# Conformational States of the CXCR4 Inhibitor Peptide EPI-X4—A Theoretical Analysis

**DOI:** 10.3390/ijms242216229

**Published:** 2023-11-12

**Authors:** Christoph Karsten Jung, Jan Münch, Timo Jacob

**Affiliations:** 1 Helmholtz Institute Ulm (HIU) Electrochemical Energy Storage, Helmholtzstr. 11, D-89081 Ulm, Germany; 2 Karlsruhe Institute of Technology (KIT), D-76021 Karlsruhe, Germany; 3 Institute of Electrochemistry, Ulm University, Albert-Einstein-Allee 47, D-89081 Ulm, Germany; 4 Institute of Molecular Virology, Ulm University Medical Center, Meyerhofstr. 1, D-89081 Ulm, Germany

**Keywords:** DFT, ReaxFF, MD

## Abstract

EPI-X4, an endogenous peptide inhibitor, has exhibited potential as a blocker of CXCR4—a G protein-coupled receptor. This unique inhibitor demonstrates the ability to impede HIV-1 infection and halt CXCR4-dependent processes such as tumor cell migration and invagination. Despite its promising effects, a comprehensive understanding of the interaction between EPI-X4 and CXCR4 under natural conditions remains elusive due to experimental limitations. To bridge this knowledge gap, a simulation approach was undertaken. Approximately 150,000 secondary structures of EPI-X4 were subjected to simulations to identify thermodynamically stable candidates. This simulation process harnessed a self-developed reactive force field operating within the ReaxFF framework. The application of the Two-Phase Thermodynamic methodology to ReaxFF facilitated the derivation of crucial thermodynamic attributes of the EPI-X4 conformers. To deepen insights, an ab initio density functional theory calculation method was employed to assess the electrostatic potentials of the most relevant (i.e., stable) EPI-X4 structures. This analytical endeavor aimed to enhance comprehension of the inhibitor’s structural characteristics. As a result of these investigations, predictions were made regarding how EPI-X4 interacts with CXCR4. Two pivotal requirements emerged. Firstly, the spatial conformation of EPI-X4 must align effectively with the CXCR4 receptor protein. Secondly, the functional groups present on the surface of the inhibitor’s structure must complement the corresponding features of CXCR4 to induce attraction between the two entities. These predictive outcomes were based on a meticulous analysis of the conformers, conducted in a gaseous environment. Ultimately, this rigorous exploration yielded a suitable EPI-X4 structure that fulfills the spatial and functional prerequisites for interacting with CXCR4, thus potentially shedding light on new avenues for therapeutic development.

## 1. Introduction

C-X-C chemokine receptor type 4 (CXCR4) [1] is a G protein-coupled receptor (GPCR) with CXCL12 as the chemokine ligand. Binding of CXCL12 to CXCR4 induces intracellular signaling cascades that drive cellular trafficking processes important for organ development, hematopoiesis, vascularization, inflammation and immune control, as well as stem cell homing and mobilization. Aberrant CXCR4/CXCL12 signaling is associated with a variety of pathophysiological conditions including cancer metastasis, enhanced tumor growth, chronic inflammation, leukemia and altered immune responses [2,3]. Furthermore, CXCR4 is a major coreceptor of HIV-1 during the late stages of infection and is associated with rapid disease progression [4,5]. Thus, CXCR4 represents an important drug target [2].

EPI-X4 (Endogenous Peptide Inhibitor of CXCR4) is a peptide antagonist of CXCR4 [6,7]. This 16-amino-acid peptide is derived via proteolytic processing of the highly abundant protein albumin and competes with CXCL12 binding to the CXCR4 receptor. EPI-X4 likely represents an important regulator of CXCR4 function in vivo and holds great prospect for clinical development because the peptide is highly specific for CXCR4, which should result in lower off-target effects and hence adverse effects [6].

Thus, EPI-X4 represents an elegant substance to regulate the receptor, though the required concentrations are relatively high, rendering a therapeutic application difficult [6,8,9]. Crucial for the improvement in anti-CXCR4 activity is an understanding of the docking process of EPI-X4 to the receptor [9]. Biochemical approaches show function and interactions of biologically relevant molecules only on larger scales, though more detailed insights even at the atomistic (or molecular) level are indispensible. The lack of suitable experimental methods to gain atomistic details for describing the dynamic evolution of the structure can be circumvented by theoretical simulations [9,10,11,12,13].

Intra- and interatomic interactions in and between molecules usually involve changes in geometry and electronic structure [14], or stabilizations thereof through traditional hydrogen bonds (H-bonds) or weakly polar interactions [15]. Density functional theory (DFT) has become an established quantum mechanical tool that has largely proven its applicability to the accurate characterization of the electronic structure of molecules [16].

However, other techniques such as molecular dynamics enable larger molecules to be characterized. By parameterizing experimentally observed and/or theoretically calculated system properties, force-field-based methods attempt to bridge larger time and length scales using an analytically formulated Hamiltonian. However, it is important to notice that the accuracy of molecular dynamics simulations often depends not only on the method chosen, but also on the underlying force field. These simulations often face a trade-off between computational efficiency and high accuracy, as highlighted in studies such as [17]. The applicability of a force field to different problems is determined by the form of the energy expression, and even more important by the quality and transferability of the parameterization [16]. Modern ab initio force fields that have been approved in extensive test simulations can provide reasonably accurate energetics and structures. In almost all force fields, the system energy is divided into several terms, which account for different types of interactions. Besides classical force field approaches, there is the class of reactive force fields being capable of even describing entire reactions [14,18]. The basis of reactive force fields is a system energy expression, where each term is explicitly bond-order-dependent. By taking the electronic configuration of each atom type into account and continuously mapping the local environment of an atom during a simulation, the bond order is regularly updated and thus certain interaction terms might vanish (bond breaking) or appear (bond formation). In addition, these force fields are able to distinguish between variations in the structure and energy of differently hybridized organic compounds.

For the present studies, we used a reactive force field [19] within the ReaxFF framework and explored the structure and properties of EPI-X4 by means of molecular dynamics simulations. The secondary structural organization of the molecules was monitored by examining both energy and entropy contributions, thus providing free energies. The lower the free energy of a particular conformation of EPI-X4, the more stable—and favored—it is. Applying this (simple) descriptor, the most stable (and probable) conformations were calculated. These EPI-X4 structures were then docked to CXCR4. The first of the two fundamental antagonist–receptor events, the antagonist binding to the receptor, is the focus of this work [20]. We will describe the suitability of the different conformers for their interaction with the receptor CXCR4.

## 2. Results

### 2.1. Characterization of the Experimentally Determined EPI-X4 Structure Using Reactive MD Simulations and DFT

The three-dimensional high-resolution NMR structure of EPI-X4 (PDB ID: 2nOx) [6] was used as the experimental reference and is henceforward labeled as structure N0.

**Figure 1:**
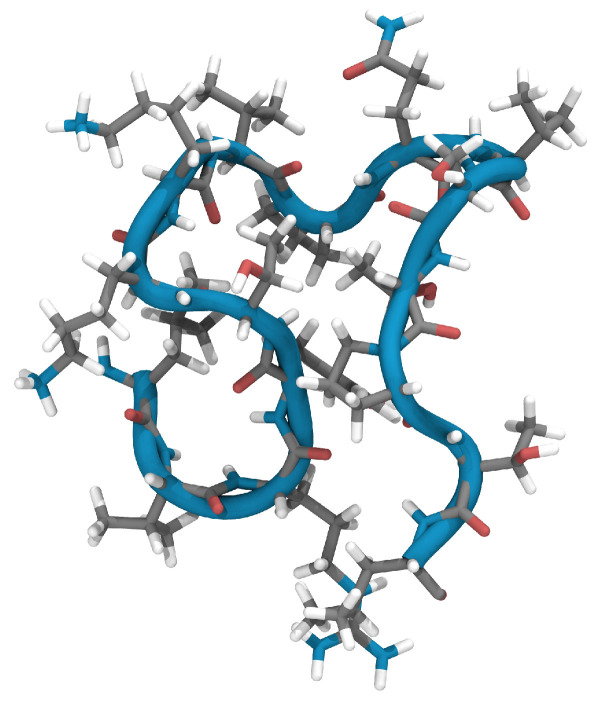
Ribbon diagram of the ReaxFF-optimized structure (N1) of EPI-X4 after optimization of structure (PDB 2nOx) [6].

Using this structure as a starting point and initiating geometry optimization within the ReaxFF framework through the conjugate gradient approach, we encountered only marginal deviations from the NMR-defined atomic positions. With an overall root mean square deviation (RMSD) of the atomic positions of 0.38 Å, the optimized configuration, now referred to as N1, remained almost equal to the NMR structure. This optimization process focuses primarily on bond lengths and distances, which may vary slightly depending on the method chosen. Figure 1 shows the three-dimensional alignment of the N1 peptide backbone and reveals the emergence of a ring-like structure accentuated by a tail of five amino acids.

**Figure 2:**
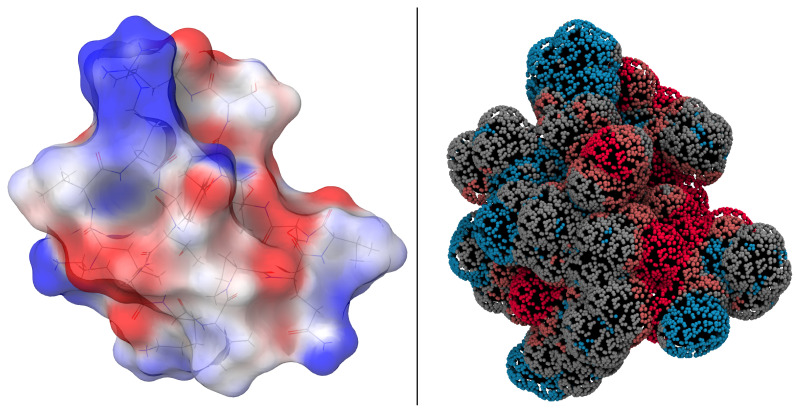
(**Left**): DFT-based electrostatic potential energy map of EPI-X4 (N1) to visualize the charge density distribution. Blue-colored areas refer to groups or sections having positive ESP, red colored areas represent negatively valued ESP regions. (**Right**): ReaxFF-based interaction potential of the N1 structure using a hydrogen-bond bridge as a probe.

Following geometry optimization, quantum mechanical calculations were performed to investigate the electronic structure of the system, with the goal of deriving the electrostatic potential created by the charge distribution of the system. The electrostatic potential distribution (ESP) of the ReaxFF-optimized N1 structure derived from density functional theory is shown in Figure 2. Molecular ESP calculations provide a quantitative assessment of the electronic charge distribution on the molecular van der Waals (vdW) surface, effectively mapping potential reactive sites.

As can be seen from the ESP distribution in Figure 2 (left), most of the molecular surface has modest values ranging from −30 to 30 kcal·mol${}^{-1}$. Positively charged regions (shown in blue) are predominantly located above and below the nine-amino-acid ring. Conversely, negatively charged regions (shown in red) are mainly located at the tail end.

Figure 2 also shows the interaction potentials calculated with ReaxFF, visualizing potentials that were not considered during force field training. A comparison with the calculations from DFT highlights the transferability and applicability of the force field for this system. The interaction potentials are visualized with a probe simulating hydrogen bonding.

While the ESP and the interaction potentials appear to show considerable agreement, it is important to recognize the different nature of the data shown by these two representations. The plot from ESP gives only the charge contributions. In contrast, the interaction potential display integrates all energy terms for each point, i.e., including contributions that exceed the Coulomb term. As a result, the absolute values do not align, but the observed trends are very similar.

In an effort to evaluate the structural dynamics in a realistic temperature-controlled environment, we performed ReaxFF molecular dynamics simulations for the N1 structure. The simulation run showed a considerable morphological change, indicated by an RMDS of more than 3 Å when comparing N1 with the structure after 1000 fs. Given these pronounced structural shifts, the two-phase thermodynamics (2PT) [21] approach was not applied to the MD simulation of N1 to evaluate the entropy contributions (see Section 4.3). The energy barrier required to leave the local minimum and induce this structural change is overcome at 300 K. The following sections deal with other conformers.

### 2.2. Creating and Analyzing Further Stable Conformations of EPI-X4

Peptides intricately fold into unique three-dimensional structures specific to the amino acid sequence of the particular peptide. While these native configurations are a result of their sequence, peptides are not rigid structures. They can exhibit a series of comparatively rapid vibrations and slower structural changes. These changes can also be influenced by rotations around the phi and psi angles, offering the potential for a wide variety of secondary structures. A comprehensive set of approximately 150,000 different conformers was created and optimized. Each conformer was generated by introducing independent random rotations about each phi and psi angle.

**Figure 3:**
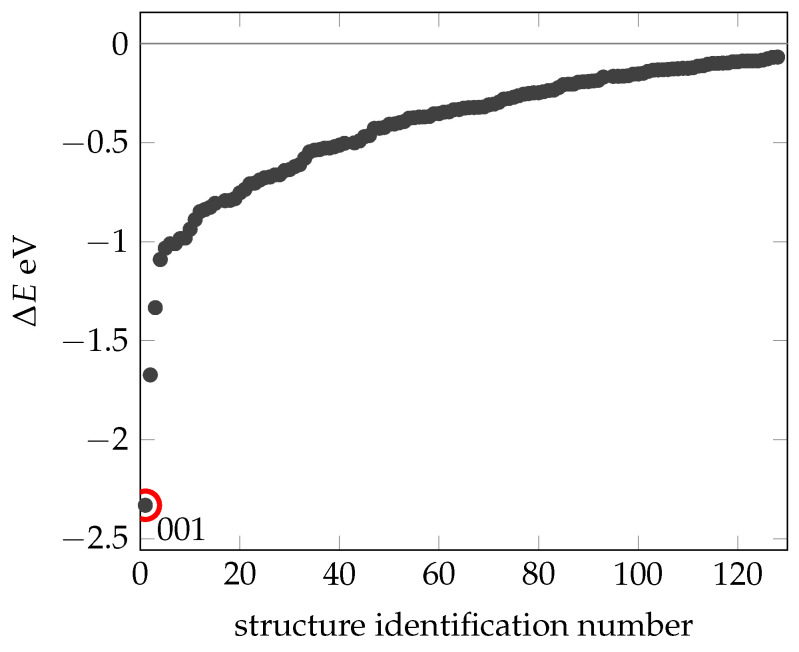
Summary of the energies (black dots) obtained for those confomers being more stable than structure N1. The x-axis shows the identification number of the structure. The dot circled in red is the most stable structure 0001. All energies are relative to N1.

The calculated energies (ΔE) for these different conformers are consolidated and plotted in Figure 3, all relative to the energy of N1 ($E_{\mathrm{Reax}}\left(N1\right)$). Using this method, we identified an additional 130 stable configurations (with lower energy) compared to the experimentally determined and geometrically optimized N1 conformation (Structures can be found in Supplementary Materials Listing S1)). The most stable of these configurations (with a total energy of −2.3 eV referencing N1), designated as 001, stands out. Short molecular dynamics simulations were performed for the structures highlighted here, as well as for a selection of others, which resemble the N1 structure. It is important to note that only structures that show no changes at 300 K by our definition (RMSD more than 3 Å) are presented. Therefore, local minima where the energy barrier to structural change is overcome at 300 K are not included in this presentation.

The range of relative energies (ΔE) extends from 0 to −2.3 V, predominantly concentrated above −1 eV. This result supports the stability of the experimentally determined NMR structure. The energy differences between the N1 structure and even slightly more stable configurations are negligible compared to the total energy of the system. These differences are mainly due to noncovalent interactions between amino acids or strands, often facilitated by hydrogen bonds.

As already observed for the N1 structure, stability within a certain temperature range has to be investigated. Incorporating temperature requires an additional descriptor describing the thermodynamic distribution of the different secondary structures. To this end, we have adapted the 2PT method to the ReaxFF trajectories of the different stable systems, which gives us insight into both the entropic contributions and the free energy. The simulations were performed at a temperature of 300 K.

**Figure 4:**
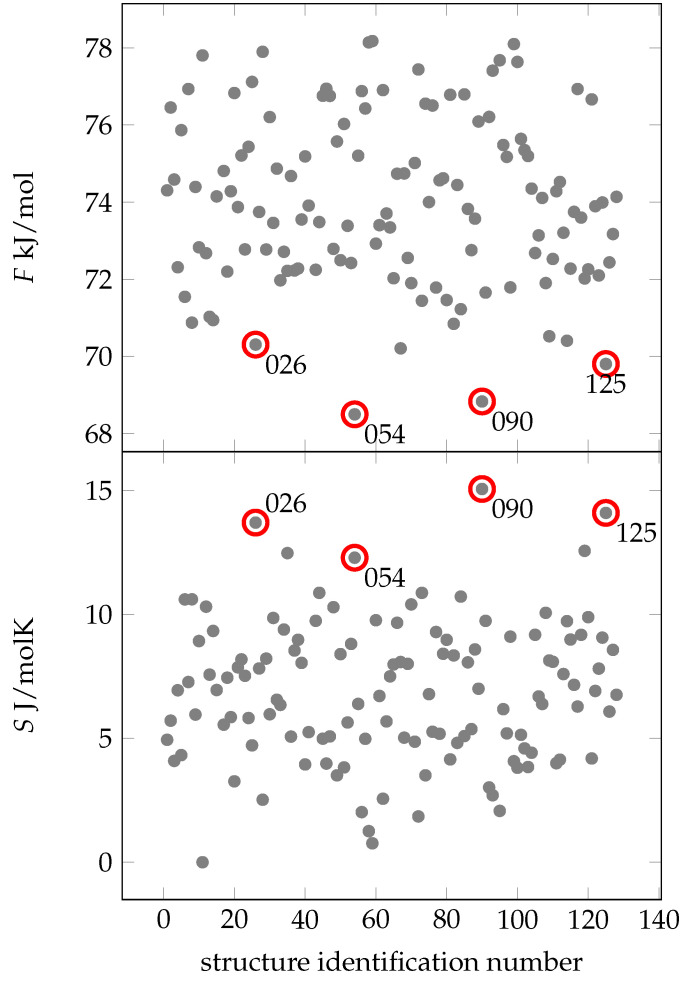
Free energies (gray dots) of different conformers of EPI-X4. All free energies are absolute values. In the lower figure are the entropic values (gray dots) relative to the lowest found entropy depicted. The conformers identified by lowest free energies (thus high entropy) are 026, 054, 090 and 125 (marked with a red circle). The x-axis shows the identification number of the structure.

Figure 4 shows that four configurations exhibit notably low free energies: C025, C054, C090 and C125. A combination of low free energy and high entropy at 300 K is crucial for the spatial arrangement of amino acids. Remarkably, the energy-minimized structure 001 has the lowest energy compared to N1, but does not have a relatively high entropy or free energy. Thus, we conclude that 001 may have no significance under biologically realistic conditions.

Looking at the MD simulations of structures C025, C054, C090 and C125 (see Supplementary Materials Figure S1), two recurring patterns are apparent. First, these structures have a lower number of hydrogen bonds (13–16), in contrast to the average of about 20 in the other structures. This suggests that these specific hydrogen bonds are exceptionally robust. Second, the residues align perpendicular to the backbone, leading to more degrees of freedom and contributing significantly to entropy at 300 K.

Contact maps (see Supplementary Materials Figure S2) were prepared to provide a clear representation of the three-dimensional coordinates of the peptide. Conformers C001, C054 and C090 reflect a similar tertiary structure to N1, consisting of a ring and a tail. However, C090 exhibits a bend in the middle of the strand.

Table 1 summarizes the SASA and energy point data. These point data are created by bringing a molecule from infinity to this point, in this case a OH with its O—H axis always oriented normal to the van der Waals surface. Using the SASA, the number of points uniformly distributed on the SASA and the energy associated with each point, we can classify it into hydrophobic, hydrophilic and other surface areas. If the energy of the point is less than −0.1 eV, then it is hydrophilic. At +0.1 eV and above, we define it as hydrophobic, and between −0.1 eV and 0.1 eV there are neither attractive nor repulsive interactions. Intramolecular hydrogen bonding is of the order of 0.3 eV. The limits were set symmetrically to one-third of this value, i.e., to the 0.1 eV already mentioned. Drawing attention to the structures N1 and C090, it is noticeable that they differ from the other structures in terms of hydrophilic and hydrophobic surface area: C090 is hydrophilic (40% of the surface is hydrophilic), while structure N1 is hydrophobic (60% of the surface is hydrophobic).

### 2.3. Docking of EPI-X4 to CXCR4

**Figure 5:**
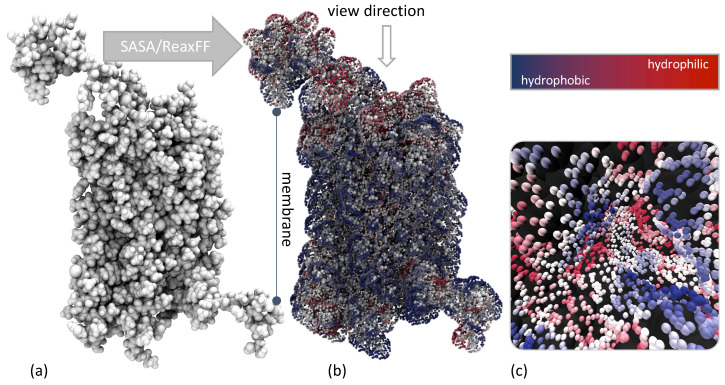
(**a**) VdW representation of CXCR4 (PDB ID: 3ODU), (**b**) ReaxFF -based interaction potential of CXCR4 using a hydrogen-bond bridge as probe and viewing direction of the Figure 5c, (**c**) identified (and not further used) by us as the most likely docking site for the EPI-X4.

As for the docking process of EPI-X4 and CXCR4, the interaction and its intensity are usually determined by the structural groups and atoms located on the outside of the peptides. For a stable interaction, the distribution of charged and uncharged groups and the hydrogen bond donors and acceptors between the corresponding EPI-X4 conformer and CXCR4 receptor must be complementary. Figure 5a shows a representation of CXCR4. Not directly visible in this representation, but present, is that CXCR4 consists of subunits with seven helical structures that cross the cell membrane and are connected by three intracellular and three extracellular loops. The regions normally surrounded by the membrane are mostly hydrophobic (blue, Figure 5b). In these interaction data, one region of the CXCR4 structure in particular caught our attention (Figure 5c). In this region, which can almost be called a pocket, hydrophilic and hydrophobic regions were close to each other. We might have assumed that this region was probably the most important one. However, it is important to clarify that our docking was conducted impartially regardless of this assumption.

To extend our analysis of the potential interaction surface of EPI-X4 (functional groups complementary to the CXCR4 pocket) with CXCR4, we performed a screening of the different conformers with respect to their spatial fit into the pocket of CXCR4.

Using the most promising stable structures we had previously identified (C001, C026, C054, C090, C125 and N1), we investigated various potential docking positions. Using a Cartesian grid, EPI-X4 was translated along the x/y/z axes and rotated around the x/y/z axes. Again, structural optimizations were performed using the ReaxFF framework. Positions requiring a minimum interatomic distance of 1.3 Å were considered to avoid unrealistic atomic overlaps. In addition, a short molecular dynamics simulation was performed at 300 K for 10,000 fs with the top 15% of CXCR4 atoms unfixed to allow for relaxation.

**Figure 6:**
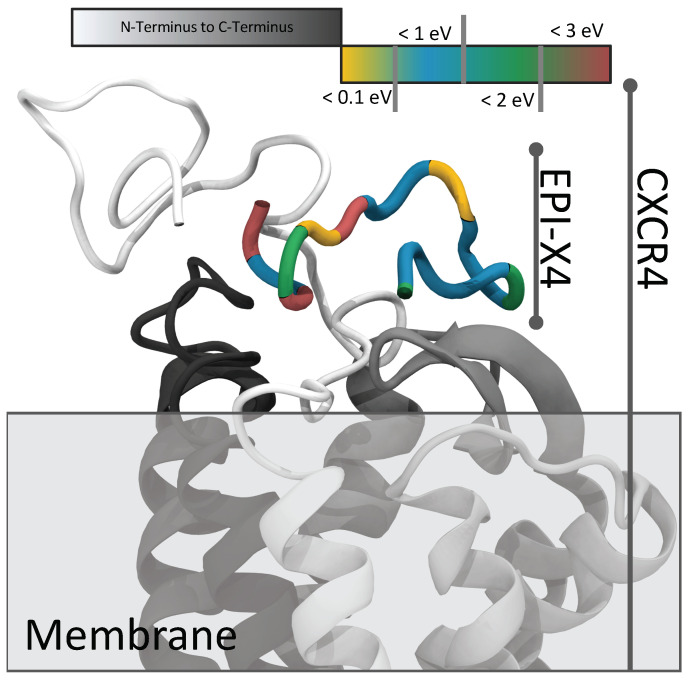
Schematic representation of the docking of EPI-X4 (colored, energy dissolved) to CXCR4 (white to black) (PDB ID: 3ODU).

Surprisingly, no feasible docking position was found for the most energetically stable structure (conformer 001). Instead, only conformer C090 showed a compatible fit to CXCR4, as shown in Figure 6.

This specificity arises from the structural features of C090: a lengthy, nearly linear strand interacting within the CXCR4 pocket complemented by a small loop-like tail extending outward. Subsequent MD simulations from this docked position show no significant structural shifts or increased interaction energy. This suggests a stable local minimum energy. However, it is important to note that no molecular dynamics simulations were performed without fixed atoms, as they are beyond the scope of this study. To achieve this, the CXCR4 structure would need to be embedded in a lipid membrane to facilitate structural relaxation. To date, CXCR4 calculations have been limited to the gas phase, requiring fixation of the atoms.

The EPI-X4 conformer C090 shows favorable potency toward CXCR4, a critical attribute for antagonists. The accurate determination of ligand affinity and efficacy is of great importance for drug discovery and basic biology. When the structural group topology and spatial compatibility of the previously generated conformers with CXCR4 were investigated, the C090 structure of EPI-X4 was found to be well suited for docking to CXCR4 in the gas phase. This makes the C090 conformer particularly interesting for further investigation.

## 3. Conclusions

Through a combination of molecular structure optimization within the ReaxFF framework, molecular dynamics simulations, and quantum mechanical methodologies, various conformations of EPI-X4 were meticulously examined. An NMR-derived structure, subsequently optimized through ReaxFF, served as the reference. The strong alignment between these outcomes and experimental data underscored the effectiveness of the ReaxFF approach.

Furthermore, the conformers displaying favorable attributes in terms of low free energy (accompanied by high entropy) exhibited shared structural characteristics: most displayed a ring-like configuration along with an accompanying tail. Utilizing quantum mechanical calculations, notably density functional theory, to analyze electrostatic potentials facilitated the assessment of potential hydrogen bonding and hydrophobic interaction sites. Consequently, EPI-X4 should not be construed as a singular explicit secondary structure, but rather as a dynamic ensemble of conformations.

In conclusion, the docking of EPI-X4 onto CXCR4 was systematically investigated. The stable conformers identified thus far underwent rigorous testing to ensure spatial compatibility and appropriate complementarity of functional groups on the conformer’s surface with the receptor protein. Among these conformers, one emerged as both suitable and energetically stable. Future investigations should delve into embedding the CXCR4 system within a lipid membrane, enabling system-wide relaxation and facilitating exploration of the antagonist’s impact on the receptor protein. In addition, solvation effects, including explicit water molecules, are generally believed to play a significant role. Consideration of these factors in future studies will provide a more comprehensive understanding of the EPI-X4-CXCR4 complex.

Drawing on the accumulated results, and with the incorporation of forthcoming CXCR4 studies, the possibility of chemically modifying EPI-X4 could be explored to enhance its potential therapeutic applications.

## 4. Theoretical Methods

### 4.1. DFT

All electronic structure calculations were performed using the Jaguar program (v2018-3) suite [22,23]. Spin-unrestricted density functional theory calculations were performed using the B3LYP gradient-corrected exchange–correlation functional [24,25], which combines the exact Hartree–Fock exchange and the Slater local exchange functional [26]. This approach also integrates the Becke gradient correction, the local Vosko–Wilk–Nusair exchange functional, and the Lee–Yang–Parr local-gradient-corrected functional [25,27,28]. All atom types (H, C, N and O) were described with the basis set 6-311G**, which is characterized by a triple split-valence basis.

### 4.2. ReaxFF

The molecular dynamics (MD) simulations in this study are based on the ReaxFF approach [18]. In particular, ReaxFF includes bond-order-dependent energy terms that are continuously updated as the local environment of the atoms is mapped during the system dynamics. For a detailed description of the ReaxFF formalism, see references [18,29]. We used C/H/O/N glycine/water parameters, originally developed by Rahaman et al. [30] and later extended by Monti et al. [19]. Rigorous testing evaluated the accuracy and transferability of this force field. The training set included amino acid structures obtained via quantum mechanical studies at the DFT-B3LYP/6-311++G** level [19,25], and the simulations were compared with the results of the classical nonreactive force field ff99SB of the AMBER family [31].

The following preparatory steps were performed for the EPI-X4 system: A peptide was first positioned in the center of a square box whose edges measured 100 Å. System optimization was performed using the conjugate gradient algorithm, with the convergence criterion for minimizing the molecular mechanical energy set to 0.5 kcal·mol${}^{-1}$. Subsequently, an NVT (constant particle number *N*, volume *V* and temperature *T*) velocity Verlet algorithm dynamics at 300 K was applied using the Berendsen thermostat [32] with a coupling constant of 100 fs. Equilibration was performed with 100,000 iteration steps (0.25 fs per time step) followed by 10,000 iterations analyzing the position and velocity of each atom in each iteration. These MD simulations formed the basis for the subsequent two-phase thermodynamic analysis of the thermodynamic properties of the system.

### 4.3. 2PT Approach

Quantum mechanical methods can access energies and enthalpies at low temperatures. These approaches reach their limits for systems of higher complexity and size [16,33]. However, entropy can be an important driving force for interactions in biological or organic systems. The evaluation of entropy contributions to the total energy of processes leads to more realistic modeling. The “2-phase thermodynamics” (2PT) method allows the precise use of MD trajectories to determine thermodynamic observables [21,34,35,36]. A detailed description of the evaluation of molecular dynamics simulations in conjunction with ReaxFF can be found in Jacob et al. [21]. For this purpose, the autocorrelation function of the velocity of atoms C(t) is extracted from an MD trajectory, which allows the calculation of the density of states DoS(ν) via Fourier transform [34]. The thermodynamic properties were obtained directly from the velocity autocorrelation function derived from our molecular dynamics simulations. The simulations were performed as described above. It is possible that the structure changes significantly during this time. We therefore defined the following: a structure remains the same during the simulation if it deviates by a maximum of 3 Å in RMSD (RMSD change during simulation <3 Å). We made the assumption that if the RMSD is larger than 3 Å, the structural change is significant enough that the similarity to the initial structure is no longer given.

### 4.4. Interaction Potential of Structures

To link the calculations based on DFT to the force field, we took the surface area accessible to the solvent (SASA) and used ReaxFF to screen for interaction with relevant model groups, e.g., OH, NH and atomic oxygen and hydrogen. Here, the interaction means the preference of the site for an electrophilic or electrophobic species. First, the SASA was created by distributing a series of analysis points (23,500 points) along the van der Waals surface of the peptide. Then, at each point, the interaction of a probe atom with the particle was evaluated using ReaxFF single-point calculations. These points were then displayed with color coding according to their reactive interaction energies, providing a picture of the system regions of attractive or repulsive character.

### 4.5. Exploration of Binding Sites through Grid-Based Docking

To investigate the potential binding sites between EPI-X4 and CXCR4, we used a docking approach based on a Cartesian grid. In this method, EPI-X4 was systematically shifted along the x, y and z axes and simultaneously rotated about these axes, while CXCR4 remained fixed in position. Structural optimization was performed using the ReaxFF framework for each point on the grid. We evaluated positions that maintained a minimum interatomic distance of 1.3 Å preventing unrealistic atomic overlaps.

We then performed a short 10,000 fs molecular dynamics simulation at 300 K with the top 15% of CXCR4 atoms unfixed to facilitate structural relaxation.

## Figures and Tables

**Table 1:** Surface characterizations of the diffrent structural arrangements.

	C001	C026	C054	C090	C125	N1
Surface [Å${}^{2}$]	1986	1868	1980	1913	1916	1969
Hydrophil [Å${}^{2}$]	601	615	600	749	635	607
Else [Å${}^{2}$]	448	435	351	541	451	178
Hydrophob [Å${}^{2}$]	935	817	1027	621	829	1183

## Data Availability

Data are contained within the article and Supplementary Materials.

## Supplementary Materials

The following supporting information can be downloaded at https://www.mdpi.com/article/10.3390/ijms242216229/s1.
