# Supplementary material for: Conformational States of the CXCR4 Inhibitor Peptide EPI-X4—A Theoretical Analysis
Source: Int J Mol Sci. 2023 Nov 12;24(22):16229. doi: 10.3390/ijms242216229 (PMC10671355; doi:10.3390/ijms242216229)
Supplement: Supplementary file 1 [file ijms-2613912-supplementary.pdf › ijms-2613912-supplementary.pdf]

Article

Conformational states of the CXCR4 inhibitor peptide EPI-X4 - a theoretical analysis

Christoph Karsten Jung [1,2,3]*, Jan Münch [4], Timo Jacob [1,2,3]

[1] Helmholtz Institute Ulm (HIU) Electrochemical Energy Storage, Helmholtzstr. 11, D-89081 Ulm, Germany
[2] Karlsruhe Institute of Technology (KIT), P.O. Box 3640, D-76021 Karlsruhe, Germany
[3] Institute of Electrochemistry, Ulm University, Albert-Einstein-Allee 47, D-89081 Ulm, Germany
[4] Institute of Molecular Virology, Ulm University Medical Center , Meyerhofstr. 1, D-89081 Ulm , Germany
* Correspondence: christoph.jung@kit.edu

1. SI

Here, the distance between all possible amino acid residue pairs of the three-dimensional peptide structure is represented using a two-dimensional matrix. The ij element of the matrix is colored darker, depending on how close the two residues are. The distance between atoms i and j (*i.e.* the bond C_i–C_j) atom has been established at the threshold of 11 Å. Figure (see SI (f)) also shows the contact map for the published EPI-X4 structure (N1).

(a) Confomer C001

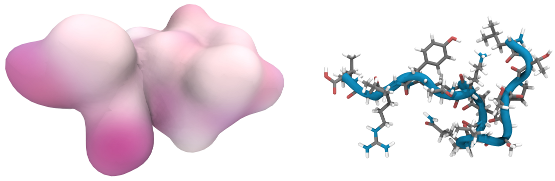

(b) Conformer C026

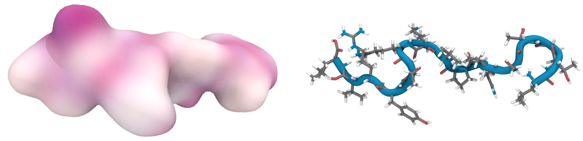

(c) Conformer C054

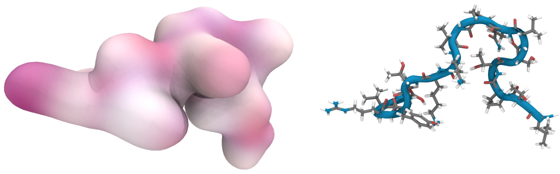

(d) Conformer C090

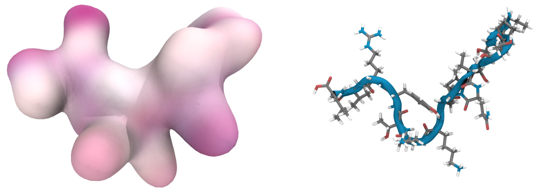

(e) Conformer C125

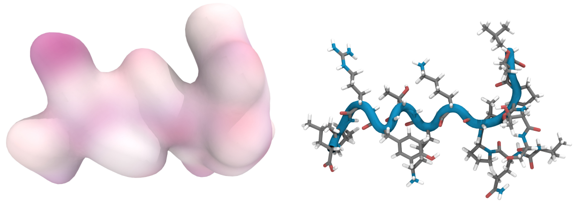

Figure S1. Charge distribution of selected conformers gained from ReaxFF structure optimizations.(a) Conformer 001 as the energetically favored conformer, (b)-(e) conformers C026, C054, C090 and C125. Red color refers to negatively charged areas, white represents charge neutrality and blue would refer to positively charged areas of the structures. On the right side are schematics of the conformers shown.

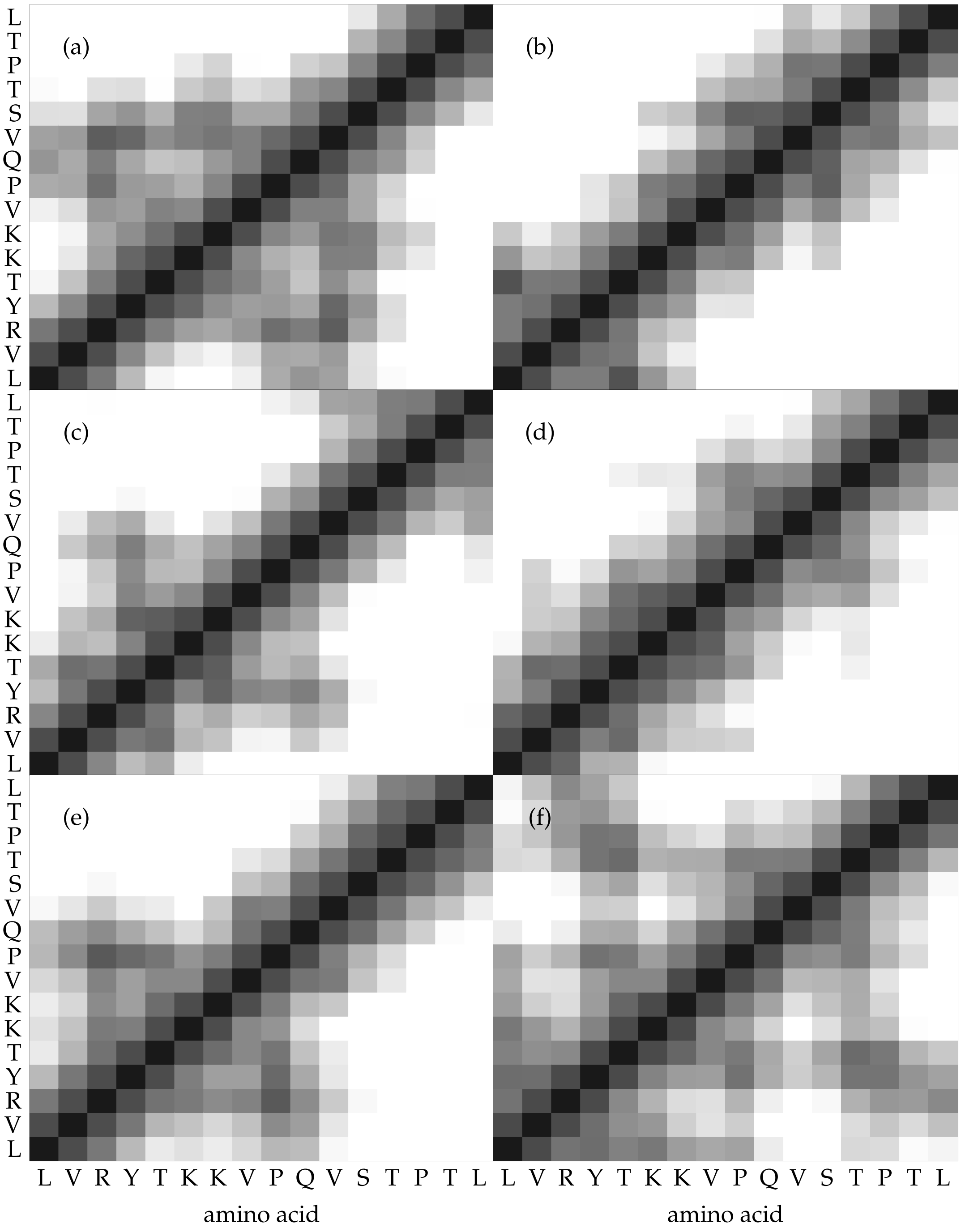

Figure S2. Contact maps of certain conformers: (a) Conformer 1 as the energetically favoured structure (b)-(e) conformers C026, C054, C090 and C125 (f) NMR conformer N1. White stands both for a distance of 11 Å and more, black for 0 Å. The gray tones are linearly interpolated between white and black.

Contents

1 EPI-X4 xyz structures **1**

2 control - SCM's ReaxFF Input **637**

3 MD - CXCR4 + EPI-X4 **638**

1 EPI-X4 xyz structures

The stable structures in comparison with the NMR structure (and this one).

Listing 1: C00001.xmolout

```
      276
 150.0000  150.0000  150.0000    0.0000    0.0000    0.0000***********    1.7765     7106
      0.2500     300.00    0.00    0.00    0.00   -1295.6273      0.0000    300.0000
H   67.50467  66.03206  73.75534    -2043.14101    -1757.64893      399.89339          1
N   67.90687  66.60675  74.49259      426.75047      490.45733     -334.42467          1
H   67.09319  66.85163  75.05133      442.25559    -1110.30854    -2448.52907          1
C   68.70489  67.69291  74.07796      415.32972     -666.39213       83.73172          1
H   69.30200  67.30130  73.22781      879.32219     2491.00447      667.35213          1
C   67.74093  68.92001  73.68223       31.35357      477.41777      628.23362          1
H   67.18670  69.25304  74.59262     1168.38489     2240.25337    -1019.71136          1
H   68.35145  69.79807  73.36012     -360.43770     2534.90498      931.67112          1
C   66.77373  68.51515  72.59811      463.18208      512.70242     -382.34388          1
H   66.25868  67.57969  72.91111     1725.29117     1863.52247     -987.07854          1
C   65.71525  69.59270  72.39998      710.42801     -144.27427     -313.15367          1
H   64.97212  69.30247  71.61282      364.20195    -1033.13725     2494.72524          1
H   65.14128  69.78840  73.34251     2567.61904      335.80679      847.48802          1
H   66.17203  70.56514  72.08157    -2587.99029      680.92867     -512.16411          1
C   67.49361  68.25718  71.28184      268.17616      741.64379      -40.42634          1
H   66.77736  67.97373  70.46770     1283.54314    -1945.37420    -1411.27812          1
H   68.05007  69.16522  70.93229    -1258.57796     1317.15249    -2026.02085          1
H   68.23920  67.42568  71.37139    -2502.84526      909.80597      575.92391          1
C   69.58912  68.12512  75.37727      619.36578     -458.64843     -172.09273          1
O   69.10705  68.76066  76.29653      -81.40433      433.87611      522.34594          1
N   70.85223  67.72967  75.22088      175.23996     -622.20190      341.55523          1
H   71.07607  67.17914  74.39208     -876.57256     -303.22879    -2561.90213          1
C   71.59349  67.54527  76.43883     -571.88214     -534.10783     -106.15033          1
H   70.79727  67.52437  77.21635    -2713.20841      -16.25800     -248.80809          1
C   72.44684  66.19917  76.31609      166.60354      288.16399     -716.09568          1
H   73.18667  66.31682  75.47840    -2097.85574     1636.78449     -586.18044          1
O   71.69000  65.01068  76.03298     -317.80580     -363.38227      484.43082          1
H   72.09925  64.71701  75.19455     1863.74748    -1451.84923     1357.29467          1
C   73.21840  66.20910  77.68776      503.80731      517.99032      318.51606          1
H   73.94022  65.36244  77.75566      640.61020    -2157.10410    -1536.29104          1
H   73.80150  67.15012  77.83254     1687.88455      465.60692     2087.56452          1
H   72.51756  66.11527  78.54959    -1293.07895    -1899.88581    -1463.57452          1
C   72.63430  68.78404  76.59303     -153.07550     -733.68902      248.70981          1
O   73.61624  68.87146  75.87299      186.15459     -480.78787     -449.34387          1
N   72.27568  69.56283  77.61520     -487.53139      543.18517       42.03850          1
C   73.04234  70.72584  77.95789     -699.35871       69.34393      360.10701          1
H   73.39053  71.14887  76.98966        2.81126     -221.99807    -2715.58072          1
C   71.98395  71.60116  78.74255     -507.46439     -592.59194      122.07761          1
H   72.46211  72.36385  79.39667    -1975.08116      525.87560    -1801.71556          1
H   71.32949  72.15301  78.03221     1588.27743    -1913.22100     1113.83566          1
C   71.18086  70.51101  79.52250     -613.62442     -457.55993     -194.14490          1
H   71.73160  70.19682  80.43719     1707.54637     -721.27992     1996.92523          1
H   70.17672  70.85174  79.86106      758.23357     2100.81767     1560.55011          1
C   71.11360  69.33173  78.46295     -352.41524      704.96076       49.21465          1
```

H	71.20805	68.30973	78.90104	1714.76886	-2070.42778	443.35792	1
H	70.22412	69.35705	77.75695	1224.15040	1801.50881	1636.97633	1
C	74.29204	70.20397	78.88857	566.85117	537.05831	117.62814	1
O	74.35944	70.43024	80.06883	-550.26779	-406.08867	4.21666	1
N	75.10344	69.50789	78.09856	566.34832	-425.77725	180.18749	1
H	74.85432	69.37418	77.09582	644.44502	2646.80550	-52.73430	1
C	76.43380	69.26456	78.58789	612.34801	-281.48662	411.56221	1
H	76.31023	68.72733	79.55389	1667.23498	-1490.75142	1556.16753	1
C	77.13853	68.41691	77.43634	-714.45746	-175.43074	286.98927	1
H	77.02126	68.98044	76.47076	-787.09484	1763.03619	-1922.46060	1
O	76.57272	67.11432	77.18389	-429.46514	516.51932	-128.40526	1
H	76.19034	67.24455	76.29290	1981.82785	436.16847	-1818.18182	1
C	78.64606	68.45608	77.88369	630.79189	-468.94448	76.03230	1
H	79.30769	67.97335	77.12756	-1775.71861	2062.30353	131.89849	1
H	79.00909	69.50177	78.02321	-1855.14608	88.94118	1993.53760	1
H	78.79966	67.91953	78.84847	-1504.82350	-780.94249	-2132.90992	1
C	77.11921	70.72276	78.76248	288.57638	618.62814	-397.00270	1
O	77.24881	71.48979	77.81134	321.70495	-185.26452	574.37218	1
N	77.55870	70.92090	80.03762	-447.05792	-446.53431	367.76425	1
H	77.33186	70.00450	80.45788	350.31316	1975.63644	-1843.31522	1
C	76.90830	72.03780	80.72782	-511.23061	374.03917	-471.51480	1
H	75.91038	72.10271	80.23481	2234.79579	-898.70890	-1273.45190	1
C	76.81895	71.56396	82.24587	529.53781	-159.34528	563.72604	1
H	76.32568	72.37159	82.84977	654.59388	2347.94251	1217.51488	1
H	76.12195	70.68409	82.26225	-1526.55661	2103.42026	817.87394	1
O	78.07639	71.21624	82.89776	-554.84278	14.12789	399.58769	1
H	78.36291	70.49401	82.31243	1297.06236	2061.84854	1220.68812	1
C	77.72757	73.39128	80.58280	-662.15484	-412.13544	123.62687	1
O	77.74185	74.22915	81.46761	-540.01537	-317.05804	274.91456	1
N	78.28211	73.47425	79.37147	-202.76299	-690.00100	-131.49145	1
H	78.04981	72.74780	78.64765	-702.62132	-2617.37191	281.70413	1
C	79.12342	74.62070	79.00829	-491.73914	576.34187	222.73509	1
H	79.66224	74.85073	79.95846	1977.17978	-1823.58850	-434.68928	1
C	80.00058	74.19279	77.84728	-649.50797	-328.66843	306.11224	1
H	79.33352	73.98493	76.97809	2075.07803	1452.16490	-1004.45904	1
C	80.95990	75.29693	77.44242	-615.18735	-489.99689	-70.96352	1
H	81.58311	75.00140	76.55391	-1226.32661	-1724.38906	-1716.47170	1
H	80.40194	76.23622	77.16543	2381.64273	-969.84735	900.46860	1
H	81.66821	75.55903	78.27621	2122.74991	1702.23946	-141.36228	1
C	80.76566	72.91784	78.15280	-501.86074	-479.61276	-376.42482	1
H	81.37147	72.57291	77.27062	993.79261	-2246.95401	-1177.81305	1
H	81.47600	73.05941	79.01298	42.71279	-626.15391	-2651.37258	1
H	80.07215	72.07731	78.43359	-2026.39012	-1590.33709	-887.82922	1
C	78.08471	75.83580	78.55982	-672.89153	382.57394	-156.34072	1
O	78.13286	76.27196	77.44050	410.04627	221.86223	500.35950	1
N	77.40525	76.24963	79.61603	-378.11782	-443.79414	-441.10984	1
H	77.57352	75.73793	80.51208	2404.15973	-568.14894	1149.30085	1
C	76.09132	76.81654	79.46788	-544.54484	466.16019	331.29119	1
H	76.02455	77.51722	80.32982	-1409.80988	-170.17955	2325.32679	1
C	75.97848	77.53701	78.07071	-184.48284	116.78282	-758.89137	1
H	76.90538	78.12634	77.87134	1893.78641	1861.14652	611.04528	1
H	75.92327	76.77468	77.25295	-2170.79665	-974.19311	1327.50117	1
C	74.73279	78.47818	78.01747	389.51668	672.81878	-138.49152	1
H	73.79768	77.87495	78.05768	510.64577	1933.03327	1851.02484	1
H	74.70548	79.17087	78.88572	-2302.30869	-1105.72939	-948.89774	1
C	74.85818	79.29245	76.64731	-559.93427	-126.34590	542.30857	1
O	75.09032	80.47114	76.58906	-670.36003	-111.83166	76.36448	1
N	74.72309	78.43315	75.62859	-216.06729	-537.07037	446.51379	1
H	74.58869	77.38111	75.74746	-1251.96487	1074.12894	-2168.52500	1
H	74.87042	78.62655	74.61232	-2451.83023	-720.73851	944.84612	1
C	75.01965	75.59585	79.61113	546.24052	-563.88337	-85.11809	1
O	75.11550	74.58121	78.97234	71.70702	-307.40463	606.69673	1

N	74.06823	75.94915	80.50356	479.90863	-504.50129	222.86849	1
C	73.04390	75.04331	80.87025	75.51268	-600.91904	506.73700	1
H	73.40228	74.02297	80.60523	-2033.61562	1795.30103	254.89480	1
C	72.87521	75.35797	82.41300	523.79067	517.99274	284.45581	1
H	71.90545	74.99835	82.82498	1599.61665	-1127.00911	1895.98173	1
H	73.67974	74.88336	83.01810	-1881.83093	1275.82488	-1501.55016	1
C	73.00690	76.91416	82.41380	-600.05639	-404.05026	316.64485	1
H	72.05545	77.39463	82.08536	-2597.05260	496.36221	657.73249	1
H	73.24746	77.34118	83.41351	-1666.24518	1294.38985	-1723.90591	1
C	74.15852	77.13912	81.34547	493.57570	-129.95376	-602.56394	1
H	74.01637	78.03004	80.68963	-1469.08415	-637.84797	2204.22581	1
H	75.19365	77.14515	81.76535	-1038.97840	1068.88701	-2280.71789	1
C	71.60616	75.44552	80.11771	188.88100	241.05089	-727.87821	1
O	71.10559	76.54103	80.22188	531.88613	-49.19943	427.07960	1
N	70.81306	74.24628	80.40650	708.14833	26.93417	-179.73509	1
H	71.36353	73.42484	80.65918	-2232.69501	-1179.09831	1023.94816	1
C	69.81847	73.97521	79.35940	-738.32412	259.92548	104.42456	1
H	69.55203	72.89351	79.41996	2028.89123	1260.02983	1311.33324	1
C	68.63477	74.91059	79.56705	-258.65678	-345.52513	661.28459	1
H	69.02888	75.95434	79.55278	-1826.06090	-1801.46097	-918.64554	1
C	67.60443	74.76279	78.46439	-541.23400	531.92970	-218.41428	1
H	66.72750	75.44939	78.62063	1311.73218	-2219.18200	882.19023	1
H	68.03982	75.00195	77.45500	-1048.71298	1223.58118	-2196.97971	1
H	67.20007	73.71433	78.41360	419.06414	1903.92898	1903.44670	1
C	67.99175	74.68767	80.92360	-423.86431	152.66566	-648.55262	1
H	67.14663	75.40575	81.10973	105.68386	-2342.50080	1387.51238	1
H	67.57499	73.64762	81.01940	-1695.63603	-1484.73621	-1531.02793	1
H	68.73971	74.82477	81.75275	-1853.57121	-487.39031	1936.59343	1
C	70.72318	74.37030	78.04811	539.71428	-510.58232	267.58633	1
O	71.75078	75.13817	78.26745	-15.93758	-278.34085	-624.49402	1
N	70.31587	73.90371	76.93477	-676.81886	12.05292	276.17960	1
H	69.47463	73.35074	77.14182	-1694.44250	-373.84189	-2100.66098	1
C	70.88770	74.15421	75.65605	412.33078	-664.70360	108.35451	1
H	70.05658	73.92956	74.95122	1765.77476	1294.44366	1621.76594	1
C	71.37992	75.65935	75.64111	-179.66635	-721.95667	-264.73915	1
H	70.79220	76.28138	76.35430	-607.34922	2587.87032	-598.10018	1
H	72.44011	75.72323	75.99688	-359.69332	-1479.13954	-2259.74260	1
C	71.26055	76.19784	74.17569	-240.01076	616.02425	431.85322	1
H	71.50853	75.40415	73.43528	-1359.17131	782.54425	2227.99181	1
H	70.21480	76.50752	73.95427	-820.36667	85.81767	-2596.78715	1
C	72.26134	77.41004	74.03350	-513.07015	236.18570	-551.87280	1
H	72.20703	78.07682	74.92474	1816.11944	-1183.11722	-1650.94321	1
H	73.31584	77.03947	74.00514	-1800.04429	-1361.53473	1526.34643	1
C	71.91881	78.20583	72.74431	726.08972	-253.19732	-179.64713	1
H	71.80074	77.57268	71.84148	1224.27271	-456.51798	-2390.90304	1
H	71.04969	78.88987	72.83150	1206.40339	-1150.93514	-2154.90344	1
N	73.13772	79.10526	72.44847	329.70796	-633.68968	-155.67358	1
H	73.31814	79.35717	71.39023	1348.31305	1938.76845	1359.00643	1
H	73.12503	80.05203	72.93782	2096.92035	1446.54551	-966.48890	1
H	74.09871	78.66677	72.76654	-1197.07217	-2303.53719	827.28733	1
C	72.18323	73.18300	75.40797	-378.01186	-556.17301	-413.96481	1
O	72.14571	72.41107	74.47659	-409.44433	-307.55053	-453.30833	1
N	73.14198	73.34845	76.31640	-429.02477	524.70884	274.08502	1
H	72.94972	74.04291	77.04966	1706.34263	-1425.96036	1574.38926	1
C	74.51473	73.25145	75.87094	-45.25864	-626.22952	478.93267	1
H	75.01963	73.12711	76.85761	1988.63164	570.90773	1772.87287	1
C	74.65264	71.99291	74.92501	551.77128	-20.49526	-564.54996	1
H	73.93594	71.19325	75.23223	1434.61535	-1753.10253	1513.99471	1
H	74.34778	72.25075	73.88459	-714.67738	1819.09770	1898.36494	1
C	76.12781	71.47209	74.96882	446.99263	-555.77305	338.97221	1
H	76.83440	72.20421	74.51178	1953.68466	1892.25957	-161.67766	1
H	76.48100	71.33424	76.03370	-403.03785	2688.07032	-188.43591	1

C	76.11684	70.10250	74.18033	567.86262	335.74681	434.04421	1
H	75.51781	69.33497	74.73000	-1080.02275	1568.26081	-1948.78900	1
H	75.61272	70.22116	73.19357	-919.54108	2111.34692	1456.13463	1
C	77.58303	69.65084	73.98318	195.81897	173.33862	-745.11536	1
H	78.18725	70.32227	73.34118	1405.67949	-2301.21089	-390.08113	1
H	78.13701	69.42822	74.91670	-940.37328	-1118.32446	2299.72133	1
N	77.54327	68.29176	73.20690	319.26422	656.36007	-42.02424	1
H	78.48794	67.87119	72.97075	1961.03633	619.91968	1787.09422	1
H	77.06596	67.47360	73.68592	854.14343	-2156.80752	1429.08711	1
H	77.07290	68.29418	72.25497	1237.02905	-1378.50857	-1998.28500	1
C	74.89673	74.67619	75.21959	532.53319	-268.57236	517.55729	1
O	74.53502	75.71279	75.78518	266.16714	-290.73804	-558.88002	1
N	75.60224	74.59206	74.10686	-226.25352	640.49678	-270.32839	1
H	75.82237	73.65102	73.78665	2007.69063	-1035.27229	1523.50231	1
C	76.56641	75.63785	73.86930	-540.76542	-573.33303	49.50000	1
H	76.63032	76.12822	74.86900	-2374.20950	-1036.76023	843.75774	1
C	77.91204	74.91408	73.40951	-77.09082	666.87422	-415.84136	1
H	77.76623	74.41621	72.40976	-985.81834	617.19825	2463.91927	1
O	78.39053	73.84684	74.25592	293.29899	-391.65602	-477.80926	1
H	78.48742	73.12263	73.60614	2528.72714	-681.76262	-751.27122	1
C	78.85893	76.15837	73.24696	252.47363	-605.14473	440.05134	1
H	79.84528	75.86684	72.81739	-2052.65063	1456.49051	-1043.51830	1
H	78.41763	76.93173	72.57468	-2152.98800	-1661.97574	-161.70652	1
H	79.05683	76.64523	74.23039	-473.12726	-1584.27339	2165.61725	1
C	75.94142	76.64861	72.76363	360.36307	488.46713	505.10056	1
O	75.34729	77.66640	73.17503	-200.08817	-528.03552	-385.82850	1
N	76.15797	76.27967	71.53397	-597.54095	-226.44775	355.20479	1
H	76.66025	75.39975	71.42034	2179.23655	-1300.70981	-991.33835	1
C	75.42998	76.81408	70.43759	-687.68921	297.39807	-249.45179	1
H	75.79384	76.24222	69.55428	-349.16256	-2467.37713	-1101.72831	1
C	73.86611	76.63250	70.74662	672.47345	361.54328	-201.62880	1
H	73.67030	76.97502	71.78822	1541.41502	582.18425	2169.97026	1
H	73.27228	77.27845	70.05690	-2534.67717	60.25153	997.72295	1
C	73.47500	75.15803	70.58690	574.26774	-389.43119	-377.02542	1
C	73.25559	74.62065	69.28259	688.82679	-287.33580	-257.96133	1
H	73.37869	75.25160	68.38369	331.14182	1470.38163	2269.80013	1
C	73.33317	74.33863	71.74701	-383.52382	-675.04808	144.24917	1
H	73.52874	74.74663	72.75619	4.09847	1517.59662	2262.86397	1
C	72.87661	73.28352	69.16874	-134.76730	428.45285	-649.50303	1
H	72.70250	72.86648	68.16148	-903.23433	1600.49020	2011.53387	1
C	72.93596	73.01034	71.57804	-335.53281	-615.00040	-364.39058	1
H	72.78930	72.38465	72.48260	1596.45398	-1369.33862	1732.02659	1
C	72.69529	72.43266	70.29780	585.48547	-340.24194	-406.23938	1
O	72.29911	71.08000	70.13044	-419.15649	-173.04408	-511.94141	1
H	72.23047	70.80930	71.07714	-1071.84350	1559.71507	-1960.13012	1
C	75.58173	78.42432	70.30475	291.41878	-183.13091	710.72246	1
O	74.57051	79.14052	70.32009	379.87695	523.47396	-222.23622	1
N	76.83314	78.76757	70.12642	564.30591	373.09380	277.24371	1
H	77.53344	78.02609	70.14890	-120.59931	-2436.07824	-1214.35098	1
C	77.18044	80.14755	70.27782	404.07514	-299.37480	608.84009	1
H	76.24760	80.63642	69.91123	-1071.07882	-841.69025	2359.66467	1
C	77.38273	80.39032	71.83507	-683.12807	68.71770	-390.13096	1
H	76.65445	79.78476	72.42215	2068.88918	1596.58590	770.89603	1
H	78.38702	80.02666	72.14829	1379.86429	2342.49779	-179.85638	1
C	77.21642	81.90638	72.14717	-176.39967	537.49466	550.97268	1
H	77.63046	82.53943	71.33246	-396.28051	1942.22168	1869.33314	1
H	76.14498	82.19358	72.22446	-94.44700	586.00678	-2659.20016	1
C	77.99881	82.16023	73.52912	-291.69381	-718.18079	-150.72929	1
H	77.47587	81.81165	74.45510	-2303.38776	1089.76242	964.62032	1
H	79.04778	81.77044	73.48823	884.16729	2568.80533	-207.74283	1
N	78.08455	83.55033	73.51091	-594.49522	-252.81478	342.29276	1
H	78.51235	83.95122	72.68696	-1735.17426	2084.05705	-263.71605	1

```
C   77.72203  84.34529  74.44212      595.23177     -271.52487      442.22352          1
N   77.15331  83.80832  75.59728      585.53813     -424.79750     -105.81303          1
H   76.94115  82.83854  75.83128      959.88797     1327.56823    -2177.11905          1
H   76.83289  84.35161  76.40007     1578.48647       16.27378    -2220.76233          1
N   78.00395  85.66779  74.08131     -452.17763     -390.33541     -421.51853          1
H   78.43013  86.05368  73.23970      470.26107     1974.25629    -1817.92087          1
H   77.81292  86.46775  74.68593     1856.67307    -1573.97321    -1224.35416          1
C   78.48465  80.41131  69.34286     -531.47271     -581.45780      -55.06206          1
O   78.54742  79.92761  68.21156     -243.00837      192.73507      609.52485          1
N   79.33313  81.24267  69.91093      343.49288     -644.25607       38.09164          1
H   79.07475  81.53375  70.85040    -1691.16043    -1535.97525    -1484.73099          1
C   80.61777  81.62827  69.35460      542.66492      350.14337      454.42563          1
H   81.11771  82.17100  70.19253     1209.57979    -2396.95920      463.86744          1
C   81.36842  80.37797  68.91061     -737.14309      145.93010      242.71942          1
H   80.71213  79.81671  68.20339    -1050.18303      159.36419    -2509.06125          1
C   82.65333  80.73495  68.18866     -706.77696     -252.88343     -245.16230          1
H   83.18063  79.82082  67.79884    -1624.64039    -1665.49085     1417.86935          1
H   82.46040  81.41104  67.30955     2566.10521      598.51109      693.22305          1
H   83.37338  81.27215  68.86589    -1972.69758      107.10018    -1876.34312          1
C   81.65749  79.45539  70.07965      412.85148      672.58832      -27.69222          1
H   82.16212  78.50680  69.74664     1447.02277     1762.76533    -1490.78944          1
H   82.33114  79.94408  70.83634     1320.77379     -145.20455     2378.68491          1
H   80.71107  79.16374  70.61396    -1512.10848     1470.52031     1724.75147          1
C   80.31721  82.65121  68.06809     -607.11113      499.56689       73.73769          1
O   80.66855  83.79339  68.17254      357.97126      460.81534      356.68785          1
N   79.76503  82.01138  67.04597      536.55407     -368.62270      332.76306          1
H   79.49418  81.00967  67.19625      524.38866     2297.69985     1367.20941          1
C   78.94277  82.76052  66.14819       46.50277     -774.29190     -147.97904          1
H   78.38937  81.95505  65.61643     1676.51875     1883.29066     1032.55549          1
C   77.95706  83.73147  66.97650     -559.41456      -34.19198     -556.30433          1
H   78.56704  84.45839  67.56767      -64.66664      395.18769     2695.05373          1
H   77.33321  84.34581  66.28359    -2703.44278      -60.40056      333.79418          1
C   77.10355  82.88559  67.88273     -519.76435      377.31045      459.42366          1
H   77.73907  82.06091  68.29506    -1746.74323     1667.50644    -1261.73691          1
C   76.57511  83.71218  69.04813      570.95554       16.46261     -545.27700          1
H   75.93722  83.09873  69.73525     2391.58273       28.05277    -1305.07271          1
H   77.40960  84.14057  69.66123      175.75588      917.63458     2559.43867          1
H   75.94936  84.57090  68.69299    -1063.97851    -2155.34650     1283.00462          1
C   75.94121  82.26462  67.11962      193.76014        2.62946     -765.53141          1
H   75.30876  81.61981  67.78309      507.39394     -846.55087     2539.60089          1
H   75.27094  83.04728  66.67941     1337.57841     1818.19054     1526.01994          1
H   76.29787  81.61956  66.27595     1100.88635    -1172.91350    -2199.08909          1
C   79.86837  83.61667  65.15852     -549.21115      475.64486     -309.38218          1
O   79.47313  84.62083  64.62520      556.06106     -390.37755      -78.24250          1
O   81.10253  83.03341  64.77164     -572.27940     -310.19032     -209.75862          1
H   81.13914  82.29878  65.44502    -1380.08193     2212.14486      790.85944          1
```

Listing 2: C00002.xmolout

```
      276
  150.0000  150.0000  150.0000     0.0000     0.0000     0.0000***********    1.1710     4684
       0.2500     300.00    0.00    0.00    0.00   -1294.9636       0.0000     300.0000
H   61.22334  76.68362  69.85415    -2043.14101    -1757.64893      399.89339          1
N   61.61283  77.38906  70.47541      426.75047      490.45733     -334.42467          1
H   60.77473  77.83945  70.83632      442.25559    -1110.30854    -2448.52907          1
C   62.55780  78.25394  69.88593      415.32972     -666.39213       83.73172          1
H   63.20906  77.56971  69.30234      879.32219     2491.00447      667.35213          1
C   61.83823  79.35068  68.95132       31.35357      477.41777      628.23362          1
H   61.01258  79.85053  69.51305     1168.38489     2240.25337    -1019.71136          1
H   62.57375  80.15579  68.70867     -360.43770     2534.90498      931.67112          1
C   61.31679  78.71576  67.68877      463.18208      512.70242     -382.34388          1
H   60.57655  77.93104  67.96201     1725.29117     1863.52247     -987.07854          1
```

C	60.62069	79.76244	66.82667	710.42801	-144.27427	-313.15367	1
H	60.20629	79.31523	65.88648	364.20195	-1033.13725	2494.72524	1
H	59.76885	80.24793	67.36891	2567.61904	335.80679	847.48802	1
H	61.32650	80.57769	66.52137	-2587.99029	680.92867	-512.16411	1
C	62.43470	78.07005	66.88100	268.17616	741.64379	-40.42634	1
H	62.06577	77.69610	65.89125	1283.54314	-1945.37420	-1411.27812	1
H	63.26358	78.79522	66.67346	-1258.57796	1317.15249	-2026.02085	1
H	62.88526	77.19425	67.41433	-2502.84526	909.80597	575.92391	1
C	63.36044	79.04730	71.04790	619.36578	-458.64843	-172.09273	1
O	63.85778	80.13073	70.81440	-81.40433	433.87611	522.34594	1
N	63.47869	78.30939	72.15171	175.23996	-622.20190	341.55523	1
H	63.00235	77.40806	72.16680	-876.57256	-303.22879	-2561.90213	1
C	64.27440	78.79927	73.21833	-571.88214	-534.10783	-106.15033	1
H	64.55346	79.83097	72.89583	-2713.20841	-16.25800	-248.80809	1
C	63.41964	78.69872	74.57272	166.60354	288.16399	-716.09568	1
H	63.31345	77.60945	74.83950	-2097.85574	1636.78449	-586.18044	1
O	62.08672	79.23331	74.51794	-317.80580	-363.38227	484.43082	1
H	61.55186	78.42999	74.68197	1863.74748	-1451.84923	1357.29467	1
C	64.38005	79.39487	75.60595	503.80731	517.99032	318.51606	1
H	63.98128	79.32105	76.64430	640.61020	-2157.10410	-1536.29104	1
H	65.39626	78.93134	75.60677	1687.88455	465.60692	2087.56452	1
H	64.50664	80.47874	75.37788	-1293.07895	-1899.88581	-1463.57452	1
C	65.53302	77.77394	73.43731	-153.07550	-733.68902	248.70981	1
O	65.46514	77.01201	74.36667	186.15459	-480.78787	-449.34387	1
N	66.48937	77.88136	72.49857	-487.53139	543.18517	42.03850	1
C	67.78042	77.31179	72.70104	-699.35871	69.34393	360.10701	1
H	67.67921	76.53089	73.48957	2.81126	-221.99807	-2715.58072	1
C	68.14202	76.79405	71.24356	-507.46439	-592.59194	122.07761	1
H	69.23345	76.62541	71.10277	-1975.08116	525.87560	-1801.71556	1
H	67.63713	75.82900	71.01443	1588.27743	-1913.22100	1113.83566	1
C	67.57218	77.95530	70.36977	-613.62442	-457.55993	-194.14490	1
H	68.25319	78.85074	70.39032	1707.54637	-721.27992	1996.92523	1
H	67.43554	77.68392	69.29825	758.23357	2100.81767	1560.55011	1
C	66.20390	78.25352	71.11510	-352.41524	704.96076	49.21465	1
H	65.87998	79.32355	71.09825	1714.76886	-2070.42778	443.35792	1
H	65.34823	77.60855	70.80571	1224.15040	1801.50881	1636.97633	1
C	68.88370	78.47902	73.07452	566.85117	537.05831	117.62814	1
O	70.00329	78.14808	73.38001	-550.26779	-406.08867	4.21666	1
N	68.36295	79.69317	72.95767	566.34832	-425.77725	180.18749	1
H	67.37962	79.71306	72.69380	644.44502	2646.80550	-52.73430	1
C	69.16173	80.86973	72.84625	612.34801	-281.48662	411.56221	1
H	68.37081	81.64380	72.72645	1667.23498	-1490.75142	1556.16753	1
C	69.98475	81.05054	74.20373	-714.45746	-175.43074	286.98927	1
H	70.82589	80.30139	74.20748	-787.09484	1763.03619	-1922.46060	1
O	69.26810	80.81854	75.43197	-429.46514	516.51932	-128.40526	1
H	69.70037	79.99733	75.74220	1981.82785	436.16847	-1818.18182	1
C	70.52674	82.51985	74.04425	630.79189	-468.94448	76.03230	1
H	71.27368	82.76759	74.83258	-1775.71861	2062.30353	131.89849	1
H	71.01363	82.69768	73.05775	-1855.14608	88.94118	1993.53760	1
H	69.70020	83.26154	74.13368	-1504.82350	-780.94249	-2132.90992	1
C	70.03949	80.70139	71.48813	288.57638	618.62814	-397.00270	1
O	69.49100	80.46298	70.43630	321.70495	-185.26452	574.37218	1
N	71.33083	80.97352	71.68846	-447.05792	-446.53431	367.76425	1
H	71.71345	81.06685	72.64906	350.31316	1975.63644	-1843.31522	1
C	72.25035	80.34538	70.77862	-511.23061	374.03917	-471.51480	1
H	71.65858	79.64695	70.14272	2234.79579	-898.70890	-1273.45190	1
C	72.95581	81.52700	69.95325	529.53781	-159.34528	563.72604	1
H	73.48566	81.06157	69.07791	654.59388	2347.94251	1217.51488	1
H	72.12590	82.15553	69.53663	-1526.55661	2103.42026	817.87394	1
O	73.93473	82.32310	70.66847	-554.84278	14.12789	399.58769	1
H	73.32749	82.90652	71.16291	1297.06236	2061.84854	1220.68812	1
C	73.28505	79.68447	71.82481	-662.15484	-412.13544	123.62687	1

O	73.15684	79.98381	73.02628	-540.01537	-317.05804	274.91456	1
N	74.17687	78.90739	71.26565	-202.76299	-690.00100	-131.49145	1
H	74.09671	78.82558	70.25172	-702.62132	-2617.37191	281.70413	1
C	75.21957	78.22650	71.99872	-491.73914	576.34187	222.73509	1
H	75.79294	77.69588	71.20091	1977.17978	-1823.58850	-434.68928	1
C	76.03897	79.23514	72.79920	-649.50797	-328.66843	306.11224	1
H	75.35569	79.68153	73.55991	2075.07803	1452.16490	-1004.45904	1
C	77.17425	78.53444	73.52021	-615.18735	-489.99689	-70.96352	1
H	77.70882	79.21966	74.23386	-1226.32661	-1724.38906	-1716.47170	1
H	76.78881	77.65568	74.11288	2381.64273	-969.84735	900.46860	1
H	77.94285	78.13443	72.80271	2122.74991	1702.23946	-141.36228	1
C	76.55501	80.36619	71.93244	-501.86074	-479.61276	-376.42482	1
H	77.15315	81.10964	72.52798	993.79261	-2246.95401	-1177.81305	1
H	77.22018	79.98596	71.10861	42.71279	-626.15391	-2651.37258	1
H	75.70638	80.92872	71.44479	-2026.39012	-1590.33709	-887.82922	1
C	74.46021	77.18880	73.08090	-672.89153	382.57394	-156.34072	1
O	74.53077	77.34620	74.28976	410.04627	221.86223	500.35950	1
N	73.82516	76.28029	72.38695	-378.11782	-443.79414	-441.10984	1
H	73.95864	76.37514	71.38303	2404.15973	-568.14894	1149.30085	1
C	73.17517	75.16851	73.00514	-544.54484	466.16019	331.29119	1
H	72.71310	75.56770	73.93535	-1409.80988	-170.17955	2325.32679	1
C	72.15620	74.64849	71.90199	-184.48284	116.78282	-758.89137	1
H	71.44276	75.45712	71.62486	1893.78641	1861.14652	611.04528	1
H	72.70364	74.39577	70.96613	-2170.79665	-974.19311	1327.50117	1
C	71.39358	73.39905	72.43716	389.51668	672.81878	-138.49152	1
H	72.11387	72.64012	72.81612	510.64577	1933.03327	1851.02484	1
H	70.78655	73.67215	73.32797	-2302.30869	-1105.72939	-948.89774	1
C	70.46223	72.74099	71.34776	-559.93427	-126.34590	542.30857	1
O	69.93065	71.68734	71.58510	-670.36003	-111.83166	76.36448	1
N	70.33309	73.46366	70.21452	-216.06729	-537.07037	446.51379	1
H	70.74239	74.37151	69.96499	-1251.96487	1074.12894	-2168.52500	1
H	69.74347	73.18586	69.42209	-2451.83023	-720.73851	944.84612	1
C	74.32086	74.05363	73.27751	546.24052	-563.88337	-85.11809	1
O	75.07989	73.72518	72.37282	71.70702	-307.40463	606.69673	1
N	74.22248	73.55711	74.50237	479.90863	-504.50129	222.86849	1
C	75.26002	72.72780	75.06974	75.51268	-600.91904	506.73700	1
H	76.21230	73.20582	74.74133	-2033.61562	1795.30103	254.89480	1
C	74.94849	72.80770	76.62778	523.79067	517.99274	284.45581	1
H	75.34912	71.93128	77.19145	1599.61665	-1127.00911	1895.98173	1
H	75.39021	73.72301	77.08890	-1881.83093	1275.82488	-1501.55016	1
C	73.38917	72.88021	76.62129	-600.05639	-404.05026	316.64485	1
H	72.94386	71.88484	76.39584	-2597.05260	496.36221	657.73249	1
H	72.95039	73.21840	77.58686	-1666.24518	1294.38985	-1723.90591	1
C	73.15606	73.90787	75.43102	493.57570	-129.95376	-602.56394	1
H	72.17604	73.81663	74.90757	-1469.08415	-637.84797	2204.22581	1
H	73.33245	74.97531	75.70713	-1038.97840	1068.88701	-2280.71789	1
C	75.12228	71.21298	74.52252	188.88100	241.05089	-727.87821	1
O	74.85960	70.29945	75.27522	531.88613	-49.19943	427.07960	1
N	75.40167	71.17057	73.22364	708.14833	26.93417	-179.73509	1
H	75.55701	72.07708	72.71655	-2232.69501	-1179.09831	1023.94816	1
C	75.05557	69.95327	72.46245	-738.32412	259.92548	104.42456	1
H	74.07894	69.65777	72.91234	2028.89123	1260.02983	1311.33324	1
C	74.98825	70.28982	70.99017	-258.65678	-345.52513	661.28459	1
H	75.96244	70.74702	70.70299	-1826.06090	-1801.46097	-918.64554	1
C	74.78418	69.05263	70.13295	-541.23400	531.92970	-218.41428	1
H	74.76394	69.30575	69.03711	1311.73218	-2219.18200	882.19023	1
H	75.61106	68.30287	70.28608	-1048.71298	1223.58118	-2196.97971	1
H	73.81457	68.53589	70.37310	419.06414	1903.92898	1903.44670	1
C	73.89526	71.29700	70.67943	-423.86431	152.66566	-648.55262	1
H	73.86281	71.55202	69.58441	105.68386	-2342.50080	1387.51238	1
H	72.87730	70.90710	70.95900	-1695.63603	-1484.73621	-1531.02793	1
H	74.05104	72.26279	71.24456	-1853.57121	-487.39031	1936.59343	1

C	76.24023	68.84572	72.79943	539.71428	-510.58232	267.58633	1
O	77.10220	68.60723	71.99177	-15.93758	-278.34085	-624.49402	1
N	76.03058	68.31581	73.98914	-676.81886	12.05292	276.17960	1
H	75.27433	68.69947	74.59607	-1694.44250	-373.84189	-2100.66098	1
C	76.85421	67.25384	74.45990	412.33078	-664.70360	108.35451	1
H	76.21835	66.77262	75.23702	1765.77476	1294.44366	1621.76594	1
C	77.21182	66.29866	73.24588	-179.66635	-721.95667	-264.73915	1
H	76.31442	66.12219	72.61077	-607.34922	2587.87032	-598.10018	1
H	77.96955	66.77720	72.57444	-359.69332	-1479.13954	-2259.74260	1
C	77.76828	64.95473	73.82435	-240.01076	616.02425	431.85322	1
H	78.71416	65.13920	74.38781	-1359.17131	782.54425	2227.99181	1
H	77.05609	64.50658	74.55281	-820.36667	85.81767	-2596.78715	1
C	78.01218	63.97677	72.60548	-513.07015	236.18570	-551.87280	1
H	77.05591	63.75509	72.07810	1816.11944	-1183.11722	-1650.94321	1
H	78.67384	64.45081	71.84438	-1800.04429	-1361.53473	1526.34643	1
C	78.65211	62.67935	73.15135	726.08972	-253.19732	-179.64713	1
H	79.68063	62.80373	73.54508	1224.27271	-456.51798	-2390.90304	1
H	78.03005	62.12920	73.88522	1206.40339	-1150.93514	-2154.90344	1
N	78.80500	61.68984	71.94669	329.70796	-633.68968	-155.67358	1
H	79.26967	60.76286	72.17018	1348.31305	1938.76845	1359.00643	1
H	77.91262	61.36539	71.47214	2096.92035	1446.54551	-966.48890	1
H	79.38479	62.02004	71.12126	-1197.07217	-2303.53719	827.28733	1
C	78.22700	67.86154	75.10515	-378.01186	-556.17301	-413.96481	1
O	79.22343	67.17648	75.16118	-409.44433	-307.55053	-453.30833	1
N	78.03199	69.06793	75.63941	-429.02477	524.70884	274.08502	1
H	77.11636	69.49370	75.48328	1706.34263	-1425.96036	1574.38926	1
C	79.20626	69.82188	75.96485	-45.25864	-626.22952	478.93267	1
H	80.02762	69.28613	75.43305	1988.63164	570.90773	1772.87287	1
C	78.97143	71.30876	75.47312	551.77128	-20.49526	-564.54996	1
H	78.59326	71.31347	74.42573	1434.61535	-1753.10253	1513.99471	1
H	78.18327	71.81320	76.08343	-714.67738	1819.09770	1898.36494	1
C	80.33443	72.07084	75.58543	446.99263	-555.77305	338.97221	1
H	80.58657	72.26781	76.65386	1953.68466	1892.25957	-161.67766	1
H	81.16502	71.44628	75.18577	-403.03785	2688.07032	-188.43591	1
C	80.23118	73.42317	74.77876	567.86262	335.74681	434.04421	1
H	80.00445	73.22461	73.70616	-1080.02275	1568.26081	-1948.78900	1
H	79.38785	74.05238	75.15354	-919.54108	2111.34692	1456.13463	1
C	81.57289	74.19023	74.92323	195.81897	173.33862	-745.11536	1
H	81.77296	74.57413	75.94573	1405.67949	-2301.21089	-390.08113	1
H	82.46592	73.65802	74.53550	-940.37328	-1118.32446	2299.72133	1
N	81.41702	75.45327	74.04327	319.26422	656.36007	-42.02424	1
H	82.17970	76.18417	74.12376	1961.03633	619.91968	1787.09422	1
H	81.42425	75.28819	72.99684	854.14343	-2156.80752	1429.08711	1
H	80.45606	76.00467	74.23665	1237.02905	-1378.50857	-1998.28500	1
C	79.46612	69.87102	77.57360	532.53319	-268.57236	517.55729	1
O	80.48276	70.44572	77.97337	266.16714	-290.73804	-558.88002	1
N	78.51080	69.29273	78.26038	-226.25352	640.49678	-270.32839	1
H	77.78280	68.88954	77.67114	2007.69063	-1035.27229	1523.50231	1
C	78.41220	69.24255	79.67209	-540.76542	-573.33303	49.50000	1
H	77.59912	68.50647	79.85655	-2374.20950	-1036.76023	843.75774	1
C	79.81913	68.81076	80.30081	-77.09082	666.87422	-415.84136	1
H	80.60442	69.54356	79.95528	-985.81834	617.19825	2463.91927	1
O	80.29618	67.50488	79.93243	293.29899	-391.65602	-477.80926	1
H	81.05466	67.73817	79.35912	2528.72714	-681.76262	-751.27122	1
C	79.53570	69.04071	81.83048	252.47363	-605.14473	440.05134	1
H	80.46504	68.95019	82.44028	-2052.65063	1456.49051	-1043.51830	1
H	79.10416	70.05667	82.02103	-2152.98800	-1661.97574	-161.70652	1
H	78.80803	68.29389	82.22522	-473.12726	-1584.27339	2165.61725	1
C	78.02964	70.72448	80.26663	360.36307	488.46713	505.10056	1
O	77.15791	70.81919	81.09589	-200.08817	-528.03552	-385.82850	1
N	78.87982	71.64264	79.82701	-597.54095	-226.44775	355.20479	1
H	79.65029	71.37654	79.16862	2179.23655	-1300.70981	-991.33835	1

C	78.53251	73.00653	79.99920	-687.68921	297.39807	-249.45179	1
H	77.70988	72.95334	80.74995	-349.16256	-2467.37713	-1101.72831	1
C	79.84913	73.75399	80.52252	672.47345	361.54328	-201.62880	1
H	80.61707	73.80749	79.71628	1541.41502	582.18425	2169.97026	1
H	79.61336	74.80165	80.82031	-2534.67717	60.25153	997.72295	1
C	80.38520	72.95807	81.72053	574.26774	-389.43119	-377.02542	1
C	79.76104	73.10145	82.99601	688.82679	-287.33580	-257.96133	1
H	78.91963	73.80382	83.13695	331.14182	1470.38163	2269.80013	1
C	81.46037	72.03559	81.54160	-383.52382	-675.04808	144.24917	1
H	81.93431	71.91066	80.55115	4.09847	1517.59662	2262.86397	1
C	80.22722	72.32709	84.05766	-134.76730	428.45285	-649.50303	1
H	79.74301	72.43697	85.04373	-903.23433	1600.49020	2011.53387	1
C	81.89338	71.28454	82.63687	-335.53281	-615.00040	-364.39058	1
H	82.72557	70.57471	82.48754	1596.45398	-1369.33862	1732.02659	1
C	81.30026	71.39848	83.92815	585.48547	-340.24194	-406.23938	1
O	81.72861	70.63629	85.04621	-419.15649	-173.04408	-511.94141	1
H	82.47367	70.13941	84.63321	-1071.84350	1559.71507	-1960.13012	1
C	78.02645	73.53448	78.53827	291.41878	-183.13091	710.72246	1
O	77.27561	72.88695	77.83571	379.87695	523.47396	-222.23622	1
N	78.51208	74.74535	78.30252	564.30591	373.09380	277.24371	1
H	79.10575	75.16556	79.01497	-120.59931	-2436.07824	-1214.35098	1
C	77.99750	75.48793	77.19713	404.07514	-299.37480	608.84009	1
H	77.41676	74.76387	76.57735	-1071.07882	-841.69025	2359.66467	1
C	77.10216	76.62200	77.86620	-683.12807	68.71770	-390.13096	1
H	76.41305	76.17451	78.61629	2068.88918	1596.58590	770.89603	1
H	77.73989	77.33354	78.43692	1379.86429	2342.49779	-179.85638	1
C	76.30253	77.37084	76.76008	-176.39967	537.49466	550.97268	1
H	76.98175	77.86720	76.03324	-396.28051	1942.22168	1869.33314	1
H	75.66283	76.68083	76.16032	-94.44700	586.00678	-2659.20016	1
C	75.42951	78.43498	77.57066	-291.69381	-718.18079	-150.72929	1
H	74.73014	77.99024	78.32116	-2303.38776	1089.76242	964.62032	1
H	76.00143	79.25273	78.07772	884.16729	2568.80533	-207.74283	1
N	74.62355	79.00458	76.58748	-594.49522	-252.81478	342.29276	1
H	74.61583	78.51486	75.68478	-1735.17426	2084.05705	-263.71605	1
C	73.91705	80.04325	76.81551	595.23177	-271.52487	442.22352	1
N	73.96270	80.63183	78.08563	585.53813	-424.79750	-105.81303	1
H	74.47856	80.34245	78.91534	959.88797	1327.56823	-2177.11905	1
H	73.42120	81.44825	78.37074	1578.48647	16.27378	-2220.76233	1
N	73.19624	80.40124	75.68088	-452.17763	-390.33541	-421.51853	1
H	73.23344	79.96570	74.72494	470.26107	1974.25629	-1817.92087	1
H	72.57633	81.21909	75.66629	1856.67307	-1573.97321	-1224.35416	1
C	79.22997	76.20786	76.42370	-531.47271	-581.45780	-55.06206	1
O	79.21155	76.32977	75.19046	-243.00837	192.73507	609.52485	1
N	80.11442	76.64855	77.26731	343.49288	-644.25607	38.09164	1
H	79.95585	76.45900	78.25961	-1691.16043	-1535.97525	-1484.73099	1
C	81.32035	77.36285	76.90022	542.66492	350.14337	454.42563	1
H	81.57830	77.04463	75.86365	1209.57979	-2396.95920	463.86744	1
C	81.04840	78.86299	77.00928	-737.14309	145.93010	242.71942	1
H	80.81346	79.08652	78.07338	-1050.18303	159.36419	-2509.06125	1
C	82.25552	79.68473	76.60814	-706.77696	-252.88343	-245.16230	1
H	82.04909	80.78793	76.68766	-1624.64039	-1665.49085	1417.86935	1
H	83.16356	79.46890	77.26762	2566.10521	598.51109	693.22305	1
H	82.55953	79.48129	75.54413	-1972.69758	107.10018	-1876.34312	1
C	79.84369	79.27022	76.18137	412.85148	672.58832	-27.69222	1
H	79.62561	80.37024	76.26646	1447.02277	1762.76533	-1490.78944	1
H	79.99776	79.04003	75.09110	1320.77379	-145.20455	2378.68491	1
H	78.91988	78.71654	76.50465	-1512.10848	1470.52031	1724.75147	1
C	82.38328	76.87131	78.09163	-607.11113	499.56689	73.73769	1
O	81.99301	76.47216	79.16250	357.97126	460.81534	356.68785	1
N	83.62145	77.01016	77.64795	536.55407	-368.62270	332.76306	1
H	83.73942	77.40908	76.71833	524.38866	2297.69985	1367.20941	1
C	84.69226	76.88550	78.57078	46.50277	-774.29190	-147.97904	1

```
H   84.23989  76.37764  79.45400     1676.51875     1883.29066     1032.55549          1
C   85.88665  76.06015  77.85928     -559.41456      -34.19198     -556.30433          1
H   86.32539  76.67930  77.04119      -64.66664      395.18769     2695.05373          1
H   86.72057  75.86861  78.57541    -2703.44278      -60.40056      333.79418          1
C   85.33820  74.77298  77.30603     -519.76435      377.31045      459.42366          1
H   84.41895  75.00799  76.72264    -1746.74323     1667.50644    -1261.73691          1
C   86.34959  74.12242  76.37036      570.95554       16.46261     -545.27700          1
H   85.95281  73.17301  75.92714     2391.58273       28.05277    -1305.07271          1
H   86.62116  74.79876  75.51922      175.75588      917.63458     2559.43867          1
H   87.30005  73.86640  76.90546    -1063.97851    -2155.34650     1283.00462          1
C   84.96608  73.80413  78.42035      193.76014        2.62946     -765.53141          1
H   84.56312  72.84112  78.01313      507.39394     -846.55087     2539.60089          1
H   85.85124  73.55043  79.05890     1337.57841     1818.19054     1526.01994          1
H   84.17996  74.23297  79.09433     1100.88635    -1172.91350    -2199.08909          1
C   85.23943  78.36301  78.89670     -549.21115      475.64486     -309.38218          1
O   84.78817  79.36918  78.40157      556.06106     -390.37755      -78.24250          1
O   86.32829  78.43394  79.79217     -572.27940     -310.19032     -209.75862          1
H   86.39802  77.46408  80.02351    -1380.08193     2212.14486      790.85944          1
```

Listing 3: C00003.xmolout

```
       276
  150.0000  150.0000  150.0000     0.0000     0.0000     0.0000***********    2.7750    11100
       0.2500    300.00    0.00    0.00    0.00   -1294.6210      0.0000    300.0000
H   71.77912  76.43050  82.74316    -2043.14101    -1757.64893      399.89339          1
N   72.75468  76.32011  83.01133      426.75047      490.45733     -334.42467          1
H   73.02148  75.48974  82.49078      442.25559    -1110.30854    -2448.52907          1
C   73.56483  77.46236  82.83258      415.32972     -666.39213       83.73172          1
H   73.13756  78.22538  83.51705      879.32219     2491.00447      667.35213          1
C   75.09137  77.07199  83.16290       31.35357      477.41777      628.23362          1
H   75.41966  76.25962  82.47031     1168.38489     2240.25337    -1019.71136          1
H   75.76836  77.93790  82.96283     -360.43770     2534.90498      931.67112          1
C   75.19765  76.62076  84.59832      463.18208      512.70242     -382.34388          1
H   74.33912  75.94678  84.81867     1725.29117     1863.52247     -987.07854          1
C   76.48919  75.84276  84.81442      710.42801     -144.27427     -313.15367          1
H   76.59169  75.49677  85.87535      364.20195    -1033.13725     2494.72524          1
H   76.53957  74.93324  84.16136     2567.61904      335.80679      847.48802          1
H   77.38938  76.46811  84.58109    -2587.99029      680.92867     -512.16411          1
C   75.14559  77.80804  85.54967      268.17616      741.64379      -40.42634          1
H   75.23623  77.48602  86.61933     1283.54314    -1945.37420    -1411.27812          1
H   75.97759  78.53198  85.34971    -1258.57796     1317.15249    -2026.02085          1
H   74.18374  78.37486  85.45383    -2502.84526      909.80597      575.92391          1
C   73.50629  77.94797  81.28548      619.36578     -458.64843     -172.09273          1
O   73.70025  79.10643  81.00759      -81.40433      433.87611      522.34594          1
N   73.36478  76.90946  80.45897      175.23996     -622.20190      341.55523          1
H   73.11560  75.95588  80.77442     -876.57256     -303.22879    -2561.90213          1
C   73.03596  77.17891  79.09421     -571.88214     -534.10783     -106.15033          1
H   72.77501  78.25854  79.00565    -2713.20841      -16.25800     -248.80809          1
C   74.35268  76.77910  78.25164      166.60354      288.16399     -716.09568          1
H   74.89632  75.99848  78.85044    -2097.85574     1636.78449     -586.18044          1
O   75.29080  77.84593  78.06236     -317.80580     -363.38227      484.43082          1
H   76.09042  77.47512  78.50323     1863.74748    -1451.84923     1357.29467          1
C   73.81915  76.11113  76.93491      503.80731      517.99032      318.51606          1
H   74.65583  75.70599  76.31874      640.61020    -2157.10410    -1536.29104          1
H   73.11940  75.26497  77.14547     1687.88455      465.60692     2087.56452          1
H   73.26539  76.84192  76.29851    -1293.07895    -1899.88581    -1463.57452          1
C   71.82639  76.12004  78.92209     -153.07550     -733.68902      248.70981          1
O   71.81462  75.15474  79.65998      186.15459     -480.78787     -449.34387          1
N   71.01398  76.44598  77.91620     -487.53139      543.18517       42.03850          1
C   69.92385  75.59205  77.53171     -699.35871       69.34393      360.10701          1
H   69.31991  75.42302  78.45065        2.81126     -221.99807    -2715.58072          1
C   69.19019  76.41313  76.39836     -507.46439     -592.59194      122.07761          1
```

H	68.70934	75.75696	75.63656	-1975.08116	525.87560	-1801.71556	1
H	68.37770	77.04738	76.81912	1588.27743	-1913.22100	1113.83566	1
C	70.34532	77.28773	75.81522	-613.62442	-457.55993	-194.14490	1
H	70.98963	76.69043	75.13194	1707.54637	-721.27992	1996.92523	1
H	69.99083	78.16995	75.23640	758.23357	2100.81767	1560.55011	1
C	71.11997	77.67818	77.14722	-352.41524	704.96076	49.21465	1
H	72.19622	77.94411	77.01653	1714.76886	-2070.42778	443.35792	1
H	70.61483	78.46376	77.75807	1224.15040	1801.50881	1636.97633	1
C	70.58426	74.19629	76.96745	566.85117	537.05831	117.62814	1
O	71.42291	74.14408	76.10025	-550.26779	-406.08867	4.21666	1
N	69.94137	73.22292	77.59625	566.34832	-425.77725	180.18749	1
H	69.23502	73.47057	78.28826	644.44502	2646.80550	-52.73430	1
C	70.35109	71.87513	77.42525	612.34801	-281.48662	411.56221	1
H	71.17917	71.95883	76.68534	1667.23498	-1490.75142	1556.16753	1
C	70.89155	71.49863	78.89372	-714.45746	-175.43074	286.98927	1
H	70.30785	72.10747	79.63997	-787.09484	1763.03619	-1922.46060	1
O	72.25491	71.92687	79.11010	-429.46514	516.51932	-128.40526	1
H	72.10430	72.83649	79.45604	1981.82785	436.16847	-1818.18182	1
C	70.56304	69.98341	79.11749	630.79189	-468.94448	76.03230	1
H	70.99439	69.60747	80.07545	-1775.71861	2062.30353	131.89849	1
H	69.46041	69.80454	79.15589	-1855.14608	88.94118	1993.53760	1
H	70.96009	69.34137	78.29188	-1504.82350	-780.94249	-2132.90992	1
C	69.12498	70.97363	76.85106	288.57638	618.62814	-397.00270	1
O	69.20622	69.74631	76.88590	321.70495	-185.26452	574.37218	1
N	68.16264	71.71367	76.35279	-447.05792	-446.53431	367.76425	1
H	68.34165	72.71605	76.35154	350.31316	1975.63644	-1843.31522	1
C	67.01339	71.19116	75.69536	-511.23061	374.03917	-471.51480	1
H	66.72640	70.31074	76.31352	2234.79579	-898.70890	-1273.45190	1
C	65.97478	72.41034	75.67613	529.53781	-159.34528	563.72604	1
H	65.05717	72.03617	75.15308	654.59388	2347.94251	1217.51488	1
H	65.66767	72.64561	76.73048	-1526.55661	2103.42026	817.87394	1
O	66.47313	73.64464	75.08819	-554.84278	14.12789	399.58769	1
H	66.59340	73.33449	74.16665	1297.06236	2061.84854	1220.68812	1
C	67.42156	70.74871	74.18860	-662.15484	-412.13544	123.62687	1
O	66.96850	71.30507	73.22519	-540.01537	-317.05804	274.91456	1
N	68.31185	69.74449	74.26101	-202.76299	-690.00100	-131.49145	1
H	68.61972	69.49197	75.23567	-702.62132	-2617.37191	281.70413	1
C	68.28684	68.61954	73.30473	-491.73914	576.34187	222.73509	1
H	67.80407	67.79839	73.88208	1977.17978	-1823.58850	-434.68928	1
C	67.57000	68.94052	72.01937	-649.50797	-328.66843	306.11224	1
H	67.78089	70.00545	71.75247	2075.07803	1452.16490	-1004.45904	1
C	68.05217	68.06395	70.87505	-615.18735	-489.99689	-70.96352	1
H	67.51968	68.30137	69.91369	-1226.32661	-1724.38906	-1716.47170	1
H	69.15406	68.19532	70.68986	2381.64273	-969.84735	900.46860	1
H	67.88201	66.97364	71.09155	2122.74991	1702.23946	-141.36228	1
C	66.06456	68.78200	72.15492	-501.86074	-479.61276	-376.42482	1
H	65.53285	69.03665	71.19743	993.79261	-2246.95401	-1177.81305	1
H	65.77985	67.72814	72.42466	42.71279	-626.15391	-2651.37258	1
H	65.65013	69.45708	72.95492	-2026.39012	-1590.33709	-887.82922	1
C	69.89286	68.28072	73.05772	-672.89153	382.57394	-156.34072	1
O	70.31828	67.17166	73.28438	410.04627	221.86223	500.35950	1
N	70.53569	69.31977	72.54473	-378.11782	-443.79414	-441.10984	1
H	69.99725	70.18161	72.48390	2404.15973	-568.14894	1149.30085	1
C	71.94668	69.24327	72.36096	-544.54484	466.16019	331.29119	1
H	72.21085	68.19656	72.64059	-1409.80988	-170.17955	2325.32679	1
C	72.21456	69.59747	70.83097	-184.48284	116.78282	-758.89137	1
H	71.73657	68.84862	70.16034	1893.78641	1861.14652	611.04528	1
H	71.75455	70.57584	70.56937	-2170.79665	-974.19311	1327.50117	1
C	73.75806	69.64129	70.62615	389.51668	672.81878	-138.49152	1
H	74.21973	70.29173	71.40033	510.64577	1933.03327	1851.02484	1
H	74.20094	68.63782	70.81192	-2302.30869	-1105.72939	-948.89774	1
C	74.17685	70.16131	69.19708	-559.93427	-126.34590	542.30857	1

O	75.33440	70.40306	68.97417	-670.36003	-111.83166	76.36448	1
N	73.15137	70.27000	68.32650	-216.06729	-537.07037	446.51379	1
H	72.15468	70.06252	68.46100	-1251.96487	1074.12894	-2168.52500	1
H	73.25751	70.55922	67.34812	-2451.83023	-720.73851	944.84612	1
C	72.60422	70.39333	73.30180	546.24052	-563.88337	-85.11809	1
O	72.70719	71.53876	72.87822	71.70702	-307.40463	606.69673	1
N	72.92535	69.91699	74.49934	479.90863	-504.50129	222.86849	1
C	73.27882	70.81746	75.56127	75.51268	-600.91904	506.73700	1
H	72.73622	71.76884	75.35499	-2033.61562	1795.30103	254.89480	1
C	72.78865	69.97780	76.81384	523.79067	517.99274	284.45581	1
H	73.21897	70.34020	77.77860	1599.61665	-1127.00911	1895.98173	1
H	71.68056	70.02055	76.91708	-1881.83093	1275.82488	-1501.55016	1
C	73.23505	68.53763	76.41467	-600.05639	-404.05026	316.64485	1
H	74.34113	68.41896	76.50622	-2597.05260	496.36221	657.73249	1
H	72.76946	67.73418	77.02943	-1666.24518	1294.38985	-1723.90591	1
C	72.76570	68.52143	74.89805	493.57570	-129.95376	-602.56394	1
H	73.38535	67.88894	74.22024	-1469.08415	-637.84797	2204.22581	1
H	71.68430	68.28037	74.74450	-1038.97840	1068.88701	-2280.71789	1
C	74.90305	71.00006	75.56643	188.88100	241.05089	-727.87821	1
O	75.65064	70.14661	75.97628	531.88613	-49.19943	427.07960	1
N	75.22168	72.22720	75.11212	708.14833	26.93417	-179.73509	1
H	74.42148	72.73395	74.72968	-2232.69501	-1179.09831	1023.94816	1
C	76.58440	72.42496	74.59569	-738.32412	259.92548	104.42456	1
H	76.99592	71.38661	74.57060	2028.89123	1260.02983	1311.33324	1
C	76.48241	73.06755	73.22718	-258.65678	-345.52513	661.28459	1
H	75.95249	74.04053	73.33931	-1826.06090	-1801.46097	-918.64554	1
C	77.86101	73.35129	72.65774	-541.23400	531.92970	-218.41428	1
H	77.80468	73.89858	71.67627	1311.73218	-2219.18200	882.19023	1
H	78.45125	73.99520	73.37602	-1048.71298	1223.58118	-2196.97971	1
H	78.44910	72.40760	72.48712	419.06414	1903.92898	1903.44670	1
C	75.70011	72.21420	72.24698	-423.86431	152.66566	-648.55262	1
H	75.66103	72.68201	71.22573	105.68386	-2342.50080	1387.51238	1
H	76.17043	71.19983	72.12189	-1695.63603	-1484.73621	-1531.02793	1
H	74.62713	72.04825	72.58749	-1853.57121	-487.39031	1936.59343	1
C	77.42348	73.36377	75.67457	539.71428	-510.58232	267.58633	1
O	78.64637	73.43868	75.57804	-15.93758	-278.34085	-624.49402	1
N	76.62585	74.00756	76.51107	-676.81886	12.05292	276.17960	1
H	75.67742	73.72813	76.24299	-1694.44250	-373.84189	-2100.66098	1
C	76.91294	74.24577	77.88700	412.33078	-664.70360	108.35451	1
H	75.90389	74.44876	78.30552	1765.77476	1294.44366	1621.76594	1
C	77.57381	72.94549	78.50029	-179.66635	-721.95667	-264.73915	1
H	76.93559	72.04664	78.34290	-607.34922	2587.87032	-598.10018	1
H	78.54389	72.71909	78.00256	-359.69332	-1479.13954	-2259.74260	1
C	77.76868	73.27283	80.01869	-240.01076	616.02425	431.85322	1
H	78.33624	74.22710	80.12626	-1359.17131	782.54425	2227.99181	1
H	76.78572	73.45245	80.50869	-820.36667	85.81767	-2596.78715	1
C	78.54297	72.10221	80.74481	-513.07015	236.18570	-551.87280	1
H	77.92720	71.17441	80.78844	1816.11944	-1183.11722	-1650.94321	1
H	79.47154	71.82765	80.19326	-1800.04429	-1361.53473	1526.34643	1
C	78.87416	72.63224	82.16051	726.08972	-253.19732	-179.64713	1
H	79.59569	73.47351	82.17751	1224.27271	-456.51798	-2390.90304	1
H	77.98841	72.88465	82.77696	1206.40339	-1150.93514	-2154.90344	1
N	79.58982	71.48858	82.95322	329.70796	-633.68968	-155.67358	1
H	79.89498	71.73942	83.93796	1348.31305	1938.76845	1359.00643	1
H	79.03527	70.60036	83.12726	2096.92035	1446.54551	-966.48890	1
H	80.49340	71.10820	82.54635	-1197.07217	-2303.53719	827.28733	1
C	77.96962	75.46071	78.18913	-378.01186	-556.17301	-413.96481	1
O	77.74457	76.19358	79.12283	-409.44433	-307.55053	-453.30833	1
N	79.07300	75.35377	77.46420	-429.02477	524.70884	274.08502	1
H	79.11570	74.65426	76.67754	1706.34263	-1425.96036	1574.38926	1
C	79.99828	76.44883	77.51336	-45.25864	-626.22952	478.93267	1
H	79.84387	76.87936	78.53120	1988.63164	570.90773	1772.87287	1

C	81.41329	75.76615	77.29242	551.77128	-20.49526	-564.54996	1
H	81.58527	75.03045	78.10926	1434.61535	-1753.10253	1513.99471	1
H	81.39138	75.15990	76.36139	-714.67738	1819.09770	1898.36494	1
C	82.61664	76.76296	77.22908	446.99263	-555.77305	338.97221	1
H	82.59115	77.34617	76.27832	1953.68466	1892.25957	-161.67766	1
H	82.57920	77.49788	78.06526	-403.03785	2688.07032	-188.43591	1
C	83.95730	75.92585	77.28006	567.86262	335.74681	434.04421	1
H	84.07170	75.40237	78.25670	-1080.02275	1568.26081	-1948.78900	1
H	83.94982	75.12784	76.50499	-919.54108	2111.34692	1456.13463	1
C	85.12489	76.91412	77.02486	195.81897	173.33862	-745.11536	1
H	84.94563	77.62850	76.19554	1405.67949	-2301.21089	-390.08113	1
H	85.48243	77.45749	77.92340	-940.37328	-1118.32446	2299.72133	1
N	86.32928	76.07576	76.52136	319.26422	656.36007	-42.02424	1
H	87.21462	76.62762	76.32510	1961.03633	619.91968	1787.09422	1
H	86.70763	75.34180	77.18780	854.14343	-2156.80752	1429.08711	1
H	86.14128	75.52894	75.57743	1237.02905	-1378.50857	-1998.28500	1
C	79.53537	77.48110	76.35296	532.53319	-268.57236	517.55729	1
O	78.41266	77.42778	75.84487	266.16714	-290.73804	-558.88002	1
N	80.46823	78.38079	76.15435	-226.25352	640.49678	-270.32839	1
H	81.30565	78.34466	76.73150	2007.69063	-1035.27229	1523.50231	1
C	80.33337	79.33246	75.09544	-540.76542	-573.33303	49.50000	1
H	79.28048	79.66751	75.22793	-2374.20950	-1036.76023	843.75774	1
C	81.44047	80.45201	75.31765	-77.09082	666.87422	-415.84136	1
H	82.45192	80.00648	75.10982	-985.81834	617.19825	2463.91927	1
O	81.51627	81.00857	76.64602	293.29899	-391.65602	-477.80926	1
H	82.43765	80.79562	76.89168	2528.72714	-681.76262	-751.27122	1
C	81.07744	81.47223	74.17517	252.47363	-605.14473	440.05134	1
H	81.81108	82.31022	74.13188	-2052.65063	1456.49051	-1043.51830	1
H	81.06910	80.98800	73.16898	-2152.98800	-1661.97574	-161.70652	1
H	80.07085	81.92279	74.33599	-473.12726	-1584.27339	2165.61725	1
C	80.53538	78.56014	73.66226	360.36307	488.46713	505.10056	1
O	81.48861	78.76495	72.95359	-200.08817	-528.03552	-385.82850	1
N	79.47317	77.79608	73.45081	-597.54095	-226.44775	355.20479	1
H	78.73136	77.67637	74.19076	2179.23655	-1300.70981	-991.33835	1
C	79.49313	76.89726	72.35717	-687.68921	297.39807	-249.45179	1
H	80.11735	77.35365	71.55689	-349.16256	-2467.37713	-1101.72831	1
C	80.02610	75.49211	72.88208	672.47345	361.54328	-201.62880	1
H	79.42144	75.19024	73.77589	1541.41502	582.18425	2169.97026	1
H	79.82611	74.73065	72.09396	-2534.67717	60.25153	997.72295	1
C	81.52321	75.38681	73.25409	574.26774	-389.43119	-377.02542	1
C	82.56416	76.13539	72.63848	688.82679	-287.33580	-257.96133	1
H	82.33290	76.92179	71.89748	331.14182	1470.38163	2269.80013	1
C	81.83290	74.37632	74.20696	-383.52382	-675.04808	144.24917	1
H	81.02799	73.78327	74.67609	4.09847	1517.59662	2262.86397	1
C	83.89976	75.85254	72.99871	-134.76730	428.45285	-649.50303	1
H	84.71695	76.42222	72.51817	-903.23433	1600.49020	2011.53387	1
C	83.18638	74.13196	74.52545	-335.53281	-615.00040	-364.39058	1
H	83.42317	73.33943	75.25968	1596.45398	-1369.33862	1732.02659	1
C	84.24029	74.86233	73.94139	585.48547	-340.24194	-406.23938	1
O	85.69454	74.60185	74.28971	-419.15649	-173.04408	-511.94141	1
H	85.54043	73.77649	74.77144	-1071.84350	1559.71507	-1960.13012	1
C	77.90359	76.62228	72.02665	291.41878	-183.13091	710.72246	1
O	77.16190	76.18271	72.88178	379.87695	523.47396	-222.23622	1
N	77.62021	76.96257	70.77958	564.30591	373.09380	277.24371	1
H	78.38170	77.33131	70.21054	-120.59931	-2436.07824	-1214.35098	1
C	76.29496	76.94893	70.26773	404.07514	-299.37480	608.84009	1
H	76.44414	77.04686	69.16901	-1071.07882	-841.69025	2359.66467	1
C	75.60070	75.58260	70.68902	-683.12807	68.71770	-390.13096	1
H	76.25603	74.72189	70.42822	2068.88918	1596.58590	770.89603	1
H	75.47671	75.53873	71.80054	1379.86429	2342.49779	-179.85638	1
C	74.22087	75.46126	69.97453	-176.39967	537.49466	550.97268	1
H	73.55887	76.31350	70.25181	-396.28051	1942.22168	1869.33314	1

```
H    74.34964  75.53567  68.87213      -94.44700      586.00678    -2659.20016          1
C    73.52600  74.07520  70.37055     -291.69381     -718.18079     -150.72929          1
H    74.05603  73.16663  69.98514    -2303.38776     1089.76242      964.62032          1
H    73.28208  73.92375  71.45267      884.16729     2568.80533     -207.74283          1
N    72.33172  74.20934  69.64461     -594.49522     -252.81478      342.29276          1
H    72.36110  74.82416  68.84494    -1735.17426     2084.05705     -263.71605          1
C    71.23646  73.60473  69.88445      595.23177     -271.52487      442.22352          1
N    71.17140  72.73100  70.96618      585.53813     -424.79750     -105.81303          1
H    71.91856  72.49230  71.65960      959.88797     1327.56823    -2177.11905          1
H    70.31179  72.23805  71.23390     1578.48647       16.27378    -2220.76233          1
N    70.24878  73.95408  68.94655     -452.17763     -390.33541     -421.51853          1
H    70.27129  74.61184  68.16975      470.26107     1974.25629    -1817.92087          1
H    69.29416  73.59709  68.98420     1856.67307    -1573.97321    -1224.35416          1
C    75.37440  78.16946  70.84382     -531.47271     -581.45780      -55.06206          1
O    74.36124  78.44158  70.24867     -243.00837      192.73507      609.52485          1
N    75.86026  78.77183  71.94248      343.49288     -644.25607       38.09164          1
H    76.70075  78.31539  72.29519    -1691.16043    -1535.97525    -1484.73099          1
C    74.93519  79.44153  72.88481      542.66492      350.14337      454.42563          1
H    74.09615  78.72480  73.04289     1209.57979    -2396.95920      463.86744          1
C    75.72987  79.74981  74.13594     -737.14309      145.93010      242.71942          1
H    76.73833  80.10015  73.81857    -1050.18303      159.36419    -2509.06125          1
C    75.08650  80.86059  74.94693     -706.77696     -252.88343     -245.16230          1
H    75.69854  81.12523  75.85320    -1624.64039    -1665.49085     1417.86935          1
H    74.96647  81.79899  74.33642     2566.10521      598.51109      693.22305          1
H    74.06191  80.56980  75.30891    -1972.69758      107.10018    -1876.34312          1
C    75.92417  78.52312  75.00768      412.85148      672.58832      -27.69222          1
H    76.57518  78.74451  75.90216     1447.02277     1762.76533    -1490.78944          1
H    74.94576  78.14065  75.41000     1320.77379     -145.20455     2378.68491          1
H    76.41281  77.67335  74.44539    -1512.10848     1470.52031     1724.75147          1
C    74.43888  80.80427  72.11204     -607.11113      499.56689       73.73769          1
O    73.32089  81.21589  72.27664      357.97126      460.81534      356.68785          1
N    75.47077  81.39144  71.48159      536.55407     -368.62270      332.76306          1
H    76.34473  80.83736  71.48968      524.38866     2297.69985     1367.20941          1
C    75.16134  81.88074  70.15564       46.50277     -774.29190     -147.97904          1
H    74.23785  81.34333  69.84685     1676.51875     1883.29066     1032.55549          1
C    74.96080  83.47753  70.18908     -559.41456      -34.19198     -556.30433          1
H    75.89035  83.95177  70.58554      -64.66664      395.18769     2695.05373          1
H    74.83038  83.87715  69.15471    -2703.44278      -60.40056      333.79418          1
C    73.78053  83.84731  71.04959     -519.76435      377.31045      459.42366          1
H    73.90551  83.35758  72.04456    -1746.74323     1667.50644    -1261.73691          1
C    73.72194  85.35471  71.26084      570.95554       16.46261     -545.27700          1
H    72.85751  85.64646  71.91160     2391.58273       28.05277    -1305.07271          1
H    74.65212  85.73982  71.75276      175.75588      917.63458     2559.43867          1
H    73.60732  85.90370  70.29106    -1063.97851    -2155.34650     1283.00462          1
C    72.47428  83.36039  70.43803      193.76014        2.62946     -765.53141          1
H    71.58602  83.73228  71.01104      507.39394     -846.55087     2539.60089          1
H    72.35587  83.70387  69.37786     1337.57841     1818.19054     1526.01994          1
H    72.41866  82.23857  70.44163     1100.88635    -1172.91350    -2199.08909          1
C    76.45972  81.50748  69.31065     -549.21115      475.64486     -309.38218          1
O    77.40070  80.92064  69.79484      556.06106     -390.37755      -78.24250          1
O    76.48698  81.90403  67.95446     -572.27940     -310.19032     -209.75862          1
H    75.57769  82.30803  67.89199    -1380.08193     2212.14486      790.85944          1
```

Listing 4: C00004.xmolout

```
      276
  150.0000  150.0000  150.0000     0.0000     0.0000     0.0000***********    2.7750    11100
      0.2500     300.00    0.00    0.00    0.00   -1294.3757       0.0000     300.0000
H    77.81839  58.44812  77.61439    -2043.14101    -1757.64893      399.89339          1
N    77.51883  59.13719  76.92911      426.75047      490.45733     -334.42467          1
H    76.97371  58.57081  76.28335      442.25559    -1110.30854    -2448.52907          1
C    78.54976  59.90111  76.34119      415.32972     -666.39213       83.73172          1
```

H	79.17710	60.18057	77.21432	879.32219	2491.00447	667.35213	1
C	79.36550	59.03481	75.25830	31.35357	477.41777	628.23362	1
H	78.68825	58.76305	74.41335	1168.38489	2240.25337	-1019.71136	1
H	80.16368	59.67409	74.80997	-360.43770	2534.90498	931.67112	1
C	79.96424	57.79406	75.86951	463.18208	512.70242	-382.34388	1
H	79.13858	57.14407	76.23611	1725.29117	1863.52247	-987.07854	1
C	80.75676	57.02638	74.81798	710.42801	-144.27427	-313.15367	1
H	81.18865	56.07966	75.23369	364.20195	-1033.13725	2494.72524	1
H	80.11922	56.74502	73.94057	2567.61904	335.80679	847.48802	1
H	81.61198	57.63407	74.42394	-2587.99029	680.92867	-512.16411	1
C	80.87728	58.12211	77.04285	268.17616	741.64379	-40.42634	1
H	81.37357	57.20296	77.44898	1283.54314	-1945.37420	-1411.27812	1
H	81.69031	58.83382	76.74585	-1258.57796	1317.15249	-2026.02085	1
H	80.31540	58.59381	77.88956	-2502.84526	909.80597	575.92391	1
C	77.87242	61.17762	75.60520	619.36578	-458.64843	-172.09273	1
O	78.29060	61.58407	74.53370	-81.40433	433.87611	522.34594	1
N	76.88932	61.66940	76.34805	175.23996	-622.20190	341.55523	1
H	76.71099	61.19434	77.23248	-876.57256	-303.22879	-2561.90213	1
C	76.32441	62.93561	76.00838	-571.88214	-534.10783	-106.15033	1
H	76.23982	62.90828	74.90143	-2713.20841	-16.25800	-248.80809	1
C	74.93450	63.04420	76.78460	166.60354	288.16399	-716.09568	1
H	75.13228	62.96087	77.88838	-2097.85574	1636.78449	-586.18044	1
O	73.96214	62.02784	76.47574	-317.80580	-363.38227	484.43082	1
H	73.84909	61.59728	77.34580	1863.74748	-1451.84923	1357.29467	1
C	74.51615	64.52097	76.43976	503.80731	517.99032	318.51606	1
H	73.57639	64.81050	76.96457	640.61020	-2157.10410	-1536.29104	1
H	75.30313	65.25304	76.73689	1687.88455	465.60692	2087.56452	1
H	74.33559	64.65079	75.34759	-1293.07895	-1899.88581	-1463.57452	1
C	77.36041	64.08298	76.54361	-153.07550	-733.68902	248.70981	1
O	77.53546	64.20191	77.72656	186.15459	-480.78787	-449.34387	1
N	77.85726	64.82224	75.52690	-487.53139	543.18517	42.03850	1
C	78.92849	65.72637	75.72731	-699.35871	69.34393	360.10701	1
H	79.45616	65.41172	76.65429	2.81126	-221.99807	-2715.58072	1
C	79.73829	65.60984	74.38070	-507.46439	-592.59194	122.07761	1
H	80.40239	66.48510	74.19535	-1975.08116	525.87560	-1801.71556	1
H	80.38527	64.70358	74.36688	1588.27743	-1913.22100	1113.83566	1
C	78.56801	65.48261	73.34968	-613.62442	-457.55993	-194.14490	1
H	78.07293	66.46441	73.17692	1707.54637	-721.27992	1996.92523	1
H	78.88612	65.10302	72.35273	758.23357	2100.81767	1560.55011	1
C	77.61741	64.48123	74.12514	-352.41524	704.96076	49.21465	1
H	76.52876	64.59478	73.90785	1714.76886	-2070.42778	443.35792	1
H	77.91674	63.38368	74.05261	1224.15040	1801.50881	1636.97633	1
C	78.40042	67.32679	75.80556	566.85117	537.05831	117.62814	1
O	79.20709	68.25353	75.96055	-550.26779	-406.08867	4.21666	1
N	77.14163	67.43054	75.64919	566.34832	-425.77725	180.18749	1
H	76.66975	66.55495	75.52845	644.44502	2646.80550	-52.73430	1
C	75.51595	69.59666	76.06148	612.34801	-281.48662	411.56221	2
H	76.22583	68.77264	75.71585	1667.23498	-1490.75142	1556.16753	2
C	74.50374	68.97590	77.06757	-714.45746	-175.43074	286.98927	2
H	75.05304	68.60857	77.97237	-787.09484	1763.03619	-1922.46060	2
O	73.76548	67.82967	76.50625	-429.46514	516.51932	-128.40526	2
H	74.02671	67.13678	77.12877	1981.82785	436.16847	-1818.18182	2
C	73.61919	70.21436	77.45989	630.79189	-468.94448	76.03230	2
H	72.84715	69.93799	78.21536	-1775.71861	2062.30353	131.89849	2
H	74.23300	71.03908	77.89861	-1855.14608	88.94118	1993.53760	2
H	73.08478	70.63184	76.57502	-1504.82350	-780.94249	-2132.90992	2
C	76.35637	70.81232	76.64082	288.57638	618.62814	-397.00270	2
O	76.47110	71.06953	77.82175	321.70495	-185.26452	574.37218	2
N	76.89658	71.45599	75.59320	-447.05792	-446.53431	367.76425	2
H	76.79116	71.09529	74.64559	350.31316	1975.63644	-1843.31522	2
C	77.66713	72.63345	75.84380	-511.23061	374.03917	-471.51480	2
H	77.01578	73.17388	76.56681	2234.79579	-898.70890	-1273.45190	2

```
C   79.00544  72.12939  76.56780     529.53781    -159.34528     563.72604      2
H   79.49185  73.04194  77.00857     654.59388    2347.94251    1217.51488      2
H   78.68257  71.48557  77.43143   -1526.55661    2103.42026     817.87394      2
O   79.96690  71.44134  75.73393    -554.84278      14.12789     399.58769      2
H   79.66992  70.51174  75.86286    1297.06236    2061.84854    1220.68812      2
C   77.83024  73.38241  74.41884    -662.15484    -412.13544     123.62687      2
O   76.88657  73.49949  73.66287    -540.01537    -317.05804     274.91456      2
N   79.06534  73.85739  74.27301    -202.76299    -690.00100    -131.49145      2
H   79.72762  73.58563  75.00178    -702.62132   -2617.37191     281.70413      2
C   79.49695  74.64146  73.12359    -491.73914     576.34187     222.73509      2
H   80.60556  74.61285  73.24481    1977.17978   -1823.58850    -434.68928      2
C   79.03052  73.96689  71.84759    -649.50797    -328.66843     306.11224      2
H   77.91219  73.95466  71.84628    2075.07803    1452.16490   -1004.45904      2
C   79.52011  74.69173  70.60807    -615.18735    -489.99689     -70.96352      2
H   79.23572  74.13207  69.66657   -1226.32661   -1724.38906   -1716.47170      2
H   79.09585  75.72983  70.52354    2381.64273    -969.84735     900.46860      2
H   80.63970  74.79455  70.60749    2122.74991    1702.23946    -141.36228      2
C   79.48706  72.51820  71.79291    -501.86074    -479.61276    -376.42482      2
H   79.07680  71.98443  70.89260     993.79261   -2246.95401   -1177.81305      2
H   80.60753  72.43986  71.74766      42.71279    -626.15391   -2651.37258      2
H   79.15285  71.95014  72.70466   -2026.39012   -1590.33709    -887.82922      2
C   78.93393  76.18744  73.41685    -672.89153     382.57394    -156.34072      2
O   79.42767  76.80471  74.33329     410.04627     221.86223     500.35950      2
N   78.07191  76.60414  72.51109    -378.11782    -443.79414    -441.10984      2
H   77.73603  75.87813  71.88409    2404.15973    -568.14894    1149.30085      2
C   77.26823  77.78639  72.72993    -544.54484     466.16019     331.29119      2
H   76.48583  77.43757  73.44026   -1409.80988    -170.17955    2325.32679      2
C   78.17458  78.90920  73.37899    -184.48284     116.78282    -758.89137      2
H   78.45679  78.63496  74.42940    1893.78641    1861.14652     611.04528      2
H   79.14209  78.99491  72.83244   -2170.79665    -974.19311    1327.50117      2
C   77.39729  80.26407  73.37705     389.51668     672.81878    -138.49152      2
H   77.10035  80.53661  72.33987     510.64577    1933.03327    1851.02484      2
H   76.43000  80.15873  73.91516   -2302.30869   -1105.72939    -948.89774      2
C   78.23566  81.43376  74.01960    -559.93427    -126.34590     542.30857      2
O   77.71717  82.50626  74.19729    -670.36003    -111.83166      76.36448      2
N   79.51488  81.10888  74.30864    -216.06729    -537.07037     446.51379      2
H   80.01773  80.22587  74.16232   -1251.96487    1074.12894   -2168.52500      2
H   80.20565  81.77322  74.67367   -2451.83023    -720.73851     944.84612      2
C   76.62124  78.07911  71.28328     546.24052    -563.88337     -85.11809      2
O   75.51910  77.55843  71.09638      71.70702    -307.40463     606.69673      2
N   77.38548  78.70094  70.39557     479.90863    -504.50129     222.86849      2
C   76.86641  79.13409  69.12328      75.51268    -600.91904     506.73700      2
H   75.88583  79.61891  69.33236   -2033.61562    1795.30103     254.89480      2
C   78.00256  80.11112  68.60543     523.79067     517.99274     284.45581      2
H   77.98097  80.24781  67.50047    1599.61665   -1127.00911    1895.98173      2
H   77.90865  81.12433  69.05645   -1881.83093    1275.82488   -1501.55016      2
C   79.28491  79.38055  69.11494    -600.05639    -404.05026     316.64485      2
H   79.52949  78.50233  68.47699   -2597.05260     496.36221     657.73249      2
H   80.19092  80.02734  69.12682   -1666.24518    1294.38985   -1723.90591      2
C   78.81533  78.93685  70.56560     493.57570    -129.95376    -602.56394      2
H   79.27414  77.99900  70.95765   -1469.08415    -637.84797    2204.22581      2
H   78.90225  79.74442  71.33142   -1038.97840    1068.88701   -2280.71789      2
C   76.71628  77.89371  68.07888     188.88100     241.05089    -727.87821      2
O   76.58862  78.11676  66.90039     531.88613     -49.19943     427.07960      2
N   76.74601  76.70547  68.68527     708.14833      26.93417    -179.73509      2
H   76.87970  76.72241  69.69873   -2232.69501   -1179.09831    1023.94816      2
C   76.57871  75.45986  67.92629    -738.32412     259.92548     104.42456      2
H   75.98387  75.78467  67.03734    2028.89123    1260.02983    1311.33324      2
C   77.96703  74.98488  67.51832    -258.65678    -345.52513     661.28459      2
H   78.56216  74.86356  68.45724   -1826.06090   -1801.46097    -918.64554      2
C   77.94470  73.65584  66.78963    -541.23400     531.92970    -218.41428      2
H   78.98154  73.31040  66.52135    1311.73218   -2219.18200     882.19023      2
```

H	77.46892	72.84141	67.41448	-1048.71298	1223.58118	-2196.97971	2
H	77.35757	73.72092	65.83190	419.06414	1903.92898	1903.44670	2
C	78.67188	76.01389	66.65275	-423.86431	152.66566	-648.55262	2
H	79.76216	75.77366	66.51595	105.68386	-2342.50080	1387.51238	2
H	78.21042	76.07447	65.62867	-1695.63603	-1484.73621	-1531.02793	2
H	78.59959	77.04587	67.09882	-1853.57121	-487.39031	1936.59343	2
C	75.78423	74.39982	68.89814	539.71428	-510.58232	267.58633	2
O	75.81731	73.21836	68.63813	-15.93758	-278.34085	-624.49402	2
N	75.13176	74.97280	69.90216	-676.81886	12.05292	276.17960	2
H	75.14173	75.99448	70.02281	-1694.44250	-373.84189	-2100.66098	2
C	74.12613	74.11816	70.49267	412.33078	-664.70360	108.35451	2
H	73.68556	73.60081	69.61041	1765.77476	1294.44366	1621.76594	2
C	74.88813	73.08351	71.41018	-179.66635	-721.95667	-264.73915	2
H	75.67963	72.56064	70.82308	-607.34922	2587.87032	-598.10018	2
H	75.42705	73.58992	72.25955	-359.69332	-1479.13954	-2259.74260	2
C	73.81625	72.06834	71.94864	-240.01076	616.02425	431.85322	2
H	73.09856	72.58499	72.62849	-1359.17131	782.54425	2227.99181	2
H	73.20684	71.65953	71.11028	-820.36667	85.81767	-2596.78715	2
C	74.57010	70.91891	72.69138	-513.07015	236.18570	-551.87280	2
H	75.29087	70.40958	72.01504	1816.11944	-1183.11722	-1650.94321	2
H	75.18037	71.32917	73.52471	-1800.04429	-1361.53473	1526.34643	2
C	73.46916	69.88608	73.22807	726.08972	-253.19732	-179.64713	2
H	72.92267	70.25389	74.13193	1224.27271	-456.51798	-2390.90304	2
H	72.73568	69.58276	72.44015	1206.40339	-1150.93514	-2154.90344	2
N	74.15748	68.69003	73.61006	329.70796	-633.68968	-155.67358	2
H	73.91042	68.30087	74.52238	1348.31305	1938.76845	1359.00643	2
H	74.01617	67.89514	72.99197	2096.92035	1446.54551	-966.48890	2
H	74.99038	69.93007	75.14194	-1197.07217	-2303.53719	827.28733	2
C	73.03608	74.95469	71.37642	-378.01186	-556.17301	-413.96481	2
O	71.91070	74.51994	71.44304	-409.44433	-307.55053	-453.30833	2
N	73.58741	75.98863	71.98159	-429.02477	524.70884	274.08502	2
H	74.58175	76.23995	71.78861	1706.34263	-1425.96036	1574.38926	2
C	72.83818	76.90187	72.77157	-45.25864	-626.22952	478.93267	2
H	73.38580	77.86136	72.63483	1988.63164	570.90773	1772.87287	2
C	71.33241	76.94008	72.29356	551.77128	-20.49526	-564.54996	2
H	71.26780	77.11538	71.19518	1434.61535	-1753.10253	1513.99471	2
H	70.82362	75.95700	72.47750	-714.67738	1819.09770	1898.36494	2
C	70.64597	78.09095	73.10454	446.99263	-555.77305	338.97221	2
H	70.66938	77.86114	74.19396	1953.68466	1892.25957	-161.67766	2
H	71.20301	79.04640	72.98442	-403.03785	2688.07032	-188.43591	2
C	69.15755	78.25033	72.59852	567.86262	335.74681	434.04421	2
H	69.12838	78.67868	71.57001	-1080.02275	1568.26081	-1948.78900	2
H	68.64844	77.26126	72.53172	-919.54108	2111.34692	1456.13463	2
C	68.43687	79.17031	73.61295	195.81897	173.33862	-745.11536	2
H	68.19912	78.69044	74.58341	1405.67949	-2301.21089	-390.08113	2
H	68.93922	80.13978	73.78783	-940.37328	-1118.32446	2299.72133	2
N	67.05017	79.56218	73.00494	319.26422	656.36007	-42.02424	2
H	66.44024	80.18045	73.61432	1961.03633	619.91968	1787.09422	2
H	67.05242	80.10301	72.09102	854.14343	-2156.80752	1429.08711	2
H	66.38653	78.76385	72.78795	1237.02905	-1378.50857	-1998.28500	2
C	72.86251	76.40228	74.34475	532.53319	-268.57236	517.55729	2
O	72.53166	75.30645	74.72575	266.16714	-290.73804	-558.88002	2
N	73.19468	77.47158	75.05543	-226.25352	640.49678	-270.32839	2
H	73.40822	78.34966	74.58457	2007.69063	-1035.27229	1523.50231	2
C	73.24662	77.36895	76.46716	-540.76542	-573.33303	49.50000	2
H	72.85560	76.35743	76.73047	-2374.20950	-1036.76023	843.75774	2
C	74.79115	77.59417	76.86794	-77.09082	666.87422	-415.84136	2
H	75.12755	78.56999	76.42248	-985.81834	617.19825	2463.91927	2
O	75.70622	76.59699	76.38977	293.29899	-391.65602	-477.80926	2
H	76.27584	77.12829	75.79734	2528.72714	-681.76262	-751.27122	2
C	74.71549	77.74050	78.43001	252.47363	-605.14473	440.05134	2
H	75.71589	77.96568	78.86667	-2052.65063	1456.49051	-1043.51830	2

193	H	74.02627	78.56065	78.74150	-2152.98800	-1661.97574	-161.70652	2
194	H	74.35165	76.80176	78.90853	-473.12726	-1584.27339	2165.61725	2
195	C	72.39680	78.63601	77.04659	360.36307	488.46713	505.10056	2
196	O	72.45922	79.75167	76.53736	-200.08817	-528.03552	-385.82850	2
197	N	71.75839	78.26010	78.13389	-597.54095	-226.44775	355.20479	2
198	H	71.86322	77.29885	78.45178	2179.23655	-1300.70981	-991.33835	2
199	C	71.08439	79.25838	78.89473	-687.68921	297.39807	-249.45179	2
200	H	70.60028	79.89125	78.11631	-349.16256	-2467.37713	-1101.72831	2
201	C	70.13143	78.62209	80.00257	672.47345	361.54328	-201.62880	2
202	H	70.75825	78.19938	80.82495	1541.41502	582.18425	2169.97026	2
203	H	69.51477	79.43684	80.45271	-2534.67717	60.25153	997.72295	2
204	C	69.22927	77.53711	79.39411	574.26774	-389.43119	-377.02542	2
205	C	68.03093	77.92898	78.72660	688.82679	-287.33580	-257.96133	2
206	H	67.75474	78.99951	78.64105	331.14182	1470.38163	2269.80013	2
207	C	69.59264	76.16085	79.49832	-383.52382	-675.04808	144.24917	2
208	H	70.52502	75.86037	80.01014	4.09847	1517.59662	2262.86397	2
209	C	67.21672	76.93062	78.19144	-134.76730	428.45285	-649.50303	2
210	H	66.28384	77.22797	77.68144	-903.23433	1600.49020	2011.53387	2
211	C	68.74377	75.20014	78.94291	-335.53281	-615.00040	-364.39058	2
212	H	69.02974	74.13720	79.02954	1596.45398	-1369.33862	1732.02659	2
213	C	67.53165	75.54314	78.27569	585.48547	-340.24194	-406.23938	2
214	O	66.66166	74.57356	77.71164	-419.15649	-173.04408	-511.94141	2
215	H	67.15452	73.75177	77.94523	-1071.84350	1559.71507	-1960.13012	2
216	C	72.19233	80.07112	79.78057	291.41878	-183.13091	710.72246	2
217	O	72.24545	79.92888	80.97978	379.87695	523.47396	-222.23622	2
218	N	72.94800	80.81606	78.97768	564.30591	373.09380	277.24371	2
219	H	72.80656	80.76960	77.94205	-120.59931	-2436.07824	-1214.35098	2
220	C	73.94118	81.62199	79.59792	404.07514	-299.37480	608.84009	2
221	H	74.00829	81.26256	80.65340	-1071.07882	-841.69025	2359.66467	2
222	C	75.27444	81.46476	78.74849	-683.12807	68.71770	-390.13096	2
223	H	75.53389	80.39212	78.60625	2068.88918	1596.58590	770.89603	2
224	H	75.13487	81.87649	77.72371	1379.86429	2342.49779	-179.85638	2
225	C	76.40667	82.22208	79.50583	-176.39967	537.49466	550.97268	2
226	H	76.10189	83.27067	79.73345	-396.28051	1942.22168	1869.33314	2
227	H	76.61535	81.75093	80.49236	-94.44700	586.00678	-2659.20016	2
228	C	77.69163	82.18476	78.55693	-291.69381	-718.18079	-150.72929	2
229	H	78.08168	81.16637	78.30297	-2303.38776	1089.76242	964.62032	2
230	H	77.59968	82.76429	77.60261	884.16729	2568.80533	-207.74283	2
231	N	78.65099	82.82390	79.35547	-594.49522	-252.81478	342.29276	2
232	H	78.32951	83.22152	80.22462	-1735.17426	2084.05705	-263.71605	2
233	C	79.88068	82.93561	79.05202	595.23177	-271.52487	442.22352	2
234	N	80.29971	82.38189	77.83721	585.53813	-424.79750	-105.81303	2
235	H	79.75183	81.91445	77.11527	959.88797	1327.56823	-2177.11905	2
236	H	81.25328	82.41913	77.47642	1578.48647	16.27378	-2220.76233	2
237	N	80.58198	83.62135	80.05333	-452.17763	-390.33541	-421.51853	2
238	H	80.26609	84.02221	80.93537	470.26107	1974.25629	-1817.92087	2
239	H	81.58265	83.81621	80.00344	1856.67307	-1573.97321	-1224.35416	2
240	C	73.51274	83.20168	79.48615	-531.47271	-581.45780	-55.06206	2
241	O	73.99972	84.01420	80.23560	-243.00837	192.73507	609.52485	2
242	N	72.72827	83.42064	78.43377	343.49288	-644.25607	38.09164	2
243	H	72.48785	82.59204	77.89465	-1691.16043	-1535.97525	-1484.73099	2
244	C	72.05492	84.70896	78.23562	542.66492	350.14337	454.42563	2
245	H	71.90093	85.08209	79.27654	1209.57979	-2396.95920	463.86744	2
246	C	72.98445	85.61824	77.44661	-737.14309	145.93010	242.71942	2
247	H	73.15220	85.14828	76.45174	-1050.18303	159.36419	-2509.06125	2
248	C	72.37756	86.99022	77.22515	-706.77696	-252.88343	-245.16230	2
249	H	73.03287	87.63851	76.58026	-1624.64039	-1665.49085	1417.86935	2
250	H	71.36624	86.91447	76.71764	2566.10521	598.51109	693.22305	2
251	H	72.22170	87.53634	78.19617	-1972.69758	107.10018	-1876.34312	2
252	C	74.33785	85.76122	78.11978	412.85148	672.58832	-27.69222	2
253	H	75.03420	86.42247	77.53432	1447.02277	1762.76533	-1490.78944	2
254	H	74.23854	86.19995	79.15137	1320.77379	-145.20455	2378.68491	2

```
H    74.84180   84.76172   78.24534    -1512.10848     1470.52031     1724.75147          2
C    70.65087   84.39596   77.42692     -607.11113      499.56689       73.73769          2
O    70.13424   85.26606   76.75879      357.97126      460.81534      356.68785          2
N    70.19649   83.17845   77.66219      536.55407     -368.62270      332.76306          2
H    70.72694   82.57466   78.28721      524.38866     2297.69985     1367.20941          2
C    69.03043   82.74078   76.97027       46.50277     -774.29190     -147.97904          2
H    68.48966   83.67894   76.70259     1676.51875     1883.29066     1032.55549          2
C    69.46518   81.89294   75.69090     -559.41456      -34.19198     -556.30433          2
H    70.05736   81.00519   76.00652      -64.66664      395.18769     2695.05373          2
H    68.56433   81.49542   75.17463    -2703.44278      -60.40056      333.79418          2
C    70.31348   82.77114   74.71334     -519.76435      377.31045      459.42366          2
H    71.18532   83.20345   75.25223    -1746.74323     1667.50644    -1261.73691          2
C    70.79599   81.87244   73.51640      570.95554       16.46261     -545.27700          2
H    71.48070   82.43051   72.83657     2391.58273       28.05277    -1305.07271          2
H    71.34775   80.97418   73.87886      175.75588      917.63458     2559.43867          2
H    69.94123   81.51602   72.89637    -1063.97851    -2155.34650     1283.00462          2
C    69.41882   83.91200   74.10527      193.76014        2.62946     -765.53141          2
H    69.94958   84.45809   73.29090      507.39394     -846.55087     2539.60089          2
H    68.47278   83.50933   73.67399     1337.57841     1818.19054     1526.01994          2
H    69.14312   84.66816   74.88213     1100.88635    -1172.91350    -2199.08909          2
C    68.22474   81.70479   77.92184     -549.21115      475.64486     -309.38218          2
O    68.74920   81.05336   78.78778      556.06106     -390.37755      -78.24250          2
O    66.87903   81.46901   77.56241     -572.27940     -310.19032     -209.75862          2
H    66.78980   82.16604   76.85032    -1380.08193     2212.14486      790.85944          2
```

Listing 5: C00005.xmolout

```
       276
   150.0000   150.0000   150.0000     0.0000     0.0000     0.0000***********    2.7750    11100
       0.2500    300.00   0.00   0.00   0.00  -1294.3184      0.0000    300.0000
H    70.86210   62.77071   79.43633    -2043.14101    -1757.64893      399.89339          1
N    71.07610   62.99383   78.46748      426.75047      490.45733     -334.42467          1
H    70.22577   63.44856   78.14689      442.25559    -1110.30854    -2448.52907          1
C    72.28532   63.67621   78.23128      415.32972     -666.39213       83.73172          1
H    73.04240   63.13716   78.83873      879.32219     2491.00447      667.35213          1
C    72.59916   63.65851   76.64949       31.35357      477.41777      628.23362          1
H    71.69606   64.01455   76.09289     1168.38489     2240.25337    -1019.71136          1
H    73.40661   64.39551   76.42848     -360.43770     2534.90498      931.67112          1
C    72.99864   62.28180   76.18253      463.18208      512.70242     -382.34388          1
H    72.21796   61.55828   76.50831     1725.29117     1863.52247     -987.07854          1
C    73.08745   62.25901   74.66156      710.42801     -144.27427     -313.15367          1
H    73.29467   61.22877   74.27265      364.20195    -1033.13725     2494.72524          1
H    72.13594   62.61211   74.18524     2567.61904      335.80679      847.48802          1
H    73.90987   62.92355   74.29047    -2587.99029      680.92867     -512.16411          1
C    74.33816   61.86699   76.77479      268.17616      741.64379      -40.42634          1
H    74.67898   60.87729   76.37435     1283.54314    -1945.37420    -1411.27812          1
H    75.13994   62.61430   76.53882    -1258.57796     1317.15249    -2026.02085          1
H    74.29001   61.77416   77.89037    -2502.84526      909.80597      575.92391          1
C    72.19985   65.24717   78.60636      619.36578     -458.64843     -172.09273          1
O    73.17326   65.95857   78.34761      -81.40433      433.87611      522.34594          1
N    71.06168   65.56875   79.16943      175.23996     -622.20190      341.55523          1
H    70.41754   64.79796   79.33097     -876.57256     -303.22879    -2561.90213          1
C    70.60077   66.88582   79.46867     -571.88214     -534.10783     -106.15033          1
H    69.50353   66.70141   79.47127    -2713.20841      -16.25800     -248.80809          1
C    71.02945   67.20385   80.97300      166.60354      288.16399     -716.09568          1
H    72.14754   67.28646   81.05363    -2097.85574     1636.78449     -586.18044          1
O    70.67218   66.20091   81.94875     -317.80580     -363.38227      484.43082          1
H    71.55217   65.94316   82.28243     1863.74748    -1451.84923     1357.29467          1
C    70.35649   68.61040   81.17571      503.80731      517.99032      318.51606          1
H    70.65892   69.07113   82.14454      640.61020    -2157.10410    -1536.29104          1
H    70.62377   69.32638   80.36409     1687.88455      465.60692     2087.56452          1
H    69.24506   68.52650   81.18567    -1293.07895    -1899.88581    -1463.57452          1
```

C	70.98469	67.91199	78.25409	-153.07550	-733.68902	248.70981	1
O	70.21648	67.86483	77.32476	186.15459	-480.78787	-449.34387	1
N	72.07054	68.68472	78.38492	-487.53139	543.18517	42.03850	1
C	72.38031	69.64698	77.36258	-699.35871	69.34393	360.10701	1
H	71.45126	70.23712	77.19081	2.81126	-221.99807	-2715.58072	1
C	73.59337	70.46110	77.98629	-507.46439	-592.59194	122.07761	1
H	74.26505	70.90055	77.20876	-1975.08116	525.87560	-1801.71556	1
H	73.23619	71.30707	78.61467	1588.27743	-1913.22100	1113.83566	1
C	74.30541	69.37525	78.85067	-613.62442	-457.55993	-194.14490	1
H	74.92932	68.70463	78.21796	1707.54637	-721.27992	1996.92523	1
H	74.98140	69.79497	79.62905	758.23357	2100.81767	1560.55011	1
C	73.05065	68.60644	79.45504	-352.41524	704.96076	49.21465	1
H	73.22199	67.52138	79.68173	1714.76886	-2070.42778	443.35792	1
H	72.60086	69.11268	80.34131	1224.15040	1801.50881	1636.97633	1
C	72.86060	68.89699	75.99510	566.85117	537.05831	117.62814	1
O	72.79350	69.51431	74.95856	-550.26779	-406.08867	4.21666	1
N	73.40063	67.71250	76.23714	566.34832	-425.77725	180.18749	1
H	73.34834	67.29152	77.19346	644.44502	2646.80550	-52.73430	1
C	73.83366	66.89460	75.15332	612.34801	-281.48662	411.56221	1
H	74.18468	65.96765	75.65598	1667.23498	-1490.75142	1556.16753	1
C	72.51380	66.60001	74.30478	-714.45746	-175.43074	286.98927	1
H	72.10073	67.58799	73.95905	-787.09484	1763.03619	-1922.46060	1
O	71.43828	65.98607	75.06251	-429.46514	516.51932	-128.40526	1
H	71.05838	66.76765	75.50992	1981.82785	436.16847	-1818.18182	1
C	72.99311	65.75478	73.07572	630.79189	-468.94448	76.03230	1
H	72.13655	65.46983	72.42060	-1775.71861	2062.30353	131.89849	1
H	73.72882	66.31098	72.43942	-1855.14608	88.94118	1993.53760	1
H	73.48611	64.80989	73.40258	-1504.82350	-780.94249	-2132.90992	1
C	75.06223	67.61741	74.36100	288.57638	618.62814	-397.00270	1
O	75.44811	67.15937	73.30759	321.70495	-185.26452	574.37218	1
N	75.59261	68.61837	75.04631	-447.05792	-446.53431	367.76425	1
H	75.14022	68.93143	75.90470	350.31316	1975.63644	-1843.31522	1
C	76.60656	69.43379	74.45100	-511.23061	374.03917	-471.51480	1
H	77.39720	68.74048	74.08881	2234.79579	-898.70890	-1273.45190	1
C	75.90174	70.15715	73.20650	529.53781	-159.34528	563.72604	1
H	76.69970	70.66306	72.59728	654.59388	2347.94251	1217.51488	1
H	75.48973	69.32171	72.57580	-1526.55661	2103.42026	817.87394	1
O	74.88992	71.14482	73.50390	-554.84278	14.12789	399.58769	1
H	74.19663	70.56442	73.89281	1297.06236	2061.84854	1220.68812	1
C	77.06829	70.44893	75.62606	-662.15484	-412.13544	123.62687	1
O	76.36553	70.70117	76.57964	-540.01537	-317.05804	274.91456	1
N	78.26913	70.92031	75.31810	-202.76299	-690.00100	-131.49145	1
H	78.80715	70.68059	74.46415	-702.62132	-2617.37191	281.70413	1
C	79.01620	71.65614	76.29276	-491.73914	576.34187	222.73509	1
H	78.34144	72.41997	76.73949	1977.17978	-1823.58850	-434.68928	1
C	79.55413	70.63071	77.38321	-649.50797	-328.66843	306.11224	1
H	80.16064	69.83572	76.89891	2075.07803	1452.16490	-1004.45904	1
C	80.40194	71.40881	78.44513	-615.18735	-489.99689	-70.96352	1
H	80.75416	70.73913	79.26476	-1226.32661	-1724.38906	-1716.47170	1
H	81.30855	71.87725	77.99031	2381.64273	-969.84735	900.46860	1
H	79.81198	72.22912	78.91901	2122.74991	1702.23946	-141.36228	1
C	78.35791	69.99292	78.17257	-501.86074	-479.61276	-376.42482	1
H	78.70493	69.47267	79.09651	993.79261	-2246.95401	-1177.81305	1
H	77.61345	70.76595	78.48337	42.71279	-626.15391	-2651.37258	1
H	77.81582	69.23647	77.55497	-2026.39012	-1590.33709	-887.82922	1
C	80.20202	72.21071	75.32351	-672.89153	382.57394	-156.34072	1
O	80.37830	71.64601	74.25143	410.04627	221.86223	500.35950	1
N	80.87544	73.17429	75.90919	-378.11782	-443.79414	-441.10984	1
H	80.59463	73.45147	76.85730	2404.15973	-568.14894	1149.30085	1
C	81.86289	73.97760	75.25461	-544.54484	466.16019	331.29119	1
H	82.64671	74.11587	76.03105	-1409.80988	-170.17955	2325.32679	1
C	82.36554	73.24211	73.94250	-184.48284	116.78282	-758.89137	1

97	H	82.59985	72.17331	74.15845	1893.78641	1861.14652	611.04528	1
98	H	81.54526	73.22340	73.18479	-2170.79665	-974.19311	1327.50117	1
99	C	83.60903	73.99539	73.37558	389.51668	672.81878	-138.49152	1
100	H	83.39891	75.08511	73.31170	510.64577	1933.03327	1851.02484	1
101	H	84.46236	73.92056	74.08484	-2302.30869	-1105.72939	-948.89774	1
102	C	84.04920	73.46434	71.95834	-559.93427	-126.34590	542.30857	1
103	O	84.85750	74.08263	71.31341	-670.36003	-111.83166	76.36448	1
104	N	83.45848	72.30472	71.59919	-216.06729	-537.07037	446.51379	1
105	H	82.79668	71.72037	72.12345	-1251.96487	1074.12894	-2168.52500	1
106	H	83.67459	71.79342	70.73657	-2451.83023	-720.73851	944.84612	1
107	C	81.16524	75.37383	74.81371	546.24052	-563.88337	-85.11809	1
108	O	80.10241	75.37511	74.20540	71.70702	-307.40463	606.69673	1
109	N	81.93925	76.40707	75.13054	479.90863	-504.50129	222.86849	1
110	C	81.49130	77.76021	74.92130	75.51268	-600.91904	506.73700	1
111	H	80.42715	77.76618	75.25396	-2033.61562	1795.30103	254.89480	1
112	C	82.50316	78.60690	75.80314	523.79067	517.99274	284.45581	1
113	H	82.60425	79.65776	75.44911	1599.61665	-1127.00911	1895.98173	1
114	H	82.18376	78.65542	76.86831	-1881.83093	1275.82488	-1501.55016	1
115	C	83.81331	77.77619	75.63832	-600.05639	-404.05026	316.64485	1
116	H	84.26641	77.94424	74.63487	-2597.05260	496.36221	657.73249	1
117	H	84.59768	78.01766	76.39049	-1666.24518	1294.38985	-1723.90591	1
118	C	83.23922	76.30035	75.77914	493.57570	-129.95376	-602.56394	1
119	H	83.83994	75.51119	75.27316	-1469.08415	-637.84797	2204.22581	1
120	H	83.03902	75.98919	76.83151	-1038.97840	1068.88701	-2280.71789	1
121	C	81.63714	78.13509	73.34384	188.88100	241.05089	-727.87821	1
122	O	82.44485	78.93604	72.96072	531.88613	-49.19943	427.07960	1
123	N	80.72871	77.45834	72.62901	708.14833	26.93417	-179.73509	1
124	H	80.13820	76.72193	73.08921	-2232.69501	-1179.09831	1023.94816	1
125	C	80.68392	77.66865	71.18057	-738.32412	259.92548	104.42456	1
126	H	81.32411	78.56708	71.00143	2028.89123	1260.02983	1311.33324	1
127	C	81.20542	76.39550	70.52691	-258.65678	-345.52513	661.28459	1
128	H	80.67597	75.53756	70.99834	-1826.06090	-1801.46097	-918.64554	1
129	C	80.92114	76.37081	69.03785	-541.23400	531.92970	-218.41428	1
130	H	81.27214	75.41271	68.56252	1311.73218	-2219.18200	882.19023	1
131	H	79.81914	76.46704	68.82598	-1048.71298	1223.58118	-2196.97971	1
132	H	81.43649	77.21697	68.50357	419.06414	1903.92898	1903.44670	1
133	C	82.69063	76.21802	70.77814	-423.86431	152.66566	-648.55262	1
134	H	83.05911	75.20211	70.45333	105.68386	-2342.50080	1387.51238	1
135	H	83.30040	76.98535	70.22464	-1695.63603	-1484.73621	-1531.02793	1
136	H	82.93604	76.33275	71.87018	-1853.57121	-487.39031	1936.59343	1
137	C	79.08419	77.86198	70.79310	539.71428	-510.58232	267.58633	1
138	O	78.24441	77.07258	71.22662	-15.93758	-278.34085	-624.49402	1
139	N	78.95246	78.82319	69.92018	-676.81886	12.05292	276.17960	1
140	H	79.82906	79.29856	69.71724	-1694.44250	-373.84189	-2100.66098	1
141	C	77.69326	79.35592	69.52678	412.33078	-664.70360	108.35451	1
142	H	77.99106	80.08402	68.73941	1765.77476	1294.44366	1621.76594	1
143	C	76.80083	78.17400	68.97464	-179.66635	-721.95667	-264.73915	1
144	H	77.35289	77.59140	68.20348	-607.34922	2587.87032	-598.10018	1
145	H	76.57146	77.44037	69.78297	-359.69332	-1479.13954	-2259.74260	1
146	C	75.49551	78.79848	68.37603	-240.01076	616.02425	431.85322	1
147	H	74.98735	79.45088	69.12084	-1359.17131	782.54425	2227.99181	1
148	H	75.72887	79.45581	67.50906	-820.36667	85.81767	-2596.78715	1
149	C	74.55131	77.60632	67.94449	-513.07015	236.18570	-551.87280	1
150	H	75.00651	77.01905	67.11416	1816.11944	-1183.11722	-1650.94321	1
151	H	74.41115	76.88594	68.78320	-1800.04429	-1361.53473	1526.34643	1
152	C	73.19591	78.21678	67.51658	726.08972	-253.19732	-179.64713	1
153	H	72.61256	78.66771	68.34420	1224.27271	-456.51798	-2390.90304	1
154	H	73.25582	78.92537	66.66658	1206.40339	-1150.93514	-2154.90344	1
155	N	72.30384	77.04497	66.98724	329.70796	-633.68968	-155.67358	1
156	H	71.32289	77.30874	66.68097	1348.31305	1938.76845	1359.00643	1
157	H	72.65934	76.53703	66.12670	2096.92035	1446.54551	-966.48890	1
158	H	72.11053	76.24015	67.65204	-1197.07217	-2303.53719	827.28733	1

C	77.06716	80.13880	70.82838	-378.01186	-556.17301	-413.96481	1
O	77.62695	81.13683	71.22193	-409.44433	-307.55053	-453.30833	1
N	75.94350	79.59770	71.27830	-429.02477	524.70884	274.08502	1
H	75.59462	78.73278	70.86772	1706.34263	-1425.96036	1574.38926	1
C	75.29565	80.22496	72.37687	-45.25864	-626.22952	478.93267	1
H	75.80442	81.21370	72.48197	1988.63164	570.90773	1772.87287	1
C	73.74556	80.32271	72.04785	551.77128	-20.49526	-564.54996	1
H	73.57904	80.98497	71.16939	1434.61535	-1753.10253	1513.99471	1
H	73.32716	79.33298	71.75868	-714.67738	1819.09770	1898.36494	1
C	73.03338	80.88011	73.32352	446.99263	-555.77305	338.97221	1
H	72.77474	80.04868	74.02363	1953.68466	1892.25957	-161.67766	1
H	73.72451	81.54688	73.88925	-403.03785	2688.07032	-188.43591	1
C	71.73157	81.67114	72.89329	567.86262	335.74681	434.04421	1
H	71.97055	82.48040	72.16593	-1080.02275	1568.26081	-1948.78900	1
H	71.00241	81.00450	72.37814	-919.54108	2111.34692	1456.13463	1
C	71.13618	82.25329	74.19697	195.81897	173.33862	-745.11536	1
H	70.78209	81.48859	74.91649	1405.67949	-2301.21089	-390.08113	1
H	71.77812	82.99630	74.71213	-940.37328	-1118.32446	2299.72133	1
N	69.83347	83.03388	73.83172	319.26422	656.36007	-42.02424	1
H	69.35280	83.51462	74.64551	1961.03633	619.91968	1787.09422	1
H	69.87671	83.80784	73.10638	854.14343	-2156.80752	1429.08711	1
H	69.04493	82.42052	73.47692	1237.02905	-1378.50857	-1998.28500	1
C	75.43417	79.30537	73.73193	532.53319	-268.57236	517.55729	1
O	74.91530	79.71758	74.74297	266.16714	-290.73804	-558.88002	1
N	76.18686	78.22718	73.54553	-226.25352	640.49678	-270.32839	1
H	76.52603	78.10834	72.58874	2007.69063	-1035.27229	1523.50231	1
C	75.96040	77.05029	74.34748	-540.76542	-573.33303	49.50000	1
H	74.89739	76.76189	74.19923	-2374.20950	-1036.76023	843.75774	1
C	76.97644	75.99716	73.71186	-77.09082	666.87422	-415.84136	1
H	77.93480	76.54998	73.48155	-985.81834	617.19825	2463.91927	1
O	76.54903	75.48098	72.45697	293.29899	-391.65602	-477.80926	1
H	77.19016	75.99676	71.82971	2528.72714	-681.76262	-751.27122	1
C	77.27469	74.94937	74.84500	252.47363	-605.14473	440.05134	1
H	78.12562	74.28267	74.55740	-2052.65063	1456.49051	-1043.51830	1
H	77.55845	75.44262	75.80544	-2152.98800	-1661.97574	-161.70652	1
H	76.39219	74.29845	75.04901	-473.12726	-1584.27339	2165.61725	1
C	76.27795	77.41046	75.89473	360.36307	488.46713	505.10056	1
O	77.21159	78.11912	76.22190	-200.08817	-528.03552	-385.82850	1
N	75.49184	76.65615	76.64948	-597.54095	-226.44775	355.20479	1
H	74.83019	76.04124	76.17377	2179.23655	-1300.70981	-991.33835	1
C	75.28407	76.94128	78.02915	-687.68921	297.39807	-249.45179	1
H	75.03293	75.94538	78.45682	-349.16256	-2467.37713	-1101.72831	1
C	76.56079	77.61996	78.71584	672.47345	361.54328	-201.62880	1
H	76.80605	78.59323	78.21708	1541.41502	582.18425	2169.97026	1
H	76.30116	77.86486	79.77280	-2534.67717	60.25153	997.72295	1
C	77.77196	76.67185	78.68179	574.26774	-389.43119	-377.02542	1
C	77.75215	75.47687	79.45908	688.82679	-287.33580	-257.96133	1
H	76.87065	75.21813	80.07279	331.14182	1470.38163	2269.80013	1
C	78.92017	77.00678	77.90085	-383.52382	-675.04808	144.24917	1
H	78.95159	77.94482	77.30512	4.09847	1517.59662	2262.86397	1
C	78.87837	74.64705	79.44072	-134.76730	428.45285	-649.50303	1
H	78.86655	73.73266	80.05884	-903.23433	1600.49020	2011.53387	1
C	80.01883	76.13595	77.90797	-335.53281	-615.00040	-364.39058	1
H	80.89725	76.39996	77.29317	1596.45398	-1369.33862	1732.02659	1
C	80.04020	74.93824	78.67339	585.48547	-340.24194	-406.23938	1
O	81.17070	74.04961	78.68374	-419.15649	-173.04408	-511.94141	1
H	81.78853	74.58442	78.13683	-1071.84350	1559.71507	-1960.13012	1
C	74.04347	77.96772	78.20826	291.41878	-183.13091	710.72246	1
O	73.81633	78.42183	79.30475	379.87695	523.47396	-222.23622	1
N	73.33880	78.14440	77.09529	564.30591	373.09380	277.24371	1
H	73.57198	77.76454	76.15896	-120.59931	-2436.07824	-1214.35098	1
C	72.27202	79.10836	77.10113	404.07514	-299.37480	608.84009	1

```
H    71.66259   78.93265   78.01590    -1071.07882     -841.69025     2359.66467          1
C    72.98121   80.52762   77.13766     -683.12807       68.71770     -390.13096          1
H    73.80934   80.50839   77.88215     2068.88918     1596.58590      770.89603          1
H    73.47059   80.74384   76.15906     1379.86429     2342.49779     -179.85638          1
C    71.94438   81.62964   77.51568     -176.39967      537.49466      550.97268          1
H    71.12744   81.70029   76.76572     -396.28051     1942.22168     1869.33314          1
H    71.44195   81.39177   78.47940      -94.44700      586.00678    -2659.20016          1
C    72.76114   82.99938   77.62284     -291.69381     -718.18079     -150.72929          1
H    73.54785   83.03725   78.41863    -2303.38776     1089.76242      964.62032          1
H    73.22643   83.34637   76.66512      884.16729     2568.80533     -207.74283          1
N    71.76031   83.91913   77.96827     -594.49522     -252.81478      342.29276          1
H    70.80694   83.59989   77.89322    -1735.17426     2084.05705     -263.71605          1
C    71.97257   85.11109   78.35472      595.23177     -271.52487      442.22352          1
N    73.29968   85.54581   78.44330      585.53813     -424.79750     -105.81303          1
H    74.16276   85.04423   78.23801      959.88797     1327.56823    -2177.11905          1
H    73.58793   86.47859   78.74067     1578.48647       16.27378    -2220.76233          1
N    70.77014   85.78074   78.62309     -452.17763     -390.33541     -421.51853          1
H    69.80249   85.46551   78.57491      470.26107     1974.25629    -1817.92087          1
H    70.73280   86.74489   78.95647     1856.67307    -1573.97321    -1224.35416          1
C    71.57877   78.72731   75.68682     -531.47271     -581.45780      -55.06206          1
O    72.20245   78.03014   74.90083     -243.00837      192.73507      609.52485          1
N    70.37747   79.25339   75.57193      343.49288     -644.25607       38.09164          1
H    69.91748   79.85080   76.28405    -1691.16043    -1535.97525    -1484.73099          1
C    69.64181   79.10930   74.35211      542.66492      350.14337      454.42563          1
H    70.35859   79.26208   73.51577     1209.57979    -2396.95920      463.86744          1
C    69.01675   77.64620   74.29996     -737.14309      145.93010      242.71942          1
H    68.47916   77.41838   75.24503    -1050.18303      159.36419    -2509.06125          1
C    68.04735   77.52108   73.07709     -706.77696     -252.88343     -245.16230          1
H    67.72544   76.46524   72.91535    -1624.64039    -1665.49085     1417.86935          1
H    67.10897   78.12964   73.24182     2566.10521      598.51109      693.22305          1
H    68.52444   77.87046   72.13101    -1972.69758      107.10018    -1876.34312          1
C    70.16157   76.60144   74.05587      412.85148      672.58832      -27.69222          1
H    69.76569   75.63885   73.65365     1447.02277     1762.76533    -1490.78944          1
H    70.91736   76.98389   73.32719     1320.77379     -145.20455     2378.68491          1
H    70.70448   76.36233   75.00243    -1512.10848     1470.52031     1724.75147          1
C    68.54649   80.29091   74.56634     -607.11113      499.56689       73.73769          1
O    68.50603   80.86422   75.64646      357.97126      460.81534      356.68785          1
N    67.87042   80.53932   73.45211      536.55407     -368.62270      332.76306          1
H    68.04031   79.92418   72.65745      524.38866     2297.69985     1367.20941          1
C    66.66096   81.28354   73.53249       46.50277     -774.29190     -147.97904          1
H    66.88105   82.10053   74.25420     1676.51875     1883.29066     1032.55549          1
C    66.23089   81.75747   72.05261     -559.41456      -34.19198     -556.30433          1
H    65.99407   80.84979   71.44703      -64.66664      395.18769     2695.05373          1
H    65.28386   82.34776   72.09354    -2703.44278      -60.40056      333.79418          1
C    67.32652   82.54890   71.38589     -519.76435      377.31045      459.42366          1
H    68.29881   82.04690   71.58931    -1746.74323     1667.50644    -1261.73691          1
C    67.11311   82.57893   69.87743      570.95554       16.46261     -545.27700          1
H    67.92839   83.14223   69.35452     2391.58273       28.05277    -1305.07271          1
H    67.08862   81.54702   69.44107      175.75588      917.63458     2559.43867          1
H    66.14432   83.07406   69.60922    -1063.97851    -2155.34650     1283.00462          1
C    67.37960   83.97300   71.92165      193.76014        2.62946     -765.53141          1
H    68.23180   84.54943   71.47732      507.39394     -846.55087     2539.60089          1
H    66.44050   84.53628   71.68427     1337.57841     1818.19054     1526.01994          1
H    67.50500   83.99487   73.03464     1100.88635    -1172.91350    -2199.08909          1
C    65.49004   80.30857   74.05239     -549.21115      475.64486     -309.38218          1
O    65.49901   79.11028   73.89802      556.06106     -390.37755      -78.24250          1
O    64.34355   80.95569   74.56183     -572.27940     -310.19032     -209.75862          1
H    64.67618   81.89790   74.53791    -1380.08193     2212.14486      790.85944          1
```

Listing 6: C00006.xmolout

```
     276
```

```
   150.0000  150.0000  150.0000    0.0000    0.0000    0.0000***********   2.7750    11100
       0.2500    300.00   0.00   0.00   0.00  -1294.2957      0.0000    300.0000
H    68.71586  73.12670  87.63958    -2043.14101    -1757.64893       399.89339          1
N    69.69982  72.89536  87.76675      426.75047      490.45733      -334.42467          1
H    69.77453  71.97324  87.32632      442.25559    -1110.30854     -2448.52907          1
C    70.60064  73.82037  87.19173      415.32972     -666.39213        83.73172          1
H    70.06952  74.79272  87.09080      879.32219     2491.00447       667.35213          1
C    71.89726  73.91184  88.15399       31.35357      477.41777       628.23362          1
H    72.33084  72.89196  88.28333     1168.38489     2240.25337     -1019.71136          1
H    72.69809  74.53150  87.68561     -360.43770     2534.90498       931.67112          1
C    71.47677  74.48055  89.48159      463.18208      512.70242      -382.34388          1
H    70.56731  73.93188  89.81759     1725.29117     1863.52247      -987.07854          1
C    72.57305  74.27785  90.52052      710.42801     -144.27427      -313.15367          1
H    72.26330  74.65786  91.52802      364.20195    -1033.13725      2494.72524          1
H    72.83441  73.19478  90.64038     2567.61904      335.80679       847.48802          1
H    73.51364  74.81683  90.23775    -2587.99029      680.92867      -512.16411          1
C    71.14426  75.96270  89.37375      268.17616      741.64379       -40.42634          1
H    70.84191  76.39108  90.36411     1283.54314    -1945.37420     -1411.27812          1
H    72.01938  76.55961  89.00601    -1258.57796     1317.15249     -2026.02085          1
H    70.30037  76.14966  88.66134    -2502.84526      909.80597       575.92391          1
C    71.03218  73.14590  85.78698      619.36578     -458.64843      -172.09273          1
O    70.76081  71.96616  85.51313      -81.40433      433.87611       522.34594          1
N    71.62319  74.05010  85.04905      175.23996     -622.20190       341.55523          1
H    71.74939  74.95829  85.49496     -876.57256     -303.22879     -2561.90213          1
C    72.45813  73.70947  83.92647     -571.88214     -534.10783      -106.15033          1
H    73.42167  73.40215  84.38907    -2713.20841      -16.25800      -248.80809          1
C    71.80477  72.48761  83.12522      166.60354      288.16399      -716.09568          1
H    70.69119  72.53526  83.30584    -2097.85574     1636.78449      -586.18044          1
O    72.18951  71.19537  83.59389     -317.80580     -363.38227       484.43082          1
H    71.51958  71.09623  84.37667     1863.74748    -1451.84923      1357.29467          1
C    72.13108  72.80559  81.62241      503.80731      517.99032       318.51606          1
H    71.67047  72.05684  80.93679      640.61020    -2157.10410     -1536.29104          1
H    71.75966  73.81135  81.31500     1687.88455      465.60692      2087.56452          1
H    73.23054  72.79217  81.43657    -1293.07895    -1899.88581     -1463.57452          1
C    72.65052  75.07021  83.07933     -153.07550     -733.68902       248.70981          1
O    73.79297  75.26855  82.70007      186.15459     -480.78787      -449.34387          1
N    71.55040  75.77496  82.81250     -487.53139      543.18517        42.03850          1
C    71.59856  77.03954  82.13358     -699.35871       69.34393       360.10701          1
H    72.32924  76.91098  81.30223        2.81126     -221.99807     -2715.58072          1
C    70.09541  77.27115  81.69720     -507.46439     -592.59194       122.07761          1
H    69.85295  78.35086  81.56442    -1975.08116      525.87560     -1801.71556          1
H    69.86783  76.76995  80.72948     1588.27743    -1913.22100      1113.83566          1
C    69.32374  76.60408  82.88032     -613.62442     -457.55993      -194.14490          1
H    69.32964  77.25990  83.77968     1707.54637     -721.27992      1996.92523          1
H    68.25743  76.38111  82.65166      758.23357     2100.81767      1560.55011          1
C    70.20374  75.30106  83.10670     -352.41524      704.96076        49.21465          1
H    70.20349  74.88836  84.14460     1714.76886    -2070.42778       443.35792          1
H    69.99955  74.47534  82.38511     1224.15040     1801.50881      1636.97633          1
C    72.05888  78.21365  83.17870      566.85117      537.05831       117.62814          1
O    71.51446  79.29068  83.17675     -550.26779     -406.08867         4.21666          1
N    73.09589  77.79947  83.89728      566.34832     -425.77725       180.18749          1
H    73.48488  76.85668  83.73309      644.44502     2646.80550       -52.73430          1
C    73.62577  78.67076  84.88045      612.34801     -281.48662       411.56221          1
H    73.01604  79.60360  84.82139     1667.23498    -1490.75142      1556.16753          1
C    73.57367  77.91017  86.29081     -714.45746     -175.43074       286.98927          1
H    74.27422  77.03154  86.24003     -787.09484     1763.03619     -1922.46060          1
O    72.30028  77.37821  86.69044     -429.46514      516.51932      -128.40526          1
H    72.49218  76.41830  86.69993     1981.82785      436.16847     -1818.18182          1
C    74.17993  79.01905  87.22723      630.79189     -468.94448        76.03230          1
H    74.31587  78.64528  88.26822    -1775.71861     2062.30353       131.89849          1
H    75.17543  79.36782  86.86513    -1855.14608       88.94118      1993.53760          1
H    73.51429  79.91170  87.28245    -1504.82350     -780.94249     -2132.90992          1
```

```
C   75.21167  78.81865  84.46830     288.57638    618.62814   -397.00270    1
O   75.99092  77.88263  84.66973     321.70495   -185.26452    574.37218    1
N   75.41637  79.97875  83.90055    -447.05792   -446.53431    367.76425    1
H   74.58367  80.56654  83.85513     350.31316   1975.63644  -1843.31522    1
C   76.56429  80.36460  83.16319    -511.23061    374.03917   -471.51480    1
H   76.46498  81.47252  83.14001    2234.79579   -898.70890  -1273.45190    1
C   77.88146  79.86846  83.92281     529.53781   -159.34528    563.72604    1
H   78.73985  80.53102  83.61934     654.59388   2347.94251   1217.51488    1
H   77.69014  80.05238  85.01405   -1526.55661   2103.42026    817.87394    1
O   78.29853  78.52027  83.69052    -554.84278     14.12789    399.58769    1
H   77.50217  78.03164  84.13853    1297.06236   2061.84854   1220.68812    1
C   76.56242  79.76427  81.65030    -662.15484   -412.13544    123.62687    1
O   77.13987  80.35981  80.77361    -540.01537   -317.05804    274.91456    1
N   75.86630  78.59228  81.52475    -202.76299   -690.00100   -131.49145    1
H   75.57224  78.33417  82.47288    -702.62132  -2617.37191    281.70413    1
C   76.48512  77.52312  80.69611    -491.73914    576.34187    222.73509    1
H   77.56719  77.54306  80.96236    1977.17978  -1823.58850   -434.68928    1
C   75.78220  76.23934  81.05329    -649.50797   -328.66843    306.11224    1
H   74.73404  76.48683  81.37650    2075.07803   1452.16490  -1004.45904    1
C   75.67863  75.30147  79.86456    -615.18735   -489.99689    -70.96352    1
H   75.11535  74.36183  80.12232   -1226.32661  -1724.38906  -1716.47170    1
H   75.13428  75.79475  79.00558    2381.64273   -969.84735    900.46860    1
H   76.69245  74.99573  79.48380    2122.74991   1702.23946   -141.36228    1
C   76.46446  75.54184  82.21691    -501.86074   -479.61276   -376.42482    1
H   75.93978  74.58094  82.48629     993.79261  -2246.95401  -1177.81305    1
H   77.53495  75.28575  81.98120      42.71279   -626.15391  -2651.37258    1
H   76.46578  76.19156  83.14048   -2026.39012  -1590.33709   -887.82922    1
C   76.19777  77.89969  79.12383    -672.89153    382.57394   -156.34072    1
O   75.08336  78.05846  78.68718     410.04627    221.86223    500.35950    1
N   77.34136  77.80439  78.43389    -378.11782   -443.79414   -441.10984    1
H   78.19641  77.71263  78.98275    2404.15973   -568.14894   1149.30085    1
C   77.35545  78.56582  77.21362    -544.54484    466.16019    331.29119    1
H   76.34310  78.38692  76.77986   -1409.80988   -170.17955   2325.32679    1
C   77.58973  80.06872  77.65372    -184.48284    116.78282   -758.89137    1
H   76.91016  80.33684  78.49919    1893.78641   1861.14652    611.04528    1
H   78.62493  80.20971  78.04147   -2170.79665   -974.19311   1327.50117    1
C   77.33024  80.96341  76.40230     389.51668    672.81878   -138.49152    1
H   78.03543  80.69352  75.58403     510.64577   1933.03327   1851.02484    1
H   76.32602  80.74630  75.97533   -2302.30869  -1105.72939   -948.89774    1
C   77.44469  82.50245  76.71944    -559.93427   -126.34590    542.30857    1
O   77.30385  83.30136  75.82998    -670.36003   -111.83166     76.36448    1
N   77.68488  82.78496  78.01805    -216.06729   -537.07037    446.51379    1
H   77.79062  82.15480  78.82262   -1251.96487   1074.12894  -2168.52500    1
H   77.76732  83.73730  78.39011   -2451.83023   -720.73851    944.84612    1
C   78.61675  77.99217  76.34891     546.24052   -563.88337    -85.11809    1
O   79.72848  78.38085  76.58865      71.70702   -307.40463    606.69673    1
N   78.20882  77.04727  75.49055     479.90863   -504.50129    222.86849    1
C   79.09489  76.54048  74.48533      75.51268   -600.91904    506.73700    1
H   80.10972  76.47926  74.94209   -2033.61562   1795.30103    254.89480    1
C   78.42340  75.14860  74.11639     523.79067    517.99274    284.45581    1
H   78.68008  74.81464  73.07625    1599.61665  -1127.00911   1895.98173    1
H   78.75800  74.34017  74.80451   -1881.83093   1275.82488  -1501.55016    1
C   76.90461  75.45250  74.29814    -600.05639   -404.05026    316.64485    1
H   76.49932  75.99689  73.41736   -2597.05260    496.36221    657.73249    1
H   76.28518  74.53561  74.41795   -1666.24518   1294.38985  -1723.90591    1
C   76.91950  76.37856  75.58981     493.57570   -129.95376   -602.56394    1
H   76.11871  77.15520  75.63009   -1469.08415   -637.84797   2204.22581    1
H   76.93832  75.83096  76.56216   -1038.97840   1068.88701  -2280.71789    1
C   79.04961  77.54183  73.20031     188.88100    241.05089   -727.87821    1
O   78.13803  78.29830  72.95086     531.88613    -49.19943    427.07960    1
N   80.19079  77.36884  72.45844     708.14833     26.93417   -179.73509    1
H   80.60934  76.54684  72.90760   -2232.69501  -1179.09831   1023.94816    1
```

C	80.05306	77.41000	70.98864	-738.32412	259.92548	104.42456	1
H	79.58915	78.40132	70.78014	2028.89123	1260.02983	1311.33324	1
C	81.43396	77.23756	70.40035	-258.65678	-345.52513	661.28459	1
H	81.78867	76.21454	70.66060	-1826.06090	-1801.46097	-918.64554	1
C	81.42292	77.36787	68.88883	-541.23400	531.92970	-218.41428	1
H	82.44594	77.20477	68.45161	1311.73218	-2219.18200	882.19023	1
H	80.72102	76.61047	68.39736	-1048.71298	1223.58118	-2196.97971	1
H	81.08558	78.39050	68.56566	419.06414	1903.92898	1903.44670	1
C	82.42737	78.22903	70.98386	-423.86431	152.66566	-648.55262	1
H	83.47068	78.04785	70.60663	105.68386	-2342.50080	1387.51238	1
H	82.15588	79.28732	70.72006	-1695.63603	-1484.73621	-1531.02793	1
H	82.45759	78.16737	72.10606	-1853.57121	-487.39031	1936.59343	1
C	79.07390	76.14246	70.57935	539.71428	-510.58232	267.58633	1
O	79.14817	75.04977	71.10257	-15.93758	-278.34085	-624.49402	1
N	78.25717	76.53348	69.61231	-676.81886	12.05292	276.17960	1
H	78.42340	77.47101	69.25275	-1694.44250	-373.84189	-2100.66098	1
C	77.62779	75.50492	68.83324	412.33078	-664.70360	108.35451	1
H	77.12215	74.83478	69.56199	1765.77476	1294.44366	1621.76594	1
C	76.66526	76.24044	67.82389	-179.66635	-721.95667	-264.73915	1
H	75.92395	76.87005	68.36466	-607.34922	2587.87032	-598.10018	1
H	77.24053	76.94061	67.17713	-359.69332	-1479.13954	-2259.74260	1
C	75.94879	75.14716	66.96128	-240.01076	616.02425	431.85322	1
H	76.68780	74.50387	66.43044	-1359.17131	782.54425	2227.99181	1
H	75.34662	74.46010	67.60031	-820.36667	85.81767	-2596.78715	1
C	75.02700	75.92292	65.93814	-513.07015	236.18570	-551.87280	1
H	74.29301	76.56450	66.47806	1816.11944	-1183.11722	-1650.94321	1
H	75.62817	76.61398	65.30363	-1800.04429	-1361.53473	1526.34643	1
C	74.29819	74.86908	65.07044	726.08972	-253.19732	-179.64713	1
H	74.94787	74.33356	64.34807	1224.27271	-456.51798	-2390.90304	1
H	73.69454	74.12755	65.66100	1206.40339	-1150.93514	-2154.90344	1
N	73.25538	75.63389	64.19507	329.70796	-633.68968	-155.67358	1
H	72.65556	75.03376	63.55852	1348.31305	1938.76845	1359.00643	1
H	72.51637	76.18761	64.71756	2096.92035	1446.54551	-966.48890	1
H	73.63127	76.35516	63.51245	-1197.07217	-2303.53719	827.28733	1
C	78.79941	74.73692	67.98285	-378.01186	-556.17301	-413.96481	1
O	79.67195	75.31901	67.36789	-409.44433	-307.55053	-453.30833	1
N	78.54835	73.44221	68.04445	-429.02477	524.70884	274.08502	1
H	77.76783	73.10650	68.60627	1706.34263	-1425.96036	1574.38926	1
C	79.37709	72.54999	67.30901	-45.25864	-626.22952	478.93267	1
H	79.93545	73.20726	66.60192	1988.63164	570.90773	1772.87287	1
C	80.32083	71.83440	68.36132	551.77128	-20.49526	-564.54996	1
H	80.96341	72.58306	68.87646	1434.61535	-1753.10253	1513.99471	1
H	79.73155	71.35173	69.17229	-714.67738	1819.09770	1898.36494	1
C	81.18846	70.78703	67.58882	446.99263	-555.77305	338.97221	1
H	80.54474	70.02249	67.09553	1953.68466	1892.25957	-161.67766	1
H	81.76066	71.27939	66.77095	-403.03785	2688.07032	-188.43591	1
C	82.16946	70.11241	68.63031	567.86262	335.74681	434.04421	1
H	82.72727	70.88564	69.20675	-1080.02275	1568.26081	-1948.78900	1
H	81.60245	69.52182	69.38667	-919.54108	2111.34692	1456.13463	1
C	83.14911	69.21247	67.84194	195.81897	173.33862	-745.11536	1
H	82.68032	68.33656	67.35065	1405.67949	-2301.21089	-390.08113	1
H	83.79000	69.75362	67.11739	-940.37328	-1118.32446	2299.72133	1
N	84.15922	68.60147	68.87139	319.26422	656.36007	-42.02424	1
H	84.91161	67.97698	68.46002	1961.03633	619.91968	1787.09422	1
H	84.74276	69.28204	69.43985	854.14343	-2156.80752	1429.08711	1
H	83.75744	67.97352	69.62685	1237.02905	-1378.50857	-1998.28500	1
C	78.32445	71.51417	66.58105	532.53319	-268.57236	517.55729	1
O	77.89317	71.78097	65.48352	266.16714	-290.73804	-558.88002	1
N	78.01737	70.49629	67.37287	-226.25352	640.49678	-270.32839	1
H	78.43369	70.43554	68.30091	2007.69063	-1035.27229	1523.50231	1
C	77.02630	69.57470	66.94700	-540.76542	-573.33303	49.50000	1
H	76.82438	69.88052	65.89215	-2374.20950	-1036.76023	843.75774	1

C	77.62531	68.09792	67.09945	-77.09082	666.87422	-415.84136	1
H	77.59858	67.80863	68.18546	-985.81834	617.19825	2463.91927	1
O	78.98364	67.91099	66.66600	293.29899	-391.65602	-477.80926	1
H	79.40824	67.61581	67.49706	2528.72714	-681.76262	-751.27122	1
C	76.54646	67.27028	66.30888	252.47363	-605.14473	440.05134	1
H	76.74921	66.17572	66.36586	-2052.65063	1456.49051	-1043.51830	1
H	75.51663	67.44187	66.71121	-2152.98800	-1661.97574	-161.70652	1
H	76.53460	67.54609	65.22836	-473.12726	-1584.27339	2165.61725	1
C	75.74827	69.77619	67.96060	360.36307	488.46713	505.10056	1
O	75.08822	68.83325	68.33479	-200.08817	-528.03552	-385.82850	1
N	75.57095	71.06580	68.22115	-597.54095	-226.44775	355.20479	1
H	76.21110	71.72167	67.77399	2179.23655	-1300.70981	-991.33835	1
C	74.54227	71.46815	69.10256	-687.68921	297.39807	-249.45179	1
H	74.10295	70.52133	69.49237	-349.16256	-2467.37713	-1101.72831	1
C	75.17994	72.41861	70.23035	672.47345	361.54328	-201.62880	1
H	75.50926	73.38824	69.79569	1541.41502	582.18425	2169.97026	1
H	74.40444	72.67213	70.99173	-2534.67717	60.25153	997.72295	1
C	76.36879	71.68707	70.87043	574.26774	-389.43119	-377.02542	1
C	76.19809	70.33203	71.28926	688.82679	-287.33580	-257.96133	1
H	75.21144	69.84031	71.21535	331.14182	1470.38163	2269.80013	1
C	77.64343	72.31768	71.01321	-383.52382	-675.04808	144.24917	1
H	77.79593	73.38514	70.75107	4.09847	1517.59662	2262.86397	1
C	77.30274	69.62111	71.75689	-134.76730	428.45285	-649.50303	1
H	77.16349	68.56856	72.06016	-903.23433	1600.49020	2011.53387	1
C	78.72068	71.56445	71.48463	-335.53281	-615.00040	-364.39058	1
H	79.70106	72.06524	71.56763	1596.45398	-1369.33862	1732.02659	1
C	78.60445	70.19295	71.85571	585.48547	-340.24194	-406.23938	1
O	79.70278	69.41886	72.31173	-419.15649	-173.04408	-511.94141	1
H	80.40724	70.10975	72.30402	-1071.84350	1559.71507	-1960.13012	1
C	73.50095	72.37920	68.23862	291.41878	-183.13091	710.72246	1
O	73.77968	72.88982	67.16807	379.87695	523.47396	-222.23622	1
N	72.38246	72.50718	68.94155	564.30591	373.09380	277.24371	1
H	72.33871	72.02478	69.83805	-120.59931	-2436.07824	-1214.35098	1
C	71.25044	73.06014	68.28645	404.07514	-299.37480	608.84009	1
H	71.59697	73.33685	67.26242	-1071.07882	-841.69025	2359.66467	1
C	70.13826	71.92799	68.33635	-683.12807	68.71770	-390.13096	1
H	70.48401	71.00754	67.81492	2068.88918	1596.58590	770.89603	1
H	69.95249	71.61890	69.38925	1379.86429	2342.49779	-179.85638	1
C	68.83107	72.48280	67.69487	-176.39967	537.49466	550.97268	1
H	68.59721	73.49911	68.08546	-396.28051	1942.22168	1869.33314	1
H	68.93438	72.59914	66.59317	-94.44700	586.00678	-2659.20016	1
C	67.67722	71.44316	68.07460	-291.69381	-718.18079	-150.72929	1
H	67.79780	70.40311	67.67893	-2303.38776	1089.76242	964.62032	1
H	67.45524	71.37692	69.17064	884.16729	2568.80533	-207.74283	1
N	66.56728	72.02895	67.44977	-594.49522	-252.81478	342.29276	1
H	66.66763	72.99017	67.16113	-1735.17426	2084.05705	-263.71605	1
C	65.45568	71.44627	67.24373	595.23177	-271.52487	442.22352	1
N	65.32259	70.11941	67.66526	585.53813	-424.79750	-105.81303	1
H	65.99417	69.52106	68.14458	959.88797	1327.56823	-2177.11905	1
H	64.47912	69.55457	67.56072	1578.48647	16.27378	-2220.76233	1
N	64.55361	72.30946	66.60676	-452.17763	-390.33541	-421.51853	1
H	64.65790	73.27538	66.29960	470.26107	1974.25629	-1817.92087	1
H	63.60531	72.03521	66.34755	1856.67307	-1573.97321	-1224.35416	1
C	70.65334	74.32966	69.14013	-531.47271	-581.45780	-55.06206	1
O	70.29033	75.34285	68.59457	-243.00837	192.73507	609.52485	1
N	70.39534	73.97044	70.44360	343.49288	-644.25607	38.09164	1
H	71.00377	73.15141	70.55548	-1691.16043	-1535.97525	-1484.73099	1
C	70.40512	75.03991	71.45849	542.66492	350.14337	454.42563	1
H	70.18657	75.97553	70.89186	1209.57979	-2396.95920	463.86744	1
C	69.33040	74.66236	72.45309	-737.14309	145.93010	242.71942	1
H	69.64830	73.72188	72.95693	-1050.18303	159.36419	-2509.06125	1
C	69.12892	75.72703	73.51629	-706.77696	-252.88343	-245.16230	1

```
H    68.32395  75.43742  74.24645    -1624.64039    -1665.49085     1417.86935          1
H    70.06865  75.90284  74.11143     2566.10521      598.51109      693.22305          1
H    68.82944  76.71293  73.06529    -1972.69758      107.10018    -1876.34312          1
C    68.00735  74.38344  71.75860      412.85148      672.58832      -27.69222          1
H    67.21934  74.03304  72.47942     1447.02277     1762.76533    -1490.78944          1
H    67.60936  75.30216  71.24751     1320.77379     -145.20455     2378.68491          1
H    68.11908  73.59176  70.96764    -1512.10848     1470.52031     1724.75147          1
C    71.92503  74.99661  72.10841     -607.11113      499.56689       73.73769          1
O    72.61721  74.00806  72.05694      357.97126      460.81534      356.68785          1
N    72.20331  76.17182  72.66217      536.55407     -368.62270      332.76306          1
H    71.46934  76.87776  72.66429      524.38866     2297.69985     1367.20941          1
C    73.35139  76.25739  73.48879       46.50277     -774.29190     -147.97904          1
H    74.01804  75.44119  73.12892     1676.51875     1883.29066     1032.55549          1
C    73.96648  77.74533  73.39216     -559.41456      -34.19198     -556.30433          1
H    73.23063  78.46528  73.82459      -64.66664      395.18769     2695.05373          1
H    74.88434  77.83567  74.02069    -2703.44278      -60.40056      333.79418          1
C    74.25913  78.10101  71.95752     -519.76435      377.31045      459.42366          1
H    73.38906  77.78636  71.33756    -1746.74323     1667.50644    -1261.73691          1
C    74.43462  79.60862  71.81848      570.95554       16.46261     -545.27700          1
H    74.63123  79.90758  70.75683     2391.58273       28.05277    -1305.07271          1
H    73.52318  80.16589  72.15715      175.75588      917.63458     2559.43867          1
H    75.29659  79.97932  72.43178    -1063.97851    -2155.34650     1283.00462          1
C    75.50435  77.39561  71.44224      193.76014        2.62946     -765.53141          1
H    75.69689  77.63329  70.36452      507.39394     -846.55087     2539.60089          1
H    76.42983  77.70778  72.01596     1337.57841     1818.19054     1526.01994          1
H    75.41516  76.28124  71.52176     1100.88635    -1172.91350    -2199.08909          1
C    72.87435  76.05752  75.02179     -549.21115      475.64486     -309.38218          1
O    71.80371  76.41503  75.43990      556.06106     -390.37755      -78.24250          1
O    73.86483  75.57257  75.90693     -572.27940     -310.19032     -209.75862          1
H    74.59200  75.40628  75.24407    -1380.08193     2212.14486      790.85944          1
```

Listing 7: C00007.xmolout

```
        276
  150.0000  150.0000  150.0000     0.0000     0.0000     0.0000***********    2.7750    11100
        0.2500    300.00    0.00    0.00    0.00   -1294.2954       0.0000    300.0000
H    75.03646  64.16014  76.53732    -2043.14101    -1757.64893      399.89339          1
N    75.65288  64.16294  75.72699      426.75047      490.45733     -334.42467          1
H    74.98111  64.17967  74.96192      442.25559    -1110.30854    -2448.52907          1
C    76.60036  65.20996  75.71036      415.32972     -666.39213       83.73172          1
H    76.75542  65.47264  76.77773      879.32219     2491.00447      667.35213          1
C    77.93731  64.66330  74.99741       31.35357      477.41777      628.23362          1
H    77.70598  64.33986  73.95420     1168.38489     2240.25337    -1019.71136          1
H    78.69318  65.48011  74.90317     -360.43770     2534.90498      931.67112          1
C    78.48518  63.50581  75.79367      463.18208      512.70242     -382.34388          1
H    77.63470  62.84301  76.07197     1725.29117     1863.52247     -987.07854          1
C    79.46837  62.69952  74.95479      710.42801     -144.27427     -313.15367          1
H    79.88193  61.82799  75.52524      364.20195    -1033.13725     2494.72524          1
H    78.98620  62.29184  74.02889     2567.61904      335.80679      847.48802          1
H    80.33912  63.32437  74.62737    -2587.99029      680.92867     -512.16411          1
C    79.17220  63.98411  77.06514      268.17616      741.64379      -40.42634          1
H    79.58707  63.12807  77.65784     1283.54314    -1945.37420    -1411.27812          1
H    80.02619  64.67318  76.83690    -1258.57796     1317.15249    -2026.02085          1
H    78.46729  64.54055  77.73501    -2502.84526      909.80597      575.92391          1
C    75.97326  66.43891  74.83731      619.36578     -458.64843     -172.09273          1
O    75.78362  67.53800  75.32794      -81.40433      433.87611      522.34594          1
N    75.74928  66.05285  73.59013      175.23996     -622.20190      341.55523          1
H    75.91348  65.08106  73.33601     -876.57256     -303.22879    -2561.90213          1
C    75.20402  67.01206  72.68867     -571.88214     -534.10783     -106.15033          1
H    75.57714  67.98673  73.07565    -2713.20841      -16.25800     -248.80809          1
C    75.66815  66.62984  71.20686      166.60354      288.16399     -716.09568          1
H    75.19304  65.64902  70.92897    -2097.85574     1636.78449     -586.18044          1
```

O	77.08188	66.45913	71.01179	-317.80580	-363.38227	484.43082	1
H	77.12323	65.52606	70.71939	1863.74748	-1451.84923	1357.29467	1
C	74.99422	67.78938	70.38339	503.80731	517.99032	318.51606	1
H	75.10120	67.62591	69.28604	640.61020	-2157.10410	-1536.29104	1
H	73.90326	67.87712	70.60222	1687.88455	465.60692	2087.56452	1
H	75.45943	68.77754	70.61153	-1293.07895	-1899.88581	-1463.57452	1
C	73.57525	66.83432	72.77348	-153.07550	-733.68902	248.70981	1
O	73.07724	65.79683	72.42858	186.15459	-480.78787	-449.34387	1
N	72.98880	67.96048	73.21114	-487.53139	543.18517	42.03850	1
C	71.57205	68.04215	73.36008	-699.35871	69.34393	360.10701	1
H	71.19483	67.01141	73.55029	2.81126	-221.99807	-2715.58072	1
C	71.45060	69.06796	74.56610	-507.46439	-592.59194	122.07761	1
H	70.43972	69.55975	74.61291	-1975.08116	525.87560	-1801.71556	1
H	71.61433	68.56676	75.54654	1588.27743	-1913.22100	1113.83566	1
C	72.59974	70.07320	74.24435	-613.62442	-457.55993	-194.14490	1
H	72.28308	70.78820	73.45101	1707.54637	-721.27992	1996.92523	1
H	72.91645	70.68956	75.11954	758.23357	2100.81767	1560.55011	1
C	73.73873	69.10449	73.71435	-352.41524	704.96076	49.21465	1
H	74.33808	69.51119	72.86533	1714.76886	-2070.42778	443.35792	1
H	74.43784	68.68539	74.50488	1224.15040	1801.50881	1636.97633	1
C	70.91211	68.73191	72.02026	566.85117	537.05831	117.62814	1
O	71.46663	69.61759	71.41933	-550.26779	-406.08867	4.21666	1
N	69.67753	68.26090	71.88379	566.34832	-425.77725	180.18749	1
H	69.40269	67.48953	72.48889	644.44502	2646.80550	-52.73430	1
C	68.93463	68.56235	70.69044	612.34801	-281.48662	411.56221	1
H	69.64783	68.41784	69.84835	1667.23498	-1490.75142	1556.16753	1
C	67.69886	67.55270	70.67828	-714.45746	-175.43074	286.98927	1
H	67.09168	67.72067	71.60991	-787.09484	1763.03619	-1922.46060	1
O	68.05249	66.15571	70.68305	-429.46514	516.51932	-128.40526	1
H	67.63247	65.85397	71.51215	1981.82785	436.16847	-1818.18182	1
C	66.86790	68.03912	69.43501	630.79189	-468.94448	76.03230	1
H	65.93482	67.44195	69.31064	-1775.71861	2062.30353	131.89849	1
H	66.56414	69.10928	69.52756	-1855.14608	88.94118	1993.53760	1
H	67.45240	67.94054	68.49088	-1504.82350	-780.94249	-2132.90992	1
C	68.42999	70.09773	70.77965	288.57638	618.62814	-397.00270	1
O	68.17360	70.71370	69.77481	321.70495	-185.26452	574.37218	1
N	68.18337	70.45551	72.05181	-447.05792	-446.53431	367.76425	1
H	68.48328	69.83890	72.83646	350.31316	1975.63644	-1843.31522	1
C	68.41876	71.86326	72.31922	-511.23061	374.03917	-471.51480	1
H	69.28874	72.15426	71.68833	2234.79579	-898.70890	-1273.45190	1
C	67.08322	72.62409	71.87649	529.53781	-159.34528	563.72604	1
H	67.31260	73.72327	71.82951	654.59388	2347.94251	1217.51488	1
H	66.88605	72.28259	70.82280	-1526.55661	2103.42026	817.87394	1
O	65.92788	72.44868	72.73368	-554.84278	14.12789	399.58769	1
H	65.63610	71.56902	72.42666	1297.06236	2061.84854	1220.68812	1
C	68.72319	71.80132	73.89065	-662.15484	-412.13544	123.62687	1
O	68.80002	70.69852	74.43738	-540.01537	-317.05804	274.91456	1
N	69.03497	73.00263	74.38199	-202.76299	-690.00100	-131.49145	1
H	68.88699	73.74408	73.69085	-702.62132	-2617.37191	281.70413	1
C	68.61530	73.33799	75.73532	-491.73914	576.34187	222.73509	1
H	67.53997	73.61820	75.66552	1977.17978	-1823.58850	-434.68928	1
C	68.86018	72.08845	76.68931	-649.50797	-328.66843	306.11224	1
H	69.65424	71.42515	76.27372	2075.07803	1452.16490	-1004.45904	1
C	69.24979	72.59102	78.12261	-615.18735	-489.99689	-70.96352	1
H	69.43059	71.74342	78.82417	-1226.32661	-1724.38906	-1716.47170	1
H	70.17966	73.20928	78.09842	2381.64273	-969.84735	900.46860	1
H	68.44708	73.22614	78.56540	2122.74991	1702.23946	-141.36228	1
C	67.50041	71.33087	76.87818	-501.86074	-479.61276	-376.42482	1
H	67.61964	70.42978	77.52420	993.79261	-2246.95401	-1177.81305	1
H	66.72897	71.98123	77.35329	42.71279	-626.15391	-2651.37258	1
H	67.08463	70.98587	75.90054	-2026.39012	-1590.33709	-887.82922	1
C	69.54111	74.55152	76.17686	-672.89153	382.57394	-156.34072	1

O	70.77969	74.68781	75.87143	410.04627	221.86223	500.35950	1
N	68.91598	75.34184	77.00889	-378.11782	-443.79414	-441.10984	1
H	67.92408	75.18499	77.17583	2404.15973	-568.14894	1149.30085	1
C	69.60008	76.59231	77.19258	-544.54484	466.16019	331.29119	1
H	69.19551	77.22153	76.37518	-1409.80988	-170.17955	2325.32679	1
C	69.25528	77.15914	78.60336	-184.48284	116.78282	-758.89137	1
H	68.15775	77.31199	78.72689	1893.78641	1861.14652	611.04528	1
H	69.56020	76.43699	79.39566	-2170.79665	-974.19311	1327.50117	1
C	70.04078	78.51028	78.73710	389.51668	672.81878	-138.49152	1
H	71.12256	78.33895	78.53655	510.64577	1933.03327	1851.02484	1
H	69.72951	79.21980	77.94004	-2302.30869	-1105.72939	-948.89774	1
C	69.86848	79.19327	80.14241	-559.93427	-126.34590	542.30857	1
O	70.40215	80.25320	80.35672	-670.36003	-111.83166	76.36448	1
N	69.10526	78.49424	81.01091	-216.06729	-537.07037	446.51379	1
H	68.63230	77.59144	80.88847	-1251.96487	1074.12894	-2168.52500	1
H	68.90282	78.79970	81.96867	-2451.83023	-720.73851	944.84612	1
C	71.24728	76.22040	77.03740	546.24052	-563.88337	-85.11809	1
O	71.84628	75.62057	77.93498	71.70702	-307.40463	606.69673	1
N	71.80544	77.24209	76.28277	479.90863	-504.50129	222.86849	1
C	73.20388	77.31384	76.12346	75.51268	-600.91904	506.73700	1
H	73.52978	76.26392	76.33344	-2033.61562	1795.30103	254.89480	1
C	73.37264	77.81140	74.61259	523.79067	517.99274	284.45581	1
H	74.34590	78.32652	74.43979	1599.61665	-1127.00911	1895.98173	1
H	73.34758	76.95201	73.90669	-1881.83093	1275.82488	-1501.55016	1
C	72.13166	78.72821	74.41241	-600.05639	-404.05026	316.64485	1
H	72.30964	79.76163	74.78416	-2597.05260	496.36221	657.73249	1
H	71.82751	78.83228	73.34746	-1666.24518	1294.38985	-1723.90591	1
C	71.03727	77.97473	75.30390	493.57570	-129.95376	-602.56394	1
H	70.34173	78.65822	75.84841	-1469.08415	-637.84797	2204.22581	1
H	70.44115	77.20451	74.75565	-1038.97840	1068.88701	-2280.71789	1
C	73.83049	78.33274	77.26259	188.88100	241.05089	-727.87821	1
O	74.05730	77.87874	78.37904	531.88613	-49.19943	427.07960	1
N	73.97143	79.56308	76.83475	708.14833	26.93417	-179.73509	1
H	73.70680	79.73235	75.86773	-2232.69501	-1179.09831	1023.94816	1
C	74.82176	80.48115	77.52201	-738.32412	259.92548	104.42456	1
H	74.67467	81.43090	76.96279	2028.89123	1260.02983	1311.33324	1
C	74.33057	80.56350	79.02958	-258.65678	-345.52513	661.28459	1
H	74.31421	79.54595	79.49973	-1826.06090	-1801.46097	-918.64554	1
C	75.23732	81.54623	79.84645	-541.23400	531.92970	-218.41428	1
H	74.88911	81.63807	80.90226	1311.73218	-2219.18200	882.19023	1
H	76.29797	81.20225	79.87653	-1048.71298	1223.58118	-2196.97971	1
H	75.23315	82.57010	79.40551	419.06414	1903.92898	1903.44670	1
C	72.88162	81.16221	79.02998	-423.86431	152.66566	-648.55262	1
H	72.45478	81.21446	80.06172	105.68386	-2342.50080	1387.51238	1
H	72.85952	82.19323	78.60596	-1695.63603	-1484.73621	-1531.02793	1
H	72.18672	80.53602	78.42166	-1853.57121	-487.39031	1936.59343	1
C	76.33300	79.89848	77.28360	539.71428	-510.58232	267.58633	1
O	76.86393	80.08548	76.19227	-15.93758	-278.34085	-624.49402	1
N	76.79591	79.27879	78.34338	-676.81886	12.05292	276.17960	1
H	76.17396	79.21520	79.14715	-1694.44250	-373.84189	-2100.66098	1
C	78.03696	78.58928	78.33560	412.33078	-664.70360	108.35451	1
H	78.43342	78.72724	77.30353	1765.77476	1294.44366	1621.76594	1
C	78.92498	79.28172	79.46053	-179.66635	-721.95667	-264.73915	1
H	78.91992	80.38758	79.33947	-607.34922	2587.87032	-598.10018	1
H	78.48380	79.09174	80.46403	-359.69332	-1479.13954	-2259.74260	1
C	80.38208	78.72505	79.39083	-240.01076	616.02425	431.85322	1
H	80.37227	77.60356	79.41503	-1359.17131	782.54425	2227.99181	1
H	80.87107	79.01029	78.43306	-820.36667	85.81767	-2596.78715	1
C	81.18979	79.30171	80.62399	-513.07015	236.18570	-551.87280	1
H	81.27545	80.41090	80.55763	1816.11944	-1183.11722	-1650.94321	1
H	80.66038	79.09180	81.58167	-1800.04429	-1361.53473	1526.34643	1
C	82.58805	78.64221	80.61639	726.08972	-253.19732	-179.64713	1

H	82.58504	77.54922	80.80076	1224.27271	-456.51798	-2390.90304	1
H	83.19945	78.87481	79.72198	1206.40339	-1150.93514	-2154.90344	1
N	83.40122	79.24618	81.80954	329.70796	-633.68968	-155.67358	1
H	84.39244	78.88617	81.92458	1348.31305	1938.76845	1359.00643	1
H	83.56086	80.29436	81.78281	2096.92035	1446.54551	-966.48890	1
H	83.01174	79.10477	82.78683	-1197.07217	-2303.53719	827.28733	1
C	77.81143	77.03066	78.75789	-378.01186	-556.17301	-413.96481	1
O	78.75248	76.36107	79.12070	-409.44433	-307.55053	-453.30833	1
N	76.53574	76.66383	78.68405	-429.02477	524.70884	274.08502	1
H	75.81041	77.32650	78.35479	1706.34263	-1425.96036	1574.38926	1
C	76.29153	75.25973	78.54494	-45.25864	-626.22952	478.93267	1
H	77.17416	74.74645	78.99753	1988.63164	570.90773	1772.87287	1
C	74.90933	74.98832	79.27231	551.77128	-20.49526	-564.54996	1
H	74.92827	75.41545	80.30004	1434.61535	-1753.10253	1513.99471	1
H	74.05934	75.50047	78.75434	-714.67738	1819.09770	1898.36494	1
C	74.71284	73.43795	79.31234	446.99263	-555.77305	338.97221	1
H	74.43872	73.04511	78.30514	1953.68466	1892.25957	-161.67766	1
H	75.66290	72.92981	79.59317	-403.03785	2688.07032	-188.43591	1
C	73.59110	73.11936	80.38057	567.86262	335.74681	434.04421	1
H	73.84177	73.57477	81.36641	-1080.02275	1568.26081	-1948.78900	1
H	72.61614	73.56615	80.07672	-919.54108	2111.34692	1456.13463	1
C	73.49180	71.58061	80.50219	195.81897	173.33862	-745.11536	1
H	73.07391	71.07349	79.60979	1405.67949	-2301.21089	-390.08113	1
H	74.43026	71.08092	80.81627	-940.37328	-1118.32446	2299.72133	1
N	72.48382	71.26675	81.65733	319.26422	656.36007	-42.02424	1
H	72.30440	70.23840	81.84669	1961.03633	619.91968	1787.09422	1
H	72.73502	71.61605	82.62807	854.14343	-2156.80752	1429.08711	1
H	71.49633	71.63491	81.53887	1237.02905	-1378.50857	-1998.28500	1
C	76.08660	74.93989	76.95521	532.53319	-268.57236	517.55729	1
O	75.60740	75.76412	76.17533	266.16714	-290.73804	-558.88002	1
N	76.41833	73.68905	76.75764	-226.25352	640.49678	-270.32839	1
H	76.79813	73.21764	77.57767	2007.69063	-1035.27229	1523.50231	1
C	76.20571	72.95837	75.56283	-540.76542	-573.33303	49.50000	1
H	76.45132	71.91257	75.85293	-2374.20950	-1036.76023	843.75774	1
C	74.68086	73.13486	75.09743	-77.09082	666.87422	-415.84136	1
H	74.45566	74.22855	74.95227	-985.81834	617.19825	2463.91927	1
O	73.67420	72.63392	75.98994	293.29899	-391.65602	-477.80926	1
H	73.08417	73.41601	76.07915	2528.72714	-681.76262	-751.27122	1
C	74.80298	72.43549	73.69535	252.47363	-605.14473	440.05134	1
H	73.88487	72.57571	73.07916	-2052.65063	1456.49051	-1043.51830	1
H	75.67313	72.84017	73.11306	-2152.98800	-1661.97574	-161.70652	1
H	74.96933	71.33927	73.80236	-473.12726	-1584.27339	2165.61725	1
C	77.20640	73.49704	74.37291	360.36307	488.46713	505.10056	1
O	77.85679	72.68238	73.76661	-200.08817	-528.03552	-385.82850	1
N	77.06792	74.79804	74.14824	-597.54095	-226.44775	355.20479	1
H	76.41700	75.37880	74.74183	2179.23655	-1300.70981	-991.33835	1
C	78.07654	75.43553	73.37730	-687.68921	297.39807	-249.45179	1
H	78.79238	74.60730	73.16693	-349.16256	-2467.37713	-1101.72831	1
C	77.37581	75.99054	72.04627	672.47345	361.54328	-201.62880	1
H	76.56116	76.70824	72.29612	1541.41502	582.18425	2169.97026	1
H	78.11768	76.53483	71.41801	-2534.67717	60.25153	997.72295	1
C	76.82337	74.77793	71.28474	574.26774	-389.43119	-377.02542	1
C	77.74454	73.90019	70.63654	688.82679	-287.33580	-257.96133	1
H	78.82749	74.12045	70.63810	331.14182	1470.38163	2269.80013	1
C	75.42539	74.48681	71.27519	-383.52382	-675.04808	144.24917	1
H	74.70698	75.16130	71.77486	4.09847	1517.59662	2262.86397	1
C	77.25602	72.74392	70.02912	-134.76730	428.45285	-649.50303	1
H	77.97419	72.05913	69.54495	-903.23433	1600.49020	2011.53387	1
C	74.98440	73.31955	70.64560	-335.53281	-615.00040	-364.39058	1
H	73.90236	73.10084	70.65006	1596.45398	-1369.33862	1732.02659	1
C	75.87243	72.40069	70.01327	585.48547	-340.24194	-406.23938	1
O	75.43364	71.20245	69.39095	-419.15649	-173.04408	-511.94141	1

215	H	74.46205	71.28315	69.54090	-1071.84350	1559.71507	-1960.13012	1
216	C	78.69149	76.60955	74.32770	291.41878	-183.13091	710.72246	1
217	O	79.04234	76.38051	75.46209	379.87695	523.47396	-222.23622	1
218	N	78.86628	77.73572	73.63823	564.30591	373.09380	277.24371	1
219	H	78.54609	77.77358	72.67205	-120.59931	-2436.07824	-1214.35098	1
220	C	78.91068	78.97567	74.38203	404.07514	-299.37480	608.84009	1
221	H	78.12181	78.91110	75.17937	-1071.07882	-841.69025	2359.66467	1
222	C	80.36663	79.02826	74.99647	-683.12807	68.71770	-390.13096	1
223	H	80.58212	78.06932	75.52609	2068.88918	1596.58590	770.89603	1
224	H	81.12587	79.10353	74.18556	1379.86429	2342.49779	-179.85638	1
225	C	80.47237	80.23814	75.97640	-176.39967	537.49466	550.97268	1
226	H	80.24257	81.19684	75.46007	-396.28051	1942.22168	1869.33314	1
227	H	79.72768	80.15877	76.79851	-94.44700	586.00678	-2659.20016	1
228	C	81.96796	80.22429	76.54110	-291.69381	-718.18079	-150.72929	1
229	H	82.33226	79.24393	76.94099	-2303.38776	1089.76242	964.62032	1
230	H	82.74344	80.60953	75.83120	884.16729	2568.80533	-207.74283	1
231	N	81.88859	81.11544	77.62113	-594.49522	-252.81478	342.29276	1
232	H	81.04006	81.65359	77.70304	-1735.17426	2084.05705	-263.71605	1
233	C	82.80425	81.29047	78.48490	595.23177	-271.52487	442.22352	1
234	N	83.98046	80.54364	78.35481	585.53813	-424.79750	-105.81303	1
235	H	84.24625	79.87371	77.63427	959.88797	1327.56823	-2177.11905	1
236	H	84.78504	80.61268	78.97894	1578.48647	16.27378	-2220.76233	1
237	N	82.42268	82.24204	79.44172	-452.17763	-390.33541	-421.51853	1
238	H	81.57692	82.80353	79.52779	470.26107	1974.25629	-1817.92087	1
239	H	83.03205	82.53617	80.20591	1856.67307	-1573.97321	-1224.35416	1
240	C	78.63418	80.10234	73.26267	-531.47271	-581.45780	-55.06206	1
241	O	79.31655	80.18466	72.24452	-243.00837	192.73507	609.52485	1
242	N	77.59406	80.85431	73.61269	343.49288	-644.25607	38.09164	1
243	H	77.09617	80.67400	74.51420	-1691.16043	-1535.97525	-1484.73099	1
244	C	76.92491	81.40840	72.45711	542.66492	350.14337	454.42563	1
245	H	77.14260	80.62763	71.69250	1209.57979	-2396.95920	463.86744	1
246	C	75.38683	81.62234	72.75298	-737.14309	145.93010	242.71942	1
247	H	75.23728	82.20384	73.68844	-1050.18303	159.36419	-2509.06125	1
248	C	74.76060	82.35864	71.51675	-706.77696	-252.88343	-245.16230	1
249	H	73.65805	82.48484	71.62396	-1624.64039	-1665.49085	1417.86935	1
250	H	75.20100	83.37727	71.38608	2566.10521	598.51109	693.22305	1
251	H	74.94125	81.79685	70.57030	-1972.69758	107.10018	-1876.34312	1
252	C	74.68541	80.22232	72.84094	412.85148	672.58832	-27.69222	1
253	H	73.58538	80.32470	72.98924	1447.02277	1762.76533	-1490.78944	1
254	H	74.83803	79.62865	71.90913	1320.77379	-145.20455	2378.68491	1
255	H	75.08670	79.61761	73.69055	-1512.10848	1470.52031	1724.75147	1
256	C	77.65029	82.81972	72.05527	-607.11113	499.56689	73.73769	1
257	O	77.20099	83.89502	72.36495	357.97126	460.81534	356.68785	1
258	N	78.72424	82.53450	71.32597	536.55407	-368.62270	332.76306	1
259	H	79.07557	81.55054	71.20937	524.38866	2297.69985	1367.20941	1
260	C	79.52043	83.62594	70.89567	46.50277	-774.29190	-147.97904	1
261	H	78.84930	84.51535	70.91974	1676.51875	1883.29066	1032.55549	1
262	C	80.76680	83.76125	71.92024	-559.41456	-34.19198	-556.30433	1
263	H	81.30427	82.78487	71.97560	-64.66664	395.18769	2695.05373	1
264	H	81.50921	84.50322	71.54231	-2703.44278	-60.40056	333.79418	1
265	C	80.25273	84.15788	73.27632	-519.76435	377.31045	459.42366	1
266	H	79.45082	83.44226	73.57122	-1746.74323	1667.50644	-1261.73691	1
267	C	81.36551	84.08663	74.31449	570.95554	16.46261	-545.27700	1
268	H	80.99931	84.37604	75.33314	2391.58273	28.05277	-1305.07271	1
269	H	81.79007	83.05270	74.39632	175.75588	917.63458	2559.43867	1
270	H	82.21191	84.77470	74.05844	-1063.97851	-2155.34650	1283.00462	1
271	C	79.65827	85.55978	73.24859	193.76014	2.62946	-765.53141	1
272	H	79.40457	85.92300	74.27743	507.39394	-846.55087	2539.60089	1
273	H	80.36572	86.30267	72.79750	1337.57841	1818.19054	1526.01994	1
274	H	78.70943	85.58888	72.64855	1100.88635	-1172.91350	-2199.08909	1
275	C	80.11279	83.25523	69.44340	-549.21115	475.64486	-309.38218	1
276	O	79.97609	82.18168	68.91827	556.06106	-390.37755	-78.24250	1

```
O    80.86744   84.27409   68.81103     -572.27940     -310.19032     -209.75862          1
H    80.75806   84.99098   69.49435    -1380.08193     2212.14486      790.85944          1
```

Listing 8: C00008.xmolout

```
      276
  150.0000  150.0000  150.0000    0.0000    0.0000    0.0000***********   2.7750   11100
      0.2500    300.00   0.00   0.00   0.00  -1294.2694      0.0000    300.0000
H    75.32361   62.83301   77.20589    -2043.14101    -1757.64893      399.89339          1
N    75.45345   63.21380   76.27261      426.75047      490.45733     -334.42467          1
H    74.53795   63.60506   76.06979      442.25559    -1110.30854    -2448.52907          1
C    76.56277   64.07660   76.09664      415.32972     -666.39213       83.73172          1
H    77.37782   63.57045   76.66102      879.32219     2491.00447      667.35213          1
C    76.89748   64.16346   74.51107       31.35357      477.41777      628.23362          1
H    76.04080   64.63315   73.97316     1168.38489     2240.25337    -1019.71136          1
H    77.77343   64.83111   74.33509     -360.43770     2534.90498      931.67112          1
C    77.15274   62.78050   73.98923      463.18208      512.70242     -382.34388          1
H    76.31747   62.12412   74.32545     1725.29117     1863.52247     -987.07854          1
C    77.19027   62.77222   72.46506      710.42801     -144.27427     -313.15367          1
H    77.36369   61.74130   72.06215      364.20195    -1033.13725     2494.72524          1
H    76.23003   63.14348   72.02377     2567.61904      335.80679      847.48802          1
H    78.01110   63.42384   72.06984    -2587.99029      680.92867     -512.16411          1
C    78.46287   62.21336   74.52269      268.17616      741.64379      -40.42634          1
H    78.67028   61.19338   74.10843     1283.54314    -1945.37420    -1411.27812          1
H    79.33360   62.86444   74.25313    -1258.57796     1317.15249    -2026.02085          1
H    78.45128   62.11995   75.63851    -2502.84526      909.80597      575.92391          1
C    76.27261   65.52282   76.67263      619.36578     -458.64843     -172.09273          1
O    77.04869   66.52108   76.45343      -81.40433      433.87611      522.34594          1
N    75.22302   65.60285   77.44337      175.23996     -622.20190      341.55523          1
H    74.62034   64.79159   77.56017     -876.57256     -303.22879    -2561.90213          1
C    74.78048   66.95451   77.63425     -571.88214     -534.10783     -106.15033          1
H    74.06601   67.10613   76.80242    -2713.20841      -16.25800     -248.80809          1
C    74.07100   66.99335   79.03659      166.60354      288.16399     -716.09568          1
H    74.78776   66.61590   79.81665    -2097.85574     1636.78449     -586.18044          1
O    72.88585   66.16554   79.16514     -317.80580     -363.38227      484.43082          1
H    73.14689   65.56365   79.88488     1863.74748    -1451.84923     1357.29467          1
C    73.87457   68.54647   79.21229      503.80731      517.99032      318.51606          1
H    73.47257   68.79681   80.22158      640.61020    -2157.10410    -1536.29104          1
H    74.84216   69.09524   79.09037     1687.88455      465.60692     2087.56452          1
H    73.15910   68.95316   78.46160    -1293.07895    -1899.88581    -1463.57452          1
C    76.17223   67.91887   77.53622     -153.07550     -733.68902      248.70981          1
O    76.83238   68.09231   78.57543      186.15459     -480.78787     -449.34387          1
N    75.92251   68.93717   76.64073     -487.53139      543.18517       42.03850          1
C    76.93249   69.88754   76.34999     -699.35871       69.34393      360.10701          1
H    77.91741   69.38870   76.51361        2.81126     -221.99807    -2715.58072          1
C    76.58364   70.30644   74.84065     -507.46439     -592.59194      122.07761          1
H    77.00160   71.29811   74.55956    -1975.08116      525.87560    -1801.71556          1
H    76.98668   69.57020   74.11093     1588.27743    -1913.22100     1113.83566          1
C    75.03130   70.28009   74.87462     -613.62442     -457.55993     -194.14490          1
H    74.62833   71.18797   75.38013     1707.54637     -721.27992     1996.92523          1
H    74.55947   70.23355   73.86808      758.23357     2100.81767     1560.55011          1
C    74.78302   68.96565   75.75561     -352.41524      704.96076       49.21465          1
H    73.85303   68.98658   76.37357     1714.76886    -2070.42778      443.35792          1
H    74.82215   68.00866   75.17969     1224.15040     1801.50881     1636.97633          1
C    76.68937   71.16304   77.34654      566.85117      537.05831      117.62814          1
O    75.61041   71.76833   77.34647     -550.26779     -406.08867        4.21666          1
N    77.77415   71.41546   78.02611      566.34832     -425.77725      180.18749          1
H    78.57695   70.79889   77.90103      644.44502     2646.80550      -52.73430          1
C    77.68780   72.37912   79.07349      612.34801     -281.48662      411.56221          1
H    76.69392   72.86569   78.93081     1667.23498    -1490.75142     1556.16753          1
C    77.76311   71.52943   80.42700     -714.45746     -175.43074      286.98927          1
H    78.72185   70.94393   80.41446     -787.09484     1763.03619    -1922.46060          1
```

57	O	76.71617	70.54881	80.60012	-429.46514	516.51932	-128.40526	1
58	H	77.06227	69.80331	80.06453	1981.82785	436.16847	-1818.18182	1
59	C	77.82568	72.65746	81.51549	630.79189	-468.94448	76.03230	1
60	H	78.02500	72.23986	82.52970	-1775.71861	2062.30353	131.89849	1
61	H	78.62267	73.40541	81.30042	-1855.14608	88.94118	1993.53760	1
62	H	76.86212	73.21602	81.57229	-1504.82350	-780.94249	-2132.90992	1
63	C	78.94013	73.42241	78.88672	288.57638	618.62814	-397.00270	1
64	O	78.66550	74.62349	78.81521	321.70495	-185.26452	574.37218	1
65	N	80.10565	72.82853	78.88057	-447.05792	-446.53431	367.76425	1
66	H	80.00888	71.81549	78.93986	350.31316	1975.63644	-1843.31522	1
67	C	81.35137	73.43003	78.56726	-511.23061	374.03917	-471.51480	1
68	H	81.96984	72.54563	78.29417	2234.79579	-898.70890	-1273.45190	1
69	C	81.16345	74.43375	77.34779	529.53781	-159.34528	563.72604	1
70	H	82.14853	74.56255	76.82075	654.59388	2347.94251	1217.51488	1
71	H	80.47447	73.92075	76.62618	-1526.55661	2103.42026	817.87394	1
72	O	80.68738	75.77171	77.62657	-554.84278	14.12789	399.58769	1
73	H	79.81481	75.51173	78.12499	1297.06236	2061.84854	1220.68812	1
74	C	82.00790	74.22439	79.83589	-662.15484	-412.13544	123.62687	1
75	O	83.14418	74.63316	79.75364	-540.01537	-317.05804	274.91456	1
76	N	81.16016	74.36638	80.87075	-202.76299	-690.00100	-131.49145	1
77	H	80.29035	73.89323	80.63060	-702.62132	-2617.37191	281.70413	1
78	C	81.62560	74.48965	82.26189	-491.73914	576.34187	222.73509	1
79	H	80.73186	74.15500	82.84114	1977.17978	-1823.58850	-434.68928	1
80	C	82.83492	73.60568	82.49749	-649.50797	-328.66843	306.11224	1
81	H	83.60817	73.87680	81.73780	2075.07803	1452.16490	-1004.45904	1
82	C	83.43802	73.81150	83.87410	-615.18735	-489.99689	-70.96352	1
83	H	84.33578	73.15466	84.04085	-1226.32661	-1724.38906	-1716.47170	1
84	H	83.77304	74.88544	84.01941	2381.64273	-969.84735	900.46860	1
85	H	82.69960	73.58061	84.69025	2122.74991	1702.23946	-141.36228	1
86	C	82.49136	72.13750	82.31241	-501.86074	-479.61276	-376.42482	1
87	H	83.39535	71.47968	82.43129	993.79261	-2246.95401	-1177.81305	1
88	H	81.72976	71.79469	83.06519	42.71279	-626.15391	-2651.37258	1
89	H	82.06198	71.93985	81.29222	-2026.39012	-1590.33709	-887.82922	1
90	C	81.91819	76.09443	82.47869	-672.89153	382.57394	-156.34072	1
91	O	82.86425	76.48577	83.11827	410.04627	221.86223	500.35950	1
92	N	80.93096	76.80487	81.96975	-378.11782	-443.79414	-441.10984	1
93	H	80.16015	76.42025	81.39779	2404.15973	-568.14894	1149.30085	1
94	C	80.98364	78.23780	82.08846	-544.54484	466.16019	331.29119	1
95	H	81.22217	78.48887	83.14704	-1409.80988	-170.17955	2325.32679	1
96	C	82.17028	78.64906	81.11029	-184.48284	116.78282	-758.89137	1
97	H	83.10649	78.15600	81.46331	1893.78641	1861.14652	611.04528	1
98	H	81.98951	78.25091	80.08607	-2170.79665	-974.19311	1327.50117	1
99	C	82.33477	80.19762	81.08896	389.51668	672.81878	-138.49152	1
100	H	81.42354	80.67799	80.66464	510.64577	1933.03327	1851.02484	1
101	H	82.39341	80.59175	82.12945	-2302.30869	-1105.72939	-948.89774	1
102	C	83.61123	80.66908	80.29317	-559.93427	-126.34590	542.30857	1
103	O	83.84363	81.84585	80.19247	-670.36003	-111.83166	76.36448	1
104	N	84.36142	79.66136	79.79824	-216.06729	-537.07037	446.51379	1
105	H	84.22659	78.64516	79.85840	-1251.96487	1074.12894	-2168.52500	1
106	H	85.23948	79.80014	79.28626	-2451.83023	-720.73851	944.84612	1
107	C	79.49057	78.60408	81.59520	546.24052	-563.88337	-85.11809	1
108	O	78.89808	77.76602	80.94467	71.70702	-307.40463	606.69673	1
109	N	79.08821	79.81428	81.98543	479.90863	-504.50129	222.86849	1
110	C	77.86883	80.38112	81.48056	75.51268	-600.91904	506.73700	1
111	H	77.10756	79.57049	81.53026	-2033.61562	1795.30103	254.89480	1
112	C	77.62541	81.61032	82.44582	523.79067	517.99274	284.45581	1
113	H	77.03091	82.42233	81.96923	1599.61665	-1127.00911	1895.98173	1
114	H	77.07116	81.30706	83.36251	-1881.83093	1275.82488	-1501.55016	1
115	C	79.08992	82.03470	82.78122	-600.05639	-404.05026	316.64485	1
116	H	79.55276	82.56683	81.91885	-2597.05260	496.36221	657.73249	1
117	H	79.17334	82.70708	83.66443	-1666.24518	1294.38985	-1723.90591	1
118	C	79.75675	80.61008	83.00632	493.57570	-129.95376	-602.56394	1

H	80.85965	80.56674	82.84872	-1469.08415	-637.84797	2204.22581	1
H	79.50613	80.13007	83.98210	-1038.97840	1068.88701	-2280.71789	1
C	78.17494	80.85025	79.93641	188.88100	241.05089	-727.87821	1
O	78.85115	81.81368	79.67740	531.88613	-49.19943	427.07960	1
N	77.54738	80.01888	79.10979	708.14833	26.93417	-179.73509	1
H	77.05088	79.23417	79.53386	-2232.69501	-1179.09831	1023.94816	1
C	77.69782	80.09787	77.66836	-738.32412	259.92548	104.42456	1
H	78.26569	81.03817	77.46407	2028.89123	1260.02983	1311.33324	1
C	78.40797	78.82019	77.22280	-258.65678	-345.52513	661.28459	1
H	77.78626	77.95908	77.55741	-1826.06090	-1801.46097	-918.64554	1
C	78.53378	78.75333	75.71344	-541.23400	531.92970	-218.41428	1
H	79.04305	77.80767	75.37855	1311.73218	-2219.18200	882.19023	1
H	77.52534	78.78541	75.21278	-1048.71298	1223.58118	-2196.97971	1
H	79.13213	79.61518	75.30716	419.06414	1903.92898	1903.44670	1
C	79.75938	78.67306	77.88891	-423.86431	152.66566	-648.55262	1
H	80.21059	77.63401	77.70612	105.68386	-2342.50080	1387.51238	1
H	80.49617	79.43681	77.51376	-1695.63603	-1484.73621	-1531.02793	1
H	79.67655	78.80224	79.00725	-1853.57121	-487.39031	1936.59343	1
C	76.14094	80.06630	77.08343	539.71428	-510.58232	267.58633	1
O	75.25646	79.44669	77.66558	-15.93758	-278.34085	-624.49402	1
N	76.08286	80.69944	75.94172	-676.81886	12.05292	276.17960	1
H	76.96069	81.09536	75.61838	-1694.44250	-373.84189	-2100.66098	1
C	74.81520	80.90812	75.30346	412.33078	-664.70360	108.35451	1
H	74.15311	81.12127	76.17374	1765.77476	1294.44366	1621.76594	1
C	74.97605	82.08718	74.26552	-179.66635	-721.95667	-264.73915	1
H	75.52133	82.94823	74.71387	-607.34922	2587.87032	-598.10018	1
H	75.59318	81.75444	73.40063	-359.69332	-1479.13954	-2259.74260	1
C	73.54371	82.51046	73.79474	-240.01076	616.02425	431.85322	1
H	72.93884	81.62042	73.48399	-1359.17131	782.54425	2227.99181	1
H	72.97571	82.99225	74.62218	-820.36667	85.81767	-2596.78715	1
C	73.72278	83.49817	72.57370	-513.07015	236.18570	-551.87280	1
H	74.23937	84.43530	72.88511	1816.11944	-1183.11722	-1650.94321	1
H	74.36670	83.04229	71.78666	-1800.04429	-1361.53473	1526.34643	1
C	72.30941	83.79205	72.01818	726.08972	-253.19732	-179.64713	1
H	71.77071	82.90046	71.63835	1224.27271	-456.51798	-2390.90304	1
H	71.65372	84.36571	72.70319	1206.40339	-1150.93514	-2154.90344	1
N	72.48619	84.71412	70.76606	329.70796	-633.68968	-155.67358	1
H	71.59078	84.99628	70.27240	1348.31305	1938.76845	1359.00643	1
H	72.94372	85.65895	70.92395	2096.92035	1446.54551	-966.48890	1
H	73.05719	84.32677	69.95930	-1197.07217	-2303.53719	827.28733	1
C	74.37341	79.53685	74.53991	-378.01186	-556.17301	-413.96481	1
O	74.36087	79.43158	73.32228	-409.44433	-307.55053	-453.30833	1
N	74.01925	78.65506	75.44725	-429.02477	524.70884	274.08502	1
H	74.11802	78.86528	76.46811	1706.34263	-1425.96036	1574.38926	1
C	73.80121	77.31358	74.98669	-45.25864	-626.22952	478.93267	1
H	74.57496	77.18364	74.19574	1988.63164	570.90773	1772.87287	1
C	73.94500	76.37239	76.24420	551.77128	-20.49526	-564.54996	1
H	74.98197	76.40144	76.64983	1434.61535	-1753.10253	1513.99471	1
H	73.29154	76.73436	77.07002	-714.67738	1819.09770	1898.36494	1
C	73.54049	74.91860	75.82162	446.99263	-555.77305	338.97221	1
H	72.51566	74.91669	75.38237	1953.68466	1892.25957	-161.67766	1
H	74.21588	74.51743	75.03311	-403.03785	2688.07032	-188.43591	1
C	73.58101	74.02108	77.11837	567.86262	335.74681	434.04421	1
H	74.62367	73.67441	77.33547	-1080.02275	1568.26081	-1948.78900	1
H	73.25770	74.59932	78.01459	-919.54108	2111.34692	1456.13463	1
C	72.63149	72.81655	76.88281	195.81897	173.33862	-745.11536	1
H	71.55322	73.03331	77.02606	1405.67949	-2301.21089	-390.08113	1
H	72.78818	72.28692	75.92097	-940.37328	-1118.32446	2299.72133	1
N	73.01395	71.77048	77.96111	319.26422	656.36007	-42.02424	1
H	72.48422	70.85169	77.93706	1961.03633	619.91968	1787.09422	1
H	74.07838	71.48268	77.90139	854.14343	-2156.80752	1429.08711	1
H	72.87139	72.05438	78.97399	1237.02905	-1378.50857	-1998.28500	1

C	72.28984	77.24796	74.35169	532.53319	-268.57236	517.55729	1
O	71.34807	76.78070	74.94461	266.16714	-290.73804	-558.88002	1
N	72.35204	77.72616	73.11476	-226.25352	640.49678	-270.32839	1
H	73.22542	78.18126	72.76449	2007.69063	-1035.27229	1523.50231	1
C	71.12161	77.81092	72.39569	-540.76542	-573.33303	49.50000	1
H	70.36449	77.41767	73.11899	-2374.20950	-1036.76023	843.75774	1
C	70.90763	79.33788	72.01142	-77.09082	666.87422	-415.84136	1
H	71.60846	79.60330	71.17203	-985.81834	617.19825	2463.91927	1
O	71.31489	80.32220	73.03168	293.29899	-391.65602	-477.80926	1
H	72.12650	79.88464	73.34640	2528.72714	-681.76262	-751.27122	1
C	69.42076	79.39480	71.53509	252.47363	-605.14473	440.05134	1
H	69.12927	80.42514	71.22189	-2052.65063	1456.49051	-1043.51830	1
H	69.23486	78.71555	70.65887	-2152.98800	-1661.97574	-161.70652	1
H	68.71928	79.08985	72.34827	-473.12726	-1584.27339	2165.61725	1
C	71.25583	76.91490	71.02931	360.36307	488.46713	505.10056	1
O	70.57663	77.17331	70.04979	-200.08817	-528.03552	-385.82850	1
N	72.11307	75.92529	71.18324	-597.54095	-226.44775	355.20479	1
H	72.56187	75.83697	72.09225	2179.23655	-1300.70981	-991.33835	1
C	72.32836	75.04231	70.08137	-687.68921	297.39807	-249.45179	1
H	72.50181	75.69376	69.19753	-349.16256	-2467.37713	-1101.72831	1
C	73.52448	74.05331	70.44176	672.47345	361.54328	-201.62880	1
H	73.26647	73.47606	71.35862	1541.41502	582.18425	2169.97026	1
H	73.64335	73.31546	69.61497	-2534.67717	60.25153	997.72295	1
C	74.84000	74.81867	70.65664	574.26774	-389.43119	-377.02542	1
C	75.60737	75.24998	69.53339	688.82679	-287.33580	-257.96133	1
H	75.24456	75.08370	68.50273	331.14182	1470.38163	2269.80013	1
C	75.30517	75.05521	71.98530	-383.52382	-675.04808	144.24917	1
H	74.70531	74.73525	72.85601	4.09847	1517.59662	2262.86397	1
C	76.83592	75.86996	69.76476	-134.76730	428.45285	-649.50303	1
H	77.43725	76.19258	68.89680	-903.23433	1600.49020	2011.53387	1
C	76.54261	75.67918	72.16528	-335.53281	-615.00040	-364.39058	1
H	76.89987	75.84382	73.19685	1596.45398	-1369.33862	1732.02659	1
C	77.35533	76.09972	71.07198	585.48547	-340.24194	-406.23938	1
O	78.62014	76.72091	71.24460	-419.15649	-173.04408	-511.94141	1
H	78.67552	76.71467	72.22919	-1071.84350	1559.71507	-1960.13012	1
C	70.98144	74.12767	69.92003	291.41878	-183.13091	710.72246	1
O	70.41290	73.59958	70.85028	379.87695	523.47396	-222.23622	1
N	70.75328	74.00777	68.61946	564.30591	373.09380	277.24371	1
H	71.36987	74.49586	67.97182	-120.59931	-2436.07824	-1214.35098	1
C	69.60824	73.30337	68.14764	404.07514	-299.37480	608.84009	1
H	69.79324	73.19824	67.05435	-1071.07882	-841.69025	2359.66467	1
C	69.56728	71.90951	68.91393	-683.12807	68.71770	-390.13096	1
H	70.58154	71.45002	68.91806	2068.88918	1596.58590	770.89603	1
H	69.30278	72.05949	69.99228	1379.86429	2342.49779	-179.85638	1
C	68.53642	70.96872	68.22120	-176.39967	537.49466	550.97268	1
H	67.50327	71.38042	68.30606	-396.28051	1942.22168	1869.33314	1
H	68.73797	70.87783	67.13059	-94.44700	586.00678	-2659.20016	1
C	68.67566	69.55455	68.95388	-291.69381	-718.18079	-150.72929	1
H	69.67262	69.05350	68.85969	-2303.38776	1089.76242	964.62032	1
H	68.39673	69.55490	70.03841	884.16729	2568.80533	-207.74283	1
N	67.73643	68.76443	68.27708	-594.49522	-252.81478	342.29276	1
H	67.12373	69.24111	67.63388	-1735.17426	2084.05705	-263.71605	1
C	67.60177	67.50980	68.43157	595.23177	-271.52487	442.22352	1
N	68.45416	66.86686	69.33607	585.53813	-424.79750	-105.81303	1
H	69.19013	67.25870	69.92205	959.88797	1327.56823	-2177.11905	1
H	68.43651	65.86859	69.54694	1578.48647	16.27378	-2220.76233	1
N	66.57198	67.01014	67.62192	-452.17763	-390.33541	-421.51853	1
H	65.94998	67.47989	66.96582	470.26107	1974.25629	-1817.92087	1
H	66.30503	66.02515	67.60367	1856.67307	-1573.97321	-1224.35416	1
C	68.26355	74.16662	68.45505	-531.47271	-581.45780	-55.06206	1
O	67.17980	73.64185	68.39210	-243.00837	192.73507	609.52485	1
N	68.53159	75.48071	68.60067	343.49288	-644.25607	38.09164	1

```
H    69.52255  75.70340  68.75339    -1691.16043    -1535.97525    -1484.73099          1
C    67.51174  76.38171  69.14405      542.66492      350.14337      454.42563          1
H    66.56401  75.97086  68.71914     1209.57979    -2396.95920      463.86744          1
C    67.56040  76.26296  70.65613     -737.14309      145.93010      242.71942          1
H    68.57379  76.59354  70.98441    -1050.18303      159.36419    -2509.06125          1
C    66.52304  77.14070  71.32900     -706.77696     -252.88343     -245.16230          1
H    66.62014  77.12263  72.44940    -1624.64039    -1665.49085     1417.86935          1
H    66.61902  78.21979  70.99351     2566.10521      598.51109      693.22305          1
H    65.47705  76.80985  71.07982    -1972.69758      107.10018    -1876.34312          1
C    67.38711  74.82074  71.09790      412.85148      672.58832      -27.69222          1
H    67.55447  74.69227  72.20222     1447.02277     1762.76533    -1490.78944          1
H    66.35381  74.44047  70.86607     1320.77379     -145.20455     2378.68491          1
H    68.11542  74.14473  70.55697    -1512.10848     1470.52031     1724.75147          1
C    67.86239  77.91197  68.62292     -607.11113      499.56689       73.73769          1
O    67.45626  78.87606  69.23552      357.97126      460.81534      356.68785          1
N    68.56082  77.89882  67.49857      536.55407     -368.62270      332.76306          1
H    68.78200  76.97948  67.11931      524.38866     2297.69985     1367.20941          1
C    68.79732  79.09970  66.79370       46.50277     -774.29190     -147.97904          1
H    68.21154  79.87926  67.33053     1676.51875     1883.29066     1032.55549          1
C    70.38899  79.39093  66.75572     -559.41456      -34.19198     -556.30433          1
H    70.90392  78.53496  66.25865      -64.66664      395.18769     2695.05373          1
H    70.60413  80.28685  66.12621    -2703.44278      -60.40056      333.79418          1
C    70.91213  79.57735  68.15253     -519.76435      377.31045      459.42366          1
H    70.56544  78.70778  68.78047    -1746.74323     1667.50644    -1261.73691          1
C    72.43565  79.58745  68.15379      570.95554       16.46261     -545.27700          1
H    72.84884  79.71083  69.18817     2391.58273       28.05277    -1305.07271          1
H    72.85558  78.63334  67.74250      175.75588      917.63458     2559.43867          1
H    72.84326  80.42553  67.53166    -1063.97851    -2155.34650     1283.00462          1
C    70.39442  80.87025  68.76760      193.76014        2.62946     -765.53141          1
H    70.84308  81.05988  69.77665      507.39394     -846.55087     2539.60089          1
H    70.63666  81.75775  68.12788     1337.57841     1818.19054     1526.01994          1
H    69.28153  80.84624  68.89897     1100.88635    -1172.91350    -2199.08909          1
C    68.36659  78.83277  65.25899     -549.21115      475.64486     -309.38218          1
O    68.36783  77.73963  64.75493      556.06106     -390.37755      -78.24250          1
O    68.13136  79.98815  64.47846     -572.27940     -310.19032     -209.75862          1
H    68.22753  80.67854  65.19159    -1380.08193     2212.14486      790.85944          1
```

Listing 9: C00009.xmolout

```
       276
  150.0000  150.0000  150.0000     0.0000     0.0000     0.0000**********    1.8560     7424
       0.2500     300.00    0.00    0.00    0.00   -1294.2665       0.0000    300.0000
H    65.16994  75.22344  73.97829    -2043.14101    -1757.64893      399.89339          1
N    65.31837  75.30906  72.97283      426.75047      490.45733     -334.42467          1
H    64.38062  75.53283  72.61417      442.25559    -1110.30854    -2448.52907          1
C    66.27337  76.28642  72.61039      415.32972     -666.39213       83.73172          1
H    66.83387  76.54225  73.53671      879.32219     2491.00447      667.35213          1
C    67.23898  75.68363  71.46510       31.35357      477.41777      628.23362          1
H    66.62508  75.43729  70.56634     1168.38489     2240.25337    -1019.71136          1
H    67.97708  76.45213  71.13368     -360.43770     2534.90498      931.67112          1
C    67.93835  74.45309  71.97405      463.18208      512.70242     -382.34388          1
H    67.15729  73.74142  72.32721     1725.29117     1863.52247     -987.07854          1
C    68.74120  73.78352  70.86518      710.42801     -144.27427     -313.15367          1
H    69.17036  72.80558  71.20290      364.20195    -1033.13725     2494.72524          1
H    68.11076  73.57519  69.96270     2567.61904      335.80679      847.48802          1
H    69.60682  74.41769  70.53135    -2587.99029      680.92867     -512.16411          1
C    68.87257  74.76858  73.13499      268.17616      741.64379      -40.42634          1
H    69.44923  73.86233  73.45844     1283.54314    -1945.37420    -1411.27812          1
H    69.62384  75.55258  72.85888    -1258.57796     1317.15249    -2026.02085          1
H    68.31226  75.14246  74.03014    -2502.84526      909.80597      575.92391          1
C    65.35983  77.48888  72.03333      619.36578     -458.64843     -172.09273          1
O    64.12204  77.35689  71.98045      -81.40433      433.87611      522.34594          1
```

N	66.10993	78.50463	71.70992	175.23996	-622.20190	341.55523	1
H	67.11110	78.33601	71.80266	-876.57256	-303.22879	-2561.90213	1
C	65.66437	79.78136	71.24613	-571.88214	-534.10783	-106.15033	1
H	66.10565	79.83254	70.22664	-2713.20841	-16.25800	-248.80809	1
C	64.07257	79.89600	71.20819	166.60354	288.16399	-716.09568	1
H	63.68332	79.58082	72.21831	-2097.85574	1636.78449	-586.18044	1
O	63.42119	79.04499	70.26365	-317.80580	-363.38227	484.43082	1
H	63.31449	78.20349	70.84917	1863.74748	-1451.84923	1357.29467	1
C	63.82730	81.43575	71.01346	503.80731	517.99032	318.51606	1
H	62.73936	81.67906	71.00553	640.61020	-2157.10410	-1536.29104	1
H	64.29931	82.04026	71.82554	1687.88455	465.60692	2087.56452	1
H	64.25358	81.79441	70.04730	-1293.07895	-1899.88581	-1463.57452	1
C	66.35103	80.95450	72.14226	-153.07550	-733.68902	248.70981	1
O	66.56462	81.97250	71.53148	186.15459	-480.78787	-449.34387	1
N	66.50805	80.73533	73.45958	-487.53139	543.18517	42.03850	1
C	67.07386	81.74372	74.29697	-699.35871	69.34393	360.10701	1
H	66.85842	82.73005	73.82440	2.81126	-221.99807	-2715.58072	1
C	66.35323	81.45228	75.68396	-507.46439	-592.59194	122.07761	1
H	66.88379	81.90699	76.55043	-1975.08116	525.87560	-1801.71556	1
H	65.31207	81.84465	75.69915	1588.27743	-1913.22100	1113.83566	1
C	66.37454	79.89246	75.69445	-613.62442	-457.55993	-194.14490	1
H	67.38636	79.51179	75.96427	1707.54637	-721.27992	1996.92523	1
H	65.66116	79.43834	76.41847	758.23357	2100.81767	1560.55011	1
C	66.02505	79.56372	74.18000	-352.41524	704.96076	49.21465	1
H	66.55308	78.67252	73.77240	1714.76886	-2070.42778	443.35792	1
H	64.93787	79.48038	73.94876	1224.15040	1801.50881	1636.97633	1
C	68.68111	81.47653	74.51894	566.85117	537.05831	117.62814	1
O	69.29948	82.15628	75.30315	-550.26779	-406.08867	4.21666	1
N	69.10759	80.45851	73.78179	566.34832	-425.77725	180.18749	1
H	68.43955	80.02982	73.14324	644.44502	2646.80550	-52.73430	1
C	70.44700	80.02015	73.90807	612.34801	-281.48662	411.56221	1
H	70.88075	80.61745	74.74374	1667.23498	-1490.75142	1556.16753	1
C	70.36926	78.44672	74.19912	-714.45746	-175.43074	286.98927	1
H	69.88887	77.94098	73.31740	-787.09484	1763.03619	-1922.46060	1
O	69.57214	78.07354	75.34057	-429.46514	516.51932	-128.40526	1
H	68.89158	77.51423	74.91643	1981.82785	436.16847	-1818.18182	1
C	71.88053	78.02214	74.27149	630.79189	-468.94448	76.03230	1
H	71.98260	76.93359	74.48816	-1775.71861	2062.30353	131.89849	1
H	72.41597	78.21704	73.30693	-1855.14608	88.94118	1993.53760	1
H	72.42450	78.56897	75.07733	-1504.82350	-780.94249	-2132.90992	1
C	71.15223	80.29697	72.44885	288.57638	618.62814	-397.00270	1
O	70.53773	80.34385	71.41128	321.70495	-185.26452	574.37218	1
N	72.45453	80.40355	72.66074	-447.05792	-446.53431	367.76425	1
H	72.80688	80.29028	73.60938	350.31316	1975.63644	-1843.31522	1
C	73.38465	80.36444	71.54853	-511.23061	374.03917	-471.51480	1
H	73.16486	81.28202	70.95349	2234.79579	-898.70890	-1273.45190	1
C	74.80487	80.33025	72.17232	529.53781	-159.34528	563.72604	1
H	75.56326	80.23745	71.37165	654.59388	2347.94251	1217.51488	1
H	75.02552	81.25688	72.75052	-1526.55661	2103.42026	817.87394	1
O	75.07393	79.27593	73.38285	-554.84278	14.12789	399.58769	1
H	74.97865	78.51730	72.81081	1297.06236	2061.84854	1220.68812	1
C	73.13334	79.00661	70.66900	-662.15484	-412.13544	123.62687	1
O	72.82323	77.92555	71.15646	-540.01537	-317.05804	274.91456	1
N	73.47500	79.30078	69.42737	-202.76299	-690.00100	-131.49145	1
H	73.75323	80.27286	69.30299	-702.62132	-2617.37191	281.70413	1
C	72.79260	78.64333	68.34546	-491.73914	576.34187	222.73509	1
H	71.78018	78.45730	68.76686	1977.17978	-1823.58850	-434.68928	1
C	72.81882	79.62646	67.10789	-649.50797	-328.66843	306.11224	1
H	73.85986	79.96051	66.90640	2075.07803	1452.16490	-1004.45904	1
C	72.21001	78.92589	65.84338	-615.18735	-489.99689	-70.96352	1
H	72.16522	79.62002	64.97133	-1226.32661	-1724.38906	-1716.47170	1
H	72.82302	78.04373	65.53273	2381.64273	-969.84735	900.46860	1

H	71.17323	78.56195	66.03279	2122.74991	1702.23946	-141.36228	1
C	71.88945	80.85434	67.41127	-501.86074	-479.61276	-376.42482	1
H	71.88117	81.58219	66.56645	993.79261	-2246.95401	-1177.81305	1
H	70.83516	80.53515	67.58421	42.71279	-626.15391	-2651.37258	1
H	72.21684	81.40662	68.32257	-2026.39012	-1590.33709	-887.82922	1
C	73.56802	77.24347	68.02517	-672.89153	382.57394	-156.34072	1
O	74.17162	77.04789	66.99710	410.04627	221.86223	500.35950	1
N	73.35228	76.40953	69.03621	-378.11782	-443.79414	-441.10984	1
H	72.86300	76.71608	69.91337	2404.15973	-568.14894	1149.30085	1
C	73.91962	75.10915	68.94874	-544.54484	466.16019	331.29119	1
H	74.48236	75.07803	67.98653	-1409.80988	-170.17955	2325.32679	1
C	74.83464	75.03518	70.24722	-184.48284	116.78282	-758.89137	1
H	75.44810	75.96178	70.30085	1893.78641	1861.14652	611.04528	1
H	74.20679	75.04065	71.16633	-2170.79665	-974.19311	1327.50117	1
C	75.75311	73.77638	70.21724	389.51668	672.81878	-138.49152	1
H	75.13429	72.85202	70.22540	510.64577	1933.03327	1851.02484	1
H	76.30669	73.73338	69.25361	-2302.30869	-1105.72939	-948.89774	1
C	76.78680	73.73625	71.40958	-559.93427	-126.34590	542.30857	1
O	77.35001	72.70399	71.68551	-670.36003	-111.83166	76.36448	1
N	76.94128	74.91767	72.04390	-216.06729	-537.07037	446.51379	1
H	76.52041	75.83043	71.83632	-1251.96487	1074.12894	-2168.52500	1
H	77.62528	75.08451	72.78974	-2451.83023	-720.73851	944.84612	1
C	72.65183	74.07630	69.03885	546.24052	-563.88337	-85.11809	1
O	71.78421	74.25588	69.85652	71.70702	-307.40463	606.69673	1
N	72.79871	73.06375	68.17483	479.90863	-504.50129	222.86849	1
C	71.72448	72.15636	67.88366	75.51268	-600.91904	506.73700	1
H	70.81345	72.78882	67.77722	-2033.61562	1795.30103	254.89480	1
C	72.19419	71.44623	66.54395	523.79067	517.99274	284.45581	1
H	71.77354	70.42101	66.43369	1599.61665	-1127.00911	1895.98173	1
H	71.87112	72.01798	65.64542	-1881.83093	1275.82488	-1501.55016	1
C	73.74731	71.46019	66.69113	-600.05639	-404.05026	316.64485	1
H	74.09423	70.65577	67.37752	-2597.05260	496.36221	657.73249	1
H	74.28905	71.31607	65.72913	-1666.24518	1294.38985	-1723.90591	1
C	73.96860	72.90276	67.32398	493.57570	-129.95376	-602.56394	1
H	74.88548	73.01218	67.94721	-1469.08415	-637.84797	2204.22581	1
H	73.92263	73.73723	66.58368	-1038.97840	1068.88701	-2280.71789	1
C	71.52700	71.04658	69.05832	188.88100	241.05089	-727.87821	1
O	70.82186	70.08309	68.85359	531.88613	-49.19943	427.07960	1
N	72.25631	71.33039	70.14855	708.14833	26.93417	-179.73509	1
H	72.69443	72.25824	70.08724	-2232.69501	-1179.09831	1023.94816	1
C	71.67809	70.94799	71.41469	-738.32412	259.92548	104.42456	1
H	70.57944	71.06547	71.30507	2028.89123	1260.02983	1311.33324	1
C	72.10551	69.44405	71.65701	-258.65678	-345.52513	661.28459	1
H	72.97376	69.18785	71.01202	-1826.06090	-1801.46097	-918.64554	1
C	72.43451	69.18708	73.16875	-541.23400	531.92970	-218.41428	1
H	72.65619	68.11217	73.36660	1311.73218	-2219.18200	882.19023	1
H	73.32411	69.77614	73.50039	-1048.71298	1223.58118	-2196.97971	1
H	71.58561	69.48135	73.82952	419.06414	1903.92898	1903.44670	1
C	70.87503	68.53133	71.32250	-423.86431	152.66566	-648.55262	1
H	71.12603	67.44805	71.41210	105.68386	-2342.50080	1387.51238	1
H	70.01618	68.73077	72.00599	-1695.63603	-1484.73621	-1531.02793	1
H	70.51888	68.71476	70.26561	-1853.57121	-487.39031	1936.59343	1
C	72.33976	71.94928	72.51995	539.71428	-510.58232	267.58633	1
O	71.66226	72.52265	73.34547	-15.93758	-278.34085	-624.49402	1
N	73.65228	71.99010	72.32212	-676.81886	12.05292	276.17960	1
H	74.00409	71.43596	71.54118	-1694.44250	-373.84189	-2100.66098	1
C	74.55535	72.51748	73.28665	412.33078	-664.70360	108.35451	1
H	75.45630	72.75071	72.67654	1765.77476	1294.44366	1621.76594	1
C	73.88755	73.76699	73.98461	-179.66635	-721.95667	-264.73915	1
H	73.45138	74.45404	73.22389	-607.34922	2587.87032	-598.10018	1
H	73.03857	73.46535	74.65000	-359.69332	-1479.13954	-2259.74260	1
C	75.04382	74.45028	74.79462	-240.01076	616.02425	431.85322	1

H	75.24904	73.89562	75.74928	-1359.17131	782.54425	2227.99181	1
H	75.99243	74.43439	74.21148	-820.36667	85.81767	-2596.78715	1
C	74.60421	75.93615	75.08139	-513.07015	236.18570	-551.87280	1
H	74.33121	76.45804	74.13553	1816.11944	-1183.11722	-1650.94321	1
H	73.69233	75.96460	75.72071	-1800.04429	-1361.53473	1526.34643	1
C	75.79580	76.65287	75.77672	726.08972	-253.19732	-179.64713	1
H	75.95841	76.36304	76.83635	1224.27271	-456.51798	-2390.90304	1
H	76.75497	76.60039	75.22004	1206.40339	-1150.93514	-2154.90344	1
N	75.44170	78.15012	75.79851	329.70796	-633.68968	-155.67358	1
H	76.15737	78.76324	76.27862	1348.31305	1938.76845	1359.00643	1
H	75.28853	78.64264	74.77411	2096.92035	1446.54551	-966.48890	1
H	74.58093	78.40884	76.35508	-1197.07217	-2303.53719	827.28733	1
C	74.90096	71.36319	74.40947	-378.01186	-556.17301	-413.96481	1
O	74.12674	70.99376	75.26062	-409.44433	-307.55053	-453.30833	1
N	76.14777	70.96327	74.19087	-429.02477	524.70884	274.08502	1
H	76.69297	71.37344	73.42873	1706.34263	-1425.96036	1574.38926	1
C	76.64536	69.95457	75.07502	-45.25864	-626.22952	478.93267	1
H	75.73350	69.38747	75.37719	1988.63164	570.90773	1772.87287	1
C	77.71770	69.09487	74.29018	551.77128	-20.49526	-564.54996	1
H	77.30999	68.74196	73.31668	1434.61535	-1753.10253	1513.99471	1
H	78.61075	69.70764	74.03540	-714.67738	1819.09770	1898.36494	1
C	78.11076	67.89267	75.21213	446.99263	-555.77305	338.97221	1
H	78.60975	68.26009	76.14002	1953.68466	1892.25957	-161.67766	1
H	77.21413	67.32456	75.54815	-403.03785	2688.07032	-188.43591	1
C	79.07549	66.98180	74.35466	567.86262	335.74681	434.04421	1
H	78.50632	66.44946	73.55775	-1080.02275	1568.26081	-1948.78900	1
H	79.83440	67.61024	73.81109	-919.54108	2111.34692	1456.13463	1
C	79.80691	65.97636	75.27391	195.81897	173.33862	-745.11536	1
H	80.39105	66.43925	76.09451	1405.67949	-2301.21089	-390.08113	1
H	79.18005	65.15152	75.66828	-940.37328	-1118.32446	2299.72133	1
N	80.87811	65.30216	74.35191	319.26422	656.36007	-42.02424	1
H	81.57285	64.64358	74.80996	1961.03633	619.91968	1787.09422	1
H	80.51340	64.70713	73.55265	854.14343	-2156.80752	1429.08711	1
H	81.52108	65.97114	73.83705	1237.02905	-1378.50857	-1998.28500	1
C	77.36267	70.63228	76.38262	532.53319	-268.57236	517.55729	1
O	77.57833	69.89970	77.35068	266.16714	-290.73804	-558.88002	1
N	77.69905	71.88964	76.19395	-226.25352	640.49678	-270.32839	1
H	77.40833	72.19120	75.26495	2007.69063	-1035.27229	1523.50231	1
C	77.86662	72.83799	77.24267	-540.76542	-573.33303	49.50000	1
H	77.83621	73.81663	76.71534	-2374.20950	-1036.76023	843.75774	1
C	76.61613	72.65389	78.21267	-77.09082	666.87422	-415.84136	1
H	76.53840	71.56951	78.49867	-985.81834	617.19825	2463.91927	1
O	75.34058	72.99680	77.60568	293.29899	-391.65602	-477.80926	1
H	74.98167	72.10928	77.41423	2528.72714	-681.76262	-751.27122	1
C	77.00831	73.49691	79.47532	252.47363	-605.14473	440.05134	1
H	76.28109	73.34704	80.30771	-2052.65063	1456.49051	-1043.51830	1
H	78.01831	73.22840	79.86391	-2152.98800	-1661.97574	-161.70652	1
H	77.02848	74.58835	79.24664	-473.12726	-1584.27339	2165.61725	1
C	79.30955	72.63472	77.99550	360.36307	488.46713	505.10056	1
O	79.99891	73.61670	78.10658	-200.08817	-528.03552	-385.82850	1
N	79.48655	71.41154	78.47164	-597.54095	-226.44775	355.20479	1
H	78.76361	70.66725	78.26842	2179.23655	-1300.70981	-991.33835	1
C	80.76887	71.00050	78.92179	-687.68921	297.39807	-249.45179	1
H	80.66849	69.89692	79.02545	-349.16256	-2467.37713	-1101.72831	1
C	81.88511	71.41780	77.84865	672.47345	361.54328	-201.62880	1
H	81.83753	72.51679	77.66145	1541.41502	582.18425	2169.97026	1
H	82.89620	71.19679	78.26158	-2534.67717	60.25153	997.72295	1
C	81.65832	70.65405	76.53689	574.26774	-389.43119	-377.02542	1
C	82.15591	69.32433	76.39227	688.82679	-287.33580	-257.96133	1
H	82.66617	68.81772	77.23112	331.14182	1470.38163	2269.80013	1
C	80.96812	71.28906	75.46033	-383.52382	-675.04808	144.24917	1
H	80.54398	72.30187	75.58499	4.09847	1517.59662	2262.86397	1

```
C   82.00199  68.68698  75.15872    -134.76730     428.45285    -649.50303          1
H   82.39748  67.66281  75.04320    -903.23433    1600.49020    2011.53387          1
C   80.84921  70.61189  74.24202    -335.53281    -615.00040    -364.39058          1
H   80.33215  71.12067  73.40874    1596.45398   -1369.33862    1732.02659          1
C   81.36923  69.30189  74.04156     585.48547    -340.24194    -406.23938          1
O   81.25889  68.60456  72.79427    -419.15649    -173.04408    -511.94141          1
H   80.83039  69.31112  72.25934   -1071.84350    1559.71507   -1960.13012          1
C   81.13743  71.73241  80.32255     291.41878    -183.13091     710.72246          1
O   82.31517  71.85201  80.67119     379.87695     523.47396    -222.23622          1
N   80.04547  72.03154  80.98917     564.30591     373.09380     277.24371          1
H   79.18542  71.80286  80.49303    -120.59931   -2436.07824   -1214.35098          1
C   80.02994  72.80230  82.18338     404.07514    -299.37480     608.84009          1
H   78.95740  72.80526  82.48117   -1071.07882    -841.69025    2359.66467          1
C   80.97935  72.07128  83.22464    -683.12807      68.71770    -390.13096          1
H   80.64133  71.02393  83.39216    2068.88918    1596.58590     770.89603          1
H   82.01439  71.98284  82.81485    1379.86429    2342.49779    -179.85638          1
C   80.97924  72.87758  84.55873    -176.39967     537.49466     550.97268          1
H   81.26766  73.93927  84.38505    -396.28051    1942.22168    1869.33314          1
H   79.96373  72.90999  85.01212     -94.44700     586.00678   -2659.20016          1
C   82.02543  72.14420  85.52018    -291.69381    -718.18079    -150.72929          1
H   81.83736  71.05782  85.71364   -2303.38776    1089.76242     964.62032          1
H   83.09707  72.26154  85.21622     884.16729    2568.80533    -207.74283          1
N   81.84626  72.83258  86.72801    -594.49522    -252.81478     342.29276          1
H   81.33901  73.70270  86.67959   -1735.17426    2084.05705    -263.71605          1
C   82.28770  72.44717  87.85626     595.23177    -271.52487     442.22352          1
N   83.00580  71.24751  87.90473     585.53813    -424.79750    -105.81303          1
H   83.26285  70.61524  87.14784     959.88797    1327.56823   -2177.11905          1
H   83.41675  70.84670  88.74845    1578.48647      16.27378   -2220.76233          1
N   81.94416  73.35570  88.86695    -452.17763    -390.33541    -421.51853          1
H   81.42034  74.22888  88.83052     470.26107    1974.25629   -1817.92087          1
H   82.20363  73.22640  89.84566    1856.67307   -1573.97321   -1224.35416          1
C   80.57096  74.31751  81.87811    -531.47271    -581.45780     -55.06206          1
O   79.79896  75.22608  82.00362    -243.00837     192.73507     609.52485          1
N   81.88088  74.34142  81.61315     343.49288    -644.25607      38.09164          1
H   82.36631  73.42173  81.47168   -1691.16043   -1535.97525   -1484.73099          1
C   82.54039  75.57962  81.15482     542.66492     350.14337     454.42563          1
H   83.57924  75.38997  81.51189    1209.57979   -2396.95920     463.86744          1
C   82.47322  75.64916  79.64519    -737.14309     145.93010     242.71942          1
H   81.40138  75.70545  79.34735   -1050.18303     159.36419   -2509.06125          1
C   83.20770  76.86614  79.10807    -706.77696    -252.88343    -245.16230          1
H   83.09658  76.95865  77.99341   -1624.64039   -1665.49085    1417.86935          1
H   82.83096  77.82326  79.56142    2566.10521     598.51109     693.22305          1
H   84.30878  76.81138  79.32831   -1972.69758     107.10018   -1876.34312          1
C   83.04850  74.40481  78.99024     412.85148     672.58832     -27.69222          1
H   82.93910  74.43408  77.87138    1447.02277    1762.76533   -1490.78944          1
H   84.14285  74.28179  79.21832    1320.77379    -145.20455    2378.68491          1
H   82.52405  73.47588  79.36095   -1512.10848    1470.52031    1724.75147          1
C   81.85467  76.85367  81.95018    -607.11113     499.56689      73.73769          1
O   82.13012  77.02040  83.11201     357.97126     460.81534     356.68785          1
N   81.17573  77.66331  81.14092     536.55407    -368.62270     332.76306          1
H   80.96893  77.38996  80.17352     524.38866    2297.69985    1367.20941          1
C   80.18844  78.51078  81.73676      46.50277    -774.29190    -147.97904          1
H   79.92735  78.03681  82.70942    1676.51875    1883.29066    1032.55549          1
C   80.79021  79.99873  81.91604    -559.41456     -34.19198    -556.30433          1
H   81.08486  80.39163  80.91391     -64.66664     395.18769    2695.05373          1
H   80.00662  80.69491  82.29941   -2703.44278     -60.40056     333.79418          1
C   81.98050  79.97613  82.83587    -519.76435     377.31045     459.42366          1
H   82.66336  79.15782  82.50418   -1746.74323    1667.50644   -1261.73691          1
C   82.72982  81.30111  82.76765     570.95554      16.46261    -545.27700          1
H   83.63350  81.29994  83.42998    2391.58273      28.05277   -1305.07271          1
H   83.08061  81.52627  81.72766     175.75588     917.63458    2559.43867          1
H   82.08329  82.15684  83.09141   -1063.97851   -2155.34650    1283.00462          1
```

```
C    81.56812  79.69183  84.27379       193.76014         2.62946      -765.53141           1
H    82.43896  79.76765  84.97494       507.39394      -846.55087      2539.60089           1
H    80.78614  80.40941  84.63345      1337.57841      1818.19054      1526.01994           1
H    81.15291  78.65379  84.37755      1100.88635     -1172.91350     -2199.08909           1
C    79.02014  78.55962  80.64047      -549.21115       475.64486      -309.38218           1
O    79.15263  78.09426  79.53329       556.06106      -390.37755       -78.24250           1
O    77.84475  79.26033  80.98927      -572.27940      -310.19032      -209.75862           1
H    78.07431  79.48643  81.93443     -1380.08193      2212.14486       790.85944           1
```

Listing 10: C00010.xmolout

```
      276
  150.0000  150.0000  150.0000     0.0000     0.0000     0.0000***********    2.7750    11100
       0.2500     300.00    0.00    0.00    0.00  -1294.2214       0.0000     300.0000
H    71.61414  87.28463  75.84055     -2043.14101     -1757.64893       399.89339           1
N    72.42419  87.12158  75.24494       426.75047       490.45733      -334.42467           1
H    71.96957  86.72832  74.42222       442.25559     -1110.30854     -2448.52907           1
C    73.17452  88.29579  74.98116       415.32972      -666.39213        83.73172           1
H    72.48753  89.15564  75.13823       879.32219      2491.00447       667.35213           1
C    74.42430  88.30739  75.99907        31.35357       477.41777       628.23362           1
H    74.94679  87.32529  75.92213      1168.38489      2240.25337     -1019.71136           1
H    75.16540  89.08760  75.69979      -360.43770      2534.90498       931.67112           1
C    73.94652  88.53769  77.41044       463.18208       512.70242      -382.34388           1
H    73.06655  87.88212  77.59695      1725.29117      1863.52247      -987.07854           1
C    75.04035  88.16323  78.40261       710.42801      -144.27427      -313.15367           1
H    74.72261  88.35204  79.46049       364.20195     -1033.13725      2494.72524           1
H    75.31401  87.07930  78.32614      2567.61904       335.80679       847.48802           1
H    75.97660  88.75366  78.22632     -2587.99029       680.92867      -512.16411           1
C    73.53363  89.98752  77.62208       268.17616       741.64379       -40.42634           1
H    73.18411  90.16948  78.67126      1283.54314     -1945.37420     -1411.27812           1
H    74.38612  90.68956  77.43078     -1258.57796      1317.15249     -2026.02085           1
H    72.69875  90.28776  76.93784     -2502.84526       909.80597       575.92391           1
C    73.71781  88.19152  73.45893       619.36578      -458.64843      -172.09273           1
O    73.78899  89.20302  72.75364       -81.40433       433.87611       522.34594           1
N    74.17657  86.97935  73.23133       175.23996      -622.20190       341.55523           1
H    74.00513  86.33728  74.00810      -876.57256      -303.22879     -2561.90213           1
C    74.22312  86.39767  71.91571      -571.88214      -534.10783      -106.15033           1
H    73.22879  85.91266  71.80919     -2713.20841       -16.25800      -248.80809           1
C    74.45121  87.51368  70.80439       166.60354       288.16399      -716.09568           1
H    75.18643  88.26896  71.21704     -2097.85574      1636.78449      -586.18044           1
O    73.30492  88.30213  70.47104      -317.80580      -363.38227       484.43082           1
H    73.40906  89.04688  71.17387      1863.74748     -1451.84923      1357.29467           1
C    75.09961  86.70738  69.62145       503.80731       517.99032       318.51606           1
H    75.39382  87.38047  68.78307       640.61020     -2157.10410     -1536.29104           1
H    76.01480  86.15571  69.94095      1687.88455       465.60692      2087.56452           1
H    74.38855  85.95499  69.20763     -1293.07895     -1899.88581     -1463.57452           1
C    75.35926  85.23428  71.99965      -153.07550      -733.68902       248.70981           1
O    74.93550  84.11810  71.84902       186.15459      -480.78787      -449.34387           1
N    76.62496  85.63935  72.19125      -487.53139       543.18517        42.03850           1
C    77.71914  84.72727  72.08409      -699.35871        69.34393       360.10701           1
H    77.43688  83.94098  71.34711         2.81126      -221.99807     -2715.58072           1
C    78.90732  85.69727  71.66514      -507.46439      -592.59194       122.07761           1
H    79.91146  85.25579  71.85332     -1975.08116       525.87560     -1801.71556           1
H    78.86617  85.95839  70.58434      1588.27743     -1913.22100      1113.83566           1
C    78.59282  86.93181  72.56534      -613.62442      -457.55993      -194.14490           1
H    78.86368  86.73873  73.62842      1707.54637      -721.27992      1996.92523           1
H    79.13080  87.85750  72.26057       758.23357      2100.81767      1560.55011           1
C    77.01790  87.03065  72.37954      -352.41524       704.96076        49.21465           1
H    76.45162  87.43669  73.25090      1714.76886     -2070.42778       443.35792           1
H    76.70680  87.57086  71.45529      1224.15040      1801.50881      1636.97633           1
C    78.15043  84.10924  73.54141       566.85117       537.05831       117.62814           1
O    79.07137  83.32834  73.60416      -550.26779      -406.08867         4.21666           1
```

```
N    77.41246   84.59692   74.53951      566.34832     -425.77725      180.18749          1
H    76.70252   85.27680   74.26861      644.44502     2646.80550      -52.73430          1
C    78.02751   84.59099   75.83869      612.34801     -281.48662      411.56221          1
H    79.11884   84.67985   75.64163     1667.23498    -1490.75142     1556.16753          1
C    77.40085   85.82075   76.63288     -714.45746     -175.43074      286.98927          1
H    76.28111   85.74277   76.59497     -787.09484     1763.03619    -1922.46060          1
O    77.74920   87.11373   76.09601     -429.46514      516.51932     -128.40526          1
H    76.86893   87.48778   75.91026     1981.82785      436.16847    -1818.18182          1
C    77.87820   85.53259   78.10380      630.79189     -468.94448       76.03230          1
H    77.46948   86.28239   78.82022    -1775.71861     2062.30353      131.89849          1
H    77.54844   84.52602   78.45360    -1855.14608       88.94118     1993.53760          1
H    78.98898   85.56703   78.18704    -1504.82350     -780.94249    -2132.90992          1
C    77.64399   83.15767   76.52177      288.57638      618.62814     -397.00270          1
O    76.48521   82.88953   76.84105      321.70495     -185.26452      574.37218          1
N    78.75054   82.49298   76.72185     -447.05792     -446.53431      367.76425          1
H    79.59083   82.94068   76.35689      350.31316     1975.63644    -1843.31522          1
C    78.86020   81.17969   77.24750     -511.23061      374.03917     -471.51480          1
H    79.86916   81.21976   77.71344     2234.79579     -898.70890    -1273.45190          1
C    77.69364   80.90123   78.30011      529.53781     -159.34528      563.72604          1
H    78.03477   80.06190   78.97012      654.59388     2347.94251     1217.51488          1
H    77.62689   81.82862   78.93049    -1526.55661     2103.42026      817.87394          1
O    76.40611   80.52447   77.80053     -554.84278       14.12789      399.58769          1
H    76.10624   81.44754   77.43373     1297.06236     2061.84854     1220.68812          1
C    78.92859   80.10463   76.01305     -662.15484     -412.13544      123.62687          1
O    80.00257   79.51413   75.88906     -540.01537     -317.05804      274.91456          1
N    77.83462   80.02924   75.30464     -202.76299     -690.00100     -131.49145          1
H    77.08828   80.64887   75.62844     -702.62132    -2617.37191      281.70413          1
C    77.61877   79.14746   74.20979     -491.73914      576.34187      222.73509          1
H    76.82004   79.64346   73.61362     1977.17978    -1823.58850     -434.68928          1
C    78.98229   78.97116   73.40617     -649.50797     -328.66843      306.11224          1
H    79.77479   78.54868   74.07106     2075.07803     1452.16490    -1004.45904          1
C    78.77313   78.04860   72.15782     -615.18735     -489.99689      -70.96352          1
H    79.71179   77.94407   71.56436    -1226.32661    -1724.38906    -1716.47170          1
H    78.45553   77.02154   72.45672     2381.64273     -969.84735      900.46860          1
H    77.99136   78.45333   71.47326     2122.74991     1702.23946     -141.36228          1
C    79.41307   80.37880   72.86929     -501.86074     -479.61276     -376.42482          1
H    80.37834   80.33185   72.31215      993.79261    -2246.95401    -1177.81305          1
H    78.64943   80.80971   72.17993       42.71279     -626.15391    -2651.37258          1
H    79.54610   81.10672   73.70800    -2026.39012    -1590.33709     -887.82922          1
C    77.12375   77.70212   74.77586     -672.89153      382.57394     -156.34072          1
O    77.41918   77.25099   75.88521      410.04627      221.86223      500.35950          1
N    76.45132   77.10637   73.82297     -378.11782     -443.79414     -441.10984          1
H    76.28630   77.62079   72.96254     2404.15973     -568.14894     1149.30085          1
C    76.05308   75.73442   73.99622     -544.54484      466.16019      331.29119          1
H    76.99531   75.18620   74.21095    -1409.80988     -170.17955     2325.32679          1
C    75.09842   75.75565   75.25813     -184.48284      116.78282     -758.89137          1
H    75.70117   75.95951   76.17657     1893.78641     1861.14652      611.04528          1
H    74.38692   76.60785   75.17019    -2170.79665     -974.19311     1327.50117          1
C    74.31378   74.41304   75.40369      389.51668      672.81878     -138.49152          1
H    73.76098   74.18517   74.46678      510.64577     1933.03327     1851.02484          1
H    75.00881   73.55153   75.51162    -2302.30869    -1105.72939     -948.89774          1
C    73.32172   74.48788   76.62469     -559.93427     -126.34590      542.30857          1
O    72.46066   73.65212   76.76537     -670.36003     -111.83166       76.36448          1
N    73.56861   75.51633   77.46017     -216.06729     -537.07037      446.51379          1
H    74.26708   76.26656   77.39899    -1251.96487     1074.12894    -2168.52500          1
H    73.01684   75.71980   78.30079    -2451.83023     -720.73851      944.84612          1
C    75.28336   75.25441   72.65022      546.24052     -563.88337      -85.11809          1
O    74.21330   75.74489   72.35276       71.70702     -307.40463      606.69673          1
N    75.95111   74.27478   72.03704      479.90863     -504.50129      222.86849          1
C    75.53555   73.77422   70.75635       75.51268     -600.91904      506.73700          1
H    75.14452   74.64747   70.18390    -2033.61562     1795.30103      254.89480          1
C    76.87345   73.09752   70.22235      523.79067      517.99274      284.45581          1
```

113	H	76.68681	72.35107	69.41810	1599.61665	-1127.00911	1895.98173	1
114	H	77.58263	73.84846	69.80876	-1881.83093	1275.82488	-1501.55016	1
115	C	77.41557	72.46085	71.53907	-600.05639	-404.05026	316.64485	1
116	H	76.84709	71.53554	71.79194	-2597.05260	496.36221	657.73249	1
117	H	78.48897	72.17037	71.48821	-1666.24518	1294.38985	-1723.90591	1
118	C	77.12488	73.61529	72.59210	493.57570	-129.95376	-602.56394	1
119	H	76.87122	73.26408	73.62062	-1469.08415	-637.84797	2204.22581	1
120	H	77.92084	74.39495	72.65069	-1038.97840	1068.88701	-2280.71789	1
121	C	74.42274	72.61514	71.01809	188.88100	241.05089	-727.87821	1
122	O	74.74702	71.44915	70.89812	531.88613	-49.19943	427.07960	1
123	N	73.22676	73.14344	71.27524	708.14833	26.93417	-179.73509	1
124	H	73.30152	74.16278	71.41898	-2232.69501	-1179.09831	1023.94816	1
125	C	72.21963	72.37449	72.05524	-738.32412	259.92548	104.42456	1
126	H	72.81020	72.04495	72.94483	2028.89123	1260.02983	1311.33324	1
127	C	71.08984	73.31747	72.39099	-258.65678	-345.52513	661.28459	1
128	H	70.75483	73.79447	71.44184	-1826.06090	-1801.46097	-918.64554	1
129	C	69.90599	72.58899	73.00459	-541.23400	531.92970	-218.41428	1
130	H	69.10482	73.30346	73.33982	1311.73218	-2219.18200	882.19023	1
131	H	69.43104	71.87613	72.27013	-1048.71298	1223.58118	-2196.97971	1
132	H	70.21806	71.98329	73.90099	419.06414	1903.92898	1903.44670	1
133	C	71.51848	74.42446	73.33789	-423.86431	152.66566	-648.55262	1
134	H	70.70752	75.19125	73.47450	105.68386	-2342.50080	1387.51238	1
135	H	71.75995	74.01776	74.35913	-1695.63603	-1484.73621	-1531.02793	1
136	H	72.44078	74.97131	72.95980	-1853.57121	-487.39031	1936.59343	1
137	C	71.71796	71.09350	71.16777	539.71428	-510.58232	267.58633	1
138	O	70.60836	71.03755	70.66606	-15.93758	-278.34085	-624.49402	1
139	N	72.64949	70.16334	71.15867	-676.81886	12.05292	276.17960	1
140	H	73.57899	70.34434	71.59634	-1694.44250	-373.84189	-2100.66098	1
141	C	72.53907	69.17530	70.12264	412.33078	-664.70360	108.35451	1
142	H	71.88792	69.66540	69.36077	1765.77476	1294.44366	1621.76594	1
143	C	74.00733	68.82490	69.65623	-179.66635	-721.95667	-264.73915	1
144	H	74.53344	69.73927	69.29381	-607.34922	2587.87032	-598.10018	1
145	H	74.61402	68.45377	70.51287	-359.69332	-1479.13954	-2259.74260	1
146	C	73.89202	67.73208	68.54223	-240.01076	616.02425	431.85322	1
147	H	73.37973	66.83052	68.95569	-1359.17131	782.54425	2227.99181	1
148	H	73.26343	68.08790	67.69533	-820.36667	85.81767	-2596.78715	1
149	C	75.35198	67.37788	68.04838	-513.07015	236.18570	-551.87280	1
150	H	75.85503	68.27512	67.61983	1816.11944	-1183.11722	-1650.94321	1
151	H	75.98981	67.05189	68.90184	-1800.04429	-1361.53473	1526.34643	1
152	C	75.23625	66.25470	66.99180	726.08972	-253.19732	-179.64713	1
153	H	74.83310	65.29767	67.37886	1224.27271	-456.51798	-2390.90304	1
154	H	74.70025	66.54414	66.06594	1206.40339	-1150.93514	-2154.90344	1
155	N	76.68684	65.90543	66.51553	329.70796	-633.68968	-155.67358	1
156	H	76.75740	65.13445	65.79041	1348.31305	1938.76845	1359.00643	1
157	H	77.24334	66.68023	66.05005	2096.92035	1446.54551	-966.48890	1
158	H	77.36662	65.55911	67.25351	-1197.07217	-2303.53719	827.28733	1
159	C	71.81330	67.88665	70.83110	-378.01186	-556.17301	-413.96481	1
160	O	72.39049	66.83732	71.01624	-409.44433	-307.55053	-453.30833	1
161	N	70.57309	68.25169	71.09695	-429.02477	524.70884	274.08502	1
162	H	70.28470	69.22193	70.85798	1706.34263	-1425.96036	1574.38926	1
163	C	69.68813	67.38479	71.79332	-45.25864	-626.22952	478.93267	1
164	H	68.69597	67.88359	71.71145	1988.63164	570.90773	1772.87287	1
165	C	69.74647	65.97695	71.06252	551.77128	-20.49526	-564.54996	1
166	H	69.61713	66.11096	69.96502	1434.61535	-1753.10253	1513.99471	1
167	H	70.76034	65.51933	71.18974	-714.67738	1819.09770	1898.36494	1
168	C	68.64512	65.02976	71.63801	446.99263	-555.77305	338.97221	1
169	H	68.81901	64.83969	72.72537	1953.68466	1892.25957	-161.67766	1
170	H	67.63443	65.49008	71.56144	-403.03785	2688.07032	-188.43591	1
171	C	68.72405	63.68735	70.80355	567.86262	335.74681	434.04421	1
172	H	68.46150	63.86851	69.73587	-1080.02275	1568.26081	-1948.78900	1
173	H	69.76459	63.28856	70.79552	-919.54108	2111.34692	1456.13463	1
174	C	67.75764	62.66549	71.44461	195.81897	173.33862	-745.11536	1

H	68.05495	62.32300	72.45595	1405.67949	-2301.21089	-390.08113	1
H	66.69292	62.97286	71.44643	-940.37328	-1118.32446	2299.72133	1
N	67.79628	61.37373	70.55929	319.26422	656.36007	-42.02424	1
H	67.19407	60.56773	70.89486	1961.03633	619.91968	1787.09422	1
H	67.47404	61.46903	69.55240	854.14343	-2156.80752	1429.08711	1
H	68.73759	60.89705	70.44394	1237.02905	-1378.50857	-1998.28500	1
C	70.16449	67.21755	73.34941	532.53319	-268.57236	517.55729	1
O	69.64429	66.37289	74.03694	266.16714	-290.73804	-558.88002	1
N	71.03037	68.15566	73.70321	-226.25352	640.49678	-270.32839	1
H	71.33376	68.90367	73.06291	2007.69063	-1035.27229	1523.50231	1
C	71.86429	67.95972	74.84400	-540.76542	-573.33303	49.50000	1
H	71.29000	67.39333	75.61186	-2374.20950	-1036.76023	843.75774	1
C	73.16614	67.18144	74.33134	-77.09082	666.87422	-415.84136	1
H	73.60780	67.78038	73.48563	-985.81834	617.19825	2463.91927	1
O	72.94586	65.87879	73.76419	293.29899	-391.65602	-477.80926	1
H	72.85480	66.09612	72.80334	2528.72714	-681.76262	-751.27122	1
C	74.11104	67.22698	75.58292	252.47363	-605.14473	440.05134	1
H	75.08379	66.72275	75.37757	-2052.65063	1456.49051	-1043.51830	1
H	74.34280	68.27346	75.89393	-2152.98800	-1661.97574	-161.70652	1
H	73.65419	66.70870	76.45933	-473.12726	-1584.27339	2165.61725	1
C	72.20687	69.52354	75.16137	360.36307	488.46713	505.10056	1
O	72.06164	70.33207	74.26114	-200.08817	-528.03552	-385.82850	1
N	72.64060	69.69867	76.40146	-597.54095	-226.44775	355.20479	1
H	72.69810	68.90019	77.03388	2179.23655	-1300.70981	-991.33835	1
C	73.19485	70.95639	76.75380	-687.68921	297.39807	-249.45179	1
H	72.68276	71.62915	76.02203	-349.16256	-2467.37713	-1101.72831	1
C	72.87874	71.25482	78.29084	672.47345	361.54328	-201.62880	1
H	73.51558	70.61729	78.94937	1541.41502	582.18425	2169.97026	1
H	73.13815	72.32215	78.49724	-2534.67717	60.25153	997.72295	1
C	71.39943	70.99660	78.61097	574.26774	-389.43119	-377.02542	1
C	70.43446	71.99387	78.28020	688.82679	-287.33580	-257.96133	1
H	70.75120	72.92794	77.76309	331.14182	1470.38163	2269.80013	1
C	71.00643	69.77868	79.24446	-383.52382	-675.04808	144.24917	1
H	71.75325	68.99928	79.48278	4.09847	1517.59662	2262.86397	1
C	69.10183	71.76355	78.62515	-134.76730	428.45285	-649.50303	1
H	68.35107	72.53605	78.38390	-903.23433	1600.49020	2011.53387	1
C	69.65921	69.59538	79.56351	-335.53281	-615.00040	-364.39058	1
H	69.36412	68.65321	80.05809	1596.45398	-1369.33862	1732.02659	1
C	68.66271	70.57460	79.27774	585.48547	-340.24194	-406.23938	1
O	67.29434	70.39784	79.61198	-419.15649	-173.04408	-511.94141	1
H	67.33878	69.50436	80.02799	-1071.84350	1559.71507	-1960.13012	1
C	74.80615	70.88590	76.47604	291.41878	-183.13091	710.72246	1
O	75.22609	70.71728	75.35435	379.87695	523.47396	-222.23622	1
N	75.50958	71.00997	77.59790	564.30591	373.09380	277.24371	1
H	75.04776	71.18030	78.49102	-120.59931	-2436.07824	-1214.35098	1
C	76.91590	71.20062	77.45774	404.07514	-299.37480	608.84009	1
H	77.16229	70.70862	76.48815	-1071.07882	-841.69025	2359.66467	1
C	77.14939	72.76920	77.44793	-683.12807	68.71770	-390.13096	1
H	76.35253	73.28798	76.87136	2068.88918	1596.58590	770.89603	1
H	77.09302	73.18051	78.48154	1379.86429	2342.49779	-179.85638	1
C	78.55379	73.01374	76.81656	-176.39967	537.49466	550.97268	1
H	79.34883	72.54373	77.43764	-396.28051	1942.22168	1869.33314	1
H	78.62812	72.53391	75.81537	-94.44700	586.00678	-2659.20016	1
C	78.76644	74.59276	76.70479	-291.69381	-718.18079	-150.72929	1
H	78.09461	75.13416	75.98782	-2303.38776	1089.76242	964.62032	1
H	78.75326	75.15525	77.67334	884.16729	2568.80533	-207.74283	1
N	80.07562	74.62819	76.20138	-594.49522	-252.81478	342.29276	1
H	80.53815	73.74180	76.07379	-1735.17426	2084.05705	-263.71605	1
C	80.71629	75.69439	75.92120	595.23177	-271.52487	442.22352	1
N	80.06748	76.90962	76.10254	585.53813	-424.79750	-105.81303	1
H	79.06955	77.03703	76.39071	959.88797	1327.56823	-2177.11905	1
H	80.48030	77.85225	75.89621	1578.48647	16.27378	-2220.76233	1

```
N    82.01604  75.41683  75.46178     -452.17763     -390.33541     -421.51853           1
H    82.50604  74.53192  75.34736      470.26107     1974.25629    -1817.92087           1
H    82.68581  76.14489  75.21369     1856.67307    -1573.97321    -1224.35416           1
C    77.64904  70.57445  78.78838     -531.47271     -581.45780      -55.06206           1
O    77.77105  71.21851  79.80853     -243.00837      192.73507      609.52485           1
N    77.99498  69.31307  78.55472      343.49288     -644.25607       38.09164           1
H    77.76984  68.99589  77.61129    -1691.16043    -1535.97525    -1484.73099           1
C    78.94363  68.57831  79.38818      542.66492      350.14337      454.42563           1
H    79.06102  67.60662  78.85123     1209.57979    -2396.95920      463.86744           1
C    80.24498  69.36269  79.44627     -737.14309      145.93010      242.71942           1
H    80.00932  70.36575  79.87024    -1050.18303      159.36419    -2509.06125           1
C    81.27541  68.69143  80.33333     -706.77696     -252.88343     -245.16230           1
H    82.22465  69.29125  80.40174    -1624.64039    -1665.49085     1417.86935           1
H    80.88278  68.55084  81.38815     2566.10521      598.51109      693.22305           1
H    81.55369  67.67246  79.94605    -1972.69758      107.10018    -1876.34312           1
C    80.83093  69.56789  78.06012      412.85148      672.58832      -27.69222           1
H    81.77829  70.17233  78.09151     1447.02277     1762.76533    -1490.78944           1
H    81.07981  68.58659  77.57016     1320.77379     -145.20455     2378.68491           1
H    80.11135  70.10353  77.38240    -1512.10848     1470.52031     1724.75147           1
C    78.24165  68.37474  80.87604     -607.11113      499.56689       73.73769           1
O    78.91433  68.25082  81.87990      357.97126      460.81534      356.68785           1
N    76.93043  68.26977  80.75244      536.55407     -368.62270      332.76306           1
H    76.55578  68.40764  79.81609      524.38866     2297.69985     1367.20941           1
C    76.13352  68.02979  81.89489       46.50277     -774.29190     -147.97904           1
H    76.84730  67.69395  82.68191     1676.51875     1883.29066     1032.55549           1
C    75.34250  69.38375  82.28682     -559.41456      -34.19198     -556.30433           1
H    74.60659  69.62177  81.48225      -64.66664      395.18769     2695.05373           1
H    74.73963  69.21835  83.21151    -2703.44278      -60.40056      333.79418           1
C    76.29912  70.53132  82.46717     -519.76435      377.31045      459.42366           1
H    76.88518  70.66946  81.51186    -1746.74323     1667.50644    -1261.73691           1
C    75.52148  71.81142  82.74648      570.95554       16.46261     -545.27700           1
H    76.20464  72.69006  82.87590     2391.58273       28.05277    -1305.07271           1
H    74.81878  72.05745  81.90869      175.75588      917.63458     2559.43867           1
H    74.90792  71.72655  83.68040    -1063.97851    -2155.34650     1283.00462           1
C    77.28118  70.26389  83.59710      193.76014        2.62946     -765.53141           1
H    77.89964  71.16932  83.83023      507.39394     -846.55087     2539.60089           1
H    76.75820  69.96593  84.54293     1337.57841     1818.19054     1526.01994           1
H    77.99761  69.43341  83.32282     1100.88635    -1172.91350    -2199.08909           1
C    74.99554  66.96562  81.47070     -549.21115      475.64486     -309.38218           1
O    74.58609  66.84934  80.34517      556.06106     -390.37755      -78.24250           1
O    74.37125  66.28032  82.53978     -572.27940     -310.19032     -209.75862           1
H    74.93887  66.61410  83.28909    -1380.08193     2212.14486      790.85944           1
```

Listing 11: C00011.xmolout

```
       276
  150.0000  150.0000  150.0000     0.0000     0.0000     0.0000**********    0.8412     3365
       0.2500    300.00   0.00    0.00    0.00   -1294.1729       0.0000    300.0000
H    77.10411  73.37372  81.69057    -2043.14101    -1757.64893      399.89339           1
N    77.99704  73.74902  82.00846      426.75047      490.45733     -334.42467           1
H    78.51201  72.90672  82.29087      442.25559    -1110.30854    -2448.52907           1
C    78.70723  74.46890  81.02212      415.32972     -666.39213       83.73172           1
H    77.98589  74.76090  80.22733      879.32219     2491.00447      667.35213           1
C    79.40510  75.74283  81.72733       31.35357      477.41777      628.23362           1
H    79.87051  75.42510  82.69046     1168.38489     2240.25337    -1019.71136           1
H    80.23343  76.14703  81.09544     -360.43770     2534.90498      931.67112           1
C    78.34191  76.78379  81.95649      463.18208      512.70242     -382.34388           1
H    77.41065  76.26478  82.27868     1725.29117     1863.52247     -987.07854           1
C    78.75812  77.74318  83.06444      710.42801     -144.27427     -313.15367           1
H    77.97008  78.51533  83.26077      364.20195    -1033.13725     2494.72524           1
H    78.94251  77.20435  84.02969     2567.61904      335.80679      847.48802           1
H    79.70096  78.28967  82.80415    -2587.99029      680.92867     -512.16411           1
```

C	78.06022	77.56451	80.67947	268.17616	741.64379	-40.42634	1
H	77.24461	78.31837	80.82793	1283.54314	-1945.37420	-1411.27812	1
H	78.96683	78.12325	80.33102	-1258.57796	1317.15249	-2026.02085	1
H	77.74431	76.89334	79.84012	-2502.84526	909.80597	575.92391	1
C	79.85352	73.42632	80.53399	619.36578	-458.64843	-172.09273	1
O	80.09312	72.38269	81.10689	-81.40433	433.87611	522.34594	1
N	80.40253	73.92333	79.42258	175.23996	-622.20190	341.55523	1
H	80.12009	74.86623	79.15553	-876.57256	-303.22879	-2561.90213	1
C	81.67119	73.42021	79.01681	-571.88214	-534.10783	-106.15033	1
H	81.76847	72.43964	79.53740	-2713.20841	-16.25800	-248.80809	1
C	81.54513	73.31316	77.42037	166.60354	288.16399	-716.09568	1
H	81.15004	74.29995	77.05835	-2097.85574	1636.78449	-586.18044	1
O	80.58234	72.33737	76.96377	-317.80580	-363.38227	484.43082	1
H	79.85704	72.92347	76.67959	1863.74748	-1451.84923	1357.29467	1
C	82.99440	73.11972	76.85182	503.80731	517.99032	318.51606	1
H	82.99663	73.17148	75.73732	640.61020	-2157.10410	-1536.29104	1
H	83.71568	73.91484	77.21859	1687.88455	465.60692	2087.56452	1
H	83.41741	72.12683	77.13061	-1293.07895	-1899.88581	-1463.57452	1
C	82.75179	74.54008	79.49014	-153.07550	-733.68902	248.70981	1
O	82.38847	75.67119	79.68462	186.15459	-480.78787	-449.34387	1
N	83.98370	74.02546	79.56261	-487.53139	543.18517	42.03850	1
C	85.13340	74.87927	79.71822	-699.35871	69.34393	360.10701	1
H	84.91803	75.52578	80.59943	2.81126	-221.99807	-2715.58072	1
C	86.31873	73.84559	79.93435	-507.46439	-592.59194	122.07761	1
H	87.27838	74.20266	79.49733	-1975.08116	525.87560	-1801.71556	1
H	86.51631	73.68093	81.01701	1588.27743	-1913.22100	1113.83566	1
C	85.78846	72.54436	79.25817	-613.62442	-457.55993	-194.14490	1
H	85.89844	72.59189	78.15150	1707.54637	-721.27992	1996.92523	1
H	86.30678	71.61880	79.59593	758.23357	2100.81767	1560.55011	1
C	84.25788	72.60438	79.69340	-352.41524	704.96076	49.21465	1
H	83.55674	72.01582	79.05923	1714.76886	-2070.42778	443.35792	1
H	84.07443	72.35979	80.76735	1224.15040	1801.50881	1636.97633	1
C	85.35710	75.75612	78.36934	566.85117	537.05831	117.62814	1
O	85.02902	75.34406	77.25470	-550.26779	-406.08867	4.21666	1
N	85.98648	76.85518	78.70378	566.34832	-425.77725	180.18749	1
H	86.17002	76.97368	79.69947	644.44502	2646.80550	-52.73430	1
C	86.51274	77.78594	77.77421	612.34801	-281.48662	411.56221	1
H	87.09742	78.48491	78.41142	1667.23498	-1490.75142	1556.16753	1
C	87.43919	76.97646	76.74742	-714.45746	-175.43074	286.98927	1
H	86.83489	76.15502	76.26758	-787.09484	1763.03619	-1922.46060	1
O	88.58044	76.33217	77.34280	-429.46514	516.51932	-128.40526	1
H	88.39020	75.39363	77.14225	1981.82785	436.16847	-1818.18182	1
C	87.75797	78.06864	75.66394	630.79189	-468.94448	76.03230	1
H	88.42243	77.66369	74.86525	-1775.71861	2062.30353	131.89849	1
H	86.82959	78.43500	75.16176	-1855.14608	88.94118	1993.53760	1
H	88.27241	78.95255	76.10877	-1504.82350	-780.94249	-2132.90992	1
C	85.29392	78.57253	77.01063	288.57638	618.62814	-397.00270	1
O	85.24900	79.77469	77.10527	321.70495	-185.26452	574.37218	1
N	84.49383	77.76775	76.31998	-447.05792	-446.53431	367.76425	1
H	84.63182	76.72578	76.29800	350.31316	1975.63644	-1843.31522	1
C	83.62867	78.43194	75.39330	-511.23061	374.03917	-471.51480	1
H	83.20232	79.30260	75.94210	2234.79579	-898.70890	-1273.45190	1
C	84.55203	78.90286	74.17409	529.53781	-159.34528	563.72604	1
H	83.86202	79.39235	73.43802	654.59388	2347.94251	1217.51488	1
H	85.24363	79.71775	74.51596	-1526.55661	2103.42026	817.87394	1
O	85.35322	77.86989	73.52862	-554.84278	14.12789	399.58769	1
H	84.67619	77.16646	73.46509	1297.06236	2061.84854	1220.68812	1
C	82.55031	77.34166	74.84917	-662.15484	-412.13544	123.62687	1
O	82.86602	76.23352	74.48378	-540.01537	-317.05804	274.91456	1
N	81.35401	77.93776	74.75887	-202.76299	-690.00100	-131.49145	1
H	81.27151	78.89951	75.09040	-702.62132	-2617.37191	281.70413	1
C	80.21287	77.08170	74.86510	-491.73914	576.34187	222.73509	1

H	80.58431	76.11239	74.46075	1977.17978	-1823.58850	-434.68928	1
C	79.80832	76.97316	76.39702	-649.50797	-328.66843	306.11224	1
H	79.20421	77.85260	76.70710	2075.07803	1452.16490	-1004.45904	1
C	78.99009	75.65058	76.59190	-615.18735	-489.99689	-70.96352	1
H	78.92636	75.35650	77.66466	-1226.32661	-1724.38906	-1716.47170	1
H	77.94240	75.75093	76.22366	2381.64273	-969.84735	900.46860	1
H	79.45287	74.80072	76.03907	2122.74991	1702.23946	-141.36228	1
C	81.09465	76.88531	77.28826	-501.86074	-479.61276	-376.42482	1
H	80.84826	76.66630	78.35471	993.79261	-2246.95401	-1177.81305	1
H	81.79377	76.09124	76.93421	42.71279	-626.15391	-2651.37258	1
H	81.66911	77.84121	77.27466	-2026.39012	-1590.33709	-887.82922	1
C	79.02056	77.78776	73.99517	-672.89153	382.57394	-156.34072	1
O	79.14778	78.88784	73.50338	410.04627	221.86223	500.35950	1
N	77.97645	76.97310	73.96308	-378.11782	-443.79414	-441.10984	1
H	78.07344	76.10926	74.49140	2404.15973	-568.14894	1149.30085	1
C	76.86453	77.11272	73.07423	-544.54484	466.16019	331.29119	1
H	76.01915	76.91399	73.76822	-1409.80988	-170.17955	2325.32679	1
C	76.82253	78.57659	72.47541	-184.48284	116.78282	-758.89137	1
H	76.97499	79.32912	73.28558	1893.78641	1861.14652	611.04528	1
H	77.67437	78.71714	71.76893	-2170.79665	-974.19311	1327.50117	1
C	75.46455	78.83235	71.74660	389.51668	672.81878	-138.49152	1
H	75.27859	78.04674	70.97757	510.64577	1933.03327	1851.02484	1
H	74.61282	78.71841	72.45409	-2302.30869	-1105.72939	-948.89774	1
C	75.42055	80.25766	71.07573	-559.93427	-126.34590	542.30857	1
O	74.43804	80.60223	70.47048	-670.36003	-111.83166	76.36448	1
N	76.54484	80.98692	71.24837	-216.06729	-537.07037	446.51379	1
H	77.40821	80.76525	71.75775	-1251.96487	1074.12894	-2168.52500	1
H	76.67299	81.93417	70.87692	-2451.83023	-720.73851	944.84612	1
C	76.83225	76.01112	71.87361	546.24052	-563.88337	-85.11809	1
O	75.73191	75.84706	71.39960	71.70702	-307.40463	606.69673	1
N	77.97205	75.40884	71.50812	479.90863	-504.50129	222.86849	1
C	78.04379	74.65223	70.29003	75.51268	-600.91904	506.73700	1
H	77.38314	75.16835	69.55506	-2033.61562	1795.30103	254.89480	1
C	79.59860	74.68663	69.95040	523.79067	517.99274	284.45581	1
H	79.90952	73.85990	69.27292	1599.61665	-1127.00911	1895.98173	1
H	79.88846	75.63739	69.45027	-1881.83093	1275.82488	-1501.55016	1
C	80.22267	74.58606	71.37541	-600.05639	-404.05026	316.64485	1
H	80.18236	73.54248	71.76135	-2597.05260	496.36221	657.73249	1
H	81.28900	74.90324	71.42346	-1666.24518	1294.38985	-1723.90591	1
C	79.24988	75.54498	72.19121	493.57570	-129.95376	-602.56394	1
H	79.10598	75.25840	73.25751	-1469.08415	-637.84797	2204.22581	1
H	79.50471	76.63030	72.13170	-1038.97840	1068.88701	-2280.71789	1
C	77.60303	73.10316	70.51637	188.88100	241.05089	-727.87821	1
O	77.66610	72.31131	69.58434	531.88613	-49.19943	427.07960	1
N	77.18755	72.87036	71.75290	708.14833	26.93417	-179.73509	1
H	77.27112	73.70365	72.33040	-2232.69501	-1179.09831	1023.94816	1
C	77.15621	71.50700	72.31324	-738.32412	259.92548	104.42456	1
H	78.03803	71.02571	71.82679	2028.89123	1260.02983	1311.33324	1
C	77.22831	71.58923	73.82371	-258.65678	-345.52513	661.28459	1
H	76.29525	72.10323	74.17661	-1826.06090	-1801.46097	-918.64554	1
C	77.27493	70.20704	74.45051	-541.23400	531.92970	-218.41428	1
H	77.30394	70.25936	75.57341	1311.73218	-2219.18200	882.19023	1
H	76.37075	69.59748	74.16599	-1048.71298	1223.58118	-2196.97971	1
H	78.18388	69.63234	74.12196	419.06414	1903.92898	1903.44670	1
C	78.40617	72.40901	74.31909	-423.86431	152.66566	-648.55262	1
H	78.43295	72.44279	75.44173	105.68386	-2342.50080	1387.51238	1
H	79.38750	71.98126	73.97478	-1695.63603	-1484.73621	-1531.02793	1
H	78.35987	73.47124	73.95672	-1853.57121	-487.39031	1936.59343	1
C	75.72958	70.82487	71.84336	539.71428	-510.58232	267.58633	1
O	74.89667	70.51189	72.67946	-15.93758	-278.34085	-624.49402	1
N	75.73117	70.64884	70.54931	-676.81886	12.05292	276.17960	1
H	76.53658	71.02302	69.99382	-1694.44250	-373.84189	-2100.66098	1

C	74.62758	70.09479	69.84875	412.33078	-664.70360	108.35451	1
H	75.03453	69.94573	68.82308	1765.77476	1294.44366	1621.76594	1
C	74.17529	68.75008	70.55810	-179.66635	-721.95667	-264.73915	1
H	75.04746	68.08275	70.73753	-607.34922	2587.87032	-598.10018	1
H	73.74729	68.95869	71.56679	-359.69332	-1479.13954	-2259.74260	1
C	73.11774	68.06833	69.62987	-240.01076	616.02425	431.85322	1
H	72.27271	68.76287	69.42240	-1359.17131	782.54425	2227.99181	1
H	73.56322	67.82588	68.63924	-820.36667	85.81767	-2596.78715	1
C	72.59856	66.75620	70.34548	-513.07015	236.18570	-551.87280	1
H	73.44608	66.07781	70.59642	1816.11944	-1183.11722	-1650.94321	1
H	72.10776	66.99964	71.31582	-1800.04429	-1361.53473	1526.34643	1
C	71.60694	66.06256	69.38298	726.08972	-253.19732	-179.64713	1
H	70.68393	66.63997	69.17605	1224.27271	-456.51798	-2390.90304	1
H	72.05619	65.71539	68.43115	1206.40339	-1150.93514	-2154.90344	1
N	71.10610	64.75085	70.07746	329.70796	-633.68968	-155.67358	1
H	70.42769	64.16382	69.51130	1348.31305	1938.76845	1359.00643	1
H	71.84165	64.02892	70.33105	2096.92035	1446.54551	-966.48890	1
H	70.57772	64.85570	70.99205	-1197.07217	-2303.53719	827.28733	1
C	73.35175	71.12678	69.83682	-378.01186	-556.17301	-413.96481	1
O	72.83440	71.39400	68.77851	-409.44433	-307.55053	-453.30833	1
N	72.96976	71.50956	71.04927	-429.02477	524.70884	274.08502	1
H	73.44836	71.16681	71.91049	1706.34263	-1425.96036	1574.38926	1
C	71.80832	72.36356	71.05679	-45.25864	-626.22952	478.93267	1
H	72.07452	73.06460	70.23231	1988.63164	570.90773	1772.87287	1
C	70.57398	71.46261	70.64481	551.77128	-20.49526	-564.54996	1
H	70.82884	70.85126	69.74667	1434.61535	-1753.10253	1513.99471	1
H	70.31270	70.74161	71.45191	-714.67738	1819.09770	1898.36494	1
C	69.39133	72.44229	70.35067	446.99263	-555.77305	338.97221	1
H	69.15246	73.02424	71.26890	1953.68466	1892.25957	-161.67766	1
H	69.68313	73.18975	69.57931	-403.03785	2688.07032	-188.43591	1
C	68.12904	71.62058	69.86846	567.86262	335.74681	434.04421	1
H	68.35702	71.04290	68.94335	-1080.02275	1568.26081	-1948.78900	1
H	67.81988	70.86909	70.63046	-919.54108	2111.34692	1456.13463	1
C	67.00710	72.65497	69.61638	195.81897	173.33862	-745.11536	1
H	66.66830	73.19500	70.52149	1405.67949	-2301.21089	-390.08113	1
H	67.22209	73.37876	68.80498	-940.37328	-1118.32446	2299.72133	1
N	65.74097	71.87782	69.12328	319.26422	656.36007	-42.02424	1
H	64.88891	72.47600	68.91904	1961.03633	619.91968	1787.09422	1
H	65.83466	71.33572	68.21543	854.14343	-2156.80752	1429.08711	1
H	65.33256	71.14682	69.77558	1237.02905	-1378.50857	-1998.28500	1
C	71.63775	73.07288	72.51308	532.53319	-268.57236	517.55729	1
O	70.62757	72.90077	73.19114	266.16714	-290.73804	-558.88002	1
N	72.65675	73.86913	72.73588	-226.25352	640.49678	-270.32839	1
H	73.40012	73.86156	72.03961	2007.69063	-1035.27229	1523.50231	1
C	72.82641	74.47609	74.02126	-540.76542	-573.33303	49.50000	1
H	72.52987	73.66002	74.71760	-2374.20950	-1036.76023	843.75774	1
C	74.34373	74.93251	74.11103	-77.09082	666.87422	-415.84136	1
H	74.50395	75.71681	73.31963	-985.81834	617.19825	2463.91927	1
O	75.32539	73.89955	73.83997	293.29899	-391.65602	-477.80926	1
H	75.64768	74.18447	72.95920	2528.72714	-681.76262	-751.27122	1
C	74.45487	75.57134	75.54284	252.47363	-605.14473	440.05134	1
H	75.46571	76.00880	75.71577	-2052.65063	1456.49051	-1043.51830	1
H	73.71018	76.38909	75.69970	-2152.98800	-1661.97574	-161.70652	1
H	74.28511	74.80961	76.33917	-473.12726	-1584.27339	2165.61725	1
C	71.81497	75.75691	74.12742	360.36307	488.46713	505.10056	1
O	72.23376	76.89092	74.13150	-200.08817	-528.03552	-385.82850	1
N	70.56036	75.33433	74.24402	-597.54095	-226.44775	355.20479	1
H	70.31347	74.31731	74.16107	2179.23655	-1300.70981	-991.33835	1
C	69.58442	76.33187	74.46668	-687.68921	297.39807	-249.45179	1
H	70.12685	77.30567	74.53498	-349.16256	-2467.37713	-1101.72831	1
C	68.50314	76.25320	73.27981	672.47345	361.54328	-201.62880	1
H	68.07921	75.22627	73.24233	1541.41502	582.18425	2169.97026	1

203	H	67.65674	76.94623	73.48846	-2534.67717	60.25153	997.72295	1
204	C	69.17281	76.60016	71.94511	574.26774	-389.43119	-377.02542	1
205	C	69.01294	77.88977	71.35343	688.82679	-287.33580	-257.96133	1
206	H	68.41302	78.67069	71.85422	331.14182	1470.38163	2269.80013	1
207	C	69.98014	75.61392	71.30144	-383.52382	-675.04808	144.24917	1
208	H	70.14460	74.63381	71.78454	4.09847	1517.59662	2262.86397	1
209	C	69.62953	78.14228	70.12845	-134.76730	428.45285	-649.50303	1
210	H	69.50861	79.14077	69.67290	-903.23433	1600.49020	2011.53387	1
211	C	70.57543	75.91034	70.07369	-335.53281	-615.00040	-364.39058	1
212	H	71.19499	75.13468	69.59009	1596.45398	-1369.33862	1732.02659	1
213	C	70.41640	77.17574	69.43646	585.48547	-340.24194	-406.23938	1
214	O	70.99712	77.48537	68.17920	-419.15649	-173.04408	-511.94141	1
215	H	71.52053	76.66260	68.02946	-1071.84350	1559.71507	-1960.13012	1
216	C	68.73430	75.89346	75.79657	291.41878	-183.13091	710.72246	1
217	O	68.24351	74.78141	75.91536	379.87695	523.47396	-222.23622	1
218	N	68.58796	76.94637	76.58142	564.30591	373.09380	277.24371	1
219	H	69.03899	77.80889	76.28167	-120.59931	-2436.07824	-1214.35098	1
220	C	68.19878	76.75682	77.94753	404.07514	-299.37480	608.84009	1
221	H	68.55958	75.72934	78.18035	-1071.07882	-841.69025	2359.66467	1
222	C	68.88630	77.91020	78.78105	-683.12807	68.71770	-390.13096	1
223	H	69.98617	77.92013	78.60737	2068.88918	1596.58590	770.89603	1
224	H	68.52081	78.90389	78.43670	1379.86429	2342.49779	-179.85638	1
225	C	68.56559	77.70564	80.29521	-176.39967	537.49466	550.97268	1
226	H	67.45924	77.63334	80.45422	-396.28051	1942.22168	1869.33314	1
227	H	68.99230	76.75029	80.67501	-94.44700	586.00678	-2659.20016	1
228	C	69.17079	78.96228	81.07317	-291.69381	-718.18079	-150.72929	1
229	H	70.28596	79.06257	81.05612	-2303.38776	1089.76242	964.62032	1
230	H	68.73082	79.95200	80.78825	884.16729	2568.80533	-207.74283	1
231	N	68.77355	78.68768	82.39031	-594.49522	-252.81478	342.29276	1
232	H	68.13720	77.91634	82.52085	-1735.17426	2084.05705	-263.71605	1
233	C	69.14334	79.34721	83.41243	595.23177	-271.52487	442.22352	1
234	N	70.01730	80.42189	83.21642	585.53813	-424.79750	-105.81303	1
235	H	70.40962	80.78661	82.34939	959.88797	1327.56823	-2177.11905	1
236	H	70.37302	81.02836	83.95610	1578.48647	16.27378	-2220.76233	1
237	N	68.56446	78.83003	84.57998	-452.17763	-390.33541	-421.51853	1
238	H	67.92276	78.05074	84.71785	470.26107	1974.25629	-1817.92087	1
239	H	68.73106	79.22173	85.50760	1856.67307	-1573.97321	-1224.35416	1
240	C	66.57319	76.84388	78.04372	-531.47271	-581.45780	-55.06206	1
241	O	66.00416	77.37763	78.97152	-243.00837	192.73507	609.52485	1
242	N	66.04308	76.18281	77.02409	343.49288	-644.25607	38.09164	1
243	H	66.67571	75.73908	76.33669	-1691.16043	-1535.97525	-1484.73099	1
244	C	64.63788	76.33521	76.78808	542.66492	350.14337	454.42563	1
245	H	64.21938	76.21571	77.81272	1209.57979	-2396.95920	463.86744	1
246	C	64.31668	77.77757	76.21134	-737.14309	145.93010	242.71942	1
247	H	64.80660	77.92134	75.22446	-1050.18303	159.36419	-2509.06125	1
248	C	62.75846	77.89422	76.09289	-706.77696	-252.88343	-245.16230	1
249	H	62.44658	78.89765	75.71911	-1624.64039	-1665.49085	1417.86935	1
250	H	62.34796	77.13146	75.38548	2566.10521	598.51109	693.22305	1
251	H	62.25887	77.73797	77.07785	-1972.69758	107.10018	-1876.34312	1
252	C	64.76494	78.89729	77.21591	412.85148	672.58832	-27.69222	1
253	H	64.41902	79.90678	76.88835	1447.02277	1762.76533	-1490.78944	1
254	H	64.35077	78.71891	78.23850	1320.77379	-145.20455	2378.68491	1
255	H	65.87653	78.94292	77.31133	-1512.10848	1470.52031	1724.75147	1
256	C	64.24437	75.15941	75.73194	-607.11113	499.56689	73.73769	1
257	O	63.57421	75.36641	74.74158	357.97126	460.81534	356.68785	1
258	N	64.75507	74.00952	76.16233	536.55407	-368.62270	332.76306	1
259	H	65.30597	74.00724	77.02110	524.38866	2297.69985	1367.20941	1
260	C	64.44422	72.82006	75.46448	46.50277	-774.29190	-147.97904	1
261	H	63.66514	73.11131	74.72282	1676.51875	1883.29066	1032.55549	1
262	C	65.79411	72.24920	74.77810	-559.41456	-34.19198	-556.30433	1
263	H	66.52819	71.97381	75.57292	-64.66664	395.18769	2695.05373	1
264	H	65.57527	71.30678	74.22218	-2703.44278	-60.40056	333.79418	1

```
C    66.38268  73.28828  73.86528     -519.76435      377.31045      459.42366          1
H    66.62378  74.19003  74.47729    -1746.74323     1667.50644    -1261.73691          1
C    67.66618  72.77031  73.22844      570.95554       16.46261     -545.27700          1
H    68.03932  73.46710  72.43416     2391.58273       28.05277    -1305.07271          1
H    68.49580  72.66003  73.97836      175.75588      917.63458     2559.43867          1
H    67.51638  71.76999  72.74670    -1063.97851    -2155.34650     1283.00462          1
C    65.40501  73.70793  72.77676      193.76014        2.62946     -765.53141          1
H    65.87546  74.42234  72.05338      507.39394     -846.55087     2539.60089          1
H    65.03573  72.83007  72.18584     1337.57841     1818.19054     1526.01994          1
H    64.50734  74.23060  73.21739     1100.88635    -1172.91350    -2199.08909          1
C    63.97463  71.71445  76.54246     -549.21115      475.64486     -309.38218          1
O    64.13322  71.82615  77.72961      556.06106     -390.37755      -78.24250          1
O    63.43517  70.52467  75.99603     -572.27940     -310.19032     -209.75862          1
H    63.45086  70.77816  75.03216    -1380.08193     2212.14486      790.85944          1
```

Listing 12: C00012.xmolout

```
       276
  150.0000  150.0000  150.0000     0.0000     0.0000     0.0000***********    2.7750    11100
       0.2500    300.00    0.00    0.00    0.00   -1294.1313       0.0000    300.0000
H    72.22800  83.22922  77.91478    -2043.14101    -1757.64893      399.89339          1
N    73.05475  82.87942  77.42850      426.75047      490.45733     -334.42467          1
H    72.67606  82.41107  76.58675      442.25559    -1110.30854    -2448.52907          1
C    73.97302  83.89811  77.09135      415.32972     -666.39213       83.73172          1
H    73.69882  84.81006  77.66589      879.32219     2491.00447      667.35213          1
C    75.47375  83.39081  77.39992       31.35357      477.41777      628.23362          1
H    75.68965  82.47917  76.79403     1168.38489     2240.25337    -1019.71136          1
H    76.22244  84.15419  77.07955     -360.43770     2534.90498      931.67112          1
C    75.59409  83.09545  78.87074      463.18208      512.70242     -382.34388          1
H    74.70517  82.50045  79.18068     1725.29117     1863.52247     -987.07854          1
C    76.84718  82.27629  79.15420      710.42801     -144.27427     -313.15367          1
H    76.94981  82.04341  80.24530      364.20195    -1033.13725     2494.72524          1
H    76.84252  81.29693  78.60868     2567.61904      335.80679      847.48802          1
H    77.77459  82.82326  78.84455    -2587.99029      680.92867     -512.16411          1
C    75.62802  84.37814  79.69148      268.17616      741.64379      -40.42634          1
H    75.72659  84.16476  80.78704     1283.54314    -1945.37420    -1411.27812          1
H    76.49204  85.03021  79.40260    -1258.57796     1317.15249    -2026.02085          1
H    74.69546  84.98355  79.55549    -2502.84526      909.80597      575.92391          1
C    73.74348  84.00209  75.49012      619.36578     -458.64843     -172.09273          1
O    73.02744  83.18213  74.92449      -81.40433      433.87611      522.34594          1
N    74.44879  84.99947  74.97420      175.23996     -622.20190      341.55523          1
H    75.00105  85.57891  75.60693     -876.57256     -303.22879    -2561.90213          1
C    74.57326  85.12414  73.56866     -571.88214     -534.10783     -106.15033          1
H    73.89865  84.33417  73.16119    -2713.20841      -16.25800     -248.80809          1
C    74.19591  86.62868  73.15513      166.60354      288.16399     -716.09568          1
H    75.00031  87.30755  73.55269    -2097.85574     1636.78449     -586.18044          1
O    72.94525  87.12348  73.65827     -317.80580     -363.38227      484.43082          1
H    73.24546  87.86134  74.22717     1863.74748    -1451.84923     1357.29467          1
C    74.30611  86.55874  71.58789      503.80731      517.99032      318.51606          1
H    74.14849  87.56059  71.12477      640.61020    -2157.10410    -1536.29104          1
H    75.30825  86.19249  71.25688     1687.88455      465.60692     2087.56452          1
H    73.54150  85.87100  71.15676    -1293.07895    -1899.88581    -1463.57452          1
C    76.16688  84.94766  73.22910     -153.07550     -733.68902      248.70981          1
O    76.80036  85.93891  72.96930      186.15459     -480.78787     -449.34387          1
N    76.58479  83.67738  73.31952     -487.53139      543.18517       42.03850          1
C    77.95999  83.32215  73.15354     -699.35871       69.34393      360.10701          1
H    78.55698  84.20482  73.47950        2.81126     -221.99807    -2715.58072          1
C    78.03494  82.02109  74.06018     -507.46439     -592.59194      122.07761          1
H    78.93561  81.40090  73.85609    -1975.08116      525.87560    -1801.71556          1
H    78.05462  82.27760  75.14272     1588.27743    -1913.22100     1113.83566          1
C    76.69729  81.31589  73.66485     -613.62442     -457.55993     -194.14490          1
H    76.80557  80.78712  72.68752     1707.54637     -721.27992     1996.92523          1
```

H	76.36159	80.55308	74.40530	758.23357	2100.81767	1560.55011	1
C	75.69694	82.54270	73.53177	-352.41524	704.96076	49.21465	1
H	75.01758	82.50561	72.63245	1714.76886	-2070.42778	443.35792	1
H	75.10177	82.77314	74.45036	1224.15040	1801.50881	1636.97633	1
C	78.16001	82.94289	71.56645	566.85117	537.05831	117.62814	1
O	78.26157	81.76756	71.22646	-550.26779	-406.08867	4.21666	1
N	78.17274	84.04515	70.85978	566.34832	-425.77725	180.18749	1
H	78.00728	84.89367	71.40940	644.44502	2646.80550	-52.73430	1
C	78.37813	84.12521	69.46200	612.34801	-281.48662	411.56221	1
H	78.52380	85.21293	69.27815	1667.23498	-1490.75142	1556.16753	1
C	79.65293	83.23767	69.06333	-714.45746	-175.43074	286.98927	1
H	79.49984	82.18955	69.45024	-787.09484	1763.03619	-1922.46060	1
O	80.91561	83.67607	69.58896	-429.46514	516.51932	-128.40526	1
H	81.15435	82.91881	70.16208	1981.82785	436.16847	-1818.18182	1
C	79.50237	83.23566	67.49842	630.79189	-468.94448	76.03230	1
H	80.19984	82.51093	67.01728	-1775.71861	2062.30353	131.89849	1
H	78.45893	82.96281	67.19332	-1855.14608	88.94118	1993.53760	1
H	79.71912	84.24098	67.06771	-1504.82350	-780.94249	-2132.90992	1
C	77.07122	83.55448	68.65057	288.57638	618.62814	-397.00270	1
O	76.62722	84.21528	67.74082	321.70495	-185.26452	574.37218	1
N	76.74108	82.32247	69.02689	-447.05792	-446.53431	367.76425	1
H	77.25417	81.85775	69.81417	350.31316	1975.63644	-1843.31522	1
C	75.44684	81.87392	68.62746	-511.23061	374.03917	-471.51480	1
H	75.10881	82.69587	67.95046	2234.79579	-898.70890	-1273.45190	1
C	75.69232	80.49666	67.85014	529.53781	-159.34528	563.72604	1
H	74.74838	80.24712	67.29511	654.59388	2347.94251	1217.51488	1
H	76.47903	80.70230	67.07765	-1526.55661	2103.42026	817.87394	1
O	76.05463	79.33640	68.65469	-554.84278	14.12789	399.58769	1
H	76.99558	79.54083	68.80566	1297.06236	2061.84854	1220.68812	1
C	74.53913	81.77244	69.97365	-662.15484	-412.13544	123.62687	1
O	74.42420	82.71802	70.72747	-540.01537	-317.05804	274.91456	1
N	73.85783	80.62272	70.00418	-202.76299	-690.00100	-131.49145	1
H	74.14645	79.91801	69.31929	-702.62132	-2617.37191	281.70413	1
C	73.38157	80.22113	71.29083	-491.73914	576.34187	222.73509	1
H	73.26547	81.15899	71.88106	1977.17978	-1823.58850	-434.68928	1
C	72.03786	79.41007	71.06251	-649.50797	-328.66843	306.11224	1
H	72.17537	78.62805	70.28491	2075.07803	1452.16490	-1004.45904	1
C	71.58855	78.76585	72.41860	-615.18735	-489.99689	-70.96352	1
H	70.61182	78.23603	72.31723	-1226.32661	-1724.38906	-1716.47170	1
H	72.33517	78.01227	72.77545	2381.64273	-969.84735	900.46860	1
H	71.47433	79.53064	73.22238	2122.74991	1702.23946	-141.36228	1
C	70.92912	80.42685	70.62170	-501.86074	-479.61276	-376.42482	1
H	69.95823	79.91651	70.41955	993.79261	-2246.95401	-1177.81305	1
H	70.74530	81.19669	71.40745	42.71279	-626.15391	-2651.37258	1
H	71.22451	80.96741	69.69180	-2026.39012	-1590.33709	-887.82922	1
C	74.51102	79.20374	71.91741	-672.89153	382.57394	-156.34072	1
O	74.99328	79.43213	73.00412	410.04627	221.86223	500.35950	1
N	74.74223	78.20232	71.08517	-378.11782	-443.79414	-441.10984	1
H	74.24051	78.20483	70.20000	2404.15973	-568.14894	1149.30085	1
C	75.54029	77.07462	71.47260	-544.54484	466.16019	331.29119	1
H	75.53670	76.48741	70.52513	-1409.80988	-170.17955	2325.32679	1
C	76.97724	77.70490	71.77078	-184.48284	116.78282	-758.89137	1
H	77.00597	78.74241	71.36871	1893.78641	1861.14652	611.04528	1
H	77.16618	77.80346	72.86411	-2170.79665	-974.19311	1327.50117	1
C	78.05524	76.83031	71.06300	389.51668	672.81878	-138.49152	1
H	78.04025	75.78362	71.43539	510.64577	1933.03327	1851.02484	1
H	77.80512	76.72932	69.98364	-2302.30869	-1105.72939	-948.89774	1
C	79.51300	77.41087	71.20821	-559.93427	-126.34590	542.30857	1
O	80.45203	76.73731	70.87251	-670.36003	-111.83166	76.36448	1
N	79.56450	78.67701	71.67777	-216.06729	-537.07037	446.51379	1
H	78.81015	79.33869	71.89659	-1251.96487	1074.12894	-2168.52500	1
H	80.43432	79.21547	71.75056	-2451.83023	-720.73851	944.84612	1

C	74.68598	76.33309	72.60660	546.24052	-563.88337	-85.11809	1
O	73.47401	76.18807	72.41280	71.70702	-307.40463	606.69673	1
N	75.39569	75.78947	73.58347	479.90863	-504.50129	222.86849	1
C	74.70043	75.21822	74.70701	75.51268	-600.91904	506.73700	1
H	73.66086	75.00909	74.36051	-2033.61562	1795.30103	254.89480	1
C	75.58863	73.94395	75.02254	523.79067	517.99274	284.45581	1
H	75.44927	73.56948	76.06310	1599.61665	-1127.00911	1895.98173	1
H	75.34191	73.10016	74.33943	-1881.83093	1275.82488	-1501.55016	1
C	77.02120	74.51404	74.75351	-600.05639	-404.05026	316.64485	1
H	77.33360	75.20784	75.56998	-2597.05260	496.36221	657.73249	1
H	77.82494	73.73981	74.65779	-1666.24518	1294.38985	-1723.90591	1
C	76.76629	75.30450	73.40510	493.57570	-129.95376	-602.56394	1
H	77.44196	76.17736	73.25422	-1469.08415	-637.84797	2204.22581	1
H	76.75029	74.67367	72.48594	-1038.97840	1068.88701	-2280.71789	1
C	74.74472	76.14642	76.05211	188.88100	241.05089	-727.87821	1
O	74.04638	75.80970	77.01225	531.88613	-49.19943	427.07960	1
N	75.64811	77.10419	76.00018	708.14833	26.93417	-179.73509	1
H	76.06016	77.09303	75.06734	-2232.69501	-1179.09831	1023.94816	1
C	75.71511	78.26701	76.86691	-738.32412	259.92548	104.42456	1
H	76.42089	78.94158	76.32616	2028.89123	1260.02983	1311.33324	1
C	74.31574	78.85250	76.99152	-258.65678	-345.52513	661.28459	1
H	73.63702	78.02954	77.31486	-1826.06090	-1801.46097	-918.64554	1
C	74.25605	79.95690	78.02996	-541.23400	531.92970	-218.41428	1
H	73.21004	80.34816	78.16655	1311.73218	-2219.18200	882.19023	1
H	74.61969	79.60469	79.03521	-1048.71298	1223.58118	-2196.97971	1
H	74.89465	80.83236	77.73356	419.06414	1903.92898	1903.44670	1
C	73.80844	79.37280	75.65908	-423.86431	152.66566	-648.55262	1
H	72.72217	79.66002	75.70616	105.68386	-2342.50080	1387.51238	1
H	74.37486	80.28546	75.32429	-1695.63603	-1484.73621	-1531.02793	1
H	73.92037	78.60183	74.84441	-1853.57121	-487.39031	1936.59343	1
C	76.32240	77.85194	78.36607	539.71428	-510.58232	267.58633	1
O	77.16319	78.56792	78.84143	-15.93758	-278.34085	-624.49402	1
N	75.74025	76.78949	78.88514	-676.81886	12.05292	276.17960	1
H	74.96214	76.30404	78.36981	-1694.44250	-373.84189	-2100.66098	1
C	76.14471	76.36884	80.17998	412.33078	-664.70360	108.35451	1
H	76.99397	77.03947	80.45777	1765.77476	1294.44366	1621.76594	1
C	74.88180	76.53163	81.13328	-179.66635	-721.95667	-264.73915	1
H	74.39476	77.52072	80.98500	-607.34922	2587.87032	-598.10018	1
H	74.10842	75.77651	80.86911	-359.69332	-1479.13954	-2259.74260	1
C	75.34599	76.35090	82.61399	-240.01076	616.02425	431.85322	1
H	76.01690	75.45472	82.69829	-1359.17131	782.54425	2227.99181	1
H	75.95099	77.22170	82.95392	-820.36667	85.81767	-2596.78715	1
C	74.07395	76.14801	83.53319	-513.07015	236.18570	-551.87280	1
H	73.45722	77.07462	83.57842	1816.11944	-1183.11722	-1650.94321	1
H	73.40703	75.35825	83.11721	-1800.04429	-1361.53473	1526.34643	1
C	74.58283	75.74042	84.93535	726.08972	-253.19732	-179.64713	1
H	75.13909	74.78187	84.96136	1224.27271	-456.51798	-2390.90304	1
H	75.15864	76.52517	85.46513	1206.40339	-1150.93514	-2154.90344	1
N	73.33286	75.48261	85.84294	329.70796	-633.68968	-155.67358	1
H	73.54570	75.17379	86.83525	1348.31305	1938.76845	1359.00643	1
H	72.68023	76.30163	86.01524	2096.92035	1446.54551	-966.48890	1
H	72.65829	74.72473	85.53104	-1197.07217	-2303.53719	827.28733	1
C	76.50774	74.77604	80.19456	-378.01186	-556.17301	-413.96481	1
O	76.66871	74.18743	81.24692	-409.44433	-307.55053	-453.30833	1
N	76.53011	74.21285	78.96134	-429.02477	524.70884	274.08502	1
H	76.59556	74.98613	78.29949	1706.34263	-1425.96036	1574.38926	1
C	77.34451	73.00201	78.91075	-45.25864	-626.22952	478.93267	1
H	78.17289	73.29196	79.59501	1988.63164	570.90773	1772.87287	1
C	76.47858	71.83300	79.51645	551.77128	-20.49526	-564.54996	1
H	75.92375	72.19315	80.41473	1434.61535	-1753.10253	1513.99471	1
H	75.69663	71.51020	78.79135	-714.67738	1819.09770	1898.36494	1
C	77.42317	70.64066	79.89425	446.99263	-555.77305	338.97221	1

H	78.09785	70.37520	79.05034	1953.68466	1892.25957	-161.67766	1
H	78.09126	70.90946	80.74274	-403.03785	2688.07032	-188.43591	1
C	76.48532	69.42356	80.26417	567.86262	335.74681	434.04421	1
H	75.94032	69.62044	81.21605	-1080.02275	1568.26081	-1948.78900	1
H	75.69864	69.28456	79.48624	-919.54108	2111.34692	1456.13463	1
C	77.35492	68.14898	80.37442	195.81897	173.33862	-745.11536	1
H	77.74649	67.77731	79.40668	1405.67949	-2301.21089	-390.08113	1
H	78.17524	68.20668	81.11764	-940.37328	-1118.32446	2299.72133	1
N	76.41907	67.01374	80.90924	319.26422	656.36007	-42.02424	1
H	76.84054	66.04789	81.03968	1961.03633	619.91968	1787.09422	1
H	75.99145	67.17910	81.86497	854.14343	-2156.80752	1429.08711	1
H	75.55951	66.80013	80.32535	1237.02905	-1378.50857	-1998.28500	1
C	77.82392	72.80819	77.38518	532.53319	-268.57236	517.55729	1
O	78.38482	73.73172	76.85118	266.16714	-290.73804	-558.88002	1
N	77.64880	71.55531	76.93265	-226.25352	640.49678	-270.32839	1
H	77.13997	70.85088	77.51793	2007.69063	-1035.27229	1523.50231	1
C	77.31433	71.40887	75.53623	-540.76542	-573.33303	49.50000	1
H	76.98745	72.40321	75.16344	-2374.20950	-1036.76023	843.75774	1
C	78.63547	70.93352	74.77558	-77.09082	666.87422	-415.84136	1
H	79.20533	70.26918	75.47816	-985.81834	617.19825	2463.91927	1
O	79.51301	72.02063	74.41521	293.29899	-391.65602	-477.80926	1
H	80.24633	71.88768	75.04455	2528.72714	-681.76262	-751.27122	1
C	78.14156	70.07734	73.55401	252.47363	-605.14473	440.05134	1
H	78.99997	69.62554	73.00471	-2052.65063	1456.49051	-1043.51830	1
H	77.46808	69.23831	73.86446	-2152.98800	-1661.97574	-161.70652	1
H	77.58202	70.70295	72.82056	-473.12726	-1584.27339	2165.61725	1
C	76.12039	70.32280	75.66118	360.36307	488.46713	505.10056	1
O	75.93292	69.77984	76.74592	-200.08817	-528.03552	-385.82850	1
N	75.46317	70.19500	74.52457	-597.54095	-226.44775	355.20479	1
H	75.77223	70.73659	73.71888	2179.23655	-1300.70981	-991.33835	1
C	74.40538	69.24583	74.39537	-687.68921	297.39807	-249.45179	1
H	73.72066	69.41987	75.25506	-349.16256	-2467.37713	-1101.72831	1
C	73.75664	69.45291	72.94891	672.47345	361.54328	-201.62880	1
H	74.53800	69.35195	72.16092	1541.41502	582.18425	2169.97026	1
H	72.99685	68.65947	72.76222	-2534.67717	60.25153	997.72295	1
C	73.11464	70.84624	72.90073	574.26774	-389.43119	-377.02542	1
C	71.82584	71.03733	73.47702	688.82679	-287.33580	-257.96133	1
H	71.27987	70.19306	73.93641	331.14182	1470.38163	2269.80013	1
C	73.81305	71.94254	72.31421	-383.52382	-675.04808	144.24917	1
H	74.81206	71.80228	71.86272	4.09847	1517.59662	2262.86397	1
C	71.27921	72.31602	73.45642	-134.76730	428.45285	-649.50303	1
H	70.28118	72.46034	73.90508	-903.23433	1600.49020	2011.53387	1
C	73.20697	73.19826	72.32722	-335.53281	-615.00040	-364.39058	1
H	73.76070	74.03517	71.86411	1596.45398	-1369.33862	1732.02659	1
C	71.92930	73.44576	72.89739	585.48547	-340.24194	-406.23938	1
O	71.32595	74.69979	72.92911	-419.15649	-173.04408	-511.94141	1
H	72.14642	75.32283	72.70952	-1071.84350	1559.71507	-1960.13012	1
C	75.03338	67.74869	74.39419	291.41878	-183.13091	710.72246	1
O	76.06640	67.45271	73.84053	379.87695	523.47396	-222.23622	1
N	74.11215	66.92284	74.92282	564.30591	373.09380	277.24371	1
H	73.30686	67.34168	75.39405	-120.59931	-2436.07824	-1214.35098	1
C	74.64901	65.68802	75.42708	404.07514	-299.37480	608.84009	1
H	75.38693	65.41941	74.63564	-1071.07882	-841.69025	2359.66467	1
C	75.35607	65.98184	76.81150	-683.12807	68.71770	-390.13096	1
H	75.99717	66.89162	76.75218	2068.88918	1596.58590	770.89603	1
H	74.60173	66.19208	77.60365	1379.86429	2342.49779	-179.85638	1
C	76.19318	64.70867	77.14955	-176.39967	537.49466	550.97268	1
H	75.54682	63.79833	77.11141	-396.28051	1942.22168	1869.33314	1
H	76.99722	64.54366	76.39792	-94.44700	586.00678	-2659.20016	1
C	76.80221	64.90519	78.61335	-291.69381	-718.18079	-150.72929	1
H	77.48640	65.77857	78.75413	-2303.38776	1089.76242	964.62032	1
H	76.02563	64.91469	79.42003	884.16729	2568.80533	-207.74283	1

```
N    77.54179   63.72325   78.75263      -594.49522      -252.81478       342.29276          1
H    77.23468   62.93529   78.20229     -1735.17426      2084.05705      -263.71605          1
C    78.53211   63.56434   79.53551       595.23177      -271.52487       442.22352          1
N    78.93864   64.65027   80.31641       585.53813      -424.79750      -105.81303          1
H    78.56513   65.59703   80.36519       959.88797      1327.56823     -2177.11905          1
H    79.71331   64.63010   80.98045      1578.48647        16.27378     -2220.76233          1
N    79.02462   62.25530   79.44485      -452.17763      -390.33541      -421.51853          1
H    78.72611   61.46647   78.87303       470.26107      1974.25629     -1817.92087          1
H    79.81732   61.91680   79.99136      1856.67307     -1573.97321     -1224.35416          1
C    73.42630   64.62170   75.62482      -531.47271      -581.45780       -55.06206          1
O    73.45859   63.81934   76.53797      -243.00837       192.73507       609.52485          1
N    72.58121   64.69588   74.60997       343.49288      -644.25607        38.09164          1
H    72.79159   65.42873   73.93348     -1691.16043     -1535.97525     -1484.73099          1
C    71.39225   63.91994   74.54729       542.66492       350.14337       454.42563          1
H    71.18553   63.89565   73.45465      1209.57979     -2396.95920       463.86744          1
C    71.62924   62.49301   75.19549      -737.14309       145.93010       242.71942          1
H    71.85684   62.59014   76.28974     -1050.18303       159.36419     -2509.06125          1
C    70.35080   61.61023   74.97734      -706.77696      -252.88343      -245.16230          1
H    70.49357   60.57827   75.37570     -1624.64039     -1665.49085      1417.86935          1
H    69.46346   62.04669   75.49800      2566.10521       598.51109       693.22305          1
H    70.09285   61.51727   73.89661     -1972.69758       107.10018     -1876.34312          1
C    72.81035   61.78473   74.44281       412.85148       672.58832       -27.69222          1
H    73.00508   60.76439   74.84779      1447.02277      1762.76533     -1490.78944          1
H    72.59929   61.67852   73.35326      1320.77379      -145.20455      2378.68491          1
H    73.75467   62.36955   74.54530     -1512.10848      1470.52031      1724.75147          1
C    70.19981   64.72130   75.34635      -607.11113       499.56689        73.73769          1
O    69.57324   64.18946   76.23629       357.97126       460.81534       356.68785          1
N    69.98819   65.88193   74.74151       536.55407      -368.62270       332.76306          1
H    70.65464   66.12137   74.00906       524.38866      2297.69985      1367.20941          1
C    69.10900   66.86251   75.27424        46.50277      -774.29190      -147.97904          1
H    68.99694   67.58159   74.43435      1676.51875      1883.29066      1032.55549          1
C    67.71036   66.17226   75.68836      -559.41456       -34.19198      -556.30433          1
H    67.89960   65.39236   76.47577       -64.66664       395.18769      2695.05373          1
H    67.00551   66.91067   76.13963     -2703.44278       -60.40056       333.79418          1
C    67.10464   65.54325   74.46378      -519.76435       377.31045       459.42366          1
H    67.90087   64.96125   73.94639     -1746.74323      1667.50644     -1261.73691          1
C    65.98340   64.58408   74.84494       570.95554        16.46261      -545.27700          1
H    65.53716   64.09083   73.94309      2391.58273        28.05277     -1305.07271          1
H    66.34887   63.76999   75.52271       175.75588       917.63458      2559.43867          1
H    65.15156   65.11228   75.37797     -1063.97851     -2155.34650      1283.00462          1
C    66.56313   66.59717   73.50651       193.76014         2.62946      -765.53141          1
H    66.11245   66.13196   72.59232       507.39394      -846.55087      2539.60089          1
H    65.76535   67.21926   73.98784      1337.57841      1818.19054      1526.01994          1
H    67.36540   67.29724   73.15974      1100.88635     -1172.91350     -2199.08909          1
C    69.86677   67.57342   76.49781      -549.21115       475.64486      -309.38218          1
O    71.06449   67.69703   76.53389       556.06106      -390.37755       -78.24250          1
O    69.05637   68.24238   77.44940      -572.27940      -310.19032      -209.75862          1
H    68.16266   67.98597   77.10201     -1380.08193      2212.14486       790.85944          1
```

Listing 13: C00013.xmolout

```
        276
   150.0000  150.0000  150.0000     0.0000     0.0000     0.0000***********     2.7750    11100
         0.2500     300.00    0.00    0.00    0.00   -1294.1210       0.0000     300.0000
H    78.97967   60.06429   80.19138     -2043.14101     -1757.64893       399.89339          1
N    78.94044   60.45535   79.25218       426.75047       490.45733      -334.42467          1
H    77.96928   60.75758   79.20454       442.25559     -1110.30854     -2448.52907          1
C    79.87231   61.49996   79.04045       415.32972      -666.39213        83.73172          1
H    80.80267   61.05332   79.45327       879.32219      2491.00447       667.35213          1
C    79.98498   61.80571   77.46127        31.35357       477.41777       628.23362          1
H    79.04109   62.28645   77.10945      1168.38489      2240.25337     -1019.71136          1
H    80.79998   62.54143   77.25902      -360.43770      2534.90498       931.67112          1
```

C	80.23276	60.52630	76.70743	463.18208	512.70242	-382.34388	1
H	79.47307	59.77898	77.03061	1725.29117	1863.52247	-987.07854	1
C	80.08424	60.75677	75.20835	710.42801	-144.27427	-313.15367	1
H	80.23931	59.81164	74.62686	364.20195	-1033.13725	2494.72524	1
H	79.06627	61.14480	74.94679	2567.61904	335.80679	847.48802	1
H	80.83021	61.50304	74.83140	-2587.99029	680.92867	-512.16411	1
C	81.61929	59.96718	76.99749	268.17616	741.64379	-40.42634	1
H	81.81686	59.03024	76.41537	1283.54314	-1945.37420	-1411.27812	1
H	82.42375	60.69988	76.72983	-1258.57796	1317.15249	-2026.02085	1
H	81.74579	59.71552	78.08173	-2502.84526	909.80597	575.92391	1
C	79.35164	62.73416	79.94253	619.36578	-458.64843	-172.09273	1
O	78.86953	62.53698	81.06439	-81.40433	433.87611	522.34594	1
N	79.65823	63.87832	79.38590	175.23996	-622.20190	341.55523	1
H	80.10378	63.84312	78.47196	-876.57256	-303.22879	-2561.90213	1
C	79.00696	65.11101	79.74412	-571.88214	-534.10783	-106.15033	1
H	77.96523	65.03215	79.36703	-2713.20841	-16.25800	-248.80809	1
C	79.04559	65.22908	81.33677	166.60354	288.16399	-716.09568	1
H	79.91890	64.59636	81.66811	-2097.85574	1636.78449	-586.18044	1
O	77.91740	64.68153	82.01395	-317.80580	-363.38227	484.43082	1
H	78.16100	63.67180	81.95805	1863.74748	-1451.84923	1357.29467	1
C	79.34646	66.75049	81.58857	503.80731	517.99032	318.51606	1
H	79.52970	66.95904	82.66844	640.61020	-2157.10410	-1536.29104	1
H	80.24809	67.09215	81.02210	1687.88455	465.60692	2087.56452	1
H	78.49831	67.40171	81.26465	-1293.07895	-1899.88581	-1463.57452	1
C	79.87921	66.25359	78.99869	-153.07550	-733.68902	248.70981	1
O	81.08209	66.22715	79.07728	186.15459	-480.78787	-449.34387	1
N	79.09608	67.18520	78.43360	-487.53139	543.18517	42.03850	1
C	79.66531	68.39356	77.91406	-699.35871	69.34393	360.10701	1
H	80.65951	68.13554	77.47896	2.81126	-221.99807	-2715.58072	1
C	78.54402	68.81557	76.86290	-507.46439	-592.59194	122.07761	1
H	78.59488	69.89616	76.56993	-1975.08116	525.87560	-1801.71556	1
H	78.63179	68.22775	75.92171	1588.27743	-1913.22100	1113.83566	1
C	77.24078	68.45205	77.63469	-613.62442	-457.55993	-194.14490	1
H	77.00793	69.21902	78.40644	1707.54637	-721.27992	1996.92523	1
H	76.34389	68.37600	76.98005	758.23357	2100.81767	1560.55011	1
C	77.65266	67.06773	78.30169	-352.41524	704.96076	49.21465	1
H	77.22324	66.88972	79.31581	1714.76886	-2070.42778	443.35792	1
H	77.47418	66.17074	77.66240	1224.15040	1801.50881	1636.97633	1
C	79.77729	69.45423	79.13581	566.85117	537.05831	117.62814	1
O	79.10281	69.38851	80.15252	-550.26779	-406.08867	4.21666	1
N	80.70988	70.42629	78.85387	566.34832	-425.77725	180.18749	1
H	80.82108	70.24354	77.83702	644.44502	2646.80550	-52.73430	1
C	81.97980	70.21605	79.54978	612.34801	-281.48662	411.56221	1
H	82.23679	69.15294	79.34835	1667.23498	-1490.75142	1556.16753	1
C	82.97810	71.21863	78.83815	-714.45746	-175.43074	286.98927	1
H	82.73971	72.26174	79.18249	-787.09484	1763.03619	-1922.46060	1
O	82.91244	71.26954	77.39320	-429.46514	516.51932	-128.40526	1
H	82.65836	72.20454	77.24969	1981.82785	436.16847	-1818.18182	1
C	84.36870	70.77685	79.42872	630.79189	-468.94448	76.03230	1
H	85.18143	71.47777	79.12666	-1775.71861	2062.30353	131.89849	1
H	84.35601	70.73611	80.54774	-1855.14608	88.94118	1993.53760	1
H	84.65733	69.76307	79.06516	-1504.82350	-780.94249	-2132.90992	1
C	81.85272	70.52837	81.12444	288.57638	618.62814	-397.00270	1
O	82.76301	70.22180	81.86809	321.70495	-185.26452	574.37218	1
N	80.76454	71.24328	81.43771	-447.05792	-446.53431	367.76425	1
H	80.08366	71.38403	80.68919	350.31316	1975.63644	-1843.31522	1
C	80.47010	71.35148	82.84746	-511.23061	374.03917	-471.51480	1
H	80.73694	70.34452	83.24275	2234.79579	-898.70890	-1273.45190	1
C	81.49330	72.45760	83.40188	529.53781	-159.34528	563.72604	1
H	81.34099	72.57300	84.50773	654.59388	2347.94251	1217.51488	1
H	82.51782	72.01207	83.26441	-1526.55661	2103.42026	817.87394	1
O	81.44251	73.76353	82.77173	-554.84278	14.12789	399.58769	1

73	H	81.59775	73.49231	81.84548	1297.06236	2061.84854	1220.68812	1
74	C	78.92749	71.72515	83.03929	-662.15484	-412.13544	123.62687	1
75	O	78.57277	72.33081	84.01275	-540.01537	-317.05804	274.91456	1
76	N	78.15881	71.09816	82.11425	-202.76299	-690.00100	-131.49145	1
77	H	78.65290	70.46492	81.42800	-702.62132	-2617.37191	281.70413	1
78	C	76.97694	71.79747	81.55576	-491.73914	576.34187	222.73509	1
79	H	76.39197	70.95535	81.12121	1977.17978	-1823.58850	-434.68928	1
80	C	76.22924	72.59546	82.58878	-649.50797	-328.66843	306.11224	1
81	H	76.93169	73.35489	83.01420	2075.07803	1452.16490	-1004.45904	1
82	C	75.05943	73.33850	81.96308	-615.18735	-489.99689	-70.96352	1
83	H	74.51046	73.96252	82.71929	-1226.32661	-1724.38906	-1716.47170	1
84	H	75.40410	74.03049	81.14636	2381.64273	-969.84735	900.46860	1
85	H	74.31395	72.63169	81.50767	2122.74991	1702.23946	-141.36228	1
86	C	75.73262	71.73073	83.73489	-501.86074	-479.61276	-376.42482	1
87	H	75.19193	72.33790	84.51125	993.79261	-2246.95401	-1177.81305	1
88	H	75.02551	70.93266	83.37903	42.71279	-626.15391	-2651.37258	1
89	H	76.59147	71.21512	84.24957	-2026.39012	-1590.33709	-887.82922	1
90	C	77.55108	72.82138	80.38904	-672.89153	382.57394	-156.34072	1
91	O	78.01406	73.90488	80.65590	410.04627	221.86223	500.35950	1
92	N	77.38717	72.27735	79.19457	-378.11782	-443.79414	-441.10984	1
93	H	77.03011	71.32740	79.12245	2404.15973	-568.14894	1149.30085	1
94	C	77.76953	73.07067	78.05558	-544.54484	466.16019	331.29119	1
95	H	77.29935	74.05528	78.27303	-1409.80988	-170.17955	2325.32679	1
96	C	79.35028	73.17751	78.12042	-184.48284	116.78282	-758.89137	1
97	H	79.64584	73.68294	79.07039	1893.78641	1861.14652	611.04528	1
98	H	79.81164	72.16619	78.16039	-2170.79665	-974.19311	1327.50117	1
99	C	79.84631	73.95928	76.86416	389.51668	672.81878	-138.49152	1
100	H	79.53646	73.42677	75.93520	510.64577	1933.03327	1851.02484	1
101	H	79.33757	74.94694	76.80633	-2302.30869	-1105.72939	-948.89774	1
102	C	81.41318	74.15523	76.85347	-559.93427	-126.34590	542.30857	1
103	O	81.98860	74.34088	75.81345	-670.36003	-111.83166	76.36448	1
104	N	81.97764	74.13321	78.08524	-216.06729	-537.07037	446.51379	1
105	H	81.49090	74.13739	78.98736	-1251.96487	1074.12894	-2168.52500	1
106	H	82.93793	74.44095	78.27144	-2451.83023	-720.73851	944.84612	1
107	C	77.20168	72.32228	76.73565	546.24052	-563.88337	-85.11809	1
108	O	77.98145	71.75498	75.99536	71.70702	-307.40463	606.69673	1
109	N	75.88208	72.46733	76.60799	479.90863	-504.50129	222.86849	1
110	C	75.09680	71.60096	75.77512	75.51268	-600.91904	506.73700	1
111	H	75.48192	70.56532	75.91416	-2033.61562	1795.30103	254.89480	1
112	C	73.62664	71.86620	76.31487	523.79067	517.99274	284.45581	1
113	H	72.83886	71.58583	75.57958	1599.61665	-1127.00911	1895.98173	1
114	H	73.41529	71.29720	77.24743	-1881.83093	1275.82488	-1501.55016	1
115	C	73.70912	73.40080	76.58468	-600.05639	-404.05026	316.64485	1
116	H	73.72639	73.96927	75.62109	-2597.05260	496.36221	657.73249	1
117	H	72.86414	73.80572	77.18971	-1666.24518	1294.38985	-1723.90591	1
118	C	75.11718	73.48683	77.31691	493.57570	-129.95376	-602.56394	1
119	H	75.64455	74.46435	77.21946	-1469.08415	-637.84797	2204.22581	1
120	H	75.10789	73.17499	78.38843	-1038.97840	1068.88701	-2280.71789	1
121	C	75.14504	72.06886	74.21238	188.88100	241.05089	-727.87821	1
122	O	74.64077	71.33406	73.36107	531.88613	-49.19943	427.07960	1
123	N	75.62209	73.29066	74.06098	708.14833	26.93417	-179.73509	1
124	H	75.91394	73.62937	74.97737	-2232.69501	-1179.09831	1023.94816	1
125	C	76.19633	73.83769	72.84262	-738.32412	259.92548	104.42456	1
126	H	76.79116	74.71248	73.20009	2028.89123	1260.02983	1311.33324	1
127	C	77.04723	72.76666	72.17412	-258.65678	-345.52513	661.28459	1
128	H	76.41967	71.85074	72.07244	-1826.06090	-1801.46097	-918.64554	1
129	C	77.48831	73.19926	70.78778	-541.23400	531.92970	-218.41428	1
130	H	78.08591	72.39888	70.27085	1311.73218	-2219.18200	882.19023	1
131	H	76.60740	73.43910	70.12997	-1048.71298	1223.58118	-2196.97971	1
132	H	78.13120	74.12143	70.82848	419.06414	1903.92898	1903.44670	1
133	C	78.25943	72.40376	73.00793	-423.86431	152.66566	-648.55262	1
134	H	78.83711	71.55281	72.55255	105.68386	-2342.50080	1387.51238	1

H	78.97017	73.27084	73.10216	-1695.63603	-1484.73621	-1531.02793	1
H	77.97343	72.09060	74.07104	-1853.57121	-487.39031	1936.59343	1
C	74.96373	74.31567	71.82845	539.71428	-510.58232	267.58633	1
O	75.00723	75.41394	71.33666	-15.93758	-278.34085	-624.49402	1
N	74.08328	73.35511	71.64064	-676.81886	12.05292	276.17960	1
H	74.17856	72.43164	72.13435	-1694.44250	-373.84189	-2100.66098	1
C	73.00295	73.63041	70.74763	412.33078	-664.70360	108.35451	1
H	73.44492	74.30052	69.97422	1765.77476	1294.44366	1621.76594	1
C	72.48496	72.23002	70.22029	-179.66635	-721.95667	-264.73915	1
H	73.32867	71.60807	69.84582	-607.34922	2587.87032	-598.10018	1
H	72.04094	71.64942	71.05998	-359.69332	-1479.13954	-2259.74260	1
C	71.42441	72.47087	69.09635	-240.01076	616.02425	431.85322	1
H	70.68137	73.24020	69.41485	-1359.17131	782.54425	2227.99181	1
H	71.90180	72.86871	68.17286	-820.36667	85.81767	-2596.78715	1
C	70.70782	71.08730	68.82228	-513.07015	236.18570	-551.87280	1
H	71.44354	70.31545	68.49909	1816.11944	-1183.11722	-1650.94321	1
H	70.24576	70.69168	69.75586	-1800.04429	-1361.53473	1526.34643	1
C	69.63048	71.31171	67.73629	726.08972	-253.19732	-179.64713	1
H	68.84441	72.04356	68.00997	1224.27271	-456.51798	-2390.90304	1
H	70.03036	71.54487	66.72937	1206.40339	-1150.93514	-2154.90344	1
N	68.85935	69.95981	67.55949	329.70796	-633.68968	-155.67358	1
H	68.09020	69.96205	66.82913	1348.31305	1938.76845	1359.00643	1
H	69.42646	69.11294	67.26332	2096.92035	1446.54551	-966.48890	1
H	68.33892	69.58937	68.40726	-1197.07217	-2303.53719	827.28733	1
C	71.78218	74.37622	71.54502	-378.01186	-556.17301	-413.96481	1
O	70.89650	74.88997	70.89561	-409.44433	-307.55053	-453.30833	1
N	71.85865	74.25181	72.86566	-429.02477	524.70884	274.08502	1
H	72.68173	73.78940	73.25058	1706.34263	-1425.96036	1574.38926	1
C	70.95023	75.04099	73.63145	-45.25864	-626.22952	478.93267	1
H	70.41871	75.64850	72.85853	1988.63164	570.90773	1772.87287	1
C	69.99529	74.06247	74.43023	551.77128	-20.49526	-564.54996	1
H	69.61263	73.25539	73.76614	1434.61535	-1753.10253	1513.99471	1
H	70.53800	73.54943	75.25575	-714.67738	1819.09770	1898.36494	1
C	68.82963	74.95392	74.97264	446.99263	-555.77305	338.97221	1
H	69.21181	75.67916	75.72585	1953.68466	1892.25957	-161.67766	1
H	68.39920	75.56783	74.14989	-403.03785	2688.07032	-188.43591	1
C	67.71028	74.02924	75.59946	567.86262	335.74681	434.04421	1
H	67.35707	73.27336	74.86086	-1080.02275	1568.26081	-1948.78900	1
H	68.10587	73.44885	76.46441	-919.54108	2111.34692	1456.13463	1
C	66.55137	74.95788	76.03198	195.81897	173.33862	-745.11536	1
H	66.79978	75.64936	76.86165	1405.67949	-2301.21089	-390.08113	1
H	66.07795	75.51949	75.20192	-940.37328	-1118.32446	2299.72133	1
N	65.40007	74.05923	76.59612	319.26422	656.36007	-42.02424	1
H	64.53555	74.57791	76.92641	1961.03633	619.91968	1787.09422	1
H	64.97393	73.34729	75.93404	854.14343	-2156.80752	1429.08711	1
H	65.62279	73.46383	77.44603	1237.02905	-1378.50857	-1998.28500	1
C	71.82837	75.91579	74.71823	532.53319	-268.57236	517.55729	1
O	72.22117	75.44920	75.75870	266.16714	-290.73804	-558.88002	1
N	71.92728	77.13687	74.23225	-226.25352	640.49678	-270.32839	1
H	71.45790	77.44312	73.36396	2007.69063	-1035.27229	1523.50231	1
C	72.84864	78.08097	74.75968	-540.76542	-573.33303	49.50000	1
H	72.97951	77.89808	75.84933	-2374.20950	-1036.76023	843.75774	1
C	74.21083	77.89262	73.93130	-77.09082	666.87422	-415.84136	1
H	73.94682	77.80551	72.84014	-985.81834	617.19825	2463.91927	1
O	74.95879	76.70005	74.22467	293.29899	-391.65602	-477.80926	1
H	74.92153	76.23748	73.35775	2528.72714	-681.76262	-751.27122	1
C	74.97983	79.24038	74.17304	252.47363	-605.14473	440.05134	1
H	75.94929	79.25165	73.62208	-2052.65063	1456.49051	-1043.51830	1
H	74.39649	80.14472	73.82795	-2152.98800	-1661.97574	-161.70652	1
H	75.21571	79.38619	75.25290	-473.12726	-1584.27339	2165.61725	1
C	72.06004	79.44336	74.32091	360.36307	488.46713	505.10056	1
O	71.25871	79.36683	73.40414	-200.08817	-528.03552	-385.82850	1

N	72.44451	80.49282	75.02738	-597.54095	-226.44775	355.20479	1
H	73.16245	80.37552	75.74171	2179.23655	-1300.70981	-991.33835	1
C	71.96161	81.78656	74.67115	-687.68921	297.39807	-249.45179	1
H	70.86760	81.66816	74.50228	-349.16256	-2467.37713	-1101.72831	1
C	72.36603	82.81248	75.82720	672.47345	361.54328	-201.62880	1
H	73.46558	82.98890	75.80151	1541.41502	582.18425	2169.97026	1
H	71.88091	83.79575	75.62731	-2534.67717	60.25153	997.72295	1
C	71.96053	82.28066	77.20959	574.26774	-389.43119	-377.02542	1
C	70.59051	82.26868	77.60886	688.82679	-287.33580	-257.96133	1
H	69.80008	82.62796	76.92516	331.14182	1470.38163	2269.80013	1
C	72.97565	81.80441	78.09368	-383.52382	-675.04808	144.24917	1
H	74.03661	81.80589	77.78474	4.09847	1517.59662	2262.86397	1
C	70.27234	81.79622	78.88226	-134.76730	428.45285	-649.50303	1
H	69.21353	81.79029	79.19450	-903.23433	1600.49020	2011.53387	1
C	72.60639	81.34336	79.35941	-335.53281	-615.00040	-364.39058	1
H	73.40089	80.98442	80.03685	1596.45398	-1369.33862	1732.02659	1
C	71.25169	81.32281	79.80300	585.48547	-340.24194	-406.23938	1
O	70.86697	80.85781	81.08763	-419.15649	-173.04408	-511.94141	1
H	71.75417	80.63659	81.45739	-1071.84350	1559.71507	-1960.13012	1
C	72.74586	82.27255	73.33209	291.41878	-183.13091	710.72246	1
O	73.87501	81.94083	73.05007	379.87695	523.47396	-222.23622	1
N	71.96060	83.15055	72.72700	564.30591	373.09380	277.24371	1
H	70.98963	83.39060	73.00193	-120.59931	-2436.07824	-1214.35098	1
C	72.37664	83.68676	71.45683	404.07514	-299.37480	608.84009	1
H	73.37173	84.16582	71.59352	-1071.07882	-841.69025	2359.66467	1
C	72.45754	82.44166	70.47578	-683.12807	68.71770	-390.13096	1
H	73.15723	81.67934	70.89546	2068.88918	1596.58590	770.89603	1
H	71.46626	81.93752	70.41835	1379.86429	2342.49779	-179.85638	1
C	72.92458	82.90643	69.06832	-176.39967	537.49466	550.97268	1
H	72.28717	83.75066	68.68216	-396.28051	1942.22168	1869.33314	1
H	73.96715	83.29403	69.09250	-94.44700	586.00678	-2659.20016	1
C	72.79386	81.64349	68.07408	-291.69381	-718.18079	-150.72929	1
H	73.46363	80.77640	68.30604	-2303.38776	1089.76242	964.62032	1
H	71.72841	81.32945	67.91830	884.16729	2568.80533	-207.74283	1
N	73.25142	82.34852	66.96008	-594.49522	-252.81478	342.29276	1
H	72.62558	83.06096	66.60294	-1735.17426	2084.05705	-263.71605	1
C	74.36835	82.16825	66.35599	595.23177	-271.52487	442.22352	1
N	75.23016	81.18612	66.83737	585.53813	-424.79750	-105.81303	1
H	75.10833	80.56170	67.63459	959.88797	1327.56823	-2177.11905	1
H	76.13860	80.94473	66.43848	1578.48647	16.27378	-2220.76233	1
N	74.48129	83.04936	65.27942	-452.17763	-390.33541	-421.51853	1
H	73.83900	83.76806	64.94653	470.26107	1974.25629	-1817.92087	1
H	75.28628	83.08415	64.65224	1856.67307	-1573.97321	-1224.35416	1
C	71.14006	84.69390	71.19178	-531.47271	-581.45780	-55.06206	1
O	70.13761	84.59369	71.88360	-243.00837	192.73507	609.52485	1
N	71.40319	85.59130	70.24218	343.49288	-644.25607	38.09164	1
H	72.30631	85.47292	69.77957	-1691.16043	-1535.97525	-1484.73099	1
C	70.31045	86.26750	69.54650	542.66492	350.14337	454.42563	1
H	69.42427	86.12717	70.21180	1209.57979	-2396.95920	463.86744	1
C	70.72350	87.71976	69.36201	-737.14309	145.93010	242.71942	1
H	71.63933	87.73524	68.73003	-1050.18303	159.36419	-2509.06125	1
C	69.65721	88.54155	68.66724	-706.77696	-252.88343	-245.16230	1
H	69.94864	89.62624	68.60104	-1624.64039	-1665.49085	1417.86935	1
H	69.47037	88.18059	67.59631	2566.10521	598.51109	693.22305	1
H	68.67530	88.49593	69.21477	-1972.69758	107.10018	-1876.34312	1
C	71.07336	88.36550	70.69165	412.85148	672.58832	-27.69222	1
H	71.44937	89.41728	70.56098	1447.02277	1762.76533	-1490.78944	1
H	70.18037	88.41212	71.37379	1320.77379	-145.20455	2378.68491	1
H	71.87070	87.78519	71.23231	-1512.10848	1470.52031	1724.75147	1
C	70.16233	85.50452	68.07838	-607.11113	499.56689	73.73769	1
O	71.06275	84.85630	67.58698	357.97126	460.81534	356.68785	1
N	68.95198	85.72497	67.59601	536.55407	-368.62270	332.76306	1

```
H    68.34965  86.33776  68.14075      524.38866     2297.69985     1367.20941         1
C    68.68617  85.35868  66.24875       46.50277     -774.29190     -147.97904         1
H    69.40650  84.53357  66.04477     1676.51875     1883.29066     1032.55549         1
C    67.12935  84.95480  66.10079     -559.41456      -34.19198     -556.30433         1
H    66.49829  85.86040  66.26597      -64.66664      395.18769     2695.05373         1
H    66.91008  84.61994  65.05894    -2703.44278      -60.40056      333.79418         1
C    66.77149  83.88246  67.09449     -519.76435      377.31045      459.42366         1
H    67.06576  84.23297  68.10966    -1746.74323     1667.50644    -1261.73691         1
C    65.26790  83.63490  67.08147      570.95554       16.46261     -545.27700         1
H    64.96874  82.86095  67.83435     2391.58273       28.05277    -1305.07271         1
H    64.69217  84.56718  67.31600      175.75588      917.63458     2559.43867         1
H    64.91902  83.27444  66.07954    -1063.97851    -2155.34650     1283.00462         1
C    67.50844  82.58249  66.80226      193.76014        2.62946     -765.53141         1
H    67.21673  81.77277  67.52000      507.39394     -846.55087     2539.60089         1
H    67.28883  82.20638  65.76979     1337.57841     1818.19054     1526.01994         1
H    68.61891  82.71099  66.88218     1100.88635    -1172.91350    -2199.08909         1
C    68.93544  86.63649  65.30528     -549.21115      475.64486     -309.38218         1
O    69.18329  87.74519  65.72062      556.06106     -390.37755      -78.24250         1
O    68.79059  86.41014  63.91983     -572.27940     -310.19032     -209.75862         1
H    68.61287  85.42634  63.94544    -1380.08193     2212.14486      790.85944         1
```

Listing 14: C00014.xmolout

```
      276
  150.0000  150.0000  150.0000    0.0000    0.0000    0.0000***********    2.7750    11100
       0.2500    300.00   0.00   0.00   0.00  -1294.1102      0.0000    300.0000
H    67.49412  74.41689  73.80266    -2043.14101    -1757.64893      399.89339         1
N    67.69003  75.20669  74.41096      426.75047      490.45733     -334.42467         1
H    67.80482  74.72324  75.30205      442.25559    -1110.30854    -2448.52907         1
C    68.85057  75.92805  74.02451      415.32972     -666.39213       83.73172         1
H    69.63080  75.15281  73.86769      879.32219     2491.00447      667.35213         1
C    68.53017  76.74960  72.67794       31.35357      477.41777      628.23362         1
H    67.67588  77.44286  72.87121     1168.38489     2240.25337    -1019.71136         1
H    69.39932  77.39947  72.41287     -360.43770     2534.90498      931.67112         1
C    68.20199  75.81146  71.54360      463.18208      512.70242     -382.34388         1
H    67.38354  75.13160  71.86934     1725.29117     1863.52247     -987.07854         1
C    67.71070  76.60002  70.33608      710.42801     -144.27427     -313.15367         1
H    67.44024  75.92525  69.48317      364.20195    -1033.13725     2494.72524         1
H    66.80116  77.20735  70.58150     2567.61904      335.80679      847.48802         1
H    68.49259  77.31049  69.96256    -2587.99029      680.92867     -512.16411         1
C    69.40891  74.97111  71.15067      268.17616      741.64379      -40.42634         1
H    69.18138  74.30531  70.27842     1283.54314    -1945.37420    -1411.27812         1
H    70.28055  75.61281  70.86019    -1258.57796     1317.15249    -2026.02085         1
H    69.74865  74.31096  71.98963    -2502.84526      909.80597      575.92391         1
C    69.26887  76.99094  75.17871      619.36578     -458.64843     -172.09273         1
O    70.47092  77.32732  75.25766      -81.40433      433.87611      522.34594         1
N    68.23271  77.39720  75.84662      175.23996     -622.20190      341.55523         1
H    67.38893  76.91970  75.52599     -876.57256     -303.22879    -2561.90213         1
C    68.14706  78.31939  76.94759     -571.88214     -534.10783     -106.15033         1
H    67.73624  77.71339  77.78583    -2713.20841      -16.25800     -248.80809         1
C    69.54212  78.92206  77.28372      166.60354      288.16399     -716.09568         1
H    70.04855  79.28101  76.35593    -2097.85574     1636.78449     -586.18044         1
O    70.55290  77.79674  77.81991     -317.80580     -363.38227      484.43082         1
H    70.86381  77.54051  76.92581     1863.74748    -1451.84923     1357.29467         1
C    69.37388  80.06072  78.31010      503.80731      517.99032      318.51606         1
H    70.37699  80.47385  78.59194      640.61020    -2157.10410    -1536.29104         1
H    68.75258  80.90261  77.91053     1687.88455      465.60692     2087.56452         1
H    68.88867  79.70401  79.25145    -1293.07895    -1899.88581    -1463.57452         1
C    67.07815  79.47429  76.46922     -153.07550     -733.68902      248.70981         1
O    67.06344  79.87683  75.33659      186.15459     -480.78787     -449.34387         1
N    66.35144  79.89229  77.51903     -487.53139      543.18517       42.03850         1
C    65.50223  81.03472  77.39609     -699.35871       69.34393      360.10701         1
```

H	65.22438	81.09119	76.31760	2.81126	-221.99807	-2715.58072	1	
C	64.33906	80.72939	78.43258	-507.46439	-592.59194	122.07761	1	
H	63.83264	81.65815	78.78608	-1975.08116	525.87560	-1801.71556	1	
H	63.55307	80.07509	77.99279	1588.27743	-1913.22100	1113.83566	1	
C	65.12829	79.99578	79.56283	-613.62442	-457.55993	-194.14490	1	
H	65.68010	80.72746	80.19457	1707.54637	-721.27992	1996.92523	1	
H	64.48057	79.40646	80.25031	758.23357	2100.81767	1560.55011	1	
C	66.12715	79.09490	78.71858	-352.41524	704.96076	49.21465	1	
H	67.10651	78.89775	79.21130	1714.76886	-2070.42778	443.35792	1	
H	65.69563	78.12946	78.36291	1224.15040	1801.50881	1636.97633	1	
C	66.24110	82.39589	77.95068	566.85117	537.05831	117.62814	1	
O	65.66286	83.02191	78.84685	-550.26779	-406.08867	4.21666	1	
N	67.35909	82.64069	77.33926	566.34832	-425.77725	180.18749	1	
H	67.55331	81.96277	76.60164	644.44502	2646.80550	-52.73430	1	
C	68.18165	83.80393	77.50206	612.34801	-281.48662	411.56221	1	
H	68.02624	84.38509	76.56795	1667.23498	-1490.75142	1556.16753	1	
C	67.78113	84.60679	78.81568	-714.45746	-175.43074	286.98927	1	
H	67.63917	83.84021	79.63004	-787.09484	1763.03619	-1922.46060	1	
O	66.56407	85.34642	78.73506	-429.46514	516.51932	-128.40526	1	
H	65.90717	84.61004	79.04045	1981.82785	436.16847	-1818.18182	1	
C	69.06592	85.46060	79.12315	630.79189	-468.94448	76.03230	1	
H	68.94418	86.05066	80.06109	-1775.71861	2062.30353	131.89849	1	
H	69.97204	84.82193	79.25154	-1855.14608	88.94118	1993.53760	1	
H	69.28099	86.18469	78.30364	-1504.82350	-780.94249	-2132.90992	1	
C	69.70254	83.23164	77.60766	288.57638	618.62814	-397.00270	1	
O	70.06944	82.62844	78.62220	321.70495	-185.26452	574.37218	1	
N	70.39387	83.63081	76.57070	-447.05792	-446.53431	367.76425	1	
H	69.86126	84.16286	75.88148	350.31316	1975.63644	-1843.31522	1	
C	71.62626	83.05672	76.15225	-511.23061	374.03917	-471.51480	1	
H	71.94354	83.78912	75.37613	2234.79579	-898.70890	-1273.45190	1	
C	72.64572	82.99331	77.38299	529.53781	-159.34528	563.72604	1	
H	73.69754	82.99962	76.98301	654.59388	2347.94251	1217.51488	1	
H	72.50274	83.95975	77.93741	-1526.55661	2103.42026	817.87394	1	
O	72.53540	81.87749	78.27113	-554.84278	14.12789	399.58769	1	
H	71.58026	82.05841	78.62527	1297.06236	2061.84854	1220.68812	1	
C	71.32910	81.61118	75.47557	-662.15484	-412.13544	123.62687	1	
O	70.21507	81.23249	75.18293	-540.01537	-317.05804	274.91456	1	
N	72.48128	81.00558	75.19651	-202.76299	-690.00100	-131.49145	1	
H	73.36317	81.43671	75.46620	-702.62132	-2617.37191	281.70413	1	
C	72.43572	79.85283	74.35930	-491.73914	576.34187	222.73509	1	
H	71.41878	79.41114	74.49896	1977.17978	-1823.58850	-434.68928	1	
C	72.67979	80.38520	72.87356	-649.50797	-328.66843	306.11224	1	
H	73.48437	81.15127	72.87086	2075.07803	1452.16490	-1004.45904	1	
C	73.03138	79.21029	71.90151	-615.18735	-489.99689	-70.96352	1	
H	73.05275	79.54809	70.83870	-1226.32661	-1724.38906	-1716.47170	1	
H	74.03672	78.78544	72.12847	2381.64273	-969.84735	900.46860	1	
H	72.28952	78.38010	71.97235	2122.74991	1702.23946	-141.36228	1	
C	71.32968	80.99057	72.36205	-501.86074	-479.61276	-376.42482	1	
H	71.44694	81.48105	71.36670	993.79261	-2246.95401	-1177.81305	1	
H	70.54409	80.20563	72.25730	42.71279	-626.15391	-2651.37258	1	
H	70.93232	81.75422	73.07530	-2026.39012	-1590.33709	-887.82922	1	
C	73.69098	78.91843	74.81156	-672.89153	382.57394	-156.34072	1	
O	74.83353	79.29792	74.76763	410.04627	221.86223	500.35950	1	
N	73.22501	77.68954	75.07896	-378.11782	-443.79414	-441.10984	1	
H	72.18940	77.51113	75.06390	2404.15973	-568.14894	1149.30085	1	
C	74.05981	76.92558	75.98452	-544.54484	466.16019	331.29119	1	
H	74.81565	77.66252	76.34719	-1409.80988	-170.17955	2325.32679	1	
C	73.04872	76.45385	77.11599	-184.48284	116.78282	-758.89137	1	
H	72.75269	77.34856	77.71286	1893.78641	1861.14652	611.04528	1	
H	72.10854	76.07205	76.65691	-2170.79665	-974.19311	1327.50117	1	
C	73.64179	75.35964	78.05303	389.51668	672.81878	-138.49152	1	
H	73.64875	74.36705	77.53819	510.64577	1933.03327	1851.02484	1	

H	74.71912	75.55707	78.23791	-2302.30869	-1105.72939	-948.89774	1
C	72.85918	75.25434	79.42456	-559.93427	-126.34590	542.30857	1
O	73.26436	74.52913	80.30113	-670.36003	-111.83166	76.36448	1
N	71.76819	76.03194	79.49490	-216.06729	-537.07037	446.51379	1
H	71.33217	76.68876	78.76822	-1251.96487	1074.12894	-2168.52500	1
H	71.17867	76.02517	80.33851	-2451.83023	-720.73851	944.84612	1
C	74.77252	75.64422	75.33915	546.24052	-563.88337	-85.11809	1
O	75.36884	74.91504	76.13657	71.70702	-307.40463	606.69673	1
N	74.77584	75.54689	74.01335	479.90863	-504.50129	222.86849	1
C	74.88983	74.30129	73.31293	75.51268	-600.91904	506.73700	1
H	74.33152	73.54268	73.90626	-2033.61562	1795.30103	254.89480	1
C	74.26688	74.65920	71.90223	523.79067	517.99274	284.45581	1
H	74.57354	73.94774	71.10228	1599.61665	-1127.00911	1895.98173	1
H	73.15414	74.65510	71.92984	-1881.83093	1275.82488	-1501.55016	1
C	74.82079	76.10299	71.68261	-600.05639	-404.05026	316.64485	1
H	75.89483	76.08082	71.39141	-2597.05260	496.36221	657.73249	1
H	74.28783	76.67386	70.88889	-1666.24518	1294.38985	-1723.90591	1
C	74.61145	76.70500	73.13574	493.57570	-129.95376	-602.56394	1
H	75.33609	77.49458	73.45538	-1469.08415	-637.84797	2204.22581	1
H	73.57218	77.05842	73.34098	-1038.97840	1068.88701	-2280.71789	1
C	76.44336	73.85005	73.03755	188.88100	241.05089	-727.87821	1
O	76.65677	72.84445	72.40001	531.88613	-49.19943	427.07960	1
N	77.31367	74.74536	73.49261	708.14833	26.93417	-179.73509	1
H	76.90465	75.49621	74.04677	-2232.69501	-1179.09831	1023.94816	1
C	78.70964	74.43175	73.51682	-738.32412	259.92548	104.42456	1
H	78.77957	73.78205	72.61345	2028.89123	1260.02983	1311.33324	1
C	79.47571	75.81640	73.38953	-258.65678	-345.52513	661.28459	1
H	79.24588	76.44198	74.27875	-1826.06090	-1801.46097	-918.64554	1
C	81.02179	75.79583	73.13192	-541.23400	531.92970	-218.41428	1
H	81.40501	76.82813	72.95160	1311.73218	-2219.18200	882.19023	1
H	81.60520	75.39097	73.99472	-1048.71298	1223.58118	-2196.97971	1
H	81.29549	75.18931	72.22383	419.06414	1903.92898	1903.44670	1
C	78.94281	76.48921	72.07714	-423.86431	152.66566	-648.55262	1
H	79.37957	77.50413	71.92502	105.68386	-2342.50080	1387.51238	1
H	79.19408	75.87976	71.17605	-1695.63603	-1484.73621	-1531.02793	1
H	77.83429	76.60269	72.09795	-1853.57121	-487.39031	1936.59343	1
C	78.92795	73.57102	74.87607	539.71428	-510.58232	267.58633	1
O	78.10570	72.72354	75.23113	-15.93758	-278.34085	-624.49402	1
N	80.05157	73.87745	75.48311	-676.81886	12.05292	276.17960	1
H	80.63309	74.60759	75.08100	-1694.44250	-373.84189	-2100.66098	1
C	80.57765	72.98918	76.48637	412.33078	-664.70360	108.35451	1
H	80.53522	71.97602	76.02555	1765.77476	1294.44366	1621.76594	1
C	82.03616	73.49321	76.81891	-179.66635	-721.95667	-264.73915	1
H	82.63887	73.67271	75.90095	-607.34922	2587.87032	-598.10018	1
H	81.97995	74.47980	77.33127	-359.69332	-1479.13954	-2259.74260	1
C	82.70928	72.42751	77.74808	-240.01076	616.02425	431.85322	1
H	81.95715	72.04406	78.49351	-1359.17131	782.54425	2227.99181	1
H	83.05467	71.53939	77.17212	-820.36667	85.81767	-2596.78715	1
C	83.90441	73.12877	78.50819	-513.07015	236.18570	-551.87280	1
H	84.77818	73.28040	77.83394	1816.11944	-1183.11722	-1650.94321	1
H	83.60908	74.14478	78.85777	-1800.04429	-1361.53473	1526.34643	1
C	84.26499	72.23525	79.71839	726.08972	-253.19732	-179.64713	1
H	83.46545	72.15282	80.48216	1224.27271	-456.51798	-2390.90304	1
H	84.64076	71.22673	79.45484	1206.40339	-1150.93514	-2154.90344	1
N	85.45227	72.92395	80.47059	329.70796	-633.68968	-155.67358	1
H	85.79074	72.42833	81.34523	1348.31305	1938.76845	1359.00643	1
H	86.35969	73.04370	79.93320	2096.92035	1446.54551	-966.48890	1
H	85.28419	73.90504	80.83956	-1197.07217	-2303.53719	827.28733	1
C	79.70142	73.04897	77.85293	-378.01186	-556.17301	-413.96481	1
O	80.03478	72.40894	78.83159	-409.44433	-307.55053	-453.30833	1
N	78.69551	73.91714	77.76409	-429.02477	524.70884	274.08502	1
H	78.50120	74.30166	76.83733	1706.34263	-1425.96036	1574.38926	1

C	77.80003	73.97939	78.87848	-45.25864	-626.22952	478.93267	1
H	78.44390	73.76144	79.76280	1988.63164	570.90773	1772.87287	1
C	77.17691	75.43522	78.85900	551.77128	-20.49526	-564.54996	1
H	77.97906	76.20585	78.82231	1434.61535	-1753.10253	1513.99471	1
H	76.57943	75.58425	77.92884	-714.67738	1819.09770	1898.36494	1
C	76.29797	75.60611	80.14134	446.99263	-555.77305	338.97221	1
H	75.49088	74.83047	80.17268	1953.68466	1892.25957	-161.67766	1
H	76.90011	75.45238	81.06494	-403.03785	2688.07032	-188.43591	1
C	75.70350	77.07151	80.09290	567.86262	335.74681	434.04421	1
H	76.51893	77.82844	80.15357	-1080.02275	1568.26081	-1948.78900	1
H	75.19261	77.26362	79.12204	-919.54108	2111.34692	1456.13463	1
C	74.70656	77.24553	81.26227	195.81897	173.33862	-745.11536	1
H	73.89791	76.48603	81.29802	1405.67949	-2301.21089	-390.08113	1
H	75.17323	77.34984	82.26260	-940.37328	-1118.32446	2299.72133	1
N	73.94436	78.58920	81.01101	319.26422	656.36007	-42.02424	1
H	73.22700	78.86144	81.74314	1961.03633	619.91968	1787.09422	1
H	74.51969	79.47807	80.93641	854.14343	-2156.80752	1429.08711	1
H	73.36037	78.63282	80.12615	1237.02905	-1378.50857	-1998.28500	1
C	76.63407	72.84599	78.71244	532.53319	-268.57236	517.55729	1
O	75.90719	72.64116	79.65438	266.16714	-290.73804	-558.88002	1
N	76.58964	72.32515	77.49238	-226.25352	640.49678	-270.32839	1
H	77.28383	72.58105	76.75001	2007.69063	-1035.27229	1523.50231	1
C	75.76829	71.17314	77.31469	-540.76542	-573.33303	49.50000	1
H	75.20643	71.06311	78.27552	-2374.20950	-1036.76023	843.75774	1
C	74.84829	71.51417	76.06281	-77.09082	666.87422	-415.84136	1
H	75.52011	71.85267	75.21887	-985.81834	617.19825	2463.91927	1
O	73.97940	72.65438	76.21075	293.29899	-391.65602	-477.80926	1
H	74.67328	73.40165	76.17992	2528.72714	-681.76262	-751.27122	1
C	74.14590	70.15432	75.72444	252.47363	-605.14473	440.05134	1
H	73.54470	70.22686	74.78825	-2052.65063	1456.49051	-1043.51830	1
H	74.88169	69.32974	75.58328	-2152.98800	-1661.97574	-161.70652	1
H	73.45240	69.84417	76.54081	-473.12726	-1584.27339	2165.61725	1
C	76.82054	69.94174	77.03759	360.36307	488.46713	505.10056	1
O	77.27796	69.33625	77.97628	-200.08817	-528.03552	-385.82850	1
N	77.09115	69.77713	75.74830	-597.54095	-226.44775	355.20479	1
H	76.65603	70.38151	75.05286	2179.23655	-1300.70981	-991.33835	1
C	77.66165	68.52378	75.36456	-687.68921	297.39807	-249.45179	1
H	78.54388	68.43111	76.03484	-349.16256	-2467.37713	-1101.72831	1
C	76.61821	67.32690	75.54395	672.47345	361.54328	-201.62880	1
H	75.72194	67.50980	74.90964	1541.41502	582.18425	2169.97026	1
H	77.08768	66.39428	75.15480	-2534.67717	60.25153	997.72295	1
C	76.19994	67.12414	77.01275	574.26774	-389.43119	-377.02542	1
C	77.15914	66.69699	77.98211	688.82679	-287.33580	-257.96133	1
H	78.22005	66.56373	77.70278	331.14182	1470.38163	2269.80013	1
C	74.83372	67.29991	77.39069	-383.52382	-675.04808	144.24917	1
H	74.07999	67.61554	76.64724	4.09847	1517.59662	2262.86397	1
C	76.73562	66.46156	79.29176	-134.76730	428.45285	-649.50303	1
H	77.48151	66.14064	80.03984	-903.23433	1600.49020	2011.53387	1
C	74.45774	67.05531	78.71432	-335.53281	-615.00040	-364.39058	1
H	73.39864	67.19890	78.99117	1596.45398	-1369.33862	1732.02659	1
C	75.38338	66.62571	79.71036	585.48547	-340.24194	-406.23938	1
O	75.00648	66.35626	81.05249	-419.15649	-173.04408	-511.94141	1
H	74.05507	66.61159	81.01322	-1071.84350	1559.71507	-1960.13012	1
C	77.95896	68.54480	73.76728	291.41878	-183.13091	710.72246	1
O	77.39221	67.72651	73.07205	379.87695	523.47396	-222.23622	1
N	78.80996	69.49747	73.37929	564.30591	373.09380	277.24371	1
H	79.20082	70.12289	74.08459	-120.59931	-2436.07824	-1214.35098	1
C	79.30167	69.35882	72.03313	404.07514	-299.37480	608.84009	1
H	79.55236	68.27938	71.93333	-1071.07882	-841.69025	2359.66467	1
C	78.11365	69.76998	71.07669	-683.12807	68.71770	-390.13096	1
H	77.17495	69.24715	71.37944	2068.88918	1596.58590	770.89603	1
H	77.89529	70.86077	71.15895	1379.86429	2342.49779	-179.85638	1

```
C    78.53824   69.38403   69.62455      -176.39967       537.49466       550.97268           1
H    79.50715   69.85428   69.34276      -396.28051      1942.22168      1869.33314           1
H    78.70004   68.28706   69.53071       -94.44700       586.00678     -2659.20016           1
C    77.34839   69.88529   68.68558      -291.69381      -718.18079      -150.72929           1
H    76.34734   69.44106   68.91337     -2303.38776      1089.76242       964.62032           1
H    77.22229   70.99554   68.60566       884.16729      2568.80533      -207.74283           1
N    77.72508   69.38595   67.43176      -594.49522      -252.81478       342.29276           1
H    78.52969   68.77931   67.40770     -1735.17426      2084.05705      -263.71605           1
C    77.10746   69.63123   66.34754       595.23177      -271.52487       442.22352           1
N    75.98421   70.46221   66.42206       585.53813      -424.79750      -105.81303           1
H    75.57416   70.92106   67.23465       959.88797      1327.56823     -2177.11905           1
H    75.40093   70.73378   65.63030      1578.48647        16.27378     -2220.76233           1
N    77.70876   68.98226   65.26059      -452.17763      -390.33541      -421.51853           1
H    78.51492   68.36103   65.20949       470.26107      1974.25629     -1817.92087           1
H    77.34733   69.04361   64.30777      1856.67307     -1573.97321     -1224.35416           1
C    80.54507   70.37827   71.73666      -531.47271      -581.45780       -55.06206           1
O    81.44093   70.01543   71.00761      -243.00837       192.73507       609.52485           1
N    80.30636   71.57746   72.25524       343.49288      -644.25607        38.09164           1
H    79.45799   71.65663   72.81680     -1691.16043     -1535.97525     -1484.73099           1
C    81.25472   72.62937   72.19922       542.66492       350.14337       454.42563           1
H    80.89004   73.35492   72.95837      1209.57979     -2396.95920       463.86744           1
C    82.69090   72.04533   72.56282      -737.14309       145.93010       242.71942           1
H    82.98846   71.26902   71.81765     -1050.18303       159.36419     -2509.06125           1
C    83.74415   73.20242   72.62123      -706.77696      -252.88343      -245.16230           1
H    84.74562   72.83047   72.94266     -1624.64039     -1665.49085      1417.86935           1
H    83.87476   73.68720   71.62280      2566.10521       598.51109       693.22305           1
H    83.43888   73.99942   73.33897     -1972.69758       107.10018     -1876.34312           1
C    82.60999   71.42614   73.99988       412.85148       672.58832       -27.69222           1
H    83.59967   71.04495   74.34452      1447.02277      1762.76533     -1490.78944           1
H    82.26369   72.17850   74.74329      1320.77379      -145.20455      2378.68491           1
H    81.89108   70.57416   74.03370     -1512.10848      1470.52031      1724.75147           1
C    81.30058   73.27416   70.69662      -607.11113       499.56689        73.73769           1
O    81.97749   74.26651   70.51107       357.97126       460.81534       356.68785           1
N    80.58007   72.60507   69.80395       536.55407      -368.62270       332.76306           1
H    80.04402   71.81315   70.15644       524.38866      2297.69985      1367.20941           1
C    80.38530   73.20214   68.53148        46.50277      -774.29190      -147.97904           1
H    81.30109   73.81591   68.37650      1676.51875      1883.29066      1032.55549           1
C    80.10784   72.08549   67.40250      -559.41456       -34.19198      -556.30433           1
H    79.19040   71.50692   67.66772       -64.66664       395.18769      2695.05373           1
H    79.87584   72.58814   66.43275     -2703.44278       -60.40056       333.79418           1
C    81.27755   71.15076   67.23845      -519.76435       377.31045       459.42366           1
H    81.45540   70.63519   68.20949     -1746.74323      1667.50644     -1261.73691           1
C    80.96015   70.11010   66.17049       570.95554        16.46261      -545.27700           1
H    81.77681   69.34905   66.07381      2391.58273        28.05277     -1305.07271           1
H    80.01389   69.55844   66.39889       175.75588       917.63458      2559.43867           1
H    80.82651   70.58210   65.16281     -1063.97851     -2155.34650      1283.00462           1
C    82.54696   71.89342   66.84495       193.76014         2.62946      -765.53141           1
H    83.39650   71.18642   66.65762       507.39394      -846.55087      2539.60089           1
H    82.40174   72.49218   65.90849      1337.57841      1818.19054      1526.01994           1
H    82.87774   72.60199   67.64775      1100.88635     -1172.91350     -2199.08909           1
C    79.04982   74.10850   68.60316      -549.21115       475.64486      -309.38218           1
O    78.10012   73.84405   69.29207       556.06106      -390.37755       -78.24250           1
O    78.98758   75.17320   67.67203      -572.27940      -310.19032      -209.75862           1
H    79.89584   75.08040   67.27135     -1380.08193      2212.14486       790.85944           1
```

Listing 15: C00015.xmolout

```
        276
   150.0000  150.0000  150.0000    0.0000    0.0000    0.0000**********    1.0837    4335
        0.2500    300.00   0.00   0.00   0.00  -1294.0898     0.0000   300.0000
H    74.40700   81.18762   86.05284     -2043.14101     -1757.64893       399.89339           1
N    74.98250   80.68799   85.37747       426.75047       490.45733      -334.42467           1
```

H	75.22072	79.83376	85.87685	442.25559	-1110.30854	-2448.52907	1
C	76.09979	81.40186	84.89445	415.32972	-666.39213	83.73172	1
H	75.72219	82.41201	84.63075	879.32219	2491.00447	667.35213	1
C	77.24028	81.42918	86.03071	31.35357	477.41777	628.23362	1
H	77.56681	80.38413	86.24957	1168.38489	2240.25337	-1019.71136	1
H	78.14366	81.95520	85.63853	-360.43770	2534.90498	931.67112	1
C	76.74319	82.09630	87.28837	463.18208	512.70242	-382.34388	1
H	75.84311	81.54977	87.64821	1725.29117	1863.52247	-987.07854	1
C	77.80853	82.03151	88.37572	710.42801	-144.27427	-313.15367	1
H	77.45117	82.49168	89.33309	364.20195	-1033.13725	2494.72524	1
H	78.10444	80.97502	88.60425	2567.61904	335.80679	847.48802	1
H	78.73886	82.57820	88.07308	-2587.99029	680.92867	-512.16411	1
C	76.36138	83.54870	87.03919	268.17616	741.64379	-40.42634	1
H	76.02677	84.05492	87.98152	1283.54314	-1945.37420	-1411.27812	1
H	77.22494	84.13775	86.63503	-1258.57796	1317.15249	-2026.02085	1
H	75.52325	83.63836	86.30112	-2502.84526	909.80597	575.92391	1
C	76.68700	80.54253	83.62682	619.36578	-458.64843	-172.09273	1
O	77.00451	79.37981	83.71900	-81.40433	433.87611	522.34594	1
N	76.78765	81.37044	82.59242	175.23996	-622.20190	341.55523	1
H	76.56728	82.35498	82.74035	-876.57256	-303.22879	-2561.90213	1
C	77.19255	80.91318	81.30922	-571.88214	-534.10783	-106.15033	1
H	77.14485	81.83007	80.67912	-2713.20841	-16.25800	-248.80809	1
C	78.70022	80.39291	81.50756	166.60354	288.16399	-716.09568	1
H	78.64050	79.45474	82.12845	-2097.85574	1636.78449	-586.18044	1
O	79.57270	81.29633	82.21061	-317.80580	-363.38227	484.43082	1
H	79.72381	80.79429	83.03681	1863.74748	-1451.84923	1357.29467	1
C	79.18904	80.04094	80.05654	503.80731	517.99032	318.51606	1
H	80.22140	79.61921	80.06956	640.61020	-2157.10410	-1536.29104	1
H	78.52498	79.28160	79.55197	1687.88455	465.60692	2087.56452	1
H	79.21476	80.94703	79.40696	-1293.07895	-1899.88581	-1463.57452	1
C	76.18938	79.74503	80.79682	-153.07550	-733.68902	248.70981	1
O	76.63741	78.94030	80.01202	186.15459	-480.78787	-449.34387	1
N	74.91444	79.88459	81.17888	-487.53139	543.18517	42.03850	1
C	74.01559	78.77145	81.24464	-699.35871	69.34393	360.10701	1
H	74.62238	77.87838	81.52245	2.81126	-221.99807	-2715.58072	1
C	72.99376	79.28942	82.35003	-507.46439	-592.59194	122.07761	1
H	72.04986	78.69989	82.37975	-1975.08116	525.87560	-1801.71556	1
H	73.43817	79.25266	83.37049	1588.27743	-1913.22100	1113.83566	1
C	72.79868	80.76041	81.87165	-613.62442	-457.55993	-194.14490	1
H	72.14845	80.80644	80.96905	1707.54637	-721.27992	1996.92523	1
H	72.33124	81.42199	82.63531	758.23357	2100.81767	1560.55011	1
C	74.29901	81.15874	81.53202	-352.41524	704.96076	49.21465	1
H	74.43042	81.84821	80.66099	1714.76886	-2070.42778	443.35792	1
H	74.87625	81.54310	82.40377	1224.15040	1801.50881	1636.97633	1
C	73.19793	78.57658	79.84916	566.85117	537.05831	117.62814	1
O	72.16366	77.90693	79.84049	-550.26779	-406.08867	4.21666	1
N	73.79453	79.12645	78.77809	566.34832	-425.77725	180.18749	1
H	74.61723	79.59264	79.18050	644.44502	2646.80550	-52.73430	1
C	72.93122	79.90863	77.90894	612.34801	-281.48662	411.56221	1
H	72.06722	80.19870	78.54621	1667.23498	-1490.75142	1556.16753	1
C	73.80540	81.14949	77.44930	-714.45746	-175.43074	286.98927	1
H	74.60848	80.78690	76.75177	-787.09484	1763.03619	-1922.46060	1
O	74.50797	81.85870	78.50487	-429.46514	516.51932	-128.40526	1
H	75.43312	81.73194	78.22455	1981.82785	436.16847	-1818.18182	1
C	72.77205	82.02416	76.65086	630.79189	-468.94448	76.03230	1
H	73.25438	82.93417	76.22435	-1775.71861	2062.30353	131.89849	1
H	72.31114	81.46346	75.80059	-1855.14608	88.94118	1993.53760	1
H	71.94100	82.36918	77.30912	-1504.82350	-780.94249	-2132.90992	1
C	72.48352	79.01939	76.64277	288.57638	618.62814	-397.00270	1
O	71.59151	79.43141	75.89244	321.70495	-185.26452	574.37218	1
N	73.28936	77.96313	76.43229	-447.05792	-446.53431	367.76425	1
H	73.98642	78.04791	77.18363	350.31316	1975.63644	-1843.31522	1

C	72.78350	76.62493	76.23683	-511.23061	374.03917	-471.51480	1
H	73.73701	76.06283	76.12514	2234.79579	-898.70890	-1273.45190	1
C	72.07122	76.11712	77.57549	529.53781	-159.34528	563.72604	1
H	71.91639	75.01298	77.42216	654.59388	2347.94251	1217.51488	1
H	72.80470	76.23859	78.42245	-1526.55661	2103.42026	817.87394	1
O	70.81915	76.70490	77.94554	-554.84278	14.12789	399.58769	1
H	71.13992	77.25779	78.75670	1297.06236	2061.84854	1220.68812	1
C	71.80883	76.40689	74.99303	-662.15484	-412.13544	123.62687	1
O	71.35235	75.31010	74.80971	-540.01537	-317.05804	274.91456	1
N	71.66634	77.50034	74.20077	-202.76299	-690.00100	-131.49145	1
H	72.06674	78.38676	74.59273	-702.62132	-2617.37191	281.70413	1
C	70.37072	77.60841	73.45815	-491.73914	576.34187	222.73509	1
H	70.28164	76.61729	72.94649	1977.17978	-1823.58850	-434.68928	1
C	69.24248	77.91242	74.43185	-649.50797	-328.66843	306.11224	1
H	69.57022	78.77724	75.05619	2075.07803	1452.16490	-1004.45904	1
C	67.97623	78.30673	73.69271	-615.18735	-489.99689	-70.96352	1
H	67.15765	78.61502	74.39981	-1226.32661	-1724.38906	-1716.47170	1
H	68.15464	79.16830	72.99093	2381.64273	-969.84735	900.46860	1
H	67.57889	77.45687	73.07237	2122.74991	1702.23946	-141.36228	1
C	68.93924	76.76759	75.37826	-501.86074	-479.61276	-376.42482	1
H	68.09181	77.01710	76.07617	993.79261	-2246.95401	-1177.81305	1
H	68.65485	75.83159	74.82184	42.71279	-626.15391	-2651.37258	1
H	69.83518	76.51418	76.02049	-2026.39012	-1590.33709	-887.82922	1
C	70.48505	78.85871	72.46014	-672.89153	382.57394	-156.34072	1
O	70.07010	78.79623	71.25027	410.04627	221.86223	500.35950	1
N	70.97453	79.94055	72.99680	-378.11782	-443.79414	-441.10984	1
H	71.19765	79.93716	74.00180	2404.15973	-568.14894	1149.30085	1
C	70.56021	81.09491	72.24641	-544.54484	466.16019	331.29119	1
H	69.50720	81.20399	72.57731	-1409.80988	-170.17955	2325.32679	1
C	71.39800	82.34769	72.65378	-184.48284	116.78282	-758.89137	1
H	70.98236	82.83271	73.56764	1893.78641	1861.14652	611.04528	1
H	72.44463	82.06940	72.92052	-2170.79665	-974.19311	1327.50117	1
C	71.34589	83.32753	71.42898	389.51668	672.81878	-138.49152	1
H	71.56712	82.77895	70.48677	510.64577	1933.03327	1851.02484	1
H	70.30679	83.69298	71.27569	-2302.30869	-1105.72939	-948.89774	1
C	72.33288	84.54543	71.54757	-559.93427	-126.34590	542.30857	1
O	72.48575	85.28414	70.60725	-670.36003	-111.83166	76.36448	1
N	72.93640	84.65382	72.75237	-216.06729	-537.07037	446.51379	1
H	72.81357	84.08453	73.59748	-1251.96487	1074.12894	-2168.52500	1
H	73.57566	85.41520	73.00435	-2451.83023	-720.73851	944.84612	1
C	70.53465	80.60501	70.61402	546.24052	-563.88337	-85.11809	1
O	69.62876	81.08327	69.92147	71.70702	-307.40463	606.69673	1
N	71.81283	80.42461	70.11943	479.90863	-504.50129	222.86849	1
C	72.03249	80.26554	68.73724	75.51268	-600.91904	506.73700	1
H	71.19798	80.80302	68.22249	-2033.61562	1795.30103	254.89480	1
C	73.50488	80.88175	68.59192	523.79067	517.99274	284.45581	1
H	74.00491	80.58689	67.64314	1599.61665	-1127.00911	1895.98173	1
H	73.48756	81.99381	68.61742	-1881.83093	1275.82488	-1501.55016	1
C	74.18500	80.29505	69.86101	-600.05639	-404.05026	316.64485	1
H	74.45824	79.22532	69.72461	-2597.05260	496.36221	657.73249	1
H	75.11868	80.82460	70.15317	-1666.24518	1294.38985	-1723.90591	1
C	73.00860	80.46149	70.93076	493.57570	-129.95376	-602.56394	1
H	72.93331	79.65186	71.69657	-1469.08415	-637.84797	2204.22581	1
H	73.00169	81.45161	71.44420	-1038.97840	1068.88701	-2280.71789	1
C	72.19607	78.70002	68.23516	188.88100	241.05089	-727.87821	1
O	72.45506	78.49464	67.07492	531.88613	-49.19943	427.07960	1
N	72.04423	77.80733	69.20689	708.14833	26.93417	-179.73509	1
H	71.82197	78.20869	70.11916	-2232.69501	-1179.09831	1023.94816	1
C	72.50775	76.43041	69.04723	-738.32412	259.92548	104.42456	1
H	72.33048	76.20054	67.96978	2028.89123	1260.02983	1311.33324	1
C	73.98145	76.40845	69.41939	-258.65678	-345.52513	661.28459	1
H	74.13808	77.14067	70.25578	-1826.06090	-1801.46097	-918.64554	1

129	C	74.43565	75.05048	69.91761	-541.23400	531.92970	-218.41428	1
130	H	75.55141	75.01356	70.06297	1311.73218	-2219.18200	882.19023	1
131	H	73.96794	74.79902	70.91200	-1048.71298	1223.58118	-2196.97971	1
132	H	74.16044	74.22820	69.19955	419.06414	1903.92898	1903.44670	1
133	C	74.84441	76.84398	68.25005	-423.86431	152.66566	-648.55262	1
134	H	75.92596	76.94808	68.54311	105.68386	-2342.50080	1387.51238	1
135	H	74.79688	76.10395	67.40325	-1695.63603	-1484.73621	-1531.02793	1
136	H	74.50816	77.83735	67.83555	-1853.57121	-487.39031	1936.59343	1
137	C	71.61716	75.50557	70.09921	539.71428	-510.58232	267.58633	1
138	O	71.58644	75.74182	71.28587	-15.93758	-278.34085	-624.49402	1
139	N	71.07927	74.48584	69.45551	-676.81886	12.05292	276.17960	1
140	H	71.24717	74.40930	68.45500	-1694.44250	-373.84189	-2100.66098	1
141	C	70.17876	73.63708	70.16437	412.33078	-664.70360	108.35451	1
142	H	69.84609	74.28519	71.00906	1765.77476	1294.44366	1621.76594	1
143	C	69.05193	73.20948	69.14295	-179.66635	-721.95667	-264.73915	1
144	H	68.62774	74.10524	68.63556	-607.34922	2587.87032	-598.10018	1
145	H	69.48447	72.58307	68.33074	-359.69332	-1479.13954	-2259.74260	1
146	C	67.93221	72.42040	69.89988	-240.01076	616.02425	431.85322	1
147	H	68.36184	71.51743	70.39485	-1359.17131	782.54425	2227.99181	1
148	H	67.48479	73.03854	70.71019	-820.36667	85.81767	-2596.78715	1
149	C	66.84115	72.01979	68.82617	-513.07015	236.18570	-551.87280	1
150	H	66.46552	72.92271	68.29209	1816.11944	-1183.11722	-1650.94321	1
151	H	67.28719	71.36896	68.03936	-1800.04429	-1361.53473	1526.34643	1
152	C	65.67688	71.29128	69.53537	726.08972	-253.19732	-179.64713	1
153	H	65.95037	70.32016	69.99386	1224.27271	-456.51798	-2390.90304	1
154	H	65.11877	71.91136	70.26491	1206.40339	-1150.93514	-2154.90344	1
155	N	64.61560	70.92369	68.44182	329.70796	-633.68968	-155.67358	1
156	H	63.75788	70.40625	68.79067	1348.31305	1938.76845	1359.00643	1
157	H	64.17064	71.72608	67.90811	2096.92035	1446.54551	-966.48890	1
158	H	64.93533	70.28757	67.65471	-1197.07217	-2303.53719	827.28733	1
159	C	71.01906	72.32900	70.69777	-378.01186	-556.17301	-413.96481	1
160	O	70.62398	71.20363	70.50268	-409.44433	-307.55053	-453.30833	1
161	N	72.08470	72.72481	71.38193	-429.02477	524.70884	274.08502	1
162	H	72.25034	73.73074	71.47745	1706.34263	-1425.96036	1574.38926	1
163	C	72.96575	71.73197	71.89685	-45.25864	-626.22952	478.93267	1
164	H	72.33731	70.82847	72.07844	1988.63164	570.90773	1772.87287	1
165	C	74.09329	71.48313	70.80503	551.77128	-20.49526	-564.54996	1
166	H	73.64617	71.34240	69.79622	1434.61535	-1753.10253	1513.99471	1
167	H	74.74558	72.38053	70.71947	-714.67738	1819.09770	1898.36494	1
168	C	74.91901	70.22935	71.23927	446.99263	-555.77305	338.97221	1
169	H	74.60699	69.88618	72.25046	1953.68466	1892.25957	-161.67766	1
170	H	74.72822	69.36953	70.55896	-403.03785	2688.07032	-188.43591	1
171	C	76.45400	70.60630	71.25859	567.86262	335.74681	434.04421	1
172	H	76.81468	70.86770	70.23789	-1080.02275	1568.26081	-1948.78900	1
173	H	76.63019	71.51249	71.87982	-919.54108	2111.34692	1456.13463	1
174	C	77.22348	69.38461	71.82507	195.81897	173.33862	-745.11536	1
175	H	76.92149	69.07632	72.84756	1405.67949	-2301.21089	-390.08113	1
176	H	77.22251	68.50303	71.15231	-940.37328	-1118.32446	2299.72133	1
177	N	78.71766	69.77201	71.95110	319.26422	656.36007	-42.02424	1
178	H	79.43618	68.91429	71.97014	1961.03633	619.91968	1787.09422	1
179	H	79.09610	70.39846	71.18458	854.14343	-2156.80752	1429.08711	1
180	H	78.97402	70.33808	72.81134	1237.02905	-1378.50857	-1998.28500	1
181	C	73.77089	72.35464	73.17713	532.53319	-268.57236	517.55729	1
182	O	74.19509	73.48107	73.16062	266.16714	-290.73804	-558.88002	1
183	N	74.07226	71.35075	74.02492	-226.25352	640.49678	-270.32839	1
184	H	73.50745	70.50967	73.88734	2007.69063	-1035.27229	1523.50231	1
185	C	74.33939	71.72363	75.39628	-540.76542	-573.33303	49.50000	1
186	H	73.56583	72.48466	75.63991	-2374.20950	-1036.76023	843.75774	1
187	C	74.19071	70.38186	76.22664	-77.09082	666.87422	-415.84136	1
188	H	75.08697	69.73123	76.03147	-985.81834	617.19825	2463.91927	1
189	O	73.03847	69.56581	75.90403	293.29899	-391.65602	-477.80926	1
190	H	73.46540	68.72334	75.65993	2528.72714	-681.76262	-751.27122	1

C	74.24576	70.91470	77.70582	252.47363	-605.14473	440.05134	1
H	74.23954	70.07661	78.44081	-2052.65063	1456.49051	-1043.51830	1
H	75.16372	71.52312	77.89765	-2152.98800	-1661.97574	-161.70652	1
H	73.36578	71.55966	77.93553	-473.12726	-1584.27339	2165.61725	1
C	75.85060	72.31790	75.52118	360.36307	488.46713	505.10056	1
O	76.15661	72.92313	76.52947	-200.08817	-528.03552	-385.82850	1
N	76.62385	71.92353	74.51151	-597.54095	-226.44775	355.20479	1
H	76.09014	71.44728	73.78478	2179.23655	-1300.70981	-991.33835	1
C	77.88124	72.51117	74.21257	-687.68921	297.39807	-249.45179	1
H	77.92902	72.47161	73.10238	-349.16256	-2467.37713	-1101.72831	1
C	78.04263	73.99339	74.78905	672.47345	361.54328	-201.62880	1
H	77.76587	74.00462	75.87803	1541.41502	582.18425	2169.97026	1
H	79.11801	74.28343	74.72229	-2534.67717	60.25153	997.72295	1
C	77.17629	74.99563	74.00830	574.26774	-389.43119	-377.02542	1
C	77.64223	75.60480	72.80396	688.82679	-287.33580	-257.96133	1
H	78.63123	75.33954	72.38892	331.14182	1470.38163	2269.80013	1
C	75.89012	75.33064	74.52443	-383.52382	-675.04808	144.24917	1
H	75.51280	74.83062	75.43840	4.09847	1517.59662	2262.86397	1
C	76.83375	76.55353	72.16750	-134.76730	428.45285	-649.50303	1
H	77.20510	77.02473	71.24059	-903.23433	1600.49020	2011.53387	1
C	75.11832	76.28259	73.84808	-335.53281	-615.00040	-364.39058	1
H	74.11975	76.53412	74.24363	1596.45398	-1369.33862	1732.02659	1
C	75.55361	76.93778	72.66269	585.48547	-340.24194	-406.23938	1
O	74.75162	77.93145	72.01136	-419.15649	-173.04408	-511.94141	1
H	73.92747	77.83977	72.54909	-1071.84350	1559.71507	-1960.13012	1
C	79.08410	71.65510	74.88623	291.41878	-183.13091	710.72246	1
O	80.18906	72.14761	74.96038	379.87695	523.47396	-222.23622	1
N	78.73867	70.38725	75.16412	564.30591	373.09380	277.24371	1
H	77.74057	70.19171	75.08421	-120.59931	-2436.07824	-1214.35098	1
C	79.59142	69.67717	76.07251	404.07514	-299.37480	608.84009	1
H	80.55294	70.24569	76.06549	-1071.07882	-841.69025	2359.66467	1
C	78.88482	69.68575	77.49664	-683.12807	68.71770	-390.13096	1
H	78.55816	70.71414	77.76864	2068.88918	1596.58590	770.89603	1
H	77.95545	69.07387	77.47036	1379.86429	2342.49779	-179.85638	1
C	79.88949	69.11758	78.54512	-176.39967	537.49466	550.97268	1
H	80.23950	68.09555	78.24285	-396.28051	1942.22168	1869.33314	1
H	80.80873	69.74363	78.59112	-94.44700	586.00678	-2659.20016	1
C	79.17173	69.05532	79.97005	-291.69381	-718.18079	-150.72929	1
H	78.97366	70.04334	80.45811	-2303.38776	1089.76242	964.62032	1
H	78.23208	68.44812	80.01981	884.16729	2568.80533	-207.74283	1
N	80.15613	68.38265	80.71288	-594.49522	-252.81478	342.29276	1
H	81.01508	68.15646	80.23532	-1735.17426	2084.05705	-263.71605	1
C	80.03876	68.04211	81.93219	595.23177	-271.52487	442.22352	1
N	78.84339	68.36313	82.58445	585.53813	-424.79750	-105.81303	1
H	78.02112	68.84520	82.22376	959.88797	1327.56823	-2177.11905	1
H	78.63106	68.14481	83.55836	1578.48647	16.27378	-2220.76233	1
N	81.19147	67.38582	82.38699	-452.17763	-390.33541	-421.51853	1
H	82.05564	67.14346	81.90466	470.26107	1974.25629	-1817.92087	1
H	81.29319	67.02650	83.33684	1856.67307	-1573.97321	-1224.35416	1
C	79.73670	68.12372	75.64735	-531.47271	-581.45780	-55.06206	1
O	80.35423	67.34570	76.34310	-243.00837	192.73507	609.52485	1
N	79.02858	67.81020	74.54913	343.49288	-644.25607	38.09164	1
H	78.69011	68.61804	74.02988	-1691.16043	-1535.97525	-1484.73099	1
C	79.27798	66.49576	73.93289	542.66492	350.14337	454.42563	1
H	79.58662	65.85056	74.79297	1209.57979	-2396.95920	463.86744	1
C	78.00553	66.05992	73.22173	-737.14309	145.93010	242.71942	1
H	77.76807	66.82844	72.45369	-1050.18303	159.36419	-2509.06125	1
C	78.20208	64.73150	72.51563	-706.77696	-252.88343	-245.16230	1
H	77.28341	64.42090	71.94558	-1624.64039	-1665.49085	1417.86935	1
H	79.05414	64.77617	71.78146	2566.10521	598.51109	693.22305	1
H	78.43790	63.90712	73.24442	-1972.69758	107.10018	-1876.34312	1
C	76.82649	65.98259	74.17301	412.85148	672.58832	-27.69222	1

```
H    75.87286   65.71897   73.63731      1447.02277      1762.76533     -1490.78944           1
H    76.99024   65.20246   74.96735      1320.77379      -145.20455      2378.68491           1
H    76.65923   66.96234   74.70094     -1512.10848      1470.52031      1724.75147           1
C    80.46239   66.69328   72.80626      -607.11113       499.56689        73.73769           1
O    80.44460   67.65924   72.03841       357.97126       460.81534       356.68785           1
N    81.30008   65.70082   72.86342       536.55407      -368.62270       332.76306           1
H    81.14627   64.98911   73.57611       524.38866      2297.69985      1367.20941           1
C    82.12635   65.39275   71.72790        46.50277      -774.29190      -147.97904           1
H    81.38475   65.12502   70.94333      1676.51875      1883.29066      1032.55549           1
C    82.94296   66.71833   71.31932      -559.41456       -34.19198      -556.30433           1
H    82.74048   67.51019   72.08339       -64.66664       395.18769      2695.05373           1
H    84.04515   66.53761   71.32977     -2703.44278       -60.40056       333.79418           1
C    82.48154   67.17342   69.95848      -519.76435       377.31045       459.42366           1
H    81.37049   67.27098   69.98803     -1746.74323      1667.50644     -1261.73691           1
C    83.07042   68.54170   69.63762       570.95554        16.46261      -545.27700           1
H    82.71967   68.92027   68.64309      2391.58273        28.05277     -1305.07271           1
H    82.77951   69.30437   70.40579       175.75588       917.63458      2559.43867           1
H    84.19002   68.51177   69.60580     -1063.97851     -2155.34650      1283.00462           1
C    82.85730   66.18325   68.86573       193.76014         2.62946      -765.53141           1
H    82.50448   66.52840   67.85961       507.39394      -846.55087      2539.60089           1
H    83.96727   66.04602   68.79681      1337.57841      1818.19054      1526.01994           1
H    82.40822   65.17207   69.04347      1100.88635     -1172.91350     -2199.08909           1
C    83.03585   64.15828   72.16682      -549.21115       475.64486      -309.38218           1
O    82.77863   63.46707   73.12032       556.06106      -390.37755       -78.24250           1
O    84.12741   63.83156   71.32007      -572.27940      -310.19032      -209.75862           1
H    84.01138   64.54073   70.63466     -1380.08193      2212.14486       790.85944           1
```

Listing 16: C00016.xmolout

```
       276
  150.0000  150.0000  150.0000    0.0000    0.0000    0.0000***********    2.7750    11100
       0.2500    300.00   0.00   0.00   0.00  -1294.0794      0.0000    300.0000
H    73.51008   68.00272   85.91873     -2043.14101     -1757.64893       399.89339           1
N    74.37950   67.76352   85.44411       426.75047       490.45733      -334.42467           1
H    74.05490   67.38443   84.54964       442.25559     -1110.30854     -2448.52907           1
C    75.24949   68.85554   85.24031       415.32972      -666.39213        83.73172           1
H    75.06718   69.60390   86.04164       879.32219      2491.00447       667.35213           1
C    76.76091   68.28828   85.22154        31.35357       477.41777       628.23362           1
H    76.85394   67.54584   84.39280      1168.38489      2240.25337     -1019.71136           1
H    77.48320   69.10935   84.98806      -360.43770      2534.90498       931.67112           1
C    77.10442   67.63903   86.53644       463.18208       512.70242      -382.34388           1
H    76.26026   66.97419   86.82841      1725.29117      1863.52247      -987.07854           1
C    78.36202   66.79175   86.38525       710.42801      -144.27427      -313.15367           1
H    78.64021   66.28699   87.34604       364.20195     -1033.13725      2494.72524           1
H    78.23008   65.98938   85.61409      2567.61904       335.80679       847.48802           1
H    79.24036   67.41222   86.07008     -2587.99029       680.92867      -512.16411           1
C    77.31541   68.67821   87.62873       268.17616       741.64379       -40.42634           1
H    77.58415   68.20093   88.60651      1283.54314     -1945.37420     -1411.27812           1
H    78.14346   69.38697   87.36759     -1258.57796      1317.15249     -2026.02085           1
H    76.39418   69.29232   87.80044     -2502.84526       909.80597       575.92391           1
C    74.90008   69.36208   83.73387       619.36578      -458.64843      -172.09273           1
O    74.47561   68.60167   82.88531       -81.40433       433.87611       522.34594           1
N    75.26659   70.63353   83.59993       175.23996      -622.20190       341.55523           1
H    75.64141   71.12784   84.40901      -876.57256      -303.22879     -2561.90213           1
C    75.27334   71.20099   82.30027      -571.88214      -534.10783      -106.15033           1
H    74.83011   70.40809   81.65141     -2713.20841       -16.25800      -248.80809           1
C    74.47431   72.59246   82.33937       166.60354       288.16399      -716.09568           1
H    75.08064   73.32554   82.94268     -2097.85574      1636.78449      -586.18044           1
O    73.16870   72.55429   82.93758      -317.80580      -363.38227       484.43082           1
H    73.29194   73.15247   83.70297      1863.74748     -1451.84923      1357.29467           1
C    74.50987   73.01300   80.82388       503.80731       517.99032       318.51606           1
H    74.06967   74.02592   80.67223       640.61020     -2157.10410     -1536.29104           1
```

H	75.55303	73.04096	80.42533	1687.88455	465.60692	2087.56452	1
H	73.92927	72.30287	80.18971	-1293.07895	-1899.88581	-1463.57452	1
C	76.81649	71.62406	81.93992	-153.07550	-733.68902	248.70981	1
O	77.11848	72.78109	82.07155	186.15459	-480.78787	-449.34387	1
N	77.58508	70.57208	81.62892	-487.53139	543.18517	42.03850	1
C	78.84231	70.74302	80.96483	-699.35871	69.34393	360.10701	1
H	79.06304	71.83074	81.07181	2.81126	-221.99807	-2715.58072	1
C	79.75922	69.76556	81.82149	-507.46439	-592.59194	122.07761	1
H	80.71710	69.50456	81.31902	-1975.08116	525.87560	-1801.71556	1
H	80.03429	70.23000	82.79420	1588.27743	-1913.22100	1113.83566	1
C	78.81002	68.54605	82.04105	-613.62442	-457.55993	-194.14490	1
H	78.75585	67.89729	81.13930	1707.54637	-721.27992	1996.92523	1
H	79.11846	67.88681	82.88384	758.23357	2100.81767	1560.55011	1
C	77.43214	69.28860	82.30739	-352.41524	704.96076	49.21465	1
H	76.52952	68.77502	81.89638	1714.76886	-2070.42778	443.35792	1
H	77.24681	69.56290	83.37215	1224.15040	1801.50881	1636.97633	1
C	78.60270	70.30087	79.41111	566.85117	537.05831	117.62814	1
O	77.78181	70.92951	78.74268	-550.26779	-406.08867	4.21666	1
N	79.31014	69.25740	79.07295	566.34832	-425.77725	180.18749	1
H	79.97002	68.88358	79.75117	644.44502	2646.80550	-52.73430	1
C	79.38377	68.81591	77.71055	612.34801	-281.48662	411.56221	1
H	79.69928	69.70440	77.12081	1667.23498	-1490.75142	1556.16753	1
C	80.44341	67.62853	77.72468	-714.45746	-175.43074	286.98927	1
H	80.05523	66.80452	78.38601	-787.09484	1763.03619	-1922.46060	1
O	81.74594	67.95611	78.25328	-429.46514	516.51932	-128.40526	1
H	81.82714	67.30845	78.98026	1981.82785	436.16847	-1818.18182	1
C	80.39513	67.14900	76.22759	630.79189	-468.94448	76.03230	1
H	80.99225	66.21919	76.07880	-1775.71861	2062.30353	131.89849	1
H	79.34965	66.93350	75.89600	-1855.14608	88.94118	1993.53760	1
H	80.80921	67.92205	75.53977	-1504.82350	-780.94249	-2132.90992	1
C	77.90459	68.28806	77.25565	288.57638	618.62814	-397.00270	1
O	77.58107	68.36858	76.09340	321.70495	-185.26452	574.37218	1
N	77.33435	67.62602	78.26932	-447.05792	-446.53431	367.76425	1
H	77.88104	67.71199	79.12793	350.31316	1975.63644	-1843.31522	1
C	75.90723	67.55549	78.40817	-511.23061	374.03917	-471.51480	1
H	75.83881	66.82199	79.24191	2234.79579	-898.70890	-1273.45190	1
C	75.41835	68.98570	78.91009	529.53781	-159.34528	563.72604	1
H	74.37986	68.83498	79.31306	654.59388	2347.94251	1217.51488	1
H	76.07170	69.27956	79.77559	-1526.55661	2103.42026	817.87394	1
O	75.37679	70.07086	77.96190	-554.84278	14.12789	399.58769	1
H	76.28710	70.49593	78.15014	1297.06236	2061.84854	1220.68812	1
C	75.28544	66.95786	77.04371	-662.15484	-412.13544	123.62687	1
O	75.59402	65.84877	76.67748	-540.01537	-317.05804	274.91456	1
N	74.38241	67.79589	76.46709	-202.76299	-690.00100	-131.49145	1
H	74.27040	68.68310	76.98124	-702.62132	-2617.37191	281.70413	1
C	74.72183	68.01919	75.06511	-491.73914	576.34187	222.73509	1
H	75.82555	67.84320	75.01996	1977.17978	-1823.58850	-434.68928	1
C	73.87673	67.03280	74.17197	-649.50797	-328.66843	306.11224	1
H	72.80220	67.07168	74.45480	2075.07803	1452.16490	-1004.45904	1
C	74.07200	67.47294	72.67822	-615.18735	-489.99689	-70.96352	1
H	73.51930	66.80607	71.97589	-1226.32661	-1724.38906	-1716.47170	1
H	73.70010	68.51208	72.51003	2381.64273	-969.84735	900.46860	1
H	75.14635	67.45623	72.38033	2122.74991	1702.23946	-141.36228	1
C	74.43340	65.57334	74.33138	-501.86074	-479.61276	-376.42482	1
H	74.14885	64.92263	73.47090	993.79261	-2246.95401	-1177.81305	1
H	75.54848	65.56821	74.40915	42.71279	-626.15391	-2651.37258	1
H	74.04280	65.08876	75.25983	-2026.39012	-1590.33709	-887.82922	1
C	74.27965	69.54618	74.71837	-672.89153	382.57394	-156.34072	1
O	73.12239	69.91523	74.65123	410.04627	221.86223	500.35950	1
N	75.38464	70.25036	74.51176	-378.11782	-443.79414	-441.10984	1
H	76.29727	69.79953	74.59244	2404.15973	-568.14894	1149.30085	1
C	75.21000	71.58465	74.02384	-544.54484	466.16019	331.29119	1

H	74.28596	71.92448	74.54323	-1409.80988	-170.17955	2325.32679	1
C	76.52404	72.38244	74.38454	-184.48284	116.78282	-758.89137	1
H	76.74466	72.29047	75.47243	1893.78641	1861.14652	611.04528	1
H	77.41133	71.97020	73.85337	-2170.79665	-974.19311	1327.50117	1
C	76.24689	73.87585	73.98096	389.51668	672.81878	-138.49152	1
H	76.32275	73.99280	72.87409	510.64577	1933.03327	1851.02484	1
H	75.18718	74.14001	74.19492	-2302.30869	-1105.72939	-948.89774	1
C	77.14541	74.90865	74.69171	-559.93427	-126.34590	542.30857	1
O	76.76148	76.13740	74.73126	-670.36003	-111.83166	76.36448	1
N	78.23259	74.40058	75.19287	-216.06729	-537.07037	446.51379	1
H	78.46753	73.40047	75.10268	-1251.96487	1074.12894	-2168.52500	1
H	78.97357	74.90159	75.77677	-2451.83023	-720.73851	944.84612	1
C	75.03651	71.50171	72.39297	546.24052	-563.88337	-85.11809	1
O	75.91389	71.04020	71.71311	71.70702	-307.40463	606.69673	1
N	73.85856	72.02928	72.02129	479.90863	-504.50129	222.86849	1
C	73.52681	72.20717	70.64395	75.51268	-600.91904	506.73700	1
H	74.05372	71.41759	70.06025	-2033.61562	1795.30103	254.89480	1
C	71.93903	72.10108	70.68917	523.79067	517.99274	284.45581	1
H	71.44866	72.53855	69.79087	1599.61665	-1127.00911	1895.98173	1
H	71.59790	71.04443	70.76100	-1881.83093	1275.82488	-1501.55016	1
C	71.63423	72.88908	71.99938	-600.05639	-404.05026	316.64485	1
H	71.70675	73.98401	71.81063	-2597.05260	496.36221	657.73249	1
H	70.61772	72.70012	72.41284	-1666.24518	1294.38985	-1723.90591	1
C	72.80348	72.39852	72.95526	493.57570	-129.95376	-602.56394	1
H	73.20157	73.18841	73.63495	-1469.08415	-637.84797	2204.22581	1
H	72.59368	71.45809	73.55237	-1038.97840	1068.88701	-2280.71789	1
C	73.95461	73.72751	70.21443	188.88100	241.05089	-727.87821	1
O	74.16011	74.60664	71.01315	531.88613	-49.19943	427.07960	1
N	73.83096	73.87293	68.85185	708.14833	26.93417	-179.73509	1
H	73.77854	72.89242	68.55337	-2232.69501	-1179.09831	1023.94816	1
C	74.74668	74.82670	68.17829	-738.32412	259.92548	104.42456	1
H	75.74789	74.63469	68.62685	2028.89123	1260.02983	1311.33324	1
C	74.68442	74.54612	66.69776	-258.65678	-345.52513	661.28459	1
H	73.60992	74.48172	66.41179	-1826.06090	-1801.46097	-918.64554	1
C	75.32095	75.66495	65.89071	-541.23400	531.92970	-218.41428	1
H	75.18676	75.51187	64.78471	1311.73218	-2219.18200	882.19023	1
H	74.87281	76.66534	66.15332	-1048.71298	1223.58118	-2196.97971	1
H	76.42533	75.73886	66.08744	419.06414	1903.92898	1903.44670	1
C	75.33984	73.22504	66.33202	-423.86431	152.66566	-648.55262	1
H	75.26319	73.01441	65.23027	105.68386	-2342.50080	1387.51238	1
H	76.43278	73.22122	66.59584	-1695.63603	-1484.73621	-1531.02793	1
H	74.86717	72.35925	66.87049	-1853.57121	-487.39031	1936.59343	1
C	74.20831	76.34976	68.49562	539.71428	-510.58232	267.58633	1
O	74.95511	77.29030	68.35123	-15.93758	-278.34085	-624.49402	1
N	72.89595	76.36835	68.73640	-676.81886	12.05292	276.17960	1
H	72.48093	75.43984	68.81219	-1694.44250	-373.84189	-2100.66098	1
C	72.39301	77.44214	69.54198	412.33078	-664.70360	108.35451	1
H	73.28726	77.97938	69.94416	1765.77476	1294.44366	1621.76594	1
C	71.52379	78.31164	68.54615	-179.66635	-721.95667	-264.73915	1
H	72.13421	78.62517	67.66983	-607.34922	2587.87032	-598.10018	1
H	70.69491	77.69748	68.12742	-359.69332	-1479.13954	-2259.74260	1
C	70.96051	79.55673	69.30713	-240.01076	616.02425	431.85322	1
H	70.35809	79.24509	70.19003	-1359.17131	782.54425	2227.99181	1
H	71.78243	80.19359	69.70372	-820.36667	85.81767	-2596.78715	1
C	70.07316	80.34973	68.26416	-513.07015	236.18570	-551.87280	1
H	70.68452	80.66693	67.38837	1816.11944	-1183.11722	-1650.94321	1
H	69.27328	79.69342	67.85038	-1800.04429	-1361.53473	1526.34643	1
C	69.45362	81.57775	68.96935	726.08972	-253.19732	-179.64713	1
H	68.73711	81.33378	69.77892	1224.27271	-456.51798	-2390.90304	1
H	70.18826	82.32635	69.32767	1206.40339	-1150.93514	-2154.90344	1
N	68.59878	82.33848	67.89890	329.70796	-633.68968	-155.67358	1
H	68.10372	83.21041	68.24442	1348.31305	1938.76845	1359.00643	1

157	H	69.11153	82.71446	67.04897	2096.92035	1446.54551	-966.48890	1
158	H	67.79603	81.80616	67.45266	-1197.07217	-2303.53719	827.28733	1
159	C	71.38586	76.79954	70.65245	-378.01186	-556.17301	-413.96481	1
160	O	70.48942	76.05724	70.33375	-409.44433	-307.55053	-453.30833	1
161	N	71.71827	77.25175	71.86351	-429.02477	524.70884	274.08502	1
162	H	72.52675	77.90769	71.97232	1706.34263	-1425.96036	1574.38926	1
163	C	70.64784	77.21985	72.84002	-45.25864	-626.22952	478.93267	1
164	H	69.78123	77.71503	72.34938	1988.63164	570.90773	1772.87287	1
165	C	70.36182	75.69981	73.14880	551.77128	-20.49526	-564.54996	1
166	H	70.04090	75.17432	72.21476	1434.61535	-1753.10253	1513.99471	1
167	H	71.29778	75.19408	73.47835	-714.67738	1819.09770	1898.36494	1
168	C	69.25946	75.57514	74.25316	446.99263	-555.77305	338.97221	1
169	H	69.59554	76.04763	75.20384	1953.68466	1892.25957	-161.67766	1
170	H	68.32826	76.10731	73.95611	-403.03785	2688.07032	-188.43591	1
171	C	68.99608	74.02678	74.44218	567.86262	335.74681	434.04421	1
172	H	68.62555	73.57303	73.49433	-1080.02275	1568.26081	-1948.78900	1
173	H	69.94422	73.49238	74.68213	-919.54108	2111.34692	1456.13463	1
174	C	67.96404	73.83505	75.57703	195.81897	173.33862	-745.11536	1
175	H	68.32318	74.13154	76.58273	1405.67949	-2301.21089	-390.08113	1
176	H	66.97072	74.28574	75.38117	-940.37328	-1118.32446	2299.72133	1
177	N	67.68547	72.29747	75.69254	319.26422	656.36007	-42.02424	1
178	H	67.00473	72.00735	76.45247	1961.03633	619.91968	1787.09422	1
179	H	67.26772	71.81666	74.84342	854.14343	-2156.80752	1429.08711	1
180	H	68.51707	71.67453	75.90933	1237.02905	-1378.50857	-1998.28500	1
181	C	71.14098	77.95965	74.19348	532.53319	-268.57236	517.55729	1
182	O	70.35631	78.61849	74.87200	266.16714	-290.73804	-558.88002	1
183	N	72.38969	77.64429	74.47619	-226.25352	640.49678	-270.32839	1
184	H	72.90728	77.09093	73.79581	2007.69063	-1035.27229	1523.50231	1
185	C	72.93233	78.10745	75.71766	-540.76542	-573.33303	49.50000	1
186	H	72.04708	78.08152	76.39495	-2374.20950	-1036.76023	843.75774	1
187	C	74.10632	77.09373	76.09800	-77.09082	666.87422	-415.84136	1
188	H	74.83821	77.08989	75.24398	-985.81834	617.19825	2463.91927	1
189	O	73.67319	75.73120	76.28689	293.29899	-391.65602	-477.80926	1
190	H	74.25372	75.26961	75.64890	2528.72714	-681.76262	-751.27122	1
191	C	74.81384	77.75016	77.34006	252.47363	-605.14473	440.05134	1
192	H	75.63046	77.09275	77.72590	-2052.65063	1456.49051	-1043.51830	1
193	H	75.28811	78.72757	77.07232	-2152.98800	-1661.97574	-161.70652	1
194	H	74.10239	77.93167	78.17964	-473.12726	-1584.27339	2165.61725	1
195	C	73.41320	79.63774	75.43534	360.36307	488.46713	505.10056	1
196	O	74.59706	79.93031	75.32532	-200.08817	-528.03552	-385.82850	1
197	N	72.33539	80.38359	75.41838	-597.54095	-226.44775	355.20479	1
198	H	71.40024	79.91496	75.49180	2179.23655	-1300.70981	-991.33835	1
199	C	72.37184	81.76006	75.10046	-687.68921	297.39807	-249.45179	1
200	H	71.34531	82.10441	75.35343	-349.16256	-2467.37713	-1101.72831	1
201	C	73.52567	82.51035	75.91878	672.47345	361.54328	-201.62880	1
202	H	74.52834	82.16468	75.57281	1541.41502	582.18425	2169.97026	1
203	H	73.47287	83.60449	75.71520	-2534.67717	60.25153	997.72295	1
204	C	73.36567	82.22416	77.41839	574.26774	-389.43119	-377.02542	1
205	C	72.36773	82.89245	78.18873	688.82679	-287.33580	-257.96133	1
206	H	71.70211	83.64198	77.72348	331.14182	1470.38163	2269.80013	1
207	C	74.22161	81.25645	78.02584	-383.52382	-675.04808	144.24917	1
208	H	74.99279	80.73939	77.42343	4.09847	1517.59662	2262.86397	1
209	C	72.25001	82.57210	79.54110	-134.76730	428.45285	-649.50303	1
210	H	71.47927	83.08796	80.13964	-903.23433	1600.49020	2011.53387	1
211	C	74.06223	80.96654	79.38227	-335.53281	-615.00040	-364.39058	1
212	H	74.72692	80.21168	79.83789	1596.45398	-1369.33862	1732.02659	1
213	C	73.07707	81.60870	80.18826	585.48547	-340.24194	-406.23938	1
214	O	72.90457	81.32189	81.56745	-419.15649	-173.04408	-511.94141	1
215	H	73.62348	80.65742	81.68974	-1071.84350	1559.71507	-1960.13012	1
216	C	72.68838	81.92957	73.50165	291.41878	-183.13091	710.72246	1
217	O	71.91360	82.52639	72.79624	379.87695	523.47396	-222.23622	1
218	N	73.87698	81.41662	73.19545	564.30591	373.09380	277.24371	1

219	H	74.45228	80.92459	73.92165	-120.59931	-2436.07824	-1214.35098	1
220	C	74.11970	81.17619	71.80157	404.07514	-299.37480	608.84009	1
221	H	73.09791	81.20253	71.35902	-1071.07882	-841.69025	2359.66467	1
222	C	74.96659	82.43483	71.34436	-683.12807	68.71770	-390.13096	1
223	H	74.52211	83.35146	71.79368	2068.88918	1596.58590	770.89603	1
224	H	76.00734	82.39587	71.73592	1379.86429	2342.49779	-179.85638	1
225	C	74.95464	82.54463	69.78938	-176.39967	537.49466	550.97268	1
226	H	75.49737	81.69075	69.31130	-396.28051	1942.22168	1869.33314	1
227	H	73.91682	82.51324	69.38756	-94.44700	586.00678	-2659.20016	1
228	C	75.65313	83.94851	69.47733	-291.69381	-718.18079	-150.72929	1
229	H	75.13562	84.84735	69.89927	-2303.38776	1089.76242	964.62032	1
230	H	76.73163	84.01206	69.77173	884.16729	2568.80533	-207.74283	1
231	N	75.59243	84.05285	68.08183	-594.49522	-252.81478	342.29276	1
232	H	75.27976	83.23717	67.57909	-1735.17426	2084.05705	-263.71605	1
233	C	75.90980	85.09377	67.42525	595.23177	-271.52487	442.22352	1
234	N	76.34836	86.21125	68.14504	585.53813	-424.79750	-105.81303	1
235	H	76.46416	86.33100	69.15050	959.88797	1327.56823	-2177.11905	1
236	H	76.63205	87.09854	67.72856	1578.48647	16.27378	-2220.76233	1
237	N	75.74843	84.89576	66.04675	-452.17763	-390.33541	-421.51853	1
238	H	75.43099	84.08409	65.51898	470.26107	1974.25629	-1817.92087	1
239	H	75.95496	85.61663	65.35398	1856.67307	-1573.97321	-1224.35416	1
240	C	74.81471	79.70523	71.72642	-531.47271	-581.45780	-55.06206	1
241	O	74.08151	78.72404	71.76392	-243.00837	192.73507	609.52485	1
242	N	76.14168	79.73473	71.72342	343.49288	-644.25607	38.09164	1
243	H	76.49407	80.69177	71.72408	-1691.16043	-1535.97525	-1484.73099	1
244	C	76.90819	78.67389	71.06322	542.66492	350.14337	454.42563	1
245	H	76.12789	78.00440	70.62460	1209.57979	-2396.95920	463.86744	1
246	C	77.80331	79.33874	70.03025	-737.14309	145.93010	242.71942	1
247	H	78.14706	80.31407	70.44127	-1050.18303	159.36419	-2509.06125	1
248	C	79.04395	78.51678	69.72413	-706.77696	-252.88343	-245.16230	1
249	H	79.60888	78.91222	68.83434	-1624.64039	-1665.49085	1417.86935	1
250	H	79.75741	78.50515	70.59892	2566.10521	598.51109	693.22305	1
251	H	78.77844	77.43947	69.51748	-1972.69758	107.10018	-1876.34312	1
252	C	77.04308	79.62482	68.75066	412.85148	672.58832	-27.69222	1
253	H	77.70879	80.07369	67.96091	1447.02277	1762.76533	-1490.78944	1
254	H	76.58529	78.68154	68.32109	1320.77379	-145.20455	2378.68491	1
255	H	76.19379	80.35188	68.93081	-1512.10848	1470.52031	1724.75147	1
256	C	77.87031	77.86509	72.14075	-607.11113	499.56689	73.73769	1
257	O	78.54139	76.93919	71.73618	357.97126	460.81534	356.68785	1
258	N	77.85165	78.37684	73.36185	536.55407	-368.62270	332.76306	1
259	H	77.19078	79.13024	73.54460	524.38866	2297.69985	1367.20941	1
260	C	78.73059	77.79058	74.30125	46.50277	-774.29190	-147.97904	1
261	H	79.23105	76.96538	73.73774	1676.51875	1883.29066	1032.55549	1
262	C	79.74314	78.90872	74.88036	-559.41456	-34.19198	-556.30433	1
263	H	79.15873	79.78804	75.24253	-64.66664	395.18769	2695.05373	1
264	H	80.27141	78.49390	75.77390	-2703.44278	-60.40056	333.79418	1
265	C	80.73037	79.32218	73.82190	-519.76435	377.31045	459.42366	1
266	H	80.17133	79.56041	72.88903	-1746.74323	1667.50644	-1261.73691	1
267	C	81.48963	80.56606	74.26728	570.95554	16.46261	-545.27700	1
268	H	82.23947	80.89130	73.50084	2391.58273	28.05277	-1305.07271	1
269	H	80.80029	81.43094	74.44713	175.75588	917.63458	2559.43867	1
270	H	82.04932	80.38567	75.22127	-1063.97851	-2155.34650	1283.00462	1
271	C	81.71934	78.20143	73.53014	193.76014	2.62946	-765.53141	1
272	H	82.49605	78.51692	72.78657	507.39394	-846.55087	2539.60089	1
273	H	82.26017	77.87934	74.45768	1337.57841	1818.19054	1526.01994	1
274	H	81.20942	77.29516	73.11240	1100.88635	-1172.91350	-2199.08909	1
275	C	77.93656	77.26277	75.62103	-549.21115	475.64486	-309.38218	1
276	O	77.30984	77.90113	76.40118	556.06106	-390.37755	-78.24250	1
277	O	79.23324	76.46331	76.35169	-572.27940	-310.19032	-209.75862	1
278	H	79.10041	76.79193	77.21994	-1380.08193	2212.14486	790.85944	1

Listing 17: C00017.xmolout

```
      276
  150.0000  150.0000  150.0000     0.0000     0.0000     0.0000***********    2.7750    11100
      0.2500    300.00   0.00   0.00   0.00  -1294.0761       0.0000    300.0000
H    68.01668  88.30775  68.92051    -2043.14101    -1757.64893     399.89339        1
N    68.77492  88.78997  69.39946      426.75047      490.45733    -334.42467        1
H    69.10167  88.05903  70.02897      442.25559    -1110.30854   -2448.52907        1
C    69.75801  89.32942  68.53898      415.32972     -666.39213      83.73172        1
H    69.37033  89.19704  67.50595      879.32219     2491.00447     667.35213        1
C    69.95625  90.88049  68.92487       31.35357      477.41777     628.23362        1
H    70.28243  90.95806  69.98956     1168.38489     2240.25337   -1019.71136        1
H    70.77430  91.33790  68.31727     -360.43770     2534.90498     931.67112        1
C    68.65113  91.60692  68.71661      463.18208      512.70242    -382.34388        1
H    67.82892  90.95512  69.08962     1725.29117     1863.52247    -987.07854        1
C    68.62674  92.90631  69.51101      710.42801     -144.27427    -313.15367        1
H    67.65847  93.45468  69.37729      364.20195    -1033.13725    2494.72524        1
H    68.75527  92.72259  70.60908     2567.61904      335.80679     847.48802        1
H    69.44710  93.59991  69.19215    -2587.99029      680.92867    -512.16411        1
C    68.41361  91.90473  67.24270      268.17616      741.64379     -40.42634        1
H    67.43571  92.42731  67.07887     1283.54314    -1945.37420   -1411.27812        1
H    69.21553  92.56522  66.82216    -1258.57796     1317.15249   -2026.02085        1
H    68.39800  90.96956  66.62587    -2502.84526      909.80597     575.92391        1
C    71.13753  88.51167  68.81918      619.36578     -458.64843    -172.09273        1
O    71.81314  88.06746  67.90818      -81.40433      433.87611     522.34594        1
N    71.40173  88.49679  70.12273      175.23996     -622.20190     341.55523        1
H    70.69990  88.89389  70.74589     -876.57256     -303.22879   -2561.90213        1
C    72.39742  87.58164  70.57843     -571.88214     -534.10783    -106.15033        1
H    73.13086  87.53936  69.74307    -2713.20841      -16.25800    -248.80809        1
C    72.97343  88.13466  71.96291      166.60354      288.16399    -716.09568        1
H    72.12405  88.21899  72.69434    -2097.85574     1636.78449    -586.18044        1
O    73.57580  89.44036  71.89506     -317.80580     -363.38227     484.43082        1
H    72.98504  89.95307  72.48152     1863.74748    -1451.84923    1357.29467        1
C    73.93199  86.97179  72.41413      503.80731      517.99032     318.51606        1
H    74.43941  87.21733  73.37578      640.61020    -2157.10410   -1536.29104        1
H    73.39261  86.00484  72.57616     1687.88455      465.60692    2087.56452        1
H    74.73177  86.78592  71.66029    -1293.07895    -1899.88581   -1463.57452        1
C    71.60797  86.17328  70.84481     -153.07550     -733.68902     248.70981        1
O    70.76648  86.12932  71.70155      186.15459     -480.78787    -449.34387        1
N    72.05555  85.19714  70.03584     -487.53139      543.18517      42.03850        1
C    71.49541  83.88631  70.09228     -699.35871       69.34393     360.10701        1
H    70.46659  83.98249  70.50946        2.81126     -221.99807   -2715.58072        1
C    71.59105  83.43732  68.57064     -507.46439     -592.59194     122.07761        1
H    71.52876  82.33364  68.44090    -1975.08116      525.87560   -1801.71556        1
H    70.77114  83.87963  67.96174     1588.27743    -1913.22100    1113.83566        1
C    72.97746  84.03090  68.16804     -613.62442     -457.55993    -194.14490        1
H    73.80799  83.38808  68.53972     1707.54637     -721.27992    1996.92523        1
H    73.11899  84.12608  67.06758      758.23357     2100.81767    1560.55011        1
C    72.96095  85.42965  68.91654     -352.41524      704.96076      49.21465        1
H    73.94793  85.74621  69.32962     1714.76886    -2070.42778     443.35792        1
H    72.51031  86.29019  68.33411     1224.15040     1801.50881    1636.97633        1
C    72.44986  82.89276  70.98700      566.85117      537.05831     117.62814        1
O    72.11266  81.74777  71.17755     -550.26779     -406.08867       4.21666        1
N    73.56588  83.49989  71.37252      566.34832     -425.77725     180.18749        1
H    73.67283  84.48229  71.12943      644.44502     2646.80550     -52.73430        1
C    74.52483  82.76522  72.11309      612.34801     -281.48662     411.56221        1
H    74.25508  81.69151  71.97555     1667.23498    -1490.75142    1556.16753        1
C    75.96081  83.20401  71.56102     -714.45746     -175.43074     286.98927        1
H    76.10540  84.29948  71.77224     -787.09484     1763.03619   -1922.46060        1
O    76.16635  83.04923  70.14504     -429.46514      516.51932    -128.40526        1
H    76.35086  83.97221  69.87777     1981.82785      436.16847   -1818.18182        1
C    76.90922  82.36293  72.49211      630.79189     -468.94448      76.03230        1
```

60	H	77.98137	82.60543	72.30804	-1775.71861	2062.30353	131.89849	1
61	H	76.70703	82.55174	73.57225	-1855.14608	88.94118	1993.53760	1
62	H	76.78401	81.26892	72.31777	-1504.82350	-780.94249	-2132.90992	1
63	C	74.42973	83.29639	73.66972	288.57638	618.62814	-397.00270	1
64	O	74.43679	84.47673	73.93304	321.70495	-185.26452	574.37218	1
65	N	74.36127	82.23887	74.47697	-447.05792	-446.53431	367.76425	1
66	H	74.47797	81.34548	73.99752	350.31316	1975.63644	-1843.31522	1
67	C	74.78557	82.38504	75.84355	-511.23061	374.03917	-471.51480	1
68	H	75.83267	82.75474	75.78986	2234.79579	-898.70890	-1273.45190	1
69	C	73.82421	83.45651	76.53375	529.53781	-159.34528	563.72604	1
70	H	74.21389	83.61937	77.57553	654.59388	2347.94251	1217.51488	1
71	H	73.95548	84.43006	75.97627	-1526.55661	2103.42026	817.87394	1
72	O	72.41787	83.09437	76.63735	-554.84278	14.12789	399.58769	1
73	H	72.10843	83.42293	75.77240	1297.06236	2061.84854	1220.68812	1
74	C	74.65682	80.96749	76.60558	-662.15484	-412.13544	123.62687	1
75	O	75.28903	80.73767	77.60947	-540.01537	-317.05804	274.91456	1
76	N	73.76913	80.09964	75.99200	-202.76299	-690.00100	-131.49145	1
77	H	73.34845	80.72163	75.29108	-702.62132	-2617.37191	281.70413	1
78	C	72.84261	79.35361	76.88705	-491.73914	576.34187	222.73509	1
79	H	72.61857	80.06098	77.71859	1977.17978	-1823.58850	-434.68928	1
80	C	71.64220	79.00270	76.04134	-649.50797	-328.66843	306.11224	1
81	H	72.01793	78.54890	75.09577	2075.07803	1452.16490	-1004.45904	1
82	C	70.74746	77.98841	76.73275	-615.18735	-489.99689	-70.96352	1
83	H	69.86814	77.70385	76.09190	-1226.32661	-1724.38906	-1716.47170	1
84	H	71.30673	77.04247	76.97436	2381.64273	-969.84735	900.46860	1
85	H	70.34018	78.38882	77.70173	2122.74991	1702.23946	-141.36228	1
86	C	70.82412	80.22966	75.67664	-501.86074	-479.61276	-376.42482	1
87	H	69.95108	79.96744	75.01798	993.79261	-2246.95401	-1177.81305	1
88	H	70.41191	80.73737	76.59194	42.71279	-626.15391	-2651.37258	1
89	H	71.43551	80.99668	75.12631	-2026.39012	-1590.33709	-887.82922	1
90	C	73.59319	77.98814	77.38892	-672.89153	382.57394	-156.34072	1
91	O	74.12105	77.21079	76.59507	410.04627	221.86223	500.35950	1
92	N	73.37977	77.81304	78.67237	-378.11782	-443.79414	-441.10984	1
93	H	72.89726	78.56127	79.16578	2404.15973	-568.14894	1149.30085	1
94	C	74.18091	76.85886	79.36594	-544.54484	466.16019	331.29119	1
95	H	74.51014	76.14461	78.57693	-1409.80988	-170.17955	2325.32679	1
96	C	75.37273	77.70227	79.98912	-184.48284	116.78282	-758.89137	1
97	H	75.88771	78.28881	79.19383	1893.78641	1861.14652	611.04528	1
98	H	74.98561	78.45085	80.71756	-2170.79665	-974.19311	1327.50117	1
99	C	76.34540	76.69953	80.68122	389.51668	672.81878	-138.49152	1
100	H	75.81191	76.14284	81.48389	510.64577	1933.03327	1851.02484	1
101	H	76.65836	75.90872	79.96419	-2302.30869	-1105.72939	-948.89774	1
102	C	77.62096	77.40291	81.28311	-559.93427	-126.34590	542.30857	1
103	O	78.42217	76.74470	81.89451	-670.36003	-111.83166	76.36448	1
104	N	77.69788	78.72688	81.03004	-216.06729	-537.07037	446.51379	1
105	H	77.05727	79.33838	80.51002	-1251.96487	1074.12894	-2168.52500	1
106	H	78.47337	79.32366	81.33737	-2451.83023	-720.73851	944.84612	1
107	C	73.27936	76.21722	80.57470	546.24052	-563.88337	-85.11809	1
108	O	73.17920	76.80363	81.61965	71.70702	-307.40463	606.69673	1
109	N	72.74158	75.04120	80.20602	479.90863	-504.50129	222.86849	1
110	C	71.80631	74.36273	81.04247	75.51268	-600.91904	506.73700	1
111	H	71.39472	75.11419	81.75611	-2033.61562	1795.30103	254.89480	1
112	C	70.78430	73.78032	79.96934	523.79067	517.99274	284.45581	1
113	H	70.16863	72.94520	80.37242	1599.61665	-1127.00911	1895.98173	1
114	H	70.07700	74.55960	79.60870	-1881.83093	1275.82488	-1501.55016	1
115	C	71.76943	73.33732	78.84456	-600.05639	-404.05026	316.64485	1
116	H	72.26222	72.37315	79.10088	-2597.05260	496.36221	657.73249	1
117	H	71.27754	73.19157	77.85708	-1666.24518	1294.38985	-1723.90591	1
118	C	72.82472	74.52391	78.84499	493.57570	-129.95376	-602.56394	1
119	H	73.87693	74.20267	78.65786	-1469.08415	-637.84797	2204.22581	1
120	H	72.57299	75.37957	78.17445	-1038.97840	1068.88701	-2280.71789	1
121	C	72.45184	73.05092	81.78086	188.88100	241.05089	-727.87821	1

O	71.70018	72.20760	82.25709	531.88613	-49.19943	427.07960	1
N	73.77944	73.00564	81.71370	708.14833	26.93417	-179.73509	1
H	74.07436	73.84788	81.21663	-2232.69501	-1179.09831	1023.94816	1
C	74.63866	72.38415	82.71780	-738.32412	259.92548	104.42456	1
H	75.62973	72.87135	82.55551	2028.89123	1260.02983	1311.33324	1
C	74.07477	72.64565	84.10405	-258.65678	-345.52513	661.28459	1
H	73.02504	72.26931	84.11606	-1826.06090	-1801.46097	-918.64554	1
C	74.85752	71.90239	85.17086	-541.23400	531.92970	-218.41428	1
H	74.38615	72.00630	86.18674	1311.73218	-2219.18200	882.19023	1
H	74.92489	70.80068	84.94011	-1048.71298	1223.58118	-2196.97971	1
H	75.91245	72.28419	85.25133	419.06414	1903.92898	1903.44670	1
C	74.03651	74.12742	84.43084	-423.86431	152.66566	-648.55262	1
H	73.55866	74.32160	85.43039	105.68386	-2342.50080	1387.51238	1
H	75.06991	74.57018	84.46518	-1695.63603	-1484.73621	-1531.02793	1
H	73.45403	74.70683	83.66183	-1853.57121	-487.39031	1936.59343	1
C	74.72350	70.74351	82.47283	539.71428	-510.58232	267.58633	1
O	75.68759	70.14781	82.88096	-15.93758	-278.34085	-624.49402	1
N	73.61911	70.26032	81.93270	-676.81886	12.05292	276.17960	1
H	72.83084	70.90703	81.73650	-1694.44250	-373.84189	-2100.66098	1
C	73.61296	68.88879	81.55847	412.33078	-664.70360	108.35451	1
H	74.44438	68.41741	82.13500	1765.77476	1294.44366	1621.76594	1
C	72.16068	68.34115	81.88436	-179.66635	-721.95667	-264.73915	1
H	71.89288	68.53146	82.94757	-607.34922	2587.87032	-598.10018	1
H	71.39816	68.88791	81.28533	-359.69332	-1479.13954	-2259.74260	1
C	72.13236	66.81275	81.55670	-240.01076	616.02425	431.85322	1
H	72.48036	66.63456	80.51298	-1359.17131	782.54425	2227.99181	1
H	72.83705	66.25340	82.21188	-820.36667	85.81767	-2596.78715	1
C	70.65301	66.28096	81.73270	-513.07015	236.18570	-551.87280	1
H	70.32237	66.35428	82.79420	1816.11944	-1183.11722	-1650.94321	1
H	69.93399	66.89876	81.14702	-1800.04429	-1361.53473	1526.34643	1
C	70.64339	64.81470	81.24089	726.08972	-253.19732	-179.64713	1
H	70.87656	64.68943	80.16451	1224.27271	-456.51798	-2390.90304	1
H	71.26311	64.12147	81.84379	1206.40339	-1150.93514	-2154.90344	1
N	69.18289	64.27041	81.38804	329.70796	-633.68968	-155.67358	1
H	69.03493	63.27013	81.06730	1348.31305	1938.76845	1359.00643	1
H	68.77443	64.23790	82.36714	2096.92035	1446.54551	-966.48890	1
H	68.42163	64.77625	80.84830	-1197.07217	-2303.53719	827.28733	1
C	73.79740	68.70692	79.93531	-378.01186	-556.17301	-413.96481	1
O	74.25452	67.66702	79.51469	-409.44433	-307.55053	-453.30833	1
N	73.25746	69.70217	79.23822	-429.02477	524.70884	274.08502	1
H	72.96085	70.52729	79.75455	1706.34263	-1425.96036	1574.38926	1
C	73.40088	69.63584	77.82417	-45.25864	-626.22952	478.93267	1
H	73.64078	68.56558	77.61685	1988.63164	570.90773	1772.87287	1
C	72.06940	70.15795	77.14630	551.77128	-20.49526	-564.54996	1
H	71.18017	69.62727	77.55396	1434.61535	-1753.10253	1513.99471	1
H	71.90562	71.23467	77.36647	-714.67738	1819.09770	1898.36494	1
C	72.21452	69.92013	75.60619	446.99263	-555.77305	338.97221	1
H	73.10445	70.46051	75.21409	1953.68466	1892.25957	-161.67766	1
H	72.39099	68.84343	75.38673	-403.03785	2688.07032	-188.43591	1
C	70.90130	70.42755	74.88598	567.86262	335.74681	434.04421	1
H	70.00924	69.85829	75.23496	-1080.02275	1568.26081	-1948.78900	1
H	70.69789	71.49411	75.13602	-919.54108	2111.34692	1456.13463	1
C	71.10988	70.25093	73.36358	195.81897	173.33862	-745.11536	1
H	71.92274	70.87020	72.93422	1405.67949	-2301.21089	-390.08113	1
H	71.22170	69.19958	73.03098	-940.37328	-1118.32446	2299.72133	1
N	69.81173	70.75252	72.64596	319.26422	656.36007	-42.02424	1
H	69.82461	70.68917	71.58716	1961.03633	619.91968	1787.09422	1
H	68.90698	70.24565	72.87240	854.14343	-2156.80752	1429.08711	1
H	69.53785	71.76779	72.79077	1237.02905	-1378.50857	-1998.28500	1
C	74.60806	70.67512	77.38241	532.53319	-268.57236	517.55729	1
O	74.61118	71.85286	77.63927	266.16714	-290.73804	-558.88002	1
N	75.40201	69.97654	76.57584	-226.25352	640.49678	-270.32839	1

H	75.20707	68.98094	76.47407	2007.69063	-1035.27229	1523.50231	1
C	76.71331	70.46692	76.31350	-540.76542	-573.33303	49.50000	1
H	76.64663	71.52988	76.64377	-2374.20950	-1036.76023	843.75774	1
C	77.69392	69.60300	77.23538	-77.09082	666.87422	-415.84136	1
H	77.66719	68.54282	76.86518	-985.81834	617.19825	2463.91927	1
O	77.33942	69.56087	78.63211	293.29899	-391.65602	-477.80926	1
H	77.00475	68.64825	78.72006	2528.72714	-681.76262	-751.27122	1
C	79.11127	70.21942	76.95520	252.47363	-605.14473	440.05134	1
H	79.91412	69.65234	77.48181	-2052.65063	1456.49051	-1043.51830	1
H	79.35878	70.21299	75.85916	-2152.98800	-1661.97574	-161.70652	1
H	79.17131	71.27580	77.30690	-473.12726	-1584.27339	2165.61725	1
C	76.99311	70.28872	74.71713	360.36307	488.46713	505.10056	1
O	78.13855	70.06045	74.27717	-200.08817	-528.03552	-385.82850	1
N	75.89421	70.48239	74.02763	-597.54095	-226.44775	355.20479	1
H	75.13785	70.64648	74.69045	2179.23655	-1300.70981	-991.33835	1
C	75.80210	70.77449	72.64943	-687.68921	297.39807	-249.45179	1
H	74.73305	71.04164	72.50345	-349.16256	-2467.37713	-1101.72831	1
C	76.79925	71.99473	72.36384	672.47345	361.54328	-201.62880	1
H	77.76403	71.83726	72.90060	1541.41502	582.18425	2169.97026	1
H	77.02658	72.04992	71.27469	-2534.67717	60.25153	997.72295	1
C	76.11527	73.28761	72.83283	574.26774	-389.43119	-377.02542	1
C	75.28022	73.98617	71.91239	688.82679	-287.33580	-257.96133	1
H	75.12122	73.59976	70.88953	331.14182	1470.38163	2269.80013	1
C	76.28423	73.79542	74.15539	-383.52382	-675.04808	144.24917	1
H	76.91391	73.25455	74.88462	4.09847	1517.59662	2262.86397	1
C	74.67547	75.16934	72.32580	-134.76730	428.45285	-649.50303	1
H	74.04058	75.70802	71.60114	-903.23433	1600.49020	2011.53387	1
C	75.64477	74.98624	74.51345	-335.53281	-615.00040	-364.39058	1
H	75.79074	75.36613	75.54058	1596.45398	-1369.33862	1732.02659	1
C	74.82463	75.72981	73.62163	585.48547	-340.24194	-406.23938	1
O	74.18315	76.92417	73.95055	-419.15649	-173.04408	-511.94141	1
H	74.27982	76.93480	74.99111	-1071.84350	1559.71507	-1960.13012	1
C	76.23747	69.48318	71.74453	291.41878	-183.13091	710.72246	1
O	75.42382	68.96278	71.02133	379.87695	523.47396	-222.23622	1
N	77.52844	69.19320	71.88205	564.30591	373.09380	277.24371	1
H	78.10874	69.72484	72.56793	-120.59931	-2436.07824	-1214.35098	1
C	77.98904	67.99651	71.21856	404.07514	-299.37480	608.84009	1
H	77.18213	67.25909	71.43421	-1071.07882	-841.69025	2359.66467	1
C	77.98568	68.34842	69.66624	-683.12807	68.71770	-390.13096	1
H	77.02251	68.83996	69.39197	2068.88918	1596.58590	770.89603	1
H	78.78011	69.09466	69.44235	1379.86429	2342.49779	-179.85638	1
C	78.20190	67.05186	68.83041	-176.39967	537.49466	550.97268	1
H	79.08081	66.47511	69.21748	-396.28051	1942.22168	1869.33314	1
H	77.32811	66.36729	68.91906	-94.44700	586.00678	-2659.20016	1
C	78.44074	67.49204	67.31235	-291.69381	-718.18079	-150.72929	1
H	77.56694	67.95996	66.79178	-2303.38776	1089.76242	964.62032	1
H	79.33380	68.14571	67.14037	884.16729	2568.80533	-207.74283	1
N	78.70305	66.25437	66.70527	-594.49522	-252.81478	342.29276	1
H	78.77694	65.45560	67.31629	-1735.17426	2084.05705	-263.71605	1
C	78.86480	66.07576	65.45717	595.23177	-271.52487	442.22352	1
N	78.76935	67.19020	64.61686	585.53813	-424.79750	-105.81303	1
H	78.59098	68.16538	64.85325	959.88797	1327.56823	-2177.11905	1
H	78.88578	67.16953	63.60316	1578.48647	16.27378	-2220.76233	1
N	79.11989	64.72678	65.17151	-452.17763	-390.33541	-421.51853	1
H	79.19851	63.91833	65.78661	470.26107	1974.25629	-1817.92087	1
H	79.28612	64.37660	64.22737	1856.67307	-1573.97321	-1224.35416	1
C	79.44400	67.54432	71.72255	-531.47271	-581.45780	-55.06206	1
O	80.05796	66.76394	71.03914	-243.00837	192.73507	609.52485	1
N	79.82296	68.01916	72.92718	343.49288	-644.25607	38.09164	1
H	79.19256	68.66892	73.42380	-1691.16043	-1535.97525	-1484.73099	1
C	81.28773	68.16378	73.13884	542.66492	350.14337	454.42563	1
H	81.64911	68.63214	72.19500	1209.57979	-2396.95920	463.86744	1

```
C    81.53103  69.01330  74.36250      -737.14309      145.93010      242.71942          1
H    80.88163  68.62775  75.18050     -1050.18303      159.36419    -2509.06125          1
C    82.97411  68.92986  74.82971      -706.77696     -252.88343     -245.16230          1
H    83.13979  69.50578  75.78148     -1624.64039    -1665.49085     1417.86935          1
H    83.28864  67.86448  75.01931      2566.10521      598.51109      693.22305          1
H    83.68008  69.34524  74.05876     -1972.69758      107.10018    -1876.34312          1
C    81.17083  70.46865  74.12314       412.85148      672.58832      -27.69222          1
H    81.36896  71.10068  75.03243      1447.02277     1762.76533    -1490.78944          1
H    81.76452  70.90884  73.27515      1320.77379     -145.20455     2378.68491          1
H    80.07437  70.58426  73.86417     -1512.10848     1470.52031     1724.75147          1
C    81.96156  66.67511  73.35077      -607.11113      499.56689       73.73769          1
O    83.15544  66.53265  73.16622       357.97126      460.81534      356.68785          1
N    81.09543  65.79047  73.81163       536.55407     -368.62270      332.76306          1
H    80.14150  66.13086  73.91701       524.38866     2297.69985     1367.20941          1
C    81.52193  64.45767  74.04555        46.50277     -774.29190     -147.97904          1
H    82.50673  64.39874  73.52612      1676.51875     1883.29066     1032.55549          1
C    81.64703  64.17732  75.63373      -559.41456      -34.19198     -556.30433          1
H    80.64324  64.28601  76.10788       -64.66664      395.18769     2695.05373          1
H    81.94399  63.11418  75.79651     -2703.44278      -60.40056      333.79418          1
C    82.63263  65.10717  76.28398      -519.76435      377.31045      459.42366          1
H    82.26384  66.15085  76.16099     -1746.74323     1667.50644    -1261.73691          1
C    82.74745  64.80040  77.77321       570.95554       16.46261     -545.27700          1
H    83.45200  65.50356  78.28728      2391.58273       28.05277    -1305.07271          1
H    81.75822  64.88194  78.29216       175.75588      917.63458     2559.43867          1
H    83.12997  63.76250  77.95068     -1063.97851    -2155.34650     1283.00462          1
C    84.01031  65.00627  75.64566       193.76014        2.62946     -765.53141          1
H    84.78053  65.57318  76.22977       507.39394     -846.55087     2539.60089          1
H    84.36127  63.94422  75.57767      1337.57841     1818.19054     1526.01994          1
H    84.00426  65.43673  74.59530      1100.88635    -1172.91350    -2199.08909          1
C    80.38920  63.48592  73.45256      -549.21115      475.64486     -309.38218          1
O    80.11667  62.44103  73.98531       556.06106     -390.37755      -78.24250          1
O    79.79403  63.84460  72.22163      -572.27940     -310.19032     -209.75862          1
H    80.19481  64.75251  72.10358     -1380.08193     2212.14486      790.85944          1
```

Listing 18: C00018.xmolout

```
      276
  150.0000  150.0000  150.0000    0.0000    0.0000    0.0000***********    2.7750    11100
      0.2500    300.00    0.00    0.00    0.00   -1294.0746      0.0000    300.0000
H    82.46862  61.03615  72.92558     -2043.14101    -1757.64893      399.89339          1
N    82.53466  61.20196  71.92144       426.75047      490.45733     -334.42467          1
H    81.60387  61.56208  71.71056       442.25559    -1110.30854    -2448.52907          1
C    83.59720  62.05695  71.54210       415.32972     -666.39213       83.73172          1
H    84.39788  61.81344  72.27293       879.32219     2491.00447      667.35213          1
C    84.01145  61.70600  70.02462        31.35357      477.41777      628.23362          1
H    83.12890  61.82920  69.35274      1168.38489     2240.25337    -1019.71136          1
H    84.77573  62.43659  69.66731      -360.43770     2534.90498      931.67112          1
C    84.52834  60.29687  69.89541       463.18208      512.70242     -382.34388          1
H    83.75696  59.60359  70.30061      1725.29117     1863.52247     -987.07854          1
C    84.75829  59.96828  68.42444       710.42801     -144.27427     -313.15367          1
H    85.11436  58.91530  68.28337       364.20195    -1033.13725     2494.72524          1
H    83.82144  60.08639  67.82123      2567.61904      335.80679      847.48802          1
H    85.53009  60.64166  67.96972     -2587.99029      680.92867     -512.16411          1
C    85.82379  60.07982  70.66428       268.17616      741.64379      -40.42634          1
H    86.23775  59.05227  70.49130      1283.54314    -1945.37420    -1411.27812          1
H    86.61487  60.81108  70.35543     -1258.57796     1317.15249    -2026.02085          1
H    85.67602  60.19366  71.76904     -2502.84526      909.80597      575.92391          1
C    83.05603  63.59109  71.72360       619.36578     -458.64843     -172.09273          1
O    83.21594  64.26122  72.91384       -81.40433      433.87611      522.34594          1
N    82.45865  64.05993  70.74600       175.23996     -622.20190      341.55523          1
H    82.42773  63.40092  69.97569      -876.57256     -303.22879    -2561.90213          1
C    83.87059  66.28440  71.39087      -571.88214     -534.10783     -106.15033          1
```

26	H	84.96888	66.19500	71.54866	-2713.20841	-16.25800	-248.80809	1
27	C	80.90006	67.17024	69.91478	166.60354	288.16399	-716.09568	1
28	H	81.25383	65.31339	72.01758	-2097.85574	1636.78449	-586.18044	1
29	O	83.37747	66.37513	67.78090	-317.80580	-363.38227	484.43082	2
30	H	82.71245	66.21938	68.43937	1863.74748	-1451.84923	1357.29467	2
31	C	80.79417	68.31137	70.70769	503.80731	517.99032	318.51606	1
32	H	80.78062	69.18311	68.33914	640.61020	-2157.10410	-1536.29104	3
33	H	79.64966	69.02912	72.43645	1687.88455	465.60692	2087.56452	1
34	H	84.15078	68.04809	70.04585	-1293.07895	-1899.88581	-1463.57452	1
35	C	83.09196	65.92318	72.66878	-153.07550	-733.68902	248.70981	1
36	O	81.27348	66.17139	72.52321	186.15459	-480.78787	-449.34387	1
37	N	83.53398	66.58592	73.65935	-487.53139	543.18517	42.03850	1
38	C	82.72054	66.91384	74.81875	-699.35871	69.34393	360.10701	1
39	H	81.76899	66.34315	74.74130	2.81126	-221.99807	-2715.58072	1
40	C	83.67974	66.45600	75.98549	-507.46439	-592.59194	122.07761	1
41	H	83.39888	66.89064	76.97170	-1975.08116	525.87560	-1801.71556	1
42	H	83.66387	65.34964	76.10742	1588.27743	-1913.22100	1113.83566	1
43	C	85.07611	66.94489	75.47008	-613.62442	-457.55993	-194.14490	1
44	H	85.20225	68.04031	75.63327	1707.54637	-721.27992	1996.92523	1
45	H	85.93420	66.44899	75.97919	758.23357	2100.81767	1560.55011	1
46	C	84.99154	66.60633	73.93366	-352.41524	704.96076	49.21465	1
47	H	85.45207	67.36443	73.26053	1714.76886	-2070.42778	443.35792	1
48	H	85.34751	65.58652	73.65935	1224.15040	1801.50881	1636.97633	1
49	C	82.53148	68.52607	74.88723	566.85117	537.05831	117.62814	1
50	O	83.50779	69.26524	74.74265	-550.26779	-406.08867	4.21666	1
51	N	81.28033	68.83646	75.14169	566.34832	-425.77725	180.18749	1
52	H	80.66565	68.02467	75.20264	644.44502	2646.80550	-52.73430	1
53	C	80.82623	70.06421	75.68491	612.34801	-281.48662	411.56221	1
54	H	79.78648	69.83127	76.00296	1667.23498	-1490.75142	1556.16753	1
55	C	81.77398	70.46315	76.91048	-714.45746	-175.43074	286.98927	1
56	H	82.84026	70.48905	76.54761	-787.09484	1763.03619	-1922.46060	1
57	O	81.76229	69.56717	78.03569	-429.46514	516.51932	-128.40526	1
58	H	82.70405	69.30578	78.07884	1981.82785	436.16847	-1818.18182	1
59	C	81.27954	71.93138	77.18278	630.79189	-468.94448	76.03230	1
60	H	81.92337	72.45305	77.92844	-1775.71861	2062.30353	131.89849	1
61	H	81.28262	72.54355	76.24453	-1855.14608	88.94118	1993.53760	1
62	H	80.23740	71.94356	77.57882	-1504.82350	-780.94249	-2132.90992	1
63	C	80.85879	71.30715	74.61074	288.57638	618.62814	-397.00270	1
64	O	79.89437	72.02408	74.50553	321.70495	-185.26452	574.37218	1
65	N	82.06603	71.43848	74.07146	-447.05792	-446.53431	367.76425	1
66	H	82.81651	70.73307	74.30617	350.31316	1975.63644	-1843.31522	1
67	C	82.17027	72.20916	72.86732	-511.23061	374.03917	-471.51480	1
68	H	81.84437	73.23629	73.14123	2234.79579	-898.70890	-1273.45190	1
69	C	83.70761	72.07966	72.47696	529.53781	-159.34528	563.72604	1
70	H	83.91193	72.77147	71.61721	654.59388	2347.94251	1217.51488	1
71	H	84.29679	72.46398	73.35098	-1526.55661	2103.42026	817.87394	1
72	O	84.17200	70.74930	72.10291	-554.84278	14.12789	399.58769	1
73	H	84.13421	70.31348	72.97684	1297.06236	2061.84854	1220.68812	1
74	C	81.20951	71.59168	71.70670	-662.15484	-412.13544	123.62687	1
75	O	80.87131	72.30398	70.75424	-540.01537	-317.05804	274.91456	1
76	N	81.02929	70.30739	71.90202	-202.76299	-690.00100	-131.49145	1
77	H	81.52746	70.04048	72.75490	-702.62132	-2617.37191	281.70413	1
78	C	80.00754	69.39202	71.44201	-491.73914	576.34187	222.73509	1
79	H	81.19198	69.66624	68.75394	1977.17978	-1823.58850	-434.68928	3
80	C	82.05063	67.73178	70.49562	-649.50797	-328.66843	306.11224	1
81	H	80.31448	66.43047	72.43414	2075.07803	1452.16490	-1004.45904	1
82	C	80.19826	66.07580	69.15706	-615.18735	-489.99689	-70.96352	1
83	H	80.41701	65.07075	69.59983	-1226.32661	-1724.38906	-1716.47170	1
84	H	79.08806	66.21850	69.16457	2381.64273	-969.84735	900.46860	1
85	H	80.52865	66.03103	68.08892	2122.74991	1702.23946	-141.36228	1
86	C	83.49644	67.75361	70.89918	-501.86074	-479.61276	-376.42482	1
87	H	83.60026	65.55345	70.59089	993.79261	-2246.95401	-1177.81305	1

H	83.70686	67.22557	68.04038	42.71279	-626.15391	-2651.37258	2
H	83.67244	68.51251	71.70247	-2026.39012	-1590.33709	-887.82922	1
C	78.71856	69.76835	70.47107	-672.89153	382.57394	-156.34072	1
O	77.84486	68.93495	70.45390	410.04627	221.86223	500.35950	1
N	78.87838	70.83842	69.72688	-378.11782	-443.79414	-441.10984	1
H	79.74165	71.43460	69.85768	2404.15973	-568.14894	1149.30085	1
C	77.70142	71.48360	69.21807	-544.54484	466.16019	331.29119	1
H	78.12209	72.24015	68.51971	-1409.80988	-170.17955	2325.32679	1
C	76.75850	70.41382	68.52890	-184.48284	116.78282	-758.89137	1
H	77.31654	69.84925	67.74643	1893.78641	1861.14652	611.04528	1
H	76.40856	69.64830	69.27736	-2170.79665	-974.19311	1327.50117	1
C	75.52219	71.15055	67.92297	389.51668	672.81878	-138.49152	1
H	75.01849	71.75448	68.71304	510.64577	1933.03327	1851.02484	1
H	75.84102	71.89923	67.16501	-2302.30869	-1105.72939	-948.89774	1
C	74.48297	70.14937	67.29089	-559.93427	-126.34590	542.30857	1
O	73.45899	70.57687	66.82348	-670.36003	-111.83166	76.36448	1
N	74.86377	68.85378	67.33297	-216.06729	-537.07037	446.51379	1
H	75.70319	68.42612	67.74133	-1251.96487	1074.12894	-2168.52500	1
H	74.28468	68.07685	66.99664	-2451.83023	-720.73851	944.84612	1
C	76.92095	72.15214	70.48080	546.24052	-563.88337	-85.11809	1
O	75.85327	71.70231	70.83924	71.70702	-307.40463	606.69673	1
N	77.55145	73.24874	70.91322	479.90863	-504.50129	222.86849	1
C	77.32864	73.79064	72.22202	75.51268	-600.91904	506.73700	1
H	77.38365	72.93871	72.93787	-2033.61562	1795.30103	254.89480	1
C	78.50572	74.84430	72.34532	523.79067	517.99274	284.45581	1
H	78.31535	75.61854	73.12299	1599.61665	-1127.00911	1895.98173	1
H	79.46844	74.34937	72.60739	-1881.83093	1275.82488	-1501.55016	1
C	78.53776	75.41203	70.89193	-600.05639	-404.05026	316.64485	1
H	77.66933	76.08405	70.70140	-2597.05260	496.36221	657.73249	1
H	79.45606	75.99512	70.65508	-1666.24518	1294.38985	-1723.90591	1
C	78.42396	74.05717	70.07004	493.57570	-129.95376	-602.56394	1
H	77.96929	74.14752	69.05511	-1469.08415	-637.84797	2204.22581	1
H	79.38749	73.47799	70.01406	-1038.97840	1068.88701	-2280.71789	1
C	75.87906	74.55592	72.29069	188.88100	241.05089	-727.87821	1
O	75.58443	75.16255	73.29333	531.88613	-49.19943	427.07960	1
N	75.20030	74.40761	71.16216	708.14833	26.93417	-179.73509	1
H	75.63610	73.81651	70.44988	-2232.69501	-1179.09831	1023.94816	1
C	73.86298	74.86361	71.00748	-738.32412	259.92548	104.42456	1
H	73.56307	74.43279	70.02584	2028.89123	1260.02983	1311.33324	1
C	73.04932	74.21129	72.20993	-258.65678	-345.52513	661.28459	1
H	73.54324	74.45285	73.18012	-1826.06090	-1801.46097	-918.64554	1
C	71.52327	74.55828	72.27712	-541.23400	531.92970	-218.41428	1
H	70.95440	73.76902	72.82354	1311.73218	-2219.18200	882.19023	1
H	71.33052	75.51602	72.82275	-1048.71298	1223.58118	-2196.97971	1
H	71.07276	74.64281	71.25945	419.06414	1903.92898	1903.44670	1
C	73.12532	72.67314	71.91698	-423.86431	152.66566	-648.55262	1
H	72.77393	72.06908	72.78635	105.68386	-2342.50080	1387.51238	1
H	72.49121	72.39448	71.04228	-1695.63603	-1484.73621	-1531.02793	1
H	74.18564	72.34324	71.67318	-1853.57121	-487.39031	1936.59343	1
C	74.00962	76.48600	70.90398	539.71428	-510.58232	267.58633	1
O	74.96419	77.01396	70.37541	-15.93758	-278.34085	-624.49402	1
N	72.89314	77.06844	71.33224	-676.81886	12.05292	276.17960	1
H	72.13447	76.49975	71.70691	-1694.44250	-373.84189	-2100.66098	1
C	72.87468	78.48803	71.40544	412.33078	-664.70360	108.35451	1
H	73.87385	78.79780	71.01192	1765.77476	1294.44366	1621.76594	1
C	71.66471	78.98334	70.50570	-179.66635	-721.95667	-264.73915	1
H	71.67873	78.48031	69.51310	-607.34922	2587.87032	-598.10018	1
H	70.69269	78.70558	70.97076	-359.69332	-1479.13954	-2259.74260	1
C	71.78821	80.53382	70.35180	-240.01076	616.02425	431.85322	1
H	71.82882	81.01619	71.36278	-1359.17131	782.54425	2227.99181	1
H	72.73566	80.81127	69.83641	-820.36667	85.81767	-2596.78715	1
C	70.53227	81.04787	69.53897	-513.07015	236.18570	-551.87280	1

```
H   70.54281  80.65604  68.49591    1816.11944   -1183.11722   -1650.94321    1
H   69.58617  80.67514  69.99437   -1800.04429   -1361.53473    1526.34643    1
C   70.57276  82.59351  69.55572     726.08972    -253.19732    -179.64713    1
H   70.45525  83.04541  70.56097    1224.27271    -456.51798   -2390.90304    1
H   71.44683  83.03948  69.04065    1206.40339   -1150.93514   -2154.90344    1
N   69.33159  83.10238  68.74813     329.70796    -633.68968    -155.67358    1
H   69.21556  84.15565  68.70063    1348.31305    1938.76845    1359.00643    1
H   69.28357  82.84049  67.72064    2096.92035    1446.54551    -966.48890    1
H   68.37450  82.80290  69.09596   -1197.07217   -2303.53719     827.28733    1
C   72.60041  78.92956  72.96435    -378.01186    -556.17301    -413.96481    1
O   72.16127  80.04413  73.17417    -409.44433    -307.55053    -453.30833    1
N   72.98286  77.99926  73.81371    -429.02477     524.70884     274.08502    1
H   73.38931  77.16891  73.38586    1706.34263   -1425.96036    1574.38926    1
C   72.87752  78.10056  75.23374     -45.25864    -626.22952     478.93267    1
H   73.82448  77.66032  75.61881    1988.63164     570.90773    1772.87287    1
C   72.66358  79.62141  75.60540     551.77128     -20.49526    -564.54996    1
H   73.42438  80.23567  75.06910    1434.61535   -1753.10253    1513.99471    1
H   71.67251  79.96593  75.21137    -714.67738    1819.09770    1898.36494    1
C   72.71840  79.90937  77.14276     446.99263    -555.77305     338.97221    1
H   71.85241  79.44612  77.66981    1953.68466    1892.25957    -161.67766    1
H   73.63762  79.49535  77.61392    -403.03785    2688.07032    -188.43591    1
C   72.67365  81.48812  77.23295     567.86262     335.74681     434.04421    1
H   73.57935  81.93932  76.76664   -1080.02275    1568.26081   -1948.78900    1
H   71.80316  81.88383  76.65980    -919.54108    2111.34692    1456.13463    1
C   72.54871  81.89750  78.71987     195.81897     173.33862    -745.11536    1
H   71.57953  81.60838  79.18624    1405.67949   -2301.21089    -390.08113    1
H   73.39657  81.59491  79.36861    -940.37328   -1118.32446    2299.72133    1
N   72.47240  83.44596  78.82035     319.26422     656.36007     -42.02424    1
H   72.07639  83.76125  79.80711    1961.03633     619.91968    1787.09422    1
H   73.35918  84.01307  78.68262     854.14343   -2156.80752    1429.08711    1
H   71.81452  83.90656  78.12780    1237.02905   -1378.50857   -1998.28500    1
C   71.57606  77.25429  75.74585     532.53319    -268.57236     517.55729    1
O   70.59257  77.02306  75.06033     266.16714    -290.73804    -558.88002    1
N   71.76521  76.98681  77.02653    -226.25352     640.49678    -270.32839    1
H   72.64428  77.29276  77.43600    2007.69063   -1035.27229    1523.50231    1
C   70.76014  76.33763  77.80237    -540.76542    -573.33303      49.50000    1
H   70.23559  75.71942  77.03772   -2374.20950   -1036.76023     843.75774    1
C   71.48973  75.51793  78.96211     -77.09082     666.87422    -415.84136    1
H   71.90589  76.24921  79.70755    -985.81834     617.19825    2463.91927    1
O   72.59423  74.69360  78.54400     293.29899    -391.65602    -477.80926    1
H   73.32775  75.08168  79.06060    2528.72714    -681.76262    -751.27122    1
C   70.28977  74.74054  79.61784     252.47363    -605.14473     440.05134    1
H   70.62243  74.16289  80.51149   -2052.65063    1456.49051   -1043.51830    1
H   69.47738  75.43138  79.95247   -2152.98800   -1661.97574    -161.70652    1
H   69.83838  74.01221  78.90497    -473.12726   -1584.27339    2165.61725    1
C   69.79963  77.50965  78.40876     360.36307     488.46713     505.10056    1
O   69.51457  77.58929  79.59239    -200.08817    -528.03552    -385.82850    1
N   69.40255  78.24449  77.39266    -597.54095    -226.44775     355.20479    1
H   69.70316  77.96640  76.44130    2179.23655   -1300.70981    -991.33835    1
C   68.51363  79.33108  77.55981    -687.68921     297.39807    -249.45179    1
H   68.36361  79.68963  76.51809    -349.16256   -2467.37713   -1101.72831    1
C   67.16857  78.78297  78.23493     672.47345     361.54328    -201.62880    1
H   67.38790  78.34447  79.23583    1541.41502     582.18425    2169.97026    1
H   66.45959  79.62768  78.39261   -2534.67717      60.25153     997.72295    1
C   66.54791  77.70921  77.33090     574.26774    -389.43119    -377.02542    1
C   65.77989  78.07818  76.18594     688.82679    -287.33580    -257.96133    1
H   65.62924  79.14130  75.92497     331.14182    1470.38163    2269.80013    1
C   66.75601  76.33260  77.64882    -383.52382    -675.04808     144.24917    1
H   67.36821  76.04602  78.52326       4.09847    1517.59662    2262.86397    1
C   65.22157  77.06616  75.40522    -134.76730     428.45285    -649.50303    1
H   64.62362  77.34977  74.52168    -903.23433    1600.49020    2011.53387    1
C   66.17352  75.35632  76.83760    -335.53281    -615.00040    -364.39058    1
```

```
H   66.33496  74.29561  77.09808    1596.45398   -1369.33862    1732.02659        1
C   65.38504  75.68031  75.69470     585.48547    -340.24194    -406.23938        1
O   64.78430  74.69655  74.86667    -419.15649    -173.04408    -511.94141        1
H   65.08012  73.88092  75.33575   -1071.84350    1559.71507   -1960.13012        1
C   69.20124  80.47398  78.51612     291.41878    -183.13091     710.72246        1
O   69.64670  81.45324  77.97983     379.87695     523.47396    -222.23622        1
N   69.08196  80.17370  79.80630     564.30591     373.09380     277.24371        1
H   68.83210  79.18969  80.04801    -120.59931   -2436.07824   -1214.35098        1
C   69.69954  80.95583  80.84282     404.07514    -299.37480     608.84009        1
H   68.99288  80.76258  81.68116   -1071.07882    -841.69025    2359.66467        1
C   71.08838  80.25135  81.14865    -683.12807      68.71770    -390.13096        1
H   70.94373  79.15809  81.30304    2068.88918    1596.58590     770.89603        1
H   71.77670  80.32947  80.27798    1379.86429    2342.49779    -179.85638        1
C   71.71152  80.91865  82.41346    -176.39967     537.49466     550.97268        1
H   71.76914  82.02861  82.28578    -396.28051    1942.22168    1869.33314        1
H   71.07167  80.75170  83.30910     -94.44700     586.00678   -2659.20016        1
C   73.17221  80.30865  82.62438    -291.69381    -718.18079    -150.72929        1
H   73.21658  79.21115  82.84116   -2303.38776    1089.76242     964.62032        1
H   73.90822  80.52817  81.80929     884.16729    2568.80533    -207.74283        1
N   73.58903  80.99581  83.77560    -594.49522    -252.81478     342.29276        1
H   72.95064  81.67684  84.15707   -1735.17426    2084.05705    -263.71605        1
C   74.70384  80.82110  84.36058     595.23177    -271.52487     442.22352        1
N   75.58261  79.87580  83.82002     585.53813    -424.79750    -105.81303        1
H   75.47022  79.28444  82.99767     959.88797    1327.56823   -2177.11905        1
H   76.50285  79.65090  84.19949    1578.48647      16.27378   -2220.76233        1
N   74.82355  81.65504  85.48168    -452.17763    -390.33541    -421.51853        1
H   74.18167  82.34095  85.87630     470.26107    1974.25629   -1817.92087        1
H   75.64546  81.66145  86.08685    1856.67307   -1573.97321   -1224.35416        1
C   69.81840  82.52333  80.49854    -531.47271    -581.45780     -55.06206        1
O   70.87528  83.13028  80.73743    -243.00837     192.73507     609.52485        1
N   68.67958  83.04456  80.09610     343.49288    -644.25607      38.09164        1
H   67.95127  82.34527  79.94812   -1691.16043   -1535.97525   -1484.73099        1
C   68.63590  84.37694  79.49355     542.66492     350.14337     454.42563        1
H   69.60819  84.46609  78.95383    1209.57979   -2396.95920     463.86744        1
C   67.40740  84.48706  78.60637    -737.14309     145.93010     242.71942        1
H   66.52467  84.16677  79.20343   -1050.18303     159.36419   -2509.06125        1
C   67.17484  85.91808  78.15701    -706.77696    -252.88343    -245.16230        1
H   66.22502  86.02124  77.56309   -1624.64039   -1665.49085    1417.86935        1
H   67.10189  86.62066  79.03279    2566.10521     598.51109     693.22305        1
H   68.01651  86.28987  77.51015   -1972.69758     107.10018   -1876.34312        1
C   67.50300  83.57663  77.39618     412.85148     672.58832     -27.69222        1
H   66.58430  83.64008  76.74987    1447.02277    1762.76533   -1490.78944        1
H   68.38540  83.84338  76.75100    1320.77379    -145.20455    2378.68491        1
H   67.63403  82.49902  77.69681   -1512.10848    1470.52031    1724.75147        1
C   68.52439  85.49432  80.72063    -607.11113     499.56689      73.73769        1
O   69.09097  86.55947  80.61036     357.97126     460.81534     356.68785        1
N   67.67325  85.10499  81.65450     536.55407    -368.62270     332.76306        1
H   67.30995  84.15658  81.57198     524.38866    2297.69985    1367.20941        1
C   67.59921  85.85468  82.85594      46.50277    -774.29190    -147.97904        1
H   68.47261  86.54490  82.81112    1676.51875    1883.29066    1032.55549        1
C   66.19112  86.64580  82.92520    -559.41456     -34.19198    -556.30433        1
H   65.35641  85.90643  82.96081     -64.66664     395.18769    2695.05373        1
H   66.12536  87.22379  83.87753   -2703.44278     -60.40056     333.79418        1
C   66.02518  87.55646  81.73995    -519.76435     377.31045     459.42366        1
H   66.07971  86.93602  80.81718   -1746.74323    1667.50644   -1261.73691        1
C   64.66671  88.24687  81.77845     570.95554      16.46261    -545.27700        1
H   64.51833  88.91581  80.89193    2391.58273      28.05277   -1305.07271        1
H   63.82540  87.50731  81.78076     175.75588     917.63458    2559.43867        1
H   64.55271  88.88207  82.69402   -1063.97851   -2155.34650    1283.00462        1
C   67.12387  88.60745  81.67736     193.76014       2.62946    -765.53141        1
H   66.91227  89.38148  80.89510     507.39394    -846.55087    2539.60089        1
H   67.24296  89.14585  82.65292    1337.57841    1818.19054    1526.01994        1
```

```
H    68.12074   88.13630   81.41649      1100.88635     -1172.91350     -2199.08909          1
C    67.61087   84.78877   84.06491      -549.21115       475.64486      -309.38218          1
O    67.31776   83.62981   83.92705       556.06106      -390.37755       -78.24250          1
O    67.87827   85.31842   85.35012      -572.27940      -310.19032      -209.75862          1
H    68.05385   86.26561   85.09322     -1380.08193      2212.14486       790.85944          1
```

Listing 19: C00019.xmolout

```
       276
  150.0000  150.0000  150.0000    0.0000    0.0000    0.0000***********    1.5875     6350
       0.2500    300.00   0.00   0.00   0.00  -1294.0657      0.0000    300.0000
H    69.78210   73.61447   67.11124     -2043.14101     -1757.64893       399.89339          1
N    70.77008   73.85433   67.04981       426.75047       490.45733      -334.42467          1
H    71.01159   73.51292   66.11992       442.25559     -1110.30854     -2448.52907          1
C    71.06397   75.22668   67.22701       415.32972      -666.39213        83.73172          1
H    70.35683   75.63511   67.98304       879.32219      2491.00447       667.35213          1
C    72.59742   75.32217   67.63800        31.35357       477.41777       628.23362          1
H    73.20713   74.66050   66.98365      1168.38489      2240.25337     -1019.71136          1
H    72.98960   76.35342   67.48759      -360.43770      2534.90498       931.67112          1
C    72.73932   74.89609   69.13711       463.18208       512.70242      -382.34388          1
H    72.20146   73.94011   69.32339      1725.29117      1863.52247      -987.07854          1
C    74.27070   74.74100   69.45953       710.42801      -144.27427      -313.15367          1
H    74.43192   74.31757   70.47704       364.20195     -1033.13725      2494.72524          1
H    74.77258   74.05430   68.73913      2567.61904       335.80679       847.48802          1
H    74.81031   75.71905   69.41639     -2587.99029       680.92867      -512.16411          1
C    72.16545   76.05420   70.03144       268.17616       741.64379       -40.42634          1
H    72.42223   75.90076   71.10310      1283.54314     -1945.37420     -1411.27812          1
H    72.57743   77.04532   69.72931     -1258.57796      1317.15249     -2026.02085          1
H    71.05503   76.12174   69.96364     -2502.84526       909.80597       575.92391          1
C    70.95202   75.92236   65.73956       619.36578      -458.64843      -172.09273          1
O    71.42768   75.41965   64.74779       -81.40433       433.87611       522.34594          1
N    70.35118   77.09725   65.88204       175.23996      -622.20190       341.55523          1
H    69.97228   77.34230   66.79500      -876.57256      -303.22879     -2561.90213          1
C    70.24591   77.94617   64.74263      -571.88214      -534.10783      -106.15033          1
H    70.29492   77.21553   63.90078     -2713.20841       -16.25800      -248.80809          1
C    68.86081   78.74375   64.84773       166.60354       288.16399      -716.09568          1
H    68.94867   79.53770   65.63775     -2097.85574      1636.78449      -586.18044          1
O    67.71053   77.95544   65.19788      -317.80580      -363.38227       484.43082          1
H    67.41531   78.40571   66.01529      1863.74748     -1451.84923      1357.29467          1
C    68.79698   79.44330   63.44083       503.80731       517.99032       318.51606          1
H    67.90140   80.10099   63.35326       640.61020     -2157.10410     -1536.29104          1
H    69.69512   80.07733   63.24691      1687.88455       465.60692      2087.56452          1
H    68.73743   78.69318   62.61815     -1293.07895     -1899.88581     -1463.57452          1
C    71.49283   79.00237   64.66745      -153.07550      -733.68902       248.70981          1
O    71.82346   79.30726   63.54888       186.15459      -480.78787      -449.34387          1
N    71.93257   79.52480   65.82386      -487.53139       543.18517        42.03850          1
C    73.04339   80.42549   65.81497      -699.35871        69.34393       360.10701          1
H    73.08274   80.90071   64.80635         2.81126      -221.99807     -2715.58072          1
C    72.65488   81.37750   67.03364      -507.46439      -592.59194       122.07761          1
H    73.52441   81.94908   67.42740     -1975.08116       525.87560     -1801.71556          1
H    71.89278   82.12798   66.72812      1588.27743     -1913.22100      1113.83566          1
C    72.06740   80.37021   68.06791      -613.62442      -457.55993      -194.14490          1
H    72.87646   79.89165   68.66799      1707.54637      -721.27992      1996.92523          1
H    71.38008   80.84732   68.80220       758.23357      2100.81767      1560.55011          1
C    71.33964   79.30856   67.13535      -352.41524       704.96076        49.21465          1
H    71.55189   78.24690   67.40107      1714.76886     -2070.42778       443.35792          1
H    70.24090   79.45306   67.02386      1224.15040      1801.50881      1636.97633          1
C    74.40541   79.61807   66.21130       566.85117       537.05831       117.62814          1
O    74.42597   78.69779   67.02304      -550.26779      -406.08867         4.21666          1
N    75.41676   80.21151   65.61895       566.34832      -425.77725       180.18749          1
H    75.21960   80.98134   64.98381       644.44502      2646.80550       -52.73430          1
C    76.68878   79.55305   65.65615       612.34801      -281.48662       411.56221          1
```

H	76.39932	78.47988	65.59110	1667.23498	-1490.75142	1556.16753	1
C	77.58012	80.09019	64.45019	-714.45746	-175.43074	286.98927	1
H	77.78389	81.18401	64.60995	-787.09484	1763.03619	-1922.46060	1
O	76.99091	79.96795	63.14255	-429.46514	516.51932	-128.40526	1
H	76.96074	80.90115	62.85455	1981.82785	436.16847	-1818.18182	1
C	78.90870	79.27598	64.67379	630.79189	-468.94448	76.03230	1
H	79.69438	79.57125	63.94063	-1775.71861	2062.30353	131.89849	1
H	79.33078	79.43825	65.69427	-1855.14608	88.94118	1993.53760	1
H	78.73904	78.18098	64.55351	-1504.82350	-780.94249	-2132.90992	1
C	77.36045	79.91671	67.10433	288.57638	618.62814	-397.00270	1
O	78.23182	80.74486	67.21467	321.70495	-185.26452	574.37218	1
N	76.81658	79.14011	68.03868	-447.05792	-446.53431	367.76425	1
H	75.98803	78.53591	67.82585	350.31316	1975.63644	-1843.31522	1
C	77.23540	79.40357	69.37374	-511.23061	374.03917	-471.51480	1
H	78.16537	80.01534	69.28531	2234.79579	-898.70890	-1273.45190	1
C	76.02588	80.25846	69.98455	529.53781	-159.34528	563.72604	1
H	76.31273	80.60902	71.00916	654.59388	2347.94251	1217.51488	1
H	75.96041	81.18508	69.35194	-1526.55661	2103.42026	817.87394	1
O	74.71276	79.63181	70.00669	-554.84278	14.12789	399.58769	1
H	74.66016	79.36491	69.06477	1297.06236	2061.84854	1220.68812	1
C	77.47168	77.94807	70.07390	-662.15484	-412.13544	123.62687	1
O	76.70255	77.02188	70.00232	-540.01537	-317.05804	274.91456	1
N	78.62644	78.02030	70.75851	-202.76299	-690.00100	-131.49145	1
H	79.11254	78.91251	70.66587	-702.62132	-2617.37191	281.70413	1
C	79.35885	76.78673	71.06816	-491.73914	576.34187	222.73509	1
H	78.85821	76.01607	70.43513	1977.17978	-1823.58850	-434.68928	1
C	80.82862	76.99170	70.76284	-649.50797	-328.66843	306.11224	1
H	81.20329	77.82336	71.40060	2075.07803	1452.16490	-1004.45904	1
C	81.63933	75.75026	71.09218	-615.18735	-489.99689	-70.96352	1
H	82.73507	75.90492	70.89329	-1226.32661	-1724.38906	-1716.47170	1
H	81.53226	75.46190	72.17419	2381.64273	-969.84735	900.46860	1
H	81.31182	74.86600	70.48020	2122.74991	1702.23946	-141.36228	1
C	81.05892	77.39023	69.31549	-501.86074	-479.61276	-376.42482	1
H	82.14978	77.55200	69.09716	993.79261	-2246.95401	-1177.81305	1
H	80.69032	76.60015	68.60608	42.71279	-626.15391	-2651.37258	1
H	80.52074	78.34250	69.05561	-2026.39012	-1590.33709	-887.82922	1
C	79.14019	76.49031	72.68345	-672.89153	382.57394	-156.34072	1
O	78.83690	75.38410	73.06247	410.04627	221.86223	500.35950	1
N	79.38511	77.57930	73.49545	-378.11782	-443.79414	-441.10984	1
H	79.53857	78.29564	72.76672	2404.15973	-568.14894	1149.30085	1
C	78.24566	77.79284	74.37547	-544.54484	466.16019	331.29119	1
H	77.88235	76.76659	74.61096	-1409.80988	-170.17955	2325.32679	1
C	78.68344	78.61913	75.63924	-184.48284	116.78282	-758.89137	1
H	79.63985	78.23896	76.06544	1893.78641	1861.14652	611.04528	1
H	78.85867	79.68803	75.37831	-2170.79665	-974.19311	1327.50117	1
C	77.49453	78.44852	76.64426	389.51668	672.81878	-138.49152	1
H	76.55225	78.84493	76.18971	510.64577	1933.03327	1851.02484	1
H	77.27611	77.36020	76.80899	-2302.30869	-1105.72939	-948.89774	1
C	77.74555	79.12837	78.04048	-559.93427	-126.34590	542.30857	1
O	77.07571	78.81798	78.99521	-670.36003	-111.83166	76.36448	1
N	78.73000	80.05249	78.02324	-216.06729	-537.07037	446.51379	1
H	79.25195	80.41243	77.21675	-1251.96487	1074.12894	-2168.52500	1
H	78.95112	80.66840	78.81322	-2451.83023	-720.73851	944.84612	1
C	77.20378	78.72570	73.53443	546.24052	-563.88337	-85.11809	1
O	77.49091	79.85756	73.24837	71.70702	-307.40463	606.69673	1
N	76.10570	78.02111	73.23071	479.90863	-504.50129	222.86849	1
C	74.91031	78.68370	72.80304	75.51268	-600.91904	506.73700	1
H	75.23793	79.58138	72.22988	-2033.61562	1795.30103	254.89480	1
C	74.22600	77.55137	71.92250	523.79067	517.99274	284.45581	1
H	73.12871	77.69572	71.81008	1599.61665	-1127.00911	1895.98173	1
H	74.66231	77.53839	70.89317	-1881.83093	1275.82488	-1501.55016	1
C	74.60147	76.25039	72.69611	-600.05639	-404.05026	316.64485	1

H	73.94898	76.11317	73.58587	-2597.05260	496.36221	657.73249	1
H	74.50813	75.32234	72.09113	-1666.24518	1294.38985	-1723.90591	1
C	76.09598	76.56752	73.12596	493.57570	-129.95376	-602.56394	1
H	76.39787	76.13522	74.11043	-1469.08415	-637.84797	2204.22581	1
H	76.87057	76.31791	72.35759	-1038.97840	1068.88701	-2280.71789	1
C	74.07922	79.10696	74.13692	188.88100	241.05089	-727.87821	1
O	74.50695	78.98862	75.26170	531.88613	-49.19943	427.07960	1
N	72.88650	79.70013	73.79572	708.14833	26.93417	-179.73509	1
H	72.86259	79.54127	72.78326	-2232.69501	-1179.09831	1023.94816	1
C	71.72030	79.34161	74.63840	-738.32412	259.92548	104.42456	1
H	72.15103	79.40868	75.66531	2028.89123	1260.02983	1311.33324	1
C	70.61558	80.32855	74.34652	-258.65678	-345.52513	661.28459	1
H	70.37071	80.25174	73.26255	-1826.06090	-1801.46097	-918.64554	1
C	69.35624	80.00971	75.13270	-541.23400	531.92970	-218.41428	1
H	68.53600	80.75080	74.92599	1311.73218	-2219.18200	882.19023	1
H	68.96129	78.98756	74.86799	-1048.71298	1223.58118	-2196.97971	1
H	69.54452	80.01864	76.24126	419.06414	1903.92898	1903.44670	1
C	71.05258	81.75827	74.61524	-423.86431	152.66566	-648.55262	1
H	70.26102	82.50091	74.32163	105.68386	-2342.50080	1387.51238	1
H	71.28012	81.92342	75.70383	-1695.63603	-1484.73621	-1531.02793	1
H	71.98582	82.01306	74.04113	-1853.57121	-487.39031	1936.59343	1
C	71.29366	77.80174	74.22615	539.71428	-510.58232	267.58633	1
O	70.27839	77.60813	73.60771	-15.93758	-278.34085	-624.49402	1
N	72.21782	76.93716	74.60300	-676.81886	12.05292	276.17960	1
H	73.05349	77.23183	75.13400	-1694.44250	-373.84189	-2100.66098	1
C	71.87944	75.53306	74.68505	412.33078	-664.70360	108.35451	1
H	71.01753	75.53686	75.38831	1765.77476	1294.44366	1621.76594	1
C	71.46995	74.97686	73.26127	-179.66635	-721.95667	-264.73915	1
H	71.11497	75.80379	72.60330	-607.34922	2587.87032	-598.10018	1
H	72.35094	74.53034	72.74760	-359.69332	-1479.13954	-2259.74260	1
C	70.33917	73.91003	73.43845	-240.01076	616.02425	431.85322	1
H	70.57170	73.20002	74.26271	-1359.17131	782.54425	2227.99181	1
H	69.37812	74.39750	73.71775	-820.36667	85.81767	-2596.78715	1
C	70.21374	73.14480	72.05947	-513.07015	236.18570	-551.87280	1
H	70.01789	73.85589	71.22399	1816.11944	-1183.11722	-1650.94321	1
H	71.16884	72.63205	71.80138	-1800.04429	-1361.53473	1526.34643	1
C	69.05962	72.12537	72.19876	726.08972	-253.19732	-179.64713	1
H	69.20763	71.36620	72.99258	1224.27271	-456.51798	-2390.90304	1
H	68.05309	72.57947	72.29333	1206.40339	-1150.93514	-2154.90344	1
N	68.99999	71.29842	70.87026	329.70796	-633.68968	-155.67358	1
H	68.23645	70.56358	70.82111	1348.31305	1938.76845	1359.00643	1
H	68.82950	71.83847	69.97259	2096.92035	1446.54551	-966.48890	1
H	69.85704	70.72416	70.62065	-1197.07217	-2303.53719	827.28733	1
C	73.24086	74.91932	75.28805	-378.01186	-556.17301	-413.96481	1
O	74.06763	75.68475	75.81453	-409.44433	-307.55053	-453.30833	1
N	73.30608	73.62044	75.12954	-429.02477	524.70884	274.08502	1
H	72.51033	73.17835	74.67036	1706.34263	-1425.96036	1574.38926	1
C	74.41767	72.84147	75.54472	-45.25864	-626.22952	478.93267	1
H	74.10420	71.81201	75.26043	1988.63164	570.90773	1772.87287	1
C	75.67251	73.35141	74.71669	551.77128	-20.49526	-564.54996	1
H	75.34503	73.91800	73.81678	1434.61535	-1753.10253	1513.99471	1
H	76.26641	74.07534	75.32162	-714.67738	1819.09770	1898.36494	1
C	76.55726	72.13165	74.29979	446.99263	-555.77305	338.97221	1
H	76.70577	71.42726	75.14790	1953.68466	1892.25957	-161.67766	1
H	76.06964	71.53918	73.49351	-403.03785	2688.07032	-188.43591	1
C	77.94468	72.72017	73.81825	567.86262	335.74681	434.04421	1
H	77.81050	73.38271	72.92967	-1080.02275	1568.26081	-1948.78900	1
H	78.39587	73.37047	74.60376	-919.54108	2111.34692	1456.13463	1
C	78.88781	71.54054	73.48772	195.81897	173.33862	-745.11536	1
H	79.14582	70.89862	74.35361	1405.67949	-2301.21089	-390.08113	1
H	78.56790	70.91293	72.63168	-940.37328	-1118.32446	2299.72133	1
N	80.25092	72.17045	73.04271	319.26422	656.36007	-42.02424	1

178	H	81.04076	71.49778	72.81672	1961.03633	619.91968	1787.09422	1
179	H	80.23460	72.78618	72.17841	854.14343	-2156.80752	1429.08711	1
180	H	80.71467	72.81240	73.74777	1237.02905	-1378.50857	-1998.28500	1
181	C	74.57718	73.02955	77.16401	532.53319	-268.57236	517.55729	1
182	O	73.62525	73.27506	77.86552	266.16714	-290.73804	-558.88002	1
183	N	75.83264	72.71747	77.53912	-226.25352	640.49678	-270.32839	1
184	H	76.40772	72.51161	76.72121	2007.69063	-1035.27229	1523.50231	1
185	C	76.48626	73.48808	78.56160	-540.76542	-573.33303	49.50000	1
186	H	77.56672	73.34693	78.33928	-2374.20950	-1036.76023	843.75774	1
187	C	76.07099	75.00062	78.29999	-77.09082	666.87422	-415.84136	1
188	H	74.94666	75.07531	78.33500	-985.81834	617.19825	2463.91927	1
189	O	76.44318	75.50127	77.00427	293.29899	-391.65602	-477.80926	1
190	H	75.50714	75.62912	76.58043	2528.72714	-681.76262	-751.27122	1
191	C	76.68050	75.78503	79.51255	252.47363	-605.14473	440.05134	1
192	H	76.43378	76.87979	79.45548	-2052.65063	1456.49051	-1043.51830	1
193	H	76.29792	75.40674	80.49003	-2152.98800	-1661.97574	-161.70652	1
194	H	77.79332	75.70739	79.53385	-473.12726	-1584.27339	2165.61725	1
195	C	76.11329	72.88138	80.01884	360.36307	488.46713	505.10056	1
196	O	75.10317	72.26179	80.29069	-200.08817	-528.03552	-385.82850	1
197	N	77.08101	73.25059	80.93094	-597.54095	-226.44775	355.20479	1
198	H	77.74294	73.72647	80.30065	2179.23655	-1300.70981	-991.33835	1
199	C	77.63430	72.10739	81.64178	-687.68921	297.39807	-249.45179	1
200	H	77.77907	71.31918	80.87254	-349.16256	-2467.37713	-1101.72831	1
201	C	78.99745	72.61145	82.28216	672.47345	361.54328	-201.62880	1
202	H	78.81695	73.50139	82.92826	1541.41502	582.18425	2169.97026	1
203	H	79.43143	71.81485	82.93012	-2534.67717	60.25153	997.72295	1
204	C	79.94884	72.95851	81.12443	574.26774	-389.43119	-377.02542	1
205	C	80.61179	71.89724	80.44042	688.82679	-287.33580	-257.96133	1
206	H	80.47002	70.84724	80.75516	331.14182	1470.38163	2269.80013	1
207	C	80.12654	74.31279	80.71146	-383.52382	-675.04808	144.24917	1
208	H	79.60747	75.13490	81.23714	4.09847	1517.59662	2262.86397	1
209	C	81.44004	72.21484	79.36382	-134.76730	428.45285	-649.50303	1
210	H	81.95866	71.39508	78.83724	-903.23433	1600.49020	2011.53387	1
211	C	80.96582	74.57928	79.62602	-335.53281	-615.00040	-364.39058	1
212	H	81.09977	75.63008	79.31489	1596.45398	-1369.33862	1732.02659	1
213	C	81.64968	73.55062	78.91468	585.48547	-340.24194	-406.23938	1
214	O	82.49949	73.80871	77.80682	-419.15649	-173.04408	-511.94141	1
215	H	82.45883	74.79372	77.79225	-1071.84350	1559.71507	-1960.13012	1
216	C	76.64095	71.64342	82.82044	291.41878	-183.13091	710.72246	1
217	O	76.67685	70.49688	83.22284	379.87695	523.47396	-222.23622	1
218	N	75.94449	72.68107	83.31960	564.30591	373.09380	277.24371	1
219	H	76.05794	73.53579	82.77077	-120.59931	-2436.07824	-1214.35098	1
220	C	74.67868	72.39088	83.93601	404.07514	-299.37480	608.84009	1
221	H	74.18323	73.38709	83.93997	-1071.07882	-841.69025	2359.66467	1
222	C	73.97865	71.35141	82.96780	-683.12807	68.71770	-390.13096	1
223	H	74.24380	71.58960	81.88812	2068.88918	1596.58590	770.89603	1
224	H	74.39734	70.33144	83.15369	1379.86429	2342.49779	-179.85638	1
225	C	72.44034	71.39009	83.19415	-176.39967	537.49466	550.97268	1
226	H	72.18098	71.59187	84.25646	-396.28051	1942.22168	1869.33314	1
227	H	71.96379	72.21165	82.61620	-94.44700	586.00678	-2659.20016	1
228	C	71.88624	69.94685	82.73771	-291.69381	-718.18079	-150.72929	1
229	H	72.04073	69.74666	81.64552	-2303.38776	1089.76242	964.62032	1
230	H	72.25125	69.09883	83.39445	884.16729	2568.80533	-207.74283	1
231	N	70.54290	70.18676	82.99411	-594.49522	-252.81478	342.29276	1
232	H	70.19013	69.82444	83.87118	-1735.17426	2084.05705	-263.71605	1
233	C	69.72579	70.80782	82.22306	595.23177	-271.52487	442.22352	1
234	N	70.21799	71.31701	81.02338	585.53813	-424.79750	-105.81303	1
235	H	71.17092	71.28900	80.66028	959.88797	1327.56823	-2177.11905	1
236	H	69.68236	71.84520	80.33391	1578.48647	16.27378	-2220.76233	1
237	N	68.45518	70.84027	82.80005	-452.17763	-390.33541	-421.51853	1
238	H	68.11955	70.44771	83.67916	470.26107	1974.25629	-1817.92087	1
239	H	67.64014	71.26762	82.35825	1856.67307	-1573.97321	-1224.35416	1

```
C    74.99098  71.83928  85.42649     -531.47271     -581.45780      -55.06206         1
O    75.75240  72.40762  86.17011     -243.00837      192.73507      609.52485         1
N    74.21574  70.75303  85.74544      343.49288     -644.25607       38.09164         1
H    73.61717  70.52525  84.94345    -1691.16043    -1535.97525    -1484.73099         1
C    74.95227  69.57408  86.27598      542.66492      350.14337      454.42563         1
H    76.02250  69.87899  86.32677     1209.57979    -2396.95920      463.86744         1
C    74.35084  69.20410  87.61277     -737.14309      145.93010      242.71942         1
H    73.27387  68.97553  87.44611    -1050.18303      159.36419    -2509.06125         1
C    75.01689  67.97148  88.19754     -706.77696     -252.88343     -245.16230         1
H    74.51822  67.63821  89.14897    -1624.64039    -1665.49085     1417.86935         1
H    74.98902  67.10221  87.48150     2566.10521      598.51109      693.22305         1
H    76.09946  68.16059  88.43757    -1972.69758      107.10018    -1876.34312         1
C    74.42969  70.35731  88.59746      412.85148      672.58832      -27.69222         1
H    73.89722  70.12685  89.56111     1447.02277     1762.76533    -1490.78944         1
H    75.49619  70.60783  88.85299     1320.77379     -145.20455     2378.68491         1
H    73.96936  71.29026  88.16772    -1512.10848     1470.52031     1724.75147         1
C    74.62022  68.38397  85.19173     -607.11113      499.56689       73.73769         1
O    73.50657  68.25080  84.70896      357.97126      460.81534      356.68785         1
N    75.69117  67.63835  85.00568      536.55407     -368.62270      332.76306         1
H    76.54073  67.88858  85.50873      524.38866     2297.69985     1367.20941         1
C    75.56383  66.39577  84.33549       46.50277     -774.29190     -147.97904         1
H    74.46572  66.26158  84.19927     1676.51875     1883.29066     1032.55549         1
C    76.35904  66.45451  82.92851     -559.41456      -34.19198     -556.30433         1
H    77.45703  66.45326  83.12625      -64.66664      395.18769     2695.05373         1
H    76.15894  65.53266  82.33280    -2703.44278      -60.40056      333.79418         1
C    75.97457  67.68475  82.15554     -519.76435      377.31045      459.42366         1
H    76.23033  68.59138  82.77537    -1746.74323     1667.50644    -1261.73691         1
C    76.75574  67.75941  80.84907      570.95554       16.46261     -545.27700         1
H    76.52505  68.70110  80.28691     2391.58273       28.05277    -1305.07271         1
H    77.86169  67.73997  81.02689      175.75588      917.63458     2559.43867         1
H    76.51229  66.89993  80.17273    -1063.97851    -2155.34650     1283.00462         1
C    74.48426  67.71854  81.85321      193.76014        2.62946     -765.53141         1
H    74.21670  68.58565  81.19502      507.39394     -846.55087     2539.60089         1
H    74.14055  66.78718  81.33340     1337.57841     1818.19054     1526.01994         1
H    73.88215  67.82248  82.81013     1100.88635    -1172.91350    -2199.08909         1
C    76.27440  65.29709  85.27417     -549.21115      475.64486     -309.38218         1
O    76.71072  65.54493  86.36814      556.06106     -390.37755      -78.24250         1
O    76.35066  63.98263  84.75229     -572.27940     -310.19032     -209.75862         1
H    75.87977  64.13282  83.88813    -1380.08193     2212.14486      790.85944         1
```

Listing 20: C00020.xmolout

```
        276
   150.0000  150.0000  150.0000     0.0000     0.0000     0.0000***********    0.8085     3234
        0.2500     300.00    0.00    0.00    0.00  -1294.0363       0.0000    300.0000
H    75.02491  70.21798  67.06212    -2043.14101    -1757.64893      399.89339         1
N    75.27755  70.77793  66.24906      426.75047      490.45733     -334.42467         1
H    74.56010  71.50013  66.25008      442.25559    -1110.30854    -2448.52907         1
C    76.60637  71.25210  66.20595      415.32972     -666.39213       83.73172         1
H    77.23499  70.36096  66.41421      879.32219     2491.00447      667.35213         1
C    76.88367  71.92619  64.77038       31.35357      477.41777      628.23362         1
H    76.03540  72.60534  64.51266     1168.38489     2240.25337    -1019.71136         1
H    77.79382  72.57276  64.82154     -360.43770     2534.90498      931.67112         1
C    77.05327  70.84892  63.72893      463.18208      512.70242     -382.34388         1
H    76.24898  70.09278  63.87340     1725.29117     1863.52247     -987.07854         1
C    76.92389  71.43090  62.32729      710.42801     -144.27427     -313.15367         1
H    77.05070  70.64378  61.53950      364.20195    -1033.13725     2494.72524         1
H    75.92147  71.90521  62.16537     2567.61904      335.80679      847.48802         1
H    77.69795  72.21852  62.13554    -2587.99029      680.92867     -512.16411         1
C    78.40714  70.16588  63.86809      268.17616      741.64379      -40.42634         1
H    78.54454  69.35906  63.10252     1283.54314    -1945.37420    -1411.27812         1
H    79.24957  70.89354  63.73656    -1258.57796     1317.15249    -2026.02085         1
```

H	78.53479	69.69269	64.87543	-2502.84526	909.80597	575.92391	1
C	76.83978	72.44725	67.33216	619.36578	-458.64843	-172.09273	1
O	78.09245	73.02542	67.31352	-81.40433	433.87611	522.34594	1
N	75.73038	73.38413	67.10132	175.23996	-622.20190	341.55523	1
H	74.82330	73.00393	67.37902	-876.57256	-303.22879	-2561.90213	1
C	76.01478	74.64510	67.73108	-571.88214	-534.10783	-106.15033	1
H	76.97320	74.96651	67.26537	-2713.20841	-16.25800	-248.80809	1
C	74.78597	75.61009	67.41777	166.60354	288.16399	-716.09568	1
H	73.88533	75.18551	67.93693	-2097.85574	1636.78449	-586.18044	1
O	74.44297	75.74856	66.02381	-317.80580	-363.38227	484.43082	1
H	73.60424	75.24869	65.98372	1863.74748	-1451.84923	1357.29467	1
C	75.23751	76.93214	68.14074	503.80731	517.99032	318.51606	1
H	74.45044	77.71866	68.08490	640.61020	-2157.10410	-1536.29104	1
H	75.45984	76.76142	69.22197	1687.88455	465.60692	2087.56452	1
H	76.15798	77.35494	67.67542	-1293.07895	-1899.88581	-1463.57452	1
C	76.11471	74.39852	69.33356	-153.07550	-733.68902	248.70981	1
O	75.14619	74.63755	70.00698	186.15459	-480.78787	-449.34387	1
N	77.30520	73.84849	69.71889	-487.53139	543.18517	42.03850	1
C	77.57172	73.60477	71.12251	-699.35871	69.34393	360.10701	1
H	76.62037	73.32712	71.62932	2.81126	-221.99807	-2715.58072	1
C	78.67062	72.47656	71.00902	-507.46439	-592.59194	122.07761	1
H	79.24981	72.33393	71.94935	-1975.08116	525.87560	-1801.71556	1
H	78.23008	71.49003	70.74478	1588.27743	-1913.22100	1113.83566	1
C	79.51995	73.07197	69.82781	-613.62442	-457.55993	-194.14490	1
H	80.20557	73.87225	70.19855	1707.54637	-721.27992	1996.92523	1
H	80.16391	72.32038	69.31452	758.23357	2100.81767	1560.55011	1
C	78.42557	73.67518	68.88949	-352.41524	704.96076	49.21465	1
H	78.66550	74.64245	68.37207	1714.76886	-2070.42778	443.35792	1
H	76.53427	72.09330	68.35626	1224.15040	1801.50881	1636.97633	1
C	78.30312	74.93279	71.72832	566.85117	537.05831	117.62814	1
O	79.03634	75.63258	71.07095	-550.26779	-406.08867	4.21666	1
N	77.99555	75.04304	73.02975	566.34832	-425.77725	180.18749	1
H	77.45236	74.26968	73.41818	644.44502	2646.80550	-52.73430	1
C	79.00363	75.65090	73.85438	612.34801	-281.48662	411.56221	1
H	79.63155	76.28012	73.18174	1667.23498	-1490.75142	1556.16753	1
C	78.12072	76.52257	74.87230	-714.45746	-175.43074	286.98927	1
H	77.20449	75.92419	75.12549	-787.09484	1763.03619	-1922.46060	1
O	77.62354	77.75238	74.30522	-429.46514	516.51932	-128.40526	1
H	76.68520	77.52670	74.15598	1981.82785	436.16847	-1818.18182	1
C	78.99964	76.67781	76.16187	630.79189	-468.94448	76.03230	1
H	78.45655	77.25421	76.94734	-1775.71861	2062.30353	131.89849	1
H	79.26941	75.68661	76.60086	-1855.14608	88.94118	1993.53760	1
H	79.94925	77.22147	75.94963	-1504.82350	-780.94249	-2132.90992	1
C	79.81875	74.42435	74.51279	288.57638	618.62814	-397.00270	1
O	79.28947	73.30659	74.63102	321.70495	-185.26452	574.37218	1
N	80.97999	74.83280	74.97406	-447.05792	-446.53431	367.76425	1
H	81.20411	75.80276	74.75428	350.31316	1975.63644	-1843.31522	1
C	82.02640	73.86452	75.15057	-511.23061	374.03917	-471.51480	1
H	82.08883	73.31915	74.18327	2234.79579	-898.70890	-1273.45190	1
C	83.32954	74.71880	75.49260	529.53781	-159.34528	563.72604	1
H	84.19574	74.00667	75.56824	654.59388	2347.94251	1217.51488	1
H	83.52971	75.38210	74.61095	-1526.55661	2103.42026	817.87394	1
O	83.29781	75.48941	76.72989	-554.84278	14.12789	399.58769	1
H	82.72921	76.22483	76.44258	1297.06236	2061.84854	1220.68812	1
C	81.66627	72.85056	76.36552	-662.15484	-412.13544	123.62687	1
O	82.02901	71.69898	76.29858	-540.01537	-317.05804	274.91456	1
N	81.13325	73.48792	77.42863	-202.76299	-690.00100	-131.49145	1
H	80.90036	74.44598	77.16476	-702.62132	-2617.37191	281.70413	1
C	80.38615	72.73736	78.45771	-491.73914	576.34187	222.73509	1
H	79.93522	71.91869	77.85108	1977.17978	-1823.58850	-434.68928	1
C	81.34659	72.22087	79.50392	-649.50797	-328.66843	306.11224	1
H	81.72765	73.09399	80.08044	2075.07803	1452.16490	-1004.45904	1

82	C	80.65922	71.26290	80.46254	-615.18735	-489.99689	-70.96352	1
83	H	81.33727	70.96324	81.30730	-1226.32661	-1724.38906	-1716.47170	1
84	H	79.73468	71.71261	80.91698	2381.64273	-969.84735	900.46860	1
85	H	80.34189	70.31885	79.94200	2122.74991	1702.23946	-141.36228	1
86	C	82.56524	71.53867	78.90159	-501.86074	-479.61276	-376.42482	1
87	H	83.27980	71.17805	79.69129	993.79261	-2246.95401	-1177.81305	1
88	H	82.27309	70.64766	78.27712	42.71279	-626.15391	-2651.37258	1
89	H	83.13238	72.23931	78.22557	-2026.39012	-1590.33709	-887.82922	1
90	C	79.27556	73.83261	78.99369	-672.89153	382.57394	-156.34072	1
91	O	78.46337	74.27600	78.20852	410.04627	221.86223	500.35950	1
92	N	79.41220	74.13479	80.27098	-378.11782	-443.79414	-441.10984	1
93	H	80.15649	73.70306	80.81341	2404.15973	-568.14894	1149.30085	1
94	C	78.48160	75.02443	80.87452	-544.54484	466.16019	331.29119	1
95	H	77.78943	75.34842	80.05962	-1409.80988	-170.17955	2325.32679	1
96	C	77.81476	74.19828	82.05719	-184.48284	116.78282	-758.89137	1
97	H	77.32145	73.28050	81.63665	1893.78641	1861.14652	611.04528	1
98	H	78.59200	73.83865	82.77018	-2170.79665	-974.19311	1327.50117	1
99	C	76.77924	75.10870	82.78431	389.51668	672.81878	-138.49152	1
100	H	77.31426	75.94854	83.28897	510.64577	1933.03327	1851.02484	1
101	H	76.11210	75.60784	82.04722	-2302.30869	-1105.72939	-948.89774	1
102	C	75.90561	74.31153	83.82562	-559.93427	-126.34590	542.30857	1
103	O	75.00536	74.86983	84.39613	-670.36003	-111.83166	76.36448	1
104	N	76.26669	73.01647	83.97431	-216.06729	-537.07037	446.51379	1
105	H	77.07890	72.52402	83.58589	-1251.96487	1074.12894	-2168.52500	1
106	H	75.87285	72.39462	84.68870	-2451.83023	-720.73851	944.84612	1
107	C	79.33141	76.22195	81.60223	546.24052	-563.88337	-85.11809	1
108	O	79.53588	76.13459	82.78373	71.70702	-307.40463	606.69673	1
109	N	79.73054	77.15677	80.72300	479.90863	-504.50129	222.86849	1
110	C	80.53495	78.25317	81.15023	75.51268	-600.91904	506.73700	1
111	H	81.05439	77.90193	82.07145	-2033.61562	1795.30103	254.89480	1
112	C	81.45256	78.48263	79.87941	523.79067	517.99274	284.45581	1
113	H	81.80775	79.53432	79.79791	1599.61665	-1127.00911	1895.98173	1
114	H	82.36824	77.85316	79.94248	-1881.83093	1275.82488	-1501.55016	1
115	C	80.57590	78.04553	78.66004	-600.05639	-404.05026	316.64485	1
116	H	80.05619	78.91154	78.19125	-2597.05260	496.36221	657.73249	1
117	H	81.18347	77.58998	77.84617	-1666.24518	1294.38985	-1723.90591	1
118	C	79.54428	77.01751	79.28412	493.57570	-129.95376	-602.56394	1
119	H	78.45306	77.24155	79.06427	-1469.08415	-637.84797	2204.22581	1
120	H	79.75581	75.94588	79.05002	-1038.97840	1068.88701	-2280.71789	1
121	C	79.57238	79.55008	81.47134	188.88100	241.05089	-727.87821	1
122	O	79.60486	80.04561	82.57376	531.88613	-49.19943	427.07960	1
123	N	78.92027	79.96096	80.39430	708.14833	26.93417	-179.73509	1
124	H	79.02587	79.40736	79.54700	-2232.69501	-1179.09831	1023.94816	1
125	C	77.79627	80.83079	80.54480	-738.32412	259.92548	104.42456	1
126	H	77.32538	80.56221	81.51610	2028.89123	1260.02983	1311.33324	1
127	C	78.33640	82.32026	80.52147	-258.65678	-345.52513	661.28459	1
128	H	78.81261	82.54105	79.54139	-1826.06090	-1801.46097	-918.64554	1
129	C	77.10736	83.25939	80.77440	-541.23400	531.92970	-218.41428	1
130	H	77.40367	84.33448	80.76861	1311.73218	-2219.18200	882.19023	1
131	H	76.32547	83.12565	79.98982	-1048.71298	1223.58118	-2196.97971	1
132	H	76.62853	83.05155	81.75989	419.06414	1903.92898	1903.44670	1
133	C	79.36428	82.53539	81.68748	-423.86431	152.66566	-648.55262	1
134	H	79.65858	83.60684	81.78707	105.68386	-2342.50080	1387.51238	1
135	H	78.94376	82.20827	82.67010	-1695.63603	-1484.73621	-1531.02793	1
136	H	80.29961	81.94901	81.51714	-1853.57121	-487.39031	1936.59343	1
137	C	76.88659	80.58109	79.20222	539.71428	-510.58232	267.58633	1
138	O	77.29763	80.88673	78.10585	-15.93758	-278.34085	-624.49402	1
139	N	75.69097	80.13189	79.56463	-676.81886	12.05292	276.17960	1
140	H	75.53892	79.97335	80.56094	-1694.44250	-373.84189	-2100.66098	1
141	C	74.85550	79.44430	78.63314	412.33078	-664.70360	108.35451	1
142	H	73.84711	79.46958	79.10351	1765.77476	1294.44366	1621.76594	1
143	C	74.94862	80.22609	77.26065	-179.66635	-721.95667	-264.73915	1

H	75.14168	81.30432	77.46950	-607.34922	2587.87032	-598.10018	1
H	75.83775	79.88600	76.67821	-359.69332	-1479.13954	-2259.74260	1
C	73.64107	80.04803	76.41927	-240.01076	616.02425	431.85322	1
H	73.58256	79.03210	75.96798	-1359.17131	782.54425	2227.99181	1
H	72.73339	80.15726	77.05423	-820.36667	85.81767	-2596.78715	1
C	73.68026	81.17139	75.30591	-513.07015	236.18570	-551.87280	1
H	73.69403	82.18287	75.77366	1816.11944	-1183.11722	-1650.94321	1
H	74.62112	81.10228	74.71269	-1800.04429	-1361.53473	1526.34643	1
C	72.44847	81.02050	74.38372	726.08972	-253.19732	-179.64713	1
H	72.47569	80.13960	73.70910	1224.27271	-456.51798	-2390.90304	1
H	71.47087	81.06996	74.90382	1206.40339	-1150.93514	-2154.90344	1
N	72.44206	82.26234	73.42845	329.70796	-633.68968	-155.67358	1
H	71.65610	82.28940	72.71700	1348.31305	1938.76845	1359.00643	1
H	72.34739	83.21954	73.87813	2096.92035	1446.54551	-966.48890	1
H	73.29137	82.39595	72.80619	-1197.07217	-2303.53719	827.28733	1
C	75.41071	77.91733	78.44032	-378.01186	-556.17301	-413.96481	1
O	76.55409	77.55020	78.62632	-409.44433	-307.55053	-453.30833	1
N	74.35103	77.13655	78.07021	-429.02477	524.70884	274.08502	1
H	73.60368	77.83533	77.97096	1706.34263	-1425.96036	1574.38926	1
C	74.59402	76.30651	76.89638	-45.25864	-626.22952	478.93267	1
H	74.88623	77.00557	76.08252	1988.63164	570.90773	1772.87287	1
C	75.72504	75.27108	77.24069	551.77128	-20.49526	-564.54996	1
H	76.73354	75.74877	77.24981	1434.61535	-1753.10253	1513.99471	1
H	75.58994	74.85820	78.26604	-714.67738	1819.09770	1898.36494	1
C	75.62954	74.13937	76.15720	446.99263	-555.77305	338.97221	1
H	74.65102	73.60779	76.22747	1953.68466	1892.25957	-161.67766	1
H	75.66776	74.55781	75.12606	-403.03785	2688.07032	-188.43591	1
C	76.84100	73.16691	76.42738	567.86262	335.74681	434.04421	1
H	77.81395	73.70291	76.27021	-1080.02275	1568.26081	-1948.78900	1
H	76.84554	72.83141	77.49212	-919.54108	2111.34692	1456.13463	1
C	76.73172	71.95183	75.46549	195.81897	173.33862	-745.11536	1
H	75.96005	71.20637	75.74130	1405.67949	-2301.21089	-390.08113	1
H	76.63391	72.21978	74.39319	-940.37328	-1118.32446	2299.72133	1
N	78.09219	71.22369	75.55541	319.26422	656.36007	-42.02424	1
H	78.18176	70.32256	75.00573	1961.03633	619.91968	1787.09422	1
H	78.95167	71.86057	75.19724	854.14343	-2156.80752	1429.08711	1
H	78.38398	70.89060	76.51741	1237.02905	-1378.50857	-1998.28500	1
C	73.23747	75.49394	76.56087	532.53319	-268.57236	517.55729	1
O	73.09038	75.06346	75.41128	266.16714	-290.73804	-558.88002	1
N	72.50926	75.26568	77.63637	-226.25352	640.49678	-270.32839	1
H	72.91864	75.69688	78.46578	2007.69063	-1035.27229	1523.50231	1
C	71.13128	74.92720	77.62800	-540.76542	-573.33303	49.50000	1
H	70.79970	75.16568	78.66249	-2374.20950	-1036.76023	843.75774	1
C	70.40989	75.81994	76.51430	-77.09082	666.87422	-415.84136	1
H	70.87060	75.57881	75.51367	-985.81834	617.19825	2463.91927	1
O	70.53133	77.24551	76.66707	293.29899	-391.65602	-477.80926	1
H	71.01676	77.47967	75.84912	2528.72714	-681.76262	-751.27122	1
C	68.95323	75.23109	76.59417	252.47363	-605.14473	440.05134	1
H	68.29601	75.64609	75.79583	-2052.65063	1456.49051	-1043.51830	1
H	68.95029	74.11824	76.49011	-2152.98800	-1661.97574	-161.70652	1
H	68.47521	75.46813	77.57312	-473.12726	-1584.27339	2165.61725	1
C	70.88143	73.34519	77.26028	360.36307	488.46713	505.10056	1
O	70.07737	72.73498	77.91949	-200.08817	-528.03552	-385.82850	1
N	71.54026	72.99030	76.17201	-597.54095	-226.44775	355.20479	1
H	72.15655	73.70162	75.68898	2179.23655	-1300.70981	-991.33835	1
C	71.66717	71.63018	75.79717	-687.68921	297.39807	-249.45179	1
H	72.57532	71.62778	75.15381	-349.16256	-2467.37713	-1101.72831	1
C	71.79093	70.66048	77.06496	672.47345	361.54328	-201.62880	1
H	70.82934	70.67801	77.63337	1541.41502	582.18425	2169.97026	1
H	71.93040	69.61490	76.70483	-2534.67717	60.25153	997.72295	1
C	72.94878	71.06371	77.99119	574.26774	-389.43119	-377.02542	1
C	74.29971	70.73761	77.66738	688.82679	-287.33580	-257.96133	1

206	H	74.53988	70.22764	76.71778	331.14182	1470.38163	2269.80013	1
207	C	72.65145	71.73377	79.21615	-383.52382	-675.04808	144.24917	1
208	H	71.60794	72.01592	79.45655	4.09847	1517.59662	2262.86397	1
209	C	75.30506	71.04536	78.58940	-134.76730	428.45285	-649.50303	1
210	H	76.34701	70.77942	78.33869	-903.23433	1600.49020	2011.53387	1
211	C	73.69038	72.01026	80.11175	-335.53281	-615.00040	-364.39058	1
212	H	73.44012	72.50874	81.06505	1596.45398	-1369.33862	1732.02659	1
213	C	75.04690	71.66738	79.84512	585.48547	-340.24194	-406.23938	1
214	O	76.10788	71.92731	80.77040	-419.15649	-173.04408	-511.94141	1
215	H	75.57624	72.27088	81.52831	-1071.84350	1559.71507	-1960.13012	1
216	C	70.33454	71.17974	74.97911	291.41878	-183.13091	710.72246	1
217	O	70.08535	70.00001	74.85188	379.87695	523.47396	-222.23622	1
218	N	69.69223	72.21507	74.43113	564.30591	373.09380	277.24371	1
219	H	70.03402	73.14698	74.66211	-120.59931	-2436.07824	-1214.35098	1
220	C	68.43685	71.94131	73.82024	404.07514	-299.37480	608.84009	1
221	H	68.35688	70.82672	73.86355	-1071.07882	-841.69025	2359.66467	1
222	C	67.31553	72.68324	74.66869	-683.12807	68.71770	-390.13096	1
223	H	67.45902	72.50855	75.75866	2068.88918	1596.58590	770.89603	1
224	H	67.38477	73.78505	74.52838	1379.86429	2342.49779	-179.85638	1
225	C	65.93329	72.14562	74.18831	-176.39967	537.49466	550.97268	1
226	H	65.87485	72.16462	73.07290	-396.28051	1942.22168	1869.33314	1
227	H	65.80458	71.07673	74.47090	-94.44700	586.00678	-2659.20016	1
228	C	64.77777	73.04556	74.82620	-291.69381	-718.18079	-150.72929	1
229	H	64.58356	72.89826	75.91899	-2303.38776	1089.76242	964.62032	1
230	H	64.86186	74.14565	74.63427	884.16729	2568.80533	-207.74283	1
231	N	63.66734	72.57395	74.10667	-594.49522	-252.81478	342.29276	1
232	H	63.85047	71.90486	73.37479	-1735.17426	2084.05705	-263.71605	1
233	C	62.46363	72.93441	74.30097	595.23177	-271.52487	442.22352	1
234	N	62.21322	73.86575	75.31447	585.53813	-424.79750	-105.81303	1
235	H	62.86677	74.31894	75.95150	959.88797	1327.56823	-2177.11905	1
236	H	61.29096	74.22577	75.56183	1578.48647	16.27378	-2220.76233	1
237	N	61.59535	72.28921	73.40800	-452.17763	-390.33541	-421.51853	1
238	H	61.78013	71.61660	72.66557	470.26107	1974.25629	-1817.92087	1
239	H	60.58722	72.44697	73.39645	1856.67307	-1573.97321	-1224.35416	1
240	C	68.33345	72.53498	72.29822	-531.47271	-581.45780	-55.06206	1
241	O	67.42827	72.12612	71.59614	-243.00837	192.73507	609.52485	1
242	N	69.21255	73.47868	72.00213	343.49288	-644.25607	38.09164	1
243	H	69.90959	73.73610	72.69757	-1691.16043	-1535.97525	-1484.73099	1
244	C	69.05858	74.10640	70.72539	542.66492	350.14337	454.42563	1
245	H	68.69727	73.26571	70.09088	1209.57979	-2396.95920	463.86744	1
246	C	67.93128	75.21510	70.86711	-737.14309	145.93010	242.71942	1
247	H	68.17548	75.93809	71.67396	-1050.18303	159.36419	-2509.06125	1
248	C	67.79566	75.92907	69.47939	-706.77696	-252.88343	-245.16230	1
249	H	66.91664	76.61500	69.45104	-1624.64039	-1665.49085	1417.86935	1
250	H	68.70733	76.54285	69.25025	2566.10521	598.51109	693.22305	1
251	H	67.67025	75.19286	68.65037	-1972.69758	107.10018	-1876.34312	1
252	C	66.53217	74.55694	71.13841	412.85148	672.58832	-27.69222	1
253	H	65.70342	75.29842	71.04503	1447.02277	1762.76533	-1490.78944	1
254	H	66.32331	73.73000	70.41672	1320.77379	-145.20455	2378.68491	1
255	H	66.47445	74.12288	72.16519	-1512.10848	1470.52031	1724.75147	1
256	C	70.50259	74.71249	70.23754	-607.11113	499.56689	73.73769	1
257	O	71.17213	74.04601	69.48183	357.97126	460.81534	356.68785	1
258	N	70.77508	75.91122	70.73562	536.55407	-368.62270	332.76306	1
259	H	70.11556	76.35905	71.36961	524.38866	2297.69985	1367.20941	1
260	C	72.01246	76.52034	70.40020	46.50277	-774.29190	-147.97904	1
261	H	72.62114	75.73026	69.89807	1676.51875	1883.29066	1032.55549	1
262	C	71.74273	77.81185	69.50470	-559.41456	-34.19198	-556.30433	1
263	H	71.19022	78.56551	70.10806	-64.66664	395.18769	2695.05373	1
264	H	72.70758	78.29306	69.23467	-2703.44278	-60.40056	333.79418	1
265	C	70.92114	77.49308	68.21210	-519.76435	377.31045	459.42366	1
266	H	69.91359	77.09055	68.48426	-1746.74323	1667.50644	-1261.73691	1
267	C	70.78828	78.82567	67.38676	570.95554	16.46261	-545.27700	1

```
H    70.20114  78.67242  66.45158     2391.58273       28.05277    -1305.07271          1
H    70.27496  79.62311  67.97256      175.75588      917.63458     2559.43867          1
H    71.78584  79.22334  67.08807    -1063.97851    -2155.34650     1283.00462          1
C    71.67746  76.45690  67.30558      193.76014        2.62946     -765.53141          1
H    71.12399  76.26026  66.35777      507.39394     -846.55087     2539.60089          1
H    72.69370  76.82399  67.02461     1337.57841     1818.19054     1526.01994          1
H    71.79554  75.47601  67.82891     1100.88635    -1172.91350    -2199.08909          1
C    72.63380  77.14195  71.77271     -549.21115      475.64486     -309.38218          1
O    71.99941  77.88263  72.47735      556.06106     -390.37755      -78.24250          1
O    73.99623  76.89395  72.02314     -572.27940     -310.19032     -209.75862          1
H    74.17442  76.21257  71.31089    -1380.08193     2212.14486      790.85944          1
```

Listing 21: C00021.xmolout

```
        276
  150.0000  150.0000  150.0000     0.0000     0.0000     0.0000***********    2.7750    11100
         0.2500     300.00    0.00    0.00    0.00   -1294.0188       0.0000     300.0000
H    74.92939  76.99095  81.98003    -2043.14101    -1757.64893      399.89339          1
N    74.85370  77.74287  81.29333      426.75047      490.45733     -334.42467          1
H    73.97356  78.22362  81.56500      442.25559    -1110.30854    -2448.52907          1
C    76.01257  78.56109  81.27914      415.32972     -666.39213       83.73172          1
H    76.41291  78.56091  82.31597      879.32219     2491.00447      667.35213          1
C    77.05677  77.98184  80.19701       31.35357      477.41777      628.23362          1
H    76.54281  77.91706  79.20810     1168.38489     2240.25337    -1019.71136          1
H    77.89233  78.70989  80.06030     -360.43770     2534.90498      931.67112          1
C    77.59894  76.63039  80.58742      463.18208      512.70242     -382.34388          1
H    76.74894  75.97924  80.89015     1725.29117     1863.52247     -987.07854          1
C    78.29898  75.98735  79.39628      710.42801     -144.27427     -313.15367          1
H    78.71362  74.97882  79.65388      364.20195    -1033.13725     2494.72524          1
H    77.60167  75.84901  78.52988     2567.61904      335.80679      847.48802          1
H    79.15501  76.61436  79.03567    -2587.99029      680.92867     -512.16411          1
C    78.57578  76.74455  81.74938      268.17616      741.64379      -40.42634          1
H    79.00304  75.74722  82.03060     1283.54314    -1945.37420    -1411.27812          1
H    79.43877  77.41353  81.49559    -1258.57796     1317.15249    -2026.02085          1
H    78.08772  77.16801  82.66469    -2502.84526      909.80597      575.92391          1
C    75.57971  80.04526  80.74324      619.36578     -458.64843     -172.09273          1
O    76.07535  81.06334  81.17175      -81.40433      433.87611      522.34594          1
N    74.76467  79.88835  79.69703      175.23996     -622.20190      341.55523          1
H    74.47910  78.92652  79.51144     -876.57256     -303.22879    -2561.90213          1
C    74.12604  81.03393  79.14868     -571.88214     -534.10783     -106.15033          1
H    74.76101  81.87762  79.50979    -2713.20841      -16.25800     -248.80809          1
C    74.11982  80.86754  77.55381      166.60354      288.16399     -716.09568          1
H    73.55483  79.93528  77.27767    -2097.85574     1636.78449     -586.18044          1
O    75.40623  80.70675  76.92842     -317.80580     -363.38227      484.43082          1
H    75.30492  79.82106  76.52374     1863.74748    -1451.84923     1357.29467          1
C    73.29213  82.12805  77.11126      503.80731      517.99032      318.51606          1
H    73.11138  82.13332  76.01143      640.61020    -2157.10410    -1536.29104          1
H    72.29500  82.16905  77.61292     1687.88455      465.60692     2087.56452          1
H    73.82659  83.07305  77.36550    -1293.07895    -1899.88581    -1463.57452          1
C    72.60407  81.12541  79.73832     -153.07550     -733.68902      248.70981          1
O    72.18464  82.24324  79.89478      186.15459     -480.78787     -449.34387          1
N    71.93216  79.97200  79.83336     -487.53139      543.18517       42.03850          1
C    70.80580  79.87977  80.71760     -699.35871       69.34393      360.10701          1
H    70.42635  80.91727  80.87714        2.81126     -221.99807    -2715.58072          1
C    69.84464  78.88827  79.91673     -507.46439     -592.59194      122.07761          1
H    69.08445  78.39937  80.56526    -1975.08116      525.87560    -1801.71556          1
H    69.28808  79.41630  79.11149     1588.27743    -1913.22100     1113.83566          1
C    70.88528  77.88971  79.32926     -613.62442     -457.55993     -194.14490          1
H    71.28111  77.20856  80.11692     1707.54637     -721.27992     1996.92523          1
H    70.48417  77.24501  78.51534      758.23357     2100.81767     1560.55011          1
C    71.98834  78.91802  78.82650     -352.41524      704.96076       49.21465          1
H    73.02142  78.50199  78.78344     1714.76886    -2070.42778      443.35792          1
```

H	71.74858	79.41325	77.85614	1224.15040	1801.50881	1636.97633	1
C	71.22344	79.11157	82.09059	566.85117	537.05831	117.62814	1
O	72.33354	78.60291	82.28164	-550.26779	-406.08867	4.21666	1
N	70.16775	79.11643	82.86215	566.34832	-425.77725	180.18749	1
H	69.34543	79.58258	82.48706	644.44502	2646.80550	-52.73430	1
C	70.11807	78.33773	84.05777	612.34801	-281.48662	411.56221	1
H	71.09455	78.55170	84.54777	1667.23498	-1490.75142	1556.16753	1
C	68.81211	78.73392	84.88272	-714.45746	-175.43074	286.98927	1
H	67.91481	78.36507	84.31213	-787.09484	1763.03619	-1922.46060	1
O	68.59773	80.13837	85.10669	-429.46514	516.51932	-128.40526	1
H	67.73873	80.27742	84.65847	1981.82785	436.16847	-1818.18182	1
C	68.99845	77.85016	86.17257	630.79189	-468.94448	76.03230	1
H	68.09007	77.87686	86.81839	-1775.71861	2062.30353	131.89849	1
H	69.19979	76.77797	85.91957	-1855.14608	88.94118	1993.53760	1
H	69.85250	78.21039	86.79157	-1504.82350	-780.94249	-2132.90992	1
C	69.95547	76.76985	83.61804	288.57638	618.62814	-397.00270	1
O	68.88274	76.23813	83.72098	321.70495	-185.26452	574.37218	1
N	71.10514	76.32136	83.10333	-447.05792	-446.53431	367.76425	1
H	71.86005	77.03393	82.95237	350.31316	1975.63644	-1843.31522	1
C	71.56498	75.01800	83.48147	-511.23061	374.03917	-471.51480	1
H	72.50226	74.96047	82.88452	2234.79579	-898.70890	-1273.45190	1
C	71.90712	75.01283	85.04731	529.53781	-159.34528	563.72604	1
H	72.55977	74.11547	85.22100	654.59388	2347.94251	1217.51488	1
H	72.53045	75.92344	85.25969	-1526.55661	2103.42026	817.87394	1
O	70.79224	75.01040	85.97596	-554.84278	14.12789	399.58769	1
H	70.54493	74.06446	85.91818	1297.06236	2061.84854	1220.68812	1
C	70.51519	73.85837	83.09285	-662.15484	-412.13544	123.62687	1
O	70.48145	72.81682	83.71467	-540.01537	-317.05804	274.91456	1
N	69.84815	74.14306	81.95613	-202.76299	-690.00100	-131.49145	1
H	69.91163	75.12059	81.66896	-702.62132	-2617.37191	281.70413	1
C	68.68147	73.33536	81.72790	-491.73914	576.34187	222.73509	1
H	69.06239	72.32051	81.98654	1977.17978	-1823.58850	-434.68928	1
C	67.53430	73.81719	82.70944	-649.50797	-328.66843	306.11224	1
H	67.37643	74.91601	82.62091	2075.07803	1452.16490	-1004.45904	1
C	66.23268	73.02754	82.33991	-615.18735	-489.99689	-70.96352	1
H	65.38066	73.29929	83.00667	-1226.32661	-1724.38906	-1716.47170	1
H	65.91926	73.24625	81.28888	2381.64273	-969.84735	900.46860	1
H	66.38251	71.92521	82.42187	2122.74991	1702.23946	-141.36228	1
C	67.94642	73.44778	84.17791	-501.86074	-479.61276	-376.42482	1
H	67.09796	73.57276	84.89147	993.79261	-2246.95401	-1177.81305	1
H	68.30184	72.39092	84.25413	42.71279	-626.15391	-2651.37258	1
H	68.78078	74.10458	84.53412	-2026.39012	-1590.33709	-887.82922	1
C	68.27642	73.54365	80.15864	-672.89153	382.57394	-156.34072	1
O	67.36017	74.25182	79.82557	410.04627	221.86223	500.35950	1
N	69.08713	72.80270	79.41853	-378.11782	-443.79414	-441.10984	1
H	69.86385	72.23387	79.81346	2404.15973	-568.14894	1149.30085	1
C	69.18610	73.01951	77.99525	-544.54484	466.16019	331.29119	1
H	68.18437	72.78229	77.57196	-1409.80988	-170.17955	2325.32679	1
C	69.55526	74.54872	77.78503	-184.48284	116.78282	-758.89137	1
H	68.85752	75.18125	78.37992	1893.78641	1861.14652	611.04528	1
H	70.56999	74.76236	78.19092	-2170.79665	-974.19311	1327.50117	1
C	69.48120	74.92471	76.27264	389.51668	672.81878	-138.49152	1
H	70.29518	74.41408	75.70996	510.64577	1933.03327	1851.02484	1
H	68.53077	74.54322	75.83936	-2302.30869	-1105.72939	-948.89774	1
C	69.56059	76.49143	76.04101	-559.93427	-126.34590	542.30857	1
O	70.12236	76.94536	75.07830	-670.36003	-111.83166	76.36448	1
N	68.87848	77.19008	76.97507	-216.06729	-537.07037	446.51379	1
H	68.29854	76.83433	77.74329	-1251.96487	1074.12894	-2168.52500	1
H	68.70937	78.20044	76.92874	-2451.83023	-720.73851	944.84612	1
C	70.35817	71.97382	77.64481	546.24052	-563.88337	-85.11809	1
O	70.88158	71.36687	78.56381	71.70702	-307.40463	606.69673	1
N	70.57528	71.84981	76.33582	479.90863	-504.50129	222.86849	1

C	71.85916	71.43240	75.83467	75.51268	-600.91904	506.73700	1
H	72.14262	70.60452	76.52444	-2033.61562	1795.30103	254.89480	1
C	71.53402	70.97470	74.35167	523.79067	517.99274	284.45581	1
H	72.39239	71.11065	73.64875	1599.61665	-1127.00911	1895.98173	1
H	71.27085	69.89346	74.31531	-1881.83093	1275.82488	-1501.55016	1
C	70.30322	71.86171	73.98666	-600.05639	-404.05026	316.64485	1
H	70.61750	72.89959	73.73352	-2597.05260	496.36221	657.73249	1
H	69.72084	71.48087	73.11736	-1666.24518	1294.38985	-1723.90591	1
C	69.49717	71.80668	75.35606	493.57570	-129.95376	-602.56394	1
H	68.79502	72.65138	75.53511	-1469.08415	-637.84797	2204.22581	1
H	68.96189	70.84504	75.54091	-1038.97840	1068.88701	-2280.71789	1
C	72.91492	72.66296	75.95757	188.88100	241.05089	-727.87821	1
O	72.76129	73.56969	76.74140	531.88613	-49.19943	427.07960	1
N	73.98355	72.41854	75.19830	708.14833	26.93417	-179.73509	1
H	74.01643	71.62907	74.55162	-2232.69501	-1179.09831	1023.94816	1
C	74.95086	73.47270	75.11250	-738.32412	259.92548	104.42456	1
H	75.00942	73.85828	76.15661	2028.89123	1260.02983	1311.33324	1
C	76.29665	72.83857	74.55790	-258.65678	-345.52513	661.28459	1
H	76.18926	72.56475	73.48384	-1826.06090	-1801.46097	-918.64554	1
C	77.51322	73.81288	74.73219	-541.23400	531.92970	-218.41428	1
H	78.48829	73.28430	74.57244	1311.73218	-2219.18200	882.19023	1
H	77.47322	74.65097	73.99607	-1048.71298	1223.58118	-2196.97971	1
H	77.54290	74.25781	75.75542	419.06414	1903.92898	1903.44670	1
C	76.63540	71.55595	75.39166	-423.86431	152.66566	-648.55262	1
H	77.63301	71.13965	75.11673	105.68386	-2342.50080	1387.51238	1
H	76.64626	71.76603	76.48709	-1695.63603	-1484.73621	-1531.02793	1
H	75.88117	70.75151	75.20902	-1853.57121	-487.39031	1936.59343	1
C	74.34227	74.59435	74.09895	539.71428	-510.58232	267.58633	1
O	73.35592	74.43876	73.37854	-15.93758	-278.34085	-624.49402	1
N	75.16468	75.61962	74.12100	-676.81886	12.05292	276.17960	1
H	75.93283	75.55558	74.78376	-1694.44250	-373.84189	-2100.66098	1
C	74.87714	76.82543	73.40039	412.33078	-664.70360	108.35451	1
H	73.78688	76.97762	73.57243	1765.77476	1294.44366	1621.76594	1
C	75.79790	77.94167	74.04206	-179.66635	-721.95667	-264.73915	1
H	75.75505	77.91098	75.15356	-607.34922	2587.87032	-598.10018	1
H	76.86163	77.74696	73.78477	-359.69332	-1479.13954	-2259.74260	1
C	75.35044	79.33977	73.50103	-240.01076	616.02425	431.85322	1
H	75.08522	79.27309	72.41481	-1359.17131	782.54425	2227.99181	1
H	74.44059	79.71022	74.02546	-820.36667	85.81767	-2596.78715	1
C	76.57978	80.31277	73.70329	-513.07015	236.18570	-551.87280	1
H	76.83566	80.41775	74.78311	1816.11944	-1183.11722	-1650.94321	1
H	77.48834	79.89236	73.21464	-1800.04429	-1361.53473	1526.34643	1
C	76.23497	81.68633	73.08101	726.08972	-253.19732	-179.64713	1
H	75.94568	81.64906	72.01147	1224.27271	-456.51798	-2390.90304	1
H	75.50099	82.28820	73.65430	1206.40339	-1150.93514	-2154.90344	1
N	77.56168	82.51301	73.09641	329.70796	-633.68968	-155.67358	1
H	77.52394	83.47725	72.65622	1348.31305	1938.76845	1359.00643	1
H	77.97222	82.72923	74.04979	2096.92035	1446.54551	-966.48890	1
H	78.38878	82.07549	72.59322	-1197.07217	-2303.53719	827.28733	1
C	75.19176	76.65988	71.81970	-378.01186	-556.17301	-413.96481	1
O	75.24770	77.64474	71.11317	-409.44433	-307.55053	-453.30833	1
N	75.40079	75.38190	71.44399	-429.02477	524.70884	274.08502	1
H	75.25656	74.72933	72.21917	1706.34263	-1425.96036	1574.38926	1
C	74.73556	75.03558	70.18549	-45.25864	-626.22952	478.93267	1
H	73.75009	75.54564	70.27629	1988.63164	570.90773	1772.87287	1
C	75.63780	75.64159	69.04867	551.77128	-20.49526	-564.54996	1
H	75.80800	76.73130	69.23349	1434.61535	-1753.10253	1513.99471	1
H	76.64671	75.17012	69.06550	-714.67738	1819.09770	1898.36494	1
C	74.94571	75.40999	67.66212	446.99263	-555.77305	338.97221	1
H	74.81255	74.32021	67.46865	1953.68466	1892.25957	-161.67766	1
H	73.92696	75.85788	67.63518	-403.03785	2688.07032	-188.43591	1
C	75.89046	76.07069	66.58050	567.86262	335.74681	434.04421	1

H	75.93178	77.17609	66.71573	-1080.02275	1568.26081	-1948.78900	1
H	76.93846	75.71214	66.70237	-919.54108	2111.34692	1456.13463	1
C	75.35599	75.70752	65.17562	195.81897	173.33862	-745.11536	1
H	75.37632	74.62473	64.94005	1405.67949	-2301.21089	-390.08113	1
H	74.35983	76.12911	64.93431	-940.37328	-1118.32446	2299.72133	1
N	76.32958	76.35662	64.13474	319.26422	656.36007	-42.02424	1
H	76.10759	76.16463	63.11554	1961.03633	619.91968	1787.09422	1
H	76.39930	77.41576	64.13661	854.14343	-2156.80752	1429.08711	1
H	77.35039	76.06934	64.18365	1237.02905	-1378.50857	-1998.28500	1
C	74.61704	73.43172	70.11176	532.53319	-268.57236	517.55729	1
O	75.11924	72.82482	69.16830	266.16714	-290.73804	-558.88002	1
N	73.87644	72.99471	71.10275	-226.25352	640.49678	-270.32839	1
H	73.47683	73.67250	71.79093	2007.69063	-1035.27229	1523.50231	1
C	73.73677	71.62164	71.42733	-540.76542	-573.33303	49.50000	1
H	73.00574	71.63617	72.27232	-2374.20950	-1036.76023	843.75774	1
C	73.17761	70.82605	70.15502	-77.09082	666.87422	-415.84136	1
H	73.83345	71.05261	69.26697	-985.81834	617.19825	2463.91927	1
O	71.83599	71.17519	69.76963	293.29899	-391.65602	-477.80926	1
H	71.99230	71.57066	68.88907	2528.72714	-681.76262	-751.27122	1
C	73.39736	69.33076	70.58796	252.47363	-605.14473	440.05134	1
H	73.10343	68.62375	69.77785	-2052.65063	1456.49051	-1043.51830	1
H	74.46534	69.12102	70.83585	-2152.98800	-1661.97574	-161.70652	1
H	72.79213	69.07288	71.48788	-473.12726	-1584.27339	2165.61725	1
C	75.18469	71.02699	71.92366	360.36307	488.46713	505.10056	1
O	75.24996	70.53216	73.02246	-200.08817	-528.03552	-385.82850	1
N	76.09608	71.10829	70.96764	-597.54095	-226.44775	355.20479	1
H	75.86387	71.56033	70.04642	2179.23655	-1300.70981	-991.33835	1
C	77.44654	70.86514	71.31370	-687.68921	297.39807	-249.45179	1
H	77.39339	70.35052	72.29982	-349.16256	-2467.37713	-1101.72831	1
C	78.07436	69.98932	70.12855	672.47345	361.54328	-201.62880	1
H	77.97833	70.50586	69.14705	1541.41502	582.18425	2169.97026	1
H	79.16081	69.82189	70.30675	-2534.67717	60.25153	997.72295	1
C	77.30877	68.65972	70.10501	574.26774	-389.43119	-377.02542	1
C	77.59513	67.67871	71.10107	688.82679	-287.33580	-257.96133	1
H	78.38062	67.85572	71.85808	331.14182	1470.38163	2269.80013	1
C	76.28643	68.43294	69.13412	-383.52382	-675.04808	144.24917	1
H	76.05555	69.19688	68.36982	4.09847	1517.59662	2262.86397	1
C	76.85212	66.49914	71.10454	-134.76730	428.45285	-649.50303	1
H	77.06884	65.74118	71.87731	-903.23433	1600.49020	2011.53387	1
C	75.57335	67.23273	69.17774	-335.53281	-615.00040	-364.39058	1
H	74.78464	67.06567	68.42345	1596.45398	-1369.33862	1732.02659	1
C	75.82304	66.22740	70.15715	585.48547	-340.24194	-406.23938	1
O	75.09974	65.00785	70.21516	-419.15649	-173.04408	-511.94141	1
H	74.50270	65.13135	69.44003	-1071.84350	1559.71507	-1960.13012	1
C	78.20061	72.32316	71.39626	291.41878	-183.13091	710.72246	1
O	78.43977	72.78145	72.49427	379.87695	523.47396	-222.23622	1
N	78.43796	72.84451	70.20555	564.30591	373.09380	277.24371	1
H	78.07459	72.34586	69.39558	-120.59931	-2436.07824	-1214.35098	1
C	79.20375	74.03554	70.05356	404.07514	-299.37480	608.84009	1
H	78.98862	74.35842	69.00894	-1071.07882	-841.69025	2359.66467	1
C	78.70162	75.07476	71.14767	-683.12807	68.71770	-390.13096	1
H	77.58767	75.08522	71.17355	2068.88918	1596.58590	770.89603	1
H	79.02235	74.75288	72.17207	1379.86429	2342.49779	-179.85638	1
C	79.26445	76.48967	70.82207	-176.39967	537.49466	550.97268	1
H	80.37075	76.52252	70.95823	-396.28051	1942.22168	1869.33314	1
H	79.08390	76.76362	69.75929	-94.44700	586.00678	-2659.20016	1
C	78.51370	77.50260	71.81711	-291.69381	-718.18079	-150.72929	1
H	77.42879	77.61910	71.54717	-2303.38776	1089.76242	964.62032	1
H	78.63053	77.28161	72.90762	884.16729	2568.80533	-207.74283	1
N	79.16129	78.70026	71.51602	-594.49522	-252.81478	342.29276	1
H	79.20825	78.93940	70.53567	-1735.17426	2084.05705	-263.71605	1
C	79.65420	79.52281	72.35672	595.23177	-271.52487	442.22352	1

```
N    79.56892   79.22238   73.71716       585.53813      -424.79750      -105.81303           1
H    79.17015   78.40496   74.17768       959.88797      1327.56823     -2177.11905           1
H    79.91897   79.81715   74.46951      1578.48647        16.27378     -2220.76233           1
N    80.19458   80.63838   71.70350      -452.17763      -390.33541      -421.51853           1
H    80.29781   80.83545   70.70926       470.26107      1974.25629     -1817.92087           1
H    80.68494   81.39056   72.18868      1856.67307     -1573.97321     -1224.35416           1
C    80.78467   73.72488   70.26729      -531.47271      -581.45780       -55.06206           1
O    81.57282   74.64789   70.44542      -243.00837       192.73507       609.52485           1
N    81.06425   72.44088   70.15438       343.49288      -644.25607        38.09164           1
H    80.25455   71.85775   69.95979     -1691.16043     -1535.97525     -1484.73099           1
C    82.43397   71.93363   70.21696       542.66492       350.14337       454.42563           1
H    82.85790   72.49923   71.08106      1209.57979     -2396.95920       463.86744           1
C    82.42682   70.42446   70.37662      -737.14309       145.93010       242.71942           1
H    81.86908   69.99608   69.51352     -1050.18303       159.36419     -2509.06125           1
C    83.83669   69.86142   70.34810      -706.77696      -252.88343      -245.16230           1
H    83.84144   68.73933   70.42272     -1624.64039     -1665.49085      1417.86935           1
H    84.36758   70.13548   69.39225      2566.10521       598.51109       693.22305           1
H    84.45894   70.25480   71.19843     -1972.69758       107.10018     -1876.34312           1
C    81.72238   69.98360   71.64698       412.85148       672.58832       -27.69222           1
H    81.63159   68.86390   71.70541      1447.02277      1762.76533     -1490.78944           1
H    82.27949   70.32067   72.56384      1320.77379      -145.20455      2378.68491           1
H    80.68486   70.41133   71.71726     -1512.10848      1470.52031      1724.75147           1
C    83.15443   72.37751   68.78564      -607.11113       499.56689        73.73769           1
O    83.37007   71.57054   67.91835       357.97126       460.81534       356.68785           1
N    83.41986   73.67325   68.81319       536.55407      -368.62270       332.76306           1
H    83.10614   74.27152   69.61085       524.38866      2297.69985      1367.20941           1
C    84.00861   74.23103   67.65524        46.50277      -774.29190      -147.97904           1
H    84.17388   73.37527   66.95886      1676.51875      1883.29066      1032.55549           1
C    83.01160   75.37243   67.08615      -559.41456       -34.19198      -556.30433           1
H    82.83772   76.13409   67.88318       -64.66664       395.18769      2695.05373           1
H    83.48306   75.91722   66.23393     -2703.44278       -60.40056       333.79418           1
C    81.71196   74.73888   66.67222      -519.76435       377.31045       459.42366           1
H    81.38270   74.05419   67.48725     -1746.74323      1667.50644     -1261.73691           1
C    80.63775   75.80107   66.47332       570.95554        16.46261      -545.27700           1
H    79.65987   75.34980   66.16398      2391.58273        28.05277     -1305.07271           1
H    80.45324   76.38286   67.41310       175.75588       917.63458      2559.43867           1
H    80.92910   76.53616   65.67938     -1063.97851     -2155.34650      1283.00462           1
C    81.87950   73.93130   65.39186       193.76014         2.62946      -765.53141           1
H    80.90739   73.49222   65.04895       507.39394      -846.55087      2539.60089           1
H    82.27168   74.56315   64.55342      1337.57841      1818.19054      1526.01994           1
H    82.59544   73.08020   65.53048      1100.88635     -1172.91350     -2199.08909           1
C    85.36155   74.99622   68.10985      -549.21115       475.64486      -309.38218           1
O    85.54158   75.43436   69.21506       556.06106      -390.37755       -78.24250           1
O    86.28950   75.25206   67.07343      -572.27940      -310.19032      -209.75862           1
H    85.82836   74.76884   66.33214     -1380.08193      2212.14486       790.85944           1
```

Listing 22: C00022.xmolout

```
       276
  150.0000  150.0000  150.0000    0.0000    0.0000    0.0000***********    1.0227     4091
       0.2500    300.00    0.00    0.00    0.00   -1293.9901       0.0000    300.0000
H    69.64461   64.28404   66.57395     -2043.14101     -1757.64893       399.89339           1
N    69.66103   65.29891   66.51328       426.75047       490.45733      -334.42467           1
H    68.82788   65.49263   65.96341       442.25559     -1110.30854     -2448.52907           1
C    70.86071   65.86502   66.03854       415.32972      -666.39213        83.73172           1
H    71.65041   65.28495   66.56193       879.32219      2491.00447       667.35213           1
C    70.97374   65.77580   64.43145        31.35357       477.41777       628.23362           1
H    70.01617   66.10902   63.96394      1168.38489      2240.25337     -1019.71136           1
H    71.74824   66.51068   64.09656      -360.43770      2534.90498       931.67112           1
C    71.33212   64.38703   63.97385       463.18208       512.70242      -382.34388           1
H    70.55640   63.67788   64.33984      1725.29117      1863.52247      -987.07854           1
C    71.36237   64.33381   62.45058       710.42801      -144.27427      -313.15367           1
```

H	71.60904	63.30698	62.07605	364.20195	-1033.13725	2494.72524	1
H	70.37629	64.62349	62.00443	2567.61904	335.80679	847.48802	1
H	72.13229	65.03201	62.03122	-2587.99029	680.92867	-512.16411	1
C	72.68756	63.94920	64.51245	268.17616	741.64379	-40.42634	1
H	72.99435	62.95393	64.09856	1283.54314	-1945.37420	-1411.27812	1
H	73.49258	64.68184	64.24507	-1258.57796	1317.15249	-2026.02085	1
H	72.68450	63.85397	65.62847	-2502.84526	909.80597	575.92391	1
C	70.88749	67.44245	66.37150	619.36578	-458.64843	-172.09273	1
O	71.44419	68.21155	65.59214	-81.40433	433.87611	522.34594	1
N	70.39948	67.75795	67.60796	175.23996	-622.20190	341.55523	1
H	70.02784	66.84382	67.91063	-876.57256	-303.22879	-2561.90213	1
C	69.42582	68.84536	67.60185	-571.88214	-534.10783	-106.15033	1
H	68.71352	68.55183	66.80067	-2713.20841	-16.25800	-248.80809	1
C	68.75238	68.76021	69.03629	166.60354	288.16399	-716.09568	1
H	69.54555	68.86868	69.82786	-2097.85574	1636.78449	-586.18044	1
O	68.09563	67.50593	69.33190	-317.80580	-363.38227	484.43082	1
H	68.59616	67.20782	70.11398	1863.74748	-1451.84923	1357.29467	1
C	67.83832	70.04036	69.03518	503.80731	517.99032	318.51606	1
H	67.37014	70.21082	70.03247	640.61020	-2157.10410	-1536.29104	1
H	68.41441	70.96060	68.77097	1687.88455	465.60692	2087.56452	1
H	67.01237	69.94663	68.29234	-1293.07895	-1899.88581	-1463.57452	1
C	70.08454	70.28543	67.27665	-153.07550	-733.68902	248.70981	1
O	69.34579	71.02990	66.68360	186.15459	-480.78787	-449.34387	1
N	71.27286	70.57163	67.82528	-487.53139	543.18517	42.03850	1
C	72.01228	71.73959	67.43611	-699.35871	69.34393	360.10701	1
H	71.30175	72.42082	66.90924	2.81126	-221.99807	-2715.58072	1
C	72.56222	72.24316	68.84811	-507.46439	-592.59194	122.07761	1
H	73.41652	72.94975	68.75110	-1975.08116	525.87560	-1801.71556	1
H	71.77431	72.76650	69.43372	1588.27743	-1913.22100	1113.83566	1
C	72.95294	70.88182	69.49784	-613.62442	-457.55993	-194.14490	1
H	73.91375	70.50560	69.07945	1707.54637	-721.27992	1996.92523	1
H	73.08906	70.94136	70.60085	758.23357	2100.81767	1560.55011	1
C	71.73024	69.96075	69.06673	-352.41524	704.96076	49.21465	1
H	71.99129	68.90100	68.83590	1714.76886	-2070.42778	443.35792	1
H	70.85828	69.98558	69.76192	1224.15040	1801.50881	1636.97633	1
C	73.31366	71.31970	66.56514	566.85117	537.05831	117.62814	1
O	74.30992	72.04648	66.54715	-550.26779	-406.08867	4.21666	1
N	73.06417	70.23313	65.89256	566.34832	-425.77725	180.18749	1
H	72.13380	69.75369	65.98679	644.44502	2646.80550	-52.73430	1
C	74.09442	69.50694	65.23678	612.34801	-281.48662	411.56221	1
H	73.53731	68.65180	64.78917	1667.23498	-1490.75142	1556.16753	1
C	74.76755	70.45005	64.13081	-714.45746	-175.43074	286.98927	1
H	75.11579	71.40590	64.61778	-787.09484	1763.03619	-1922.46060	1
O	73.89892	70.85536	63.05816	-429.46514	516.51932	-128.40526	1
H	73.84088	71.81883	63.21739	1981.82785	436.16847	-1818.18182	1
C	76.01807	69.59433	63.71339	630.79189	-468.94448	76.03230	1
H	76.64283	70.12738	62.95985	-1775.71861	2062.30353	131.89849	1
H	76.67458	69.36937	64.58658	-1855.14608	88.94118	1993.53760	1
H	75.71456	68.62021	63.26438	-1504.82350	-780.94249	-2132.90992	1
C	75.15288	68.99136	66.38094	288.57638	618.62814	-397.00270	1
O	75.12950	67.82717	66.69232	321.70495	-185.26452	574.37218	1
N	75.97817	69.96103	66.74956	-447.05792	-446.53431	367.76425	1
H	75.76873	70.94504	66.48520	350.31316	1975.63644	-1843.31522	1
C	76.84283	69.73908	67.88032	-511.23061	374.03917	-471.51480	1
H	76.15614	69.59192	68.74378	2234.79579	-898.70890	-1273.45190	1
C	77.74908	68.44366	67.63286	529.53781	-159.34528	563.72604	1
H	78.26490	68.21493	68.60747	654.59388	2347.94251	1217.51488	1
H	77.05204	67.58108	67.42032	-1526.55661	2103.42026	817.87394	1
O	78.80787	68.55878	66.64429	-554.84278	14.12789	399.58769	1
H	78.30354	68.32314	65.84397	1297.06236	2061.84854	1220.68812	1
C	77.76397	71.05291	68.00009	-662.15484	-412.13544	123.62687	1
O	77.88188	71.88211	67.13026	-540.01537	-317.05804	274.91456	1

N	78.41428	70.98247	69.17788	-202.76299	-690.00100	-131.49145	1
H	78.14380	70.21475	69.79402	-702.62132	-2617.37191	281.70413	1
C	79.19745	72.11352	69.67622	-491.73914	576.34187	222.73509	1
H	79.53104	71.76048	70.67991	1977.17978	-1823.58850	-434.68928	1
C	80.34992	72.33606	68.71965	-649.50797	-328.66843	306.11224	1
H	79.92630	72.69287	67.75116	2075.07803	1452.16490	-1004.45904	1
C	81.34712	73.35816	69.23563	-615.18735	-489.99689	-70.96352	1
H	82.23806	73.44376	68.55570	-1226.32661	-1724.38906	-1716.47170	1
H	80.90383	74.38596	69.32253	2381.64273	-969.84735	900.46860	1
H	81.73338	73.07984	70.25378	2122.74991	1702.23946	-141.36228	1
C	81.07945	71.03680	68.41722	-501.86074	-479.61276	-376.42482	1
H	81.91461	71.18786	67.68037	993.79261	-2246.95401	-1177.81305	1
H	81.52898	70.58903	69.34486	42.71279	-626.15391	-2651.37258	1
H	80.38082	70.26959	67.98003	-2026.39012	-1590.33709	-887.82922	1
C	78.05808	73.32426	69.81403	-672.89153	382.57394	-156.34072	1
O	77.10993	73.14637	70.53755	410.04627	221.86223	500.35950	1
N	78.40658	74.41047	69.14559	-378.11782	-443.79414	-441.10984	1
H	79.22716	74.32193	68.55107	2404.15973	-568.14894	1149.30085	1
C	77.53081	75.52861	68.99131	-544.54484	466.16019	331.29119	1
H	78.15377	76.24999	68.41267	-1409.80988	-170.17955	2325.32679	1
C	76.28332	75.00638	68.13734	-184.48284	116.78282	-758.89137	1
H	76.62314	74.32831	67.32155	1893.78641	1861.14652	611.04528	1
H	75.59816	74.39763	68.76976	-2170.79665	-974.19311	1327.50117	1
C	75.56716	76.26727	67.57383	389.51668	672.81878	-138.49152	1
H	75.22081	76.90930	68.42096	510.64577	1933.03327	1851.02484	1
H	76.29859	76.90625	67.03097	-2302.30869	-1105.72939	-948.89774	1
C	74.34695	75.96811	66.62048	-559.93427	-126.34590	542.30857	1
O	73.71990	76.89516	66.17266	-670.36003	-111.83166	76.36448	1
N	74.11501	74.66403	66.37739	-216.06729	-537.07037	446.51379	1
H	74.60668	73.78407	66.67345	-1251.96487	1074.12894	-2168.52500	1
H	73.34779	74.38483	65.74806	-2451.83023	-720.73851	944.84612	1
C	77.04363	76.10967	70.41280	546.24052	-563.88337	-85.11809	1
O	75.98343	76.72160	70.41840	71.70702	-307.40463	606.69673	1
N	77.92768	75.99318	71.39542	479.90863	-504.50129	222.86849	1
C	77.54069	75.83367	72.77409	75.51268	-600.91904	506.73700	1
H	76.60132	75.23333	72.76059	-2033.61562	1795.30103	254.89480	1
C	78.82000	75.12227	73.39678	523.79067	517.99274	284.45581	1
H	78.88098	75.23576	74.50276	1599.61665	-1127.00911	1895.98173	1
H	78.82899	74.03020	73.18254	-1881.83093	1275.82488	-1501.55016	1
C	79.96612	75.86027	72.63810	-600.05639	-404.05026	316.64485	1
H	80.12716	76.88005	73.05491	-2597.05260	496.36221	657.73249	1
H	80.94766	75.33724	72.69126	-1666.24518	1294.38985	-1723.90591	1
C	79.36649	75.91861	71.16790	493.57570	-129.95376	-602.56394	1
H	79.67442	76.79530	70.55128	-1469.08415	-637.84797	2204.22581	1
H	79.50783	74.98383	70.57628	-1038.97840	1068.88701	-2280.71789	1
C	77.36275	77.29013	73.46109	188.88100	241.05089	-727.87821	1
O	77.87763	77.52071	74.52325	531.88613	-49.19943	427.07960	1
N	76.54108	78.07120	72.74427	708.14833	26.93417	-179.73509	1
H	76.16254	77.66541	71.84714	-2232.69501	-1179.09831	1023.94816	1
C	76.74938	79.53508	72.82502	-738.32412	259.92548	104.42456	1
H	77.84849	79.65236	72.96887	2028.89123	1260.02983	1311.33324	1
C	76.24185	80.14490	71.53963	-258.65678	-345.52513	661.28459	1
H	75.21172	79.75957	71.36442	-1826.06090	-1801.46097	-918.64554	1
C	76.17523	81.66000	71.61961	-541.23400	531.92970	-218.41428	1
H	75.74391	82.10894	70.68305	1311.73218	-2219.18200	882.19023	1
H	75.53571	82.00078	72.48281	-1048.71298	1223.58118	-2196.97971	1
H	77.19507	82.11023	71.76866	419.06414	1903.92898	1903.44670	1
C	77.09415	79.74276	70.34774	-423.86431	152.66566	-648.55262	1
H	76.66949	80.13352	69.38277	105.68386	-2342.50080	1387.51238	1
H	78.14254	80.13916	70.43703	-1695.63603	-1484.73621	-1531.02793	1
H	77.17088	78.62398	70.25034	-1853.57121	-487.39031	1936.59343	1
C	75.91951	80.10109	74.12908	539.71428	-510.58232	267.58633	1

```
O    76.09432   81.21943   74.55661      -15.93758      -278.34085      -624.49402         1
N    74.96182   79.22092   74.56661     -676.81886        12.05292       276.17960         1
H    75.20647   78.38104   74.02709    -1694.44250      -373.84189     -2100.66098         1
C    74.97249   79.13325   76.02762      412.33078      -664.70360       108.35451         1
H    76.04173   79.27557   76.29341     1765.77476      1294.44366      1621.76594         1
C    74.03812   80.29275   76.52357     -179.66635      -721.95667      -264.73915         1
H    74.42688   81.27153   76.15270     -607.34922      2587.87032      -598.10018         1
H    73.02350   80.18090   76.07727     -359.69332     -1479.13954     -2259.74260         1
C    73.95597   80.26393   78.08942     -240.01076       616.02425       431.85322         1
H    73.55954   79.28605   78.44494    -1359.17131       782.54425      2227.99181         1
H    74.96275   80.37468   78.54768     -820.36667        85.81767     -2596.78715         1
C    73.00318   81.44849   78.52191     -513.07015       236.18570      -551.87280         1
H    73.40042   82.42563   78.16239     1816.11944     -1183.11722     -1650.94321         1
H    72.00043   81.33942   78.04766    -1800.04429     -1361.53473      1526.34643         1
C    72.87902   81.44229   80.06371      726.08972      -253.19732      -179.64713         1
H    72.40148   80.53530   80.48251     1224.27271      -456.51798     -2390.90304         1
H    73.82037   81.65622   80.61002     1206.40339     -1150.93514     -2154.90344         1
N    71.91862   82.61787   80.44980      329.70796      -633.68968      -155.67358         1
H    71.72839   82.73693   81.48648     1348.31305      1938.76845      1359.00643         1
H    72.23174   83.59676   80.18347     2096.92035      1446.54551      -966.48890         1
H    70.93238   82.59243   80.05736    -1197.07217     -2303.53719       827.28733         1
C    74.40323   77.66730   76.40212     -378.01186      -556.17301      -413.96481         1
O    73.21330   77.47640   76.65002     -409.44433      -307.55053      -453.30833         1
N    75.38829   76.79386   76.36033     -429.02477       524.70884       274.08502         1
H    76.33148   77.10728   76.12548     1706.34263     -1425.96036      1574.38926         1
C    75.09817   75.44633   76.73983      -45.25864      -626.22952       478.93267         1
H    74.08697   75.29441   76.29579     1988.63164       570.90773      1772.87287         1
C    76.24217   74.50701   76.18912      551.77128       -20.49526      -564.54996         1
H    76.39426   74.64303   75.09467     1434.61535     -1753.10253      1513.99471         1
H    77.21751   74.75818   76.66517     -714.67738      1819.09770      1898.36494         1
C    75.80597   73.04338   76.53513      446.99263      -555.77305       338.97221         1
H    75.52934   72.97728   77.61351     1953.68466      1892.25957      -161.67766         1
H    74.89541   72.75451   75.96386     -403.03785      2688.07032      -188.43591         1
C    77.00462   72.06132   76.22372      567.86262       335.74681       434.04421         1
H    77.28628   72.09799   75.14611    -1080.02275      1568.26081     -1948.78900         1
H    77.91779   72.35979   76.78857     -919.54108      2111.34692      1456.13463         1
C    76.54237   70.64334   76.63511      195.81897       173.33862      -745.11536         1
H    76.27251   70.53971   77.70516     1405.67949     -2301.21089      -390.08113         1
H    75.73975   70.21710   76.00080     -940.37328     -1118.32446      2299.72133         1
N    77.75658   69.67270   76.45002      319.26422       656.36007       -42.02424         1
H    77.56866   68.65573   76.68676     1961.03633       619.91968      1787.09422         1
H    78.16838   69.58769   75.47551      854.14343     -2156.80752      1429.08711         1
H    78.61932   69.86537   77.03778     1237.02905     -1378.50857     -1998.28500         1
C    75.04392   75.36630   78.38202      532.53319      -268.57236       517.55729         1
O    75.87053   74.75639   79.00868      266.16714      -290.73804      -558.88002         1
N    73.98301   76.04811   78.79440     -226.25352       640.49678      -270.32839         1
H    73.35695   76.49759   78.08061     2007.69063     -1035.27229      1523.50231         1
C    73.51100   75.87942   80.12614     -540.76542      -573.33303        49.50000         1
H    72.55171   76.44415   80.12160    -2374.20950     -1036.76023       843.75774         1
C    73.26548   74.31220   80.33915      -77.09082       666.87422      -415.84136         1
H    74.27096   73.80557   80.33086     -985.81834       617.19825      2463.91927         1
O    72.47167   73.67093   79.32174      293.29899      -391.65602      -477.80926         1
H    73.12955   73.06412   78.92981     2528.72714      -681.76262      -751.27122         1
C    72.64660   74.24166   81.78185      252.47363      -605.14473       440.05134         1
H    72.50202   73.18618   82.11136    -2052.65063      1456.49051     -1043.51830         1
H    73.30445   74.74883   82.54284    -2152.98800     -1661.97574      -161.70652         1
H    71.64744   74.73555   81.81927     -473.12726     -1584.27339      2165.61725         1
C    74.60571   76.54095   81.13604      360.36307       488.46713       505.10056         1
O    74.70630   76.15366   82.28896     -200.08817      -528.03552      -385.82850         1
N    75.20250   77.58881   80.57943     -597.54095      -226.44775       355.20479         1
H    74.97165   77.79222   79.60911     2179.23655     -1300.70981      -991.33835         1
C    76.03437   78.40826   81.39139     -687.68921       297.39807      -249.45179         1
```

H	76.51299	77.68657	82.09220	-349.16256	-2467.37713	-1101.72831	1
C	77.01037	79.35195	80.55476	672.47345	361.54328	-201.62880	1
H	76.41727	80.15550	80.06163	1541.41502	582.18425	2169.97026	1
H	77.69192	79.87451	81.26564	-2534.67717	60.25153	997.72295	1
C	77.82393	78.58755	79.49593	574.26774	-389.43119	-377.02542	1
C	78.67176	77.49488	79.84413	688.82679	-287.33580	-257.96133	1
H	78.74697	77.14912	80.89120	331.14182	1470.38163	2269.80013	1
C	77.74196	79.04747	78.14840	-383.52382	-675.04808	144.24917	1
H	77.11015	79.91481	77.88441	4.09847	1517.59662	2262.86397	1
C	79.40002	76.86982	78.83062	-134.76730	428.45285	-649.50303	1
H	80.05506	76.02152	79.09534	-903.23433	1600.49020	2011.53387	1
C	78.48768	78.38545	77.17126	-335.53281	-615.00040	-364.39058	1
H	78.41591	78.74705	76.12323	1596.45398	-1369.33862	1732.02659	1
C	79.32894	77.27388	77.46669	585.48547	-340.24194	-406.23938	1
O	80.08134	76.60462	76.46698	-419.15649	-173.04408	-511.94141	1
H	79.65692	76.99318	75.65822	-1071.84350	1559.71507	-1960.13012	1
C	75.13168	79.53726	82.14567	291.41878	-183.13091	710.72246	1
O	75.54098	80.66989	82.27432	379.87695	523.47396	-222.23622	1
N	73.96419	79.02272	82.54154	564.30591	373.09380	277.24371	1
H	73.82560	78.01479	82.39882	-120.59931	-2436.07824	-1214.35098	1
C	73.22728	79.73061	83.53701	404.07514	-299.37480	608.84009	1
H	73.59455	80.78010	83.44656	-1071.07882	-841.69025	2359.66467	1
C	71.70137	79.51386	83.17856	-683.12807	68.71770	-390.13096	1
H	71.51075	79.77516	82.11409	2068.88918	1596.58590	770.89603	1
H	71.42743	78.43925	83.27579	1379.86429	2342.49779	-179.85638	1
C	70.82850	80.39375	84.12531	-176.39967	537.49466	550.97268	1
H	70.94677	80.07859	85.18927	-396.28051	1942.22168	1869.33314	1
H	71.12338	81.46614	84.08375	-94.44700	586.00678	-2659.20016	1
C	69.34098	80.16596	83.58802	-291.69381	-718.18079	-150.72929	1
H	69.16056	80.48474	82.53122	-2303.38776	1089.76242	964.62032	1
H	68.95405	79.12062	83.69492	884.16729	2568.80533	-207.74283	1
N	68.54725	80.99144	84.39159	-594.49522	-252.81478	342.29276	1
H	69.01201	81.48846	85.13523	-1735.17426	2084.05705	-263.71605	1
C	67.29297	81.13738	84.24085	595.23177	-271.52487	442.22352	1
N	66.68127	80.42751	83.20079	585.53813	-424.79750	-105.81303	1
H	67.09160	79.78054	82.52859	959.88797	1327.56823	-2177.11905	1
H	65.68479	80.45295	82.98367	1578.48647	16.27378	-2220.76233	1
N	66.76592	82.02096	85.19260	-452.17763	-390.33541	-421.51853	1
H	67.21348	82.52883	85.95417	470.26107	1974.25629	-1817.92087	1
H	65.77488	82.26108	85.24092	1856.67307	-1573.97321	-1224.35416	1
C	73.57462	79.04202	84.98220	-531.47271	-581.45780	-55.06206	1
O	72.70759	78.74039	85.76808	-243.00837	192.73507	609.52485	1
N	74.89761	78.95663	85.14015	343.49288	-644.25607	38.09164	1
H	75.43407	79.23760	84.31785	-1691.16043	-1535.97525	-1484.73099	1
C	75.49301	78.13315	86.19206	542.66492	350.14337	454.42563	1
H	74.95201	78.42002	87.12424	1209.57979	-2396.95920	463.86744	1
C	75.31635	76.66493	85.84676	-737.14309	145.93010	242.71942	1
H	75.88326	76.45991	84.91051	-1050.18303	159.36419	-2509.06125	1
C	75.87081	75.78003	86.94804	-706.77696	-252.88343	-245.16230	1
H	75.79698	74.68886	86.68579	-1624.64039	-1665.49085	1417.86935	1
H	76.95692	76.00143	87.15653	2566.10521	598.51109	693.22305	1
H	75.31602	75.92686	87.91558	-1972.69758	107.10018	-1876.34312	1
C	73.86214	76.31950	85.57883	412.85148	672.58832	-27.69222	1
H	73.71501	75.22057	85.38791	1447.02277	1762.76533	-1490.78944	1
H	73.20289	76.61308	86.44494	1320.77379	-145.20455	2378.68491	1
H	73.47183	76.87843	84.68052	-1512.10848	1470.52031	1724.75147	1
C	77.11042	78.51463	86.17056	-607.11113	499.56689	73.73769	1
O	77.72717	78.61090	85.13830	357.97126	460.81534	356.68785	1
N	77.54411	78.63236	87.41893	536.55407	-368.62270	332.76306	1
H	76.88310	78.45466	88.17363	524.38866	2297.69985	1367.20941	1
C	78.94108	78.67817	87.64550	46.50277	-774.29190	-147.97904	1
H	79.38965	78.84742	86.63937	1676.51875	1883.29066	1032.55549	1

```
C    79.26801  79.83626  88.72399     -559.41456      -34.19198     -556.30433          1
H    78.88806  79.52345  89.72553      -64.66664      395.18769     2695.05373          1
H    80.37055  79.96054  88.84393    -2703.44278      -60.40056      333.79418          1
C    78.61989  81.12703  88.30175     -519.76435      377.31045      459.42366          1
H    77.54130  80.92707  88.10880    -1746.74323     1667.50644    -1261.73691          1
C    78.72981  82.16426  89.41260      570.95554       16.46261     -545.27700          1
H    78.22387  83.12439  89.13411     2391.58273       28.05277    -1305.07271          1
H    78.25918  81.80417  90.36360      175.75588      917.63458     2559.43867          1
H    79.79851  82.40817  89.64414    -1063.97851    -2155.34650     1283.00462          1
C    79.24076  81.67396  87.02356      193.76014        2.62946     -765.53141          1
H    78.77515  82.64796  86.72262      507.39394     -846.55087     2539.60089          1
H    80.34043  81.85410  87.14152     1337.57841     1818.19054     1526.01994          1
H    79.10703  80.96528  86.16515     1100.88635    -1172.91350    -2199.08909          1
C    79.36109  77.27264  88.32042     -549.21115      475.64486     -309.38218          1
O    78.56928  76.45586  88.71288      556.06106     -390.37755      -78.24250          1
O    80.75049  77.06789  88.49343     -572.27940     -310.19032     -209.75862          1
H    81.08234  77.90702  88.06790    -1380.08193     2212.14486      790.85944          1
```

Listing 23: C00023.xmolout

```
       276
  150.0000  150.0000  150.0000     0.0000     0.0000     0.0000***********    2.7750    11100
       0.2500     300.00    0.00    0.00    0.00   -1293.9865       0.0000     300.0000
H    70.24329  64.92098  78.43268    -2043.14101    -1757.64893      399.89339          1
N    70.07379  65.33491  77.51672      426.75047      490.45733     -334.42467          1
H    69.12054  65.69838  77.61394      442.25559    -1110.30854    -2448.52907          1
C    70.99129  66.35360  77.16564      415.32972     -666.39213       83.73172          1
H    71.78995  66.29215  77.93732      879.32219     2491.00447      667.35213          1
C    71.56401  66.08885  75.67739       31.35357      477.41777      628.23362          1
H    70.73310  66.19372  74.94031     1168.38489     2240.25337    -1019.71136          1
H    72.32057  66.86010  75.40117     -360.43770     2534.90498      931.67112          1
C    72.15519  64.70810  75.61729      463.18208      512.70242     -382.34388          1
H    71.42469  64.00720  76.08191     1725.29117     1863.52247     -987.07854          1
C    72.39302  64.27567  74.17538      710.42801     -144.27427     -313.15367          1
H    72.68189  63.19530  74.11206      364.20195    -1033.13725     2494.72524          1
H    71.47779  64.41290  73.54397     2567.61904      335.80679      847.48802          1
H    73.21928  64.86245  73.69859    -2587.99029      680.92867     -512.16411          1
C    73.47079  64.63936  76.38257      268.17616      741.64379      -40.42634          1
H    73.89520  63.60262  76.38268     1283.54314    -1945.37420    -1411.27812          1
H    74.24225  65.31298  75.92839    -1258.57796     1317.15249    -2026.02085          1
H    73.34675  64.94834  77.45217    -2502.84526      909.80597      575.92391          1
C    70.12237  67.71303  77.22188      619.36578     -458.64843     -172.09273          1
O    68.98516  67.74802  77.71898      -81.40433      433.87611      522.34594          1
N    70.86590  68.69376  76.78698      175.23996     -622.20190      341.55523          1
H    71.77524  68.42529  76.41395     -876.57256     -303.22879    -2561.90213          1
C    70.38060  70.00387  76.44592     -571.88214     -534.10783     -106.15033          1
H    70.18069  69.95912  75.35282    -2713.20841      -16.25800     -248.80809          1
C    69.05478  70.37017  77.25306      166.60354      288.16399     -716.09568          1
H    69.15084  69.94716  78.29627    -2097.85574     1636.78449     -586.18044          1
O    67.85404  69.76936  76.76141     -317.80580     -363.38227      484.43082          1
H    67.90371  68.86881  77.26648     1863.74748    -1451.84923     1357.29467          1
C    69.07887  71.94127  77.28066      503.80731      517.99032      318.51606          1
H    68.24453  72.34898  77.89747      640.61020    -2157.10410    -1536.29104          1
H    70.03004  72.34042  77.70346     1687.88455      465.60692     2087.56452          1
H    68.96849  72.36639  76.25602    -1293.07895    -1899.88581    -1463.57452          1
C    71.70914  70.90449  76.68358     -153.07550     -733.68902      248.70981          1
O    72.28285  71.21223  75.65124      186.15459     -480.78787     -449.34387          1
N    72.07962  71.10282  77.94757     -487.53139      543.18517       42.03850          1
C    73.19827  71.91992  78.32865     -699.35871       69.34393      360.10701          1
H    73.13912  72.85827  77.73327        2.81126     -221.99807    -2715.58072          1
C    73.00268  72.07988  79.89402     -507.46439     -592.59194      122.07761          1
H    73.94872  72.34854  80.41699    -1975.08116      525.87560    -1801.71556          1
```

H	72.25514	72.86529	80.14547	1588.27743	-1913.22100	1113.83566	1
C	72.48280	70.65365	80.25346	-613.62442	-457.55993	-194.14490	1
H	73.30471	69.90222	80.21072	1707.54637	-721.27992	1996.92523	1
H	72.02806	70.58032	81.26689	758.23357	2100.81767	1560.55011	1
C	71.43763	70.43719	79.07549	-352.41524	704.96076	49.21465	1
H	71.24368	69.37599	78.79390	1714.76886	-2070.42778	443.35792	1
H	70.46776	70.96297	79.23757	1224.15040	1801.50881	1636.97633	1
C	74.61496	71.12903	78.12453	566.85117	537.05831	117.62814	1
O	75.56252	71.37041	78.83570	-550.26779	-406.08867	4.21666	1
N	74.55054	70.34321	77.05887	566.34832	-425.77725	180.18749	1
H	73.67340	70.30898	76.51410	644.44502	2646.80550	-52.73430	1
C	75.48815	69.27576	76.94015	612.34801	-281.48662	411.56221	1
H	76.26285	69.53574	77.69926	1667.23498	-1490.75142	1556.16753	1
C	74.70168	67.92356	77.29390	-714.45746	-175.43074	286.98927	1
H	74.02434	67.67615	76.43180	-787.09484	1763.03619	-1922.46060	1
O	73.84816	67.97101	78.45171	-429.46514	516.51932	-128.40526	1
H	72.96704	68.05112	78.02953	1981.82785	436.16847	-1818.18182	1
C	75.87904	66.88472	77.36153	630.79189	-468.94448	76.03230	1
H	75.51229	65.85927	77.59837	-1775.71861	2062.30353	131.89849	1
H	76.43601	66.82517	76.38662	-1855.14608	88.94118	1993.53760	1
H	76.61836	67.15534	78.15146	-1504.82350	-780.94249	-2132.90992	1
C	76.03079	69.26925	75.40503	288.57638	618.62814	-397.00270	1
O	76.36221	68.19854	74.87810	321.70495	-185.26452	574.37218	1
N	76.10414	70.48315	74.92130	-447.05792	-446.53431	367.76425	1
H	75.78880	71.18727	75.59024	350.31316	1975.63644	-1843.31522	1
C	76.64236	70.87178	73.66966	-511.23061	374.03917	-471.51480	1
H	76.53773	71.98261	73.71600	2234.79579	-898.70890	-1273.45190	1
C	78.17421	70.41593	73.64141	529.53781	-159.34528	563.72604	1
H	78.71571	70.95519	72.81591	654.59388	2347.94251	1217.51488	1
H	78.62636	70.79795	74.59670	-1526.55661	2103.42026	817.87394	1
O	78.46986	69.01845	73.49019	-554.84278	14.12789	399.58769	1
H	77.67048	68.62234	74.00074	1297.06236	2061.84854	1220.68812	1
C	75.77229	70.25700	72.44379	-662.15484	-412.13544	123.62687	1
O	76.26956	70.07908	71.35753	-540.01537	-317.05804	274.91456	1
N	74.48822	70.10631	72.78950	-202.76299	-690.00100	-131.49145	1
H	74.26199	70.34202	73.75562	-702.62132	-2617.37191	281.70413	1
C	73.50364	69.71302	71.78247	-491.73914	576.34187	222.73509	1
H	74.15847	69.28373	70.98440	1977.17978	-1823.58850	-434.68928	1
C	72.55775	68.71376	72.42217	-649.50797	-328.66843	306.11224	1
H	72.08254	69.18869	73.30861	2075.07803	1452.16490	-1004.45904	1
C	71.46546	68.29121	71.45671	-615.18735	-489.99689	-70.96352	1
H	70.74164	67.57436	71.93323	-1226.32661	-1724.38906	-1716.47170	1
H	70.87285	69.17344	71.08828	2381.64273	-969.84735	900.46860	1
H	71.89283	67.77935	70.55065	2122.74991	1702.23946	-141.36228	1
C	73.30903	67.49176	72.92180	-501.86074	-479.61276	-376.42482	1
H	72.62045	66.76293	73.42718	993.79261	-2246.95401	-1177.81305	1
H	73.81339	66.94438	72.07860	42.71279	-626.15391	-2651.37258	1
H	74.11386	67.77257	73.65789	-2026.39012	-1590.33709	-887.82922	1
C	72.78502	71.11979	71.25332	-672.89153	382.57394	-156.34072	1
O	73.26980	71.69321	70.30739	410.04627	221.86223	500.35950	1
N	71.74779	71.49083	71.98458	-378.11782	-443.79414	-441.10984	1
H	71.46298	70.89139	72.75571	2404.15973	-568.14894	1149.30085	1
C	70.93106	72.55363	71.47926	-544.54484	466.16019	331.29119	1
H	71.26938	72.65329	70.41958	-1409.80988	-170.17955	2325.32679	1
C	69.43523	72.02773	71.62749	-184.48284	116.78282	-758.89137	1
H	69.36572	70.96830	71.29373	1893.78641	1861.14652	611.04528	1
H	69.11663	72.03019	72.69415	-2170.79665	-974.19311	1327.50117	1
C	68.51187	72.94127	70.76706	389.51668	672.81878	-138.49152	1
H	68.55237	73.98984	71.14445	510.64577	1933.03327	1851.02484	1
H	68.90772	73.01671	69.72993	-2302.30869	-1105.72939	-948.89774	1
C	67.01730	72.44374	70.71533	-559.93427	-126.34590	542.30857	1
O	66.20754	73.07399	70.08623	-670.36003	-111.83166	76.36448	1

```
N    66.78536  71.30231  71.39855     -216.06729     -537.07037      446.51379          1
H    67.42162  70.73704  71.97268    -1251.96487     1074.12894    -2168.52500          1
H    65.86289  70.85983  71.47615    -2451.83023     -720.73851      944.84612          1
C    71.06794  73.96610  72.26131      546.24052     -563.88337      -85.11809          1
O    70.56050  74.92741  71.69798       71.70702     -307.40463      606.69673          1
N    71.72774  73.95673  73.41975      479.90863     -504.50129      222.86849          1
C    71.38315  74.81378  74.51774       75.51268     -600.91904      506.73700          1
H    70.27060  74.85466  74.55186    -2033.61562     1795.30103      254.89480          1
C    72.10344  74.12527  75.74812      523.79067      517.99274      284.45581          1
H    72.28442  74.83820  76.58747     1599.61665    -1127.00911     1895.98173          1
H    71.49869  73.28464  76.15866    -1881.83093     1275.82488    -1501.55016          1
C    73.42036  73.61366  75.07810     -600.05639     -404.05026      316.64485          1
H    74.16821  74.43878  74.97997    -2597.05260      496.36221      657.73249          1
H    73.92832  72.79684  75.64648    -1666.24518     1294.38985    -1723.90591          1
C    72.91290  73.13897  73.65147      493.57570     -129.95376     -602.56394          1
H    73.61755  73.30952  72.80033    -1469.08415     -637.84797     2204.22581          1
H    72.55728  72.08040  73.62247    -1038.97840     1068.88701    -2280.71789          1
C    72.07572  76.29720  74.41280      188.88100      241.05089     -727.87821          1
O    72.47179  76.80381  75.43611      531.88613      -49.19943      427.07960          1
N    71.97693  76.78549  73.19174      708.14833       26.93417     -179.73509          1
H    71.47117  76.21775  72.47123    -2232.69501    -1179.09831     1023.94816          1
C    72.71233  77.92562  72.77670     -738.32412      259.92548      104.42456          1
H    72.21889  78.22640  71.82637     2028.89123     1260.02983     1311.33324          1
C    72.64399  79.03427  73.91233     -258.65678     -345.52513      661.28459          1
H    73.09883  78.65837  74.86574    -1826.06090    -1801.46097     -918.64554          1
C    73.36658  80.33506  73.42265     -541.23400      531.92970     -218.41428          1
H    73.15111  81.19422  74.10080     1311.73218    -2219.18200      882.19023          1
H    74.48967  80.21214  73.38891    -1048.71298     1223.58118    -2196.97971          1
H    73.03017  80.63121  72.40119      419.06414     1903.92898     1903.44670          1
C    71.13753  79.41181  74.13081     -423.86431      152.66566     -648.55262          1
H    71.01877  80.20759  74.90308      105.68386    -2342.50080     1387.51238          1
H    70.66602  79.78431  73.19148    -1695.63603    -1484.73621    -1531.02793          1
H    70.54865  78.52729  74.47022    -1853.57121     -487.39031     1936.59343          1
C    74.28472  77.50825  72.54699      539.71428     -510.58232      267.58633          1
O    74.97464  76.97899  73.38273      -15.93758     -278.34085     -624.49402          1
N    74.66755  78.01976  71.36198     -676.81886       12.05292      276.17960          1
H    73.88272  78.36577  70.80923    -1694.44250     -373.84189    -2100.66098          1
C    75.72380  77.28734  70.68821      412.33078     -664.70360      108.35451          1
H    75.43331  76.21452  70.72528     1765.77476     1294.44366     1621.76594          1
C    75.78682  77.88433  69.23445     -179.66635     -721.95667     -264.73915          1
H    74.86451  77.64475  68.65851     -607.34922     2587.87032     -598.10018          1
H    75.82694  78.99592  69.28533     -359.69332    -1479.13954    -2259.74260          1
C    77.06128  77.32654  68.51232     -240.01076      616.02425      431.85322          1
H    77.95630  77.43488  69.16693    -1359.17131      782.54425     2227.99181          1
H    76.96441  76.23765  68.30397     -820.36667       85.81767    -2596.78715          1
C    77.26033  78.15485  67.18105     -513.07015      236.18570     -551.87280          1
H    76.44531  77.94343  66.45111     1816.11944    -1183.11722    -1650.94321          1
H    77.20933  79.24933  67.38448    -1800.04429    -1361.53473     1526.34643          1
C    78.64453  77.77900  66.60197      726.08972     -253.19732     -179.64713          1
H    79.50073  78.02060  67.26318     1224.27271     -456.51798    -2390.90304          1
H    78.72764  76.73417  66.24223     1206.40339    -1150.93514    -2154.90344          1
N    78.87532  78.66266  65.33023      329.70796     -633.68968     -155.67358          1
H    79.79993  78.51725  64.83105     1348.31305     1938.76845     1359.00643          1
H    78.18673  78.54659  64.53100     2096.92035     1446.54551     -966.48890          1
H    78.87615  79.71533  65.46778    -1197.07217    -2303.53719      827.28733          1
C    77.13393  77.57193  71.44559     -378.01186     -556.17301     -413.96481          1
O    78.03859  76.77193  71.35218     -409.44433     -307.55053     -453.30833          1
N    77.15561  78.81412  71.92035     -429.02477      524.70884      274.08502          1
H    76.25746  79.30193  71.95656     1706.34263    -1425.96036     1574.38926          1
C    78.19051  79.23954  72.83018      -45.25864     -626.22952      478.93267          1
H    78.18061  78.43082  73.59410     1988.63164      570.90773     1772.87287          1
C    79.55651  79.28661  72.04278      551.77128      -20.49526     -564.54996          1
```

H	79.62210	78.41621	71.34963	1434.61535	-1753.10253	1513.99471	1
H	79.58769	80.18507	71.38590	-714.67738	1819.09770	1898.36494	1
C	80.77273	79.28353	73.03131	446.99263	-555.77305	338.97221	1
H	80.71613	80.11698	73.76786	1953.68466	1892.25957	-161.67766	1
H	80.79818	78.35377	73.64326	-403.03785	2688.07032	-188.43591	1
C	82.06848	79.41792	72.13516	567.86262	335.74681	434.04421	1
H	82.17530	78.53980	71.45747	-1080.02275	1568.26081	-1948.78900	1
H	81.99520	80.30720	71.46762	-919.54108	2111.34692	1456.13463	1
C	83.29083	79.54571	73.07346	195.81897	173.33862	-745.11536	1
H	83.27335	80.43537	73.73419	1405.67949	-2301.21089	-390.08113	1
H	83.51498	78.63772	73.66792	-940.37328	-1118.32446	2299.72133	1
N	84.55281	79.75708	72.16980	319.26422	656.36007	-42.02424	1
H	85.47130	79.87461	72.68711	1961.03633	619.91968	1787.09422	1
H	84.79618	78.98074	71.48820	854.14343	-2156.80752	1429.08711	1
H	84.55930	80.61180	71.54014	1237.02905	-1378.50857	-1998.28500	1
C	77.61845	80.65651	73.36714	532.53319	-268.57236	517.55729	1
O	76.41439	80.77165	73.57416	266.16714	-290.73804	-558.88002	1
N	78.58798	81.52244	73.58916	-226.25352	640.49678	-270.32839	1
H	79.52739	81.23059	73.32045	2007.69063	-1035.27229	1523.50231	1
C	78.39345	82.89438	73.89280	-540.76542	-573.33303	49.50000	1
H	79.40614	83.33486	73.75290	-2374.20950	-1036.76023	843.75774	1
C	77.35724	83.51504	72.83360	-77.09082	666.87422	-415.84136	1
H	76.45765	82.84317	72.74411	-985.81834	617.19825	2463.91927	1
O	77.88901	83.62891	71.50300	293.29899	-391.65602	-477.80926	1
H	77.29648	83.02316	71.01238	2528.72714	-681.76262	-751.27122	1
C	76.92104	84.85581	73.52607	252.47363	-605.14473	440.05134	1
H	76.37484	85.52264	72.81845	-2052.65063	1456.49051	-1043.51830	1
H	76.24048	84.67440	74.39401	-2152.98800	-1661.97574	-161.70652	1
H	77.80171	85.42198	73.91718	-473.12726	-1584.27339	2165.61725	1
C	77.90458	83.08947	75.43530	360.36307	488.46713	505.10056	1
O	77.90329	84.21902	75.88101	-200.08817	-528.03552	-385.82850	1
N	77.58096	81.95977	76.06293	-597.54095	-226.44775	355.20479	1
H	77.59795	81.10647	75.50133	2179.23655	-1300.70981	-991.33835	1
C	76.89469	82.09466	77.29703	-687.68921	297.39807	-249.45179	1
H	76.91422	83.19187	77.50539	-349.16256	-2467.37713	-1101.72831	1
C	75.41989	81.49900	77.08691	672.47345	361.54328	-201.62880	1
H	75.45546	80.58142	76.45469	1541.41502	582.18425	2169.97026	1
H	74.97012	81.21504	78.06559	-2534.67717	60.25153	997.72295	1
C	74.59761	82.60532	76.41341	574.26774	-389.43119	-377.02542	1
C	73.82174	83.51416	77.19430	688.82679	-287.33580	-257.96133	1
H	73.77936	83.42090	78.29458	331.14182	1470.38163	2269.80013	1
C	74.66568	82.74998	74.99636	-383.52382	-675.04808	144.24917	1
H	75.30431	82.04244	74.39319	4.09847	1517.59662	2262.86397	1
C	73.12642	84.52738	76.53629	-134.76730	428.45285	-649.50303	1
H	72.52771	85.23222	77.13908	-903.23433	1600.49020	2011.53387	1
C	73.94797	83.77715	74.38104	-335.53281	-615.00040	-364.39058	1
H	74.00946	83.87391	73.28288	1596.45398	-1369.33862	1732.02659	1
C	73.15489	84.70110	75.12185	585.48547	-340.24194	-406.23938	1
O	72.42231	85.75167	74.51129	-419.15649	-173.04408	-511.94141	1
H	72.66807	85.59940	73.56811	-1071.84350	1559.71507	-1960.13012	1
C	77.58164	81.21488	78.49064	291.41878	-183.13091	710.72246	1
O	77.66521	81.70999	79.59450	379.87695	523.47396	-222.23622	1
N	77.91781	79.97656	78.14764	564.30591	373.09380	277.24371	1
H	77.74842	79.63016	77.20434	-120.59931	-2436.07824	-1214.35098	1
C	78.41449	79.17086	79.23238	404.07514	-299.37480	608.84009	1
H	79.10176	79.90313	79.71574	-1071.07882	-841.69025	2359.66467	1
C	77.18774	78.87732	80.19081	-683.12807	68.71770	-390.13096	1
H	76.69550	79.83718	80.47625	2068.88918	1596.58590	770.89603	1
H	76.40056	78.28795	79.66861	1379.86429	2342.49779	-179.85638	1
C	77.71967	78.13020	81.45399	-176.39967	537.49466	550.97268	1
H	78.28927	77.22020	81.16375	-396.28051	1942.22168	1869.33314	1
H	78.44459	78.76438	82.01053	-94.44700	586.00678	-2659.20016	1

```
C    76.47717  77.74375  82.38008      -291.69381      -718.18079      -150.72929          1
H    75.96316  78.59544  82.89411     -2303.38776      1089.76242       964.62032          1
H    75.69249  77.11138  81.89152       884.16729      2568.80533      -207.74283          1
N    77.10819  76.95599  83.35656      -594.49522      -252.81478       342.29276          1
H    78.08526  76.75187  83.21389     -1735.17426      2084.05705      -263.71605          1
C    76.54106  76.48026  84.38982       595.23177      -271.52487       442.22352          1
N    75.18382  76.76062  84.58187       585.53813      -424.79750      -105.81303          1
H    74.55032  77.31815  84.01069       959.88797      1327.56823     -2177.11905          1
H    74.62972  76.44125  85.37717      1578.48647        16.27378     -2220.76233          1
N    77.44329  75.73018  85.15858      -452.17763      -390.33541      -421.51853          1
H    78.43226  75.52678  85.02292       470.26107      1974.25629     -1817.92087          1
H    77.18176  75.26763  86.02991      1856.67307     -1573.97321     -1224.35416          1
C    79.17418  77.87409  78.60344      -531.47271      -581.45780       -55.06206          1
O    80.23589  77.99209  78.04494      -243.00837       192.73507       609.52485          1
N    78.53116  76.72306  78.91699       343.49288      -644.25607        38.09164          1
H    77.60065  76.92423  79.28879     -1691.16043     -1535.97525     -1484.73099          1
C    78.69310  75.54654  78.05065       542.66492       350.14337       454.42563          1
H    79.79585  75.42114  77.94535      1209.57979     -2396.95920       463.86744          1
C    78.01208  74.40773  78.78036      -737.14309       145.93010       242.71942          1
H    76.97128  74.72821  79.01393     -1050.18303       159.36419     -2509.06125          1
C    77.92536  73.13586  77.96195      -706.77696      -252.88343      -245.16230          1
H    77.40738  72.30923  78.53869     -1624.64039     -1665.49085      1417.86935          1
H    77.31827  73.29684  77.00635      2566.10521       598.51109       693.22305          1
H    78.94187  72.75620  77.66408     -1972.69758       107.10018     -1876.34312          1
C    78.70314  74.10601  80.10054       412.85148       672.58832       -27.69222          1
H    78.17242  73.29672  80.67294      1447.02277      1762.76533     -1490.78944          1
H    79.76222  73.76310  79.94281      1320.77379      -145.20455      2378.68491          1
H    78.74467  75.01438  80.76129     -1512.10848      1470.52031      1724.75147          1
C    77.98903  75.93980  76.60338      -607.11113       499.56689        73.73769          1
O    77.24855  76.88332  76.46621       357.97126       460.81534       356.68785          1
N    78.39246  75.07254  75.68239       536.55407      -368.62270       332.76306          1
H    78.97396  74.30205  76.00399       524.38866      2297.69985      1367.20941          1
C    77.73407  75.09207  74.42040        46.50277      -774.29190      -147.97904          1
H    77.48078  76.16766  74.26611      1676.51875      1883.29066      1032.55549          1
C    78.67972  74.42096  73.29533      -559.41456       -34.19198      -556.30433          1
H    78.56887  73.31246  73.36208       -64.66664       395.18769      2695.05373          1
H    78.30799  74.69945  72.27725     -2703.44278       -60.40056       333.79418          1
C    80.13082  74.78961  73.44907      -519.76435       377.31045       459.42366          1
H    80.40451  74.75263  74.52712     -1746.74323      1667.50644     -1261.73691          1
C    81.01048  73.79810  72.69666       570.95554        16.46261      -545.27700          1
H    82.09698  74.05594  72.78857      2391.58273        28.05277     -1305.07271          1
H    80.88309  72.75374  73.08221       175.75588       917.63458      2559.43867          1
H    80.76575  73.77909  71.60347     -1063.97851     -2155.34650      1283.00462          1
C    80.38721  76.19680  72.92909       193.76014         2.62946      -765.53141          1
H    81.47675  76.46099  72.95303       507.39394      -846.55087      2539.60089          1
H    80.03253  76.30888  71.86526      1337.57841      1818.19054      1526.01994          1
H    79.83759  76.96147  73.53991      1100.88635     -1172.91350     -2199.08909          1
C    76.43975  74.15178  74.50348      -549.21115       475.64486      -309.38218          1
O    76.16847  73.43530  75.44172       556.06106      -390.37755       -78.24250          1
O    75.68062  74.06053  73.31152      -572.27940      -310.19032      -209.75862          1
H    75.91943  74.95593  72.92598     -1380.08193      2212.14486       790.85944          1
```

Listing 24: C00024.xmolout

```
       276
  150.0000  150.0000  150.0000      0.0000      0.0000      0.0000***********    2.7750    11100
        0.2500     300.00    0.00    0.00    0.00  -1293.9706        0.0000     300.0000
H    71.43720  78.94080  86.11812     -2043.14101     -1757.64893       399.89339          1
N    71.38973  79.41802  85.21883       426.75047       490.45733      -334.42467          1
H    70.43836  79.78301  85.25721       442.25559     -1110.30854     -2448.52907          1
C    72.36730  80.43794  85.09025       415.32972      -666.39213        83.73172          1
H    72.45879  80.90359  86.09531       879.32219      2491.00447       667.35213          1
```

C	73.74606	79.76737	84.60098	31.35357	477.41777	628.23362	1
H	73.56990	79.25612	83.62384	1168.38489	2240.25337	-1019.71136	1
H	74.50401	80.56287	84.40071	-360.43770	2534.90498	931.67112	1
C	74.26231	78.78423	85.62231	463.18208	512.70242	-382.34388	1
H	73.43357	78.09638	85.90197	1725.29117	1863.52247	-987.07854	1
C	75.39268	77.95360	85.02796	710.42801	-144.27427	-313.15367	1
H	75.78599	77.20381	85.76220	364.20195	-1033.13725	2494.72524	1
H	75.05788	77.38733	84.12057	2567.61904	335.80679	847.48802	1
H	76.25609	78.59704	84.71726	-2587.99029	680.92867	-512.16411	1
C	74.75576	79.49445	86.87492	268.17616	741.64379	-40.42634	1
H	75.16067	78.77027	87.62849	1283.54314	-1945.37420	-1411.27812	1
H	75.57678	80.22028	86.63932	-1258.57796	1317.15249	-2026.02085	1
H	73.93759	80.07222	87.37696	-2502.84526	909.80597	575.92391	1
C	71.89961	81.49962	83.95023	619.36578	-458.64843	-172.09273	1
O	72.32623	82.63380	83.95762	-81.40433	433.87611	522.34594	1
N	71.14598	80.91290	83.01710	175.23996	-622.20190	341.55523	1
H	70.85897	79.95138	83.19463	-876.57256	-303.22879	-2561.90213	1
C	70.51891	81.74050	82.04268	-571.88214	-534.10783	-106.15033	1
H	71.09741	82.69329	82.03688	-2713.20841	-16.25800	-248.80809	1
C	70.55026	80.90531	80.66796	166.60354	288.16399	-716.09568	1
H	70.04401	79.91601	80.85869	-2097.85574	1636.78449	-586.18044	1
O	71.85733	80.59563	80.16051	-317.80580	-363.38227	484.43082	1
H	71.82569	79.61728	80.14820	1863.74748	-1451.84923	1357.29467	1
C	69.63464	81.75577	79.71460	503.80731	517.99032	318.51606	1
H	69.42327	81.20718	78.76733	640.61020	-2157.10410	-1536.29104	1
H	68.64992	81.99493	80.18245	1687.88455	465.60692	2087.56452	1
H	70.11728	82.72092	79.43645	-1293.07895	-1899.88581	-1463.57452	1
C	68.96339	81.88184	82.48361	-153.07550	-733.68902	248.70981	1
O	68.33318	80.87188	82.75722	186.15459	-480.78787	-449.34387	1
N	68.53583	83.13434	82.37997	-487.53139	543.18517	42.03850	1
C	67.20436	83.51347	82.78723	-699.35871	69.34393	360.10701	1
H	67.03694	82.99484	83.75995	2.81126	-221.99807	-2715.58072	1
C	67.26527	85.10038	82.83889	-507.46439	-592.59194	122.07761	1
H	66.27203	85.56085	82.62374	-1975.08116	525.87560	-1801.71556	1
H	67.58624	85.47100	83.83823	1588.27743	-1913.22100	1113.83566	1
C	68.33018	85.41756	81.74312	-613.62442	-457.55993	-194.14490	1
H	67.88087	85.35530	80.72654	1707.54637	-721.27992	1996.92523	1
H	68.77590	86.43381	81.83327	758.23357	2100.81767	1560.55011	1
C	69.38198	84.24934	81.97959	-352.41524	704.96076	49.21465	1
H	69.97158	83.95726	81.08032	1714.76886	-2070.42778	443.35792	1
H	70.07711	84.42170	82.83618	1224.15040	1801.50881	1636.97633	1
C	66.10695	83.11244	81.66851	566.85117	537.05831	117.62814	1
O	65.35545	83.98619	81.24515	-550.26779	-406.08867	4.21666	1
N	66.15866	81.83424	81.35295	566.34832	-425.77725	180.18749	1
H	66.84195	81.22643	81.86364	644.44502	2646.80550	-52.73430	1
C	64.91836	81.25123	80.90094	612.34801	-281.48662	411.56221	1
H	64.12461	81.74373	81.50706	1667.23498	-1490.75142	1556.16753	1
C	64.74403	81.52352	79.34532	-714.45746	-175.43074	286.98927	1
H	65.58821	81.04636	78.76417	-787.09484	1763.03619	-1922.46060	1
O	64.81882	82.88029	78.87649	-429.46514	516.51932	-128.40526	1
H	64.97366	83.37030	79.76229	1981.82785	436.16847	-1818.18182	1
C	63.41534	80.74357	79.04404	630.79189	-468.94448	76.03230	1
H	63.19364	80.72103	77.95158	-1775.71861	2062.30353	131.89849	1
H	63.46615	79.68467	79.39881	-1855.14608	88.94118	1993.53760	1
H	62.54180	81.21657	79.55049	-1504.82350	-780.94249	-2132.90992	1
C	65.02243	79.63728	81.10658	288.57638	618.62814	-397.00270	1
O	64.06110	79.02573	81.50513	321.70495	-185.26452	574.37218	1
N	66.17574	79.16829	80.65185	-447.05792	-446.53431	367.76425	1
H	66.97102	79.72320	80.28047	350.31316	1975.63644	-1843.31522	1
C	66.39657	77.75256	80.71344	-511.23061	374.03917	-471.51480	1
H	66.15490	77.42096	81.74804	2234.79579	-898.70890	-1273.45190	1
C	65.41210	77.08392	79.63905	529.53781	-159.34528	563.72604	1

```
H    65.46865  75.96893  79.77200     654.59388    2347.94251    1217.51488    1
H    64.37407  77.39668  79.93606   -1526.55661    2103.42026     817.87394    1
O    65.67648  77.36702  78.24217    -554.84278      14.12789     399.58769    1
H    65.33013  78.27874  78.19810    1297.06236    2061.84854    1220.68812    1
C    67.96064  77.71440  80.30685    -662.15484    -412.13544     123.62687    1
O    68.47970  78.73336  79.86386    -540.01537    -317.05804     274.91456    1
N    68.49754  76.52970  80.55313    -202.76299    -690.00100    -131.49145    1
H    67.91744  75.77947  80.93052    -702.62132   -2617.37191     281.70413    1
C    69.83321  76.27241  80.16410    -491.73914     576.34187     222.73509    1
H    70.14546  77.16212  79.56967    1977.17978   -1823.58850    -434.68928    1
C    70.66106  76.06101  81.50894    -649.50797    -328.66843     306.11224    1
H    70.17110  75.28566  82.13627    2075.07803    1452.16490   -1004.45904    1
C    72.12329  75.64232  81.13883    -615.18735    -489.99689     -70.96352    1
H    72.75393  75.50691  82.04906   -1226.32661   -1724.38906   -1716.47170    1
H    72.14074  74.67931  80.57460    2381.64273    -969.84735     900.46860    1
H    72.62092  76.40953  80.50016    2122.74991    1702.23946    -141.36228    1
C    70.73499  77.42258  82.28096    -501.86074    -479.61276    -376.42482    1
H    71.21049  77.30861  83.28392     993.79261   -2246.95401   -1177.81305    1
H    71.32950  78.18021  81.71984      42.71279    -626.15391   -2651.37258    1
H    69.71797  77.85357  82.44139   -2026.39012   -1590.33709    -887.82922    1
C    69.77745  74.84196  79.34896    -672.89153     382.57394    -156.34072    1
O    69.49314  73.80296  79.89981     410.04627     221.86223     500.35950    1
N    70.05367  75.05090  78.06702    -378.11782    -443.79414    -441.10984    1
H    70.32955  75.98321  77.76172    2404.15973    -568.14894    1149.30085    1
C    70.24411  73.88976  77.25802    -544.54484     466.16019     331.29119    1
H    69.59130  73.11498  77.72101   -1409.80988    -170.17955    2325.32679    1
C    69.89108  74.27659  75.76264    -184.48284     116.78282    -758.89137    1
H    68.83266  74.61444  75.68944    1893.78641    1861.14652     611.04528    1
H    70.50890  75.14155  75.43143   -2170.79665    -974.19311    1327.50117    1
C    70.15018  73.03709  74.84868     389.51668     672.81878    -138.49152    1
H    71.17287  72.62498  75.01868     510.64577    1933.03327    1851.02484    1
H    69.47571  72.20082  75.13768   -2302.30869   -1105.72939    -948.89774    1
C    69.96628  73.37450  73.31826    -559.93427    -126.34590     542.30857    1
O    70.35835  72.60650  72.47761    -670.36003    -111.83166      76.36448    1
N    69.32122  74.54029  73.08967    -216.06729    -537.07037     446.51379    1
H    68.90363  75.19199  73.76331   -1251.96487    1074.12894   -2168.52500    1
H    69.02720  74.86005  72.16076   -2451.83023    -720.73851     944.84612    1
C    71.84259  73.52552  77.31639     546.24052    -563.88337     -85.11809    1
O    72.66995  74.34851  77.03259      71.70702    -307.40463     606.69673    1
N    71.99479  72.23959  77.66821     479.90863    -504.50129     222.86849    1
C    73.26201  71.58629  77.59493      75.51268    -600.91904     506.73700    1
H    74.04657  72.36539  77.73783   -2033.61562    1795.30103     254.89480    1
C    73.12125  70.50285  78.75276     523.79067     517.99274     284.45581    1
H    73.84575  69.66423  78.65252    1599.61665   -1127.00911    1895.98173    1
H    73.28742  70.95248  79.75668   -1881.83093    1275.82488   -1501.55016    1
C    71.63770  70.05844  78.57007    -600.05639    -404.05026     316.64485    1
H    71.53340  69.36279  77.70713   -2597.05260     496.36221     657.73249    1
H    71.21532  69.53172  79.45556   -1666.24518    1294.38985   -1723.90591    1
C    70.92926  71.44847  78.27260     493.57570    -129.95376    -602.56394    1
H    70.08347  71.39263  77.54719   -1469.08415    -637.84797    2204.22581    1
H    70.60434  72.02047  79.17893   -1038.97840    1068.88701   -2280.71789    1
C    73.38808  70.79777  76.16730     188.88100     241.05089    -727.87821    1
O    72.46526  70.64635  75.40551     531.88613     -49.19943     427.07960    1
N    74.60691  70.17254  76.11935     708.14833      26.93417    -179.73509    1
H    75.12249  70.64100  76.87058   -2232.69501   -1179.09831    1023.94816    1
C    75.26480  69.93429  74.81542    -738.32412     259.92548     104.42456    1
H    76.22648  69.49527  75.16739    2028.89123    1260.02983    1311.33324    1
C    74.44964  68.97195  73.99027    -258.65678    -345.52513     661.28459    1
H    73.66840  69.56288  73.45832   -1826.06090   -1801.46097    -918.64554    1
C    75.29835  68.23836  72.96371    -541.23400     531.92970    -218.41428    1
H    74.66529  67.61899  72.27258    1311.73218   -2219.18200     882.19023    1
H    75.90312  68.93868  72.32482   -1048.71298    1223.58118   -2196.97971    1
```

132	H	76.02625	67.54045	73.45854	419.06414	1903.92898	1903.44670	1
133	C	73.72254	67.93989	74.83875	-423.86431	152.66566	-648.55262	1
134	H	73.10275	67.24358	74.21179	105.68386	-2342.50080	1387.51238	1
135	H	74.44099	67.31110	75.43078	-1695.63603	-1484.73621	-1531.02793	1
136	H	73.03032	68.43789	75.57363	-1853.57121	-487.39031	1936.59343	1
137	C	75.49824	71.43563	74.16854	539.71428	-510.58232	267.58633	1
138	O	75.76272	72.36218	74.89384	-15.93758	-278.34085	-624.49402	1
139	N	75.49174	71.39741	72.84468	-676.81886	12.05292	276.17960	1
140	H	75.26164	70.50472	72.41196	-1694.44250	-373.84189	-2100.66098	1
141	C	75.50328	72.57074	72.04187	412.33078	-664.70360	108.35451	1
142	H	75.52423	72.14742	71.01136	1765.77476	1294.44366	1621.76594	1
143	C	74.18792	73.41291	72.31887	-179.66635	-721.95667	-264.73915	1
144	H	73.28318	72.76440	72.36880	-607.34922	2587.87032	-598.10018	1
145	H	74.24874	73.93106	73.30316	-359.69332	-1479.13954	-2259.74260	1
146	C	74.10930	74.42950	71.13270	-240.01076	616.02425	431.85322	1
147	H	75.05804	75.02102	71.08644	-1359.17131	782.54425	2227.99181	1
148	H	74.03457	73.89160	70.16048	-820.36667	85.81767	-2596.78715	1
149	C	72.88157	75.40807	71.31465	-513.07015	236.18570	-551.87280	1
150	H	71.91182	74.86040	71.25812	1816.11944	-1183.11722	-1650.94321	1
151	H	72.90003	75.89781	72.31552	-1800.04429	-1361.53473	1526.34643	1
152	C	73.02115	76.45067	70.17959	726.08972	-253.19732	-179.64713	1
153	H	73.92316	77.08863	70.25313	1224.27271	-456.51798	-2390.90304	1
154	H	72.93850	76.03314	69.15631	1206.40339	-1150.93514	-2154.90344	1
155	N	71.83367	77.46258	70.29389	329.70796	-633.68968	-155.67358	1
156	H	71.83210	78.24506	69.57714	1348.31305	1938.76845	1359.00643	1
157	H	70.85339	77.07221	70.17922	2096.92035	1446.54551	-966.48890	1
158	H	71.75047	78.01002	71.19952	-1197.07217	-2303.53719	827.28733	1
159	C	76.80589	73.50525	72.38173	-378.01186	-556.17301	-413.96481	1
160	O	76.71726	74.72013	72.32371	-409.44433	-307.55053	-453.30833	1
161	N	77.88202	72.76538	72.54919	-429.02477	524.70884	274.08502	1
162	H	77.71864	71.76185	72.59207	1706.34263	-1425.96036	1574.38926	1
163	C	79.15178	73.37429	72.85182	-45.25864	-626.22952	478.93267	1
164	H	79.37974	73.92405	71.90194	1988.63164	570.90773	1772.87287	1
165	C	78.97789	74.38783	74.05185	551.77128	-20.49526	-564.54996	1
166	H	78.14808	75.10993	73.84470	1434.61535	-1753.10253	1513.99471	1
167	H	78.69945	73.85581	74.99018	-714.67738	1819.09770	1898.36494	1
168	C	80.35265	75.12413	74.18990	446.99263	-555.77305	338.97221	1
169	H	81.15371	74.39025	74.44317	1953.68466	1892.25957	-161.67766	1
170	H	80.64840	75.58999	73.21940	-403.03785	2688.07032	-188.43591	1
171	C	80.21465	76.21888	75.32130	567.86262	335.74681	434.04421	1
172	H	79.54311	77.04562	74.99313	-1080.02275	1568.26081	-1948.78900	1
173	H	79.74066	75.78670	76.23235	-919.54108	2111.34692	1456.13463	1
174	C	81.63062	76.74836	75.64940	195.81897	173.33862	-745.11536	1
175	H	82.31805	75.98926	76.07343	1405.67949	-2301.21089	-390.08113	1
176	H	82.12979	77.28997	74.82087	-940.37328	-1118.32446	2299.72133	1
177	N	81.47605	77.81262	76.78752	319.26422	656.36007	-42.02424	1
178	H	82.37987	78.23453	77.14859	1961.03633	619.91968	1787.09422	1
179	H	80.91448	78.68442	76.56110	854.14343	-2156.80752	1429.08711	1
180	H	81.02289	77.48789	77.69083	1237.02905	-1378.50857	-1998.28500	1
181	C	80.20853	72.19859	73.19177	532.53319	-268.57236	517.55729	1
182	O	80.76017	72.14477	74.29469	266.16714	-290.73804	-558.88002	1
183	N	80.39338	71.39743	72.16322	-226.25352	640.49678	-270.32839	1
184	H	79.84682	71.52853	71.31369	2007.69063	-1035.27229	1523.50231	1
185	C	81.52095	70.52114	72.23240	-540.76542	-573.33303	49.50000	1
186	H	82.26814	71.09175	72.82927	-2374.20950	-1036.76023	843.75774	1
187	C	81.04483	69.22630	73.02300	-77.09082	666.87422	-415.84136	1
188	H	80.18828	68.72219	72.48986	-985.81834	617.19825	2463.91927	1
189	O	80.49375	69.44777	74.33763	293.29899	-391.65602	-477.80926	1
190	H	80.62687	70.44959	74.42119	2528.72714	-681.76262	-751.27122	1
191	C	82.31053	68.30660	72.93004	252.47363	-605.14473	440.05134	1
192	H	82.12731	67.30958	73.39517	-2052.65063	1456.49051	-1043.51830	1
193	H	82.60901	68.13132	71.86832	-2152.98800	-1661.97574	-161.70652	1

```
H    83.18806   68.75806   73.45006      -473.12726     -1584.27339      2165.61725          1
C    81.97847   70.17947   70.68562       360.36307       488.46713       505.10056          1
O    81.27135   69.55621   69.93336      -200.08817      -528.03552      -385.82850          1
N    83.18303   70.70333   70.50092      -597.54095      -226.44775       355.20479          1
H    83.60096   71.17625   71.30276      2179.23655     -1300.70981      -991.33835          1
C    83.97261   70.45811   69.35572      -687.68921       297.39807      -249.45179          1
H    84.96962   70.86442   69.63712      -349.16256     -2467.37713     -1101.72831          1
C    84.01872   68.88952   69.01791       672.47345       361.54328      -201.62880          1
H    82.99729   68.44091   69.06427      1541.41502       582.18425      2169.97026          1
H    84.39168   68.75324   67.97556     -2534.67717        60.25153       997.72295          1
C    84.96942   68.22314   70.02141       574.26774      -389.43119      -377.02542          1
C    86.34683   68.59922   70.02196       688.82679      -287.33580      -257.96133          1
H    86.73028   69.33575   69.29275       331.14182      1470.38163      2269.80013          1
C    84.48727   67.25664   70.95442      -383.52382      -675.04808       144.24917          1
H    83.42327   66.95231   70.94945         4.09847      1517.59662      2262.86397          1
C    87.19670   68.02565   70.96618      -134.76730       428.45285      -649.50303          1
H    88.26010   68.32235   70.96914      -903.23433      1600.49020      2011.53387          1
C    85.37743   66.71091   71.88257      -335.53281      -615.00040      -364.39058          1
H    84.98948   65.97196   72.60521      1596.45398     -1369.33862      1732.02659          1
C    86.75532   67.07245   71.92955       585.48547      -340.24194      -406.23938          1
O    87.66748   66.53008   72.87170      -419.15649      -173.04408      -511.94141          1
H    87.06456   65.91598   73.35316     -1071.84350      1559.71507     -1960.13012          1
C    83.37974   71.17352   68.01789       291.41878      -183.13091       710.72246          1
O    83.87019   70.91043   66.94196       379.87695       523.47396      -222.23622          1
N    82.46312   72.10905   68.28926       564.30591       373.09380       277.24371          1
H    82.11175   72.10636   69.24706      -120.59931     -2436.07824     -1214.35098          1
C    81.72692   72.63328   67.17622       404.07514      -299.37480       608.84009          1
H    82.42921   72.51395   66.31655     -1071.07882      -841.69025      2359.66467          1
C    80.43005   71.72603   67.00328      -683.12807        68.71770      -390.13096          1
H    80.69586   70.64693   67.07376      2068.88918      1596.58590       770.89603          1
H    79.69696   71.90937   67.82515      1379.86429      2342.49779      -179.85638          1
C    79.81414   72.07019   65.61515      -176.39967       537.49466       550.97268          1
H    79.54507   73.15584   65.57121      -396.28051      1942.22168      1869.33314          1
H    80.55607   71.91251   64.80028       -94.44700       586.00678     -2659.20016          1
C    78.51961   71.15581   65.38860      -291.69381      -718.18079      -150.72929          1
H    78.69165   70.05165   65.32820     -2303.38776      1089.76242       964.62032          1
H    77.69498   71.37346   66.11729       884.16729      2568.80533      -207.74283          1
N    78.12961   71.63773   64.13139      -594.49522      -252.81478       342.29276          1
H    78.31209   72.61436   63.95238     -1735.17426      2084.05705      -263.71605          1
C    77.53356   70.97218   63.22425       595.23177      -271.52487       442.22352          1
N    77.24120   69.62608   63.45967       585.53813      -424.79750      -105.81303          1
H    77.39972   69.06533   64.29560       959.88797      1327.56823     -2177.11905          1
H    76.73891   69.01600   62.81355      1578.48647        16.27378     -2220.76233          1
N    77.27320   71.77866   62.10798      -452.17763      -390.33541      -421.51853          1
H    77.47131   72.76330   61.93659       470.26107      1974.25629     -1817.92087          1
H    76.78447   71.44499   61.27642      1856.67307     -1573.97321     -1224.35416          1
C    81.26364   74.15799   67.45137      -531.47271      -581.45780       -55.06206          1
O    80.45878   74.69193   66.68342      -243.00837       192.73507       609.52485          1
N    81.87376   74.69656   68.48636       343.49288      -644.25607        38.09164          1
H    82.51842   74.05221   68.94482     -1691.16043     -1535.97525     -1484.73099          1
C    81.42696   75.90222   69.16912       542.66492       350.14337       454.42563          1
H    82.21632   76.09510   69.93416      1209.57979     -2396.95920       463.86744          1
C    81.26511   77.02949   68.15742      -737.14309       145.93010       242.71942          1
H    80.61188   76.65178   67.32894     -1050.18303       159.36419     -2509.06125          1
C    80.60007   78.24075   68.77978      -706.77696      -252.88343      -245.16230          1
H    80.43833   79.06021   68.02592     -1624.64039     -1665.49085      1417.86935          1
H    79.58909   77.98031   69.21799      2566.10521       598.51109       693.22305          1
H    81.22170   78.67188   69.61270     -1972.69758       107.10018     -1876.34312          1
C    82.59596   77.41601   67.54044       412.85148       672.58832       -27.69222          1
H    82.47522   78.20607   66.74871      1447.02277      1762.76533     -1490.78944          1
H    83.30876   77.82083   68.31091      1320.77379      -145.20455      2378.68491          1
H    83.09325   76.52881   67.05859     -1512.10848      1470.52031      1724.75147          1
```

```
C    79.96330  75.55399  69.88488      -607.11113      499.56689       73.73769          1
O    79.86237  75.71411  71.07675       357.97126      460.81534      356.68785          1
N    79.06549  75.17938  69.00135       536.55407     -368.62270      332.76306          1
H    79.32159  75.11776  67.99401       524.38866     2297.69985     1367.20941          1
C    77.77607  74.78120  69.44148        46.50277     -774.29190     -147.97904          1
H    77.82319  74.82833  70.55900      1676.51875     1883.29066     1032.55549          1
C    76.71133  75.77271  68.79036      -559.41456      -34.19198     -556.30433          1
H    76.75489  75.70288  67.68101       -64.66664      395.18769     2695.05373          1
H    75.67429  75.48852  69.07293     -2703.44278      -60.40056      333.79418          1
C    77.03882  77.22996  69.25635      -519.76435      377.31045      459.42366          1
H    78.10064  77.48057  69.00952     -1746.74323     1667.50644    -1261.73691          1
C    76.05693  78.21119  68.51824       570.95554       16.46261     -545.27700          1
H    76.21070  79.26615  68.84395      2391.58273       28.05277    -1305.07271          1
H    76.20373  78.18154  67.41354       175.75588      917.63458     2559.43867          1
H    74.99059  77.95589  68.71551     -1063.97851    -2155.34650     1283.00462          1
C    76.79976  77.35470  70.80520       193.76014        2.62946     -765.53141          1
H    77.00440  78.38820  71.16945       507.39394     -846.55087     2539.60089          1
H    75.75085  77.10669  71.08567      1337.57841     1818.19054     1526.01994          1
H    77.46397  76.66314  71.38024      1100.88635    -1172.91350    -2199.08909          1
C    77.52790  73.29673  68.81964      -549.21115      475.64486     -309.38218          1
O    76.97412  73.11532  67.76831       556.06106     -390.37755      -78.24250          1
O    78.08622  72.21807  69.53976      -572.27940     -310.19032     -209.75862          1
H    78.33224  72.69964  70.38011     -1380.08193     2212.14486      790.85944          1
```

Listing 25: C00025.xmolout

```
      276
  150.0000  150.0000  150.0000     0.0000     0.0000     0.0000***********    0.7890     3156
       0.2500     300.00    0.00    0.00    0.00   -1293.9590       0.0000     300.0000
H    66.80058  61.07021  72.04983     -2043.14101    -1757.64893      399.89339          1
N    66.86297  62.06785  72.25056       426.75047      490.45733     -334.42467          1
H    66.83971  62.10426  73.27819       442.25559    -1110.30854    -2448.52907          1
C    68.03550  62.68018  71.75648       415.32972     -666.39213       83.73172          1
H    68.42401  62.05782  70.92112       879.32219     2491.00447      667.35213          1
C    67.66151  64.18160  71.29986        31.35357      477.41777      628.23362          1
H    67.23850  64.73423  72.17253      1168.38489     2240.25337    -1019.71136          1
H    68.57769  64.74048  70.99164      -360.43770     2534.90498      931.67112          1
C    66.65769  64.12182  70.17853       463.18208      512.70242     -382.34388          1
H    65.85822  63.40304  70.46960      1725.29117     1863.52247     -987.07854          1
C    66.02132  65.48885  69.95963       710.42801     -144.27427     -313.15367          1
H    65.25378  65.46219  69.14361       364.20195    -1033.13725     2494.72524          1
H    65.50996  65.86063  70.88487      2567.61904      335.80679      847.48802          1
H    66.78474  66.25672  69.67127     -2587.99029      680.92867     -512.16411          1
C    67.29541  63.64563  68.88053       268.17616      741.64379      -40.42634          1
H    66.55099  63.61187  68.04344      1283.54314    -1945.37420    -1411.27812          1
H    68.12645  64.32409  68.55683     -1258.57796     1317.15249    -2026.02085          1
H    67.72648  62.61664  68.98141     -2502.84526      909.80597      575.92391          1
C    68.99548  62.72613  73.06802       619.36578     -458.64843     -172.09273          1
O    68.54325  62.61061  74.19291       -81.40433      433.87611      522.34594          1
N    70.24903  62.95593  72.69613       175.23996     -622.20190      341.55523          1
H    70.45923  63.08166  71.70665      -876.57256     -303.22879    -2561.90213          1
C    71.26758  62.92166  73.68644      -571.88214     -534.10783     -106.15033          1
H    70.76401  62.55434  74.60995     -2713.20841      -16.25800     -248.80809          1
C    72.43260  61.96339  73.12266       166.60354      288.16399     -716.09568          1
H    72.76164  62.36547  72.12581     -2097.85574     1636.78449     -586.18044          1
O    72.04671  60.59944  72.89773      -317.80580     -363.38227      484.43082          1
H    72.19274  60.51940  71.93297      1863.74748    -1451.84923     1357.29467          1
C    73.58979  62.18219  74.16310       503.80731      517.99032      318.51606          1
H    74.51987  61.65036  73.85395       640.61020    -2157.10410    -1536.29104          1
H    73.85474  63.26150  74.27633      1687.88455      465.60692     2087.56452          1
H    73.31183  61.79747  75.17147     -1293.07895    -1899.88581    -1463.57452          1
C    71.88875  64.42062  73.78985      -153.07550     -733.68902      248.70981          1
```

O	72.32365	64.96483	72.78702	186.15459	-480.78787	-449.34387	1
N	71.91628	64.84169	75.05266	-487.53139	543.18517	42.03850	1
C	72.37182	66.16257	75.38731	-699.35871	69.34393	360.10701	1
H	71.94397	66.82172	74.59771	2.81126	-221.99807	-2715.58072	1
C	71.80775	66.37587	76.84977	-507.46439	-592.59194	122.07761	1
H	72.39451	67.12251	77.43182	-1975.08116	525.87560	-1801.71556	1
H	70.75452	66.73595	76.83447	1588.27743	-1913.22100	1113.83566	1
C	71.90517	64.92883	77.42973	-613.62442	-457.55993	-194.14490	1
H	72.95498	64.68620	77.70952	1707.54637	-721.27992	1996.92523	1
H	71.28530	64.76834	78.34038	758.23357	2100.81767	1560.55011	1
C	71.42565	64.06646	76.18548	-352.41524	704.96076	49.21465	1
H	71.85367	63.03857	76.13252	1714.76886	-2070.42778	443.35792	1
H	70.31777	64.01384	76.05684	1224.15040	1801.50881	1636.97633	1
C	74.01173	66.16825	75.35635	566.85117	537.05831	117.62814	1
O	74.67086	66.26097	76.36132	-550.26779	-406.08867	4.21666	1
N	74.40777	66.04431	74.09449	566.34832	-425.77725	180.18749	1
H	73.71358	65.92696	73.32094	644.44502	2646.80550	-52.73430	1
C	75.79632	66.21243	73.84018	612.34801	-281.48662	411.56221	1
H	76.31493	65.99294	74.80219	1667.23498	-1490.75142	1556.16753	1
C	76.13103	65.21853	72.63073	-714.45746	-175.43074	286.98927	1
H	75.50081	65.51787	71.74786	-787.09484	1763.03619	-1922.46060	1
O	75.83473	63.82818	72.86012	-429.46514	516.51932	-128.40526	1
H	75.09992	63.68851	72.22831	1981.82785	436.16847	-1818.18182	1
C	77.63798	65.56033	72.34050	630.79189	-468.94448	76.03230	1
H	78.01512	65.00999	71.44745	-1775.71861	2062.30353	131.89849	1
H	77.78657	66.64869	72.14825	-1855.14608	88.94118	1993.53760	1
H	78.29089	65.28107	73.19961	-1504.82350	-780.94249	-2132.90992	1
C	75.99052	67.75987	73.32395	288.57638	618.62814	-397.00270	1
O	75.39272	68.15461	72.32180	321.70495	-185.26452	574.37218	1
N	76.90567	68.34725	74.04536	-447.05792	-446.53431	367.76425	1
H	77.26008	67.76669	74.80430	350.31316	1975.63644	-1843.31522	1
C	77.28748	69.71442	73.96515	-511.23061	374.03917	-471.51480	1
H	78.27857	69.69558	74.47251	2234.79579	-898.70890	-1273.45190	1
C	77.39164	70.12198	72.42876	529.53781	-159.34528	563.72604	1
H	78.12617	70.96737	72.33102	654.59388	2347.94251	1217.51488	1
H	77.83382	69.23873	71.89657	-1526.55661	2103.42026	817.87394	1
O	76.18166	70.54983	71.76958	-554.84278	14.12789	399.58769	1
H	75.67231	69.64724	71.79659	1297.06236	2061.84854	1220.68812	1
C	76.24618	70.65219	74.79662	-662.15484	-412.13544	123.62687	1
O	75.30632	70.22452	75.42277	-540.01537	-317.05804	274.91456	1
N	76.71187	71.91387	74.78123	-202.76299	-690.00100	-131.49145	1
H	77.50785	72.02459	74.15396	-702.62132	-2617.37191	281.70413	1
C	75.89785	73.07004	75.16348	-491.73914	576.34187	222.73509	1
H	76.52553	73.92780	74.82070	1977.17978	-1823.58850	-434.68928	1
C	74.55842	72.99961	74.45740	-649.50797	-328.66843	306.11224	1
H	74.08349	72.02876	74.73584	2075.07803	1452.16490	-1004.45904	1
C	73.63086	74.12901	74.86752	-615.18735	-489.99689	-70.96352	1
H	72.64877	74.08191	74.32168	-1226.32661	-1724.38906	-1716.47170	1
H	73.40351	74.10189	75.97925	2381.64273	-969.84735	900.46860	1
H	74.08753	75.13244	74.64877	2122.74991	1702.23946	-141.36228	1
C	74.71182	73.02604	72.94720	-501.86074	-479.61276	-376.42482	1
H	73.71621	72.97715	72.42677	993.79261	-2246.95401	-1177.81305	1
H	75.22104	73.96740	72.60096	42.71279	-626.15391	-2651.37258	1
H	75.33071	72.14789	72.57021	-2026.39012	-1590.33709	-887.82922	1
C	75.74023	73.05441	76.81257	-672.89153	382.57394	-156.34072	1
O	74.74614	73.49817	77.35216	410.04627	221.86223	500.35950	1
N	76.85581	72.63695	77.38566	-378.11782	-443.79414	-441.10984	1
H	77.57271	72.33528	76.73011	2404.15973	-568.14894	1149.30085	1
C	76.81818	72.30826	78.77693	-544.54484	466.16019	331.29119	1
H	75.94500	72.88999	79.15333	-1409.80988	-170.17955	2325.32679	1
C	76.54561	70.74764	78.84633	-184.48284	116.78282	-758.89137	1
H	75.55221	70.49771	78.40826	1893.78641	1861.14652	611.04528	1

H	77.27995	70.18649	78.22522	-2170.79665	-974.19311	1327.50117	1
C	76.63314	70.31979	80.34440	389.51668	672.81878	-138.49152	1
H	77.51468	70.79444	80.83028	510.64577	1933.03327	1851.02484	1
H	75.76638	70.72261	80.91302	-2302.30869	-1105.72939	-948.89774	1
C	76.72649	68.75817	80.52867	-559.93427	-126.34590	542.30857	1
O	76.94750	68.30336	81.62084	-670.36003	-111.83166	76.36448	1
N	76.53802	68.05143	79.39153	-216.06729	-537.07037	446.51379	1
H	76.25606	68.37292	78.45810	-1251.96487	1074.12894	-2168.52500	1
H	76.48606	67.02747	79.35980	-2451.83023	-720.73851	944.84612	1
C	78.22859	72.80937	79.43857	546.24052	-563.88337	-85.11809	1
O	78.13165	73.79526	80.12472	71.70702	-307.40463	606.69673	1
N	79.32578	72.08219	79.18027	479.90863	-504.50129	222.86849	1
C	80.61279	72.57342	79.56890	75.51268	-600.91904	506.73700	1
H	80.47685	73.20265	80.47919	-2033.61562	1795.30103	254.89480	1
C	81.40652	71.20681	79.77018	523.79067	517.99274	284.45581	1
H	82.51066	71.34254	79.78105	1599.61665	-1127.00911	1895.98173	1
H	81.13295	70.71436	80.72994	-1881.83093	1275.82488	-1501.55016	1
C	80.89330	70.38944	78.54685	-600.05639	-404.05026	316.64485	1
H	81.39875	70.71632	77.61058	-2597.05260	496.36221	657.73249	1
H	81.06858	69.29422	78.63982	-1666.24518	1294.38985	-1723.90591	1
C	79.35128	70.77850	78.53284	493.57570	-129.95376	-602.56394	1
H	78.90978	70.89905	77.51582	-1469.08415	-637.84797	2204.22581	1
H	78.70345	70.10798	79.14649	-1038.97840	1068.88701	-2280.71789	1
C	81.24541	73.39261	78.30248	188.88100	241.05089	-727.87821	1
O	80.89583	73.18526	77.14557	531.88613	-49.19943	427.07960	1
N	82.19506	74.18575	78.74419	708.14833	26.93417	-179.73509	1
H	82.29918	74.16220	79.75709	-2232.69501	-1179.09831	1023.94816	1
C	83.01989	75.04743	77.91738	-738.32412	259.92548	104.42456	1
H	83.81875	75.39990	78.61395	2028.89123	1260.02983	1311.33324	1
C	83.56045	74.24258	76.74048	-258.65678	-345.52513	661.28459	1
H	82.68178	73.82600	76.18811	-1826.06090	-1801.46097	-918.64554	1
C	84.35652	75.11507	75.78948	-541.23400	531.92970	-218.41428	1
H	84.67661	74.54764	74.87208	1311.73218	-2219.18200	882.19023	1
H	83.75800	76.00355	75.44418	-1048.71298	1223.58118	-2196.97971	1
H	85.28936	75.51375	76.27622	419.06414	1903.92898	1903.44670	1
C	84.40519	73.07022	77.20260	-423.86431	152.66566	-648.55262	1
H	84.76646	72.44875	76.33744	105.68386	-2342.50080	1387.51238	1
H	85.31480	73.41246	77.76909	-1695.63603	-1484.73621	-1531.02793	1
H	83.82391	72.39062	77.88510	-1853.57121	-487.39031	1936.59343	1
C	82.08913	76.32518	77.38484	539.71428	-510.58232	267.58633	1
O	82.57198	77.42704	77.42776	-15.93758	-278.34085	-624.49402	1
N	80.92361	75.95876	76.88301	-676.81886	12.05292	276.17960	1
H	80.65785	74.95530	76.84040	-1694.44250	-373.84189	-2100.66098	1
C	79.96791	77.02284	76.73231	412.33078	-664.70360	108.35451	1
H	80.09778	77.66840	77.62969	1765.77476	1294.44366	1621.76594	1
C	80.42551	77.77868	75.42249	-179.66635	-721.95667	-264.73915	1
H	81.52725	77.95071	75.46251	-607.34922	2587.87032	-598.10018	1
H	80.25234	77.13697	74.52945	-359.69332	-1479.13954	-2259.74260	1
C	79.66505	79.13964	75.28210	-240.01076	616.02425	431.85322	1
H	78.56839	78.98029	75.19982	-1359.17131	782.54425	2227.99181	1
H	79.81137	79.78166	76.17951	-820.36667	85.81767	-2596.78715	1
C	80.23473	79.83977	73.98346	-513.07015	236.18570	-551.87280	1
H	81.23968	80.27648	74.18555	1816.11944	-1183.11722	-1650.94321	1
H	80.39056	79.09189	73.17268	-1800.04429	-1361.53473	1526.34643	1
C	79.24291	80.92970	73.51225	726.08972	-253.19732	-179.64713	1
H	78.30012	80.54086	73.06947	1224.27271	-456.51798	-2390.90304	1
H	79.01749	81.71432	74.26485	1206.40339	-1150.93514	-2154.90344	1
N	79.92154	81.70668	72.33233	329.70796	-633.68968	-155.67358	1
H	79.33964	82.48800	71.91324	1348.31305	1938.76845	1359.00643	1
H	80.82749	82.21495	72.54897	2096.92035	1446.54551	-966.48890	1
H	80.18187	81.15180	71.46599	-1197.07217	-2303.53719	827.28733	1
C	78.46917	76.39519	76.54107	-378.01186	-556.17301	-413.96481	1

O	78.23168	75.44412	75.83478	-409.44433	-307.55053	-453.30833	1
N	77.61335	77.19561	77.16909	-429.02477	524.70884	274.08502	1
H	77.97561	78.00052	77.67838	1706.34263	-1425.96036	1574.38926	1
C	76.22028	76.92240	77.10054	-45.25864	-626.22952	478.93267	1
H	76.16492	75.88854	76.68370	1988.63164	570.90773	1772.87287	1
C	75.64124	77.08733	78.56340	551.77128	-20.49526	-564.54996	1
H	76.29054	76.57449	79.30907	1434.61535	-1753.10253	1513.99471	1
H	75.63747	78.16346	78.85195	-714.67738	1819.09770	1898.36494	1
C	74.18823	76.50991	78.59256	446.99263	-555.77305	338.97221	1
H	73.58526	76.92613	77.75169	1953.68466	1892.25957	-161.67766	1
H	74.18861	75.40080	78.46155	-403.03785	2688.07032	-188.43591	1
C	73.57695	76.94187	79.98611	567.86262	335.74681	434.04421	1
H	74.12990	76.46516	80.82824	-1080.02275	1568.26081	-1948.78900	1
H	73.69230	78.04101	80.14227	-919.54108	2111.34692	1456.13463	1
C	72.08026	76.55630	80.01721	195.81897	173.33862	-745.11536	1
H	71.46540	77.02583	79.22339	1405.67949	-2301.21089	-390.08113	1
H	71.87859	75.46709	80.06065	-940.37328	-1118.32446	2299.72133	1
N	71.50620	77.13784	81.35348	319.26422	656.36007	-42.02424	1
H	70.47065	76.99237	81.53715	1961.03633	619.91968	1787.09422	1
H	71.93610	76.78034	82.25532	854.14343	-2156.80752	1429.08711	1
H	71.59897	78.18702	81.47766	1237.02905	-1378.50857	-1998.28500	1
C	75.52485	78.06244	76.13971	532.53319	-268.57236	517.55729	1
O	74.40729	78.51708	76.36209	266.16714	-290.73804	-558.88002	1
N	76.33642	78.31021	75.13704	-226.25352	640.49678	-270.32839	1
H	77.18155	77.74275	75.17212	2007.69063	-1035.27229	1523.50231	1
C	76.03806	79.16565	74.04622	-540.76542	-573.33303	49.50000	1
H	76.91430	79.03287	73.37482	-2374.20950	-1036.76023	843.75774	1
C	74.67932	78.63713	73.38654	-77.09082	666.87422	-415.84136	1
H	73.85706	78.67624	74.15508	-985.81834	617.19825	2463.91927	1
O	74.69810	77.27553	72.91634	293.29899	-391.65602	-477.80926	1
H	74.01947	76.87188	73.49334	2528.72714	-681.76262	-751.27122	1
C	74.43172	79.73932	72.29325	252.47363	-605.14473	440.05134	1
H	73.46408	79.57858	71.76439	-2052.65063	1456.49051	-1043.51830	1
H	74.40000	80.75910	72.73830	-2152.98800	-1661.97574	-161.70652	1
H	75.23613	79.73794	71.52177	-473.12726	-1584.27339	2165.61725	1
C	75.90970	80.70826	74.58709	360.36307	488.46713	505.10056	1
O	76.77826	81.48236	74.26334	-200.08817	-528.03552	-385.82850	1
N	74.79389	80.92695	75.27228	-597.54095	-226.44775	355.20479	1
H	74.22827	80.09946	75.57810	2179.23655	-1300.70981	-991.33835	1
C	74.64844	82.14928	75.97183	-687.68921	297.39807	-249.45179	1
H	73.66451	82.03819	76.47800	-349.16256	-2467.37713	-1101.72831	1
C	75.87273	82.30017	76.99008	672.47345	361.54328	-201.62880	1
H	76.83029	82.03323	76.48154	1541.41502	582.18425	2169.97026	1
H	75.96402	83.36850	77.30424	-2534.67717	60.25153	997.72295	1
C	75.63369	81.41727	78.22619	574.26774	-389.43119	-377.02542	1
C	74.38102	81.49834	78.90608	688.82679	-287.33580	-257.96133	1
H	73.57999	82.16513	78.53872	331.14182	1470.38163	2269.80013	1
C	76.66611	80.56788	78.72462	-383.52382	-675.04808	144.24917	1
H	77.64071	80.50421	78.20796	4.09847	1517.59662	2262.86397	1
C	74.18198	80.72299	80.04775	-134.76730	428.45285	-649.50303	1
H	73.20707	80.78345	80.56248	-903.23433	1600.49020	2011.53387	1
C	76.42786	79.82633	79.88728	-335.53281	-615.00040	-364.39058	1
H	77.23788	79.18130	80.27070	1596.45398	-1369.33862	1732.02659	1
C	75.18554	79.86716	80.58582	585.48547	-340.24194	-406.23938	1
O	74.91967	79.09898	81.75193	-419.15649	-173.04408	-511.94141	1
H	75.81046	78.70080	81.89282	-1071.84350	1559.71507	-1960.13012	1
C	74.75051	83.48990	75.04399	291.41878	-183.13091	710.72246	1
O	75.19655	84.51509	75.51504	379.87695	523.47396	-222.23622	1
N	74.13810	83.37206	73.81859	564.30591	373.09380	277.24371	1
H	73.99583	82.35582	73.77126	-120.59931	-2436.07824	-1214.35098	1
C	74.84291	84.09547	72.77975	404.07514	-299.37480	608.84009	1
H	75.66287	84.63637	73.30537	-1071.07882	-841.69025	2359.66467	1

```
C   75.37619  83.02937  71.74839     -683.12807      68.71770    -390.13096        1
H   75.97502  82.24125  72.26306     2068.88918    1596.58590     770.89603        1
H   74.52313  82.49856  71.26812     1379.86429    2342.49779    -179.85638        1
C   76.23855  83.77100  70.67716     -176.39967     537.49466     550.97268        1
H   75.72182  84.70858  70.34103     -396.28051    1942.22168    1869.33314        1
H   77.20974  84.10524  71.10641      -94.44700     586.00678   -2659.20016        1
C   76.46003  82.77981  69.44572     -291.69381    -718.18079    -150.72929        1
H   77.19400  81.95019  69.60967    -2303.38776    1089.76242     964.62032        1
H   75.52958  82.33632  69.00745      884.16729    2568.80533    -207.74283        1
N   76.98737  83.66985  68.49567     -594.49522    -252.81478     342.29276        1
H   77.08162  84.63445  68.77450    -1735.17426    2084.05705    -263.71605        1
C   77.33955  83.35349  67.31589      595.23177    -271.52487     442.22352        1
N   77.20783  82.01565  66.92828      585.53813    -424.79750    -105.81303        1
H   76.85802  81.21963  67.45992      959.88797    1327.56823   -2177.11905        1
H   77.45504  81.64763  66.00902     1578.48647      16.27378   -2220.76233        1
N   77.81077  84.47244  66.61325     -452.17763    -390.33541    -421.51853        1
H   77.88998  85.44959  66.89051      470.26107    1974.25629   -1817.92087        1
H   78.12780  84.43130  65.64409     1856.67307   -1573.97321   -1224.35416        1
C   73.83081  85.10718  72.02066     -531.47271    -581.45780     -55.06206        1
O   74.27202  85.86761  71.17611     -243.00837     192.73507     609.52485        1
N   72.55621  84.94604  72.36234      343.49288    -644.25607      38.09164        1
H   72.34852  84.27677  73.10261    -1691.16043   -1535.97525   -1484.73099        1
C   71.60357  85.78999  71.71747      542.66492     350.14337     454.42563        1
H   72.17583  86.71034  71.45828     1209.57979   -2396.95920     463.86744        1
C   71.07870  85.02945  70.42148     -737.14309     145.93010     242.71942        1
H   70.74046  84.00451  70.68471    -1050.18303     159.36419   -2509.06125        1
C   69.90882  85.86404  69.79780     -706.77696    -252.88343    -245.16230        1
H   69.49445  85.37558  68.88478    -1624.64039   -1665.49085    1417.86935        1
H   69.06711  85.99102  70.51941     2566.10521     598.51109     693.22305        1
H   70.24627  86.88607  69.50535    -1972.69758     107.10018   -1876.34312        1
C   72.22576  84.97506  69.35617      412.85148     672.58832     -27.69222        1
H   71.91556  84.42147  68.43882     1447.02277    1762.76533   -1490.78944        1
H   72.53640  85.99903  69.04101     1320.77379    -145.20455    2378.68491        1
H   73.13629  84.47238  69.76441    -1512.10848    1470.52031    1724.75147        1
C   70.30859  86.03309  72.68810     -607.11113     499.56689      73.73769        1
O   69.84318  87.14259  72.80378      357.97126     460.81534     356.68785        1
N   69.81476  84.87565  73.13237      536.55407    -368.62270     332.76306        1
H   70.33969  84.02261  72.93968      524.38866    2297.69985    1367.20941        1
C   68.91782  84.93148  74.22792       46.50277    -774.29190    -147.97904        1
H   68.73458  86.01644  74.40464     1676.51875    1883.29066    1032.55549        1
C   67.58368  84.09782  73.85988     -559.41456     -34.19198    -556.30433        1
H   67.84156  83.01622  73.76604      -64.66664     395.18769    2695.05373        1
H   66.84437  84.16353  74.69335    -2703.44278     -60.40056     333.79418        1
C   66.99116  84.60283  72.57212     -519.76435     377.31045     459.42366        1
H   67.78358  84.57290  71.79029    -1746.74323    1667.50644   -1261.73691        1
C   65.83948  83.70866  72.12906      570.95554      16.46261    -545.27700        1
H   65.39015  84.05889  71.16418     2391.58273      28.05277   -1305.07271        1
H   66.17219  82.64984  71.97543      175.75588     917.63458    2559.43867        1
H   65.01647  83.68969  72.88905    -1063.97851   -2155.34650    1283.00462        1
C   66.50115  86.03837  72.70466      193.76014       2.62946    -765.53141        1
H   66.00622  86.39505  71.76455      507.39394    -846.55087    2539.60089        1
H   65.75438  86.14559  73.53333     1337.57841    1818.19054    1526.01994        1
H   67.34637  86.74308  72.92068     1100.88635   -1172.91350   -2199.08909        1
C   69.65247  84.15444  75.44055     -549.21115     475.64486    -309.38218        1
O   70.45461  83.27434  75.26857      556.06106    -390.37755     -78.24250        1
O   69.18811  84.46573  76.73921     -572.27940    -310.19032    -209.75862        1
H   68.55724  85.20297  76.50532    -1380.08193    2212.14486     790.85944        1
```

Listing 26: C00026.xmolout

```
       276
  150.0000  150.0000  150.0000    0.0000    0.0000    0.0000***********    2.7750    11100
```

```
        0.2500    300.00   0.00    0.00    0.00  -1293.9554     0.0000    300.0000
H    72.57283  88.15144  73.34531    -2043.14101    -1757.64893     399.89339         1
N    73.38582  87.87093  72.80265      426.75047      490.45733    -334.42467         1
H    72.96830  87.50991  71.94767      442.25559    -1110.30854   -2448.52907         1
C    74.37926  88.85863  72.63170      415.32972     -666.39213      83.73172         1
H    74.41333  89.36381  73.62059      879.32219     2491.00447     667.35213         1
C    75.77006  88.12568  72.27449       31.35357      477.41777     628.23362         1
H    75.64421  87.52141  71.34449     1168.38489     2240.25337   -1019.71136         1
H    76.56247  88.88015  72.05069     -360.43770     2534.90498     931.67112         1
C    76.16563  87.24679  73.43245      463.18208      512.70242    -382.34388         1
H    75.26959  86.66799  73.74814     1725.29117     1863.52247    -987.07854         1
C    77.25055  86.26173  73.01591      710.42801     -144.27427    -313.15367         1
H    77.54190  85.58637  73.86139      364.20195    -1033.13725    2494.72524         1
H    76.91510  85.60878  72.16934     2567.61904      335.80679     847.48802         1
H    78.17936  86.79107  72.68013    -2587.99029      680.92867    -512.16411         1
C    76.65411  88.07007  74.61670      268.17616      741.64379     -40.42634         1
H    76.96996  87.41763  75.47129     1283.54314    -1945.37420   -1411.27812         1
H    77.53554  88.70500  74.34173    -1258.57796     1317.15249   -2026.02085         1
H    75.85866  88.75760  75.00305    -2502.84526      909.80597     575.92391         1
C    74.00458  89.88902  71.44395      619.36578     -458.64843    -172.09273         1
O    74.90037  90.59616  70.96381      -81.40433      433.87611     522.34594         1
N    72.72809  89.87916  71.17085      175.23996     -622.20190     341.55523         1
H    72.17179  89.25105  71.74788     -876.57256     -303.22879   -2561.90213         1
C    72.04889  90.61246  70.15074     -571.88214     -534.10783    -106.15033         1
H    71.27402  91.16872  70.72337    -2713.20841      -16.25800    -248.80809         1
C    73.02143  91.59010  69.35604      166.60354      288.16399    -716.09568         1
H    73.89595  90.99173  68.95796    -2097.85574     1636.78449    -586.18044         1
O    73.65479  92.61364  70.12761     -317.80580     -363.38227     484.43082         1
H    74.47592  92.07533  70.45711     1863.74748    -1451.84923    1357.29467         1
C    72.09646  92.06014  68.17489      503.80731      517.99032     318.51606         1
H    72.65886  92.67947  67.43825      640.61020    -2157.10410   -1536.29104         1
H    71.65633  91.19839  67.62002     1687.88455      465.60692    2087.56452         1
H    71.24829  92.68014  68.54765    -1293.07895    -1899.88581   -1463.57452         1
C    71.25908  89.45544  69.30514     -153.07550     -733.68902     248.70981         1
O    70.09228  89.34938  69.58247      186.15459     -480.78787    -449.34387         1
N    71.98112  88.74568  68.42638     -487.53139      543.18517      42.03850         1
C    71.38906  87.63986  67.73641     -699.35871       69.34393     360.10701         1
H    70.32607  87.92070  67.55601        2.81126     -221.99807   -2715.58072         1
C    72.32233  87.52868  66.45897     -507.46439     -592.59194     122.07761         1
H    72.25485  86.53718  65.95785    -1975.08116      525.87560   -1801.71556         1
H    72.06200  88.29438  65.69435     1588.27743    -1913.22100    1113.83566         1
C    73.72271  87.80715  67.09018     -613.62442     -457.55993    -194.14490         1
H    74.10133  86.90442  67.62453     1707.54637     -721.27992    1996.92523         1
H    74.50116  88.08688  66.34485      758.23357     2100.81767    1560.55011         1
C    73.38286  88.97783  68.10874     -352.41524      704.96076      49.21465         1
H    73.95356  88.95904  69.06788     1714.76886    -2070.42778     443.35792         1
H    73.43776  89.99864  67.66135     1224.15040     1801.50881    1636.97633         1
C    71.54589  86.31957  68.70516      566.85117      537.05831     117.62814         1
O    72.57824  85.65422  68.68068     -550.26779     -406.08867       4.21666         1
N    70.46855  86.18433  69.43717      566.34832     -425.77725     180.18749         1
H    69.79336  86.93876  69.30641      644.44502     2646.80550     -52.73430         1
C    70.10698  85.01923  70.16165      612.34801     -281.48662     411.56221         1
H    69.03745  85.19385  70.41636     1667.23498    -1490.75142    1556.16753         1
C    70.32893  83.73138  69.22468     -714.45746     -175.43074     286.98927         1
H    71.39468  83.72431  68.86030     -787.09484     1763.03619   -1922.46060         1
O    69.50484  83.65959  68.05312     -429.46514      516.51932    -128.40526         1
H    70.18198  83.67698  67.34473     1981.82785      436.16847   -1818.18182         1
C    70.14873  82.58961  70.28978      630.79189     -468.94448      76.03230         1
H    70.42303  81.59193  69.87413    -1775.71861     2062.30353     131.89849         1
H    70.79653  82.76957  71.18818    -1855.14608       88.94118    1993.53760         1
H    69.09428  82.52496  70.64638    -1504.82350     -780.94249   -2132.90992         1
C    71.01926  84.82771  71.49874      288.57638      618.62814    -397.00270         1
```

O	70.43462	84.66409	72.56818	321.70495	-185.26452	574.37218	1
N	72.29726	84.82772	71.24359	-447.05792	-446.53431	367.76425	1
H	72.63562	84.98469	70.25942	350.31316	1975.63644	-1843.31522	1
C	73.24373	84.45682	72.24065	-511.23061	374.03917	-471.51480	1
H	74.17535	84.89208	71.81129	2234.79579	-898.70890	-1273.45190	1
C	72.80314	85.13519	73.61579	529.53781	-159.34528	563.72604	1
H	73.72923	85.45810	74.16324	654.59388	2347.94251	1217.51488	1
H	72.23359	86.06453	73.34661	-1526.55661	2103.42026	817.87394	1
O	72.00504	84.36042	74.53331	-554.84278	14.12789	399.58769	1
H	71.11497	84.39442	74.00905	1297.06236	2061.84854	1220.68812	1
C	73.32718	82.83173	72.30189	-662.15484	-412.13544	123.62687	1
O	72.97584	82.11373	71.36860	-540.01537	-317.05804	274.91456	1
N	73.93940	82.49147	73.41806	-202.76299	-690.00100	-131.49145	1
H	74.13004	83.28229	74.03146	-702.62132	-2617.37191	281.70413	1
C	73.93745	81.17246	73.94474	-491.73914	576.34187	222.73509	1
H	74.29971	81.31044	74.98722	1977.17978	-1823.58850	-434.68928	1
C	72.45203	80.60377	73.87251	-649.50797	-328.66843	306.11224	1
H	71.98996	80.81929	72.88269	2075.07803	1452.16490	-1004.45904	1
C	72.45763	79.06681	74.17061	-615.18735	-489.99689	-70.96352	1
H	71.42350	78.65091	74.21440	-1226.32661	-1724.38906	-1716.47170	1
H	73.01601	78.48861	73.38153	2381.64273	-969.84735	900.46860	1
H	72.94489	78.84959	75.14825	2122.74991	1702.23946	-141.36228	1
C	71.64422	81.28909	75.02629	-501.86074	-479.61276	-376.42482	1
H	70.56794	80.99643	75.00923	993.79261	-2246.95401	-1177.81305	1
H	72.04841	81.01501	76.02934	42.71279	-626.15391	-2651.37258	1
H	71.69517	82.41134	74.93926	-2026.39012	-1590.33709	-887.82922	1
C	74.94955	80.21076	73.08491	-672.89153	382.57394	-156.34072	1
O	75.95300	79.82135	73.64103	410.04627	221.86223	500.35950	1
N	74.50209	79.98371	71.86903	-378.11782	-443.79414	-441.10984	1
H	73.66462	80.51061	71.52252	2404.15973	-568.14894	1149.30085	1
C	75.08020	78.96187	71.04759	-544.54484	466.16019	331.29119	1
H	74.37252	78.98805	70.18815	-1409.80988	-170.17955	2325.32679	1
C	76.50516	79.51072	70.61166	-184.48284	116.78282	-758.89137	1
H	76.41915	80.55743	70.24143	1893.78641	1861.14652	611.04528	1
H	77.18721	79.57456	71.48983	-2170.79665	-974.19311	1327.50117	1
C	77.06448	78.57623	69.49399	389.51668	672.81878	-138.49152	1
H	76.96442	77.50706	69.78612	510.64577	1933.03327	1851.02484	1
H	76.43835	78.65660	68.57813	-2302.30869	-1105.72939	-948.89774	1
C	78.56753	78.88998	69.14037	-559.93427	-126.34590	542.30857	1
O	79.14808	78.19501	68.34680	-670.36003	-111.83166	76.36448	1
N	79.07580	79.95914	69.79091	-216.06729	-537.07037	446.51379	1
H	78.61960	80.60683	70.44390	-1251.96487	1074.12894	-2168.52500	1
H	80.02476	80.31955	69.64438	-2451.83023	-720.73851	944.84612	1
C	74.97570	77.53139	71.79998	546.24052	-563.88337	-85.11809	1
O	73.82906	77.16535	72.04973	71.70702	-307.40463	606.69673	1
N	76.10881	76.89212	72.06025	479.90863	-504.50129	222.86849	1
C	76.19260	75.45433	72.13444	75.51268	-600.91904	506.73700	1
H	75.62850	75.07109	71.25320	-2033.61562	1795.30103	254.89480	1
C	77.75938	75.19288	72.13833	523.79067	517.99274	284.45581	1
H	78.02455	74.19927	72.56997	1599.61665	-1127.00911	1895.98173	1
H	78.19248	75.23230	71.11355	-1881.83093	1275.82488	-1501.55016	1
C	78.25100	76.38899	73.01196	-600.05639	-404.05026	316.64485	1
H	78.03276	76.21548	74.09004	-2597.05260	496.36221	657.73249	1
H	79.34396	76.58604	72.93176	-1666.24518	1294.38985	-1723.90591	1
C	77.35735	77.55673	72.41153	493.57570	-129.95376	-602.56394	1
H	77.08848	78.39904	73.12480	-1469.08415	-637.84797	2204.22581	1
H	77.76807	77.98317	71.46384	-1038.97840	1068.88701	-2280.71789	1
C	75.66689	74.83167	73.53839	188.88100	241.05089	-727.87821	1
O	76.34186	74.01105	74.10583	531.88613	-49.19943	427.07960	1
N	74.46442	75.28991	73.89198	708.14833	26.93417	-179.73509	1
H	73.95394	75.95597	73.25498	-2232.69501	-1179.09831	1023.94816	1
C	73.79679	74.68669	75.05118	-738.32412	259.92548	104.42456	1

H	74.66652	74.29727	75.63499	2028.89123	1260.02983	1311.33324	1
C	72.99891	75.76824	75.75444	-258.65678	-345.52513	661.28459	1
H	72.30657	76.22848	75.01413	-1826.06090	-1801.46097	-918.64554	1
C	72.16982	75.20428	76.89004	-541.23400	531.92970	-218.41428	1
H	71.70079	76.01524	77.51310	1311.73218	-2219.18200	882.19023	1
H	71.33436	74.55221	76.50174	-1048.71298	1223.58118	-2196.97971	1
H	72.80597	74.55132	77.58454	419.06414	1903.92898	1903.44670	1
C	73.91379	76.86375	76.27451	-423.86431	152.66566	-648.55262	1
H	73.33720	77.69261	76.76927	105.68386	-2342.50080	1387.51238	1
H	74.63961	76.46483	77.03522	-1695.63603	-1484.73621	-1531.02793	1
H	74.52230	77.31848	75.44352	-1853.57121	-487.39031	1936.59343	1
C	72.85047	73.44910	74.47549	539.71428	-510.58232	267.58633	1
O	71.65159	73.45213	74.62113	-15.93758	-278.34085	-624.49402	1
N	73.60493	72.53497	73.88208	-676.81886	12.05292	276.17960	1
H	74.61281	72.70783	73.87967	-1694.44250	-373.84189	-2100.66098	1
C	73.03239	71.26158	73.62399	412.33078	-664.70360	108.35451	1
H	71.92973	71.40219	73.71823	1765.77476	1294.44366	1621.76594	1
C	73.55136	70.75841	72.21690	-179.66635	-721.95667	-264.73915	1
H	73.18693	71.41435	71.39471	-607.34922	2587.87032	-598.10018	1
H	74.66298	70.80474	72.17446	-359.69332	-1479.13954	-2259.74260	1
C	73.04910	69.28668	72.04005	-240.01076	616.02425	431.85322	1
H	73.40225	68.66341	72.89517	-1359.17131	782.54425	2227.99181	1
H	71.93725	69.23883	72.05761	-820.36667	85.81767	-2596.78715	1
C	73.61755	68.72175	70.67708	-513.07015	236.18570	-551.87280	1
H	73.24562	69.31631	69.81121	1816.11944	-1183.11722	-1650.94321	1
H	74.72855	68.80305	70.64786	-1800.04429	-1361.53473	1526.34643	1
C	73.17456	67.24400	70.56717	726.08972	-253.19732	-179.64713	1
H	73.58556	66.58039	71.35401	1224.27271	-456.51798	-2390.90304	1
H	72.07975	67.09518	70.48090	1206.40339	-1150.93514	-2154.90344	1
N	73.75824	66.67408	69.23061	329.70796	-633.68968	-155.67358	1
H	73.53013	65.65837	69.02658	1348.31305	1938.76845	1359.00643	1
H	73.44863	67.14182	68.32955	2096.92035	1446.54551	-966.48890	1
H	74.81464	66.67488	69.12686	-1197.07217	-2303.53719	827.28733	1
C	73.68606	70.21778	74.72783	-378.01186	-556.17301	-413.96481	1
O	74.72653	69.64508	74.47987	-409.44433	-307.55053	-453.30833	1
N	72.92934	70.16163	75.80972	-429.02477	524.70884	274.08502	1
H	72.09339	70.74569	75.81508	1706.34263	-1425.96036	1574.38926	1
C	73.25264	69.33305	76.91909	-45.25864	-626.22952	478.93267	1
H	72.27852	69.18180	77.43563	1988.63164	570.90773	1772.87287	1
C	73.89054	68.00670	76.36381	551.77128	-20.49526	-564.54996	1
H	73.32031	67.64504	75.47951	1434.61535	-1753.10253	1513.99471	1
H	74.92651	68.20339	75.98609	-714.67738	1819.09770	1898.36494	1
C	73.92279	66.90161	77.50752	446.99263	-555.77305	338.97221	1
H	74.52061	67.28283	78.37740	1953.68466	1892.25957	-161.67766	1
H	72.89262	66.70640	77.89636	-403.03785	2688.07032	-188.43591	1
C	74.53588	65.64324	76.93243	567.86262	335.74681	434.04421	1
H	73.85530	65.21240	76.16342	-1080.02275	1568.26081	-1948.78900	1
H	75.46800	65.90391	76.38010	-919.54108	2111.34692	1456.13463	1
C	74.84815	64.63624	77.94652	195.81897	173.33862	-745.11536	1
H	75.68859	64.91533	78.61264	1405.67949	-2301.21089	-390.08113	1
H	73.97893	64.29864	78.54682	-940.37328	-1118.32446	2299.72133	1
N	75.35958	63.30937	77.29535	319.26422	656.36007	-42.02424	1
H	75.61331	62.56047	78.00634	1961.03633	619.91968	1787.09422	1
H	74.69347	62.77761	76.66174	854.14343	-2156.80752	1429.08711	1
H	76.24595	63.36101	76.71265	1237.02905	-1378.50857	-1998.28500	1
C	74.32919	70.10267	77.90993	532.53319	-268.57236	517.55729	1
O	74.37511	69.77167	79.07021	266.16714	-290.73804	-558.88002	1
N	75.05556	70.98877	77.25088	-226.25352	640.49678	-270.32839	1
H	74.86259	71.05583	76.24914	2007.69063	-1035.27229	1523.50231	1
C	75.93756	71.91390	77.87228	-540.76542	-573.33303	49.50000	1
H	76.19377	72.59180	77.02693	-2374.20950	-1036.76023	843.75774	1
C	75.14918	72.65957	79.03248	-77.09082	666.87422	-415.84136	1

H	74.78817	71.90089	79.78058	-985.81834	617.19825	2463.91927	1
O	73.93690	73.37493	78.62851	293.29899	-391.65602	-477.80926	1
H	73.26781	72.84283	79.09628	2528.72714	-681.76262	-751.27122	1
C	76.26880	73.55916	79.66026	252.47363	-605.14473	440.05134	1
H	75.91394	74.06429	80.58874	-2052.65063	1456.49051	-1043.51830	1
H	77.17809	72.97141	79.92816	-2152.98800	-1661.97574	-161.70652	1
H	76.59225	74.35718	78.95194	-473.12726	-1584.27339	2165.61725	1
C	77.26611	71.07899	78.36866	360.36307	488.46713	505.10056	1
O	78.13676	70.86486	77.55845	-200.08817	-528.03552	-385.82850	1
N	77.24462	70.74712	79.65520	-597.54095	-226.44775	355.20479	1
H	76.41720	70.93023	80.22134	2179.23655	-1300.70981	-991.33835	1
C	78.36079	70.05570	80.16753	-687.68921	297.39807	-249.45179	1
H	79.05085	69.88508	79.30660	-349.16256	-2467.37713	-1101.72831	1
C	78.97622	70.83147	81.42965	672.47345	361.54328	-201.62880	1
H	78.18492	70.97836	82.20237	1541.41502	582.18425	2169.97026	1
H	79.76281	70.19558	81.89817	-2534.67717	60.25153	997.72295	1
C	79.57982	72.16954	80.98438	574.26774	-389.43119	-377.02542	1
C	80.72702	72.16512	80.13457	688.82679	-287.33580	-257.96133	1
H	81.16755	71.21367	79.78522	331.14182	1470.38163	2269.80013	1
C	79.01118	73.40124	81.42851	-383.52382	-675.04808	144.24917	1
H	78.12318	73.40697	82.08618	4.09847	1517.59662	2262.86397	1
C	81.27957	73.38702	79.75416	-134.76730	428.45285	-649.50303	1
H	82.16779	73.38416	79.09833	-903.23433	1600.49020	2011.53387	1
C	79.60151	74.59907	81.01887	-335.53281	-615.00040	-364.39058	1
H	79.15589	75.54625	81.36924	1596.45398	-1369.33862	1732.02659	1
C	80.75005	74.64123	80.17571	585.48547	-340.24194	-406.23938	1
O	81.35788	75.85341	79.75727	-419.15649	-173.04408	-511.94141	1
H	80.78528	76.49970	80.23394	-1071.84350	1559.71507	-1960.13012	1
C	77.82848	68.71705	80.96735	291.41878	-183.13091	710.72246	1
O	77.48472	68.79323	82.12334	379.87695	523.47396	-222.23622	1
N	77.93439	67.66111	80.17570	564.30591	373.09380	277.24371	1
H	78.17919	67.75427	79.18655	-120.59931	-2436.07824	-1214.35098	1
C	77.39853	66.40598	80.61176	404.07514	-299.37480	608.84009	1
H	77.78998	66.20713	81.63477	-1071.07882	-841.69025	2359.66467	1
C	75.82359	66.59904	80.63130	-683.12807	68.71770	-390.13096	1
H	75.53986	67.41526	81.33489	2068.88918	1596.58590	770.89603	1
H	75.46500	66.93709	79.62846	1379.86429	2342.49779	-179.85638	1
C	75.16891	65.24514	81.04312	-176.39967	537.49466	550.97268	1
H	75.58090	64.40257	80.44367	-396.28051	1942.22168	1869.33314	1
H	75.39779	64.99011	82.10158	-94.44700	586.00678	-2659.20016	1
C	73.59614	65.40736	80.80982	-291.69381	-718.18079	-150.72929	1
H	73.01813	65.82942	81.67074	-2303.38776	1089.76242	964.62032	1
H	73.29473	65.97066	79.89111	884.16729	2568.80533	-207.74283	1
N	73.21761	64.07471	80.61769	-594.49522	-252.81478	342.29276	1
H	73.95760	63.37788	80.70945	-1735.17426	2084.05705	-263.71605	1
C	72.03056	63.70934	80.33115	595.23177	-271.52487	442.22352	1
N	71.05530	64.69856	80.20019	585.53813	-424.79750	-105.81303	1
H	71.14454	65.71005	80.29623	959.88797	1327.56823	-2177.11905	1
H	70.07584	64.53290	79.96408	1578.48647	16.27378	-2220.76233	1
N	72.01407	62.31602	80.20475	-452.17763	-390.33541	-421.51853	1
H	72.79854	61.65592	80.24675	470.26107	1974.25629	-1817.92087	1
H	71.18221	61.77419	79.96326	1856.67307	-1573.97321	-1224.35416	1
C	77.88518	65.37285	79.45319	-531.47271	-581.45780	-55.06206	1
O	78.06604	65.72694	78.31149	-243.00837	192.73507	609.52485	1
N	77.92889	64.13670	79.95746	343.49288	-644.25607	38.09164	1
H	77.75930	64.05158	80.96095	-1691.16043	-1535.97525	-1484.73099	1
C	78.57658	63.04925	79.22930	542.66492	350.14337	454.42563	1
H	78.31510	63.20809	78.15725	1209.57979	-2396.95920	463.86744	1
C	80.06972	63.11394	79.49951	-737.14309	145.93010	242.71942	1
H	80.20629	63.17306	80.60241	-1050.18303	159.36419	-2509.06125	1
C	80.78465	61.87312	78.99711	-706.77696	-252.88343	-245.16230	1
H	81.88517	61.90521	79.22889	-1624.64039	-1665.49085	1417.86935	1

```
H    80.37140   60.93820   79.46668      2566.10521       598.51109       693.22305          1
H    80.68238   61.75868   77.88263     -1972.69758       107.10018     -1876.34312          1
C    80.70088   64.35049   78.88659       412.85148       672.58832       -27.69222          1
H    81.76977   64.48187   79.21224      1447.02277      1762.76533     -1490.78944          1
H    80.69724   64.30091   77.76279      1320.77379      -145.20455      2378.68491          1
H    80.13822   65.28547   79.17110     -1512.10848      1470.52031      1724.75147          1
C    77.90539   61.69435   79.93583      -607.11113       499.56689        73.73769          1
O    78.05467   61.48058   81.11395       357.97126       460.81534       356.68785          1
N    77.35189   60.94382   78.99688       536.55407      -368.62270       332.76306          1
H    77.41651   61.30714   78.04643       524.38866      2297.69985      1367.20941          1
C    76.37861   59.95771   79.31073        46.50277      -774.29190      -147.97904          1
H    76.30469   59.36874   78.36988      1676.51875      1883.29066      1032.55549          1
C    76.83028   59.08389   80.58862      -559.41456       -34.19198      -556.30433          1
H    76.70744   59.71638   81.50321       -64.66664       395.18769      2695.05373          1
H    76.14439   58.21370   80.72531     -2703.44278       -60.40056       333.79418          1
C    78.26265   58.63147   80.48085      -519.76435       377.31045       459.42366          1
H    78.88519   59.53003   80.26034     -1746.74323      1667.50644     -1261.73691          1
C    78.72856   58.04479   81.80808       570.95554        16.46261      -545.27700          1
H    79.80231   57.72749   81.76757      2391.58273        28.05277     -1305.07271          1
H    78.63366   58.78918   82.64067       175.75588       917.63458      2559.43867          1
H    78.12679   57.14488   82.09634     -1063.97851     -2155.34650      1283.00462          1
C    78.46347   57.61188   79.36913       193.76014         2.62946      -765.53141          1
H    79.53072   57.27477   79.30749       507.39394      -846.55087      2539.60089          1
H    77.83647   56.69717   79.52912      1337.57841      1818.19054      1526.01994          1
H    78.19036   58.03345   78.36768      1100.88635     -1172.91350     -2199.08909          1
C    74.99008   60.67670   79.67540      -549.21115       475.64486      -309.38218          1
O    74.87567   61.82714   80.04641       556.06106      -390.37755       -78.24250          1
O    73.84961   59.84866   79.62083      -572.27940      -310.19032      -209.75862          1
H    74.30113   59.00354   79.32060     -1380.08193      2212.14486       790.85944          1
```

Listing 27: C00027.xmolout

```
        276
  150.0000  150.0000  150.0000     0.0000     0.0000     0.0000***********    1.1695     4678
        0.2500    300.00   0.00    0.00    0.00   -1293.9452       0.0000    300.0000
H    66.79296   76.53065   74.29401     -2043.14101     -1757.64893       399.89339          1
N    66.68826   77.51405   74.05073       426.75047       490.45733      -334.42467          1
H    66.09303   77.86696   74.80431       442.25559     -1110.30854     -2448.52907          1
C    67.90228   78.23254   73.97682       415.32972      -666.39213        83.73172          1
H    68.70690   77.53404   73.65516       879.32219      2491.00447       667.35213          1
C    67.67988   79.45501   72.94403        31.35357       477.41777       628.23362          1
H    66.81731   80.07910   73.28054      1168.38489      2240.25337     -1019.71136          1
H    68.57090   80.12832   72.93719      -360.43770      2534.90498       931.67112          1
C    67.42055   78.88057   71.57714       463.18208       512.70242      -382.34388          1
H    66.65934   78.07379   71.67989      1725.29117      1863.52247      -987.07854          1
C    66.86629   79.94534   70.63913       710.42801      -144.27427      -313.15367          1
H    66.65025   79.52843   69.62158       364.20195     -1033.13725      2494.72524          1
H    65.91074   80.38331   71.02781      2567.61904       335.80679       847.48802          1
H    67.58820   80.79153   70.50398     -2587.99029       680.92867      -512.16411          1
C    68.69147   78.28700   70.98406       268.17616       741.64379       -40.42634          1
H    68.51567   77.86976   69.95892      1283.54314     -1945.37420     -1411.27812          1
H    69.50308   79.05501   70.89662     -1258.57796      1317.15249     -2026.02085          1
H    69.09806   77.45582   71.61628     -2502.84526       909.80597       575.92391          1
C    68.10150   78.82359   75.47362       619.36578      -458.64843      -172.09273          1
O    67.17137   79.05395   76.21440       -81.40433       433.87611       522.34594          1
N    69.40044   79.07210   75.65005       175.23996      -622.20190       341.55523          1
H    70.10597   78.84411   74.90991      -876.57256      -303.22879     -2561.90213          1
C    69.79696   79.40022   76.99436      -571.88214      -534.10783      -106.15033          1
H    68.85520   79.37780   77.59525     -2713.20841       -16.25800      -248.80809          1
C    70.82483   78.24824   77.40608       166.60354       288.16399      -716.09568          1
H    71.65144   78.23908   76.63871     -2097.85574      1636.78449      -586.18044          1
O    70.33120   76.87182   77.26504      -317.80580      -363.38227       484.43082          1
```

H	70.09199	76.68464	78.19075	1863.74748	-1451.84923	1357.29467	1
C	71.37065	78.62685	78.81902	503.80731	517.99032	318.51606	1
H	72.17183	77.92163	79.14611	640.61020	-2157.10410	-1536.29104	1
H	71.81270	79.66194	78.83964	1687.88455	465.60692	2087.56452	1
H	70.56879	78.58558	79.59410	-1293.07895	-1899.88581	-1463.57452	1
C	70.46685	80.86167	76.91275	-153.07550	-733.68902	248.70981	1
O	70.86464	81.32214	75.84648	186.15459	-480.78787	-449.34387	1
N	70.57902	81.42432	78.11249	-487.53139	543.18517	42.03850	1
C	71.49434	82.53051	78.26237	-699.35871	69.34393	360.10701	1
H	71.31823	83.18205	77.37602	2.81126	-221.99807	-2715.58072	1
C	71.06078	83.15189	79.65213	-507.46439	-592.59194	122.07761	1
H	71.91652	83.60373	80.20264	-1975.08116	525.87560	-1801.71556	1
H	70.31652	83.96737	79.51080	1588.27743	-1913.22100	1113.83566	1
C	70.42728	81.93668	80.40041	-613.62442	-457.55993	-194.14490	1
H	71.21588	81.26849	80.81314	1707.54637	-721.27992	1996.92523	1
H	69.77127	82.22843	81.25108	758.23357	2100.81767	1560.55011	1
C	69.64065	81.23727	79.21015	-352.41524	704.96076	49.21465	1
H	69.42656	80.15333	79.34685	1714.76886	-2070.42778	443.35792	1
H	68.70427	81.75902	78.90005	1224.15040	1801.50881	1636.97633	1
C	73.01792	81.94971	78.29605	566.85117	537.05831	117.62814	1
O	73.33860	81.01917	79.04289	-550.26779	-406.08867	4.21666	1
N	73.74854	82.63407	77.46825	566.34832	-425.77725	180.18749	1
H	73.27176	83.34659	76.91957	644.44502	2646.80550	-52.73430	1
C	75.15299	82.43141	77.26177	612.34801	-281.48662	411.56221	1
H	75.67757	82.90877	78.11791	1667.23498	-1490.75142	1556.16753	1
C	75.40187	80.85345	77.27182	-714.45746	-175.43074	286.98927	1
H	74.41341	80.37019	77.02618	-787.09484	1763.03619	-1922.46060	1
O	75.75323	80.34471	78.55983	-429.46514	516.51932	-128.40526	1
H	74.81968	80.35849	79.00652	1981.82785	436.16847	-1818.18182	1
C	76.42606	80.60177	76.10988	630.79189	-468.94448	76.03230	1
H	76.63634	79.51485	75.97425	-1775.71861	2062.30353	131.89849	1
H	76.05310	80.99047	75.13208	-1855.14608	88.94118	1993.53760	1
H	77.40244	81.10118	76.31351	-1504.82350	-780.94249	-2132.90992	1
C	75.45023	83.17985	75.85427	288.57638	618.62814	-397.00270	1
O	74.66000	83.13931	74.94055	321.70495	-185.26452	574.37218	1
N	76.70865	83.66095	75.85132	-447.05792	-446.53431	367.76425	1
H	77.21040	83.62096	76.73989	350.31316	1975.63644	-1843.31522	1
C	76.94700	84.73979	74.92831	-511.23061	374.03917	-471.51480	1
H	76.03774	85.37762	74.98130	2234.79579	-898.70890	-1273.45190	1
C	78.27377	85.47286	75.41628	529.53781	-159.34528	563.72604	1
H	78.38264	86.41026	74.80451	654.59388	2347.94251	1217.51488	1
H	78.10576	85.79219	76.47790	-1526.55661	2103.42026	817.87394	1
O	79.51927	84.73141	75.26646	-554.84278	14.12789	399.58769	1
H	79.48651	84.19594	76.07749	1297.06236	2061.84854	1220.68812	1
C	77.14682	84.13419	73.43619	-662.15484	-412.13544	123.62687	1
O	77.02210	84.86252	72.47554	-540.01537	-317.05804	274.91456	1
N	77.51633	82.85279	73.45212	-202.76299	-690.00100	-131.49145	1
H	77.62849	82.39881	74.35731	-702.62132	-2617.37191	281.70413	1
C	77.72509	82.19702	72.21204	-491.73914	576.34187	222.73509	1
H	77.19761	82.82368	71.45601	1977.17978	-1823.58850	-434.68928	1
C	79.30392	82.16587	71.99849	-649.50797	-328.66843	306.11224	1
H	79.80452	81.71374	72.88129	2075.07803	1452.16490	-1004.45904	1
C	79.63031	81.35989	70.69626	-615.18735	-489.99689	-70.96352	1
H	80.71728	81.40259	70.44996	-1226.32661	-1724.38906	-1716.47170	1
H	79.35914	80.28386	70.80699	2381.64273	-969.84735	900.46860	1
H	79.07299	81.75684	69.81555	2122.74991	1702.23946	-141.36228	1
C	79.79675	83.63857	71.79022	-501.86074	-479.61276	-376.42482	1
H	80.89610	83.68773	71.60815	993.79261	-2246.95401	-1177.81305	1
H	79.29055	84.12052	70.92065	42.71279	-626.15391	-2651.37258	1
H	79.57710	84.26721	72.68745	-2026.39012	-1590.33709	-887.82922	1
C	77.17986	80.65770	72.38417	-672.89153	382.57394	-156.34072	1
O	77.62309	79.90863	73.22239	410.04627	221.86223	500.35950	1

92	N	76.30614	80.39316	71.41479	-378.11782	-443.79414	-441.10984	1
93	H	76.00754	81.15957	70.81225	2404.15973	-568.14894	1149.30085	1
94	C	75.54180	79.19047	71.53181	-544.54484	466.16019	331.29119	1
95	H	76.21437	78.50194	72.09564	-1409.80988	-170.17955	2325.32679	1
96	C	74.21834	79.58680	72.31296	-184.48284	116.78282	-758.89137	1
97	H	74.48924	80.17185	73.22276	1893.78641	1861.14652	611.04528	1
98	H	73.60541	80.27745	71.69026	-2170.79665	-974.19311	1327.50117	1
99	C	73.37197	78.32574	72.69030	389.51668	672.81878	-138.49152	1
100	H	73.00037	77.82021	71.76705	510.64577	1933.03327	1851.02484	1
101	H	74.01497	77.57618	73.20919	-2302.30869	-1105.72939	-948.89774	1
102	C	72.16887	78.74111	73.62467	-559.93427	-126.34590	542.30857	1
103	O	71.03846	78.30836	73.49502	-670.36003	-111.83166	76.36448	1
104	N	72.59017	79.60339	74.56056	-216.06729	-537.07037	446.51379	1
105	H	73.53756	80.00397	74.60494	-1251.96487	1074.12894	-2168.52500	1
106	H	71.94371	80.16857	75.17474	-2451.83023	-720.73851	944.84612	1
107	C	75.06601	78.69262	70.05325	546.24052	-563.88337	-85.11809	1
108	O	73.88049	78.68912	69.84457	71.70702	-307.40463	606.69673	1
109	N	76.07192	78.33561	69.23719	479.90863	-504.50129	222.86849	1
110	C	75.84490	78.05046	67.85898	75.51268	-600.91904	506.73700	1
111	H	74.84606	78.46866	67.59303	-2033.61562	1795.30103	254.89480	1
112	C	77.11883	78.71721	67.17679	523.79067	517.99274	284.45581	1
113	H	77.30797	78.32189	66.15158	1599.61665	-1127.00911	1895.98173	1
114	H	77.01156	79.82124	67.08823	-1881.83093	1275.82488	-1501.55016	1
115	C	78.23053	78.32869	68.20135	-600.05639	-404.05026	316.64485	1
116	H	78.54427	77.27008	68.06264	-2597.05260	496.36221	657.73249	1
117	H	79.15346	78.94500	68.11242	-1666.24518	1294.38985	-1723.90591	1
118	C	77.47631	78.53077	69.58378	493.57570	-129.95376	-602.56394	1
119	H	77.74079	77.79691	70.38043	-1469.08415	-637.84797	2204.22581	1
120	H	77.54237	79.56173	69.99966	-1038.97840	1068.88701	-2280.71789	1
121	C	76.03434	76.46117	67.48557	188.88100	241.05089	-727.87821	1
122	O	76.54725	76.18441	66.42832	531.88613	-49.19943	427.07960	1
123	N	75.57319	75.64837	68.41805	708.14833	26.93417	-179.73509	1
124	H	75.10155	75.96812	69.27954	-2232.69501	-1179.09831	1023.94816	1
125	C	75.55868	74.24315	68.15696	-738.32412	259.92548	104.42456	1
126	H	75.47801	74.07785	67.05858	2028.89123	1260.02983	1311.33324	1
127	C	76.89596	73.63372	68.78491	-258.65678	-345.52513	661.28459	1
128	H	77.12199	74.13268	69.75138	-1826.06090	-1801.46097	-918.64554	1
129	C	76.73989	72.08845	68.97854	-541.23400	531.92970	-218.41428	1
130	H	77.66928	71.62807	69.38853	1311.73218	-2219.18200	882.19023	1
131	H	75.91078	71.84692	69.68648	-1048.71298	1223.58118	-2196.97971	1
132	H	76.51221	71.57502	68.01497	419.06414	1903.92898	1903.44670	1
133	C	78.05564	73.85622	67.75768	-423.86431	152.66566	-648.55262	1
134	H	79.04023	73.52703	68.16580	105.68386	-2342.50080	1387.51238	1
135	H	77.87962	73.28961	66.81313	-1695.63603	-1484.73621	-1531.02793	1
136	H	78.14669	74.93512	67.47943	-1853.57121	-487.39031	1936.59343	1
137	C	74.28427	73.82297	69.07007	539.71428	-510.58232	267.58633	1
138	O	73.97555	74.57775	69.99821	-15.93758	-278.34085	-624.49402	1
139	N	73.74687	72.68117	68.69957	-676.81886	12.05292	276.17960	1
140	H	74.19194	72.18510	67.92801	-1694.44250	-373.84189	-2100.66098	1
141	C	72.79768	72.03867	69.55247	412.33078	-664.70360	108.35451	1
142	H	72.19764	72.89654	69.93437	1765.77476	1294.44366	1621.76594	1
143	C	72.01392	70.98468	68.67481	-179.66635	-721.95667	-264.73915	1
144	H	71.57062	71.46526	67.77374	-607.34922	2587.87032	-598.10018	1
145	H	72.71651	70.21224	68.28890	-359.69332	-1479.13954	-2259.74260	1
146	C	70.90751	70.32041	69.56064	-240.01076	616.02425	431.85322	1
147	H	71.34538	69.95758	70.52041	-1359.17131	782.54425	2227.99181	1
148	H	70.11475	71.05259	69.83338	-820.36667	85.81767	-2596.78715	1
149	C	70.31717	69.11386	68.72571	-513.07015	236.18570	-551.87280	1
150	H	69.96006	69.45969	67.72849	1816.11944	-1183.11722	-1650.94321	1
151	H	71.11082	68.35976	68.51783	-1800.04429	-1361.53473	1526.34643	1
152	C	69.15901	68.47513	69.52649	726.08972	-253.19732	-179.64713	1
153	H	69.44070	68.10373	70.53191	1224.27271	-456.51798	-2390.90304	1

H	68.24474	69.09788	69.59424	1206.40339	-1150.93514	-2154.90344	1
N	68.71577	67.19057	68.74698	329.70796	-633.68968	-155.67358	1
H	67.89654	66.65929	69.16113	1348.31305	1938.76845	1359.00643	1
H	68.39720	67.31753	67.74253	2096.92035	1446.54551	-966.48890	1
H	69.43714	66.41765	68.65187	-1197.07217	-2303.53719	827.28733	1
C	73.61208	71.29086	70.76217	-378.01186	-556.17301	-413.96481	1
O	73.59541	70.09433	70.90249	-409.44433	-307.55053	-453.30833	1
N	74.26649	72.21322	71.46960	-429.02477	524.70884	274.08502	1
H	74.12992	73.21754	71.22587	1706.34263	-1425.96036	1574.38926	1
C	74.58055	71.87080	72.82460	-45.25864	-626.22952	478.93267	1
H	74.25608	70.81087	72.95515	1988.63164	570.90773	1772.87287	1
C	76.15112	72.06096	72.98148	551.77128	-20.49526	-564.54996	1
H	76.66895	71.55239	72.13855	1434.61535	-1753.10253	1513.99471	1
H	76.42295	73.13520	72.88578	-714.67738	1819.09770	1898.36494	1
C	76.65652	71.48453	74.34378	446.99263	-555.77305	338.97221	1
H	76.30385	72.10782	75.19554	1953.68466	1892.25957	-161.67766	1
H	76.23847	70.46625	74.51066	-403.03785	2688.07032	-188.43591	1
C	78.23618	71.42591	74.39630	567.86262	335.74681	434.04421	1
H	78.64842	70.78588	73.58297	-1080.02275	1568.26081	-1948.78900	1
H	78.68391	72.43594	74.25224	-919.54108	2111.34692	1456.13463	1
C	78.57415	70.85370	75.79474	195.81897	173.33862	-745.11536	1
H	78.23686	71.48245	76.64630	1405.67949	-2301.21089	-390.08113	1
H	78.25896	69.79947	75.94734	-940.37328	-1118.32446	2299.72133	1
N	80.13155	70.81852	75.94499	319.26422	656.36007	-42.02424	1
H	80.49756	70.35781	76.82785	1961.03633	619.91968	1787.09422	1
H	80.67747	70.30011	75.19829	854.14343	-2156.80752	1429.08711	1
H	80.63470	71.75290	75.97276	1237.02905	-1378.50857	-1998.28500	1
C	73.77522	72.95256	73.71896	532.53319	-268.57236	517.55729	1
O	73.11137	73.87715	73.24055	266.16714	-290.73804	-558.88002	1
N	73.95926	72.71722	75.05073	-226.25352	640.49678	-270.32839	1
H	74.55827	71.87876	75.00521	2007.69063	-1035.27229	1523.50231	1
C	74.46732	73.89108	75.75244	-540.76542	-573.33303	49.50000	1
H	75.31431	74.24118	75.12391	-2374.20950	-1036.76023	843.75774	1
C	73.27240	74.94212	75.68749	-77.09082	666.87422	-415.84136	1
H	72.35952	74.35827	75.36487	-985.81834	617.19825	2463.91927	1
O	73.41231	75.93838	74.66504	293.29899	-391.65602	-477.80926	1
H	73.03985	75.41516	73.86901	2528.72714	-681.76262	-751.27122	1
C	73.08955	75.45320	77.15827	252.47363	-605.14473	440.05134	1
H	72.12986	76.03751	77.27056	-2052.65063	1456.49051	-1043.51830	1
H	73.06306	74.60797	77.88996	-2152.98800	-1661.97574	-161.70652	1
H	73.94208	76.10718	77.46789	-473.12726	-1584.27339	2165.61725	1
C	74.95917	73.49313	77.22730	360.36307	488.46713	505.10056	1
O	75.34257	74.37218	77.97492	-200.08817	-528.03552	-385.82850	1
N	74.91702	72.18312	77.47558	-597.54095	-226.44775	355.20479	1
H	74.56662	71.53383	76.77417	2179.23655	-1300.70981	-991.33835	1
C	75.26177	71.72146	78.76961	-687.68921	297.39807	-249.45179	1
H	75.52903	72.62467	79.36860	-349.16256	-2467.37713	-1101.72831	1
C	73.97117	70.93270	79.32379	672.47345	361.54328	-201.62880	1
H	73.68657	70.11976	78.61815	1541.41502	582.18425	2169.97026	1
H	74.19736	70.45540	80.30412	-2534.67717	60.25153	997.72295	1
C	72.84342	71.96220	79.45574	574.26774	-389.43119	-377.02542	1
C	72.62437	72.63115	80.69855	688.82679	-287.33580	-257.96133	1
H	73.22204	72.37066	81.59058	331.14182	1470.38163	2269.80013	1
C	72.05947	72.31170	78.31310	-383.52382	-675.04808	144.24917	1
H	72.23245	71.81266	77.34277	4.09847	1517.59662	2262.86397	1
C	71.65345	73.63163	80.76258	-134.76730	428.45285	-649.50303	1
H	71.49202	74.14643	81.72573	-903.23433	1600.49020	2011.53387	1
C	71.10075	73.32107	78.42600	-335.53281	-615.00040	-364.39058	1
H	70.51594	73.58857	77.52836	1596.45398	-1369.33862	1732.02659	1
C	70.86349	74.02547	79.64296	585.48547	-340.24194	-406.23938	1
O	69.90227	75.06701	79.75556	-419.15649	-173.04408	-511.94141	1
H	69.55706	75.06953	78.82990	-1071.84350	1559.71507	-1960.13012	1

C	76.47624	70.66162	78.56547	291.41878	-183.13091	710.72246	1
O	76.69826	70.08403	77.49835	379.87695	523.47396	-222.23622	1
N	77.08782	70.51915	79.71878	564.30591	373.09380	277.24371	1
H	76.73309	71.06797	80.49852	-120.59931	-2436.07824	-1214.35098	1
C	78.37325	69.87800	79.75300	404.07514	-299.37480	608.84009	1
H	78.84246	70.21637	78.80085	-1071.07882	-841.69025	2359.66467	1
C	79.11507	70.33696	81.07453	-683.12807	68.71770	-390.13096	1
H	79.21562	71.44493	81.11901	2068.88918	1596.58590	770.89603	1
H	78.52490	70.04826	81.97341	1379.86429	2342.49779	-179.85638	1
C	80.50978	69.63680	81.07843	-176.39967	537.49466	550.97268	1
H	80.38567	68.53389	80.97224	-396.28051	1942.22168	1869.33314	1
H	81.11997	69.95787	80.20500	-94.44700	586.00678	-2659.20016	1
C	81.24378	69.98482	82.45329	-291.69381	-718.18079	-150.72929	1
H	81.52287	71.05781	82.61039	-2303.38776	1089.76242	964.62032	1
H	80.72931	69.61770	83.37810	884.16729	2568.80533	-207.74283	1
N	82.42000	69.23494	82.28955	-594.49522	-252.81478	342.29276	1
H	82.46160	68.62910	81.48415	-1735.17426	2084.05705	-263.71605	1
C	83.41433	69.25292	83.08222	595.23177	-271.52487	442.22352	1
N	83.34288	70.08687	84.20234	585.53813	-424.79750	-105.81303	1
H	82.58452	70.69710	84.50456	959.88797	1327.56823	-2177.11905	1
H	84.07078	70.17995	84.91155	1578.48647	16.27378	-2220.76233	1
N	84.41958	68.38444	82.63435	-452.17763	-390.33541	-421.51853	1
H	84.45427	67.76800	81.82376	470.26107	1974.25629	-1817.92087	1
H	85.29948	68.24093	83.13087	1856.67307	-1573.97321	-1224.35416	1
C	78.14011	68.27108	79.76136	-531.47271	-581.45780	-55.06206	1
O	78.31598	67.59294	80.73430	-243.00837	192.73507	609.52485	1
N	77.83954	67.87611	78.49768	343.49288	-644.25607	38.09164	1
H	77.58041	68.63474	77.80954	-1691.16043	-1535.97525	-1484.73099	1
C	77.16706	66.56642	78.35575	542.66492	350.14337	454.42563	1
H	77.48666	66.03306	79.28304	1209.57979	-2396.95920	463.86744	1
C	75.67537	66.79559	78.26551	-737.14309	145.93010	242.71942	1
H	75.48519	67.44673	77.38194	-1050.18303	159.36419	-2509.06125	1
C	74.92398	65.49142	78.06525	-706.77696	-252.88343	-245.16230	1
H	73.81392	65.65585	77.99237	-1624.64039	-1665.49085	1417.86935	1
H	75.24994	64.97301	77.11895	2566.10521	598.51109	693.22305	1
H	75.10108	64.77636	78.91519	-1972.69758	107.10018	-1876.34312	1
C	75.13493	67.51813	79.48723	412.85148	672.58832	-27.69222	1
H	74.03320	67.72523	79.40024	1447.02277	1762.76533	-1490.78944	1
H	75.29071	66.91565	80.42412	1320.77379	-145.20455	2378.68491	1
H	75.65290	68.50505	79.64117	-1512.10848	1470.52031	1724.75147	1
C	77.78535	65.91330	76.97058	-607.11113	499.56689	73.73769	1
O	77.09255	65.69718	76.00110	357.97126	460.81534	356.68785	1
N	79.08125	65.68767	77.12113	536.55407	-368.62270	332.76306	1
H	79.50872	65.94178	78.01117	524.38866	2297.69985	1367.20941	1
C	79.76567	64.93490	76.13650	46.50277	-774.29190	-147.97904	1
H	78.96240	64.47385	75.51711	1676.51875	1883.29066	1032.55549	1
C	80.72575	65.91458	75.27888	-559.41456	-34.19198	-556.30433	1
H	81.49362	66.35933	75.95488	-64.66664	395.18769	2695.05373	1
H	81.29304	65.32742	74.51818	-2703.44278	-60.40056	333.79418	1
C	79.91910	67.00052	74.62175	-519.76435	377.31045	459.42366	1
H	79.35767	67.54874	75.41264	-1746.74323	1667.50644	-1261.73691	1
C	80.84203	67.98177	73.90866	570.95554	16.46261	-545.27700	1
H	80.26906	68.82518	73.44425	2391.58273	28.05277	-1305.07271	1
H	81.58927	68.43121	74.61188	175.75588	917.63458	2559.43867	1
H	81.41958	67.48222	73.08896	-1063.97851	-2155.34650	1283.00462	1
C	78.91452	66.42908	73.63153	193.76014	2.62946	-765.53141	1
H	78.42190	67.23594	73.02964	507.39394	-846.55087	2539.60089	1
H	79.39853	65.72339	72.90742	1337.57841	1818.19054	1526.01994	1
H	78.09347	65.86701	74.16532	1100.88635	-1172.91350	-2199.08909	1
C	80.73083	63.90316	76.91324	-549.21115	475.64486	-309.38218	1
O	80.98923	63.99213	78.08502	556.06106	-390.37755	-78.24250	1
O	81.35637	62.91955	76.10980	-572.27940	-310.19032	-209.75862	1

```
H    80.93093  63.14148  75.23619    -1380.08193      2212.14486       790.85944          1
```

Listing 28: C00028.xmolout

```
      276
  150.0000  150.0000  150.0000     0.0000     0.0000     0.0000**********    0.9687     3875
      0.2500    300.00   0.00   0.00   0.00  -1293.9439       0.0000    300.0000
H    65.84523  73.21692  90.01490    -2043.14101     -1757.64893       399.89339          1
N    66.67251  72.67887  90.26539      426.75047       490.45733      -334.42467          1
H    66.50239  71.80438  89.77243      442.25559     -1110.30854     -2448.52907          1
C    67.88179  73.31427  89.90043      415.32972      -666.39213        83.73172          1
H    67.71398  74.34169  90.28740      879.32219      2491.00447       667.35213          1
C    69.11706  72.57614  90.61870       31.35357       477.41777       628.23362          1
H    69.21220  71.53672  90.22135     1168.38489      2240.25337     -1019.71136          1
H    70.07717  73.09308  90.37503     -360.43770      2534.90498       931.67112          1
C    68.89394  72.54637  92.10968      463.18208       512.70242      -382.34388          1
H    67.87822  72.13142  92.29992     1725.29117      1863.52247      -987.07854          1
C    69.92247  71.64489  92.78070      710.42801      -144.27427      -313.15367          1
H    69.75887  71.58218  93.88754      364.20195     -1033.13725      2494.72524          1
H    69.88294  70.59940  92.37929     2567.61904       335.80679       847.48802          1
H    70.96578  72.02173  92.62156    -2587.99029       680.92867      -512.16411          1
C    68.97078  73.94080  92.71553      268.17616       741.64379       -40.42634          1
H    68.83830  73.91563  93.82812     1283.54314     -1945.37420     -1411.27812          1
H    69.95982  74.42630  92.51043    -1258.57796      1317.15249     -2026.02085          1
H    68.17740  74.61652  92.30442    -2502.84526       909.80597       575.92391          1
C    67.91901  73.30041  88.27523      619.36578      -458.64843      -172.09273          1
O    66.97344  73.69005  87.62379      -81.40433       433.87611       522.34594          1
N    69.10286  72.90122  87.81355      175.23996      -622.20190       341.55523          1
H    69.82762  72.56132  88.44153     -876.57256      -303.22879     -2561.90213          1
C    69.25252  72.85674  86.39121     -571.88214      -534.10783      -106.15033          1
H    68.59261  73.67315  86.01097    -2713.20841       -16.25800      -248.80809          1
C    70.81817  73.01453  86.07647      166.60354       288.16399      -716.09568          1
H    71.35281  72.13591  86.53157    -2097.85574      1636.78449      -586.18044          1
O    71.44795  74.18856  86.61352     -317.80580      -363.38227       484.43082          1
H    72.11295  73.78874  87.21068     1863.74748     -1451.84923      1357.29467          1
C    70.85734  72.87520  84.51045      503.80731       517.99032       318.51606          1
H    71.90524  72.84395  84.13171      640.61020     -2157.10410     -1536.29104          1
H    70.35269  71.94206  84.15667     1687.88455       465.60692      2087.56452          1
H    70.35264  73.73446  84.01107    -1293.07895     -1899.88581     -1463.57452          1
C    68.82029  71.37759  85.88496     -153.07550      -733.68902       248.70981          1
O    69.29435  70.37407  86.40068      186.15459      -480.78787      -449.34387          1
N    68.03051  71.47187  84.81905     -487.53139       543.18517        42.03850          1
C    67.65608  70.30977  84.06170     -699.35871        69.34393       360.10701          1
H    67.40250  69.51011  84.79354        2.81126      -221.99807     -2715.58072          1
C    66.44987  70.84709  83.18617     -507.46439      -592.59194       122.07761          1
H    66.29479  70.25511  82.25582    -1975.08116       525.87560     -1801.71556          1
H    65.48903  70.81787  83.74736     1588.27743     -1913.22100      1113.83566          1
C    66.90471  72.31477  82.91435     -613.62442      -457.55993      -194.14490          1
H    67.72456  72.33997  82.16010     1707.54637      -721.27992      1996.92523          1
H    66.09244  72.97705  82.53817      758.23357      2100.81767      1560.55011          1
C    67.43778  72.72086  84.35404     -352.41524       704.96076        49.21465          1
H    68.21327  73.52324  84.36465     1714.76886     -2070.42778       443.35792          1
H    66.64063  72.96830  85.09612     1224.15040      1801.50881      1636.97633          1
C    68.91445  69.90498  83.09512      566.85117       537.05831       117.62814          1
O    69.50829  70.71192  82.42428     -550.26779      -406.08867         4.21666          1
N    68.99832  68.56750  83.07922      566.34832      -425.77725       180.18749          1
H    68.34962  68.08307  83.70272      644.44502      2646.80550       -52.73430          1
C    70.31342  68.00324  82.97987      612.34801      -281.48662       411.56221          1
H    70.89187  68.67296  82.30562     1667.23498     -1490.75142      1556.16753          1
C    70.88242  67.97254  84.47332     -714.45746      -175.43074       286.98927          1
H    70.04955  67.64402  85.15704     -787.09484      1763.03619     -1922.46060          1
O    71.31822  69.22831  84.99501     -429.46514       516.51932      -128.40526          1
```

H	70.55379	69.47595	85.60710	1981.82785	436.16847	-1818.18182	1
C	71.96517	66.83934	84.38507	630.79189	-468.94448	76.03230	1
H	72.44163	66.65324	85.37582	-1775.71861	2062.30353	131.89849	1
H	71.52268	65.87137	84.04322	-1855.14608	88.94118	1993.53760	1
H	72.77816	67.10561	83.66975	-1504.82350	-780.94249	-2132.90992	1
C	70.04025	66.50487	82.40400	288.57638	618.62814	-397.00270	1
O	69.47135	65.66396	83.05651	321.70495	-185.26452	574.37218	1
N	70.55567	66.39431	81.15779	-447.05792	-446.53431	367.76425	1
H	70.98072	67.25616	80.80399	350.31316	1975.63644	-1843.31522	1
C	69.64008	65.75211	80.22783	-511.23061	374.03917	-471.51480	1
H	69.55266	64.70705	80.59991	2234.79579	-898.70890	-1273.45190	1
C	68.28351	66.55493	80.34966	529.53781	-159.34528	563.72604	1
H	67.54706	66.10003	79.64067	654.59388	2347.94251	1217.51488	1
H	67.87270	66.40797	81.38050	-1526.55661	2103.42026	817.87394	1
O	68.35844	68.02187	80.05130	-554.84278	14.12789	399.58769	1
H	68.59845	68.32878	80.93855	1297.06236	2061.84854	1220.68812	1
C	70.32449	65.86441	78.75959	-662.15484	-412.13544	123.62687	1
O	70.75214	64.89145	78.19708	-540.01537	-317.05804	274.91456	1
N	70.27393	67.12971	78.32080	-202.76299	-690.00100	-131.49145	1
H	69.84193	67.84313	78.93553	-702.62132	-2617.37191	281.70413	1
C	70.98867	67.51320	77.10619	-491.73914	576.34187	222.73509	1
H	70.95784	66.60349	76.46006	1977.17978	-1823.58850	-434.68928	1
C	70.26847	68.72232	76.53963	-649.50797	-328.66843	306.11224	1
H	70.38584	69.55926	77.26472	2075.07803	1452.16490	-1004.45904	1
C	70.84926	69.16336	75.21010	-615.18735	-489.99689	-70.96352	1
H	70.28003	70.03200	74.77816	-1226.32661	-1724.38906	-1716.47170	1
H	71.93558	69.49406	75.30855	2381.64273	-969.84735	900.46860	1
H	70.81799	68.33551	74.44931	2122.74991	1702.23946	-141.36228	1
C	68.77904	68.46454	76.38395	-501.86074	-479.61276	-376.42482	1
H	68.23374	69.37462	76.01236	993.79261	-2246.95401	-1177.81305	1
H	68.57879	67.63348	75.65390	42.71279	-626.15391	-2651.37258	1
H	68.30857	68.16677	77.36176	-2026.39012	-1590.33709	-887.82922	1
C	72.54141	67.91236	77.55967	-672.89153	382.57394	-156.34072	1
O	72.82144	68.32620	78.65787	410.04627	221.86223	500.35950	1
N	73.34899	67.72595	76.51930	-378.11782	-443.79414	-441.10984	1
H	72.87925	67.45329	75.65920	2404.15973	-568.14894	1149.30085	1
C	74.61697	68.40065	76.53814	-544.54484	466.16019	331.29119	1
H	75.04440	68.18009	77.54031	-1409.80988	-170.17955	2325.32679	1
C	75.44586	67.85847	75.30430	-184.48284	116.78282	-758.89137	1
H	75.53009	66.74822	75.32311	1893.78641	1861.14652	611.04528	1
H	74.92016	68.11092	74.35534	-2170.79665	-974.19311	1327.50117	1
C	76.84976	68.53986	75.35820	389.51668	672.81878	-138.49152	1
H	76.74330	69.61868	75.60668	510.64577	1933.03327	1851.02484	1
H	77.44487	68.12768	76.20201	-2302.30869	-1105.72939	-948.89774	1
C	77.65579	68.40244	74.01177	-559.93427	-126.34590	542.30857	1
O	78.66856	69.03834	73.86138	-670.36003	-111.83166	76.36448	1
N	77.10845	67.54850	73.12085	-216.06729	-537.07037	446.51379	1
H	76.27659	66.95416	73.21458	-1251.96487	1074.12894	-2168.52500	1
H	77.53415	67.31139	72.21808	-2451.83023	-720.73851	944.84612	1
C	74.35064	69.98451	76.26895	546.24052	-563.88337	-85.11809	1
O	73.74891	70.32047	75.27318	71.70702	-307.40463	606.69673	1
N	74.94314	70.75070	77.19008	479.90863	-504.50129	222.86849	1
C	74.58150	72.13478	77.30913	75.51268	-600.91904	506.73700	1
H	73.48085	72.14535	77.14560	-2033.61562	1795.30103	254.89480	1
C	75.03822	72.49997	78.77866	523.79067	517.99274	284.45581	1
H	75.34673	73.56503	78.87876	1599.61665	-1127.00911	1895.98173	1
H	74.20367	72.35003	79.50006	-1881.83093	1275.82488	-1501.55016	1
C	76.20244	71.49989	79.07098	-600.05639	-404.05026	316.64485	1
H	77.16488	71.85427	78.63901	-2597.05260	496.36221	657.73249	1
H	76.38789	71.33737	80.15674	-1666.24518	1294.38985	-1723.90591	1
C	75.66516	70.20158	78.33169	493.57570	-129.95376	-602.56394	1
H	76.45507	69.50784	77.95901	-1469.08415	-637.84797	2204.22581	1

H	74.90085	69.62455	78.90822	-1038.97840	1068.88701	-2280.71789	1
C	75.31557	73.06441	76.17523	188.88100	241.05089	-727.87821	1
O	74.64018	73.94294	75.63562	531.88613	-49.19943	427.07960	1
N	76.60203	72.81795	76.05984	708.14833	26.93417	-179.73509	1
H	76.84942	72.02847	76.65591	-2232.69501	-1179.09831	1023.94816	1
C	77.41325	73.22326	74.96804	-738.32412	259.92548	104.42456	1
H	78.32869	72.60114	75.08317	2028.89123	1260.02983	1311.33324	1
C	76.60322	72.91850	73.63206	-258.65678	-345.52513	661.28459	1
H	75.65685	73.50575	73.61629	-1826.06090	-1801.46097	-918.64554	1
C	77.53240	73.28993	72.42691	-541.23400	531.92970	-218.41428	1
H	77.02628	73.12188	71.44752	1311.73218	-2219.18200	882.19023	1
H	77.84169	74.36168	72.46401	-1048.71298	1223.58118	-2196.97971	1
H	78.46421	72.67649	72.42946	419.06414	1903.92898	1903.44670	1
C	76.28425	71.38800	73.54276	-423.86431	152.66566	-648.55262	1
H	75.78016	71.12679	72.58265	105.68386	-2342.50080	1387.51238	1
H	77.21394	70.77088	73.61066	-1695.63603	-1484.73621	-1531.02793	1
H	75.60182	71.06753	74.37067	-1853.57121	-487.39031	1936.59343	1
C	77.75448	74.82380	74.95416	539.71428	-510.58232	267.58633	1
O	78.92795	75.15417	74.88496	-15.93758	-278.34085	-624.49402	1
N	76.67061	75.55213	74.94219	-676.81886	12.05292	276.17960	1
H	75.73009	75.07945	75.01449	-1694.44250	-373.84189	-2100.66098	1
C	76.68992	76.97307	74.98454	412.33078	-664.70360	108.35451	1
H	75.62070	77.21570	75.18090	1765.77476	1294.44366	1621.76594	1
C	77.65865	77.43570	76.15391	-179.66635	-721.95667	-264.73915	1
H	77.40027	76.91510	77.10297	-607.34922	2587.87032	-598.10018	1
H	78.71601	77.15593	75.94004	-359.69332	-1479.13954	-2259.74260	1
C	77.50280	78.98448	76.29455	-240.01076	616.02425	431.85322	1
H	77.88777	79.50829	75.38700	-1359.17131	782.54425	2227.99181	1
H	76.42825	79.26352	76.37508	-820.36667	85.81767	-2596.78715	1
C	78.28972	79.44362	77.58802	-513.07015	236.18570	-551.87280	1
H	77.92574	78.90120	78.49065	1816.11944	-1183.11722	-1650.94321	1
H	79.37444	79.20232	77.50497	-1800.04429	-1361.53473	1526.34643	1
C	78.06817	80.96726	77.73173	726.08972	-253.19732	-179.64713	1
H	78.55718	81.57822	76.94509	1224.27271	-456.51798	-2390.90304	1
H	77.00700	81.26867	77.83976	1206.40339	-1150.93514	-2154.90344	1
N	78.73005	81.41693	79.07745	329.70796	-633.68968	-155.67358	1
H	78.62621	82.44488	79.31790	1348.31305	1938.76845	1359.00643	1
H	78.37239	80.96075	79.96706	2096.92035	1446.54551	-966.48890	1
H	79.77913	81.28593	79.17037	-1197.07217	-2303.53719	827.28733	1
C	77.18443	77.62148	73.56332	-378.01186	-556.17301	-413.96481	1
O	76.39592	78.34424	72.97226	-409.44433	-307.55053	-453.30833	1
N	78.41314	77.28349	73.27567	-429.02477	524.70884	274.08502	1
H	78.91713	76.62403	73.91350	1706.34263	-1425.96036	1574.38926	1
C	79.02221	77.64074	72.04377	-45.25864	-626.22952	478.93267	1
H	79.95122	77.02843	72.05335	1988.63164	570.90773	1772.87287	1
C	78.03124	77.23889	70.87702	551.77128	-20.49526	-564.54996	1
H	77.68055	76.19403	71.03212	1434.61535	-1753.10253	1513.99471	1
H	77.10704	77.86206	70.89243	-714.67738	1819.09770	1898.36494	1
C	78.77673	77.38125	69.50931	446.99263	-555.77305	338.97221	1
H	78.63435	78.41424	69.06351	1953.68466	1892.25957	-161.67766	1
H	79.87431	77.24765	69.64268	-403.03785	2688.07032	-188.43591	1
C	78.20152	76.26164	68.55507	567.86262	335.74681	434.04421	1
H	78.24402	75.26133	69.04415	-1080.02275	1568.26081	-1948.78900	1
H	77.12580	76.44392	68.32919	-919.54108	2111.34692	1456.13463	1
C	79.06252	76.27062	67.26505	195.81897	173.33862	-745.11536	1
H	79.00645	77.21155	66.67585	1405.67949	-2301.21089	-390.08113	1
H	80.12429	75.98247	67.41137	-940.37328	-1118.32446	2299.72133	1
N	78.48713	75.24239	66.26476	319.26422	656.36007	-42.02424	1
H	79.18507	74.53599	65.89492	1961.03633	619.91968	1787.09422	1
H	77.69028	74.60456	66.55464	854.14343	-2156.80752	1429.08711	1
H	78.11774	75.76301	65.33204	1237.02905	-1378.50857	-1998.28500	1
C	79.38260	79.25281	72.00226	532.53319	-268.57236	517.55729	1

182	O	80.54658	79.53680	71.89716	266.16714	-290.73804	-558.88002	1
183	N	78.30467	80.01209	72.05671	-226.25352	640.49678	-270.32839	1
184	H	77.37314	79.54660	72.18250	2007.69063	-1035.27229	1523.50231	1
185	C	78.36937	81.42169	72.18228	-540.76542	-573.33303	49.50000	1
186	H	77.31557	81.67219	72.43540	-2374.20950	-1036.76023	843.75774	1
187	C	79.40222	81.81818	73.33007	-77.09082	666.87422	-415.84136	1
188	H	80.43151	81.51005	72.99319	-985.81834	617.19825	2463.91927	1
189	O	79.18793	81.20661	74.62014	293.29899	-391.65602	-477.80926	1
190	H	79.97441	80.62790	74.67713	2528.72714	-681.76262	-751.27122	1
191	C	79.28879	83.38720	73.31272	252.47363	-605.14473	440.05134	1
192	H	80.03078	83.85668	73.99895	-2052.65063	1456.49051	-1043.51830	1
193	H	79.46894	83.80459	72.29383	-2152.98800	-1661.97574	-161.70652	1
194	H	78.27793	83.72764	73.63626	-473.12726	-1584.27339	2165.61725	1
195	C	78.83212	82.06832	70.73496	360.36307	488.46713	505.10056	1
196	O	79.96851	82.42790	70.53949	-200.08817	-528.03552	-385.82850	1
197	N	77.75874	82.19371	69.96888	-597.54095	-226.44775	355.20479	1
198	H	76.88571	81.82265	70.34208	2179.23655	-1300.70981	-991.33835	1
199	C	77.85577	82.55514	68.59944	-687.68921	297.39807	-249.45179	1
200	H	76.79055	82.71485	68.32238	-349.16256	-2467.37713	-1101.72831	1
201	C	78.74804	83.87410	68.43781	672.47345	361.54328	-201.62880	1
202	H	79.80430	83.65132	68.72736	1541.41502	582.18425	2169.97026	1
203	H	78.76242	84.19435	67.36998	-2534.67717	60.25153	997.72295	1
204	C	78.18834	85.00537	69.31267	574.26774	-389.43119	-377.02542	1
205	C	76.91441	85.58015	69.02371	688.82679	-287.33580	-257.96133	1
206	H	76.30885	85.21565	68.17422	331.14182	1470.38163	2269.80013	1
207	C	78.96639	85.48221	70.41013	-383.52382	-675.04808	144.24917	1
208	H	79.95379	85.03670	70.63010	4.09847	1517.59662	2262.86397	1
209	C	76.45219	86.61372	69.83775	-134.76730	428.45285	-649.50303	1
210	H	75.46775	87.06133	69.61554	-903.23433	1600.49020	2011.53387	1
211	C	78.46007	86.51961	71.19657	-335.53281	-615.00040	-364.39058	1
212	H	79.07083	86.88095	72.04242	1596.45398	-1369.33862	1732.02659	1
213	C	77.19394	87.12343	70.94280	585.48547	-340.24194	-406.23938	1
214	O	76.66922	88.17801	71.73455	-419.15649	-173.04408	-511.94141	1
215	H	77.41894	88.30318	72.36299	-1071.84350	1559.71507	-1960.13012	1
216	C	78.49985	81.28861	67.80494	291.41878	-183.13091	710.72246	1
217	O	78.34028	80.14941	68.21153	379.87695	523.47396	-222.23622	1
218	N	79.07723	81.70528	66.69638	564.30591	373.09380	277.24371	1
219	H	79.07475	82.70699	66.51860	-120.59931	-2436.07824	-1214.35098	1
220	C	79.66904	80.84590	65.72260	404.07514	-299.37480	608.84009	1
221	H	80.52125	81.44973	65.33693	-1071.07882	-841.69025	2359.66467	1
222	C	80.11991	79.52455	66.47368	-683.12807	68.71770	-390.13096	1
223	H	80.48178	79.80623	67.49108	2068.88918	1596.58590	770.89603	1
224	H	79.22296	78.88283	66.65048	1379.86429	2342.49779	-179.85638	1
225	C	81.22586	78.71823	65.73104	-176.39967	537.49466	550.97268	1
226	H	80.79861	77.85958	65.16569	-396.28051	1942.22168	1869.33314	1
227	H	81.75888	79.34379	64.98141	-94.44700	586.00678	-2659.20016	1
228	C	82.23196	78.22565	66.88145	-291.69381	-718.18079	-150.72929	1
229	H	82.90801	79.04673	67.23532	-2303.38776	1089.76242	964.62032	1
230	H	81.76650	77.70121	67.75364	884.16729	2568.80533	-207.74283	1
231	N	82.98261	77.31282	66.13786	-594.49522	-252.81478	342.29276	1
232	H	83.29410	77.62980	65.23046	-1735.17426	2084.05705	-263.71605	1
233	C	83.32744	76.14547	66.52111	595.23177	-271.52487	442.22352	1
234	N	82.92337	75.71513	67.78672	585.53813	-424.79750	-105.81303	1
235	H	82.41885	76.22650	68.51042	959.88797	1327.56823	-2177.11905	1
236	H	83.18063	74.82325	68.21151	1578.48647	16.27378	-2220.76233	1
237	N	84.08709	75.51463	65.53021	-452.17763	-390.33541	-421.51853	1
238	H	84.38188	75.83812	64.60968	470.26107	1974.25629	-1817.92087	1
239	H	84.47585	74.57610	65.63071	1856.67307	-1573.97321	-1224.35416	1
240	C	78.56556	80.61343	64.55231	-531.47271	-581.45780	-55.06206	1
241	O	77.51034	81.20306	64.48667	-243.00837	192.73507	609.52485	1
242	N	79.08860	79.78764	63.62069	343.49288	-644.25607	38.09164	1
243	H	79.95164	79.33396	63.91532	-1691.16043	-1535.97525	-1484.73099	1

```
C    78.09440   79.01643   62.90920       542.66492       350.14337       454.42563           1
H    77.31823   79.75547   62.60697      1209.57979     -2396.95920       463.86744           1
C    78.81973   78.26692   61.71683      -737.14309       145.93010       242.71942           1
H    79.53920   77.51924   62.11599     -1050.18303       159.36419     -2509.06125           1
C    77.70665   77.57134   60.85926      -706.77696      -252.88343      -245.16230           1
H    78.14178   76.99785   60.00752     -1624.64039     -1665.49085      1417.86935           1
H    77.10695   76.85562   61.47074      2566.10521       598.51109       693.22305           1
H    76.99619   78.31585   60.42948     -1972.69758       107.10018     -1876.34312           1
C    79.56354   79.30115   60.80329       412.85148       672.58832       -27.69222           1
H    80.05322   78.80538   59.93228      1447.02277      1762.76533     -1490.78944           1
H    78.86220   80.06895   60.40060      1320.77379      -145.20455      2378.68491           1
H    80.35731   79.84376   61.36863     -1512.10848      1470.52031      1724.75147           1
C    77.56212   77.90907   63.96746      -607.11113       499.56689        73.73769           1
O    78.32668   77.08889   64.49948       357.97126       460.81534       356.68785           1
N    76.26349   78.00837   64.09505       536.55407      -368.62270       332.76306           1
H    75.77833   78.71155   63.53732       524.38866      2297.69985      1367.20941           1
C    75.67574   77.51243   65.29610        46.50277      -774.29190      -147.97904           1
H    76.50784   77.50095   66.03415      1676.51875      1883.29066      1032.55549           1
C    74.43223   78.46515   65.66767      -559.41456       -34.19198      -556.30433           1
H    73.72848   78.50166   64.80126       -64.66664       395.18769      2695.05373           1
H    73.84991   78.03806   66.51973     -2703.44278       -60.40056       333.79418           1
C    74.93862   79.84628   66.00222      -519.76435       377.31045       459.42366           1
H    75.75048   80.12286   65.28599     -1746.74323      1667.50644     -1261.73691           1
C    73.81602   80.86656   65.86707       570.95554        16.46261      -545.27700           1
H    74.16948   81.90357   66.10210      2391.58273        28.05277     -1305.07271           1
H    73.39902   80.88742   64.82682       175.75588       917.63458      2559.43867           1
H    72.96525   80.63904   66.55991     -1063.97851     -2155.34650      1283.00462           1
C    75.51066   79.87861   67.41242       193.76014         2.62946      -765.53141           1
H    75.86974   80.90311   67.68795       507.39394      -846.55087      2539.60089           1
H    74.75083   79.57500   68.17848      1337.57841      1818.19054      1526.01994           1
H    76.38768   79.18592   67.51862      1100.88635     -1172.91350     -2199.08909           1
C    75.10805   76.03329   64.98401      -549.21115       475.64486      -309.38218           1
O    74.28584   75.80077   64.13853       556.06106      -390.37755       -78.24250           1
O    75.55607   75.02106   65.86626      -572.27940      -310.19032      -209.75862           1
H    76.26294   75.52927   66.34885     -1380.08193      2212.14486       790.85944           1
```

Listing 29: C00029.xmolout

```
      276
  150.0000  150.0000  150.0000     0.0000     0.0000     0.0000***********    2.7750    11100
       0.2500    300.00    0.00    0.00    0.00   -1293.9222       0.0000     300.0000
H    73.83440   70.01963   87.99284     -2043.14101     -1757.64893       399.89339           1
N    74.78120   69.79032   87.69688       426.75047       490.45733      -334.42467           1
H    75.14626   69.30328   88.51221       442.25559     -1110.30854     -2448.52907           1
C    75.54966   70.88451   87.25121       415.32972      -666.39213        83.73172           1
H    74.82964   71.51831   86.69253       879.32219      2491.00447       667.35213           1
C    76.19753   71.66164   88.50589        31.35357       477.41777       628.23362           1
H    76.70398   70.93351   89.18436      1168.38489      2240.25337     -1019.71136           1
H    76.99833   72.34949   88.14062      -360.43770      2534.90498       931.67112           1
C    75.13385   72.43230   89.24405       463.18208       512.70242      -382.34388           1
H    74.29215   71.74408   89.48125      1725.29117      1863.52247      -987.07854           1
C    75.69353   72.97892   90.55172       710.42801      -144.27427      -313.15367           1
H    74.91991   73.55142   91.12576       364.20195     -1033.13725      2494.72524           1
H    76.06501   72.15898   91.21915      2567.61904       335.80679       847.48802           1
H    76.55375   73.67422   90.37108     -2587.99029       680.92867      -512.16411           1
C    74.60375   73.58495   88.40182       268.17616       741.64379       -40.42634           1
H    73.85450   74.19836   88.96568      1283.54314     -1945.37420     -1411.27812           1
H    75.42802   74.27603   88.08688     -1258.57796      1317.15249     -2026.02085           1
H    74.09974   73.22507   87.46851     -2502.84526       909.80597       575.92391           1
C    76.78977   70.38921   86.33298       619.36578      -458.64843      -172.09273           1
O    77.64726   71.19038   86.01652       -81.40433       433.87611       522.34594           1
N    76.69998   69.10797   85.96637       175.23996      -622.20190       341.55523           1
```

H	75.89340	68.57875	86.29661	-876.57256	-303.22879	-2561.90213	1
C	77.63604	68.60542	85.02724	-571.88214	-534.10783	-106.15033	1
H	78.39594	69.41934	84.93387	-2713.20841	-16.25800	-248.80809	1
C	78.18424	67.19936	85.56670	166.60354	288.16399	-716.09568	1
H	77.33681	66.46044	85.51265	-2097.85574	1636.78449	-586.18044	1
O	78.66401	67.19451	86.92187	-317.80580	-363.38227	484.43082	1
H	78.03126	66.58075	87.34652	1863.74748	-1451.84923	1357.29467	1
C	79.25741	66.82810	84.47785	503.80731	517.99032	318.51606	1
H	79.68677	65.81562	84.66027	640.61020	-2157.10410	-1536.29104	1
H	78.82104	66.82371	83.44988	1687.88455	465.60692	2087.56452	1
H	80.10572	67.55138	84.47856	-1293.07895	-1899.88581	-1463.57452	1
C	76.81650	68.25862	83.65244	-153.07550	-733.68902	248.70981	1
O	76.55261	67.10302	83.44458	186.15459	-480.78787	-449.34387	1
N	76.48598	69.34624	82.94217	-487.53139	543.18517	42.03850	1
C	75.99671	69.24300	81.60262	-699.35871	69.34393	360.10701	1
H	75.61298	68.20761	81.44860	2.81126	-221.99807	-2715.58072	1
C	74.90877	70.41137	81.61574	-507.46439	-592.59194	122.07761	1
H	74.59098	70.71984	80.59533	-1975.08116	525.87560	-1801.71556	1
H	73.98849	70.10640	82.16131	1588.27743	-1913.22100	1113.83566	1
C	75.68374	71.52481	82.38212	-613.62442	-457.55993	-194.14490	1
H	76.43090	72.01548	81.71416	1707.54637	-721.27992	1996.92523	1
H	75.02968	72.33232	82.78348	758.23357	2100.81767	1560.55011	1
C	76.41108	70.68369	83.51652	-352.41524	704.96076	49.21465	1
H	77.45375	71.01362	83.74610	1714.76886	-2070.42778	443.35792	1
H	75.84325	70.57797	84.47336	1224.15040	1801.50881	1636.97633	1
C	77.17998	69.68466	80.57545	566.85117	537.05831	117.62814	1
O	77.97970	70.58166	80.83983	-550.26779	-406.08867	4.21666	1
N	77.07949	68.98197	79.46436	566.34832	-425.77725	180.18749	1
H	76.31772	68.30502	79.43255	644.44502	2646.80550	-52.73430	1
C	77.53432	69.62677	78.25874	612.34801	-281.48662	411.56221	1
H	77.37079	70.71419	78.43534	1667.23498	-1490.75142	1556.16753	1
C	79.08333	69.30362	78.11339	-714.45746	-175.43074	286.98927	1
H	79.26040	68.18967	78.06255	-787.09484	1763.03619	-1922.46060	1
O	79.92935	69.67437	79.22044	-429.46514	516.51932	-128.40526	1
H	79.23668	70.07157	79.85408	1981.82785	436.16847	-1818.18182	1
C	79.42154	69.93545	76.71902	630.79189	-468.94448	76.03230	1
H	80.47810	69.73093	76.42641	-1775.71861	2062.30353	131.89849	1
H	78.76653	69.53413	75.90996	-1855.14608	88.94118	1993.53760	1
H	79.28949	71.04280	76.73036	-1504.82350	-780.94249	-2132.90992	1
C	76.65850	69.03256	77.02238	288.57638	618.62814	-397.00270	1
O	76.05801	69.80845	76.29380	321.70495	-185.26452	574.37218	1
N	76.62841	67.67114	76.98182	-447.05792	-446.53431	367.76425	1
H	77.21849	67.43864	77.79601	350.31316	1975.63644	-1843.31522	1
C	77.02009	67.04442	75.73363	-511.23061	374.03917	-471.51480	1
H	78.02873	67.44578	75.49688	2234.79579	-898.70890	-1273.45190	1
C	77.03776	65.49060	76.06727	529.53781	-159.34528	563.72604	1
H	77.34135	64.93963	75.13603	654.59388	2347.94251	1217.51488	1
H	77.84566	65.31991	76.82500	-1526.55661	2103.42026	817.87394	1
O	75.76715	64.91267	76.50840	-554.84278	14.12789	399.58769	1
H	75.73957	65.27759	77.40872	1297.06236	2061.84854	1220.68812	1
C	75.97780	67.23985	74.52642	-662.15484	-412.13544	123.62687	1
O	76.17964	66.66700	73.47690	-540.01537	-317.05804	274.91456	1
N	74.94770	68.02373	74.85700	-202.76299	-690.00100	-131.49145	1
H	74.95178	68.52390	75.76888	-702.62132	-2617.37191	281.70413	1
C	73.85861	68.11998	73.95269	-491.73914	576.34187	222.73509	1
H	74.19462	67.51863	73.07513	1977.17978	-1823.58850	-434.68928	1
C	72.62300	67.48107	74.71717	-649.50797	-328.66843	306.11224	1
H	72.42375	68.02616	75.66481	2075.07803	1452.16490	-1004.45904	1
C	71.36124	67.47449	73.78844	-615.18735	-489.99689	-70.96352	1
H	70.47654	67.03178	74.30377	-1226.32661	-1724.38906	-1716.47170	1
H	71.07750	68.50365	73.46659	2381.64273	-969.84735	900.46860	1
H	71.53907	66.87823	72.86326	2122.74991	1702.23946	-141.36228	1

C	72.97347	65.98451	75.02940	-501.86074	-479.61276	-376.42482	1
H	72.16944	65.48554	75.61993	993.79261	-2246.95401	-1177.81305	1
H	73.11422	65.39437	74.09363	42.71279	-626.15391	-2651.37258	1
H	73.92403	65.89635	75.61460	-2026.39012	-1590.33709	-887.82922	1
C	73.74990	69.73244	73.62437	-672.89153	382.57394	-156.34072	1
O	74.58977	70.23138	72.91286	410.04627	221.86223	500.35950	1
N	72.70358	70.30556	74.20067	-378.11782	-443.79414	-441.10984	1
H	72.11697	69.71625	74.78891	2404.15973	-568.14894	1149.30085	1
C	72.58555	71.72540	74.28509	-544.54484	466.16019	331.29119	1
H	71.69964	71.85925	74.94693	-1409.80988	-170.17955	2325.32679	1
C	73.93801	72.29037	74.90164	-184.48284	116.78282	-758.89137	1
H	74.20314	71.74957	75.83925	1893.78641	1861.14652	611.04528	1
H	74.78883	72.10886	74.20291	-2170.79665	-974.19311	1327.50117	1
C	73.69451	73.80899	75.16848	389.51668	672.81878	-138.49152	1
H	73.82064	74.38407	74.22119	510.64577	1933.03327	1851.02484	1
H	72.63644	73.98012	75.46539	-2302.30869	-1105.72939	-948.89774	1
C	74.62654	74.39330	76.30880	-559.93427	-126.34590	542.30857	1
O	74.28329	75.30987	77.01384	-670.36003	-111.83166	76.36448	1
N	75.85159	73.74894	76.37652	-216.06729	-537.07037	446.51379	1
H	76.26003	73.39304	75.49747	-1251.96487	1074.12894	-2168.52500	1
H	76.59851	74.31070	76.79914	-2451.83023	-720.73851	944.84612	1
C	72.41155	72.38313	72.80962	546.24052	-563.88337	-85.11809	1
O	73.28974	73.12464	72.39141	71.70702	-307.40463	606.69673	1
N	71.22608	72.10160	72.27706	479.90863	-504.50129	222.86849	1
C	70.95161	72.29597	70.87952	75.51268	-600.91904	506.73700	1
H	71.81736	71.87398	70.32108	-2033.61562	1795.30103	254.89480	1
C	69.58799	71.50859	70.69621	523.79067	517.99274	284.45581	1
H	69.01122	71.84757	69.80625	1599.61665	-1127.00911	1895.98173	1
H	69.75945	70.41619	70.57110	-1881.83093	1275.82488	-1501.55016	1
C	68.87273	71.81618	72.04987	-600.05639	-404.05026	316.64485	1
H	68.45706	72.84870	72.05504	-2597.05260	496.36221	657.73249	1
H	68.02469	71.13081	72.27447	-1666.24518	1294.38985	-1723.90591	1
C	70.07912	71.67439	73.07154	493.57570	-129.95376	-602.56394	1
H	70.01381	72.32286	73.97629	-1469.08415	-637.84797	2204.22581	1
H	70.30301	70.62696	73.38212	-1038.97840	1068.88701	-2280.71789	1
C	70.72803	73.87821	70.54317	188.88100	241.05089	-727.87821	1
O	70.37627	74.22196	69.44180	531.88613	-49.19943	427.07960	1
N	70.95353	74.64630	71.61132	708.14833	26.93417	-179.73509	1
H	71.30086	74.13600	72.43017	-2232.69501	-1179.09831	1023.94816	1
C	70.80465	76.09419	71.56929	-738.32412	259.92548	104.42456	1
H	70.00759	76.26184	70.80545	2028.89123	1260.02983	1311.33324	1
C	70.43352	76.55858	72.96784	-258.65678	-345.52513	661.28459	1
H	71.25357	76.25604	73.65722	-1826.06090	-1801.46097	-918.64554	1
C	70.28819	78.06725	73.04408	-541.23400	531.92970	-218.41428	1
H	69.99516	78.40807	74.07501	1311.73218	-2219.18200	882.19023	1
H	71.25231	78.58291	72.78091	-1048.71298	1223.58118	-2196.97971	1
H	69.50517	78.44608	72.33102	419.06414	1903.92898	1903.44670	1
C	69.15810	75.89138	73.45177	-423.86431	152.66566	-648.55262	1
H	68.90881	76.18155	74.50890	105.68386	-2342.50080	1387.51238	1
H	68.27834	76.17483	72.81137	-1695.63603	-1484.73621	-1531.02793	1
H	69.24101	74.77039	73.41963	-1853.57121	-487.39031	1936.59343	1
C	72.24321	76.77610	71.07638	539.71428	-510.58232	267.58633	1
O	72.25388	77.95769	70.82344	-15.93758	-278.34085	-624.49402	1
N	73.29067	75.93191	71.14338	-676.81886	12.05292	276.17960	1
H	72.86375	75.04151	71.42743	-1694.44250	-373.84189	-2100.66098	1
C	74.24656	75.87814	70.04378	412.33078	-664.70360	108.35451	1
H	73.83907	75.02995	69.45049	1765.77476	1294.44366	1621.76594	1
C	74.19322	77.22506	69.23313	-179.66635	-721.95667	-264.73915	1
H	73.16635	77.37957	68.82559	-607.34922	2587.87032	-598.10018	1
H	74.35613	78.07767	69.93421	-359.69332	-1479.13954	-2259.74260	1
C	75.21478	77.25672	68.04472	-240.01076	616.02425	431.85322	1
H	76.25464	77.10523	68.41510	-1359.17131	782.54425	2227.99181	1

H	75.02425	76.43419	67.31983	-820.36667	85.81767	-2596.78715	1
C	75.04614	78.67286	67.36089	-513.07015	236.18570	-551.87280	1
H	74.07326	78.73538	66.82155	1816.11944	-1183.11722	-1650.94321	1
H	75.00905	79.48091	68.12595	-1800.04429	-1361.53473	1526.34643	1
C	76.23117	78.91553	66.39689	726.08972	-253.19732	-179.64713	1
H	77.23295	78.77721	66.85000	1224.27271	-456.51798	-2390.90304	1
H	76.17111	78.36957	65.43411	1206.40339	-1150.93514	-2154.90344	1
N	76.21082	80.43450	66.02027	329.70796	-633.68968	-155.67358	1
H	76.98292	80.75306	65.36667	1348.31305	1938.76845	1359.00643	1
H	75.34679	80.81561	65.53530	2096.92035	1446.54551	-966.48890	1
H	76.33330	81.11289	66.82662	-1197.07217	-2303.53719	827.28733	1
C	75.66952	75.47564	70.66215	-378.01186	-556.17301	-413.96481	1
O	76.72425	75.96385	70.23815	-409.44433	-307.55053	-453.30833	1
N	75.50396	74.51371	71.53366	-429.02477	524.70884	274.08502	1
H	74.53604	74.18572	71.76972	1706.34263	-1425.96036	1574.38926	1
C	76.56662	73.85403	72.20096	-45.25864	-626.22952	478.93267	1
H	76.02842	73.06328	72.77055	1988.63164	570.90773	1772.87287	1
C	77.60765	73.26966	71.15738	551.77128	-20.49526	-564.54996	1
H	77.15516	72.45496	70.54903	1434.61535	-1753.10253	1513.99471	1
H	77.92932	74.04702	70.42692	-714.67738	1819.09770	1898.36494	1
C	78.81531	72.75034	72.00590	446.99263	-555.77305	338.97221	1
H	79.21034	73.57314	72.64813	1953.68466	1892.25957	-161.67766	1
H	78.48321	71.95226	72.70718	-403.03785	2688.07032	-188.43591	1
C	79.96838	72.21683	71.06532	567.86262	335.74681	434.04421	1
H	79.63493	71.33050	70.47825	-1080.02275	1568.26081	-1948.78900	1
H	80.26844	72.98614	70.31708	-919.54108	2111.34692	1456.13463	1
C	81.14749	71.86407	72.00327	195.81897	173.33862	-745.11536	1
H	81.58791	72.73300	72.53416	1405.67949	-2301.21089	-390.08113	1
H	80.93207	71.05169	72.72587	-940.37328	-1118.32446	2299.72133	1
N	82.31616	71.30412	71.12659	319.26422	656.36007	-42.02424	1
H	83.18284	71.00716	71.66176	1961.03633	619.91968	1787.09422	1
H	82.11727	70.43277	70.55363	854.14343	-2156.80752	1429.08711	1
H	82.73564	71.95513	70.40076	1237.02905	-1378.50857	-1998.28500	1
C	77.38962	74.86861	73.20547	532.53319	-268.57236	517.55729	1
O	77.34117	74.67143	74.39800	266.16714	-290.73804	-558.88002	1
N	78.09845	75.73651	72.50921	-226.25352	640.49678	-270.32839	1
H	77.99774	75.75361	71.47160	2007.69063	-1035.27229	1523.50231	1
C	78.93246	76.65196	73.19818	-540.76542	-573.33303	49.50000	1
H	78.83181	76.38270	74.27628	-2374.20950	-1036.76023	843.75774	1
C	80.41312	76.42384	72.61456	-77.09082	666.87422	-415.84136	1
H	80.39534	76.65057	71.51326	-985.81834	617.19825	2463.91927	1
O	80.91740	75.07878	72.72569	293.29899	-391.65602	-477.80926	1
H	81.05092	74.85838	71.78115	2528.72714	-681.76262	-751.27122	1
C	81.27913	77.51299	73.34231	252.47363	-605.14473	440.05134	1
H	82.35491	77.42808	73.06193	-2052.65063	1456.49051	-1043.51830	1
H	80.95701	78.55094	73.08358	-2152.98800	-1661.97574	-161.70652	1
H	81.21709	77.41205	74.45104	-473.12726	-1584.27339	2165.61725	1
C	78.45790	78.17401	72.85502	360.36307	488.46713	505.10056	1
O	78.85441	79.08836	73.53686	-200.08817	-528.03552	-385.82850	1
N	77.75376	78.24786	71.72210	-597.54095	-226.44775	355.20479	1
H	77.39248	77.39467	71.26655	2179.23655	-1300.70981	-991.33835	1
C	77.02373	79.45798	71.53337	-687.68921	297.39807	-249.45179	1
H	77.63768	80.26423	71.99924	-349.16256	-2467.37713	-1101.72831	1
C	76.72152	79.62519	69.97270	672.47345	361.54328	-201.62880	1
H	76.01186	78.82025	69.68508	1541.41502	582.18425	2169.97026	1
H	76.21039	80.59598	69.78080	-2534.67717	60.25153	997.72295	1
C	77.97802	79.49940	69.10236	574.26774	-389.43119	-377.02542	1
C	78.57277	80.66871	68.53915	688.82679	-287.33580	-257.96133	1
H	78.18407	81.67489	68.77939	331.14182	1470.38163	2269.80013	1
C	78.49224	78.19914	68.80691	-383.52382	-675.04808	144.24917	1
H	78.03606	77.29277	69.26577	4.09847	1517.59662	2262.86397	1
C	79.64156	80.50697	67.65876	-134.76730	428.45285	-649.50303	1

210	H	80.09842	81.40696	67.21146	-903.23433	1600.49020	2011.53387	1
211	C	79.56137	78.09375	67.91466	-335.53281	-615.00040	-364.39058	1
212	H	79.94336	77.08617	67.67508	1596.45398	-1369.33862	1732.02659	1
213	C	80.16812	79.23108	67.30429	585.48547	-340.24194	-406.23938	1
214	O	81.24818	79.13312	66.38904	-419.15649	-173.04408	-511.94141	1
215	H	81.35280	78.15285	66.35976	-1071.84350	1559.71507	-1960.13012	1
216	C	75.57889	79.22279	72.24958	291.41878	-183.13091	710.72246	1
217	O	75.25995	78.21185	72.83117	379.87695	523.47396	-222.23622	1
218	N	74.85353	80.31895	72.05507	564.30591	373.09380	277.24371	1
219	H	75.22571	81.15242	71.59977	-120.59931	-2436.07824	-1214.35098	1
220	C	73.54126	80.35906	72.64101	404.07514	-299.37480	608.84009	1
221	H	73.03752	79.40426	72.35880	-1071.07882	-841.69025	2359.66467	1
222	C	73.83918	80.44252	74.19849	-683.12807	68.71770	-390.13096	1
223	H	74.54672	79.62703	74.47886	2068.88918	1596.58590	770.89603	1
224	H	74.37300	81.39078	74.43140	1379.86429	2342.49779	-179.85638	1
225	C	72.53309	80.32623	75.03857	-176.39967	537.49466	550.97268	1
226	H	71.82637	81.16167	74.83563	-396.28051	1942.22168	1869.33314	1
227	H	71.97683	79.38956	74.81377	-94.44700	586.00678	-2659.20016	1
228	C	73.07760	80.34335	76.54304	-291.69381	-718.18079	-150.72929	1
229	H	73.63766	79.41792	76.83572	-2303.38776	1089.76242	964.62032	1
230	H	73.70960	81.23185	76.82235	884.16729	2568.80533	-207.74283	1
231	N	71.91545	80.38144	77.32044	-594.49522	-252.81478	342.29276	1
232	H	71.05549	80.13141	76.85734	-1735.17426	2084.05705	-263.71605	1
233	C	71.88637	80.67532	78.55741	595.23177	-271.52487	442.22352	1
234	N	73.09634	81.00448	79.17887	585.53813	-424.79750	-105.81303	1
235	H	74.04267	80.99638	78.80047	959.88797	1327.56823	-2177.11905	1
236	H	73.20124	81.21906	80.17090	1578.48647	16.27378	-2220.76233	1
237	N	70.57951	80.60770	79.05787	-452.17763	-390.33541	-421.51853	1
238	H	69.70306	80.34731	78.60794	470.26107	1974.25629	-1817.92087	1
239	H	70.34902	80.78342	80.03678	1856.67307	-1573.97321	-1224.35416	1
240	C	72.87322	81.71432	72.06387	-531.47271	-581.45780	-55.06206	1
241	O	73.54541	82.70991	71.80742	-243.00837	192.73507	609.52485	1
242	N	71.55968	81.57864	72.01003	343.49288	-644.25607	38.09164	1
243	H	71.20411	80.67341	72.31054	-1691.16043	-1535.97525	-1484.73099	1
244	C	70.73777	82.53251	71.26411	542.66492	350.14337	454.42563	1
245	H	71.45170	82.92185	70.49869	1209.57979	-2396.95920	463.86744	1
246	C	69.54155	81.79727	70.68606	-737.14309	145.93010	242.71942	1
247	H	68.93259	81.41366	71.53457	-1050.18303	159.36419	-2509.06125	1
248	C	68.67037	82.72417	69.85831	-706.77696	-252.88343	-245.16230	1
249	H	67.74840	82.20292	69.47908	-1624.64039	-1665.49085	1417.86935	1
250	H	68.33213	83.61335	70.46106	2566.10521	598.51109	693.22305	1
251	H	69.22099	83.11932	68.96014	-1972.69758	107.10018	-1876.34312	1
252	C	69.97339	80.60700	69.84798	412.85148	672.58832	-27.69222	1
253	H	69.09501	79.99985	69.49662	1447.02277	1762.76533	-1490.78944	1
254	H	70.53974	80.93334	68.93298	1320.77379	-145.20455	2378.68491	1
255	H	70.65186	79.91457	70.41988	-1512.10848	1470.52031	1724.75147	1
256	C	70.37304	83.70583	72.38659	-607.11113	499.56689	73.73769	1
257	O	69.24555	83.95714	72.72138	357.97126	460.81534	356.68785	1
258	N	71.50828	84.26996	72.75851	536.55407	-368.62270	332.76306	1
259	H	72.41216	83.94094	72.34521	524.38866	2297.69985	1367.20941	1
260	C	71.48211	85.23256	73.79790	46.50277	-774.29190	-147.97904	1
261	H	70.52707	85.03757	74.33702	1676.51875	1883.29066	1032.55549	1
262	C	72.82837	85.11938	74.67975	-559.41456	-34.19198	-556.30433	1
263	H	73.71100	85.25207	74.00863	-64.66664	395.18769	2695.05373	1
264	H	72.87492	85.95943	75.41378	-2703.44278	-60.40056	333.79418	1
265	C	72.90709	83.79187	75.38601	-519.76435	377.31045	459.42366	1
266	H	72.75539	82.98617	74.63320	-1746.74323	1667.50644	-1261.73691	1
267	C	74.28112	83.61616	76.02009	570.95554	16.46261	-545.27700	1
268	H	74.35546	82.64759	76.59239	2391.58273	28.05277	-1305.07271	1
269	H	75.09529	83.61450	75.24995	175.75588	917.63458	2559.43867	1
270	H	74.51004	84.44395	76.73998	-1063.97851	-2155.34650	1283.00462	1
271	C	71.83774	83.66772	76.46309	193.76014	2.62946	-765.53141	1

```
H    71.94236  82.71414  77.04292      507.39394     -846.55087     2539.60089          1
H    71.89623  84.50950  77.20060     1337.57841     1818.19054     1526.01994          1
H    70.80582  83.67734  76.02720     1100.88635    -1172.91350    -2199.08909          1
C    71.49093  86.66643  73.05543     -549.21115      475.64486     -309.38218          1
O    72.38312  87.46463  73.16343      556.06106     -390.37755      -78.24250          1
O    70.34789  86.94607  72.27252     -572.27940     -310.19032     -209.75862          1
H    69.88056  86.06195  72.35948    -1380.08193     2212.14486      790.85944          1
```

Listing 30: C00030.xmolout

```
      276
  150.0000  150.0000  150.0000    0.0000    0.0000    0.0000***********   0.7397     2959
      0.2500    300.00    0.00    0.00    0.00  -1293.9175      0.0000   300.0000
H    78.62264  63.84704  87.94986    -2043.14101    -1757.64893      399.89339          1
N    78.80462  64.55328  87.23881      426.75047      490.45733     -334.42467          1
H    77.97384  65.13605  87.32135      442.25559    -1110.30854    -2448.52907          1
C    80.02957  65.23756  87.40265      415.32972     -666.39213       83.73172          1
H    80.65037  64.57071  88.03759      879.32219     2491.00447      667.35213          1
C    80.66873  65.48275  85.94383       31.35357      477.41777      628.23362          1
H    80.00100  66.13560  85.33121     1168.38489     2240.25337    -1019.71136          1
H    81.64006  66.02772  86.02156     -360.43770     2534.90498      931.67112          1
C    80.84798  64.14299  85.27530      463.18208      512.70242     -382.34388          1
H    79.95403  63.51880  85.50195     1725.29117     1863.52247     -987.07854          1
C    80.94098  64.31424  83.76495      710.42801     -144.27427     -313.15367          1
H    81.07976  63.33318  83.24118      364.20195    -1033.13725     2494.72524          1
H    80.01350  64.78934  83.34534     2567.61904      335.80679      847.48802          1
H    81.80065  64.97304  83.47507    -2587.99029      680.92867     -512.16411          1
C    82.08888  63.43087  85.79501      268.17616      741.64379      -40.42634          1
H    82.22175  62.42742  85.31394     1283.54314    -1945.37420    -1411.27812          1
H    83.01884  64.02211  85.59024    -1258.57796     1317.15249    -2026.02085          1
H    82.03890  63.26436  86.90190    -2502.84526      909.80597      575.92391          1
C    79.69309  66.66772  88.11414      619.36578     -458.64843     -172.09273          1
O    80.20702  66.96994  89.19762      -81.40433      433.87611      522.34594          1
N    78.87909  67.34351  87.35504      175.23996     -622.20190      341.55523          1
H    78.57444  66.84374  86.52174     -876.57256     -303.22879    -2561.90213          1
C    78.45684  68.70215  87.54243     -571.88214     -534.10783     -106.15033          1
H    77.36795  68.60161  87.74491    -2713.20841      -16.25800     -248.80809          1
C    79.22574  69.39868  88.76163      166.60354      288.16399     -716.09568          1
H    80.32794  69.17174  88.65973    -2097.85574     1636.78449     -586.18044          1
O    78.86735  68.91134  90.05247     -317.80580     -363.38227      484.43082          1
H    79.52871  68.11441  90.11419     1863.74748    -1451.84923     1357.29467          1
C    78.97679  70.92629  88.49403      503.80731      517.99032      318.51606          1
H    79.49466  71.56042  89.25029      640.61020    -2157.10410    -1536.29104          1
H    79.34559  71.23889  87.48909     1687.88455      465.60692     2087.56452          1
H    77.89149  71.17584  88.54238    -1293.07895    -1899.88581    -1463.57452          1
C    78.61735  69.39211  86.09339     -153.07550     -733.68902      248.70981          1
O    77.54996  69.61358  85.50258      186.15459     -480.78787     -449.34387          1
N    79.84785  69.63009  85.68120     -487.53139      543.18517       42.03850          1
C    80.12171  70.44205  84.51955     -699.35871       69.34393      360.10701          1
H    79.44303  71.32288  84.58983        2.81126     -221.99807    -2715.58072          1
C    81.67854  70.72156  84.62562     -507.46439     -592.59194      122.07761          1
H    82.13626  70.92433  83.62872    -1975.08116      525.87560    -1801.71556          1
H    81.90337  71.59761  85.27464     1588.27743    -1913.22100     1113.83566          1
C    82.18325  69.38811  85.26463     -613.62442     -457.55993     -194.14490          1
H    82.22632  68.57688  84.50315     1707.54637     -721.27992     1996.92523          1
H    83.20038  69.46216  85.71150      758.23357     2100.81767     1560.55011          1
C    81.04579  69.11512  86.33715     -352.41524      704.96076       49.21465          1
H    80.86962  68.04469  86.58973     1714.76886    -2070.42778      443.35792          1
H    81.16162  69.70412  87.27785     1224.15040     1801.50881     1636.97633          1
C    79.93027  69.59125  83.14938      566.85117      537.05831      117.62814          1
O    80.89033  69.44944  82.39681     -550.26779     -406.08867        4.21666          1
N    78.69246  69.17712  82.99052      566.34832     -425.77725      180.18749          1
```

H	77.99311	69.33588	83.73924	644.44502	2646.80550	-52.73430	1
C	78.38218	68.52665	81.75072	612.34801	-281.48662	411.56221	1
H	78.99422	69.13007	81.04032	1667.23498	-1490.75142	1556.16753	1
C	78.89653	67.01900	81.79693	-714.45746	-175.43074	286.98927	1
H	78.20548	66.37928	82.41403	-787.09484	1763.03619	-1922.46060	1
O	80.18786	66.79610	82.39308	-429.46514	516.51932	-128.40526	1
H	80.56168	67.72812	82.34858	1981.82785	436.16847	-1818.18182	1
C	78.75512	66.57453	80.29905	630.79189	-468.94448	76.03230	1
H	79.01239	65.49760	80.16887	-1775.71861	2062.30353	131.89849	1
H	77.71427	66.71541	79.91790	-1855.14608	88.94118	1993.53760	1
H	79.43482	67.15798	79.63484	-1504.82350	-780.94249	-2132.90992	1
C	76.77177	68.61099	81.52598	288.57638	618.62814	-397.00270	1
O	76.13418	67.63289	81.21143	321.70495	-185.26452	574.37218	1
N	76.32884	69.88555	81.62111	-447.05792	-446.53431	367.76425	1
H	77.04956	70.50945	81.99042	350.31316	1975.63644	-1843.31522	1
C	74.98348	69.99534	82.12054	-511.23061	374.03917	-471.51480	1
H	74.48960	69.03704	81.83378	2234.79579	-898.70890	-1273.45190	1
C	75.10437	70.17757	83.70317	529.53781	-159.34528	563.72604	1
H	74.05419	70.31281	84.07836	654.59388	2347.94251	1217.51488	1
H	75.47416	69.21387	84.14688	-1526.55661	2103.42026	817.87394	1
O	75.92405	71.26292	84.16615	-554.84278	14.12789	399.58769	1
H	76.62395	70.73598	84.67744	1297.06236	2061.84854	1220.68812	1
C	74.22632	71.30189	81.55090	-662.15484	-412.13544	123.62687	1
O	73.08199	71.51039	81.88006	-540.01537	-317.05804	274.91456	1
N	74.96346	72.03886	80.69424	-202.76299	-690.00100	-131.49145	1
H	75.90940	71.67891	80.56321	-702.62132	-2617.37191	281.70413	1
C	74.64662	73.46783	80.56891	-491.73914	576.34187	222.73509	1
H	73.72195	73.59308	81.18206	1977.17978	-1823.58850	-434.68928	1
C	75.83600	74.27082	81.05348	-649.50797	-328.66843	306.11224	1
H	76.71324	73.98489	80.43084	2075.07803	1452.16490	-1004.45904	1
C	75.60593	75.76274	80.88893	-615.18735	-489.99689	-70.96352	1
H	76.50229	76.35991	81.21261	-1226.32661	-1724.38906	-1716.47170	1
H	75.39132	76.03207	79.81802	2381.64273	-969.84735	900.46860	1
H	74.73162	76.11638	81.50135	2122.74991	1702.23946	-141.36228	1
C	76.17379	73.95807	82.49998	-501.86074	-479.61276	-376.42482	1
H	77.12143	74.46527	82.83133	993.79261	-2246.95401	-1177.81305	1
H	75.35552	74.28960	83.19706	42.71279	-626.15391	-2651.37258	1
H	76.30845	72.85124	82.66011	-2026.39012	-1590.33709	-887.82922	1
C	74.41708	73.76022	78.94923	-672.89153	382.57394	-156.34072	1
O	75.31791	73.67970	78.14932	410.04627	221.86223	500.35950	1
N	73.17728	74.19428	78.75464	-378.11782	-443.79414	-441.10984	1
H	72.56484	74.20186	79.56872	2404.15973	-568.14894	1149.30085	1
C	72.64035	74.11072	77.43388	-544.54484	466.16019	331.29119	1
H	73.53311	73.94809	76.78635	-1409.80988	-170.17955	2325.32679	1
C	71.62090	72.89552	77.45396	-184.48284	116.78282	-758.89137	1
H	72.12345	71.97112	77.81993	1893.78641	1861.14652	611.04528	1
H	70.78931	73.09344	78.16716	-2170.79665	-974.19311	1327.50117	1
C	71.07883	72.70885	76.00368	389.51668	672.81878	-138.49152	1
H	70.56554	73.64232	75.67418	510.64577	1933.03327	1851.02484	1
H	71.92184	72.59172	75.28745	-2302.30869	-1105.72939	-948.89774	1
C	70.09661	71.48514	75.86141	-559.93427	-126.34590	542.30857	1
O	69.64394	71.21633	74.77864	-670.36003	-111.83166	76.36448	1
N	69.84958	70.82993	77.01601	-216.06729	-537.07037	446.51379	1
H	70.19438	71.01515	77.96505	-1251.96487	1074.12894	-2168.52500	1
H	69.22362	70.02100	77.09172	-2451.83023	-720.73851	944.84612	1
C	71.82209	75.49413	77.13567	546.24052	-563.88337	-85.11809	1
O	70.62297	75.46773	77.04734	71.70702	-307.40463	606.69673	1
N	72.65999	76.53099	76.97020	479.90863	-504.50129	222.86849	1
C	72.22630	77.87522	77.17436	75.51268	-600.91904	506.73700	1
H	71.36439	77.84305	77.87918	-2033.61562	1795.30103	254.89480	1
C	73.56060	78.53705	77.73485	523.79067	517.99274	284.45581	1
H	73.55186	79.64759	77.66891	1599.61665	-1127.00911	1895.98173	1

H	73.72912	78.27668	78.80352	-1881.83093	1275.82488	-1501.55016	1
C	74.62706	77.86800	76.81453	-600.05639	-404.05026	316.64485	1
H	74.62214	78.32455	75.79844	-2597.05260	496.36221	657.73249	1
H	75.66851	77.95716	77.19791	-1666.24518	1294.38985	-1723.90591	1
C	74.09767	76.37082	76.78471	493.57570	-129.95376	-602.56394	1
H	74.26348	75.82831	75.82422	-1469.08415	-637.84797	2204.22581	1
H	74.45333	75.73199	77.62957	-1038.97840	1068.88701	-2280.71789	1
C	71.85853	78.64885	75.77724	188.88100	241.05089	-727.87821	1
O	71.45431	79.79531	75.83091	531.88613	-49.19943	427.07960	1
N	72.19130	77.96064	74.67117	708.14833	26.93417	-179.73509	1
H	72.45818	77.03006	74.99563	-2232.69501	-1179.09831	1023.94816	1
C	71.54208	78.16173	73.37103	-738.32412	259.92548	104.42456	1
H	71.73162	77.20872	72.82225	2028.89123	1260.02983	1311.33324	1
C	70.06304	78.42310	73.58898	-258.65678	-345.52513	661.28459	1
H	69.97416	79.32121	74.24617	-1826.06090	-1801.46097	-918.64554	1
C	69.35288	78.72161	72.28104	-541.23400	531.92970	-218.41428	1
H	68.26241	78.94731	72.43708	1311.73218	-2219.18200	882.19023	1
H	69.80964	79.61058	71.76164	-1048.71298	1223.58118	-2196.97971	1
H	69.41706	77.85402	71.56803	419.06414	1903.92898	1903.44670	1
C	69.38317	77.26745	74.30029	-423.86431	152.66566	-648.55262	1
H	68.30704	77.49748	74.53239	105.68386	-2342.50080	1387.51238	1
H	69.39626	76.33176	73.67648	-1695.63603	-1484.73621	-1531.02793	1
H	69.89077	77.02094	75.27554	-1853.57121	-487.39031	1936.59343	1
C	72.24301	79.45225	72.61461	539.71428	-510.58232	267.58633	1
O	72.06828	79.61634	71.42526	-15.93758	-278.34085	-624.49402	1
N	72.88968	80.22294	73.46319	-676.81886	12.05292	276.17960	1
H	72.88218	79.95713	74.45497	-1694.44250	-373.84189	-2100.66098	1
C	73.58684	81.35927	72.96660	412.33078	-664.70360	108.35451	1
H	73.11367	81.56390	71.97821	1765.77476	1294.44366	1621.76594	1
C	73.44962	82.49080	74.06200	-179.66635	-721.95667	-264.73915	1
H	72.38046	82.72399	74.26645	-607.34922	2587.87032	-598.10018	1
H	73.87165	82.14407	75.03239	-359.69332	-1479.13954	-2259.74260	1
C	74.22104	83.74861	73.54238	-240.01076	616.02425	431.85322	1
H	75.28236	83.47775	73.33213	-1359.17131	782.54425	2227.99181	1
H	73.79476	84.11025	72.58069	-820.36667	85.81767	-2596.78715	1
C	74.15143	84.88137	74.64512	-513.07015	236.18570	-551.87280	1
H	73.09845	85.18957	74.84192	1816.11944	-1183.11722	-1650.94321	1
H	74.54907	84.51584	75.61991	-1800.04429	-1361.53473	1526.34643	1
C	74.99415	86.06189	74.11018	726.08972	-253.19732	-179.64713	1
H	76.07189	85.83572	73.98729	1224.27271	-456.51798	-2390.90304	1
H	74.59916	86.52862	73.18574	1206.40339	-1150.93514	-2154.90344	1
N	74.95773	87.20952	75.17453	329.70796	-633.68968	-155.67358	1
H	75.52016	88.07438	74.92746	1348.31305	1938.76845	1359.00643	1
H	74.01162	87.63467	75.40057	2096.92035	1446.54551	-966.48890	1
H	75.33640	86.98565	76.14060	-1197.07217	-2303.53719	827.28733	1
C	75.18740	80.98189	72.84770	-378.01186	-556.17301	-413.96481	1
O	75.96341	81.32740	73.70904	-409.44433	-307.55053	-453.30833	1
N	75.43349	80.30229	71.73770	-429.02477	524.70884	274.08502	1
H	74.65637	80.04557	71.13065	1706.34263	-1425.96036	1574.38926	1
C	76.81694	80.11560	71.39271	-45.25864	-626.22952	478.93267	1
H	77.28013	81.11708	71.53727	1988.63164	570.90773	1772.87287	1
C	77.37727	79.06880	72.43406	551.77128	-20.49526	-564.54996	1
H	77.23718	79.44395	73.47608	1434.61535	-1753.10253	1513.99471	1
H	76.81292	78.11153	72.37294	-714.67738	1819.09770	1898.36494	1
C	78.89063	78.86241	72.09054	446.99263	-555.77305	338.97221	1
H	79.00770	78.55265	71.02785	1953.68466	1892.25957	-161.67766	1
H	79.45924	79.81345	72.19592	-403.03785	2688.07032	-188.43591	1
C	79.45726	77.75236	73.06303	567.86262	335.74681	434.04421	1
H	79.38995	78.08037	74.12599	-1080.02275	1568.26081	-1948.78900	1
H	78.85489	76.81708	72.99488	-919.54108	2111.34692	1456.13463	1
C	80.92539	77.49328	72.65158	195.81897	173.33862	-745.11536	1
H	81.04974	77.04595	71.64546	1405.67949	-2301.21089	-390.08113	1

H	81.59756	78.36766	72.76105	-940.37328	-1118.32446	2299.72133	1
N	81.49810	76.42312	73.63979	319.26422	656.36007	-42.02424	1
H	82.50137	76.12604	73.46564	1961.03633	619.91968	1787.09422	1
H	81.53425	76.68847	74.66705	854.14343	-2156.80752	1429.08711	1
H	81.00420	75.48408	73.67387	1237.02905	-1378.50857	-1998.28500	1
C	76.94805	79.50240	69.88169	532.53319	-268.57236	517.55729	1
O	76.52801	78.37453	69.59443	266.16714	-290.73804	-558.88002	1
N	77.58638	80.37426	69.14825	-226.25352	640.49678	-270.32839	1
H	77.81367	81.22067	69.67154	2007.69063	-1035.27229	1523.50231	1
C	78.13631	80.21395	67.85398	-540.76542	-573.33303	49.50000	1
H	78.45625	81.25217	67.61094	-2374.20950	-1036.76023	843.75774	1
C	79.38217	79.20376	67.90609	-77.09082	666.87422	-415.84136	1
H	79.01632	78.17240	68.16186	-985.81834	617.19825	2463.91927	1
O	80.42284	79.51119	68.84334	293.29899	-391.65602	-477.80926	1
H	80.46240	78.68215	69.36395	2528.72714	-681.76262	-751.27122	1
C	79.77059	79.26053	66.38201	252.47363	-605.14473	440.05134	1
H	80.56937	78.52620	66.12844	-2052.65063	1456.49051	-1043.51830	1
H	78.88689	79.04123	65.72868	-2152.98800	-1661.97574	-161.70652	1
H	80.14952	80.26888	66.09468	-473.12726	-1584.27339	2165.61725	1
C	77.09693	79.59328	66.74512	360.36307	488.46713	505.10056	1
O	76.90410	80.22673	65.73683	-200.08817	-528.03552	-385.82850	1
N	76.68152	78.37012	67.05419	-597.54095	-226.44775	355.20479	1
H	76.89884	77.93503	67.98466	2179.23655	-1300.70981	-991.33835	1
C	75.88658	77.70287	66.08434	-687.68921	297.39807	-249.45179	1
H	75.98716	78.29845	65.14553	-349.16256	-2467.37713	-1101.72831	1
C	76.41979	76.18596	65.96923	672.47345	361.54328	-201.62880	1
H	76.51158	75.72059	66.97644	1541.41502	582.18425	2169.97026	1
H	75.69205	75.57645	65.38701	-2534.67717	60.25153	997.72295	1
C	77.77610	76.23874	65.25936	574.26774	-389.43119	-377.02542	1
C	77.79931	76.42470	63.84343	688.82679	-287.33580	-257.96133	1
H	76.85815	76.49058	63.26831	331.14182	1470.38163	2269.80013	1
C	78.99688	76.15716	65.99616	-383.52382	-675.04808	144.24917	1
H	78.98489	76.01951	67.09201	4.09847	1517.59662	2262.86397	1
C	79.03251	76.53383	63.20394	-134.76730	428.45285	-649.50303	1
H	79.04764	76.68200	62.10999	-903.23433	1600.49020	2011.53387	1
C	80.20728	76.26707	65.30835	-335.53281	-615.00040	-364.39058	1
H	81.14505	76.20434	65.88788	1596.45398	-1369.33862	1732.02659	1
C	80.27547	76.46510	63.89801	585.48547	-340.24194	-406.23938	1
O	81.49889	76.59383	63.19097	-419.15649	-173.04408	-511.94141	1
H	82.13495	76.49018	63.93773	-1071.84350	1559.71507	-1960.13012	1
C	74.35064	77.58737	66.59472	291.41878	-183.13091	710.72246	1
O	73.48794	77.33546	65.76047	379.87695	523.47396	-222.23622	1
N	74.25503	77.70669	67.89652	564.30591	373.09380	277.24371	1
H	75.11020	77.91060	68.44270	-120.59931	-2436.07824	-1214.35098	1
C	73.00431	77.89865	68.55313	404.07514	-299.37480	608.84009	1
H	73.31411	78.21495	69.57684	-1071.07882	-841.69025	2359.66467	1
C	72.20185	79.02501	67.77044	-683.12807	68.71770	-390.13096	1
H	72.82344	79.94092	67.65308	2068.88918	1596.58590	770.89603	1
H	71.93975	78.70323	66.73593	1379.86429	2342.49779	-179.85638	1
C	70.93021	79.29974	68.62678	-176.39967	537.49466	550.97268	1
H	70.23152	78.43286	68.59628	-396.28051	1942.22168	1869.33314	1
H	71.22004	79.42640	69.71527	-94.44700	586.00678	-2659.20016	1
C	70.24205	80.63038	68.06885	-291.69381	-718.18079	-150.72929	1
H	70.83714	81.55997	68.25888	-2303.38776	1089.76242	964.62032	1
H	69.91804	80.61818	66.99900	884.16729	2568.80533	-207.74283	1
N	69.11203	80.65836	68.89907	-594.49522	-252.81478	342.29276	1
H	69.20294	80.20741	69.79680	-1735.17426	2084.05705	-263.71605	1
C	68.00995	81.23571	68.62615	595.23177	-271.52487	442.22352	1
N	67.89163	81.88476	67.39324	585.53813	-424.79750	-105.81303	1
H	68.58682	82.00861	66.65896	959.88797	1327.56823	-2177.11905	1
H	67.07084	82.40461	67.08214	1578.48647	16.27378	-2220.76233	1
N	67.10438	81.09048	69.68561	-452.17763	-390.33541	-421.51853	1

```
H    67.20138  80.63032  70.58956      470.26107     1974.25629    -1817.92087          1
H    66.16823  81.49687  69.67904     1856.67307    -1573.97321    -1224.35416          1
C    72.10246  76.53454  68.59867     -531.47271     -581.45780      -55.06206          1
O    71.77185  76.13230  69.67740     -243.00837      192.73507      609.52485          1
N    71.75579  76.09722  67.38064      343.49288     -644.25607       38.09164          1
H    72.18230  76.60578  66.55445    -1691.16043    -1535.97525    -1484.73099          1
C    71.44638  74.66771  67.16768      542.66492      350.14337      454.42563          1
H    71.59403  74.56972  66.06668     1209.57979    -2396.95920      463.86744          1
C    72.39672  73.80830  67.97460     -737.14309      145.93010      242.71942          1
H    72.21399  74.02629  69.05406    -1050.18303      159.36419    -2509.06125          1
C    72.12870  72.33155  67.74251     -706.77696     -252.88343     -245.16230          1
H    72.79917  71.68202  68.36897    -1624.64039    -1665.49085     1417.86935          1
H    71.06520  72.06790  68.00411     2566.10521      598.51109      693.22305          1
H    72.29325  72.04251  66.66792    -1972.69758      107.10018    -1876.34312          1
C    73.84897  74.12896  67.67040      412.85148      672.58832      -27.69222          1
H    74.55284  73.52570  68.30590     1447.02277     1762.76533    -1490.78944          1
H    74.10305  73.91768  66.59573     1320.77379     -145.20455     2378.68491          1
H    74.07722  75.21497  67.85534    -1512.10848     1470.52031     1724.75147          1
C    69.88182  74.44300  67.61869     -607.11113      499.56689       73.73769          1
O    69.60770  73.85443  68.66700      357.97126      460.81534      356.68785          1
N    69.02184  74.87180  66.68276      536.55407     -368.62270      332.76306          1
H    69.65739  75.29325  65.98820      524.38866     2297.69985     1367.20941          1
C    67.91139  75.70047  67.10916       46.50277     -774.29190     -147.97904          1
H    68.36755  76.39848  67.84465     1676.51875     1883.29066     1032.55549          1
C    67.30955  76.42724  65.80451     -559.41456      -34.19198     -556.30433          1
H    67.14403  75.64612  65.02251      -64.66664      395.18769     2695.05373          1
H    66.29637  76.84208  66.03994    -2703.44278      -60.40056      333.79418          1
C    68.19061  77.52573  65.25854     -519.76435      377.31045      459.42366          1
H    69.24521  77.17318  65.23172    -1746.74323     1667.50644    -1261.73691          1
C    67.75854  77.86639  63.83781      570.95554       16.46261     -545.27700          1
H    68.38097  78.68875  63.39885     2391.58273       28.05277    -1305.07271          1
H    67.84559  76.98228  63.15408      175.75588      917.63458     2559.43867          1
H    66.69014  78.20557  63.80337    -1063.97851    -2155.34650     1283.00462          1
C    68.11369  78.77723  66.12127      193.76014        2.62946     -765.53141          1
H    68.68620  79.62601  65.66582      507.39394     -846.55087     2539.60089          1
H    67.05690  79.12920  66.24937     1337.57841     1818.19054     1526.01994          1
H    68.52933  78.60803  67.14816     1100.88635    -1172.91350    -2199.08909          1
C    66.70924  74.86141  67.70429     -549.21115      475.64486     -309.38218          1
O    65.58573  75.21232  67.44433      556.06106     -390.37755      -78.24250          1
O    66.97158  73.81782  68.56465     -572.27940     -310.19032     -209.75862          1
H    68.03754  73.85633  68.56446    -1380.08193     2212.14486      790.85944          1
```

Listing 31: C00031.xmolout

```
       276
   150.0000  150.0000  150.0000     0.0000     0.0000     0.0000***********    2.7750    11100
       0.2500    300.00    0.00    0.00    0.00   -1293.9028       0.0000    300.0000
H    88.06052  71.69157  76.34736    -2043.14101    -1757.64893      399.89339          1
N    88.33511  71.92573  75.39598      426.75047      490.45733     -334.42467          1
H    87.45597  72.21694  74.97664      442.25559    -1110.30854    -2448.52907          1
C    89.41160  72.82548  75.27339      415.32972     -666.39213       83.73172          1
H    90.15425  72.45848  76.01249      879.32219     2491.00447      667.35213          1
C    89.96395  72.77494  73.76276       31.35357      477.41777      628.23362          1
H    89.22069  73.24324  73.07432     1168.38489     2240.25337    -1019.71136          1
H    90.89088  73.39092  73.66708     -360.43770     2534.90498      931.67112          1
C    90.21046  71.34184  73.35902      463.18208      512.70242     -382.34388          1
H    89.34149  70.73231  73.69577     1725.29117     1863.52247     -987.07854          1
C    90.31690  71.23003  71.84383      710.42801     -144.27427     -313.15367          1
H    90.47483  70.16974  71.51732      364.20195    -1033.13725     2494.72524          1
H    89.38932  71.60007  71.33498     2567.61904      335.80679      847.48802          1
H    91.17526  71.83076  71.44627    -2587.99029      680.92867     -512.16411          1
C    91.47115  70.79267  74.01116      268.17616      741.64379      -40.42634          1
```

H	91.65780	69.72728	73.71734	1283.54314	-1945.37420	-1411.27812	1
H	92.37777	71.38211	73.71661	-1258.57796	1317.15249	-2026.02085	1
H	91.40434	70.82210	75.12941	-2502.84526	909.80597	575.92391	1
C	88.95983	74.35894	75.56220	619.36578	-458.64843	-172.09273	1
O	89.76845	75.24612	75.39406	-81.40433	433.87611	522.34594	1
N	87.67999	74.46231	75.91861	175.23996	-622.20190	341.55523	1
H	87.16548	73.60084	76.08873	-876.57256	-303.22879	-2561.90213	1
C	87.05139	75.71084	76.17669	-571.88214	-534.10783	-106.15033	1
H	86.02975	75.40539	76.49609	-2713.20841	-16.25800	-248.80809	1
C	87.89205	76.35109	77.38856	166.60354	288.16399	-716.09568	1
H	88.89672	76.64438	76.97394	-2097.85574	1636.78449	-586.18044	1
O	88.14692	75.45504	78.48662	-317.80580	-363.38227	484.43082	1
H	89.11399	75.33551	78.39868	1863.74748	-1451.84923	1357.29467	1
C	87.10553	77.64602	77.80322	503.80731	517.99032	318.51606	1
H	87.62785	78.18529	78.62824	640.61020	-2157.10410	-1536.29104	1
H	86.99359	78.37512	76.95030	1687.88455	465.60692	2087.56452	1
H	86.08207	77.39970	78.17107	-1293.07895	-1899.88581	-1463.57452	1
C	87.02529	76.64479	74.85112	-153.07550	-733.68902	248.70981	1
O	87.10163	77.83734	75.05026	186.15459	-480.78787	-449.34387	1
N	86.81760	76.03712	73.67007	-487.53139	543.18517	42.03850	1
C	86.83510	76.82762	72.48123	-699.35871	69.34393	360.10701	1
H	87.49910	77.69712	72.69546	2.81126	-221.99807	-2715.58072	1
C	87.37092	75.78139	71.41572	-507.46439	-592.59194	122.07761	1
H	87.12981	76.07086	70.36842	-1975.08116	525.87560	-1801.71556	1
H	88.47792	75.68080	71.47301	1588.27743	-1913.22100	1113.83566	1
C	86.66813	74.46302	71.86828	-613.62442	-457.55993	-194.14490	1
H	85.60983	74.43107	71.52554	1707.54637	-721.27992	1996.92523	1
H	87.15262	73.54065	71.47570	758.23357	2100.81767	1560.55011	1
C	86.77649	74.59787	73.44788	-352.41524	704.96076	49.21465	1
H	85.92357	74.17494	74.02964	1714.76886	-2070.42778	443.35792	1
H	87.73149	74.21311	73.87033	1224.15040	1801.50881	1636.97633	1
C	85.31195	77.31448	72.07237	566.85117	537.05831	117.62814	1
O	85.17231	78.34888	71.46828	-550.26779	-406.08867	4.21666	1
N	84.40750	76.41926	72.43061	566.34832	-425.77725	180.18749	1
H	84.73106	75.61354	72.96339	644.44502	2646.80550	-52.73430	1
C	83.04081	76.52617	72.06902	612.34801	-281.48662	411.56221	1
H	82.82435	77.60364	71.88760	1667.23498	-1490.75142	1556.16753	1
C	82.82148	75.62397	70.75418	-714.45746	-175.43074	286.98927	1
H	83.17330	74.57941	70.97630	-787.09484	1763.03619	-1922.46060	1
O	83.53324	76.04377	69.58155	-429.46514	516.51932	-128.40526	1
H	84.11214	75.26990	69.42261	1981.82785	436.16847	-1818.18182	1
C	81.25532	75.61632	70.61244	630.79189	-468.94448	76.03230	1
H	80.93628	75.07522	69.68839	-1775.71861	2062.30353	131.89849	1
H	80.75910	75.11278	71.47946	-1855.14608	88.94118	1993.53760	1
H	80.84156	76.64922	70.54399	-1504.82350	-780.94249	-2132.90992	1
C	82.25235	75.80580	73.29541	288.57638	618.62814	-397.00270	1
O	82.70638	74.81025	73.85132	321.70495	-185.26452	574.37218	1
N	81.10045	76.41069	73.51272	-447.05792	-446.53431	367.76425	1
H	80.81569	77.19197	72.92426	350.31316	1975.63644	-1843.31522	1
C	80.18702	75.76263	74.40205	-511.23061	374.03917	-471.51480	1
H	80.86412	75.37087	75.19650	2234.79579	-898.70890	-1273.45190	1
C	79.09053	76.82596	74.85930	529.53781	-159.34528	563.72604	1
H	78.52512	76.34829	75.70445	654.59388	2347.94251	1217.51488	1
H	79.59482	77.72663	75.29782	-1526.55661	2103.42026	817.87394	1
O	78.17116	77.23475	73.83276	-554.84278	14.12789	399.58769	1
H	77.91360	76.31509	73.46924	1297.06236	2061.84854	1220.68812	1
C	79.42917	74.56691	73.58876	-662.15484	-412.13544	123.62687	1
O	78.25905	74.68062	73.25247	-540.01537	-317.05804	274.91456	1
N	80.28089	73.58655	73.42882	-202.76299	-690.00100	-131.49145	1
H	81.26373	73.71603	73.75402	-702.62132	-2617.37191	281.70413	1
C	79.91112	72.38826	72.76176	-491.73914	576.34187	222.73509	1
H	80.78451	71.73580	72.98433	1977.17978	-1823.58850	-434.68928	1

C	78.55412	71.83578	73.37461	-649.50797	-328.66843	306.11224	1
H	77.71593	72.54124	73.17132	2075.07803	1452.16490	-1004.45904	1
C	78.27350	70.41587	72.77301	-615.18735	-489.99689	-70.96352	1
H	77.31847	69.98886	73.15854	-1226.32661	-1724.38906	-1716.47170	1
H	78.20083	70.44157	71.66037	2381.64273	-969.84735	900.46860	1
H	79.08621	69.69756	73.03184	2122.74991	1702.23946	-141.36228	1
C	78.73516	71.65557	74.92114	-501.86074	-479.61276	-376.42482	1
H	77.81656	71.24129	75.39850	993.79261	-2246.95401	-1177.81305	1
H	79.57661	70.96306	75.15729	42.71279	-626.15391	-2651.37258	1
H	78.95892	72.62984	75.41593	-2026.39012	-1590.33709	-887.82922	1
C	79.84270	72.72622	71.15564	-672.89153	382.57394	-156.34072	1
O	80.86772	73.01469	70.57703	410.04627	221.86223	500.35950	1
N	78.62899	72.60627	70.64719	-378.11782	-443.79414	-441.10984	1
H	77.84060	72.39821	71.25470	2404.15973	-568.14894	1149.30085	1
C	78.46643	72.80153	69.23363	-544.54484	466.16019	331.29119	1
H	79.27519	73.53957	69.02284	-1409.80988	-170.17955	2325.32679	1
C	78.81946	71.41144	68.54976	-184.48284	116.78282	-758.89137	1
H	79.86284	71.10761	68.79332	1893.78641	1861.14652	611.04528	1
H	78.18397	70.59714	68.96506	-2170.79665	-974.19311	1327.50117	1
C	78.63408	71.57250	67.00766	389.51668	672.81878	-138.49152	1
H	77.73632	72.19248	66.78705	510.64577	1933.03327	1851.02484	1
H	79.47278	72.16620	66.58190	-2302.30869	-1105.72939	-948.89774	1
C	78.50846	70.19888	66.24501	-559.93427	-126.34590	542.30857	1
O	78.25138	70.19868	65.06965	-670.36003	-111.83166	76.36448	1
N	78.70070	69.11094	67.02235	-216.06729	-537.07037	446.51379	1
H	78.98838	69.05457	68.00613	-1251.96487	1074.12894	-2168.52500	1
H	78.70643	68.14885	66.66587	-2451.83023	-720.73851	944.84612	1
C	76.97698	73.41291	69.04389	546.24052	-563.88337	-85.11809	1
O	76.86496	74.59266	69.36024	71.70702	-307.40463	606.69673	1
N	76.02616	72.59798	68.59802	479.90863	-504.50129	222.86849	1
C	74.79404	73.12769	68.07111	75.51268	-600.91904	506.73700	1
H	75.08344	74.04060	67.50077	-2033.61562	1795.30103	254.89480	1
C	74.26488	71.90025	67.21045	523.79067	517.99274	284.45581	1
H	73.17529	71.96521	66.99205	1599.61665	-1127.00911	1895.98173	1
H	74.78903	71.82496	66.23124	-1881.83093	1275.82488	-1501.55016	1
C	74.63436	70.70683	68.14405	-600.05639	-404.05026	316.64485	1
H	73.92341	70.63456	68.99770	-2597.05260	496.36221	657.73249	1
H	74.62061	69.71812	67.63211	-1666.24518	1294.38985	-1723.90591	1
C	76.08704	71.14272	68.62006	493.57570	-129.95376	-602.56394	1
H	76.37558	70.81608	69.64712	-1469.08415	-637.84797	2204.22581	1
H	76.89428	70.85682	67.90058	-1038.97840	1068.88701	-2280.71789	1
C	73.76072	73.45222	69.28205	188.88100	241.05089	-727.87821	1
O	72.67969	72.93431	69.30541	531.88613	-49.19943	427.07960	1
N	74.25826	74.36916	70.13624	708.14833	26.93417	-179.73509	1
H	75.23400	74.72609	69.96327	-2232.69501	-1179.09831	1023.94816	1
C	73.76899	74.30230	71.52360	-738.32412	259.92548	104.42456	1
H	72.69797	74.00601	71.42553	2028.89123	1260.02983	1311.33324	1
C	74.60795	73.29394	72.28530	-258.65678	-345.52513	661.28459	1
H	75.65366	73.67821	72.30928	-1826.06090	-1801.46097	-918.64554	1
C	74.12349	73.14286	73.71622	-541.23400	531.92970	-218.41428	1
H	74.74364	72.39956	74.28896	1311.73218	-2219.18200	882.19023	1
H	74.17046	74.11939	74.27348	-1048.71298	1223.58118	-2196.97971	1
H	73.05657	72.78768	73.75576	419.06414	1903.92898	1903.44670	1
C	74.64107	71.93356	71.61325	-423.86431	152.66566	-648.55262	1
H	75.26530	71.19307	72.18581	105.68386	-2342.50080	1387.51238	1
H	73.60807	71.49868	71.51019	-1695.63603	-1484.73621	-1531.02793	1
H	75.07000	72.00760	70.57760	-1853.57121	-487.39031	1936.59343	1
C	74.03182	75.77766	72.22794	539.71428	-510.58232	267.58633	1
O	75.05739	76.38908	72.05124	-15.93758	-278.34085	-624.49402	1
N	73.04405	76.06096	73.06862	-676.81886	12.05292	276.17960	1
H	72.22618	75.45552	73.04905	-1694.44250	-373.84189	-2100.66098	1
C	73.15611	77.23550	73.86999	412.33078	-664.70360	108.35451	1

142	H	74.24357	77.49037	73.83928	1765.77476	1294.44366	1621.76594	1
143	C	72.26452	78.36062	73.17999	-179.66635	-721.95667	-264.73915	1
144	H	72.41891	78.36675	72.07836	-607.34922	2587.87032	-598.10018	1
145	H	71.18229	78.15254	73.33565	-359.69332	-1479.13954	-2259.74260	1
146	C	72.67007	79.72766	73.81634	-240.01076	616.02425	431.85322	1
147	H	72.54870	79.69017	74.92903	-1359.17131	782.54425	2227.99181	1
148	H	73.74780	79.94139	73.63408	-820.36667	85.81767	-2596.78715	1
149	C	71.76254	80.86712	73.19953	-513.07015	236.18570	-551.87280	1
150	H	71.92518	80.95965	72.10139	1816.11944	-1183.11722	-1650.94321	1
151	H	70.68039	80.63426	73.32615	-1800.04429	-1361.53473	1526.34643	1
152	C	72.14479	82.17333	73.93509	726.08972	-253.19732	-179.64713	1
153	H	71.96349	82.15785	75.02843	1224.27271	-456.51798	-2390.90304	1
154	H	73.17454	82.52939	73.73241	1206.40339	-1150.93514	-2154.90344	1
155	N	71.21866	83.30920	73.39112	329.70796	-633.68968	-155.67358	1
156	H	71.40180	84.27709	73.78699	1348.31305	1938.76845	1359.00643	1
157	H	71.27403	83.50202	72.34907	2096.92035	1446.54551	-966.48890	1
158	H	70.16993	83.21171	73.55009	-1197.07217	-2303.53719	827.28733	1
159	C	72.54137	77.02505	75.35807	-378.01186	-556.17301	-413.96481	1
160	O	72.52797	77.98418	76.13999	-409.44433	-307.55053	-453.30833	1
161	N	72.10608	75.81076	75.56505	-429.02477	524.70884	274.08502	1
162	H	72.21938	75.15271	74.79746	1706.34263	-1425.96036	1574.38926	1
163	C	71.37596	75.47090	76.74568	-45.25864	-626.22952	478.93267	1
164	H	71.15772	74.38619	76.61701	1988.63164	570.90773	1772.87287	1
165	C	72.40390	75.76405	77.92517	551.77128	-20.49526	-564.54996	1
166	H	73.42428	75.50172	77.56778	1434.61535	-1753.10253	1513.99471	1
167	H	72.44770	76.85987	78.12489	-714.67738	1819.09770	1898.36494	1
168	C	72.11119	74.99247	79.24926	446.99263	-555.77305	338.97221	1
169	H	71.27609	75.45532	79.82501	1953.68466	1892.25957	-161.67766	1
170	H	71.79787	73.94311	79.04841	-403.03785	2688.07032	-188.43591	1
171	C	73.44422	75.02739	80.10383	567.86262	335.74681	434.04421	1
172	H	74.27234	74.49875	79.57860	-1080.02275	1568.26081	-1948.78900	1
173	H	73.79613	76.07463	80.25110	-919.54108	2111.34692	1456.13463	1
174	C	73.12299	74.35604	81.45930	195.81897	173.33862	-745.11536	1
175	H	72.43687	74.93932	82.10804	1405.67949	-2301.21089	-390.08113	1
176	H	72.76458	73.30851	81.38080	-940.37328	-1118.32446	2299.72133	1
177	N	74.44942	74.24177	82.28318	319.26422	656.36007	-42.02424	1
178	H	74.33877	73.84456	83.26041	1961.03633	619.91968	1787.09422	1
179	H	75.20875	73.61229	81.89129	854.14343	-2156.80752	1429.08711	1
180	H	74.98358	75.13722	82.48076	1237.02905	-1378.50857	-1998.28500	1
181	C	70.03886	76.38305	76.73919	532.53319	-268.57236	517.55729	1
182	O	69.55897	76.85196	75.71012	266.16714	-290.73804	-558.88002	1
183	N	69.54455	76.41365	77.96401	-226.25352	640.49678	-270.32839	1
184	H	69.98900	75.77718	78.63529	2007.69063	-1035.27229	1523.50231	1
185	C	69.06705	77.63177	78.54086	-540.76542	-573.33303	49.50000	1
186	H	69.21260	77.47263	79.63201	-2374.20950	-1036.76023	843.75774	1
187	C	69.97333	78.82792	77.98610	-77.09082	666.87422	-415.84136	1
188	H	69.99888	78.77101	76.85960	-985.81834	617.19825	2463.91927	1
189	O	71.33675	78.80018	78.38715	293.29899	-391.65602	-477.80926	1
190	H	71.80511	78.54325	77.49534	2528.72714	-681.76262	-751.27122	1
191	C	69.14329	80.08632	78.42843	252.47363	-605.14473	440.05134	1
192	H	69.60181	81.03040	78.05208	-2052.65063	1456.49051	-1043.51830	1
193	H	68.09681	80.05184	78.04308	-2152.98800	-1661.97574	-161.70652	1
194	H	69.08628	80.16502	79.53928	-473.12726	-1584.27339	2165.61725	1
195	C	67.48245	77.80488	78.19820	360.36307	488.46713	505.10056	1
196	O	66.71742	78.15767	79.07142	-200.08817	-528.03552	-385.82850	1
197	N	67.22086	77.56691	76.91964	-597.54095	-226.44775	355.20479	1
198	H	67.98711	77.26927	76.26666	2179.23655	-1300.70981	-991.33835	1
199	C	65.92851	77.91933	76.43791	-687.68921	297.39807	-249.45179	1
200	H	65.57593	78.68842	77.16331	-349.16256	-2467.37713	-1101.72831	1
201	C	66.09061	78.42921	74.93197	672.47345	361.54328	-201.62880	1
202	H	66.62061	77.66218	74.32211	1541.41502	582.18425	2169.97026	1
203	H	65.09083	78.58806	74.46663	-2534.67717	60.25153	997.72295	1

204	C	66.89280	79.73897	74.98318	574.26774	-389.43119	-377.02542	1
205	C	66.19884	80.98536	74.97478	688.82679	-287.33580	-257.96133	1
206	H	65.09731	81.01879	74.89344	331.14182	1470.38163	2269.80013	1
207	C	68.31494	79.70328	75.10183	-383.52382	-675.04808	144.24917	1
208	H	68.85319	78.72915	75.13008	4.09847	1517.59662	2262.86397	1
209	C	66.94212	82.16295	75.07585	-134.76730	428.45285	-649.50303	1
210	H	66.40644	83.12848	75.06439	-903.23433	1600.49020	2011.53387	1
211	C	69.01206	80.91101	75.21590	-335.53281	-615.00040	-364.39058	1
212	H	70.10916	80.87166	75.33287	1596.45398	-1369.33862	1732.02659	1
213	C	68.36049	82.17733	75.19661	585.48547	-340.24194	-406.23938	1
214	O	69.05862	83.41945	75.29768	-419.15649	-173.04408	-511.94141	1
215	H	69.98254	83.08245	75.33102	-1071.84350	1559.71507	-1960.13012	1
216	C	65.01199	76.57108	76.49942	291.41878	-183.13091	710.72246	1
217	O	64.25932	76.25282	75.61240	379.87695	523.47396	-222.23622	1
218	N	65.17651	76.01259	77.70484	564.30591	373.09380	277.24371	1
219	H	65.89339	76.44284	78.30690	-120.59931	-2436.07824	-1214.35098	1
220	C	64.83993	74.61234	77.83023	404.07514	-299.37480	608.84009	1
221	H	63.73962	74.63588	77.66588	-1071.07882	-841.69025	2359.66467	1
222	C	65.56774	73.79337	76.69277	-683.12807	68.71770	-390.13096	1
223	H	65.40272	74.27493	75.69912	2068.88918	1596.58590	770.89603	1
224	H	66.67017	73.77521	76.85151	1379.86429	2342.49779	-179.85638	1
225	C	64.95723	72.35665	76.74151	-176.39967	537.49466	550.97268	1
226	H	65.17517	71.87886	77.72574	-396.28051	1942.22168	1869.33314	1
227	H	63.84800	72.39842	76.66418	-94.44700	586.00678	-2659.20016	1
228	C	65.57262	71.51001	75.53577	-291.69381	-718.18079	-150.72929	1
229	H	65.45945	71.95494	74.51452	-2303.38776	1089.76242	964.62032	1
230	H	66.64291	71.20783	75.66797	884.16729	2568.80533	-207.74283	1
231	N	64.78088	70.35204	75.58434	-594.49522	-252.81478	342.29276	1
232	H	64.20130	70.23106	76.40059	-1735.17426	2084.05705	-263.71605	1
233	C	64.75043	69.45358	74.68589	595.23177	-271.52487	442.22352	1
234	N	65.56833	69.62110	73.56323	585.53813	-424.79750	-105.81303	1
235	H	66.22201	70.37147	73.34320	959.88797	1327.56823	-2177.11905	1
236	H	65.63208	68.96374	72.78518	1578.48647	16.27378	-2220.76233	1
237	N	63.85694	68.42795	75.02662	-452.17763	-390.33541	-421.51853	1
238	H	63.25526	68.30664	75.83993	470.26107	1974.25629	-1817.92087	1
239	H	63.68572	67.61689	74.43096	1856.67307	-1573.97321	-1224.35416	1
240	C	65.28252	74.13198	79.31068	-531.47271	-581.45780	-55.06206	1
241	O	65.96197	73.11861	79.47644	-243.00837	192.73507	609.52485	1
242	N	64.80686	74.96389	80.22100	343.49288	-644.25607	38.09164	1
243	H	64.31352	75.74505	79.78684	-1691.16043	-1535.97525	-1484.73099	1
244	C	64.59585	74.63334	81.62044	542.66492	350.14337	454.42563	1
245	H	63.86189	75.39891	81.96968	1209.57979	-2396.95920	463.86744	1
246	C	64.08940	73.20241	81.74196	-737.14309	145.93010	242.71942	1
247	H	64.85807	72.53072	81.29061	-1050.18303	159.36419	-2509.06125	1
248	C	63.90745	72.80125	83.19311	-706.77696	-252.88343	-245.16230	1
249	H	63.54771	71.73976	83.28929	-1624.64039	-1665.49085	1417.86935	1
250	H	64.87579	72.88330	83.77228	2566.10521	598.51109	693.22305	1
251	H	63.15447	73.45622	83.71185	-1972.69758	107.10018	-1876.34312	1
252	C	62.79903	72.98658	80.97311	412.85148	672.58832	-27.69222	1
253	H	62.45575	71.91701	81.02836	1447.02277	1762.76533	-1490.78944	1
254	H	61.96579	73.62595	81.37448	1320.77379	-145.20455	2378.68491	1
255	H	62.92159	73.24307	79.88486	-1512.10848	1470.52031	1724.75147	1
256	C	66.05046	74.76532	82.42287	-607.11113	499.56689	73.73769	1
257	O	66.10131	75.39677	83.44653	357.97126	460.81534	356.68785	1
258	N	66.99388	74.06633	81.82045	536.55407	-368.62270	332.76306	1
259	H	66.77125	73.51154	80.95744	524.38866	2297.69985	1367.20941	1
260	C	68.26009	74.00937	82.46300	46.50277	-774.29190	-147.97904	1
261	H	68.24112	74.85929	83.18571	1676.51875	1883.29066	1032.55549	1
262	C	68.42717	72.56967	83.19133	-559.41456	-34.19198	-556.30433	1
263	H	68.25053	71.75613	82.44873	-64.66664	395.18769	2695.05373	1
264	H	69.47990	72.44579	83.53929	-2703.44278	-60.40056	333.79418	1
265	C	67.46935	72.45103	84.34133	-519.76435	377.31045	459.42366	1

```
H    66.42760  72.60324  83.95956    -1746.74323     1667.50644    -1261.73691          1
C    67.56561  71.05871  84.95447      570.95554       16.46261     -545.27700          1
H    66.85808  70.93467  85.81405     2391.58273       28.05277    -1305.07271          1
H    67.32395  70.26075  84.20596      175.75588      917.63458     2559.43867          1
H    68.59667  70.84987  85.34001    -1063.97851    -2155.34650     1283.00462          1
C    67.75218  73.50149  85.40685      193.76014        2.62946     -765.53141          1
H    67.16018  73.31584  86.33966      507.39394     -846.55087     2539.60089          1
H    68.83468  73.51572  85.69706     1337.57841     1818.19054     1526.01994          1
H    67.48498  74.53169  85.04750     1100.88635    -1172.91350    -2199.08909          1
C    69.42728  74.09140  81.36620     -549.21115      475.64486     -309.38218          1
O    70.55327  73.74016  81.61468      556.06106     -390.37755      -78.24250          1
O    69.13475  74.72082  80.12417     -572.27940     -310.19032     -209.75862          1
H    68.14849  74.81136  80.23576    -1380.08193     2212.14486      790.85944          1
```

Listing 32: C00032.xmolout

```
       276
  150.0000  150.0000  150.0000     0.0000     0.0000     0.0000***********    2.7750    11100
       0.2500     300.00    0.00    0.00    0.00   -1293.8923       0.0000    300.0000
H    75.92431  66.32038  83.84679    -2043.14101    -1757.64893      399.89339          1
N    76.30681  66.42121  82.90814      426.75047      490.45733     -334.42467          1
H    75.47084  66.58558  82.34694      442.25559    -1110.30854    -2448.52907          1
C    77.23606  67.47390  82.76453      415.32972     -666.39213       83.73172          1
H    77.83959  67.51467  83.69615      879.32219     2491.00447      667.35213          1
C    78.10009  67.16860  81.44120       31.35357      477.41777      628.23362          1
H    77.43072  67.22582  80.54873     1168.38489     2240.25337    -1019.71136          1
H    78.88367  67.95014  81.28944     -360.43770     2534.90498      931.67112          1
C    78.70873  65.79222  81.55144      463.18208      512.70242     -382.34388          1
H    77.90940  65.08979  81.88058     1725.29117     1863.52247     -987.07854          1
C    79.23045  65.32350  80.19968      710.42801     -144.27427     -313.15367          1
H    79.65889  64.28931  80.25769      364.20195    -1033.13725     2494.72524          1
H    78.41917  65.30382  79.42641     2567.61904      335.80679      847.48802          1
H    80.03964  65.99620  79.81420    -2587.99029      680.92867     -512.16411          1
C    79.83439  65.75898  82.57529      268.17616      741.64379      -40.42634          1
H    80.28456  64.73590  82.66077     1283.54314    -1945.37420    -1411.27812          1
H    80.66192  66.46362  82.30173    -1258.57796     1317.15249    -2026.02085          1
H    79.47415  66.04850  83.59608    -2502.84526      909.80597      575.92391          1
C    76.30241  68.79353  82.50561      619.36578     -458.64843     -172.09273          1
O    75.41442  68.77615  81.67636      -81.40433      433.87611      522.34594          1
N    76.68021  69.77483  83.30827      175.23996     -622.20190      341.55523          1
H    77.48696  69.61887  83.91053     -876.57256     -303.22879    -2561.90213          1
C    76.07206  71.06127  83.20234     -571.88214     -534.10783     -106.15033          1
H    75.00387  70.87963  82.95100    -2713.20841      -16.25800     -248.80809          1
C    76.32167  71.75734  84.62172      166.60354      288.16399     -716.09568          1
H    77.42683  71.74458  84.82704    -2097.85574     1636.78449     -586.18044          1
O    75.70638  71.08144  85.73713     -317.80580     -363.38227      484.43082          1
H    76.49012  70.83797  86.26592     1863.74748    -1451.84923     1357.29467          1
C    75.86924  73.24718  84.41326      503.80731      517.99032      318.51606          1
H    76.08150  73.86322  85.31851      640.61020    -2157.10410    -1536.29104          1
H    76.39401  73.74599  83.54394     1687.88455      465.60692     2087.56452          1
H    74.76996  73.31549  84.22643    -1293.07895    -1899.88581    -1463.57452          1
C    76.86784  71.85845  82.02852     -153.07550     -733.68902      248.70981          1
O    78.05458  71.69952  81.91231      186.15459     -480.78787     -449.34387          1
N    76.06215  72.70355  81.36449     -487.53139      543.18517       42.03850          1
C    76.58825  73.58375  80.36910     -699.35871       69.34393      360.10701          1
H    77.50685  73.08667  79.97759        2.81126     -221.99807    -2715.58072          1
C    75.34695  73.67409  79.37598     -507.46439     -592.59194      122.07761          1
H    75.40753  74.51488  78.65200    -1975.08116      525.87560    -1801.71556          1
H    75.24029  72.74563  78.77304     1588.27743    -1913.22100     1113.83566          1
C    74.19526  73.83236  80.41442     -613.62442     -457.55993     -194.14490          1
H    74.16276  74.86878  80.81953     1707.54637     -721.27992     1996.92523          1
H    73.18270  73.62971  79.99202      758.23357     2100.81767     1560.55011          1
```

C	74.61987	72.79466	81.54111	-352.41524	704.96076	49.21465	1
H	74.40652	73.13351	82.58237	1714.76886	-2070.42778	443.35792	1
H	74.20682	71.76390	81.41088	1224.15040	1801.50881	1636.97633	1
C	76.89437	75.03484	81.07342	566.85117	537.05831	117.62814	1
O	77.12453	75.10994	82.26098	-550.26779	-406.08867	4.21666	1
N	76.87925	75.99895	80.16971	566.34832	-425.77725	180.18749	1
H	76.69909	75.75646	79.19875	644.44502	2646.80550	-52.73430	1
C	77.07892	77.34703	80.60510	612.34801	-281.48662	411.56221	1
H	77.95436	77.30605	81.29215	1667.23498	-1490.75142	1556.16753	1
C	77.28473	78.24270	79.30179	-714.45746	-175.43074	286.98927	1
H	76.39968	78.08588	78.62755	-787.09484	1763.03619	-1922.46060	1
O	78.45467	77.94413	78.50958	-429.46514	516.51932	-128.40526	1
H	78.04797	77.63869	77.67777	1981.82785	436.16847	-1818.18182	1
C	77.22172	79.69938	79.88943	630.79189	-468.94448	76.03230	1
H	77.24407	80.46853	79.08319	-1775.71861	2062.30353	131.89849	1
H	76.29175	79.87008	80.48340	-1855.14608	88.94118	1993.53760	1
H	78.08526	79.90046	80.56516	-1504.82350	-780.94249	-2132.90992	1
C	75.74296	77.85319	81.39596	288.57638	618.62814	-397.00270	1
O	75.87447	78.59946	82.33784	321.70495	-185.26452	574.37218	1
N	74.60700	77.44184	80.81210	-447.05792	-446.53431	367.76425	1
H	74.72149	76.85903	79.98131	350.31316	1975.63644	-1843.31522	1
C	73.53078	78.38515	80.88277	-511.23061	374.03917	-471.51480	1
H	73.98401	79.30674	81.31559	2234.79579	-898.70890	-1273.45190	1
C	72.99004	78.55262	79.39034	529.53781	-159.34528	563.72604	1
H	72.18868	79.33508	79.42647	654.59388	2347.94251	1217.51488	1
H	73.82963	78.97909	78.77804	-1526.55661	2103.42026	817.87394	1
O	72.54078	77.33942	78.71657	-554.84278	14.12789	399.58769	1
H	71.64874	77.26779	79.10188	1297.06236	2061.84854	1220.68812	1
C	72.36939	77.80151	81.86843	-662.15484	-412.13544	123.62687	1
O	72.00329	78.50240	82.78484	-540.01537	-317.05804	274.91456	1
N	71.83480	76.62581	81.45679	-202.76299	-690.00100	-131.49145	1
H	72.42511	76.25041	80.70816	-702.62132	-2617.37191	281.70413	1
C	71.40720	75.77427	82.54682	-491.73914	576.34187	222.73509	1
H	72.09129	76.00284	83.39254	1977.17978	-1823.58850	-434.68928	1
C	69.89097	76.07065	82.88013	-649.50797	-328.66843	306.11224	1
H	69.26455	75.90102	81.97762	2075.07803	1452.16490	-1004.45904	1
C	69.49553	75.07982	84.03023	-615.18735	-489.99689	-70.96352	1
H	68.41387	75.15753	84.29008	-1226.32661	-1724.38906	-1716.47170	1
H	69.69721	74.01931	83.73821	2381.64273	-969.84735	900.46860	1
H	70.07751	75.27907	84.96026	2122.74991	1702.23946	-141.36228	1
C	69.65166	77.52149	83.42669	-501.86074	-479.61276	-376.42482	1
H	68.62985	77.63944	83.85873	993.79261	-2246.95401	-1177.81305	1
H	70.38859	77.78326	84.22530	42.71279	-626.15391	-2651.37258	1
H	69.75926	78.28414	82.61721	-2026.39012	-1590.33709	-887.82922	1
C	71.42079	74.22236	82.06461	-672.89153	382.57394	-156.34072	1
O	71.68715	73.33416	82.84516	410.04627	221.86223	500.35950	1
N	70.99817	74.11079	80.80315	-378.11782	-443.79414	-441.10984	1
H	70.82100	74.96624	80.27774	2404.15973	-568.14894	1149.30085	1
C	70.54188	72.83774	80.36871	-544.54484	466.16019	331.29119	1
H	70.43825	72.21815	81.29066	-1409.80988	-170.17955	2325.32679	1
C	69.21462	73.11597	79.55304	-184.48284	116.78282	-758.89137	1
H	68.42479	73.53018	80.21975	1893.78641	1861.14652	611.04528	1
H	69.40218	73.89748	78.78249	-2170.79665	-974.19311	1327.50117	1
C	68.73176	71.79452	78.87426	389.51668	672.81878	-138.49152	1
H	69.54042	71.39441	78.21869	510.64577	1933.03327	1851.02484	1
H	68.57063	71.00246	79.64144	-2302.30869	-1105.72939	-948.89774	1
C	67.42341	71.95835	78.01056	-559.93427	-126.34590	542.30857	1
O	66.95035	70.98709	77.48104	-670.36003	-111.83166	76.36448	1
N	66.95507	73.22196	77.93647	-216.06729	-537.07037	446.51379	1
H	67.34197	74.08608	78.33355	-1251.96487	1074.12894	-2168.52500	1
H	66.14520	73.49920	77.37069	-2451.83023	-720.73851	944.84612	1
C	71.67498	72.26252	79.31547	546.24052	-563.88337	-85.11809	1

O	71.87839	72.94721	78.28559	71.70702	-307.40463	606.69673	1
N	72.25988	71.12905	79.64931	479.90863	-504.50129	222.86849	1
C	73.53364	70.51029	79.43135	75.51268	-600.91904	506.73700	1
H	74.27553	71.19821	79.89774	-2033.61562	1795.30103	254.89480	1
C	73.29493	69.15986	80.22576	523.79067	517.99274	284.45581	1
H	74.00566	68.35067	79.92665	1599.61665	-1127.00911	1895.98173	1
H	73.44179	69.31927	81.32666	-1881.83093	1275.82488	-1501.55016	1
C	71.80460	68.85397	79.92593	-600.05639	-404.05026	316.64485	1
H	71.66800	68.48418	78.88869	-2597.05260	496.36221	657.73249	1
H	71.36679	68.08555	80.59644	-1666.24518	1294.38985	-1723.90591	1
C	71.12111	70.32901	80.12521	493.57570	-129.95376	-602.56394	1
H	70.19756	70.52698	79.51225	-1469.08415	-637.84797	2204.22581	1
H	70.94009	70.60177	81.20239	-1038.97840	1068.88701	-2280.71789	1
C	73.88130	70.31447	77.85661	188.88100	241.05089	-727.87821	1
O	74.19385	69.24702	77.39129	531.88613	-49.19943	427.07960	1
N	73.88663	71.52443	77.28875	708.14833	26.93417	-179.73509	1
H	73.47172	72.30963	77.83059	-2232.69501	-1179.09831	1023.94816	1
C	73.92073	71.59874	75.85691	-738.32412	259.92548	104.42456	1
H	74.74884	70.92573	75.54041	2028.89123	1260.02983	1311.33324	1
C	72.50874	71.13529	75.28858	-258.65678	-345.52513	661.28459	1
H	71.68931	71.74363	75.72955	-1826.06090	-1801.46097	-918.64554	1
C	72.57595	71.33367	73.73629	-541.23400	531.92970	-218.41428	1
H	71.62518	71.02945	73.23896	1311.73218	-2219.18200	882.19023	1
H	72.76229	72.40257	73.47079	-1048.71298	1223.58118	-2196.97971	1
H	73.39866	70.73348	73.28119	419.06414	1903.92898	1903.44670	1
C	72.27049	69.61690	75.59482	-423.86431	152.66566	-648.55262	1
H	71.41898	69.20521	75.00269	105.68386	-2342.50080	1387.51238	1
H	73.17494	69.00405	75.35916	-1695.63603	-1484.73621	-1531.02793	1
H	72.03295	69.45236	76.67410	-1853.57121	-487.39031	1936.59343	1
C	74.09727	73.16178	75.45692	539.71428	-510.58232	267.58633	1
O	74.93826	73.47921	74.62734	-15.93758	-278.34085	-624.49402	1
N	73.22259	73.90678	76.09266	-676.81886	12.05292	276.17960	1
H	72.55506	73.49774	76.79032	-1694.44250	-373.84189	-2100.66098	1
C	73.08825	75.28872	75.79099	412.33078	-664.70360	108.35451	1
H	72.37880	75.64668	76.57212	1765.77476	1294.44366	1621.76594	1
C	74.52518	75.94799	75.89189	-179.66635	-721.95667	-264.73915	1
H	75.06876	75.56686	76.78483	-607.34922	2587.87032	-598.10018	1
H	75.15888	75.66864	75.01806	-359.69332	-1479.13954	-2259.74260	1
C	74.33555	77.49826	75.97290	-240.01076	616.02425	431.85322	1
H	73.71916	77.88414	75.13093	-1359.17131	782.54425	2227.99181	1
H	73.79977	77.78229	76.90999	-820.36667	85.81767	-2596.78715	1
C	75.80181	78.08903	75.92997	-513.07015	236.18570	-551.87280	1
H	76.45222	77.54128	76.64666	1816.11944	-1183.11722	-1650.94321	1
H	76.26198	77.92466	74.92839	-1800.04429	-1361.53473	1526.34643	1
C	75.76408	79.59530	76.27596	726.08972	-253.19732	-179.64713	1
H	75.31216	80.23968	75.49505	1224.27271	-456.51798	-2390.90304	1
H	75.32629	79.83468	77.26538	1206.40339	-1150.93514	-2154.90344	1
N	77.25739	80.05635	76.38759	329.70796	-633.68968	-155.67358	1
H	77.41012	81.08389	76.60102	1348.31305	1938.76845	1359.00643	1
H	77.85161	79.59368	77.13828	2096.92035	1446.54551	-966.48890	1
H	77.86207	79.93327	75.52422	-1197.07217	-2303.53719	827.28733	1
C	72.47076	75.45856	74.27277	-378.01186	-556.17301	-413.96481	1
O	71.34403	75.87833	74.18191	-409.44433	-307.55053	-453.30833	1
N	73.32901	75.12669	73.32640	-429.02477	524.70884	274.08502	1
H	74.25994	74.70967	73.55631	1706.34263	-1425.96036	1574.38926	1
C	72.93464	75.32041	71.95431	-45.25864	-626.22952	478.93267	1
H	72.05124	74.66498	71.78683	1988.63164	570.90773	1772.87287	1
C	72.56112	76.84711	71.80294	551.77128	-20.49526	-564.54996	1
H	71.74672	77.12966	72.51494	1434.61535	-1753.10253	1513.99471	1
H	73.43900	77.48346	72.07421	-714.67738	1819.09770	1898.36494	1
C	72.10872	77.12524	70.33088	446.99263	-555.77305	338.97221	1
H	72.90526	76.83778	69.60815	1953.68466	1892.25957	-161.67766	1

H	71.21103	76.52682	70.05780	-403.03785	2688.07032	-188.43591	1
C	71.81248	78.67667	70.26345	567.86262	335.74681	434.04421	1
H	70.87383	78.93194	70.80726	-1080.02275	1568.26081	-1948.78900	1
H	72.61476	79.25713	70.77163	-919.54108	2111.34692	1456.13463	1
C	71.72331	79.04858	68.76483	195.81897	173.33862	-745.11536	1
H	72.64357	78.84148	68.18231	1405.67949	-2301.21089	-390.08113	1
H	70.84049	78.63298	68.23911	-940.37328	-1118.32446	2299.72133	1
N	71.54781	80.60129	68.68491	319.26422	656.36007	-42.02424	1
H	71.43700	80.99933	67.70789	1961.03633	619.91968	1787.09422	1
H	70.70371	81.01617	69.17689	854.14343	-2156.80752	1429.08711	1
H	72.33889	81.19787	69.06579	1237.02905	-1378.50857	-1998.28500	1
C	74.21515	75.00122	71.00625	532.53319	-268.57236	517.55729	1
O	75.34265	75.20518	71.38053	266.16714	-290.73804	-558.88002	1
N	73.78014	74.74635	69.74760	-226.25352	640.49678	-270.32839	1
H	72.77239	74.56683	69.73743	2007.69063	-1035.27229	1523.50231	1
C	74.56919	73.81610	68.96802	-540.76542	-573.33303	49.50000	1
H	74.94998	73.08225	69.71113	-2374.20950	-1036.76023	843.75774	1
C	73.53197	73.21462	67.93307	-77.09082	666.87422	-415.84136	1
H	73.12294	74.03528	67.28035	-985.81834	617.19825	2463.91927	1
O	72.35257	72.61367	68.52312	293.29899	-391.65602	-477.80926	1
H	71.64205	73.12290	68.09006	2528.72714	-681.76262	-751.27122	1
C	74.44753	72.27400	67.06654	252.47363	-605.14473	440.05134	1
H	73.88080	71.82267	66.21914	-2052.65063	1456.49051	-1043.51830	1
H	75.31264	72.82561	66.62668	-2152.98800	-1661.97574	-161.70652	1
H	74.86064	71.43460	67.67255	-473.12726	-1584.27339	2165.61725	1
C	75.78904	74.59778	68.22285	360.36307	488.46713	505.10056	1
O	76.91705	74.43182	68.62910	-200.08817	-528.03552	-385.82850	1
N	75.39490	75.33210	67.18391	-597.54095	-226.44775	355.20479	1
H	74.40034	75.43247	66.98868	2179.23655	-1300.70981	-991.33835	1
C	76.38337	76.06849	66.47280	-687.68921	297.39807	-249.45179	1
H	77.33814	75.54459	66.71735	-349.16256	-2467.37713	-1101.72831	1
C	76.01758	76.09575	64.91374	672.47345	361.54328	-201.62880	1
H	75.01440	76.55184	64.75485	1541.41502	582.18425	2169.97026	1
H	76.75718	76.73186	64.37136	-2534.67717	60.25153	997.72295	1
C	76.05317	74.65397	64.38842	574.26774	-389.43119	-377.02542	1
C	77.31074	74.04376	64.09907	688.82679	-287.33580	-257.96133	1
H	78.25732	74.60394	64.24446	331.14182	1470.38163	2269.80013	1
C	74.83938	73.92266	64.21586	-383.52382	-675.04808	144.24917	1
H	73.86521	74.38670	64.45250	4.09847	1517.59662	2262.86397	1
C	77.32401	72.72866	63.63492	-134.76730	428.45285	-649.50303	1
H	78.29751	72.25733	63.41151	-903.23433	1600.49020	2011.53387	1
C	74.90460	72.60962	63.74337	-335.53281	-615.00040	-364.39058	1
H	73.95911	72.05628	63.60701	1596.45398	-1369.33862	1732.02659	1
C	76.13760	71.96456	63.43417	585.48547	-340.24194	-406.23938	1
O	76.21280	70.63075	62.95459	-419.15649	-173.04408	-511.94141	1
H	75.25142	70.41501	62.91040	-1071.84350	1559.71507	-1960.13012	1
C	76.33938	77.58550	67.05237	291.41878	-183.13091	710.72246	1
O	76.40127	78.58067	66.33545	379.87695	523.47396	-222.23622	1
N	76.26707	77.53885	68.36847	564.30591	373.09380	277.24371	1
H	76.27289	76.61980	68.81681	-120.59931	-2436.07824	-1214.35098	1
C	76.10526	78.78645	69.05580	404.07514	-299.37480	608.84009	1
H	75.47504	79.37049	68.34442	-1071.07882	-841.69025	2359.66467	1
C	75.45580	78.48980	70.46858	-683.12807	68.71770	-390.13096	1
H	74.57882	77.81231	70.36743	2068.88918	1596.58590	770.89603	1
H	76.18010	77.94345	71.11564	1379.86429	2342.49779	-179.85638	1
C	75.04990	79.85106	71.10926	-176.39967	537.49466	550.97268	1
H	75.87071	80.59409	71.00569	-396.28051	1942.22168	1869.33314	1
H	74.17922	80.30316	70.58650	-94.44700	586.00678	-2659.20016	1
C	74.73627	79.57596	72.66154	-291.69381	-718.18079	-150.72929	1
H	74.07322	78.69726	72.88220	-2303.38776	1089.76242	964.62032	1
H	75.67378	79.53577	73.27442	884.16729	2568.80533	-207.74283	1
N	74.04970	80.75558	72.94854	-594.49522	-252.81478	342.29276	1

```
H    74.54054   81.61287   72.73368    -1735.17426     2084.05705     -263.71605          1
C    72.88949   80.84597   73.47534      595.23177     -271.52487      442.22352          1
N    72.20353   79.67643   73.80337      585.53813     -424.79750     -105.81303          1
H    72.47998   78.70192   73.69749      959.88797     1327.56823    -2177.11905          1
H    71.28029   79.64122   74.23790     1578.48647       16.27378    -2220.76233          1
N    72.53100   82.18954   73.63015     -452.17763     -390.33541     -421.51853          1
H    73.02902   83.04673   73.39331      470.26107     1974.25629    -1817.92087          1
H    71.64506   82.48986   74.03921     1856.67307    -1573.97321    -1224.35416          1
C    77.59480   79.43860   69.22120     -531.47271     -581.45780      -55.06206          1
O    78.18065   79.40809   70.26990     -243.00837      192.73507      609.52485          1
N    77.93716   79.98957   68.05667      343.49288     -644.25607       38.09164          1
H    77.30657   79.82105   67.23456    -1691.16043    -1535.97525    -1484.73099          1
C    79.20753   80.68962   67.84469      542.66492      350.14337      454.42563          1
H    78.91642   81.48936   67.12298     1209.57979    -2396.95920      463.86744          1
C    79.74911   81.25794   69.13952     -737.14309      145.93010      242.71942          1
H    79.93458   80.40573   69.84457    -1050.18303      159.36419    -2509.06125          1
C    81.06503   81.99200   68.95327     -706.77696     -252.88343     -245.16230          1
H    81.43631   82.42979   69.92056    -1624.64039    -1665.49085     1417.86935          1
H    81.87284   81.30942   68.56551     2566.10521      598.51109      693.22305          1
H    80.96667   82.83867   68.22014    -1972.69758      107.10018    -1876.34312          1
C    78.74946   82.18845   69.80590      412.85148      672.58832      -27.69222          1
H    79.14209   82.59864   70.77591     1447.02277     1762.76533    -1490.78944          1
H    78.49464   83.06467   69.14961     1320.77379     -145.20455     2378.68491          1
H    77.78886   81.64867   70.03618    -1512.10848     1470.52031     1724.75147          1
C    80.27127   79.65148   67.11704     -607.11113      499.56689       73.73769          1
O    81.35105   80.07758   66.75760      357.97126      460.81534      356.68785          1
N    79.78983   78.43206   66.95680      536.55407     -368.62270      332.76306          1
H    78.86478   78.27062   67.35021      524.38866     2297.69985     1367.20941          1
C    80.66036   77.40331   66.50203       46.50277     -774.29190     -147.97904          1
H    81.48818   77.95842   66.00284     1676.51875     1883.29066     1032.55549          1
C    81.17604   76.49700   67.73632     -559.41456      -34.19198     -556.30433          1
H    80.29825   76.03013   68.24410      -64.66664      395.18769     2695.05373          1
H    81.78291   75.64903   67.33808    -2703.44278      -60.40056      333.79418          1
C    81.97974   77.30694   68.71629     -519.76435      377.31045      459.42366          1
H    81.31972   78.09473   69.14619    -1746.74323     1667.50644    -1261.73691          1
C    82.47530   76.40844   69.84395      570.95554       16.46261     -545.27700          1
H    83.04982   76.98621   70.61282     2391.58273       28.05277    -1305.07271          1
H    81.62628   75.90243   70.37203      175.75588      917.63458     2559.43867          1
H    83.15330   75.60307   69.45998    -1063.97851    -2155.34650     1283.00462          1
C    83.16830   77.98653   68.05258      193.76014        2.62946     -765.53141          1
H    83.85680   78.44736   68.80729      507.39394     -846.55087     2539.60089          1
H    83.77598   77.26578   67.44637     1337.57841     1818.19054     1526.01994          1
H    82.82972   78.81676   67.36162     1100.88635    -1172.91350    -2199.08909          1
C    79.81407   76.44563   65.53158     -549.21115      475.64486     -309.38218          1
O    79.91079   75.24566   65.55038      556.06106     -390.37755      -78.24250          1
O    79.00618   77.08614   64.55868     -572.27940     -310.19032     -209.75862          1
H    79.10265   78.02774   64.88013    -1380.08193     2212.14486      790.85944          1
```

Listing 33: C00033.xmolout

```
       276
  150.0000  150.0000  150.0000     0.0000     0.0000     0.0000***********    2.7750    11100
        0.2500    300.00   0.00   0.00   0.00  -1293.8604       0.0000    300.0000
H    74.98776   63.11486   86.00369    -2043.14101    -1757.64893      399.89339          1
N    75.69024   62.95080   85.28496      426.75047      490.45733     -334.42467          1
H    75.11769   62.71686   84.47580      442.25559    -1110.30854    -2448.52907          1
C    76.55752   64.03842   85.04698      415.32972     -666.39213       83.73172          1
H    76.76710   64.50496   86.03344      879.32219     2491.00447      667.35213          1
C    77.86406   63.46844   84.29594       31.35357      477.41777      628.23362          1
H    77.55714   63.04980   83.30707     1168.38489     2240.25337    -1019.71136          1
H    78.58148   64.29476   84.07205     -360.43770     2534.90498      931.67112          1
C    78.51001   62.39876   85.14051      463.18208      512.70242     -382.34388          1
```

12	H	77.71229	61.69925	85.47909	1725.29117	1863.52247	-987.07854	1
13	C	79.52496	61.61222	84.32127	710.42801	-144.27427	-313.15367	1
14	H	79.99472	60.79097	84.92206	364.20195	-1033.13725	2494.72524	1
15	H	79.05334	61.13789	83.42190	2567.61904	335.80679	847.48802	1
16	H	80.35467	62.26921	83.95267	-2587.99029	680.92867	-512.16411	1
17	C	79.19049	62.99296	86.36554	268.17616	741.64379	-40.42634	1
18	H	79.67512	62.20109	86.99361	1283.54314	-1945.37420	-1411.27812	1
19	H	79.99001	63.72422	86.07869	-1258.57796	1317.15249	-2026.02085	1
20	H	78.46241	63.53681	87.02100	-2502.84526	909.80597	575.92391	1
21	C	75.77734	65.01149	83.99498	619.36578	-458.64843	-172.09273	1
22	O	75.42135	64.62963	82.89395	-81.40433	433.87611	522.34594	1
23	N	75.61879	66.21055	84.54110	175.23996	-622.20190	341.55523	1
24	H	76.02900	66.39982	85.45465	-876.57256	-303.22879	-2561.90213	1
25	C	75.20500	67.25915	83.66587	-571.88214	-534.10783	-106.15033	1
26	H	74.55620	66.75515	82.91459	-2713.20841	-16.25800	-248.80809	1
27	C	74.49391	68.40419	84.52780	166.60354	288.16399	-716.09568	1
28	H	75.23390	68.78957	85.28135	-2097.85574	1636.78449	-586.18044	1
29	O	73.33257	67.99992	85.27225	-317.80580	-363.38227	484.43082	1
30	H	73.62250	68.17796	86.18926	1863.74748	-1451.84923	1357.29467	1
31	C	74.24912	69.50509	83.43030	503.80731	517.99032	318.51606	1
32	H	73.86321	70.44921	83.88001	640.61020	-2157.10410	-1536.29104	1
33	H	75.18276	69.76288	82.86728	1687.88455	465.60692	2087.56452	1
34	H	73.49822	69.16855	82.67837	-1293.07895	-1899.88581	-1463.57452	1
35	C	76.58689	67.88446	83.04676	-153.07550	-733.68902	248.70981	1
36	O	77.41336	68.34931	83.78449	186.15459	-480.78787	-449.34387	1
37	N	76.58140	67.81001	81.70443	-487.53139	543.18517	42.03850	1
38	C	77.55351	68.49052	80.91524	-699.35871	69.34393	360.10701	1
39	H	78.40285	68.72500	81.59916	2.81126	-221.99807	-2715.58072	1
40	C	77.81936	67.38166	79.80320	-507.46439	-592.59194	122.07761	1
41	H	78.35533	67.76628	78.90832	-1975.08116	525.87560	-1801.71556	1
42	H	78.43083	66.54485	80.20704	1588.27743	-1913.22100	1113.83566	1
43	C	76.36006	66.92218	79.50044	-613.62442	-457.55993	-194.14490	1
44	H	75.85407	67.63731	78.81301	1707.54637	-721.27992	1996.92523	1
45	H	76.30491	65.92453	79.01029	758.23357	2100.81767	1560.55011	1
46	C	75.69189	66.94477	80.94056	-352.41524	704.96076	49.21465	1
47	H	74.67774	67.40973	80.96873	1714.76886	-2070.42778	443.35792	1
48	H	75.66732	65.95406	81.48994	1224.15040	1801.50881	1636.97633	1
49	C	76.82953	69.82675	80.29064	566.85117	537.05831	117.62814	1
50	O	75.86181	70.32463	80.81787	-550.26779	-406.08867	4.21666	1
51	N	77.49094	70.22177	79.21161	566.34832	-425.77725	180.18749	1
52	H	78.31181	69.69278	78.92544	644.44502	2646.80550	-52.73430	1
53	C	77.13979	71.46235	78.61153	612.34801	-281.48662	411.56221	1
54	H	76.26200	71.80723	79.20818	1667.23498	-1490.75142	1556.16753	1
55	C	78.42877	72.39591	78.78360	-714.45746	-175.43074	286.98927	1
56	H	79.29822	71.93677	78.23850	-787.09484	1763.03619	-1922.46060	1
57	O	78.89319	72.60782	80.13078	-429.46514	516.51932	-128.40526	1
58	H	79.78270	72.20796	80.09234	1981.82785	436.16847	-1818.18182	1
59	C	77.93902	73.68606	78.03459	630.79189	-468.94448	76.03230	1
60	H	78.79335	74.36795	77.79722	-1775.71861	2062.30353	131.89849	1
61	H	77.41812	73.44881	77.07929	-1855.14608	88.94118	1993.53760	1
62	H	77.21512	74.25487	78.66153	-1504.82350	-780.94249	-2132.90992	1
63	C	76.75392	71.14081	77.04837	288.57638	618.62814	-397.00270	1
64	O	75.55515	71.01754	76.79483	321.70495	-185.26452	574.37218	1
65	N	77.79148	71.01807	76.26269	-447.05792	-446.53431	367.76425	1
66	H	78.69015	71.10372	76.73425	350.31316	1975.63644	-1843.31522	1
67	C	77.72591	70.79093	74.86518	-511.23061	374.03917	-471.51480	1
68	H	78.79711	70.59196	74.63648	2234.79579	-898.70890	-1273.45190	1
69	C	76.81121	69.51017	74.60549	529.53781	-159.34528	563.72604	1
70	H	77.10766	69.03623	73.62756	654.59388	2347.94251	1217.51488	1
71	H	77.08689	68.77875	75.41318	-1526.55661	2103.42026	817.87394	1
72	O	75.38612	69.67114	74.55523	-554.84278	14.12789	399.58769	1
73	H	75.24971	70.20786	75.42677	1297.06236	2061.84854	1220.68812	1

C	77.19222	72.10217	74.05257	-662.15484	-412.13544	123.62687	1
O	76.64878	72.00075	72.97437	-540.01537	-317.05804	274.91456	1
N	77.46104	73.25808	74.72489	-202.76299	-690.00100	-131.49145	1
H	78.03173	72.91858	75.50832	-702.62132	-2617.37191	281.70413	1
C	77.94996	74.41571	74.01048	-491.73914	576.34187	222.73509	1
H	79.05704	74.33717	74.05393	1977.17978	-1823.58850	-434.68928	1
C	77.42757	74.46929	72.52303	-649.50797	-328.66843	306.11224	1
H	76.38090	74.08905	72.46653	2075.07803	1452.16490	-1004.45904	1
C	77.53804	75.95893	72.03771	-615.18735	-489.99689	-70.96352	1
H	77.05793	76.10619	71.04150	-1226.32661	-1724.38906	-1716.47170	1
H	77.05004	76.65559	72.76434	2381.64273	-969.84735	900.46860	1
H	78.60082	76.28302	71.94621	2122.74991	1702.23946	-141.36228	1
C	78.36862	73.70482	71.52283	-501.86074	-479.61276	-376.42482	1
H	77.97104	73.74797	70.48185	993.79261	-2246.95401	-1177.81305	1
H	79.39438	74.14089	71.50546	42.71279	-626.15391	-2651.37258	1
H	78.47343	72.62630	71.78854	-2026.39012	-1590.33709	-887.82922	1
C	77.43714	75.70060	74.85473	-672.89153	382.57394	-156.34072	1
O	78.07213	76.74351	74.80576	410.04627	221.86223	500.35950	1
N	76.22197	75.51769	75.41952	-378.11782	-443.79414	-441.10984	1
H	75.96269	74.55131	75.19750	2404.15973	-568.14894	1149.30085	1
C	76.00885	75.92549	76.79411	-544.54484	466.16019	331.29119	1
H	75.72795	74.96297	77.27269	-1409.80988	-170.17955	2325.32679	1
C	77.33223	76.53685	77.40268	-184.48284	116.78282	-758.89137	1
H	78.17951	75.81010	77.37872	1893.78641	1861.14652	611.04528	1
H	77.69536	77.40722	76.80666	-2170.79665	-974.19311	1327.50117	1
C	76.99242	76.97923	78.86250	389.51668	672.81878	-138.49152	1
H	75.97443	77.42497	78.88079	510.64577	1933.03327	1851.02484	1
H	76.90740	76.09798	79.53472	-2302.30869	-1105.72939	-948.89774	1
C	78.02113	78.00802	79.45842	-559.93427	-126.34590	542.30857	1
O	77.82661	78.46544	80.55701	-670.36003	-111.83166	76.36448	1
N	79.04860	78.31320	78.63766	-216.06729	-537.07037	446.51379	1
H	79.30036	77.91914	77.72352	-1251.96487	1074.12894	-2168.52500	1
H	79.81619	78.94365	78.89541	-2451.83023	-720.73851	944.84612	1
C	74.74528	76.92503	76.87851	546.24052	-563.88337	-85.11809	1
O	73.84813	76.64109	77.67178	71.70702	-307.40463	606.69673	1
N	74.87995	78.04717	76.19451	479.90863	-504.50129	222.86849	1
C	73.73320	78.78650	75.71184	75.51268	-600.91904	506.73700	1
H	72.88337	78.56376	76.39144	-2033.61562	1795.30103	254.89480	1
C	74.31266	80.26245	75.90523	523.79067	517.99274	284.45581	1
H	73.71029	81.05655	75.40372	1599.61665	-1127.00911	1895.98173	1
H	74.33624	80.53770	76.98390	-1881.83093	1275.82488	-1501.55016	1
C	75.75227	80.10206	75.33356	-600.05639	-404.05026	316.64485	1
H	75.74152	79.98298	74.22582	-2597.05260	496.36221	657.73249	1
H	76.43264	80.95243	75.56474	-1666.24518	1294.38985	-1723.90591	1
C	76.15247	78.75319	76.06978	493.57570	-129.95376	-602.56394	1
H	76.85239	78.07520	75.48561	-1469.08415	-637.84797	2204.22581	1
H	76.53165	78.88639	77.11141	-1038.97840	1068.88701	-2280.71789	1
C	73.40102	78.31675	74.23039	188.88100	241.05089	-727.87821	1
O	72.35318	79.28659	73.21895	531.88613	-49.19943	427.07960	1
N	74.62636	78.08804	73.55669	708.14833	26.93417	-179.73509	1
H	75.09076	77.46005	74.23439	-2232.69501	-1179.09831	1023.94816	1
C	74.59918	77.59181	72.21120	-738.32412	259.92548	104.42456	1
H	75.45986	76.89238	72.23170	2028.89123	1260.02983	1311.33324	1
C	73.26944	76.80818	71.86053	-258.65678	-345.52513	661.28459	1
H	72.39502	77.21282	72.41356	-1826.06090	-1801.46097	-918.64554	1
C	72.93673	76.79665	70.35757	-541.23400	531.92970	-218.41428	1
H	71.95289	76.31011	70.19378	1311.73218	-2219.18200	882.19023	1
H	72.83286	77.83531	69.97284	-1048.71298	1223.58118	-2196.97971	1
H	75.08663	76.59199	69.55682	419.06414	1903.92898	1903.44670	1
C	73.54034	75.28369	72.22096	-423.86431	152.66566	-648.55262	1
H	72.98056	74.63699	71.49924	105.68386	-2342.50080	1387.51238	1
H	74.61567	75.02924	72.07569	-1695.63603	-1484.73621	-1531.02793	1

136	H	72.31661	75.52879	74.19839	-1853.57121	-487.39031	1936.59343	1
137	C	74.93423	78.89275	71.28029	539.71428	-510.58232	267.58633	1
138	O	74.08152	79.57823	70.76431	-15.93758	-278.34085	-624.49402	1
139	N	76.26108	78.95602	71.11949	-676.81886	12.05292	276.17960	1
140	H	76.82563	78.33063	71.69262	-1694.44250	-373.84189	-2100.66098	1
141	C	76.84876	80.15348	70.57077	412.33078	-664.70360	108.35451	1
142	H	76.39498	80.98186	71.15989	1765.77476	1294.44366	1621.76594	1
143	C	78.40427	80.01114	70.79335	-179.66635	-721.95667	-264.73915	1
144	H	78.63731	79.74926	71.85015	-607.34922	2587.87032	-598.10018	1
145	H	78.79857	79.16615	70.18533	-359.69332	-1479.13954	-2259.74260	1
146	C	79.09949	81.35371	70.38468	-240.01076	616.02425	431.85322	1
147	H	78.72738	81.69194	69.38432	-1359.17131	782.54425	2227.99181	1
148	H	78.84449	82.17028	71.09694	-820.36667	85.81767	-2596.78715	1
149	C	80.66092	81.10949	70.33661	-513.07015	236.18570	-551.87280	1
150	H	81.06707	80.91071	71.35501	1816.11944	-1183.11722	-1650.94321	1
151	H	80.90277	80.20517	69.73218	-1800.04429	-1361.53473	1526.34643	1
152	C	81.30913	82.36633	69.71034	726.08972	-253.19732	-179.64713	1
153	H	81.03053	82.54839	68.65300	1224.27271	-456.51798	-2390.90304	1
154	H	81.18073	83.29578	70.29993	1206.40339	-1150.93514	-2154.90344	1
155	N	82.85698	82.13285	69.67019	329.70796	-633.68968	-155.67358	1
156	H	83.42668	82.91755	69.24009	1348.31305	1938.76845	1359.00643	1
157	H	83.35137	82.00218	70.60031	2096.92035	1446.54551	-966.48890	1
158	H	83.20495	81.29662	69.11671	-1197.07217	-2303.53719	827.28733	1
159	C	76.51349	80.30636	68.99569	-378.01186	-556.17301	-413.96481	1
160	O	76.96249	81.23438	68.35895	-409.44433	-307.55053	-453.30833	1
161	N	75.80589	79.26096	68.51164	-429.02477	524.70884	274.08502	1
162	H	75.45541	78.65876	69.26125	1706.34263	-1425.96036	1574.38926	1
163	C	74.87852	79.62914	67.45647	-45.25864	-626.22952	478.93267	1
164	H	74.54691	80.66803	67.67596	1988.63164	570.90773	1772.87287	1
165	C	75.77882	79.52900	66.17250	551.77128	-20.49526	-564.54996	1
166	H	76.54639	80.33805	66.18742	1434.61535	-1753.10253	1513.99471	1
167	H	76.35533	78.57668	66.18687	-714.67738	1819.09770	1898.36494	1
168	C	74.89463	79.60798	64.88237	446.99263	-555.77305	338.97221	1
169	H	74.08547	78.84252	64.90245	1953.68466	1892.25957	-161.67766	1
170	H	74.39052	80.59564	64.78786	-403.03785	2688.07032	-188.43591	1
171	C	75.90727	79.34608	63.69607	567.86262	335.74681	434.04421	1
172	H	76.70815	80.12079	63.68668	-1080.02275	1568.26081	-1948.78900	1
173	H	76.43189	78.37335	63.83895	-919.54108	2111.34692	1456.13463	1
174	C	75.13223	79.34631	62.35886	195.81897	173.33862	-745.11536	1
175	H	74.39291	78.52751	62.25187	1405.67949	-2301.21089	-390.08113	1
176	H	74.66654	80.31615	62.09328	-940.37328	-1118.32446	2299.72133	1
177	N	76.18975	79.08351	61.23318	319.26422	656.36007	-42.02424	1
178	H	75.81425	79.05860	60.24181	1961.03633	619.91968	1787.09422	1
179	H	76.97884	79.78749	61.14064	854.14343	-2156.80752	1429.08711	1
180	H	76.72124	78.16575	61.27809	1237.02905	-1378.50857	-1998.28500	1
181	C	73.67159	78.53808	67.50837	532.53319	-268.57236	517.55729	1
182	O	72.51686	78.89730	67.53514	266.16714	-290.73804	-558.88002	1
183	N	74.16164	77.29087	67.46419	-226.25352	640.49678	-270.32839	1
184	H	75.17729	77.19603	67.42442	2007.69063	-1035.27229	1523.50231	1
185	C	73.35194	76.26932	67.99540	-540.76542	-573.33303	49.50000	1
186	H	72.33565	76.71688	68.06182	-2374.20950	-1036.76023	843.75774	1
187	C	73.43986	75.00427	67.05589	-77.09082	666.87422	-415.84136	1
188	H	74.48475	74.59434	67.09237	-985.81834	617.19825	2463.91927	1
189	O	73.14713	75.23571	65.65411	293.29899	-391.65602	-477.80926	1
190	H	74.00143	75.02189	65.24092	2528.72714	-681.76262	-751.27122	1
191	C	72.44538	74.01280	67.76778	252.47363	-605.14473	440.05134	1
192	H	72.44209	73.01425	67.27177	-2052.65063	1456.49051	-1043.51830	1
193	H	72.70616	73.84603	68.84113	-2152.98800	-1661.97574	-161.70652	1
194	H	71.39895	74.39619	67.74084	-473.12726	-1584.27339	2165.61725	1
195	C	74.10534	75.97983	69.49897	360.36307	488.46713	505.10056	1
196	O	74.49788	74.83226	69.80204	-200.08817	-528.03552	-385.82850	1
197	N	71.44721	73.26443	70.58358	-597.54095	-226.44775	355.20479	2

198	H	70.52172	73.60837	70.85780	2179.23655	-1300.70981	-991.33835	2
199	C	72.02747	72.74412	71.53329	-687.68921	297.39807	-249.45179	2
200	H	71.47921	72.74543	72.50004	-349.16256	-2467.37713	-1101.72831	2
201	C	73.50421	72.19518	71.32290	672.47345	361.54328	-201.62880	2
202	H	74.21944	73.04236	71.19577	1541.41502	582.18425	2169.97026	2
203	H	73.82815	71.64191	72.23642	-2534.67717	60.25153	997.72295	2
204	C	73.57671	71.29555	70.07843	574.26774	-389.43119	-377.02542	2
205	C	72.92520	70.02605	70.06035	688.82679	-287.33580	-257.96133	2
206	H	72.33997	69.68072	70.93204	331.14182	1470.38163	2269.80013	2
207	C	74.32350	71.74215	68.94674	-383.52382	-675.04808	144.24917	2
208	H	74.82072	72.73196	68.95853	4.09847	1517.59662	2262.86397	2
209	C	73.04968	69.22923	68.92185	-134.76730	428.45285	-649.50303	2
210	H	72.55085	68.24437	68.90849	-903.23433	1600.49020	2011.53387	2
211	C	74.41993	70.90702	67.83045	-335.53281	-615.00040	-364.39058	2
212	H	75.00593	71.25893	66.96363	1596.45398	-1369.33862	1732.02659	2
213	C	73.79719	69.62582	67.77513	585.48547	-340.24194	-406.23938	2
214	O	73.89398	68.76695	66.64875	-419.15649	-173.04408	-511.94141	2
215	H	74.47320	69.31622	66.06991	-1071.84350	1559.71507	-1960.13012	2
216	C	73.12299	74.92834	73.66258	291.41878	-183.13091	710.72246	1
217	O	73.42332	73.98951	74.35957	379.87695	523.47396	-222.23622	1
218	N	70.06111	76.76292	72.17417	564.30591	373.09380	277.24371	1
219	H	70.13522	77.41632	71.40177	-120.59931	-2436.07824	-1214.35098	1
220	C	70.32406	77.28453	73.26567	404.07514	-299.37480	608.84009	1
221	H	72.86333	77.34948	74.18956	-1071.07882	-841.69025	2359.66467	1
222	C	70.72128	78.75831	73.40208	-683.12807	68.71770	-390.13096	1
223	H	70.28261	79.39033	72.60262	2068.88918	1596.58590	770.89603	1
224	H	70.44939	79.18255	74.38802	1379.86429	2342.49779	-179.85638	1
225	C	72.40583	80.88593	73.79076	-176.39967	537.49466	550.97268	1
226	H	72.13329	80.76388	74.86219	-396.28051	1942.22168	1869.33314	1
227	H	73.49043	81.11907	73.73002	-94.44700	586.00678	-2659.20016	1
228	C	71.53921	81.97596	73.09636	-291.69381	-718.18079	-150.72929	1
229	H	71.80965	82.21977	72.03398	-2303.38776	1089.76242	964.62032	1
230	H	70.43316	81.79679	73.19321	884.16729	2568.80533	-207.74283	1
231	N	71.89584	83.03672	73.94329	-594.49522	-252.81478	342.29276	1
232	H	71.94101	82.84417	74.93504	-1735.17426	2084.05705	-263.71605	1
233	C	72.12107	84.23406	73.56673	595.23177	-271.52487	442.22352	1
234	N	72.03228	84.54336	72.20699	585.53813	-424.79750	-105.81303	1
235	H	71.79527	83.93708	71.42268	959.88797	1327.56823	-2177.11905	1
236	H	72.17720	85.46769	71.79953	1578.48647	16.27378	-2220.76233	1
237	N	72.42592	85.04622	74.66688	-452.17763	-390.33541	-421.51853	1
238	H	72.46926	84.81536	75.65864	470.26107	1974.25629	-1817.92087	1
239	H	72.62135	86.04563	74.60355	1856.67307	-1573.97321	-1224.35416	1
240	C	70.29100	76.37286	74.55937	-531.47271	-581.45780	-55.06206	1
241	O	69.83994	75.24643	74.59214	-243.00837	192.73507	609.52485	1
242	N	70.90906	77.02874	75.59150	343.49288	-644.25607	38.09164	1
243	H	71.17122	77.98919	75.33933	-1691.16043	-1535.97525	-1484.73099	1
244	C	70.24492	76.86381	76.87265	542.66492	350.14337	454.42563	1
245	H	69.18490	76.64467	76.61834	1209.57979	-2396.95920	463.86744	1
246	C	70.94700	75.67813	77.64411	-737.14309	145.93010	242.71942	1
247	H	72.05451	75.86458	77.71651	-1050.18303	159.36419	-2509.06125	1
248	C	70.29021	75.64277	79.06815	-706.77696	-252.88343	-245.16230	1
249	H	70.70481	74.81902	79.69423	-1624.64039	-1665.49085	1417.86935	1
250	H	70.46673	76.59779	79.61765	2566.10521	598.51109	693.22305	1
251	H	69.18646	75.49661	79.00812	-1972.69758	107.10018	-1876.34312	1
252	C	70.67286	74.31849	76.91021	412.85148	672.58832	-27.69222	1
253	H	70.93071	73.44443	77.55382	1447.02277	1762.76533	-1490.78944	1
254	H	69.59827	74.21927	76.61957	1320.77379	-145.20455	2378.68491	1
255	H	71.28083	74.23171	75.97475	-1512.10848	1470.52031	1724.75147	1
256	C	70.44558	78.24156	77.70290	-607.11113	499.56689	73.73769	1
257	O	69.47394	78.93752	77.87393	357.97126	460.81534	356.68785	1
258	N	71.70666	78.46432	78.10493	536.55407	-368.62270	332.76306	1
259	H	72.47968	77.77432	77.96629	524.38866	2297.69985	1367.20941	1

```
C    71.80184  79.57998  79.02210       46.50277     -774.29190     -147.97904          1
H    70.95563  79.41530  79.72793     1676.51875     1883.29066     1032.55549          1
C    73.23152  79.65359  79.68889     -559.41456      -34.19198     -556.30433          1
H    73.96795  79.97339  78.91890      -64.66664      395.18769     2695.05373          1
H    73.24404  80.45240  80.46386    -2703.44278      -60.40056      333.79418          1
C    73.72139  78.29999  80.30866     -519.76435      377.31045      459.42366          1
H    73.73863  77.49608  79.52871    -1746.74323     1667.50644    -1261.73691          1
C    75.16871  78.65074  80.81530      570.95554       16.46261     -545.27700          1
H    75.73648  77.74368  81.13511     2391.58273       28.05277    -1305.07271          1
H    75.76244  79.15492  80.01463      175.75588      917.63458     2559.43867          1
H    75.14311  79.34668  81.68572    -1063.97851    -2155.34650     1283.00462          1
C    72.84748  77.86947  81.53985      193.76014        2.62946     -765.53141          1
H    73.25449  76.95277  82.02724      507.39394     -846.55087     2539.60089          1
H    72.81127  78.66960  82.31527     1337.57841     1818.19054     1526.01994          1
H    71.79705  77.64738  81.24012     1100.88635    -1172.91350    -2199.08909          1
C    71.67903  80.97580  78.20883     -549.21115      475.64486     -309.38218          1
O    72.13948  81.15773  77.11041      556.06106     -390.37755      -78.24250          1
O    71.20984  82.06371  78.97705     -572.27940     -310.19032     -209.75862          1
H    70.92027  81.56079  79.79176    -1380.08193     2212.14486      790.85944          1
```

Listing 34: C00034.xmolout

```
       276
  150.0000  150.0000  150.0000     0.0000     0.0000     0.0000***********    1.2297     4919
      0.2500     300.00    0.00    0.00    0.00  -1293.8275       0.0000    300.0000
H    70.75458  82.90491  85.29892    -2043.14101    -1757.64893      399.89339          1
N    71.74711  82.75800  85.47461      426.75047      490.45733     -334.42467          1
H    71.93991  81.90077  84.95087      442.25559    -1110.30854    -2448.52907          1
C    72.57548  83.82556  85.05684      415.32972     -666.39213       83.73172          1
H    71.94553  84.74304  85.02769      879.32219     2491.00447      667.35213          1
C    73.79955  83.95179  86.10874       31.35357      477.41777      628.23362          1
H    74.36913  82.99042  86.13629     1168.38489     2240.25337    -1019.71136          1
H    74.52841  84.72842  85.77534     -360.43770     2534.90498      931.67112          1
C    73.24742  84.27501  87.46873      463.18208      512.70242     -382.34388          1
H    72.32777  83.66593  87.62375     1725.29117     1863.52247     -987.07854          1
C    74.25761  83.90609  88.54899      710.42801     -144.27427     -313.15367          1
H    73.86296  84.11904  89.57561      364.20195    -1033.13725     2494.72524          1
H    74.52169  82.81776  88.51277     2567.61904      335.80679      847.48802          1
H    75.21163  84.48198  88.43370    -2587.99029      680.92867     -512.16411          1
C    72.88818  85.75035  87.58683      268.17616      741.64379      -40.42634          1
H    72.45327  85.99052  88.59117     1283.54314    -1945.37420    -1411.27812          1
H    73.78731  86.40503  87.45126    -1258.57796     1317.15249    -2026.02085          1
H    72.13539  86.05844  86.81665    -2502.84526      909.80597      575.92391          1
C    73.14422  83.36606  83.62263      619.36578     -458.64843     -172.09273          1
O    73.11213  82.20803  83.21919      -81.40433      433.87611      522.34594          1
N    73.75218  84.41932  83.02660      175.23996     -622.20190      341.55523          1
H    73.61816  85.26133  83.60039     -876.57256     -303.22879    -2561.90213          1
C    73.32205  84.61364  81.64148     -571.88214     -534.10783     -106.15033          1
H    72.22287  84.45265  81.69284    -2713.20841      -16.25800     -248.80809          1
C    73.67756  86.12282  81.30269      166.60354      288.16399     -716.09568          1
H    74.79338  86.23543  81.21023    -2097.85574     1636.78449     -586.18044          1
O    73.26238  87.09104  82.29476     -317.80580     -363.38227      484.43082          1
H    74.11382  87.52003  82.50312     1863.74748    -1451.84923     1357.29467          1
C    73.02907  86.28130  79.87826      503.80731      517.99032      318.51606          1
H    73.27555  87.26894  79.42395      640.61020    -2157.10410    -1536.29104          1
H    73.37825  85.49019  79.17143     1687.88455      465.60692     2087.56452          1
H    71.91764  86.21027  79.92948    -1293.07895    -1899.88581    -1463.57452          1
C    74.01552  83.54076  80.65009     -153.07550     -733.68902      248.70981          1
O    73.36498  83.26086  79.67618      186.15459     -480.78787     -449.34387          1
N    75.28430  83.20307  80.92320     -487.53139      543.18517       42.03850          1
C    75.92211  82.12199  80.23298     -699.35871       69.34393      360.10701          1
H    75.31977  81.90528  79.31851        2.81126     -221.99807    -2715.58072          1
```

C	77.38217	82.72690	79.99636	-507.46439	-592.59194	122.07761	1
H	78.13813	81.94229	79.77067	-1975.08116	525.87560	-1801.71556	1
H	77.39721	83.44596	79.14810	1588.27743	-1913.22100	1113.83566	1
C	77.62370	83.44270	81.35927	-613.62442	-457.55993	-194.14490	1
H	77.88672	82.71061	82.15598	1707.54637	-721.27992	1996.92523	1
H	78.44830	84.18983	81.32933	758.23357	2100.81767	1560.55011	1
C	76.19930	84.09258	81.62954	-352.41524	704.96076	49.21465	1
H	75.88959	84.11089	82.70099	1714.76886	-2070.42778	443.35792	1
H	76.05356	85.10479	81.18357	1224.15040	1801.50881	1636.97633	1
C	76.14715	80.84241	81.20387	566.85117	537.05831	117.62814	1
O	76.99099	80.00774	80.90677	-550.26779	-406.08867	4.21666	1
N	75.35214	80.85111	82.25950	566.34832	-425.77725	180.18749	1
H	74.63261	81.59015	82.38883	644.44502	2646.80550	-52.73430	1
C	75.91565	80.20164	83.42170	612.34801	-281.48662	411.56221	1
H	77.00532	80.20312	83.18405	1667.23498	-1490.75142	1556.16753	1
C	75.52320	81.11762	84.66323	-714.45746	-175.43074	286.98927	1
H	74.40170	81.10993	84.74729	-787.09484	1763.03619	-1922.46060	1
O	75.90918	82.50724	84.52008	-429.46514	516.51932	-128.40526	1
H	75.08775	82.88034	84.13561	1981.82785	436.16847	-1818.18182	1
C	76.14087	80.37651	85.90084	630.79189	-468.94448	76.03230	1
H	75.86207	80.87995	86.85621	-1775.71861	2062.30353	131.89849	1
H	75.79111	79.31799	85.96853	-1855.14608	88.94118	1993.53760	1
H	77.25470	80.35886	85.85102	-1504.82350	-780.94249	-2132.90992	1
C	75.32641	78.68724	83.44562	288.57638	618.62814	-397.00270	1
O	74.57460	78.30637	84.33823	321.70495	-185.26452	574.37218	1
N	75.83625	78.03079	82.44091	-447.05792	-446.53431	367.76425	1
H	76.44406	78.54493	81.76216	350.31316	1975.63644	-1843.31522	1
C	75.59627	76.64110	82.22040	-511.23061	374.03917	-471.51480	1
H	76.59283	76.37378	81.80191	2234.79579	-898.70890	-1273.45190	1
C	75.29177	75.90783	83.60708	529.53781	-159.34528	563.72604	1
H	75.62915	74.83896	83.50656	654.59388	2347.94251	1217.51488	1
H	75.95190	76.39642	84.37431	-1526.55661	2103.42026	817.87394	1
O	73.92964	75.88307	84.06746	-554.84278	14.12789	399.58769	1
H	73.88618	76.84485	84.43997	1297.06236	2061.84854	1220.68812	1
C	74.49455	76.47444	81.04896	-662.15484	-412.13544	123.62687	1
O	74.47483	77.20496	80.08988	-540.01537	-317.05804	274.91456	1
N	73.80188	75.31991	81.21257	-202.76299	-690.00100	-131.49145	1
H	73.96953	74.92448	82.14438	-702.62132	-2617.37191	281.70413	1
C	72.43684	75.19014	80.66527	-491.73914	576.34187	222.73509	1
H	71.99393	74.42723	81.34696	1977.17978	-1823.58850	-434.68928	1
C	71.71127	76.51426	80.76923	-649.50797	-328.66843	306.11224	1
H	72.27213	77.26453	80.16106	2075.07803	1452.16490	-1004.45904	1
C	70.29369	76.43486	80.23240	-615.18735	-489.99689	-70.96352	1
H	69.75453	77.41700	80.32995	-1226.32661	-1724.38906	-1716.47170	1
H	70.28086	76.14874	79.13895	2381.64273	-969.84735	900.46860	1
H	69.68602	75.66466	80.78194	2122.74991	1702.23946	-141.36228	1
C	71.67588	77.01721	82.20269	-501.86074	-479.61276	-376.42482	1
H	71.14284	78.00312	82.28870	993.79261	-2246.95401	-1177.81305	1
H	71.14974	76.29167	82.88174	42.71279	-626.15391	-2651.37258	1
H	72.71444	77.15967	82.61620	-2026.39012	-1590.33709	-887.82922	1
C	72.55472	74.59623	79.13252	-672.89153	382.57394	-156.34072	1
O	71.61266	74.68262	78.37775	410.04627	221.86223	500.35950	1
N	73.69672	73.96115	78.92471	-378.11782	-443.79414	-441.10984	1
H	74.40320	74.02013	79.65429	2404.15973	-568.14894	1149.30085	1
C	73.88386	73.27423	77.68239	-544.54484	466.16019	331.29119	1
H	72.90030	72.81428	77.43852	-1409.80988	-170.17955	2325.32679	1
C	74.26354	74.40440	76.63583	-184.48284	116.78282	-758.89137	1
H	73.45169	75.16485	76.57342	1893.78641	1861.14652	611.04528	1
H	75.16936	74.96029	76.96686	-2170.79665	-974.19311	1327.50117	1
C	74.49403	73.71836	75.25152	389.51668	672.81878	-138.49152	1
H	75.32696	72.98104	75.30348	510.64577	1933.03327	1851.02484	1
H	73.58493	73.15498	74.94726	-2302.30869	-1105.72939	-948.89774	1

C	74.81586	74.87135	74.20753	-559.93427	-126.34590	542.30857	1
O	75.92992	75.10561	73.80526	-670.36003	-111.83166	76.36448	1
N	73.67160	75.54458	73.91353	-216.06729	-537.07037	446.51379	1
H	72.74135	75.17834	74.14103	-1251.96487	1074.12894	-2168.52500	1
H	73.53550	76.09808	73.06293	-2451.83023	-720.73851	944.84612	1
C	75.14424	72.25258	77.81528	546.24052	-563.88337	-85.11809	1
O	76.14163	72.60558	78.38700	71.70702	-307.40463	606.69673	1
N	74.91277	71.10766	77.14958	479.90863	-504.50129	222.86849	1
C	75.95521	70.14493	76.98412	75.51268	-600.91904	506.73700	1
H	76.66988	70.30754	77.82377	-2033.61562	1795.30103	254.89480	1
C	75.12832	68.78642	77.00185	523.79067	517.99274	284.45581	1
H	75.68232	67.93781	76.54192	1599.61665	-1127.00911	1895.98173	1
H	74.87307	68.47774	78.03991	-1881.83093	1275.82488	-1501.55016	1
C	73.85246	69.19394	76.20281	-600.05639	-404.05026	316.64485	1
H	74.06281	69.22062	75.10777	-2597.05260	496.36221	657.73249	1
H	72.99308	68.50074	76.34691	-1666.24518	1294.38985	-1723.90591	1
C	73.58207	70.65396	76.76880	493.57570	-129.95376	-602.56394	1
H	73.16556	71.37209	76.02468	-1469.08415	-637.84797	2204.22581	1
H	72.96815	70.68993	77.69963	-1038.97840	1068.88701	-2280.71789	1
C	76.63916	70.31173	75.50420	188.88100	241.05089	-727.87821	1
O	76.00867	70.13188	74.46866	531.88613	-49.19943	427.07960	1
N	77.91155	70.60622	75.64165	708.14833	26.93417	-179.73509	1
H	78.23247	70.68911	76.60295	-2232.69501	-1179.09831	1023.94816	1
C	78.77437	70.87748	74.49475	-738.32412	259.92548	104.42456	1
H	78.09145	71.43997	73.81369	2028.89123	1260.02983	1311.33324	1
C	79.99775	71.65014	74.96047	-258.65678	-345.52513	661.28459	1
H	80.53396	71.01964	75.70489	-1826.06090	-1801.46097	-918.64554	1
C	80.94916	71.92679	73.81108	-541.23400	531.92970	-218.41428	1
H	81.87257	72.46931	74.15476	1311.73218	-2219.18200	882.19023	1
H	81.28612	70.97021	73.31853	-1048.71298	1223.58118	-2196.97971	1
H	80.46638	72.55887	73.01559	419.06414	1903.92898	1903.44670	1
C	79.61681	72.95034	75.64436	-423.86431	152.66566	-648.55262	1
H	80.51910	73.49621	76.03534	105.68386	-2342.50080	1387.51238	1
H	79.08433	73.64606	74.93877	-1695.63603	-1484.73621	-1531.02793	1
H	78.92620	72.77527	76.51509	-1853.57121	-487.39031	1936.59343	1
C	79.18388	69.39624	73.85659	539.71428	-510.58232	267.58633	1
O	80.31412	68.98661	73.90476	-15.93758	-278.34085	-624.49402	1
N	78.11466	68.84968	73.29990	-676.81886	12.05292	276.17960	1
H	77.18405	69.31149	73.41531	-1694.44250	-373.84189	-2100.66098	1
C	78.24373	67.51389	72.82680	412.33078	-664.70360	108.35451	1
H	79.33018	67.26736	72.91212	1765.77476	1294.44366	1621.76594	1
C	77.32265	66.62502	73.76856	-179.66635	-721.95667	-264.73915	1
H	77.55717	66.81710	74.83915	-607.34922	2587.87032	-598.10018	1
H	76.25395	66.90945	73.64472	-359.69332	-1479.13954	-2259.74260	1
C	77.54443	65.12129	73.40746	-240.01076	616.02425	431.85322	1
H	77.34886	64.95234	72.32033	-1359.17131	782.54425	2227.99181	1
H	78.60380	64.82245	73.57496	-820.36667	85.81767	-2596.78715	1
C	76.56539	64.24230	74.28650	-513.07015	236.18570	-551.87280	1
H	76.87358	64.24733	75.35726	1816.11944	-1183.11722	-1650.94321	1
H	75.53100	64.65564	74.26615	-1800.04429	-1361.53473	1526.34643	1
C	76.59177	62.81210	73.69929	726.08972	-253.19732	-179.64713	1
H	76.19645	62.73314	72.66677	1224.27271	-456.51798	-2390.90304	1
H	77.57333	62.30215	73.76684	1206.40339	-1150.93514	-2154.90344	1
N	75.62405	61.92594	74.55383	329.70796	-633.68968	-155.67358	1
H	75.51787	60.92247	74.22667	1348.31305	1938.76845	1359.00643	1
H	75.87123	61.78578	75.57649	2096.92035	1446.54551	-966.48890	1
H	74.60967	62.23312	74.61225	-1197.07217	-2303.53719	827.28733	1
C	77.65540	67.38529	71.30494	-378.01186	-556.17301	-413.96481	1
O	77.73758	66.33015	70.71393	-409.44433	-307.55053	-453.30833	1
N	77.10989	68.51306	70.86873	-429.02477	524.70884	274.08502	1
H	77.09729	69.33301	71.47266	1706.34263	-1425.96036	1574.38926	1
C	76.36204	68.44723	69.65862	-45.25864	-626.22952	478.93267	1

164	H	76.81439	67.61830	69.06555	1988.63164	570.90773	1772.87287	1
165	C	74.87970	68.11369	70.11171	551.77128	-20.49526	-564.54996	1
166	H	74.81318	67.06116	70.46799	1434.61535	-1753.10253	1513.99471	1
167	H	74.58162	68.74855	70.97946	-714.67738	1819.09770	1898.36494	1
168	C	73.90801	68.36315	68.91395	446.99263	-555.77305	338.97221	1
169	H	73.81579	69.46530	68.75070	1953.68466	1892.25957	-161.67766	1
170	H	74.30201	67.93640	67.96383	-403.03785	2688.07032	-188.43591	1
171	C	72.49633	67.74692	69.27360	567.86262	335.74681	434.04421	1
172	H	72.53754	66.63383	69.30489	-1080.02275	1568.26081	-1948.78900	1
173	H	72.18068	68.07280	70.29264	-919.54108	2111.34692	1456.13463	1
174	C	71.48124	68.24532	68.21780	195.81897	173.33862	-745.11536	1
175	H	71.41091	69.35035	68.13442	1405.67949	-2301.21089	-390.08113	1
176	H	71.59567	67.79574	67.21080	-940.37328	-1118.32446	2299.72133	1
177	N	70.05500	67.82711	68.70336	319.26422	656.36007	-42.02424	1
178	H	69.25415	68.11357	68.06947	1961.03633	619.91968	1787.09422	1
179	H	69.86185	66.79130	68.83201	854.14343	-2156.80752	1429.08711	1
180	H	69.73802	68.23118	69.63175	1237.02905	-1378.50857	-1998.28500	1
181	C	76.41010	69.93771	68.95668	532.53319	-268.57236	517.55729	1
182	O	76.30589	70.95519	69.59574	266.16714	-290.73804	-558.88002	1
183	N	76.51966	69.74956	67.64996	-226.25352	640.49678	-270.32839	1
184	H	76.59371	68.78222	67.33674	2007.69063	-1035.27229	1523.50231	1
185	C	76.33864	70.77099	66.67923	-540.76542	-573.33303	49.50000	1
186	H	76.64900	70.22746	65.75871	-2374.20950	-1036.76023	843.75774	1
187	C	74.79750	71.17757	66.61928	-77.09082	666.87422	-415.84136	1
188	H	74.51629	71.75437	67.54819	-985.81834	617.19825	2463.91927	1
189	O	73.81411	70.13016	66.57064	293.29899	-391.65602	-477.80926	1
190	H	73.21076	70.40924	67.30763	2528.72714	-681.76262	-751.27122	1
191	C	74.83959	72.11524	65.35848	252.47363	-605.14473	440.05134	1
192	H	73.88617	72.67831	65.23557	-2052.65063	1456.49051	-1043.51830	1
193	H	75.66507	72.85984	65.41959	-2152.98800	-1661.97574	-161.70652	1
194	H	75.00003	71.52822	64.42476	-473.12726	-1584.27339	2165.61725	1
195	C	77.33837	72.03110	67.01790	360.36307	488.46713	505.10056	1
196	O	78.52599	71.88022	66.86409	-200.08817	-528.03552	-385.82850	1
197	N	76.69266	73.11780	67.42905	-597.54095	-226.44775	355.20479	1
198	H	75.68224	73.09232	67.55692	2179.23655	-1300.70981	-991.33835	1
199	C	77.34888	74.37145	67.27707	-687.68921	297.39807	-249.45179	1
200	H	78.42967	74.10002	67.32023	-349.16256	-2467.37713	-1101.72831	1
201	C	76.91920	75.05178	65.88642	672.47345	361.54328	-201.62880	1
202	H	75.85765	74.83255	65.63296	1541.41502	582.18425	2169.97026	1
203	H	77.00035	76.16059	65.98569	-2534.67717	60.25153	997.72295	1
204	C	77.87639	74.55272	64.79578	574.26774	-389.43119	-377.02542	1
205	C	79.27563	74.75357	64.99772	688.82679	-287.33580	-257.96133	1
206	H	79.64679	75.24819	65.91343	331.14182	1470.38163	2269.80013	1
207	C	77.40640	73.91693	63.60688	-383.52382	-675.04808	144.24917	1
208	H	76.32530	73.76541	63.43631	4.09847	1517.59662	2262.86397	1
209	C	80.16767	74.30589	64.02561	-134.76730	428.45285	-649.50303	1
210	H	81.24912	74.45106	64.19407	-903.23433	1600.49020	2011.53387	1
211	C	78.33991	73.49499	62.65620	-335.53281	-615.00040	-364.39058	1
212	H	77.96589	73.00928	61.73811	1596.45398	-1369.33862	1732.02659	1
213	C	79.74503	73.66681	62.82406	585.48547	-340.24194	-406.23938	1
214	O	80.70191	73.24315	61.86555	-419.15649	-173.04408	-511.94141	1
215	H	80.09974	72.84887	61.19181	-1071.84350	1559.71507	-1960.13012	1
216	C	76.84422	75.40083	68.41504	291.41878	-183.13091	710.72246	1
217	O	76.56900	76.55460	68.19607	379.87695	523.47396	-222.23622	1
218	N	76.99145	74.82245	69.74587	564.30591	373.09380	277.24371	1
219	H	76.79477	73.84350	69.39866	-120.59931	-2436.07824	-1214.35098	1
220	C	76.05232	75.42759	70.67319	404.07514	-299.37480	608.84009	1
221	H	76.35345	74.84800	71.57416	-1071.07882	-841.69025	2359.66467	1
222	C	76.36121	76.90051	70.83517	-683.12807	68.71770	-390.13096	1
223	H	77.39810	77.13625	70.48881	2068.88918	1596.58590	770.89603	1
224	H	75.68330	77.52879	70.20398	1379.86429	2342.49779	-179.85638	1
225	C	76.18949	77.23291	72.37951	-176.39967	537.49466	550.97268	1

```
H    75.23089  76.80649  72.75851     -396.28051     1942.22168     1869.33314          1
H    76.99262  76.72754  72.97195      -94.44700      586.00678    -2659.20016          1
C    76.21038  78.80251  72.62837     -291.69381     -718.18079     -150.72929          1
H    77.19442  79.31533  72.47346    -2303.38776     1089.76242      964.62032          1
H    75.40557  79.39981  72.12720      884.16729     2568.80533     -207.74283          1
N    75.93363  78.73304  74.00854     -594.49522     -252.81478      342.29276          1
H    75.88716  77.80823  74.41427    -1735.17426     2084.05705     -263.71605          1
C    75.74496  79.74059  74.76360      595.23177     -271.52487      442.22352          1
N    75.81674  81.00925  74.18306      585.53813     -424.79750     -105.81303          1
H    76.00860  81.26130  73.21399      959.88797     1327.56823    -2177.11905          1
H    75.68502  81.89194  74.67797     1578.48647       16.27378    -2220.76233          1
N    75.49461  79.32921  76.07849     -452.17763     -390.33541     -421.51853          1
H    75.43956  78.39763  76.48846      470.26107     1974.25629    -1817.92087          1
H    75.30976  79.97336  76.84832     1856.67307    -1573.97321    -1224.35416          1
C    74.61390  75.09579  70.30395     -531.47271     -581.45780      -55.06206          1
O    74.40617  74.04361  69.74679     -243.00837      192.73507      609.52485          1
N    73.64163  75.98476  70.77645      343.49288     -644.25607       38.09164          1
H    73.94111  76.88810  71.16589    -1691.16043    -1535.97525    -1484.73099          1
C    72.26923  75.90991  70.23927      542.66492      350.14337      454.42563          1
H    71.82145  76.86321  70.60449     1209.57979    -2396.95920      463.86744          1
C    72.35446  75.85142  68.73283     -737.14309      145.93010      242.71942          1
H    72.85286  74.89371  68.44714    -1050.18303      159.36419    -2509.06125          1
C    70.97348  75.89457  68.10076     -706.77696     -252.88343     -245.16230          1
H    71.02631  75.82119  66.98082    -1624.64039    -1665.49085     1417.86935          1
H    70.32346  75.05143  68.46127     2566.10521      598.51109      693.22305          1
H    70.43785  76.85167  68.34549    -1972.69758      107.10018    -1876.34312          1
C    73.21160  76.97373  68.17041      412.85148      672.58832      -27.69222          1
H    73.20662  76.98022  67.04733     1447.02277     1762.76533    -1490.78944          1
H    72.84903  77.98103  68.51148     1320.77379     -145.20455     2378.68491          1
H    74.28649  76.87670  68.49560    -1512.10848     1470.52031     1724.75147          1
C    71.58563  74.61240  71.00091     -607.11113      499.56689       73.73769          1
O    71.27652  74.73816  72.16122      357.97126      460.81534      356.68785          1
N    71.39030  73.55333  70.21517      536.55407     -368.62270      332.76306          1
H    71.76020  73.58282  69.26582      524.38866     2297.69985     1367.20941          1
C    71.37873  72.29132  70.88838       46.50277     -774.29190     -147.97904          1
H    71.72538  72.50936  71.92411     1676.51875     1883.29066     1032.55549          1
C    69.88574  71.67340  70.85178     -559.41456      -34.19198     -556.30433          1
H    69.52959  71.63729  69.79488      -64.66664      395.18769     2695.05373          1
H    69.90456  70.61483  71.20576    -2703.44278      -60.40056      333.79418          1
C    68.94747  72.49325  71.69497     -519.76435      377.31045      459.42366          1
H    68.98174  73.54681  71.33516    -1746.74323     1667.50644    -1261.73691          1
C    67.52277  71.97015  71.55481      570.95554       16.46261     -545.27700          1
H    66.79851  72.57403  72.15992     2391.58273       28.05277    -1305.07271          1
H    67.17278  71.99966  70.49093      175.75588      917.63458     2559.43867          1
H    67.43806  70.90814  71.90183    -1063.97851    -2155.34650     1283.00462          1
C    69.35017  72.47855  73.16326      193.76014        2.62946     -765.53141          1
H    68.57649  72.96996  73.80820      507.39394     -846.55087     2539.60089          1
H    69.48789  71.43438  73.54643     1337.57841     1818.19054     1526.01994          1
H    70.31374  73.03140  73.32839     1100.88635    -1172.91350    -2199.08909          1
C    72.29872  71.26400  70.06816     -549.21115      475.64486     -309.38218          1
O    72.41778  71.26848  68.86350      556.06106     -390.37755      -78.24250          1
O    72.82171  70.19717  70.83317     -572.27940     -310.19032     -209.75862          1
H    72.56753  70.52648  71.74292    -1380.08193     2212.14486      790.85944          1
```

Listing 35: C00035.xmolout

```
        276
  150.0000  150.0000  150.0000    0.0000    0.0000    0.0000***********    2.7750    11100
        0.2500    300.00    0.00    0.00    0.00   -1293.8187       0.0000    300.0000
H    72.58912  77.53305  86.68238    -2043.14101    -1757.64893      399.89339          1
N    73.04082  77.64417  85.77786      426.75047      490.45733     -334.42467          1
H    72.25209  77.87636  85.17693      442.25559    -1110.30854    -2448.52907          1
```

C	74.08827	78.59942	85.73578	415.32972	-666.39213	83.73172	1
H	74.47423	78.70371	86.77682	879.32219	2491.00447	667.35213	1
C	75.22677	78.03089	84.71661	31.35357	477.41777	628.23362	1
H	74.76157	77.83165	83.72336	1168.38489	2240.25337	-1019.71136	1
H	76.01081	78.80337	84.54068	-360.43770	2534.90498	931.67112	1
C	75.82504	76.77910	85.27840	463.18208	512.70242	-382.34388	1
H	74.99372	76.11075	85.60049	1725.29117	1863.52247	-987.07854	1
C	76.64842	76.05476	84.21797	710.42801	-144.27427	-313.15367	1
H	77.08858	75.10411	84.61455	364.20195	-1033.13725	2494.72524	1
H	76.02970	75.78657	83.32372	2567.61904	335.80679	847.48802	1
H	77.49927	76.68616	83.85469	-2587.99029	680.92867	-512.16411	1
C	76.71593	77.07312	86.47993	268.17616	741.64379	-40.42634	1
H	77.18093	76.13871	86.88689	1283.54314	-1945.37420	-1411.27812	1
H	77.55202	77.76884	86.21225	-1258.57796	1317.15249	-2026.02085	1
H	76.14364	77.54872	87.31650	-2502.84526	909.80597	575.92391	1
C	73.49025	79.92748	85.13115	619.36578	-458.64843	-172.09273	1
O	72.32934	80.06920	84.49629	-81.40433	433.87611	522.34594	1
N	74.33213	80.95395	85.15794	175.23996	-622.20190	341.55523	1
H	75.04286	80.61945	85.82668	-876.57256	-303.22879	-2561.90213	1
C	73.59920	82.23293	85.02769	-571.88214	-534.10783	-106.15033	1
H	73.28848	82.52218	86.05564	-2713.20841	-16.25800	-248.80809	1
C	74.64927	83.25333	84.37804	166.60354	288.16399	-716.09568	1
H	75.14426	82.72579	83.51579	-2097.85574	1636.78449	-586.18044	1
O	75.71791	83.66014	85.24869	-317.80580	-363.38227	484.43082	1
H	76.49595	83.30461	84.77248	1863.74748	-1451.84923	1357.29467	1
C	73.74740	84.40062	83.79296	503.80731	517.99032	318.51606	1
H	74.34503	85.10548	83.16811	640.61020	-2157.10410	-1536.29104	1
H	72.93447	83.99464	83.14137	1687.88455	465.60692	2087.56452	1
H	73.26555	84.99882	84.59992	-1293.07895	-1899.88581	-1463.57452	1
C	72.43434	81.76130	84.08635	-153.07550	-733.68902	248.70981	1
O	73.16324	81.81904	82.48953	186.15459	-480.78787	-449.34387	1
N	71.34939	82.37389	84.22943	-487.53139	543.18517	42.03850	1
C	70.19026	82.05432	83.40295	-699.35871	69.34393	360.10701	1
H	70.33565	80.99966	83.08624	2.81126	-221.99807	-2715.58072	1
C	68.99407	82.26237	84.40109	-507.46439	-592.59194	122.07761	1
H	68.03407	82.47864	83.87930	-1975.08116	525.87560	-1801.71556	1
H	68.82160	81.34498	85.00800	1588.27743	-1913.22100	1113.83566	1
C	69.47685	83.44293	85.31529	-613.62442	-457.55993	-194.14490	1
H	69.31679	84.43678	84.82261	1707.54637	-721.27992	1996.92523	1
H	68.93730	83.49357	86.28896	758.23357	2100.81767	1560.55011	1
C	71.00441	83.10359	85.48024	-352.41524	704.96076	49.21465	1
H	71.67567	83.98725	85.56034	1714.76886	-2070.42778	443.35792	1
H	71.22930	82.38722	86.30208	1224.15040	1801.50881	1636.97633	1
C	70.05078	83.06402	82.13721	566.85117	537.05831	117.62814	1
O	69.62749	82.65066	81.07756	-550.26779	-406.08867	4.21666	1
N	70.43350	84.35592	82.40473	566.34832	-425.77725	180.18749	1
H	70.60484	84.31628	83.41447	644.44502	2646.80550	-52.73430	1
C	69.52131	85.34193	81.84519	612.34801	-281.48662	411.56221	1
H	68.51371	84.87208	81.85804	1667.23498	-1490.75142	1556.16753	1
C	69.63390	86.62280	82.76836	-714.45746	-175.43074	286.98927	1
H	70.70987	86.94114	82.82972	-787.09484	1763.03619	-1922.46060	1
O	69.21729	86.42169	84.14845	-429.46514	516.51932	-128.40526	1
H	70.00945	86.72950	84.62445	1981.82785	436.16847	-1818.18182	1
C	68.82844	87.69100	81.94439	630.79189	-468.94448	76.03230	1
H	68.84963	88.69049	82.43804	-1775.71861	2062.30353	131.89849	1
H	69.24459	87.82198	80.91573	-1855.14608	88.94118	1993.53760	1
H	67.75835	87.39891	81.83069	-1504.82350	-780.94249	-2132.90992	1
C	70.02751	85.67452	80.34659	288.57638	618.62814	-397.00270	1
O	69.25854	86.03615	79.48743	321.70495	-185.26452	574.37218	1
N	71.39207	85.67538	80.29198	-447.05792	-446.53431	367.76425	1
H	71.76271	85.29440	81.16809	350.31316	1975.63644	-1843.31522	1
C	71.88589	85.02308	79.09412	-511.23061	374.03917	-471.51480	1

H	71.02022	84.45948	78.68231	2234.79579	-898.70890	-1273.45190	1
C	72.30315	86.23777	78.15712	529.53781	-159.34528	563.72604	1
H	72.69449	85.84122	77.18363	654.59388	2347.94251	1217.51488	1
H	71.34700	86.77876	77.91674	-1526.55661	2103.42026	817.87394	1
O	73.24969	87.20598	78.70272	-554.84278	14.12789	399.58769	1
H	72.78997	87.41389	79.53652	1297.06236	2061.84854	1220.68812	1
C	73.07500	84.06511	79.63311	-662.15484	-412.13544	123.62687	1
O	74.07797	84.46259	80.17323	-540.01537	-317.05804	274.91456	1
N	72.68927	82.79202	79.44847	-202.76299	-690.00100	-131.49145	1
H	71.81847	82.60534	78.94892	-702.62132	-2617.37191	281.70413	1
C	73.68871	81.74731	79.65139	-491.73914	576.34187	222.73509	1
H	74.43700	82.21057	80.34135	1977.17978	-1823.58850	-434.68928	1
C	72.99002	80.50583	80.21543	-649.50797	-328.66843	306.11224	1
H	72.29180	80.10916	79.43907	2075.07803	1452.16490	-1004.45904	1
C	74.03272	79.42885	80.51995	-615.18735	-489.99689	-70.96352	1
H	73.55203	78.47447	80.86355	-1226.32661	-1724.38906	-1716.47170	1
H	74.66179	79.17793	79.62354	2381.64273	-969.84735	900.46860	1
H	74.73638	79.75331	81.33154	2122.74991	1702.23946	-141.36228	1
C	72.23683	80.80555	81.39100	-501.86074	-479.61276	-376.42482	1
H	71.91943	79.89821	81.93389	993.79261	-2246.95401	-1177.81305	1
H	73.78260	81.07786	82.71467	42.71279	-626.15391	-2651.37258	1
H	71.33942	81.42473	81.18097	-2026.39012	-1590.33709	-887.82922	1
C	74.28157	81.41896	78.13340	-672.89153	382.57394	-156.34072	1
O	73.58204	81.39941	77.14699	410.04627	221.86223	500.35950	1
N	75.53774	81.02197	78.26144	-378.11782	-443.79414	-441.10984	1
H	75.94869	81.03809	79.19291	2404.15973	-568.14894	1149.30085	1
C	76.18973	80.43868	77.14037	-544.54484	466.16019	331.29119	1
H	75.54235	80.69413	76.26940	-1409.80988	-170.17955	2325.32679	1
C	77.63181	81.10324	77.16853	-184.48284	116.78282	-758.89137	1
H	77.53271	82.20616	77.28800	1893.78641	1861.14652	611.04528	1
H	78.19212	80.74882	78.06394	-2170.79665	-974.19311	1327.50117	1
C	78.42573	80.76597	75.87089	389.51668	672.81878	-138.49152	1
H	78.59252	79.66762	75.80054	510.64577	1933.03327	1851.02484	1
H	77.82119	81.01163	74.97026	-2302.30869	-1105.72939	-948.89774	1
C	79.80712	81.52355	75.80025	-559.93427	-126.34590	542.30857	1
O	80.50769	81.37765	74.83249	-670.36003	-111.83166	76.36448	1
N	80.07760	82.30031	76.87127	-216.06729	-537.07037	446.51379	1
H	79.53409	82.46023	77.72753	-1251.96487	1074.12894	-2168.52500	1
H	80.93751	82.85057	76.97524	-2451.83023	-720.73851	944.84612	1
C	76.35552	78.82886	77.40235	546.24052	-563.88337	-85.11809	1
O	77.38354	78.46358	77.90997	71.70702	-307.40463	606.69673	1
N	75.26262	78.10915	77.08182	479.90863	-504.50129	222.86849	1
C	75.32543	76.68641	77.17517	75.51268	-600.91904	506.73700	1
H	76.24910	76.49437	77.77002	-2033.61562	1795.30103	254.89480	1
C	73.95901	76.33801	77.90262	523.79067	517.99274	284.45581	1
H	73.60183	75.31172	77.66267	1599.61665	-1127.00911	1895.98173	1
H	74.09056	76.36817	79.00704	-1881.83093	1275.82488	-1501.55016	1
C	72.95271	77.44485	77.45116	-600.05639	-404.05026	316.64485	1
H	72.38428	77.16336	76.53514	-2597.05260	496.36221	657.73249	1
H	72.17786	77.66377	78.22030	-1666.24518	1294.38985	-1723.90591	1
C	73.92254	78.67600	77.20512	493.57570	-129.95376	-602.56394	1
H	73.70705	79.28272	76.29192	-1469.08415	-637.84797	2204.22581	1
H	73.98569	79.35602	78.08266	-1038.97840	1068.88701	-2280.71789	1
C	75.39926	75.94342	75.71090	188.88100	241.05089	-727.87821	1
O	76.40153	75.30050	75.41689	531.88613	-49.19943	427.07960	1
N	74.30199	76.11328	75.01552	708.14833	26.93417	-179.73509	1
H	73.63359	76.72384	75.48067	-2232.69501	-1179.09831	1023.94816	1
C	74.00287	75.51316	73.73222	-738.32412	259.92548	104.42456	1
H	73.02434	75.97586	73.46033	2028.89123	1260.02983	1311.33324	1
C	75.12863	75.87675	72.77048	-258.65678	-345.52513	661.28459	1
H	76.06645	75.41425	73.15665	-1826.06090	-1801.46097	-918.64554	1
C	74.85671	75.35453	71.37292	-541.23400	531.92970	-218.41428	1

H	75.64021	75.69643	70.63883	1311.73218	-2219.18200	882.19023	1
H	74.84327	74.23174	71.33608	-1048.71298	1223.58118	-2196.97971	1
H	73.86239	75.70908	70.98472	419.06414	1903.92898	1903.44670	1
C	75.36041	77.37631	72.72832	-423.86431	152.66566	-648.55262	1
H	76.23388	77.64582	72.07287	105.68386	-2342.50080	1387.51238	1
H	74.46202	77.92349	72.33069	-1695.63603	-1484.73621	-1531.02793	1
H	75.57147	77.78554	73.75532	-1853.57121	-487.39031	1936.59343	1
C	73.84729	73.85721	73.93634	539.71428	-510.58232	267.58633	1
O	72.77968	73.36444	73.69301	-15.93758	-278.34085	-624.49402	1
N	74.98397	73.28900	74.28966	-676.81886	12.05292	276.17960	1
H	75.80182	73.89712	74.52265	-1694.44250	-373.84189	-2100.66098	1
C	75.03591	71.89751	74.58501	412.33078	-664.70360	108.35451	1
H	76.07505	71.76086	74.96068	1765.77476	1294.44366	1621.76594	1
C	73.95348	71.61593	75.70727	-179.66635	-721.95667	-264.73915	1
H	74.03631	72.36639	76.52535	-607.34922	2587.87032	-598.10018	1
H	72.92490	71.73757	75.29221	-359.69332	-1479.13954	-2259.74260	1
C	74.19236	70.16783	76.24708	-240.01076	616.02425	431.85322	1
H	73.99876	69.42824	75.43609	-1359.17131	782.54425	2227.99181	1
H	75.25835	70.02643	76.53497	-820.36667	85.81767	-2596.78715	1
C	73.25205	69.89093	77.48818	-513.07015	236.18570	-551.87280	1
H	73.52937	70.53949	78.35074	1816.11944	-1183.11722	-1650.94321	1
H	72.19175	70.13742	77.25065	-1800.04429	-1361.53473	1526.34643	1
C	73.40218	68.39354	77.84649	726.08972	-253.19732	-179.64713	1
H	72.94210	67.69726	77.11806	1224.27271	-456.51798	-2390.90304	1
H	74.43939	68.07326	78.06984	1206.40339	-1150.93514	-2154.90344	1
N	72.61641	68.13287	79.17525	329.70796	-633.68968	-155.67358	1
H	72.60772	67.12614	79.50982	1348.31305	1938.76845	1359.00643	1
H	72.95647	68.63666	80.04541	2096.92035	1446.54551	-966.48890	1
H	71.58003	68.36208	79.17868	-1197.07217	-2303.53719	827.28733	1
C	74.73711	70.98291	73.26089	-378.01186	-556.17301	-413.96481	1
O	74.52191	69.77356	73.34928	-409.44433	-307.55053	-453.30833	1
N	74.89920	71.70504	72.17033	-429.02477	524.70884	274.08502	1
H	75.05147	72.68137	72.41070	1706.34263	-1425.96036	1574.38926	1
C	74.45289	71.26673	70.89399	-45.25864	-626.22952	478.93267	1
H	74.47729	70.15452	70.95545	1988.63164	570.90773	1772.87287	1
C	72.97426	71.81964	70.75093	551.77128	-20.49526	-564.54996	1
H	72.38795	71.61143	71.67498	1434.61535	-1753.10253	1513.99471	1
H	72.98020	72.92852	70.65384	-714.67738	1819.09770	1898.36494	1
C	72.31383	71.15712	69.49838	446.99263	-555.77305	338.97221	1
H	72.93781	71.34546	68.59034	1953.68466	1892.25957	-161.67766	1
H	72.26007	70.05100	69.61282	-403.03785	2688.07032	-188.43591	1
C	70.86818	71.77355	69.31480	567.86262	335.74681	434.04421	1
H	70.21645	71.52928	70.18504	-1080.02275	1568.26081	-1948.78900	1
H	70.91401	72.88611	69.27432	-919.54108	2111.34692	1456.13463	1
C	70.27985	71.20443	68.00309	195.81897	173.33862	-745.11536	1
H	70.83041	71.49714	67.08676	1405.67949	-2301.21089	-390.08113	1
H	70.11149	70.10905	68.00867	-940.37328	-1118.32446	2299.72133	1
N	68.85390	71.82603	67.81733	319.26422	656.36007	-42.02424	1
H	68.33524	71.53084	66.94032	1961.03633	619.91968	1787.09422	1
H	68.13232	71.61544	68.56675	854.14343	-2156.80752	1429.08711	1
H	68.79419	72.88346	67.74680	1237.02905	-1378.50857	-1998.28500	1
C	75.37533	71.91059	69.70722	532.53319	-268.57236	517.55729	1
O	75.05057	71.76086	68.55122	266.16714	-290.73804	-558.88002	1
N	76.39809	72.62965	70.18911	-226.25352	640.49678	-270.32839	1
H	76.51372	72.51489	71.19555	2007.69063	-1035.27229	1523.50231	1
C	77.56372	72.80608	69.37900	-540.76542	-573.33303	49.50000	1
H	77.20665	73.13312	68.37798	-2374.20950	-1036.76023	843.75774	1
C	78.44927	73.87613	70.15879	-77.09082	666.87422	-415.84136	1
H	78.69169	73.46453	71.17754	-985.81834	617.19825	2463.91927	1
O	77.83001	75.15773	70.41140	293.29899	-391.65602	-477.80926	1
H	77.84487	75.18335	71.38830	2528.72714	-681.76262	-751.27122	1
C	79.76137	73.92506	69.29437	252.47363	-605.14473	440.05134	1

192	H	80.51971	74.60617	69.74491	-2052.65063	1456.49051	-1043.51830	1
193	H	80.23251	72.91854	69.20430	-2152.98800	-1661.97574	-161.70652	1
194	H	79.55798	74.29311	68.26173	-473.12726	-1584.27339	2165.61725	1
195	C	78.32820	71.35951	69.34174	360.36307	488.46713	505.10056	1
196	O	78.25179	70.58278	70.27188	-200.08817	-528.03552	-385.82850	1
197	N	79.06229	71.30585	68.24807	-597.54095	-226.44775	355.20479	1
198	H	78.94651	72.06194	67.57579	2179.23655	-1300.70981	-991.33835	1
199	C	79.88977	70.19866	67.89046	-687.68921	297.39807	-249.45179	1
200	H	79.43279	69.84092	66.94275	-349.16256	-2467.37713	-1101.72831	1
201	C	81.36769	70.75368	67.67389	672.47345	361.54328	-201.62880	1
202	H	81.74885	71.18835	68.62630	1541.41502	582.18425	2169.97026	1
203	H	82.04025	69.90048	67.42334	-2534.67717	60.25153	997.72295	1
204	C	81.40483	71.80484	66.55222	574.26774	-389.43119	-377.02542	1
205	C	81.16875	71.41083	65.20141	688.82679	-287.33580	-257.96133	1
206	H	80.94802	70.35730	64.95099	331.14182	1470.38163	2269.80013	1
207	C	81.69814	73.16567	66.86608	-383.52382	-675.04808	144.24917	1
208	H	81.89539	73.47327	67.90852	4.09847	1517.59662	2262.86397	1
209	C	81.22991	72.38286	64.20248	-134.76730	428.45285	-649.50303	1
210	H	81.05375	72.07794	63.15617	-903.23433	1600.49020	2011.53387	1
211	C	81.74368	74.10353	65.83149	-335.53281	-615.00040	-364.39058	1
212	H	81.97102	75.15303	66.08784	1596.45398	-1369.33862	1732.02659	1
213	C	81.51485	73.75337	64.46903	585.48547	-340.24194	-406.23938	1
214	O	81.56168	74.69766	63.41023	-419.15649	-173.04408	-511.94141	1
215	H	81.79974	75.50780	63.91942	-1071.84350	1559.71507	-1960.13012	1
216	C	80.00606	69.06530	69.05710	291.41878	-183.13091	710.72246	1
217	O	80.86473	69.12214	69.90852	379.87695	523.47396	-222.23622	1
218	N	79.18059	68.05261	68.76688	564.30591	373.09380	277.24371	1
219	H	78.49791	68.18351	68.02000	-120.59931	-2436.07824	-1214.35098	1
220	C	78.96218	67.08653	69.80278	404.07514	-299.37480	608.84009	1
221	H	79.90738	67.16352	70.39091	-1071.07882	-841.69025	2359.66467	1
222	C	77.71143	67.62035	70.61438	-683.12807	68.71770	-390.13096	1
223	H	77.84223	68.72798	70.80057	2068.88918	1596.58590	770.89603	1
224	H	76.77591	67.53023	70.01646	1379.86429	2342.49779	-179.85638	1
225	C	77.60792	66.82020	71.94904	-176.39967	537.49466	550.97268	1
226	H	77.56589	65.72542	71.75286	-396.28051	1942.22168	1869.33314	1
227	H	78.51273	66.97515	72.57787	-94.44700	586.00678	-2659.20016	1
228	C	76.28921	67.31296	72.70421	-291.69381	-718.18079	-150.72929	1
229	H	76.30432	68.36763	73.08887	-2303.38776	1089.76242	964.62032	1
230	H	75.32953	67.17454	72.14341	884.16729	2568.80533	-207.74283	1
231	N	76.25936	66.44118	73.80288	-594.49522	-252.81478	342.29276	1
232	H	77.01494	65.77907	73.88532	-1735.17426	2084.05705	-263.71605	1
233	C	75.33546	66.41907	74.67860	595.23177	-271.52487	442.22352	1
234	N	74.30328	67.34782	74.54090	585.53813	-424.79750	-105.81303	1
235	H	74.25292	68.13637	73.85131	959.88797	1327.56823	-2177.11905	1
236	H	73.51268	67.41115	75.18957	1578.48647	16.27378	-2220.76233	1
237	N	75.55391	65.42144	75.64244	-452.17763	-390.33541	-421.51853	1
238	H	76.30248	64.73674	75.73376	470.26107	1974.25629	-1817.92087	1
239	H	74.91743	65.24208	76.41949	1856.67307	-1573.97321	-1224.35416	1
240	C	78.79495	65.65582	69.03959	-531.47271	-581.45780	-55.06206	1
241	O	79.73331	65.18338	68.44280	-243.00837	192.73507	609.52485	1
242	N	77.58730	65.11035	69.19843	343.49288	-644.25607	38.09164	1
243	H	76.89088	65.63206	69.72813	-1691.16043	-1535.97525	-1484.73099	1
244	C	77.21215	63.94751	68.38717	542.66492	350.14337	454.42563	1
245	H	77.86743	64.04534	67.48832	1209.57979	-2396.95920	463.86744	1
246	C	77.48454	62.69203	69.19652	-737.14309	145.93010	242.71942	1
247	H	76.99378	62.81950	70.18746	-1050.18303	159.36419	-2509.06125	1
248	C	76.88735	61.46891	68.52676	-706.77696	-252.88343	-245.16230	1
249	H	77.04498	60.53705	69.13739	-1624.64039	-1665.49085	1417.86935	1
250	H	75.77537	61.59574	68.37868	2566.10521	598.51109	693.22305	1
251	H	77.34207	61.28654	67.51339	-1972.69758	107.10018	-1876.34312	1
252	C	78.96949	62.49253	69.43482	412.85148	672.58832	-27.69222	1
253	H	79.17375	61.64115	70.14140	1447.02277	1762.76533	-1490.78944	1

```
H    79.51525  62.27218  68.47577     1320.77379     -145.20455     2378.68491           1
H    79.44179  63.42040  69.86837    -1512.10848     1470.52031     1724.75147           1
C    75.58829  64.12575  68.08896     -607.11113      499.56689       73.73769           1
O    74.76523  63.60333  68.80009      357.97126      460.81534      356.68785           1
N    75.40398  64.94638  67.06046      536.55407     -368.62270      332.76306           1
H    76.23509  65.27043  66.56639      524.38866     2297.69985     1367.20941           1
C    74.12851  64.95217  66.44268       46.50277     -774.29190     -147.97904           1
H    73.44556  64.47433  67.18182     1676.51875     1883.29066     1032.55549           1
C    73.73969  66.46962  66.05127     -559.41456      -34.19198     -556.30433           1
H    74.43220  66.83009  65.25360      -64.66664      395.18769     2695.05373           1
H    72.71701  66.50742  65.60569    -2703.44278      -60.40056      333.79418           1
C    73.83691  67.35508  67.26502     -519.76435      377.31045      459.42366           1
H    74.84016  67.20167  67.72396    -1746.74323     1667.50644    -1261.73691           1
C    73.70349  68.82124  66.87277      570.95554       16.46261     -545.27700           1
H    73.85989  69.50142  67.74963     2391.58273       28.05277    -1305.07271           1
H    74.45440  69.11228  66.09379      175.75588      917.63458     2559.43867           1
H    72.68859  69.04643  66.45442    -1063.97851    -2155.34650     1283.00462           1
C    72.77433  67.00449  68.29775      193.76014        2.62946     -765.53141           1
H    72.82325  67.68302  69.18820      507.39394     -846.55087     2539.60089           1
H    71.74103  67.08881  67.87226     1337.57841     1818.19054     1526.01994           1
H    72.89614  65.95548  68.67381     1100.88635    -1172.91350    -2199.08909           1
C    74.27181  64.13126  65.05731     -549.21115      475.64486     -309.38218           1
O    75.31483  63.98725  64.47574      556.06106     -390.37755      -78.24250           1
O    73.05756  63.69382  64.47822     -572.27940     -310.19032     -209.75862           1
H    72.43158  63.97481  65.20190    -1380.08193     2212.14486      790.85944           1
```

Listing 36: C00036.xmolout

```
       276
  150.0000  150.0000  150.0000     0.0000     0.0000     0.0000***********    0.8737     3495
       0.2500    300.00   0.00   0.00   0.00  -1293.8160       0.0000    300.0000
H    74.95760  72.39181  92.82325    -2043.14101    -1757.64893      399.89339           1
N    75.71557  72.13772  93.45260      426.75047      490.45733     -334.42467           1
H    75.72624  71.12585  93.34543      442.25559    -1110.30854    -2448.52907           1
C    76.95423  72.76685  93.15826      415.32972     -666.39213       83.73172           1
H    76.61511  73.81502  92.99580      879.32219     2491.00447      667.35213           1
C    77.95357  72.63705  94.42693       31.35357      477.41777      628.23362           1
H    78.33618  71.59138  94.49355     1168.38489     2240.25337    -1019.71136           1
H    78.85176  73.28165  94.27952     -360.43770     2534.90498      931.67112           1
C    77.23211  73.00836  95.68940      463.18208      512.70242     -382.34388           1
H    76.32567  72.36600  95.77142     1725.29117     1863.52247     -987.07854           1
C    78.12077  72.76144  96.90373      710.42801     -144.27427     -313.15367           1
H    77.58699  72.99415  97.86066      364.20195    -1033.13725     2494.72524           1
H    78.45779  71.69490  96.96172     2567.61904      335.80679      847.48802           1
H    79.04146  73.39881  96.87579    -2587.99029      680.92867     -512.16411           1
C    76.79024  74.46665  95.68233      268.17616      741.64379      -40.42634           1
H    76.28284  74.74841  96.64056     1283.54314    -1945.37420    -1411.27812           1
H    77.65868  75.16149  95.54976    -1258.57796     1317.15249    -2026.02085           1
H    76.06663  74.67424  94.85306    -2502.84526      909.80597      575.92391           1
C    77.47577  72.10164  91.81527      619.36578     -458.64843     -172.09273           1
O    76.69949  71.75871  90.85235      -81.40433      433.87611      522.34594           1
N    78.77126  71.97010  91.76296      175.23996     -622.20190      341.55523           1
H    79.35666  72.31555  92.51935     -876.57256     -303.22879    -2561.90213           1
C    79.25519  71.76865  90.42552     -571.88214     -534.10783     -106.15033           1
H    79.44631  72.80048  90.06531    -2713.20841      -16.25800     -248.80809           1
C    80.57506  70.92166  90.50454      166.60354      288.16399     -716.09568           1
H    80.33515  69.91600  90.94650    -2097.85574     1636.78449     -586.18044           1
O    81.63503  71.47129  91.32933     -317.80580     -363.38227      484.43082           1
H    81.75781  70.76127  91.98445     1863.74748    -1451.84923     1357.29467           1
C    80.90886  70.76064  88.97193      503.80731      517.99032      318.51606           1
H    81.77329  70.07555  88.81194      640.61020    -2157.10410    -1536.29104           1
H    80.04582  70.34487  88.39778     1687.88455      465.60692     2087.56452           1
```

H	81.17403	71.73761	88.50594	-1293.07895	-1899.88581	-1463.57452	1
C	78.00295	70.97716	89.59757	-153.07550	-733.68902	248.70981	1
O	77.88965	69.74923	89.69655	186.15459	-480.78787	-449.34387	1
N	77.85843	71.65667	88.39792	-487.53139	543.18517	42.03850	1
C	77.00531	71.16800	87.39056	-699.35871	69.34393	360.10701	1
H	76.25537	70.49734	87.87253	2.81126	-221.99807	-2715.58072	1
C	76.45250	72.51226	86.71737	-507.46439	-592.59194	122.07761	1
H	76.07170	72.35376	85.68390	-1975.08116	525.87560	-1801.71556	1
H	75.61669	72.95204	87.30477	1588.27743	-1913.22100	1113.83566	1
C	77.72484	73.40161	86.77959	-613.62442	-457.55993	-194.14490	1
H	78.46409	73.09727	86.00239	1707.54637	-721.27992	1996.92523	1
H	77.52572	74.48616	86.63374	758.23357	2100.81767	1560.55011	1
C	78.24838	73.04032	88.24955	-352.41524	704.96076	49.21465	1
H	79.35476	73.11723	88.39481	1714.76886	-2070.42778	443.35792	1
H	77.73136	73.58692	89.07676	1224.15040	1801.50881	1636.97633	1
C	77.90912	70.40431	86.22834	566.85117	537.05831	117.62814	1
O	78.93082	70.86931	85.78828	-550.26779	-406.08867	4.21666	1
N	77.23352	69.33606	85.83852	566.34832	-425.77725	180.18749	1
H	76.35418	69.10438	86.29855	644.44502	2646.80550	-52.73430	1
C	77.74257	68.54916	84.77777	612.34801	-281.48662	411.56221	1
H	78.67361	69.05211	84.42428	1667.23498	-1490.75142	1556.16753	1
C	77.98045	67.08860	85.40887	-714.45746	-175.43074	286.98927	1
H	77.02992	66.77983	85.92404	-787.09484	1763.03619	-1922.46060	1
O	79.01464	67.00687	86.40313	-429.46514	516.51932	-128.40526	1
H	78.48919	66.83350	87.20998	1981.82785	436.16847	-1818.18182	1
C	78.19242	66.19079	84.13739	630.79189	-468.94448	76.03230	1
H	78.29748	65.11516	84.41034	-1775.71861	2062.30353	131.89849	1
H	77.33584	66.26967	83.42959	-1855.14608	88.94118	1993.53760	1
H	79.11260	66.48226	83.58008	-1504.82350	-780.94249	-2132.90992	1
C	76.53329	68.43241	83.68097	288.57638	618.62814	-397.00270	1
O	75.35783	68.26375	84.04640	321.70495	-185.26452	574.37218	1
N	77.03106	68.47015	82.47528	-447.05792	-446.53431	367.76425	1
H	78.04379	68.58547	82.50613	350.31316	1975.63644	-1843.31522	1
C	76.36417	68.20928	81.25235	-511.23061	374.03917	-471.51480	1
H	77.22151	68.00895	80.57048	2234.79579	-898.70890	-1273.45190	1
C	75.39928	66.93777	81.40547	529.53781	-159.34528	563.72604	1
H	75.18548	66.51822	80.38208	654.59388	2347.94251	1217.51488	1
H	75.99788	66.15905	81.94663	-1526.55661	2103.42026	817.87394	1
O	74.12628	67.15410	82.01921	-554.84278	14.12789	399.58769	1
H	74.44601	67.58028	82.90721	1297.06236	2061.84854	1220.68812	1
C	75.46381	69.43576	80.70313	-662.15484	-412.13544	123.62687	1
O	75.02971	69.37108	79.55921	-540.01537	-317.05804	274.91456	1
N	75.35421	70.49260	81.54470	-202.76299	-690.00100	-131.49145	1
H	75.74977	70.12487	82.41590	-702.62132	-2617.37191	281.70413	1
C	74.10030	71.29743	81.50244	-491.73914	576.34187	222.73509	1
H	73.34881	70.53127	81.20120	1977.17978	-1823.58850	-434.68928	1
C	73.86391	71.87328	82.87276	-649.50797	-328.66843	306.11224	1
H	74.80428	72.37295	83.19846	2075.07803	1452.16490	-1004.45904	1
C	72.75001	72.90820	82.88116	-615.18735	-489.99689	-70.96352	1
H	72.55914	73.30701	83.91472	-1226.32661	-1724.38906	-1716.47170	1
H	72.99890	73.79185	82.22464	2381.64273	-969.84735	900.46860	1
H	71.78141	72.47738	82.50711	2122.74991	1702.23946	-141.36228	1
C	73.52163	70.80574	83.89869	-501.86074	-479.61276	-376.42482	1
H	73.45856	71.23424	84.93649	993.79261	-2246.95401	-1177.81305	1
H	72.52668	70.33075	83.67799	42.71279	-626.15391	-2651.37258	1
H	74.28751	69.96656	83.92606	-2026.39012	-1590.33709	-887.82922	1
C	74.31028	72.43207	80.33858	-672.89153	382.57394	-156.34072	1
O	74.38616	73.60575	80.60660	410.04627	221.86223	500.35950	1
N	74.28566	71.88077	79.12679	-378.11782	-443.79414	-441.10984	1
H	74.31729	70.83546	79.01640	2404.15973	-568.14894	1149.30085	1
C	74.78319	72.71995	78.07037	-544.54484	466.16019	331.29119	1
H	74.33743	73.71384	78.30542	-1409.80988	-170.17955	2325.32679	1

C	76.36169	72.71655	78.20929	-184.48284	116.78282	-758.89137	1
H	76.66052	73.09383	79.21412	1893.78641	1861.14652	611.04528	1
H	76.75332	71.67563	78.14472	-2170.79665	-974.19311	1327.50117	1
C	76.95542	73.60631	77.07313	389.51668	672.81878	-138.49152	1
H	76.66014	73.19801	76.07828	510.64577	1933.03327	1851.02484	1
H	76.49561	74.61928	77.09719	-2302.30869	-1105.72939	-948.89774	1
C	78.52383	73.73977	77.14176	-559.93427	-126.34590	542.30857	1
O	79.10163	74.37952	76.30114	-670.36003	-111.83166	76.36448	1
N	79.08965	73.10368	78.19016	-216.06729	-537.07037	446.51379	1
H	78.65595	72.54688	78.93575	-1251.96487	1074.12894	-2168.52500	1
H	80.09801	73.10065	78.37951	-2451.83023	-720.73851	944.84612	1
C	74.31858	72.04653	76.66059	546.24052	-563.88337	-85.11809	1
O	75.14574	71.48827	75.98926	71.70702	-307.40463	606.69673	1
N	73.00745	72.21017	76.41630	479.90863	-504.50129	222.86849	1
C	72.34568	71.41793	75.42899	75.51268	-600.91904	506.73700	1
H	72.96383	70.50014	75.29213	-2033.61562	1795.30103	254.89480	1
C	70.92332	71.19705	76.10620	523.79067	517.99274	284.45581	1
H	70.13829	70.90615	75.37275	1599.61665	-1127.00911	1895.98173	1
H	70.95830	70.39337	76.87542	-1881.83093	1275.82488	-1501.55016	1
C	70.67612	72.59225	76.75814	-600.05639	-404.05026	316.64485	1
H	70.35683	73.33961	75.99871	-2597.05260	496.36221	657.73249	1
H	69.88822	72.58197	77.54418	-1666.24518	1294.38985	-1723.90591	1
C	72.12133	72.92603	77.32683	493.57570	-129.95376	-602.56394	1
H	72.40048	74.00555	77.30643	-1469.08415	-637.84797	2204.22581	1
H	72.33022	72.51261	78.34164	-1038.97840	1068.88701	-2280.71789	1
C	72.09611	72.21551	74.02162	188.88100	241.05089	-727.87821	1
O	71.43759	71.68868	73.15633	531.88613	-49.19943	427.07960	1
N	72.62391	73.44038	73.99915	708.14833	26.93417	-179.73509	1
H	73.17903	73.67768	74.82030	-2232.69501	-1179.09831	1023.94816	1
C	72.64210	74.21276	72.76226	-738.32412	259.92548	104.42456	1
H	72.27157	73.50575	71.97970	2028.89123	1260.02983	1311.33324	1
C	71.75593	75.43585	72.95389	-258.65678	-345.52513	661.28459	1
H	72.11007	75.96870	73.86414	-1826.06090	-1801.46097	-918.64554	1
C	71.85405	76.39025	71.77905	-541.23400	531.92970	-218.41428	1
H	71.23070	77.31394	71.93648	1311.73218	-2219.18200	882.19023	1
H	72.91448	76.72743	71.60657	-1048.71298	1223.58118	-2196.97971	1
H	71.50250	75.90940	70.82456	419.06414	1903.92898	1903.44670	1
C	70.30952	75.03956	73.18474	-423.86431	152.66566	-648.55262	1
H	69.65609	75.93330	73.38535	105.68386	-2342.50080	1387.51238	1
H	69.88170	74.50400	72.29229	-1695.63603	-1484.73621	-1531.02793	1
H	70.21131	74.34263	74.06227	-1853.57121	-487.39031	1936.59343	1
C	74.20235	74.74644	72.54958	539.71428	-510.58232	267.58633	1
O	74.87254	75.11097	73.48448	-15.93758	-278.34085	-624.49402	1
N	74.48207	74.80772	71.25555	-676.81886	12.05292	276.17960	1
H	73.77706	74.49322	70.59219	-1694.44250	-373.84189	-2100.66098	1
C	75.80335	75.15830	70.87576	412.33078	-664.70360	108.35451	1
H	76.34563	75.32065	71.83843	1765.77476	1294.44366	1621.76594	1
C	76.37031	73.97382	69.98300	-179.66635	-721.95667	-264.73915	1
H	76.19224	72.98745	70.46629	-607.34922	2587.87032	-598.10018	1
H	75.84109	73.93220	69.00498	-359.69332	-1479.13954	-2259.74260	1
C	77.89794	74.23986	69.78617	-240.01076	616.02425	431.85322	1
H	78.07594	75.27652	69.42026	-1359.17131	782.54425	2227.99181	1
H	78.44292	74.15906	70.75354	-820.36667	85.81767	-2596.78715	1
C	78.44161	73.18746	68.73859	-513.07015	236.18570	-551.87280	1
H	78.27566	72.14488	69.09506	1816.11944	-1183.11722	-1650.94321	1
H	77.89357	73.26800	67.77168	-1800.04429	-1361.53473	1526.34643	1
C	79.95021	73.47883	68.55628	726.08972	-253.19732	-179.64713	1
H	80.19716	74.53353	68.30407	1224.27271	-456.51798	-2390.90304	1
H	80.59064	73.13898	69.39439	1206.40339	-1150.93514	-2154.90344	1
N	80.43279	72.65968	67.31315	329.70796	-633.68968	-155.67358	1
H	81.46360	72.76642	67.08738	1348.31305	1938.76845	1359.00643	1
H	80.34238	71.60291	67.36590	2096.92035	1446.54551	-966.48890	1

H	79.99715	72.87231	66.36840	-1197.07217	-2303.53719	827.28733	1
C	75.79341	76.45166	69.85471	-378.01186	-556.17301	-413.96481	1
O	75.11871	76.53798	68.82630	-409.44433	-307.55053	-453.30833	1
N	76.76253	77.22660	70.26768	-429.02477	524.70884	274.08502	1
H	77.23852	76.90475	71.10834	1706.34263	-1425.96036	1574.38926	1
C	76.95649	78.56212	69.78966	-45.25864	-626.22952	478.93267	1
H	75.92594	78.96524	69.66523	1988.63164	570.90773	1772.87287	1
C	77.81613	79.28506	70.90322	551.77128	-20.49526	-564.54996	1
H	77.36466	79.13794	71.91013	1434.61535	-1753.10253	1513.99471	1
H	78.82417	78.81614	70.96060	-714.67738	1819.09770	1898.36494	1
C	77.94838	80.80874	70.57391	446.99263	-555.77305	338.97221	1
H	78.27518	80.94815	69.51053	1953.68466	1892.25957	-161.67766	1
H	76.97011	81.33125	70.67129	-403.03785	2688.07032	-188.43591	1
C	79.02187	81.40833	71.56948	567.86262	335.74681	434.04421	1
H	78.67233	81.33576	72.62505	-1080.02275	1568.26081	-1948.78900	1
H	79.96906	80.82302	71.52465	-919.54108	2111.34692	1456.13463	1
C	79.28067	82.87928	71.17030	195.81897	173.33862	-745.11536	1
H	79.71171	83.01183	70.15778	1405.67949	-2301.21089	-390.08113	1
H	78.41297	83.55542	71.30417	-940.37328	-1118.32446	2299.72133	1
N	80.37827	83.44213	72.13612	319.26422	656.36007	-42.02424	1
H	80.67062	84.44663	71.96133	1961.03633	619.91968	1787.09422	1
H	80.14626	83.46947	73.17146	854.14343	-2156.80752	1429.08711	1
H	81.31847	82.94952	72.13461	1237.02905	-1378.50857	-1998.28500	1
C	77.75135	78.54370	68.37335	532.53319	-268.57236	517.55729	1
O	78.36784	79.51916	68.00464	266.16714	-290.73804	-558.88002	1
N	77.61695	77.37369	67.74066	-226.25352	640.49678	-270.32839	1
H	76.99207	76.67501	68.15995	2007.69063	-1035.27229	1523.50231	1
C	77.85482	77.40561	66.32659	-540.76542	-573.33303	49.50000	1
H	78.66500	78.16146	66.19408	-2374.20950	-1036.76023	843.75774	1
C	78.29231	75.92805	65.88656	-77.09082	666.87422	-415.84136	1
H	77.56262	75.18798	66.31311	-985.81834	617.19825	2463.91927	1
O	79.59769	75.53707	66.34656	293.29899	-391.65602	-477.80926	1
H	79.39248	74.72267	66.84627	2528.72714	-681.76262	-751.27122	1
C	78.12360	75.96393	64.32608	252.47363	-605.14473	440.05134	1
H	78.45042	75.00601	63.85777	-2052.65063	1456.49051	-1043.51830	1
H	77.05988	76.12452	64.02516	-2152.98800	-1661.97574	-161.70652	1
H	78.72744	76.78248	63.86913	-473.12726	-1584.27339	2165.61725	1
C	76.49186	77.87899	65.58121	360.36307	488.46713	505.10056	1
O	76.61392	78.31702	64.45722	-200.08817	-528.03552	-385.82850	1
N	75.37004	77.67826	66.26842	-597.54095	-226.44775	355.20479	1
H	75.33569	77.28919	67.24146	2179.23655	-1300.70981	-991.33835	1
C	74.17805	77.83323	65.49344	-687.68921	297.39807	-249.45179	1
H	74.57354	78.23382	64.52324	-349.16256	-2467.37713	-1101.72831	1
C	73.49736	76.38915	65.32779	672.47345	361.54328	-201.62880	1
H	73.31622	75.92375	66.32384	1541.41502	582.18425	2169.97026	1
H	72.51127	76.49082	64.81700	-2534.67717	60.25153	997.72295	1
C	74.42552	75.51079	64.47851	574.26774	-389.43119	-377.02542	1
C	74.47605	75.72696	63.06816	688.82679	-287.33580	-257.96133	1
H	73.85412	76.50715	62.59333	331.14182	1470.38163	2269.80013	1
C	75.23774	74.50859	65.08997	-383.52382	-675.04808	144.24917	1
H	75.20835	74.35046	66.18324	4.09847	1517.59662	2262.86397	1
C	75.33209	74.93619	62.30357	-134.76730	428.45285	-649.50303	1
H	75.37384	75.10421	61.21311	-903.23433	1600.49020	2011.53387	1
C	76.07361	73.73706	64.27883	-335.53281	-615.00040	-364.39058	1
H	76.69843	72.96400	64.75879	1596.45398	-1369.33862	1732.02659	1
C	76.15523	73.91820	62.86708	585.48547	-340.24194	-406.23938	1
O	77.01353	73.15066	62.03718	-419.15649	-173.04408	-511.94141	1
H	77.38707	72.53388	62.70989	-1071.84350	1559.71507	-1960.13012	1
C	73.21528	78.90396	66.24279	291.41878	-183.13091	710.72246	1
O	73.62978	80.03706	66.47925	379.87695	523.47396	-222.23622	1
N	72.01488	78.40596	66.43050	564.30591	373.09380	277.24371	1
H	71.86492	77.43981	66.13985	-120.59931	-2436.07824	-1214.35098	1

```
C    71.06155  79.00438  67.30870      404.07514     -299.37480      608.84009          1
H    70.33752  78.18128  67.50511    -1071.07882     -841.69025     2359.66467          1
C    71.86527  79.45677  68.60460     -683.12807       68.71770     -390.13096          1
H    72.53658  78.63694  68.94742     2068.88918     1596.58590      770.89603          1
H    72.54075  80.31334  68.37007     1379.86429     2342.49779     -179.85638          1
C    70.84831  79.84895  69.71683     -176.39967      537.49466      550.97268          1
H    70.19885  80.69130  69.38814     -396.28051     1942.22168     1869.33314          1
H    70.15862  79.00771  69.95121      -94.44700      586.00678    -2659.20016          1
C    71.73073  80.24980  70.98852     -291.69381     -718.18079     -150.72929          1
H    72.34765  79.42634  71.43047    -2303.38776     1089.76242      964.62032          1
H    72.39813  81.13852  70.85133      884.16729     2568.80533     -207.74283          1
N    70.74071  80.59690  71.91918     -594.49522     -252.81478      342.29276          1
H    69.78460  80.54319  71.60325    -1735.17426     2084.05705     -263.71605          1
C    70.96768  80.97456  73.11192      595.23177     -271.52487      442.22352          1
N    72.30061  81.04491  73.53013      585.53813     -424.79750     -105.81303          1
H    73.15273  80.82965  73.01386      959.88797     1327.56823    -2177.11905          1
H    72.60652  81.33160  74.46057     1578.48647       16.27378    -2220.76233          1
N    69.76799  81.25290  73.78154     -452.17763     -390.33541     -421.51853          1
H    68.80044  81.21020  73.46493      470.26107     1974.25629    -1817.92087          1
H    69.73026  81.58248  74.74674     1856.67307    -1573.97321    -1224.35416          1
C    70.34211  80.26274  66.57440     -531.47271     -581.45780      -55.06206          1
O    69.13326  80.26510  66.46292     -243.00837      192.73507      609.52485          1
N    71.19927  81.20819  66.22129      343.49288     -644.25607       38.09164          1
H    72.22974  81.04746  66.34757    -1691.16043    -1535.97525    -1484.73099          1
C    70.68672  82.41118  65.54832      542.66492      350.14337      454.42563          1
H    69.75357  82.60652  66.12841     1209.57979    -2396.95920      463.86744          1
C    71.72741  83.51257  65.61301     -737.14309      145.93010      242.71942          1
H    72.65109  83.13667  65.11706    -1050.18303      159.36419    -2509.06125          1
C    71.26686  84.75242  64.86772     -706.77696     -252.88343     -245.16230          1
H    72.04355  85.56585  64.89017    -1624.64039    -1665.49085     1417.86935          1
H    71.05275  84.52384  63.78514     2566.10521      598.51109      693.22305          1
H    70.32454  85.17777  65.31131    -1972.69758      107.10018    -1876.34312          1
C    72.08595  83.87460  67.04268      412.85148      672.58832      -27.69222          1
H    72.88550  84.66479  67.08374     1447.02277     1762.76533    -1490.78944          1
H    71.19525  84.27228  67.60296     1320.77379     -145.20455     2378.68491          1
H    72.46511  82.98201  67.61264    -1512.10848     1470.52031     1724.75147          1
C    70.34380  81.98989  63.98191     -607.11113      499.56689       73.73769          1
O    71.04679  82.35192  63.07190      357.97126      460.81534      356.68785          1
N    69.23817  81.26653  63.93841      536.55407     -368.62270      332.76306          1
H    68.76450  80.95801  64.81165      524.38866     2297.69985     1367.20941          1
C    68.78046  80.88181  62.64767       46.50277     -774.29190     -147.97904          1
H    69.16504  81.67469  61.96635     1676.51875     1883.29066     1032.55549          1
C    69.40895  79.43620  62.27985     -559.41456      -34.19198     -556.30433          1
H    69.21214  78.72616  63.11754      -64.66664      395.18769     2695.05373          1
H    68.90148  79.00510  61.38447    -2703.44278      -60.40056      333.79418          1
C    70.88496  79.57135  62.03502     -519.76435      377.31045      459.42366          1
H    71.34273  80.09143  62.91092    -1746.74323     1667.50644    -1261.73691          1
C    71.52770  78.19782  61.88397      570.95554       16.46261     -545.27700          1
H    72.63098  78.27655  61.70583     2391.58273       28.05277    -1305.07271          1
H    71.38209  77.56854  62.79931      175.75588      917.63458     2559.43867          1
H    71.09238  77.63303  61.01953    -1063.97851    -2155.34650     1283.00462          1
C    71.17130  80.40522  60.79289      193.76014        2.62946     -765.53141          1
H    72.25549  80.36925  60.51215      507.39394     -846.55087     2539.60089          1
H    70.58505  80.05026  59.90606     1337.57841     1818.19054     1526.01994          1
H    70.91448  81.48523  60.96056     1100.88635    -1172.91350    -2199.08909          1
C    67.17852  80.75210  62.69294     -549.21115      475.64486     -309.38218          1
O    66.52181  80.90927  63.68848      556.06106     -390.37755      -78.24250          1
O    66.55295  80.42032  61.46422     -572.27940     -310.19032     -209.75862          1
H    67.35966  80.40298  60.88147    -1380.08193     2212.14486      790.85944          1
```

Listing 37: C00037.xmolout

```
     276
  150.0000  150.0000  150.0000    0.0000    0.0000    0.0000***********   1.8545     7418
       0.2500    300.00   0.00   0.00   0.00  -1293.8088      0.0000    300.0000
H    69.44136  77.30548  71.89115    -2043.14101    -1757.64893      399.89339          1
N    69.29526  78.30699  71.75896      426.75047      490.45733     -334.42467          1
H    68.83847  78.55911  72.63808      442.25559    -1110.30854    -2448.52907          1
C    70.50450  79.02763  71.57384      415.32972     -666.39213       83.73172          1
H    71.31273  78.32704  71.87470      879.32219     2491.00447      667.35213          1
C    70.63020  79.46530  70.02785       31.35357      477.41777      628.23362          1
H    69.77767  80.13400  69.76085     1168.38489     2240.25337    -1019.71136          1
H    71.55907  80.06157  69.86180     -360.43770     2534.90498      931.67112          1
C    70.62396  78.23140  69.16351      463.18208      512.70242     -382.34388          1
H    69.78803  77.57673  69.49936     1725.29117     1863.52247     -987.07854          1
C    70.38555  78.60479  67.70572      710.42801     -144.27427     -313.15367          1
H    70.35091  77.69919  67.04679      364.20195    -1033.13725     2494.72524          1
H    69.41568  79.14990  67.57155     2567.61904      335.80679      847.48802          1
H    71.19677  79.27008  67.31246    -2587.99029      680.92867     -512.16411          1
C    71.93088  77.46030  69.28797      268.17616      741.64379      -40.42634          1
H    71.92214  76.52892  68.66484     1283.54314    -1945.37420    -1411.27812          1
H    72.80585  78.07501  68.95338    -1258.57796     1317.15249    -2026.02085          1
H    72.13477  77.14873  70.34526    -2502.84526      909.80597      575.92391          1
C    70.35967  80.33389  72.55998      619.36578     -458.64843     -172.09273          1
O    69.95450  80.12056  73.87222      -81.40433      433.87611      522.34594          1
N    71.66909  80.99314  72.43702      175.23996     -622.20190      341.55523          1
H    71.84368  81.37762  71.50605     -876.57256     -303.22879    -2561.90213          1
C    71.81801  81.96868  73.48467     -571.88214     -534.10783     -106.15033          1
H    71.70029  81.38287  74.42468    -2713.20841      -16.25800     -248.80809          1
C    73.27172  82.60073  73.31152      166.60354      288.16399     -716.09568          1
H    73.27558  83.24523  72.39026    -2097.85574     1636.78449     -586.18044          1
O    74.35295  81.66248  73.15294     -317.80580     -363.38227      484.43082          1
H    74.69254  81.90931  72.26978     1863.74748    -1451.84923     1357.29467          1
C    73.33337  83.51807  74.58850      503.80731      517.99032      318.51606          1
H    74.26302  84.13301  74.60190      640.61020    -2157.10410    -1536.29104          1
H    72.46825  84.22295  74.63292     1687.88455      465.60692     2087.56452          1
H    73.32314  82.91228  75.52478    -1293.07895    -1899.88581    -1463.57452          1
C    70.67457  83.10310  73.29023     -153.07550     -733.68902      248.70981          1
O    71.01396  84.17720  72.86976      186.15459     -480.78787     -449.34387          1
N    69.40352  82.68141  73.59615     -487.53139      543.18517       42.03850          1
C    68.24024  83.37059  73.06318     -699.35871       69.34393      360.10701          1
H    68.52410  83.78762  72.07117        2.81126     -221.99807    -2715.58072          1
C    67.18672  82.19848  73.05083     -507.46439     -592.59194      122.07761          1
H    66.13551  82.55530  72.96862    -1975.08116      525.87560    -1801.71556          1
H    67.35832  81.48653  72.21297     1588.27743    -1913.22100     1113.83566          1
C    67.53252  81.55697  74.44125     -613.62442     -457.55993     -194.14490          1
H    67.16484  82.18628  75.28879     1707.54637     -721.27992     1996.92523          1
H    67.09608  80.54065  74.58133      758.23357     2100.81767     1560.55011          1
C    69.09762  81.55818  74.36121     -352.41524      704.96076       49.21465          1
H    69.69690  81.53852  75.30624     1714.76886    -2070.42778      443.35792          1
H    69.69829  81.10678  72.07371     1224.15040     1801.50881     1636.97633          1
C    67.64535  84.48556  74.09345      566.85117      537.05831      117.62814          1
O    66.51425  84.93961  73.88520     -550.26779     -406.08867        4.21666          1
N    68.49465  84.81136  75.04339      566.34832     -425.77725      180.18749          1
H    69.36962  84.30751  74.89808      644.44502     2646.80550      -52.73430          1
C    68.11028  85.15707  76.38280      612.34801     -281.48662      411.56221          1
H    68.80758  85.98423  76.63545     1667.23498    -1490.75142     1556.16753          1
C    66.57964  85.55657  76.48041     -714.45746     -175.43074      286.98927          1
H    65.98190  84.68411  76.08671     -787.09484     1763.03619    -1922.46060          1
O    66.19172  86.68106  75.69049     -429.46514      516.51932     -128.40526          1
H    66.03475  86.19457  74.79015     1981.82785      436.16847    -1818.18182          1
C    66.35996  85.68246  78.03370      630.79189     -468.94448       76.03230          1
H    65.29023  85.88056  78.27836    -1775.71861     2062.30353      131.89849          1
```

H	66.65891	84.74969	78.57387	-1855.14608	88.94118	1993.53760	1
H	66.95511	86.52071	78.46469	-1504.82350	-780.94249	-2132.90992	1
C	68.43112	83.81012	77.25531	288.57638	618.62814	-397.00270	1
O	67.55680	82.98110	77.43429	321.70495	-185.26452	574.37218	1
N	69.72639	83.76438	77.53463	-447.05792	-446.53431	367.76425	1
H	70.24185	84.56217	77.15508	350.31316	1975.63644	-1843.31522	1
C	70.26915	83.17485	78.71129	-511.23061	374.03917	-471.51480	1
H	71.35301	83.33323	78.51221	2234.79579	-898.70890	-1273.45190	1
C	69.84629	84.01751	80.01346	529.53781	-159.34528	563.72604	1
H	70.47949	83.62448	80.85424	654.59388	2347.94251	1217.51488	1
H	70.15450	85.08451	79.83865	-1526.55661	2103.42026	817.87394	1
O	68.44652	84.01833	80.38322	-554.84278	14.12789	399.58769	1
H	68.39640	83.15020	80.83213	1297.06236	2061.84854	1220.68812	1
C	69.92212	81.60990	78.90713	-662.15484	-412.13544	123.62687	1
O	70.05228	81.08601	79.98720	-540.01537	-317.05804	274.91456	1
N	69.75524	80.94694	77.70316	-202.76299	-690.00100	-131.49145	1
H	69.37268	81.69707	77.11824	-702.62132	-2617.37191	281.70413	1
C	69.26282	79.55601	77.77210	-491.73914	576.34187	222.73509	1
H	68.96143	79.43448	78.84068	1977.17978	-1823.58850	-434.68928	1
C	68.14124	79.38520	76.77256	-649.50797	-328.66843	306.11224	1
H	68.46046	79.87186	75.82455	2075.07803	1452.16490	-1004.45904	1
C	67.86769	77.92486	76.45599	-615.18735	-489.99689	-70.96352	1
H	67.03766	77.81130	75.70574	-1226.32661	-1724.38906	-1716.47170	1
H	68.77792	77.42204	76.02620	2381.64273	-969.84735	900.46860	1
H	67.57190	77.34975	77.37554	2122.74991	1702.23946	-141.36228	1
C	66.86710	80.05260	77.25083	-501.86074	-479.61276	-376.42482	1
H	66.03297	79.94855	76.50414	993.79261	-2246.95401	-1177.81305	1
H	66.50384	79.60934	78.21822	42.71279	-626.15391	-2651.37258	1
H	67.03404	81.17928	77.42728	-2026.39012	-1590.33709	-887.82922	1
C	70.53239	78.58274	77.36918	-672.89153	382.57394	-156.34072	1
O	70.76718	77.61340	78.05929	410.04627	221.86223	500.35950	1
N	71.10284	78.92841	76.23204	-378.11782	-443.79414	-441.10984	1
H	70.76979	79.74415	75.71097	2404.15973	-568.14894	1149.30085	1
C	72.28548	78.20591	75.86483	-544.54484	466.16019	331.29119	1
H	72.02917	77.15168	76.11036	-1409.80988	-170.17955	2325.32679	1
C	72.57170	78.51068	74.33912	-184.48284	116.78282	-758.89137	1
H	71.62218	78.56431	73.75707	1893.78641	1861.14652	611.04528	1
H	73.06036	79.50610	74.21823	-2170.79665	-974.19311	1327.50117	1
C	73.49993	77.35183	73.85597	389.51668	672.81878	-138.49152	1
H	74.33947	77.22305	74.57325	510.64577	1933.03327	1851.02484	1
H	72.95690	76.38241	73.90311	-2302.30869	-1105.72939	-948.89774	1
C	74.08935	77.55193	72.41018	-559.93427	-126.34590	542.30857	1
O	74.77298	76.68541	71.92826	-670.36003	-111.83166	76.36448	1
N	73.77764	78.73584	71.84014	-216.06729	-537.07037	446.51379	1
H	73.26179	79.53543	72.22923	-1251.96487	1074.12894	-2168.52500	1
H	74.13647	79.04516	70.93155	-2451.83023	-720.73851	944.84612	1
C	73.55916	78.78306	76.71975	546.24052	-563.88337	-85.11809	1
O	73.88914	79.93490	76.61851	71.70702	-307.40463	606.69673	1
N	74.15124	77.78363	77.39469	479.90863	-504.50129	222.86849	1
C	75.27333	78.04067	78.24053	75.51268	-600.91904	506.73700	1
H	75.15916	79.08935	78.59915	-2033.61562	1795.30103	254.89480	1
C	75.12741	76.91850	79.35227	523.79067	517.99274	284.45581	1
H	76.09330	76.67821	79.85072	1599.61665	-1127.00911	1895.98173	1
H	74.42524	77.23619	80.15533	-1881.83093	1275.82488	-1501.55016	1
C	74.53893	75.72313	78.53826	-600.05639	-404.05026	316.64485	1
H	75.33333	75.20102	77.95939	-2597.05260	496.36221	657.73249	1
H	74.04757	74.95041	79.17149	-1666.24518	1294.38985	-1723.90591	1
C	73.52517	76.47988	77.57967	493.57570	-129.95376	-602.56394	1
H	73.38542	76.00717	76.57859	-1469.08415	-637.84797	2204.22581	1
H	72.51117	76.71423	78.02969	-1038.97840	1068.88701	-2280.71789	1
C	76.68980	77.83310	77.43391	188.88100	241.05089	-727.87821	1
O	77.69718	78.29914	77.91106	531.88613	-49.19943	427.07960	1

N	76.56391	77.03286	76.37048	708.14833	26.93417	-179.73509	1
H	75.59202	76.78431	76.18878	-2232.69501	-1179.09831	1023.94816	1
C	77.50685	77.12428	75.30073	-738.32412	259.92548	104.42456	1
H	77.01249	76.56874	74.47312	2028.89123	1260.02983	1311.33324	1
C	77.69546	78.66107	74.94685	-258.65678	-345.52513	661.28459	1
H	78.04496	79.21708	75.84715	-1826.06090	-1801.46097	-918.64554	1
C	78.70491	78.81417	73.75886	-541.23400	531.92970	-218.41428	1
H	78.81961	79.87994	73.44928	1311.73218	-2219.18200	882.19023	1
H	79.72298	78.43363	74.03670	-1048.71298	1223.58118	-2196.97971	1
H	78.37118	78.24058	72.86188	419.06414	1903.92898	1903.44670	1
C	76.32636	79.23326	74.43935	-423.86431	152.66566	-648.55262	1
H	76.40589	80.30475	74.13762	105.68386	-2342.50080	1387.51238	1
H	75.95013	78.67093	73.55179	-1695.63603	-1484.73621	-1531.02793	1
H	75.53869	79.17523	75.23013	-1853.57121	-487.39031	1936.59343	1
C	78.89703	76.41802	75.76794	539.71428	-510.58232	267.58633	1
O	79.94782	76.70259	75.23700	-15.93758	-278.34085	-624.49402	1
N	78.65938	75.39381	76.60347	-676.81886	12.05292	276.17960	1
H	77.70290	75.38592	76.95471	-1694.44250	-373.84189	-2100.66098	1
C	79.76877	74.92914	77.39691	412.33078	-664.70360	108.35451	1
H	80.39706	75.83674	77.53921	1765.77476	1294.44366	1621.76594	1
C	79.17873	74.34806	78.73748	-179.66635	-721.95667	-264.73915	1
H	78.47247	75.06788	79.20978	-607.34922	2587.87032	-598.10018	1
H	78.57844	73.43507	78.52439	-359.69332	-1479.13954	-2259.74260	1
C	80.36454	74.01239	79.70586	-240.01076	616.02425	431.85322	1
H	81.17533	73.47262	79.14542	-1359.17131	782.54425	2227.99181	1
H	80.83316	74.94200	80.10065	-820.36667	85.81767	-2596.78715	1
C	79.83066	73.10263	80.88296	-513.07015	236.18570	-551.87280	1
H	79.13196	73.66817	81.54128	1816.11944	-1183.11722	-1650.94321	1
H	79.24514	72.24222	80.48495	-1800.04429	-1361.53473	1526.34643	1
C	81.06107	72.59713	81.67277	726.08972	-253.19732	-179.64713	1
H	81.74795	71.95034	81.09067	1224.27271	-456.51798	-2390.90304	1
H	81.63677	73.39112	82.18883	1206.40339	-1150.93514	-2154.90344	1
N	80.54702	71.66825	82.82364	329.70796	-633.68968	-155.67358	1
H	81.30165	71.26216	83.44871	1348.31305	1938.76845	1359.00643	1
H	79.89079	72.09611	83.54004	2096.92035	1446.54551	-966.48890	1
H	80.02449	70.78967	82.53715	-1197.07217	-2303.53719	827.28733	1
C	80.58144	73.76186	76.61094	-378.01186	-556.17301	-413.96481	1
O	81.46753	73.14826	77.18139	-409.44433	-307.55053	-453.30833	1
N	80.06801	73.48280	75.38819	-429.02477	524.70884	274.08502	1
H	79.40282	74.24484	75.22848	1706.34263	-1425.96036	1574.38926	1
C	80.98645	73.12850	74.31900	-45.25864	-626.22952	478.93267	1
H	81.34007	74.11781	73.95591	1988.63164	570.90773	1772.87287	1
C	82.13737	72.23082	74.89634	551.77128	-20.49526	-564.54996	1
H	82.83738	72.85285	75.50568	1434.61535	-1753.10253	1513.99471	1
H	81.69613	71.50130	75.61584	-714.67738	1819.09770	1898.36494	1
C	82.96166	71.48861	73.78645	446.99263	-555.77305	338.97221	1
H	82.28837	70.89583	73.11482	1953.68466	1892.25957	-161.67766	1
H	83.50166	72.20939	73.13194	-403.03785	2688.07032	-188.43591	1
C	83.96665	70.53523	74.54750	567.86262	335.74681	434.04421	1
H	84.65983	71.12363	75.19169	-1080.02275	1568.26081	-1948.78900	1
H	83.41537	69.85663	75.23860	-919.54108	2111.34692	1456.13463	1
C	84.75403	69.71279	73.50108	195.81897	173.33862	-745.11536	1
H	84.13192	69.03451	72.88350	1405.67949	-2301.21089	-390.08113	1
H	85.42467	70.30971	72.85135	-940.37328	-1118.32446	2299.72133	1
N	85.71021	68.75640	74.29140	319.26422	656.36007	-42.02424	1
H	86.32270	68.12533	73.69842	1961.03633	619.91968	1787.09422	1
H	86.42976	69.20825	74.92769	854.14343	-2156.80752	1429.08711	1
H	85.25355	68.05174	74.94075	1237.02905	-1378.50857	-1998.28500	1
C	80.05332	72.38367	73.22650	532.53319	-268.57236	517.55729	1
O	80.27424	71.22431	72.89013	266.16714	-290.73804	-558.88002	1
N	79.08605	73.18705	72.81391	-226.25352	640.49678	-270.32839	1
H	79.08621	74.13217	73.19559	2007.69063	-1035.27229	1523.50231	1

C	78.23591	72.83770	71.73327	-540.76542	-573.33303	49.50000	1
H	77.62388	73.75577	71.58715	-2374.20950	-1036.76023	843.75774	1
C	79.15683	72.50099	70.47251	-77.09082	666.87422	-415.84136	1
H	79.72593	71.55665	70.70012	-985.81834	617.19825	2463.91927	1
O	80.13955	73.49993	70.13523	293.29899	-391.65602	-477.80926	1
H	80.96836	73.01245	70.31105	2528.72714	-681.76262	-751.27122	1
C	78.09104	72.21991	69.35114	252.47363	-605.14473	440.05134	1
H	78.57792	71.91878	68.39452	-2052.65063	1456.49051	-1043.51830	1
H	77.38821	71.39556	69.64294	-2152.98800	-1661.97574	-161.70652	1
H	77.47714	73.12570	69.13673	-473.12726	-1584.27339	2165.61725	1
C	77.34970	71.54361	72.22130	360.36307	488.46713	505.10056	1
O	77.19565	70.55920	71.52743	-200.08817	-528.03552	-385.82850	1
N	76.81849	71.83293	73.39948	-597.54095	-226.44775	355.20479	1
H	77.08665	72.74644	73.76750	2179.23655	-1300.70981	-991.33835	1
C	76.14006	70.89451	74.21285	-687.68921	297.39807	-249.45179	1
H	75.61213	71.51736	74.96727	-349.16256	-2467.37713	-1101.72831	1
C	75.15468	69.97965	73.34192	672.47345	361.54328	-201.62880	1
H	75.73499	69.44640	72.54535	1541.41502	582.18425	2169.97026	1
H	74.69730	69.20138	73.99576	-2534.67717	60.25153	997.72295	1
C	74.07048	70.86521	72.71200	574.26774	-389.43119	-377.02542	1
C	72.94266	71.28039	73.48149	688.82679	-287.33580	-257.96133	1
H	72.82653	70.95769	74.53201	331.14182	1470.38163	2269.80013	1
C	74.20945	71.28535	71.35471	-383.52382	-675.04808	144.24917	1
H	75.08592	70.96759	70.75785	4.09847	1517.59662	2262.86397	1
C	71.99126	72.10378	72.87916	-134.76730	428.45285	-649.50303	1
H	71.11793	72.42242	73.47403	-903.23433	1600.49020	2011.53387	1
C	73.23109	72.11054	70.79620	-335.53281	-615.00040	-364.39058	1
H	73.35445	72.42925	69.74628	1596.45398	-1369.33862	1732.02659	1
C	72.09198	72.55325	71.53036	585.48547	-340.24194	-406.23938	1
O	71.09577	73.39699	70.97296	-419.15649	-173.04408	-511.94141	1
H	71.45808	73.49956	70.06069	-1071.84350	1559.71507	-1960.13012	1
C	77.19144	69.86958	74.91769	291.41878	-183.13091	710.72246	1
O	76.76877	69.07658	75.72945	379.87695	523.47396	-222.23622	1
N	78.43512	70.03946	74.49125	564.30591	373.09380	277.24371	1
H	78.65510	70.76846	73.79444	-120.59931	-2436.07824	-1214.35098	1
C	79.50432	69.19524	74.89893	404.07514	-299.37480	608.84009	1
H	80.40671	69.81758	74.68863	-1071.07882	-841.69025	2359.66467	1
C	79.28917	68.83875	76.42809	-683.12807	68.71770	-390.13096	1
H	78.95203	69.74150	76.98666	2068.88918	1596.58590	770.89603	1
H	78.46298	68.09042	76.53533	1379.86429	2342.49779	-179.85638	1
C	80.61502	68.27637	77.03035	-176.39967	537.49466	550.97268	1
H	80.92848	67.34339	76.50754	-396.28051	1942.22168	1869.33314	1
H	81.45782	68.99333	76.90733	-94.44700	586.00678	-2659.20016	1
C	80.28932	68.01707	78.57397	-291.69381	-718.18079	-150.72929	1
H	79.98098	68.91677	79.16465	-2303.38776	1089.76242	964.62032	1
H	79.53107	67.21134	78.75044	884.16729	2568.80533	-207.74283	1
N	81.50634	67.56061	79.09485	-594.49522	-252.81478	342.29276	1
H	82.18468	67.22850	78.42709	-1735.17426	2084.05705	-263.71605	1
C	81.79331	67.51370	80.33227	595.23177	-271.52487	442.22352	1
N	80.83543	67.95998	81.24964	585.53813	-424.79750	-105.81303	1
H	79.89082	68.30466	81.08382	959.88797	1327.56823	-2177.11905	1
H	80.94925	67.94602	82.26366	1578.48647	16.27378	-2220.76233	1
N	83.07881	66.99183	80.53293	-452.17763	-390.33541	-421.51853	1
H	83.77577	66.65390	79.87095	470.26107	1974.25629	-1817.92087	1
H	83.48742	66.86028	81.45915	1856.67307	-1573.97321	-1224.35416	1
C	79.52018	67.81056	74.02276	-531.47271	-581.45780	-55.06206	1
O	80.50906	67.11223	74.05231	-243.00837	192.73507	609.52485	1
N	78.39653	67.64482	73.33238	343.49288	-644.25607	38.09164	1
H	77.72297	68.39379	73.50188	-1691.16043	-1535.97525	-1484.73099	1
C	77.98545	66.40384	72.69013	542.66492	350.14337	454.42563	1
H	77.12594	66.70546	72.04396	1209.57979	-2396.95920	463.86744	1
C	79.16730	65.88494	71.88967	-737.14309	145.93010	242.71942	1

```
H   80.03014  65.76681  72.59973    -1050.18303      159.36419    -2509.06125          1
C   78.90403  64.54794  71.22870     -706.77696     -252.88343     -245.16230          1
H   79.79473  64.19908  70.63644    -1624.64039    -1665.49085     1417.86935          1
H   78.67151  63.72808  71.99111     2566.10521      598.51109      693.22305          1
H   78.03512  64.60111  70.51600    -1972.69758      107.10018    -1876.34312          1
C   79.57910  66.89498  70.83138      412.85148      672.58832      -27.69222          1
H   80.50843  66.57567  70.28537     1447.02277     1762.76533    -1490.78944          1
H   78.77213  67.04032  70.06204     1320.77379     -145.20455     2378.68491          1
H   79.78443  67.90216  71.28938    -1512.10848     1470.52031     1724.75147          1
C   77.47708  65.37382  73.89447     -607.11113      499.56689       73.73769          1
O   77.72227  65.53290  75.06534      357.97126      460.81534      356.68785          1
N   76.74693  64.41600  73.33746      536.55407     -368.62270      332.76306          1
H   76.68005  64.41193  72.32260      524.38866     2297.69985     1367.20941          1
C   76.39341  63.30637  74.15434       46.50277     -774.29190     -147.97904          1
H   76.30530  63.74177  75.17598     1676.51875     1883.29066     1032.55549          1
C   75.04864  62.62843  73.57481     -559.41456      -34.19198     -556.30433          1
H   75.26609  62.18378  72.57424      -64.66664      395.18769     2695.05373          1
H   74.73328  61.77548  74.22222    -2703.44278      -60.40056      333.79418          1
C   73.94941  63.65138  73.46026     -519.76435      377.31045      459.42366          1
H   74.33793  64.52101  72.88374    -1746.74323     1667.50644    -1261.73691          1
C   72.75898  63.06575  72.71081      570.95554       16.46261     -545.27700          1
H   71.93423  63.81487  72.59209     2391.58273       28.05277    -1305.07271          1
H   73.04422  62.71974  71.68388      175.75588      917.63458     2559.43867          1
H   72.32821  62.18247  73.24903    -1063.97851    -2155.34650     1283.00462          1
C   73.50203  64.14285  74.83011      193.76014        2.62946     -765.53141          1
H   72.65884  64.87677  74.75046      507.39394     -846.55087     2539.60089          1
H   73.14713  63.29862  75.47592     1337.57841     1818.19054     1526.01994          1
H   74.33551  64.65464  75.37742     1100.88635    -1172.91350    -2199.08909          1
C   77.56351  62.20484  74.06143     -549.21115      475.64486     -309.38218          1
O   78.40818  62.18984  73.19683      556.06106     -390.37755      -78.24250          1
O   77.47658  61.15008  74.99505     -572.27940     -310.19032     -209.75862          1
H   76.71355  61.49463  75.54249    -1380.08193     2212.14486      790.85944          1
```

Listing 38: C00038.xmolout

```
      276
  150.0000  150.0000  150.0000    0.0000    0.0000    0.0000***********    1.1302     4521
      0.2500    300.00    0.00    0.00    0.00  -1293.8079      0.0000    300.0000
H   65.35693  62.33953  70.99840    -2043.14101    -1757.64893      399.89339          1
N   65.64673  62.81309  70.14344      426.75047      490.45733     -334.42467          1
H   64.91415  63.52074  70.01103      442.25559    -1110.30854    -2448.52907          1
C   66.91889  63.42016  70.22570      415.32972     -666.39213       83.73172          1
H   67.49704  62.90581  71.02404      879.32219     2491.00447      667.35213          1
C   67.61703  63.31349  68.77442       31.35357      477.41777      628.23362          1
H   66.90322  63.67297  67.99536     1168.38489     2240.25337    -1019.71136          1
H   68.51056  63.98082  68.71393     -360.43770     2534.90498      931.67112          1
C   67.99771  61.87748  68.52782      463.18208      512.70242     -382.34388          1
H   67.14927  61.23302  68.85209     1725.29117     1863.52247     -987.07854          1
C   68.24430  61.63595  67.04382      710.42801     -144.27427     -313.15367          1
H   68.51435  60.56804  66.83773      364.20195    -1033.13725     2494.72524          1
H   67.33778  61.87383  66.42942     2567.61904      335.80679      847.48802          1
H   69.08323  62.27033  66.65726    -2587.99029      680.92867     -512.16411          1
C   69.24149  61.49305  69.31789      268.17616      741.64379      -40.42634          1
H   69.52852  60.42414  69.14224     1283.54314    -1945.37420    -1411.27812          1
H   70.12222  62.12332  69.02971    -1258.57796     1317.15249    -2026.02085          1
H   69.09116  61.61908  70.42071    -2502.84526      909.80597      575.92391          1
C   66.57111  64.97729  70.54359      619.36578     -458.64843     -172.09273          1
O   65.47171  65.45378  70.33706      -81.40433      433.87611      522.34594          1
N   67.66656  65.57378  71.00159      175.23996     -622.20190      341.55523          1
H   68.53486  65.04203  71.05762     -876.57256     -303.22879    -2561.90213          1
C   67.63083  66.96839  71.25404     -571.88214     -534.10783     -106.15033          1
H   66.55366  67.25509  71.23837    -2713.20841      -16.25800     -248.80809          1
```

C	68.38918	67.19545	72.65224	166.60354	288.16399	-716.09568	1
H	69.44996	66.84229	72.53115	-2097.85574	1636.78449	-586.18044	1
O	67.85726	66.48306	73.78057	-317.80580	-363.38227	484.43082	1
H	68.61379	65.90736	74.01419	1863.74748	-1451.84923	1357.29467	1
C	68.37844	68.76208	72.77462	503.80731	517.99032	318.51606	1
H	68.98959	69.11309	73.63822	640.61020	-2157.10410	-1536.29104	1
H	68.79264	69.25266	71.86082	1687.88455	465.60692	2087.56452	1
H	67.34595	69.15379	72.92671	-1293.07895	-1899.88581	-1463.57452	1
C	68.55555	67.64964	70.08631	-153.07550	-733.68902	248.70981	1
O	69.64053	67.17684	69.87773	186.15459	-480.78787	-449.34387	1
N	67.99174	68.74693	69.56524	-487.53139	543.18517	42.03850	1
C	68.58193	69.38419	68.42379	-699.35871	69.34393	360.10701	1
H	68.88432	68.55124	67.74941	2.81126	-221.99807	-2715.58072	1
C	67.41043	70.29534	67.87292	-507.46439	-592.59194	122.07761	1
H	67.78489	71.21589	67.37073	-1975.08116	525.87560	-1801.71556	1
H	66.80405	69.75266	67.11340	1588.27743	-1913.22100	1113.83566	1
C	66.56053	70.59857	69.14765	-613.62442	-457.55993	-194.14490	1
H	67.01023	71.42044	69.74822	1707.54637	-721.27992	1996.92523	1
H	65.51447	70.90717	68.92402	758.23357	2100.81767	1560.55011	1
C	66.65087	69.20349	69.90319	-352.41524	704.96076	49.21465	1
H	66.55173	69.25915	71.01160	1714.76886	-2070.42778	443.35792	1
H	65.95839	68.42034	69.51093	1224.15040	1801.50881	1636.97633	1
C	69.90319	70.26523	68.84131	566.85117	537.05831	117.62814	1
O	70.83791	70.24771	68.08296	-550.26779	-406.08867	4.21666	1
N	69.72488	70.96647	69.94722	566.34832	-425.77725	180.18749	1
H	68.88438	70.86757	70.53983	644.44502	2646.80550	-52.73430	1
C	70.73792	71.83473	70.44360	612.34801	-281.48662	411.56221	1
H	71.73309	71.44403	70.13241	1667.23498	-1490.75142	1556.16753	1
C	70.42994	73.29521	69.84572	-714.45746	-175.43074	286.98927	1
H	69.34739	73.52304	70.04876	-787.09484	1763.03619	-1922.46060	1
O	70.60499	73.45805	68.42957	-429.46514	516.51932	-128.40526	1
H	69.68217	73.60242	68.13789	1981.82785	436.16847	-1818.18182	1
C	71.33660	74.22529	70.72899	630.79189	-468.94448	76.03230	1
H	71.19104	75.29990	70.47241	-1775.71861	2062.30353	131.89849	1
H	71.11267	74.11003	71.81589	-1855.14608	88.94118	1993.53760	1
H	72.41840	73.99742	70.58723	-1504.82350	-780.94249	-2132.90992	1
C	70.37088	71.78202	72.02615	288.57638	618.62814	-397.00270	1
O	69.23395	71.44682	72.32222	321.70495	-185.26452	574.37218	1
N	71.37706	72.13158	72.82487	-447.05792	-446.53431	367.76425	1
H	72.24453	72.44478	72.39008	350.31316	1975.63644	-1843.31522	1
C	71.03284	72.65972	74.10270	-511.23061	374.03917	-471.51480	1
H	69.91918	72.62427	74.13577	2234.79579	-898.70890	-1273.45190	1
C	71.69917	71.69836	75.19613	529.53781	-159.34528	563.72604	1
H	71.12401	71.84481	76.15264	654.59388	2347.94251	1217.51488	1
H	71.51483	70.64663	74.85295	-1526.55661	2103.42026	817.87394	1
O	73.09097	71.91898	75.52836	-554.84278	14.12789	399.58769	1
H	73.51423	71.36284	74.84159	1297.06236	2061.84854	1220.68812	1
C	71.67160	74.15819	74.21613	-662.15484	-412.13544	123.62687	1
O	72.58474	74.53603	73.51075	-540.01537	-317.05804	274.91456	1
N	71.04956	74.79680	75.19367	-202.76299	-690.00100	-131.49145	1
H	70.27344	74.32023	75.64900	-702.62132	-2617.37191	281.70413	1
C	71.31876	76.16488	75.48009	-491.73914	576.34187	222.73509	1
H	72.41223	76.31162	75.32470	1977.17978	-1823.58850	-434.68928	1
C	70.45490	77.07784	74.50497	-649.50797	-328.66843	306.11224	1
H	69.38306	76.78647	74.54633	2075.07803	1452.16490	-1004.45904	1
C	70.66318	78.55751	74.97641	-615.18735	-489.99689	-70.96352	1
H	70.16470	79.28483	74.29375	-1226.32661	-1724.38906	-1716.47170	1
H	70.24615	78.71932	75.99909	2381.64273	-969.84735	900.46860	1
H	71.74478	78.82201	75.01842	2122.74991	1702.23946	-141.36228	1
C	71.00345	76.96300	73.04220	-501.86074	-479.61276	-376.42482	1
H	70.42044	77.59105	72.32837	993.79261	-2246.95401	-1177.81305	1
H	72.06988	77.27968	72.97546	42.71279	-626.15391	-2651.37258	1

89	H	70.95590	75.90871	72.68119	-2026.39012	-1590.33709	-887.82922	1
90	C	70.76327	76.43135	76.99508	-672.89153	382.57394	-156.34072	1
91	O	69.57871	76.50728	77.21142	410.04627	221.86223	500.35950	1
92	N	71.78232	76.58136	77.84325	-378.11782	-443.79414	-441.10984	1
93	H	72.77246	76.37094	77.54971	2404.15973	-568.14894	1149.30085	1
94	C	71.41322	76.84070	79.20124	-544.54484	466.16019	331.29119	1
95	H	70.40840	77.31630	79.10347	-1409.80988	-170.17955	2325.32679	1
96	C	71.30636	75.43236	79.93453	-184.48284	116.78282	-758.89137	1
97	H	70.57736	74.76892	79.41629	1893.78641	1861.14652	611.04528	1
98	H	72.28223	74.88920	79.90142	-2170.79665	-974.19311	1327.50117	1
99	C	70.87460	75.71417	81.40540	389.51668	672.81878	-138.49152	1
100	H	71.51741	76.51313	81.84417	510.64577	1933.03327	1851.02484	1
101	H	69.85352	76.15567	81.42818	-2302.30869	-1105.72939	-948.89774	1
102	C	70.92416	74.43818	82.32816	-559.93427	-126.34590	542.30857	1
103	O	70.65752	74.54349	83.49733	-670.36003	-111.83166	76.36448	1
104	N	71.27650	73.30270	81.68780	-216.06729	-537.07037	446.51379	1
105	H	71.49602	73.14851	80.69679	-1251.96487	1074.12894	-2168.52500	1
106	H	71.34321	72.38606	82.14396	-2451.83023	-720.73851	944.84612	1
107	C	72.47793	77.76610	80.02142	546.24052	-563.88337	-85.11809	1
108	O	71.96445	78.50929	80.82287	71.70702	-307.40463	606.69673	1
109	N	73.79354	77.52795	79.85608	479.90863	-504.50129	222.86849	1
110	C	74.72030	78.28351	80.63544	75.51268	-600.91904	506.73700	1
111	H	74.12649	78.92035	81.33362	-2033.61562	1795.30103	254.89480	1
112	C	75.53337	77.07879	81.28824	523.79067	517.99274	284.45581	1
113	H	76.50241	77.40060	81.72958	1599.61665	-1127.00911	1895.98173	1
114	H	74.95180	76.60016	82.10697	-1881.83093	1275.82488	-1501.55016	1
115	C	75.70449	76.10822	80.07945	-600.05639	-404.05026	316.64485	1
116	H	76.60504	76.37120	79.47399	-2597.05260	496.36221	657.73249	1
117	H	75.84050	75.04748	80.38882	-1666.24518	1294.38985	-1723.90591	1
118	C	74.37344	76.34190	79.24340	493.57570	-129.95376	-602.56394	1
119	H	74.56331	76.61086	78.17389	-1469.08415	-637.84797	2204.22581	1
120	H	73.62158	75.51749	79.30478	-1038.97840	1068.88701	-2280.71789	1
121	C	75.73843	79.10531	79.64119	188.88100	241.05089	-727.87821	1
122	O	76.59348	78.51018	79.02911	531.88613	-49.19943	427.07960	1
123	N	75.57019	80.43013	79.77891	708.14833	26.93417	-179.73509	1
124	H	74.74241	80.60327	80.35502	-2232.69501	-1179.09831	1023.94816	1
125	C	75.85602	81.38108	78.68996	-738.32412	259.92548	104.42456	1
126	H	76.01787	82.34178	79.23246	2028.89123	1260.02983	1311.33324	1
127	C	77.07669	80.94302	77.91648	-258.65678	-345.52513	661.28459	1
128	H	76.86201	79.93420	77.47041	-1826.06090	-1801.46097	-918.64554	1
129	C	77.43782	81.87627	76.77521	-541.23400	531.92970	-218.41428	1
130	H	78.40412	81.57276	76.28699	1311.73218	-2219.18200	882.19023	1
131	H	76.64769	81.87674	75.96000	-1048.71298	1223.58118	-2196.97971	1
132	H	77.56499	82.93669	77.12701	419.06414	1903.92898	1903.44670	1
133	C	78.28648	80.80509	78.82771	-423.86431	152.66566	-648.55262	1
134	H	79.16703	80.36735	78.28549	105.68386	-2342.50080	1387.51238	1
135	H	78.60858	81.79688	79.24718	-1695.63603	-1484.73621	-1531.02793	1
136	H	78.06133	80.12941	79.69933	-1853.57121	-487.39031	1936.59343	1
137	C	74.48284	81.46928	77.77556	539.71428	-510.58232	267.58633	1
138	O	73.58884	80.66028	77.82655	-15.93758	-278.34085	-624.49402	1
139	N	74.52519	82.59224	77.05184	-676.81886	12.05292	276.17960	1
140	H	75.39784	83.11021	77.12579	-1694.44250	-373.84189	-2100.66098	1
141	C	73.75195	82.59737	75.84438	412.33078	-664.70360	108.35451	1
142	H	72.75257	82.21154	76.14571	1765.77476	1294.44366	1621.76594	1
143	C	73.78524	84.07609	75.30594	-179.66635	-721.95667	-264.73915	1
144	H	73.40638	84.78616	76.07542	-607.34922	2587.87032	-598.10018	1
145	H	74.83600	84.38508	75.10472	-359.69332	-1479.13954	-2259.74260	1
146	C	72.92964	84.16369	73.99609	-240.01076	616.02425	431.85322	1
147	H	73.26525	83.40386	73.25505	-1359.17131	782.54425	2227.99181	1
148	H	71.85852	83.94396	74.20218	-820.36667	85.81767	-2596.78715	1
149	C	73.11114	85.62534	73.42033	-513.07015	236.18570	-551.87280	1
150	H	72.82202	86.38813	74.17932	1816.11944	-1183.11722	-1650.94321	1

H	74.18194	85.82514	73.18480	-1800.04429	-1361.53473	1526.34643	1
C	72.23964	85.76337	72.15064	726.08972	-253.19732	-179.64713	1
H	72.54173	85.10871	71.30901	1224.27271	-456.51798	-2390.90304	1
H	71.14926	85.67432	72.32737	1206.40339	-1150.93514	-2154.90344	1
N	72.43165	87.21968	71.60585	329.70796	-633.68968	-155.67358	1
H	71.87496	87.46094	70.73578	1348.31305	1938.76845	1359.00643	1
H	72.16261	88.01736	72.25235	2096.92035	1446.54551	-966.48890	1
H	73.41188	87.49871	71.30934	-1197.07217	-2303.53719	827.28733	1
C	74.51734	81.60932	74.77084	-378.01186	-556.17301	-413.96481	1
O	75.54334	81.94969	74.21993	-409.44433	-307.55053	-453.30833	1
N	73.78163	80.52600	74.60046	-429.02477	524.70884	274.08502	1
H	72.94742	80.46443	75.18343	1706.34263	-1425.96036	1574.38926	1
C	74.26445	79.35641	73.95006	-45.25864	-626.22952	478.93267	1
H	73.34617	78.80488	73.65269	1988.63164	570.90773	1772.87287	1
C	75.17659	79.80954	72.74172	551.77128	-20.49526	-564.54996	1
H	74.72105	80.67459	72.20862	1434.61535	-1753.10253	1513.99471	1
H	76.16745	80.17335	73.11002	-714.67738	1819.09770	1898.36494	1
C	75.34663	78.57490	71.79345	446.99263	-555.77305	338.97221	1
H	75.87738	77.72742	72.32803	1953.68466	1892.25957	-161.67766	1
H	74.35517	78.17712	71.47932	-403.03785	2688.07032	-188.43591	1
C	76.18247	79.05175	70.54008	567.86262	335.74681	434.04421	1
H	75.69706	79.92411	70.04492	-1080.02275	1568.26081	-1948.78900	1
H	77.19445	79.40187	70.84932	-919.54108	2111.34692	1456.13463	1
C	76.28855	77.85522	69.56561	195.81897	173.33862	-745.11536	1
H	76.87029	76.99503	69.95348	1405.67949	-2301.21089	-390.08113	1
H	75.31908	77.50443	69.15864	-940.37328	-1118.32446	2299.72133	1
N	77.08701	78.34420	68.31068	319.26422	656.36007	-42.02424	1
H	77.21232	77.62816	67.53819	1961.03633	619.91968	1787.09422	1
H	76.68678	79.16572	67.77038	854.14343	-2156.80752	1429.08711	1
H	78.08934	78.65404	68.47178	1237.02905	-1378.50857	-1998.28500	1
C	75.19890	78.49016	74.99212	532.53319	-268.57236	517.55729	1
O	76.07795	78.96634	75.67139	266.16714	-290.73804	-558.88002	1
N	74.83170	77.21806	74.88880	-226.25352	640.49678	-270.32839	1
H	74.12774	76.97567	74.19379	2007.69063	-1035.27229	1523.50231	1
C	75.70753	76.22436	75.45118	-540.76542	-573.33303	49.50000	1
H	76.09597	76.68088	76.39323	-2374.20950	-1036.76023	843.75774	1
C	74.84891	74.93932	75.64994	-77.09082	666.87422	-415.84136	1
H	74.21190	74.73877	74.74718	-985.81834	617.19825	2463.91927	1
O	73.70798	75.18091	76.76571	293.29899	-391.65602	-477.80926	1
H	72.99513	74.76525	76.28943	2528.72714	-681.76262	-751.27122	1
C	75.73887	73.72046	75.97732	252.47363	-605.14473	440.05134	1
H	75.11794	72.79139	76.06956	-2052.65063	1456.49051	-1043.51830	1
H	76.49904	73.53501	75.17850	-2152.98800	-1661.97574	-161.70652	1
H	76.28644	73.85886	76.94203	-473.12726	-1584.27339	2165.61725	1
C	76.92759	75.97205	74.36009	360.36307	488.46713	505.10056	1
O	76.85725	76.30022	73.18962	-200.08817	-528.03552	-385.82850	1
N	77.90085	75.37356	75.01772	-597.54095	-226.44775	355.20479	1
H	77.68401	75.24226	76.00461	2179.23655	-1300.70981	-991.33835	1
C	78.89513	74.56203	74.40496	-687.68921	297.39807	-249.45179	1
H	78.94076	73.70117	75.10725	-349.16256	-2467.37713	-1101.72831	1
C	78.52781	74.15692	72.90922	672.47345	361.54328	-201.62880	1
H	78.28293	75.06738	72.30192	1541.41502	582.18425	2169.97026	1
H	79.41471	73.67849	72.43135	-2534.67717	60.25153	997.72295	1
C	77.34453	73.17193	72.94131	574.26774	-389.43119	-377.02542	1
C	77.52313	71.87012	73.49553	688.82679	-287.33580	-257.96133	1
H	78.50399	71.55929	73.89843	331.14182	1470.38163	2269.80013	1
C	76.06788	73.56333	72.44053	-383.52382	-675.04808	144.24917	1
H	75.91530	74.57629	72.02238	4.09847	1517.59662	2262.86397	1
C	76.43329	70.99632	73.52125	-134.76730	428.45285	-649.50303	1
H	76.57697	69.98975	73.95103	-903.23433	1600.49020	2011.53387	1
C	75.00650	72.65296	72.49369	-335.53281	-615.00040	-364.39058	1
H	74.02831	72.98463	72.10670	1596.45398	-1369.33862	1732.02659	1

213	C	75.14316	71.33810	73.02329	585.48547	-340.24194	-406.23938	1
214	O	74.07790	70.38862	73.07337	-419.15649	-173.04408	-511.94141	1
215	H	73.33439	70.95500	72.76568	-1071.84350	1559.71507	-1960.13012	1
216	C	80.30675	75.38750	74.42825	291.41878	-183.13091	710.72246	1
217	O	80.86201	75.78420	73.43002	379.87695	523.47396	-222.23622	1
218	N	80.74163	75.42519	75.68734	564.30591	373.09380	277.24371	1
219	H	80.13478	75.08095	76.43066	-120.59931	-2436.07824	-1214.35098	1
220	C	81.86808	76.25128	75.97036	404.07514	-299.37480	608.84009	1
221	H	82.43562	76.32317	75.01188	-1071.07882	-841.69025	2359.66467	1
222	C	81.25431	77.63461	76.45578	-683.12807	68.71770	-390.13096	1
223	H	80.51678	78.01254	75.71189	2068.88918	1596.58590	770.89603	1
224	H	80.67933	77.48797	77.39772	1379.86429	2342.49779	-179.85638	1
225	C	82.40815	78.65980	76.66602	-176.39967	537.49466	550.97268	1
226	H	83.17949	78.26726	77.36849	-396.28051	1942.22168	1869.33314	1
227	H	82.94441	78.86964	75.71369	-94.44700	586.00678	-2659.20016	1
228	C	81.70853	79.97619	77.24644	-291.69381	-718.18079	-150.72929	1
229	H	80.92229	80.44321	76.60070	-2303.38776	1089.76242	964.62032	1
230	H	81.29093	79.85455	78.27845	884.16729	2568.80533	-207.74283	1
231	N	82.78931	80.86312	77.33353	-594.49522	-252.81478	342.29276	1
232	H	83.70964	80.45003	77.32173	-1735.17426	2084.05705	-263.71605	1
233	C	82.68782	82.12518	77.45245	595.23177	-271.52487	442.22352	1
234	N	81.40479	82.68030	77.49237	585.53813	-424.79750	-105.81303	1
235	H	80.49563	82.22063	77.46053	959.88797	1327.56823	-2177.11905	1
236	H	81.20385	83.67528	77.59812	1578.48647	16.27378	-2220.76233	1
237	N	83.95534	82.71830	77.52454	-452.17763	-390.33541	-421.51853	1
238	H	84.88602	82.30317	77.51153	470.26107	1974.25629	-1817.92087	1
239	H	84.10050	83.72257	77.63490	1856.67307	-1573.97321	-1224.35416	1
240	C	82.62904	75.55389	77.23259	-531.47271	-581.45780	-55.06206	1
241	O	82.11419	75.47217	78.33888	-243.00837	192.73507	609.52485	1
242	N	83.88632	75.18018	76.89511	343.49288	-644.25607	38.09164	1
243	H	83.93052	75.39523	75.89479	-1691.16043	-1535.97525	-1484.73099	1
244	C	84.36846	73.85860	77.39345	542.66492	350.14337	454.42563	1
245	H	83.41939	73.32493	77.62930	1209.57979	-2396.95920	463.86744	1
246	C	85.18691	73.19745	76.31661	-737.14309	145.93010	242.71942	1
247	H	86.00804	73.89549	76.03606	-1050.18303	159.36419	-2509.06125	1
248	C	85.81424	71.89868	76.79518	-706.77696	-252.88343	-245.16230	1
249	H	86.42238	71.40732	75.98707	-1624.64039	-1665.49085	1417.86935	1
250	H	86.50157	72.07280	77.67316	2566.10521	598.51109	693.22305	1
251	H	85.03513	71.15705	77.12271	-1972.69758	107.10018	-1876.34312	1
252	C	84.36695	72.91347	75.06808	412.85148	672.58832	-27.69222	1
253	H	84.99522	72.46232	74.25248	1447.02277	1762.76533	-1490.78944	1
254	H	83.53129	72.19239	75.28086	1320.77379	-145.20455	2378.68491	1
255	H	83.89781	73.84505	74.65020	-1512.10848	1470.52031	1724.75147	1
256	C	85.22997	74.22281	78.74252	-607.11113	499.56689	73.73769	1
257	O	86.38070	73.88624	78.86408	357.97126	460.81534	356.68785	1
258	N	84.42838	74.81423	79.62033	536.55407	-368.62270	332.76306	1
259	H	83.42414	74.98064	79.35993	524.38866	2297.69985	1367.20941	1
260	C	85.00364	75.65569	80.61655	46.50277	-774.29190	-147.97904	1
261	H	84.11454	75.94445	81.21923	1676.51875	1883.29066	1032.55549	1
262	C	86.10954	74.84731	81.46593	-559.41456	-34.19198	-556.30433	1
263	H	87.00569	74.66384	80.81944	-64.66664	395.18769	2695.05373	1
264	H	86.47078	75.45905	82.32745	-2703.44278	-60.40056	333.79418	1
265	C	85.51788	73.54067	81.92286	-519.76435	377.31045	459.42366	1
266	H	85.05444	73.05352	81.03431	-1746.74323	1667.50644	-1261.73691	1
267	C	86.59934	72.61454	82.46475	570.95554	16.46261	-545.27700	1
268	H	86.17908	71.61940	82.76258	2391.58273	28.05277	-1305.07271	1
269	H	87.39773	72.42325	81.70191	175.75588	917.63458	2559.43867	1
270	H	87.09949	73.04724	83.36893	-1063.97851	-2155.34650	1283.00462	1
271	C	84.44167	73.74862	82.97978	193.76014	2.62946	-765.53141	1
272	H	83.98535	72.77680	83.30032	507.39394	-846.55087	2539.60089	1
273	H	84.85344	74.24077	83.89804	1337.57841	1818.19054	1526.01994	1
274	H	83.60890	74.39479	82.60061	1100.88635	-1172.91350	-2199.08909	1

```
C    85.65495  76.93046  79.88873      -549.21115      475.64486     -309.38218          1
O    86.53875  77.56439  80.40568       556.06106     -390.37755      -78.24250          1
O    85.03380  77.40368  78.70586      -572.27940     -310.19032     -209.75862          1
H    84.46709  76.60986  78.49902     -1380.08193     2212.14486      790.85944          1
```

Listing 39: C00039.xmolout

```
        276
  150.0000  150.0000  150.0000     0.0000     0.0000     0.0000**********    2.7750    11100
       0.2500     300.00    0.00    0.00    0.00   -1293.8012       0.0000     300.0000
H    79.56307  71.49838  77.05859     -2043.14101    -1757.64893      399.89339          1
N    79.42056  72.50538  77.13012       426.75047      490.45733     -334.42467          1
H    79.31074  72.62630  78.13889       442.25559    -1110.30854    -2448.52907          1
C    80.54121  73.22787  76.67060       415.32972     -666.39213       83.73172          1
H    81.35306  72.49281  76.49126       879.32219     2491.00447      667.35213          1
C    80.14231  74.11334  75.39398        31.35357      477.41777      628.23362          1
H    79.35720  74.83785  75.73299      1168.38489     2240.25337    -1019.71136          1
H    81.01567  74.73540  75.07777      -360.43770     2534.90498      931.67112          1
C    79.63760  73.28301  74.23444       463.18208      512.70242     -382.34388          1
H    78.84190  72.59610  74.60084      1725.29117     1863.52247     -987.07854          1
C    79.04204  74.19702  73.17170       710.42801     -144.27427     -313.15367          1
H    78.71742  73.60828  72.27340       364.20195    -1033.13725     2494.72524          1
H    78.14851  74.75514  73.55622      2567.61904      335.80679      847.48802          1
H    79.78361  74.95894  72.81610     -2587.99029      680.92867     -512.16411          1
C    80.74998  72.45250  73.61308       268.17616      741.64379      -40.42634          1
H    80.36854  71.85317  72.74151      1283.54314    -1945.37420    -1411.27812          1
H    81.58440  73.09898  73.23377     -1258.57796     1317.15249    -2026.02085          1
H    81.19428  71.73208  74.34810     -2502.84526      909.80597      575.92391          1
C    80.87729  74.39358  77.80527       619.36578     -458.64843     -172.09273          1
O    80.28667  75.45563  77.74003       -81.40433      433.87611      522.34594          1
N    82.34460  74.33609  77.71105       175.23996     -622.20190      341.55523          1
H    82.75050  74.84560  76.92398      -876.57256     -303.22879    -2561.90213          1
C    82.92889  74.79363  78.96619      -571.88214     -534.10783     -106.15033          1
H    83.96544  74.38786  79.00755     -2713.20841      -16.25800     -248.80809          1
C    82.93255  76.40015  78.79698       166.60354      288.16399     -716.09568          1
H    81.86503  76.74667  78.70313     -2097.85574     1636.78449     -586.18044          1
O    83.61388  76.84253  77.61208      -317.80580     -363.38227      484.43082          1
H    82.88234  77.29324  77.14028      1863.74748    -1451.84923     1357.29467          1
C    83.51280  76.91461  80.15996       503.80731      517.99032      318.51606          1
H    83.50802  78.02909  80.20473       640.61020    -2157.10410    -1536.29104          1
H    82.91452  76.54563  81.03611      1687.88455      465.60692     2087.56452          1
H    84.56885  76.58765  80.30509     -1293.07895    -1899.88581    -1463.57452          1
C    81.94789  74.19291  80.03764      -153.07550     -733.68902      248.70981          1
O    80.81093  73.79611  79.58802       186.15459     -480.78787     -449.34387          1
N    82.37833  74.18919  81.26330      -487.53139      543.18517       42.03850          1
C    81.45590  74.21514  82.38487      -699.35871       69.34393      360.10701          1
H    80.64620  73.48718  82.15429         2.81126     -221.99807    -2715.58072          1
C    82.40942  73.82838  83.58841      -507.46439     -592.59194      122.07761          1
H    82.02762  74.18315  84.57202     -1975.08116      525.87560    -1801.71556          1
H    82.53740  72.72637  83.67423      1588.27743    -1913.22100     1113.83566          1
C    83.74013  74.52361  83.15930      -613.62442     -457.55993     -194.14490          1
H    83.65823  75.63111  83.26974      1707.54637     -721.27992     1996.92523          1
H    84.63052  74.19729  83.74293       758.23357     2100.81767     1560.55011          1
C    83.79330  74.09772  81.63422      -352.41524      704.96076       49.21465          1
H    84.39711  74.75731  80.97256      1714.76886    -2070.42778      443.35792          1
H    84.08498  73.03602  81.45873      1224.15040     1801.50881     1636.97633          1
C    80.91665  75.72989  82.57307       566.85117      537.05831      117.62814          1
O    81.69631  76.66899  82.73226      -550.26779     -406.08867        4.21666          1
N    79.61280  75.73133  82.56504       566.34832     -425.77725      180.18749          1
H    79.05294  74.87397  82.41024       644.44502     2646.80550      -52.73430          1
C    78.86544  76.87521  83.02297       612.34801     -281.48662      411.56221          1
H    79.36822  77.27459  83.93543      1667.23498    -1490.75142     1556.16753          1
```

C	78.90726	77.95247	81.83463	-714.45746	-175.43074	286.98927	1
H	78.70250	77.42924	80.85483	-787.09484	1763.03619	-1922.46060	1
O	80.18867	78.55207	81.57008	-429.46514	516.51932	-128.40526	1
H	80.80048	77.87041	82.02345	1981.82785	436.16847	-1818.18182	1
C	77.73360	78.93101	82.16057	630.79189	-468.94448	76.03230	1
H	77.59882	79.68734	81.35099	-1775.71861	2062.30353	131.89849	1
H	76.76071	78.38942	82.26069	-1855.14608	88.94118	1993.53760	1
H	77.90920	79.48660	83.11223	-1504.82350	-780.94249	-2132.90992	1
C	77.46298	76.15713	83.28360	288.57638	618.62814	-397.00270	1
O	77.27185	75.01477	82.90900	321.70495	-185.26452	574.37218	1
N	76.56688	76.93195	83.94321	-447.05792	-446.53431	367.76425	1
H	76.63731	77.91488	84.27846	350.31316	1975.63644	-1843.31522	1
C	75.37541	76.53250	84.28752	-511.23061	374.03917	-471.51480	1
H	75.14600	75.48490	84.01456	2234.79579	-898.70890	-1273.45190	1
C	74.44116	77.47000	84.96845	529.53781	-159.34528	563.72604	1
H	73.44323	77.00656	85.11118	654.59388	2347.94251	1217.51488	1
H	74.82184	77.84959	85.97169	-1526.55661	2103.42026	817.87394	1
O	74.51405	79.12031	84.56610	-554.84278	14.12789	399.58769	1
H	73.78064	79.39346	85.09207	1297.06236	2061.84854	1220.68812	1
C	74.57068	78.95406	81.18421	-662.15484	-412.13544	123.62687	2
O	75.43792	78.71841	80.42570	-540.01537	-317.05804	274.91456	2
N	73.64532	79.20747	81.99485	-202.76299	-690.00100	-131.49145	2
H	73.81324	79.11580	83.04767	-702.62132	-2617.37191	281.70413	2
C	72.49187	79.89371	81.52474	-491.73914	576.34187	222.73509	2
H	71.92306	80.06053	82.46726	1977.17978	-1823.58850	-434.68928	2
C	72.98975	81.25787	80.87220	-649.50797	-328.66843	306.11224	2
H	73.59408	81.05958	79.96131	2075.07803	1452.16490	-1004.45904	2
C	71.76959	82.18577	80.55195	-615.18735	-489.99689	-70.96352	2
H	72.09641	83.13593	80.06755	-1226.32661	-1724.38906	-1716.47170	2
H	71.04026	81.69669	79.86455	2381.64273	-969.84735	900.46860	2
H	71.21335	82.46098	81.47834	2122.74991	1702.23946	-141.36228	2
C	73.85797	82.01788	81.93250	-501.86074	-479.61276	-376.42482	2
H	74.26307	82.97458	81.52729	993.79261	-2246.95401	-1177.81305	2
H	73.26575	82.26520	82.84446	42.71279	-626.15391	-2651.37258	2
H	74.72662	81.39981	82.26222	-2026.39012	-1590.33709	-887.82922	2
C	71.69050	78.86928	80.51136	-672.89153	382.57394	-156.34072	2
O	71.23353	77.84090	80.95655	410.04627	221.86223	500.35950	2
N	71.60373	79.32547	79.27233	-378.11782	-443.79414	-441.10984	2
H	72.02511	80.22402	79.05165	2404.15973	-568.14894	1149.30085	2
C	71.00830	78.49034	78.27764	-544.54484	466.16019	331.29119	2
H	70.28458	77.88935	78.87699	-1409.80988	-170.17955	2325.32679	2
C	70.34504	79.46849	77.21846	-184.48284	116.78282	-758.89137	2
H	69.67284	80.19057	77.73369	1893.78641	1861.14652	611.04528	2
H	71.12157	80.08947	76.71843	-2170.79665	-974.19311	1327.50117	2
C	69.56128	78.63069	76.16078	389.51668	672.81878	-138.49152	2
H	70.23541	77.87931	75.68778	510.64577	1933.03327	1851.02484	2
H	68.78441	78.01256	76.66325	-2302.30869	-1105.72939	-948.89774	2
C	68.89343	79.51297	75.03650	-559.93427	-126.34590	542.30857	2
O	68.44320	78.98754	74.05099	-670.36003	-111.83166	76.36448	2
N	68.86725	80.83385	75.31714	-216.06729	-537.07037	446.51379	2
H	69.19772	81.33205	76.15129	-1251.96487	1074.12894	-2168.52500	2
H	68.41784	81.53802	74.72151	-2451.83023	-720.73851	944.84612	2
C	72.04732	77.49861	77.50110	546.24052	-563.88337	-85.11809	2
O	71.53817	76.52821	76.99073	71.70702	-307.40463	606.69673	2
N	73.33169	77.87580	77.42267	479.90863	-504.50129	222.86849	2
C	74.28329	77.06199	76.72758	75.51268	-600.91904	506.73700	2
H	73.70851	76.51164	75.94716	-2033.61562	1795.30103	254.89480	2
C	75.32370	78.14357	76.20025	523.79067	517.99274	284.45581	2
H	76.31223	77.69761	75.94823	1599.61665	-1127.00911	1895.98173	2
H	74.95570	78.65926	75.28546	-1881.83093	1275.82488	-1501.55016	2
C	75.38233	79.11556	77.41848	-600.05639	-404.05026	316.64485	2
H	76.03530	78.70136	78.22214	-2597.05260	496.36221	657.73249	2

H	75.78783	80.12233	77.16997	-1666.24518	1294.38985	-1723.90591	2
C	73.86257	79.15096	77.88225	493.57570	-129.95376	-602.56394	2
H	73.71932	79.17404	78.98837	-1469.08415	-637.84797	2204.22581	2
H	73.23922	79.94326	77.40506	-1038.97840	1068.88701	-2280.71789	2
C	75.06719	76.06128	77.75619	188.88100	241.05089	-727.87821	2
O	76.24074	75.78209	77.52014	531.88613	-49.19943	427.07960	2
N	74.29604	75.60830	78.71587	708.14833	26.93417	-179.73509	2
H	73.33111	75.93205	78.70495	-2232.69501	-1179.09831	1023.94816	2
C	74.87331	74.79665	79.74129	-738.32412	259.92548	104.42456	2
H	75.85550	75.29030	79.91816	2028.89123	1260.02983	1311.33324	2
C	73.90314	74.73078	80.99001	-258.65678	-345.52513	661.28459	2
H	72.91351	74.32217	80.68896	-1826.06090	-1801.46097	-918.64554	2
C	74.56290	73.83741	82.09717	-541.23400	531.92970	-218.41428	2
H	73.93352	73.79175	83.01716	1311.73218	-2219.18200	882.19023	2
H	74.70502	72.78703	81.74435	-1048.71298	1223.58118	-2196.97971	2
H	75.58350	74.23445	82.39896	419.06414	1903.92898	1903.44670	2
C	73.74520	76.16451	81.60417	-423.86431	152.66566	-648.55262	2
H	73.08224	76.16353	82.50158	105.68386	-2342.50080	1387.51238	2
H	74.72941	76.58871	81.91605	-1695.63603	-1484.73621	-1531.02793	2
H	73.30003	76.87592	80.86732	-1853.57121	-487.39031	1936.59343	2
C	75.05871	73.27494	79.15108	539.71428	-510.58232	267.58633	2
O	74.33273	72.38863	79.52468	-15.93758	-278.34085	-624.49402	2
N	76.07734	73.25816	78.30645	-676.81886	12.05292	276.17960	2
H	76.55887	74.15947	78.06066	-1694.44250	-373.84189	-2100.66098	2
C	76.63222	72.03882	77.84300	412.33078	-664.70360	108.35451	2
H	77.56077	72.39344	77.33839	1765.77476	1294.44366	1621.76594	2
C	76.86866	71.07820	79.08056	-179.66635	-721.95667	-264.73915	2
H	77.44456	71.59165	79.88335	-607.34922	2587.87032	-598.10018	2
H	75.88644	70.80582	79.53686	-359.69332	-1479.13954	-2259.74260	2
C	77.61380	69.79942	78.57467	-240.01076	616.02425	431.85322	2
H	77.08139	69.36916	77.69542	-1359.17131	782.54425	2227.99181	2
H	78.64057	70.04287	78.22330	-820.36667	85.81767	-2596.78715	2
C	77.65434	68.74053	79.74872	-513.07015	236.18570	-551.87280	2
H	78.32375	69.07671	80.57366	1816.11944	-1183.11722	-1650.94321	2
H	76.64581	68.61298	80.20565	-1800.04429	-1361.53473	1526.34643	2
C	78.14211	67.41081	79.12738	726.08972	-253.19732	-179.64713	2
H	77.44463	66.96798	78.38828	1224.27271	-456.51798	-2390.90304	2
H	79.16657	67.44559	78.70630	1206.40339	-1150.93514	-2154.90344	2
N	78.21977	66.34610	80.27236	329.70796	-633.68968	-155.67358	2
H	78.52107	65.37468	79.97082	1348.31305	1938.76845	1359.00643	2
H	78.89578	66.53626	81.06828	2096.92035	1446.54551	-966.48890	2
H	77.31951	66.13188	80.79232	-1197.07217	-2303.53719	827.28733	2
C	75.61840	71.26327	76.80658	-378.01186	-556.17301	-413.96481	2
O	75.04699	70.24795	77.13559	-409.44433	-307.55053	-453.30833	2
N	75.63116	71.84419	75.61063	-429.02477	524.70884	274.08502	2
H	76.14570	72.71740	75.50954	1706.34263	-1425.96036	1574.38926	2
C	74.81394	71.21930	74.61719	-45.25864	-626.22952	478.93267	2
H	74.79431	70.14865	74.93104	1988.63164	570.90773	1772.87287	2
C	73.37534	71.87460	74.71985	551.77128	-20.49526	-564.54996	2
H	73.03817	71.94328	75.77851	1434.61535	-1753.10253	1513.99471	2
H	73.38254	72.91873	74.33480	-714.67738	1819.09770	1898.36494	2
C	72.42545	70.96039	73.86660	446.99263	-555.77305	338.97221	2
H	72.85019	70.79786	72.84771	1953.68466	1892.25957	-161.67766	2
H	72.32891	69.95004	74.32646	-403.03785	2688.07032	-188.43591	2
C	71.03794	71.67184	73.76871	567.86262	335.74681	434.04421	2
H	70.57753	71.80690	74.77213	-1080.02275	1568.26081	-1948.78900	2
H	71.15844	72.69486	73.35472	-919.54108	2111.34692	1456.13463	2
C	70.10743	70.78465	72.81689	195.81897	173.33862	-745.11536	2
H	70.44359	70.76494	71.75040	1405.67949	-2301.21089	-390.08113	2
H	69.97559	69.73549	73.18175	-940.37328	-1118.32446	2299.72133	2
N	68.80180	71.38310	72.82173	319.26422	656.36007	-42.02424	2
H	68.18427	71.04005	72.09093	1961.03633	619.91968	1787.09422	2

H	68.25233	71.17413	73.65117	854.14343	-2156.80752	1429.08711	2
H	68.65822	74.90668	72.56344	1237.02905	-1378.50857	-1998.28500	2
C	75.30428	71.45510	73.06913	532.53319	-268.57236	517.55729	2
O	75.04827	70.55645	72.27229	266.16714	-290.73804	-558.88002	2
N	75.78564	72.65457	72.84361	-226.25352	640.49678	-270.32839	2
H	75.85494	73.22552	73.68461	2007.69063	-1035.27229	1523.50231	2
C	76.33414	73.09781	71.60985	-540.76542	-573.33303	49.50000	2
H	76.90014	74.01144	71.89338	-2374.20950	-1036.76023	843.75774	2
C	77.26137	71.93853	71.00978	-77.09082	666.87422	-415.84136	2
H	76.60845	71.04841	70.78034	-985.81834	617.19825	2463.91927	2
O	78.30488	71.43815	71.87316	293.29899	-391.65602	-477.80926	2
H	77.96320	70.54082	72.06103	2528.72714	-681.76262	-751.27122	2
C	77.74942	72.58646	69.66466	252.47363	-605.14473	440.05134	2
H	78.33125	71.86047	69.05037	-2052.65063	1456.49051	-1043.51830	2
H	76.89427	72.94441	69.04085	-2152.98800	-1661.97574	-161.70652	2
H	78.41075	73.46425	69.85150	-473.12726	-1584.27339	2165.61725	2
C	75.15866	73.47426	70.54317	360.36307	488.46713	505.10056	2
O	75.34844	74.43768	69.81335	-200.08817	-528.03552	-385.82850	2
N	74.18027	72.60494	70.56408	-597.54095	-226.44775	355.20479	2
H	74.27880	71.78706	71.19847	2179.23655	-1300.70981	-991.33835	2
C	72.90983	72.85189	69.98322	-687.68921	297.39807	-249.45179	2
H	72.25582	72.10208	70.48141	-349.16256	-2467.37713	-1101.72831	2
C	72.45151	74.35371	70.21987	672.47345	361.54328	-201.62880	2
H	73.32627	75.04467	70.22340	1541.41502	582.18425	2169.97026	2
H	71.79258	74.67638	69.38291	-2534.67717	60.25153	997.72295	2
C	71.66422	74.44506	71.56783	574.26774	-389.43119	-377.02542	2
C	70.33967	74.06674	71.53902	688.82679	-287.33580	-257.96133	2
H	69.89025	73.67591	70.61843	331.14182	1470.38163	2269.80013	2
C	72.31353	74.93860	72.75076	-383.52382	-675.04808	144.24917	2
H	73.38051	75.20943	72.71983	4.09847	1517.59662	2262.86397	2
C	69.52104	74.23213	72.79739	-134.76730	428.45285	-649.50303	2
H	69.03887	73.24131	73.01976	-903.23433	1600.49020	2011.53387	2
C	71.59021	75.07622	73.93431	-335.53281	-615.00040	-364.39058	2
H	72.12540	75.46410	74.82183	1596.45398	-1369.33862	1732.02659	2
C	70.23655	74.76064	74.08495	585.48547	-340.24194	-406.23938	2
O	69.48043	74.85316	75.20468	-419.15649	-173.04408	-511.94141	2
H	70.17107	75.30143	75.82916	-1071.84350	1559.71507	-1960.13012	2
C	72.94043	72.61958	68.35515	291.41878	-183.13091	710.72246	2
O	72.11911	71.87911	67.87617	379.87695	523.47396	-222.23622	2
N	73.79196	73.45126	67.76898	564.30591	373.09380	277.24371	2
H	74.39675	74.02658	68.40591	-120.59931	-2436.07824	-1214.35098	2
C	74.37024	73.10459	66.49400	404.07514	-299.37480	608.84009	2
H	75.40986	72.78869	66.73056	-1071.07882	-841.69025	2359.66467	2
C	73.53720	72.01075	65.72833	-683.12807	68.71770	-390.13096	2
H	73.52672	71.04737	66.29114	2068.88918	1596.58590	770.89603	2
H	72.46923	72.32758	65.66233	1379.86429	2342.49779	-179.85638	2
C	74.14929	71.81326	64.30272	-176.39967	537.49466	550.97268	2
H	74.30102	72.79414	63.79638	-396.28051	1942.22168	1869.33314	2
H	75.15864	71.34954	64.36093	-94.44700	586.00678	-2659.20016	2
C	73.13458	70.90235	63.47316	-291.69381	-718.18079	-150.72929	2
H	72.92762	69.88761	63.89883	-2303.38776	1089.76242	964.62032	2
H	72.15028	71.38006	63.23248	884.16729	2568.80533	-207.74283	2
N	73.83290	70.72833	62.26860	-594.49522	-252.81478	342.29276	2
H	74.69502	71.24073	62.16422	-1735.17426	2084.05705	-263.71605	2
C	73.45430	69.98018	61.31328	595.23177	-271.52487	442.22352	2
N	72.25203	69.28125	61.46776	585.53813	-424.79750	-105.81303	2
H	71.60081	69.28097	62.25178	959.88797	1327.56823	-2177.11905	2
H	71.84676	68.65451	60.77182	1578.48647	16.27378	-2220.76233	2
N	74.36858	70.01536	60.25067	-452.17763	-390.33541	-421.51853	2
H	75.24772	70.51878	60.14197	470.26107	1974.25629	-1817.92087	2
H	74.24915	69.47467	59.39323	1856.67307	-1573.97321	-1224.35416	2
C	74.35546	74.45487	65.58384	-531.47271	-581.45780	-55.06206	2

```
O    75.20127   74.66778   64.74852      -243.00837       192.73507       609.52485          2
N    73.14585   75.11208   65.70918       343.49288      -644.25607        38.09164          2
H    72.83128   74.74659   66.61748     -1691.16043     -1535.97525     -1484.73099          2
C    73.12189   76.56092   65.40590       542.66492       350.14337       454.42563          2
H    74.18277   76.89044   65.48379      1209.57979     -2396.95920       463.86744          2
C    72.53899   76.74376   64.02286      -737.14309       145.93010       242.71942          2
H    71.58025   76.17429   63.98160     -1050.18303       159.36419     -2509.06125          2
C    72.21368   78.19875   63.73267      -706.77696      -252.88343      -245.16230          2
H    71.82838   78.33750   62.68525     -1624.64039     -1665.49085      1417.86935          2
H    71.41679   78.58299   64.43203      2566.10521       598.51109       693.22305          2
H    73.11402   78.86144   63.85257     -1972.69758       107.10018     -1876.34312          2
C    73.45428   76.20070   62.94038       412.85148       672.58832       -27.69222          2
H    73.00226   76.30498   61.91593      1447.02277      1762.76533     -1490.78944          2
H    74.44237   76.73811   62.92781      1320.77379      -145.20455      2378.68491          2
H    73.67778   75.10904   63.10201     -1512.10848      1470.52031      1724.75147          2
C    72.12902   77.27540   66.51028      -607.11113       499.56689        73.73769          2
O    72.35131   78.41491   66.84413       357.97126       460.81534       356.68785          2
N    71.15205   76.45656   66.92281       536.55407      -368.62270       332.76306          2
H    71.16889   75.56565   66.42545       524.38866      2297.69985      1367.20941          2
C    69.86720   77.02185   67.21797        46.50277      -774.29190      -147.97904          2
H    69.29543   76.14898   67.60103      1676.51875      1883.29066      1032.55549          2
C    70.01347   78.19672   68.30825      -559.41456       -34.19198      -556.30433          2
H    70.58810   79.04745   67.85603       -64.66664       395.18769      2695.05373          2
H    69.01842   78.61378   68.59619     -2703.44278       -60.40056       333.79418          2
C    70.74104   77.66366   69.51515      -519.76435       377.31045       459.42366          2
H    71.59740   77.04820   69.15641     -1746.74323      1667.50644     -1261.73691          2
C    71.29510   78.80744   70.35553       570.95554        16.46261      -545.27700          2
H    71.87063   78.42879   71.23946      2391.58273        28.05277     -1305.07271          2
H    71.98714   79.45794   69.75992       175.75588       917.63458      2559.43867          2
H    70.47621   79.46310   70.75008     -1063.97851     -2155.34650      1283.00462          2
C    69.82952   76.80011   70.37614       193.76014         2.62946      -765.53141          2
H    70.35766   76.43687   71.29556       507.39394      -846.55087      2539.60089          2
H    68.92620   77.36681   70.72086      1337.57841      1818.19054      1526.01994          2
H    69.46624   75.89714   69.82128      1100.88635     -1172.91350     -2199.08909          2
C    69.25075   77.55949   65.84539      -549.21115       475.64486      -309.38218          2
O    69.63049   77.18532   64.76380       556.06106      -390.37755       -78.24250          2
O    68.13245   78.42752   65.94162      -572.27940      -310.19032      -209.75862          2
H    68.04316   78.45714   66.92967     -1380.08193      2212.14486       790.85944          2
```

Listing 40: C00040.xmolout

```
       276
  150.0000  150.0000  150.0000     0.0000     0.0000     0.0000***********    2.3537     9415
        0.2500    300.00    0.00    0.00    0.00  -1293.7932       0.0000    300.0000
H    85.65516   69.35480   80.41490     -2043.14101     -1757.64893       399.89339          1
N    85.74069   69.77449   79.48950       426.75047       490.45733      -334.42467          1
H    84.80943   70.17133   79.30544       442.25559     -1110.30854     -2448.52907          1
C    86.83618   70.65016   79.33944       415.32972      -666.39213        83.73172          1
H    87.69461   70.14115   79.82907       879.32219      2491.00447       667.35213          1
C    87.06199   70.90420   77.76049        31.35357       477.41777       628.23362          1
H    86.14217   71.36999   77.33309      1168.38489      2240.25337     -1019.71136          1
H    87.88732   71.63600   77.59267      -360.43770      2534.90498       931.67112          1
C    87.36030   69.59576   77.07836       463.18208       512.70242      -382.34388          1
H    86.57895   68.86219   77.38146      1725.29117      1863.52247      -987.07854          1
C    87.30969   69.76557   75.56480       710.42801      -144.27427      -313.15367          1
H    87.49684   68.79671   75.03372       364.20195     -1033.13725      2494.72524          1
H    86.31214   70.14508   75.22371      2567.61904       335.80679       847.48802          1
H    88.08219   70.49384   75.20634     -2587.99029       680.92867      -512.16411          1
C    88.72341   69.04858   77.47975       268.17616       741.64379       -40.42634          1
H    88.95101   68.08434   76.95579      1283.54314     -1945.37420     -1411.27812          1
H    89.54714   69.76486   77.22677     -1258.57796      1317.15249     -2026.02085          1
H    88.78380   68.84778   78.58018     -2502.84526       909.80597       575.92391          1
```

C	86.45564	72.05891	80.04207	619.36578	-458.64843	-172.09273	1
O	85.31809	72.36712	80.39858	-81.40433	433.87611	522.34594	1
N	87.55285	72.78116	80.03384	175.23996	-622.20190	341.55523	1
H	88.40750	72.31771	79.73088	-876.57256	-303.22879	-2561.90213	1
C	87.60448	74.07037	80.65539	-571.88214	-534.10783	-106.15033	1
H	87.19912	73.87777	81.67399	-2713.20841	-16.25800	-248.80809	1
C	89.14648	74.48479	80.65712	166.60354	288.16399	-716.09568	1
H	89.49046	74.63076	79.59678	-2097.85574	1636.78449	-586.18044	1
O	90.03274	73.49916	81.22487	-317.80580	-363.38227	484.43082	1
H	90.64734	73.34738	80.48034	1863.74748	-1451.84923	1357.29467	1
C	89.13894	75.88294	81.37629	503.80731	517.99032	318.51606	1
H	90.16976	76.29678	81.47036	640.61020	-2157.10410	-1536.29104	1
H	88.53277	76.63815	80.82079	1687.88455	465.60692	2087.56452	1
H	88.71598	75.81015	82.40535	-1293.07895	-1899.88581	-1463.57452	1
C	86.66167	75.15244	79.88649	-153.07550	-733.68902	248.70981	1
O	86.34737	76.10353	80.55348	186.15459	-480.78787	-449.34387	1
N	86.49699	74.97081	78.57120	-487.53139	543.18517	42.03850	1
C	85.33788	75.48795	77.90022	-699.35871	69.34393	360.10701	1
H	85.21079	76.50769	78.32831	2.81126	-221.99807	-2715.58072	1
C	85.74378	75.44635	76.37494	-507.46439	-592.59194	122.07761	1
H	84.87323	75.25705	75.70628	-1975.08116	525.87560	-1801.71556	1
H	86.16505	76.42460	76.05213	1588.27743	-1913.22100	1113.83566	1
C	86.83448	74.32704	76.30554	-613.62442	-457.55993	-194.14490	1
H	86.38433	73.31198	76.24795	1707.54637	-721.27992	1996.92523	1
H	87.50917	74.41897	75.42520	758.23357	2100.81767	1560.55011	1
C	87.57963	74.55052	77.68920	-352.41524	704.96076	49.21465	1
H	88.05711	73.64443	78.12498	1714.76886	-2070.42778	443.35792	1
H	88.31495	75.38965	77.69142	1224.15040	1801.50881	1636.97633	1
C	84.04944	74.55147	78.30928	566.85117	537.05831	117.62814	1
O	83.46278	74.82283	79.33192	-550.26779	-406.08867	4.21666	1
N	83.87784	73.54304	77.46711	566.34832	-425.77725	180.18749	1
H	84.54446	73.48231	76.70044	644.44502	2646.80550	-52.73430	1
C	82.61969	72.88433	77.31963	612.34801	-281.48662	411.56221	1
H	82.77892	72.09231	76.55285	1667.23498	-1490.75142	1556.16753	1
C	82.31356	72.26101	78.75668	-714.45746	-175.43074	286.98927	1
H	82.41498	73.08736	79.52782	-787.09484	1763.03619	-1922.46060	1
O	83.25595	71.24394	79.16958	-429.46514	516.51932	-128.40526	1
H	83.88476	71.81053	79.74786	1981.82785	436.16847	-1818.18182	1
C	80.81586	71.82539	78.67645	630.79189	-468.94448	76.03230	1
H	80.45098	71.44376	79.65938	-1775.71861	2062.30353	131.89849	1
H	80.15345	72.67426	78.38463	-1855.14608	88.94118	1993.53760	1
H	80.66531	71.01114	77.92915	-1504.82350	-780.94249	-2132.90992	1
C	81.57340	74.01251	76.79256	288.57638	618.62814	-397.00270	1
O	81.71551	75.22707	77.03963	321.70495	-185.26452	574.37218	1
N	80.61985	73.42328	76.12985	-447.05792	-446.53431	367.76425	1
H	80.73166	72.41204	76.07643	350.31316	1975.63644	-1843.31522	1
C	79.47421	74.06575	75.58777	-511.23061	374.03917	-471.51480	1
H	78.83559	73.19483	75.31916	2234.79579	-898.70890	-1273.45190	1
C	78.84086	74.94889	76.75498	529.53781	-159.34528	563.72604	1
H	77.72953	75.01301	76.60363	654.59388	2347.94251	1217.51488	1
H	79.00248	74.36821	77.70195	-1526.55661	2103.42026	817.87394	1
O	79.32274	76.29555	76.93437	-554.84278	14.12789	399.58769	1
H	80.31055	76.09207	77.08396	1297.06236	2061.84854	1220.68812	1
C	79.86855	74.91675	74.25219	-662.15484	-412.13544	123.62687	1
O	79.24174	74.66156	73.23359	-540.01537	-317.05804	274.91456	1
N	80.79431	75.81869	74.47398	-202.76299	-690.00100	-131.49145	1
H	81.15769	75.88872	75.44552	-702.62132	-2617.37191	281.70413	1
C	81.62691	76.32624	73.43778	-491.73914	576.34187	222.73509	1
H	82.45012	76.81449	74.00494	1977.17978	-1823.58850	-434.68928	1
C	82.13315	75.09908	72.56263	-649.50797	-328.66843	306.11224	1
H	81.27687	74.47043	72.23165	2075.07803	1452.16490	-1004.45904	1
C	82.92555	75.65638	71.33172	-615.18735	-489.99689	-70.96352	1

83	H	83.34410	74.83478	70.70433	-1226.32661	-1724.38906	-1716.47170	1
84	H	82.27085	76.27692	70.67469	2381.64273	-969.84735	900.46860	1
85	H	83.77796	76.30106	71.65068	2122.74991	1702.23946	-141.36228	1
86	C	83.12195	74.26399	73.44697	-501.86074	-479.61276	-376.42482	1
87	H	83.48328	73.35343	72.91450	993.79261	-2246.95401	-1177.81305	1
88	H	84.01959	74.86120	73.73086	42.71279	-626.15391	-2651.37258	1
89	H	82.63372	73.92810	74.39261	-2026.39012	-1590.33709	-887.82922	1
90	C	80.85795	77.41261	72.48073	-672.89153	382.57394	-156.34072	1
91	O	81.36910	78.49453	72.33018	410.04627	221.86223	500.35950	1
92	N	79.76209	76.92914	71.91201	-378.11782	-443.79414	-441.10984	1
93	H	79.41712	75.95805	72.12195	2404.15973	-568.14894	1149.30085	1
94	C	78.92841	77.87421	71.21886	-544.54484	466.16019	331.29119	1
95	H	79.24110	78.85675	71.64585	-1409.80988	-170.17955	2325.32679	1
96	C	79.32725	77.76184	69.68050	-184.48284	116.78282	-758.89137	1
97	H	80.43300	77.83005	69.57155	1893.78641	1861.14652	611.04528	1
98	H	79.04110	76.76787	69.26927	-2170.79665	-974.19311	1327.50117	1
99	C	78.61993	78.91039	68.90148	389.51668	672.81878	-138.49152	1
100	H	77.51377	78.79760	68.98305	510.64577	1933.03327	1851.02484	1
101	H	78.83179	79.88700	69.39037	-2302.30869	-1105.72939	-948.89774	1
102	C	79.02261	78.99658	67.38008	-559.93427	-126.34590	542.30857	1
103	O	78.46587	79.79564	66.67266	-670.36003	-111.83166	76.36448	1
104	N	80.00404	78.14703	67.00957	-216.06729	-537.07037	446.51379	1
105	H	80.52089	77.45686	67.56673	-1251.96487	1074.12894	-2168.52500	1
106	H	80.39267	78.10050	66.06086	-2451.83023	-720.73851	944.84612	1
107	C	77.38666	77.49283	71.48699	546.24052	-563.88337	-85.11809	1
108	O	76.59306	77.50597	70.55320	71.70702	-307.40463	606.69673	1
109	N	77.12206	77.29263	72.77329	479.90863	-504.50129	222.86849	1
110	C	75.92022	76.61012	73.18111	75.51268	-600.91904	506.73700	1
111	H	75.75498	75.80415	72.42811	-2033.61562	1795.30103	254.89480	1
112	C	76.31550	76.14313	74.64797	523.79067	517.99274	284.45581	1
113	H	75.43364	75.86744	75.26989	1599.61665	-1127.00911	1895.98173	1
114	H	76.99294	75.26007	74.62359	-1881.83093	1275.82488	-1501.55016	1
115	C	77.05627	77.42266	75.14382	-600.05639	-404.05026	316.64485	1
116	H	76.33063	78.24408	75.35201	-2597.05260	496.36221	657.73249	1
117	H	77.65250	77.26707	76.07593	-1666.24518	1294.38985	-1723.90591	1
118	C	77.96139	77.74090	73.87700	493.57570	-129.95376	-602.56394	1
119	H	78.21652	78.81488	73.71900	-1469.08415	-637.84797	2204.22581	1
120	H	78.89333	77.13050	73.82575	-1038.97840	1068.88701	-2280.71789	1
121	C	74.72605	77.71374	73.20604	188.88100	241.05089	-727.87821	1
122	O	74.34468	78.22506	74.21975	531.88613	-49.19943	427.07960	1
123	N	74.24068	77.84996	71.94309	708.14833	26.93417	-179.73509	1
124	H	74.86740	77.51815	71.17622	-2232.69501	-1179.09831	1023.94816	1
125	C	73.24736	78.92953	71.71382	-738.32412	259.92548	104.42456	1
126	H	73.67505	79.77490	72.30157	2028.89123	1260.02983	1311.33324	1
127	C	73.17543	79.23345	70.23794	-258.65678	-345.52513	661.28459	1
128	H	72.83749	78.30950	69.71640	-1826.06090	-1801.46097	-918.64554	1
129	C	72.19136	80.34850	69.92260	-541.23400	531.92970	-218.41428	1
130	H	72.18424	80.59996	68.82721	1311.73218	-2219.18200	882.19023	1
131	H	71.13699	80.07377	70.20810	-1048.71298	1223.58118	-2196.97971	1
132	H	72.44747	81.29160	70.47831	419.06414	1903.92898	1903.44670	1
133	C	74.52794	79.62023	69.66440	-423.86431	152.66566	-648.55262	1
134	H	74.48743	79.75398	68.54890	105.68386	-2342.50080	1387.51238	1
135	H	74.90071	80.58663	70.10128	-1695.63603	-1484.73621	-1531.02793	1
136	H	75.31340	78.82525	69.88712	-1853.57121	-487.39031	1936.59343	1
137	C	71.79133	78.48334	72.32999	539.71428	-510.58232	267.58633	1
138	O	70.84824	79.23131	72.20898	-15.93758	-278.34085	-624.49402	1
139	N	71.75058	77.25399	72.94250	-676.81886	12.05292	276.17960	1
140	H	72.71578	76.93738	72.77297	-1694.44250	-373.84189	-2100.66098	1
141	C	71.37271	77.34502	74.35453	412.33078	-664.70360	108.35451	1
142	H	72.14265	78.01700	74.79702	1765.77476	1294.44366	1621.76594	1
143	C	69.94266	77.99211	74.32139	-179.66635	-721.95667	-264.73915	1
144	H	70.03611	79.10325	74.34102	-607.34922	2587.87032	-598.10018	1

H	69.50382	77.76058	73.32467	-359.69332	-1479.13954	-2259.74260	1
C	68.93092	77.55407	75.43420	-240.01076	616.02425	431.85322	1
H	68.97944	76.45923	75.63709	-1359.17131	782.54425	2227.99181	1
H	69.10712	78.08133	76.39765	-820.36667	85.81767	-2596.78715	1
C	67.55990	77.95293	74.75208	-513.07015	236.18570	-551.87280	1
H	67.63653	78.97894	74.32449	1816.11944	-1183.11722	-1650.94321	1
H	67.39473	77.26941	73.88620	-1800.04429	-1361.53473	1526.34643	1
C	66.32645	77.88370	75.67730	726.08972	-253.19732	-179.64713	1
H	66.02316	76.86085	75.97618	1224.27271	-456.51798	-2390.90304	1
H	66.34819	78.56287	76.55341	1206.40339	-1150.93514	-2154.90344	1
N	65.11893	78.39476	74.80900	329.70796	-633.68968	-155.67358	1
H	64.15662	78.31005	75.24740	1348.31305	1938.76845	1359.00643	1
H	65.13297	79.41853	74.53307	2096.92035	1446.54551	-966.48890	1
H	64.96805	77.92423	73.87024	-1197.07217	-2303.53719	827.28733	1
C	71.47195	75.85910	74.98020	-378.01186	-556.17301	-413.96481	1
O	72.30536	75.65163	75.85489	-409.44433	-307.55053	-453.30833	1
N	70.57044	75.05354	74.47067	-429.02477	524.70884	274.08502	1
H	69.87421	75.42993	73.78702	1706.34263	-1425.96036	1574.38926	1
C	70.60708	73.63974	74.58648	-45.25864	-626.22952	478.93267	1
H	69.89276	73.32730	73.79133	1988.63164	570.90773	1772.87287	1
C	72.08690	73.11976	74.36600	551.77128	-20.49526	-564.54996	1
H	72.49518	73.46223	73.38543	1434.61535	-1753.10253	1513.99471	1
H	72.77361	73.53129	75.14333	-714.67738	1819.09770	1898.36494	1
C	71.99187	71.55949	74.43603	446.99263	-555.77305	338.97221	1
H	71.61850	71.23650	75.43541	1953.68466	1892.25957	-161.67766	1
H	71.24791	71.18186	73.69921	-403.03785	2688.07032	-188.43591	1
C	73.41748	70.94730	74.13386	567.86262	335.74681	434.04421	1
H	73.75771	71.23975	73.11096	-1080.02275	1568.26081	-1948.78900	1
H	74.18302	71.34350	74.84029	-919.54108	2111.34692	1456.13463	1
C	73.28816	69.41108	74.26011	195.81897	173.33862	-745.11536	1
H	73.01386	69.05302	75.27260	1405.67949	-2301.21089	-390.08113	1
H	72.63037	68.94064	73.50223	-940.37328	-1118.32446	2299.72133	1
N	74.70105	68.79020	73.99675	319.26422	656.36007	-42.02424	1
H	74.75164	67.73151	74.04354	1961.03633	619.91968	1787.09422	1
H	75.14224	68.97808	73.04976	854.14343	-2156.80752	1429.08711	1
H	75.48153	69.06390	74.66206	1237.02905	-1378.50857	-1998.28500	1
C	70.14416	73.07429	76.06227	532.53319	-268.57236	517.55729	1
O	69.48157	72.07035	76.12505	266.16714	-290.73804	-558.88002	1
N	70.67400	73.79692	77.03694	-226.25352	640.49678	-270.32839	1
H	71.35459	74.56072	76.81081	2007.69063	-1035.27229	1523.50231	1
C	70.43113	73.37316	78.38328	-540.76542	-573.33303	49.50000	1
H	70.58061	72.27004	78.37822	-2374.20950	-1036.76023	843.75774	1
C	71.49248	74.17453	79.26223	-77.09082	666.87422	-415.84136	1
H	71.32204	75.27463	79.10138	-985.81834	617.19825	2463.91927	1
O	72.87003	73.93532	78.89927	293.29899	-391.65602	-477.80926	1
H	73.09778	74.78012	78.46340	2528.72714	-681.76262	-751.27122	1
C	71.10559	73.80298	80.73945	252.47363	-605.14473	440.05134	1
H	71.77639	74.30620	81.47414	-2052.65063	1456.49051	-1043.51830	1
H	70.05989	74.10645	80.98758	-2152.98800	-1661.97574	-161.70652	1
H	71.18391	72.70484	80.91699	-473.12726	-1584.27339	2165.61725	1
C	68.89145	73.73608	78.77686	360.36307	488.46713	505.10056	1
O	68.38904	73.19901	79.74053	-200.08817	-528.03552	-385.82850	1
N	68.37389	74.69524	78.00889	-597.54095	-226.44775	355.20479	1
H	68.94719	75.04081	77.24047	2179.23655	-1300.70981	-991.33835	1
C	67.04274	75.08749	78.28340	-687.68921	297.39807	-249.45179	1
H	66.78332	74.59177	79.25018	-349.16256	-2467.37713	-1101.72831	1
C	66.98611	76.69198	78.30787	672.47345	361.54328	-201.62880	1
H	67.38268	77.09206	77.34599	1541.41502	582.18425	2169.97026	1
H	65.92293	77.01589	78.38756	-2534.67717	60.25153	997.72295	1
C	67.79310	77.25450	79.48266	574.26774	-389.43119	-377.02542	1
C	67.17604	77.34864	80.76725	688.82679	-287.33580	-257.96133	1
H	66.14812	76.97616	80.92806	331.14182	1470.38163	2269.80013	1

C	69.13283	77.71199	79.28926	-383.52382	-675.04808	144.24917	1
H	69.62547	77.61452	78.30584	4.09847	1517.59662	2262.86397	1
C	67.89170	77.93546	81.80960	-134.76730	428.45285	-649.50303	1
H	67.41032	78.01904	82.79974	-903.23433	1600.49020	2011.53387	1
C	69.80453	78.29954	80.36349	-335.53281	-615.00040	-364.39058	1
H	70.83147	78.66938	80.19742	1596.45398	-1369.33862	1732.02659	1
C	69.21505	78.44197	81.65410	585.48547	-340.24194	-406.23938	1
O	69.88682	79.04196	82.75057	-419.15649	-173.04408	-511.94141	1
H	70.72605	79.30882	82.30646	-1071.84350	1559.71507	-1960.13012	1
C	66.02527	74.70155	77.06158	291.41878	-183.13091	710.72246	1
O	64.85141	74.58105	77.32976	379.87695	523.47396	-222.23622	1
N	66.61573	74.74036	75.88695	564.30591	373.09380	277.24371	1
H	67.63679	74.85603	75.77743	-120.59931	-2436.07824	-1214.35098	1
C	65.91923	74.39970	74.66494	404.07514	-299.37480	608.84009	1
H	65.91325	73.28787	74.71756	-1071.07882	-841.69025	2359.66467	1
C	64.45659	74.99275	74.59486	-683.12807	68.71770	-390.13096	1
H	63.96193	74.95468	75.60606	2068.88918	1596.58590	770.89603	1
H	64.49460	76.06942	74.31776	1379.86429	2342.49779	-179.85638	1
C	63.60568	74.18763	73.56137	-176.39967	537.49466	550.97268	1
H	64.12451	74.08036	72.58271	-396.28051	1942.22168	1869.33314	1
H	63.41462	73.14938	73.91311	-94.44700	586.00678	-2659.20016	1
C	62.25035	75.02026	73.41715	-291.69381	-718.18079	-150.72929	1
H	61.60920	75.05674	74.33458	-2303.38776	1089.76242	964.62032	1
H	62.37315	76.07130	73.05009	884.16729	2568.80533	-207.74283	1
N	61.54469	74.31480	72.43344	-594.49522	-252.81478	342.29276	1
H	62.00115	73.50862	72.03591	-1735.17426	2084.05705	-263.71605	1
C	60.38454	74.62855	72.01936	595.23177	-271.52487	442.22352	1
N	59.77489	75.75324	72.58705	585.53813	-424.79750	-105.81303	1
H	60.12088	76.38011	73.31241	959.88797	1327.56823	-2177.11905	1
H	58.84703	76.10049	72.34280	1578.48647	16.27378	-2220.76233	1
N	59.94174	73.73781	71.03165	-452.17763	-390.33541	-421.51853	1
H	60.38738	72.91972	70.61887	470.26107	1974.25629	-1817.92087	1
H	59.03337	73.82103	70.57340	1856.67307	-1573.97321	-1224.35416	1
C	66.97523	74.98852	73.60321	-531.47271	-581.45780	-55.06206	1
O	68.14923	75.11472	73.98632	-243.00837	192.73507	609.52485	1
N	66.45658	75.32436	72.44140	343.49288	-644.25607	38.09164	1
H	65.45462	75.17230	72.32411	-1691.16043	-1535.97525	-1484.73099	1
C	67.26776	75.37225	71.26643	542.66492	350.14337	454.42563	1
H	66.52291	75.60426	70.47350	1209.57979	-2396.95920	463.86744	1
C	68.34594	76.52399	71.43785	-737.14309	145.93010	242.71942	1
H	68.93514	76.34130	72.36396	-1050.18303	159.36419	-2509.06125	1
C	69.29178	76.70883	70.20196	-706.77696	-252.88343	-245.16230	1
H	70.00926	77.54723	70.37203	-1624.64039	-1665.49085	1417.86935	1
H	69.90094	75.79876	69.98983	2566.10521	598.51109	693.22305	1
H	68.71649	76.94287	69.27600	-1972.69758	107.10018	-1876.34312	1
C	67.58208	77.89234	71.51024	412.85148	672.58832	-27.69222	1
H	68.27076	78.73350	71.76237	1447.02277	1762.76533	-1490.78944	1
H	67.09872	78.14147	70.53708	1320.77379	-145.20455	2378.68491	1
H	66.77442	77.87461	72.27808	-1512.10848	1470.52031	1724.75147	1
C	67.83230	73.84576	71.10548	-607.11113	499.56689	73.73769	1
O	67.02153	72.95695	70.94990	357.97126	460.81534	356.68785	1
N	69.14719	73.78453	71.09344	536.55407	-368.62270	332.76306	1
H	69.74974	74.60254	71.26779	524.38866	2297.69985	1367.20941	1
C	69.81668	72.51615	71.20086	46.50277	-774.29190	-147.97904	1
H	69.39286	72.03261	72.10822	1676.51875	1883.29066	1032.55549	1
C	69.59654	71.60417	69.88801	-559.41456	-34.19198	-556.30433	1
H	69.35725	72.26135	69.01839	-64.66664	395.18769	2695.05373	1
H	70.53947	71.06917	69.62172	-2703.44278	-60.40056	333.79418	1
C	68.49466	70.61099	70.13047	-519.76435	377.31045	459.42366	1
H	67.56994	71.18321	70.44892	-1746.74323	1667.50644	-1261.73691	1
C	68.18402	69.84907	68.84790	570.95554	16.46261	-545.27700	1
H	67.34258	69.12299	68.99097	2391.58273	28.05277	-1305.07271	1

```
H    67.88877  70.54003  68.01659       175.75588       917.63458      2559.43867         1
H    69.07038  69.26260  68.49277     -1063.97851     -2155.34650      1283.00462         1
C    68.85953  69.61882  71.22604       193.76014         2.62946      -765.53141         1
H    68.06571  68.83828  71.35398       507.39394      -846.55087      2539.60089         1
H    69.81684  69.08387  70.99662      1337.57841      1818.19054      1526.01994         1
H    68.98914  70.11893  72.21994      1100.88635     -1172.91350     -2199.08909         1
C    71.33018  72.98479  71.33800      -549.21115       475.64486      -309.38218         1
O    71.61324  74.16397  71.39673       556.06106      -390.37755       -78.24250         1
O    72.33748  72.00157  71.42234      -572.27940      -310.19032      -209.75862         1
H    71.76226  71.18617  71.34912     -1380.08193      2212.14486       790.85944         1
```

Listing 41: C00041.xmolout

```
      276
  150.0000  150.0000  150.0000     0.0000     0.0000     0.0000***********    2.7750    11100
       0.2500     300.00    0.00    0.00    0.00   -1293.7837       0.0000    300.0000
H    72.71198  65.91386  64.16711     -2043.14101     -1757.64893       399.89339         1
N    73.60896  65.85551  64.64405       426.75047       490.45733      -334.42467         1
H    73.35058  66.04976  65.60924       442.25559     -1110.30854     -2448.52907         1
C    74.62171  66.68077  64.11745       415.32972      -666.39213        83.73172         1
H    74.45128  66.65369  63.02074       879.32219      2491.00447       667.35213         1
C    74.45543  68.16569  64.71505        31.35357       477.41777       628.23362         1
H    74.55820  68.13076  65.82741      1168.38489      2240.25337     -1019.71136         1
H    75.29152  68.81099  64.35246      -360.43770      2534.90498       931.67112         1
C    73.12189  68.74903  64.32009       463.18208       512.70242      -382.34388         1
H    72.31925  68.03524  64.61074      1725.29117      1863.52247      -987.07854         1
C    72.87695  70.06301  65.05112       710.42801      -144.27427      -313.15367         1
H    71.88321  70.50610  64.78181       364.20195     -1033.13725      2494.72524         1
H    72.89165  69.92828  66.16359      2567.61904       335.80679       847.48802         1
H    73.65799  70.82569  64.79815     -2587.99029       680.92867      -512.16411         1
C    73.04140  68.98472  62.81804       268.17616       741.64379       -40.42634         1
H    72.06950  69.46017  62.52565      1283.54314     -1945.37420     -1411.27812         1
H    73.86085  69.66220  62.46369     -1258.57796      1317.15249     -2026.02085         1
H    73.12816  68.02928  62.23957     -2502.84526       909.80597       575.92391         1
C    76.10806  66.15996  64.52750       619.36578      -458.64843      -172.09273         1
O    77.06634  66.84759  64.19953       -81.40433       433.87611       522.34594         1
N    76.08594  65.01418  65.17989       175.23996      -622.20190       341.55523         1
H    75.13578  64.66800  65.31052      -876.57256      -303.22879     -2561.90213         1
C    77.18226  64.18303  65.53632      -571.88214      -534.10783      -106.15033         1
H    76.72386  63.17120  65.47710     -2713.20841       -16.25800      -248.80809         1
C    78.39827  64.34289  64.51446       166.60354       288.16399      -716.09568         1
H    78.72831  65.41968  64.47711     -2097.85574      1636.78449      -586.18044         1
O    78.10161  64.00119  63.14377      -317.80580      -363.38227       484.43082         1
H    78.07087  64.89372  62.74228      1863.74748     -1451.84923      1357.29467         1
C    79.50083  63.47496  65.22322       503.80731       517.99032       318.51606         1
H    80.48191  63.55359  64.70018       640.61020     -2157.10410     -1536.29104         1
H    79.66009  63.78594  66.28241      1687.88455       465.60692      2087.56452         1
H    79.21834  62.39660  65.23720     -1293.07895     -1899.88581     -1463.57452         1
C    77.50601  64.40311  67.12977      -153.07550      -733.68902       248.70981         1
O    77.38427  63.40168  67.78671       186.15459      -480.78787      -449.34387         1
N    77.94955  65.60625  67.51674      -487.53139       543.18517        42.03850         1
C    77.71907  66.07920  68.85064      -699.35871        69.34393       360.10701         1
H    77.73789  65.20469  69.54092         2.81126      -221.99807     -2715.58072         1
C    78.89402  67.14049  69.01931      -507.46439      -592.59194       122.07761         1
H    78.70480  67.87247  69.83805     -1975.08116       525.87560     -1801.71556         1
H    79.86535  66.64790  69.24804      1588.27743     -1913.22100      1113.83566         1
C    78.89996  67.78732  67.60095      -613.62442      -457.55993      -194.14490         1
H    78.03121  68.47726  67.48154      1707.54637      -721.27992      1996.92523         1
H    79.81896  68.37506  67.37680       758.23357      2100.81767      1560.55011         1
C    78.73156  66.50442  66.68009      -352.41524       704.96076        49.21465         1
H    78.13103  66.67092  65.72710      1714.76886     -2070.42778       443.35792         1
H    79.68054  65.96967  66.43793      1224.15040      1801.50881      1636.97633         1
```

C	76.30413	66.89123	68.85240	566.85117	537.05831	117.62814	1
O	75.96297	67.60632	67.91338	-550.26779	-406.08867	4.21666	1
N	75.70964	66.71307	70.00837	566.34832	-425.77725	180.18749	1
H	76.15722	66.10907	70.69323	644.44502	2646.80550	-52.73430	1
C	74.40549	67.27199	70.20443	612.34801	-281.48662	411.56221	1
H	73.99223	67.22973	69.16973	1667.23498	-1490.75142	1556.16753	1
C	73.63440	66.37270	71.27641	-714.45746	-175.43074	286.98927	1
H	74.11206	66.52466	72.28192	-787.09484	1763.03619	-1922.46060	1
O	73.64621	64.95805	71.01656	-429.46514	516.51932	-128.40526	1
H	74.10858	64.62110	71.80918	1981.82785	436.16847	-1818.18182	1
C	72.21048	67.04138	71.27020	630.79189	-468.94448	76.03230	1
H	71.53658	66.56775	72.02096	-1775.71861	2062.30353	131.89849	1
H	72.26132	68.13078	71.50896	-1855.14608	88.94118	1993.53760	1
H	71.71801	66.93945	70.27534	-1504.82350	-780.94249	-2132.90992	1
C	74.61910	68.80994	70.70443	288.57638	618.62814	-397.00270	1
O	74.26750	69.17622	71.81896	321.70495	-185.26452	574.37218	1
N	75.08813	69.47978	69.68916	-447.05792	-446.53431	367.76425	1
H	75.28090	68.95399	68.80185	350.31316	1975.63644	-1843.31522	1
C	75.72353	70.75538	69.80824	-511.23061	374.03917	-471.51480	1
H	75.86806	71.04392	68.73990	2234.79579	-898.70890	-1273.45190	1
C	74.69930	71.74763	70.56218	529.53781	-159.34528	563.72604	1
H	74.90704	72.80053	70.22366	654.59388	2347.94251	1217.51488	1
H	73.68610	71.47532	70.16222	-1526.55661	2103.42026	817.87394	1
O	74.71248	71.74867	71.98482	-554.84278	14.12789	399.58769	1
H	74.48995	70.74895	72.15120	1297.06236	2061.84854	1220.68812	1
C	77.11791	70.63974	70.56963	-662.15484	-412.13544	123.62687	1
O	77.52160	69.71851	71.38578	-540.01537	-317.05804	274.91456	1
N	77.84053	71.67721	70.28542	-202.76299	-690.00100	-131.49145	1
H	77.45369	72.32941	69.60992	-702.62132	-2617.37191	281.70413	1
C	79.23124	71.48992	70.60734	-491.73914	576.34187	222.73509	1
H	79.63310	70.98472	69.70513	1977.17978	-1823.58850	-434.68928	1
C	79.85833	72.91287	70.81272	-649.50797	-328.66843	306.11224	1
H	79.19181	73.53253	71.45165	2075.07803	1452.16490	-1004.45904	1
C	81.27666	72.76155	71.47103	-615.18735	-489.99689	-70.96352	1
H	81.75628	73.75274	71.64639	-1226.32661	-1724.38906	-1716.47170	1
H	81.20900	72.24345	72.45699	2381.64273	-969.84735	900.46860	1
H	81.96645	72.16471	70.83039	2122.74991	1702.23946	-141.36228	1
C	80.05493	73.58780	69.40658	-501.86074	-479.61276	-376.42482	1
H	80.47471	74.61609	69.49983	993.79261	-2246.95401	-1177.81305	1
H	80.75324	73.00135	68.76554	42.71279	-626.15391	-2651.37258	1
H	79.09055	73.67204	68.85415	-2026.39012	-1590.33709	-887.82922	1
C	79.20977	70.44164	71.91925	-672.89153	382.57394	-156.34072	1
O	80.04738	69.57528	72.04134	410.04627	221.86223	500.35950	1
N	78.95640	71.37971	73.00339	-378.11782	-443.79414	-441.10984	1
H	78.09960	71.92714	72.87456	2404.15973	-568.14894	1149.30085	1
C	79.00717	70.76035	74.29972	-544.54484	466.16019	331.29119	1
H	79.72680	69.91313	74.24417	-1409.80988	-170.17955	2325.32679	1
C	79.38859	71.90494	75.33296	-184.48284	116.78282	-758.89137	1
H	80.40065	72.32338	75.13285	1893.78641	1861.14652	611.04528	1
H	78.67831	72.75614	75.22839	-2170.79665	-974.19311	1327.50117	1
C	79.29146	71.28011	76.75898	389.51668	672.81878	-138.49152	1
H	78.43029	70.57813	76.81476	510.64577	1933.03327	1851.02484	1
H	80.18213	70.64541	76.96048	-2302.30869	-1105.72939	-948.89774	1
C	79.10607	72.35194	77.89996	-559.93427	-126.34590	542.30857	1
O	78.63807	72.00996	78.95773	-670.36003	-111.83166	76.36448	1
N	79.51720	73.59015	77.55955	-216.06729	-537.07037	446.51379	1
H	79.98537	73.88940	76.69581	-1251.96487	1074.12894	-2168.52500	1
H	79.55501	74.38444	78.20798	-2451.83023	-720.73851	944.84612	1
C	77.49108	70.37738	74.71831	546.24052	-563.88337	-85.11809	1
O	76.67560	71.28510	74.78388	71.70702	-307.40463	606.69673	1
N	77.37840	69.11261	75.10384	479.90863	-504.50129	222.86849	1
C	76.10100	68.47084	75.27699	75.51268	-600.91904	506.73700	1

H	75.45859	68.86486	74.45426	-2033.61562	1795.30103	254.89480	1
C	76.47959	66.93196	75.18211	523.79067	517.99274	284.45581	1
H	75.73095	66.27684	75.68218	1599.61665	-1127.00911	1895.98173	1
H	76.55611	66.59099	74.12526	-1881.83093	1275.82488	-1501.55016	1
C	77.87292	66.92028	75.88466	-600.05639	-404.05026	316.64485	1
H	77.76516	67.01461	76.98864	-2597.05260	496.36221	657.73249	1
H	78.46473	65.99605	75.69779	-1666.24518	1294.38985	-1723.90591	1
C	78.52509	68.22249	75.25053	493.57570	-129.95376	-602.56394	1
H	79.29402	68.72593	75.88231	-1469.08415	-637.84797	2204.22581	1
H	78.92653	68.07962	74.21855	-1038.97840	1068.88701	-2280.71789	1
C	75.49265	68.77774	76.75258	188.88100	241.05089	-727.87821	1
O	74.98589	67.90197	77.39551	531.88613	-49.19943	427.07960	1
N	75.67406	70.06984	77.07364	708.14833	26.93417	-179.73509	1
H	76.00963	70.69510	76.29993	-2232.69501	-1179.09831	1023.94816	1
C	74.87322	70.67973	78.14855	-738.32412	259.92548	104.42456	1
H	73.90378	70.13090	78.14099	2028.89123	1260.02983	1311.33324	1
C	75.61752	70.57447	79.46135	-258.65678	-345.52513	661.28459	1
H	76.68564	70.85196	79.28924	-1826.06090	-1801.46097	-918.64554	1
C	75.04789	71.52345	80.49726	-541.23400	531.92970	-218.41428	1
H	75.56070	71.40743	81.49144	1311.73218	-2219.18200	882.19023	1
H	75.16743	72.62705	80.18237	-1048.71298	1223.58118	-2196.97971	1
H	73.95048	71.34251	80.66555	419.06414	1903.92898	1903.44670	1
C	75.59909	69.15888	80.01197	-423.86431	152.66566	-648.55262	1
H	76.12887	69.08730	81.00154	105.68386	-2342.50080	1387.51238	1
H	74.54784	68.78900	80.16656	-1695.63603	-1484.73621	-1531.02793	1
H	76.10184	68.43746	79.30853	-1853.57121	-487.39031	1936.59343	1
C	74.76332	72.25984	77.65815	539.71428	-510.58232	267.58633	1
O	73.69333	72.71855	77.33439	-15.93758	-278.34085	-624.49402	1
N	75.95842	72.81910	77.64405	-676.81886	12.05292	276.17960	1
H	76.75500	72.24783	77.92842	-1694.44250	-373.84189	-2100.66098	1
C	76.13603	74.20901	77.40575	412.33078	-664.70360	108.35451	1
H	77.21948	74.29420	77.16142	1765.77476	1294.44366	1621.76594	1
C	75.17414	74.56893	76.19549	-179.66635	-721.95667	-264.73915	1
H	75.22806	73.75844	75.43138	-607.34922	2587.87032	-598.10018	1
H	74.10361	74.60026	76.52651	-359.69332	-1479.13954	-2259.74260	1
C	75.59543	75.94428	75.58047	-240.01076	616.02425	431.85322	1
H	75.22971	76.78404	76.22047	-1359.17131	782.54425	2227.99181	1
H	76.70475	76.03444	75.53691	-820.36667	85.81767	-2596.78715	1
C	74.98428	76.06272	74.12873	-513.07015	236.18570	-551.87280	1
H	75.33166	75.22930	73.47492	1816.11944	-1183.11722	-1650.94321	1
H	73.87252	75.98721	74.15445	-1800.04429	-1361.53473	1526.34643	1
C	75.43502	77.43736	73.57295	726.08972	-253.19732	-179.64713	1
H	74.99605	78.30980	74.09887	1224.27271	-456.51798	-2390.90304	1
H	76.53292	77.56438	73.47971	1206.40339	-1150.93514	-2154.90344	1
N	74.89328	77.54230	72.11893	329.70796	-633.68968	-155.67358	1
H	75.11308	78.45427	71.61843	1348.31305	1938.76845	1359.00643	1
H	75.27067	76.83803	71.41870	2096.92035	1446.54551	-966.48890	1
H	73.81519	77.45429	72.02864	-1197.07217	-2303.53719	827.28733	1
C	75.76567	75.00816	78.78491	-378.01186	-556.17301	-413.96481	1
O	75.38416	74.48274	79.81347	-409.44433	-307.55053	-453.30833	1
N	76.07136	76.28255	78.57461	-429.02477	524.70884	274.08502	1
H	76.38475	76.50972	77.63415	1706.34263	-1425.96036	1574.38926	1
C	75.44137	77.27501	79.38568	-45.25864	-626.22952	478.93267	1
H	75.44264	76.86811	80.42221	1988.63164	570.90773	1772.87287	1
C	76.29149	78.58451	79.15617	551.77128	-20.49526	-564.54996	1
H	77.37930	78.34947	79.17566	1434.61535	-1753.10253	1513.99471	1
H	76.09356	78.99735	78.14095	-714.67738	1819.09770	1898.36494	1
C	75.93629	79.64407	80.25224	446.99263	-555.77305	338.97221	1
H	74.84488	79.88470	80.25162	1953.68466	1892.25957	-161.67766	1
H	76.18200	79.26992	81.27167	-403.03785	2688.07032	-188.43591	1
C	76.79236	80.91916	79.87779	567.86262	335.74681	434.04421	1
H	77.86286	80.64827	79.72743	-1080.02275	1568.26081	-1948.78900	1

H	76.44580	81.34409	78.90497	-919.54108	2111.34692	1456.13463	1
C	76.65429	81.97217	81.00157	195.81897	173.33862	-745.11536	1
H	75.61253	82.29098	81.20425	1405.67949	-2301.21089	-390.08113	1
H	77.16793	81.71644	81.95043	-940.37328	-1118.32446	2299.72133	1
N	77.38061	83.25083	80.46455	319.26422	656.36007	-42.02424	1
H	77.40924	84.10493	81.09344	1961.03633	619.91968	1787.09422	1
H	78.40207	83.15315	80.19426	854.14343	-2156.80752	1429.08711	1
H	76.95739	83.65167	79.57752	1237.02905	-1378.50857	-1998.28500	1
C	73.92836	77.55491	78.80229	532.53319	-268.57236	517.55729	1
O	73.63380	77.45199	77.63495	266.16714	-290.73804	-558.88002	1
N	73.19331	77.97738	79.82241	-226.25352	640.49678	-270.32839	1
H	73.64488	78.04873	80.73210	2007.69063	-1035.27229	1523.50231	1
C	71.90686	78.54016	79.57578	-540.76542	-573.33303	49.50000	1
H	71.44730	77.92572	78.76903	-2374.20950	-1036.76023	843.75774	1
C	71.13412	78.53864	80.97665	-77.09082	666.87422	-415.84136	1
H	71.75787	79.11326	81.71548	-985.81834	617.19825	2463.91927	1
O	70.91248	77.24588	81.56866	293.29899	-391.65602	-477.80926	1
H	71.45453	77.31146	82.37982	2528.72714	-681.76262	-751.27122	1
C	69.83781	79.36767	80.65051	252.47363	-605.14473	440.05134	1
H	69.23342	79.55964	81.56700	-2052.65063	1456.49051	-1043.51830	1
H	70.08068	80.35858	80.20099	-2152.98800	-1661.97574	-161.70652	1
H	69.18205	78.82843	79.92866	-473.12726	-1584.27339	2165.61725	1
C	72.12683	80.09821	79.14269	360.36307	488.46713	505.10056	1
O	72.89300	80.83505	79.76134	-200.08817	-528.03552	-385.82850	1
N	71.24234	80.38817	78.21828	-597.54095	-226.44775	355.20479	1
H	70.67178	79.61359	77.88479	2179.23655	-1300.70981	-991.33835	1
C	71.36195	81.57866	77.43371	-687.68921	297.39807	-249.45179	1
H	72.44687	81.64804	77.19607	-349.16256	-2467.37713	-1101.72831	1
C	70.36502	81.48492	76.19521	672.47345	361.54328	-201.62880	1
H	69.34323	81.22671	76.55663	1541.41502	582.18425	2169.97026	1
H	70.28961	82.49212	75.72071	-2534.67717	60.25153	997.72295	1
C	70.84709	80.43825	75.17079	574.26774	-389.43119	-377.02542	1
C	71.85311	80.79741	74.22898	688.82679	-287.33580	-257.96133	1
H	72.31482	81.80046	74.25030	331.14182	1470.38163	2269.80013	1
C	70.26965	79.13627	75.15500	-383.52382	-675.04808	144.24917	1
H	69.49964	78.85136	75.89323	4.09847	1517.59662	2262.86397	1
C	72.23283	79.84933	73.26448	-134.76730	428.45285	-649.50303	1
H	73.00707	80.12407	72.52535	-903.23433	1600.49020	2011.53387	1
C	70.69000	78.22544	74.16883	-335.53281	-615.00040	-364.39058	1
H	70.23587	77.21784	74.15476	1596.45398	-1369.33862	1732.02659	1
C	71.66437	78.55414	73.19556	585.48547	-340.24194	-406.23938	1
O	72.08853	77.61165	72.14487	-419.15649	-173.04408	-511.94141	1
H	71.59526	76.82993	72.45798	-1071.84350	1559.71507	-1960.13012	1
C	70.80585	82.87017	78.24304	291.41878	-183.13091	710.72246	1
O	69.89406	83.51637	77.78216	379.87695	523.47396	-222.23622	1
N	71.53802	83.12056	79.33669	564.30591	373.09380	277.24371	1
H	72.28072	82.42973	79.63091	-120.59931	-2436.07824	-1214.35098	1
C	70.87664	83.93573	80.32089	404.07514	-299.37480	608.84009	1
H	70.27136	84.64338	79.70742	-1071.07882	-841.69025	2359.66467	1
C	69.93326	82.97944	81.16506	-683.12807	68.71770	-390.13096	1
H	69.30055	82.35737	80.49293	2068.88918	1596.58590	770.89603	1
H	70.53512	82.26058	81.76510	1379.86429	2342.49779	-179.85638	1
C	69.05393	83.88224	82.08367	-176.39967	537.49466	550.97268	1
H	69.70601	84.54044	82.71619	-396.28051	1942.22168	1869.33314	1
H	68.42852	84.57418	81.47561	-94.44700	586.00678	-2659.20016	1
C	68.13762	82.94638	82.99711	-291.69381	-718.18079	-150.72929	1
H	67.32105	82.39061	82.46955	-2303.38776	1089.76242	964.62032	1
H	68.68301	82.22326	83.65566	884.16729	2568.80533	-207.74283	1
N	67.55484	83.92567	83.81905	-594.49522	-252.81478	342.29276	1
H	67.84869	84.87889	83.66903	-1735.17426	2084.05705	-263.71605	1
C	66.70069	83.69627	84.73289	595.23177	-271.52487	442.22352	1
N	66.30122	82.37130	84.93440	585.53813	-424.79750	-105.81303	1

```
H    66.59786   81.52676   84.44694       959.88797      1327.56823     -2177.11905          1
H    65.62613   82.06331   85.63501      1578.48647        16.27378     -2220.76233          1
N    66.32582   84.88540   85.37404      -452.17763      -390.33541      -421.51853          1
H    66.63173   85.84631   85.22817       470.26107      1974.25629     -1817.92087          1
H    65.65799   84.92031   86.14492      1856.67307     -1573.97321     -1224.35416          1
C    71.96969   84.66163   81.27189      -531.47271      -581.45780       -55.06206          1
O    71.63772   85.14314   82.33705      -243.00837       192.73507       609.52485          1
N    73.19737   84.66185   80.73595       343.49288      -644.25607        38.09164          1
H    73.26383   84.25455   79.80232     -1691.16043     -1535.97525     -1484.73099          1
C    74.02635   85.76759   81.12714       542.66492       350.14337       454.42563          1
H    73.34126   86.49143   81.62545      1209.57979     -2396.95920       463.86744          1
C    75.11043   85.17004   82.12317      -737.14309       145.93010       242.71942          1
H    75.47214   84.19084   81.74812     -1050.18303       159.36419     -2509.06125          1
C    76.29558   86.18155   82.28571      -706.77696      -252.88343      -245.16230          1
H    77.04838   85.81844   83.02409     -1624.64039     -1665.49085      1417.86935          1
H    76.82828   86.34207   81.31892      2566.10521       598.51109       693.22305          1
H    75.93773   87.17829   82.63501     -1972.69758       107.10018     -1876.34312          1
C    74.42896   85.00152   83.52409       412.85148       672.58832       -27.69222          1
H    75.10143   84.49313   84.25435      1447.02277      1762.76533     -1490.78944          1
H    74.14398   85.98718   83.96128      1320.77379      -145.20455      2378.68491          1
H    73.49256   84.39606   83.44476     -1512.10848      1470.52031      1724.75147          1
C    74.74951   86.33057   79.77630      -607.11113       499.56689        73.73769          1
O    74.75743   87.51417   79.54537       357.97126       460.81534       356.68785          1
N    75.35646   85.33756   79.13276       536.55407      -368.62270       332.76306          1
H    75.23233   84.35280   79.42328       524.38866      2297.69985      1367.20941          1
C    75.80731   85.55496   77.79222        46.50277      -774.29190      -147.97904          1
H    75.12852   86.31156   77.34002      1676.51875      1883.29066      1032.55549          1
C    77.35149   86.03308   77.77458      -559.41456       -34.19198      -556.30433          1
H    77.97140   85.29421   78.33485       -64.66664       395.18769      2695.05373          1
H    77.74265   86.03120   76.72963     -2703.44278       -60.40056       333.79418          1
C    77.49289   87.39865   78.38556      -519.76435       377.31045       459.42366          1
H    77.03391   87.38113   79.40159     -1746.74323      1667.50644     -1261.73691          1
C    78.96501   87.76852   78.52452       570.95554        16.46261      -545.27700          1
H    79.09451   88.77838   78.99191      2391.58273        28.05277     -1305.07271          1
H    79.51838   87.03325   79.16327       175.75588       917.63458      2559.43867          1
H    79.47833   87.79598   77.52933     -1063.97851     -2155.34650      1283.00462          1
C    76.77402   88.45735   77.55973       193.76014         2.62946      -765.53141          1
H    76.99081   89.49072   77.93432       507.39394      -846.55087      2539.60089          1
H    77.07665   88.42226   76.48151      1337.57841      1818.19054      1526.01994          1
H    75.66062   88.31997   77.60637      1100.88635     -1172.91350     -2199.08909          1
C    75.68600   84.06886   77.22397      -549.21115       475.64486      -309.38218          1
O    75.40572   83.15250   77.97202       556.06106      -390.37755       -78.24250          1
O    75.96161   83.83529   75.86793      -572.27940      -310.19032      -209.75862          1
H    76.15301   84.77823   75.58271     -1380.08193      2212.14486       790.85944          1
```

Listing 42: C00042.xmolout

```
       276
  150.0000  150.0000  150.0000     0.0000     0.0000     0.0000***********    2.7750    11100
       0.2500    300.00   0.00    0.00   0.00  -1293.7837      0.0000    300.0000
H    80.35535   77.07638   75.71892     -2043.14101     -1757.64893       399.89339          1
N    80.21278   78.08263   75.65226       426.75047       490.45733      -334.42467          1
H    79.46964   78.14117   74.95902       442.25559     -1110.30854     -2448.52907          1
C    81.36687   78.84714   75.38252       415.32972      -666.39213        83.73172          1
H    82.11386   78.44032   76.09563       879.32219      2491.00447       667.35213          1
C    81.83344   78.67083   73.85281        31.35357       477.41777       628.23362          1
H    80.99527   78.94382   73.16735      1168.38489      2240.25337     -1019.71136          1
H    82.65371   79.39730   73.63562      -360.43770      2534.90498       931.67112          1
C    82.29608   77.26173   73.58656       463.18208       512.70242      -382.34388          1
H    81.44036   76.57259   73.75876      1725.29117      1863.52247      -987.07854          1
C    82.75561   77.12601   72.13945       710.42801      -144.27427      -313.15367          1
H    83.14485   76.09755   71.92713       364.20195     -1033.13725      2494.72524          1
```

H	81.92274	77.32658	71.41750	2567.61904	335.80679	847.48802	1
H	83.58167	77.84463	71.90088	-2587.99029	680.92867	-512.16411	1
C	83.44115	76.85209	74.50303	268.17616	741.64379	-40.42634	1
H	83.83198	75.83395	74.24495	1283.54314	-1945.37420	-1411.27812	1
H	84.30211	77.56582	74.42880	-1258.57796	1317.15249	-2026.02085	1
H	83.12709	76.82024	75.57764	-2502.84526	909.80597	575.92391	1
C	81.02553	80.41176	75.64625	619.36578	-458.64843	-172.09273	1
O	81.54218	81.28769	74.98627	-81.40433	433.87611	522.34594	1
N	80.20577	80.54397	76.68986	175.23996	-622.20190	341.55523	1
H	79.90510	79.69330	77.16314	-876.57256	-303.22879	-2561.90213	1
C	79.80644	81.85978	77.07061	-571.88214	-534.10783	-106.15033	1
H	80.00577	82.49482	76.17464	-2713.20841	-16.25800	-248.80809	1
C	78.26054	81.72829	77.49462	166.60354	288.16399	-716.09568	1
H	78.18627	80.91037	78.26667	-2097.85574	1636.78449	-586.18044	1
O	77.35907	81.33898	76.44599	-317.80580	-363.38227	484.43082	1
H	77.07204	80.45815	76.76189	1863.74748	-1451.84923	1357.29467	1
C	77.96582	83.11548	78.17155	503.80731	517.99032	318.51606	1
H	76.96581	83.12553	78.66354	640.61020	-2157.10410	-1536.29104	1
H	78.72292	83.37326	78.95137	1687.88455	465.60692	2087.56452	1
H	77.96682	83.94072	77.42277	-1293.07895	-1899.88581	-1463.57452	1
C	80.67727	82.26325	78.36814	-153.07550	-733.68902	248.70981	1
O	80.64813	81.55036	79.37192	186.15459	-480.78787	-449.34387	1
N	81.22491	83.46080	78.22468	-487.53139	543.18517	42.03850	1
C	82.06744	83.99564	79.26496	-699.35871	69.34393	360.10701	1
H	82.84243	83.23040	79.49022	2.81126	-221.99807	-2715.58072	1
C	82.59622	85.34142	78.62473	-507.46439	-592.59194	122.07761	1
H	82.90469	86.09112	79.38832	-1975.08116	525.87560	-1801.71556	1
H	83.47805	85.17055	77.96759	1588.27743	-1913.22100	1113.83566	1
C	81.34213	85.77500	77.80257	-613.62442	-457.55993	-194.14490	1
H	80.53513	86.13593	78.48332	1707.54637	-721.27992	1996.92523	1
H	81.54157	86.58466	77.06513	758.23357	2100.81767	1560.55011	1
C	80.94766	84.38978	77.13437	-352.41524	704.96076	49.21465	1
H	79.88069	84.29802	76.82891	1714.76886	-2070.42778	443.35792	1
H	81.60895	84.07877	76.29070	1224.15040	1801.50881	1636.97633	1
C	81.07811	84.29756	80.54328	566.85117	537.05831	117.62814	1
O	80.10728	85.00490	80.42514	-550.26779	-406.08867	4.21666	1
N	81.57675	83.68505	81.60615	566.34832	-425.77725	180.18749	1
H	82.43766	83.15177	81.48523	644.44502	2646.80550	-52.73430	1
C	81.01542	83.82878	82.90241	612.34801	-281.48662	411.56221	1
H	81.77867	83.35556	83.56112	1667.23498	-1490.75142	1556.16753	1
C	80.87967	85.40219	83.19110	-714.45746	-175.43074	286.98927	1
H	80.05601	85.80150	82.53530	-787.09484	1763.03619	-1922.46060	1
O	82.05108	86.18714	82.89840	-429.46514	516.51932	-128.40526	1
H	81.72982	86.70962	82.13551	1981.82785	436.16847	-1818.18182	1
C	80.42153	85.46723	84.69232	630.79189	-468.94448	76.03230	1
H	80.17852	86.51222	84.99760	-1775.71861	2062.30353	131.89849	1
H	79.50689	84.83791	84.87880	-1855.14608	88.94118	1993.53760	1
H	81.22350	85.10472	85.37715	-1504.82350	-780.94249	-2132.90992	1
C	79.60851	83.01669	82.93652	288.57638	618.62814	-397.00270	1
O	78.79963	83.29019	83.79933	321.70495	-185.26452	574.37218	1
N	79.50868	82.07197	82.00504	-447.05792	-446.53431	367.76425	1
H	80.25540	81.91974	81.31342	350.31316	1975.63644	-1843.31522	1
C	78.48349	81.10205	82.21765	-511.23061	374.03917	-471.51480	1
H	77.69769	81.65963	82.78156	2234.79579	-898.70890	-1273.45190	1
C	78.02091	80.59484	80.77406	529.53781	-159.34528	563.72604	1
H	77.03210	80.06936	80.89609	654.59388	2347.94251	1217.51488	1
H	77.82870	81.53591	80.19490	-1526.55661	2103.42026	817.87394	1
O	78.87259	79.68662	80.06474	-554.84278	14.12789	399.58769	1
H	79.60896	80.33693	79.77916	1297.06236	2061.84854	1220.68812	1
C	79.07992	79.85795	83.08352	-662.15484	-412.13544	123.62687	1
O	80.24820	79.58579	83.20403	-540.01537	-317.05804	274.91456	1
N	78.02588	79.23624	83.67762	-202.76299	-690.00100	-131.49145	1

H	77.15457	79.62846	83.31418	-702.62132	-2617.37191	281.70413	1
C	78.10244	77.79057	83.91486	-491.73914	576.34187	222.73509	1
H	79.17309	77.58034	84.14522	1977.17978	-1823.58850	-434.68928	1
C	77.13299	77.46330	85.03172	-649.50797	-328.66843	306.11224	1
H	76.13154	77.84558	84.72982	2075.07803	1452.16490	-1004.45904	1
C	77.01167	75.96788	85.26266	-615.18735	-489.99689	-70.96352	1
H	76.27283	75.73004	86.07637	-1226.32661	-1724.38906	-1716.47170	1
H	76.66684	75.43416	84.33295	2381.64273	-969.84735	900.46860	1
H	77.99560	75.51260	85.56117	2122.74991	1702.23946	-141.36228	1
C	77.51563	78.15634	86.32763	-501.86074	-479.61276	-376.42482	1
H	76.75530	77.97888	87.13715	993.79261	-2246.95401	-1177.81305	1
H	78.50694	77.79180	86.71366	42.71279	-626.15391	-2651.37258	1
H	77.60506	79.26858	86.18409	-2026.39012	-1590.33709	-887.82922	1
C	77.54808	77.06093	82.52977	-672.89153	382.57394	-156.34072	1
O	76.68033	77.55494	81.85188	410.04627	221.86223	500.35950	1
N	78.00325	75.77561	82.47375	-378.11782	-443.79414	-441.10984	1
H	78.81171	75.81581	83.10355	2404.15973	-568.14894	1149.30085	1
C	78.10933	75.20335	81.14412	-544.54484	466.16019	331.29119	1
H	78.94479	75.74287	80.65112	-1409.80988	-170.17955	2325.32679	1
C	78.42084	73.68638	81.41715	-184.48284	116.78282	-758.89137	1
H	79.35403	73.57705	82.01559	1893.78641	1861.14652	611.04528	1
H	77.60830	73.24129	82.03607	-2170.79665	-974.19311	1327.50117	1
C	78.54673	72.93555	80.05161	389.51668	672.81878	-138.49152	1
H	77.58853	73.00495	79.48755	510.64577	1933.03327	1851.02484	1
H	79.28521	73.44328	79.39355	-2302.30869	-1105.72939	-948.89774	1
C	78.95457	71.42400	80.23021	-559.93427	-126.34590	542.30857	1
O	79.23553	70.76451	79.26246	-670.36003	-111.83166	76.36448	1
N	78.94505	70.99330	81.51115	-216.06729	-537.07037	446.51379	1
H	78.67209	71.48793	82.36802	-1251.96487	1074.12894	-2168.52500	1
H	79.14037	70.02537	81.78959	-2451.83023	-720.73851	944.84612	1
C	76.67618	75.37294	80.40254	546.24052	-563.88337	-85.11809	1
O	75.73825	74.74531	80.82530	71.70702	-307.40463	606.69673	1
N	76.75395	76.14320	79.30205	479.90863	-504.50129	222.86849	1
C	75.56683	76.59116	78.64807	75.51268	-600.91904	506.73700	1
H	74.77576	76.51419	79.43018	-2033.61562	1795.30103	254.89480	1
C	76.00978	78.06640	78.23835	523.79067	517.99274	284.45581	1
H	75.34412	78.54208	77.48337	1599.61665	-1127.00911	1895.98173	1
H	76.03354	78.74313	79.12138	-1881.83093	1275.82488	-1501.55016	1
C	77.44738	77.78306	77.71292	-600.05639	-404.05026	316.64485	1
H	77.41709	77.26437	76.70937	-2597.05260	496.36221	657.73249	1
H	78.07171	78.69579	77.58542	-1666.24518	1294.38985	-1723.90591	1
C	77.96371	76.84282	78.88823	493.57570	-129.95376	-602.56394	1
H	78.72687	76.08451	78.59162	-1469.08415	-637.84797	2204.22581	1
H	78.31639	77.40439	79.78970	-1038.97840	1068.88701	-2280.71789	1
C	75.26190	75.62230	77.35943	188.88100	241.05089	-727.87821	1
O	75.44238	74.42951	77.39245	531.88613	-49.19943	427.07960	1
N	74.66517	76.33606	76.40745	708.14833	26.93417	-179.73509	1
H	74.60565	77.34690	76.52346	-2232.69501	-1179.09831	1023.94816	1
C	74.58567	75.75284	75.10468	-738.32412	259.92548	104.42456	1
H	74.50274	74.66194	75.32130	2028.89123	1260.02983	1311.33324	1
C	73.29654	76.39630	74.43660	-258.65678	-345.52513	661.28459	1
H	73.46265	77.48176	74.26824	-1826.06090	-1801.46097	-918.64554	1
C	72.86171	75.70606	73.09610	-541.23400	531.92970	-218.41428	1
H	71.87300	76.08288	72.74292	1311.73218	-2219.18200	882.19023	1
H	73.59415	75.90479	72.27413	-1048.71298	1223.58118	-2196.97971	1
H	72.76250	74.60039	73.20430	419.06414	1903.92898	1903.44670	1
C	72.09446	76.17437	75.41774	-423.86431	152.66566	-648.55262	1
H	71.14779	76.60979	75.02030	105.68386	-2342.50080	1387.51238	1
H	71.91249	75.08955	75.60172	-1695.63603	-1484.73621	-1531.02793	1
H	72.28895	76.64627	76.40934	-1853.57121	-487.39031	1936.59343	1
C	75.98419	76.11957	74.34644	539.71428	-510.58232	267.58633	1
O	76.91751	76.69228	74.87426	-15.93758	-278.34085	-624.49402	1

N	75.93469	75.58925	73.13155	-676.81886	12.05292	276.17960	1
H	75.05726	75.16075	72.84632	-1694.44250	-373.84189	-2100.66098	1
C	76.93498	75.99193	72.17856	412.33078	-664.70360	108.35451	1
H	76.99742	77.09996	72.26033	1765.77476	1294.44366	1621.76594	1
C	78.28294	75.33041	72.67287	-179.66635	-721.95667	-264.73915	1
H	78.61441	75.80381	73.62823	-607.34922	2587.87032	-598.10018	1
H	78.10129	74.25481	72.92435	-359.69332	-1479.13954	-2259.74260	1
C	79.38485	75.43707	71.56818	-240.01076	616.02425	431.85322	1
H	78.97372	75.14524	70.57178	-1359.17131	782.54425	2227.99181	1
H	79.76094	76.47874	71.45555	-820.36667	85.81767	-2596.78715	1
C	80.51996	74.44116	72.04155	-513.07015	236.18570	-551.87280	1
H	81.00468	74.81360	72.97190	1816.11944	-1183.11722	-1650.94321	1
H	80.08098	73.45329	72.31281	-1800.04429	-1361.53473	1526.34643	1
C	81.56517	74.25861	70.91801	726.08972	-253.19732	-179.64713	1
H	81.23038	73.62832	70.07037	1224.27271	-456.51798	-2390.90304	1
H	82.00731	75.19870	70.53487	1206.40339	-1150.93514	-2154.90344	1
N	82.76375	73.47895	71.56097	329.70796	-633.68968	-155.67358	1
H	83.59545	73.28019	70.93237	1348.31305	1938.76845	1359.00643	1
H	83.23644	73.92418	72.40078	2096.92035	1446.54551	-966.48890	1
H	82.54371	72.50945	71.92992	-1197.07217	-2303.53719	827.28733	1
C	76.51227	75.47865	70.69756	-378.01186	-556.17301	-413.96481	1
O	77.02800	76.00504	69.73670	-409.44433	-307.55053	-453.30833	1
N	75.68484	74.43042	70.70137	-429.02477	524.70884	274.08502	1
H	75.36610	74.05247	71.59279	1706.34263	-1425.96036	1574.38926	1
C	74.91014	74.24269	69.50481	-45.25864	-626.22952	478.93267	1
H	74.97657	75.25974	69.05182	1988.63164	570.90773	1772.87287	1
C	75.66744	73.17833	68.61868	551.77128	-20.49526	-564.54996	1
H	76.75439	73.41720	68.58234	1434.61535	-1753.10253	1513.99471	1
H	75.60338	72.16526	69.07537	-714.67738	1819.09770	1898.36494	1
C	75.05268	73.19637	67.17800	446.99263	-555.77305	338.97221	1
H	73.94986	73.04520	67.20309	1953.68466	1892.25957	-161.67766	1
H	75.20866	74.18546	66.69223	-403.03785	2688.07032	-188.43591	1
C	75.76521	72.04577	66.36029	567.86262	335.74681	434.04421	1
H	76.86375	72.22465	66.30752	-1080.02275	1568.26081	-1948.78900	1
H	75.64357	71.06303	66.87119	-919.54108	2111.34692	1456.13463	1
C	75.15083	72.00297	64.94196	195.81897	173.33862	-745.11536	1
H	74.09217	71.67723	64.90595	1405.67949	-2301.21089	-390.08113	1
H	75.28009	72.93345	64.35407	-940.37328	-1118.32446	2299.72133	1
N	75.93385	70.91596	64.12969	319.26422	656.36007	-42.02424	1
H	75.60921	70.76638	63.13103	1961.03633	619.91968	1787.09422	1
H	76.97265	71.08059	63.98603	854.14343	-2156.80752	1429.08711	1
H	75.92017	69.92300	64.50459	1237.02905	-1378.50857	-1998.28500	1
C	73.40994	73.81884	69.98439	532.53319	-268.57236	517.55729	1
O	72.80026	74.55495	70.73621	266.16714	-290.73804	-558.88002	1
N	73.00330	72.65779	69.47887	-226.25352	640.49678	-270.32839	1
H	73.62513	72.13772	68.86432	2007.69063	-1035.27229	1523.50231	1
C	71.61517	72.35919	69.58781	-540.76542	-573.33303	49.50000	1
H	71.15771	73.28948	70.00203	-2374.20950	-1036.76023	843.75774	1
C	71.06810	71.92146	68.14546	-77.09082	666.87422	-415.84136	1
H	71.47406	70.90314	67.89269	-985.81834	617.19825	2463.91927	1
O	71.42581	72.76882	67.04337	293.29899	-391.65602	-477.80926	1
H	71.86454	72.12738	66.44606	2528.72714	-681.76262	-751.27122	1
C	69.53140	71.79848	68.45934	252.47363	-605.14473	440.05134	1
H	68.96804	71.37473	67.59504	-2052.65063	1456.49051	-1043.51830	1
H	69.33455	71.13288	69.34424	-2152.98800	-1661.97574	-161.70652	1
H	69.08127	72.79391	68.68331	-473.12726	-1584.27339	2165.61725	1
C	71.42607	71.05629	70.56448	360.36307	488.46713	505.10056	1
O	70.50252	71.07963	71.38693	-200.08817	-528.03552	-385.82850	1
N	72.24777	70.08929	70.24353	-597.54095	-226.44775	355.20479	1
H	72.88269	70.37840	69.49965	2179.23655	-1300.70981	-991.33835	1
C	72.44674	68.88859	70.95574	-687.68921	297.39807	-249.45179	1
H	73.38940	68.47946	70.53148	-349.16256	-2467.37713	-1101.72831	1

201	C	72.51771	69.20509	72.52274	672.47345	361.54328	-201.62880	1
202	H	71.56916	69.69131	72.85274	1541.41502	582.18425	2169.97026	1
203	H	72.61630	68.25138	73.09050	-2534.67717	60.25153	997.72295	1
204	C	73.71067	70.12872	72.80392	574.26774	-389.43119	-377.02542	1
205	C	75.02633	69.58712	72.91202	688.82679	-287.33580	-257.96133	1
206	H	75.20659	68.50514	72.77748	331.14182	1470.38163	2269.80013	1
207	C	73.47841	71.52794	72.96274	-383.52382	-675.04808	144.24917	1
208	H	72.46164	71.94761	72.85069	4.09847	1517.59662	2262.86397	1
209	C	76.07770	70.45723	73.20337	-134.76730	428.45285	-649.50303	1
210	H	77.09706	70.04370	73.29878	-903.23433	1600.49020	2011.53387	1
211	C	74.56405	72.35268	73.26587	-335.53281	-615.00040	-364.39058	1
212	H	74.37601	73.42850	73.41377	1596.45398	-1369.33862	1732.02659	1
213	C	75.89188	71.85563	73.39434	585.48547	-340.24194	-406.23938	1
214	O	77.00334	72.69748	73.69379	-419.15649	-173.04408	-511.94141	1
215	H	76.53045	73.56303	73.76359	-1071.84350	1559.71507	-1960.13012	1
216	C	71.19215	67.84289	70.80753	291.41878	-183.13091	710.72246	1
217	O	71.40298	66.69563	70.49756	379.87695	523.47396	-222.23622	1
218	N	70.05125	68.41464	71.16547	564.30591	373.09380	277.24371	1
219	H	70.01010	69.43718	71.40234	-120.59931	-2436.07824	-1214.35098	1
220	C	68.86571	67.62702	71.13917	404.07514	-299.37480	608.84009	1
221	H	69.17967	66.60099	70.82833	-1071.07882	-841.69025	2359.66467	1
222	C	68.29971	67.70237	72.62905	-683.12807	68.71770	-390.13096	1
223	H	69.13241	67.54072	73.34914	2068.88918	1596.58590	770.89603	1
224	H	67.90888	68.71918	72.85472	1379.86429	2342.49779	-179.85638	1
225	C	67.19529	66.62493	72.83189	-176.39967	537.49466	550.97268	1
226	H	66.26719	66.89365	72.27924	-396.28051	1942.22168	1869.33314	1
227	H	67.52021	65.64193	72.42360	-94.44700	586.00678	-2659.20016	1
228	C	66.91663	66.51476	74.40291	-291.69381	-718.18079	-150.72929	1
229	H	67.79004	66.21334	75.03534	-2303.38776	1089.76242	964.62032	1
230	H	66.45158	67.42056	74.86866	884.16729	2568.80533	-207.74283	1
231	N	65.97610	65.47429	74.44940	-594.49522	-252.81478	342.29276	1
232	H	65.61819	65.14889	73.56477	-1735.17426	2084.05705	-263.71605	1
233	C	65.54426	64.93022	75.51379	595.23177	-271.52487	442.22352	1
234	N	66.04110	65.39778	76.73514	585.53813	-424.79750	-105.81303	1
235	H	66.71498	66.13941	76.92094	959.88797	1327.56823	-2177.11905	1
236	H	65.76496	65.04159	77.65080	1578.48647	16.27378	-2220.76233	1
237	N	64.61159	63.92464	75.22206	-452.17763	-390.33541	-421.51853	1
238	H	64.24870	63.58431	74.33292	470.26107	1974.25629	-1817.92087	1
239	H	64.14811	63.36927	75.94221	1856.67307	-1573.97321	-1224.35416	1
240	C	67.84373	68.36184	70.11385	-531.47271	-581.45780	-55.06206	1
241	O	68.04353	69.49201	69.66314	-243.00837	192.73507	609.52485	1
242	N	66.80820	67.58793	69.88487	343.49288	-644.25607	38.09164	1
243	H	66.83658	66.68863	70.35529	-1691.16043	-1535.97525	-1484.73099	1
244	C	65.75749	67.98976	68.94576	542.66492	350.14337	454.42563	1
245	H	66.35283	68.28893	68.05113	1209.57979	-2396.95920	463.86744	1
246	C	64.81472	66.82105	68.71645	-737.14309	145.93010	242.71942	1
247	H	64.43084	66.49319	69.70826	-1050.18303	159.36419	-2509.06125	1
248	C	63.62813	67.23914	67.86795	-706.77696	-252.88343	-245.16230	1
249	H	62.90546	66.39144	67.71187	-1624.64039	-1665.49085	1417.86935	1
250	H	63.05936	68.08349	68.35335	2566.10521	598.51109	693.22305	1
251	H	63.95039	67.59561	66.85059	-1972.69758	107.10018	-1876.34312	1
252	C	65.52151	65.63975	68.07806	412.85148	672.58832	-27.69222	1
253	H	64.82912	64.76433	67.93962	1447.02277	1762.76533	-1490.78944	1
254	H	65.93092	65.90384	67.06417	1320.77379	-145.20455	2378.68491	1
255	H	66.38870	65.28714	68.70146	-1512.10848	1470.52031	1724.75147	1
256	C	64.99769	69.30685	69.61994	-607.11113	499.56689	73.73769	1
257	O	63.92622	69.19628	70.15343	357.97126	460.81534	356.68785	1
258	N	65.74629	70.38219	69.44856	536.55407	-368.62270	332.76306	1
259	H	66.70557	70.34348	69.01699	524.38866	2297.69985	1367.20941	1
260	C	65.24698	71.61555	69.92329	46.50277	-774.29190	-147.97904	1
261	H	64.26502	71.39365	70.40274	1676.51875	1883.29066	1032.55549	1
262	C	66.35399	72.26826	70.90784	-559.41456	-34.19198	-556.30433	1

```
H    67.26282   72.53046   70.31587       -64.66664       395.18769      2695.05373           1
H    65.97952   73.22855   71.33509     -2703.44278       -60.40056       333.79418           1
C    66.70886   71.28823   71.99215      -519.76435       377.31045       459.42366           1
H    66.97831   70.31887   71.50577     -1746.74323      1667.50644     -1261.73691           1
C    67.90677   71.77654   72.79175       570.95554        16.46261      -545.27700           1
H    68.03844   71.19127   73.73808      2391.58273        28.05277     -1305.07271           1
H    68.87748   71.66873   72.19704       175.75588       917.63458      2559.43867           1
H    67.80232   72.85454   73.08027     -1063.97851     -2155.34650      1283.00462           1
C    65.53221   71.05639   72.93117       193.76014         2.62946      -765.53141           1
H    65.79884   70.35549   73.76374       507.39394      -846.55087      2539.60089           1
H    65.18470   72.01274   73.40077      1337.57841      1818.19054      1526.01994           1
H    64.65648   70.61039   72.39202      1100.88635     -1172.91350     -2199.08909           1
C    65.17001   72.61117   68.64776      -549.21115       475.64486      -309.38218           1
O    65.78220   72.43457   67.62784       556.06106      -390.37755       -78.24250           1
O    64.43323   73.80122   68.84951      -572.27940      -310.19032      -209.75862           1
H    64.08585   73.61192   69.76591     -1380.08193      2212.14486       790.85944           1
```

Listing 43: C00043.xmolout

```
       276
  150.0000  150.0000  150.0000     0.0000     0.0000     0.0000***********    2.7750    11100
       0.2500    300.00   0.00   0.00   0.00  -1293.7815       0.0000    300.0000
H    65.75450   67.48575   76.09365     -2043.14101     -1757.64893       399.89339           1
N    66.51773   67.71593   76.72913       426.75047       490.45733      -334.42467           1
H    66.85335   66.79428   77.03788       442.25559     -1110.30854     -2448.52907           1
C    67.56031   68.45672   76.12960       415.32972      -666.39213        83.73172           1
H    67.15071   68.92167   75.20600       879.32219      2491.00447       667.35213           1
C    68.09278   69.55151   77.18911        31.35357       477.41777       628.23362           1
H    68.45826   69.02695   78.10394      1168.38489      2240.25337     -1019.71136           1
H    68.97143   70.10300   76.77655      -360.43770      2534.90498       931.67112           1
C    66.97504   70.49807   77.53673       463.18208       512.70242      -382.34388           1
H    66.07995   69.89060   77.80166      1725.29117      1863.52247      -987.07854           1
C    67.35588   71.35233   78.73991       710.42801      -144.27427      -313.15367           1
H    66.52391   72.04106   79.03825       364.20195     -1033.13725      2494.72524           1
H    67.60354   70.72306   79.63334      2567.61904       335.80679       847.48802           1
H    68.25177   71.98993   78.52469     -2587.99029       680.92867      -512.16411           1
C    66.62197   71.40579   76.36616       268.17616       741.64379       -40.42634           1
H    65.79696   72.11685   76.63006      1283.54314     -1945.37420     -1411.27812           1
H    67.49970   72.02338   76.04394     -1258.57796      1317.15249     -2026.02085           1
H    66.28134   70.82108   75.47357     -2502.84526       909.80597       575.92391           1
C    68.70320   67.33425   75.86416       619.36578      -458.64843      -172.09273           1
O    68.62910   66.21449   76.32961       -81.40433       433.87611       522.34594           1
N    69.69661   67.89257   75.16917       175.23996      -622.20190       341.55523           1
H    69.52689   68.83899   74.82719      -876.57256      -303.22879     -2561.90213           1
C    70.58003   67.01960   74.43198      -571.88214      -534.10783      -106.15033           1
H    69.93104   66.22951   73.99655     -2713.20841       -16.25800      -248.80809           1
C    71.26812   67.97966   73.35928       166.60354       288.16399      -716.09568           1
H    71.83599   68.78467   73.90229     -2097.85574      1636.78449      -586.18044           1
O    70.35920   68.67006   72.47864      -317.80580      -363.38227       484.43082           1
H    70.62184   69.60057   72.62540      1863.74748     -1451.84923      1357.29467           1
C    72.29755   67.00720   72.67688       503.80731       517.99032       318.51606           1
H    72.89321   67.53339   71.89222       640.61020     -2157.10410     -1536.29104           1
H    73.01992   66.59785   73.42037      1687.88455       465.60692      2087.56452           1
H    71.79081   66.14148   72.18685     -1293.07895     -1899.88581     -1463.57452           1
C    71.67790   66.44360   75.46534      -153.07550      -733.68902       248.70981           1
O    72.33994   67.23154   76.12072       186.15459      -480.78787      -449.34387           1
N    71.82368   65.12426   75.35784      -487.53139       543.18517        42.03850           1
C    72.38815   64.34657   76.42577      -699.35871        69.34393       360.10701           1
H    71.91221   64.72851   77.35616         2.81126      -221.99807     -2715.58072           1
C    72.00614   62.86745   76.02265      -507.46439      -592.59194       122.07761           1
H    72.69653   62.10814   76.45526     -1975.08116       525.87560     -1801.71556           1
H    70.98099   62.60008   76.36507      1588.27743     -1913.22100      1113.83566           1
```

C	72.07895	62.95042	74.46533	-613.62442	-457.55993	-194.14490	1
H	73.13867	62.98657	74.12657	1707.54637	-721.27992	1996.92523	1
H	71.59610	62.09449	73.94261	758.23357	2100.81767	1560.55011	1
C	71.33454	64.33475	74.23342	-352.41524	704.96076	49.21465	1
H	71.57827	64.86198	73.28032	1714.76886	-2070.42778	443.35792	1
H	70.22680	64.27969	74.35752	1224.15040	1801.50881	1636.97633	1
C	74.02070	64.56556	76.43117	566.85117	537.05831	117.62814	1
O	74.72822	64.04240	75.60294	-550.26779	-406.08867	4.21666	1
N	74.33373	65.33593	77.45979	566.34832	-425.77725	180.18749	1
H	73.56454	65.66020	78.04520	644.44502	2646.80550	-52.73430	1
C	75.65768	65.81057	77.69444	612.34801	-281.48662	411.56221	1
H	75.55220	66.33830	78.67005	1667.23498	-1490.75142	1556.16753	1
C	76.58645	64.50542	77.83143	-714.45746	-175.43074	286.98927	1
H	76.66730	64.04495	76.80757	-787.09484	1763.03619	-1922.46060	1
O	76.07771	63.48187	78.70607	-429.46514	516.51932	-128.40526	1
H	75.85240	62.78676	78.05521	1981.82785	436.16847	-1818.18182	1
C	77.98295	65.07603	78.26881	630.79189	-468.94448	76.03230	1
H	78.75018	64.26913	78.32885	-1775.71861	2062.30353	131.89849	1
H	78.36839	65.85340	77.55360	-1855.14608	88.94118	1993.53760	1
H	77.92739	65.54979	79.27675	-1504.82350	-780.94249	-2132.90992	1
C	76.09484	66.82577	76.52104	288.57638	618.62814	-397.00270	1
O	77.30156	67.03141	76.31768	321.70495	-185.26452	574.37218	1
N	75.06179	67.39124	75.96398	-447.05792	-446.53431	367.76425	1
H	74.10748	67.11073	76.28555	350.31316	1975.63644	-1843.31522	1
C	75.10311	68.35468	74.92414	-511.23061	374.03917	-471.51480	1
H	74.05183	68.72132	74.92765	2234.79579	-898.70890	-1273.45190	1
C	76.16968	69.49165	75.28034	529.53781	-159.34528	563.72604	1
H	75.86672	70.44801	74.77130	654.59388	2347.94251	1217.51488	1
H	76.06299	69.66571	76.38374	-1526.55661	2103.42026	817.87394	1
O	77.53287	69.23661	74.91562	-554.84278	14.12789	399.58769	1
H	77.67402	68.36984	75.44454	1297.06236	2061.84854	1220.68812	1
C	75.49039	67.70104	73.47724	-662.15484	-412.13544	123.62687	1
O	74.98384	68.16249	72.48649	-540.01537	-317.05804	274.91456	1
N	76.41800	66.75097	73.58038	-202.76299	-690.00100	-131.49145	1
H	76.71263	66.51363	74.54010	-702.62132	-2617.37191	281.70413	1
C	76.78210	65.88731	72.46459	-491.73914	576.34187	222.73509	1
H	77.41684	65.10146	72.93905	1977.17978	-1823.58850	-434.68928	1
C	75.51251	65.31341	71.85721	-649.50797	-328.66843	306.11224	1
H	74.93076	66.16618	71.43414	2075.07803	1452.16490	-1004.45904	1
C	75.80624	64.33395	70.73734	-615.18735	-489.99689	-70.96352	1
H	74.86386	63.96245	70.24829	-1226.32661	-1724.38906	-1716.47170	1
H	76.44444	64.80935	69.93118	2381.64273	-969.84735	900.46860	1
H	76.36459	63.43226	71.11206	2122.74991	1702.23946	-141.36228	1
C	74.64915	64.63894	72.90879	-501.86074	-479.61276	-376.42482	1
H	73.68054	64.26502	72.47767	993.79261	-2246.95401	-1177.81305	1
H	75.17323	63.75954	73.37900	42.71279	-626.15391	-2651.37258	1
H	74.39902	65.34244	73.75143	-2026.39012	-1590.33709	-887.82922	1
C	77.64778	66.79142	71.36080	-672.89153	382.57394	-156.34072	1
O	77.81409	66.33429	70.24884	410.04627	221.86223	500.35950	1
N	78.09215	67.91401	71.87173	-378.11782	-443.79414	-441.10984	1
H	77.80717	68.06323	72.83780	2404.15973	-568.14894	1149.30085	1
C	79.09708	68.74754	71.26870	-544.54484	466.16019	331.29119	1
H	79.99134	68.60461	71.91070	-1409.80988	-170.17955	2325.32679	1
C	79.31248	68.33470	69.76346	-184.48284	116.78282	-758.89137	1
H	79.70696	67.29208	69.68733	1893.78641	1861.14652	611.04528	1
H	78.32326	68.32388	69.24549	-2170.79665	-974.19311	1327.50117	1
C	80.30104	69.33505	69.08002	389.51668	672.81878	-138.49152	1
H	79.97650	70.38425	69.25570	510.64577	1933.03327	1851.02484	1
H	81.30352	69.27962	69.55810	-2302.30869	-1105.72939	-948.89774	1
C	80.43227	69.07327	67.53243	-559.93427	-126.34590	542.30857	1
O	81.13695	69.78706	66.86547	-670.36003	-111.83166	76.36448	1
N	79.71026	68.02229	67.08549	-216.06729	-537.07037	446.51379	1

H	79.09458	67.37856	67.59660	-1251.96487	1074.12894	-2168.52500	1
H	79.68785	67.71893	66.10606	-2451.83023	-720.73851	944.84612	1
C	78.51171	70.26823	71.34049	546.24052	-563.88337	-85.11809	1
O	77.54268	70.56685	70.68963	71.70702	-307.40463	606.69673	1
N	79.30219	71.05985	72.07825	479.90863	-504.50129	222.86849	1
C	78.83935	72.34190	72.52219	75.51268	-600.91904	506.73700	1
H	77.77765	72.21351	72.83286	-2033.61562	1795.30103	254.89480	1
C	79.86831	72.67884	73.68275	523.79067	517.99274	284.45581	1
H	79.93381	73.76856	73.90047	1599.61665	-1127.00911	1895.98173	1
H	79.59170	72.17653	74.63684	-1881.83093	1275.82488	-1501.55016	1
C	81.18189	72.08678	73.08602	-600.05639	-404.05026	316.64485	1
H	81.56865	72.73238	72.26273	-2597.05260	496.36221	657.73249	1
H	82.00448	71.97375	73.82744	-1666.24518	1294.38985	-1723.90591	1
C	80.63995	70.70093	72.52533	493.57570	-129.95376	-602.56394	1
H	81.20459	70.27815	71.66118	-1469.08415	-637.84797	2204.22581	1
H	80.50280	69.91497	73.30597	-1038.97840	1068.88701	-2280.71789	1
C	79.01984	73.41046	71.29374	188.88100	241.05089	-727.87821	1
O	80.08683	73.65608	70.78693	531.88613	-49.19943	427.07960	1
N	77.83740	74.02507	71.09040	708.14833	26.93417	-179.73509	1
H	77.06070	73.53534	71.53839	-2232.69501	-1179.09831	1023.94816	1
C	77.61782	74.82326	69.88101	-738.32412	259.92548	104.42456	1
H	78.55813	75.41837	69.80171	2028.89123	1260.02983	1311.33324	1
C	77.46799	73.88162	68.70168	-258.65678	-345.52513	661.28459	1
H	76.51906	73.31461	68.83496	-1826.06090	-1801.46097	-918.64554	1
C	77.38762	74.63742	67.38827	-541.23400	531.92970	-218.41428	1
H	77.18015	73.95104	66.52175	1311.73218	-2219.18200	882.19023	1
H	76.56618	75.41513	67.41278	-1048.71298	1223.58118	-2196.97971	1
H	78.34811	75.17695	67.16172	419.06414	1903.92898	1903.44670	1
C	78.59935	72.87009	68.64658	-423.86431	152.66566	-648.55262	1
H	78.50215	72.16586	67.77484	105.68386	-2342.50080	1387.51238	1
H	79.60326	73.37732	68.56984	-1695.63603	-1484.73621	-1531.02793	1
H	78.63118	72.24902	69.59149	-1853.57121	-487.39031	1936.59343	1
C	76.26830	75.76400	70.10735	539.71428	-510.58232	267.58633	1
O	75.77050	76.31439	69.14833	-15.93758	-278.34085	-624.49402	1
N	75.87514	75.80014	71.36306	-676.81886	12.05292	276.17960	1
H	76.47271	75.27834	71.99661	-1694.44250	-373.84189	-2100.66098	1
C	74.90784	76.74836	71.84885	412.33078	-664.70360	108.35451	1
H	75.39559	77.72844	71.65348	1765.77476	1294.44366	1621.76594	1
C	73.58013	76.57552	71.02081	-179.66635	-721.95667	-264.73915	1
H	73.78580	76.64938	69.92357	-607.34922	2587.87032	-598.10018	1
H	73.15258	75.55989	71.18265	-359.69332	-1479.13954	-2259.74260	1
C	72.57872	77.68610	71.49085	-240.01076	616.02425	431.85322	1
H	72.45953	77.65770	72.59962	-1359.17131	782.54425	2227.99181	1
H	72.96232	78.70243	71.24763	-820.36667	85.81767	-2596.78715	1
C	71.19544	77.41081	70.77845	-513.07015	236.18570	-551.87280	1
H	71.29350	77.49113	69.67120	1816.11944	-1183.11722	-1650.94321	1
H	70.84984	76.37030	70.97791	-1800.04429	-1361.53473	1526.34643	1
C	70.17270	78.43580	71.32345	726.08972	-253.19732	-179.64713	1
H	69.98352	78.36195	72.41322	1224.27271	-456.51798	-2390.90304	1
H	70.37995	79.48853	71.04577	1206.40339	-1150.93514	-2154.90344	1
N	68.79178	78.10805	70.66476	329.70796	-633.68968	-155.67358	1
H	67.98314	78.73341	70.94829	1348.31305	1938.76845	1359.00643	1
H	68.73077	78.14824	69.60543	2096.92035	1446.54551	-966.48890	1
H	68.39152	77.14803	70.87250	-1197.07217	-2303.53719	827.28733	1
C	74.71147	76.41320	73.43035	-378.01186	-556.17301	-413.96481	1
O	73.61078	76.20370	73.89572	-409.44433	-307.55053	-453.30833	1
N	75.88062	76.41185	74.06728	-429.02477	524.70884	274.08502	1
H	76.72514	76.64851	73.54833	1706.34263	-1425.96036	1574.38926	1
C	75.87490	76.14334	75.46291	-45.25864	-626.22952	478.93267	1
H	74.84488	75.75049	75.64269	1988.63164	570.90773	1772.87287	1
C	77.05102	75.13331	75.78201	551.77128	-20.49526	-564.54996	1
H	77.01841	74.24879	75.10665	1434.61535	-1753.10253	1513.99471	1

H	78.03772	75.61562	75.60103	-714.67738	1819.09770	1898.36494	1
C	76.90614	74.70954	77.28152	446.99263	-555.77305	338.97221	1
H	76.86329	75.61446	77.93088	1953.68466	1892.25957	-161.67766	1
H	75.95228	74.16261	77.45425	-403.03785	2688.07032	-188.43591	1
C	78.15052	73.81802	77.67739	567.86262	335.74681	434.04421	1
H	78.12424	72.83299	77.15669	-1080.02275	1568.26081	-1948.78900	1
H	79.10312	74.30360	77.36373	-919.54108	2111.34692	1456.13463	1
C	78.10838	73.64774	79.21429	195.81897	173.33862	-745.11536	1
H	78.22437	74.59172	79.78385	1405.67949	-2301.21089	-390.08113	1
H	77.23332	73.08347	79.59372	-940.37328	-1118.32446	2299.72133	1
N	79.34926	72.78988	79.63176	319.26422	656.36007	-42.02424	1
H	79.45080	72.60864	80.67210	1961.03633	619.91968	1787.09422	1
H	79.41295	71.80530	79.24036	854.14343	-2156.80752	1429.08711	1
H	80.30662	73.18306	79.39597	1237.02905	-1378.50857	-1998.28500	1
C	76.18436	77.54238	76.27886	532.53319	-268.57236	517.55729	1
O	77.22030	77.64284	76.94628	266.16714	-290.73804	-558.88002	1
N	75.21937	78.39394	76.10852	-226.25352	640.49678	-270.32839	1
H	74.50108	78.02749	75.48543	2007.69063	-1035.27229	1523.50231	1
C	75.10526	79.70360	76.67813	-540.76542	-573.33303	49.50000	1
H	74.45568	79.60223	77.57497	-2374.20950	-1036.76023	843.75774	1
C	76.55930	80.21407	77.08236	-77.09082	666.87422	-415.84136	1
H	77.26420	79.82453	76.29491	-985.81834	617.19825	2463.91927	1
O	77.01887	79.71652	78.33866	293.29899	-391.65602	-477.80926	1
H	77.43958	78.82765	78.01640	2528.72714	-681.76262	-751.27122	1
C	76.50217	81.77990	76.97487	252.47363	-605.14473	440.05134	1
H	77.49813	82.23792	77.19115	-2052.65063	1456.49051	-1043.51830	1
H	76.19131	82.12057	75.95606	-2152.98800	-1661.97574	-161.70652	1
H	75.78209	82.21283	77.70872	-473.12726	-1584.27339	2165.61725	1
C	74.35591	80.55933	75.52048	360.36307	488.46713	505.10056	1
O	74.50680	80.30725	74.34472	-200.08817	-528.03552	-385.82850	1
N	73.66272	81.56715	76.06100	-597.54095	-226.44775	355.20479	1
H	73.63186	81.69987	77.07305	2179.23655	-1300.70981	-991.33835	1
C	72.88884	82.35119	75.16562	-687.68921	297.39807	-249.45179	1
H	72.74794	81.71193	74.26388	-349.16256	-2467.37713	-1101.72831	1
C	71.53459	82.79910	75.89169	672.47345	361.54328	-201.62880	1
H	71.75285	83.26683	76.87958	1541.41502	582.18425	2169.97026	1
H	71.01235	83.56347	75.27098	-2534.67717	60.25153	997.72295	1
C	70.66075	81.54788	76.05445	574.26774	-389.43119	-377.02542	1
C	69.98022	81.01465	74.91821	688.82679	-287.33580	-257.96133	1
H	70.06872	81.49823	73.92841	331.14182	1470.38163	2269.80013	1
C	70.55688	80.91110	77.32759	-383.52382	-675.04808	144.24917	1
H	71.09483	81.31479	78.20509	4.09847	1517.59662	2262.86397	1
C	69.20289	79.86894	75.08361	-134.76730	428.45285	-649.50303	1
H	68.67469	79.45668	74.20616	-903.23433	1600.49020	2011.53387	1
C	69.76243	79.76768	77.44218	-335.53281	-615.00040	-364.39058	1
H	69.68344	79.28593	78.43240	1596.45398	-1369.33862	1732.02659	1
C	69.05833	79.20598	76.33726	585.48547	-340.24194	-406.23938	1
O	68.24804	78.04588	76.44774	-419.15649	-173.04408	-511.94141	1
H	68.36670	77.85151	77.40760	-1071.84350	1559.71507	-1960.13012	1
C	73.68627	83.73301	74.79818	291.41878	-183.13091	710.72246	1
O	73.41682	84.32751	73.77883	379.87695	523.47396	-222.23622	1
N	74.52225	84.09259	75.76432	564.30591	373.09380	277.24371	1
H	74.56799	83.52199	76.60674	-120.59931	-2436.07824	-1214.35098	1
C	75.36704	85.22350	75.53580	404.07514	-299.37480	608.84009	1
H	75.39266	85.26592	74.42208	-1071.07882	-841.69025	2359.66467	1
C	74.59696	86.47176	76.13529	-683.12807	68.71770	-390.13096	1
H	73.54439	86.47407	75.77231	2068.88918	1596.58590	770.89603	1
H	74.52292	86.40567	77.24383	1379.86429	2342.49779	-179.85638	1
C	75.33336	87.77195	75.68594	-176.39967	537.49466	550.97268	1
H	76.37951	87.79892	76.06204	-396.28051	1942.22168	1869.33314	1
H	75.41321	87.82528	74.57736	-94.44700	586.00678	-2659.20016	1
C	74.47347	88.98933	76.26092	-291.69381	-718.18079	-150.72929	1

```
H    73.43668  89.08291  75.84833    -2303.38776     1089.76242      964.62032          1
H    74.39865  89.03668  77.37716      884.16729     2568.80533     -207.74283          1
N    75.19819  90.11256  75.83777     -594.49522     -252.81478      342.29276          1
H    76.05841  89.93894  75.34172    -1735.17426     2084.05705     -263.71605          1
C    74.84134  91.31535  76.03945      595.23177     -271.52487      442.22352          1
N    73.64418  91.53021  76.73174      585.53813     -424.79750     -105.81303          1
H    72.98505  90.85190  77.11175      959.88797     1327.56823    -2177.11905          1
H    73.25311  92.44686  76.95125     1578.48647       16.27378    -2220.76233          1
N    75.76875  92.21804  75.50062     -452.17763     -390.33541     -421.51853          1
H    76.63897  92.06362  74.99338      470.26107     1974.25629    -1817.92087          1
H    75.65678  93.23114  75.55875     1856.67307    -1573.97321    -1224.35416          1
C    76.81117  84.87001  76.21086     -531.47271     -581.45780      -55.06206          1
O    77.48505  83.99103  75.72613     -243.00837      192.73507      609.52485          1
N    77.12188  85.64770  77.24765      343.49288     -644.25607       38.09164          1
H    76.42782  86.33378  77.53843    -1691.16043    -1535.97525    -1484.73099          1
C    78.31742  85.36258  78.04120      542.66492      350.14337      454.42563          1
H    79.03277  84.92441  77.30375     1209.57979    -2396.95920      463.86744          1
C    78.78634  86.65547  78.69028     -737.14309      145.93010      242.71942          1
H    77.93622  87.06798  79.27827    -1050.18303      159.36419    -2509.06125          1
C    79.94231  86.41249  79.64200     -706.77696     -252.88343     -245.16230          1
H    80.24086  87.35091  80.18619    -1624.64039    -1665.49085     1417.86935          1
H    79.68127  85.63717  80.41838     2566.10521      598.51109      693.22305          1
H    80.85286  86.03769  79.09778    -1972.69758      107.10018    -1876.34312          1
C    79.17341  87.69346  77.65288      412.85148      672.58832      -27.69222          1
H    79.45856  88.67292  78.12655     1447.02277     1762.76533    -1490.78944          1
H    80.05094  87.35213  77.03728     1320.77379     -145.20455     2378.68491          1
H    78.32942  87.89784  76.93872    -1512.10848     1470.52031     1724.75147          1
C    77.92481  84.26252  79.22123     -607.11113      499.56689       73.73769          1
O    78.81140  83.63039  79.75407      357.97126      460.81534      356.68785          1
N    76.63778  84.27902  79.51269      536.55407     -368.62270      332.76306          1
H    76.03509  84.87544  78.94944      524.38866     2297.69985     1367.20941          1
C    76.12484  83.28963  80.39183       46.50277     -774.29190     -147.97904          1
H    76.93324  82.52372  80.44170     1676.51875     1883.29066     1032.55549          1
C    75.82155  83.94466  81.83847     -559.41456      -34.19198     -556.30433          1
H    74.97247  84.66099  81.73784      -64.66664      395.18769     2695.05373          1
H    75.46824  83.15738  82.54588    -2703.44278      -60.40056      333.79418          1
C    77.02257  84.65449  82.39894     -519.76435      377.31045      459.42366          1
H    77.36898  85.39954  81.64681    -1746.74323     1667.50644    -1261.73691          1
C    76.65598  85.39338  83.68148      570.95554       16.46261     -545.27700          1
H    77.52888  85.96004  84.09651     2391.58273       28.05277    -1305.07271          1
H    75.83029  86.13155  83.51487      175.75588      917.63458     2559.43867          1
H    76.31246  84.68690  84.47999    -1063.97851    -2155.34650     1283.00462          1
C    78.16935  83.69708  82.68899      193.76014        2.62946     -765.53141          1
H    79.00464  84.20713  83.23542      507.39394     -846.55087     2539.60089          1
H    77.84158  82.83079  83.31958     1337.57841     1818.19054     1526.01994          1
H    78.60532  83.28159  81.72970     1100.88635    -1172.91350    -2199.08909          1
C    74.73008  82.79369  79.76078     -549.21115      475.64486     -309.38218          1
O    74.17200  83.36028  78.85505      556.06106     -390.37755      -78.24250          1
O    74.13545  81.66083  80.36518     -572.27940     -310.19032     -209.75862          1
H    74.85013  81.45180  81.02960    -1380.08193     2212.14486      790.85944          1
```

Listing 44: C00044.xmolout

```
      276
  150.0000  150.0000  150.0000    0.0000    0.0000    0.0000***********    2.7750    11100
      0.2500     300.00   0.00   0.00   0.00  -1293.7722      0.0000    300.0000
H    65.03120  79.95604  82.05380    -2043.14101    -1757.64893      399.89339          1
N    64.97378  80.60698  82.83598      426.75047      490.45733     -334.42467          1
H    64.82898  79.97577  83.62896      442.25559    -1110.30854    -2448.52907          1
C    66.12690  81.41037  83.00294      415.32972     -666.39213       83.73172          1
H    66.68481  81.27106  82.05167      879.32219     2491.00447      667.35213          1
C    65.70781  82.95337  83.22529       31.35357      477.41777      628.23362          1
```

H	65.33133	83.08305	84.26782	1168.38489	2240.25337	-1019.71136	1
H	66.60407	83.61356	83.14245	-360.43770	2534.90498	931.67112	1
C	64.65296	83.37007	82.23717	463.18208	512.70242	-382.34388	1
H	63.78093	82.68725	82.35357	1725.29117	1863.52247	-987.07854	1
C	64.20427	84.79795	82.52595	710.42801	-144.27427	-313.15367	1
H	63.38021	85.12096	81.83942	364.20195	-1033.13725	2494.72524	1
H	63.82684	84.90974	83.57493	2567.61904	335.80679	847.48802	1
H	65.04579	85.52661	82.39838	-2587.99029	680.92867	-512.16411	1
C	65.15515	83.27353	80.80278	268.17616	741.64379	-40.42634	1
H	64.37841	83.61401	80.07051	1283.54314	-1945.37420	-1411.27812	1
H	66.06540	83.90608	80.64015	-1258.57796	1317.15249	-2026.02085	1
H	65.42809	82.22139	80.53102	-2502.84526	909.80597	575.92391	1
C	66.87647	80.78444	84.28757	619.36578	-458.64843	-172.09273	1
O	66.54856	79.68210	84.75154	-81.40433	433.87611	522.34594	1
N	67.78490	81.62874	84.71772	175.23996	-622.20190	341.55523	1
H	67.83878	82.47593	84.14868	-876.57256	-303.22879	-2561.90213	1
C	69.00998	81.26722	85.37686	-571.88214	-534.10783	-106.15033	1
H	69.02877	81.88095	86.30394	-2713.20841	-16.25800	-248.80809	1
C	69.04154	79.70808	85.70384	166.60354	288.16399	-716.09568	1
H	68.76963	79.17746	84.74405	-2097.85574	1636.78449	-586.18044	1
O	68.09876	79.30205	86.69543	-317.80580	-363.38227	484.43082	1
H	67.27784	79.14344	86.08732	1863.74748	-1451.84923	1357.29467	1
C	70.53470	79.37705	86.06422	503.80731	517.99032	318.51606	1
H	70.69263	78.27703	86.15455	640.61020	-2157.10410	-1536.29104	1
H	71.26197	79.75268	85.29311	1687.88455	465.60692	2087.56452	1
H	70.82697	79.82996	87.04173	-1293.07895	-1899.88581	-1463.57452	1
C	70.11631	81.78367	84.31224	-153.07550	-733.68902	248.70981	1
O	69.81468	81.90739	83.15113	186.15459	-480.78787	-449.34387	1
N	71.29830	82.02072	84.90129	-487.53139	543.18517	42.03850	1
C	72.46994	82.23933	84.10423	-699.35871	69.34393	360.10701	1
H	72.17613	82.95031	83.29970	2.81126	-221.99807	-2715.58072	1
C	73.50187	82.81141	85.16141	-507.46439	-592.59194	122.07761	1
H	74.55803	82.57905	84.89925	-1975.08116	525.87560	-1801.71556	1
H	73.43509	83.92040	85.22933	1588.27743	-1913.22100	1113.83566	1
C	73.03809	82.14128	86.49195	-613.62442	-457.55993	-194.14490	1
H	73.36323	81.07776	86.54108	1707.54637	-721.27992	1996.92523	1
H	73.42507	82.64232	87.40765	758.23357	2100.81767	1560.55011	1
C	71.46065	82.26144	86.32676	-352.41524	704.96076	49.21465	1
H	70.86274	81.51926	86.90676	1714.76886	-2070.42778	443.35792	1
H	71.05270	83.28387	86.51207	1224.15040	1801.50881	1636.97633	1
C	72.95420	80.79762	83.49183	566.85117	537.05831	117.62814	1
O	72.99315	79.79950	84.17404	-550.26779	-406.08867	4.21666	1
N	73.24105	80.95112	82.20011	566.34832	-425.77725	180.18749	1
H	73.13688	81.90695	81.85751	644.44502	2646.80550	-52.73430	1
C	74.22599	80.12769	81.56591	612.34801	-281.48662	411.56221	1
H	74.32459	80.62045	80.57257	1667.23498	-1490.75142	1556.16753	1
C	75.59064	80.21985	82.39230	-714.45746	-175.43074	286.98927	1
H	75.46408	79.65329	83.35420	-787.09484	1763.03619	-1922.46060	1
O	76.01763	81.54912	82.74367	-429.46514	516.51932	-128.40526	1
H	76.05921	81.47905	83.71803	1981.82785	436.16847	-1818.18182	1
C	76.57837	79.44215	81.44637	630.79189	-468.94448	76.03230	1
H	77.59569	79.36280	81.89524	-1775.71861	2062.30353	131.89849	1
H	76.21898	78.39538	81.23969	-1855.14608	88.94118	1993.53760	1
H	76.69303	79.95679	80.46377	-1504.82350	-780.94249	-2132.90992	1
C	73.64929	78.61490	81.45296	288.57638	618.62814	-397.00270	1
O	74.37367	77.63408	81.48306	321.70495	-185.26452	574.37218	1
N	72.35543	78.65909	81.13604	-447.05792	-446.53431	367.76425	1
H	71.92174	79.57526	81.24860	350.31316	1975.63644	-1843.31522	1
C	71.61948	77.44259	81.21197	-511.23061	374.03917	-471.51480	1
H	72.36844	76.64107	81.01555	2234.79579	-898.70890	-1273.45190	1
C	71.08344	77.38157	82.72759	529.53781	-159.34528	563.72604	1
H	70.51466	76.42014	82.83753	654.59388	2347.94251	1217.51488	1

H	71.99351	77.30499	83.38225	-1526.55661	2103.42026	817.87394	1
O	70.25716	78.48816	83.15761	-554.84278	14.12789	399.58769	1
H	70.96930	79.13824	83.35860	1297.06236	2061.84854	1220.68812	1
C	70.38394	77.45249	80.16423	-662.15484	-412.13544	123.62687	1
O	69.65291	76.49082	80.09595	-540.01537	-317.05804	274.91456	1
N	70.33225	78.55795	79.41536	-202.76299	-690.00100	-131.49145	1
H	71.06373	79.24626	79.58129	-702.62132	-2617.37191	281.70413	1
C	69.26255	78.76410	78.45421	-491.73914	576.34187	222.73509	1
H	68.55013	77.94440	78.71728	1977.17978	-1823.58850	-434.68928	1
C	68.72295	80.17094	78.63531	-649.50797	-328.66843	306.11224	1
H	69.56204	80.88102	78.45840	2075.07803	1452.16490	-1004.45904	1
C	67.62703	80.48284	77.63292	-615.18735	-489.99689	-70.96352	1
H	67.23442	81.52956	77.75703	-1226.32661	-1724.38906	-1716.47170	1
H	68.00474	80.38836	76.57508	2381.64273	-969.84735	900.46860	1
H	66.75308	79.78326	77.74098	2122.74991	1702.23946	-141.36228	1
C	68.21756	80.40260	80.04805	-501.86074	-479.61276	-376.42482	1
H	67.89521	81.46826	80.20609	993.79261	-2246.95401	-1177.81305	1
H	67.33460	79.74696	80.28298	42.71279	-626.15391	-2651.37258	1
H	69.00922	80.17358	80.81539	-2026.39012	-1590.33709	-887.82922	1
C	69.96769	78.55836	76.94363	-672.89153	382.57394	-156.34072	1
O	70.06768	79.48653	76.17685	410.04627	221.86223	500.35950	1
N	70.33292	77.30011	76.78658	-378.11782	-443.79414	-441.10984	1
H	70.11436	76.71499	77.59184	2404.15973	-568.14894	1149.30085	1
C	70.92843	76.79899	75.59384	-544.54484	466.16019	331.29119	1
H	70.98853	75.70183	75.78091	-1409.80988	-170.17955	2325.32679	1
C	69.93035	77.18939	74.41186	-184.48284	116.78282	-758.89137	1
H	68.88051	76.97544	74.71325	1893.78641	1861.14652	611.04528	1
H	69.97420	78.28993	74.23113	-2170.79665	-974.19311	1327.50117	1
C	70.30780	76.39324	73.12788	389.51668	672.81878	-138.49152	1
H	71.33287	76.68480	72.78303	510.64577	1933.03327	1851.02484	1
H	70.39533	75.30884	73.36101	-2302.30869	-1105.72939	-948.89774	1
C	69.28735	76.58844	71.94262	-559.93427	-126.34590	542.30857	1
O	69.45306	75.97631	70.91988	-670.36003	-111.83166	76.36448	1
N	68.28813	77.45830	72.20351	-216.06729	-537.07037	446.51379	1
H	68.12912	78.04182	73.03331	-1251.96487	1074.12894	-2168.52500	1
H	67.57065	77.72628	71.52070	-2451.83023	-720.73851	944.84612	1
C	72.36540	77.46322	75.26894	546.24052	-563.88337	-85.11809	1
O	72.64171	77.62360	74.08677	71.70702	-307.40463	606.69673	1
N	73.15506	77.64419	76.31961	479.90863	-504.50129	222.86849	1
C	74.29447	78.52448	76.25217	75.51268	-600.91904	506.73700	1
H	73.94482	79.40274	75.66195	-2033.61562	1795.30103	254.89480	1
C	74.58964	78.78641	77.78942	523.79067	517.99274	284.45581	1
H	75.62564	79.14702	77.97840	1599.61665	-1127.00911	1895.98173	1
H	73.89656	79.54029	78.22667	-1881.83093	1275.82488	-1501.55016	1
C	74.31654	77.36195	78.36141	-600.05639	-404.05026	316.64485	1
H	75.13127	76.65878	78.07188	-2597.05260	496.36221	657.73249	1
H	74.24079	77.33435	79.48028	-1666.24518	1294.38985	-1723.90591	1
C	72.95181	77.02177	77.62214	493.57570	-129.95376	-602.56394	1
H	72.73278	75.93864	77.47250	-1469.08415	-637.84797	2204.22581	1
H	72.05869	77.52543	78.06191	-1038.97840	1068.88701	-2280.71789	1
C	75.54073	77.75349	75.53554	188.88100	241.05089	-727.87821	1
O	76.50791	77.45170	76.17972	531.88613	-49.19943	427.07960	1
N	75.29839	77.59317	74.23385	708.14833	26.93417	-179.73509	1
H	74.35006	77.85616	73.86178	-2232.69501	-1179.09831	1023.94816	1
C	76.30241	77.04329	73.32097	-738.32412	259.92548	104.42456	1
H	75.86763	77.30009	72.32645	2028.89123	1260.02983	1311.33324	1
C	77.63820	77.71545	73.56643	-258.65678	-345.52513	661.28459	1
H	77.94112	77.49043	74.62049	-1826.06090	-1801.46097	-918.64554	1
C	78.72371	77.19888	72.64087	-541.23400	531.92970	-218.41428	1
H	79.71484	77.68930	72.84542	1311.73218	-2219.18200	882.19023	1
H	78.86745	76.08180	72.76007	-1048.71298	1223.58118	-2196.97971	1
H	78.47429	77.38971	71.56112	419.06414	1903.92898	1903.44670	1

C	77.53678	79.22503	73.42655	-423.86431	152.66566	-648.55262	1
H	78.51097	79.73320	73.66348	105.68386	-2342.50080	1387.51238	1
H	77.24973	79.52445	72.38176	-1695.63603	-1484.73621	-1531.02793	1
H	76.75884	79.64907	74.11851	-1853.57121	-487.39031	1936.59343	1
C	76.35805	75.39925	73.53319	539.71428	-510.58232	267.58633	1
O	77.37791	74.77769	73.34043	-15.93758	-278.34085	-624.49402	1
N	75.10719	74.87635	73.66422	-676.81886	12.05292	276.17960	1
H	74.55755	75.72618	73.84940	-1694.44250	-373.84189	-2100.66098	1
C	74.98597	73.77966	74.60842	412.33078	-664.70360	108.35451	1
H	75.24566	74.20787	75.60051	1765.77476	1294.44366	1621.76594	1
C	73.47640	73.36603	74.47988	-179.66635	-721.95667	-264.73915	1
H	72.80653	74.11094	74.96631	-607.34922	2587.87032	-598.10018	1
H	73.18622	73.36751	73.40482	-359.69332	-1479.13954	-2259.74260	1
C	73.27550	71.93543	75.08972	-240.01076	616.02425	431.85322	1
H	74.14088	71.28046	74.83286	-1359.17131	782.54425	2227.99181	1
H	73.23437	71.96529	76.20122	-820.36667	85.81767	-2596.78715	1
C	71.95270	71.33449	74.46847	-513.07015	236.18570	-551.87280	1
H	71.04977	71.86869	74.84417	1816.11944	-1183.11722	-1650.94321	1
H	71.94556	71.46086	73.36077	-1800.04429	-1361.53473	1526.34643	1
C	71.91237	69.82927	74.82460	726.08972	-253.19732	-179.64713	1
H	72.75497	69.23934	74.41043	1224.27271	-456.51798	-2390.90304	1
H	71.78704	69.60796	75.90368	1206.40339	-1150.93514	-2154.90344	1
N	70.64819	69.22060	74.13368	329.70796	-633.68968	-155.67358	1
H	70.49416	68.18158	74.28258	1348.31305	1938.76845	1359.00643	1
H	69.70046	69.61611	74.40257	2096.92035	1446.54551	-966.48890	1
H	70.62427	69.28514	73.07518	-1197.07217	-2303.53719	827.28733	1
C	75.93365	72.52163	74.21556	-378.01186	-556.17301	-413.96481	1
O	76.35431	71.79138	75.08614	-409.44433	-307.55053	-453.30833	1
N	75.91204	72.24871	72.86437	-429.02477	524.70884	274.08502	1
H	75.49225	73.12454	72.51383	1706.34263	-1425.96036	1574.38926	1
C	77.20026	71.90929	72.28002	-45.25864	-626.22952	478.93267	1
H	77.63074	72.90911	72.04718	1988.63164	570.90773	1772.87287	1
C	78.07795	71.08463	73.28126	551.77128	-20.49526	-564.54996	1
H	78.42534	71.73251	74.12121	1434.61535	-1753.10253	1513.99471	1
H	77.46967	70.28932	73.77295	-714.67738	1819.09770	1898.36494	1
C	79.29150	70.47439	72.49168	446.99263	-555.77305	338.97221	1
H	78.93704	69.89636	71.60917	1953.68466	1892.25957	-161.67766	1
H	79.95272	71.27162	72.08534	-403.03785	2688.07032	-188.43591	1
C	80.08062	69.53532	73.48517	567.86262	335.74681	434.04421	1
H	80.50348	70.12060	74.33389	-1080.02275	1568.26081	-1948.78900	1
H	79.40009	68.78234	73.94621	-919.54108	2111.34692	1456.13463	1
C	81.20103	68.83678	72.67616	195.81897	173.33862	-745.11536	1
H	80.84145	68.09448	71.93317	1405.67949	-2301.21089	-390.08113	1
H	81.93854	69.52463	72.20410	-940.37328	-1118.32446	2299.72133	1
N	82.03274	67.97664	73.68200	319.26422	656.36007	-42.02424	1
H	82.83411	67.44269	73.23762	1961.03633	619.91968	1787.09422	1
H	82.52767	68.48497	74.47147	854.14343	-2156.80752	1429.08711	1
H	81.52628	67.19934	74.19862	1237.02905	-1378.50857	-1998.28500	1
C	76.81633	71.06685	70.93829	532.53319	-268.57236	517.55729	1
O	76.81193	71.62840	69.86940	266.16714	-290.73804	-558.88002	1
N	76.57747	69.77067	71.22163	-226.25352	640.49678	-270.32839	1
H	76.57032	69.57683	72.22415	2007.69063	-1035.27229	1523.50231	1
C	75.61676	69.05356	70.43229	-540.76542	-573.33303	49.50000	1
H	75.44053	68.13115	71.02762	-2374.20950	-1036.76023	843.75774	1
C	74.31048	69.96006	70.40691	-77.09082	666.87422	-415.84136	1
H	74.60107	70.92973	69.91667	-985.81834	617.19825	2463.91927	1
O	73.79639	70.30787	71.72070	293.29899	-391.65602	-477.80926	1
H	74.35702	71.07445	71.93399	2528.72714	-681.76262	-751.27122	1
C	73.29911	69.16956	69.50404	252.47363	-605.14473	440.05134	1
H	72.36821	69.75650	69.32223	-2052.65063	1456.49051	-1043.51830	1
H	73.74567	68.91844	68.49945	-2152.98800	-1661.97574	-161.70652	1
H	72.99263	68.20982	69.98313	-473.12726	-1584.27339	2165.61725	1

195	C	76.31824	68.71908	68.99988	360.36307	488.46713	505.10056	1
196	O	75.65784	68.67956	67.97923	-200.08817	-528.03552	-385.82850	1
197	N	77.58398	68.37706	69.19601	-597.54095	-226.44775	355.20479	1
198	H	77.89838	68.44948	70.16379	2179.23655	-1300.70981	-991.33835	1
199	C	78.46282	67.97788	68.16959	-687.68921	297.39807	-249.45179	1
200	H	79.32469	67.53232	68.71523	-349.16256	-2467.37713	-1101.72831	1
201	C	77.74312	67.01282	67.11365	672.47345	361.54328	-201.62880	1
202	H	76.80032	67.49550	66.73867	1541.41502	582.18425	2169.97026	1
203	H	78.41459	66.88236	66.23030	-2534.67717	60.25153	997.72295	1
204	C	77.43938	65.66643	67.78455	574.26774	-389.43119	-377.02542	1
205	C	78.49239	64.73511	68.02880	688.82679	-287.33580	-257.96133	1
206	H	79.53416	64.97074	67.74407	331.14182	1470.38163	2269.80013	1
207	C	76.09813	65.36734	68.16969	-383.52382	-675.04808	144.24917	1
208	H	75.28834	66.09941	67.99345	4.09847	1517.59662	2262.86397	1
209	C	78.17496	63.52089	68.63604	-134.76730	428.45285	-649.50303	1
210	H	78.98666	62.79697	68.82555	-903.23433	1600.49020	2011.53387	1
211	C	75.83071	64.13481	68.76996	-335.53281	-615.00040	-364.39058	1
212	H	74.78899	63.90703	69.05608	1596.45398	-1369.33862	1732.02659	1
213	C	76.84895	63.16951	69.02261	585.48547	-340.24194	-406.23938	1
214	O	76.58975	61.91467	69.63293	-419.15649	-173.04408	-511.94141	1
215	H	75.61244	61.98306	69.74528	-1071.84350	1559.71507	-1960.13012	1
216	C	78.89349	69.24315	67.22431	291.41878	-183.13091	710.72246	1
217	O	78.79609	69.14883	66.01918	379.87695	523.47396	-222.23622	1
218	N	79.42986	70.22630	67.93848	564.30591	373.09380	277.24371	1
219	H	79.44260	70.13444	68.95410	-120.59931	-2436.07824	-1214.35098	1
220	C	79.75677	71.43060	67.24749	404.07514	-299.37480	608.84009	1
221	H	79.82087	71.11861	66.17752	-1071.07882	-841.69025	2359.66467	1
222	C	78.54656	72.42285	67.52178	-683.12807	68.71770	-390.13096	1
223	H	77.58321	71.95348	67.21771	2068.88918	1596.58590	770.89603	1
224	H	78.42857	72.64067	68.60883	1379.86429	2342.49779	-179.85638	1
225	C	78.83556	73.71687	66.70283	-176.39967	537.49466	550.97268	1
226	H	79.79346	74.18420	67.02469	-396.28051	1942.22168	1869.33314	1
227	H	78.96545	73.48050	65.62313	-94.44700	586.00678	-2659.20016	1
228	C	77.60442	74.71058	66.92429	-291.69381	-718.18079	-150.72929	1
229	H	76.60052	74.32192	66.61640	-2303.38776	1089.76242	964.62032	1
230	H	77.50965	75.13002	67.95831	884.16729	2568.80533	-207.74283	1
231	N	77.94263	75.75933	66.05510	-594.49522	-252.81478	342.29276	1
232	H	78.85445	75.71908	65.62639	-1735.17426	2084.05705	-263.71605	1
233	C	77.18766	76.74388	65.77818	595.23177	-271.52487	442.22352	1
234	N	75.92910	76.78972	66.38765	585.53813	-424.79750	-105.81303	1
235	H	75.50376	76.13646	67.04426	959.88797	1327.56823	-2177.11905	1
236	H	75.23652	77.52514	66.24367	1578.48647	16.27378	-2220.76233	1
237	N	77.81004	77.61921	64.87684	-452.17763	-390.33541	-421.51853	1
238	H	78.72391	77.57922	64.42799	470.26107	1974.25629	-1817.92087	1
239	H	77.36442	78.46712	64.52426	1856.67307	-1573.97321	-1224.35416	1
240	C	81.16105	72.01303	67.84950	-531.47271	-581.45780	-55.06206	1
241	O	82.14660	72.06448	67.12552	-243.00837	192.73507	609.52485	1
242	N	81.05308	72.43184	69.09745	343.49288	-644.25607	38.09164	1
243	H	80.15686	72.23287	69.53407	-1691.16043	-1535.97525	-1484.73099	1
244	C	82.21221	72.94737	69.81925	542.66492	350.14337	454.42563	1
245	H	82.76521	73.46580	68.99920	1209.57979	-2396.95920	463.86744	1
246	C	81.74837	73.85469	70.94741	-737.14309	145.93010	242.71942	1
247	H	81.14685	73.24369	71.65639	-1050.18303	159.36419	-2509.06125	1
248	C	82.93240	74.42457	71.70620	-706.77696	-252.88343	-245.16230	1
249	H	82.60578	75.07248	72.56567	-1624.64039	-1665.49085	1417.86935	1
250	H	83.57725	73.60056	72.12770	2566.10521	598.51109	693.22305	1
251	H	83.58667	75.05513	71.04264	-1972.69758	107.10018	-1876.34312	1
252	C	80.86175	74.97564	70.43695	412.85148	672.58832	-27.69222	1
253	H	80.49142	75.62867	71.27350	1447.02277	1762.76533	-1490.78944	1
254	H	81.41192	75.63940	69.71458	1320.77379	-145.20455	2378.68491	1
255	H	79.95994	74.57631	69.89593	-1512.10848	1470.52031	1724.75147	1
256	C	83.05882	71.61491	70.36310	-607.11113	499.56689	73.73769	1

```
O    83.22287  71.42117  71.53740      357.97126      460.81534      356.68785          1
N    83.51792  70.96897  69.30707      536.55407     -368.62270      332.76306          1
H    83.26083  71.32721  68.36342      524.38866     2297.69985     1367.20941          1
C    84.07205  69.67012  69.43173       46.50277     -774.29190     -147.97904          1
H    84.37111  69.42340  68.38896     1676.51875     1883.29066     1032.55549          1
C    85.33000  69.72520  70.44461     -559.41456      -34.19198     -556.30433          1
H    84.96524  70.04571  71.45257      -64.66664      395.18769     2695.05373          1
H    85.78246  68.71502  70.58461    -2703.44278      -60.40056      333.79418          1
C    86.35281  70.69796  69.92686     -519.76435      377.31045      459.42366          1
H    85.82557  71.64012  69.65310    -1746.74323     1667.50644    -1261.73691          1
C    87.38077  71.01604  71.00656      570.95554       16.46261     -545.27700          1
H    88.13794  71.76139  70.65077     2391.58273       28.05277    -1305.07271          1
H    86.89862  71.44337  71.92322      175.75588      917.63458     2559.43867          1
H    87.94258  70.09999  71.32338    -1063.97851    -2155.34650     1283.00462          1
C    87.06904  70.16231  68.69401      193.76014        2.62946     -765.53141          1
H    87.84115  70.88320  68.31999      507.39394     -846.55087     2539.60089          1
H    87.59541  69.19696  68.90921     1337.57841     1818.19054     1526.01994          1
H    86.35849  69.97263  67.84924     1100.88635    -1172.91350    -2199.08909          1
C    82.94606  68.66221  69.98014     -549.21115      475.64486     -309.38218          1
O    81.76680  68.90987  70.00801      556.06106     -390.37755      -78.24250          1
O    83.40385  67.37905  70.37164     -572.27940     -310.19032     -209.75862          1
H    84.36135  67.47863  70.11653    -1380.08193     2212.14486      790.85944          1
```

Listing 45: C00045.xmolout

```
      276
  150.0000  150.0000  150.0000     0.0000     0.0000     0.0000***********    1.7062     6825
      0.2500     300.00    0.00    0.00    0.00   -1293.7502       0.0000     300.0000
H    79.81510  83.67426  78.64968    -2043.14101    -1757.64893      399.89339          1
N    80.48079  83.44081  77.91540      426.75047      490.45733     -334.42467          1
H    80.78883  82.51091  78.19175      442.25559    -1110.30854    -2448.52907          1
C    81.52316  84.37474  77.73452      415.32972     -666.39213       83.73172          1
H    81.01395  85.35656  77.82982      879.32219     2491.00447      667.35213          1
C    82.67335  84.13389  78.83060       31.35357      477.41777      628.23362          1
H    83.10774  83.11525  78.68514     1168.38489     2240.25337    -1019.71136          1
H    83.51399  84.84836  78.65774     -360.43770     2534.90498      931.67112          1
C    82.13054  84.28012  80.23128      463.18208      512.70242     -382.34388          1
H    81.24365  83.61705  80.33979     1725.29117     1863.52247     -987.07854          1
C    83.17679  83.84821  81.25085      710.42801     -144.27427     -313.15367          1
H    82.78808  83.92251  82.29940      364.20195    -1033.13725     2494.72524          1
H    83.49955  82.78707  81.09011     2567.61904      335.80679      847.48802          1
H    84.09619  84.48638  81.19296    -2587.99029      680.92867     -512.16411          1
C    81.70459  85.71359  80.51542      268.17616      741.64379      -40.42634          1
H    81.31824  85.82990  81.56110     1283.54314    -1945.37420    -1411.27812          1
H    82.55978  86.42828  80.39713    -1258.57796     1317.15249    -2026.02085          1
H    80.89146  86.05181  79.82287    -2502.84526      909.80597      575.92391          1
C    82.15241  84.09692  76.24359      619.36578     -458.64843     -172.09273          1
O    83.19054  83.49314  76.10104      -81.40433      433.87611      522.34594          1
N    81.33807  84.61524  75.33173      175.23996     -622.20190      341.55523          1
H    80.52245  85.11651  75.68322     -876.57256     -303.22879    -2561.90213          1
C    81.63483  84.59875  73.94445     -571.88214     -534.10783     -106.15033          1
H    80.80131  85.19364  73.50824    -2713.20841      -16.25800     -248.80809          1
C    83.06028  85.31737  73.79223      166.60354      288.16399     -716.09568          1
H    83.82831  84.60890  74.21454    -2097.85574     1636.78449     -586.18044          1
O    83.21004  86.56041  74.50296     -317.80580     -363.38227      484.43082          1
H    83.87091  86.30483  75.17809     1863.74748    -1451.84923     1357.29467          1
C    83.24378  85.44418  72.23616      503.80731      517.99032      318.51606          1
H    84.24054  85.87374  71.98085      640.61020    -2157.10410    -1536.29104          1
H    83.16795  84.45312  71.71889     1687.88455      465.60692     2087.56452          1
H    82.47384  86.11551  71.78936    -1293.07895    -1899.88581    -1463.57452          1
C    81.66085  83.05908  73.40927     -153.07550     -733.68902      248.70981          1
O    82.61655  82.71742  72.75732      186.15459     -480.78787     -449.34387          1
```

N	80.55510	82.35355	73.68671	-487.53139	543.18517	42.03850	1
C	80.42589	80.98994	73.26950	-699.35871	69.34393	360.10701	1
H	81.44086	80.52928	73.29481	2.81126	-221.99807	-2715.58072	1
C	79.37033	80.43228	74.32309	-507.46439	-592.59194	122.07761	1
H	78.86834	79.50001	73.98005	-1975.08116	525.87560	-1801.71556	1
H	79.84766	80.20141	75.30127	1588.27743	-1913.22100	1113.83566	1
C	78.40075	81.64710	74.43264	-613.62442	-457.55993	-194.14490	1
H	77.74794	81.71285	73.53222	1707.54637	-721.27992	1996.92523	1
H	77.72538	81.60553	75.31664	758.23357	2100.81767	1560.55011	1
C	79.44611	82.84466	74.49252	-352.41524	704.96076	49.21465	1
H	79.09577	83.81052	74.05896	1714.76886	-2070.42778	443.35792	1
H	79.87782	83.02908	75.50534	1224.15040	1801.50881	1636.97633	1
C	79.71829	80.91930	71.79727	566.85117	537.05831	117.62814	1
O	79.06537	81.82023	71.33976	-550.26779	-406.08867	4.21666	1
N	79.81070	79.64288	71.37857	566.34832	-425.77725	180.18749	1
H	80.50038	79.08613	71.88840	644.44502	2646.80550	-52.73430	1
C	79.72704	79.41644	69.94860	612.34801	-281.48662	411.56221	1
H	80.42092	80.15858	69.49578	1667.23498	-1490.75142	1556.16753	1
C	80.19249	77.91123	69.74377	-714.45746	-175.43074	286.98927	1
H	79.36449	77.22842	70.07965	-787.09484	1763.03619	-1922.46060	1
O	81.36051	77.50532	70.49314	-429.46514	516.51932	-128.40526	1
H	81.00591	76.75003	71.00060	1981.82785	436.16847	-1818.18182	1
C	80.32399	77.83564	68.17800	630.79189	-468.94448	76.03230	1
H	80.53630	76.79591	67.83637	-1775.71861	2062.30353	131.89849	1
H	79.39154	78.17321	67.66316	-1855.14608	88.94118	1993.53760	1
H	81.15703	78.47899	67.81027	-1504.82350	-780.94249	-2132.90992	1
C	78.19870	79.61535	69.46576	288.57638	618.62814	-397.00270	1
O	77.94825	79.76895	68.29374	321.70495	-185.26452	574.37218	1
N	77.31524	79.39399	70.47488	-447.05792	-446.53431	367.76425	1
H	77.79577	79.33978	71.37642	350.31316	1975.63644	-1843.31522	1
C	76.17979	80.30800	70.45473	-511.23061	374.03917	-471.51480	1
H	76.56964	81.26659	70.04248	2234.79579	-898.70890	-1273.45190	1
C	75.13713	79.61316	69.45885	529.53781	-159.34528	563.72604	1
H	74.30802	80.34214	69.25159	654.59388	2347.94251	1217.51488	1
H	75.69357	79.46541	68.49263	-1526.55661	2103.42026	817.87394	1
O	74.54367	78.36863	69.91244	-554.84278	14.12789	399.58769	1
H	75.33063	77.79303	69.85282	1297.06236	2061.84854	1220.68812	1
C	75.64942	80.42218	71.95285	-662.15484	-412.13544	123.62687	1
O	75.99650	79.64830	72.86173	-540.01537	-317.05804	274.91456	1
N	74.80093	81.42537	72.03314	-202.76299	-690.00100	-131.49145	1
H	74.59068	81.92487	71.17008	-702.62132	-2617.37191	281.70413	1
C	74.21369	81.80860	73.29811	-491.73914	576.34187	222.73509	1
H	74.78651	81.21843	74.05567	1977.17978	-1823.58850	-434.68928	1
C	74.33889	83.32470	73.42542	-649.50797	-328.66843	306.11224	1
H	73.83238	83.77893	72.54500	2075.07803	1452.16490	-1004.45904	1
C	73.65249	83.83047	74.67897	-615.18735	-489.99689	-70.96352	1
H	73.69916	84.95227	74.75572	-1226.32661	-1724.38906	-1716.47170	1
H	72.56537	83.53711	74.70123	2381.64273	-969.84735	900.46860	1
H	74.12822	83.41160	75.60881	2122.74991	1702.23946	-141.36228	1
C	75.78983	83.76549	73.39803	-501.86074	-479.61276	-376.42482	1
H	75.88747	84.88588	73.37407	993.79261	-2246.95401	-1177.81305	1
H	76.34858	83.40207	74.30413	42.71279	-626.15391	-2651.37258	1
H	76.32639	83.36135	72.49446	-2026.39012	-1590.33709	-887.82922	1
C	72.58661	81.39750	73.22030	-672.89153	382.57394	-156.34072	1
O	71.85517	81.85436	72.37787	410.04627	221.86223	500.35950	1
N	72.29741	80.61157	74.24178	-378.11782	-443.79414	-441.10984	1
H	73.05063	80.35371	74.87578	2404.15973	-568.14894	1149.30085	1
C	70.98747	80.07892	74.36761	-544.54484	466.16019	331.29119	1
H	70.41312	80.52460	73.52431	-1409.80988	-170.17955	2325.32679	1
C	71.16652	78.50329	74.27742	-184.48284	116.78282	-758.89137	1
H	71.68772	78.26135	73.32403	1893.78641	1861.14652	611.04528	1
H	71.83455	78.13568	75.08890	-2170.79665	-974.19311	1327.50117	1

C	69.78587	77.78159	74.34177	389.51668	672.81878	-138.49152	1
H	69.35345	77.85477	75.36771	510.64577	1933.03327	1851.02484	1
H	69.04656	78.30464	73.69549	-2302.30869	-1105.72939	-948.89774	1
C	69.87230	76.26613	73.91154	-559.93427	-126.34590	542.30857	1
O	68.88016	75.58308	73.90702	-670.36003	-111.83166	76.36448	1
N	71.11434	75.85860	73.56822	-216.06729	-537.07037	446.51379	1
H	71.97530	76.40994	73.48759	-1251.96487	1074.12894	-2168.52500	1
H	71.32685	74.93354	73.17933	-2451.83023	-720.73851	944.84612	1
C	70.46882	80.48972	75.86972	546.24052	-563.88337	-85.11809	1
O	70.98602	79.97820	76.83045	71.70702	-307.40463	606.69673	1
N	69.46314	81.37589	75.81811	479.90863	-504.50129	222.86849	1
C	68.98147	82.04572	76.98902	75.51268	-600.91904	506.73700	1
H	69.87092	82.24598	77.62961	-2033.61562	1795.30103	254.89480	1
C	68.26697	83.33288	76.39230	523.79067	517.99274	284.45581	1
H	67.49866	83.75429	77.07945	1599.61665	-1127.00911	1895.98173	1
H	68.99463	84.14930	76.18541	-1881.83093	1275.82488	-1501.55016	1
C	67.66652	82.75928	75.07158	-600.05639	-404.05026	316.64485	1
H	66.74505	82.16974	75.27653	-2597.05260	496.36221	657.73249	1
H	67.38555	83.54221	74.33157	-1666.24518	1294.38985	-1723.90591	1
C	68.85272	81.82455	74.57454	493.57570	-129.95376	-602.56394	1
H	68.53470	80.92613	73.99493	-1469.08415	-637.84797	2204.22581	1
H	69.65988	82.35878	74.01758	-1038.97840	1068.88701	-2280.71789	1
C	67.87176	81.12447	77.75756	188.88100	241.05089	-727.87821	1
O	67.06227	81.64281	78.49497	531.88613	-49.19943	427.07960	1
N	68.00351	79.83879	77.43208	708.14833	26.93417	-179.73509	1
H	68.80397	79.62870	76.82545	-2232.69501	-1179.09831	1023.94816	1
C	67.56242	78.87015	78.39755	-738.32412	259.92548	104.42456	1
H	67.62318	79.38599	79.38113	2028.89123	1260.02983	1311.33324	1
C	66.06845	78.50553	78.00271	-258.65678	-345.52513	661.28459	1
H	65.90823	78.68417	76.91760	-1826.06090	-1801.46097	-918.64554	1
C	65.73719	77.02260	78.38570	-541.23400	531.92970	-218.41428	1
H	64.66967	76.77308	78.18003	1311.73218	-2219.18200	882.19023	1
H	66.36485	76.30511	77.80277	-1048.71298	1223.58118	-2196.97971	1
H	65.92481	76.82556	79.46729	419.06414	1903.92898	1903.44670	1
C	65.12763	79.40830	78.87244	-423.86431	152.66566	-648.55262	1
H	64.06817	79.36183	78.52603	105.68386	-2342.50080	1387.51238	1
H	65.13944	79.09954	79.94449	-1695.63603	-1484.73621	-1531.02793	1
H	65.45879	80.48715	78.83400	-1853.57121	-487.39031	1936.59343	1
C	68.53624	77.57320	78.24368	539.71428	-510.58232	267.58633	1
O	68.50882	76.86203	77.24338	-15.93758	-278.34085	-624.49402	1
N	69.24319	77.45293	79.34472	-676.81886	12.05292	276.17960	1
H	69.11775	78.18674	80.04184	-1694.44250	-373.84189	-2100.66098	1
C	69.95375	76.27482	79.68984	412.33078	-664.70360	108.35451	1
H	70.28686	76.46963	80.73388	1765.77476	1294.44366	1621.76594	1
C	68.94217	75.06113	79.56358	-179.66635	-721.95667	-264.73915	1
H	68.04803	75.20808	80.20962	-607.34922	2587.87032	-598.10018	1
H	68.54602	74.98459	78.52494	-359.69332	-1479.13954	-2259.74260	1
C	69.73032	73.76777	79.95615	-240.01076	616.02425	431.85322	1
H	70.71152	73.73235	79.42425	-1359.17131	782.54425	2227.99181	1
H	69.96847	73.75379	81.04354	-820.36667	85.81767	-2596.78715	1
C	68.84993	72.52570	79.54270	-513.07015	236.18570	-551.87280	1
H	67.89907	72.49016	80.12194	1816.11944	-1183.11722	-1650.94321	1
H	68.55425	72.58698	78.47008	-1800.04429	-1361.53473	1526.34643	1
C	69.71971	71.26184	79.79579	726.08972	-253.19732	-179.64713	1
H	70.66984	71.24231	79.22001	1224.27271	-456.51798	-2390.90304	1
H	69.92036	71.04452	80.86595	1206.40339	-1150.93514	-2154.90344	1
N	68.92269	70.06329	79.26543	329.70796	-633.68968	-155.67358	1
H	69.37277	69.12372	79.43415	1348.31305	1938.76845	1359.00643	1
H	67.96991	69.89414	79.68563	2096.92035	1446.54551	-966.48890	1
H	68.75055	70.08316	78.11416	-1197.07217	-2303.53719	827.28733	1
C	71.22821	75.99142	78.68401	-378.01186	-556.17301	-413.96481	1
O	72.33404	76.11059	79.14375	-409.44433	-307.55053	-453.30833	1

N	70.84518	75.64678	77.46473	-429.02477	524.70884	274.08502	1
H	69.84173	75.76610	77.17490	1706.34263	-1425.96036	1574.38926	1
C	71.71563	74.89736	76.62719	-45.25864	-626.22952	478.93267	1
H	71.20864	74.96814	75.63963	1988.63164	570.90773	1772.87287	1
C	73.13002	75.60150	76.69326	551.77128	-20.49526	-564.54996	1
H	72.99130	76.69972	76.81851	1434.61535	-1753.10253	1513.99471	1
H	73.70138	75.27146	77.59445	-714.67738	1819.09770	1898.36494	1
C	73.91969	75.27794	75.37970	446.99263	-555.77305	338.97221	1
H	74.39166	74.27023	75.43263	1953.68466	1892.25957	-161.67766	1
H	73.23711	75.24513	74.50332	-403.03785	2688.07032	-188.43591	1
C	74.99890	76.41764	75.19837	567.86262	335.74681	434.04421	1
H	74.52102	77.42419	75.22619	-1080.02275	1568.26081	-1948.78900	1
H	75.72460	76.40172	76.04981	-919.54108	2111.34692	1456.13463	1
C	75.73007	76.20469	73.84691	195.81897	173.33862	-745.11536	1
H	76.24846	75.22787	73.76330	1405.67949	-2301.21089	-390.08113	1
H	75.10126	76.37487	72.94812	-940.37328	-1118.32446	2299.72133	1
N	76.85831	77.25785	73.74465	319.26422	656.36007	-42.02424	1
H	77.57055	77.06056	72.98521	1961.03633	619.91968	1787.09422	1
H	76.53240	78.31893	73.53205	854.14343	-2156.80752	1429.08711	1
H	77.49674	77.33304	74.58584	1237.02905	-1378.50857	-1998.28500	1
C	71.83172	73.34618	77.16109	532.53319	-268.57236	517.55729	1
O	72.42096	73.02310	78.16587	266.16714	-290.73804	-558.88002	1
N	71.13652	72.54963	76.33690	-226.25352	640.49678	-270.32839	1
H	70.76587	73.01187	75.50602	2007.69063	-1035.27229	1523.50231	1
C	71.66416	71.20290	76.22483	-540.76542	-573.33303	49.50000	1
H	72.00868	70.93438	77.25207	-2374.20950	-1036.76023	843.75774	1
C	70.53861	70.29663	75.66509	-77.09082	666.87422	-415.84136	1
H	70.01584	70.76868	74.80676	-985.81834	617.19825	2463.91927	1
O	69.23875	70.08246	76.68861	293.29899	-391.65602	-477.80926	1
H	68.62027	70.64641	76.22888	2528.72714	-681.76262	-751.27122	1
C	71.13702	68.92400	75.30270	252.47363	-605.14473	440.05134	1
H	70.36355	68.24741	74.86134	-2052.65063	1456.49051	-1043.51830	1
H	71.97051	69.01391	74.55989	-2152.98800	-1661.97574	-161.70652	1
H	71.55273	68.41284	76.20670	-473.12726	-1584.27339	2165.61725	1
C	72.90841	71.27359	75.13755	360.36307	488.46713	505.10056	1
O	72.93296	72.05248	74.21654	-200.08817	-528.03552	-385.82850	1
N	73.74611	70.28895	75.43917	-597.54095	-226.44775	355.20479	1
H	73.53907	69.72238	76.26010	2179.23655	-1300.70981	-991.33835	1
C	74.84062	70.03714	74.58489	-687.68921	297.39807	-249.45179	1
H	74.78866	70.83355	73.80764	-349.16256	-2467.37713	-1101.72831	1
C	76.16762	70.07021	75.48238	672.47345	361.54328	-201.62880	1
H	76.11194	69.30105	76.28584	1541.41502	582.18425	2169.97026	1
H	77.05652	69.82061	74.85849	-2534.67717	60.25153	997.72295	1
C	76.28867	71.47674	76.08536	574.26774	-389.43119	-377.02542	1
C	77.00281	72.49433	75.38463	688.82679	-287.33580	-257.96133	1
H	77.48223	72.27815	74.41291	331.14182	1470.38163	2269.80013	1
C	75.64756	71.77541	77.32556	-383.52382	-675.04808	144.24917	1
H	75.06643	71.00053	77.85705	4.09847	1517.59662	2262.86397	1
C	77.08802	73.76565	75.95491	-134.76730	428.45285	-649.50303	1
H	77.65049	74.54799	75.41657	-903.23433	1600.49020	2011.53387	1
C	75.75437	73.06601	77.85157	-335.53281	-615.00040	-364.39058	1
H	75.24517	73.28198	78.80773	1596.45398	-1369.33862	1732.02659	1
C	76.48477	74.10201	77.20094	585.48547	-340.24194	-406.23938	1
O	76.61176	75.41718	77.73558	-419.15649	-173.04408	-511.94141	1
H	76.10525	75.30091	78.57303	-1071.84350	1559.71507	-1960.13012	1
C	74.72359	68.51228	73.97669	291.41878	-183.13091	710.72246	1
O	74.77887	68.36756	72.77664	379.87695	523.47396	-222.23622	1
N	74.53273	67.60078	74.92267	564.30591	373.09380	277.24371	1
H	74.48559	67.96430	75.87313	-120.59931	-2436.07824	-1214.35098	1
C	74.86264	66.22892	74.71450	404.07514	-299.37480	608.84009	1
H	74.33544	65.68740	75.53113	-1071.07882	-841.69025	2359.66467	1
C	74.36306	65.85888	73.25939	-683.12807	68.71770	-390.13096	1

```
H   73.48364  66.49873  73.01180     2068.88918     1596.58590      770.89603          1
H   75.14019  66.13872  72.50944     1379.86429     2342.49779     -179.85638          1
C   73.99217  64.35099  73.11025     -176.39967      537.49466      550.97268          1
H   74.88894  63.69395  73.15800     -396.28051     1942.22168     1869.33314          1
H   73.31824  64.00972  73.92667      -94.44700      586.00678    -2659.20016          1
C   73.26814  64.26452  71.68469     -291.69381     -718.18079     -150.72929          1
H   72.36803  64.91642  71.55635    -2303.38776     1089.76242      964.62032          1
H   73.96243  64.44491  70.82437      884.16729     2568.80533     -207.74283          1
N   72.85160  62.93113  71.61039     -594.49522     -252.81478      342.29276          1
H   73.39207  62.26513  72.14090    -1735.17426     2084.05705     -263.71605          1
C   71.88103  62.50563  70.90722      595.23177     -271.52487      442.22352          1
N   71.15066  63.42923  70.15194      585.53813     -424.79750     -105.81303          1
H   71.28327  64.43363  70.04261      959.88797     1327.56823    -2177.11905          1
H   70.36596  63.19149  69.54433     1578.48647       16.27378    -2220.76233          1
N   71.73666  61.11726  71.03510     -452.17763     -390.33541     -421.51853          1
H   72.26714  60.43477  71.57432      470.26107     1974.25629    -1817.92087          1
H   71.01724  60.58603  70.54278     1856.67307    -1573.97321    -1224.35416          1
C   76.48557  66.05806  74.80792     -531.47271     -581.45780      -55.06206          1
O   77.28247  66.96814  74.76144     -243.00837      192.73507      609.52485          1
N   76.79304  64.72586  74.97814      343.49288     -644.25607       38.09164          1
H   75.85136  64.31259  74.94398    -1691.16043    -1535.97525    -1484.73099          1
C   77.74111  64.21309  73.99926      542.66492      350.14337      454.42563          1
H   77.28506  64.41599  73.00721     1209.57979    -2396.95920      463.86744          1
C   79.10005  64.98655  74.21751     -737.14309      145.93010      242.71942          1
H   79.05350  65.57967  75.15897    -1050.18303      159.36419    -2509.06125          1
C   80.31999  63.99906  74.23534     -706.77696     -252.88343     -245.16230          1
H   81.28845  64.54197  74.33978    -1624.64039    -1665.49085     1417.86935          1
H   80.25046  63.27960  75.08511     2566.10521      598.51109      693.22305          1
H   80.37284  63.39729  73.29803    -1972.69758      107.10018    -1876.34312          1
C   79.34698  65.91588  72.97719      412.85148      672.58832      -27.69222          1
H   80.30555  66.47801  73.07116     1447.02277     1762.76533    -1490.78944          1
H   79.39433  65.33342  72.02748     1320.77379     -145.20455     2378.68491          1
H   78.53056  66.67071  72.86871    -1512.10848     1470.52031     1724.75147          1
C   77.82637  62.63005  74.32625     -607.11113      499.56689       73.73769          1
O   77.72573  61.80471  73.44407      357.97126      460.81534      356.68785          1
N   78.10496  62.46648  75.61580      536.55407     -368.62270      332.76306          1
H   78.13545  63.28227  76.25564      524.38866     2297.69985     1367.20941          1
C   77.85758  61.19934  76.24970       46.50277     -774.29190     -147.97904          1
H   76.84016  60.89218  75.92210     1676.51875     1883.29066     1032.55549          1
C   78.97079  60.10565  75.83734     -559.41456      -34.19198     -556.30433          1
H   79.90779  60.62306  75.52055      -64.66664      395.18769     2695.05373          1
H   79.24701  59.46817  76.71128    -2703.44278      -60.40056      333.79418          1
C   78.41698  59.25734  74.72733     -519.76435      377.31045      459.42366          1
H   78.01041  59.95316  73.93086    -1746.74323     1667.50644    -1261.73691          1
C   79.51134  58.39632  74.10919      570.95554       16.46261     -545.27700          1
H   79.11506  57.76289  73.27389     2391.58273       28.05277    -1305.07271          1
H   80.34093  59.02045  73.68760      175.75588      917.63458     2559.43867          1
H   79.96613  57.70314  74.86313    -1063.97851    -2155.34650     1283.00462          1
C   77.29942  58.35363  75.23141      193.76014        2.62946     -765.53141          1
H   76.86665  57.73294  74.40515      507.39394     -846.55087     2539.60089          1
H   77.66709  57.64599  76.01864     1337.57841     1818.19054     1526.01994          1
H   76.45661  58.93745  75.68185     1100.88635    -1172.91350    -2199.08909          1
C   77.96341  61.63438  77.77407     -549.21115      475.64486     -309.38218          1
O   78.10419  62.80561  78.06622      556.06106     -390.37755      -78.24250          1
O   77.86954  60.64843  78.77202     -572.27940     -310.19032     -209.75862          1
H   77.73779  59.84515  78.18968    -1380.08193     2212.14486      790.85944          1
```

Listing 46: C00046.xmolout

```
      276
  150.0000  150.0000  150.0000     0.0000     0.0000     0.0000***********    0.7550     3020
       0.2500     300.00    0.00    0.00    0.00   -1293.7445       0.0000     300.0000
```

H	87.38199	78.63726	71.23284	-2043.14101	-1757.64893	399.89339	1
N	87.26963	78.73093	70.22582	426.75047	490.45733	-334.42467	1
H	86.26341	78.86670	70.15689	442.25559	-1110.30854	-2448.52907	1
C	88.04032	79.77897	69.65540	415.32972	-666.39213	83.73172	1
H	89.03757	79.55975	70.09902	879.32219	2491.00447	667.35213	1
C	88.03448	79.66078	68.04247	31.35357	477.41777	628.23362	1
H	87.03891	79.97152	67.64606	1168.38489	2240.25337	-1019.71136	1
H	88.77469	80.37219	67.60513	-360.43770	2534.90498	931.67112	1
C	88.33299	78.24841	67.62728	463.18208	512.70242	-382.34388	1
H	87.57039	77.58373	68.09375	1725.29117	1863.52247	-987.07854	1
C	88.23856	78.12170	66.11063	710.42801	-144.27427	-313.15367	1
H	88.38582	77.06391	65.77328	364.20195	-1033.13725	2494.72524	1
H	87.24087	78.45825	65.72836	2567.61904	335.80679	847.48802	1
H	89.01618	78.74424	65.59809	-2587.99029	680.92867	-512.16411	1
C	89.71118	77.79236	68.08808	268.17616	741.64379	-40.42634	1
H	89.93384	76.74784	67.74934	1283.54314	-1945.37420	-1411.27812	1
H	90.52199	78.45225	67.68574	-1258.57796	1317.15249	-2026.02085	1
H	89.79652	77.80123	69.20505	-2502.84526	909.80597	575.92391	1
C	87.42201	81.12261	70.23958	619.36578	-458.64843	-172.09273	1
O	87.27320	81.29788	71.50661	-81.40433	433.87611	522.34594	1
N	87.09350	82.00872	69.35253	175.23996	-622.20190	341.55523	1
H	87.26665	81.85438	68.36306	-876.57256	-303.22879	-2561.90213	1
C	86.75403	83.28795	69.90692	-571.88214	-534.10783	-106.15033	1
H	87.66148	83.90490	69.74573	-2713.20841	-16.25800	-248.80809	1
C	85.50300	83.83748	69.12791	166.60354	288.16399	-716.09568	1
H	84.65696	83.10832	69.26384	-2097.85574	1636.78449	-586.18044	1
O	85.66451	83.98910	67.69617	-317.80580	-363.38227	484.43082	1
H	84.95992	83.40502	67.35966	1863.74748	-1451.84923	1357.29467	1
C	85.17497	85.15664	69.92823	503.80731	517.99032	318.51606	1
H	84.23427	85.62975	69.56301	640.61020	-2157.10410	-1536.29104	1
H	85.03962	84.96098	71.01985	1687.88455	465.60692	2087.56452	1
H	85.98618	85.91285	69.82434	-1293.07895	-1899.88581	-1463.57452	1
C	86.35484	83.01086	71.52722	-153.07550	-733.68902	248.70981	1
O	85.11550	82.79822	71.71878	186.15459	-480.78787	-449.34387	1
N	87.14549	83.82227	72.28988	-487.53139	543.18517	42.03850	1
C	87.21104	83.65144	73.70138	-699.35871	69.34393	360.10701	1
H	87.20376	82.54979	73.87817	2.81126	-221.99807	-2715.58072	1
C	88.55237	84.39925	74.13278	-507.46439	-592.59194	122.07761	1
H	88.46560	84.88625	75.12990	-1975.08116	525.87560	-1801.71556	1
H	89.40406	83.68737	74.20878	1588.27743	-1913.22100	1113.83566	1
C	88.77647	85.42211	72.98118	-613.62442	-457.55993	-194.14490	1
H	88.17493	86.34557	73.13509	1707.54637	-721.27992	1996.92523	1
H	89.83511	85.74665	72.87329	758.23357	2100.81767	1560.55011	1
C	88.25019	84.57250	71.73235	-352.41524	704.96076	49.21465	1
H	87.87679	85.17063	70.86423	1714.76886	-2070.42778	443.35792	1
H	88.97514	83.80431	71.36582	1224.15040	1801.50881	1636.97633	1
C	85.89679	84.35839	74.37279	566.85117	537.05831	117.62814	1
O	85.37234	83.88082	75.37143	-550.26779	-406.08867	4.21666	1
N	85.60187	85.48439	73.75584	566.34832	-425.77725	180.18749	1
H	86.14575	85.70303	72.92474	644.44502	2646.80550	-52.73430	1
C	84.51868	86.26090	74.26756	612.34801	-281.48662	411.56221	1
H	84.57627	86.02336	75.35630	1667.23498	-1490.75142	1556.16753	1
C	84.73307	87.79548	73.88532	-714.45746	-175.43074	286.98927	1
H	84.56234	87.92297	72.78175	-787.09484	1763.03619	-1922.46060	1
O	86.03436	88.33825	74.16687	-429.46514	516.51932	-128.40526	1
H	86.31082	88.64333	73.27930	1981.82785	436.16847	-1818.18182	1
C	83.53231	88.43747	74.67510	630.79189	-468.94448	76.03230	1
H	83.44605	89.52970	74.47181	-1775.71861	2062.30353	131.89849	1
H	82.55832	87.96693	74.39607	-1855.14608	88.94118	1993.53760	1
H	83.65917	88.31620	75.77614	-1504.82350	-780.94249	-2132.90992	1
C	83.15972	85.65156	73.58014	288.57638	618.62814	-397.00270	1
O	82.46148	86.29814	72.83688	321.70495	-185.26452	574.37218	1

N	83.01286	84.39503	73.98570	-447.05792	-446.53431	367.76425	1
H	83.71686	83.94841	74.60731	350.31316	1975.63644	-1843.31522	1
C	81.78128	83.76969	73.62772	-511.23061	374.03917	-471.51480	1
H	81.01577	84.55944	73.79260	2234.79579	-898.70890	-1273.45190	1
C	81.91486	83.44114	72.06321	529.53781	-159.34528	563.72604	1
H	80.87361	83.37099	71.63486	654.59388	2347.94251	1217.51488	1
H	82.39152	84.36286	71.62491	-1526.55661	2103.42026	817.87394	1
O	82.59216	82.24847	71.69538	-554.84278	14.12789	399.58769	1
H	83.57975	82.53424	71.95489	1297.06236	2061.84854	1220.68812	1
C	81.63462	82.44987	74.56741	-662.15484	-412.13544	123.62687	1
O	82.46740	81.57489	74.65195	-540.01537	-317.05804	274.91456	1
N	80.47630	82.54335	75.21487	-202.76299	-690.00100	-131.49145	1
H	79.84067	83.33413	75.09720	-702.62132	-2617.37191	281.70413	1
C	79.95286	81.33130	75.76614	-491.73914	576.34187	222.73509	1
H	80.33009	80.50226	75.12615	1977.17978	-1823.58850	-434.68928	1
C	80.50871	81.20967	77.24975	-649.50797	-328.66843	306.11224	1
H	80.19043	82.08173	77.86040	2075.07803	1452.16490	-1004.45904	1
C	79.95857	79.86799	77.84147	-615.18735	-489.99689	-70.96352	1
H	80.32262	79.69719	78.88177	-1226.32661	-1724.38906	-1716.47170	1
H	78.84281	79.86147	77.87212	2381.64273	-969.84735	900.46860	1
H	80.27635	78.99065	77.23028	2122.74991	1702.23946	-141.36228	1
C	82.07379	81.11035	77.21318	-501.86074	-479.61276	-376.42482	1
H	82.49982	80.87248	78.21609	993.79261	-2246.95401	-1177.81305	1
H	82.41846	80.31604	76.50448	42.71279	-626.15391	-2651.37258	1
H	82.53628	82.07085	76.87578	-2026.39012	-1590.33709	-887.82922	1
C	78.33480	81.57763	75.73965	-672.89153	382.57394	-156.34072	1
O	77.83718	82.64486	76.01892	410.04627	221.86223	500.35950	1
N	77.73845	80.44358	75.38721	-378.11782	-443.79414	-441.10984	1
H	78.29722	79.60489	75.22956	2404.15973	-568.14894	1149.30085	1
C	76.32001	80.38549	75.42108	-544.54484	466.16019	331.29119	1
H	75.96310	81.41490	75.65883	-1409.80988	-170.17955	2325.32679	1
C	75.87553	79.82722	73.99666	-184.48284	116.78282	-758.89137	1
H	76.21026	80.50682	73.18136	1893.78641	1861.14652	611.04528	1
H	76.36891	78.84941	73.79812	-2170.79665	-974.19311	1327.50117	1
C	74.32569	79.67133	74.00476	389.51668	672.81878	-138.49152	1
H	73.99089	79.17930	74.94395	510.64577	1933.03327	1851.02484	1
H	73.83843	80.67096	74.02625	-2302.30869	-1105.72939	-948.89774	1
C	73.80526	78.83207	72.77326	-559.93427	-126.34590	542.30857	1
O	72.70320	78.34879	72.81270	-670.36003	-111.83166	76.36448	1
N	74.68367	78.75180	71.75252	-216.06729	-537.07037	446.51379	1
H	75.61573	79.17356	71.66255	-1251.96487	1074.12894	-2168.52500	1
H	74.48315	78.29698	70.85527	-2451.83023	-720.73851	944.84612	1
C	75.94173	79.22550	76.51191	546.24052	-563.88337	-85.11809	1
O	76.50890	78.16322	76.46013	71.70702	-307.40463	606.69673	1
N	74.93103	79.61127	77.29864	479.90863	-504.50129	222.86849	1
C	74.61245	78.84770	78.47119	75.51268	-600.91904	506.73700	1
H	75.59813	78.55642	78.89953	-2033.61562	1795.30103	254.89480	1
C	73.78083	79.86433	79.35412	523.79067	517.99274	284.45581	1
H	73.00865	79.35645	79.97484	1599.61665	-1127.00911	1895.98173	1
H	74.44311	80.40494	80.06680	-1881.83093	1275.82488	-1501.55016	1
C	73.16664	80.85114	78.30992	-600.05639	-404.05026	316.64485	1
H	72.23930	80.43705	77.85566	-2597.05260	496.36221	657.73249	1
H	72.89149	81.83985	78.74197	-1666.24518	1294.38985	-1723.90591	1
C	74.34461	80.94455	77.24945	493.57570	-129.95376	-602.56394	1
H	74.02437	81.16083	76.20366	-1469.08415	-637.84797	2204.22581	1
H	75.16658	81.64361	77.53591	-1038.97840	1068.88701	-2280.71789	1
C	73.74849	77.50141	78.12156	188.88100	241.05089	-727.87821	1
O	73.93468	76.50788	78.82680	531.88613	-49.19943	427.07960	1
N	72.82034	77.68741	77.19871	708.14833	26.93417	-179.73509	1
H	72.95255	78.64126	76.85554	-2232.69501	-1179.09831	1023.94816	1
C	72.19308	76.65364	76.39213	-738.32412	259.92548	104.42456	1
H	71.72426	77.23020	75.55824	2028.89123	1260.02983	1311.33324	1

C	73.25911	75.68074	75.91430	-258.65678	-345.52513	661.28459	1
H	73.77793	75.27867	76.81498	-1826.06090	-1801.46097	-918.64554	1
C	72.64557	74.52113	75.15092	-541.23400	531.92970	-218.41428	1
H	73.41532	73.75055	74.87059	1311.73218	-2219.18200	882.19023	1
H	71.85000	73.99898	75.75226	-1048.71298	1223.58118	-2196.97971	1
H	72.16005	74.87012	74.19778	419.06414	1903.92898	1903.44670	1
C	74.29913	76.36922	75.05111	-423.86431	152.66566	-648.55262	1
H	75.05477	75.64081	74.64685	105.68386	-2342.50080	1387.51238	1
H	73.82559	76.88924	74.16814	-1695.63603	-1484.73621	-1531.02793	1
H	74.87220	77.14906	75.63848	-1853.57121	-487.39031	1936.59343	1
C	71.04498	75.80164	77.25851	539.71428	-510.58232	267.58633	1
O	69.99343	75.58786	76.71773	-15.93758	-278.34085	-624.49402	1
N	71.50328	75.37967	78.41479	-676.81886	12.05292	276.17960	1
H	72.47819	75.65150	78.70695	-1694.44250	-373.84189	-2100.66098	1
C	70.64292	74.79237	79.39235	412.33078	-664.70360	108.35451	1
H	71.08726	75.15051	80.34895	1765.77476	1294.44366	1621.76594	1
C	69.14480	75.26647	79.18594	-179.66635	-721.95667	-264.73915	1
H	69.09456	76.35452	78.95504	-607.34922	2587.87032	-598.10018	1
H	68.69705	74.75147	78.30120	-359.69332	-1479.13954	-2259.74260	1
C	68.35679	74.91708	80.49122	-240.01076	616.02425	431.85322	1
H	68.53532	73.84342	80.76644	-1359.17131	782.54425	2227.99181	1
H	68.72013	75.52082	81.35342	-820.36667	85.81767	-2596.78715	1
C	66.81621	75.16554	80.23735	-513.07015	236.18570	-551.87280	1
H	66.59522	76.25217	80.12737	1816.11944	-1183.11722	-1650.94321	1
H	66.48665	74.69000	79.28485	-1800.04429	-1361.53473	1526.34643	1
C	66.06449	74.55350	81.44272	726.08972	-253.19732	-179.64713	1
H	66.14875	73.45120	81.52434	1224.27271	-456.51798	-2390.90304	1
H	66.29790	75.02278	82.41912	1206.40339	-1150.93514	-2154.90344	1
N	64.53591	74.81747	81.23213	329.70796	-633.68968	-155.67358	1
H	63.89539	74.43363	81.98574	1348.31305	1938.76845	1359.00643	1
H	64.22400	75.83121	81.19164	2096.92035	1446.54551	-966.48890	1
H	64.09186	74.41639	80.35530	-1197.07217	-2303.53719	827.28733	1
C	70.68463	73.16745	79.30759	-378.01186	-556.17301	-413.96481	1
O	69.89307	72.52152	80.00309	-409.44433	-307.55053	-453.30833	1
N	71.61528	72.73296	78.49297	-429.02477	524.70884	274.08502	1
H	72.11376	73.49100	78.02875	1706.34263	-1425.96036	1574.38926	1
C	72.03389	71.38245	78.36968	-45.25864	-626.22952	478.93267	1
H	72.98377	71.45566	77.79382	1988.63164	570.90773	1772.87287	1
C	72.21377	70.77284	79.82099	551.77128	-20.49526	-564.54996	1
H	72.97801	71.34087	80.39791	1434.61535	-1753.10253	1513.99471	1
H	71.26791	70.86728	80.40725	-714.67738	1819.09770	1898.36494	1
C	72.62233	69.27115	79.66495	446.99263	-555.77305	338.97221	1
H	71.86827	68.71950	79.06042	1953.68466	1892.25957	-161.67766	1
H	73.58419	69.17622	79.11311	-403.03785	2688.07032	-188.43591	1
C	72.73572	68.64407	81.11255	567.86262	335.74681	434.04421	1
H	73.48840	69.19111	81.72543	-1080.02275	1568.26081	-1948.78900	1
H	71.77070	68.73810	81.66191	-919.54108	2111.34692	1456.13463	1
C	73.13334	67.15888	80.94621	195.81897	173.33862	-745.11536	1
H	72.33510	66.50900	80.53544	1405.67949	-2301.21089	-390.08113	1
H	74.07804	66.99373	80.39056	-940.37328	-1118.32446	2299.72133	1
N	73.42359	66.59434	82.37763	319.26422	656.36007	-42.02424	1
H	73.71575	65.57545	82.42166	1961.03633	619.91968	1787.09422	1
H	74.20719	67.05773	82.92382	854.14343	-2156.80752	1429.08711	1
H	72.62704	66.61687	83.07866	1237.02905	-1378.50857	-1998.28500	1
C	70.92782	70.45849	77.58284	532.53319	-268.57236	517.55729	1
O	71.34179	69.70824	76.72499	266.16714	-290.73804	-558.88002	1
N	69.72481	70.57000	78.10743	-226.25352	640.49678	-270.32839	1
H	69.61076	71.22368	78.92442	2007.69063	-1035.27229	1523.50231	1
C	68.54528	70.18425	77.41110	-540.76542	-573.33303	49.50000	1
H	67.76273	70.83794	77.85639	-2374.20950	-1036.76023	843.75774	1
C	68.72274	70.42780	75.83993	-77.09082	666.87422	-415.84136	1
H	69.56290	69.77158	75.47139	-985.81834	617.19825	2463.91927	1

O	69.04971	71.78055	75.47066	293.29899	-391.65602	-477.80926	1
H	69.98178	71.67285	75.19279	2528.72714	-681.76262	-751.27122	1
C	67.36998	69.88863	75.24791	252.47363	-605.14473	440.05134	1
H	67.35694	69.96126	74.13615	-2052.65063	1456.49051	-1043.51830	1
H	67.19818	68.81731	75.50834	-2152.98800	-1661.97574	-161.70652	1
H	66.49823	70.46963	75.62915	-473.12726	-1584.27339	2165.61725	1
C	68.23416	68.61292	77.72142	360.36307	488.46713	505.10056	1
O	67.07415	68.23100	77.71293	-200.08817	-528.03552	-385.82850	1
N	69.34988	67.92163	77.86377	-597.54095	-226.44775	355.20479	1
H	70.22390	68.45419	77.73901	2179.23655	-1300.70981	-991.33835	1
C	69.41663	66.61308	78.39222	-687.68921	297.39807	-249.45179	1
H	70.46940	66.51275	78.73594	-349.16256	-2467.37713	-1101.72831	1
C	68.33322	66.39225	79.54894	672.47345	361.54328	-201.62880	1
H	67.30982	66.49764	79.11452	1541.41502	582.18425	2169.97026	1
H	68.41807	65.34969	79.93357	-2534.67717	60.25153	997.72295	1
C	68.51895	67.39940	80.69225	574.26774	-389.43119	-377.02542	1
C	69.49726	67.18359	81.70887	688.82679	-287.33580	-257.96133	1
H	70.16427	66.30317	81.67559	331.14182	1470.38163	2269.80013	1
C	67.66989	68.54643	80.73779	-383.52382	-675.04808	144.24917	1
H	66.92216	68.72040	79.93985	4.09847	1517.59662	2262.86397	1
C	69.57985	68.10179	82.75558	-134.76730	428.45285	-649.50303	1
H	70.32515	67.92872	83.55109	-903.23433	1600.49020	2011.53387	1
C	67.79387	69.43933	81.80385	-335.53281	-615.00040	-364.39058	1
H	67.12666	70.31886	81.82974	1596.45398	-1369.33862	1732.02659	1
C	68.74197	69.25074	82.85162	585.48547	-340.24194	-406.23938	1
O	68.86757	70.14613	83.94543	-419.15649	-173.04408	-511.94141	1
H	68.15808	70.79157	83.71349	-1071.84350	1559.71507	-1960.13012	1
C	69.02806	65.49696	77.26302	291.41878	-183.13091	710.72246	1
O	69.47405	64.37670	77.32426	379.87695	523.47396	-222.23622	1
N	68.08533	65.96816	76.44573	564.30591	373.09380	277.24371	1
H	67.77965	66.95000	76.55700	-120.59931	-2436.07824	-1214.35098	1
C	67.91125	65.28132	75.20349	404.07514	-299.37480	608.84009	1
H	68.06180	64.20147	75.43537	-1071.07882	-841.69025	2359.66467	1
C	66.46375	65.68504	74.70563	-683.12807	68.71770	-390.13096	1
H	65.73150	65.57130	75.53661	2068.88918	1596.58590	770.89603	1
H	66.42780	66.76130	74.42253	1379.86429	2342.49779	-179.85638	1
C	66.07416	64.77886	73.49745	-176.39967	537.49466	550.97268	1
H	66.70708	64.99665	72.60178	-396.28051	1942.22168	1869.33314	1
H	66.23856	63.70474	73.73737	-94.44700	586.00678	-2659.20016	1
C	64.53120	65.07761	73.20769	-291.69381	-718.18079	-150.72929	1
H	63.85612	65.02627	74.10006	-2303.38776	1089.76242	964.62032	1
H	64.31612	66.03788	72.67359	884.16729	2568.80533	-207.74283	1
N	64.17349	64.01547	72.36504	-594.49522	-252.81478	342.29276	1
H	64.89814	63.35164	72.14009	-1735.17426	2084.05705	-263.71605	1
C	63.01209	63.83627	71.88104	595.23177	-271.52487	442.22352	1
N	62.02113	64.77036	72.20189	585.53813	-424.79750	-105.81303	1
H	62.08587	65.61294	72.77189	959.88797	1327.56823	-2177.11905	1
H	61.05781	64.73791	71.86648	1578.48647	16.27378	-2220.76233	1
N	62.96969	62.67738	71.09349	-452.17763	-390.33541	-421.51853	1
H	63.69368	61.99842	70.86277	470.26107	1974.25629	-1817.92087	1
H	62.12569	62.35720	70.61695	1856.67307	-1573.97321	-1224.35416	1
C	69.00416	65.86829	74.13636	-531.47271	-581.45780	-55.06206	1
O	69.40745	67.00397	74.14027	-243.00837	192.73507	609.52485	1
N	69.28434	64.88417	73.27072	343.49288	-644.25607	38.09164	1
H	68.74763	64.02896	73.41044	-1691.16043	-1535.97525	-1484.73099	1
C	69.98245	65.17569	72.01646	542.66492	350.14337	454.42563	1
H	70.90312	65.72310	72.32654	1209.57979	-2396.95920	463.86744	1
C	70.22847	63.88081	71.26977	-737.14309	145.93010	242.71942	1
H	69.23568	63.43460	71.03419	-1050.18303	159.36419	-2509.06125	1
C	70.95390	64.13874	69.96034	-706.77696	-252.88343	-245.16230	1
H	71.05496	63.20219	69.34647	-1624.64039	-1665.49085	1417.86935	1
H	70.41461	64.89810	69.32855	2566.10521	598.51109	693.22305	1

```
H    71.99103  64.53781  70.13269    -1972.69758      107.10018    -1876.34312          1
C    71.00046  62.86804  72.09602      412.85148      672.58832      -27.69222          1
H    71.13392  61.89623  71.54587     1447.02277     1762.76533    -1490.78944          1
H    72.02610  63.24662  72.35852     1320.77379     -145.20455     2378.68491          1
H    70.47718  62.63717  73.06413    -1512.10848     1470.52031     1724.75147          1
C    68.95127  66.11702  71.10908     -607.11113      499.56689       73.73769          1
O    67.82492  65.77390  70.84617      357.97126      460.81534      356.68785          1
N    69.60133  67.21442  70.73932      536.55407     -368.62270      332.76306          1
H    70.56737  67.31432  71.05120      524.38866     2297.69985     1367.20941          1
C    69.18749  67.86581  69.54511       46.50277     -774.29190     -147.97904          1
H    68.33672  67.24993  69.17227     1676.51875     1883.29066     1032.55549          1
C    68.75939  69.38058  69.91914     -559.41456      -34.19198     -556.30433          1
H    69.57704  69.86551  70.50377      -64.66664      395.18769     2695.05373          1
H    68.62153  70.00198  69.00286    -2703.44278      -60.40056      333.79418          1
C    67.49082  69.31869  70.72422     -519.76435      377.31045      459.42366          1
H    67.59500  68.50041  71.47378    -1746.74323     1667.50644    -1261.73691          1
C    67.26080  70.62984  71.46556      570.95554       16.46261     -545.27700          1
H    66.32315  70.60011  72.07820     2391.58273       28.05277    -1305.07271          1
H    68.10642  70.86626  72.16217      175.75588      917.63458     2559.43867          1
H    67.16524  71.49223  70.75674    -1063.97851    -2155.34650     1283.00462          1
C    66.29272  69.01955  69.83226      193.76014        2.62946     -765.53141          1
H    65.33612  69.00497  70.41507      507.39394     -846.55087     2539.60089          1
H    66.17742  69.78825  69.02503     1337.57841     1818.19054     1526.01994          1
H    66.38762  68.02232  69.33042     1100.88635    -1172.91350    -2199.08909          1
C    70.47675  67.90316  68.58413     -549.21115      475.64486     -309.38218          1
O    71.51716  67.36543  68.86271      556.06106     -390.37755      -78.24250          1
O    70.33161  68.59552  67.35710     -572.27940     -310.19032     -209.75862          1
H    69.37586  68.86151  67.44273    -1380.08193     2212.14486      790.85944          1
```

Listing 47: C00047.xmolout

```
      276
  150.0000  150.0000  150.0000     0.0000     0.0000     0.0000***********    2.7750    11100
      0.2500    300.00    0.00    0.00    0.00   -1293.7078       0.0000    300.0000
H    76.71315  73.25631  82.38942    -2043.14101    -1757.64893      399.89339          1
N    77.61161  72.89208  82.07683      426.75047      490.45733     -334.42467          1
H    77.32416  72.32704  81.27854      442.25559    -1110.30854    -2448.52907          1
C    78.54684  73.90031  81.72965      415.32972     -666.39213       83.73172          1
H    77.89164  74.72760  81.38330      879.32219     2491.00447      667.35213          1
C    79.37578  74.31727  83.04416       31.35357      477.41777      628.23362          1
H    79.89793  73.41561  83.44579     1168.38489     2240.25337    -1019.71136          1
H    80.17602  75.04920  82.77590     -360.43770     2534.90498      931.67112          1
C    78.48071  74.90339  84.10654      463.18208      512.70242     -382.34388          1
H    77.65750  74.18264  84.30967     1725.29117     1863.52247     -987.07854          1
C    79.26264  75.11867  85.39692      710.42801     -144.27427     -313.15367          1
H    78.61438  75.53700  86.20985      364.20195    -1033.13725     2494.72524          1
H    79.70179  74.16238  85.78186     2567.61904      335.80679      847.48802          1
H    80.11051  75.83663  85.25025    -2587.99029      680.92867     -512.16411          1
C    77.87494  76.22915  83.66693      268.17616      741.64379      -40.42634          1
H    77.25296  76.68773  84.47894     1283.54314    -1945.37420    -1411.27812          1
H    78.66755  76.97458  83.39809    -1258.57796     1317.15249    -2026.02085          1
H    77.21269  76.10974  82.77138    -2502.84526      909.80597      575.92391          1
C    79.41965  73.27712  80.50520      619.36578     -458.64843     -172.09273          1
O    78.88612  72.90865  79.47654      -81.40433      433.87611      522.34594          1
N    80.71533  73.27954  80.79156      175.23996     -622.20190      341.55523          1
H    81.03178  73.59340  81.70639     -876.57256     -303.22879    -2561.90213          1
C    81.63591  72.72131  79.86072     -571.88214     -534.10783     -106.15033          1
H    81.03800  72.54026  78.93911    -2713.20841      -16.25800     -248.80809          1
C    82.83023  73.78150  79.70289      166.60354      288.16399     -716.09568          1
H    83.33753  73.88913  80.69980    -2097.85574     1636.78449     -586.18044          1
O    82.42125  75.10943  79.31616     -317.80580     -363.38227      484.43082          1
H    82.71196  75.62416  80.09493     1863.74748    -1451.84923     1357.29467          1
```

```
C    83.80025  73.04361  78.71306     503.80731     517.99032     318.51606    1
H    84.71925  73.64487  78.52023     640.61020   -2157.10410   -1536.29104    1
H    84.12982  72.05430  79.11460    1687.88455     465.60692    2087.56452    1
H    83.31766  72.84806  77.72214   -1293.07895   -1899.88581   -1463.57452    1
C    82.25374  71.37402  80.53557    -153.07550    -733.68902     248.70981    1
O    83.29937  71.46316  81.15942     186.15459    -480.78787    -449.34387    1
N    81.49819  70.29989  80.31469    -487.53139     543.18517      42.03850    1
C    81.86865  69.00916  80.82400    -699.35871      69.34393     360.10701    1
H    82.27816  69.19515  81.84322       2.81126    -221.99807   -2715.58072    1
C    80.50560  68.20572  80.75292    -507.46439    -592.59194     122.07761    1
H    80.65624  67.10250  80.72826   -1975.08116     525.87560   -1801.71556    1
H    79.85385  68.42300  81.62885    1588.27743   -1913.22100    1113.83566    1
C    79.89449  68.77871  79.43545    -613.62442    -457.55993    -194.14490    1
H    80.41103  68.35676  78.54301    1707.54637    -721.27992    1996.92523    1
H    78.80955  68.56583  79.31115     758.23357    2100.81767    1560.55011    1
C    80.21565  70.32367  79.62224    -352.41524     704.96076      49.21465    1
H    80.33169  70.90160  78.67992    1714.76886   -2070.42778     443.35792    1
H    79.51400  70.87043  80.29849    1224.15040    1801.50881    1636.97633    1
C    82.99703  68.39564  79.80081     566.85117     537.05831     117.62814    1
O    82.75467  67.39945  79.16674    -550.26779    -406.08867       4.21666    1
N    84.07891  69.15282  79.85725     566.34832    -425.77725     180.18749    1
H    84.07302  69.97783  80.49669     644.44502    2646.80550     -52.73430    1
C    85.22429  68.92116  79.04723     612.34801    -281.48662     411.56221    1
H    86.04670  69.37111  79.64686    1667.23498   -1490.75142    1556.16753    1
C    85.42883  67.35130  78.83070    -714.45746    -175.43074     286.98927    1
H    84.56276  66.96615  78.21424    -787.09484    1763.03619   -1922.46060    1
O    85.44985  66.58405  80.05665    -429.46514     516.51932    -128.40526    1
H    84.51932  66.27272  80.07078    1981.82785     436.16847   -1818.18182    1
C    86.73533  67.23270  77.96632     630.79189    -468.94448      76.03230    1
H    86.97610  66.16672  77.74436   -1775.71861    2062.30353     131.89849    1
H    86.64156  67.76071  76.98585   -1855.14608      88.94118    1993.53760    1
H    87.61536  67.66632  78.49589   -1504.82350    -780.94249   -2132.90992    1
C    85.05396  69.73969  77.64866     288.57638     618.62814    -397.00270    1
O    86.02481  69.86589  76.93168     321.70495    -185.26452     574.37218    1
N    83.83055  70.23311  77.44563    -447.05792    -446.53431     367.76425    1
H    83.10539  69.98526  78.11809     350.31316    1975.63644   -1843.31522    1
C    83.57187  70.69161  76.12216    -511.23061     374.03917    -471.51480    1
H    84.56882  71.04316  75.76738    2234.79579    -898.70890   -1273.45190    1
C    83.07037  69.40963  75.31028     529.53781    -159.34528     563.72604    1
H    83.15047  69.66926  74.22212     654.59388    2347.94251    1217.51488    1
H    83.80927  68.58480  75.50740   -1526.55661    2103.42026     817.87394    1
O    81.75761  68.89116  75.63951    -554.84278      14.12789     399.58769    1
H    81.19376  69.53460  75.15777    1297.06236    2061.84854    1220.68812    1
C    82.41301  71.85433  76.10008    -662.15484    -412.13544     123.62687    1
O    82.67285  72.89530  75.53071    -540.01537    -317.05804     274.91456    1
N    81.32388  71.51768  76.77824    -202.76299    -690.00100    -131.49145    1
H    81.36535  70.56128  77.13511    -702.62132   -2617.37191     281.70413    1
C    80.06501  72.17530  76.62628    -491.73914     576.34187     222.73509    1
H    79.56360  72.11576  77.62652    1977.17978   -1823.58850    -434.68928    1
C    80.23032  73.66573  76.12418    -649.50797    -328.66843     306.11224    1
H    80.75716  73.70364  75.13897    2075.07803    1452.16490   -1004.45904    1
C    78.78430  74.26538  76.01936    -615.18735    -489.99689     -70.96352    1
H    78.80331  75.33485  75.70422   -1226.32661   -1724.38906   -1716.47170    1
H    78.16408  73.71195  75.27541    2381.64273    -969.84735     900.46860    1
H    78.25069  74.21530  76.99778    2122.74991    1702.23946    -141.36228    1
C    80.97889  74.54805  77.18725    -501.86074    -479.61276    -376.42482    1
H    80.93531  75.63247  76.93039     993.79261   -2246.95401   -1177.81305    1
H    80.54229  74.43108  78.20975      42.71279    -626.15391   -2651.37258    1
H    82.05980  74.27182  77.24668   -2026.39012   -1590.33709    -887.82922    1
C    79.23581  71.32798  75.49387    -672.89153     382.57394    -156.34072    1
O    79.72850  70.89961  74.47059     410.04627     221.86223     500.35950    1
N    77.98805  71.22965  75.92685    -378.11782    -443.79414    -441.10984    1
```

H	77.75742	71.73028	76.78370	2404.15973	-568.14894	1149.30085	1
C	76.94262	70.80104	75.03133	-544.54484	466.16019	331.29119	1
H	76.92818	71.58505	74.24234	-1409.80988	-170.17955	2325.32679	1
C	77.33026	69.38989	74.42882	-184.48284	116.78282	-758.89137	1
H	78.33283	69.43221	73.92926	1893.78641	1861.14652	611.04528	1
H	77.42293	68.62857	75.23796	-2170.79665	-974.19311	1327.50117	1
C	76.20294	68.99933	73.42143	389.51668	672.81878	-138.49152	1
H	75.20649	69.07251	73.91750	510.64577	1933.03327	1851.02484	1
H	76.14470	69.74716	72.59992	-2302.30869	-1105.72939	-948.89774	1
C	76.39001	67.55896	72.81223	-559.93427	-126.34590	542.30857	1
O	75.55613	67.11558	72.06509	-670.36003	-111.83166	76.36448	1
N	77.52563	66.93901	73.20042	-216.06729	-537.07037	446.51379	1
H	78.28613	67.27578	73.80234	-1251.96487	1074.12894	-2168.52500	1
H	77.81336	66.01225	72.86811	-2451.83023	-720.73851	944.84612	1
C	75.53623	70.68105	75.82621	546.24052	-563.88337	-85.11809	1
O	74.54519	70.87032	75.16445	71.70702	-307.40463	606.69673	1
N	75.60587	70.23347	77.09054	479.90863	-504.50129	222.86849	1
C	74.39933	70.17850	77.85817	75.51268	-600.91904	506.73700	1
H	73.57495	70.15265	77.10620	-2033.61562	1795.30103	254.89480	1
C	74.62426	68.86474	78.71928	523.79067	517.99274	284.45581	1
H	73.99567	68.83589	79.63719	1599.61665	-1127.00911	1895.98173	1
H	74.38527	67.94727	78.13642	-1881.83093	1275.82488	-1501.55016	1
C	76.15175	68.96307	79.02249	-600.05639	-404.05026	316.64485	1
H	76.34875	69.73448	79.80372	-2597.05260	496.36221	657.73249	1
H	76.60119	68.01081	79.38408	-1666.24518	1294.38985	-1723.90591	1
C	76.70295	69.42267	77.60627	493.57570	-129.95376	-602.56394	1
H	77.62654	70.04407	77.64720	-1469.08415	-637.84797	2204.22581	1
H	76.84916	68.60138	76.86453	-1038.97840	1068.88701	-2280.71789	1
C	74.33572	71.49433	78.84233	188.88100	241.05089	-727.87821	1
O	74.93199	71.52620	79.89641	531.88613	-49.19943	427.07960	1
N	73.57581	72.43686	78.29968	708.14833	26.93417	-179.73509	1
H	73.19652	72.27646	77.36783	-2232.69501	-1179.09831	1023.94816	1
C	73.39041	73.61542	79.08058	-738.32412	259.92548	104.42456	1
H	73.41659	73.22658	80.12452	2028.89123	1260.02983	1311.33324	1
C	74.61955	74.59144	78.82207	-258.65678	-345.52513	661.28459	1
H	74.66897	74.88622	77.75155	-1826.06090	-1801.46097	-918.64554	1
C	74.40731	75.83623	79.74883	-541.23400	531.92970	-218.41428	1
H	75.19588	76.60935	79.59413	1311.73218	-2219.18200	882.19023	1
H	73.41677	76.31882	79.55734	-1048.71298	1223.58118	-2196.97971	1
H	74.42739	75.55014	80.82694	419.06414	1903.92898	1903.44670	1
C	75.94811	73.88902	79.26929	-423.86431	152.66566	-648.55262	1
H	76.82903	74.56911	79.18964	105.68386	-2342.50080	1387.51238	1
H	75.88459	73.54817	80.33056	-1695.63603	-1484.73621	-1531.02793	1
H	76.16918	72.98660	78.64789	-1853.57121	-487.39031	1936.59343	1
C	72.02076	74.43007	78.67750	539.71428	-510.58232	267.58633	1
O	71.49497	75.08089	79.55508	-15.93758	-278.34085	-624.49402	1
N	71.76353	74.42277	77.37777	-676.81886	12.05292	276.17960	1
H	72.38883	73.81370	76.85404	-1694.44250	-373.84189	-2100.66098	1
C	70.47221	74.75365	76.83841	412.33078	-664.70360	108.35451	1
H	70.09245	73.80393	76.40032	1765.77476	1294.44366	1621.76594	1
C	69.52541	75.36737	77.93882	-179.66635	-721.95667	-264.73915	1
H	69.38646	74.65275	78.78388	-607.34922	2587.87032	-598.10018	1
H	70.00589	76.26698	78.39280	-359.69332	-1479.13954	-2259.74260	1
C	68.15142	75.75585	77.29264	-240.01076	616.02425	431.85322	1
H	68.30814	76.43024	76.41991	-1359.17131	782.54425	2227.99181	1
H	67.62120	74.86119	76.89724	-820.36667	85.81767	-2596.78715	1
C	67.30542	76.47119	78.42100	-513.07015	236.18570	-551.87280	1
H	67.09274	75.76787	79.25879	1816.11944	-1183.11722	-1650.94321	1
H	67.88349	77.30938	78.87380	-1800.04429	-1361.53473	1526.34643	1
C	65.99022	76.99902	77.80294	726.08972	-253.19732	-179.64713	1
H	66.12360	77.79873	77.04735	1224.27271	-456.51798	-2390.90304	1
H	65.31559	76.21346	77.40838	1206.40339	-1150.93514	-2154.90344	1

N	65.17424	77.67875	78.95540	329.70796	-633.68968	-155.67358	1
H	64.24413	78.10358	78.67323	1348.31305	1938.76845	1359.00643	1
H	64.88290	77.06161	79.76829	2096.92035	1446.54551	-966.48890	1
H	65.63323	78.49521	79.45473	-1197.07217	-2303.53719	827.28733	1
C	70.69986	75.87776	75.68339	-378.01186	-556.17301	-413.96481	1
O	69.96744	75.91487	74.69462	-409.44433	-307.55053	-453.30833	1
N	71.66117	76.70805	76.02893	-429.02477	524.70884	274.08502	1
H	72.15487	76.46964	76.88728	1706.34263	-1425.96036	1574.38926	1
C	71.91952	77.87766	75.25338	-45.25864	-626.22952	478.93267	1
H	70.90313	78.15346	74.89017	1988.63164	570.90773	1772.87287	1
C	72.61661	78.94511	76.18505	551.77128	-20.49526	-564.54996	1
H	71.96903	79.18796	77.05756	1434.61535	-1753.10253	1513.99471	1
H	73.56320	78.54443	76.61167	-714.67738	1819.09770	1898.36494	1
C	72.88802	80.21693	75.31286	446.99263	-555.77305	338.97221	1
H	73.61762	79.98329	74.50191	1953.68466	1892.25957	-161.67766	1
H	71.95049	80.56037	74.79930	-403.03785	2688.07032	-188.43591	1
C	73.43428	81.36837	76.24730	567.86262	335.74681	434.04421	1
H	72.70466	81.60271	77.05657	-1080.02275	1568.26081	-1948.78900	1
H	74.37474	81.06079	76.75951	-919.54108	2111.34692	1456.13463	1
C	73.66234	82.60290	75.34252	195.81897	173.33862	-745.11536	1
H	74.45001	82.47182	74.57344	1405.67949	-2301.21089	-390.08113	1
H	72.73876	83.00802	74.87666	-940.37328	-1118.32446	2299.72133	1
N	74.17741	83.76471	76.25194	319.26422	656.36007	-42.02424	1
H	74.41189	84.67188	75.75443	1961.03633	619.91968	1787.09422	1
H	73.50115	84.10665	76.99406	854.14343	-2156.80752	1429.08711	1
H	75.06139	83.59570	76.81546	1237.02905	-1378.50857	-1998.28500	1
C	72.88600	77.44030	73.99970	532.53319	-268.57236	517.55729	1
O	74.01102	77.84135	73.84668	266.16714	-290.73804	-558.88002	1
N	72.12655	76.69216	73.19923	-226.25352	640.49678	-270.32839	1
H	71.16541	76.42435	73.53281	2007.69063	-1035.27229	1523.50231	1
C	72.82075	75.83775	72.29123	-540.76542	-573.33303	49.50000	1
H	73.86390	76.23066	72.25345	-2374.20950	-1036.76023	843.75774	1
C	72.73255	74.39805	72.98444	-77.09082	666.87422	-415.84136	1
H	71.64355	74.13580	73.08669	-985.81834	617.19825	2463.91927	1
O	73.26076	74.33480	74.32810	293.29899	-391.65602	-477.80926	1
H	72.45557	74.51274	74.85436	2528.72714	-681.76262	-751.27122	1
C	73.41918	73.43592	71.95216	252.47363	-605.14473	440.05134	1
H	73.43451	72.38450	72.32454	-2052.65063	1456.49051	-1043.51830	1
H	72.89260	73.42986	70.96641	-2152.98800	-1661.97574	-161.70652	1
H	74.47712	73.73291	71.76037	-473.12726	-1584.27339	2165.61725	1
C	72.06586	75.85856	70.84601	360.36307	488.46713	505.10056	1
O	72.59997	75.35156	69.88499	-200.08817	-528.03552	-385.82850	1
N	70.83394	76.37561	70.90094	-597.54095	-226.44775	355.20479	1
H	70.57126	76.82551	71.77644	2179.23655	-1300.70981	-991.33835	1
C	70.25490	76.78044	69.66735	-687.68921	297.39807	-249.45179	1
H	70.66257	76.07877	68.90229	-349.16256	-2467.37713	-1101.72831	1
C	68.66466	76.78387	69.83987	672.47345	361.54328	-201.62880	1
H	68.39864	77.55627	70.59277	1541.41502	582.18425	2169.97026	1
H	68.18785	77.08731	68.87983	-2534.67717	60.25153	997.72295	1
C	68.12895	75.42109	70.29703	574.26774	-389.43119	-377.02542	1
C	67.82525	74.40615	69.34008	688.82679	-287.33580	-257.96133	1
H	68.01298	74.56975	68.26347	331.14182	1470.38163	2269.80013	1
C	67.90647	75.19296	71.68959	-383.52382	-675.04808	144.24917	1
H	68.16564	75.96596	72.43599	4.09847	1517.59662	2262.86397	1
C	67.27268	73.20841	69.79331	-134.76730	428.45285	-649.50303	1
H	67.02777	72.42618	69.05372	-903.23433	1600.49020	2011.53387	1
C	67.34668	73.97849	72.09201	-335.53281	-615.00040	-364.39058	1
H	67.16635	73.81967	73.16966	1596.45398	-1369.33862	1732.02659	1
C	67.00232	72.94814	71.16835	585.48547	-340.24194	-406.23938	1
O	66.42664	71.71389	71.56741	-419.15649	-173.04408	-511.94141	1
H	66.35334	71.87050	72.53851	-1071.84350	1559.71507	-1960.13012	1
C	70.63209	78.34893	69.40818	291.41878	-183.13091	710.72246	1

217	O	70.99169	79.11093	70.27262	379.87695	523.47396	-222.23622	1
218	N	70.25160	78.62206	68.14479	564.30591	373.09380	277.24371	1
219	H	70.04818	77.77239	67.61699	-120.59931	-2436.07824	-1214.35098	1
220	C	70.92042	79.71924	67.47427	404.07514	-299.37480	608.84009	1
221	H	72.00767	79.48868	67.50427	-1071.07882	-841.69025	2359.66467	1
222	C	70.30548	79.74028	66.02721	-683.12807	68.71770	-390.13096	1
223	H	70.47487	78.77305	65.50177	2068.88918	1596.58590	770.89603	1
224	H	69.19973	79.85494	66.09677	1379.86429	2342.49779	-179.85638	1
225	C	70.92846	80.92792	65.22347	-176.39967	537.49466	550.97268	1
226	H	70.91510	81.86761	65.82016	-396.28051	1942.22168	1869.33314	1
227	H	71.99722	80.73845	64.97996	-94.44700	586.00678	-2659.20016	1
228	C	70.03687	81.08139	63.90782	-291.69381	-718.18079	-150.72929	1
229	H	70.02242	80.18664	63.23441	-2303.38776	1089.76242	964.62032	1
230	H	68.97534	81.39528	64.07810	884.16729	2568.80533	-207.74283	1
231	N	70.67815	82.11852	63.21633	-594.49522	-252.81478	342.29276	1
232	H	71.53981	82.46265	63.61026	-1735.17426	2084.05705	-263.71605	1
233	C	70.25033	82.63828	62.13793	595.23177	-271.52487	442.22352	1
234	N	69.05357	82.14488	61.60559	585.53813	-424.79750	-105.81303	1
235	H	68.44429	81.40794	61.95786	959.88797	1327.56823	-2177.11905	1
236	H	68.61250	82.48149	60.74936	1578.48647	16.27378	-2220.76233	1
237	N	71.11388	83.64600	61.68473	-452.17763	-390.33541	-421.51853	1
238	H	71.98549	84.00426	62.07166	470.26107	1974.25629	-1817.92087	1
239	H	70.94644	84.18090	60.83179	1856.67307	-1573.97321	-1224.35416	1
240	C	70.51872	81.10245	68.22465	-531.47271	-581.45780	-55.06206	1
241	O	69.38191	81.38869	68.51637	-243.00837	192.73507	609.52485	1
242	N	71.62100	81.93788	68.31484	343.49288	-644.25607	38.09164	1
243	H	72.31069	81.36954	67.79575	-1691.16043	-1535.97525	-1484.73099	1
244	C	72.05779	82.32593	69.68171	542.66492	350.14337	454.42563	1
245	H	72.43032	81.36788	70.11113	1209.57979	-2396.95920	463.86744	1
246	C	73.12976	83.36780	69.49363	-737.14309	145.93010	242.71942	1
247	H	72.76727	84.07755	68.71494	-1050.18303	159.36419	-2509.06125	1
248	C	73.37968	84.15797	70.76665	-706.77696	-252.88343	-245.16230	1
249	H	74.15758	84.95613	70.61606	-1624.64039	-1665.49085	1417.86935	1
250	H	72.43807	84.66872	71.11943	2566.10521	598.51109	693.22305	1
251	H	73.73447	83.49700	71.60454	-1972.69758	107.10018	-1876.34312	1
252	C	74.43772	82.77045	69.00360	412.85148	672.58832	-27.69222	1
253	H	75.21449	83.56375	68.82440	1447.02277	1762.76533	-1490.78944	1
254	H	74.86731	82.04767	69.75050	1320.77379	-145.20455	2378.68491	1
255	H	74.30409	82.21076	68.03781	-1512.10848	1470.52031	1724.75147	1
256	C	70.79755	82.94925	70.54416	-607.11113	499.56689	73.73769	1
257	O	70.38118	84.06145	70.32662	357.97126	460.81534	356.68785	1
258	N	70.44326	82.10072	71.50085	536.55407	-368.62270	332.76306	1
259	H	70.90251	81.19106	71.55897	524.38866	2297.69985	1367.20941	1
260	C	69.34327	82.45504	72.31756	46.50277	-774.29190	-147.97904	1
261	H	68.86402	83.31901	71.80226	1676.51875	1883.29066	1032.55549	1
262	C	68.40461	81.15680	72.52845	-559.41456	-34.19198	-556.30433	1
263	H	68.93551	80.42293	73.18183	-64.66664	395.18769	2695.05373	1
264	H	67.48039	81.44470	73.08442	-2703.44278	-60.40056	333.79418	1
265	C	68.06472	80.51927	71.20834	-519.76435	377.31045	459.42366	1
266	H	69.00831	80.14581	70.73989	-1746.74323	1667.50644	-1261.73691	1
267	C	67.11563	79.34868	71.42967	570.95554	16.46261	-545.27700	1
268	H	66.84580	78.84523	70.46524	2391.58273	28.05277	-1305.07271	1
269	H	67.56391	78.57332	72.10428	175.75588	917.63458	2559.43867	1
270	H	66.15725	79.68147	71.90566	-1063.97851	-2155.34650	1283.00462	1
271	C	67.43441	81.51972	70.24679	193.76014	2.62946	-765.53141	1
272	H	67.23953	81.05505	69.24283	507.39394	-846.55087	2539.60089	1
273	H	66.45930	81.91692	70.63425	1337.57841	1818.19054	1526.01994	1
274	H	68.11208	82.39939	70.06732	1100.88635	-1172.91350	-2199.08909	1
275	C	69.92105	82.81853	73.78446	-549.21115	475.64486	-309.38218	1
276	O	70.69985	82.10639	74.37166	556.06106	-390.37755	-78.24250	1
277	O	69.35515	83.94172	74.41819	-572.27940	-310.19032	-209.75862	1
278	H	68.77531	84.26934	73.67158	-1380.08193	2212.14486	790.85944	1

Listing 48: C00048.xmolout

```
      276
  150.0000  150.0000  150.0000    0.0000    0.0000    0.0000**********    2.7750   11100
      0.2500    300.00   0.00   0.00   0.00  -1293.7071       0.0000    300.0000
H    60.08894  78.48963  69.93480    -2043.14101    -1757.64893     399.89339         1
N    59.97632  79.50315  69.93002      426.75047      490.45733    -334.42467         1
H    59.71229  79.72098  70.90112      442.25559    -1110.30854   -2448.52907         1
C    61.14284  80.20622  69.56222      415.32972     -666.39213      83.73172         1
H    61.78246  79.52905  68.95599      879.32219     2491.00447     667.35213         1
C    60.70163  81.54069  68.77378       31.35357      477.41777     628.23362         1
H    60.07683  82.17260  69.44967     1168.38489     2240.25337   -1019.71136         1
H    61.59548  82.15722  68.51309     -360.43770     2534.90498     931.67112         1
C    59.92105  81.16474  67.54034      463.18208      512.70242    -382.34388         1
H    59.13179  80.43833  67.83990     1725.29117     1863.52247    -987.07854         1
C    59.24685  82.39459  66.94540      710.42801     -144.27427    -313.15367         1
H    58.63795  82.13623  66.04085      364.20195    -1033.13725    2494.72524         1
H    58.55834  82.88595  67.68056     2567.61904      335.80679     847.48802         1
H    59.99835  83.16377  66.63023    -2587.99029      680.92867    -512.16411         1
C    60.81099  80.51254  66.49155      268.17616      741.64379     -40.42634         1
H    60.23535  80.25430  65.56522     1283.54314    -1945.37420   -1411.27812         1
H    61.64881  81.18971  66.18287    -1258.57796     1317.15249   -2026.02085         1
H    61.27162  79.56393  66.86990    -2502.84526      909.80597     575.92391         1
C    61.77046  80.63628  71.00728      619.36578     -458.64843    -172.09273         1
O    61.05916  80.66637  71.99599      -81.40433      433.87611     522.34594         1
N    63.03400  80.98329  70.83993      175.23996     -622.20190     341.55523         1
H    63.37978  80.92707  69.88157     -876.57256     -303.22879   -2561.90213         1
C    63.91854  81.38846  71.86915     -571.88214     -534.10783    -106.15033         1
H    64.75117  81.86083  71.30529    -2713.20841      -16.25800    -248.80809         1
C    63.16351  82.39827  72.85617      166.60354      288.16399    -716.09568         1
H    62.25203  81.86930  73.26565    -2097.85574     1636.78449    -586.18044         1
O    62.70850  83.62153  72.25236     -317.80580     -363.38227     484.43082         1
H    61.73792  83.49335  72.28842     1863.74748    -1451.84923    1357.29467         1
C    64.22822  82.55029  74.00344      503.80731      517.99032     318.51606         1
H    63.84474  83.18756  74.83397      640.61020    -2157.10410   -1536.29104         1
H    64.50265  81.55930  74.44364     1687.88455      465.60692    2087.56452         1
H    65.16751  83.02208  73.63131    -1293.07895    -1899.88581   -1463.57452         1
C    64.40027  80.06844  72.71390     -153.07550     -733.68902     248.70981         1
O    63.67946  79.61557  73.56451      186.15459     -480.78787    -449.34387         1
N    65.63438  79.67921  72.35887     -487.53139      543.18517      42.03850         1
C    66.24665  78.54103  72.96937     -699.35871       69.34393     360.10701         1
H    65.44806  77.78980  73.16418        2.81126     -221.99807   -2715.58072         1
C    67.33167  78.14999  71.87771     -507.46439     -592.59194     122.07761         1
H    68.14251  77.50503  72.28440    -1975.08116      525.87560   -1801.71556         1
H    66.87879  77.59838  71.02377     1588.27743    -1913.22100    1113.83566         1
C    67.82451  79.56553  71.44701     -613.62442     -457.55993    -194.14490         1
H    68.48247  80.00688  72.23263     1707.54637     -721.27992    1996.92523         1
H    68.40317  79.57242  70.49581      758.23357     2100.81767    1560.55011         1
C    66.44713  80.35198  71.35439     -352.41524      704.96076      49.21465         1
H    66.50627  81.43401  71.61847     1714.76886    -2070.42778     443.35792         1
H    65.90356  80.23422  70.38685     1224.15040     1801.50881    1636.97633         1
C    67.02221  79.02197  74.33761      566.85117      537.05831     117.62814         1
O    67.69588  80.02345  74.37934     -550.26779     -406.08867       4.21666         1
N    66.85032  78.09007  75.26519      566.34832     -425.77725     180.18749         1
H    66.28971  77.26725  75.04744      644.44502     2646.80550     -52.73430         1
C    67.50215  78.23880  76.51389      612.34801     -281.48662     411.56221         1
H    67.96305  79.25473  76.51679     1667.23498    -1490.75142    1556.16753         1
C    66.37290  78.00247  77.63373     -714.45746     -175.43074     286.98927         1
H    65.86166  77.02788  77.40366     -787.09484     1763.03619   -1922.46060         1
O    65.33682  78.99737  77.67580     -429.46514      516.51932    -128.40526         1
```

H	64.56691	78.48235	77.36078	1981.82785	436.16847	-1818.18182	1
C	67.21218	77.84227	78.95234	630.79189	-468.94448	76.03230	1
H	66.56080	77.59444	79.82222	-1775.71861	2062.30353	131.89849	1
H	67.97208	77.02951	78.86666	-1855.14608	88.94118	1993.53760	1
H	67.75838	78.78065	79.20420	-1504.82350	-780.94249	-2132.90992	1
C	68.56672	77.00241	76.62729	288.57638	618.62814	-397.00270	1
O	68.28845	75.87859	76.28642	321.70495	-185.26452	574.37218	1
N	69.64043	77.42048	77.31598	-447.05792	-446.53431	367.76425	1
H	69.72187	78.42858	77.45238	350.31316	1975.63644	-1843.31522	1
C	70.83906	76.63004	77.10265	-511.23061	374.03917	-471.51480	1
H	70.42966	75.59423	77.03653	2234.79579	-898.70890	-1273.45190	1
C	71.45262	77.10710	75.74924	529.53781	-159.34528	563.72604	1
H	72.15705	76.32721	75.36459	654.59388	2347.94251	1217.51488	1
H	70.65768	77.25538	74.98026	-1526.55661	2103.42026	817.87394	1
O	72.17057	78.53293	75.64668	-554.84278	14.12789	399.58769	1
H	73.01929	78.18800	75.38590	1297.06236	2061.84854	1220.68812	1
C	71.77275	76.87276	78.42081	-662.15484	-412.13544	123.62687	1
O	71.70547	76.08925	79.33034	-540.01537	-317.05804	274.91456	1
N	72.57021	77.92866	78.25618	-202.76299	-690.00100	-131.49145	1
H	72.46875	78.43151	77.33326	-702.62132	-2617.37191	281.70413	1
C	73.09255	78.72673	79.38394	-491.73914	576.34187	222.73509	1
H	72.44589	79.63085	79.30714	1977.17978	-1823.58850	-434.68928	1
C	72.96522	78.04521	80.72055	-649.50797	-328.66843	306.11224	1
H	73.18655	76.95820	80.58649	2075.07803	1452.16490	-1004.45904	1
C	73.92586	78.58933	81.76415	-615.18735	-489.99689	-70.96352	1
H	73.76878	78.10526	82.76707	-1226.32661	-1724.38906	-1716.47170	1
H	75.00588	78.41091	81.47192	2381.64273	-969.84735	900.46860	1
H	73.79802	79.69756	81.90553	2122.74991	1702.23946	-141.36228	1
C	71.55406	78.17366	81.27535	-501.86074	-479.61276	-376.42482	1
H	71.43023	77.59181	82.22907	993.79261	-2246.95401	-1177.81305	1
H	71.29342	79.24330	81.50192	42.71279	-626.15391	-2651.37258	1
H	70.78534	77.79022	80.54835	-2026.39012	-1590.33709	-887.82922	1
C	74.62907	79.09666	78.88903	-672.89153	382.57394	-156.34072	1
O	75.60032	78.75674	79.54705	410.04627	221.86223	500.35950	1
N	74.58610	79.79671	77.78020	-378.11782	-443.79414	-441.10984	1
H	73.64471	79.93254	77.41960	2404.15973	-568.14894	1149.30085	1
C	75.71445	80.36831	77.12430	-544.54484	466.16019	331.29119	1
H	75.21907	81.03616	76.38615	-1409.80988	-170.17955	2325.32679	1
C	76.52763	81.19621	78.20151	-184.48284	116.78282	-758.89137	1
H	75.86269	81.90754	78.74193	1893.78641	1861.14652	611.04528	1
H	76.93087	80.52999	78.99619	-2170.79665	-974.19311	1327.50117	1
C	77.66012	81.95754	77.44239	389.51668	672.81878	-138.49152	1
H	78.13147	81.29541	76.68281	510.64577	1933.03327	1851.02484	1
H	77.22733	82.78273	76.83549	-2302.30869	-1105.72939	-948.89774	1
C	78.77521	82.52600	78.39791	-559.93427	-126.34590	542.30857	1
O	79.74209	83.06584	77.92487	-670.36003	-111.83166	76.36448	1
N	78.52733	82.34318	79.71339	-216.06729	-537.07037	446.51379	1
H	77.70995	81.93071	80.17758	-1251.96487	1074.12894	-2168.52500	1
H	79.14020	82.68344	80.46252	-2451.83023	-720.73851	944.84612	1
C	76.47378	79.18120	76.28868	546.24052	-563.88337	-85.11809	1
O	75.98427	78.97151	75.20123	71.70702	-307.40463	606.69673	1
N	77.50584	78.54584	76.84747	479.90863	-504.50129	222.86849	1
C	78.06451	77.38833	76.18137	75.51268	-600.91904	506.73700	1
H	77.96251	77.63636	75.09910	-2033.61562	1795.30103	254.89480	1
C	79.56416	77.37070	76.75105	523.79067	517.99274	284.45581	1
H	80.05685	76.38044	76.63529	1599.61665	-1127.00911	1895.98173	1
H	80.20617	78.11559	76.23174	-1881.83093	1275.82488	-1501.55016	1
C	79.31250	77.76921	78.23374	-600.05639	-404.05026	316.64485	1
H	78.95576	76.89575	78.82429	-2597.05260	496.36221	657.73249	1
H	80.22288	78.14949	78.74922	-1666.24518	1294.38985	-1723.90591	1
C	78.17168	78.87163	78.09302	493.57570	-129.95376	-602.56394	1
H	77.36119	78.81980	78.88907	-1469.08415	-637.84797	2204.22581	1

H	78.55268	79.91483	78.00237	-1038.97840	1068.88701	-2280.71789	1
C	77.31553	76.04584	76.59106	188.88100	241.05089	-727.87821	1
O	77.80987	74.82113	76.32565	531.88613	-49.19943	427.07960	1
N	76.20006	76.23459	77.19436	708.14833	26.93417	-179.73509	1
H	76.07939	77.24385	77.33272	-2232.69501	-1179.09831	1023.94816	1
C	75.21887	75.21565	77.28875	-738.32412	259.92548	104.42456	1
H	75.78293	74.26035	77.20100	2028.89123	1260.02983	1311.33324	1
C	74.43446	75.39171	78.64219	-258.65678	-345.52513	661.28459	1
H	73.87041	76.35217	78.64448	-1826.06090	-1801.46097	-918.64554	1
C	73.44624	74.18259	78.81080	-541.23400	531.92970	-218.41428	1
H	72.92260	74.21294	79.79760	1311.73218	-2219.18200	882.19023	1
H	72.65571	74.19455	78.01791	-1048.71298	1223.58118	-2196.97971	1
H	73.97444	73.20322	78.74027	419.06414	1903.92898	1903.44670	1
C	75.45602	75.37201	79.83418	-423.86431	152.66566	-648.55262	1
H	74.94089	75.44232	80.82064	105.68386	-2342.50080	1387.51238	1
H	76.06233	74.43618	79.83796	-1695.63603	-1484.73621	-1531.02793	1
H	76.16590	76.23033	79.76581	-1853.57121	-487.39031	1936.59343	1
C	74.12484	75.42883	76.03403	539.71428	-510.58232	267.58633	1
O	73.18642	74.61972	75.90693	-15.93758	-278.34085	-624.49402	1
N	74.34638	76.44841	75.29407	-676.81886	12.05292	276.17960	1
H	75.14667	76.99915	75.54905	-1694.44250	-373.84189	-2100.66098	1
C	74.93767	74.86294	72.52554	412.33078	-664.70360	108.35451	1
H	74.60439	75.04681	71.47913	1765.77476	1294.44366	1621.76594	1
C	77.23500	74.48708	71.64100	-179.66635	-721.95667	-264.73915	1
H	78.28497	74.86230	71.64756	-607.34922	2587.87032	-598.10018	1
H	77.30226	73.39434	71.83948	-359.69332	-1479.13954	-2259.74260	1
C	76.58691	74.73363	70.22569	-240.01076	616.02425	431.85322	1
H	75.88509	73.90797	69.97141	-1359.17131	782.54425	2227.99181	1
H	75.98068	75.66691	70.22677	-820.36667	85.81767	-2596.78715	1
C	77.73376	74.85524	69.15005	-513.07015	236.18570	-551.87280	1
H	78.23617	75.84742	69.22682	1816.11944	-1183.11722	-1650.94321	1
H	78.53135	74.09644	69.32185	-1800.04429	-1361.53473	1526.34643	1
C	77.09194	74.66523	67.75368	726.08972	-253.19732	-179.64713	1
H	76.83475	73.61612	67.50564	1224.27271	-456.51798	-2390.90304	1
H	76.22640	75.32589	67.54577	1206.40339	-1150.93514	-2154.90344	1
N	78.15421	75.08355	66.68301	329.70796	-633.68968	-155.67358	1
H	77.87400	74.92175	65.67262	1348.31305	1938.76845	1359.00643	1
H	78.42855	76.10900	66.65833	2096.92035	1446.54551	-966.48890	1
H	79.10001	74.60309	66.71786	-1197.07217	-2303.53719	827.28733	1
C	74.87939	73.32295	72.95043	-378.01186	-556.17301	-413.96481	1
O	75.74859	72.97353	73.88301	-409.44433	-307.55053	-453.30833	1
N	74.08341	72.57149	72.29491	-429.02477	524.70884	274.08502	1
H	73.56871	73.15538	71.63116	1706.34263	-1425.96036	1574.38926	1
C	73.86170	71.18029	72.54649	-45.25864	-626.22952	478.93267	1
H	73.64325	71.12753	73.63632	1988.63164	570.90773	1772.87287	1
C	72.63481	70.80172	71.63111	551.77128	-20.49526	-564.54996	1
H	71.78513	71.49627	71.81907	1434.61535	-1753.10253	1513.99471	1
H	72.90503	70.94953	70.56128	-714.67738	1819.09770	1898.36494	1
C	72.18893	69.31985	71.87096	446.99263	-555.77305	338.97221	1
H	73.03704	68.61687	71.69735	1953.68466	1892.25957	-161.67766	1
H	71.86211	69.15775	72.92249	-403.03785	2688.07032	-188.43591	1
C	71.00940	69.05150	70.85165	567.86262	335.74681	434.04421	1
H	70.13603	69.70502	71.07943	-1080.02275	1568.26081	-1948.78900	1
H	71.32147	69.31731	69.81560	-919.54108	2111.34692	1456.13463	1
C	70.60908	67.56007	70.92015	195.81897	173.33862	-745.11536	1
H	71.40879	66.85350	70.62085	1405.67949	-2301.21089	-390.08113	1
H	70.16278	67.24236	71.88343	-940.37328	-1118.32446	2299.72133	1
N	69.47563	67.34156	69.86012	319.26422	656.36007	-42.02424	1
H	69.10610	66.35089	69.77728	1961.03633	619.91968	1787.09422	1
H	68.57621	67.88915	69.99352	854.14343	-2156.80752	1429.08711	1
H	69.71557	67.55882	68.84920	1237.02905	-1378.50857	-1998.28500	1
C	75.18340	70.28991	72.18898	532.53319	-268.57236	517.55729	1

O	75.14441	69.09364	72.37761	266.16714	-290.73804	-558.88002	1
N	76.16267	71.00294	71.63004	-226.25352	640.49678	-270.32839	1
H	75.99067	72.00642	71.59678	2007.69063	-1035.27229	1523.50231	1
C	77.47146	70.44314	71.59914	-540.76542	-573.33303	49.50000	1
H	77.28985	69.35518	71.75974	-2374.20950	-1036.76023	843.75774	1
C	78.08170	70.79734	70.17153	-77.09082	666.87422	-415.84136	1
H	78.09423	71.91573	70.03314	-985.81834	617.19825	2463.91927	1
O	77.33734	70.32010	69.02865	293.29899	-391.65602	-477.80926	1
H	77.05981	71.16223	68.61989	2528.72714	-681.76262	-751.27122	1
C	79.54825	70.24634	70.31111	252.47363	-605.14473	440.05134	1
H	80.15671	70.48632	69.40853	-2052.65063	1456.49051	-1043.51830	1
H	80.09092	70.67052	71.20048	-2152.98800	-1661.97574	-161.70652	1
H	79.55148	69.13698	70.42225	-473.12726	-1584.27339	2165.61725	1
C	78.32943	71.12483	72.83016	360.36307	488.46713	505.10056	1
O	78.31752	70.58066	73.90722	-200.08817	-528.03552	-385.82850	1
N	78.92748	72.24477	72.45363	-597.54095	-226.44775	355.20479	1
H	78.83957	72.54387	71.48390	2179.23655	-1300.70981	-991.33835	1
C	79.82564	72.93654	73.30635	-687.68921	297.39807	-249.45179	1
H	80.14648	73.80167	72.68431	-349.16256	-2467.37713	-1101.72831	1
C	79.03197	73.37938	74.61772	672.47345	361.54328	-201.62880	1
H	78.41053	72.52087	74.97224	1541.41502	582.18425	2169.97026	1
H	79.75708	73.60716	75.43510	-2534.67717	60.25153	997.72295	1
C	78.14684	74.63540	74.54769	574.26774	-389.43119	-377.02542	1
C	78.86209	75.79330	73.94009	688.82679	-287.33580	-257.96133	1
H	79.92556	75.95719	74.16618	331.14182	1470.38163	2269.80013	1
C	76.72214	74.49846	74.06792	-383.52382	-675.04808	144.24917	1
H	75.99717	74.77017	74.87436	4.09847	1517.59662	2262.86397	1
C	78.18452	76.73388	73.20064	-134.76730	428.45285	-649.50303	1
H	78.73109	77.65355	72.92583	-903.23433	1600.49020	2011.53387	1
C	76.43182	75.16646	72.77640	-335.53281	-615.00040	-364.39058	1
H	74.29220	75.50018	73.17722	1596.45398	-1369.33862	1732.02659	1
C	76.78857	76.59519	72.79314	585.48547	-340.24194	-406.23938	1
O	75.95107	77.51143	72.23025	-419.15649	-173.04408	-511.94141	1
H	75.84402	78.12459	73.06116	-1071.84350	1559.71507	-1960.13012	1
C	81.06141	71.91209	73.60171	291.41878	-183.13091	710.72246	1
O	81.48406	71.18609	72.73003	379.87695	523.47396	-222.23622	1
N	81.57155	72.11546	74.80996	564.30591	373.09380	277.24371	1
H	81.15546	72.77238	75.47029	-120.59931	-2436.07824	-1214.35098	1
C	82.54647	71.21719	75.31569	404.07514	-299.37480	608.84009	1
H	82.70658	70.42979	74.54246	-1071.07882	-841.69025	2359.66467	1
C	83.81083	72.13926	75.59911	-683.12807	68.71770	-390.13096	1
H	84.11241	72.67523	74.67166	2068.88918	1596.58590	770.89603	1
H	83.54005	72.93578	76.32791	1379.86429	2342.49779	-179.85638	1
C	84.99082	71.28318	76.14499	-176.39967	537.49466	550.97268	1
H	84.68145	70.71575	77.05425	-396.28051	1942.22168	1869.33314	1
H	85.31190	70.51506	75.40652	-94.44700	586.00678	-2659.20016	1
C	86.17978	72.30302	76.47619	-291.69381	-718.18079	-150.72929	1
H	86.60683	72.87190	75.61291	-2303.38776	1089.76242	964.62032	1
H	85.94465	73.02227	77.30007	884.16729	2568.80533	-207.74283	1
N	87.14857	71.43304	76.97609	-594.49522	-252.81478	342.29276	1
H	86.77006	70.53842	77.32364	-1735.17426	2084.05705	-263.71605	1
C	88.39093	71.70227	77.05434	595.23177	-271.52487	442.22352	1
N	88.84151	72.93605	76.59522	585.53813	-424.79750	-105.81303	1
H	88.30815	73.71763	76.21355	959.88797	1327.56823	-2177.11905	1
H	89.80947	73.26081	76.63316	1578.48647	16.27378	-2220.76233	1
N	89.03307	70.60217	77.63148	-452.17763	-390.33541	-421.51853	1
H	88.63374	69.74868	78.02336	470.26107	1974.25629	-1817.92087	1
H	90.02845	70.57716	77.85636	1856.67307	-1573.97321	-1224.35416	1
C	81.99786	70.70738	76.77746	-531.47271	-581.45780	-55.06206	1
O	81.40586	71.46513	77.51178	-243.00837	192.73507	609.52485	1
N	82.40544	69.46246	77.03032	343.49288	-644.25607	38.09164	1
H	82.90369	68.97942	76.28360	-1691.16043	-1535.97525	-1484.73099	1

```
C    81.72338   68.77175   78.07211       542.66492       350.14337       454.42563           1
H    81.06503   69.54157   78.54023      1209.57979     -2396.95920       463.86744           1
C    80.93446   67.56171   77.40094      -737.14309       145.93010       242.71942           1
H    81.62066   66.95970   76.76737     -1050.18303       159.36419     -2509.06125           1
C    80.31021   66.67924   78.53423      -706.77696      -252.88343      -245.16230           1
H    79.75369   65.80559   78.12086     -1624.64039     -1665.49085      1417.86935           1
H    81.09797   66.27950   79.21960      2566.10521       598.51109       693.22305           1
H    79.59781   67.26390   79.16244     -1972.69758       107.10018     -1876.34312           1
C    79.75489   68.13555   76.54496       412.85148       672.58832       -27.69222           1
H    79.17548   67.32395   76.04494      1447.02277      1762.76533     -1490.78944           1
H    79.03864   68.71620   77.17282      1320.77379      -145.20455      2378.68491           1
H    80.12246   68.82412   75.74702     -1512.10848      1470.52031      1724.75147           1
C    82.74483   68.07099   79.14219      -607.11113       499.56689        73.73769           1
O    82.35936   67.86950   80.27195       357.97126       460.81534       356.68785           1
N    83.86697   67.61160   78.57319       536.55407      -368.62270       332.76306           1
H    84.03663   67.94681   77.62494       524.38866      2297.69985      1367.20941           1
C    84.96158   67.39483   79.46740        46.50277      -774.29190      -147.97904           1
H    84.51679   67.36573   80.49015      1676.51875      1883.29066      1032.55549           1
C    85.74806   66.04538   79.05042      -559.41456       -34.19198      -556.30433           1
H    86.19801   66.18283   78.03861       -64.66664       395.18769      2695.05373           1
H    86.60482   65.86773   79.74352     -2703.44278       -60.40056       333.79418           1
C    84.80700   64.87161   79.04663      -519.76435       377.31045       459.42366           1
H    83.92285   65.13550   78.42302     -1746.74323      1667.50644     -1261.73691           1
C    85.49307   63.65563   78.43534       570.95554        16.46261      -545.27700           1
H    84.80884   62.76957   78.39471      2391.58273        28.05277     -1305.07271           1
H    85.83866   63.85767   77.38881       175.75588       917.63458      2559.43867           1
H    86.39431   63.35126   79.02742     -1063.97851     -2155.34650      1283.00462           1
C    84.32453   64.53706   80.45146       193.76014         2.62946      -765.53141           1
H    83.65858   63.63578   80.45566       507.39394      -846.55087      2539.60089           1
H    85.18047   64.31994   81.14147      1337.57841      1818.19054      1526.01994           1
H    83.74015   65.38253   80.89912      1100.88635     -1172.91350     -2199.08909           1
C    85.98697   68.59812   79.21864      -549.21115       475.64486      -309.38218           1
O    86.00333   69.22660   78.17403       556.06106      -390.37755       -78.24250           1
O    86.96129   68.83394   80.19431      -572.27940      -310.19032      -209.75862           1
H    86.63401   68.18706   80.89439     -1380.08193      2212.14486       790.85944           1
```

Listing 49: C00049.xmolout

```
      276
  150.0000  150.0000  150.0000     0.0000     0.0000     0.0000***********    2.7750    11100
      0.2500     300.00    0.00    0.00    0.00   -1293.7032       0.0000    300.0000
H    79.11725   85.79260   78.33612     -2043.14101     -1757.64893       399.89339           1
N    79.94656   85.78227   78.92696       426.75047       490.45733      -334.42467           1
H    79.62704   86.30149   79.74227       442.25559     -1110.30854     -2448.52907           1
C    81.10257   86.30588   78.31318       415.32972      -666.39213        83.73172           1
H    80.95392   86.13786   77.22600       879.32219      2491.00447       667.35213           1
C    81.23918   87.86571   78.68611        31.35357       477.41777       628.23362           1
H    81.33613   87.97056   79.79367      1168.38489      2240.25337     -1019.71136           1
H    82.18366   88.28165   78.25825      -360.43770      2534.90498       931.67112           1
C    80.04241   88.63516   78.18538       463.18208       512.70242      -382.34388           1
H    79.11860   88.08067   78.46322      1725.29117      1863.52247      -987.07854           1
C    79.98363   90.01048   78.83791       710.42801      -144.27427      -313.15367           1
H    79.09136   90.59425   78.49277       364.20195     -1033.13725      2494.72524           1
H    79.92157   89.93754   79.95453      2567.61904       335.80679       847.48802           1
H    80.89226   90.62006   78.59592     -2587.99029       680.92867      -512.16411           1
C    80.08076   88.78979   76.67133       268.17616       741.64379       -40.42634           1
H    79.19530   89.36272   76.29200      1283.54314     -1945.37420     -1411.27812           1
H    80.99851   89.33968   76.33738     -1258.57796      1317.15249     -2026.02085           1
H    80.08171   87.79764   76.15095     -2502.84526       909.80597       575.92391           1
C    82.43932   85.56211   78.87537       619.36578      -458.64843      -172.09273           1
O    83.48224   85.66381   78.26400       -81.40433       433.87611       522.34594           1
N    82.22939   84.95033   80.03998       175.23996      -622.20190       341.55523           1
```

H	81.28797	85.03800	80.41677	-876.57256	-303.22879	-2561.90213	1
C	83.31955	84.33941	80.71428	-571.88214	-534.10783	-106.15033	1
H	84.20797	84.75830	80.18324	-2713.20841	-16.25800	-248.80809	1
C	83.22048	84.74468	82.26200	166.60354	288.16399	-716.09568	1
H	82.29780	84.28117	82.70525	-2097.85574	1636.78449	-586.18044	1
O	83.12504	86.15355	82.53179	-317.80580	-363.38227	484.43082	1
H	82.26614	86.19872	82.99839	1863.74748	-1451.84923	1357.29467	1
C	84.48123	84.01775	82.85720	503.80731	517.99032	318.51606	1
H	84.51748	84.10545	83.96787	640.61020	-2157.10410	-1536.29104	1
H	84.49105	82.92896	82.60709	1687.88455	465.60692	2087.56452	1
H	85.42616	84.45582	82.45956	-1293.07895	-1899.88581	-1463.57452	1
C	83.27592	82.70590	80.61124	-153.07550	-733.68902	248.70981	1
O	84.35554	82.19575	80.44678	186.15459	-480.78787	-449.34387	1
N	82.10071	82.09733	80.84582	-487.53139	543.18517	42.03850	1
C	82.07121	80.69731	81.12669	-699.35871	69.34393	360.10701	1
H	83.02814	80.44699	81.63878	2.81126	-221.99807	-2715.58072	1
C	80.75500	80.57982	82.00760	-507.46439	-592.59194	122.07761	1
H	80.36194	79.53998	82.06295	-1975.08116	525.87560	-1801.71556	1
H	80.92746	80.91729	83.05414	1588.27743	-1913.22100	1113.83566	1
C	79.80932	81.55689	81.24345	-613.62442	-457.55993	-194.14490	1
H	79.42647	81.08123	80.30791	1707.54637	-721.27992	1996.92523	1
H	78.91679	81.86883	81.83145	758.23357	2100.81767	1560.55011	1
C	80.80463	82.75689	80.93442	-352.41524	704.96076	49.21465	1
H	80.62214	83.28647	79.96965	1714.76886	-2070.42778	443.35792	1
H	80.88863	83.50802	81.75268	1224.15040	1801.50881	1636.97633	1
C	81.85644	79.80709	79.75525	566.85117	537.05831	117.62814	1
O	82.04188	78.61315	79.80706	-550.26779	-406.08867	4.21666	1
N	81.44033	80.55868	78.74910	566.34832	-425.77725	180.18749	1
H	81.40290	81.55592	78.94803	644.44502	2646.80550	-52.73430	1
C	81.00048	80.03220	77.50585	612.34801	-281.48662	411.56221	1
H	80.79149	80.94100	76.90000	1667.23498	-1490.75142	1556.16753	1
C	82.28289	79.26127	76.94362	-714.45746	-175.43074	286.98927	1
H	82.54591	78.45904	77.68915	-787.09484	1763.03619	-1922.46060	1
O	83.44862	80.11703	76.83860	-429.46514	516.51932	-128.40526	1
H	83.93466	79.85940	77.64242	1981.82785	436.16847	-1818.18182	1
C	81.90632	78.58571	75.57953	630.79189	-468.94448	76.03230	1
H	82.80578	78.13295	75.10091	-1775.71861	2062.30353	131.89849	1
H	81.16449	77.76333	75.69892	-1855.14608	88.94118	1993.53760	1
H	81.48944	79.32457	74.85544	-1504.82350	-780.94249	-2132.90992	1
C	79.61832	79.23257	77.82290	288.57638	618.62814	-397.00270	1
O	79.03114	79.34185	78.87824	321.70495	-185.26452	574.37218	1
N	79.20876	78.57965	76.74107	-447.05792	-446.53431	367.76425	1
H	79.72543	78.63706	75.86587	350.31316	1975.63644	-1843.31522	1
C	78.00318	77.83290	76.81159	-511.23061	374.03917	-471.51480	1
H	77.42219	78.27983	77.65239	2234.79579	-898.70890	-1273.45190	1
C	77.32661	77.95734	75.35934	529.53781	-159.34528	563.72604	1
H	76.33630	77.43639	75.42427	654.59388	2347.94251	1217.51488	1
H	77.11353	79.04624	75.17876	-1526.55661	2103.42026	817.87394	1
O	78.12511	77.49340	74.24264	-554.84278	14.12789	399.58769	1
H	77.95580	76.53426	74.30555	1297.06236	2061.84854	1220.68812	1
C	78.24845	76.24011	77.01991	-662.15484	-412.13544	123.62687	1
O	77.29052	75.50873	77.22150	-540.01537	-317.05804	274.91456	1
N	79.52308	75.84118	76.78637	-202.76299	-690.00100	-131.49145	1
H	80.00766	76.73343	76.64093	-702.62132	-2617.37191	281.70413	1
C	80.17073	74.80363	77.61106	-491.73914	576.34187	222.73509	1
H	81.25883	75.01803	77.49707	1977.17978	-1823.58850	-434.68928	1
C	79.69525	74.94913	79.03967	-649.50797	-328.66843	306.11224	1
H	78.58345	74.84854	79.02767	2075.07803	1452.16490	-1004.45904	1
C	80.26774	73.85883	79.92880	-615.18735	-489.99689	-70.96352	1
H	79.87228	73.92397	80.97893	-1226.32661	-1724.38906	-1716.47170	1
H	80.01680	72.83273	79.53812	2381.64273	-969.84735	900.46860	1
H	81.38864	73.92156	79.98902	2122.74991	1702.23946	-141.36228	1

C	80.02405	76.30976	79.62807	-501.86074	-479.61276	-376.42482	1
H	79.69149	76.39082	80.69879	993.79261	-2246.95401	-1177.81305	1
H	81.12992	76.52618	79.60798	42.71279	-626.15391	-2651.37258	1
H	79.51999	77.14280	79.06166	-2026.39012	-1590.33709	-887.82922	1
C	79.77782	73.30319	77.05124	-672.89153	382.57394	-156.34072	1
O	80.44906	72.34535	77.34677	410.04627	221.86223	500.35950	1
N	78.66444	73.33122	76.33356	-378.11782	-443.79414	-441.10984	1
H	78.15328	74.22675	76.23957	2404.15973	-568.14894	1149.30085	1
C	78.05162	72.06057	76.06342	-544.54484	466.16019	331.29119	1
H	78.29626	71.43503	76.95025	-1409.80988	-170.17955	2325.32679	1
C	76.52287	72.35493	75.81409	-184.48284	116.78282	-758.89137	1
H	76.09894	73.01395	76.60607	1893.78641	1861.14652	611.04528	1
H	76.40303	72.91025	74.85660	-2170.79665	-974.19311	1327.50117	1
C	75.78552	70.97220	75.76683	389.51668	672.81878	-138.49152	1
H	76.31377	70.28744	75.06567	510.64577	1933.03327	1851.02484	1
H	75.85157	70.45866	76.75197	-2302.30869	-1105.72939	-948.89774	1
C	74.29173	71.09570	75.33747	-559.93427	-126.34590	542.30857	1
O	73.54467	70.13347	75.45868	-670.36003	-111.83166	76.36448	1
N	73.97793	72.27437	74.78738	-216.06729	-537.07037	446.51379	1
H	74.57413	73.09720	74.63863	-1251.96487	1074.12894	-2168.52500	1
H	73.08863	72.48954	74.32182	-2451.83023	-720.73851	944.84612	1
C	78.66183	71.48540	74.65478	546.24052	-563.88337	-85.11809	1
O	78.40385	72.04719	73.62328	71.70702	-307.40463	606.69673	1
N	79.29795	70.32560	74.85405	479.90863	-504.50129	222.86849	1
C	80.28773	69.84278	73.93196	75.51268	-600.91904	506.73700	1
H	80.92632	70.71979	73.68013	-2033.61562	1795.30103	254.89480	1
C	81.02351	68.70517	74.75459	523.79067	517.99274	284.45581	1
H	81.43533	67.90188	74.10279	1599.61665	-1127.00911	1895.98173	1
H	81.88397	69.11539	75.32922	-1881.83093	1275.82488	-1501.55016	1
C	79.90177	68.20246	75.71626	-600.05639	-404.05026	316.64485	1
H	79.18353	67.53500	75.19010	-2597.05260	496.36221	657.73249	1
H	80.28412	67.63362	76.59335	-1666.24518	1294.38985	-1723.90591	1
C	79.23104	69.58970	76.10912	493.57570	-129.95376	-602.56394	1
H	78.16755	69.52969	76.44130	-1469.08415	-637.84797	2204.22581	1
H	79.82422	70.20359	76.83515	-1038.97840	1068.88701	-2280.71789	1
C	79.59823	69.22402	72.58921	188.88100	241.05089	-727.87821	1
O	80.27887	68.98692	71.61855	531.88613	-49.19943	427.07960	1
N	78.29886	68.96593	72.75654	708.14833	26.93417	-179.73509	1
H	77.94596	69.19491	73.68435	-2232.69501	-1179.09831	1023.94816	1
C	77.51573	68.36263	71.68974	-738.32412	259.92548	104.42456	1
H	78.25491	68.24163	70.85877	2028.89123	1260.02983	1311.33324	1
C	76.98201	67.03679	72.21299	-258.65678	-345.52513	661.28459	1
H	76.39936	67.24650	73.13731	-1826.06090	-1801.46097	-918.64554	1
C	76.06430	66.34670	71.22241	-541.23400	531.92970	-218.41428	1
H	75.72856	65.33995	71.59654	1311.73218	-2219.18200	882.19023	1
H	75.13646	66.95273	71.01825	-1048.71298	1223.58118	-2196.97971	1
H	76.57494	66.18210	70.23309	419.06414	1903.92898	1903.44670	1
C	78.11888	66.10253	72.58842	-423.86431	152.66566	-648.55262	1
H	77.74550	65.16099	73.07730	105.68386	-2342.50080	1387.51238	1
H	78.71376	65.79622	71.68402	-1695.63603	-1484.73621	-1531.02793	1
H	78.83708	66.59354	73.30073	-1853.57121	-487.39031	1936.59343	1
C	76.26668	69.39704	71.28688	539.71428	-510.58232	267.58633	1
O	75.55885	69.07880	70.36463	-15.93758	-278.34085	-624.49402	1
N	76.16822	70.43416	72.09658	-676.81886	12.05292	276.17960	1
H	76.91822	70.52558	72.78223	-1694.44250	-373.84189	-2100.66098	1
C	75.29519	71.53692	71.86854	412.33078	-664.70360	108.35451	1
H	75.31913	72.06823	72.84678	1765.77476	1294.44366	1621.76594	1
C	75.83130	72.40799	70.65644	-179.66635	-721.95667	-264.73915	1
H	76.92888	72.58176	70.73176	-607.34922	2587.87032	-598.10018	1
H	75.65733	71.87590	69.69147	-359.69332	-1479.13954	-2259.74260	1
C	75.01933	73.74479	70.72398	-240.01076	616.02425	431.85322	1
H	73.92745	73.52524	70.66820	-1359.17131	782.54425	2227.99181	1

H	75.17796	74.25452	71.70086	-820.36667	85.81767	-2596.78715	1
C	75.46396	74.68129	69.53105	-513.07015	236.18570	-551.87280	1
H	76.54831	74.92823	69.60388	1816.11944	-1183.11722	-1650.94321	1
H	75.33320	74.16800	68.55062	-1800.04429	-1361.53473	1526.34643	1
C	74.60024	75.96273	69.60345	726.08972	-253.19732	-179.64713	1
H	73.52815	75.81294	69.36781	1224.27271	-456.51798	-2390.90304	1
H	74.70235	76.53462	70.54730	1206.40339	-1150.93514	-2154.90344	1
N	75.11784	76.94214	68.49721	329.70796	-633.68968	-155.67358	1
H	74.60153	77.86599	68.42568	1348.31305	1938.76845	1359.00643	1
H	76.12241	77.27414	68.58512	2096.92035	1446.54551	-966.48890	1
H	75.08429	76.60391	67.49155	-1197.07217	-2303.53719	827.28733	1
C	73.81471	71.08056	71.35357	-378.01186	-556.17301	-413.96481	1
O	73.47502	71.33634	70.19664	-409.44433	-307.55053	-453.30833	1
N	73.10379	70.57957	72.34421	-429.02477	524.70884	274.08502	1
H	73.63894	70.56279	73.21887	1706.34263	-1425.96036	1574.38926	1
C	72.30432	69.39858	72.10030	-45.25864	-626.22952	478.93267	1
H	72.99078	68.74860	71.51390	1988.63164	570.90773	1772.87287	1
C	71.99149	68.86955	73.54666	551.77128	-20.49526	-564.54996	1
H	72.91387	68.46838	74.02862	1434.61535	-1753.10253	1513.99471	1
H	71.68965	69.73006	74.18876	-714.67738	1819.09770	1898.36494	1
C	70.86416	67.78224	73.53117	446.99263	-555.77305	338.97221	1
H	70.35711	67.74045	72.54001	1953.68466	1892.25957	-161.67766	1
H	71.26496	66.76052	73.71783	-403.03785	2688.07032	-188.43591	1
C	69.85017	68.23689	74.65693	567.86262	335.74681	434.04421	1
H	70.30601	68.13391	75.66898	-1080.02275	1568.26081	-1948.78900	1
H	69.60756	69.31898	74.54308	-919.54108	2111.34692	1456.13463	1
C	68.55771	67.39765	74.53705	195.81897	173.33862	-745.11536	1
H	68.00101	67.54477	73.58986	1405.67949	-2301.21089	-390.08113	1
H	68.68124	66.31516	74.74134	-940.37328	-1118.32446	2299.72133	1
N	67.58378	67.91397	75.65055	319.26422	656.36007	-42.02424	1
H	66.62032	67.46942	75.68415	1961.03633	619.91968	1787.09422	1
H	67.90185	67.80289	76.65691	854.14343	-2156.80752	1429.08711	1
H	67.33767	68.94505	75.61786	1237.02905	-1378.50857	-1998.28500	1
C	70.96433	69.77961	71.24716	532.53319	-268.57236	517.55729	1
O	69.87553	69.83493	71.76139	266.16714	-290.73804	-558.88002	1
N	71.30406	69.87609	69.96279	-226.25352	640.49678	-270.32839	1
H	72.31088	69.88431	69.73507	2007.69063	-1035.27229	1523.50231	1
C	70.36985	70.36167	68.99731	-540.76542	-573.33303	49.50000	1
H	70.96996	70.35630	68.05891	-2374.20950	-1036.76023	843.75774	1
C	69.11082	69.37534	68.94384	-77.09082	666.87422	-415.84136	1
H	68.56861	69.46397	69.92668	-985.81834	617.19825	2463.91927	1
O	69.41841	67.98344	68.75508	293.29899	-391.65602	-477.80926	1
H	69.11798	67.60981	69.60807	2528.72714	-681.76262	-751.27122	1
C	68.25437	70.01964	67.79232	252.47363	-605.14473	440.05134	1
H	67.29035	69.47844	67.65112	-2052.65063	1456.49051	-1043.51830	1
H	68.00783	71.08957	68.00282	-2152.98800	-1661.97574	-161.70652	1
H	68.79484	69.98756	66.81758	-473.12726	-1584.27339	2165.61725	1
C	69.98119	71.86229	69.48829	360.36307	488.46713	505.10056	1
O	68.82490	72.23046	69.64184	-200.08817	-528.03552	-385.82850	1
N	71.10771	72.53277	69.57147	-597.54095	-226.44775	355.20479	1
H	72.01328	72.02410	69.45217	2179.23655	-1300.70981	-991.33835	1
C	71.15057	73.86356	70.05100	-687.68921	297.39807	-249.45179	1
H	72.17496	74.19603	69.77926	-349.16256	-2467.37713	-1101.72831	1
C	69.99408	74.75224	69.39531	672.47345	361.54328	-201.62880	1
H	68.99392	74.43107	69.77337	1541.41502	582.18425	2169.97026	1
H	70.12499	75.81416	69.70768	-2534.67717	60.25153	997.72295	1
C	70.04210	74.62235	67.86516	574.26774	-389.43119	-377.02542	1
C	71.09897	75.21703	67.11334	688.82679	-287.33580	-257.96133	1
H	71.89300	75.79872	67.61584	331.14182	1470.38163	2269.80013	1
C	69.02124	73.87387	67.20492	-383.52382	-675.04808	144.24917	1
H	68.20256	73.41073	67.78757	4.09847	1517.59662	2262.86397	1
C	71.10962	75.04148	65.72923	-134.76730	428.45285	-649.50303	1

H	71.92617	75.50093	65.14584	-903.23433	1600.49020	2011.53387	1
C	69.07647	73.72282	65.81783	-335.53281	-615.00040	-364.39058	1
H	68.28279	73.13604	65.32273	1596.45398	-1369.33862	1732.02659	1
C	70.11649	74.29644	65.02959	585.48547	-340.24194	-406.23938	1
O	70.18587	74.14762	63.61980	-419.15649	-173.04408	-511.94141	1
H	69.36656	73.61979	63.46759	-1071.84350	1559.71507	-1960.13012	1
C	70.94862	73.81069	71.67944	291.41878	-183.13091	710.72246	1
O	71.85989	74.17524	72.38405	379.87695	523.47396	-222.23622	1
N	69.75781	73.32794	71.98863	564.30591	373.09380	277.24371	1
H	69.13169	72.99835	71.22205	-120.59931	-2436.07824	-1214.35098	1
C	69.28007	73.35011	73.32632	404.07514	-299.37480	608.84009	1
H	68.43642	72.62355	73.33056	-1071.07882	-841.69025	2359.66467	1
C	70.50042	72.91003	74.24052	-683.12807	68.71770	-390.13096	1
H	71.03385	72.06597	73.74363	2068.88918	1596.58590	770.89603	1
H	71.24754	73.74465	74.30275	1379.86429	2342.49779	-179.85638	1
C	70.06063	72.49935	75.67827	-176.39967	537.49466	550.97268	1
H	69.56076	73.33733	76.21268	-396.28051	1942.22168	1869.33314	1
H	69.32730	71.66261	75.66959	-94.44700	586.00678	-2659.20016	1
C	71.42213	72.08288	76.41245	-291.69381	-718.18079	-150.72929	1
H	71.94297	71.15635	76.02371	-2303.38776	1089.76242	964.62032	1
H	72.15827	72.92513	76.45981	884.16729	2568.80533	-207.74283	1
N	70.99282	71.87280	77.72152	-594.49522	-252.81478	342.29276	1
H	70.27107	72.50013	78.05007	-1735.17426	2084.05705	-263.71605	1
C	71.45563	71.00809	78.53858	595.23177	-271.52487	442.22352	1
N	72.46025	70.14831	78.09850	585.53813	-424.79750	-105.81303	1
H	72.86297	70.07324	77.14688	959.88797	1327.56823	-2177.11905	1
H	72.88538	69.42130	78.68239	1578.48647	16.27378	-2220.76233	1
N	70.81272	71.14263	79.77595	-452.17763	-390.33541	-421.51853	1
H	70.06180	71.78481	80.04622	470.26107	1974.25629	-1817.92087	1
H	71.01374	70.55796	80.58913	1856.67307	-1573.97321	-1224.35416	1
C	68.77546	74.87225	73.64448	-531.47271	-581.45780	-55.06206	1
O	68.93284	75.82551	72.91082	-243.00837	192.73507	609.52485	1
N	68.13450	74.85752	74.80989	343.49288	-644.25607	38.09164	1
H	68.03266	73.97057	75.29896	-1691.16043	-1535.97525	-1484.73099	1
C	67.75950	76.13411	75.33373	542.66492	350.14337	454.42563	1
H	68.52690	76.83106	74.92394	1209.57979	-2396.95920	463.86744	1
C	66.31272	76.49047	74.76966	-737.14309	145.93010	242.71942	1
H	65.57408	75.71399	75.06272	-1050.18303	159.36419	-2509.06125	1
C	65.91794	77.88882	75.35204	-706.77696	-252.88343	-245.16230	1
H	64.88434	78.18523	75.05536	-1624.64039	-1665.49085	1417.86935	1
H	65.96244	77.88900	76.46954	2566.10521	598.51109	693.22305	1
H	66.60995	78.68862	74.99730	-1972.69758	107.10018	-1876.34312	1
C	66.40763	76.60513	73.20933	412.85148	672.58832	-27.69222	1
H	65.44746	76.95219	72.75921	1447.02277	1762.76533	-1490.78944	1
H	67.20614	77.32376	72.90139	1320.77379	-145.20455	2378.68491	1
H	66.66174	75.62161	72.74414	-1512.10848	1470.52031	1724.75147	1
C	67.65671	76.08835	76.96115	-607.11113	499.56689	73.73769	1
O	67.79365	77.11176	77.59584	357.97126	460.81534	356.68785	1
N	67.31125	74.88498	77.39944	536.55407	-368.62270	332.76306	1
H	67.17543	74.11332	76.74692	524.38866	2297.69985	1367.20941	1
C	67.20930	74.67544	78.79601	46.50277	-774.29190	-147.97904	1
H	67.62806	75.59492	79.26659	1676.51875	1883.29066	1032.55549	1
C	65.65581	74.41283	79.16296	-559.41456	-34.19198	-556.30433	1
H	65.36508	73.39710	78.80448	-64.66664	395.18769	2695.05373	1
H	65.51581	74.39473	80.26967	-2703.44278	-60.40056	333.79418	1
C	64.78447	75.45638	78.51951	-519.76435	377.31045	459.42366	1
H	65.02705	75.48940	77.43281	-1746.74323	1667.50644	-1261.73691	1
C	63.31313	75.09056	78.67292	570.95554	16.46261	-545.27700	1
H	62.64613	75.84034	78.17476	2391.58273	28.05277	-1305.07271	1
H	63.08606	74.09120	78.21996	175.75588	917.63458	2559.43867	1
H	63.01065	75.04197	79.75042	-1063.97851	-2155.34650	1283.00462	1
C	65.03868	76.83824	79.10485	193.76014	2.62946	-765.53141	1

```
H    64.35124  77.60625  78.66489       507.39394      -846.55087      2539.60089           1
H    64.88679  76.85352  80.21493      1337.57841      1818.19054      1526.01994           1
H    66.09285  77.17964  78.90047      1100.88635     -1172.91350     -2199.08909           1
C    68.01606  73.30734  79.07995      -549.21115       475.64486      -309.38218           1
O    68.00603  72.37342  78.32228       556.06106      -390.37755       -78.24250           1
O    68.69014  73.21582  80.33052      -572.27940      -310.19032      -209.75862           1
H    68.44464  74.11113  80.69302     -1380.08193      2212.14486       790.85944           1
```

Listing 50: C00050.xmolout

```
       276
  150.0000  150.0000  150.0000     0.0000     0.0000     0.0000***********    2.7750    11100
       0.2500    300.00    0.00    0.00    0.00  -1293.6872       0.0000    300.0000
H    61.16985  80.83259  69.48001     -2043.14101     -1757.64893       399.89339           1
N    62.12888  80.50896  69.61162       426.75047       490.45733      -334.42467           1
H    62.20793  79.68091  68.99573       442.25559     -1110.30854     -2448.52907           1
C    63.10329  81.48350  69.30867       415.32972      -666.39213        83.73172           1
H    62.63764  82.49151  69.36073       879.32219      2491.00447       667.35213           1
C    64.34844  81.29317  70.31870        31.35357       477.41777       628.23362           1
H    64.73368  80.24967  70.22318      1168.38489      2240.25337     -1019.71136           1
H    65.19229  81.96625  70.02632      -360.43770      2534.90498       931.67112           1
C    63.92514  81.54983  71.74042       463.18208       512.70242      -382.34388           1
H    63.00945  80.94968  71.94434      1725.29117      1863.52247      -987.07854           1
C    65.02548  81.10247  72.69544       710.42801      -144.27427      -313.15367           1
H    64.73283  81.25511  73.76608       364.20195     -1033.13725      2494.72524           1
H    65.26849  80.01541  72.56930      2567.61904       335.80679       847.48802           1
H    65.97434  81.67402  72.52650     -2587.99029       680.92867      -512.16411           1
C    63.60926  83.02084  71.97234       268.17616       741.64379       -40.42634           1
H    63.31798  83.21735  73.03650      1283.54314     -1945.37420     -1411.27812           1
H    64.49171  83.67277  71.74415     -1258.57796      1317.15249     -2026.02085           1
H    62.76231  83.36981  71.32724     -2502.84526       909.80597       575.92391           1
C    63.55442  81.00194  67.82950       619.36578      -458.64843      -172.09273           1
O    63.25721  79.87023  67.46349       -81.40433       433.87611       522.34594           1
N    64.30842  81.90308  67.21579       175.23996      -622.20190       341.55523           1
H    64.47509  82.80181  67.66836      -876.57256      -303.22879     -2561.90213           1
C    64.90282  81.55689  65.97551      -571.88214      -534.10783      -106.15033           1
H    64.45678  80.55605  65.75477     -2713.20841       -16.25800      -248.80809           1
C    64.56767  82.71205  64.91234       166.60354       288.16399      -716.09568           1
H    65.23168  83.59522  65.13080     -2097.85574      1636.78449      -586.18044           1
O    63.21890  83.20807  64.92094      -317.80580      -363.38227       484.43082           1
H    63.36361  84.14898  65.14945      1863.74748     -1451.84923      1357.29467           1
C    65.02120  82.04716  63.56082       503.80731       517.99032       318.51606           1
H    64.95401  82.76720  62.71238       640.61020     -2157.10410     -1536.29104           1
H    66.07666  81.68500  63.60704      1687.88455       465.60692      2087.56452           1
H    64.37867  81.17327  63.30297     -1293.07895     -1899.88581     -1463.57452           1
C    66.53420  81.56215  66.12717      -153.07550      -733.68902       248.70981           1
O    67.13758  82.39536  65.50266       186.15459      -480.78787      -449.34387           1
N    67.00394  80.66518  67.01023      -487.53139       543.18517        42.03850           1
C    68.33207  80.14871  66.94693      -699.35871        69.34393       360.10701           1
H    68.87898  80.80338  66.22894         2.81126      -221.99807     -2715.58072           1
C    68.75825  80.26518  68.47549      -507.46439      -592.59194       122.07761           1
H    69.67352  79.68116  68.72643     -1975.08116       525.87560     -1801.71556           1
H    68.96688  81.32120  68.75634      1588.27743     -1913.22100      1113.83566           1
C    67.46751  79.73761  69.17612      -613.62442      -457.55993      -194.14490           1
H    67.40943  78.62718  69.13716      1707.54637      -721.27992      1996.92523           1
H    67.39706  80.01763  70.25158       758.23357      2100.81767      1560.55011           1
C    66.33653  80.40214  68.28154      -352.41524       704.96076        49.21465           1
H    65.45991  79.74372  68.06241      1714.76886     -2070.42778       443.35792           1
H    65.97991  81.39868  68.63679      1224.15040      1801.50881      1636.97633           1
C    68.21302  78.58539  66.45544       566.85117       537.05831       117.62814           1
O    67.56094  78.32174  65.47461      -550.26779      -406.08867         4.21666           1
N    68.95763  77.77095  67.19986       566.34832      -425.77725       180.18749           1
```

H	69.48029	78.16149	67.98380	644.44502	2646.80550	-52.73430	1
C	68.66267	76.38176	67.12954	612.34801	-281.48662	411.56221	1
H	68.22923	76.23260	66.11341	1667.23498	-1490.75142	1556.16753	1
C	70.01057	75.58173	67.43604	-714.45746	-175.43074	286.98927	1
H	70.35338	75.83500	68.47890	-787.09484	1763.03619	-1922.46060	1
O	71.14333	75.87455	66.59766	-429.46514	516.51932	-128.40526	1
H	71.76391	76.26308	67.24764	1981.82785	436.16847	-1818.18182	1
C	69.47928	74.10114	67.39617	630.79189	-468.94448	76.03230	1
H	70.24826	73.37937	67.75663	-1775.71861	2062.30353	131.89849	1
H	68.56828	73.96922	68.02984	-1855.14608	88.94118	1993.53760	1
H	69.20262	73.80002	66.35915	-1504.82350	-780.94249	-2132.90992	1
C	67.61342	75.99050	68.34254	288.57638	618.62814	-397.00270	1
O	66.68152	75.25912	68.10186	321.70495	-185.26452	574.37218	1
N	68.05321	76.43720	69.50672	-447.05792	-446.53431	367.76425	1
H	68.87251	77.03870	69.47927	350.31316	1975.63644	-1843.31522	1
C	67.31519	76.20177	70.70998	-511.23061	374.03917	-471.51480	1
H	66.31279	76.65084	70.53732	2234.79579	-898.70890	-1273.45190	1
C	67.21478	74.61892	70.90928	529.53781	-159.34528	563.72604	1
H	66.51718	74.43242	71.77147	654.59388	2347.94251	1217.51488	1
H	66.71754	74.20394	69.98808	-1526.55661	2103.42026	817.87394	1
O	68.45141	73.91139	71.20829	-554.84278	14.12789	399.58769	1
H	68.82523	73.86146	70.31025	1297.06236	2061.84854	1220.68812	1
C	68.13521	76.86003	71.94683	-662.15484	-412.13544	123.62687	1
O	67.53920	77.26252	72.91667	-540.01537	-317.05804	274.91456	1
N	69.44296	76.77582	71.73607	-202.76299	-690.00100	-131.49145	1
H	69.84190	76.40148	70.85877	-702.62132	-2617.37191	281.70413	1
C	70.40188	77.29867	72.64461	-491.73914	576.34187	222.73509	1
H	69.93942	78.14745	73.19735	1977.17978	-1823.58850	-434.68928	1
C	70.81459	76.10545	73.61639	-649.50797	-328.66843	306.11224	1
H	70.96035	75.17228	73.03003	2075.07803	1452.16490	-1004.45904	1
C	72.09587	76.44782	74.44711	-615.18735	-489.99689	-70.96352	1
H	72.29355	75.67709	75.22851	-1226.32661	-1724.38906	-1716.47170	1
H	73.01756	76.49417	73.78230	2381.64273	-969.84735	900.46860	1
H	72.00111	77.42928	74.96902	2122.74991	1702.23946	-141.36228	1
C	69.66817	75.91835	74.66765	-501.86074	-479.61276	-376.42482	1
H	69.86831	75.05864	75.35018	993.79261	-2246.95401	-1177.81305	1
H	69.54241	76.82678	75.30266	42.71279	-626.15391	-2651.37258	1
H	68.68800	75.72944	74.16458	-2026.39012	-1590.33709	-887.82922	1
C	71.56558	77.67605	71.56313	-672.89153	382.57394	-156.34072	1
O	71.46634	77.28497	70.40967	410.04627	221.86223	500.35950	1
N	72.49010	78.44674	72.11963	-378.11782	-443.79414	-441.10984	1
H	72.39497	78.60443	73.12265	2404.15973	-568.14894	1149.30085	1
C	73.76381	78.59081	71.47326	-544.54484	466.16019	331.29119	1
H	73.52893	78.89344	70.42896	-1409.80988	-170.17955	2325.32679	1
C	74.52429	79.68062	72.33541	-184.48284	116.78282	-758.89137	1
H	73.91279	80.60838	72.40530	1893.78641	1861.14652	611.04528	1
H	74.64729	79.32151	73.38201	-2170.79665	-974.19311	1327.50117	1
C	75.91628	79.99068	71.70235	389.51668	672.81878	-138.49152	1
H	76.53589	79.06496	71.66470	510.64577	1933.03327	1851.02484	1
H	75.79915	80.28539	70.63461	-2302.30869	-1105.72939	-948.89774	1
C	76.69909	81.12529	72.46689	-559.93427	-126.34590	542.30857	1
O	77.75567	81.50950	72.03624	-670.36003	-111.83166	76.36448	1
N	76.07845	81.57795	73.57755	-216.06729	-537.07037	446.51379	1
H	75.18943	81.28476	73.99881	-1251.96487	1074.12894	-2168.52500	1
H	76.46011	82.30757	74.18958	-2451.83023	-720.73851	944.84612	1
C	74.54399	77.16836	71.55015	546.24052	-563.88337	-85.11809	1
O	74.49877	76.49993	72.55860	71.70702	-307.40463	606.69673	1
N	75.24074	76.95850	70.43124	479.90863	-504.50129	222.86849	1
C	75.94222	75.72151	70.21969	75.51268	-600.91904	506.73700	1
H	75.22642	74.90183	70.45666	-2033.61562	1795.30103	254.89480	1
C	76.36498	75.82042	68.69368	523.79067	517.99274	284.45581	1
H	77.28109	75.23202	68.46210	1599.61665	-1127.00911	1895.98173	1

114	H	75.56440	75.43466	68.02365	-1881.83093	1275.82488	-1501.55016	1
115	C	76.55937	77.35665	68.50848	-600.05639	-404.05026	316.64485	1
116	H	77.51197	77.70129	68.97108	-2597.05260	496.36221	657.73249	1
117	H	76.57617	77.68478	67.44473	-1666.24518	1294.38985	-1723.90591	1
118	C	75.29614	77.89036	69.31426	493.57570	-129.95376	-602.56394	1
119	H	75.38588	78.93246	69.70151	-1469.08415	-637.84797	2204.22581	1
120	H	74.32614	77.77708	68.77301	-1038.97840	1068.88701	-2280.71789	1
121	C	77.25851	75.67887	71.18357	188.88100	241.05089	-727.87821	1
122	O	77.86209	76.67828	71.49308	531.88613	-49.19943	427.07960	1
123	N	77.61388	74.39646	71.34832	708.14833	26.93417	-179.73509	1
124	H	76.92513	73.76900	70.92860	-2232.69501	-1179.09831	1023.94816	1
125	C	78.40051	73.90278	72.48329	-738.32412	259.92548	104.42456	1
126	H	78.92517	73.02971	72.03186	2028.89123	1260.02983	1311.33324	1
127	C	79.34635	74.95729	73.00871	-258.65678	-345.52513	661.28459	1
128	H	78.76265	75.88205	73.24359	-1826.06090	-1801.46097	-918.64554	1
129	C	80.03809	74.50673	74.28155	-541.23400	531.92970	-218.41428	1
130	H	80.78757	75.26583	74.63665	1311.73218	-2219.18200	882.19023	1
131	H	79.29184	74.35017	75.13252	-1048.71298	1223.58118	-2196.97971	1
132	H	80.58782	73.53726	74.13479	419.06414	1903.92898	1903.44670	1
133	C	80.38660	75.33698	71.96763	-423.86431	152.66566	-648.55262	1
134	H	81.08104	76.13616	72.34475	105.68386	-2342.50080	1387.51238	1
135	H	81.01747	74.45671	71.66720	-1695.63603	-1484.73621	-1531.02793	1
136	H	79.89389	75.73380	71.03596	-1853.57121	-487.39031	1936.59343	1
137	C	77.30929	73.34974	73.61665	539.71428	-510.58232	267.58633	1
138	O	77.17508	72.15533	73.73131	-15.93758	-278.34085	-624.49402	1
139	N	76.68442	74.32458	74.25351	-676.81886	12.05292	276.17960	1
140	H	76.90814	75.27365	73.96039	-1694.44250	-373.84189	-2100.66098	1
141	C	75.96515	74.05518	75.45622	412.33078	-664.70360	108.35451	1
142	H	75.57179	75.06627	75.70557	1765.77476	1294.44366	1621.76594	1
143	C	74.82349	73.02871	75.07504	-179.66635	-721.95667	-264.73915	1
144	H	74.41270	73.28238	74.07165	-607.34922	2587.87032	-598.10018	1
145	H	75.25355	72.00539	74.96139	-359.69332	-1479.13954	-2259.74260	1
146	C	73.68129	73.06046	76.14494	-240.01076	616.02425	431.85322	1
147	H	74.06565	72.83341	77.16439	-1359.17131	782.54425	2227.99181	1
148	H	73.22864	74.07389	76.21492	-820.36667	85.81767	-2596.78715	1
149	C	72.60226	71.99084	75.70491	-513.07015	236.18570	-551.87280	1
150	H	72.12772	72.28033	74.73897	1816.11944	-1183.11722	-1650.94321	1
151	H	73.07919	71.00016	75.52303	-1800.04429	-1361.53473	1526.34643	1
152	C	71.54619	71.88359	76.82905	726.08972	-253.19732	-179.64713	1
153	H	71.93279	71.48183	77.78700	1224.27271	-456.51798	-2390.90304	1
154	H	70.96878	72.81155	77.01250	1206.40339	-1150.93514	-2154.90344	1
155	N	70.47747	70.83563	76.36646	329.70796	-633.68968	-155.67358	1
156	H	69.69732	70.63537	77.05676	1348.31305	1938.76845	1359.00643	1
157	H	69.92521	71.06707	75.49007	2096.92035	1446.54551	-966.48890	1
158	H	70.82407	69.85514	76.15374	-1197.07217	-2303.53719	827.28733	1
159	C	77.05762	73.54582	76.56050	-378.01186	-556.17301	-413.96481	1
160	O	78.11234	74.16650	76.68977	-409.44433	-307.55053	-453.30833	1
161	N	76.60882	72.55495	77.30119	-429.02477	524.70884	274.08502	1
162	H	75.69902	72.20909	77.00239	1706.34263	-1425.96036	1574.38926	1
163	C	77.40720	71.71290	78.12709	-45.25864	-626.22952	478.93267	1
164	H	76.71395	70.86290	78.33283	1988.63164	570.90773	1772.87287	1
165	C	78.69051	71.25375	77.31360	551.77128	-20.49526	-564.54996	1
166	H	78.44068	71.04561	76.24855	1434.61535	-1753.10253	1513.99471	1
167	H	79.45854	72.05975	77.29483	-714.67738	1819.09770	1898.36494	1
168	C	79.21127	69.97518	78.04852	446.99263	-555.77305	338.97221	1
169	H	79.34901	70.18143	79.13602	1953.68466	1892.25957	-161.67766	1
170	H	78.45176	69.15567	77.99131	-403.03785	2688.07032	-188.43591	1
171	C	80.58152	69.50272	77.42021	567.86262	335.74681	434.04421	1
172	H	80.47475	69.28078	76.33356	-1080.02275	1568.26081	-1948.78900	1
173	H	81.35929	70.30005	77.49750	-919.54108	2111.34692	1456.13463	1
174	C	80.98594	68.23807	78.21701	195.81897	173.33862	-745.11536	1
175	H	81.16714	68.41048	79.29938	1405.67949	-2301.21089	-390.08113	1

H	80.30589	67.37111	78.08615	-940.37328	-1118.32446	2299.72133	1
N	82.36937	67.76076	77.67221	319.26422	656.36007	-42.02424	1
H	82.80857	66.95773	78.20857	1961.03633	619.91968	1787.09422	1
H	82.41023	67.40118	76.67398	854.14343	-2156.80752	1429.08711	1
H	83.15570	68.47100	77.68608	1237.02905	-1378.50857	-1998.28500	1
C	77.94946	72.40464	79.50158	532.53319	-268.57236	517.55729	1
O	78.31459	71.66292	80.40577	266.16714	-290.73804	-558.88002	1
N	77.97611	73.70696	79.41792	-226.25352	640.49678	-270.32839	1
H	77.71683	74.12941	78.50975	2007.69063	-1035.27229	1523.50231	1
C	78.15862	74.57134	80.53486	-540.76542	-573.33303	49.50000	1
H	77.52924	75.44209	80.24601	-2374.20950	-1036.76023	843.75774	1
C	77.69160	73.88293	81.89030	-77.09082	666.87422	-415.84136	1
H	78.37554	73.01344	82.11649	-985.81834	617.19825	2463.91927	1
O	76.36360	73.29602	81.90294	293.29899	-391.65602	-477.80926	1
H	76.59852	72.36668	81.69476	2528.72714	-681.76262	-751.27122	1
C	77.94582	75.04669	82.91587	252.47363	-605.14473	440.05134	1
H	77.81641	74.70182	83.96770	-2052.65063	1456.49051	-1043.51830	1
H	78.97681	75.46314	82.82509	-2152.98800	-1661.97574	-161.70652	1
H	77.23573	75.89071	82.75471	-473.12726	-1584.27339	2165.61725	1
C	79.74445	75.00124	80.50170	360.36307	488.46713	505.10056	1
O	80.11374	75.84264	79.71719	-200.08817	-528.03552	-385.82850	1
N	80.46445	74.32209	81.38806	-597.54095	-226.44775	355.20479	1
H	80.02681	73.60845	81.96897	2179.23655	-1300.70981	-991.33835	1
C	81.85794	74.54008	81.43007	-687.68921	297.39807	-249.45179	1
H	82.06512	75.30005	80.63955	-349.16256	-2467.37713	-1101.72831	1
C	82.33985	74.95307	82.90508	672.47345	361.54328	-201.62880	1
H	82.06826	74.16507	83.64248	1541.41502	582.18425	2169.97026	1
H	83.45187	75.03175	82.91711	-2534.67717	60.25153	997.72295	1
C	81.68789	76.29093	83.28162	574.26774	-389.43119	-377.02542	1
C	82.08249	77.49031	82.61575	688.82679	-287.33580	-257.96133	1
H	82.86558	77.47217	81.83619	331.14182	1470.38163	2269.80013	1
C	80.67054	76.32227	84.28255	-383.52382	-675.04808	144.24917	1
H	80.36082	75.39557	84.79859	4.09847	1517.59662	2262.86397	1
C	81.45201	78.68482	82.96201	-134.76730	428.45285	-649.50303	1
H	81.75475	79.61192	82.44429	-903.23433	1600.49020	2011.53387	1
C	80.06810	77.54367	84.59351	-335.53281	-615.00040	-364.39058	1
H	79.28068	77.55506	85.36713	1596.45398	-1369.33862	1732.02659	1
C	80.43082	78.76431	83.95289	585.48547	-340.24194	-406.23938	1
O	79.82561	80.01030	84.26178	-419.15649	-173.04408	-511.94141	1
H	79.19879	79.72128	84.96605	-1071.84350	1559.71507	-1960.13012	1
C	82.57772	73.07909	81.23507	291.41878	-183.13091	710.72246	1
O	83.53147	72.79819	81.93210	379.87695	523.47396	-222.23622	1
N	81.98376	72.36998	80.28387	564.30591	373.09380	277.24371	1
H	81.19774	72.82287	79.81575	-120.59931	-2436.07824	-1214.35098	1
C	82.52049	71.12710	79.83846	404.07514	-299.37480	608.84009	1
H	82.12666	71.03178	78.79970	-1071.07882	-841.69025	2359.66467	1
C	84.10665	71.20134	79.92598	-683.12807	68.71770	-390.13096	1
H	84.47342	72.18133	79.54531	2068.88918	1596.58590	770.89603	1
H	84.43319	71.15219	80.99678	1379.86429	2342.49779	-179.85638	1
C	84.72555	70.02028	79.11788	-176.39967	537.49466	550.97268	1
H	84.30249	69.04623	79.45951	-396.28051	1942.22168	1869.33314	1
H	84.48394	70.09951	78.03479	-94.44700	586.00678	-2659.20016	1
C	86.30460	70.07824	79.36682	-291.69381	-718.18079	-150.72929	1
H	86.82575	71.00937	79.02985	-2303.38776	1089.76242	964.62032	1
H	86.60297	69.86480	80.42538	884.16729	2568.80533	-207.74283	1
N	86.74688	69.00763	78.57861	-594.49522	-252.81478	342.29276	1
H	86.06985	68.28669	78.38073	-1735.17426	2084.05705	-263.71605	1
C	87.92943	68.86671	78.13018	595.23177	-271.52487	442.22352	1
N	88.87393	69.85464	78.42807	585.53813	-424.79750	-105.81303	1
H	88.77558	70.69747	78.99191	959.88797	1327.56823	-2177.11905	1
H	89.85059	69.83540	78.13347	1578.48647	16.27378	-2220.76233	1
N	88.04495	67.67939	77.39338	-452.17763	-390.33541	-421.51853	1

```
H    87.36097   66.95579   77.17890       470.26107      1974.25629     -1817.92087           1
H    88.91532   67.38009   76.95263      1856.67307     -1573.97321     -1224.35416           1
C    81.99731   69.93313   80.80503      -531.47271      -581.45780       -55.06206           1
O    82.65074   68.92907   80.95949      -243.00837       192.73507       609.52485           1
N    80.73448   70.14710   81.23651       343.49288      -644.25607        38.09164           1
H    80.40035   71.08273   80.98905     -1691.16043     -1535.97525     -1484.73099           1
C    80.36164   69.54425   82.53079       542.66492       350.14337       454.42563           1
H    81.27591   68.97971   82.83511      1209.57979     -2396.95920       463.86744           1
C    79.96163   70.66586   83.46932      -737.14309       145.93010       242.71942           1
H    79.12854   71.22491   82.98683     -1050.18303       159.36419     -2509.06125           1
C    79.45552   70.11943   84.79214      -706.77696      -252.88343      -245.16230           1
H    79.06376   70.93473   85.46051     -1624.64039     -1665.49085      1417.86935           1
H    78.62236   69.37732   84.64000      2566.10521       598.51109       693.22305           1
H    80.26982   69.58634   85.35625     -1972.69758       107.10018     -1876.34312           1
C    81.09761   71.64335   83.70498       412.85148       672.58832       -27.69222           1
H    80.76323   72.52918   84.31222      1447.02277      1762.76533     -1490.78944           1
H    81.94673   71.15874   84.26107      1320.77379      -145.20455      2378.68491           1
H    81.52978   72.03615   82.73569     -1512.10848      1470.52031      1724.75147           1
C    79.05384   68.56292   82.29224      -607.11113       499.56689        73.73769           1
O    78.94003   67.54737   82.93107       357.97126       460.81534       356.68785           1
N    78.20428   69.10408   81.43499       536.55407      -368.62270       332.76306           1
H    78.39832   70.03056   80.98405       524.38866      2297.69985      1367.20941           1
C    77.00624   68.39186   81.17484        46.50277      -774.29190      -147.97904           1
H    77.05619   67.47886   81.81571      1676.51875      1883.29066      1032.55549           1
C    75.74625   69.35265   81.48975      -559.41456       -34.19198      -556.30433           1
H    75.81335   70.26910   80.85670       -64.66664       395.18769      2695.05373           1
H    74.79284   68.84812   81.20298     -2703.44278       -60.40056       333.79418           1
C    75.74828   69.71169   82.95078      -519.76435       377.31045       459.42366           1
H    76.75712   70.10266   83.21488     -1746.74323      1667.50644     -1261.73691           1
C    74.71748   70.79674   83.23611       570.95554        16.46261      -545.27700           1
H    74.67512   71.04931   84.32693      2391.58273        28.05277     -1305.07271           1
H    74.94841   71.74857   82.68543       175.75588       917.63458      2559.43867           1
H    73.68790   70.47701   82.93059     -1063.97851     -2155.34650      1283.00462           1
C    75.45849   68.49628   83.82199       193.76014         2.62946      -765.53141           1
H    75.37980   68.77277   84.90497       507.39394      -846.55087      2539.60089           1
H    74.49383   68.00302   83.53590      1337.57841      1818.19054      1526.01994           1
H    76.26593   67.72351   83.73788      1100.88635     -1172.91350     -2199.08909           1
C    77.05520   68.08740   79.58882      -549.21115       475.64486      -309.38218           1
O    76.36386   68.64800   78.77958       556.06106      -390.37755       -78.24250           1
O    78.04116   67.15744   79.18406      -572.27940      -310.19032      -209.75862           1
H    78.43162   66.94896   80.08314     -1380.08193      2212.14486       790.85944           1
```

Listing 51: C00051.xmolout

```
       276
  150.0000  150.0000  150.0000     0.0000     0.0000     0.0000***********    2.7750    11100
       0.2500    300.00    0.00    0.00    0.00   -1293.6855       0.0000     300.0000
H    64.75932   75.73609   74.33608     -2043.14101     -1757.64893       399.89339           1
N    65.63330   75.22322   74.22643       426.75047       490.45733      -334.42467           1
H    65.38206   74.48753   73.55695       442.25559     -1110.30854     -2448.52907           1
C    66.71825   76.00602   73.76681       415.32972      -666.39213        83.73172           1
H    66.38018   77.06232   73.84907       879.32219      2491.00447       667.35213           1
C    68.01162   75.71433   74.68881        31.35357       477.41777       628.23362           1
H    68.33869   74.65790   74.54022      1168.38489      2240.25337     -1019.71136           1
H    68.87167   76.35110   74.37173      -360.43770      2534.90498       931.67112           1
C    67.66386   75.95876   76.13193       463.18208       512.70242      -382.34388           1
H    66.76618   75.34739   76.37914      1725.29117      1863.52247      -987.07854           1
C    68.81414   75.52599   77.03321       710.42801      -144.27427      -313.15367           1
H    68.56387   75.66009   78.11704       364.20195     -1033.13725      2494.72524           1
H    69.07520   74.44669   76.88322      2567.61904       335.80679       847.48802           1
H    69.74065   76.12290   76.83149     -2587.99029       680.92867      -512.16411           1
C    67.34078   77.42421   76.39135       268.17616       741.64379       -40.42634           1
```

H	67.12697	77.61440	77.47472	1283.54314	-1945.37420	-1411.27812	1
H	68.19123	78.09303	76.09870	-1258.57796	1317.15249	-2026.02085	1
H	66.44101	77.75916	75.81424	-2502.84526	909.80597	575.92391	1
C	66.95551	75.50732	72.24907	619.36578	-458.64843	-172.09273	1
O	66.20626	74.66413	71.72628	-81.40433	433.87611	522.34594	1
N	67.95142	76.16497	71.72572	175.23996	-622.20190	341.55523	1
H	68.44181	76.80282	72.35058	-876.57256	-303.22879	-2561.90213	1
C	68.44205	75.98997	70.38854	-571.88214	-534.10783	-106.15033	1
H	69.28162	75.26918	70.50302	-2713.20841	-16.25800	-248.80809	1
C	67.30732	75.38505	69.43362	166.60354	288.16399	-716.09568	1
H	66.31835	75.81627	69.76244	-2097.85574	1636.78449	-586.18044	1
O	67.15769	73.96974	69.51229	-317.80580	-363.38227	484.43082	1
H	66.52327	73.91471	70.33026	1863.74748	-1451.84923	1357.29467	1
C	67.70255	75.97018	68.02955	503.80731	517.99032	318.51606	1
H	67.01538	75.61335	67.22717	640.61020	-2157.10410	-1536.29104	1
H	67.67689	77.08740	68.02133	1687.88455	465.60692	2087.56452	1
H	68.74051	75.66948	67.74406	-1293.07895	-1899.88581	-1463.57452	1
C	69.00174	77.41443	69.86366	-153.07550	-733.68902	248.70981	1
O	69.76062	77.33735	68.92697	186.15459	-480.78787	-449.34387	1
N	68.45245	78.51598	70.39143	-487.53139	543.18517	42.03850	1
C	69.06570	79.78961	70.14416	-699.35871	69.34393	360.10701	1
H	69.64838	79.60366	69.21018	2.81126	-221.99807	-2715.58072	1
C	67.79024	80.72459	69.98250	-507.46439	-592.59194	122.07761	1
H	68.01549	81.80936	70.10070	-1975.08116	525.87560	-1801.71556	1
H	67.32866	80.60850	68.97686	1588.27743	-1913.22100	1113.83566	1
C	66.85757	80.14842	71.09171	-613.62442	-457.55993	-194.14490	1
H	67.19738	80.45729	72.10624	1707.54637	-721.27992	1996.92523	1
H	65.79518	80.46772	70.99884	758.23357	2100.81767	1560.55011	1
C	67.07035	78.59198	70.85221	-352.41524	704.96076	49.21465	1
H	66.96369	77.97018	71.76906	1714.76886	-2070.42778	443.35792	1
H	66.45061	78.14825	70.03840	1224.15040	1801.50881	1636.97633	1
C	70.04645	80.14588	71.40284	566.85117	537.05831	117.62814	1
O	70.94068	79.38172	71.69814	-550.26779	-406.08867	4.21666	1
N	69.70502	81.28850	71.98966	566.34832	-425.77725	180.18749	1
H	68.94629	81.80852	71.54506	644.44502	2646.80550	-52.73430	1
C	70.77429	82.09589	72.54010	612.34801	-281.48662	411.56221	1
H	71.57034	82.11590	71.76292	1667.23498	-1490.75142	1556.16753	1
C	70.10144	83.51292	72.80324	-714.45746	-175.43074	286.98927	1
H	69.30022	83.39949	73.58420	-787.09484	1763.03619	-1922.46060	1
O	69.45219	84.10614	71.65856	-429.46514	516.51932	-128.40526	1
H	68.54729	84.24198	72.00054	1981.82785	436.16847	-1818.18182	1
C	71.31359	84.30894	73.41351	630.79189	-468.94448	76.03230	1
H	71.01172	85.33537	73.72638	-1775.71861	2062.30353	131.89849	1
H	71.73330	83.79717	74.31091	-1855.14608	88.94118	1993.53760	1
H	72.14320	84.41659	72.67642	-1504.82350	-780.94249	-2132.90992	1
C	71.26354	81.41413	73.92607	288.57638	618.62814	-397.00270	1
O	70.52060	81.13180	74.83033	321.70495	-185.26452	574.37218	1
N	72.61521	81.41796	73.91395	-447.05792	-446.53431	367.76425	1
H	73.06728	81.70227	73.02373	350.31316	1975.63644	-1843.31522	1
C	73.18695	80.29204	74.62833	-511.23061	374.03917	-471.51480	1
H	72.42012	80.03060	75.39520	2234.79579	-898.70890	-1273.45190	1
C	73.36432	79.15257	73.53094	529.53781	-159.34528	563.72604	1
H	73.42199	78.14485	73.99914	654.59388	2347.94251	1217.51488	1
H	72.46144	79.15225	72.84077	-1526.55661	2103.42026	817.87394	1
O	74.30993	82.41244	71.85120	-554.84278	14.12789	399.58769	2
H	74.62929	80.51703	72.34469	1297.06236	2061.84854	1220.68812	1
C	74.60087	80.71579	75.25664	-662.15484	-412.13544	123.62687	1
O	75.02850	80.09199	76.21965	-540.01537	-317.05804	274.91456	1
N	75.19966	81.66858	74.55221	-202.76299	-690.00100	-131.49145	1
H	74.67675	81.97303	73.71421	-702.62132	-2617.37191	281.70413	1
C	76.02683	82.71219	75.17291	-491.73914	576.34187	222.73509	1
H	76.01274	83.52631	74.41053	1977.17978	-1823.58850	-434.68928	1

C	75.37144	83.12060	76.47896	-649.50797	-328.66843	306.11224	1
H	75.22150	82.19600	77.08481	2075.07803	1452.16490	-1004.45904	1
C	76.24949	84.07339	77.26843	-615.18735	-489.99689	-70.96352	1
H	75.77733	84.36449	78.24706	-1226.32661	-1724.38906	-1716.47170	1
H	77.25080	83.61451	77.50074	2381.64273	-969.84735	900.46860	1
H	76.44758	85.02230	76.69726	2122.74991	1702.23946	-141.36228	1
C	74.00586	83.74033	76.24280	-501.86074	-479.61276	-376.42482	1
H	73.49594	84.01055	77.20797	993.79261	-2246.95401	-1177.81305	1
H	74.07797	84.68026	75.62893	42.71279	-626.15391	-2651.37258	1
H	73.32652	83.03359	75.68946	-2026.39012	-1590.33709	-887.82922	1
C	77.57693	82.17898	75.42662	-672.89153	382.57394	-156.34072	1
O	78.49118	82.92290	75.17954	410.04627	221.86223	500.35950	1
N	77.60207	80.98748	75.99295	-378.11782	-443.79414	-441.10984	1
H	76.70283	80.48780	76.18862	2404.15973	-568.14894	1149.30085	1
C	78.87226	80.43098	76.30935	-544.54484	466.16019	331.29119	1
H	79.59424	81.25783	76.10129	-1409.80988	-170.17955	2325.32679	1
C	78.78224	80.02700	77.84657	-184.48284	116.78282	-758.89137	1
H	78.60796	80.92168	78.48532	1893.78641	1861.14652	611.04528	1
H	77.89855	79.37613	78.02743	-2170.79665	-974.19311	1327.50117	1
C	80.11460	79.31392	78.23278	389.51668	672.81878	-138.49152	1
H	80.41725	78.63472	77.40163	510.64577	1933.03327	1851.02484	1
H	80.93967	80.05668	78.30328	-2302.30869	-1105.72939	-948.89774	1
C	80.03903	78.47637	79.57196	-559.93427	-126.34590	542.30857	1
O	81.03334	78.29183	80.22364	-670.36003	-111.83166	76.36448	1
N	78.81258	77.92354	79.81000	-216.06729	-537.07037	446.51379	1
H	78.05720	77.93584	79.11788	-1251.96487	1074.12894	-2168.52500	1
H	78.72510	77.03374	80.32967	-2451.83023	-720.73851	944.84612	1
C	79.18823	79.11241	75.39006	546.24052	-563.88337	-85.11809	1
O	80.35273	78.93497	75.13678	71.70702	-307.40463	606.69673	1
N	78.15905	78.27564	75.17283	479.90863	-504.50129	222.86849	1
C	78.26757	77.30771	74.12391	75.51268	-600.91904	506.73700	1
H	79.32191	77.41468	73.77520	-2033.61562	1795.30103	254.89480	1
C	77.94864	75.96887	74.91181	523.79067	517.99274	284.45581	1
H	77.68321	75.11704	74.24580	1599.61665	-1127.00911	1895.98173	1
H	78.82963	75.63718	75.50561	-1881.83093	1275.82488	-1501.55016	1
C	76.78858	76.42133	75.85582	-600.05639	-404.05026	316.64485	1
H	75.80983	76.45964	75.32840	-2597.05260	496.36221	657.73249	1
H	76.64610	75.74688	76.72994	-1666.24518	1294.38985	-1723.90591	1
C	77.27473	77.87392	76.26497	493.57570	-129.95376	-602.56394	1
H	76.45142	78.63809	76.32976	-1469.08415	-637.84797	2204.22581	1
H	77.90594	77.90665	77.18319	-1038.97840	1068.88701	-2280.71789	1
C	77.17845	77.68030	72.94499	188.88100	241.05089	-727.87821	1
O	77.39564	78.66361	72.27378	531.88613	-49.19943	427.07960	1
N	76.15994	76.84389	72.89662	708.14833	26.93417	-179.73509	1
H	76.15245	76.07794	73.56563	-2232.69501	-1179.09831	1023.94816	1
C	75.09501	76.97148	71.96510	-738.32412	259.92548	104.42456	1
H	74.23596	76.50940	72.49962	2028.89123	1260.02983	1311.33324	1
C	74.88723	78.47506	71.51419	-258.65678	-345.52513	661.28459	1
H	75.78313	78.80689	70.93144	-1826.06090	-1801.46097	-918.64554	1
C	73.61126	78.50442	70.60148	-541.23400	531.92970	-218.41428	1
H	73.34090	79.54417	70.30173	1311.73218	-2219.18200	882.19023	1
H	73.77415	77.91545	69.66519	-1048.71298	1223.58118	-2196.97971	1
H	72.71894	78.07003	71.11436	419.06414	1903.92898	1903.44670	1
C	74.66873	79.45426	72.72281	-423.86431	152.66566	-648.55262	1
H	74.26655	82.10899	70.94358	105.68386	-2342.50080	1387.51238	2
H	75.55338	79.40826	73.40351	-1695.63603	-1484.73621	-1531.02793	1
H	75.15299	82.86807	71.84342	-1853.57121	-487.39031	1936.59343	2
C	75.49880	76.13396	70.60298	539.71428	-510.58232	267.58633	1
O	75.72575	76.72851	69.57175	-15.93758	-278.34085	-624.49402	1
N	75.42385	74.83604	70.83348	-676.81886	12.05292	276.17960	1
H	75.18346	74.55938	71.78467	-1694.44250	-373.84189	-2100.66098	1
C	75.76704	73.86425	69.85375	412.33078	-664.70360	108.35451	1

H	75.19525	72.96977	70.18840	1765.77476	1294.44366	1621.76594	1
C	75.40711	74.39294	68.40807	-179.66635	-721.95667	-264.73915	1
H	74.36785	74.79271	68.37769	-607.34922	2587.87032	-598.10018	1
H	76.07318	75.25241	68.13137	-359.69332	-1479.13954	-2259.74260	1
C	75.59669	73.20649	67.40243	-240.01076	616.02425	431.85322	1
H	76.64549	72.82914	67.44667	-1359.17131	782.54425	2227.99181	1
H	74.94492	72.34564	67.67140	-820.36667	85.81767	-2596.78715	1
C	75.25903	73.73951	65.95327	-513.07015	236.18570	-551.87280	1
H	74.21155	74.11618	65.90157	1816.11944	-1183.11722	-1650.94321	1
H	75.90681	74.60777	65.69091	-1800.04429	-1361.53473	1526.34643	1
C	75.47636	72.57450	64.95933	726.08972	-253.19732	-179.64713	1
H	76.52821	72.23773	64.86609	1224.27271	-456.51798	-2390.90304	1
H	74.81254	71.70149	65.11870	1206.40339	-1150.93514	-2154.90344	1
N	75.10118	73.09819	63.53200	329.70796	-633.68968	-155.67358	1
H	75.19983	72.39419	62.74464	1348.31305	1938.76845	1359.00643	1
H	74.10499	73.43466	63.38662	2096.92035	1446.54551	-966.48890	1
H	75.66589	73.91352	63.15387	-1197.07217	-2303.53719	827.28733	1
C	77.38770	73.59420	69.87714	-378.01186	-556.17301	-413.96481	1
O	78.07441	73.84674	68.91207	-409.44433	-307.55053	-453.30833	1
N	77.74754	73.03439	71.02459	-429.02477	524.70884	274.08502	1
H	77.03506	72.88351	71.73690	1706.34263	-1425.96036	1574.38926	1
C	79.08394	72.54418	71.12305	-45.25864	-626.22952	478.93267	1
H	79.51632	72.70280	70.10471	1988.63164	570.90773	1772.87287	1
C	79.78448	73.42089	72.25049	551.77128	-20.49526	-564.54996	1
H	79.69921	74.50297	72.00497	1434.61535	-1753.10253	1513.99471	1
H	79.28001	73.29883	73.23461	-714.67738	1819.09770	1898.36494	1
C	81.27901	72.97529	72.32779	446.99263	-555.77305	338.97221	1
H	81.35853	71.93343	72.71265	1953.68466	1892.25957	-161.67766	1
H	81.74292	72.96431	71.31594	-403.03785	2688.07032	-188.43591	1
C	82.03586	73.99239	73.27511	567.86262	335.74681	434.04421	1
H	82.00632	75.02473	72.85663	-1080.02275	1568.26081	-1948.78900	1
H	81.54082	74.06050	74.27069	-919.54108	2111.34692	1456.13463	1
C	83.49226	73.48887	73.40484	195.81897	173.33862	-745.11536	1
H	83.59251	72.45449	73.78870	1405.67949	-2301.21089	-390.08113	1
H	84.10473	73.60917	72.48903	-940.37328	-1118.32446	2299.72133	1
N	84.19954	74.37816	74.48174	319.26422	656.36007	-42.02424	1
H	85.21645	74.14573	74.67396	1961.03633	619.91968	1787.09422	1
H	84.25990	75.41819	74.27683	854.14343	-2156.80752	1429.08711	1
H	83.79467	74.37281	75.46303	1237.02905	-1378.50857	-1998.28500	1
C	79.05258	70.97944	71.58846	532.53319	-268.57236	517.55729	1
O	79.65454	70.15493	70.90858	266.16714	-290.73804	-558.88002	1
N	78.42200	70.82935	72.71091	-226.25352	640.49678	-270.32839	1
H	77.97978	71.62560	73.21291	2007.69063	-1035.27229	1523.50231	1
C	78.25914	69.56143	73.36412	-540.76542	-573.33303	49.50000	1
H	77.46801	69.03055	72.78971	-2374.20950	-1036.76023	843.75774	1
C	79.64317	68.77310	73.21037	-77.09082	666.87422	-415.84136	1
H	80.41062	69.56562	72.97694	-985.81834	617.19825	2463.91927	1
O	79.64412	67.89785	72.07962	293.29899	-391.65602	-477.80926	1
H	79.81020	68.58980	71.32977	2528.72714	-681.76262	-751.27122	1
C	80.03369	68.12123	74.58178	252.47363	-605.14473	440.05134	1
H	81.07056	67.71166	74.55312	-2052.65063	1456.49051	-1043.51830	1
H	79.98933	68.85619	75.42064	-2152.98800	-1661.97574	-161.70652	1
H	79.35996	67.27049	74.83772	-473.12726	-1584.27339	2165.61725	1
C	77.72230	70.08282	74.79805	360.36307	488.46713	505.10056	1
O	77.44484	71.27473	74.92344	-200.08817	-528.03552	-385.82850	1
N	77.60077	69.09003	75.65980	-597.54095	-226.44775	355.20479	1
H	77.83523	68.16228	75.30638	2179.23655	-1300.70981	-991.33835	1
C	77.47407	69.26139	77.06071	-687.68921	297.39807	-249.45179	1
H	78.01407	68.39363	77.49980	-349.16256	-2467.37713	-1101.72831	1
C	78.07050	70.67398	77.51615	672.47345	361.54328	-201.62880	1
H	77.53570	71.50735	76.98984	1541.41502	582.18425	2169.97026	1
H	77.88967	70.81676	78.60760	-2534.67717	60.25153	997.72295	1

C	79.57833	70.73008	77.22084	574.26774	-389.43119	-377.02542	1
C	80.48593	69.92328	77.97036	688.82679	-287.33580	-257.96133	1
H	80.11504	69.24740	78.76192	331.14182	1470.38163	2269.80013	1
C	80.07483	71.59818	76.20193	-383.52382	-675.04808	144.24917	1
H	79.38129	72.22608	75.61153	4.09847	1517.59662	2262.86397	1
C	81.85201	70.01464	77.69733	-134.76730	428.45285	-649.50303	1
H	82.55079	69.39884	78.28981	-903.23433	1600.49020	2011.53387	1
C	81.44922	71.65255	75.96122	-335.53281	-615.00040	-364.39058	1
H	81.81598	72.32910	75.17173	1596.45398	-1369.33862	1732.02659	1
C	82.39043	70.87696	76.69824	585.48547	-340.24194	-406.23938	1
O	83.78840	70.93843	76.45890	-419.15649	-173.04408	-511.94141	1
H	83.81336	71.67419	75.80143	-1071.84350	1559.71507	-1960.13012	1
C	75.88231	69.21869	77.42712	291.41878	-183.13091	710.72246	1
O	75.00172	69.66341	76.72745	379.87695	523.47396	-222.23622	1
N	75.77598	68.72018	78.66229	564.30591	373.09380	277.24371	1
H	76.62321	68.39158	79.12450	-120.59931	-2436.07824	-1214.35098	1
C	74.48413	68.26682	79.06914	404.07514	-299.37480	608.84009	1
H	73.79292	68.79615	78.36996	-1071.07882	-841.69025	2359.66467	1
C	74.42715	66.68873	78.90387	-683.12807	68.71770	-390.13096	1
H	74.79975	66.37977	77.90151	2068.88918	1596.58590	770.89603	1
H	75.09169	66.18762	79.64279	1379.86429	2342.49779	-179.85638	1
C	72.94083	66.26401	79.10925	-176.39967	537.49466	550.97268	1
H	72.57616	66.61778	80.10663	-396.28051	1942.22168	1869.33314	1
H	72.28420	66.75303	78.35506	-94.44700	586.00678	-2659.20016	1
C	72.83376	64.67382	79.00549	-291.69381	-718.18079	-150.72929	1
H	73.12306	64.22011	78.02359	-2303.38776	1089.76242	964.62032	1
H	73.34973	64.10367	79.81980	884.16729	2568.80533	-207.74283	1
N	71.44828	64.51388	79.17283	-594.49522	-252.81478	342.29276	1
H	70.92342	65.33868	79.42045	-1735.17426	2084.05705	-263.71605	1
C	70.81923	63.41607	79.04567	595.23177	-271.52487	442.22352	1
N	71.55496	62.27412	78.71251	585.53813	-424.79750	-105.81303	1
H	72.55787	62.17253	78.56294	959.88797	1327.56823	-2177.11905	1
H	71.16230	61.33984	78.59282	1578.48647	16.27378	-2220.76233	1
N	69.44834	63.59534	79.27937	-452.17763	-390.33541	-421.51853	1
H	68.91625	64.42879	79.52475	470.26107	1974.25629	-1817.92087	1
H	68.76813	62.83628	79.22840	1856.67307	-1573.97321	-1224.35416	1
C	74.27589	68.65054	80.64049	-531.47271	-581.45780	-55.06206	1
O	73.59460	67.96792	81.38064	-243.00837	192.73507	609.52485	1
N	74.89974	69.78510	80.93007	343.49288	-644.25607	38.09164	1
H	75.42805	70.24679	80.19143	-1691.16043	-1535.97525	-1484.73099	1
C	74.66016	70.34100	82.22534	542.66492	350.14337	454.42563	1
H	73.63448	69.97966	82.46658	1209.57979	-2396.95920	463.86744	1
C	75.72807	69.69855	83.20940	-737.14309	145.93010	242.71942	1
H	76.76557	69.87121	82.85227	-1050.18303	159.36419	-2509.06125	1
C	75.51199	70.31424	84.63340	-706.77696	-252.88343	-245.16230	1
H	76.18606	69.84782	85.38962	-1624.64039	-1665.49085	1417.86935	1
H	75.71678	71.41028	84.63885	2566.10521	598.51109	693.22305	1
H	74.46419	70.16908	84.98710	-1972.69758	107.10018	-1876.34312	1
C	75.43350	68.15976	83.29697	412.85148	672.58832	-27.69222	1
H	76.05076	67.66277	84.08226	1447.02277	1762.76533	-1490.78944	1
H	74.35972	67.96150	83.53419	1320.77379	-145.20455	2378.68491	1
H	75.64965	67.64841	82.32763	-1512.10848	1470.52031	1724.75147	1
C	74.77489	71.96941	82.07462	-607.11113	499.56689	73.73769	1
O	73.75729	72.61098	81.94580	357.97126	460.81534	356.68785	1
N	76.02868	72.40299	82.09357	536.55407	-368.62270	332.76306	1
H	76.80186	71.74624	82.18840	524.38866	2297.69985	1367.20941	1
C	76.25709	73.79079	81.89508	46.50277	-774.29190	-147.97904	1
H	75.31243	74.17429	81.44496	1676.51875	1883.29066	1032.55549	1
C	76.58012	74.49077	83.32175	-559.41456	-34.19198	-556.30433	1
H	77.44635	73.97583	83.80006	-64.66664	395.18769	2695.05373	1
H	76.90220	75.54774	83.16869	-2703.44278	-60.40056	333.79418	1
C	75.37496	74.43270	84.21514	-519.76435	377.31045	459.42366	1

```
H    75.05619  73.37037  84.31067    -1746.74323     1667.50644    -1261.73691          1
C    75.71874  74.96771  85.60075      570.95554       16.46261     -545.27700          1
H    74.83820  74.92276  86.29185     2391.58273       28.05277    -1305.07271          1
H    76.54633  74.38242  86.07771      175.75588      917.63458     2559.43867          1
H    76.05145  76.03663  85.55793    -1063.97851    -2155.34650     1283.00462          1
C    74.20939  75.22600  83.63868      193.76014        2.62946     -765.53141          1
H    73.35521  75.28803  84.36101      507.39394     -846.55087     2539.60089          1
H    74.50665  76.27574  83.38329     1337.57841     1818.19054     1526.01994          1
H    73.81215  74.74824  82.70305     1100.88635    -1172.91350    -2199.08909          1
C    77.56235  73.96490  80.98040     -549.21115      475.64486     -309.38218          1
O    78.27330  73.06469  80.62703      556.06106     -390.37755      -78.24250          1
O    77.88085  75.32379  80.64253     -572.27940     -310.19032     -209.75862          1
H    77.07296  75.75121  81.03340    -1380.08193     2212.14486      790.85944          1
```

Listing 52: C00052.xmolout

```
       276
  150.0000  150.0000  150.0000     0.0000     0.0000     0.0000***********    2.7750    11100
       0.2500     300.00   0.00   0.00   0.00  -1293.6794       0.0000    300.0000
H    77.25715  83.10635  78.88329    -2043.14101    -1757.64893      399.89339          1
N    78.26404  82.96360  78.86084      426.75047      490.45733     -334.42467          1
H    78.35030  82.14461  78.26200      442.25559    -1110.30854    -2448.52907          1
C    79.03086  84.06656  78.41456      415.32972     -666.39213       83.73172          1
H    78.52172  84.99785  78.75252      879.32219     2491.00447      667.35213          1
C    80.52273  83.88592  79.03341       31.35357      477.41777      628.23362          1
H    80.94988  82.92364  78.66483     1168.38489     2240.25337    -1019.71136          1
H    81.20029  84.68814  78.65725     -360.43770     2534.90498      931.67112          1
C    80.45509  83.89149  80.53269      463.18208      512.70242     -382.34388          1
H    79.61299  83.23203  80.84458     1725.29117     1863.52247     -987.07854          1
C    81.74710  83.33857  81.12372      710.42801     -144.27427     -313.15367          1
H    81.71478  83.31752  82.24333      364.20195    -1033.13725     2494.72524          1
H    81.94542  82.29169  80.77692     2567.61904      335.80679      847.48802          1
H    82.63278  83.95812  80.82899    -2587.99029      680.92867     -512.16411          1
C    80.20877  85.29394  81.07400      268.17616      741.64379      -40.42634          1
H    80.16592  85.30252  82.19363     1283.54314    -1945.37420    -1411.27812          1
H    81.02157  86.00204  80.76849    -1258.57796     1317.15249    -2026.02085          1
H    79.24136  85.71858  80.70188    -2502.84526      909.80597      575.92391          1
C    79.16631  83.91840  76.83373      619.36578     -458.64843     -172.09273          1
O    79.23645  82.80007  76.20587      -81.40433      433.87611      522.34594          1
N    79.30665  85.07515  76.25249      175.23996     -622.20190      341.55523          1
H    79.21058  85.92549  76.80370     -876.57256     -303.22879    -2561.90213          1
C    79.08958  85.00110  74.83449     -571.88214     -534.10783     -106.15033          1
H    78.00090  85.17175  74.73045    -2713.20841      -16.25800     -248.80809          1
C    79.93202  86.15489  74.17748      166.60354      288.16399     -716.09568          1
H    81.00199  86.06071  74.51138    -2097.85574     1636.78449     -586.18044          1
O    79.53490  87.50500  74.52770     -317.80580     -363.38227      484.43082          1
H    80.34975  87.84873  74.93614     1863.74748    -1451.84923     1357.29467          1
C    79.82018  85.75882  72.65650      503.80731      517.99032      318.51606          1
H    80.44164  86.42425  72.01339      640.61020    -2157.10410    -1536.29104          1
H    80.16405  84.70785  72.48345     1687.88455      465.60692     2087.56452          1
H    78.77034  85.83028  72.28857    -1293.07895    -1899.88581    -1463.57452          1
C    79.59178  83.44634  74.38047     -153.07550     -733.68902      248.70981          1
O    80.79547  83.27002  74.14622      186.15459     -480.78787     -449.34387          1
N    78.59008  82.87855  73.60650     -487.53139      543.18517       42.03850          1
C    78.78882  81.62308  73.00367     -699.35871       69.34393      360.10701          1
H    79.65606  81.13232  73.50613        2.81126     -221.99807    -2715.58072          1
C    77.35328  80.93123  73.18518     -507.46439     -592.59194      122.07761          1
H    77.19119  80.07962  72.48793    -1975.08116      525.87560    -1801.71556          1
H    77.21227  80.53803  74.21616     1588.27743    -1913.22100     1113.83566          1
C    76.42898  82.14712  72.90237     -613.62442     -457.55993     -194.14490          1
H    76.38379  82.36255  71.80859     1707.54637     -721.27992     1996.92523          1
H    75.38210  82.00836  73.25271      758.23357     2100.81767     1560.55011          1
```

C	77.21225	83.30678	73.67925	-352.41524	704.96076	49.21465	1
H	77.13528	84.31620	73.20710	1714.76886	-2070.42778	443.35792	1
H	76.97476	83.38764	74.76831	1224.15040	1801.50881	1636.97633	1
C	78.99928	81.83040	71.37130	566.85117	537.05831	117.62814	1
O	78.38628	82.66091	70.74746	-550.26779	-406.08867	4.21666	1
N	79.80821	80.87878	70.93534	566.34832	-425.77725	180.18749	1
H	80.22989	80.25240	71.61914	644.44502	2646.80550	-52.73430	1
C	80.12056	80.84140	69.54439	612.34801	-281.48662	411.56221	1
H	80.11508	81.90524	69.21483	1667.23498	-1490.75142	1556.16753	1
C	81.52882	80.10172	69.41588	-714.45746	-175.43074	286.98927	1
H	81.43279	79.07384	69.86170	-787.09484	1763.03619	-1922.46060	1
O	82.61955	80.73918	70.11022	-429.46514	516.51932	-128.40526	1
H	82.85532	80.05548	70.76643	1981.82785	436.16847	-1818.18182	1
C	81.70953	79.96854	67.85991	630.79189	-468.94448	76.03230	1
H	82.63984	79.40900	67.60601	-1775.71861	2062.30353	131.89849	1
H	80.85704	79.42593	67.38562	-1855.14608	88.94118	1993.53760	1
H	81.77957	80.96818	67.37136	-1504.82350	-780.94249	-2132.90992	1
C	78.94952	79.98761	68.78259	288.57638	618.62814	-397.00270	1
O	78.71566	80.19113	67.61350	321.70495	-185.26452	574.37218	1
N	78.40956	79.07123	69.58530	-447.05792	-446.53431	367.76425	1
H	78.80893	78.99398	70.51896	350.31316	1975.63644	-1843.31522	1
C	77.68916	78.00005	68.95732	-511.23061	374.03917	-471.51480	1
H	76.92895	78.53436	68.34478	2234.79579	-898.70890	-1273.45190	1
C	78.72883	77.17623	68.07429	529.53781	-159.34528	563.72604	1
H	78.12057	76.48124	67.43396	654.59388	2347.94251	1217.51488	1
H	79.24335	77.87088	67.35929	-1526.55661	2103.42026	817.87394	1
O	79.69023	76.41416	68.82388	-554.84278	14.12789	399.58769	1
H	79.05759	76.02240	69.53468	1297.06236	2061.84854	1220.68812	1
C	77.10498	77.04516	70.13037	-662.15484	-412.13544	123.62687	1
O	77.68101	76.00179	70.44839	-540.01537	-317.05804	274.91456	1
N	76.01920	77.57553	70.64371	-202.76299	-690.00100	-131.49145	1
H	75.69751	78.47712	70.29275	-702.62132	-2617.37191	281.70413	1
C	75.35753	76.86429	71.68437	-491.73914	576.34187	222.73509	1
H	75.66626	75.80784	71.50702	1977.17978	-1823.58850	-434.68928	1
C	75.91786	77.40574	73.07329	-649.50797	-328.66843	306.11224	1
H	75.72367	78.49029	73.21378	2075.07803	1452.16490	-1004.45904	1
C	75.19231	76.56443	74.17546	-615.18735	-489.99689	-70.96352	1
H	75.68817	76.66702	75.17473	-1226.32661	-1724.38906	-1716.47170	1
H	74.12255	76.88415	74.28963	2381.64273	-969.84735	900.46860	1
H	75.18902	75.47867	73.91960	2122.74991	1702.23946	-141.36228	1
C	77.45760	77.12224	73.13000	-501.86074	-479.61276	-376.42482	1
H	77.85722	77.18706	74.16911	993.79261	-2246.95401	-1177.81305	1
H	77.69791	76.10575	72.73052	42.71279	-626.15391	-2651.37258	1
H	78.02522	77.85162	72.50595	-2026.39012	-1590.33709	-887.82922	1
C	73.74464	77.13853	71.52088	-672.89153	382.57394	-156.34072	1
O	73.04654	76.24041	71.11029	410.04627	221.86223	500.35950	1
N	73.41581	78.39765	71.78244	-378.11782	-443.79414	-441.10984	1
H	74.15841	79.00788	72.11889	2404.15973	-568.14894	1149.30085	1
C	72.03472	78.73385	71.97486	-544.54484	466.16019	331.29119	1
H	72.08644	79.84540	71.99236	-1409.80988	-170.17955	2325.32679	1
C	71.32007	78.17346	70.66932	-184.48284	116.78282	-758.89137	1
H	72.09477	77.86758	69.92767	1893.78641	1861.14652	611.04528	1
H	70.72869	77.25407	70.89124	-2170.79665	-974.19311	1327.50117	1
C	70.43710	79.29344	70.03497	389.51668	672.81878	-138.49152	1
H	69.55824	79.52951	70.67143	510.64577	1933.03327	1851.02484	1
H	71.00373	80.24986	70.00113	-2302.30869	-1105.72939	-948.89774	1
C	69.94545	78.94130	68.58072	-559.93427	-126.34590	542.30857	1
O	69.26089	79.73102	67.98244	-670.36003	-111.83166	76.36448	1
N	70.36861	77.74361	68.12133	-216.06729	-537.07037	446.51379	1
H	70.92724	77.01714	68.58451	-1251.96487	1074.12894	-2168.52500	1
H	70.13382	77.37328	67.19374	-2451.83023	-720.73851	944.84612	1
C	71.63211	78.16959	73.43640	546.24052	-563.88337	-85.11809	1

O	72.46909	78.24237	74.30538	71.70702	-307.40463	606.69673	1
N	70.34028	77.83075	73.58595	479.90863	-504.50129	222.86849	1
C	69.90549	77.34125	74.85760	75.51268	-600.91904	506.73700	1
H	70.68623	77.63309	75.59987	-2033.61562	1795.30103	254.89480	1
C	68.49297	78.06006	74.99772	523.79067	517.99274	284.45581	1
H	67.83188	77.57139	75.74762	1599.61665	-1127.00911	1895.98173	1
H	68.60099	79.12280	75.30875	-1881.83093	1275.82488	-1501.55016	1
C	67.95741	77.94680	73.53807	-600.05639	-404.05026	316.64485	1
H	67.61707	76.90763	73.31792	-2597.05260	496.36221	657.73249	1
H	67.09994	78.62224	73.31724	-1666.24518	1294.38985	-1723.90591	1
C	69.26901	78.30456	72.71720	493.57570	-129.95376	-602.56394	1
H	69.35569	77.77489	71.73923	-1469.08415	-637.84797	2204.22581	1
H	69.45436	79.39542	72.57716	-1038.97840	1068.88701	-2280.71789	1
C	69.58524	75.73394	74.82130	188.88100	241.05089	-727.87821	1
O	69.00787	75.19290	73.91080	531.88613	-49.19943	427.07960	1
N	69.82375	75.20601	76.06513	708.14833	26.93417	-179.73509	1
H	70.36058	75.95184	76.51994	-2232.69501	-1179.09831	1023.94816	1
C	70.34204	73.81792	76.15543	-738.32412	259.92548	104.42456	1
H	70.56861	73.79716	77.24494	2028.89123	1260.02983	1311.33324	1
C	69.28568	72.81905	75.75435	-258.65678	-345.52513	661.28459	1
H	69.30133	72.76060	74.64085	-1826.06090	-1801.46097	-918.64554	1
C	69.54063	71.42961	76.31407	-541.23400	531.92970	-218.41428	1
H	69.03733	70.63585	75.69844	1311.73218	-2219.18200	882.19023	1
H	70.63776	71.17857	76.35271	-1048.71298	1223.58118	-2196.97971	1
H	69.14720	71.30951	77.36249	419.06414	1903.92898	1903.44670	1
C	67.88750	73.26354	76.15467	-423.86431	152.66566	-648.55262	1
H	67.10295	72.53255	75.81972	105.68386	-2342.50080	1387.51238	1
H	67.79046	73.37346	77.26805	-1695.63603	-1484.73621	-1531.02793	1
H	67.63980	74.26091	75.69708	-1853.57121	-487.39031	1936.59343	1
C	71.72897	73.82390	75.26960	539.71428	-510.58232	267.58633	1
O	72.45075	74.80743	75.30926	-15.93758	-278.34085	-624.49402	1
N	71.96002	72.66520	74.70720	-676.81886	12.05292	276.17960	1
H	71.22285	71.97396	74.79859	-1694.44250	-373.84189	-2100.66098	1
C	73.07130	72.38768	73.83903	412.33078	-664.70360	108.35451	1
H	72.60699	72.33503	72.82975	1765.77476	1294.44366	1621.76594	1
C	74.17952	73.49644	73.95868	-179.66635	-721.95667	-264.73915	1
H	73.80132	74.47434	73.57023	-607.34922	2587.87032	-598.10018	1
H	74.41681	73.67192	75.03276	-359.69332	-1479.13954	-2259.74260	1
C	75.44407	73.02419	73.16023	-240.01076	616.02425	431.85322	1
H	75.48763	71.91294	73.11204	-1359.17131	782.54425	2227.99181	1
H	75.40467	73.37574	72.10504	-820.36667	85.81767	-2596.78715	1
C	76.72026	73.58039	73.90806	-513.07015	236.18570	-551.87280	1
H	76.67566	74.68893	74.01823	1816.11944	-1183.11722	-1650.94321	1
H	76.77617	73.17605	74.94535	-1800.04429	-1361.53473	1526.34643	1
C	77.96231	73.16729	73.08407	726.08972	-253.19732	-179.64713	1
H	78.11916	72.07299	73.00190	1224.27271	-456.51798	-2390.90304	1
H	78.03004	73.63822	72.08224	1206.40339	-1150.93514	-2154.90344	1
N	79.21945	73.69378	73.85298	329.70796	-633.68968	-155.67358	1
H	80.15223	73.46829	73.40143	1348.31305	1938.76845	1359.00643	1
H	79.30164	74.74394	73.98398	2096.92035	1446.54551	-966.48890	1
H	79.37171	73.33299	74.83991	-1197.07217	-2303.53719	827.28733	1
C	73.62785	70.92650	74.33803	-378.01186	-556.17301	-413.96481	1
O	74.28813	70.75602	75.36643	-409.44433	-307.55053	-453.30833	1
N	73.22270	70.08964	73.42334	-429.02477	524.70884	274.08502	1
H	72.67242	70.50791	72.67339	1706.34263	-1425.96036	1574.38926	1
C	73.35279	68.67491	73.45650	-45.25864	-626.22952	478.93267	1
H	72.50642	68.36269	72.80513	1988.63164	570.90773	1772.87287	1
C	74.73555	68.31055	72.77727	551.77128	-20.49526	-564.54996	1
H	74.83620	68.81915	71.79263	1434.61535	-1753.10253	1513.99471	1
H	75.59235	68.67794	73.38274	-714.67738	1819.09770	1898.36494	1
C	74.77070	66.75447	72.60963	446.99263	-555.77305	338.97221	1
H	74.68324	66.24737	73.59804	1953.68466	1892.25957	-161.67766	1

H	73.90053	66.39990	72.01348	-403.03785	2688.07032	-188.43591	1
C	76.12783	66.37041	71.89474	567.86262	335.74681	434.04421	1
H	76.16656	66.79178	70.86394	-1080.02275	1568.26081	-1948.78900	1
H	76.99616	66.81185	72.43229	-919.54108	2111.34692	1456.13463	1
C	76.23059	64.82763	71.86437	195.81897	173.33862	-745.11536	1
H	76.31883	64.35147	72.86114	1405.67949	-2301.21089	-390.08113	1
H	75.43947	64.32385	71.27394	-940.37328	-1118.32446	2299.72133	1
N	77.56439	64.45605	71.13191	319.26422	656.36007	-42.02424	1
H	77.76578	63.41785	71.04904	1961.03633	619.91968	1787.09422	1
H	77.66334	64.76642	70.12159	854.14343	-2156.80752	1429.08711	1
H	78.46921	64.80900	71.56032	1237.02905	-1378.50857	-1998.28500	1
C	73.16211	68.15289	75.00195	532.53319	-268.57236	517.55729	1
O	72.07977	68.21150	75.53703	266.16714	-290.73804	-558.88002	1
N	74.30857	67.69709	75.48591	-226.25352	640.49678	-270.32839	1
H	75.15462	67.74431	74.92136	2007.69063	-1035.27229	1523.50231	1
C	74.32818	67.10910	76.77934	-540.76542	-573.33303	49.50000	1
H	73.27143	67.20225	77.12866	-2374.20950	-1036.76023	843.75774	1
C	74.80328	65.58162	76.59901	-77.09082	666.87422	-415.84136	1
H	75.91300	65.56845	76.41877	-985.81834	617.19825	2463.91927	1
O	74.21139	64.86396	75.50620	293.29899	-391.65602	-477.80926	1
H	75.01003	64.58198	75.01345	2528.72714	-681.76262	-751.27122	1
C	74.50473	64.99530	78.02609	252.47363	-605.14473	440.05134	1
H	74.79495	63.92089	78.09259	-2052.65063	1456.49051	-1043.51830	1
H	75.07051	65.54472	78.82472	-2152.98800	-1661.97574	-161.70652	1
H	73.41966	65.05934	78.27586	-473.12726	-1584.27339	2165.61725	1
C	75.40680	67.94361	77.66441	360.36307	488.46713	505.10056	1
O	75.95744	67.39519	78.59826	-200.08817	-528.03552	-385.82850	1
N	75.49659	69.22005	77.30362	-597.54095	-226.44775	355.20479	1
H	74.97735	69.60935	76.47579	2179.23655	-1300.70981	-991.33835	1
C	76.51506	69.95627	77.96721	-687.68921	297.39807	-249.45179	1
H	76.97342	69.24027	78.69543	-349.16256	-2467.37713	-1101.72831	1
C	77.54631	70.50229	76.85980	672.47345	361.54328	-201.62880	1
H	77.00755	71.01823	76.03264	1541.41502	582.18425	2169.97026	1
H	78.23902	71.24288	77.32115	-2534.67717	60.25153	997.72295	1
C	78.31445	69.27822	76.35020	574.26774	-389.43119	-377.02542	1
C	79.28652	68.66394	77.19769	688.82679	-287.33580	-257.96133	1
H	79.51881	69.08810	78.19154	331.14182	1470.38163	2269.80013	1
C	78.00974	68.72241	75.07105	-383.52382	-675.04808	144.24917	1
H	77.25732	69.19851	74.41787	4.09847	1517.59662	2262.86397	1
C	79.92159	67.50610	76.75255	-134.76730	428.45285	-649.50303	1
H	80.66740	67.02569	77.41009	-903.23433	1600.49020	2011.53387	1
C	78.67700	67.56294	74.67032	-335.53281	-615.00040	-364.39058	1
H	78.44135	67.13791	73.68041	1596.45398	-1369.33862	1732.02659	1
C	79.64652	66.91112	75.48671	585.48547	-340.24194	-406.23938	1
O	80.32094	65.72908	75.08521	-419.15649	-173.04408	-511.94141	1
H	79.91608	65.60172	74.19446	-1071.84350	1559.71507	-1960.13012	1
C	75.89225	71.28227	78.68442	291.41878	-183.13091	710.72246	1
O	76.38600	71.66885	79.72047	379.87695	523.47396	-222.23622	1
N	74.93235	71.86498	77.97166	564.30591	373.09380	277.24371	1
H	74.59785	71.47694	77.06944	-120.59931	-2436.07824	-1214.35098	1
C	74.29685	72.96623	78.62720	404.07514	-299.37480	608.84009	1
H	74.83960	73.12175	79.59370	-1071.07882	-841.69025	2359.66467	1
C	74.41799	74.17061	77.59954	-683.12807	68.71770	-390.13096	1
H	75.45816	74.24432	77.20876	2068.88918	1596.58590	770.89603	1
H	73.77405	73.95649	76.71277	1379.86429	2342.49779	-179.85638	1
C	73.98656	75.50930	78.26797	-176.39967	537.49466	550.97268	1
H	72.96939	75.43072	78.71161	-396.28051	1942.22168	1869.33314	1
H	74.66402	75.79128	79.10417	-94.44700	586.00678	-2659.20016	1
C	74.03286	76.58605	77.08137	-291.69381	-718.18079	-150.72929	1
H	75.04622	76.77045	76.63463	-2303.38776	1089.76242	964.62032	1
H	73.29444	76.33585	76.25789	884.16729	2568.80533	-207.74283	1
N	73.58801	77.75546	77.69812	-594.49522	-252.81478	342.29276	1

```
H    72.83529   77.64045   78.36085     -1735.17426      2084.05705      -263.71605          1
C    74.01365   78.93281   77.46521       595.23177      -271.52487       442.22352          1
N    75.03809   79.12443   76.53356       585.53813      -424.79750      -105.81303          1
H    75.49041   78.45267   75.91685       959.88797      1327.56823     -2177.11905          1
H    75.41033   80.03263   76.25373      1578.48647        16.27378     -2220.76233          1
N    73.31987   79.86289   78.24938      -452.17763      -390.33541      -421.51853          1
H    72.56955   79.74117   78.92791       470.26107      1974.25629     -1817.92087          1
H    73.50105   80.86664   78.21020      1856.67307     -1573.97321     -1224.35416          1
C    72.71367   72.58733   78.77642      -531.47271      -581.45780       -55.06206          1
O    72.06955   72.18305   77.80914      -243.00837       192.73507       609.52485          1
N    72.29173   72.85902   79.98879       343.49288      -644.25607        38.09164          1
H    73.02704   73.19638   80.61100     -1691.16043     -1535.97525     -1484.73099          1
C    71.02373   72.41751   80.54307       542.66492       350.14337       454.42563          1
H    70.98265   72.90171   81.54801      1209.57979     -2396.95920       463.86744          1
C    69.88595   72.82841   79.61819      -737.14309       145.93010       242.71942          1
H    70.14407   72.45743   78.59598     -1050.18303       159.36419     -2509.06125          1
C    68.57194   72.19693   80.03531      -706.77696      -252.88343      -245.16230          1
H    67.73477   72.46930   79.33521     -1624.64039     -1665.49085      1417.86935          1
H    68.63993   71.07080   80.04921      2566.10521       598.51109       693.22305          1
H    68.26366   72.52257   81.06736     -1972.69758       107.10018     -1876.34312          1
C    69.73917   74.33589   79.53503       412.85148       672.58832       -27.69222          1
H    68.97229   74.63953   78.76960      1447.02277      1762.76533     -1490.78944          1
H    69.41994   74.77533   80.52007      1320.77379      -145.20455      2378.68491          1
H    70.71000   74.82726   79.24976     -1512.10848      1470.52031      1724.75147          1
C    71.04104   70.74923   80.67798      -607.11113       499.56689        73.73769          1
O    70.79403   70.27401   81.75406       357.97126       460.81534       356.68785          1
N    71.34482   70.17146   79.53685       536.55407      -368.62270       332.76306          1
H    71.50709   70.76779   78.69738       524.38866      2297.69985      1367.20941          1
C    71.23183   68.76800   79.34721        46.50277      -774.29190      -147.97904          1
H    71.91664   68.58614   78.48891      1676.51875      1883.29066      1032.55549          1
C    71.64522   67.95373   80.67654      -559.41456       -34.19198      -556.30433          1
H    70.84040   68.08628   81.44335       -64.66664       395.18769      2695.05373          1
H    71.69432   66.85735   80.47173     -2703.44278       -60.40056       333.79418          1
C    72.95464   68.45560   81.22235      -519.76435       377.31045       459.42366          1
H    72.91026   69.56947   81.25683     -1746.74323      1667.50644     -1261.73691          1
C    73.17482   67.94679   82.64153       570.95554        16.46261      -545.27700          1
H    74.14568   68.31266   83.06548      2391.58273        28.05277     -1305.07271          1
H    72.36078   68.29151   83.33084       175.75588       917.63458      2559.43867          1
H    73.19560   66.82732   82.68242     -1063.97851     -2155.34650      1283.00462          1
C    74.13216   68.04088   80.34834       193.76014         2.62946      -765.53141          1
H    75.10945   68.40420   80.76245       507.39394      -846.55087      2539.60089          1
H    74.21205   66.92629   80.25593      1337.57841      1818.19054      1526.01994          1
H    74.05476   68.45068   79.30430      1100.88635     -1172.91350     -2199.08909          1
C    69.70718   68.49469   78.92506      -549.21115       475.64486      -309.38218          1
O    68.95015   69.36025   78.56378       556.06106      -390.37755       -78.24250          1
O    69.29789   67.13848   78.90698      -572.27940      -310.19032      -209.75862          1
H    70.17100   66.71169   79.12145     -1380.08193      2212.14486       790.85944          1
```

Listing 53: C00053.xmolout

```
       276
   150.0000  150.0000  150.0000      0.0000      0.0000      0.0000***********     1.4410      5764
         0.2500     300.00    0.00    0.00    0.00   -1293.6730        0.0000     300.0000
H    80.89357   79.22160   85.63356     -2043.14101     -1757.64893       399.89339          1
N    81.43146   79.31452   84.77479       426.75047       490.45733      -334.42467          1
H    80.71450   79.60539   84.11376       442.25559     -1110.30854     -2448.52907          1
C    82.53701   80.19070   84.86681       415.32972      -666.39213        83.73172          1
H    82.93187   80.03662   85.89339       879.32219      2491.00447       667.35213          1
C    83.58697   79.77982   83.71308        31.35357       477.41777       628.23362          1
H    83.07093   79.73479   82.72427      1168.38489      2240.25337     -1019.71136          1
H    84.38357   80.55599   83.61411      -360.43770      2534.90498       931.67112          1
C    84.18105   78.43766   84.05211       463.18208       512.70242      -382.34388          1
```

H	83.35283	77.75753	84.35494	1725.29117	1863.52247	-987.07854	1
C	84.87359	77.84177	82.83267	710.42801	-144.27427	-313.15367	1
H	85.31309	76.83556	83.05647	364.20195	-1033.13725	2494.72524	1
H	84.16330	77.71110	81.97569	2567.61904	335.80679	847.48802	1
H	85.70960	78.49585	82.47355	-2587.99029	680.92867	-512.16411	1
C	85.17651	78.54373	85.19929	268.17616	741.64379	-40.42634	1
H	85.63507	77.55024	85.44142	1283.54314	-1945.37420	-1411.27812	1
H	86.01575	79.24412	84.95213	-1258.57796	1317.15249	-2026.02085	1
H	84.69451	78.92306	86.13669	-2502.84526	909.80597	575.92391	1
C	81.97767	81.69878	84.64661	619.36578	-458.64843	-172.09273	1
O	81.89481	82.47794	85.59268	-81.40433	433.87611	522.34594	1
N	81.65100	81.89942	83.39484	175.23996	-622.20190	341.55523	1
H	81.76290	81.19518	82.64732	-876.57256	-303.22879	-2561.90213	1
C	80.96381	83.10073	83.01343	-571.88214	-534.10783	-106.15033	1
H	80.23308	83.36010	83.81364	-2713.20841	-16.25800	-248.80809	1
C	82.11209	84.20371	82.87169	166.60354	288.16399	-716.09568	1
H	82.92610	83.80399	82.19942	-2097.85574	1636.78449	-586.18044	1
O	82.86880	84.49931	84.06470	-317.80580	-363.38227	484.43082	1
H	82.49434	83.80251	84.69441	1863.74748	-1451.84923	1357.29467	1
C	81.42370	85.42062	82.16916	503.80731	517.99032	318.51606	1
H	82.17472	86.19025	81.86313	640.61020	-2157.10410	-1536.29104	1
H	80.86443	85.11278	81.24991	1687.88455	465.60692	2087.56452	1
H	80.70083	85.92879	82.85005	-1293.07895	-1899.88581	-1463.57452	1
C	80.34160	82.59985	81.60670	-153.07550	-733.68902	248.70981	1
O	80.94238	81.70444	81.03489	186.15459	-480.78787	-449.34387	1
N	79.23811	83.24285	81.24262	-487.53139	543.18517	42.03850	1
C	78.53538	82.92160	80.02516	-699.35871	69.34393	360.10701	1
H	78.55330	81.80868	79.96935	2.81126	-221.99807	-2715.58072	1
C	77.10249	83.56628	80.23699	-507.46439	-592.59194	122.07761	1
H	76.63977	83.88262	79.27326	-1975.08116	525.87560	-1801.71556	1
H	76.39553	82.84825	80.70963	1588.27743	-1913.22100	1113.83566	1
C	77.41682	84.76281	81.18895	-613.62442	-457.55993	-194.14490	1
H	77.85911	85.61660	80.62871	1707.54637	-721.27992	1996.92523	1
H	76.52366	85.15734	81.72366	758.23357	2100.81767	1560.55011	1
C	78.48283	84.09091	82.15758	-352.41524	704.96076	49.21465	1
H	79.17455	84.79785	82.66728	1714.76886	-2070.42778	443.35792	1
H	78.04467	83.40085	82.91720	1224.15040	1801.50881	1636.97633	1
C	79.21360	83.59922	78.71565	566.85117	537.05831	117.62814	1
O	78.49166	83.97750	77.79413	-550.26779	-406.08867	4.21666	1
N	80.52566	83.58913	78.77830	566.34832	-425.77725	180.18749	1
H	80.95655	83.17709	79.61266	644.44502	2646.80550	-52.73430	1
C	81.25301	84.39215	77.84348	612.34801	-281.48662	411.56221	1
H	80.55482	85.24401	77.68042	1667.23498	-1490.75142	1556.16753	1
C	82.65677	84.77932	78.48076	-714.45746	-175.43074	286.98927	1
H	83.28674	83.85223	78.56436	-787.09484	1763.03619	-1922.46060	1
O	82.61278	85.34640	79.81127	-429.46514	516.51932	-128.40526	1
H	83.21666	84.74297	80.28631	1981.82785	436.16847	-1818.18182	1
C	83.26287	85.71710	77.37264	630.79189	-468.94448	76.03230	1
H	84.29916	86.03481	77.63320	-1775.71861	2062.30353	131.89849	1
H	83.31296	85.21025	76.37901	-1855.14608	88.94118	1993.53760	1
H	82.65659	86.64354	77.24426	-1504.82350	-780.94249	-2132.90992	1
C	81.44621	83.50561	76.47655	288.57638	618.62814	-397.00270	1
O	82.52440	83.09164	76.13015	321.70495	-185.26452	574.37218	1
N	80.26666	83.39787	75.87550	-447.05792	-446.53431	367.76425	1
H	79.40996	83.78810	76.34022	350.31316	1975.63644	-1843.31522	1
C	80.18879	82.48781	74.77511	-511.23061	374.03917	-471.51480	1
H	80.99454	82.82318	74.08654	2234.79579	-898.70890	-1273.45190	1
C	80.50857	81.02519	75.34498	529.53781	-159.34528	563.72604	1
H	80.58878	80.34462	74.45391	654.59388	2347.94251	1217.51488	1
H	81.52848	81.06996	75.81558	-1526.55661	2103.42026	817.87394	1
O	79.54427	80.45249	76.26457	-554.84278	14.12789	399.58769	1
H	79.83986	80.88045	77.09134	1297.06236	2061.84854	1220.68812	1

C	78.69666	82.53218	74.13163	-662.15484	-412.13544	123.62687	1
O	78.59694	82.41567	72.93083	-540.01537	-317.05804	274.91456	1
N	77.72949	82.56029	75.04868	-202.76299	-690.00100	-131.49145	1
H	78.08047	82.61009	76.00921	-702.62132	-2617.37191	281.70413	1
C	76.33442	82.91240	74.79567	-491.73914	576.34187	222.73509	1
H	76.18789	83.82969	75.41287	1977.17978	-1823.58850	-434.68928	1
C	76.01719	83.11293	73.33238	-649.50797	-328.66843	306.11224	1
H	76.52111	82.30532	72.74291	2075.07803	1452.16490	-1004.45904	1
C	74.52340	83.02239	73.07213	-615.18735	-489.99689	-70.96352	1
H	74.28156	83.15156	71.98188	-1226.32661	-1724.38906	-1716.47170	1
H	74.10419	82.02597	73.39195	2381.64273	-969.84735	900.46860	1
H	73.95690	83.81312	73.63594	2122.74991	1702.23946	-141.36228	1
C	76.54287	84.44437	72.82386	-501.86074	-479.61276	-376.42482	1
H	76.30288	84.59953	71.73654	993.79261	-2246.95401	-1177.81305	1
H	76.10422	85.31068	73.39084	42.71279	-626.15391	-2651.37258	1
H	77.66288	84.50237	72.93031	-2026.39012	-1590.33709	-887.82922	1
C	75.44100	81.65359	75.45045	-672.89153	382.57394	-156.34072	1
O	74.70617	81.92147	76.37620	410.04627	221.86223	500.35950	1
N	75.64419	80.51222	74.82705	-378.11782	-443.79414	-441.10984	1
H	76.38140	80.58765	74.12928	2404.15973	-568.14894	1149.30085	1
C	75.00029	79.27729	75.13198	-544.54484	466.16019	331.29119	1
H	75.79809	78.51558	75.00119	-1409.80988	-170.17955	2325.32679	1
C	74.45545	79.40909	76.60749	-184.48284	116.78282	-758.89137	1
H	75.24834	79.87525	77.23940	1893.78641	1861.14652	611.04528	1
H	73.59706	80.12889	76.61546	-2170.79665	-974.19311	1327.50117	1
C	74.00003	78.05249	77.24089	389.51668	672.81878	-138.49152	1
H	73.17411	77.59638	76.64799	510.64577	1933.03327	1851.02484	1
H	74.81350	77.29423	77.19265	-2302.30869	-1105.72939	-948.89774	1
C	73.55575	78.27327	78.72399	-559.93427	-126.34590	542.30857	1
O	73.23237	77.31666	79.40775	-670.36003	-111.83166	76.36448	1
N	73.61433	79.55345	79.11750	-216.06729	-537.07037	446.51379	1
H	73.87470	80.40404	78.60156	-1251.96487	1074.12894	-2168.52500	1
H	73.36439	79.86713	80.06283	-2451.83023	-720.73851	944.84612	1
C	73.74165	79.05842	74.10827	546.24052	-563.88337	-85.11809	1
O	72.90389	79.91476	73.98898	71.70702	-307.40463	606.69673	1
N	73.79508	77.84954	73.52274	479.90863	-504.50129	222.86849	1
C	72.74651	77.39778	72.66200	75.51268	-600.91904	506.73700	1
H	72.20102	78.30607	72.31240	-2033.61562	1795.30103	254.89480	1
C	73.58269	76.61175	71.55255	523.79067	517.99274	284.45581	1
H	72.96041	75.91021	70.95375	1599.61665	-1127.00911	1895.98173	1
H	74.06427	77.30957	70.83228	-1881.83093	1275.82488	-1501.55016	1
C	74.63899	75.89113	72.44185	-600.05639	-404.05026	316.64485	1
H	74.19276	75.00834	72.95193	-2597.05260	496.36221	657.73249	1
H	75.52040	75.51958	71.87232	-1666.24518	1294.38985	-1723.90591	1
C	75.00256	77.03637	73.48374	493.57570	-129.95376	-602.56394	1
H	75.20700	76.67341	74.51872	-1469.08415	-637.84797	2204.22581	1
H	75.82318	77.71927	73.15852	-1038.97840	1068.88701	-2280.71789	1
C	71.79026	76.31500	73.41175	188.88100	241.05089	-727.87821	1
O	72.04391	75.85130	74.50905	531.88613	-49.19943	427.07960	1
N	70.79173	75.91112	72.57596	708.14833	26.93417	-179.73509	1
H	70.88837	76.59739	71.81498	-2232.69501	-1179.09831	1023.94816	1
C	69.45704	75.61954	73.15297	-738.32412	259.92548	104.42456	1
H	69.28067	76.43266	73.89290	2028.89123	1260.02983	1311.33324	1
C	68.45812	75.59557	72.02615	-258.65678	-345.52513	661.28459	1
H	68.87407	74.94768	71.22096	-1826.06090	-1801.46097	-918.64554	1
C	67.12806	75.00426	72.46409	-541.23400	531.92970	-218.41428	1
H	66.41513	74.89402	71.60183	1311.73218	-2219.18200	882.19023	1
H	67.25891	73.98485	72.92541	-1048.71298	1223.58118	-2196.97971	1
H	66.62446	75.64884	73.23519	419.06414	1903.92898	1903.44670	1
C	68.21557	76.97346	71.43258	-423.86431	152.66566	-648.55262	1
H	67.47901	76.93497	70.58397	105.68386	-2342.50080	1387.51238	1
H	67.80165	77.68469	72.19870	-1695.63603	-1484.73621	-1531.02793	1

H	69.16073	77.43087	71.03258	-1853.57121	-487.39031	1936.59343	1
C	69.49158	74.12850	73.86142	539.71428	-510.58232	267.58633	1
O	68.67276	73.87117	74.71836	-15.93758	-278.34085	-624.49402	1
N	70.34787	73.30837	73.26839	-676.81886	12.05292	276.17960	1
H	70.92567	73.78554	72.57758	-1694.44250	-373.84189	-2100.66098	1
C	70.72628	72.06419	73.84632	412.33078	-664.70360	108.35451	1
H	71.78598	71.93408	73.52409	1765.77476	1294.44366	1621.76594	1
C	70.52759	72.14277	75.41588	-179.66635	-721.95667	-264.73915	1
H	70.98142	73.07336	75.82414	-607.34922	2587.87032	-598.10018	1
H	69.43996	72.20214	75.68229	-359.69332	-1479.13954	-2259.74260	1
C	71.17888	70.86493	76.04390	-240.01076	616.02425	431.85322	1
H	70.56957	69.95819	75.82232	-1359.17131	782.54425	2227.99181	1
H	72.18291	70.66748	75.60345	-820.36667	85.81767	-2596.78715	1
C	71.28601	71.11225	77.60111	-513.07015	236.18570	-551.87280	1
H	71.89153	72.02232	77.82323	1816.11944	-1183.11722	-1650.94321	1
H	70.28170	71.30133	78.04534	-1800.04429	-1361.53473	1526.34643	1
C	71.93484	69.84520	78.20698	726.08972	-253.19732	-179.64713	1
H	71.34393	68.91682	78.07407	1224.27271	-456.51798	-2390.90304	1
H	72.98507	69.67180	77.89803	1206.40339	-1150.93514	-2154.90344	1
N	71.99769	70.05584	79.75543	329.70796	-633.68968	-155.67358	1
H	72.41712	69.25968	80.31746	1348.31305	1938.76845	1359.00643	1
H	72.55943	70.88456	80.10851	2096.92035	1446.54551	-966.48890	1
H	71.06935	70.19463	80.24944	-1197.07217	-2303.53719	827.28733	1
C	69.78558	70.82878	73.33847	-378.01186	-556.17301	-413.96481	1
O	70.09271	69.68899	73.61480	-409.44433	-307.55053	-453.30833	1
N	68.66466	71.24551	72.74409	-429.02477	524.70884	274.08502	1
H	68.61317	72.24287	72.54079	1706.34263	-1425.96036	1574.38926	1
C	67.93763	70.24073	72.03165	-45.25864	-626.22952	478.93267	1
H	68.07046	69.31236	72.63454	1988.63164	570.90773	1772.87287	1
C	66.44646	70.74748	71.93177	551.77128	-20.49526	-564.54996	1
H	66.10143	71.12276	72.92182	1434.61535	-1753.10253	1513.99471	1
H	66.35998	71.61311	71.23725	-714.67738	1819.09770	1898.36494	1
C	65.56070	69.54703	71.45558	446.99263	-555.77305	338.97221	1
H	65.80260	69.27080	70.40456	1953.68466	1892.25957	-161.67766	1
H	65.76644	68.63946	72.06602	-403.03785	2688.07032	-188.43591	1
C	64.04421	69.97239	71.60033	567.86262	335.74681	434.04421	1
H	63.81212	70.25243	72.65370	-1080.02275	1568.26081	-1948.78900	1
H	63.82779	70.88012	70.99179	-919.54108	2111.34692	1456.13463	1
C	63.16116	68.78636	71.14809	195.81897	173.33862	-745.11536	1
H	63.24395	68.53338	70.07232	1405.67949	-2301.21089	-390.08113	1
H	63.26825	67.86960	71.76167	-940.37328	-1118.32446	2299.72133	1
N	61.66474	69.20972	71.33914	319.26422	656.36007	-42.02424	1
H	60.94451	68.48580	71.05200	1961.03633	619.91968	1787.09422	1
H	61.34714	69.43710	72.32619	854.14343	-2156.80752	1429.08711	1
H	61.33027	70.05991	70.79900	1237.02905	-1378.50857	-1998.28500	1
C	68.64344	70.12059	70.54590	532.53319	-268.57236	517.55729	1
O	68.98395	71.08361	69.89725	266.16714	-290.73804	-558.88002	1
N	68.64258	68.83854	70.22812	-226.25352	640.49678	-270.32839	1
H	68.27518	68.20240	70.93410	2007.69063	-1035.27229	1523.50231	1
C	69.26826	68.29904	69.07410	-540.76542	-573.33303	49.50000	1
H	68.75955	67.31741	68.95195	-2374.20950	-1036.76023	843.75774	1
C	69.05693	69.28653	67.83962	-77.09082	666.87422	-415.84136	1
H	69.62924	70.23988	68.03947	-985.81834	617.19825	2463.91927	1
O	67.68887	69.69156	67.60222	293.29899	-391.65602	-477.80926	1
H	67.68126	70.54019	68.09860	2528.72714	-681.76262	-751.27122	1
C	69.76324	68.49201	66.68184	252.47363	-605.14473	440.05134	1
H	69.83279	69.10138	65.75075	-2052.65063	1456.49051	-1043.51830	1
H	70.79967	68.18883	66.96234	-2152.98800	-1661.97574	-161.70652	1
H	69.20631	67.56079	66.42654	-473.12726	-1584.27339	2165.61725	1
C	70.86759	68.07215	69.38792	360.36307	488.46713	505.10056	1
O	71.29541	66.95452	69.21746	-200.08817	-528.03552	-385.82850	1
N	71.53324	69.16751	69.73439	-597.54095	-226.44775	355.20479	1

H	71.02549	70.05012	69.82867	2179.23655	-1300.70981	-991.33835	1
C	72.91983	69.00951	69.99652	-687.68921	297.39807	-249.45179	1
H	73.07792	67.93578	70.26075	-349.16256	-2467.37713	-1101.72831	1
C	73.73855	69.47375	68.68917	672.47345	361.54328	-201.62880	1
H	73.45800	70.51678	68.42046	1541.41502	582.18425	2169.97026	1
H	74.82380	69.48889	68.94074	-2534.67717	60.25153	997.72295	1
C	73.49751	68.54021	67.49993	574.26774	-389.43119	-377.02542	1
C	73.92799	67.17965	67.56346	688.82679	-287.33580	-257.96133	1
H	74.42374	66.78552	68.46937	331.14182	1470.38163	2269.80013	1
C	72.85404	69.04210	66.32712	-383.52382	-675.04808	144.24917	1
H	72.52270	70.09469	66.27316	4.09847	1517.59662	2262.86397	1
C	73.69013	66.35439	66.46610	-134.76730	428.45285	-649.50303	1
H	74.01273	65.29971	66.51971	-903.23433	1600.49020	2011.53387	1
C	72.64026	68.17488	65.25414	-335.53281	-615.00040	-364.39058	1
H	72.13505	68.57094	64.35596	1596.45398	-1369.33862	1732.02659	1
C	73.04239	66.80719	65.27977	585.48547	-340.24194	-406.23938	1
O	72.82580	65.91840	64.19549	-419.15649	-173.04408	-511.94141	1
H	72.37131	66.52936	63.56805	-1071.84350	1559.71507	-1960.13012	1
C	73.46359	70.11692	71.04880	291.41878	-183.13091	710.72246	1
O	73.15715	71.28226	70.97937	379.87695	523.47396	-222.23622	1
N	74.42862	69.56577	71.81188	564.30591	373.09380	277.24371	1
H	74.52929	68.53395	71.91178	-120.59931	-2436.07824	-1214.35098	1
C	75.15161	70.53711	72.58896	404.07514	-299.37480	608.84009	1
H	75.26846	71.37205	71.85994	-1071.07882	-841.69025	2359.66467	1
C	74.18222	70.99173	73.75271	-683.12807	68.71770	-390.13096	1
H	73.15482	71.14289	73.33790	2068.88918	1596.58590	770.89603	1
H	74.08690	70.19503	74.52487	1379.86429	2342.49779	-179.85638	1
C	74.74387	72.31013	74.37707	-176.39967	537.49466	550.97268	1
H	75.71376	72.12131	74.89783	-396.28051	1942.22168	1869.33314	1
H	74.96465	73.06443	73.58957	-94.44700	586.00678	-2659.20016	1
C	73.63250	72.84633	75.37913	-291.69381	-718.18079	-150.72929	1
H	72.84208	73.48715	74.91958	-2303.38776	1089.76242	964.62032	1
H	73.13351	72.02707	75.94996	884.16729	2568.80533	-207.74283	1
N	74.26770	73.60644	76.42940	-594.49522	-252.81478	342.29276	1
H	75.23138	73.50979	76.72745	-1735.17426	2084.05705	-263.71605	1
C	73.61806	74.41407	77.21464	595.23177	-271.52487	442.22352	1
N	72.20524	74.65484	76.97257	585.53813	-424.79750	-105.81303	1
H	71.93644	74.93071	76.00170	959.88797	1327.56823	-2177.11905	1
H	71.90391	75.41783	77.58051	1578.48647	16.27378	-2220.76233	1
N	74.22656	75.05835	78.29287	-452.17763	-390.33541	-421.51853	1
H	75.21310	74.98927	78.56538	470.26107	1974.25629	-1817.92087	1
H	73.71946	75.74493	78.91111	1856.67307	-1573.97321	-1224.35416	1
C	76.55438	69.98445	73.21951	-531.47271	-581.45780	-55.06206	1
O	77.09241	70.66371	74.07164	-243.00837	192.73507	609.52485	1
N	76.95996	68.85245	72.68293	343.49288	-644.25607	38.09164	1
H	76.37695	68.48487	71.93413	-1691.16043	-1535.97525	-1484.73099	1
C	78.12224	68.12339	73.10018	542.66492	350.14337	454.42563	1
H	78.76982	68.11795	72.19629	1209.57979	-2396.95920	463.86744	1
C	78.84701	68.72993	74.37455	-737.14309	145.93010	242.71942	1
H	78.11452	69.13387	75.11453	-1050.18303	159.36419	-2509.06125	1
C	79.69211	67.57010	75.01192	-706.77696	-252.88343	-245.16230	1
H	80.38953	67.94555	75.79809	-1624.64039	-1665.49085	1417.86935	1
H	79.03451	66.80118	75.48642	2566.10521	598.51109	693.22305	1
H	80.29828	67.03644	74.24069	-1972.69758	107.10018	-1876.34312	1
C	79.83450	69.84916	73.89485	412.85148	672.58832	-27.69222	1
H	80.40688	70.28567	74.74662	1447.02277	1762.76533	-1490.78944	1
H	80.57579	69.45848	73.15888	1320.77379	-145.20455	2378.68491	1
H	79.27865	70.68515	73.40437	-1512.10848	1470.52031	1724.75147	1
C	77.68964	66.60656	73.47682	-607.11113	499.56689	73.73769	1
O	78.52964	65.73195	73.53060	357.97126	460.81534	356.68785	1
N	76.40732	66.53874	73.86659	536.55407	-368.62270	332.76306	1
H	75.86153	67.39281	73.72590	524.38866	2297.69985	1367.20941	1

```
C    75.75478  65.28632  73.60415        46.50277      -774.29190      -147.97904          1
H    76.55735  64.62204  73.20656      1676.51875      1883.29066      1032.55549          1
C    75.14977  64.74166  74.99868      -559.41456       -34.19198      -556.30433          1
H    74.28227  65.37433  75.30139       -64.66664       395.18769      2695.05373          1
H    74.74792  63.70731  74.88190     -2703.44278       -60.40056       333.79418          1
C    76.22925  64.79538  76.04583      -519.76435       377.31045       459.42366          1
H    76.65877  65.82353  76.04267     -1746.74323      1667.50644     -1261.73691          1
C    75.65870  64.52033  77.43130       570.95554        16.46261      -545.27700          1
H    76.44988  64.58037  78.22257      2391.58273        28.05277     -1305.07271          1
H    74.86090  65.25834  77.70354       175.75588       917.63458      2559.43867          1
H    75.20300  63.49918  77.49571     -1063.97851     -2155.34650      1283.00462          1
C    77.35380  63.81509  75.74323       193.76014         2.62946      -765.53141          1
H    78.10994  63.78527  76.56970       507.39394      -846.55087      2539.60089          1
H    76.97053  62.77091  75.60717      1337.57841      1818.19054      1526.01994          1
H    77.90383  64.10466  74.80318      1100.88635     -1172.91350     -2199.08909          1
C    74.60709  65.63443  72.55236      -549.21115       475.64486      -309.38218          1
O    74.40982  66.76778  72.14655       556.06106      -390.37755       -78.24250          1
O    73.82293  64.56446  72.09383      -572.27940      -310.19032      -209.75862          1
H    74.25177  63.82389  72.61939     -1380.08193      2212.14486       790.85944          1
```

Listing 54: C00054.xmolout

```
      276
  150.0000  150.0000  150.0000     0.0000     0.0000     0.0000***********    0.9262     3705
      0.2500    300.00    0.00    0.00    0.00  -1293.6558       0.0000    300.0000
H    72.18135  64.80745  83.08062     -2043.14101     -1757.64893       399.89339          1
N    73.10615  64.54146  82.74919       426.75047       490.45733      -334.42467          1
H    72.90729  64.25040  81.79388       442.25559     -1110.30854     -2448.52907          1
C    74.09936  65.53669  82.86639       415.32972      -666.39213        83.73172          1
H    73.84715  66.04987  83.81830       879.32219      2491.00447       667.35213          1
C    75.54475  64.83151  82.86168        31.35357       477.41777       628.23362          1
H    75.65045  64.22251  81.93143      1168.38489      2240.25337     -1019.71136          1
H    76.35715  65.59740  82.82361      -360.43770      2534.90498       931.67112          1
C    75.67384  63.96171  84.08845       463.18208       512.70242      -382.34388          1
H    74.72476  63.39258  84.21200      1725.29117      1863.52247      -987.07854          1
C    76.81406  62.96655  83.91836       710.42801      -144.27427      -313.15367          1
H    76.92433  62.30589  84.81705       364.20195     -1033.13725      2494.72524          1
H    76.65173  62.29907  83.03263      2567.61904       335.80679       847.48802          1
H    77.79537  63.48651  83.76657     -2587.99029       680.92867      -512.16411          1
C    75.90952  64.80247  85.33529       268.17616       741.64379       -40.42634          1
H    76.00457  64.16476  86.25201      1283.54314     -1945.37420     -1411.27812          1
H    76.85069  65.40552  85.25286     -1258.57796      1317.15249     -2026.02085          1
H    75.07015  65.52185  85.51749     -2502.84526       909.80597       575.92391          1
C    73.98482  66.50533  81.55782       619.36578      -458.64843      -172.09273          1
O    74.83596  66.52393  80.69145       -81.40433       433.87611       522.34594          1
N    72.86497  67.21470  81.62577       175.23996      -622.20190       341.55523          1
H    72.26201  67.10963  82.44039      -876.57256      -303.22879     -2561.90213          1
C    72.55476  68.07906  80.53422      -571.88214      -534.10783      -106.15033          1
H    73.15294  67.68831  79.67596     -2713.20841       -16.25800      -248.80809          1
C    70.95996  68.06765  80.33235       166.60354       288.16399      -716.09568          1
H    70.48231  68.32741  81.31710     -2097.85574      1636.78449      -586.18044          1
O    70.37142  66.81969  79.94499      -317.80580      -363.38227       484.43082          1
H    69.80069  66.62877  80.71936      1863.74748     -1451.84923      1357.29467          1
C    70.81731  69.27251  79.33131       503.80731       517.99032       318.51606          1
H    69.75293  69.56681  79.18034       640.61020     -2157.10410     -1536.29104          1
H    71.37374  70.17308  79.70348      1687.88455       465.60692      2087.56452          1
H    71.23400  69.02145  78.32832     -1293.07895     -1899.88581     -1463.57452          1
C    72.94649  69.59448  80.97657      -153.07550      -733.68902       248.70981          1
O    72.26774  70.18881  81.80454       186.15459      -480.78787      -449.34387          1
N    73.98672  70.02301  80.26916      -487.53139       543.18517        42.03850          1
C    74.48390  71.36054  80.41235      -699.35871        69.34393       360.10701          1
H    74.44905  71.61109  81.49602         2.81126      -221.99807     -2715.58072          1
```

C	75.92561	71.24192	79.77154	-507.46439	-592.59194	122.07761	1
H	76.33619	72.22388	79.44401	-1975.08116	525.87560	-1801.71556	1
H	76.65964	70.81207	80.48988	1588.27743	-1913.22100	1113.83566	1
C	75.64913	70.26231	78.58708	-613.62442	-457.55993	-194.14490	1
H	75.13249	70.79347	77.75358	1707.54637	-721.27992	1996.92523	1
H	76.56800	69.80286	78.15742	758.23357	2100.81767	1560.55011	1
C	74.68170	69.21050	79.27445	-352.41524	704.96076	49.21465	1
H	73.91707	68.75749	78.59746	1714.76886	-2070.42778	443.35792	1
H	75.19377	68.39334	79.85217	1224.15040	1801.50881	1636.97633	1
C	73.50723	72.32463	79.50187	566.85117	537.05831	117.62814	1
O	73.36228	72.12472	78.32060	-550.26779	-406.08867	4.21666	1
N	73.05346	73.30493	80.26874	566.34832	-425.77725	180.18749	1
H	73.34482	73.30799	81.24631	644.44502	2646.80550	-52.73430	1
C	71.99766	74.16274	79.86765	612.34801	-281.48662	411.56221	1
H	71.94095	74.86948	80.72575	1667.23498	-1490.75142	1556.16753	1
C	72.37223	74.85905	78.48086	-714.45746	-175.43074	286.98927	1
H	72.35272	74.06737	77.67913	-787.09484	1763.03619	-1922.46060	1
O	73.66330	75.49043	78.41592	-429.46514	516.51932	-128.40526	1
H	74.09817	74.95812	77.71934	1981.82785	436.16847	-1818.18182	1
C	71.15068	75.83571	78.30952	630.79189	-468.94448	76.03230	1
H	71.14207	76.32365	77.29200	-1775.71861	2062.30353	131.89849	1
H	70.16940	75.30918	78.42039	-1855.14608	88.94118	1993.53760	1
H	71.17779	76.65700	79.06207	-1504.82350	-780.94249	-2132.90992	1
C	70.64253	73.24075	79.71123	288.57638	618.62814	-397.00270	1
O	70.10282	73.05586	78.64577	321.70495	-185.26452	574.37218	1
N	70.28791	72.79096	80.90591	-447.05792	-446.53431	367.76425	1
H	70.84838	73.03118	81.72227	350.31316	1975.63644	-1843.31522	1
C	69.05324	72.10827	81.04317	-511.23061	374.03917	-471.51480	1
H	68.75951	71.80692	80.01004	2234.79579	-898.70890	-1273.45190	1
C	69.37724	70.89292	82.03747	529.53781	-159.34528	563.72604	1
H	68.37775	70.48777	82.34910	654.59388	2347.94251	1217.51488	1
H	69.86742	70.09724	81.41453	-1526.55661	2103.42026	817.87394	1
O	70.20659	71.14317	83.17328	-554.84278	14.12789	399.58769	1
H	71.11276	70.82490	82.79725	1297.06236	2061.84854	1220.68812	1
C	67.98391	73.13774	81.72735	-662.15484	-412.13544	123.62687	1
O	68.20484	73.73516	82.77767	-540.01537	-317.05804	274.91456	1
N	66.91826	73.20444	80.96173	-202.76299	-690.00100	-131.49145	1
H	66.95828	72.61063	80.13395	-702.62132	-2617.37191	281.70413	1
C	65.69089	73.88467	81.32622	-491.73914	576.34187	222.73509	1
H	65.00464	73.61532	80.48728	1977.17978	-1823.58850	-434.68928	1
C	65.22991	73.35936	82.67773	-649.50797	-328.66843	306.11224	1
H	66.01635	73.62732	83.42231	2075.07803	1452.16490	-1004.45904	1
C	63.91913	73.98757	83.10976	-615.18735	-489.99689	-70.96352	1
H	63.57844	73.60202	84.10995	-1226.32661	-1724.38906	-1716.47170	1
H	64.01134	75.11189	83.19598	2381.64273	-969.84735	900.46860	1
H	63.09514	73.77577	82.37337	2122.74991	1702.23946	-141.36228	1
C	65.09966	71.84669	82.66817	-501.86074	-479.61276	-376.42482	1
H	64.79486	71.44482	83.67302	993.79261	-2246.95401	-1177.81305	1
H	64.33059	71.50328	81.92240	42.71279	-626.15391	-2651.37258	1
H	66.07314	71.35580	82.39200	-2026.39012	-1590.33709	-887.82922	1
C	65.98547	75.52835	81.39244	-672.89153	382.57394	-156.34072	1
O	65.54988	76.22876	82.27792	410.04627	221.86223	500.35950	1
N	66.60374	75.94661	80.26617	-378.11782	-443.79414	-441.10984	1
H	66.83571	75.07296	79.78553	2404.15973	-568.14894	1149.30085	1
C	67.63090	76.94694	80.45221	-544.54484	466.16019	331.29119	1
H	67.17087	77.76085	81.05188	-1409.80988	-170.17955	2325.32679	1
C	68.75341	76.16696	81.24494	-184.48284	116.78282	-758.89137	1
H	68.30736	75.76483	82.18835	1893.78641	1861.14652	611.04528	1
H	69.08571	75.27422	80.66639	-2170.79665	-974.19311	1327.50117	1
C	69.97129	77.07476	81.59740	389.51668	672.81878	-138.49152	1
H	70.41867	77.51210	80.67810	510.64577	1933.03327	1851.02484	1
H	69.63078	77.95736	82.18132	-2302.30869	-1105.72939	-948.89774	1

C	71.06945	76.29612	82.42078	-559.93427	-126.34590	542.30857	1
O	72.13845	76.82073	82.60400	-670.36003	-111.83166	76.36448	1
N	70.69466	75.08543	82.88553	-216.06729	-537.07037	446.51379	1
H	69.77399	74.58460	82.84357	-1251.96487	1074.12894	-2168.52500	1
H	71.30766	74.55805	83.52339	-2451.83023	-720.73851	944.84612	1
C	68.06201	77.36003	78.94588	546.24052	-563.88337	-85.11809	1
O	68.22929	76.48227	78.13842	71.70702	-307.40463	606.69673	1
N	68.24547	78.68190	78.80296	479.90863	-504.50129	222.86849	1
C	68.32157	79.28349	77.50725	75.51268	-600.91904	506.73700	1
H	67.66479	78.69290	76.82803	-2033.61562	1795.30103	254.89480	1
C	67.85606	80.77201	77.82086	523.79067	517.99274	284.45581	1
H	68.17447	81.49869	77.04015	1599.61665	-1127.00911	1895.98173	1
H	66.74950	80.85151	77.90285	-1881.83093	1275.82488	-1501.55016	1
C	68.54902	81.00015	79.19766	-600.05639	-404.05026	316.64485	1
H	69.64957	81.12337	79.07419	-2597.05260	496.36221	657.73249	1
H	68.17822	81.89376	79.74864	-1666.24518	1294.38985	-1723.90591	1
C	68.19875	79.62391	79.91287	493.57570	-129.95376	-602.56394	1
H	68.92375	79.29190	80.69162	-1469.08415	-637.84797	2204.22581	1
H	67.16269	79.57246	80.32417	-1038.97840	1068.88701	-2280.71789	1
C	69.86760	79.38479	76.97659	188.88100	241.05089	-727.87821	1
O	70.80688	79.45871	77.73415	531.88613	-49.19943	427.07960	1
N	69.85156	79.64521	75.64577	708.14833	26.93417	-179.73509	1
H	68.87658	79.49586	75.37425	-2232.69501	-1179.09831	1023.94816	1
C	70.92897	79.19775	74.74074	-738.32412	259.92548	104.42456	1
H	70.68694	79.68379	73.76700	2028.89123	1260.02983	1311.33324	1
C	72.25410	79.64637	75.31200	-258.65678	-345.52513	661.28459	1
H	72.32762	79.26086	76.36378	-1826.06090	-1801.46097	-918.64554	1
C	73.43241	79.10771	74.52224	-541.23400	531.92970	-218.41428	1
H	74.41181	79.46425	74.94411	1311.73218	-2219.18200	882.19023	1
H	73.46152	77.97377	74.52739	-1048.71298	1223.58118	-2196.97971	1
H	73.39585	79.43479	73.44678	419.06414	1903.92898	1903.44670	1
C	72.34366	81.16197	75.38027	-423.86431	152.66566	-648.55262	1
H	73.30742	81.50170	75.84869	105.68386	-2342.50080	1387.51238	1
H	72.28485	81.62873	74.35907	-1695.63603	-1484.73621	-1531.02793	1
H	71.50299	81.58907	75.99303	-1853.57121	-487.39031	1936.59343	1
C	70.83156	77.55907	74.63326	539.71428	-510.58232	267.58633	1
O	70.40045	76.87708	75.54275	-15.93758	-278.34085	-624.49402	1
N	71.29804	77.14053	73.46666	-676.81886	12.05292	276.17960	1
H	71.64966	77.84923	72.82645	-1694.44250	-373.84189	-2100.66098	1
C	71.70267	75.76880	73.39740	412.33078	-664.70360	108.35451	1
H	71.38469	75.35522	74.38583	1765.77476	1294.44366	1621.76594	1
C	70.91611	75.13124	72.18376	-179.66635	-721.95667	-264.73915	1
H	69.82428	75.31153	72.30379	-607.34922	2587.87032	-598.10018	1
H	71.18655	75.61852	71.22094	-359.69332	-1479.13954	-2259.74260	1
C	71.21856	73.59571	72.15507	-240.01076	616.02425	431.85322	1
H	72.29882	73.40044	71.97030	-1359.17131	782.54425	2227.99181	1
H	70.98458	73.12810	73.13770	-820.36667	85.81767	-2596.78715	1
C	70.32081	72.97472	71.01042	-513.07015	236.18570	-551.87280	1
H	69.24974	73.23929	71.16611	1816.11944	-1183.11722	-1650.94321	1
H	70.59611	73.40061	70.01834	-1800.04429	-1361.53473	1526.34643	1
C	70.50261	71.43942	71.01945	726.08972	-253.19732	-179.64713	1
H	71.51924	71.09174	70.74836	1224.27271	-456.51798	-2390.90304	1
H	70.16680	70.94008	71.95021	1206.40339	-1150.93514	-2154.90344	1
N	69.56926	70.85730	69.90381	329.70796	-633.68968	-155.67358	1
H	69.58582	69.80267	69.79115	1348.31305	1938.76845	1359.00643	1
H	68.52813	71.03852	70.00412	2096.92035	1446.54551	-966.48890	1
H	69.75135	71.17678	68.90808	-1197.07217	-2303.53719	827.28733	1
C	73.33360	75.80164	73.22325	-378.01186	-556.17301	-413.96481	1
O	74.02183	76.01719	74.19805	-409.44433	-307.55053	-453.30833	1
N	73.72601	75.61410	71.97583	-429.02477	524.70884	274.08502	1
H	73.06486	75.42964	71.22357	1706.34263	-1425.96036	1574.38926	1
C	75.10318	75.83914	71.66790	-45.25864	-626.22952	478.93267	1

H	75.46638	76.62334	72.37064	1988.63164	570.90773	1772.87287	1
C	75.82049	74.44864	71.92452	551.77128	-20.49526	-564.54996	1
H	75.70163	74.14903	72.99027	1434.61535	-1753.10253	1513.99471	1
H	75.34525	73.63772	71.32878	-714.67738	1819.09770	1898.36494	1
C	77.33022	74.61227	71.55357	446.99263	-555.77305	338.97221	1
H	77.45577	74.76484	70.45732	1953.68466	1892.25957	-161.67766	1
H	77.76497	75.51236	72.04469	-403.03785	2688.07032	-188.43591	1
C	78.08008	73.30469	72.03054	567.86262	335.74681	434.04421	1
H	77.89817	73.11065	73.11270	-1080.02275	1568.26081	-1948.78900	1
H	77.70268	72.40528	71.49054	-919.54108	2111.34692	1456.13463	1
C	79.58580	73.52795	71.75714	195.81897	173.33862	-745.11536	1
H	79.84642	73.61684	70.68400	1405.67949	-2301.21089	-390.08113	1
H	80.04779	74.35808	72.33093	-940.37328	-1118.32446	2299.72133	1
N	80.31881	72.23552	72.24297	319.26422	656.36007	-42.02424	1
H	81.36036	72.18104	72.04957	1961.03633	619.91968	1787.09422	1
H	80.28343	72.02283	73.28243	854.14343	-2156.80752	1429.08711	1
H	79.97625	71.32360	71.82324	1237.02905	-1378.50857	-1998.28500	1
C	75.16527	76.21207	70.07559	532.53319	-268.57236	517.55729	1
O	74.45658	75.66150	69.26403	266.16714	-290.73804	-558.88002	1
N	76.12571	77.10852	69.86370	-226.25352	640.49678	-270.32839	1
H	76.65479	77.47902	70.65262	2007.69063	-1035.27229	1523.50231	1
C	76.38744	77.45141	68.50640	-540.76542	-573.33303	49.50000	1
H	75.41093	77.30235	67.98910	-2374.20950	-1036.76023	843.75774	1
C	76.98510	78.93373	68.46046	-77.09082	666.87422	-415.84136	1
H	77.95951	78.94564	69.02327	-985.81834	617.19825	2463.91927	1
O	76.17000	79.95744	69.05619	293.29899	-391.65602	-477.80926	1
H	76.76501	80.28721	69.75999	2528.72714	-681.76262	-751.27122	1
C	77.28678	79.09278	66.92466	252.47363	-605.14473	440.05134	1
H	77.80049	80.05876	66.71010	-2052.65063	1456.49051	-1043.51830	1
H	77.95103	78.27258	66.54718	-2152.98800	-1661.97574	-161.70652	1
H	76.35117	79.07009	66.31845	-473.12726	-1584.27339	2165.61725	1
C	77.54747	76.43159	67.94346	360.36307	488.46713	505.10056	1
O	77.48824	76.04961	66.79591	-200.08817	-528.03552	-385.82850	1
N	78.44836	76.18843	68.88394	-597.54095	-226.44775	355.20479	1
H	78.26184	76.62418	69.78693	2179.23655	-1300.70981	-991.33835	1
C	79.76556	75.75385	68.60519	-687.68921	297.39807	-249.45179	1
H	80.19306	75.47755	69.59347	-349.16256	-2467.37713	-1101.72831	1
C	79.72908	74.55570	67.54602	672.47345	361.54328	-201.62880	1
H	79.14650	74.87881	66.63984	1541.41502	582.18425	2169.97026	1
H	80.76304	74.31419	67.20737	-2534.67717	60.25153	997.72295	1
C	79.07147	73.32431	68.18510	574.26774	-389.43119	-377.02542	1
C	79.87331	72.28270	68.74094	688.82679	-287.33580	-257.96133	1
H	80.97664	72.34558	68.72305	331.14182	1470.38163	2269.80013	1
C	77.64735	73.23097	68.21261	-383.52382	-675.04808	144.24917	1
H	77.02640	74.03761	67.77700	4.09847	1517.59662	2262.86397	1
C	79.23265	71.18293	69.31043	-134.76730	428.45285	-649.50303	1
H	79.85232	70.37452	69.73580	-903.23433	1600.49020	2011.53387	1
C	77.05231	72.11126	68.79748	-335.53281	-615.00040	-364.39058	1
H	75.94965	72.05406	68.80999	1596.45398	-1369.33862	1732.02659	1
C	77.81488	71.05162	69.36950	585.48547	-340.24194	-406.23938	1
O	77.21999	69.91690	69.98001	-419.15649	-173.04408	-511.94141	1
H	76.27155	70.09807	69.77674	-1071.84350	1559.71507	-1960.13012	1
C	80.59169	76.98081	67.89435	291.41878	-183.13091	710.72246	1
O	80.07394	77.85971	67.24114	379.87695	523.47396	-222.23622	1
N	81.87450	76.70090	68.06418	564.30591	373.09380	277.24371	1
H	82.08808	75.87057	68.61455	-120.59931	-2436.07824	-1214.35098	1
C	82.92566	77.56379	67.64905	404.07514	-299.37480	608.84009	1
H	83.81694	76.90060	67.58177	-1071.07882	-841.69025	2359.66467	1
C	82.48785	78.22155	66.27583	-683.12807	68.71770	-390.13096	1
H	81.99606	77.46105	65.62783	2068.88918	1596.58590	770.89603	1
H	81.70980	79.00415	66.45877	1379.86429	2342.49779	-179.85638	1
C	83.73338	78.83561	65.56406	-176.39967	537.49466	550.97268	1

```
H   84.20994  79.62694  66.18474     -396.28051     1942.22168     1869.33314          1
H   84.52264  78.07136  65.38693      -94.44700      586.00678    -2659.20016          1
C   83.16250  79.42107  64.19158     -291.69381     -718.18079     -150.72929          1
H   82.71275  78.66654  63.49693    -2303.38776     1089.76242      964.62032          1
H   82.42639  80.25740  64.30239      884.16729     2568.80533     -207.74283          1
N   84.30203  79.94291  63.56549     -594.49522     -252.81478      342.29276          1
H   85.16096  79.91750  64.09146    -1735.17426     2084.05705     -263.71605          1
C   84.31472  80.44195  62.39606      595.23177     -271.52487      442.22352          1
N   83.10899  80.46989  61.68556      585.53813     -424.79750     -105.81303          1
H   82.18456  80.14598  61.96531      959.88797     1327.56823    -2177.11905          1
H   82.99627  80.84523  60.74350     1578.48647       16.27378    -2220.76233          1
N   85.59954  80.88183  62.04721     -452.17763     -390.33541     -421.51853          1
H   86.47897  80.85691  62.56030      470.26107     1974.25629    -1817.92087          1
H   85.81423  81.30802  61.14488     1856.67307    -1573.97321    -1224.35416          1
C   83.11197  78.75998  68.75487     -531.47271     -581.45780      -55.06206          1
O   82.20609  79.48739  69.15069     -243.00837      192.73507      609.52485          1
N   84.39711  78.84333  69.01951      343.49288     -644.25607       38.09164          1
H   84.96555  78.13898  68.55042    -1691.16043    -1535.97525    -1484.73099          1
C   84.95371  79.62584  70.11150      542.66492      350.14337      454.42563          1
H   86.05681  79.51432  69.98241     1209.57979    -2396.95920      463.86744          1
C   84.47349  81.06701  69.99153     -737.14309      145.93010      242.71942          1
H   83.35713  81.06099  70.04286    -1050.18303      159.36419    -2509.06125          1
C   85.00096  81.92890  71.12128     -706.77696     -252.88343     -245.16230          1
H   84.64026  82.99131  71.03728    -1624.64039    -1665.49085     1417.86935          1
H   84.67174  81.53974  72.13332     2566.10521      598.51109      693.22305          1
H   86.12546  81.96049  71.12557    -1972.69758      107.10018    -1876.34312          1
C   84.85766  81.67378  68.65452      412.85148      672.58832      -27.69222          1
H   84.47884  82.72770  68.54851     1447.02277     1762.76533    -1490.78944          1
H   85.97386  81.70402  68.51862     1320.77379     -145.20455     2378.68491          1
H   84.43304  81.07744  67.80089    -1512.10848     1470.52031     1724.75147          1
C   84.44185  78.92499  71.53646     -607.11113      499.56689       73.73769          1
O   85.28555  78.53663  72.30253      357.97126      460.81534      356.68785          1
N   83.12787  78.90982  71.66170      536.55407     -368.62270      332.76306          1
H   82.50831  79.27586  70.90787      524.38866     2297.69985     1367.20941          1
C   82.61442  78.39715  72.88106       46.50277     -774.29190     -147.97904          1
H   83.43790  77.76904  73.29162     1676.51875     1883.29066     1032.55549          1
C   82.27323  79.64357  73.85422     -559.41456      -34.19198     -556.30433          1
H   81.54397  80.31367  73.34113      -64.66664      395.18769     2695.05373          1
H   81.76382  79.28485  74.78001    -2703.44278      -60.40056      333.79418          1
C   83.52329  80.40342  74.19843     -519.76435      377.31045      459.42366          1
H   84.06040  80.64823  73.24410    -1746.74323     1667.50644    -1261.73691          1
C   83.18093  81.70518  74.91308      570.95554       16.46261     -545.27700          1
H   84.10023  82.30008  75.15117     2391.58273       28.05277    -1305.07271          1
H   82.51480  82.35748  74.29196      175.75588      917.63458     2559.43867          1
H   82.65076  81.51508  75.88141    -1063.97851    -2155.34650     1283.00462          1
C   84.45934  79.58599  75.07843      193.76014        2.62946     -765.53141          1
H   85.34928  80.18580  75.40140      507.39394     -846.55087     2539.60089          1
H   83.94697  79.22479  76.00710     1337.57841     1818.19054     1526.01994          1
H   84.85192  78.68558  74.53477     1100.88635    -1172.91350    -2199.08909          1
C   81.24151  77.60119  72.58473     -549.21115      475.64486     -309.38218          1
O   80.52288  77.81639  71.64449      556.06106     -390.37755      -78.24250          1
O   80.84952  76.67412  73.58267     -572.27940     -310.19032     -209.75862          1
H   81.61820  76.79284  74.20711    -1380.08193     2212.14486      790.85944          1
```

Listing 55: C00055.xmolout

```
       276
  150.0000  150.0000  150.0000     0.0000     0.0000     0.0000***********    2.7750    11100
       0.2500     300.00    0.00    0.00    0.00   -1293.6534       0.0000     300.0000
H   71.37730  64.98872  62.03234    -2043.14101    -1757.64893      399.89339          1
N   71.39791  65.91270  62.46494      426.75047      490.45733     -334.42467          1
H   71.53437  65.70940  63.46823      442.25559    -1110.30854    -2448.52907          1
```

C	72.41840	66.75283	61.96331	415.32972	-666.39213	83.73172	1
H	72.92220	66.16850	61.16353	879.32219	2491.00447	667.35213	1
C	71.77798	68.12007	61.39828	31.35357	477.41777	628.23362	1
H	71.17617	68.60529	62.20364	1168.38489	2240.25337	-1019.71136	1
H	72.58014	68.84889	61.13162	-360.43770	2534.90498	931.67112	1
C	70.91693	67.82211	60.19997	463.18208	512.70242	-382.34388	1
H	70.15777	67.06334	60.49615	1725.29117	1863.52247	-987.07854	1
C	70.19564	69.08501	59.74359	710.42801	-144.27427	-313.15367	1
H	69.52819	68.88664	58.86568	364.20195	-1033.13725	2494.72524	1
H	69.55688	69.51516	60.55739	2567.61904	335.80679	847.48802	1
H	70.91949	69.88259	59.43453	-2587.99029	680.92867	-512.16411	1
C	71.73247	67.26808	59.03924	268.17616	741.64379	-40.42634	1
H	71.09995	67.11480	58.12702	1283.54314	-1945.37420	-1411.27812	1
H	72.56473	67.96107	58.75203	-1258.57796	1317.15249	-2026.02085	1
H	72.19468	66.27870	59.28789	-2502.84526	909.80597	575.92391	1
C	73.33603	66.98586	63.27851	619.36578	-458.64843	-172.09273	1
O	73.13617	66.33332	64.29105	-81.40433	433.87611	522.34594	1
N	74.28845	67.87474	63.03146	175.23996	-622.20190	341.55523	1
H	74.29180	68.34498	62.12785	-876.57256	-303.22879	-2561.90213	1
C	75.14052	68.30156	64.09471	-571.88214	-534.10783	-106.15033	1
H	75.38711	67.36739	64.64390	-2713.20841	-16.25800	-248.80809	1
C	76.37712	69.03912	63.40357	166.60354	288.16399	-716.09568	1
H	75.97440	69.90885	62.81565	-2097.85574	1636.78449	-586.18044	1
O	77.11922	68.23774	62.46185	-317.80580	-363.38227	484.43082	1
H	76.97688	68.73845	61.63550	1863.74748	-1451.84923	1357.29467	1
C	77.21272	69.59313	64.61510	503.80731	517.99032	318.51606	1
H	78.04151	70.25131	64.26453	640.61020	-2157.10410	-1536.29104	1
H	76.59220	70.19977	65.33550	1687.88455	465.60692	2087.56452	1
H	77.68382	68.76630	65.19508	-1293.07895	-1899.88581	-1463.57452	1
C	74.30527	69.37200	64.99883	-153.07550	-733.68902	248.70981	1
O	73.63415	70.21016	64.45892	186.15459	-480.78787	-449.34387	1
N	74.59982	69.22195	66.29752	-487.53139	543.18517	42.03850	1
C	74.09621	70.12664	67.28737	-699.35871	69.34393	360.10701	1
H	73.04388	70.35729	67.00194	2.81126	-221.99807	-2715.58072	1
C	74.28127	69.30409	68.63374	-507.46439	-592.59194	122.07761	1
H	74.35152	69.95574	69.53366	-1975.08116	525.87560	-1801.71556	1
H	73.43245	68.60630	68.80963	1588.27743	-1913.22100	1113.83566	1
C	75.59999	68.52725	68.33679	-613.62442	-457.55993	-194.14490	1
H	76.48589	69.19822	68.41236	1707.54637	-721.27992	1996.92523	1
H	75.78403	67.67145	69.02466	758.23357	2100.81767	1560.55011	1
C	75.33215	68.08279	66.83347	-352.41524	704.96076	49.21465	1
H	76.24922	67.90913	66.22589	1714.76886	-2070.42778	443.35792	1
H	74.64604	67.20882	66.72218	1224.15040	1801.50881	1636.97633	1
C	75.02936	71.46647	67.35422	566.85117	537.05831	117.62814	1
O	76.10007	71.56720	66.79898	-550.26779	-406.08867	4.21666	1
N	74.48082	72.33647	68.26172	566.34832	-425.77725	180.18749	1
H	73.54415	71.93600	68.39161	644.44502	2646.80550	-52.73430	1
C	74.56213	73.73275	67.88085	612.34801	-281.48662	411.56221	1
H	74.61343	73.74924	66.77072	1667.23498	-1490.75142	1556.16753	1
C	73.24793	74.38621	68.47868	-714.45746	-175.43074	286.98927	1
H	73.29357	74.33128	69.60139	-787.09484	1763.03619	-1922.46060	1
O	72.01000	73.72894	68.11613	-429.46514	516.51932	-128.40526	1
H	71.64308	73.51667	68.99492	1981.82785	436.16847	-1818.18182	1
C	73.38237	75.88817	68.03020	630.79189	-468.94448	76.03230	1
H	72.54735	76.50807	68.43185	-1775.71861	2062.30353	131.89849	1
H	74.33589	76.34775	68.38515	-1855.14608	88.94118	1993.53760	1
H	73.36125	75.98423	66.91975	-1504.82350	-780.94249	-2132.90992	1
C	75.86542	74.38937	68.58789	288.57638	618.62814	-397.00270	1
O	76.37957	75.38454	68.12472	321.70495	-185.26452	574.37218	1
N	76.13160	73.80065	69.75404	-447.05792	-446.53431	367.76425	1
H	75.60626	72.96490	70.00701	350.31316	1975.63644	-1843.31522	1
C	77.23373	74.31546	70.51249	-511.23061	374.03917	-471.51480	1

H	77.90908	74.72500	69.72375	2234.79579	-898.70890	-1273.45190	1
C	76.62773	75.46531	71.42205	529.53781	-159.34528	563.72604	1
H	77.45995	75.93412	72.00464	654.59388	2347.94251	1217.51488	1
H	76.21245	76.26297	70.75810	-1526.55661	2103.42026	817.87394	1
O	75.58831	75.02559	72.41483	-554.84278	14.12789	399.58769	1
H	74.84125	74.96035	71.80495	1297.06236	2061.84854	1220.68812	1
C	77.84999	73.05562	71.34164	-662.15484	-412.13544	123.62687	1
O	78.82623	72.48762	70.92720	-540.01537	-317.05804	274.91456	1
N	77.13034	72.77283	72.43458	-202.76299	-690.00100	-131.49145	1
H	76.31958	73.37289	72.67643	-702.62132	-2617.37191	281.70413	1
C	77.52801	71.62148	73.24746	-491.73914	576.34187	222.73509	1
H	78.11974	71.01027	72.52297	1977.17978	-1823.58850	-434.68928	1
C	78.35244	72.11493	74.42572	-649.50797	-328.66843	306.11224	1
H	77.73698	72.84529	74.99621	2075.07803	1452.16490	-1004.45904	1
C	78.71226	70.97198	75.35674	-615.18735	-489.99689	-70.96352	1
H	79.28392	71.33015	76.25687	-1226.32661	-1724.38906	-1716.47170	1
H	77.79376	70.43982	75.73101	2381.64273	-969.84735	900.46860	1
H	79.35056	70.20366	74.83883	2122.74991	1702.23946	-141.36228	1
C	79.60716	72.83743	73.97197	-501.86074	-479.61276	-376.42482	1
H	80.21186	73.21711	74.84134	993.79261	-2246.95401	-1177.81305	1
H	80.27377	72.16306	73.36571	42.71279	-626.15391	-2651.37258	1
H	79.35785	73.72116	73.32128	-2026.39012	-1590.33709	-887.82922	1
C	76.15023	70.87256	73.79900	-672.89153	382.57394	-156.34072	1
O	75.99410	69.69853	73.56564	410.04627	221.86223	500.35950	1
N	75.36808	71.68368	74.49106	-378.11782	-443.79414	-441.10984	1
H	75.63853	72.66001	74.58909	2404.15973	-568.14894	1149.30085	1
C	74.26444	71.11750	75.19327	-544.54484	466.16019	331.29119	1
H	74.17468	70.07281	74.81042	-1409.80988	-170.17955	2325.32679	1
C	74.62714	71.20494	76.74641	-184.48284	116.78282	-758.89137	1
H	75.70276	70.97194	76.91008	1893.78641	1861.14652	611.04528	1
H	74.47644	72.23867	77.13613	-2170.79665	-974.19311	1327.50117	1
C	73.72204	70.17959	77.49370	389.51668	672.81878	-138.49152	1
H	72.65697	70.49507	77.41509	510.64577	1933.03327	1851.02484	1
H	73.76413	69.18976	76.98819	-2302.30869	-1105.72939	-948.89774	1
C	74.11771	69.97072	79.00509	-559.93427	-126.34590	542.30857	1
O	73.78377	68.96323	79.58671	-670.36003	-111.83166	76.36448	1
N	74.80514	71.00727	79.52975	-216.06729	-537.07037	446.51379	1
H	74.88084	71.96945	79.11515	-1251.96487	1074.12894	-2168.52500	1
H	74.99699	71.06559	80.53921	-2451.83023	-720.73851	944.84612	1
C	72.97797	72.08097	74.96730	546.24052	-563.88337	-85.11809	1
O	73.09594	73.29496	75.05280	71.70702	-307.40463	606.69673	1
N	71.87763	71.36373	74.76008	479.90863	-504.50129	222.86849	1
C	70.61649	72.00918	74.50043	75.51268	-600.91904	506.73700	1
H	70.87180	72.85764	73.82416	-2033.61562	1795.30103	254.89480	1
C	69.75417	70.84804	73.84844	523.79067	517.99274	284.45581	1
H	68.65943	70.99782	73.98414	1599.61665	-1127.00911	1895.98173	1
H	69.93280	70.77214	72.75248	-1881.83093	1275.82488	-1501.55016	1
C	70.29868	69.59245	74.59827	-600.05639	-404.05026	316.64485	1
H	69.87321	69.53013	75.62506	-2597.05260	496.36221	657.73249	1
H	70.06045	68.62927	74.09302	-1666.24518	1294.38985	-1723.90591	1
C	71.85475	69.91255	74.63593	493.57570	-129.95376	-602.56394	1
H	72.40434	69.46462	75.49459	-1469.08415	-637.84797	2204.22581	1
H	72.39851	69.67855	73.68975	-1038.97840	1068.88701	-2280.71789	1
C	69.99552	72.53602	75.91108	188.88100	241.05089	-727.87821	1
O	69.04989	71.99181	76.40925	531.88613	-49.19943	427.07960	1
N	70.72828	73.56313	76.36020	708.14833	26.93417	-179.73509	1
H	71.58783	73.80355	75.80293	-2232.69501	-1179.09831	1023.94816	1
C	70.14898	74.65208	77.16613	-738.32412	259.92548	104.42456	1
H	70.37780	75.57172	76.57673	2028.89123	1260.02983	1311.33324	1
C	68.67594	74.48048	77.46500	-258.65678	-345.52513	661.28459	1
H	68.52545	73.45253	77.87917	-1826.06090	-1801.46097	-918.64554	1
C	68.21750	75.48468	78.50904	-541.23400	531.92970	-218.41428	1

130	H	67.15941	75.29475	78.83788	1311.73218	-2219.18200	882.19023	1
131	H	68.87124	75.44851	79.42693	-1048.71298	1223.58118	-2196.97971	1
132	H	68.26333	76.53719	78.11707	419.06414	1903.92898	1903.44670	1
133	C	67.80965	74.60775	76.22525	-423.86431	152.66566	-648.55262	1
134	H	66.71742	74.48500	76.46363	105.68386	-2342.50080	1387.51238	1
135	H	67.93695	75.61357	75.73410	-1695.63603	-1484.73621	-1531.02793	1
136	H	68.07508	73.82690	75.45772	-1853.57121	-487.39031	1936.59343	1
137	C	70.97529	74.62989	78.59354	539.71428	-510.58232	267.58633	1
138	O	71.13892	75.66825	79.22520	-15.93758	-278.34085	-624.49402	1
139	N	71.28193	73.40793	78.96002	-676.81886	12.05292	276.17960	1
140	H	71.08243	72.67964	78.27895	-1694.44250	-373.84189	-2100.66098	1
141	C	71.91064	73.23736	80.24027	412.33078	-664.70360	108.35451	1
142	H	71.29741	73.84575	80.94376	1765.77476	1294.44366	1621.76594	1
143	C	71.96731	71.69792	80.58916	-179.66635	-721.95667	-264.73915	1
144	H	70.95382	71.23769	80.58112	-607.34922	2587.87032	-598.10018	1
145	H	72.56100	71.13766	79.82733	-359.69332	-1479.13954	-2259.74260	1
146	C	72.63371	71.59378	82.00351	-240.01076	616.02425	431.85322	1
147	H	73.58780	72.16721	82.01333	-1359.17131	782.54425	2227.99181	1
148	H	71.99491	72.05245	82.79079	-820.36667	85.81767	-2596.78715	1
149	C	72.90998	70.06858	82.30602	-513.07015	236.18570	-551.87280	1
150	H	71.97414	69.54408	82.60972	1816.11944	-1183.11722	-1650.94321	1
151	H	73.26822	69.53860	81.36961	-1800.04429	-1361.53473	1526.34643	1
152	C	73.98265	69.99048	83.41816	726.08972	-253.19732	-179.64713	1
153	H	74.92296	70.53140	83.19008	1224.27271	-456.51798	-2390.90304	1
154	H	73.62655	70.25984	84.43287	1206.40339	-1150.93514	-2154.90344	1
155	N	74.41894	68.49149	83.51237	329.70796	-633.68968	-155.67358	1
156	H	75.19017	68.26827	84.20602	1348.31305	1938.76845	1359.00643	1
157	H	73.67611	67.78351	83.78327	2096.92035	1446.54551	-966.48890	1
158	H	74.80720	68.06761	82.62062	-1197.07217	-2303.53719	827.28733	1
159	C	73.42280	73.84022	80.08525	-378.01186	-556.17301	-413.96481	1
160	O	74.17698	73.58614	79.14521	-409.44433	-307.55053	-453.30833	1
161	N	73.67130	74.49836	81.19119	-429.02477	524.70884	274.08502	1
162	H	72.91042	74.53739	81.86748	1706.34263	-1425.96036	1574.38926	1
163	C	74.81648	75.33322	81.34623	-45.25864	-626.22952	478.93267	1
164	H	74.91586	75.45431	82.44882	1988.63164	570.90773	1772.87287	1
165	C	76.05495	74.59881	80.68099	551.77128	-20.49526	-564.54996	1
166	H	76.09204	73.53828	81.01971	1434.61535	-1753.10253	1513.99471	1
167	H	75.93200	74.55413	79.56625	-714.67738	1819.09770	1898.36494	1
168	C	77.38376	75.34441	81.02846	446.99263	-555.77305	338.97221	1
169	H	77.38286	76.37316	80.59592	1953.68466	1892.25957	-161.67766	1
170	H	77.51678	75.46728	82.12683	-403.03785	2688.07032	-188.43591	1
171	C	78.52444	74.44201	80.40399	567.86262	335.74681	434.04421	1
172	H	78.59375	73.46908	80.94259	-1080.02275	1568.26081	-1948.78900	1
173	H	78.28366	74.18482	79.34689	-919.54108	2111.34692	1456.13463	1
174	C	79.86681	75.20396	80.47056	195.81897	173.33862	-745.11536	1
175	H	79.93193	76.07286	79.78698	1405.67949	-2301.21089	-390.08113	1
176	H	80.18759	75.49150	81.49181	-940.37328	-1118.32446	2299.72133	1
177	N	80.98586	74.23445	79.95833	319.26422	656.36007	-42.02424	1
178	H	81.96758	74.63551	79.94379	1961.03633	619.91968	1787.09422	1
179	H	81.13550	73.33580	80.50274	854.14343	-2156.80752	1429.08711	1
180	H	80.89243	73.87761	78.96315	1237.02905	-1378.50857	-1998.28500	1
181	C	74.54972	76.77237	80.62432	532.53319	-268.57236	517.55729	1
182	O	75.44302	77.57991	80.67402	266.16714	-290.73804	-558.88002	1
183	N	73.35521	76.87305	80.05764	-226.25352	640.49678	-270.32839	1
184	H	72.66432	76.09003	80.07055	2007.69063	-1035.27229	1523.50231	1
185	C	72.87030	78.13617	79.60667	-540.76542	-573.33303	49.50000	1
186	H	71.80733	77.88808	79.37497	-2374.20950	-1036.76023	843.75774	1
187	C	73.00904	79.17374	80.82097	-77.09082	666.87422	-415.84136	1
188	H	74.10458	79.40905	80.94172	-985.81834	617.19825	2463.91927	1
189	O	72.54297	78.69709	82.09679	293.29899	-391.65602	-477.80926	1
190	H	73.38136	78.68058	82.60059	2528.72714	-681.76262	-751.27122	1
191	C	72.25712	80.45505	80.30816	252.47363	-605.14473	440.05134	1

192	H	72.33439	81.28959	81.04407	-2052.65063	1456.49051	-1043.51830	1
193	H	72.67583	80.83039	79.33043	-2152.98800	-1661.97574	-161.70652	1
194	H	71.17107	80.25458	80.15326	-473.12726	-1584.27339	2165.61725	1
195	C	73.69789	78.61834	78.29637	360.36307	488.46713	505.10056	1
196	O	73.66032	79.80518	77.96171	-200.08817	-528.03552	-385.82850	1
197	N	74.26239	77.60528	77.68046	-597.54095	-226.44775	355.20479	1
198	H	74.12915	76.70681	78.14510	2179.23655	-1300.70981	-991.33835	1
199	C	75.03384	77.69690	76.50178	-687.68921	297.39807	-249.45179	1
200	H	75.17726	76.63476	76.20423	-349.16256	-2467.37713	-1101.72831	1
201	C	74.25558	78.57670	75.41715	672.47345	361.54328	-201.62880	1
202	H	74.15718	79.62713	75.78152	1541.41502	582.18425	2169.97026	1
203	H	74.85740	78.61684	74.47999	-2534.67717	60.25153	997.72295	1
204	C	72.86185	77.99668	75.13310	574.26774	-389.43119	-377.02542	1
205	C	72.70985	76.77235	74.41469	688.82679	-287.33580	-257.96133	1
206	H	73.59309	76.18362	74.10435	331.14182	1470.38163	2269.80013	1
207	C	71.71404	78.72493	75.57090	-383.52382	-675.04808	144.24917	1
208	H	71.83103	79.65092	76.16332	4.09847	1517.59662	2262.86397	1
209	C	71.41983	76.34324	74.09750	-134.76730	428.45285	-649.50303	1
210	H	71.30276	75.40252	73.53204	-903.23433	1600.49020	2011.53387	1
211	C	70.44394	78.25904	75.22049	-335.53281	-615.00040	-364.39058	1
212	H	69.56409	78.84274	75.54399	1596.45398	-1369.33862	1732.02659	1
213	C	70.24853	77.07051	74.45862	585.48547	-340.24194	-406.23938	1
214	O	68.95737	76.60399	74.08038	-419.15649	-173.04408	-511.94141	1
215	H	68.41296	77.36584	74.39292	-1071.84350	1559.71507	-1960.13012	1
216	C	76.46002	78.45916	76.77222	291.41878	-183.13091	710.72246	1
217	O	77.48311	77.92931	76.40916	379.87695	523.47396	-222.23622	1
218	N	76.29178	79.67272	77.28632	564.30591	373.09380	277.24371	1
219	H	75.33516	80.00610	77.55635	-120.59931	-2436.07824	-1214.35098	1
220	C	77.45828	80.37806	77.67903	404.07514	-299.37480	608.84009	1
221	H	78.32911	79.80804	77.27602	-1071.07882	-841.69025	2359.66467	1
222	C	77.31065	81.85737	77.12090	-683.12807	68.71770	-390.13096	1
223	H	77.06748	81.84298	76.03493	2068.88918	1596.58590	770.89603	1
224	H	76.45845	82.37798	77.61271	1379.86429	2342.49779	-179.85638	1
225	C	78.65167	82.60916	77.38143	-176.39967	537.49466	550.97268	1
226	H	78.88752	82.63717	78.46893	-396.28051	1942.22168	1869.33314	1
227	H	79.50299	82.07758	76.90105	-94.44700	586.00678	-2659.20016	1
228	C	78.49203	84.08773	76.79807	-291.69381	-718.18079	-150.72929	1
229	H	78.22168	84.14165	75.71257	-2303.38776	1089.76242	964.62032	1
230	H	77.79362	84.76078	77.35693	884.16729	2568.80533	-207.74283	1
231	N	79.79450	84.59490	76.92214	-594.49522	-252.81478	342.29276	1
232	H	80.50988	83.94776	77.21476	-1735.17426	2084.05705	-263.71605	1
233	C	80.13030	85.79397	76.66874	595.23177	-271.52487	442.22352	1
234	N	79.12443	86.67820	76.26307	585.53813	-424.79750	-105.81303	1
235	H	78.13223	86.50380	76.10869	959.88797	1327.56823	-2177.11905	1
236	H	79.27482	87.65624	76.01400	1578.48647	16.27378	-2220.76233	1
237	N	81.50626	85.98806	76.86121	-452.17763	-390.33541	-421.51853	1
238	H	82.24763	85.33354	77.10660	470.26107	1974.25629	-1817.92087	1
239	H	81.97133	86.87752	76.67676	1856.67307	-1573.97321	-1224.35416	1
240	C	77.44039	80.53935	79.31883	-531.47271	-581.45780	-55.06206	1
241	O	76.42442	80.69047	79.95390	-243.00837	192.73507	609.52485	1
242	N	78.70057	80.59958	79.73253	343.49288	-644.25607	38.09164	1
243	H	79.44603	80.48671	79.04755	-1691.16043	-1535.97525	-1484.73099	1
244	C	78.94289	80.55920	81.13637	542.66492	350.14337	454.42563	1
245	H	77.98978	80.12875	81.52339	1209.57979	-2396.95920	463.86744	1
246	C	80.23843	79.67529	81.37218	-737.14309	145.93010	242.71942	1
247	H	81.13599	80.16671	80.93449	-1050.18303	159.36419	-2509.06125	1
248	C	80.40920	79.44730	82.91299	-706.77696	-252.88343	-245.16230	1
249	H	81.34137	78.87930	83.14338	-1624.64039	-1665.49085	1417.86935	1
250	H	80.45879	80.42692	83.47929	2566.10521	598.51109	693.22305	1
251	H	79.55536	78.86661	83.33501	-1972.69758	107.10018	-1876.34312	1
252	C	80.04354	78.26330	80.72039	412.85148	672.58832	-27.69222	1
253	H	80.93566	77.61365	80.88235	1447.02277	1762.76533	-1490.78944	1

```
H    79.15917  77.73679  81.14899      1320.77379      -145.20455      2378.68491          1
H    79.88051  78.33971  79.61964     -1512.10848      1470.52031      1724.75147          1
C    79.13589  82.10981  81.64844      -607.11113       499.56689        73.73769          1
O    78.15399  82.80362  81.77826       357.97126       460.81534       356.68785          1
N    80.41230  82.43992  81.78869       536.55407      -368.62270       332.76306          1
H    81.11771  81.72460  81.61411       524.38866      2297.69985      1367.20941          1
C    80.76442  83.58572  82.56113        46.50277      -774.29190      -147.97904          1
H    81.85255  83.70141  82.36104      1676.51875      1883.29066      1032.55549          1
C    79.89599  84.85544  82.07170      -559.41456       -34.19198      -556.30433          1
H    78.82197  84.70442  82.36330       -64.66664       395.18769      2695.05373          1
H    80.22631  85.79330  82.57883     -2703.44278       -60.40056       333.79418          1
C    80.01479  84.98116  80.57692      -519.76435       377.31045       459.42366          1
H    79.87330  83.96999  80.13145     -1746.74323      1667.50644     -1261.73691          1
C    78.92772  85.89319  80.02228       570.95554        16.46261      -545.27700          1
H    79.00406  85.99337  78.90837      2391.58273        28.05277     -1305.07271          1
H    77.90433  85.49778  80.25251       175.75588       917.63458      2559.43867          1
H    78.99281  86.92480  80.45425     -1063.97851     -2155.34650      1283.00462          1
C    81.38553  85.50861  80.17456       193.76014         2.62946      -765.53141          1
H    81.46018  85.64338  79.06562       507.39394      -846.55087      2539.60089          1
H    81.59950  86.50339  80.64332      1337.57841      1818.19054      1526.01994          1
H    82.20629  84.81112  80.48272      1100.88635     -1172.91350     -2199.08909          1
C    80.49244  83.23835  84.10117      -549.21115       475.64486      -309.38218          1
O    80.46308  82.10416  84.52213       556.06106      -390.37755       -78.24250          1
O    80.43217  84.32628  85.00047      -572.27940      -310.19032      -209.75862          1
H    80.53748  85.07178  84.34841     -1380.08193      2212.14486       790.85944          1
```

Listing 56: C00056.xmolout

```
       276
   150.0000  150.0000  150.0000     0.0000     0.0000     0.0000***********    2.7750    11100
        0.2500     300.00    0.00    0.00    0.00  -1293.6502       0.0000     300.0000
H    72.77505  69.33813  69.64068     -2043.14101     -1757.64893       399.89339          1
N    73.53534  68.77266  69.26137       426.75047       490.45733      -334.42467          1
H    73.07585  68.19540  68.53833       442.25559     -1110.30854     -2448.52907          1
C    74.59368  69.53908  68.72342       415.32972      -666.39213        83.73172          1
H    74.38038  70.59958  68.98400       879.32219      2491.00447       667.35213          1
C    76.00682  69.03949  69.31837        31.35357       477.41777       628.23362          1
H    76.20649  68.00141  68.96012      1168.38489      2240.25337     -1019.71136          1
H    76.84049  69.66558  68.91913      -360.43770      2534.90498       931.67112          1
C    75.98591  69.07711  70.82308       463.18208       512.70242      -382.34388          1
H    75.11799  68.47237  71.17112      1725.29117      1863.52247      -987.07854          1
C    77.26698  68.46467  71.37726       710.42801      -144.27427      -313.15367          1
H    77.26164  68.43402  72.49709       364.20195     -1033.13725      2494.72524          1
H    77.41448  67.41439  71.01586      2567.61904       335.80679       847.48802          1
H    78.17015  69.05090  71.06702     -2587.99029       680.92867      -512.16411          1
C    75.83354  70.49652  71.35268       268.17616       741.64379       -40.42634          1
H    75.84612  70.52219  72.47286      1283.54314     -1945.37420     -1411.27812          1
H    76.66266  71.16148  70.99770     -1258.57796      1317.15249     -2026.02085          1
H    74.86948  70.96181  71.02246     -2502.84526       909.80597       575.92391          1
C    74.47449  69.20022  67.14226       619.36578      -458.64843      -172.09273          1
O    73.58612  68.45713  66.73136       -81.40433       433.87611       522.34594          1
N    75.46704  69.77693  66.49669       175.23996      -622.20190       341.55523          1
H    76.09087  70.34876  67.06598      -876.57256      -303.22879     -2561.90213          1
C    75.52612  69.90893  65.07413      -571.88214      -534.10783      -106.15033          1
H    76.54844  70.32975  64.94886     -2713.20841       -16.25800      -248.80809          1
C    75.44648  68.44785  64.42575       166.60354       288.16399      -716.09568          1
H    74.45290  67.97917  64.67733     -2097.85574      1636.78449      -586.18044          1
O    76.40843  67.48839  64.90507      -317.80580      -363.38227       484.43082          1
H    75.82228  66.86681  65.38203      1863.74748     -1451.84923      1357.29467          1
C    75.54789  68.78491  62.89253       503.80731       517.99032       318.51606          1
H    75.34448  67.88756  62.26270       640.61020     -2157.10410     -1536.29104          1
H    74.83127  69.58464  62.58458      1687.88455       465.60692      2087.56452          1
```

H	76.56947	69.14469	62.62915	-1293.07895	-1899.88581	-1463.57452	1
C	74.46182	71.08040	64.72264	-153.07550	-733.68902	248.70981	1
O	74.81607	72.19881	65.08459	186.15459	-480.78787	-449.34387	1
N	73.33636	70.74783	64.10037	-487.53139	543.18517	42.03850	1
C	72.41696	71.76106	63.66083	-699.35871	69.34393	360.10701	1
H	73.01636	72.61868	63.27842	2.81126	-221.99807	-2715.58072	1
C	71.56965	70.96160	62.58359	-507.46439	-592.59194	122.07761	1
H	70.61514	71.46706	62.31502	-1975.08116	525.87560	-1801.71556	1
H	72.13744	70.81643	61.63739	1588.27743	-1913.22100	1113.83566	1
C	71.36160	69.61305	63.34185	-613.62442	-457.55993	-194.14490	1
H	70.58117	69.71257	64.12883	1707.54637	-721.27992	1996.92523	1
H	71.04290	68.77525	62.68129	758.23357	2100.81767	1560.55011	1
C	72.79918	69.39632	63.97591	-352.41524	704.96076	49.21465	1
H	72.82101	68.94869	65.01660	1714.76886	-2070.42778	443.35792	1
H	73.49526	68.83391	63.30881	1224.15040	1801.50881	1636.97633	1
C	71.43198	72.17057	64.90730	566.85117	537.05831	117.62814	1
O	71.47913	71.62451	65.98324	-550.26779	-406.08867	4.21666	1
N	70.62006	73.13183	64.48506	566.34832	-425.77725	180.18749	1
H	70.71654	73.43448	63.51755	644.44502	2646.80550	-52.73430	1
C	69.63911	73.69889	65.34045	612.34801	-281.48662	411.56221	1
H	69.10526	74.41697	64.67894	1667.23498	-1490.75142	1556.16753	1
C	68.68573	72.50232	65.81182	-714.45746	-175.43074	286.98927	1
H	69.29083	71.81499	66.46745	-787.09484	1763.03619	-1922.46060	1
O	68.15625	71.68477	64.74858	-429.46514	516.51932	-128.40526	1
H	68.58495	70.82919	64.94605	1981.82785	436.16847	-1818.18182	1
C	67.59041	73.21360	66.68534	630.79189	-468.94448	76.03230	1
H	66.91643	72.47180	67.17497	-1775.71861	2062.30353	131.89849	1
H	68.04952	73.84314	67.50081	-1855.14608	88.94118	1993.53760	1
H	66.94689	73.88197	66.06680	-1504.82350	-780.94249	-2132.90992	1
C	70.41449	74.44928	66.56426	288.57638	618.62814	-397.00270	1
O	69.88041	74.59697	67.64499	321.70495	-185.26452	574.37218	1
N	71.57536	74.94746	66.15678	-447.05792	-446.53431	367.76425	1
H	71.87311	74.76238	65.20032	350.31316	1975.63644	-1843.31522	1
C	72.47823	75.46084	67.11894	-511.23061	374.03917	-471.51480	1
H	71.97927	75.30067	68.10278	2234.79579	-898.70890	-1273.45190	1
C	73.83667	74.63328	66.93689	529.53781	-159.34528	563.72604	1
H	74.50102	74.94615	67.78903	654.59388	2347.94251	1217.51488	1
H	73.60535	73.54556	67.10501	-1526.55661	2103.42026	817.87394	1
O	74.56571	74.77975	65.71440	-554.84278	14.12789	399.58769	1
H	74.65305	73.79662	65.42892	1297.06236	2061.84854	1220.68812	1
C	72.69413	77.04287	66.75004	-662.15484	-412.13544	123.62687	1
O	72.91183	77.48686	65.64986	-540.01537	-317.05804	274.91456	1
N	72.65311	77.70984	67.91744	-202.76299	-690.00100	-131.49145	1
H	72.33088	77.13489	68.69387	-702.62132	-2617.37191	281.70413	1
C	72.54472	79.16830	67.92065	-491.73914	576.34187	222.73509	1
H	73.19281	79.45686	67.05992	1977.17978	-1823.58850	-434.68928	1
C	71.09312	79.56163	67.72137	-649.50797	-328.66843	306.11224	1
H	70.50427	79.18790	68.58806	2075.07803	1452.16490	-1004.45904	1
C	70.94752	81.07253	67.67302	-615.18735	-489.99689	-70.96352	1
H	69.87340	81.38517	67.56013	-1226.32661	-1724.38906	-1716.47170	1
H	71.34176	81.55461	68.60988	2381.64273	-969.84735	900.46860	1
H	71.51606	81.51390	66.80891	2122.74991	1702.23946	-141.36228	1
C	70.49481	78.93863	66.47319	-501.86074	-479.61276	-376.42482	1
H	69.45617	79.31769	66.26866	993.79261	-2246.95401	-1177.81305	1
H	71.12282	79.15772	65.56501	42.71279	-626.15391	-2651.37258	1
H	70.43340	77.81839	66.56052	-2026.39012	-1590.33709	-887.82922	1
C	73.11339	79.69357	69.39562	-672.89153	382.57394	-156.34072	1
O	74.06595	80.43542	69.43078	410.04627	221.86223	500.35950	1
N	72.40084	79.22711	70.40686	-378.11782	-443.79414	-441.10984	1
H	71.62367	78.61579	70.16950	2404.15973	-568.14894	1149.30085	1
C	72.77977	79.56897	71.74203	-544.54484	466.16019	331.29119	1
H	73.30633	80.54064	71.58571	-1409.80988	-170.17955	2325.32679	1

C	71.40592	79.68839	72.53347	-184.48284	116.78282	-758.89137	1
H	70.68675	80.30126	71.94676	1893.78641	1861.14652	611.04528	1
H	70.92629	78.68995	72.64186	-2170.79665	-974.19311	1327.50117	1
C	71.67083	80.31969	73.93773	389.51668	672.81878	-138.49152	1
H	72.37357	79.67839	74.51800	510.64577	1933.03327	1851.02484	1
H	72.21669	81.28169	73.81251	-2302.30869	-1105.72939	-948.89774	1
C	70.37525	80.55808	74.78698	-559.93427	-126.34590	542.30857	1
O	70.46972	80.65636	75.99642	-670.36003	-111.83166	76.36448	1
N	69.26225	80.67066	74.05043	-216.06729	-537.07037	446.51379	1
H	69.14617	80.61704	73.03135	-1251.96487	1074.12894	-2168.52500	1
H	68.32870	80.85847	74.43641	-2451.83023	-720.73851	944.84612	1
C	73.75961	78.52626	72.50767	546.24052	-563.88337	-85.11809	1
O	74.30056	78.98425	73.50971	71.70702	-307.40463	606.69673	1
N	73.85925	77.28711	72.03160	479.90863	-504.50129	222.86849	1
C	74.43179	76.22632	72.81628	75.51268	-600.91904	506.73700	1
H	74.00914	76.36378	73.83752	-2033.61562	1795.30103	254.89480	1
C	73.98647	74.91901	72.04189	523.79067	517.99274	284.45581	1
H	74.66485	74.05704	72.23424	1599.61665	-1127.00911	1895.98173	1
H	72.96256	74.59566	72.33475	-1881.83093	1275.82488	-1501.55016	1
C	74.02518	75.40430	70.55828	-600.05639	-404.05026	316.64485	1
H	75.07008	75.44403	70.17694	-2597.05260	496.36221	657.73249	1
H	73.45095	74.75618	69.85876	-1666.24518	1294.38985	-1723.90591	1
C	73.41046	76.86192	70.71251	493.57570	-129.95376	-602.56394	1
H	73.77840	77.61558	69.97703	-1469.08415	-637.84797	2204.22581	1
H	72.29557	76.88669	70.75854	-1038.97840	1068.88701	-2280.71789	1
C	76.06463	76.33190	72.80931	188.88100	241.05089	-727.87821	1
O	76.72410	75.46943	72.28496	531.88613	-49.19943	427.07960	1
N	76.46600	77.40368	73.48810	708.14833	26.93417	-179.73509	1
H	75.77590	78.10971	73.84485	-2232.69501	-1179.09831	1023.94816	1
C	77.85283	77.70132	73.43751	-738.32412	259.92548	104.42456	1
H	78.37054	76.76926	73.11056	2028.89123	1260.02983	1311.33324	1
C	78.01643	78.90374	72.40425	-258.65678	-345.52513	661.28459	1
H	77.37671	79.76116	72.70611	-1826.06090	-1801.46097	-918.64554	1
C	79.52573	79.31717	72.35308	-541.23400	531.92970	-218.41428	1
H	79.70430	80.13478	71.61568	1311.73218	-2219.18200	882.19023	1
H	79.88167	79.68129	73.34599	-1048.71298	1223.58118	-2196.97971	1
H	80.17669	78.45926	72.06275	419.06414	1903.92898	1903.44670	1
C	77.61670	78.40861	70.97325	-423.86431	152.66566	-648.55262	1
H	77.75713	79.20558	70.20509	105.68386	-2342.50080	1387.51238	1
H	78.22747	77.52928	70.65922	-1695.63603	-1484.73621	-1531.02793	1
H	76.54537	78.09638	70.93766	-1853.57121	-487.39031	1936.59343	1
C	78.29336	78.27787	74.91503	539.71428	-510.58232	267.58633	1
O	77.57244	78.99824	75.56599	-15.93758	-278.34085	-624.49402	1
N	79.54796	77.91548	75.13185	-676.81886	12.05292	276.17960	1
H	79.97960	77.31308	74.43319	-1694.44250	-373.84189	-2100.66098	1
C	80.23059	78.21660	76.34314	412.33078	-664.70360	108.35451	1
H	81.26727	77.87514	76.12582	1765.77476	1294.44366	1621.76594	1
C	80.12814	79.77888	76.57120	-179.66635	-721.95667	-264.73915	1
H	80.55669	80.33079	75.70455	-607.34922	2587.87032	-598.10018	1
H	79.05800	80.10086	76.61701	-359.69332	-1479.13954	-2259.74260	1
C	80.88923	80.13762	77.89053	-240.01076	616.02425	431.85322	1
H	80.53854	79.50423	78.73640	-1359.17131	782.54425	2227.99181	1
H	81.97939	79.93670	77.79238	-820.36667	85.81767	-2596.78715	1
C	80.61450	81.66689	78.18631	-513.07015	236.18570	-551.87280	1
H	81.03567	82.31074	77.38025	1816.11944	-1183.11722	-1650.94321	1
H	79.52032	81.87771	78.20064	-1800.04429	-1361.53473	1526.34643	1
C	81.25193	82.00120	79.55481	726.08972	-253.19732	-179.64713	1
H	80.78603	81.48480	80.41776	1224.27271	-456.51798	-2390.90304	1
H	82.35305	81.88239	79.59324	1206.40339	-1150.93514	-2154.90344	1
N	81.00851	83.52432	79.82604	329.70796	-633.68968	-155.67358	1
H	81.37657	83.88936	80.75146	1348.31305	1938.76845	1359.00643	1
H	81.44350	84.21165	79.14408	2096.92035	1446.54551	-966.48890	1

H	80.00055	83.85556	79.85756	-1197.07217	-2303.53719	827.28733	1
C	79.56506	77.29471	77.52816	-378.01186	-556.17301	-413.96481	1
O	79.92271	76.12173	77.59764	-409.44433	-307.55053	-453.30833	1
N	78.73418	77.96051	78.29663	-429.02477	524.70884	274.08502	1
H	78.59210	78.92886	78.01456	1706.34263	-1425.96036	1574.38926	1
C	77.74503	77.33699	79.11116	-45.25864	-626.22952	478.93267	1
H	77.02775	78.16067	79.32670	1988.63164	570.90773	1772.87287	1
C	77.11797	76.16391	78.24650	551.77128	-20.49526	-564.54996	1
H	76.71123	76.55016	77.28431	1434.61535	-1753.10253	1513.99471	1
H	77.89779	75.41837	77.96114	-714.67738	1819.09770	1898.36494	1
C	76.00719	75.48987	79.11424	446.99263	-555.77305	338.97221	1
H	76.39462	75.26408	80.13386	1953.68466	1892.25957	-161.67766	1
H	75.14489	76.18112	79.24931	-403.03785	2688.07032	-188.43591	1
C	75.55730	74.15600	78.39630	567.86262	335.74681	434.04421	1
H	75.18033	74.35828	77.36763	-1080.02275	1568.26081	-1948.78900	1
H	76.41358	73.45324	78.27752	-919.54108	2111.34692	1456.13463	1
C	74.45027	73.54724	79.29489	195.81897	173.33862	-745.11536	1
H	74.78555	73.25287	80.31078	1405.67949	-2301.21089	-390.08113	1
H	73.53489	74.16990	79.37241	-940.37328	-1118.32446	2299.72133	1
N	73.95182	72.23477	78.64496	319.26422	656.36007	-42.02424	1
H	73.00101	71.86644	79.12954	1961.03633	619.91968	1787.09422	1
H	73.71464	72.28130	77.61377	854.14343	-2156.80752	1429.08711	1
H	74.60753	71.40338	78.66392	1237.02905	-1378.50857	-1998.28500	1
C	78.37506	76.71453	80.48647	532.53319	-268.57236	517.55729	1
O	77.91965	77.10103	81.55320	266.16714	-290.73804	-558.88002	1
N	79.27319	75.80313	80.22457	-226.25352	640.49678	-270.32839	1
H	79.54800	75.64118	79.22402	2007.69063	-1035.27229	1523.50231	1
C	80.05786	75.18690	81.23426	-540.76542	-573.33303	49.50000	1
H	80.79175	74.59431	80.64291	-2374.20950	-1036.76023	843.75774	1
C	80.75634	76.33922	82.10164	-77.09082	666.87422	-415.84136	1
H	79.94901	76.95605	82.58661	-985.81834	617.19825	2463.91927	1
O	81.58439	77.26184	81.37345	293.29899	-391.65602	-477.80926	1
H	81.07184	78.08953	81.47095	2528.72714	-681.76262	-751.27122	1
C	81.49771	75.47314	83.18454	252.47363	-605.14473	440.05134	1
H	81.97845	76.11157	83.96111	-2052.65063	1456.49051	-1043.51830	1
H	80.80176	74.77738	83.71052	-2152.98800	-1661.97574	-161.70652	1
H	82.30143	74.85023	82.72752	-473.12726	-1584.27339	2165.61725	1
C	79.13956	74.22781	82.19520	360.36307	488.46713	505.10056	1
O	79.36290	73.03676	82.17018	-200.08817	-528.03552	-385.82850	1
N	78.26552	74.89463	82.93010	-597.54095	-226.44775	355.20479	1
H	78.13393	75.92954	82.80826	2179.23655	-1300.70981	-991.33835	1
C	77.63814	74.18523	83.97748	-687.68921	297.39807	-249.45179	1
H	78.01949	73.13931	83.90083	-349.16256	-2467.37713	-1101.72831	1
C	77.98175	74.88975	85.37914	672.47345	361.54328	-201.62880	1
H	77.91374	75.99788	85.29561	1541.41502	582.18425	2169.97026	1
H	77.22188	74.57145	86.13090	-2534.67717	60.25153	997.72295	1
C	79.38100	74.46003	85.83725	574.26774	-389.43119	-377.02542	1
C	79.62264	73.08361	86.13119	688.82679	-287.33580	-257.96133	1
H	78.81298	72.33766	86.04222	331.14182	1470.38163	2269.80013	1
C	80.42985	75.41839	85.98073	-383.52382	-675.04808	144.24917	1
H	80.24917	76.48650	85.76390	4.09847	1517.59662	2262.86397	1
C	80.89994	72.69669	86.53286	-134.76730	428.45285	-649.50303	1
H	81.08429	71.63052	86.75275	-903.23433	1600.49020	2011.53387	1
C	81.69267	74.98225	86.38996	-335.53281	-615.00040	-364.39058	1
H	82.49637	75.73222	86.49215	1596.45398	-1369.33862	1732.02659	1
C	81.98008	73.61557	86.67562	585.48547	-340.24194	-406.23938	1
O	83.25979	73.15978	87.08612	-419.15649	-173.04408	-511.94141	1
H	83.74674	74.01711	87.09777	-1071.84350	1559.71507	-1960.13012	1
C	76.00778	74.30580	83.92592	291.41878	-183.13091	710.72246	1
O	75.32824	73.83348	84.81011	379.87695	523.47396	-222.23622	1
N	75.53937	74.90017	82.77748	564.30591	373.09380	277.24371	1
H	76.42287	75.17253	82.32751	-120.59931	-2436.07824	-1214.35098	1

```
C   74.59501  75.97758  83.04551      404.07514     -299.37480      608.84009          1
H   74.96563  76.50363  83.95079    -1071.07882     -841.69025     2359.66467          1
C   74.66807  76.81360  81.72108     -683.12807       68.71770     -390.13096          1
H   75.64932  77.34036  81.63850     2068.88918     1596.58590      770.89603          1
H   74.63649  76.10301  80.86474     1379.86429     2342.49779     -179.85638          1
C   73.47541  77.81565  81.58652     -176.39967      537.49466      550.97268          1
H   72.50083  77.32171  81.79896     -396.28051     1942.22168     1869.33314          1
H   73.55299  78.66611  82.29895      -94.44700      586.00678    -2659.20016          1
C   73.58073  78.27852  80.06106     -291.69381     -718.18079     -150.72929          1
H   74.48796  78.88255  79.80651    -2303.38776     1089.76242      964.62032          1
H   73.51696  77.44740  79.31636      884.16729     2568.80533     -207.74283          1
N   72.45154  79.06083  79.79145     -594.49522     -252.81478      342.29276          1
H   71.77066  79.16632  80.52645    -1735.17426     2084.05705     -263.71605          1
C   72.24010  79.61580  78.66639      595.23177     -271.52487      442.22352          1
N   73.20284  79.44815  77.65939      585.53813     -424.79750     -105.81303          1
H   74.09354  78.95359  77.66843      959.88797     1327.56823    -2177.11905          1
H   73.12812  79.86577  76.73180     1578.48647       16.27378    -2220.76233          1
N   71.03212  80.32011  78.62147     -452.17763     -390.33541     -421.51853          1
H   70.30493  80.40934  79.33653      470.26107     1974.25629    -1817.92087          1
H   70.69275  80.74084  77.72106     1856.67307    -1573.97321    -1224.35416          1
C   73.08624  75.40054  83.22947     -531.47271     -581.45780      -55.06206          1
O   72.33918  75.87340  84.05440     -243.00837      192.73507      609.52485          1
N   72.78152  74.57905  82.20517      343.49288     -644.25607       38.09164          1
H   73.62189  74.32841  81.68204    -1691.16043    -1535.97525    -1484.73099          1
C   71.76155  73.58266  82.41755      542.66492      350.14337      454.42563          1
H   71.64392  73.48682  83.51967     1209.57979    -2396.95920      463.86744          1
C   70.46014  74.08482  81.66414     -737.14309      145.93010      242.71942          1
H   70.72430  74.39760  80.63063    -1050.18303      159.36419    -2509.06125          1
C   69.43010  72.90344  81.64314     -706.77696     -252.88343     -245.16230          1
H   68.49841  73.17722  81.09442    -1624.64039    -1665.49085     1417.86935          1
H   69.86394  72.00517  81.13848     2566.10521      598.51109      693.22305          1
H   69.13303  72.59610  82.67330    -1972.69758      107.10018    -1876.34312          1
C   69.82797  75.28184  82.45411      412.85148      672.58832      -27.69222          1
H   68.96045  75.72496  81.91077     1447.02277     1762.76533    -1490.78944          1
H   69.46724  74.96398  83.46030     1320.77379     -145.20455     2378.68491          1
H   70.57610  76.09555  82.61789    -1512.10848     1470.52031     1724.75147          1
C   72.34081  72.28483  81.64624     -607.11113      499.56689       73.73769          1
O   72.13033  72.12626  80.42893      357.97126      460.81534      356.68785          1
N   73.05292  71.53916  82.44964      536.55407     -368.62270      332.76306          1
H   73.13617  71.78669  83.43693      524.38866     2297.69985     1367.20941          1
C   73.81002  70.48366  81.86096       46.50277     -774.29190     -147.97904          1
H   74.17811  70.92219  80.90778     1676.51875     1883.29066     1032.55549          1
C   74.95924  70.01208  82.88372     -559.41456      -34.19198     -556.30433          1
H   74.48826  69.70249  83.84776      -64.66664      395.18769     2695.05373          1
H   75.47576  69.10358  82.48910    -2703.44278      -60.40056      333.79418          1
C   75.94976  71.12649  83.11392     -519.76435      377.31045      459.42366          1
H   75.39278  72.05722  83.37488    -1746.74323     1667.50644    -1261.73691          1
C   76.86559  70.77032  84.27835      570.95554       16.46261     -545.27700          1
H   77.65706  71.54830  84.43577     2391.58273       28.05277    -1305.07271          1
H   76.29493  70.67767  85.23875      175.75588      917.63458     2559.43867          1
H   77.39026  69.79501  84.10733    -1063.97851    -2155.34650     1283.00462          1
C   76.77751  71.38396  81.86472      193.76014        2.62946     -765.53141          1
H   77.60428  72.14100  82.05104      507.39394     -846.55087     2539.60089          1
H   77.27426  70.44709  81.50216     1337.57841     1818.19054     1526.01994          1
H   76.15023  71.77478  81.02265     1100.88635    -1172.91350    -2199.08909          1
C   72.82589  69.23288  81.58650     -549.21115      475.64486     -309.38218          1
O   71.89186  68.94056  82.28497      556.06106     -390.37755      -78.24250          1
O   73.24510  68.37250  80.54269     -572.27940     -310.19032     -209.75862          1
H   74.02261  68.90032  80.20973    -1380.08193     2212.14486      790.85944          1
```

Listing 57: C00057.xmolout

```
    276
150.0000  150.0000  150.0000     0.0000     0.0000     0.0000***********    2.7750    11100
     0.2500     300.00    0.00    0.00    0.00  -1293.6500       0.0000     300.0000
H    72.99638  72.88041  69.49698    -2043.14101    -1757.64893       399.89339          1
N    73.08255  73.87711  69.31273      426.75047      490.45733      -334.42467          1
H    72.67322  74.27735  70.15403      442.25559    -1110.30854     -2448.52907          1
C    74.37967  74.32038  68.98704      415.32972     -666.39213        83.73172          1
H    74.92774  73.40416  68.68160      879.32219     2491.00447       667.35213          1
C    74.30040  75.46107  67.84977       31.35357      477.41777       628.23362          1
H    73.70777  76.32292  68.24073     1168.38489     2240.25337     -1019.71136          1
H    75.31825  75.87210  67.64045     -360.43770     2534.90498       931.67112          1
C    73.66897  74.91033  66.59380      463.18208      512.70242      -382.34388          1
H    72.83091  74.23939  66.88853     1725.29117     1863.52247      -987.07854          1
C    73.10251  76.04018  65.74442      710.42801     -144.27427      -313.15367          1
H    72.63175  75.65500  64.80300      364.20195    -1033.13725      2494.72524          1
H    72.31719  76.61964  66.29595     2567.61904      335.80679       847.48802          1
H    73.89982  76.76789  65.44265    -2587.99029      680.92867      -512.16411          1
C    74.68177  74.11349  65.78403      268.17616      741.64379       -40.42634          1
H    74.22509  73.68719  64.85342     1283.54314    -1945.37420     -1411.27812          1
H    75.54621  74.75251  65.46553    -1258.57796     1317.15249     -2026.02085          1
H    75.10195  73.25737  66.37251    -2502.84526      909.80597       575.92391          1
C    74.98968  75.08688  70.28250      619.36578     -458.64843      -172.09273          1
O    75.03399  76.31434  70.22811      -81.40433      433.87611       522.34594          1
N    75.36273  74.28218  71.25178      175.23996     -622.20190       341.55523          1
H    75.19525  73.28294  71.13537     -876.57256     -303.22879     -2561.90213          1
C    75.82285  74.82645  72.48833     -571.88214     -534.10783      -106.15033          1
H    75.09072  75.61973  72.77556    -2713.20841      -16.25800      -248.80809          1
C    75.96830  73.63589  73.52693      166.60354      288.16399      -716.09568          1
H    76.50947  72.78342  73.02160    -2097.85574     1636.78449      -586.18044          1
O    74.72335  72.93600  73.87880     -317.80580     -363.38227       484.43082          1
H    74.66398  73.13342  74.83141     1863.74748    -1451.84923      1357.29467          1
C    76.83586  74.24588  74.67582      503.80731      517.99032       318.51606          1
H    77.06649  73.49047  75.46389      640.61020    -2157.10410     -1536.29104          1
H    77.80830  74.63760  74.29220     1687.88455      465.60692      2087.56452          1
H    76.31790  75.09998  75.17310    -1293.07895    -1899.88581     -1463.57452          1
C    77.04728  75.91377  72.30897     -153.07550     -733.68902       248.70981          1
O    76.99177  76.85362  73.05792      186.15459     -480.78787      -449.34387          1
N    78.01220  75.58668  71.44355     -487.53139      543.18517        42.03850          1
C    79.00919  76.54750  71.07023     -699.35871       69.34393       360.10701          1
H    79.25465  77.09527  72.00939        2.81126     -221.99807     -2715.58072          1
C    80.15399  75.65144  70.44352     -507.46439     -592.59194       122.07761          1
H    80.75674  76.20809  69.68861    -1975.08116      525.87560     -1801.71556          1
H    80.86357  75.28641  71.21976     1588.27743    -1913.22100      1113.83566          1
C    79.33770  74.47456  69.81820     -613.62442     -457.55993      -194.14490          1
H    78.88585  74.78218  68.84849     1707.54637     -721.27992      1996.92523          1
H    79.94768  73.56638  69.61186      758.23357     2100.81767      1560.55011          1
C    78.22099  74.24195  70.92214     -352.41524      704.96076        49.21465          1
H    77.24921  73.85758  70.53803     1714.76886    -2070.42778       443.35792          1
H    78.54227  73.62228  71.79263     1224.15040     1801.50881      1636.97633          1
C    78.45670  77.54322  69.89020      566.85117      537.05831       117.62814          1
O    79.03128  77.52988  68.82463     -550.26779     -406.08867         4.21666          1
N    77.45574  78.32968  70.27062      566.34832     -425.77725       180.18749          1
H    77.11260  78.22100  71.22695      644.44502     2646.80550       -52.73430          1
C    77.10363  79.40504  69.37764      612.34801     -281.48662       411.56221          1
H    77.34770  78.96655  68.37917     1667.23498    -1490.75142      1556.16753          1
C    75.63927  79.86521  69.55529     -714.45746     -175.43074       286.98927          1
H    75.46174  80.39421  70.52452     -787.09484     1763.03619     -1922.46060          1
O    74.36752  78.80285  69.89674     -429.46514      516.51932      -128.40526          1
H    74.96388  78.00233  69.96208     1981.82785      436.16847     -1818.18182          1
C    75.25059  80.69898  68.31937      630.79189     -468.94448        76.03230          1
H    74.22492  81.12937  68.43672    -1775.71861     2062.30353       131.89849          1
```

H	75.95318	81.55996	68.13652	-1855.14608	88.94118	1993.53760	1
H	75.24846	80.07304	67.39231	-1504.82350	-780.94249	-2132.90992	1
C	77.99712	80.77298	69.69974	288.57638	618.62814	-397.00270	1
O	77.38712	81.84711	69.58708	321.70495	-185.26452	574.37218	1
N	79.24608	80.54781	69.98704	-447.05792	-446.53431	367.76425	1
H	79.35413	79.52767	69.99369	350.31316	1975.63644	-1843.31522	1
C	80.36026	81.40685	70.13566	-511.23061	374.03917	-471.51480	1
H	81.16031	80.68116	70.40531	2234.79579	-898.70890	-1273.45190	1
C	80.63341	82.08080	68.70603	529.53781	-159.34528	563.72604	1
H	81.65388	82.55252	68.70790	654.59388	2347.94251	1217.51488	1
H	80.67251	81.23808	67.96682	-1526.55661	2103.42026	817.87394	1
O	79.70581	83.10541	68.26060	-554.84278	14.12789	399.58769	1
H	78.86542	82.60980	68.34756	1297.06236	2061.84854	1220.68812	1
C	80.22180	82.56810	71.26413	-662.15484	-412.13544	123.62687	1
O	81.17094	82.81196	71.98295	-540.01537	-317.05804	274.91456	1
N	79.07590	83.20764	71.16599	-202.76299	-690.00100	-131.49145	1
H	78.37126	82.87569	70.45028	-702.62132	-2617.37191	281.70413	1
C	78.62854	84.19753	72.14608	-491.73914	576.34187	222.73509	1
H	79.51102	84.87072	72.26330	1977.17978	-1823.58850	-434.68928	1
C	77.39594	84.83742	71.53762	-649.50797	-328.66843	306.11224	1
H	76.65831	84.01992	71.36181	2075.07803	1452.16490	-1004.45904	1
C	76.74791	85.85573	72.45688	-615.18735	-489.99689	-70.96352	1
H	75.81716	86.29163	71.99893	-1226.32661	-1724.38906	-1716.47170	1
H	76.44894	85.39257	73.44002	2381.64273	-969.84735	900.46860	1
H	77.44047	86.71177	72.68528	2122.74991	1702.23946	-141.36228	1
C	77.69088	85.47567	70.19151	-501.86074	-479.61276	-376.42482	1
H	76.75184	85.82925	69.68363	993.79261	-2246.95401	-1177.81305	1
H	78.36970	86.36572	70.29574	42.71279	-626.15391	-2651.37258	1
H	78.19953	84.75513	69.49177	-2026.39012	-1590.33709	-887.82922	1
C	78.28084	83.37800	73.55396	-672.89153	382.57394	-156.34072	1
O	77.13886	83.30691	73.93192	410.04627	221.86223	500.35950	1
N	79.38468	82.90363	74.07742	-378.11782	-443.79414	-441.10984	1
H	80.27452	83.02995	73.55561	2404.15973	-568.14894	1149.30085	1
C	79.44832	82.33341	75.39576	-544.54484	466.16019	331.29119	1
H	79.98469	83.09332	76.00364	-1409.80988	-170.17955	2325.32679	1
C	78.00407	82.01098	75.93849	-184.48284	116.78282	-758.89137	1
H	77.40740	82.94520	76.07882	1893.78641	1861.14652	611.04528	1
H	77.44602	81.41481	75.17823	-2170.79665	-974.19311	1327.50117	1
C	78.14337	81.25549	77.30212	389.51668	672.81878	-138.49152	1
H	78.76386	80.34094	77.14808	510.64577	1933.03327	1851.02484	1
H	78.70670	81.87578	78.03740	-2302.30869	-1105.72939	-948.89774	1
C	76.74332	80.85010	77.92337	-559.93427	-126.34590	542.30857	1
O	76.54250	80.85267	79.11202	-670.36003	-111.83166	76.36448	1
N	75.87745	80.42869	76.96275	-216.06729	-537.07037	446.51379	1
H	76.22958	80.22063	76.02026	-1251.96487	1074.12894	-2168.52500	1
H	75.05946	79.78377	77.15802	-2451.83023	-720.73851	944.84612	1
C	80.32961	80.97175	75.21704	546.24052	-563.88337	-85.11809	1
O	79.98668	80.14724	74.41032	71.70702	-307.40463	606.69673	1
N	81.30387	80.90234	76.13232	479.90863	-504.50129	222.86849	1
C	82.48550	80.11390	75.90610	75.51268	-600.91904	506.73700	1
H	82.86762	80.44010	74.91210	-2033.61562	1795.30103	254.89480	1
C	83.42092	80.50100	77.12620	523.79067	517.99274	284.45581	1
H	84.05134	79.64870	77.46590	1599.61665	-1127.00911	1895.98173	1
H	84.12647	81.31490	76.84664	-1881.83093	1275.82488	-1501.55016	1
C	82.41681	80.98450	78.21878	-600.05639	-404.05026	316.64485	1
H	81.95117	80.12668	78.75357	-2597.05260	496.36221	657.73249	1
H	82.87865	81.63262	78.99700	-1666.24518	1294.38985	-1723.90591	1
C	81.35901	81.75210	77.31086	493.57570	-129.95376	-602.56394	1
H	80.33354	81.84940	77.74290	-1469.08415	-637.84797	2204.22581	1
H	81.71103	82.75072	76.95688	-1038.97840	1068.88701	-2280.71789	1
C	82.13910	78.52159	75.87656	188.88100	241.05089	-727.87821	1
O	82.79713	77.80174	75.16196	531.88613	-49.19943	427.07960	1

N	81.24655	78.20072	76.80280	708.14833	26.93417	-179.73509	1
H	80.81169	78.94554	77.38576	-2232.69501	-1179.09831	1023.94816	1
C	80.36849	77.08622	76.55438	-738.32412	259.92548	104.42456	1
H	80.22302	76.98646	75.45606	2028.89123	1260.02983	1311.33324	1
C	81.05920	75.80702	77.19905	-258.65678	-345.52513	661.28459	1
H	81.66559	76.11818	78.07677	-1826.06090	-1801.46097	-918.64554	1
C	79.99902	74.72693	77.60447	-541.23400	531.92970	-218.41428	1
H	80.48382	73.81803	78.03200	1311.73218	-2219.18200	882.19023	1
H	79.29195	75.11817	78.37296	-1048.71298	1223.58118	-2196.97971	1
H	79.39073	74.39796	76.72958	419.06414	1903.92898	1903.44670	1
C	81.95804	75.12726	76.11101	-423.86431	152.66566	-648.55262	1
H	82.62785	74.35094	76.55131	105.68386	-2342.50080	1387.51238	1
H	81.34439	74.62929	75.32356	-1695.63603	-1484.73621	-1531.02793	1
H	82.60884	75.89437	75.59219	-1853.57121	-487.39031	1936.59343	1
C	79.07225	77.57421	77.39299	539.71428	-510.58232	267.58633	1
O	79.22506	78.48794	78.19881	-15.93758	-278.34085	-624.49402	1
N	77.95788	76.95203	77.02333	-676.81886	12.05292	276.17960	1
H	78.12509	76.24152	76.30698	-1694.44250	-373.84189	-2100.66098	1
C	76.92635	76.64273	77.98709	412.33078	-664.70360	108.35451	1
H	77.05840	75.55705	78.19394	1765.77476	1294.44366	1621.76594	1
C	77.05112	77.55974	79.25893	-179.66635	-721.95667	-264.73915	1
H	78.01746	77.38290	79.78731	-607.34922	2587.87032	-598.10018	1
H	77.07235	78.63567	78.95178	-359.69332	-1479.13954	-2259.74260	1
C	75.84028	77.26467	80.21007	-240.01076	616.02425	431.85322	1
H	74.87396	77.34537	79.66282	-1359.17131	782.54425	2227.99181	1
H	75.88450	76.22702	80.61023	-820.36667	85.81767	-2596.78715	1
C	75.91864	78.33513	81.36968	-513.07015	236.18570	-551.87280	1
H	76.91485	78.31030	81.86854	1816.11944	-1183.11722	-1650.94321	1
H	75.81188	79.36775	80.95865	-1800.04429	-1361.53473	1526.34643	1
C	74.79190	78.01141	82.37967	726.08972	-253.19732	-179.64713	1
H	73.76120	78.12386	81.98151	1224.27271	-456.51798	-2390.90304	1
H	74.89621	77.03223	82.88798	1206.40339	-1150.93514	-2154.90344	1
N	74.87112	79.06400	83.53196	329.70796	-633.68968	-155.67358	1
H	74.10874	78.97695	84.26440	1348.31305	1938.76845	1359.00643	1
H	75.74730	79.08505	84.13149	2096.92035	1446.54551	-966.48890	1
H	74.76872	80.08026	83.24693	-1197.07217	-2303.53719	827.28733	1
C	75.50739	76.95290	77.25596	-378.01186	-556.17301	-413.96481	1
O	75.05947	78.08892	77.11351	-409.44433	-307.55053	-453.30833	1
N	74.95342	75.81351	76.92473	-429.02477	524.70884	274.08502	1
H	75.38260	74.90120	77.09528	1706.34263	-1425.96036	1574.38926	1
C	73.64003	75.84975	76.35905	-45.25864	-626.22952	478.93267	1
H	73.11758	76.73949	76.78156	1988.63164	570.90773	1772.87287	1
C	73.85865	75.95781	74.79093	551.77128	-20.49526	-564.54996	1
H	74.60936	76.75170	74.57257	1434.61535	-1753.10253	1513.99471	1
H	74.29479	75.00752	74.40535	-714.67738	1819.09770	1898.36494	1
C	72.50857	76.25623	74.06108	446.99263	-555.77305	338.97221	1
H	71.79439	75.40294	74.16737	1953.68466	1892.25957	-161.67766	1
H	72.00621	77.15208	74.49071	-403.03785	2688.07032	-188.43591	1
C	72.91357	76.49307	72.55432	567.86262	335.74681	434.04421	1
H	73.77161	77.20119	72.48534	-1080.02275	1568.26081	-1948.78900	1
H	73.27605	75.54234	72.10074	-919.54108	2111.34692	1456.13463	1
C	71.72315	77.05537	71.72541	195.81897	173.33862	-745.11536	1
H	70.86695	76.35582	71.61696	1405.67949	-2301.21089	-390.08113	1
H	71.35955	78.05418	72.04808	-940.37328	-1118.32446	2299.72133	1
N	72.28606	77.25295	70.31883	319.26422	656.36007	-42.02424	1
H	71.58438	77.46341	69.56423	1961.03633	619.91968	1787.09422	1
H	73.12886	78.08203	70.17588	854.14343	-2156.80752	1429.08711	1
H	72.73669	76.38568	69.92870	1237.02905	-1378.50857	-1998.28500	1
C	73.05675	74.37980	76.76504	532.53319	-268.57236	517.55729	1
O	73.81971	73.42046	76.87753	266.16714	-290.73804	-558.88002	1
N	71.75215	74.45266	76.93327	-226.25352	640.49678	-270.32839	1
H	71.36816	75.38512	76.77545	2007.69063	-1035.27229	1523.50231	1

C	70.88279	73.33225	76.85550	-540.76542	-573.33303	49.50000	1
H	69.88536	73.79322	76.68191	-2374.20950	-1036.76023	843.75774	1
C	71.36265	72.44577	75.61358	-77.09082	666.87422	-415.84136	1
H	72.36866	71.99387	75.84688	-985.81834	617.19825	2463.91927	1
O	71.52586	73.14271	74.36408	293.29899	-391.65602	-477.80926	1
H	72.48972	73.01676	74.20387	2528.72714	-681.76262	-751.27122	1
C	70.26573	71.32136	75.63843	252.47363	-605.14473	440.05134	1
H	70.47507	70.54299	74.87030	-2052.65063	1456.49051	-1043.51830	1
H	70.21564	70.80906	76.63118	-2152.98800	-1661.97574	-161.70652	1
H	69.25155	71.73341	75.42689	-473.12726	-1584.27339	2165.61725	1
C	70.90362	72.48454	78.26172	360.36307	488.46713	505.10056	1
O	69.88488	71.93169	78.59479	-200.08817	-528.03552	-385.82850	1
N	72.10499	72.46479	78.81290	-597.54095	-226.44775	355.20479	1
H	72.88079	72.95291	78.31863	2179.23655	-1300.70981	-991.33835	1
C	72.37283	71.85276	80.05694	-687.68921	297.39807	-249.45179	1
H	73.36506	72.26206	80.34913	-349.16256	-2467.37713	-1101.72831	1
C	71.22226	72.16640	81.12505	672.47345	361.54328	-201.62880	1
H	70.21680	71.92346	80.69563	1541.41502	582.18425	2169.97026	1
H	71.36597	71.51679	82.02019	-2534.67717	60.25153	997.72295	1
C	71.31401	73.64858	81.51390	574.26774	-389.43119	-377.02542	1
C	72.37319	74.08370	82.36589	688.82679	-287.33580	-257.96133	1
H	73.12193	73.36581	82.74709	331.14182	1470.38163	2269.80013	1
C	70.35223	74.57963	81.01929	-383.52382	-675.04808	144.24917	1
H	69.53743	74.24341	80.35226	4.09847	1517.59662	2262.86397	1
C	72.43854	75.43331	82.71154	-134.76730	428.45285	-649.50303	1
H	73.25055	75.76597	83.38041	-903.23433	1600.49020	2011.53387	1
C	70.45947	75.92073	81.39751	-335.53281	-615.00040	-364.39058	1
H	69.70477	76.63162	81.01820	1596.45398	-1369.33862	1732.02659	1
C	71.49718	76.39852	82.24971	585.48547	-340.24194	-406.23938	1
O	71.61645	77.76252	82.63275	-419.15649	-173.04408	-511.94141	1
H	70.80818	78.12517	82.19956	-1071.84350	1559.71507	-1960.13012	1
C	72.40104	70.22172	79.94112	291.41878	-183.13091	710.72246	1
O	72.52236	69.55823	80.95132	379.87695	523.47396	-222.23622	1
N	72.27639	69.82269	78.69149	564.30591	373.09380	277.24371	1
H	72.21982	70.55169	77.98321	-120.59931	-2436.07824	-1214.35098	1
C	72.40380	68.44919	78.28363	404.07514	-299.37480	608.84009	1
H	73.48729	68.26440	78.45181	-1071.07882	-841.69025	2359.66467	1
C	71.51681	67.54564	79.22646	-683.12807	68.71770	-390.13096	1
H	71.66781	67.85489	80.29751	2068.88918	1596.58590	770.89603	1
H	70.43382	67.70938	79.02012	1379.86429	2342.49779	-179.85638	1
C	71.90272	66.04377	79.04351	-176.39967	537.49466	550.97268	1
H	71.78639	65.71143	77.98816	-396.28051	1942.22168	1869.33314	1
H	72.97055	65.86437	79.29776	-94.44700	586.00678	-2659.20016	1
C	70.92683	65.23507	80.02156	-291.69381	-718.18079	-150.72929	1
H	71.01628	65.47293	81.11116	-2303.38776	1089.76242	964.62032	1
H	69.85165	65.30146	79.71219	884.16729	2568.80533	-207.74283	1
N	71.34407	63.91825	79.80339	-594.49522	-252.81478	342.29276	1
H	71.64485	63.70092	78.86455	-1735.17426	2084.05705	-263.71605	1
C	71.32950	62.98059	80.66399	595.23177	-271.52487	442.22352	1
N	70.88239	63.26270	81.95741	585.53813	-424.79750	-105.81303	1
H	70.51956	64.13199	82.34646	959.88797	1327.56823	-2177.11905	1
H	70.80942	62.57865	82.71153	1578.48647	16.27378	-2220.76233	1
N	71.79011	61.78508	80.09609	-452.17763	-390.33541	-421.51853	1
H	72.07474	61.57110	79.14137	470.26107	1974.25629	-1817.92087	1
H	71.82207	60.89933	80.60205	1856.67307	-1573.97321	-1224.35416	1
C	72.01170	68.51287	76.70698	-531.47271	-581.45780	-55.06206	1
O	72.59345	69.28603	75.97814	-243.00837	192.73507	609.52485	1
N	71.09572	67.60315	76.41880	343.49288	-644.25607	38.09164	1
H	70.62809	67.06017	77.17098	-1691.16043	-1535.97525	-1484.73099	1
C	70.36070	67.61906	75.18227	542.66492	350.14337	454.42563	1
H	69.99778	68.66481	75.09784	1209.57979	-2396.95920	463.86744	1
C	71.35435	67.29314	73.98887	-737.14309	145.93010	242.71942	1

```
H    72.24777  66.77254  74.39510    -1050.18303      159.36419    -2509.06125          1
C    70.75783  66.47822  72.78664     -706.77696     -252.88343     -245.16230          1
H    71.50565  66.35638  71.96760    -1624.64039    -1665.49085     1417.86935          1
H    70.44981  65.45126  73.10189     2566.10521      598.51109      693.22305          1
H    69.86869  66.98656  72.34118    -1972.69758      107.10018    -1876.34312          1
C    71.74873  68.65417  73.31803      412.85148      672.58832      -27.69222          1
H    72.44557  68.50583  72.45969     1447.02277     1762.76533    -1490.78944          1
H    70.85608  69.19710  72.92623     1320.77379     -145.20455     2378.68491          1
H    72.26653  69.33740  74.05922    -1512.10848     1470.52031     1724.75147          1
C    69.19147  66.58597  75.60377     -607.11113      499.56689       73.73769          1
O    68.94431  66.44342  76.79809      357.97126      460.81534      356.68785          1
N    68.54608  66.05400  74.56885      536.55407     -368.62270      332.76306          1
H    68.89442  66.25423  73.63035      524.38866     2297.69985     1367.20941          1
C    67.83937  64.83340  74.81300       46.50277     -774.29190     -147.97904          1
H    67.10097  65.11264  75.59498     1676.51875     1883.29066     1032.55549          1
C    67.20667  64.29742  73.43904     -559.41456      -34.19198     -556.30433          1
H    68.03429  63.87306  72.81976      -64.66664      395.18769     2695.05373          1
H    66.51826  63.44181  73.64317    -2703.44278      -60.40056      333.79418          1
C    66.50017  65.38486  72.66304     -519.76435      377.31045      459.42366          1
H    67.14128  66.29398  72.65625    -1746.74323     1667.50644    -1261.73691          1
C    66.29521  64.93749  71.22144      570.95554       16.46261     -545.27700          1
H    65.79770  65.73115  70.60667     2391.58273       28.05277    -1305.07271          1
H    67.26868  64.69185  70.72287      175.75588      917.63458     2559.43867          1
H    65.65361  64.02068  71.16257    -1063.97851    -2155.34650     1283.00462          1
C    65.16529  65.74397  73.29814      193.76014        2.62946     -765.53141          1
H    64.63739  66.54760  72.72167      507.39394     -846.55087     2539.60089          1
H    64.47734  64.86015  73.34078     1337.57841     1818.19054     1526.01994          1
H    65.29543  66.11691  74.34719     1100.88635    -1172.91350    -2199.08909          1
C    68.88339  63.72134  75.33104     -549.21115      475.64486     -309.38218          1
O    70.02805  63.65530  74.96586      556.06106     -390.37755      -78.24250          1
O    68.31859  62.68566  76.11587     -572.27940     -310.19032     -209.75862          1
H    67.39595  63.04836  76.21122    -1380.08193     2212.14486      790.85944          1
```

Listing 58: C00058.xmolout

```
       276
  150.0000  150.0000  150.0000    0.0000    0.0000    0.0000***********    2.7750    11100
      0.2500    300.00    0.00    0.00    0.00  -1293.6480      0.0000    300.0000
H    74.65997  81.04377  73.22073    -2043.14101    -1757.64893      399.89339          1
N    74.74850  81.53136  72.33032      426.75047      490.45733     -334.42467          1
H    73.77435  81.78300  72.16680      442.25559    -1110.30854    -2448.52907          1
C    75.58823  82.67195  72.42155      415.32972     -666.39213       83.73172          1
H    75.19848  83.24987  73.28718      879.32219     2491.00447      667.35213          1
C    77.10207  82.17923  72.67998       31.35357      477.41777      628.23362          1
H    77.36797  81.40843  71.92053     1168.38489     2240.25337    -1019.71136          1
H    77.80926  83.02867  72.52084     -360.43770     2534.90498      931.67112          1
C    77.27797  81.60214  74.06006      463.18208      512.70242     -382.34388          1
H    76.40469  80.95371  74.29364     1725.29117     1863.52247     -987.07854          1
C    78.53449  80.73999  74.10510      710.42801     -144.27427     -313.15367          1
H    78.69892  80.30234  75.12377      364.20195    -1033.13725     2494.72524          1
H    78.47739  79.88691  73.38040     2567.61904      335.80679      847.48802          1
H    79.44971  81.33258  73.84702    -2587.99029      680.92867     -512.16411          1
C    77.37785  82.69395  75.11627      268.17616      741.64379      -40.42634          1
H    77.52085  82.26335  76.14200     1283.54314    -1945.37420    -1411.27812          1
H    78.24529  83.37619  74.92056    -1258.57796     1317.15249    -2026.02085          1
H    76.45676  83.33093  75.14652    -2502.84526      909.80597      575.92391          1
C    75.51182  83.52692  71.05056      619.36578     -458.64843     -172.09273          1
O    75.90072  84.70183  71.09586      -81.40433      433.87611      522.34594          1
N    75.09274  82.83297  70.02403      175.23996     -622.20190      341.55523          1
H    74.77158  81.89417  70.25767     -876.57256     -303.22879    -2561.90213          1
C    75.09280  83.25959  68.66036     -571.88214     -534.10783     -106.15033          1
H    74.02949  83.09631  68.37775    -2713.20841      -16.25800     -248.80809          1
```

C	75.53459	84.78678	68.51340	166.60354	288.16399	-716.09568	1
H	76.55228	84.90691	68.98694	-2097.85574	1636.78449	-586.18044	1
O	74.68064	85.72009	69.17408	-317.80580	-363.38227	484.43082	1
H	75.15105	85.73902	70.09464	1863.74748	-1451.84923	1357.29467	1
C	75.68625	84.96217	66.95685	503.80731	517.99032	318.51606	1
H	75.76636	86.03852	66.67468	640.61020	-2157.10410	-1536.29104	1
H	76.60019	84.45191	66.56807	1687.88455	465.60692	2087.56452	1
H	74.81338	84.53288	66.40856	-1293.07895	-1899.88581	-1463.57452	1
C	76.00860	82.32826	67.68649	-153.07550	-733.68902	248.70981	1
O	75.66920	82.36817	66.52776	186.15459	-480.78787	-449.34387	1
N	77.07069	81.70230	68.21003	-487.53139	543.18517	42.03850	1
C	78.00836	80.99217	67.38709	-699.35871	69.34393	360.10701	1
H	78.07950	81.54750	66.42301	2.81126	-221.99807	-2715.58072	1
C	79.32256	81.01683	68.28850	-507.46439	-592.59194	122.07761	1
H	80.05163	80.22526	68.00481	-1975.08116	525.87560	-1801.71556	1
H	79.85650	81.98939	68.20598	1588.27743	-1913.22100	1113.83566	1
C	78.72670	80.82088	69.71561	-613.62442	-457.55993	-194.14490	1
H	78.48640	79.75066	69.90559	1707.54637	-721.27992	1996.92523	1
H	79.40993	81.14055	70.53483	758.23357	2100.81767	1560.55011	1
C	77.40403	81.69973	69.62641	-352.41524	704.96076	49.21465	1
H	76.52729	81.27241	70.16622	1714.76886	-2070.42778	443.35792	1
H	77.52647	82.77164	69.91266	1224.15040	1801.50881	1636.97633	1
C	77.56034	79.44627	67.17268	566.85117	537.05831	117.62814	1
O	78.36468	78.65588	66.69176	-550.26779	-406.08867	4.21666	1
N	76.35871	79.23653	67.62702	566.34832	-425.77725	180.18749	1
H	75.80771	80.02156	68.03494	644.44502	2646.80550	-52.73430	1
C	75.56691	78.08300	67.29508	612.34801	-281.48662	411.56221	1
H	75.51845	78.05198	66.18350	1667.23498	-1490.75142	1556.16753	1
C	76.37115	76.80619	67.82601	-714.45746	-175.43074	286.98927	1
H	77.02596	77.19797	68.65452	-787.09484	1763.03619	-1922.46060	1
O	77.28763	76.27428	66.86113	-429.46514	516.51932	-128.40526	1
H	77.88960	77.10024	66.71234	1981.82785	436.16847	-1818.18182	1
C	75.35761	75.77337	68.42367	630.79189	-468.94448	76.03230	1
H	75.89140	74.95458	68.96223	-1775.71861	2062.30353	131.89849	1
H	74.65762	76.25168	69.14866	-1855.14608	88.94118	1993.53760	1
H	74.74351	75.29011	67.62727	-1504.82350	-780.94249	-2132.90992	1
C	74.17764	78.52382	67.97772	288.57638	618.62814	-397.00270	1
O	74.09061	79.66949	68.46991	321.70495	-185.26452	574.37218	1
N	73.25970	77.59699	67.90987	-447.05792	-446.53431	367.76425	1
H	73.51046	76.72287	67.45333	350.31316	1975.63644	-1843.31522	1
C	71.93477	77.75619	68.41982	-511.23061	374.03917	-471.51480	1
H	71.61956	78.73778	68.00012	2234.79579	-898.70890	-1273.45190	1
C	71.12755	76.48233	67.88445	529.53781	-159.34528	563.72604	1
H	70.04731	76.66344	68.12468	654.59388	2347.94251	1217.51488	1
H	71.21249	76.45000	66.76570	-1526.55661	2103.42026	817.87394	1
O	71.57072	75.19657	68.40541	-554.84278	14.12789	399.58769	1
H	71.22482	75.28068	69.31887	1297.06236	2061.84854	1220.68812	1
C	71.84834	77.74248	70.03570	-662.15484	-412.13544	123.62687	1
O	70.95154	77.15753	70.58398	-540.01537	-317.05804	274.91456	1
N	72.82413	78.47460	70.58742	-202.76299	-690.00100	-131.49145	1
H	73.43255	79.04094	69.95178	-702.62132	-2617.37191	281.70413	1
C	72.92417	78.68341	72.02948	-491.73914	576.34187	222.73509	1
H	73.77891	79.39469	72.11059	1977.17978	-1823.58850	-434.68928	1
C	71.61649	79.28273	72.51594	-649.50797	-328.66843	306.11224	1
H	70.84210	78.48156	72.44404	2075.07803	1452.16490	-1004.45904	1
C	71.72510	79.73459	73.96026	-615.18735	-489.99689	-70.96352	1
H	70.73966	80.10003	74.35962	-1226.32661	-1724.38906	-1716.47170	1
H	72.07242	78.89018	74.62532	2381.64273	-969.84735	900.46860	1
H	72.46323	80.57533	74.07576	2122.74991	1702.23946	-141.36228	1
C	71.15442	80.43832	71.64603	-501.86074	-479.61276	-376.42482	1
H	70.17735	80.86517	72.00144	993.79261	-2246.95401	-1177.81305	1
H	71.90077	81.27773	71.63597	42.71279	-626.15391	-2651.37258	1

```
H    71.01122  80.11498  70.57872   -2026.39012   -1590.33709    -887.82922        1
C    73.25240  77.23438  72.77835    -672.89153     382.57394    -156.34072        1
O    73.16448  77.14079  73.98829     410.04627     221.86223     500.35950        1
N    73.65146  76.32702  71.91545    -378.11782    -443.79414    -441.10984        1
H    73.68101  76.65851  70.95564    2404.15973    -568.14894    1149.30085        1
C    74.00600  75.00880  72.32936    -544.54484     466.16019     331.29119        1
H    73.34008  74.79389  73.19312   -1409.80988    -170.17955    2325.32679        1
C    73.78278  74.09173  71.06325    -184.48284     116.78282    -758.89137        1
H    72.70829  74.08805  70.76877    1893.78641    1861.14652     611.04528        1
H    74.33427  74.49059  70.18521   -2170.79665    -974.19311    1327.50117        1
C    74.27338  72.64855  71.41581     389.51668     672.81878    -138.49152        1
H    75.35840  72.67642  71.67051     510.64577    1933.03327    1851.02484        1
H    73.77707  72.29520  72.37012   -2302.30869   -1105.72939    -948.89774        1
C    74.03398  71.62933  70.25616    -559.93427    -126.34590     542.30857        1
O    74.25963  70.44477  70.43479    -670.36003    -111.83166      76.36448        1
N    73.56436  72.19003  69.13317    -216.06729    -537.07037     446.51379        1
H    73.32695  73.17013  68.93082   -1251.96487    1074.12894   -2168.52500        1
H    73.33217  71.66721  68.27974   -2451.83023    -720.73851     944.84612        1
C    75.59272  75.07179  72.74041     546.24052    -563.88337     -85.11809        1
O    76.41266  75.23951  71.87731      71.70702    -307.40463     606.69673        1
N    75.76328  74.89076  74.05815     479.90863    -504.50129     222.86849        1
C    76.94327  75.38216  74.71091      75.51268    -600.91904     506.73700        1
H    77.19440  76.33230  74.18660   -2033.61562    1795.30103     254.89480        1
C    76.48781  75.54579  76.21904     523.79067     517.99274     284.45581        1
H    77.32535  75.37249  76.93209    1599.61665   -1127.00911    1895.98173        1
H    76.11217  76.57480  76.41531   -1881.83093    1275.82488   -1501.55016        1
C    75.34075  74.49622  76.36581    -600.05639    -404.05026     316.64485        1
H    75.74434  73.46790  76.50866   -2597.05260     496.36221     657.73249        1
H    74.65423  74.69179  77.22063   -1666.24518    1294.38985   -1723.90591        1
C    74.63778  74.65810  74.95256     493.57570    -129.95376    -602.56394        1
H    74.05228  73.74202  74.60260   -1469.08415    -637.84797    2204.22581        1
H    73.99791  75.59057  74.83943   -1038.97840    1068.88701   -2280.71789        1
C    78.21729  74.35827  74.68283     188.88100     241.05089    -727.87821        1
O    79.32925  74.80123  74.85218     531.88613     -49.19943     427.07960        1
N    77.85838  73.06685  74.64221     708.14833      26.93417    -179.73509        1
H    76.86181  72.95780  74.45610   -2232.69501   -1179.09831    1023.94816        1
C    78.87450  72.04446  74.39301    -738.32412     259.92548     104.42456        1
H    79.76318  72.62154  74.04176    2028.89123    1260.02983    1311.33324        1
C    79.11034  71.29883  75.69377    -258.65678    -345.52513     661.28459        1
H    78.11814  71.01839  76.11331   -1826.06090   -1801.46097    -918.64554        1
C    79.90142  70.02277  75.47410    -541.23400     531.92970    -218.41428        1
H    80.04907  69.45349  76.43339    1311.73218   -2219.18200     882.19023        1
H    79.37780  69.33334  74.75413   -1048.71298    1223.58118   -2196.97971        1
H    80.92156  70.23398  75.04929     419.06414    1903.92898    1903.44670        1
C    79.80093  72.17160  76.72573    -423.86431     152.66566    -648.55262        1
H    79.89323  71.65158  77.71956     105.68386   -2342.50080    1387.51238        1
H    80.83910  72.45669  76.39855   -1695.63603   -1484.73621   -1531.02793        1
H    79.23487  73.12985  76.89747   -1853.57121    -487.39031    1936.59343        1
C    78.22973  71.01914  73.24996     539.71428    -510.58232     267.58633        1
O    78.85092  70.75753  72.24756     -15.93758    -278.34085    -624.49402        1
N    77.09311  70.51429  73.68597    -676.81886      12.05292     276.17960        1
H    76.76546  70.86504  74.58313   -1694.44250    -373.84189   -2100.66098        1
C    76.18871  69.84119  72.79223     412.33078    -664.70360     108.35451        1
H    75.93874  70.56103  71.97989    1765.77476    1294.44366    1621.76594        1
C    76.94845  68.57625  72.23352    -179.66635    -721.95667    -264.73915        1
H    77.92335  68.87787  71.77497    -607.34922    2587.87032    -598.10018        1
H    77.20237  67.86760  73.05442    -359.69332   -1479.13954   -2259.74260        1
C    76.00304  67.91560  71.17437    -240.01076     616.02425     431.85322        1
H    75.06380  67.55496  71.65120   -1359.17131     782.54425    2227.99181        1
H    75.68726  68.66097  70.40610    -820.36667      85.81767   -2596.78715        1
C    76.80697  66.72330  70.51732    -513.07015     236.18570    -551.87280        1
H    77.75399  67.09108  70.05915    1816.11944   -1183.11722   -1650.94321        1
```

H	77.11365	65.97997	71.28887	-1800.04429	-1361.53473	1526.34643	1
C	75.90432	66.06454	69.44853	726.08972	-253.19732	-179.64713	1
H	74.99371	65.57647	69.84950	1224.27271	-456.51798	-2390.90304	1
H	75.63702	66.72503	68.59963	1206.40339	-1150.93514	-2154.90344	1
N	76.72783	64.90083	68.80002	329.70796	-633.68968	-155.67358	1
H	76.22900	64.35223	68.04160	1348.31305	1938.76845	1359.00643	1
H	77.62888	65.16661	68.30583	2096.92035	1446.54551	-966.48890	1
H	77.05227	64.12068	69.44255	-1197.07217	-2303.53719	827.28733	1
C	74.93417	69.42455	73.74199	-378.01186	-556.17301	-413.96481	1
O	75.09334	68.89299	74.81573	-409.44433	-307.55053	-453.30833	1
N	73.80552	69.66295	73.07323	-429.02477	524.70884	274.08502	1
H	73.84585	70.07363	72.11337	1706.34263	-1425.96036	1574.38926	1
C	72.60385	69.76866	73.84612	-45.25864	-626.22952	478.93267	1
H	71.83313	69.83664	73.04603	1988.63164	570.90773	1772.87287	1
C	72.50552	68.45209	74.71825	551.77128	-20.49526	-564.54996	1
H	72.94302	67.59685	74.15492	1434.61535	-1753.10253	1513.99471	1
H	73.14269	68.54734	75.63366	-714.67738	1819.09770	1898.36494	1
C	71.02108	68.14100	75.10541	446.99263	-555.77305	338.97221	1
H	70.52599	69.01160	75.59033	1953.68466	1892.25957	-161.67766	1
H	70.41099	67.90594	74.20485	-403.03785	2688.07032	-188.43591	1
C	71.09529	66.90500	76.08974	567.86262	335.74681	434.04421	1
H	71.58242	66.03596	75.59041	-1080.02275	1568.26081	-1948.78900	1
H	71.73531	67.14158	76.97102	-919.54108	2111.34692	1456.13463	1
C	69.66368	66.53840	76.54288	195.81897	173.33862	-745.11536	1
H	69.17276	67.29677	77.18459	1405.67949	-2301.21089	-390.08113	1
H	68.98187	66.23630	75.72318	-940.37328	-1118.32446	2299.72133	1
N	69.78488	65.26223	77.44464	319.26422	656.36007	-42.02424	1
H	68.87606	64.89253	77.84736	1961.03633	619.91968	1787.09422	1
H	70.17874	64.38869	76.98770	854.14343	-2156.80752	1429.08711	1
H	70.38433	65.34068	78.31706	1237.02905	-1378.50857	-1998.28500	1
C	72.70055	71.15182	74.70468	532.53319	-268.57236	517.55729	1
O	73.09163	72.18886	74.18350	266.16714	-290.73804	-558.88002	1
N	72.24595	70.96381	75.92367	-226.25352	640.49678	-270.32839	1
H	72.00331	70.01354	76.19298	2007.69063	-1035.27229	1523.50231	1
C	72.03325	72.03364	76.82617	-540.76542	-573.33303	49.50000	1
H	72.24512	72.96757	76.25705	-2374.20950	-1036.76023	843.75774	1
C	70.49073	71.85706	77.23031	-77.09082	666.87422	-415.84136	1
H	70.34338	70.80311	77.59318	-985.81834	617.19825	2463.91927	1
O	69.57309	72.01306	76.12633	293.29899	-391.65602	-477.80926	1
H	69.17355	71.12345	76.08922	2528.72714	-681.76262	-751.27122	1
C	70.22903	72.82908	78.43399	252.47363	-605.14473	440.05134	1
H	69.16742	72.77385	78.77032	-2052.65063	1456.49051	-1043.51830	1
H	70.86299	72.57761	79.31604	-2152.98800	-1661.97574	-161.70652	1
H	70.42845	73.89045	78.15597	-473.12726	-1584.27339	2165.61725	1
C	73.06995	71.77186	78.06132	360.36307	488.46713	505.10056	1
O	73.74809	70.76506	78.11813	-200.08817	-528.03552	-385.82850	1
N	73.06681	72.81343	78.89111	-597.54095	-226.44775	355.20479	1
H	72.42416	73.55261	78.60844	2179.23655	-1300.70981	-991.33835	1
C	73.36777	72.64552	80.27661	-687.68921	297.39807	-249.45179	1
H	72.55974	73.22875	80.77026	-349.16256	-2467.37713	-1101.72831	1
C	73.48129	71.13145	80.77277	672.47345	361.54328	-201.62880	1
H	74.13456	70.52948	80.08048	1541.41502	582.18425	2169.97026	1
H	73.97920	71.11504	81.78511	-2534.67717	60.25153	997.72295	1
C	72.06760	70.53257	80.87003	574.26774	-389.43119	-377.02542	1
C	71.27151	70.87017	82.00411	688.82679	-287.33580	-257.96133	1
H	71.66837	71.53794	82.79079	331.14182	1470.38163	2269.80013	1
C	71.55495	69.65271	79.87013	-383.52382	-675.04808	144.24917	1
H	72.17036	69.37952	78.99211	4.09847	1517.59662	2262.86397	1
C	69.99198	70.32474	82.11115	-134.76730	428.45285	-649.50303	1
H	69.38195	70.57908	82.99547	-903.23433	1600.49020	2011.53387	1
C	70.26726	69.13082	80.01912	-335.53281	-615.00040	-364.39058	1
H	69.88676	68.44618	79.24082	1596.45398	-1369.33862	1732.02659	1

C	69.44033	69.44309	81.13734	585.48547	-340.24194	-406.23938	1
O	68.13040	68.92069	81.30180	-419.15649	-173.04408	-511.94141	1
H	68.08321	68.33652	80.50902	-1071.84350	1559.71507	-1960.13012	1
C	74.83207	73.26128	80.59304	291.41878	-183.13091	710.72246	1
O	75.65096	72.60748	81.22267	379.87695	523.47396	-222.23622	1
N	74.88492	74.58236	80.29091	564.30591	373.09380	277.24371	1
H	74.02051	74.76058	79.76514	-120.59931	-2436.07824	-1214.35098	1
C	76.10221	75.04020	79.63977	404.07514	-299.37480	608.84009	1
H	76.13005	74.51094	78.66437	-1071.07882	-841.69025	2359.66467	1
C	75.86025	76.58784	79.50604	-683.12807	68.71770	-390.13096	1
H	75.01350	76.79453	78.81293	2068.88918	1596.58590	770.89603	1
H	75.53884	76.99218	80.49260	1379.86429	2342.49779	-179.85638	1
C	77.15319	77.32628	79.03032	-176.39967	537.49466	550.97268	1
H	78.03224	77.04021	79.65027	-396.28051	1942.22168	1869.33314	1
H	77.42502	77.06162	77.98647	-94.44700	586.00678	-2659.20016	1
C	76.81713	78.88110	79.17539	-291.69381	-718.18079	-150.72929	1
H	75.92248	79.23330	78.60409	-2303.38776	1089.76242	964.62032	1
H	76.71481	79.25381	80.22657	884.16729	2568.80533	-207.74283	1
N	77.92626	79.51345	78.59948	-594.49522	-252.81478	342.29276	1
H	78.65115	78.91597	78.23366	-1735.17426	2084.05705	-263.71605	1
C	78.06874	80.77328	78.50924	595.23177	-271.52487	442.22352	1
N	77.04954	81.58347	79.02275	585.53813	-424.79750	-105.81303	1
H	76.18879	81.31443	79.49776	959.88797	1327.56823	-2177.11905	1
H	77.06097	82.60376	79.03173	1578.48647	16.27378	-2220.76233	1
N	79.27585	81.10473	77.87771	-452.17763	-390.33541	-421.51853	1
H	80.02896	80.51442	77.52778	470.26107	1974.25629	-1817.92087	1
H	79.58651	82.06698	77.73802	1856.67307	-1573.97321	-1224.35416	1
C	77.43736	74.75571	80.51088	-531.47271	-581.45780	-55.06206	1
O	78.48608	74.56307	79.94223	-243.00837	192.73507	609.52485	1
N	77.27878	75.00881	81.86179	343.49288	-644.25607	38.09164	1
H	76.24956	74.98863	81.92544	-1691.16043	-1535.97525	-1484.73099	1
C	78.05500	74.13551	82.78297	542.66492	350.14337	454.42563	1
H	78.12175	73.18585	82.20261	1209.57979	-2396.95920	463.86744	1
C	79.38611	74.76008	83.11206	-737.14309	145.93010	242.71942	1
H	79.18322	75.72362	83.63428	-1050.18303	159.36419	-2509.06125	1
C	80.18681	73.87944	84.05772	-706.77696	-252.88343	-245.16230	1
H	81.17160	74.34781	84.33005	-1624.64039	-1665.49085	1417.86935	1
H	79.62508	73.69905	85.01752	2566.10521	598.51109	693.22305	1
H	80.40786	72.87496	83.60456	-1972.69758	107.10018	-1876.34312	1
C	80.22496	75.07878	81.88503	412.85148	672.58832	-27.69222	1
H	81.25677	75.43085	82.16103	1447.02277	1762.76533	-1490.78944	1
H	80.34204	74.17716	81.21990	1320.77379	-145.20455	2378.68491	1
H	79.74978	75.88882	81.26302	-1512.10848	1470.52031	1724.75147	1
C	77.17210	74.00867	84.16295	-607.11113	499.56689	73.73769	1
O	77.52208	74.58219	85.16822	357.97126	460.81534	356.68785	1
N	76.09937	73.24532	83.98635	536.55407	-368.62270	332.76306	1
H	75.90350	72.81910	83.06392	524.38866	2297.69985	1367.20941	1
C	75.48049	72.78601	85.18590	46.50277	-774.29190	-147.97904	1
H	76.29443	72.81651	85.94569	1676.51875	1883.29066	1032.55549	1
C	74.25142	73.75365	85.59407	-559.41456	-34.19198	-556.30433	1
H	73.54081	73.81356	84.73613	-64.66664	395.18769	2695.05373	1
H	73.67638	73.30227	86.43794	-2703.44278	-60.40056	333.79418	1
C	74.73829	75.12618	85.96864	-519.76435	377.31045	459.42366	1
H	75.42275	75.48640	85.16374	-1746.74323	1667.50644	-1261.73691	1
C	73.55641	76.08144	86.08988	570.95554	16.46261	-545.27700	1
H	73.88891	77.12474	86.32636	2391.58273	28.05277	-1305.07271	1
H	72.96088	76.12800	85.14224	175.75588	917.63458	2559.43867	1
H	72.85599	75.76532	86.90544	-1063.97851	-2155.34650	1283.00462	1
C	75.51277	75.11951	87.27967	193.76014	2.62946	-765.53141	1
H	75.78597	76.15799	87.60079	507.39394	-846.55087	2539.60089	1
H	74.92134	74.65823	88.11219	1337.57841	1818.19054	1526.01994	1
H	76.47181	74.54377	87.18522	1100.88635	-1172.91350	-2199.08909	1

```
C    74.83189   71.33892   84.91460      -549.21115      475.64486     -309.38218          1
O    74.42528   70.98382   83.83629       556.06106     -390.37755      -78.24250          1
O    74.59818   70.55200   86.06626      -572.27940     -310.19032     -209.75862          1
H    75.00540   71.15421   86.75012     -1380.08193     2212.14486      790.85944          1
```

Listing 59: C00059.xmolout

```
       276
  150.0000  150.0000  150.0000     0.0000     0.0000     0.0000***********    1.5102     6041
       0.2500    300.00    0.00    0.00    0.00   -1293.6332       0.0000    300.0000
H    73.97867   76.67287   82.18158     -2043.14101    -1757.64893      399.89339          1
N    74.98291   76.60699   82.33795       426.75047      490.45733     -334.42467          1
H    75.18724   75.64694   82.06382       442.25559    -1110.30854    -2448.52907          1
C    75.76083   77.54555   81.62857       415.32972     -666.39213       83.73172          1
H    75.14980   78.46710   81.51134       879.32219     2491.00447      667.35213          1
C    77.13634   77.77437   82.43997        31.35357      477.41777      628.23362          1
H    77.71833   76.81865   82.45082      1168.38489     2240.25337    -1019.71136          1
H    77.78355   78.51737   81.91474      -360.43770     2534.90498      931.67112          1
C    76.82609   78.21652   83.84716       463.18208      512.70242     -382.34388          1
H    75.99579   77.58306   84.23422      1725.29117     1863.52247     -987.07854          1
C    78.04549   78.01106   84.73699       710.42801     -144.27427     -313.15367          1
H    77.83689   78.29666   85.80026       364.20195    -1033.13725     2494.72524          1
H    78.37932   76.94083   84.73763      2567.61904      335.80679      847.48802          1
H    78.91512   78.62734   84.39093     -2587.99029      680.92867     -512.16411          1
C    76.39050   79.67413   83.89072       268.17616      741.64379      -40.42634          1
H    76.14979   80.00192   84.93517      1283.54314    -1945.37420    -1411.27812          1
H    77.19323   80.35554   83.50688     -1258.57796     1317.15249    -2026.02085          1
H    75.47648   79.85274   83.26757     -2502.84526      909.80597      575.92391          1
C    76.19044   76.81845   80.23516       619.36578     -458.64843     -172.09273          1
O    76.58242   75.67783   80.19115       -81.40433      433.87611      522.34594          1
N    76.21610   77.76127   79.27347       175.23996     -622.20190      341.55523          1
H    75.75001   78.63052   79.54021      -876.57256     -303.22879    -2561.90213          1
C    76.08493   77.29120   77.89560      -571.88214     -534.10783     -106.15033          1
H    75.36663   76.44222   77.96666     -2713.20841      -16.25800     -248.80809          1
C    75.51177   78.48520   77.07862       166.60354      288.16399     -716.09568          1
H    76.05526   79.43135   77.28984     -2097.85574     1636.78449     -586.18044          1
O    74.01451   78.84478   77.57482      -317.80580     -363.38227      484.43082          1
H    74.16202   79.76824   77.75088      1863.74748    -1451.84923     1357.29467          1
C    75.55667   78.13378   75.57596       503.80731      517.99032      318.51606          1
H    75.16637   78.97021   74.94332       640.61020    -2157.10410    -1536.29104          1
H    76.60335   77.91449   75.24018      1687.88455      465.60692     2087.56452          1
H    74.94138   77.22832   75.35323     -1293.07895    -1899.88581    -1463.57452          1
C    77.49945   76.77463   77.27872      -153.07550     -733.68902      248.70981          1
O    77.37654   76.05032   76.31403       186.15459     -480.78787     -449.34387          1
N    78.61545   77.33995   77.74801      -487.53139      543.18517       42.03850          1
C    79.89762   76.81981   77.38033      -699.35871       69.34393      360.10701          1
H    79.70455   76.31996   76.40307         2.81126     -221.99807    -2715.58072          1
C    80.76034   78.14501   77.32018      -507.46439     -592.59194      122.07761          1
H    81.85610   77.95844   77.38445     -1975.08116      525.87560    -1801.71556          1
H    80.59344   78.68555   76.36196      1588.27743    -1913.22100     1113.83566          1
C    80.18979   78.96621   78.52220      -613.62442     -457.55993     -194.14490          1
H    80.59883   78.61474   79.49526      1707.54637     -721.27992     1996.92523          1
H    80.41566   80.05468   78.46337       758.23357     2100.81767     1560.55011          1
C    78.64198   78.64285   78.40782      -352.41524      704.96076       49.21465          1
H    78.11719   78.52981   79.38447      1714.76886    -2070.42778      443.35792          1
H    78.06197   79.33330   77.75251      1224.15040     1801.50881     1636.97633          1
C    80.36746   75.72169   78.51570       566.85117      537.05831      117.62814          1
O    79.81506   74.64780   78.51816      -550.26779     -406.08867        4.21666          1
N    81.33510   76.16829   79.29567       566.34832     -425.77725      180.18749          1
H    81.69206   77.10478   79.12435       644.44502     2646.80550      -52.73430          1
C    81.85566   75.41289   80.38341       612.34801     -281.48662      411.56221          1
H    82.43844   76.18264   80.93658      1667.23498    -1490.75142     1556.16753          1
```

C	80.63997	74.83241	81.24215	-714.45746	-175.43074	286.98927	1
H	80.06431	74.08838	80.61765	-787.09484	1763.03619	-1922.46060	1
O	79.65434	75.78601	81.68548	-429.46514	516.51932	-128.40526	1
H	78.89171	75.54434	81.11233	1981.82785	436.16847	-1818.18182	1
C	81.44021	74.08813	82.37261	630.79189	-468.94448	76.03230	1
H	80.76007	73.52142	83.04921	-1775.71861	2062.30353	131.89849	1
H	82.17444	73.35922	81.95571	-1855.14608	88.94118	1993.53760	1
H	82.01338	74.80513	83.00517	-1504.82350	-780.94249	-2132.90992	1
C	82.86685	74.30884	79.71306	288.57638	618.62814	-397.00270	1
O	83.95003	74.69143	79.34654	321.70495	-185.26452	574.37218	1
N	82.38331	73.07606	79.70358	-447.05792	-446.53431	367.76425	1
H	81.41642	72.79358	79.95361	350.31316	1975.63644	-1843.31522	1
C	83.12025	72.07893	78.96493	-511.23061	374.03917	-471.51480	1
H	83.29800	72.52517	77.95992	2234.79579	-898.70890	-1273.45190	1
C	84.49632	71.76948	79.72078	529.53781	-159.34528	563.72604	1
H	85.11849	71.13947	79.02899	654.59388	2347.94251	1217.51488	1
H	85.03683	72.74538	79.84371	-1526.55661	2103.42026	817.87394	1
O	84.39071	71.04583	80.98530	-554.84278	14.12789	399.58769	1
H	84.30534	71.80623	81.58822	1297.06236	2061.84854	1220.68812	1
C	82.06082	70.87157	79.00037	-662.15484	-412.13544	123.62687	1
O	80.94420	71.07464	79.48349	-540.01537	-317.05804	274.91456	1
N	82.52395	69.77867	78.40942	-202.76299	-690.00100	-131.49145	1
H	83.49186	69.87043	78.09385	-702.62132	-2617.37191	281.70413	1
C	81.94466	68.45194	78.56693	-491.73914	576.34187	222.73509	1
H	82.77534	67.75743	78.29441	1977.17978	-1823.58850	-434.68928	1
C	81.45951	68.25882	79.99770	-649.50797	-328.66843	306.11224	1
H	80.72470	69.07309	80.21453	2075.07803	1452.16490	-1004.45904	1
C	80.75813	66.92269	80.15236	-615.18735	-489.99689	-70.96352	1
H	80.24784	66.82851	81.15054	-1226.32661	-1724.38906	-1716.47170	1
H	79.97744	66.77701	79.35043	2381.64273	-969.84735	900.46860	1
H	81.48019	66.06457	80.06548	2122.74991	1702.23946	-141.36228	1
C	82.58928	68.37937	81.00134	-501.86074	-479.61276	-376.42482	1
H	82.21623	68.30821	82.06014	993.79261	-2246.95401	-1177.81305	1
H	83.35290	67.56501	80.86383	42.71279	-626.15391	-2651.37258	1
H	83.13662	69.36751	80.90131	-2026.39012	-1590.33709	-887.82922	1
C	80.66567	68.30664	77.51332	-672.89153	382.57394	-156.34072	1
O	80.34764	67.20700	77.13446	410.04627	221.86223	500.35950	1
N	80.08250	69.46222	77.25031	-378.11782	-443.79414	-441.10984	1
H	80.40194	70.31130	77.73314	2404.15973	-568.14894	1149.30085	1
C	79.11615	69.47157	76.19587	-544.54484	466.16019	331.29119	1
H	79.34441	68.56377	75.58899	-1409.80988	-170.17955	2325.32679	1
C	77.70694	69.38213	76.93427	-184.48284	116.78282	-758.89137	1
H	77.71149	68.53683	77.65881	1893.78641	1861.14652	611.04528	1
H	77.52662	70.30017	77.53823	-2170.79665	-974.19311	1327.50117	1
C	76.59623	69.18345	75.85974	389.51668	672.81878	-138.49152	1
H	76.46325	70.12677	75.27872	510.64577	1933.03327	1851.02484	1
H	76.91424	68.42774	75.10866	-2302.30869	-1105.72939	-948.89774	1
C	75.21721	68.72875	76.48347	-559.93427	-126.34590	542.30857	1
O	74.38823	68.18668	75.79667	-670.36003	-111.83166	76.36448	1
N	75.09657	69.04023	77.78960	-216.06729	-537.07037	446.51379	1
H	75.74843	69.56619	78.38344	-1251.96487	1074.12894	-2168.52500	1
H	74.24751	68.89096	78.34520	-2451.83023	-720.73851	944.84612	1
C	79.18291	70.90349	75.43337	546.24052	-563.88337	-85.11809	1
O	79.05585	71.92606	76.08959	71.70702	-307.40463	606.69673	1
N	79.31350	70.77479	74.11606	479.90863	-504.50129	222.86849	1
C	79.50069	71.92386	73.26776	75.51268	-600.91904	506.73700	1
H	80.17920	72.59613	73.84202	-2033.61562	1795.30103	254.89480	1
C	80.10981	71.28715	71.95048	523.79067	517.99274	284.45581	1
H	79.92754	71.91327	71.04792	1599.61665	-1127.00911	1895.98173	1
H	81.21211	71.15554	72.03141	-1881.83093	1275.82488	-1501.55016	1
C	79.37548	69.91026	71.90041	-600.05639	-404.05026	316.64485	1
H	78.33416	70.03120	71.52668	-2597.05260	496.36221	657.73249	1

117	H	79.86952	69.16543	71.23667	-1666.24518	1294.38985	-1723.90591	1
118	C	79.39361	69.49105	73.43288	493.57570	-129.95376	-602.56394	1
119	H	78.53657	68.85810	73.76090	-1469.08415	-637.84797	2204.22581	1
120	H	80.34892	69.02514	73.77378	-1038.97840	1068.88701	-2280.71789	1
121	C	78.05520	72.61987	72.98052	188.88100	241.05089	-727.87821	1
122	O	77.61527	72.70680	71.84617	531.88613	-49.19943	427.07960	1
123	N	77.58511	73.10469	74.10726	708.14833	26.93417	-179.73509	1
124	H	78.12886	72.95844	74.99023	-2232.69501	-1179.09831	1023.94816	1
125	C	76.22049	73.49491	74.21583	-738.32412	259.92548	104.42456	1
126	H	76.16067	73.85905	75.26711	2028.89123	1260.02983	1311.33324	1
127	C	75.87144	74.61573	73.13691	-258.65678	-345.52513	661.28459	1
128	H	75.91997	74.21772	72.09965	-1826.06090	-1801.46097	-918.64554	1
129	C	74.43482	75.15459	73.45266	-541.23400	531.92970	-218.41428	1
130	H	74.15979	76.02527	72.80368	1311.73218	-2219.18200	882.19023	1
131	H	73.65572	74.36939	73.29708	-1048.71298	1223.58118	-2196.97971	1
132	H	74.34786	75.49079	74.51210	419.06414	1903.92898	1903.44670	1
133	C	76.90956	75.77603	73.28928	-423.86431	152.66566	-648.55262	1
134	H	76.56698	76.71372	72.78998	105.68386	-2342.50080	1387.51238	1
135	H	77.09698	76.00454	74.38059	-1695.63603	-1484.73621	-1531.02793	1
136	H	77.89262	75.49461	72.84167	-1853.57121	-487.39031	1936.59343	1
137	C	75.29136	72.17217	73.96849	539.71428	-510.58232	267.58633	1
138	O	74.78159	71.63613	74.94059	-15.93758	-278.34085	-624.49402	1
139	N	75.25040	71.86012	72.69926	-676.81886	12.05292	276.17960	1
140	H	75.86892	72.37508	72.03457	-1694.44250	-373.84189	-2100.66098	1
141	C	74.51608	70.75665	72.18834	412.33078	-664.70360	108.35451	1
142	H	74.77550	70.77654	71.10529	1765.77476	1294.44366	1621.76594	1
143	C	75.03378	69.44853	72.92702	-179.66635	-721.95667	-264.73915	1
144	H	76.14592	69.40205	72.93339	-607.34922	2587.87032	-598.10018	1
145	H	74.72235	69.44274	73.99927	-359.69332	-1479.13954	-2259.74260	1
146	C	74.40776	68.24815	72.14708	-240.01076	616.02425	431.85322	1
147	H	73.29896	68.34599	72.11716	-1359.17131	782.54425	2227.99181	1
148	H	74.74355	68.25133	71.08583	-820.36667	85.81767	-2596.78715	1
149	C	74.82202	66.89760	72.85852	-513.07015	236.18570	-551.87280	1
150	H	75.92898	66.76958	72.87153	1816.11944	-1183.11722	-1650.94321	1
151	H	74.50028	66.89424	73.92882	-1800.04429	-1361.53473	1526.34643	1
152	C	74.13440	65.76851	72.05541	726.08972	-253.19732	-179.64713	1
153	H	73.02922	65.75424	72.13750	1224.27271	-456.51798	-2390.90304	1
154	H	74.43294	65.71477	70.98934	1206.40339	-1150.93514	-2154.90344	1
155	N	74.59192	64.39678	72.65390	329.70796	-633.68968	-155.67358	1
156	H	74.17049	63.53700	72.19674	1348.31305	1938.76845	1359.00643	1
157	H	75.62777	64.16894	72.61101	2096.92035	1446.54551	-966.48890	1
158	H	74.36960	64.21985	73.67644	-1197.07217	-2303.53719	827.28733	1
159	C	72.90463	70.90232	72.43291	-378.01186	-556.17301	-413.96481	1
160	O	72.16108	70.94993	71.46639	-409.44433	-307.55053	-453.30833	1
161	N	72.58376	70.88076	73.70619	-429.02477	524.70884	274.08502	1
162	H	73.30339	70.90060	74.47024	1706.34263	-1425.96036	1574.38926	1
163	C	71.18586	70.80772	73.99392	-45.25864	-626.22952	478.93267	1
164	H	70.79544	70.20455	73.14055	1988.63164	570.90773	1772.87287	1
165	C	71.01927	70.16996	75.42947	551.77128	-20.49526	-564.54996	1
166	H	71.49598	69.16469	75.48633	1434.61535	-1753.10253	1513.99471	1
167	H	71.53964	70.79266	76.19213	-714.67738	1819.09770	1898.36494	1
168	C	69.48302	70.09848	75.72289	446.99263	-555.77305	338.97221	1
169	H	69.01926	71.09573	75.53962	1953.68466	1892.25957	-161.67766	1
170	H	68.97257	69.38850	75.03424	-403.03785	2688.07032	-188.43591	1
171	C	69.28296	69.67557	77.23269	567.86262	335.74681	434.04421	1
172	H	69.64968	68.63864	77.41178	-1080.02275	1568.26081	-1948.78900	1
173	H	69.88095	70.33045	77.90719	-919.54108	2111.34692	1456.13463	1
174	C	67.77430	69.80177	77.55222	195.81897	173.33862	-745.11536	1
175	H	67.35374	70.81436	77.38951	1405.67949	-2301.21089	-390.08113	1
176	H	67.13007	69.04281	77.06443	-940.37328	-1118.32446	2299.72133	1
177	N	67.59871	69.55706	79.08767	319.26422	656.36007	-42.02424	1
178	H	66.60651	69.63145	79.45585	1961.03633	619.91968	1787.09422	1

179	H	67.89472	68.61183	79.46943	854.14343	-2156.80752	1429.08711	1
180	H	68.11005	70.22596	79.73302	1237.02905	-1378.50857	-1998.28500	1
181	C	70.56790	72.34117	73.97288	532.53319	-268.57236	517.55729	1
182	O	70.30159	72.90951	74.99976	266.16714	-290.73804	-558.88002	1
183	N	70.43623	72.69331	72.70833	-226.25352	640.49678	-270.32839	1
184	H	70.79913	72.03816	71.98209	2007.69063	-1035.27229	1523.50231	1
185	C	69.66331	73.80355	72.27881	-540.76542	-573.33303	49.50000	1
186	H	69.32226	73.50095	71.26434	-2374.20950	-1036.76023	843.75774	1
187	C	68.46175	74.07180	73.29692	-77.09082	666.87422	-415.84136	1
188	H	68.90104	74.32625	74.30663	-985.81834	617.19825	2463.91927	1
189	O	67.55839	72.96574	73.49569	293.29899	-391.65602	-477.80926	1
190	H	67.78691	72.71660	74.41523	2528.72714	-681.76262	-751.27122	1
191	C	67.81501	75.37251	72.69325	252.47363	-605.14473	440.05134	1
192	H	66.99159	75.75392	73.34059	-2052.65063	1456.49051	-1043.51830	1
193	H	68.56083	76.19468	72.58408	-2152.98800	-1661.97574	-161.70652	1
194	H	67.38036	75.18077	71.68522	-473.12726	-1584.27339	2165.61725	1
195	C	70.63798	75.11930	72.22530	360.36307	488.46713	505.10056	1
196	O	71.19325	75.55919	73.20063	-200.08817	-528.03552	-385.82850	1
197	N	70.53182	75.67886	70.99521	-597.54095	-226.44775	355.20479	1
198	H	70.03501	75.05598	70.35424	2179.23655	-1300.70981	-991.33835	1
199	C	71.78506	76.20625	70.49346	-687.68921	297.39807	-249.45179	1
200	H	72.53114	75.39914	70.65211	-349.16256	-2467.37713	-1101.72831	1
201	C	71.55437	76.58195	68.96905	672.47345	361.54328	-201.62880	1
202	H	70.68662	77.27498	68.87916	1541.41502	582.18425	2169.97026	1
203	H	72.44832	77.13059	68.59206	-2534.67717	60.25153	997.72295	1
204	C	71.31208	75.32882	68.10921	574.26774	-389.43119	-377.02542	1
205	C	72.43246	74.55613	67.68213	688.82679	-287.33580	-257.96133	1
206	H	73.45622	74.82597	67.99885	331.14182	1470.38163	2269.80013	1
207	C	69.99117	74.96408	67.70992	-383.52382	-675.04808	144.24917	1
208	H	69.11825	75.55141	68.04795	4.09847	1517.59662	2262.86397	1
209	C	72.21134	73.46320	66.84316	-134.76730	428.45285	-649.50303	1
210	H	73.08012	72.87252	66.50428	-903.23433	1600.49020	2011.53387	1
211	C	69.82071	73.86072	66.86827	-335.53281	-615.00040	-364.39058	1
212	H	68.79570	73.59487	66.55583	1596.45398	-1369.33862	1732.02659	1
213	C	70.91426	73.07669	66.39769	585.48547	-340.24194	-406.23938	1
214	O	70.75457	71.96180	65.53283	-419.15649	-173.04408	-511.94141	1
215	H	69.77532	71.97618	65.42100	-1071.84350	1559.71507	-1960.13012	1
216	C	72.15293	77.57175	71.29208	291.41878	-183.13091	710.72246	1
217	O	73.29070	77.79011	71.63874	379.87695	523.47396	-222.23622	1
218	N	71.08962	78.40216	71.29951	564.30591	373.09380	277.24371	1
219	H	70.25016	77.91330	70.97980	-120.59931	-2436.07824	-1214.35098	1
220	C	70.96316	79.38047	72.34023	404.07514	-299.37480	608.84009	1
221	H	69.86780	79.57854	72.33212	-1071.07882	-841.69025	2359.66467	1
222	C	71.47559	78.74622	73.69388	-683.12807	68.71770	-390.13096	1
223	H	71.02639	77.73730	73.85815	2068.88918	1596.58590	770.89603	1
224	H	72.58062	78.58496	73.66139	1379.86429	2342.49779	-179.85638	1
225	C	71.05454	79.76135	74.80251	-176.39967	537.49466	550.97268	1
226	H	71.64128	80.70539	74.71444	-396.28051	1942.22168	1869.33314	1
227	H	69.99233	80.06211	74.66234	-94.44700	586.00678	-2659.20016	1
228	C	71.26053	79.09523	76.23933	-291.69381	-718.18079	-150.72929	1
229	H	70.64029	78.17602	76.40249	-2303.38776	1089.76242	964.62032	1
230	H	72.32777	78.89866	76.57025	884.16729	2568.80533	-207.74283	1
231	N	70.73215	80.13402	77.01906	-594.49522	-252.81478	342.29276	1
232	H	70.01542	80.70181	76.59162	-1735.17426	2084.05705	-263.71605	1
233	C	71.05664	80.39860	78.22465	595.23177	-271.52487	442.22352	1
234	N	72.02325	79.61166	78.84277	585.53813	-424.79750	-105.81303	1
235	H	72.55722	78.80789	78.41445	959.88797	1327.56823	-2177.11905	1
236	H	72.34307	79.75735	79.80677	1578.48647	16.27378	-2220.76233	1
237	N	70.32273	81.49198	78.71396	-452.17763	-390.33541	-421.51853	1
238	H	69.62946	82.08073	78.25597	470.26107	1974.25629	-1817.92087	1
239	H	70.43700	81.87295	79.65301	1856.67307	-1573.97321	-1224.35416	1
240	C	71.83741	80.70770	71.97733	-531.47271	-581.45780	-55.06206	1

```
O    72.66855  81.15956  72.72775     -243.00837      192.73507      609.52485         1
N    71.37940  81.25960  70.83553      343.49288     -644.25607       38.09164         1
H    70.72973  80.63886  70.35155    -1691.16043    -1535.97525    -1484.73099         1
C    72.21709  82.24900  70.14569      542.66492      350.14337      454.42563         1
H    73.16966  82.24445  70.73013     1209.57979    -2396.95920      463.86744         1
C    72.36563  81.81138  68.69935     -737.14309      145.93010      242.71942         1
H    71.34748  81.77427  68.25056    -1050.18303      159.36419    -2509.06125         1
C    73.19442  82.80004  67.90076     -706.77696     -252.88343     -245.16230         1
H    73.25411  82.51547  66.81426    -1624.64039    -1665.49085     1417.86935         1
H    72.76109  83.83941  67.95468     2566.10521      598.51109      693.22305         1
H    74.24834  82.86325  68.28863    -1972.69758      107.10018    -1876.34312         1
C    72.96246  80.41933  68.59699      412.85148      672.58832      -27.69222         1
H    72.99370  80.05367  67.53412     1447.02277     1762.76533    -1490.78944         1
H    74.01534  80.39415  68.99121     1320.77379     -145.20455     2378.68491         1
H    72.37287  79.67059  69.19563    -1512.10848     1470.52031     1724.75147         1
C    71.45183  83.71396  70.20613     -607.11113      499.56689       73.73769         1
O    72.07217  84.72369  69.94679      357.97126      460.81534      356.68785         1
N    70.15460  83.59002  70.44196      536.55407     -368.62270      332.76306         1
H    69.82405  82.65442  70.67383      524.38866     2297.69985     1367.20941         1
C    69.43718  84.74838  70.85002       46.50277     -774.29190     -147.97904         1
H    70.22312  85.51713  71.03228     1676.51875     1883.29066     1032.55549         1
C    68.40393  85.19003  69.68961     -559.41456      -34.19198     -556.30433         1
H    67.62699  84.39810  69.57080      -64.66664      395.18769     2695.05373         1
H    67.85273  86.11072  69.99617    -2703.44278      -60.40056      333.79418         1
C    69.12146  85.41090  68.38547     -519.76435      377.31045      459.42366         1
H    69.65898  84.47191  68.12003    -1746.74323     1667.50644    -1261.73691         1
C    68.12217  85.73009  67.27978      570.95554       16.46261     -545.27700         1
H    68.62919  85.86181  66.28944     2391.58273       28.05277    -1305.07271         1
H    67.36329  84.91493  67.15881      175.75588      917.63458     2559.43867         1
H    67.56109  86.67611  67.49232    -1063.97851    -2155.34650     1283.00462         1
C    70.14463  86.53170  68.48708      193.76014        2.62946     -765.53141         1
H    70.54929  86.81545  67.48118      507.39394     -846.55087     2539.60089         1
H    69.70737  87.45785  68.94198     1337.57841     1818.19054     1526.01994         1
H    71.02865  86.21443  69.12436     1100.88635    -1172.91350    -2199.08909         1
C    68.57010  84.31987  72.13507     -549.21115      475.64486     -309.38218         1
O    68.44392  83.17896  72.49763      556.06106     -390.37755      -78.24250         1
O    67.88009  85.36605  72.79514     -572.27940     -310.19032     -209.75862         1
H    68.20149  86.13278  72.24582    -1380.08193     2212.14486      790.85944         1
```

Listing 60: C00060.xmolout

```
      276
  150.0000  150.0000  150.0000    0.0000    0.0000    0.0000***********    2.7750    11100
      0.2500    300.00    0.00    0.00    0.00  -1293.6324      0.0000    300.0000
H    61.06076  70.95545  83.37899    -2043.14101    -1757.64893      399.89339         1
N    61.65840  70.74764  82.58071      426.75047      490.45733     -334.42467         1
H    60.99405  70.72897  81.80654      442.25559    -1110.30854    -2448.52907         1
C    62.69569  71.67690  82.35951      415.32972     -666.39213       83.73172         1
H    63.00447  72.08448  83.34614      879.32219     2491.00447      667.35213         1
C    63.87867  70.92111  81.56693       31.35357      477.41777      628.23362         1
H    63.48494  70.58175  80.57862     1168.38489     2240.25337    -1019.71136         1
H    64.70975  71.63204  81.34081     -360.43770     2534.90498      931.67112         1
C    64.37747  69.73906  82.35945      463.18208      512.70242     -382.34388         1
H    63.49555  69.14010  82.68130     1725.29117     1863.52247     -987.07854         1
C    65.26712  68.86235  81.48718      710.42801     -144.27427     -313.15367         1
H    65.63628  67.96228  82.04333      364.20195    -1033.13725     2494.72524         1
H    64.72222  68.49256  80.58020     2567.61904      335.80679      847.48802         1
H    66.16866  69.42182  81.12640    -2587.99029      680.92867     -512.16411         1
C    65.14601  70.17922  83.59711      268.17616      741.64379      -40.42634         1
H    65.54655  69.30167  84.16812     1283.54314    -1945.37420    -1411.27812         1
H    66.02016  70.82807  83.33034    -1258.57796     1317.15249    -2026.02085         1
H    64.49906  70.76502  84.29995    -2502.84526      909.80597      575.92391         1
```

C	62.09099	72.77975	81.31821	619.36578	-458.64843	-172.09273	1
O	61.39462	72.46521	80.37772	-81.40433	433.87611	522.34594	1
N	62.58402	73.97637	81.62648	175.23996	-622.20190	341.55523	1
H	63.19902	74.07683	82.43359	-876.57256	-303.22879	-2561.90213	1
C	62.29899	75.06668	80.76987	-571.88214	-534.10783	-106.15033	1
H	61.68675	74.65077	79.93602	-2713.20841	-16.25800	-248.80809	1
C	61.55564	76.20089	81.63572	166.60354	288.16399	-716.09568	1
H	62.18516	76.42224	82.54086	-2097.85574	1636.78449	-586.18044	1
O	60.24828	75.87393	82.12289	-317.80580	-363.38227	484.43082	1
H	60.38795	75.91244	83.09195	1863.74748	-1451.84923	1357.29467	1
C	61.65325	77.40282	80.62712	503.80731	517.99032	318.51606	1
H	61.29855	78.35581	81.08390	640.61020	-2157.10410	-1536.29104	1
H	62.71206	77.55748	80.29165	1687.88455	465.60692	2087.56452	1
H	61.03870	77.22221	79.71413	-1293.07895	-1899.88581	-1463.57452	1
C	63.74166	75.73009	80.34877	-153.07550	-733.68902	248.70981	1
O	64.39217	76.29392	81.18812	186.15459	-480.78787	-449.34387	1
N	64.01020	75.54346	79.04517	-487.53139	543.18517	42.03850	1
C	65.12884	76.17250	78.42322	-699.35871	69.34393	360.10701	1
H	65.85133	76.45287	79.22513	2.81126	-221.99807	-2715.58072	1
C	65.59376	75.02596	77.42007	-507.46439	-592.59194	122.07761	1
H	66.25260	75.40366	76.60671	-1975.08116	525.87560	-1801.71556	1
H	66.16093	74.22714	77.94698	1588.27743	-1913.22100	1113.83566	1
C	64.21655	74.50414	76.90838	-613.62442	-457.55993	-194.14490	1
H	63.79032	75.19366	76.14524	1707.54637	-721.27992	1996.92523	1
H	64.26967	73.49871	76.43419	758.23357	2100.81767	1560.55011	1
C	63.34908	74.52400	78.23917	-352.41524	704.96076	49.21465	1
H	62.28841	74.83603	78.09325	1714.76886	-2070.42778	443.35792	1
H	63.37893	73.58525	78.84240	1224.15040	1801.50881	1636.97633	1
C	64.64115	77.43643	77.49957	566.85117	537.05831	117.62814	1
O	63.50380	77.58443	77.12729	-550.26779	-406.08867	4.21666	1
N	65.73100	78.10898	77.12363	566.34832	-425.77725	180.18749	1
H	66.60065	77.86695	77.59573	644.44502	2646.80550	-52.73430	1
C	65.54158	79.42850	76.56044	612.34801	-281.48662	411.56221	1
H	64.53497	79.74635	76.92301	1667.23498	-1490.75142	1556.16753	1
C	66.72265	80.31125	77.05639	-714.45746	-175.43074	286.98927	1
H	67.68538	79.95757	76.62052	-787.09484	1763.03619	-1922.46060	1
O	67.03716	80.05767	78.60521	-429.46514	516.51932	-128.40526	1
H	67.08571	80.96305	78.89516	1981.82785	436.16847	-1818.18182	1
C	66.45378	81.79508	76.71698	630.79189	-468.94448	76.03230	1
H	67.30635	82.44871	77.02849	-1775.71861	2062.30353	131.89849	1
H	66.29966	81.94416	75.61500	-1855.14608	88.94118	1993.53760	1
H	65.53512	82.17073	77.23272	-1504.82350	-780.94249	-2132.90992	1
C	65.62292	79.37011	74.92212	288.57638	618.62814	-397.00270	1
O	65.51105	80.43651	74.27904	321.70495	-185.26452	574.37218	1
N	65.85838	78.16910	74.47503	-447.05792	-446.53431	367.76425	1
H	65.89239	77.53280	75.27334	350.31316	1975.63644	-1843.31522	1
C	65.93741	77.71794	73.13555	-511.23061	374.03917	-471.51480	1
H	65.81008	76.61711	73.23410	2234.79579	-898.70890	-1273.45190	1
C	64.78048	78.42543	72.29361	529.53781	-159.34528	563.72604	1
H	64.52835	77.78140	71.40769	654.59388	2347.94251	1217.51488	1
H	63.87591	78.43391	72.95784	-1526.55661	2103.42026	817.87394	1
O	65.05513	79.74658	71.79391	-554.84278	14.12789	399.58769	1
H	65.21544	80.21776	72.69625	1297.06236	2061.84854	1220.68812	1
C	67.38439	78.10166	72.48535	-662.15484	-412.13544	123.62687	1
O	67.93172	77.34687	71.72222	-540.01537	-317.05804	274.91456	1
N	67.76342	79.32442	72.85978	-202.76299	-690.00100	-131.49145	1
H	67.12951	79.85665	73.47556	-702.62132	-2617.37191	281.70413	1
C	69.05670	79.85433	72.44066	-491.73914	576.34187	222.73509	1
H	69.39782	79.11913	71.66798	1977.17978	-1823.58850	-434.68928	1
C	68.81423	81.25612	71.89590	-649.50797	-328.66843	306.11224	1
H	68.44442	81.88189	72.73904	2075.07803	1452.16490	-1004.45904	1
C	70.08247	81.89032	71.35787	-615.18735	-489.99689	-70.96352	1

83	H	69.90348	82.94063	70.99642	-1226.32661	-1724.38906	-1716.47170	1
84	H	70.88983	81.93808	72.14611	2381.64273	-969.84735	900.46860	1
85	H	70.50145	81.30696	70.49205	2122.74991	1702.23946	-141.36228	1
86	C	67.74252	81.26093	70.82188	-501.86074	-479.61276	-376.42482	1
87	H	67.45662	82.30657	70.52148	993.79261	-2246.95401	-1177.81305	1
88	H	68.08914	80.73063	69.89258	42.71279	-626.15391	-2651.37258	1
89	H	66.79963	80.73526	71.16871	-2026.39012	-1590.33709	-887.82922	1
90	C	70.05307	79.90423	73.76302	-672.89153	382.57394	-156.34072	1
91	O	71.14438	80.43127	73.66108	410.04627	221.86223	500.35950	1
92	N	69.51126	79.39308	74.86624	-378.11782	-443.79414	-441.10984	1
93	H	68.63402	78.90995	74.68150	2404.15973	-568.14894	1149.30085	1
94	C	70.44603	78.98352	75.87507	-544.54484	466.16019	331.29119	1
95	H	71.21990	79.78173	75.85155	-1409.80988	-170.17955	2325.32679	1
96	C	69.68980	78.82653	77.25668	-184.48284	116.78282	-758.89137	1
97	H	69.17708	79.77346	77.54440	1893.78641	1861.14652	611.04528	1
98	H	68.88952	78.05513	77.18464	-2170.79665	-974.19311	1327.50117	1
99	C	70.76207	78.43222	78.31932	389.51668	672.81878	-138.49152	1
100	H	71.32035	77.50835	78.00254	510.64577	1933.03327	1851.02484	1
101	H	71.55246	79.21408	78.35687	-2302.30869	-1105.72939	-948.89774	1
102	C	70.18029	78.21606	79.77174	-559.93427	-126.34590	542.30857	1
103	O	70.89135	77.74827	80.62791	-670.36003	-111.83166	76.36448	1
104	N	68.91101	78.62350	79.94316	-216.06729	-537.07037	446.51379	1
105	H	68.21403	79.08372	79.27919	-1251.96487	1074.12894	-2168.52500	1
106	H	68.46135	78.52658	80.86483	-2451.83023	-720.73851	944.84612	1
107	C	71.01221	77.50474	75.45701	546.24052	-563.88337	-85.11809	1
108	O	70.25084	76.58342	75.33354	71.70702	-307.40463	606.69673	1
109	N	72.35144	77.52907	75.35596	479.90863	-504.50129	222.86849	1
110	C	73.12822	76.33490	75.26423	75.51268	-600.91904	506.73700	1
111	H	72.46750	75.56171	74.80708	-2033.61562	1795.30103	254.89480	1
112	C	74.34325	76.84251	74.36749	523.79067	517.99274	284.45581	1
113	H	75.22982	76.17126	74.39886	1599.61665	-1127.00911	1895.98173	1
114	H	74.04869	76.92977	73.29769	-1881.83093	1275.82488	-1501.55016	1
115	C	74.60786	78.24236	75.00107	-600.05639	-404.05026	316.64485	1
116	H	75.19752	78.14701	75.94049	-2597.05260	496.36221	657.73249	1
117	H	75.18297	78.92707	74.33843	-1666.24518	1294.38985	-1723.90591	1
118	C	73.13462	78.75627	75.30021	493.57570	-129.95376	-602.56394	1
119	H	73.01872	79.27403	76.28171	-1469.08415	-637.84797	2204.22581	1
120	H	72.66405	79.38140	74.49000	-1038.97840	1068.88701	-2280.71789	1
121	C	73.60783	75.95747	76.78323	188.88100	241.05089	-727.87821	1
122	O	73.02086	76.38483	77.75348	531.88613	-49.19943	427.07960	1
123	N	74.63096	75.11688	76.74488	708.14833	26.93417	-179.73509	1
124	H	75.00928	74.80940	75.85057	-2232.69501	-1179.09831	1023.94816	1
125	C	75.20541	74.69515	77.97135	-738.32412	259.92548	104.42456	1
126	H	74.79664	75.36112	78.76688	2028.89123	1260.02983	1311.33324	1
127	C	74.78005	73.16688	78.14145	-258.65678	-345.52513	661.28459	1
128	H	75.08072	72.58330	77.24487	-1826.06090	-1801.46097	-918.64554	1
129	C	75.45359	72.59439	79.43281	-541.23400	531.92970	-218.41428	1
130	H	75.17404	71.52802	79.60384	1311.73218	-2219.18200	882.19023	1
131	H	76.56725	72.64062	79.36462	-1048.71298	1223.58118	-2196.97971	1
132	H	75.15251	73.16787	80.34083	419.06414	1903.92898	1903.44670	1
133	C	73.22723	73.11510	78.33769	-423.86431	152.66566	-648.55262	1
134	H	72.85868	72.07047	78.46735	105.68386	-2342.50080	1387.51238	1
135	H	72.90972	73.69780	79.23452	-1695.63603	-1484.73621	-1531.02793	1
136	H	72.69494	73.55304	77.46007	-1853.57121	-487.39031	1936.59343	1
137	C	76.83389	74.72168	77.76569	539.71428	-510.58232	267.58633	1
138	O	77.33549	74.22683	76.78765	-15.93758	-278.34085	-624.49402	1
139	N	77.42820	75.18621	78.88029	-676.81886	12.05292	276.17960	1
140	H	76.75153	75.58231	79.53559	-1694.44250	-373.84189	-2100.66098	1
141	C	78.68506	75.88752	78.69511	412.33078	-664.70360	108.35451	1
142	H	78.49712	76.66963	77.92844	1765.77476	1294.44366	1621.76594	1
143	C	79.03090	76.44995	80.12055	-179.66635	-721.95667	-264.73915	1
144	H	78.32553	77.25751	80.42075	-607.34922	2587.87032	-598.10018	1

H	78.89695	75.64043	80.87322	-359.69332	-1479.13954	-2259.74260	1
C	80.51200	76.96508	80.14255	-240.01076	616.02425	431.85322	1
H	81.18321	76.23926	79.63078	-1359.17131	782.54425	2227.99181	1
H	80.62033	77.92538	79.59120	-820.36667	85.81767	-2596.78715	1
C	80.92893	77.11321	81.66010	-513.07015	236.18570	-551.87280	1
H	80.34120	77.91786	82.15869	1816.11944	-1183.11722	-1650.94321	1
H	80.69815	76.17833	82.22100	-1800.04429	-1361.53473	1526.34643	1
C	82.44525	77.40994	81.72639	726.08972	-253.19732	-179.64713	1
H	83.08895	76.61866	81.29276	1224.27271	-456.51798	-2390.90304	1
H	82.74236	78.40063	81.32874	1206.40339	-1150.93514	-2154.90344	1
N	82.84588	77.44599	83.24038	329.70796	-633.68968	-155.67358	1
H	83.87049	77.63302	83.44121	1348.31305	1938.76845	1359.00643	1
H	82.37636	78.17845	83.84823	2096.92035	1446.54551	-966.48890	1
H	82.68338	76.55910	83.80067	-1197.07217	-2303.53719	827.28733	1
C	79.86049	74.84290	78.26819	-378.01186	-556.17301	-413.96481	1
O	80.06850	73.84221	78.91820	-409.44433	-307.55053	-453.30833	1
N	80.61455	75.41440	77.33392	-429.02477	524.70884	274.08502	1
H	80.25499	76.28952	76.95629	1706.34263	-1425.96036	1574.38926	1
C	81.52934	74.67318	76.49751	-45.25864	-626.22952	478.93267	1
H	82.52422	75.14739	76.64962	1988.63164	570.90773	1772.87287	1
C	81.48357	73.15672	76.91902	551.77128	-20.49526	-564.54996	1
H	81.82748	73.04439	77.97766	1434.61535	-1753.10253	1513.99471	1
H	80.42454	72.80257	76.91292	-714.67738	1819.09770	1898.36494	1
C	82.36741	72.26987	75.97709	446.99263	-555.77305	338.97221	1
H	82.00192	72.28793	74.92108	1953.68466	1892.25957	-161.67766	1
H	83.42252	72.62362	75.94907	-403.03785	2688.07032	-188.43591	1
C	82.25545	70.82069	76.59997	567.86262	335.74681	434.04421	1
H	82.60502	70.81030	77.65822	-1080.02275	1568.26081	-1948.78900	1
H	81.19146	70.48989	76.63048	-919.54108	2111.34692	1456.13463	1
C	83.09782	69.86010	75.72948	195.81897	173.33862	-745.11536	1
H	82.77682	69.82250	74.65369	1405.67949	-2301.21089	-390.08113	1
H	84.19339	70.01805	75.79354	-940.37328	-1118.32446	2299.72133	1
N	82.86771	68.41932	76.29424	319.26422	656.36007	-42.02424	1
H	83.40046	67.63087	75.82364	1961.03633	619.91968	1787.09422	1
H	83.12526	68.26709	77.31221	854.14343	-2156.80752	1429.08711	1
H	81.87408	68.04685	76.26576	1237.02905	-1378.50857	-1998.28500	1
C	81.00758	74.84868	74.97237	532.53319	-268.57236	517.55729	1
O	79.83076	74.66063	74.66841	266.16714	-290.73804	-558.88002	1
N	82.01105	75.12721	74.16510	-226.25352	640.49678	-270.32839	1
H	82.95465	75.21922	74.53444	2007.69063	-1035.27229	1523.50231	1
C	81.68279	75.50168	72.81794	-540.76542	-573.33303	49.50000	1
H	80.65399	75.91526	72.94509	-2374.20950	-1036.76023	843.75774	1
C	82.75876	76.55630	72.28349	-77.09082	666.87422	-415.84136	1
H	83.73684	76.02253	72.12636	-985.81834	617.19825	2463.91927	1
O	83.03830	77.66905	73.14591	293.29899	-391.65602	-477.80926	1
H	83.99139	77.52814	73.33494	2528.72714	-681.76262	-751.27122	1
C	82.10408	76.89863	70.89312	252.47363	-605.14473	440.05134	1
H	82.70789	77.64840	70.32064	-2052.65063	1456.49051	-1043.51830	1
H	82.00823	75.98478	70.25692	-2152.98800	-1661.97574	-161.70652	1
H	81.08361	77.32952	71.01928	-473.12726	-1584.27339	2165.61725	1
C	81.67715	74.13376	71.92759	360.36307	488.46713	505.10056	1
O	82.50081	73.89969	71.04864	-200.08817	-528.03552	-385.82850	1
N	80.65431	73.44634	72.35342	-597.54095	-226.44775	355.20479	1
H	80.06911	73.83212	73.13271	2179.23655	-1300.70981	-991.33835	1
C	80.33475	72.16889	71.83534	-687.68921	297.39807	-249.45179	1
H	79.47767	71.86373	72.47512	-349.16256	-2467.37713	-1101.72831	1
C	79.91529	72.36496	70.30009	672.47345	361.54328	-201.62880	1
H	80.67593	72.96903	69.75259	1541.41502	582.18425	2169.97026	1
H	79.82870	71.37853	69.78851	-2534.67717	60.25153	997.72295	1
C	78.55790	73.07974	70.33986	574.26774	-389.43119	-377.02542	1
C	77.41175	72.35699	70.79046	688.82679	-287.33580	-257.96133	1
H	77.48871	71.28810	71.06022	331.14182	1470.38163	2269.80013	1

207	C	78.45334	74.46231	70.00019	-383.52382	-675.04808	144.24917	1
208	H	79.34032	75.02643	69.65933	4.09847	1517.59662	2262.86397	1
209	C	76.19751	73.03245	70.90396	-134.76730	428.45285	-649.50303	1
210	H	75.31452	72.47472	71.26178	-903.23433	1600.49020	2011.53387	1
211	C	77.21165	75.09164	70.12168	-335.53281	-615.00040	-364.39058	1
212	H	77.14026	76.16037	69.85475	1596.45398	-1369.33862	1732.02659	1
213	C	76.04551	74.41255	70.58126	585.48547	-340.24194	-406.23938	1
214	O	74.78508	75.04944	70.72553	-419.15649	-173.04408	-511.94141	1
215	H	75.01186	75.94580	70.38221	-1071.84350	1559.71507	-1960.13012	1
216	C	81.63510	71.20186	72.06328	291.41878	-183.13091	710.72246	1
217	O	81.93037	70.81305	73.17682	379.87695	523.47396	-222.23622	1
218	N	82.21564	70.91693	70.90966	564.30591	373.09380	277.24371	1
219	H	81.84394	71.32346	70.05417	-120.59931	-2436.07824	-1214.35098	1
220	C	83.46022	70.21268	70.92534	404.07514	-299.37480	608.84009	1
221	H	83.81033	70.29592	71.98271	-1071.07882	-841.69025	2359.66467	1
222	C	83.09270	68.70888	70.53141	-683.12807	68.71770	-390.13096	1
223	H	82.26422	68.33797	71.17480	2068.88918	1596.58590	770.89603	1
224	H	82.70341	68.66563	69.49026	1379.86429	2342.49779	-179.85638	1
225	C	84.35393	67.81143	70.68475	-176.39967	537.49466	550.97268	1
226	H	85.17179	68.15804	70.00744	-396.28051	1942.22168	1869.33314	1
227	H	84.76875	67.86868	71.71619	-94.44700	586.00678	-2659.20016	1
228	C	83.89158	66.32235	70.32821	-291.69381	-718.18079	-150.72929	1
229	H	83.15464	65.85531	71.02969	-2303.38776	1089.76242	964.62032	1
230	H	83.50859	66.18402	69.28503	884.16729	2568.80533	-207.74283	1
231	N	85.10012	65.61878	70.43003	-594.49522	-252.81478	342.29276	1
232	H	85.93840	66.17044	70.52803	-1735.17426	2084.05705	-263.71605	1
233	C	85.20719	64.35217	70.38795	595.23177	-271.52487	442.22352	1
234	N	84.03561	63.60198	70.23941	585.53813	-424.79750	-105.81303	1
235	H	83.07237	63.91994	70.14154	959.88797	1327.56823	-2177.11905	1
236	H	83.99624	62.58397	70.18304	1578.48647	16.27378	-2220.76233	1
237	N	86.54853	63.96098	70.50412	-452.17763	-390.33541	-421.51853	1
238	H	87.39883	64.51367	70.60110	470.26107	1974.25629	-1817.92087	1
239	H	86.84984	62.98619	70.48343	1856.67307	-1573.97321	-1224.35416	1
240	C	84.43162	70.88546	69.82595	-531.47271	-581.45780	-55.06206	1
241	O	85.30581	70.22860	69.32735	-243.00837	192.73507	609.52485	1
242	N	84.17274	72.19298	69.63760	343.49288	-644.25607	38.09164	1
243	H	83.39272	72.66038	70.16052	-1691.16043	-1535.97525	-1484.73099	1
244	C	84.76813	72.81665	68.43414	542.66492	350.14337	454.42563	1
245	H	85.57381	72.09216	68.15714	1209.57979	-2396.95920	463.86744	1
246	C	83.70543	72.90792	67.34991	-737.14309	145.93010	242.71942	1
247	H	82.82790	73.44469	67.77223	-1050.18303	159.36419	-2509.06125	1
248	C	84.20379	73.68189	66.14438	-706.77696	-252.88343	-245.16230	1
249	H	83.42314	73.75019	65.33673	-1624.64039	-1665.49085	1417.86935	1
250	H	84.49941	74.73451	66.42047	2566.10521	598.51109	693.22305	1
251	H	85.11236	73.19680	65.69013	-1972.69758	107.10018	-1876.34312	1
252	C	83.24133	71.53052	66.91289	412.85148	672.58832	-27.69222	1
253	H	82.43176	71.58739	66.13377	1447.02277	1762.76533	-1490.78944	1
254	H	84.08782	70.93471	66.47048	1320.77379	-145.20455	2378.68491	1
255	H	82.83864	70.93477	67.77820	-1512.10848	1470.52031	1724.75147	1
256	C	85.38995	74.28822	68.81500	-607.11113	499.56689	73.73769	1
257	O	86.13319	74.80821	68.01618	357.97126	460.81534	356.68785	1
258	N	84.96734	74.78602	69.97189	536.55407	-368.62270	332.76306	1
259	H	84.26075	74.28874	70.54295	524.38866	2297.69985	1367.20941	1
260	C	85.88898	75.69966	70.57923	46.50277	-774.29190	-147.97904	1
261	H	86.85192	75.52986	70.04440	1676.51875	1883.29066	1032.55549	1
262	C	85.35291	77.20801	70.35018	-559.41456	-34.19198	-556.30433	1
263	H	84.23591	77.21683	70.40515	-64.66664	395.18769	2695.05373	1
264	H	85.70577	77.88369	71.16555	-2703.44278	-60.40056	333.79418	1
265	C	85.81719	77.71903	69.01466	-519.76435	377.31045	459.42366	1
266	H	85.64894	76.92188	68.25000	-1746.74323	1667.50644	-1261.73691	1
267	C	85.01898	78.95520	68.61792	570.95554	16.46261	-545.27700	1
268	H	85.35573	79.36686	67.63175	2391.58273	28.05277	-1305.07271	1

```
H    83.92467  78.73215  68.52866      175.75588      917.63458     2559.43867          1
H    85.12878  79.77471  69.37416    -1063.97851    -2155.34650     1283.00462          1
C    87.30128  78.06064  69.04524      193.76014        2.62946     -765.53141          1
H    87.64539  78.49121  68.06967      507.39394     -846.55087     2539.60089          1
H    87.53495  78.81354  69.84158     1337.57841     1818.19054     1526.01994          1
H    87.93140  77.15658  69.24683     1100.88635    -1172.91350    -2199.08909          1
C    85.95883  75.42369  72.15595     -549.21115      475.64486     -309.38218          1
O    85.36595  74.53626  72.71300      556.06106     -390.37755      -78.24250          1
O    86.76105  76.32433  72.90538     -572.27940     -310.19032     -209.75862          1
H    87.15810  76.83641  72.14972    -1380.08193     2212.14486      790.85944          1
```

Listing 61: C00061.xmolout

```
      276
  150.0000  150.0000  150.0000     0.0000     0.0000     0.0000***********    1.1117     4447
      0.2500    300.00    0.00    0.00    0.00   -1293.6252       0.0000    300.0000
H    83.51876  65.34336  81.39617    -2043.14101    -1757.64893      399.89339          1
N    83.61168  65.30731  80.38272      426.75047      490.45733     -334.42467          1
H    82.63503  65.30755  80.09399      442.25559    -1110.30854    -2448.52907          1
C    84.35497  66.38232  79.84253      415.32972     -666.39213       83.73172          1
H    85.13301  66.53827  80.62064      879.32219     2491.00447      667.35213          1
C    84.98596  65.95044  78.42562       31.35357      477.41777      628.23362          1
H    84.18226  65.93996  77.65075     1168.38489     2240.25337    -1019.71136          1
H    85.73156  66.70733  78.08227     -360.43770     2534.90498      931.67112          1
C    85.62102  64.58846  78.53629      463.18208      512.70242     -382.34388          1
H    84.85691  63.88244  78.93364     1725.29117     1863.52247     -987.07854          1
C    86.07046  64.10177  77.16421      710.42801     -144.27427     -313.15367          1
H    86.51143  63.07287  77.21377      364.20195    -1033.13725     2494.72524          1
H    85.21803  64.06409  76.43794     2567.61904      335.80679      847.48802          1
H    86.85050  64.77521  76.72431    -2587.99029      680.92867     -512.16411          1
C    86.80926  64.59665  79.48815      268.17616      741.64379      -40.42634          1
H    87.29953  63.59050  79.54499     1283.54314    -1945.37420    -1411.27812          1
H    87.59254  65.33015  79.16591    -1258.57796     1317.15249    -2026.02085          1
H    86.50138  64.87320  80.52940    -2502.84526      909.80597      575.92391          1
C    83.31565  67.62217  79.75044      619.36578     -458.64843     -172.09273          1
O    82.40227  67.74351  80.54775      -81.40433      433.87611      522.34594          1
N    83.65860  68.45563  78.77580      175.23996     -622.20190      341.55523          1
H    84.44604  68.22366  78.17431     -876.57256     -303.22879    -2561.90213          1
C    82.93695  69.66573  78.60845     -571.88214     -534.10783     -106.15033          1
H    82.33677  69.79385  79.53869    -2713.20841      -16.25800     -248.80809          1
C    84.01947  70.81031  78.30485      166.60354      288.16399     -716.09568          1
H    84.60945  70.49961  77.39875    -2097.85574     1636.78449     -586.18044          1
O    84.98294  71.04993  79.34308     -317.80580     -363.38227      484.43082          1
H    85.81775  70.81626  78.88776     1863.74748    -1451.84923     1357.29467          1
C    83.09814  72.02427  77.91814      503.80731      517.99032      318.51606          1
H    83.70001  72.90544  77.59528      640.61020    -2157.10410    -1536.29104          1
H    82.41183  71.76321  77.07455     1687.88455      465.60692     2087.56452          1
H    82.46723  72.35083  78.77710    -1293.07895    -1899.88581    -1463.57452          1
C    82.05366  69.52568  77.23589     -153.07550     -733.68902      248.70981          1
O    82.61892  69.66692  76.18318      186.15459     -480.78787     -449.34387          1
N    80.75186  69.28118  77.47551     -487.53139      543.18517       42.03850          1
C    79.85665  69.07844  76.38673     -699.35871       69.34393      360.10701          1
H    80.48705  68.87273  75.49066        2.81126     -221.99807    -2715.58072          1
C    78.99633  67.85966  76.93231     -507.46439     -592.59194      122.07761          1
H    78.02695  67.74179  76.39816    -1975.08116      525.87560    -1801.71556          1
H    79.54219  66.89611  76.82573     1588.27743    -1913.22100     1113.83566          1
C    78.82639  68.24759  78.43463     -613.62442     -457.55993     -194.14490          1
H    78.00918  69.01176  78.55356     1707.54637     -721.27992     1996.92523          1
H    78.55131  67.38908  79.08913      758.23357     2100.81767     1560.55011          1
C    80.25002  68.85604  78.77838     -352.41524      704.96076       49.21465          1
H    80.22729  69.74377  79.45375     1714.76886    -2070.42778      443.35792          1
H    81.00563  68.12008  79.17132     1224.15040     1801.50881     1636.97633          1
```

C	78.82656	70.34788	76.16323	566.85117	537.05831	117.62814	1
O	78.18441	70.40879	75.14219	-550.26779	-406.08867	4.21666	1
N	78.81600	71.16745	77.20700	566.34832	-425.77725	180.18749	1
H	79.46582	70.90453	77.94488	644.44502	2646.80550	-52.73430	1
C	77.71551	72.03839	77.47262	612.34801	-281.48662	411.56221	1
H	78.00929	72.45035	78.46388	1667.23498	-1490.75142	1556.16753	1
C	77.68136	73.16241	76.33960	-714.45746	-175.43074	286.98927	1
H	77.33797	72.68050	75.38098	-787.09484	1763.03619	-1922.46060	1
O	78.94052	73.78026	76.00320	-429.46514	516.51932	-128.40526	1
H	79.10153	73.39462	75.11918	1981.82785	436.16847	-1818.18182	1
C	76.59648	74.15318	76.90495	630.79189	-468.94448	76.03230	1
H	76.23717	74.86212	76.12384	-1775.71861	2062.30353	131.89849	1
H	75.69895	73.62851	77.32084	-1855.14608	88.94118	1993.53760	1
H	77.01275	74.77101	77.73409	-1504.82350	-780.94249	-2132.90992	1
C	76.38962	71.10497	77.58952	288.57638	618.62814	-397.00270	1
O	76.38447	70.18588	78.41688	321.70495	-185.26452	574.37218	1
N	75.45422	71.44902	76.74037	-447.05792	-446.53431	367.76425	1
H	75.70515	72.18197	76.07952	350.31316	1975.63644	-1843.31522	1
C	74.06313	71.27341	77.00511	-511.23061	374.03917	-471.51480	1
H	73.59504	71.78844	76.13494	2234.79579	-898.70890	-1273.45190	1
C	73.77674	69.70705	77.01213	529.53781	-159.34528	563.72604	1
H	72.70769	69.52182	76.72490	654.59388	2347.94251	1217.51488	1
H	74.40294	69.27095	76.18951	-1526.55661	2103.42026	817.87394	1
O	74.03399	69.00039	78.24690	-554.84278	14.12789	399.58769	1
H	74.99015	69.34373	78.41867	1297.06236	2061.84854	1220.68812	1
C	73.70539	72.01243	78.40968	-662.15484	-412.13544	123.62687	1
O	74.50290	72.76458	78.97869	-540.01537	-317.05804	274.91456	1
N	72.44562	71.78553	78.72568	-202.76299	-690.00100	-131.49145	1
H	72.04374	71.14112	78.04768	-702.62132	-2617.37191	281.70413	1
C	71.85370	71.98591	80.03759	-491.73914	576.34187	222.73509	1
H	70.88220	71.44192	79.96427	1977.17978	-1823.58850	-434.68928	1
C	72.78841	71.40737	81.09229	-649.50797	-328.66843	306.11224	1
H	73.80836	71.81680	80.90531	2075.07803	1452.16490	-1004.45904	1
C	72.36555	71.80762	82.49329	-615.18735	-489.99689	-70.96352	1
H	73.06731	71.40066	83.27275	-1226.32661	-1724.38906	-1716.47170	1
H	72.34163	72.92636	82.61399	2381.64273	-969.84735	900.46860	1
H	71.33631	71.42589	82.73924	2122.74991	1702.23946	-141.36228	1
C	72.87826	69.89755	80.99231	-501.86074	-479.61276	-376.42482	1
H	73.58819	69.47073	81.75364	993.79261	-2246.95401	-1177.81305	1
H	71.87840	69.40959	81.15933	42.71279	-626.15391	-2651.37258	1
H	73.24825	69.57097	79.96363	-2026.39012	-1590.33709	-887.82922	1
C	71.61485	73.61334	80.30491	-672.89153	382.57394	-156.34072	1
O	70.50245	73.99394	80.56106	410.04627	221.86223	500.35950	1
N	72.74195	74.29790	80.27633	-378.11782	-443.79414	-441.10984	1
H	73.64720	73.85004	79.97676	2404.15973	-568.14894	1149.30085	1
C	72.64647	75.71140	80.39607	-544.54484	466.16019	331.29119	1
H	71.59581	75.92716	80.70074	-1409.80988	-170.17955	2325.32679	1
C	73.75196	76.13551	81.45506	-184.48284	116.78282	-758.89137	1
H	73.64201	75.54044	82.38974	1893.78641	1861.14652	611.04528	1
H	74.77228	75.91219	81.06827	-2170.79665	-974.19311	1327.50117	1
C	73.57950	77.65878	81.73792	389.51668	672.81878	-138.49152	1
H	73.74564	78.24220	80.80397	510.64577	1933.03327	1851.02484	1
H	72.52450	77.88137	82.01141	-2302.30869	-1105.72939	-948.89774	1
C	74.53348	78.18800	82.87554	-559.93427	-126.34590	542.30857	1
O	74.47093	79.34371	83.20635	-670.36003	-111.83166	76.36448	1
N	75.35494	77.25103	83.39575	-216.06729	-537.07037	446.51379	1
H	75.46577	76.26172	83.14467	-1251.96487	1074.12894	-2168.52500	1
H	76.03881	77.43792	84.13734	-2451.83023	-720.73851	944.84612	1
C	73.09607	76.33054	78.94095	546.24052	-563.88337	-85.11809	1
O	74.24040	76.24134	78.58325	71.70702	-307.40463	606.69673	1
N	72.04298	76.85851	78.30108	479.90863	-504.50129	222.86849	1
C	72.21637	77.52434	77.04541	75.51268	-600.91904	506.73700	1

H	73.18053	77.11321	76.66440	-2033.61562	1795.30103	254.89480	1
C	70.91351	77.02470	76.28432	523.79067	517.99274	284.45581	1
H	70.67257	77.62321	75.37770	1599.61665	-1127.00911	1895.98173	1
H	71.03556	75.97202	75.94589	-1881.83093	1275.82488	-1501.55016	1
C	69.83817	77.11627	77.41036	-600.05639	-404.05026	316.64485	1
H	69.49705	78.16457	77.56093	-2597.05260	496.36221	657.73249	1
H	68.92359	76.51855	77.19861	-1666.24518	1294.38985	-1723.90591	1
C	70.65951	76.57486	78.65896	493.57570	-129.95376	-602.56394	1
H	70.43707	77.09469	79.62047	-1469.08415	-637.84797	2204.22581	1
H	70.60513	75.46942	78.81764	-1038.97840	1068.88701	-2280.71789	1
C	72.32884	79.12849	77.34424	188.88100	241.05089	-727.87821	1
O	73.24947	79.52245	78.05680	531.88613	-49.19943	427.07960	1
N	71.43059	79.83953	76.70436	708.14833	26.93417	-179.73509	1
H	70.79532	79.27349	76.14411	-2232.69501	-1179.09831	1023.94816	1
C	71.06952	81.20811	77.02550	-738.32412	259.92548	104.42456	1
H	70.07216	81.34540	76.51648	2028.89123	1260.02983	1311.33324	1
C	71.00399	81.40914	78.53376	-258.65678	-345.52513	661.28459	1
H	71.98229	81.09549	78.96784	-1826.06090	-1801.46097	-918.64554	1
C	70.77845	82.87027	78.87494	-541.23400	531.92970	-218.41428	1
H	70.75487	83.04265	79.98631	1311.73218	-2219.18200	882.19023	1
H	71.59153	83.52337	78.45121	-1048.71298	1223.58118	-2196.97971	1
H	69.80328	83.24528	78.45672	419.06414	1903.92898	1903.44670	1
C	69.92814	80.55208	79.17344	-423.86431	152.66566	-648.55262	1
H	69.85506	80.73370	80.28141	105.68386	-2342.50080	1387.51238	1
H	68.91494	80.76471	78.73190	-1695.63603	-1484.73621	-1531.02793	1
H	70.13609	79.45679	79.02685	-1853.57121	-487.39031	1936.59343	1
C	72.21218	82.24458	76.38211	539.71428	-510.58232	267.58633	1
O	71.85239	83.14975	75.67877	-15.93758	-278.34085	-624.49402	1
N	73.41828	81.93185	76.80570	-676.81886	12.05292	276.17960	1
H	73.56285	81.10440	77.43268	-1694.44250	-373.84189	-2100.66098	1
C	74.51732	82.73001	76.34874	412.33078	-664.70360	108.35451	1
H	74.23846	83.78370	76.57450	1765.77476	1294.44366	1621.76594	1
C	75.78176	82.19419	77.12533	-179.66635	-721.95667	-264.73915	1
H	75.65069	82.28991	78.22694	-607.34922	2587.87032	-598.10018	1
H	75.90929	81.10355	76.93623	-359.69332	-1479.13954	-2259.74260	1
C	77.03760	82.98778	76.63052	-240.01076	616.02425	431.85322	1
H	77.06409	83.01744	75.51643	-1359.17131	782.54425	2227.99181	1
H	77.00517	84.04868	76.96537	-820.36667	85.81767	-2596.78715	1
C	78.31492	82.24254	77.18900	-513.07015	236.18570	-551.87280	1
H	78.36600	82.31007	78.30017	1816.11944	-1183.11722	-1650.94321	1
H	78.27126	81.15406	76.95295	-1800.04429	-1361.53473	1526.34643	1
C	79.55499	82.88904	76.52754	726.08972	-253.19732	-179.64713	1
H	79.58302	82.80215	75.42277	1224.27271	-456.51798	-2390.90304	1
H	79.75020	83.93773	76.82939	1206.40339	-1150.93514	-2154.90344	1
N	80.80855	82.08725	77.01054	329.70796	-633.68968	-155.67358	1
H	81.74023	82.40202	76.61271	1348.31305	1938.76845	1359.00643	1
H	81.01405	82.08269	78.05197	2096.92035	1446.54551	-966.48890	1
H	80.82128	81.05218	76.77848	-1197.07217	-2303.53719	827.28733	1
C	74.72616	82.52468	74.73704	-378.01186	-556.17301	-413.96481	1
O	75.30066	83.38798	74.11080	-409.44433	-307.55053	-453.30833	1
N	74.41127	81.27142	74.32945	-429.02477	524.70884	274.08502	1
H	73.96054	80.84426	75.14374	1706.34263	-1425.96036	1574.38926	1
C	73.75029	81.10587	73.05012	-45.25864	-626.22952	478.93267	1
H	72.67232	81.07675	73.31985	1988.63164	570.90773	1772.87287	1
C	74.13834	82.26838	72.07361	551.77128	-20.49526	-564.54996	1
H	73.73948	83.24109	72.44834	1434.61535	-1753.10253	1513.99471	1
H	75.24865	82.38497	72.06649	-714.67738	1819.09770	1898.36494	1
C	73.61416	81.98774	70.62090	446.99263	-555.77305	338.97221	1
H	74.00465	81.01437	70.24347	1953.68466	1892.25957	-161.67766	1
H	72.50431	81.91300	70.59036	-403.03785	2688.07032	-188.43591	1
C	74.14137	83.18437	69.73350	567.86262	335.74681	434.04421	1
H	73.64648	84.14008	70.02266	-1080.02275	1568.26081	-1948.78900	1

173	H	75.23065	83.34642	69.90465	-919.54108	2111.34692	1456.13463	1
174	C	73.88216	82.85395	68.24509	195.81897	173.33862	-745.11536	1
175	H	74.42854	81.96599	67.86889	1405.67949	-2301.21089	-390.08113	1
176	H	72.81268	82.78964	67.96110	-940.37328	-1118.32446	2299.72133	1
177	N	74.45047	84.04722	67.40412	319.26422	656.36007	-42.02424	1
178	H	74.35846	83.95461	66.35148	1961.03633	619.91968	1787.09422	1
179	H	74.01905	85.00301	67.56820	854.14343	-2156.80752	1429.08711	1
180	H	75.48660	84.25514	67.50452	1237.02905	-1378.50857	-1998.28500	1
181	C	74.29109	79.67068	72.50404	532.53319	-268.57236	517.55729	1
182	O	75.13554	79.60209	71.63555	266.16714	-290.73804	-558.88002	1
183	N	73.66323	78.66232	73.11736	-226.25352	640.49678	-270.32839	1
184	H	73.02892	78.89183	73.88313	2007.69063	-1035.27229	1523.50231	1
185	C	74.25387	77.36364	72.99637	-540.76542	-573.33303	49.50000	1
186	H	74.66222	77.38946	71.95356	-2374.20950	-1036.76023	843.75774	1
187	C	75.41011	77.20600	74.07825	-77.09082	666.87422	-415.84136	1
188	H	74.99245	76.98521	75.10133	-985.81834	617.19825	2463.91927	1
189	O	76.24038	78.36958	74.32576	293.29899	-391.65602	-477.80926	1
190	H	76.00877	78.91602	73.54328	2528.72714	-681.76262	-751.27122	1
191	C	76.14568	75.92019	73.55740	252.47363	-605.14473	440.05134	1
192	H	76.99688	75.63783	74.22120	-2052.65063	1456.49051	-1043.51830	1
193	H	75.45487	75.04293	73.50361	-2152.98800	-1661.97574	-161.70652	1
194	H	76.56582	76.07538	72.52973	-473.12726	-1584.27339	2165.61725	1
195	C	73.05464	76.28054	73.24638	360.36307	488.46713	505.10056	1
196	O	73.21671	75.27843	73.90244	-200.08817	-528.03552	-385.82850	1
197	N	71.98457	76.66228	72.54537	-597.54095	-226.44775	355.20479	1
198	H	72.06222	77.56521	72.07731	2179.23655	-1300.70981	-991.33835	1
199	C	70.72433	76.07259	72.86805	-687.68921	297.39807	-249.45179	1
200	H	70.73105	75.90979	73.96618	-349.16256	-2467.37713	-1101.72831	1
201	C	69.57054	77.03815	72.34262	672.47345	361.54328	-201.62880	1
202	H	69.77831	77.33167	71.28839	1541.41502	582.18425	2169.97026	1
203	H	68.60870	76.47478	72.34163	-2534.67717	60.25153	997.72295	1
204	C	69.42524	78.28927	73.22424	574.26774	-389.43119	-377.02542	1
205	C	68.60595	78.20012	74.38987	688.82679	-287.33580	-257.96133	1
206	H	68.17564	77.23257	74.70508	331.14182	1470.38163	2269.80013	1
207	C	70.02401	79.53150	72.85394	-383.52382	-675.04808	144.24917	1
208	H	70.68972	79.59732	71.97471	4.09847	1517.59662	2262.86397	1
209	C	68.33006	79.37011	75.10299	-134.76730	428.45285	-649.50303	1
210	H	67.67869	79.30697	75.99230	-903.23433	1600.49020	2011.53387	1
211	C	69.71216	80.67561	73.60083	-335.53281	-615.00040	-364.39058	1
212	H	70.15518	81.63967	73.29335	1596.45398	-1369.33862	1732.02659	1
213	C	68.83755	80.64424	74.72327	585.48547	-340.24194	-406.23938	1
214	O	68.47633	81.81504	75.47551	-419.15649	-173.04408	-511.94141	1
215	H	68.98469	82.48619	74.96569	-1071.84350	1559.71507	-1960.13012	1
216	C	70.51920	74.66814	72.07834	291.41878	-183.13091	710.72246	1
217	O	69.63326	73.91157	72.42212	379.87695	523.47396	-222.23622	1
218	N	71.26188	74.58457	70.92534	564.30591	373.09380	277.24371	1
219	H	71.96433	75.31686	71.08108	-120.59931	-2436.07824	-1214.35098	1
220	C	71.71381	73.23427	70.62390	404.07514	-299.37480	608.84009	1
221	H	72.18755	72.86190	71.55795	-1071.07882	-841.69025	2359.66467	1
222	C	70.43831	72.41210	70.21211	-683.12807	68.71770	-390.13096	1
223	H	69.66957	72.44337	71.03218	2068.88918	1596.58590	770.89603	1
224	H	69.95027	72.88401	69.32840	1379.86429	2342.49779	-179.85638	1
225	C	70.84189	70.93705	69.88913	-176.39967	537.49466	550.97268	1
226	H	71.57988	70.89303	69.05689	-396.28051	1942.22168	1869.33314	1
227	H	71.33734	70.44907	70.75749	-94.44700	586.00678	-2659.20016	1
228	C	69.47317	70.20720	69.50866	-291.69381	-718.18079	-150.72929	1
229	H	68.72767	70.11641	70.33940	-2303.38776	1089.76242	964.62032	1
230	H	68.93857	70.63497	68.62228	884.16729	2568.80533	-207.74283	1
231	N	69.88156	68.91249	69.16195	-594.49522	-252.81478	342.29276	1
232	H	70.87427	68.73813	69.14433	-1735.17426	2084.05705	-263.71605	1
233	C	69.08854	67.96467	68.86470	595.23177	-271.52487	442.22352	1
234	N	67.71500	68.23231	68.89031	585.53813	-424.79750	-105.81303	1

```
H    67.22808   69.09735   69.12145      959.88797     1327.56823    -2177.11905          1
H    66.98369   67.55438   68.67392     1578.48647       16.27378    -2220.76233          1
N    69.79266   66.79252   68.55526     -452.17763     -390.33541     -421.51853          1
H    70.79269   66.59824   68.53493      470.26107     1974.25629    -1817.92087          1
H    69.33338   65.92052   68.28932     1856.67307    -1573.97321    -1224.35416          1
C    72.73264   73.29475   69.37011     -531.47271     -581.45780      -55.06206          1
O    73.58209   72.44552   69.23370     -243.00837      192.73507      609.52485          1
N    72.46335   74.34074   68.55494      343.49288     -644.25607       38.09164          1
H    71.62247   74.84765   68.83704    -1691.16043    -1535.97525    -1484.73099          1
C    72.77692   74.12670   67.15574      542.66492      350.14337      454.42563          1
H    72.50022   73.07032   66.94402     1209.57979    -2396.95920      463.86744          1
C    71.91667   75.17656   66.33902     -737.14309      145.93010      242.71942          1
H    71.90341   76.15969   66.85740    -1050.18303      159.36419    -2509.06125          1
C    72.50681   75.31581   64.89334     -706.77696     -252.88343     -245.16230          1
H    71.90347   76.01283   64.26557    -1624.64039    -1665.49085     1417.86935          1
H    73.55183   75.70670   64.91749     2566.10521      598.51109      693.22305          1
H    72.53811   74.33275   64.36778    -1972.69758      107.10018    -1876.34312          1
C    70.46472   74.60108   66.19339      412.85148      672.58832      -27.69222          1
H    69.80037   75.29884   65.63210     1447.02277     1762.76533    -1490.78944          1
H    70.46444   73.63061   65.64402     1320.77379     -145.20455     2378.68491          1
H    69.99335   74.41876   67.18733    -1512.10848     1470.52031     1724.75147          1
C    74.35886   74.36110   66.94811     -607.11113      499.56689       73.73769          1
O    74.91882   73.83819   66.01092      357.97126      460.81534      356.68785          1
N    74.84546   75.27554   67.80794      536.55407     -368.62270      332.76306          1
H    74.18916   75.59774   68.54129      524.38866     2297.69985     1367.20941          1
C    76.14361   74.93584   68.36620       46.50277     -774.29190     -147.97904          1
H    76.13145   73.82545   68.43265     1676.51875     1883.29066     1032.55549          1
C    77.31010   75.46349   67.38919     -559.41456      -34.19198     -556.30433          1
H    76.91283   76.28488   66.74576      -64.66664      395.18769     2695.05373          1
H    78.15776   75.90252   67.96826    -2703.44278      -60.40056      333.79418          1
C    77.78568   74.30407   66.55520     -519.76435      377.31045      459.42366          1
H    76.87560   73.80514   66.10712    -1746.74323     1667.50644    -1261.73691          1
C    78.67833   74.78074   65.41719      570.95554       16.46261     -545.27700          1
H    79.03797   73.92437   64.78969     2391.58273       28.05277    -1305.07271          1
H    78.13768   75.48685   64.73528      175.75588      917.63458     2559.43867          1
H    79.58580   75.31546   65.79959    -1063.97851    -2155.34650     1283.00462          1
C    78.54930   73.29760   67.40513      193.76014        2.62946     -765.53141          1
H    78.89061   72.41978   66.79813      507.39394     -846.55087     2539.60089          1
H    79.46196   73.75689   67.86551     1337.57841     1818.19054     1526.01994          1
H    77.92416   72.89512   68.24306     1100.88635    -1172.91350    -2199.08909          1
C    76.11052   75.65607   69.77842     -549.21115      475.64486     -309.38218          1
O    75.08325   76.10392   70.24007      556.06106     -390.37755      -78.24250          1
O    77.30689   75.69028   70.53490     -572.27940     -310.19032     -209.75862          1
H    77.90638   75.21787   69.89336    -1380.08193     2212.14486      790.85944          1
```

Listing 62: C00062.xmolout

```
        276
   150.0000  150.0000  150.0000     0.0000     0.0000     0.0000***********    2.7750    11100
        0.2500     300.00    0.00     0.00    0.00   -1293.6237       0.0000    300.0000
H    56.91105   76.10418   68.20016    -2043.14101    -1757.64893      399.89339          1
N    57.44298   76.31099   69.04273      426.75047      490.45733     -334.42467          1
H    56.70990   76.56972   69.69955      442.25559    -1110.30854    -2448.52907          1
C    58.47584   77.25812   68.89167      415.32972     -666.39213       83.73172          1
H    58.94971   76.96582   67.93033      879.32219     2491.00447      667.35213          1
C    57.86162   78.74344   68.83012       31.35357      477.41777      628.23362          1
H    57.45543   79.01552   69.83401     1168.38489     2240.25337    -1019.71136          1
H    58.68321   79.47334   68.63209     -360.43770     2534.90498      931.67112          1
C    56.78174   78.85833   67.78390      463.18208      512.70242     -382.34388          1
H    55.97233   78.13487   68.02905     1725.29117     1863.52247     -987.07854          1
C    56.18879   80.26201   67.79173      710.42801     -144.27427     -313.15367          1
H    55.35713   80.36430   67.04759      364.20195    -1033.13725     2494.72524          1
```

H	55.77168	80.53091	68.79643	2567.61904	335.80679	847.48802	1
H	56.95863	81.03491	67.53524	-2587.99029	680.92867	-512.16411	1
C	57.30741	78.54065	66.39099	268.17616	741.64379	-40.42634	1
H	56.51055	78.66089	65.61203	1283.54314	-1945.37420	-1411.27812	1
H	58.15423	79.21699	66.10605	-1258.57796	1317.15249	-2026.02085	1
H	57.68403	77.48801	66.31771	-2502.84526	909.80597	575.92391	1
C	59.49019	77.16854	70.15521	619.36578	-458.64843	-172.09273	1
O	60.14366	78.13315	70.49232	-81.40433	433.87611	522.34594	1
N	59.51495	75.94595	70.68948	175.23996	-622.20190	341.55523	1
H	58.94195	75.23208	70.24069	-876.57256	-303.22879	-2561.90213	1
C	60.46814	75.68198	71.71283	-571.88214	-534.10783	-106.15033	1
H	60.61822	76.66767	72.20867	-2713.20841	-16.25800	-248.80809	1
C	59.82936	74.53415	72.62647	166.60354	288.16399	-716.09568	1
H	59.61817	73.64642	71.96892	-2097.85574	1636.78449	-586.18044	1
O	58.57569	74.86703	73.25212	-317.80580	-363.38227	484.43082	1
H	57.97433	74.22738	72.82272	1863.74748	-1451.84923	1357.29467	1
C	61.00270	74.17494	73.60927	503.80731	517.99032	318.51606	1
H	60.75589	73.27889	74.22498	640.61020	-2157.10410	-1536.29104	1
H	61.95332	73.95226	73.06687	1687.88455	465.60692	2087.56452	1
H	61.21095	75.00958	74.31776	-1293.07895	-1899.88581	-1463.57452	1
C	61.80906	75.10912	70.97471	-153.07550	-733.68902	248.70981	1
O	61.70118	74.24083	70.15053	186.15459	-480.78787	-449.34387	1
N	62.91878	75.66051	71.48776	-487.53139	543.18517	42.03850	1
C	64.21711	75.28127	71.02356	-699.35871	69.34393	360.10701	1
H	64.13517	75.05849	69.93567	2.81126	-221.99807	-2715.58072	1
C	65.08691	76.54875	71.42142	-507.46439	-592.59194	122.07761	1
H	66.17566	76.32272	71.48717	-1975.08116	525.87560	-1801.71556	1
H	64.97463	77.37631	70.68576	1588.27743	-1913.22100	1113.83566	1
C	64.44678	76.91652	72.79473	-613.62442	-457.55993	-194.14490	1
H	64.75198	76.18597	73.58113	1707.54637	-721.27992	1996.92523	1
H	64.71862	77.93072	73.16496	758.23357	2100.81767	1560.55011	1
C	62.90536	76.76401	72.43973	-352.41524	704.96076	49.21465	1
H	62.24816	76.49532	73.29888	1714.76886	-2070.42778	443.35792	1
H	62.46371	77.63135	71.89206	1224.15040	1801.50881	1636.97633	1
C	64.77866	74.00993	71.90143	566.85117	537.05831	117.62814	1
O	64.64174	73.95431	73.09995	-550.26779	-406.08867	4.21666	1
N	65.50164	73.23944	71.09792	566.34832	-425.77725	180.18749	1
H	65.53155	73.47018	70.10587	644.44502	2646.80550	-52.73430	1
C	65.97855	71.99259	71.57928	612.34801	-281.48662	411.56221	1
H	65.79866	72.05468	72.67806	1667.23498	-1490.75142	1556.16753	1
C	65.14791	70.83604	70.84883	-714.45746	-175.43074	286.98927	1
H	65.37576	70.88873	69.74962	-787.09484	1763.03619	-1922.46060	1
O	63.71777	70.92371	70.97690	-429.46514	516.51932	-128.40526	1
H	63.45419	71.12132	70.05668	1981.82785	436.16847	-1818.18182	1
C	65.78387	69.53006	71.45130	630.79189	-468.94448	76.03230	1
H	65.35954	68.61674	70.97332	-1775.71861	2062.30353	131.89849	1
H	66.89090	69.49346	71.30548	-1855.14608	88.94118	1993.53760	1
H	65.58890	69.45015	72.54579	-1504.82350	-780.94249	-2132.90992	1
C	67.55433	71.90604	71.13798	288.57638	618.62814	-397.00270	1
O	67.93976	71.14719	70.27783	321.70495	-185.26452	574.37218	1
N	68.29341	72.72725	71.89280	-447.05792	-446.53431	367.76425	1
H	67.78842	73.40094	72.46922	350.31316	1975.63644	-1843.31522	1
C	69.61268	72.96771	71.38694	-511.23061	374.03917	-471.51480	1
H	69.94608	71.96389	71.03397	2234.79579	-898.70890	-1273.45190	1
C	69.45390	74.03544	70.21126	529.53781	-159.34528	563.72604	1
H	70.46225	74.12236	69.72389	654.59388	2347.94251	1217.51488	1
H	68.77964	73.62484	69.41489	-1526.55661	2103.42026	817.87394	1
O	68.99992	75.33574	70.62488	-554.84278	14.12789	399.58769	1
H	69.50778	75.40538	71.51296	1297.06236	2061.84854	1220.68812	1
C	70.50525	73.65547	72.56235	-662.15484	-412.13544	123.62687	1
O	70.40534	74.85693	72.80437	-540.01537	-317.05804	274.91456	1
N	71.28209	72.75788	73.11357	-202.76299	-690.00100	-131.49145	1

H	71.21039	71.78422	72.82006	-702.62132	-2617.37191	281.70413	1
C	72.19192	73.13494	74.14148	-491.73914	576.34187	222.73509	1
H	72.55332	74.15617	73.88650	1977.17978	-1823.58850	-434.68928	1
C	71.36845	73.12737	75.50229	-649.50797	-328.66843	306.11224	1
H	70.91342	72.12897	75.67887	2075.07803	1452.16490	-1004.45904	1
C	72.34644	73.50581	76.66544	-615.18735	-489.99689	-70.96352	1
H	71.82621	73.52283	77.65205	-1226.32661	-1724.38906	-1716.47170	1
H	73.18532	72.77390	76.74619	2381.64273	-969.84735	900.46860	1
H	72.79958	74.51285	76.51067	2122.74991	1702.23946	-141.36228	1
C	70.25613	74.22781	75.40185	-501.86074	-479.61276	-376.42482	1
H	69.75802	74.40827	76.38384	993.79261	-2246.95401	-1177.81305	1
H	70.67476	75.20113	75.04669	42.71279	-626.15391	-2651.37258	1
H	69.46296	73.93664	74.67063	-2026.39012	-1590.33709	-887.82922	1
C	73.33680	71.95885	74.21512	-672.89153	382.57394	-156.34072	1
O	73.01230	70.79175	74.22484	410.04627	221.86223	500.35950	1
N	74.52055	72.51478	74.40992	-378.11782	-443.79414	-441.10984	1
H	74.53723	73.53300	74.43653	2404.15973	-568.14894	1149.30085	1
C	75.75263	71.79667	74.31743	-544.54484	466.16019	331.29119	1
H	76.40280	72.30007	75.06484	-1409.80988	-170.17955	2325.32679	1
C	75.47510	70.27115	74.61466	-184.48284	116.78282	-758.89137	1
H	74.80504	70.17577	75.50213	1893.78641	1861.14652	611.04528	1
H	74.91475	69.81033	73.76271	-2170.79665	-974.19311	1327.50117	1
C	76.83205	69.53018	74.83934	389.51668	672.81878	-138.49152	1
H	77.45527	69.57866	73.91801	510.64577	1933.03327	1851.02484	1
H	77.44375	70.05570	75.60697	-2302.30869	-1105.72939	-948.89774	1
C	76.61787	68.02809	75.26465	-559.93427	-126.34590	542.30857	1
O	77.57348	67.32823	75.48193	-670.36003	-111.83166	76.36448	1
N	75.32160	67.65811	75.35632	-216.06729	-537.07037	446.51379	1
H	74.46584	68.19320	75.16687	-1251.96487	1074.12894	-2168.52500	1
H	75.01317	66.71454	75.61431	-2451.83023	-720.73851	944.84612	1
C	76.32649	71.94413	72.79116	546.24052	-563.88337	-85.11809	1
O	75.82488	71.31227	71.89739	71.70702	-307.40463	606.69673	1
N	77.34682	72.81732	72.73325	479.90863	-504.50129	222.86849	1
C	78.00601	73.08515	71.49420	75.51268	-600.91904	506.73700	1
H	77.33878	72.72863	70.67328	-2033.61562	1795.30103	254.89480	1
C	78.19102	74.66574	71.63110	523.79067	517.99274	284.45581	1
H	78.93062	75.08346	70.90866	1599.61665	-1127.00911	1895.98173	1
H	77.23239	75.19810	71.44768	-1881.83093	1275.82488	-1501.55016	1
C	78.63560	74.80014	73.11742	-600.05639	-404.05026	316.64485	1
H	79.71227	74.54444	73.23764	-2597.05260	496.36221	657.73249	1
H	78.50986	75.82870	73.52662	-1666.24518	1294.38985	-1723.90591	1
C	77.69578	73.72477	73.81770	493.57570	-129.95376	-602.56394	1
H	78.19761	73.11419	74.60589	-1469.08415	-637.84797	2204.22581	1
H	76.73388	74.12965	74.21129	-1038.97840	1068.88701	-2280.71789	1
C	79.50174	72.44507	71.46478	188.88100	241.05089	-727.87821	1
O	79.99168	71.81816	72.39002	531.88613	-49.19943	427.07960	1
N	80.04845	72.70744	70.27584	708.14833	26.93417	-179.73509	1
H	79.45310	73.31941	69.71974	-2232.69501	-1179.09831	1023.94816	1
C	81.51066	72.69255	70.11700	-738.32412	259.92548	104.42456	1
H	81.76260	71.68752	70.52895	2028.89123	1260.02983	1311.33324	1
C	81.87357	72.89328	68.65904	-258.65678	-345.52513	661.28459	1
H	81.46932	73.88096	68.34086	-1826.06090	-1801.46097	-918.64554	1
C	83.38043	72.92335	68.47405	-541.23400	531.92970	-218.41428	1
H	83.66675	73.10598	67.40219	1311.73218	-2219.18200	882.19023	1
H	83.84818	73.74207	69.09167	-1048.71298	1223.58118	-2196.97971	1
H	83.85721	71.95391	68.78675	419.06414	1903.92898	1903.44670	1
C	81.26034	71.83226	67.76371	-423.86431	152.66566	-648.55262	1
H	81.50088	72.01182	66.67982	105.68386	-2342.50080	1387.51238	1
H	81.63683	70.80491	68.02353	-1695.63603	-1484.73621	-1531.02793	1
H	80.14018	71.80840	67.86152	-1853.57121	-487.39031	1936.59343	1
C	82.13386	73.91175	71.05092	539.71428	-510.58232	267.58633	1
O	82.56142	74.92626	70.56505	-15.93758	-278.34085	-624.49402	1

N	82.04467	73.56791	72.33031	-676.81886	12.05292	276.17960	1
H	81.60593	72.65428	72.56554	-1694.44250	-373.84189	-2100.66098	1
C	83.03417	74.15983	73.19749	412.33078	-664.70360	108.35451	1
H	84.00129	74.08068	72.65418	1765.77476	1294.44366	1621.76594	1
C	82.61341	75.66150	73.42998	-179.66635	-721.95667	-264.73915	1
H	82.52675	76.20402	72.45896	-607.34922	2587.87032	-598.10018	1
H	81.60386	75.71365	73.89863	-359.69332	-1479.13954	-2259.74260	1
C	83.68948	76.31014	74.36591	-240.01076	616.02425	431.85322	1
H	83.85956	75.66575	75.26157	-1359.17131	782.54425	2227.99181	1
H	84.67477	76.40168	73.85665	-820.36667	85.81767	-2596.78715	1
C	83.11344	77.72145	74.78268	-513.07015	236.18570	-551.87280	1
H	82.97339	78.37106	73.88721	1816.11944	-1183.11722	-1650.94321	1
H	82.09362	77.58068	75.23919	-1800.04429	-1361.53473	1526.34643	1
C	84.03808	78.40364	75.81626	726.08972	-253.19732	-179.64713	1
H	84.07909	77.89770	76.80179	1224.27271	-456.51798	-2390.90304	1
H	85.06330	78.62300	75.45632	1206.40339	-1150.93514	-2154.90344	1
N	83.39951	79.80268	76.11925	329.70796	-633.68968	-155.67358	1
H	83.89545	80.38927	76.85003	1348.31305	1938.76845	1359.00643	1
H	83.33554	80.48856	75.31181	2096.92035	1446.54551	-966.48890	1
H	82.40282	79.81105	76.48841	-1197.07217	-2303.53719	827.28733	1
C	82.99779	73.37413	74.63056	-378.01186	-556.17301	-413.96481	1
O	84.00658	73.34937	75.30376	-409.44433	-307.55053	-453.30833	1
N	81.76459	73.00974	74.93027	-429.02477	524.70884	274.08502	1
H	81.06365	73.16733	74.20763	1706.34263	-1425.96036	1574.38926	1
C	81.40574	72.14733	76.02492	-45.25864	-626.22952	478.93267	1
H	81.25486	71.14368	75.56609	1988.63164	570.90773	1772.87287	1
C	82.55045	72.17170	77.10919	551.77128	-20.49526	-564.54996	1
H	83.51351	71.81230	76.66506	1434.61535	-1753.10253	1513.99471	1
H	82.75044	73.22052	77.42951	-714.67738	1819.09770	1898.36494	1
C	82.17116	71.27519	78.33646	446.99263	-555.77305	338.97221	1
H	81.24924	71.64468	78.83979	1953.68466	1892.25957	-161.67766	1
H	81.95276	70.23274	78.01366	-403.03785	2688.07032	-188.43591	1
C	83.41867	71.32005	79.30836	567.86262	335.74681	434.04421	1
H	84.36069	71.12317	78.74640	-1080.02275	1568.26081	-1948.78900	1
H	83.53827	72.33734	79.74786	-919.54108	2111.34692	1456.13463	1
C	83.23307	70.25980	80.41742	195.81897	173.33862	-745.11536	1
H	82.39050	70.45690	81.10850	1405.67949	-2301.21089	-390.08113	1
H	83.18508	69.21409	80.05378	-940.37328	-1118.32446	2299.72133	1
N	84.51989	70.29997	81.31242	319.26422	656.36007	-42.02424	1
H	84.54023	69.63995	82.14382	1961.03633	619.91968	1787.09422	1
H	85.43027	70.04902	80.83021	854.14343	-2156.80752	1429.08711	1
H	84.76790	71.22541	81.76948	1237.02905	-1378.50857	-1998.28500	1
C	80.00194	72.75136	76.57100	532.53319	-268.57236	517.55729	1
O	79.04869	72.02065	76.82397	266.16714	-290.73804	-558.88002	1
N	80.12960	74.04232	76.74527	-226.25352	640.49678	-270.32839	1
H	81.02679	74.52128	76.50915	2007.69063	-1035.27229	1523.50231	1
C	78.99659	74.91998	76.80108	-540.76542	-573.33303	49.50000	1
H	78.21167	74.54433	76.11056	-2374.20950	-1036.76023	843.75774	1
C	78.49246	74.83068	78.31271	-77.09082	666.87422	-415.84136	1
H	79.39566	74.83444	78.99782	-985.81834	617.19825	2463.91927	1
O	77.85093	73.58238	78.65370	293.29899	-391.65602	-477.80926	1
H	78.28060	72.96439	77.96570	2528.72714	-681.76262	-751.27122	1
C	77.65119	76.13030	78.53277	252.47363	-605.14473	440.05134	1
H	77.29368	76.20304	79.58715	-2052.65063	1456.49051	-1043.51830	1
H	78.24290	77.05094	78.31944	-2152.98800	-1661.97574	-161.70652	1
H	76.75143	76.15244	77.87426	-473.12726	-1584.27339	2165.61725	1
C	79.71615	76.29016	76.31743	360.36307	488.46713	505.10056	1
O	80.95032	76.32723	76.27838	-200.08817	-528.03552	-385.82850	1
N	78.87138	77.25557	76.00215	-597.54095	-226.44775	355.20479	1
H	77.86827	77.09888	76.09321	2179.23655	-1300.70981	-991.33835	1
C	79.42514	78.46853	75.50029	-687.68921	297.39807	-249.45179	1
H	80.34513	78.11258	74.98636	-349.16256	-2467.37713	-1101.72831	1

201	C	78.37778	79.21567	74.55521	672.47345	361.54328	-201.62880	1
202	H	77.35846	79.22958	75.00425	1541.41502	582.18425	2169.97026	1
203	H	78.68784	80.28148	74.44166	-2534.67717	60.25153	997.72295	1
204	C	78.36839	78.51906	73.18356	574.26774	-389.43119	-377.02542	1
205	C	79.59997	78.24431	72.51598	688.82679	-287.33580	-257.96133	1
206	H	80.56686	78.51110	72.97921	331.14182	1470.38163	2269.80013	1
207	C	77.12501	78.18501	72.56899	-383.52382	-675.04808	144.24917	1
208	H	76.16842	78.40048	73.07843	4.09847	1517.59662	2262.86397	1
209	C	79.56259	77.63176	71.26320	-134.76730	428.45285	-649.50303	1
210	H	80.51638	77.40680	70.75371	-903.23433	1600.49020	2011.53387	1
211	C	77.13716	77.59064	71.30379	-335.53281	-615.00040	-364.39058	1
212	H	76.16922	77.34327	70.83396	1596.45398	-1369.33862	1732.02659	1
213	C	78.34402	77.28972	70.60865	585.48547	-340.24194	-406.23938	1
214	O	78.36462	76.64598	69.34086	-419.15649	-173.04408	-511.94141	1
215	H	77.39748	76.63269	69.14859	-1071.84350	1559.71507	-1960.13012	1
216	C	79.81233	79.48065	76.71873	291.41878	-183.13091	710.72246	1
217	O	79.90599	80.67318	76.53348	379.87695	523.47396	-222.23622	1
218	N	80.13596	78.79647	77.81827	564.30591	373.09380	277.24371	1
219	H	80.00342	77.78312	77.76423	-120.59931	-2436.07824	-1214.35098	1
220	C	80.16255	79.51506	79.05435	404.07514	-299.37480	608.84009	1
221	H	80.47384	80.54027	78.74464	-1071.07882	-841.69025	2359.66467	1
222	C	78.68148	79.52335	79.62986	-683.12807	68.71770	-390.13096	1
223	H	77.95334	79.74117	78.81645	2068.88918	1596.58590	770.89603	1
224	H	78.40443	78.51987	80.02375	1379.86429	2342.49779	-179.85638	1
225	C	78.59573	80.59812	80.75525	-176.39967	537.49466	550.97268	1
226	H	79.26422	80.32805	81.60651	-396.28051	1942.22168	1869.33314	1
227	H	78.95480	81.58684	80.39204	-94.44700	586.00678	-2659.20016	1
228	C	77.06823	80.68803	81.22155	-291.69381	-718.18079	-150.72929	1
229	H	76.32890	80.99542	80.43952	-2303.38776	1089.76242	964.62032	1
230	H	76.69172	79.76124	81.72569	884.16729	2568.80533	-207.74283	1
231	N	77.13540	81.69925	82.19043	-594.49522	-252.81478	342.29276	1
232	H	78.03804	81.85578	82.61254	-1735.17426	2084.05705	-263.71605	1
233	C	76.15335	82.39837	82.59476	595.23177	-271.52487	442.22352	1
234	N	74.89468	82.16663	82.03078	585.53813	-424.79750	-105.81303	1
235	H	74.61240	81.48242	81.33040	959.88797	1327.56823	-2177.11905	1
236	H	74.03945	82.65840	82.29250	1578.48647	16.27378	-2220.76233	1
237	N	76.55695	83.30719	83.58317	-452.17763	-390.33541	-421.51853	1
238	H	77.46938	83.47392	84.00471	470.26107	1974.25629	-1817.92087	1
239	H	75.91452	83.95332	84.04324	1856.67307	-1573.97321	-1224.35416	1
240	C	81.16318	78.79260	80.11224	-531.47271	-581.45780	-55.06206	1
241	O	81.21823	79.22480	81.24362	-243.00837	192.73507	609.52485	1
242	N	81.88652	77.81774	79.56971	343.49288	-644.25607	38.09164	1
243	H	81.68519	77.63461	78.58698	-1691.16043	-1535.97525	-1484.73099	1
244	C	82.75010	76.92036	80.33799	542.66492	350.14337	454.42563	1
245	H	83.34834	76.45441	79.52053	1209.57979	-2396.95920	463.86744	1
246	C	83.60110	77.75454	81.27789	-737.14309	145.93010	242.71942	1
247	H	82.91415	78.34223	81.94386	-1050.18303	159.36419	-2509.06125	1
248	C	84.51569	76.91006	82.14416	-706.77696	-252.88343	-245.16230	1
249	H	85.27849	77.54241	82.67666	-1624.64039	-1665.49085	1417.86935	1
250	H	83.94596	76.34326	82.93360	2566.10521	598.51109	693.22305	1
251	H	85.07929	76.15183	81.53453	-1972.69758	107.10018	-1876.34312	1
252	C	84.43207	78.75857	80.49609	412.85148	672.58832	-27.69222	1
253	H	85.00185	79.44952	81.17521	1447.02277	1762.76533	-1490.78944	1
254	H	85.18126	78.24823	79.83143	1320.77379	-145.20455	2378.68491	1
255	H	83.78292	79.39668	79.83554	-1512.10848	1470.52031	1724.75147	1
256	C	81.74187	75.77053	80.98962	-607.11113	499.56689	73.73769	1
257	O	81.15488	75.03680	80.23012	357.97126	460.81534	356.68785	1
258	N	81.76091	75.73804	82.31219	536.55407	-368.62270	332.76306	1
259	H	82.25218	76.47278	82.81976	524.38866	2297.69985	1367.20941	1
260	C	80.90196	74.82180	82.96544	46.50277	-774.29190	-147.97904	1
261	H	80.60109	74.08692	82.18370	1676.51875	1883.29066	1032.55549	1
262	C	81.66427	74.19264	84.24400	-559.41456	-34.19198	-556.30433	1

```
H    82.06177  75.02247  84.87558       -64.66664       395.18769      2695.05373           1
H    80.94529  73.63301  84.88954     -2703.44278       -60.40056       333.79418           1
C    82.78111  73.29088  83.79155      -519.76435       377.31045       459.42366           1
H    83.29695  73.77201  82.93014     -1746.74323      1667.50644     -1261.73691           1
C    83.79512  73.09276  84.91171       570.95554        16.46261      -545.27700           1
H    84.64274  72.43314  84.59215      2391.58273        28.05277     -1305.07271           1
H    84.23878  74.06566  85.24671       175.75588       917.63458      2559.43867           1
H    83.32669  72.61571  85.81102     -1063.97851     -2155.34650      1283.00462           1
C    82.24295  71.93482  83.35375       193.76014         2.62946      -765.53141           1
H    83.06849  71.24309  83.04740       507.39394      -846.55087      2539.60089           1
H    81.68151  71.42762  84.18042      1337.57841      1818.19054      1526.01994           1
H    81.54323  72.02704  82.48369      1100.88635     -1172.91350     -2199.08909           1
C    79.65988  75.66161  83.57499      -549.21115       475.64486      -309.38218           1
O    79.76222  76.78323  83.99651       556.06106      -390.37755       -78.24250           1
O    78.46214  74.93153  83.75588      -572.27940      -310.19032      -209.75862           1
H    78.73042  74.08027  83.31106     -1380.08193      2212.14486       790.85944           1
```

Listing 63: C00063.xmolout

```
       276
  150.0000  150.0000  150.0000     0.0000     0.0000     0.0000***********    0.8935     3574
       0.2500    300.00   0.00    0.00   0.00  -1293.6126      0.0000    300.0000
H    72.13158  63.34573  73.50435     -2043.14101     -1757.64893       399.89339           1
N    72.14281  64.34379  73.70612       426.75047       490.45733      -334.42467           1
H    72.25416  64.34471  74.71876       442.25559     -1110.30854     -2448.52907           1
C    73.11976  65.07655  72.99760       415.32972      -666.39213        83.73172           1
H    73.24409  64.53631  72.03596       879.32219      2491.00447       667.35213           1
C    72.58535  66.58440  72.81604        31.35357       477.41777       628.23362           1
H    72.35426  67.02494  73.81493      1168.38489      2240.25337     -1019.71136           1
H    73.37868  67.23157  72.36946      -360.43770      2534.90498       931.67112           1
C    71.34835  66.56779  71.95218       463.18208       512.70242      -382.34388           1
H    70.72253  65.69909  72.25808      1725.29117      1863.52247      -987.07854           1
C    70.53730  67.84002  72.16102       710.42801      -144.27427      -313.15367           1
H    69.60990  67.84761  71.53193       364.20195     -1033.13725      2494.72524           1
H    70.21540  67.95484  73.22842      2567.61904       335.80679       847.48802           1
H    71.12779  68.75374  71.89215     -2587.99029       680.92867      -512.16411           1
C    71.70954  66.42397  70.48049       268.17616       741.64379       -40.42634           1
H    70.79675  66.40582  69.83042      1283.54314     -1945.37420     -1411.27812           1
H    72.34890  67.27504  70.12935     -1258.57796      1317.15249     -2026.02085           1
H    72.27841  65.47927  70.28197     -2502.84526       909.80597       575.92391           1
C    74.48515  65.09018  73.90148       619.36578      -458.64843      -172.09273           1
O    75.51991  64.60834  73.49906       -81.40433       433.87611       522.34594           1
N    74.26223  65.73131  75.04873       175.23996      -622.20190       341.55523           1
H    73.30878  66.02186  75.25958      -876.57256      -303.22879     -2561.90213           1
C    75.25520  65.64846  76.06294      -571.88214      -534.10783      -106.15033           1
H    76.19985  65.37761  75.53836     -2713.20841       -16.25800      -248.80809           1
C    75.26842  67.08448  76.77726       166.60354       288.16399      -716.09568           1
H    74.23651  67.29814  77.16863     -2097.85574      1636.78449      -586.18044           1
O    75.60980  68.18634  75.91871      -317.80580      -363.38227       484.43082           1
H    74.80752  68.74039  75.99645      1863.74748     -1451.84923      1357.29467           1
C    76.23198  66.86950  78.00213       503.80731       517.99032       318.51606           1
H    76.32907  67.81346  78.59850       640.61020     -2157.10410     -1536.29104           1
H    75.86307  66.07013  78.69387      1687.88455       465.60692      2087.56452           1
H    77.26160  66.58414  77.68235     -1293.07895     -1899.88581     -1463.57452           1
C    74.71164  64.51991  77.11241      -153.07550      -733.68902       248.70981           1
O    73.56468  64.55215  77.47052       186.15459      -480.78787      -449.34387           1
N    75.69678  63.69412  77.49245      -487.53139       543.18517        42.03850           1
C    75.49306  62.77183  78.56850      -699.35871        69.34393       360.10701           1
H    74.42808  62.44908  78.52201         2.81126      -221.99807     -2715.58072           1
C    76.57757  61.65722  78.24548      -507.46439      -592.59194       122.07761           1
H    76.85264  61.05075  79.13737     -1975.08116       525.87560     -1801.71556           1
H    76.21137  60.94280  77.47485      1588.27743     -1913.22100      1113.83566           1
```

C	77.75569	62.52248	77.70080	-613.62442	-457.55993	-194.14490	1
H	78.29366	63.02638	78.53746	1707.54637	-721.27992	1996.92523	1
H	78.51612	61.94043	77.13258	758.23357	2100.81767	1560.55011	1
C	76.97635	63.57993	76.80690	-352.41524	704.96076	49.21465	1
H	77.44933	64.58832	76.75997	1714.76886	-2070.42778	443.35792	1
H	76.73672	63.23615	75.77201	1224.15040	1801.50881	1636.97633	1
C	75.86692	63.55365	79.95986	566.85117	537.05831	117.62814	1
O	76.98806	64.03265	80.11892	-550.26779	-406.08867	4.21666	1
N	74.82378	63.54005	80.75355	566.34832	-425.77725	180.18749	1
H	73.99708	63.10552	80.34327	644.44502	2646.80550	-52.73430	1
C	74.84560	63.75483	82.15452	612.34801	-281.48662	411.56221	1
H	73.86193	63.36029	82.49274	1667.23498	-1490.75142	1556.16753	1
C	76.08722	62.94391	82.75718	-714.45746	-175.43074	286.98927	1
H	77.03151	63.34868	82.29444	-787.09484	1763.03619	-1922.46060	1
O	76.09940	61.52707	82.50817	-429.46514	516.51932	-128.40526	1
H	76.91264	61.43909	81.97067	1981.82785	436.16847	-1818.18182	1
C	76.00880	63.37767	84.26674	630.79189	-468.94448	76.03230	1
H	76.90060	63.03279	84.83983	-1775.71861	2062.30353	131.89849	1
H	75.94484	64.48832	84.37596	-1855.14608	88.94118	1993.53760	1
H	75.10953	62.94642	84.76496	-1504.82350	-780.94249	-2132.90992	1
C	75.01954	65.34237	82.53113	288.57638	618.62814	-397.00270	1
O	74.34957	65.81703	83.41840	321.70495	-185.26452	574.37218	1
N	76.03413	65.87701	81.87463	-447.05792	-446.53431	367.76425	1
H	76.54083	65.30651	81.15226	350.31316	1975.63644	-1843.31522	1
C	76.16661	67.29800	81.90445	-511.23061	374.03917	-471.51480	1
H	76.02336	67.58729	82.96977	2234.79579	-898.70890	-1273.45190	1
C	77.63735	67.55108	81.33887	529.53781	-159.34528	563.72604	1
H	77.83657	68.65606	81.36389	654.59388	2347.94251	1217.51488	1
H	78.34417	67.07897	82.07198	-1526.55661	2103.42026	817.87394	1
O	77.93432	67.07579	79.99467	-554.84278	14.12789	399.58769	1
H	77.87876	66.11250	80.14644	1297.06236	2061.84854	1220.68812	1
C	74.99039	67.94846	80.98003	-662.15484	-412.13544	123.62687	1
O	74.56998	67.47442	79.94849	-540.01537	-317.05804	274.91456	1
N	74.66198	69.11142	81.53977	-202.76299	-690.00100	-131.49145	1
H	75.09207	69.39462	82.41929	-702.62132	-2617.37191	281.70413	1
C	73.44408	69.71394	81.11604	-491.73914	576.34187	222.73509	1
H	73.30222	69.39770	80.05619	1977.17978	-1823.58850	-434.68928	1
C	72.29584	69.17445	82.08045	-649.50797	-328.66843	306.11224	1
H	72.51816	69.45486	83.13257	2075.07803	1452.16490	-1004.45904	1
C	70.93163	69.78600	81.61466	-615.18735	-489.99689	-70.96352	1
H	70.08663	69.44087	82.25560	-1226.32661	-1724.38906	-1716.47170	1
H	70.94826	70.90081	81.65994	2381.64273	-969.84735	900.46860	1
H	70.69067	69.49938	80.56408	2122.74991	1702.23946	-141.36228	1
C	72.16300	67.61951	81.95322	-501.86074	-479.61276	-376.42482	1
H	71.30048	67.22952	82.54376	993.79261	-2246.95401	-1177.81305	1
H	72.01238	67.30745	80.89228	42.71279	-626.15391	-2651.37258	1
H	73.08395	67.09525	82.32627	-2026.39012	-1590.33709	-887.82922	1
C	73.57726	71.32524	81.39003	-672.89153	382.57394	-156.34072	1
O	74.12048	71.75509	82.38087	410.04627	221.86223	500.35950	1
N	72.81951	71.96625	80.49754	-378.11782	-443.79414	-441.10984	1
H	72.41827	71.34780	79.79158	2404.15973	-568.14894	1149.30085	1
C	73.14598	73.30135	80.06397	-544.54484	466.16019	331.29119	1
H	72.16063	73.75077	79.81901	-1409.80988	-170.17955	2325.32679	1
C	73.92904	74.05616	81.20543	-184.48284	116.78282	-758.89137	1
H	73.40823	73.91771	82.18285	1893.78641	1861.14652	611.04528	1
H	74.94034	73.60153	81.33696	-2170.79665	-974.19311	1327.50117	1
C	74.05829	75.57092	80.84088	389.51668	672.81878	-138.49152	1
H	74.59380	75.69821	79.86880	510.64577	1933.03327	1851.02484	1
H	73.05550	76.01374	80.65394	-2302.30869	-1105.72939	-948.89774	1
C	74.79079	76.38804	81.97162	-559.93427	-126.34590	542.30857	1
O	74.93617	77.57782	81.85250	-670.36003	-111.83166	76.36448	1
N	75.19305	75.63877	83.02168	-216.06729	-537.07037	446.51379	1

H	75.11108	74.62802	83.18292	-1251.96487	1074.12894	-2168.52500	1
H	75.68826	76.01588	83.83682	-2451.83023	-720.73851	944.84612	1
C	74.12525	73.17291	78.77118	546.24052	-563.88337	-85.11809	1
O	75.07701	72.43649	78.79232	71.70702	-307.40463	606.69673	1
N	73.73147	74.01108	77.79407	479.90863	-504.50129	222.86849	1
C	74.58933	74.28371	76.68838	75.51268	-600.91904	506.73700	1
H	75.25521	73.39875	76.55816	-2033.61562	1795.30103	254.89480	1
C	73.52354	74.54339	75.53524	523.79067	517.99274	284.45581	1
H	73.94634	75.10406	74.67214	1599.61665	-1127.00911	1895.98173	1
H	73.12474	73.58705	75.13033	-1881.83093	1275.82488	-1501.55016	1
C	72.42069	75.33607	76.30188	-600.05639	-404.05026	316.64485	1
H	72.70299	76.40756	76.40894	-2597.05260	496.36221	657.73249	1
H	71.43110	75.32099	75.79216	-1666.24518	1294.38985	-1723.90591	1
C	72.40481	74.60676	77.71308	493.57570	-129.95376	-602.56394	1
H	72.28453	75.29213	78.58458	-1469.08415	-637.84797	2204.22581	1
H	71.67857	73.76449	77.80064	-1038.97840	1068.88701	-2280.71789	1
C	75.40483	75.67551	76.97820	188.88100	241.05089	-727.87821	1
O	75.03113	76.50171	77.77427	531.88613	-49.19943	427.07960	1
N	76.53638	75.72355	76.21100	708.14833	26.93417	-179.73509	1
H	76.44479	74.86647	75.65197	-2232.69501	-1179.09831	1023.94816	1
C	76.92784	76.97343	75.51326	-738.32412	259.92548	104.42456	1
H	76.16147	77.11811	74.71900	2028.89123	1260.02983	1311.33324	1
C	76.96983	78.09384	76.51481	-258.65678	-345.52513	661.28459	1
H	77.00223	77.62899	77.52965	-1826.06090	-1801.46097	-918.64554	1
C	78.19436	78.97890	76.35359	-541.23400	531.92970	-218.41428	1
H	78.20388	79.81889	77.10157	1311.73218	-2219.18200	882.19023	1
H	79.14816	78.39563	76.49497	-1048.71298	1223.58118	-2196.97971	1
H	78.24153	79.44148	75.32970	419.06414	1903.92898	1903.44670	1
C	75.72824	78.96826	76.44012	-423.86431	152.66566	-648.55262	1
H	75.68415	79.69585	77.29679	105.68386	-2342.50080	1387.51238	1
H	75.69214	79.56739	75.48973	-1695.63603	-1484.73621	-1531.02793	1
H	74.78497	78.35407	76.47849	-1853.57121	-487.39031	1936.59343	1
C	78.41051	76.58195	74.90397	539.71428	-510.58232	267.58633	1
O	79.32284	76.29094	75.64312	-15.93758	-278.34085	-624.49402	1
N	78.43479	76.67923	73.58019	-676.81886	12.05292	276.17960	1
H	77.58228	76.94901	73.09245	-1694.44250	-373.84189	-2100.66098	1
C	79.60661	76.19084	72.92598	412.33078	-664.70360	108.35451	1
H	80.07843	75.50671	73.67008	1765.77476	1294.44366	1621.76594	1
C	79.17382	75.48867	71.57491	-179.66635	-721.95667	-264.73915	1
H	78.36314	74.74651	71.74895	-607.34922	2587.87032	-598.10018	1
H	78.75231	76.22866	70.86008	-359.69332	-1479.13954	-2259.74260	1
C	80.45317	74.80391	70.98903	-240.01076	616.02425	431.85322	1
H	81.27948	75.54812	70.89239	-1359.17131	782.54425	2227.99181	1
H	80.83141	74.02265	71.68587	-820.36667	85.81767	-2596.78715	1
C	80.12013	74.17606	69.57671	-513.07015	236.18570	-551.87280	1
H	79.34107	73.38402	69.66152	1816.11944	-1183.11722	-1650.94321	1
H	79.70209	74.94305	68.88475	-1800.04429	-1361.53473	1526.34643	1
C	81.44777	73.60025	69.02984	726.08972	-253.19732	-179.64713	1
H	82.23387	74.35829	68.84036	1224.27271	-456.51798	-2390.90304	1
H	81.86685	72.76432	69.62472	1206.40339	-1150.93514	-2154.90344	1
N	81.16165	72.97584	67.62329	329.70796	-633.68968	-155.67358	1
H	82.00175	72.55147	67.13364	1348.31305	1938.76845	1359.00643	1
H	80.46996	72.17179	67.58255	2096.92035	1446.54551	-966.48890	1
H	80.78878	73.62576	66.87142	-1197.07217	-2303.53719	827.28733	1
C	80.60758	77.43346	72.54714	-378.01186	-556.17301	-413.96481	1
O	81.75759	77.21218	72.23195	-409.44433	-307.55053	-453.30833	1
N	79.98168	78.60449	72.54578	-429.02477	524.70884	274.08502	1
H	79.00948	78.60252	72.84762	1706.34263	-1425.96036	1574.38926	1
C	80.80232	79.76243	72.32194	-45.25864	-626.22952	478.93267	1
H	81.77923	79.49426	72.78619	1988.63164	570.90773	1772.87287	1
C	80.93988	79.91591	70.75051	551.77128	-20.49526	-564.54996	1
H	81.23911	78.94705	70.29109	1434.61535	-1753.10253	1513.99471	1

167	H	79.95679	80.17397	70.29811	-714.67738	1819.09770	1898.36494	1
168	C	81.99178	81.03102	70.44410	446.99263	-555.77305	338.97221	1
169	H	81.68961	81.99602	70.91076	1953.68466	1892.25957	-161.67766	1
170	H	82.98564	80.77630	70.87590	-403.03785	2688.07032	-188.43591	1
171	C	82.06974	81.15731	68.86868	567.86262	335.74681	434.04421	1
172	H	82.39745	80.19502	68.41204	-1080.02275	1568.26081	-1948.78900	1
173	H	81.05946	81.36074	68.43935	-919.54108	2111.34692	1456.13463	1
174	C	83.05259	82.29465	68.50902	195.81897	173.33862	-745.11536	1
175	H	82.73589	83.30255	68.84379	1405.67949	-2301.21089	-390.08113	1
176	H	84.10482	82.11007	68.80405	-940.37328	-1118.32446	2299.72133	1
177	N	83.08437	82.38691	66.94527	319.26422	656.36007	-42.02424	1
178	H	83.70282	83.14565	66.53660	1961.03633	619.91968	1787.09422	1
179	H	83.42697	81.53123	66.41914	854.14343	-2156.80752	1429.08711	1
180	H	82.16158	82.58296	66.45888	1237.02905	-1378.50857	-1998.28500	1
181	C	80.07955	81.08107	72.94184	532.53319	-268.57236	517.55729	1
182	O	80.78364	81.99246	73.38487	266.16714	-290.73804	-558.88002	1
183	N	78.79074	81.04970	72.74031	-226.25352	640.49678	-270.32839	1
184	H	78.44310	80.20596	72.28442	2007.69063	-1035.27229	1523.50231	1
185	C	77.84218	81.82996	73.48760	-540.76542	-573.33303	49.50000	1
186	H	77.58694	81.20102	74.36754	-2374.20950	-1036.76023	843.75774	1
187	C	78.48820	83.21680	73.91267	-77.09082	666.87422	-415.84136	1
188	H	79.13934	83.54343	73.05088	-985.81834	617.19825	2463.91927	1
189	O	79.35119	83.16446	75.05090	293.29899	-391.65602	-477.80926	1
190	H	80.23918	82.95190	74.57200	2528.72714	-681.76262	-751.27122	1
191	C	77.25205	84.17627	74.07001	252.47363	-605.14473	440.05134	1
192	H	77.57307	85.20803	74.34371	-2052.65063	1456.49051	-1043.51830	1
193	H	76.65703	84.25556	73.12683	-2152.98800	-1661.97574	-161.70652	1
194	H	76.56286	83.82244	74.87161	-473.12726	-1584.27339	2165.61725	1
195	C	76.58996	81.90947	72.44780	360.36307	488.46713	505.10056	1
196	O	76.48996	82.76159	71.57940	-200.08817	-528.03552	-385.82850	1
197	N	75.76272	80.91619	72.73157	-597.54095	-226.44775	355.20479	1
198	H	75.99018	80.24899	73.46882	2179.23655	-1300.70981	-991.33835	1
199	C	74.61611	80.73869	71.92774	-687.68921	297.39807	-249.45179	1
200	H	74.47736	81.67935	71.34560	-349.16256	-2467.37713	-1101.72831	1
201	C	74.84848	79.43337	71.02157	672.47345	361.54328	-201.62880	1
202	H	74.89438	78.52931	71.66929	1541.41502	582.18425	2169.97026	1
203	H	73.96702	79.29808	70.35393	-2534.67717	60.25153	997.72295	1
204	C	76.13361	79.53936	70.19026	574.26774	-389.43119	-377.02542	1
205	C	76.29552	80.59652	69.24425	688.82679	-287.33580	-257.96133	1
206	H	75.52018	81.37572	69.13344	331.14182	1470.38163	2269.80013	1
207	C	77.15482	78.55281	70.34792	-383.52382	-675.04808	144.24917	1
208	H	77.04368	77.74131	71.08873	4.09847	1517.59662	2262.86397	1
209	C	77.45292	80.62850	68.46611	-134.76730	428.45285	-649.50303	1
210	H	77.57620	81.44795	67.73669	-903.23433	1600.49020	2011.53387	1
211	C	78.29018	78.62116	69.53733	-335.53281	-615.00040	-364.39058	1
212	H	79.07044	77.85016	69.66141	1596.45398	-1369.33862	1732.02659	1
213	C	78.48069	79.64639	68.56530	585.48547	-340.24194	-406.23938	1
214	O	79.63507	79.71798	67.73943	-419.15649	-173.04408	-511.94141	1
215	H	80.08738	78.88633	68.01873	-1071.84350	1559.71507	-1960.13012	1
216	C	73.40062	80.28209	72.92738	291.41878	-183.13091	710.72246	1
217	O	73.56772	79.44910	73.78863	379.87695	523.47396	-222.23622	1
218	N	72.26369	80.85023	72.52800	564.30591	373.09380	277.24371	1
219	H	72.29119	81.57335	71.80779	-120.59931	-2436.07824	-1214.35098	1
220	C	71.11344	80.64275	73.34280	404.07514	-299.37480	608.84009	1
221	H	71.37949	79.76654	73.98166	-1071.07882	-841.69025	2359.66467	1
222	C	70.90356	81.97311	74.19055	-683.12807	68.71770	-390.13096	1
223	H	71.87592	82.33919	74.59008	2068.88918	1596.58590	770.89603	1
224	H	70.51444	82.79128	73.53553	1379.86429	2342.49779	-179.85638	1
225	C	69.90854	81.65490	75.34379	-176.39967	537.49466	550.97268	1
226	H	68.91773	81.35275	74.93073	-396.28051	1942.22168	1869.33314	1
227	H	70.25805	80.78685	75.94547	-94.44700	586.00678	-2659.20016	1
228	C	69.77974	82.96462	76.26197	-291.69381	-718.18079	-150.72929	1

```
H   70.71390  83.30382  76.77513    -2303.38776     1089.76242      964.62032          1
H   69.29598  83.82136  75.72581      884.16729     2568.80533     -207.74283          1
N   68.87574  82.45655  77.20169     -594.49522     -252.81478      342.29276          1
H   68.10750  81.91836  76.82618    -1735.17426     2084.05705     -263.71605          1
C   68.91134  82.63851  78.46393      595.23177     -271.52487      442.22352          1
N   69.94196  83.40520  79.01132      585.53813     -424.79750     -105.81303          1
H   70.70825  83.89281  78.54997      959.88797     1327.56823    -2177.11905          1
H   70.04368  83.62069  80.00382     1578.48647       16.27378    -2220.76233          1
N   67.83003  81.99222  79.07699     -452.17763     -390.33541     -421.51853          1
H   67.07426  81.43568  78.68107      470.26107     1974.25629    -1817.92087          1
H   67.66079  82.01621  80.08295     1856.67307    -1573.97321    -1224.35416          1
C   69.78715  80.43518  72.41978     -531.47271     -581.45780      -55.06206          1
O   68.68411  80.39820  72.91088     -243.00837      192.73507      609.52485          1
N   70.08581  80.25074  71.08967      343.49288     -644.25607       38.09164          1
H   71.11298  80.21634  71.11907    -1691.16043    -1535.97525    -1484.73099          1
C   69.49242  81.18599  70.10119      542.66492      350.14337      454.42563          1
H   69.79705  80.75227  69.12226     1209.57979    -2396.95920      463.86744          1
C   68.00038  81.26233  70.28758     -737.14309      145.93010      242.71942          1
H   67.80403  81.73444  71.27940    -1050.18303      159.36419    -2509.06125          1
C   67.35273  82.11779  69.21121     -706.77696     -252.88343     -245.16230          1
H   66.24638  82.22789  69.37563    -1624.64039    -1665.49085     1417.86935          1
H   67.79295  83.15254  69.18108     2566.10521      598.51109      693.22305          1
H   67.49391  81.67410  68.18815    -1972.69758      107.10018    -1876.34312          1
C   67.36056  79.88409  70.30754      412.85148      672.58832      -27.69222          1
H   66.24969  79.93828  70.46846     1447.02277     1762.76533    -1490.78944          1
H   67.53496  79.32948  69.34562     1320.77379     -145.20455     2378.68491          1
H   67.79234  79.25944  71.14022    -1512.10848     1470.52031     1724.75147          1
C   70.17872  82.66196  70.37064     -607.11113      499.56689       73.73769          1
O   70.13512  83.23291  71.43759      357.97126      460.81534      356.68785          1
N   70.76831  83.06272  69.24887      536.55407     -368.62270      332.76306          1
H   70.66937  82.47659  68.42071      524.38866     2297.69985     1367.20941          1
C   70.99339  84.45969  69.10101       46.50277     -774.29190     -147.97904          1
H   70.68260  84.91408  70.07107     1676.51875     1883.29066     1032.55549          1
C   72.55443  84.70153  68.76688     -559.41456      -34.19198     -556.30433          1
H   72.77447  84.35763  67.72842      -64.66664      395.18769     2695.05373          1
H   72.79118  85.79185  68.78106    -2703.44278      -60.40056      333.79418          1
C   73.40426  83.94923  69.75654     -519.76435      377.31045      459.42366          1
H   73.14145  82.86869  69.69200    -1746.74323     1667.50644    -1261.73691          1
C   74.87822  84.11307  69.42192      570.95554       16.46261     -545.27700          1
H   75.54224  83.60483  70.21316     2391.58273       28.05277    -1305.07271          1
H   75.13210  83.66701  68.42579      175.75588      917.63458     2559.43867          1
H   75.17489  85.19290  69.38473    -1063.97851    -2155.34650     1283.00462          1
C   73.14553  84.41145  71.18413      193.76014        2.62946     -765.53141          1
H   73.83375  83.90908  71.91241      507.39394     -846.55087     2539.60089          1
H   73.29637  85.51622  71.29443     1337.57841     1818.19054     1526.01994          1
H   72.09050  84.17754  71.50378     1100.88635    -1172.91350    -2199.08909          1
C   70.11097  84.92201  67.83503     -549.21115      475.64486     -309.38218          1
O   69.44846  84.16226  67.17751      556.06106     -390.37755      -78.24250          1
O   70.16243  86.29892  67.50972     -572.27940     -310.19032     -209.75862          1
H   70.76145  86.60782  68.24360    -1380.08193     2212.14486      790.85944          1
```

Listing 64: C00064.xmolout

```
      276
  150.0000  150.0000  150.0000     0.0000     0.0000     0.0000***********    2.7750    11100
        0.2500     300.00    0.00    0.00    0.00   -1293.6118       0.0000     300.0000
H   70.86456  70.38264  90.46485    -2043.14101    -1757.64893      399.89339          1
N   71.80658  69.99925  90.47458      426.75047      490.45733     -334.42467          1
H   71.71069  69.16223  89.90548      442.25559    -1110.30854    -2448.52907          1
C   72.83715  70.88415  90.09649      415.32972     -666.39213       83.73172          1
H   72.57003  71.84158  90.59306      879.32219     2491.00447      667.35213          1
C   74.24872  70.29569  90.60647       31.35357      477.41777      628.23362          1
```

H	74.46175	69.33946	90.07081	1168.38489	2240.25337	-1019.71136	1
H	75.07730	70.99155	90.32944	-360.43770	2534.90498	931.67112	1
C	74.20722	70.06533	92.09506	463.18208	512.70242	-382.34388	1
H	73.30501	69.45528	92.32770	1725.29117	1863.52247	-987.07854	1
C	75.44389	69.29722	92.54439	710.42801	-144.27427	-313.15367	1
H	75.42718	69.09280	93.64602	364.20195	-1033.13725	2494.72524	1
H	75.52980	68.30863	92.02356	2567.61904	335.80679	847.48802	1
H	76.38344	69.86829	92.32759	-2587.99029	680.92867	-512.16411	1
C	74.11602	71.37792	92.86090	268.17616	741.64379	-40.42634	1
H	74.13110	71.21158	93.96911	1283.54314	-1945.37420	-1411.27812	1
H	74.97178	72.05872	92.61556	-1258.57796	1317.15249	-2026.02085	1
H	73.17161	71.93183	92.62279	-2502.84526	909.80597	575.92391	1
C	72.93921	71.03142	88.49148	619.36578	-458.64843	-172.09273	1
O	73.89841	71.60576	88.00006	-81.40433	433.87611	522.34594	1
N	71.91748	70.48047	87.84853	175.23996	-622.20190	341.55523	1
H	71.13713	70.09702	88.37724	-876.57256	-303.22879	-2561.90213	1
C	71.87538	70.62607	86.43096	-571.88214	-534.10783	-106.15033	1
H	72.94571	70.69310	86.13271	-2713.20841	-16.25800	-248.80809	1
C	71.06905	69.37766	85.83846	166.60354	288.16399	-716.09568	1
H	70.03775	69.37040	86.28520	-2097.85574	1636.78449	-586.18044	1
O	71.63676	68.08280	86.09796	-317.80580	-363.38227	484.43082	1
H	70.92395	67.66267	86.62009	1863.74748	-1451.84923	1357.29467	1
C	70.98097	69.79086	84.32236	503.80731	517.99032	318.51606	1
H	70.41117	69.04037	83.72779	640.61020	-2157.10410	-1536.29104	1
H	70.47909	70.77936	84.17740	1687.88455	465.60692	2087.56452	1
H	71.99790	69.87582	83.87123	-1293.07895	-1899.88581	-1463.57452	1
C	71.01673	71.99345	86.14453	-153.07550	-733.68902	248.70981	1
O	69.84411	71.99966	86.40537	186.15459	-480.78787	-449.34387	1
N	71.77443	72.95137	85.58836	-487.53139	543.18517	42.03850	1
C	71.26571	74.27826	85.43054	-699.35871	69.34393	360.10701	1
H	70.56548	74.45232	86.27904	2.81126	-221.99807	-2715.58072	1
C	72.59200	75.14778	85.44988	-507.46439	-592.59194	122.07761	1
H	72.47090	76.12642	84.93320	-1975.08116	525.87560	-1801.71556	1
H	72.90738	75.37711	86.49217	1588.27743	-1913.22100	1113.83566	1
C	73.62525	74.20842	84.75019	-613.62442	-457.55993	-194.14490	1
H	73.52017	74.25409	83.64295	1707.54637	-721.27992	1996.92523	1
H	74.68503	74.46487	84.97584	758.23357	2100.81767	1560.55011	1
C	73.19632	72.78923	85.31474	-352.41524	704.96076	49.21465	1
H	73.31806	71.94167	84.59864	1714.76886	-2070.42778	443.35792	1
H	73.65640	72.50352	86.31431	1224.15040	1801.50881	1636.97633	1
C	70.49232	74.41864	83.98911	566.85117	537.05831	117.62814	1
O	69.61955	75.24188	83.87131	-550.26779	-406.08867	4.21666	1
N	71.04299	73.64020	83.06356	566.34832	-425.77725	180.18749	1
H	71.76211	72.97441	83.35590	644.44502	2646.80550	-52.73430	1
C	70.35344	73.38809	81.84843	612.34801	-281.48662	411.56221	1
H	69.33779	73.83699	81.93959	1667.23498	-1490.75142	1556.16753	1
C	71.23003	74.02952	80.66832	-714.45746	-175.43074	286.98927	1
H	72.23688	73.52868	80.65504	-787.09484	1763.03619	-1922.46060	1
O	71.49262	75.43930	80.76356	-429.46514	516.51932	-128.40526	1
H	72.46938	75.44900	80.82268	1981.82785	436.16847	-1818.18182	1
C	70.40297	73.58661	79.40730	630.79189	-468.94448	76.03230	1
H	70.87680	73.96100	78.47142	-1775.71861	2062.30353	131.89849	1
H	70.32519	72.47637	79.32115	-1855.14608	88.94118	1993.53760	1
H	69.36585	73.99492	79.43500	-1504.82350	-780.94249	-2132.90992	1
C	70.44136	71.75692	81.71863	288.57638	618.62814	-397.00270	1
O	71.42235	71.17467	82.13326	321.70495	-185.26452	574.37218	1
N	69.38622	71.27097	81.08526	-447.05792	-446.53431	367.76425	1
H	68.65698	71.89210	80.73746	350.31316	1975.63644	-1843.31522	1
C	69.33793	69.86448	80.84077	-511.23061	374.03917	-471.51480	1
H	69.65877	69.39671	81.79656	2234.79579	-898.70890	-1273.45190	1
C	67.84026	69.55292	80.39652	529.53781	-159.34528	563.72604	1
H	67.77978	68.44342	80.24799	654.59388	2347.94251	1217.51488	1

71	H	67.13277	69.78347	81.23660	-1526.55661	2103.42026	817.87394	1
72	O	67.35719	70.25934	79.21203	-554.84278	14.12789	399.58769	1
73	H	68.13813	70.15177	78.63353	1297.06236	2061.84854	1220.68812	1
74	C	70.36599	69.52392	79.61843	-662.15484	-412.13544	123.62687	1
75	O	70.28974	70.03039	78.51951	-540.01537	-317.05804	274.91456	1
76	N	71.17105	68.50980	80.01761	-202.76299	-690.00100	-131.49145	1
77	H	70.95772	68.30967	80.99866	-702.62132	-2617.37191	281.70413	1
78	C	72.58477	68.74254	79.75966	-491.73914	576.34187	222.73509	1
79	H	72.74597	69.82679	79.94255	1977.17978	-1823.58850	-434.68928	1
80	C	73.36632	67.78524	80.74063	-649.50797	-328.66843	306.11224	1
81	H	72.87599	66.78786	80.78024	2075.07803	1452.16490	-1004.45904	1
82	C	74.83710	67.65340	80.21030	-615.18735	-489.99689	-70.96352	1
83	H	75.45900	67.00559	80.87139	-1226.32661	-1724.38906	-1716.47170	1
84	H	74.84846	67.19892	79.18787	2381.64273	-969.84735	900.46860	1
85	H	75.34149	68.64524	80.13983	2122.74991	1702.23946	-141.36228	1
86	C	73.41860	68.43022	82.16985	-501.86074	-479.61276	-376.42482	1
87	H	74.04616	67.83216	82.87151	993.79261	-2246.95401	-1177.81305	1
88	H	73.83983	69.46247	82.14092	42.71279	-626.15391	-2651.37258	1
89	H	72.40080	68.49971	82.61632	-2026.39012	-1590.33709	-887.82922	1
90	C	72.92788	68.28369	78.24307	-672.89153	382.57394	-156.34072	1
91	O	72.94825	67.11948	77.91553	410.04627	221.86223	500.35950	1
92	N	73.30033	69.35110	77.50832	-378.11782	-443.79414	-441.10984	1
93	H	73.16135	70.26510	77.94214	2404.15973	-568.14894	1149.30085	1
94	C	73.03250	69.18519	76.09329	-544.54484	466.16019	331.29119	1
95	H	73.64813	68.29759	75.82917	-1409.80988	-170.17955	2325.32679	1
96	C	71.49530	68.83108	76.01146	-184.48284	116.78282	-758.89137	1
97	H	71.29745	67.85416	76.51556	1893.78641	1861.14652	611.04528	1
98	H	70.89062	69.57433	76.59427	-2170.79665	-974.19311	1327.50117	1
99	C	71.06354	68.76828	74.51288	389.51668	672.81878	-138.49152	1
100	H	71.38573	69.68842	73.97692	510.64577	1933.03327	1851.02484	1
101	H	71.60378	67.94933	73.98827	-2302.30869	-1105.72939	-948.89774	1
102	C	69.50869	68.57287	74.35103	-559.93427	-126.34590	542.30857	1
103	O	69.04382	68.42014	73.25101	-670.36003	-111.83166	76.36448	1
104	N	68.81899	68.62124	75.51158	-216.06729	-537.07037	446.51379	1
105	H	69.14865	68.74737	76.47624	-1251.96487	1074.12894	-2168.52500	1
106	H	67.80022	68.51851	75.57461	-2451.83023	-720.73851	944.84612	1
107	C	73.52057	70.53115	75.32167	546.24052	-563.88337	-85.11809	1
108	O	74.46588	70.37881	74.59005	71.70702	-307.40463	606.69673	1
109	N	72.82462	71.65638	75.54689	479.90863	-504.50129	222.86849	1
110	C	73.25025	72.87587	74.92959	75.51268	-600.91904	506.73700	1
111	H	73.74690	72.60639	73.96915	-2033.61562	1795.30103	254.89480	1
112	C	71.87339	73.66429	74.81747	523.79067	517.99274	284.45581	1
113	H	72.01109	74.75963	74.67814	1599.61665	-1127.00911	1895.98173	1
114	H	71.25748	73.30413	73.96359	-1881.83093	1275.82488	-1501.55016	1
115	C	71.22596	73.29196	76.18497	-600.05639	-404.05026	316.64485	1
116	H	71.73054	73.84126	77.01538	-2597.05260	496.36221	657.73249	1
117	H	70.13778	73.51995	76.25070	-1666.24518	1294.38985	-1723.90591	1
118	C	71.54869	71.73674	76.24323	493.57570	-129.95376	-602.56394	1
119	H	71.67107	71.31707	77.27062	-1469.08415	-637.84797	2204.22581	1
120	H	70.83719	71.10094	75.66547	-1038.97840	1068.88701	-2280.71789	1
121	C	74.23160	73.69640	75.95558	188.88100	241.05089	-727.87821	1
122	O	74.01036	73.78333	77.14287	531.88613	-49.19943	427.07960	1
123	N	75.19414	74.28684	75.25669	708.14833	26.93417	-179.73509	1
124	H	75.26426	74.09409	74.25823	-2232.69501	-1179.09831	1023.94816	1
125	C	76.16143	75.02020	76.00974	-738.32412	259.92548	104.42456	1
126	H	75.56366	75.46243	76.83960	2028.89123	1260.02983	1311.33324	1
127	C	77.22828	73.98640	76.58309	-258.65678	-345.52513	661.28459	1
128	H	77.72359	73.43711	75.75391	-1826.06090	-1801.46097	-918.64554	1
129	C	78.27123	74.79954	77.42130	-541.23400	531.92970	-218.41428	1
130	H	79.06904	74.14240	77.84056	1311.73218	-2219.18200	882.19023	1
131	H	78.77241	75.58101	76.79858	-1048.71298	1223.58118	-2196.97971	1
132	H	77.78693	75.32527	78.27756	419.06414	1903.92898	1903.44670	1

C	76.50898	72.98646	77.55295	-423.86431	152.66566	-648.55262	1
H	77.23554	72.31052	78.06305	105.68386	-2342.50080	1387.51238	1
H	75.93708	73.52760	78.34577	-1695.63603	-1484.73621	-1531.02793	1
H	75.77821	72.34083	77.00613	-1853.57121	-487.39031	1936.59343	1
C	76.95137	76.10095	75.07354	539.71428	-510.58232	267.58633	1
O	77.48513	77.04718	75.60684	-15.93758	-278.34085	-624.49402	1
N	77.03864	75.72479	73.80210	-676.81886	12.05292	276.17960	1
H	76.55311	74.88034	73.50215	-1694.44250	-373.84189	-2100.66098	1
C	77.45878	76.72616	72.85851	412.33078	-664.70360	108.35451	1
H	76.88512	77.63730	73.13428	1765.77476	1294.44366	1621.76594	1
C	79.00443	76.92566	73.10148	-179.66635	-721.95667	-264.73915	1
H	79.16428	77.39179	74.10225	-607.34922	2587.87032	-598.10018	1
H	79.53920	75.95052	73.14348	-359.69332	-1479.13954	-2259.74260	1
C	79.57390	77.82823	71.95651	-240.01076	616.02425	431.85322	1
H	79.96766	77.21307	71.11484	-1359.17131	782.54425	2227.99181	1
H	78.50351	80.01396	71.66786	-820.36667	85.81767	-2596.78715	1
C	80.70422	78.73401	72.59157	-513.07015	236.18570	-551.87280	1
H	80.29974	79.33752	73.43658	1816.11944	-1183.11722	-1650.94321	1
H	81.51349	78.10533	73.02868	-1800.04429	-1361.53473	1526.34643	1
C	81.26652	79.65575	71.48439	726.08972	-253.19732	-179.64713	1
H	81.77082	79.11493	70.63843	1224.27271	-456.51798	-2390.90304	1
H	80.54160	80.39751	71.08077	1206.40339	-1150.93514	-2154.90344	1
N	82.39197	80.54094	72.11005	329.70796	-633.68968	-155.67358	1
H	82.83120	81.23570	71.43962	1348.31305	1938.76845	1359.00643	1
H	82.12680	81.17309	72.92079	2096.92035	1446.54551	-966.48890	1
H	83.25169	80.04255	72.48304	-1197.07217	-2303.53719	827.28733	1
C	77.14163	76.18115	71.34663	-378.01186	-556.17301	-413.96481	1
O	76.42174	76.84008	70.62768	-409.44433	-307.55053	-453.30833	1
N	77.75040	75.02719	71.11443	-429.02477	524.70884	274.08502	1
H	78.27676	74.64319	71.89888	1706.34263	-1425.96036	1574.38926	1
C	77.87596	74.48267	69.80886	-45.25864	-626.22952	478.93267	1
H	78.33908	73.48958	70.00695	1988.63164	570.90773	1772.87287	1
C	76.42735	74.41161	69.17912	551.77128	-20.49526	-564.54996	1
H	75.69159	74.06654	69.94012	1434.61535	-1753.10253	1513.99471	1
H	76.08863	75.43064	68.87420	-714.67738	1819.09770	1898.36494	1
C	76.46992	73.43245	67.95939	446.99263	-555.77305	338.97221	1
H	77.10873	73.84207	67.14498	1953.68466	1892.25957	-161.67766	1
H	76.92532	72.45908	68.24957	-403.03785	2688.07032	-188.43591	1
C	74.98014	73.22347	67.47040	567.86262	335.74681	434.04421	1
H	74.33479	72.87487	68.30931	-1080.02275	1568.26081	-1948.78900	1
H	74.53626	74.18700	67.12910	-919.54108	2111.34692	1456.13463	1
C	74.98777	72.18312	66.32667	195.81897	173.33862	-745.11536	1
H	75.51926	72.50748	65.41001	1405.67949	-2301.21089	-390.08113	1
H	75.32920	71.17145	66.62411	-940.37328	-1118.32446	2299.72133	1
N	73.50179	71.97901	65.87457	319.26422	656.36007	-42.02424	1
H	73.35227	71.29206	65.08026	1961.03633	619.91968	1787.09422	1
H	72.82283	71.60769	66.60108	854.14343	-2156.80752	1429.08711	1
H	72.98700	72.83342	65.51182	1237.02905	-1378.50857	-1998.28500	1
C	78.84812	75.46136	68.91098	532.53319	-268.57236	517.55729	1
O	78.42026	76.10016	67.95050	266.16714	-290.73804	-558.88002	1
N	80.06126	75.40095	69.40783	-226.25352	640.49678	-270.32839	1
H	80.10556	74.80873	70.23620	2007.69063	-1035.27229	1523.50231	1
C	81.24469	75.80510	68.74013	-540.76542	-573.33303	49.50000	1
H	82.05677	75.36362	69.35921	-2374.20950	-1036.76023	843.75774	1
C	81.21265	75.21324	67.25115	-77.09082	666.87422	-415.84136	1
H	80.26895	75.55534	66.74165	-985.81834	617.19825	2463.91927	1
O	81.20852	73.77727	67.16472	293.29899	-391.65602	-477.80926	1
H	80.34367	73.61768	66.73586	2528.72714	-681.76262	-751.27122	1
C	82.44203	75.94130	66.59574	252.47363	-605.14473	440.05134	1
H	82.54344	75.68057	65.51672	-2052.65063	1456.49051	-1043.51830	1
H	82.34983	77.05201	66.65808	-2152.98800	-1661.97574	-161.70652	1
H	83.39781	75.65907	67.09554	-473.12726	-1584.27339	2165.61725	1

195	C	81.34416	77.43652	68.69921	360.36307	488.46713	505.10056	1
196	O	82.33643	77.96842	69.15313	-200.08817	-528.03552	-385.82850	1
197	N	80.31400	77.98483	68.08081	-597.54095	-226.44775	355.20479	1
198	H	79.50811	77.39593	67.74165	2179.23655	-1300.70981	-991.33835	1
199	C	80.34050	79.39014	67.90800	-687.68921	297.39807	-249.45179	1
200	H	81.40417	79.70190	68.02817	-349.16256	-2467.37713	-1101.72831	1
201	C	79.68332	79.71578	66.48075	672.47345	361.54328	-201.62880	1
202	H	78.72992	79.15365	66.35108	1541.41502	582.18425	2169.97026	1
203	H	79.43617	80.80028	66.41479	-2534.67717	60.25153	997.72295	1
204	C	80.71384	79.32330	65.41541	574.26774	-389.43119	-377.02542	1
205	C	81.74971	80.25341	65.09948	688.82679	-287.33580	-257.96133	1
206	H	81.78840	81.24265	65.59057	331.14182	1470.38163	2269.80013	1
207	C	80.67730	78.03979	64.79069	-383.52382	-675.04808	144.24917	1
208	H	79.88613	77.31192	65.04578	4.09847	1517.59662	2262.86397	1
209	C	82.71534	79.88441	64.16480	-134.76730	428.45285	-649.50303	1
210	H	83.51629	80.60398	63.92044	-903.23433	1600.49020	2011.53387	1
211	C	81.66795	77.71805	63.85959	-335.53281	-615.00040	-364.39058	1
212	H	81.63199	76.72394	63.38079	1596.45398	-1369.33862	1732.02659	1
213	C	82.71702	78.61818	63.51080	585.48547	-340.24194	-406.23938	1
214	O	83.72978	78.30213	62.56829	-419.15649	-173.04408	-511.94141	1
215	H	83.43950	77.39623	62.30894	-1071.84350	1559.71507	-1960.13012	1
216	C	79.35244	80.09377	69.00926	291.41878	-183.13091	710.72246	1
217	O	79.56941	81.22431	69.38360	379.87695	523.47396	-222.23622	1
218	N	78.37435	79.26611	69.35772	564.30591	373.09380	277.24371	1
219	H	78.37909	78.33789	68.93341	-120.59931	-2436.07824	-1214.35098	1
220	C	77.16003	79.77629	69.90498	404.07514	-299.37480	608.84009	1
221	H	76.56024	78.83686	70.02201	-1071.07882	-841.69025	2359.66467	1
222	C	77.54459	80.44839	71.29101	-683.12807	68.71770	-390.13096	1
223	H	78.76365	78.45571	71.51077	2068.88918	1596.58590	770.89603	1
224	H	77.74824	81.54289	71.17131	1379.86429	2342.49779	-179.85638	1
225	C	76.40163	80.19253	72.31903	-176.39967	537.49466	550.97268	1
226	H	75.58947	80.94771	72.23367	-396.28051	1942.22168	1869.33314	1
227	H	75.91662	79.20722	72.13994	-94.44700	586.00678	-2659.20016	1
228	C	77.08737	80.23753	73.76186	-291.69381	-718.18079	-150.72929	1
229	H	77.84555	79.43692	73.95985	-2303.38776	1089.76242	964.62032	1
230	H	77.53351	81.21846	74.06560	884.16729	2568.80533	-207.74283	1
231	N	75.97630	79.98189	74.57973	-594.49522	-252.81478	342.29276	1
232	H	75.11666	79.73367	74.11446	-1735.17426	2084.05705	-263.71605	1
233	C	75.98728	80.02570	75.85065	595.23177	-271.52487	442.22352	1
234	N	77.18873	80.36823	76.48135	585.53813	-424.79750	-105.81303	1
235	H	78.10663	80.52638	76.06803	959.88797	1327.56823	-2177.11905	1
236	H	77.33433	80.38739	77.49095	1578.48647	16.27378	-2220.76233	1
237	N	74.72183	79.71754	76.36750	-452.17763	-390.33541	-421.51853	1
238	H	73.85560	79.45243	75.90108	470.26107	1974.25629	-1817.92087	1
239	H	74.52080	79.67859	77.36734	1856.67307	-1573.97321	-1224.35416	1
240	C	76.51377	80.80924	68.82491	-531.47271	-581.45780	-55.06206	1
241	O	76.84467	80.82771	67.65255	-243.00837	192.73507	609.52485	1
242	N	75.48392	81.46956	69.37117	343.49288	-644.25607	38.09164	1
243	H	75.44927	81.24043	70.36284	-1691.16043	-1535.97525	-1484.73099	1
244	C	75.02242	82.77321	68.87553	542.66492	350.14337	454.42563	1
245	H	74.29348	83.09371	69.65745	1209.57979	-2396.95920	463.86744	1
246	C	76.19669	83.72696	68.77306	-737.14309	145.93010	242.71942	1
247	H	76.89520	83.31398	68.00973	-1050.18303	159.36419	-2509.06125	1
248	C	75.76567	85.11542	68.33644	-706.77696	-252.88343	-245.16230	1
249	H	76.64672	85.79441	68.17167	-1624.64039	-1665.49085	1417.86935	1
250	H	75.17337	85.08217	67.36943	2566.10521	598.51109	693.22305	1
251	H	75.11039	85.60537	69.10792	-1972.69758	107.10018	-1876.34312	1
252	C	76.96117	83.84490	70.08019	412.85148	672.58832	-27.69222	1
253	H	77.83786	84.54292	69.98830	1447.02277	1762.76533	-1490.78944	1
254	H	76.30839	84.23708	70.90774	1320.77379	-145.20455	2378.68491	1
255	H	77.36297	82.84305	70.41452	-1512.10848	1470.52031	1724.75147	1
256	C	74.29857	82.55026	67.41043	-607.11113	499.56689	73.73769	1

```
O    74.10518   83.50150   66.67486       357.97126       460.81534       356.68785          1
N    73.92231   81.29805   67.22877       536.55407      -368.62270       332.76306          1
H    74.17778   80.64215   67.96397       524.38866      2297.69985      1367.20941          1
C    73.33011   80.91390   66.00170        46.50277      -774.29190      -147.97904          1
H    73.00478   81.86919   65.52954      1676.51875      1883.29066      1032.55549          1
C    74.40270   80.10272   65.10374      -559.41456       -34.19198      -556.30433          1
H    74.64420   79.13918   65.61264       -64.66664       395.18769      2695.05373          1
H    73.94684   79.82391   64.12412     -2703.44278       -60.40056       333.79418          1
C    75.66037   80.90102   64.89974      -519.76435       377.31045       459.42366          1
H    76.10350   81.12088   65.92255     -1746.74323      1667.50644     -1261.73691          1
C    76.67938   80.09272   64.10645       570.95554        16.46261      -545.27700          1
H    77.64161   80.65262   63.97766      2391.58273        28.05277     -1305.07271          1
H    76.92588   79.12591   64.61661       175.75588       917.63458      2559.43867          1
H    76.30119   79.84181   63.08210     -1063.97851     -2155.34650      1283.00462          1
C    75.38253   82.21663   64.19021       193.76014         2.62946      -765.53141          1
H    76.33080   82.74671   63.91417       507.39394      -846.55087      2539.60089          1
H    74.79890   82.06563   63.24520      1337.57841      1818.19054      1526.01994          1
H    74.78928   82.91751   64.85317      1100.88635     -1172.91350     -2199.08909          1
C    72.15230   79.86710   66.34205      -549.21115       475.64486      -309.38218          1
O    72.06381   79.27848   67.38750       556.06106      -390.37755       -78.24250          1
O    71.27354   79.57012   65.27177      -572.27940      -310.19032      -209.75862          1
H    71.62382   80.21887   64.60119     -1380.08193      2212.14486       790.85944          1
```

Listing 65: C00065.xmolout

```
      276
  150.0000  150.0000  150.0000     0.0000     0.0000     0.0000***********    2.7750    11100
      0.2500    300.00   0.00   0.00   0.00  -1293.6047       0.0000    300.0000
H    77.68201   85.57534   79.91449     -2043.14101     -1757.64893       399.89339          1
N    78.18837   86.21783   80.52109       426.75047       490.45733      -334.42467          1
H    78.65034   85.58419   81.17055       442.25559     -1110.30854     -2448.52907          1
C    79.06743   87.11451   79.87739       415.32972      -666.39213        83.73172          1
H    78.61109   87.32671   78.88829       879.32219      2491.00447       667.35213          1
C    79.20590   88.40956   80.82071        31.35357       477.41777       628.23362          1
H    79.60463   88.09689   81.81617      1168.38489      2240.25337     -1019.71136          1
H    79.94675   89.13219   80.40016      -360.43770      2534.90498       931.67112          1
C    77.84304   89.04307   80.96662       463.18208       512.70242      -382.34388          1
H    77.11046   88.23434   81.18950      1725.29117      1863.52247      -987.07854          1
C    77.82974   90.03299   82.12342       710.42801      -144.27427      -313.15367          1
H    76.81887   90.49870   82.25719       364.20195     -1033.13725      2494.72524          1
H    78.09925   89.54016   83.09351      2567.61904       335.80679       847.48802          1
H    78.56002   90.86741   81.96048     -2587.99029       680.92867      -512.16411          1
C    77.41851   89.74546   79.68490       268.17616       741.64379       -40.42634          1
H    76.41156   90.22632   79.79122      1283.54314     -1945.37420     -1411.27812          1
H    78.14231   90.55163   79.39768     -1258.57796      1317.15249     -2026.02085          1
H    77.35556   89.03354   78.82192     -2502.84526       909.80597       575.92391          1
C    80.52176   86.35302   79.79438       619.36578      -458.64843      -172.09273          1
O    81.17408   86.08732   80.77790       -81.40433       433.87611       522.34594          1
N    80.78194   86.10612   78.51304       175.23996      -622.20190       341.55523          1
H    80.13981   86.47716   77.81298      -876.57256      -303.22879     -2561.90213          1
C    81.93392   85.38037   78.10396      -571.88214      -534.10783      -106.15033          1
H    81.84768   85.36415   76.99422     -2713.20841       -16.25800      -248.80809          1
C    83.18004   86.27414   78.58477       166.60354       288.16399      -716.09568          1
H    83.18639   86.24471   79.71055     -2097.85574      1636.78449      -586.18044          1
O    83.10444   87.66268   78.21002      -317.80580      -363.38227       484.43082          1
H    82.98190   88.07862   79.08650      1863.74748     -1451.84923      1357.29467          1
C    84.45583   85.54436   78.03034       503.80731       517.99032       318.51606          1
H    85.38802   86.07046   78.34410       640.61020     -2157.10410     -1536.29104          1
H    84.53902   84.47884   78.38961      1687.88455       465.60692      2087.56452          1
H    84.45551   85.51796   76.91545     -1293.07895     -1899.88581     -1463.57452          1
C    81.93123   83.89141   78.74506      -153.07550      -733.68902       248.70981          1
O    82.99269   83.31042   78.77366       186.15459      -480.78787      -449.34387          1
```

N	80.72706	83.39898	79.06749	-487.53139	543.18517	42.03850	1
C	80.58736	82.40227	80.08542	-699.35871	69.34393	360.10701	1
H	81.31831	82.64841	80.88877	2.81126	-221.99807	-2715.58072	1
C	79.05843	82.53815	80.47932	-507.46439	-592.59194	122.07761	1
H	78.65445	81.61825	80.95943	-1975.08116	525.87560	-1801.71556	1
H	78.89632	83.37136	81.19861	1588.27743	-1913.22100	1113.83566	1
C	78.40382	82.84768	79.09557	-613.62442	-457.55993	-194.14490	1
H	78.30260	81.92002	78.48830	1707.54637	-721.27992	1996.92523	1
H	77.38490	83.28914	79.17182	758.23357	2100.81767	1560.55011	1
C	79.47241	83.84323	78.47192	-352.41524	704.96076	49.21465	1
H	79.57293	83.82086	77.36118	1714.76886	-2070.42778	443.35792	1
H	79.37019	84.90316	78.81132	1224.15040	1801.50881	1636.97633	1
C	80.85861	80.91119	79.45245	566.85117	537.05831	117.62814	1
O	81.12174	79.98418	80.18258	-550.26779	-406.08867	4.21666	1
N	80.63670	80.90629	78.14058	566.34832	-425.77725	180.18749	1
H	80.43316	81.81514	77.73043	644.44502	2646.80550	-52.73430	1
C	80.85348	79.71950	77.40392	612.34801	-281.48662	411.56221	1
H	81.26837	78.99645	78.14497	1667.23498	-1490.75142	1556.16753	1
C	79.45959	79.25782	76.75807	-714.45746	-175.43074	286.98927	1
H	79.12417	80.03369	76.01617	-787.09484	1763.03619	-1922.46060	1
O	78.36519	79.08624	77.67155	-429.46514	516.51932	-128.40526	1
H	77.71200	79.72016	77.31200	1981.82785	436.16847	-1818.18182	1
C	79.91921	77.96638	75.98782	630.79189	-468.94448	76.03230	1
H	79.08674	77.53232	75.38387	-1775.71861	2062.30353	131.89849	1
H	80.77013	78.17325	75.29334	-1855.14608	88.94118	1993.53760	1
H	80.25753	77.17470	76.69589	-1504.82350	-780.94249	-2132.90992	1
C	81.84806	80.08588	76.14065	288.57638	618.62814	-397.00270	1
O	82.69763	79.26844	75.85209	321.70495	-185.26452	574.37218	1
N	81.42302	81.15082	75.47621	-447.05792	-446.53431	367.76425	1
H	80.61966	81.59376	75.92217	350.31316	1975.63644	-1843.31522	1
C	82.20399	81.95156	74.59608	-511.23061	374.03917	-471.51480	1
H	81.42995	82.64023	74.18814	2234.79579	-898.70890	-1273.45190	1
C	83.24603	82.82266	75.45802	529.53781	-159.34528	563.72604	1
H	83.72323	83.53164	74.72690	654.59388	2347.94251	1217.51488	1
H	82.65171	83.44430	76.17974	-1526.55661	2103.42026	817.87394	1
O	84.29597	82.11039	76.15264	-554.84278	14.12789	399.58769	1
H	83.94490	82.15838	77.06980	1297.06236	2061.84854	1220.68812	1
C	82.93153	81.12029	73.42244	-662.15484	-412.13544	123.62687	1
O	83.74718	81.65421	72.71223	-540.01537	-317.05804	274.91456	1
N	82.38044	79.89005	73.25838	-202.76299	-690.00100	-131.49145	1
H	81.77357	79.65189	74.05658	-702.62132	-2617.37191	281.70413	1
C	83.19339	78.79681	72.69232	-491.73914	576.34187	222.73509	1
H	83.56478	79.23491	71.73660	1977.17978	-1823.58850	-434.68928	1
C	84.31996	78.46126	73.64310	-649.50797	-328.66843	306.11224	1
H	83.86518	78.02828	74.57038	2075.07803	1452.16490	-1004.45904	1
C	85.26380	77.42964	73.05187	-615.18735	-489.99689	-70.96352	1
H	86.06678	77.13237	73.78065	-1226.32661	-1724.38906	-1716.47170	1
H	84.70575	76.48982	72.75827	2381.64273	-969.84735	900.46860	1
H	85.77741	77.81648	72.12970	2122.74991	1702.23946	-141.36228	1
C	85.10731	79.68589	74.07448	-501.86074	-479.61276	-376.42482	1
H	85.98567	79.41987	74.72366	993.79261	-2246.95401	-1177.81305	1
H	85.49784	80.26259	73.18861	42.71279	-626.15391	-2651.37258	1
H	84.45234	80.39662	74.66551	-2026.39012	-1590.33709	-887.82922	1
C	82.18701	77.50276	72.48940	-672.89153	382.57394	-156.34072	1
O	82.63072	76.36722	72.50185	410.04627	221.86223	500.35950	1
N	80.93923	77.87592	72.28965	-378.11782	-443.79414	-441.10984	1
H	80.81086	78.88355	72.23629	2404.15973	-568.14894	1149.30085	1
C	79.89109	76.92567	72.14359	-544.54484	466.16019	331.29119	1
H	80.23917	76.02806	72.69908	-1409.80988	-170.17955	2325.32679	1
C	78.59443	77.63200	72.71292	-184.48284	116.78282	-758.89137	1
H	78.75706	77.93747	73.77176	1893.78641	1861.14652	611.04528	1
H	78.38935	78.57355	72.15431	-2170.79665	-974.19311	1327.50117	1

C	77.38628	76.64983	72.59058	389.51668	672.81878	-138.49152	1
H	77.21309	76.38318	71.52169	510.64577	1933.03327	1851.02484	1
H	77.62412	75.67483	73.07167	-2302.30869	-1105.72939	-948.89774	1
C	76.06567	77.23755	73.20747	-559.93427	-126.34590	542.30857	1
O	75.02451	76.64074	73.06825	-670.36003	-111.83166	76.36448	1
N	76.23539	78.39484	73.87710	-216.06729	-537.07037	446.51379	1
H	77.09552	78.92468	74.06045	-1251.96487	1074.12894	-2168.52500	1
H	75.48840	78.87773	74.38889	-2451.83023	-720.73851	944.84612	1
C	79.70937	76.67429	70.53222	546.24052	-563.88337	-85.11809	1
O	79.24032	77.54367	69.84732	71.70702	-307.40463	606.69673	1
N	80.11440	75.44231	70.18913	479.90863	-504.50129	222.86849	1
C	80.30526	75.09049	68.81432	75.51268	-600.91904	506.73700	1
H	80.65754	76.01150	68.29539	-2033.61562	1795.30103	254.89480	1
C	81.36935	73.91411	68.90630	523.79067	517.99274	284.45581	1
H	81.33060	73.22957	68.02940	1599.61665	-1127.00911	1895.98173	1
H	82.40831	74.30970	68.95335	-1881.83093	1275.82488	-1501.55016	1
C	80.97358	73.22080	70.24711	-600.05639	-404.05026	316.64485	1
H	80.08188	72.56804	70.11179	-2597.05260	496.36221	657.73249	1
H	81.77679	72.57839	70.67369	-1666.24518	1294.38985	-1723.90591	1
C	80.63308	74.48057	71.15201	493.57570	-129.95376	-602.56394	1
H	79.84964	74.30298	71.92674	-1469.08415	-637.84797	2204.22581	1
H	81.52658	74.99436	71.62731	-1038.97840	1068.88701	-2280.71789	1
C	78.91641	74.53250	68.15614	188.88100	241.05089	-727.87821	1
O	78.84314	74.44566	66.95632	531.88613	-49.19943	427.07960	1
N	78.05703	74.11369	69.07337	708.14833	26.93417	-179.73509	1
H	78.19889	74.24214	70.09333	-2232.69501	-1179.09831	1023.94816	1
C	76.72740	73.77302	68.67476	-738.32412	259.92548	104.42456	1
H	76.46596	74.36585	67.77007	2028.89123	1260.02983	1311.33324	1
C	76.70314	72.20388	68.40460	-258.65678	-345.52513	661.28459	1
H	77.17164	71.66303	69.25480	-1826.06090	-1801.46097	-918.64554	1
C	75.22677	71.73315	68.18538	-541.23400	531.92970	-218.41428	1
H	75.17838	70.65083	67.91840	1311.73218	-2219.18200	882.19023	1
H	74.60057	71.87570	69.12027	-1048.71298	1223.58118	-2196.97971	1
H	74.73370	72.29931	67.36003	419.06414	1903.92898	1903.44670	1
C	77.46378	71.89120	67.07092	-423.86431	152.66566	-648.55262	1
H	77.43085	70.80513	66.81888	105.68386	-2342.50080	1387.51238	1
H	77.02506	72.44853	66.20994	-1695.63603	-1484.73621	-1531.02793	1
H	78.53868	72.18793	67.13749	-1853.57121	-487.39031	1936.59343	1
C	75.94799	74.14918	70.05609	539.71428	-510.58232	267.58633	1
O	76.61462	74.22377	71.07969	-15.93758	-278.34085	-624.49402	1
N	74.65696	74.35377	69.86357	-676.81886	12.05292	276.17960	1
H	74.29606	74.22334	68.91828	-1694.44250	-373.84189	-2100.66098	1
C	73.77145	74.45827	70.97286	412.33078	-664.70360	108.35451	1
H	74.48732	74.61971	71.81653	1765.77476	1294.44366	1621.76594	1
C	72.81425	75.68749	70.68161	-179.66635	-721.95667	-264.73915	1
H	73.36100	76.47021	70.10992	-607.34922	2587.87032	-598.10018	1
H	71.97647	75.36916	70.02179	-359.69332	-1479.13954	-2259.74260	1
C	72.26660	76.28911	72.01809	-240.01076	616.02425	431.85322	1
H	71.88285	75.49782	72.70246	-1359.17131	782.54425	2227.99181	1
H	73.08546	76.80551	72.59245	-820.36667	85.81767	-2596.78715	1
C	71.10702	77.28670	71.61655	-513.07015	236.18570	-551.87280	1
H	71.52309	78.23955	71.21553	1816.11944	-1183.11722	-1650.94321	1
H	70.48645	76.85940	70.79562	-1800.04429	-1361.53473	1526.34643	1
C	70.22650	77.53644	72.86267	726.08972	-253.19732	-179.64713	1
H	69.69464	76.63824	73.23636	1224.27271	-456.51798	-2390.90304	1
H	70.73795	78.05123	73.70053	1206.40339	-1150.93514	-2154.90344	1
N	69.08133	78.51077	72.42333	329.70796	-633.68968	-155.67358	1
H	68.36235	78.74549	73.16677	1348.31305	1938.76845	1359.00643	1
H	69.36677	79.47828	72.09261	2096.92035	1446.54551	-966.48890	1
H	68.45621	78.18321	71.63042	-1197.07217	-2303.53719	827.28733	1
C	72.95154	73.04563	71.05753	-378.01186	-556.17301	-413.96481	1
O	73.47470	72.00067	70.71829	-409.44433	-307.55053	-453.30833	1

N	71.75592	73.21220	71.59063	-429.02477	524.70884	274.08502	1
H	71.41943	74.14747	71.79740	1706.34263	-1425.96036	1574.38926	1
C	70.84714	72.09589	71.59859	-45.25864	-626.22952	478.93267	1
H	70.83818	71.75006	70.54159	1988.63164	570.90773	1772.87287	1
C	71.47621	71.01484	72.55624	551.77128	-20.49526	-564.54996	1
H	72.45469	70.65802	72.15504	1434.61535	-1753.10253	1513.99471	1
H	71.71233	71.45156	73.55168	-714.67738	1819.09770	1898.36494	1
C	70.45208	69.82830	72.68603	446.99263	-555.77305	338.97221	1
H	69.43583	70.19658	72.95686	1953.68466	1892.25957	-161.67766	1
H	70.33988	69.29425	71.71492	-403.03785	2688.07032	-188.43591	1
C	71.02077	68.88476	73.79366	567.86262	335.74681	434.04421	1
H	72.08936	68.64681	73.59953	-1080.02275	1568.26081	-1948.78900	1
H	71.00153	69.37324	74.79191	-919.54108	2111.34692	1456.13463	1
C	70.13965	67.54866	73.78452	195.81897	173.33862	-745.11536	1
H	69.06870	67.69660	74.07538	1405.67949	-2301.21089	-390.08113	1
H	70.20201	67.00173	72.81091	-940.37328	-1118.32446	2299.72133	1
N	70.71149	66.68206	74.76888	319.26422	656.36007	-42.02424	1
H	69.36968	64.43813	75.97378	1961.03633	619.91968	1787.09422	2
H	71.71655	66.71511	74.89840	854.14343	-2156.80752	1429.08711	1
H	70.36109	66.79144	75.71517	1237.02905	-1378.50857	-1998.28500	1
C	69.38636	72.60996	72.12492	532.53319	-268.57236	517.55729	1
O	68.48443	72.73030	71.29127	266.16714	-290.73804	-558.88002	1
N	69.35743	72.78956	73.41653	-226.25352	640.49678	-270.32839	1
H	70.26802	72.70196	73.86156	2007.69063	-1035.27229	1523.50231	1
C	68.18979	73.12644	74.17599	-540.76542	-573.33303	49.50000	1
H	68.23381	74.23298	74.27097	-2374.20950	-1036.76023	843.75774	1
C	66.87061	72.67747	73.39650	-77.09082	666.87422	-415.84136	1
H	67.11955	71.73858	72.82498	-985.81834	617.19825	2463.91927	1
O	66.42371	73.62563	72.42839	293.29899	-391.65602	-477.80926	1
H	67.03220	73.35093	71.63564	2528.72714	-681.76262	-751.27122	1
C	65.86192	72.32027	74.54866	252.47363	-605.14473	440.05134	1
H	64.87100	72.00550	74.14493	-2052.65063	1456.49051	-1043.51830	1
H	66.24202	71.48803	75.19049	-2152.98800	-1661.97574	-161.70652	1
H	65.68988	73.19555	75.22130	-473.12726	-1584.27339	2165.61725	1
C	68.31149	72.43287	75.63771	360.36307	488.46713	505.10056	1
O	67.54912	72.82976	76.49891	-200.08817	-528.03552	-385.82850	1
N	69.20135	71.45215	75.69875	-597.54095	-226.44775	355.20479	1
H	69.74292	71.26786	74.85577	2179.23655	-1300.70981	-991.33835	1
C	69.44164	70.71299	76.88021	-687.68921	297.39807	-249.45179	1
H	70.46221	70.29049	76.74738	-349.16256	-2467.37713	-1101.72831	1
C	69.24727	71.57263	78.21815	672.47345	361.54328	-201.62880	1
H	68.21497	72.01859	78.24553	1541.41502	582.18425	2169.97026	1
H	69.32797	70.88891	79.09838	-2534.67717	60.25153	997.72295	1
C	70.31189	72.67731	78.27614	574.26774	-389.43119	-377.02542	1
C	71.66003	72.36055	78.61857	688.82679	-287.33580	-257.96133	1
H	71.95884	71.32016	78.84065	331.14182	1470.38163	2269.80013	1
C	69.92986	74.01910	77.97538	-383.52382	-675.04808	144.24917	1
H	68.88507	74.25233	77.69070	4.09847	1517.59662	2262.86397	1
C	72.58841	73.39934	78.67078	-134.76730	428.45285	-649.50303	1
H	73.63244	73.16133	78.93891	-903.23433	1600.49020	2011.53387	1
C	70.89662	75.02461	78.04638	-335.53281	-615.00040	-364.39058	1
H	70.59147	76.06169	77.82196	1596.45398	-1369.33862	1732.02659	1
C	72.25184	74.75670	78.39767	585.48547	-340.24194	-406.23938	1
O	73.24390	75.76817	78.47557	-419.15649	-173.04408	-511.94141	1
H	72.69809	76.55612	78.24511	-1071.84350	1559.71507	-1960.13012	1
C	68.31105	69.56338	77.11521	291.41878	-183.13091	710.72246	1
O	68.13735	69.13546	78.24061	379.87695	523.47396	-222.23622	1
N	67.72316	69.16979	75.98817	564.30591	373.09380	277.24371	1
H	68.01930	69.60872	75.11675	-120.59931	-2436.07824	-1214.35098	1
C	66.77763	68.12165	76.07354	404.07514	-299.37480	608.84009	1
H	66.37966	68.20869	77.10964	-1071.07882	-841.69025	2359.66467	1
C	65.74459	68.29209	74.90426	-683.12807	68.71770	-390.13096	1

```
H   65.26876  69.29891  74.93456     2068.88918     1596.58590      770.89603         1
H   66.26060  68.23065  73.91882     1379.86429     2342.49779     -179.85638         1
C   64.67414  67.15867  75.03780     -176.39967      537.49466      550.97268         1
H   65.16563  66.15722  75.05131     -396.28051     1942.22168     1869.33314         1
H   64.11473  67.23931  75.99617      -94.44700      586.00678    -2659.20016         1
C   63.70342  67.31095  73.77958     -291.69381     -718.18079     -150.72929         1
H   63.22980  68.31645  73.64578    -2303.38776     1089.76242      964.62032         1
H   64.16233  67.01185  72.80245      884.16729     2568.80533     -207.74283         1
N   62.68246  66.39459  74.06660     -594.49522     -252.81478      342.29276         1
H   62.86670  65.72164  74.79423    -1735.17426     2084.05705     -263.71605         1
C   61.56292  66.34766  73.46520      595.23177     -271.52487      442.22352         1
N   61.32878  67.27734  72.44492      585.53813     -424.79750     -105.81303         1
H   61.94183  68.00078  72.07260      959.88797     1327.56823    -2177.11905         1
H   60.47640  67.32571  71.88664     1578.48647       16.27378    -2220.76233         1
N   60.75455  65.32072  73.97430     -452.17763     -390.33541     -421.51853         1
H   60.92052  64.64425  74.71740      470.26107     1974.25629    -1817.92087         1
H   59.81637  65.12568  73.62322     1856.67307    -1573.97321    -1224.35416         1
C   67.60134  66.66576  75.84146     -531.47271     -581.45780      -55.06206         1
O   67.43176  65.99267  74.80161     -243.00837      192.73507      609.52485         1
N   68.37081  66.38429  76.82074      343.49288     -644.25607       38.09164         1
H   68.35903  67.08075  77.54994    -1691.16043    -1535.97525    -1484.73099         1
C   70.17755  63.75833  75.60728      542.66492      350.14337      454.42563         2
H   69.87712  62.73400  75.93316     1209.57979    -2396.95920      463.86744         2
C   70.28462  63.81329  74.10441     -737.14309      145.93010      242.71942         2
H   70.75756  64.78284  73.82621    -1050.18303      159.36419    -2509.06125         2
C   71.15257  62.68517  73.56704     -706.77696     -252.88343     -245.16230         2
H   71.26948  62.74305  72.45356    -1624.64039    -1665.49085     1417.86935         2
H   72.18353  62.72206  74.01903     2566.10521      598.51109      693.22305         2
H   70.71946  61.67907  73.80508    -1972.69758      107.10018    -1876.34312         2
C   68.90060  63.76768  73.48105      412.85148      672.58832      -27.69222         2
H   68.93746  63.89804  72.36855     1447.02277     1762.76533    -1490.78944         2
H   68.38476  62.79378  73.68492     1320.77379     -145.20455     2378.68491         2
H   68.23399  64.61306  73.91356    -1512.10848     1470.52031     1724.75147         2
C   71.59563  64.17000  76.29125     -607.11113      499.56689       73.73769         2
O   72.65739  63.90179  75.77358      357.97126      460.81534      356.68785         2
N   71.37744  64.81990  77.43393      536.55407     -368.62270      332.76306         2
H   70.41192  64.95285  77.73849      524.38866     2297.69985     1367.20941         2
C   72.43636  64.82573  78.38041       46.50277     -774.29190     -147.97904         2
H   73.29437  64.33959  77.86042     1676.51875     1883.29066     1032.55549         2
C   72.73832  66.34921  78.82514     -559.41456      -34.19198     -556.30433         2
H   71.85779  66.76343  79.37195      -64.66664      395.18769     2695.05373         2
H   73.58861  66.37860  79.54744    -2703.44278      -60.40056      333.79418         2
C   73.03574  67.18000  77.60569     -519.76435      377.31045      459.42366         2
H   72.13683  67.16467  76.94976    -1746.74323     1667.50644    -1261.73691         2
C   73.31776  68.62368  78.00209      570.95554       16.46261     -545.27700         2
H   73.52377  69.26706  77.10822     2391.58273       28.05277    -1305.07271         2
H   72.45005  69.07772  78.54636      175.75588      917.63458     2559.43867         2
H   74.20875  68.69914  78.67729    -1063.97851    -2155.34650     1283.00462         2
C   74.20795  66.61036  76.81945      193.76014        2.62946     -765.53141         2
H   74.50511  67.28078  75.97234      507.39394     -846.55087     2539.60089         2
H   75.11268  66.47311  77.46663     1337.57841     1818.19054     1526.01994         2
H   73.95373  65.60701  76.37573     1100.88635    -1172.91350    -2199.08909         2
C   71.91894  64.03707  79.68923     -549.21115      475.64486     -309.38218         2
O   70.76289  63.77686  79.89604      556.06106     -390.37755      -78.24250         2
O   72.90984  63.73737  80.65494     -572.27940     -310.19032     -209.75862         2
H   73.70904  64.07738  80.16564    -1380.08193     2212.14486      790.85944         2
```

Listing 66: C00066.xmolout

```
       276
  150.0000  150.0000  150.0000     0.0000     0.0000     0.0000***********    2.7750    11100
       0.2500     300.00    0.00    0.00    0.00   -1293.6022       0.0000     300.0000
```

H	79.78072	61.88150	67.99571	-2043.14101	-1757.64893	399.89339	1
N	79.48041	62.84407	67.84501	426.75047	490.45733	-334.42467	1
H	79.07510	63.05209	68.75748	442.25559	-1110.30854	-2448.52907	1
C	80.54964	63.70911	67.49957	415.32972	-666.39213	83.73172	1
H	81.42382	63.36009	68.09132	879.32219	2491.00447	667.35213	1
C	80.81239	63.61287	65.91464	31.35357	477.41777	628.23362	1
H	79.92400	64.02815	65.38013	1168.38489	2240.25337	-1019.71136	1
H	81.67028	64.27175	65.63703	-360.43770	2534.90498	931.67112	1
C	81.06401	62.19297	65.47205	463.18208	512.70242	-382.34388	1
H	80.21818	61.56013	65.82255	1725.29117	1863.52247	-987.07854	1
C	81.12022	62.12159	63.95112	710.42801	-144.27427	-313.15367	1
H	81.28436	61.07325	63.59079	364.20195	-1033.13725	2494.72524	1
H	80.17035	62.48936	63.48358	2567.61904	335.80679	847.48802	1
H	81.95460	62.74719	63.54069	-2587.99029	680.92867	-512.16411	1
C	82.35899	61.64620	66.05570	268.17616	741.64379	-40.42634	1
H	82.56368	60.60273	65.70120	1283.54314	-1945.37420	-1411.27812	1
H	83.23977	62.27435	65.76256	-1258.57796	1317.15249	-2026.02085	1
H	82.33075	61.61630	67.17541	-2502.84526	909.80597	575.92391	1
C	80.13574	65.24317	67.83333	619.36578	-458.64843	-172.09273	1
O	80.98812	66.08670	67.99831	-81.40433	433.87611	522.34594	1
N	78.80946	65.42940	67.73192	175.23996	-622.20190	341.55523	1
H	78.25386	64.58026	67.62970	-876.57256	-303.22879	-2561.90213	1
C	78.28745	66.59864	68.37442	-571.88214	-534.10783	-106.15033	1
H	79.04382	67.39505	68.18287	-2713.20841	-16.25800	-248.80809	1
C	76.85194	66.86054	67.72744	166.60354	288.16399	-716.09568	1
H	76.18980	65.98738	67.97910	-2097.85574	1636.78449	-586.18044	1
O	76.82704	66.97458	66.28985	-317.80580	-363.38227	484.43082	1
H	76.29225	66.19424	66.04757	1863.74748	-1451.84923	1357.29467	1
C	76.34939	68.13428	68.49945	503.80731	517.99032	318.51606	1
H	75.30802	68.40002	68.20219	640.61020	-2157.10410	-1536.29104	1
H	76.34712	67.99202	69.62531	1687.88455	465.60692	2087.56452	1
H	76.98630	69.02143	68.27566	-1293.07895	-1899.88581	-1463.57452	1
C	78.12296	66.22180	69.95269	-153.07550	-733.68902	248.70981	1
O	77.70207	65.14092	70.26625	186.15459	-480.78787	-449.34387	1
N	78.43823	67.27270	70.72002	-487.53139	543.18517	42.03850	1
C	78.14729	67.29117	72.12528	-699.35871	69.34393	360.10701	1
H	78.45663	66.29805	72.52436	2.81126	-221.99807	-2715.58072	1
C	79.00547	68.51913	72.64737	-507.46439	-592.59194	122.07761	1
H	78.56031	69.00238	73.54650	-1975.08116	525.87560	-1801.71556	1
H	80.03117	68.20042	72.93873	1588.27743	-1913.22100	1113.83566	1
C	79.04262	69.45998	71.40336	-613.62442	-457.55993	-194.14490	1
H	78.08564	70.01716	71.29326	1707.54637	-721.27992	1996.92523	1
H	79.85579	70.21954	71.43969	758.23357	2100.81767	1560.55011	1
C	79.22942	68.39471	70.23753	-352.41524	704.96076	49.21465	1
H	78.85107	68.71007	69.23599	1714.76886	-2070.42778	443.35792	1
H	80.26905	68.00137	70.12800	1224.15040	1801.50881	1636.97633	1
C	76.55384	67.55559	72.35516	566.85117	537.05831	117.62814	1
O	75.82470	68.03082	71.50891	-550.26779	-406.08867	4.21666	1
N	76.27148	67.24824	73.61543	566.34832	-425.77725	180.18749	1
H	77.02560	66.89181	74.20022	644.44502	2646.80550	-52.73430	1
C	74.90320	67.03208	73.95379	612.34801	-281.48662	411.56221	1
H	74.38935	66.78108	72.99833	1667.23498	-1490.75142	1556.16753	1
C	74.88319	65.89300	75.07247	-714.45746	-175.43074	286.98927	1
H	75.47980	66.25105	75.95631	-787.09484	1763.03619	-1922.46060	1
O	75.46387	64.63290	74.68888	-429.46514	516.51932	-128.40526	1
H	76.18901	64.54840	75.33931	1981.82785	436.16847	-1818.18182	1
C	73.35891	65.85807	75.46060	630.79189	-468.94448	76.03230	1
H	73.16746	65.15141	76.30086	-1775.71861	2062.30353	131.89849	1
H	72.99154	66.86086	75.78040	-1855.14608	88.94118	1993.53760	1
H	72.72695	65.53140	74.60249	-1504.82350	-780.94249	-2132.90992	1
C	74.32988	68.40521	74.64455	288.57638	618.62814	-397.00270	1
O	74.86931	68.95071	75.57780	321.70495	-185.26452	574.37218	1

N	73.14640	68.66914	74.10292	-447.05792	-446.53431	367.76425	1
H	72.78744	68.13374	73.31357	350.31316	1975.63644	-1843.31522	1
C	72.44176	69.80145	74.59687	-511.23061	374.03917	-471.51480	1
H	72.82024	69.96420	75.63391	2234.79579	-898.70890	-1273.45190	1
C	72.90290	71.00065	73.62455	529.53781	-159.34528	563.72604	1
H	72.44913	71.95135	74.01466	654.59388	2347.94251	1217.51488	1
H	74.01421	71.08989	73.76250	-1526.55661	2103.42026	817.87394	1
O	72.58010	70.87620	72.22087	-554.84278	14.12789	399.58769	1
H	73.19525	70.15287	71.98158	1297.06236	2061.84854	1220.68812	1
C	70.86231	69.45074	74.48458	-662.15484	-412.13544	123.62687	1
O	70.36755	68.82173	73.58161	-540.01537	-317.05804	274.91456	1
N	70.20942	70.06117	75.50718	-202.76299	-690.00100	-131.49145	1
H	70.86274	70.44962	76.19000	-702.62132	-2617.37191	281.70413	1
C	68.94322	69.45472	75.95604	-491.73914	576.34187	222.73509	1
H	68.57644	69.01964	74.99535	1977.17978	-1823.58850	-434.68928	1
C	69.21088	68.40548	77.01248	-649.50797	-328.66843	306.11224	1
H	69.79332	68.88236	77.83232	2075.07803	1452.16490	-1004.45904	1
C	67.92290	67.86342	77.60477	-615.18735	-489.99689	-70.96352	1
H	68.11779	67.08513	78.39294	-1226.32661	-1724.38906	-1716.47170	1
H	67.31533	68.69171	78.08635	2381.64273	-969.84735	900.46860	1
H	67.27355	67.38757	76.81922	2122.74991	1702.23946	-141.36228	1
C	70.03753	67.25309	76.46754	-501.86074	-479.61276	-376.42482	1
H	70.26359	66.48657	77.25849	993.79261	-2246.95401	-1177.81305	1
H	69.50225	66.72340	75.63208	42.71279	-626.15391	-2651.37258	1
H	71.01913	67.60969	76.05212	-2026.39012	-1590.33709	-887.82922	1
C	68.02954	70.72736	76.49708	-672.89153	382.57394	-156.34072	1
O	67.37825	70.66305	77.51668	410.04627	221.86223	500.35950	1
N	68.08488	71.70634	75.61083	-378.11782	-443.79414	-441.10984	1
H	68.67587	71.48864	74.80957	2404.15973	-568.14894	1149.30085	1
C	67.43278	72.95702	75.78207	-544.54484	466.16019	331.29119	1
H	67.66831	73.50625	74.84140	-1409.80988	-170.17955	2325.32679	1
C	65.88095	72.65850	76.00201	-184.48284	116.78282	-758.89137	1
H	65.54913	71.82567	75.34220	1893.78641	1861.14652	611.04528	1
H	65.71687	72.30660	77.04857	-2170.79665	-974.19311	1327.50117	1
C	65.07102	73.95774	75.70946	389.51668	672.81878	-138.49152	1
H	65.38461	74.77106	76.40445	510.64577	1933.03327	1851.02484	1
H	65.32788	74.34936	74.70060	-2302.30869	-1105.72939	-948.89774	1
C	63.51050	73.76698	75.81096	-559.93427	-126.34590	542.30857	1
O	62.78859	74.70898	75.60798	-670.36003	-111.83166	76.36448	1
N	63.12060	72.51063	76.11612	-216.06729	-537.07037	446.51379	1
H	63.68798	71.68389	76.33625	-1251.96487	1074.12894	-2168.52500	1
H	62.14202	72.23173	76.24729	-2451.83023	-720.73851	944.84612	1
C	67.90217	73.72076	77.13364	546.24052	-563.88337	-85.11809	1
O	67.00467	74.18068	77.82858	71.70702	-307.40463	606.69673	1
N	69.21583	73.86393	77.27003	479.90863	-504.50129	222.86849	1
C	69.82984	74.23531	78.51807	75.51268	-600.91904	506.73700	1
H	69.25457	73.67931	79.29394	-2033.61562	1795.30103	254.89480	1
C	71.33469	73.77419	78.33361	523.79067	517.99274	284.45581	1
H	72.04059	74.38631	78.94049	1599.61665	-1127.00911	1895.98173	1
H	71.47502	72.71728	78.65354	-1881.83093	1275.82488	-1501.55016	1
C	71.56180	73.92684	76.79618	-600.05639	-404.05026	316.64485	1
H	71.77857	74.99952	76.49930	-2597.05260	496.36221	657.73249	1
H	72.40872	73.31399	76.40995	-1666.24518	1294.38985	-1723.90591	1
C	70.15440	73.46597	76.22648	493.57570	-129.95376	-602.56394	1
H	69.84341	73.94780	75.26982	-1469.08415	-637.84797	2204.22581	1
H	70.03826	72.36135	76.13567	-1038.97840	1068.88701	-2280.71789	1
C	69.79203	75.83861	78.78009	188.88100	241.05089	-727.87821	1
O	70.71783	76.38927	79.31230	531.88613	-49.19943	427.07960	1
N	68.63663	76.36392	78.34733	708.14833	26.93417	-179.73509	1
H	67.90605	75.69423	78.01974	-2232.69501	-1179.09831	1023.94816	1
C	68.26291	77.75944	78.58954	-738.32412	259.92548	104.42456	1
H	67.23684	77.82767	78.15591	2028.89123	1260.02983	1311.33324	1

C	68.24868	77.97095	80.09214	-258.65678	-345.52513	661.28459	1
H	69.26041	77.70614	80.47961	-1826.06090	-1801.46097	-918.64554	1
C	67.94654	79.40778	80.47326	-541.23400	531.92970	-218.41428	1
H	67.86950	79.53652	81.58819	1311.73218	-2219.18200	882.19023	1
H	68.74692	80.11220	80.10903	-1048.71298	1223.58118	-2196.97971	1
H	66.97358	79.75679	80.02903	419.06414	1903.92898	1903.44670	1
C	67.24123	77.04765	80.75658	-423.86431	152.66566	-648.55262	1
H	67.24913	77.15154	81.87578	105.68386	-2342.50080	1387.51238	1
H	66.19358	77.26328	80.40911	-1695.63603	-1484.73621	-1531.02793	1
H	67.45134	75.96916	80.51531	-1853.57121	-487.39031	1936.59343	1
C	69.30558	78.76399	77.78342	539.71428	-510.58232	267.58633	1
O	69.17704	79.95977	77.88150	-15.93758	-278.34085	-624.49402	1
N	70.14331	78.09582	76.99898	-676.81886	12.05292	276.17960	1
H	70.13488	77.08175	77.12220	-1694.44250	-373.84189	-2100.66098	1
C	71.29839	78.77607	76.49872	412.33078	-664.70360	108.35451	1
H	70.96017	79.78183	76.16013	1765.77476	1294.44366	1621.76594	1
C	72.32747	78.85397	77.70192	-179.66635	-721.95667	-264.73915	1
H	71.90448	79.42972	78.55535	-607.34922	2587.87032	-598.10018	1
H	72.53824	77.83662	78.10301	-359.69332	-1479.13954	-2259.74260	1
C	73.61923	79.53179	77.13474	-240.01076	616.02425	431.85322	1
H	73.97541	78.99632	76.22517	-1359.17131	782.54425	2227.99181	1
H	73.40758	80.57486	76.80892	-820.36667	85.81767	-2596.78715	1
C	74.73417	79.50567	78.25109	-513.07015	236.18570	-551.87280	1
H	74.39606	80.03599	79.17088	1816.11944	-1183.11722	-1650.94321	1
H	74.95859	78.46175	78.56983	-1800.04429	-1361.53473	1526.34643	1
C	75.99222	80.18514	77.64614	726.08972	-253.19732	-179.64713	1
H	76.39923	79.69319	76.73787	1224.27271	-456.51798	-2390.90304	1
H	75.88574	81.27464	77.46536	1206.40339	-1150.93514	-2154.90344	1
N	77.09987	80.05299	78.71480	329.70796	-633.68968	-155.67358	1
H	78.00498	80.56236	78.49214	1348.31305	1938.76845	1359.00643	1
H	76.85981	80.41255	79.74528	2096.92035	1446.54551	-966.48890	1
H	77.48396	79.07331	78.87061	-1197.07217	-2303.53719	827.28733	1
C	71.97794	77.81717	75.36611	-378.01186	-556.17301	-413.96481	1
O	72.07162	76.61282	75.49692	-409.44433	-307.55053	-453.30833	1
N	72.47629	78.59104	74.41512	-429.02477	524.70884	274.08502	1
H	72.33905	79.60134	74.44118	1706.34263	-1425.96036	1574.38926	1
C	73.32298	77.99833	73.44437	-45.25864	-626.22952	478.93267	1
H	73.58663	76.98395	73.82628	1988.63164	570.90773	1772.87287	1
C	72.47009	77.96973	72.10820	551.77128	-20.49526	-564.54996	1
H	71.48014	77.49869	72.29968	1434.61535	-1753.10253	1513.99471	1
H	72.24625	79.00402	71.76361	-714.67738	1819.09770	1898.36494	1
C	73.25964	77.17453	71.01862	446.99263	-555.77305	338.97221	1
H	74.19656	77.70685	70.72632	1953.68466	1892.25957	-161.67766	1
H	73.57540	76.17754	71.40002	-403.03785	2688.07032	-188.43591	1
C	72.26899	77.02098	69.79397	567.86262	335.74681	434.04421	1
H	71.33150	76.50887	70.11122	-1080.02275	1568.26081	-1948.78900	1
H	71.95325	78.02009	69.41516	-919.54108	2111.34692	1456.13463	1
C	72.97342	76.21254	68.68095	195.81897	173.33862	-745.11536	1
H	73.82642	76.73249	68.20094	1405.67949	-2301.21089	-390.08113	1
H	73.26989	75.18507	68.97234	-940.37328	-1118.32446	2299.72133	1
N	71.92700	76.00657	67.53312	319.26422	656.36007	-42.02424	1
H	72.27383	75.47159	66.68572	1961.03633	619.91968	1787.09422	1
H	71.04863	75.46225	67.77624	854.14343	-2156.80752	1429.08711	1
H	71.52669	76.87905	67.08030	1237.02905	-1378.50857	-1998.28500	1
C	74.57947	79.04963	73.21376	532.53319	-268.57236	517.55729	1
O	74.37986	80.23625	73.11790	266.16714	-290.73804	-558.88002	1
N	75.69182	78.33853	73.11935	-226.25352	640.49678	-270.32839	1
H	75.56975	77.32959	73.20669	2007.69063	-1035.27229	1523.50231	1
C	76.92784	78.82641	72.62873	-540.76542	-573.33303	49.50000	1
H	77.47862	77.90322	72.34319	-2374.20950	-1036.76023	843.75774	1
C	76.62672	79.78798	71.38848	-77.09082	666.87422	-415.84136	1
H	76.02811	80.66906	71.75978	-985.81834	617.19825	2463.91927	1

O	75.86366	79.20198	70.31271	293.29899	-391.65602	-477.80926	1
H	75.05260	79.74887	70.35040	2528.72714	-681.76262	-751.27122	1
C	78.07483	80.27151	71.01364	252.47363	-605.14473	440.05134	1
H	78.05036	81.03972	70.20593	-2052.65063	1456.49051	-1043.51830	1
H	78.60170	80.72392	71.88735	-2152.98800	-1661.97574	-161.70652	1
H	78.70427	79.42796	70.64654	-473.12726	-1584.27339	2165.61725	1
C	77.69032	79.65570	73.82519	360.36307	488.46713	505.10056	1
O	77.27606	80.71072	74.24696	-200.08817	-528.03552	-385.82850	1
N	78.84024	79.04141	74.09931	-597.54095	-226.44775	355.20479	1
H	78.98490	78.12934	73.66795	2179.23655	-1300.70981	-991.33835	1
C	79.41762	79.31870	75.39124	-687.68921	297.39807	-249.45179	1
H	78.62029	79.12834	76.14182	-349.16256	-2467.37713	-1101.72831	1
C	80.71347	78.41331	75.56137	672.47345	361.54328	-201.62880	1
H	81.42070	78.61656	74.72403	1541.41502	582.18425	2169.97026	1
H	81.23005	78.70431	76.50616	-2534.67717	60.25153	997.72295	1
C	80.35652	76.91911	75.60337	574.26774	-389.43119	-377.02542	1
C	79.86741	76.35031	76.81713	688.82679	-287.33580	-257.96133	1
H	79.70888	76.98021	77.71494	331.14182	1470.38163	2269.80013	1
C	80.55719	76.11424	74.44190	-383.52382	-675.04808	144.24917	1
H	80.93211	76.56126	73.50316	4.09847	1517.59662	2262.86397	1
C	79.61510	74.97800	76.84412	-134.76730	428.45285	-649.50303	1
H	79.24957	74.52664	77.78316	-903.23433	1600.49020	2011.53387	1
C	80.28951	74.74557	74.52270	-335.53281	-615.00040	-364.39058	1
H	80.45971	74.12544	73.62517	1596.45398	-1369.33862	1732.02659	1
C	79.81738	74.12816	75.71766	585.48547	-340.24194	-406.23938	1
O	79.54788	72.73714	75.80643	-419.15649	-173.04408	-511.94141	1
H	79.81807	72.45721	74.89997	-1071.84350	1559.71507	-1960.13012	1
C	79.97273	80.84028	75.40371	291.41878	-183.13091	710.72246	1
O	80.74406	81.26850	74.57685	379.87695	523.47396	-222.23622	1
N	79.58818	81.42482	76.56024	564.30591	373.09380	277.24371	1
H	78.87747	80.92800	77.10261	-120.59931	-2436.07824	-1214.35098	1
C	79.54733	82.86245	76.47426	404.07514	-299.37480	608.84009	1
H	80.45052	83.11253	75.87193	-1071.07882	-841.69025	2359.66467	1
C	78.19628	83.21332	75.73257	-683.12807	68.71770	-390.13096	1
H	78.03077	82.52107	74.86246	2068.88918	1596.58590	770.89603	1
H	77.31576	83.06242	76.39665	1379.86429	2342.49779	-179.85638	1
C	78.30604	84.70534	75.28943	-176.39967	537.49466	550.97268	1
H	78.51559	85.35866	76.16596	-396.28051	1942.22168	1869.33314	1
H	79.16233	84.85230	74.59401	-94.44700	586.00678	-2659.20016	1
C	76.92621	85.10583	74.59220	-291.69381	-718.18079	-150.72929	1
H	76.69343	84.58953	73.62612	-2303.38776	1089.76242	964.62032	1
H	76.02026	85.04131	75.24762	884.16729	2568.80533	-207.74283	1
N	77.17094	86.46214	74.32356	-594.49522	-252.81478	342.29276	1
H	78.02515	86.85005	74.69387	-1735.17426	2084.05705	-263.71605	1
C	76.39740	87.22608	73.66403	595.23177	-271.52487	442.22352	1
N	75.21312	86.67776	73.16167	585.53813	-424.79750	-105.81303	1
H	74.85564	85.72666	73.24346	959.88797	1327.56823	-2177.11905	1
H	74.51449	87.18881	72.62108	1578.48647	16.27378	-2220.76233	1
N	76.92816	88.52098	73.58220	-452.17763	-390.33541	-421.51853	1
H	77.79300	88.91309	73.95185	470.26107	1974.25629	-1817.92087	1
H	76.46222	89.28747	73.09530	1856.67307	-1573.97321	-1224.35416	1
C	79.57194	83.46068	77.99595	-531.47271	-581.45780	-55.06206	1
O	80.54618	84.05073	78.40155	-243.00837	192.73507	609.52485	1
N	78.41720	83.24060	78.62241	343.49288	-644.25607	38.09164	1
H	77.71291	82.67092	78.15536	-1691.16043	-1535.97525	-1484.73099	1
C	78.40137	83.40571	80.04152	542.66492	350.14337	454.42563	1
H	79.31458	84.00656	80.26813	1209.57979	-2396.95920	463.86744	1
C	77.02097	84.10292	80.41607	-737.14309	145.93010	242.71942	1
H	76.16925	83.44054	80.14949	-1050.18303	159.36419	-2509.06125	1
C	77.02393	84.41689	81.95003	-706.77696	-252.88343	-245.16230	1
H	76.09043	84.94123	82.26311	-1624.64039	-1665.49085	1417.86935	1
H	77.09954	83.48171	82.55434	2566.10521	598.51109	693.22305	1

```
H    77.88291  85.06926  82.23406    -1972.69758      107.10018    -1876.34312          1
C    76.92910  85.45642  79.63184      412.85148      672.58832      -27.69222          1
H    75.98006  85.99972  79.85057     1447.02277     1762.76533    -1490.78944          1
H    77.77262  86.13722  79.89412     1320.77379     -145.20455     2378.68491          1
H    76.97354  85.28857  78.52955    -1512.10848     1470.52031     1724.75147          1
C    78.43523  81.88498  80.63045     -607.11113      499.56689       73.73769          1
O    77.38640  81.31967  80.98318      357.97126      460.81534      356.68785          1
N    79.65695  81.43054  80.57702      536.55407     -368.62270      332.76306          1
H    80.36255  82.07385  80.21071      524.38866     2297.69985     1367.20941          1
C    80.03699  80.18857  81.14889       46.50277     -774.29190     -147.97904          1
H    81.14791  80.23606  81.13080     1676.51875     1883.29066     1032.55549          1
C    79.43613  80.07162  82.64398     -559.41456      -34.19198     -556.30433          1
H    78.32387  80.19816  82.60988      -64.66664      395.18769     2695.05373          1
H    79.62082  79.05433  83.06492    -2703.44278      -60.40056      333.79418          1
C    80.06998  81.13626  83.49530     -519.76435      377.31045      459.42366          1
H    79.97901  82.10848  82.95930    -1746.74323     1667.50644    -1261.73691          1
C    79.35898  81.26058  84.83738      570.95554       16.46261     -545.27700          1
H    79.81736  82.06076  85.47419     2391.58273       28.05277    -1305.07271          1
H    78.27545  81.51617  84.71103      175.75588      917.63458     2559.43867          1
H    79.40958  80.30535  85.42061    -1063.97851    -2155.34650     1283.00462          1
C    81.54611  80.84465  83.73560      193.76014        2.62946     -765.53141          1
H    82.01768  81.61611  84.39691      507.39394     -846.55087     2539.60089          1
H    81.69378  79.84986  84.22924     1337.57841     1818.19054     1526.01994          1
H    82.12960  80.82985  82.78000     1100.88635    -1172.91350    -2199.08909          1
C    79.48525  79.01035  80.20565     -549.21115      475.64486     -309.38218          1
O    78.96240  78.01454  80.63131      556.06106     -390.37755      -78.24250          1
O    79.75394  79.14023  78.81466     -572.27940     -310.19032     -209.75862          1
H    80.08981  80.07988  78.81857    -1380.08193     2212.14486      790.85944          1
```

Listing 67: C00067.xmolout

```
      276
  150.0000  150.0000  150.0000     0.0000     0.0000     0.0000***********    2.7750    11100
      0.2500    300.00    0.00    0.00    0.00  -1293.6016      0.0000    300.0000
H    56.82512  72.04113  75.69816    -2043.14101    -1757.64893      399.89339          1
N    57.31295  72.25636  76.56732      426.75047      490.45733     -334.42467          1
H    57.45118  71.32655  76.98118      442.25559    -1110.30854    -2448.52907          1
C    58.54639  72.92617  76.38301      415.32972     -666.39213       83.73172          1
H    58.62160  73.04659  75.28013      879.32219     2491.00447      667.35213          1
C    58.53295  74.35071  77.13764       31.35357      477.41777      628.23362          1
H    58.53729  74.17884  78.24043     1168.38489     2240.25337    -1019.71136          1
H    59.46556  74.92153  76.91306     -360.43770     2534.90498      931.67112          1
C    57.32020  75.14809  76.73812      463.18208      512.70242     -382.34388          1
H    56.41732  74.52234  76.92028     1725.29117     1863.52247     -987.07854          1
C    57.22550  76.40944  77.58873      710.42801     -144.27427     -313.15367          1
H    56.31364  77.01061  77.33951      364.20195    -1033.13725     2494.72524          1
H    57.17780  76.16878  78.68198     2567.61904      335.80679      847.48802          1
H    58.11335  77.07562  77.43551    -2587.99029      680.92867     -512.16411          1
C    57.35760  75.53272  75.26531      268.17616      741.64379      -40.42634          1
H    56.47254  76.15792  74.97974     1283.54314    -1945.37420    -1411.27812          1
H    58.27558  76.12497  75.01633    -1258.57796     1317.15249    -2026.02085          1
H    57.35319  74.63102  74.60061    -2502.84526      909.80597      575.92391          1
C    59.63309  71.89669  76.99581      619.36578     -458.64843     -172.09273          1
O    59.35383  70.71489  77.17075      -81.40433      433.87611      522.34594          1
N    60.77256  72.52537  77.22141      175.23996     -622.20190      341.55523          1
H    60.79504  73.52647  77.03823     -876.57256     -303.22879    -2561.90213          1
C    61.99138  71.81908  77.40706     -571.88214     -534.10783     -106.15033          1
H    61.80858  70.78859  77.03159    -2713.20841      -16.25800     -248.80809          1
C    63.05658  72.66949  76.56704      166.60354      288.16399     -716.09568          1
H    62.98637  73.74380  76.89194    -2097.85574     1636.78449     -586.18044          1
O    62.84454  72.67575  75.14251     -317.80580     -363.38227      484.43082          1
H    62.66899  73.62353  74.97947     1863.74748    -1451.84923     1357.29467          1
```

C	64.42999	72.08515	77.05901	503.80731	517.99032	318.51606	1
H	65.29169	72.61912	76.59404	640.61020	-2157.10410	-1536.29104	1
H	64.53453	72.18568	78.17018	1687.88455	465.60692	2087.56452	1
H	64.52960	71.00305	76.79605	-1293.07895	-1899.88581	-1463.57452	1
C	62.34141	71.87408	79.00367	-153.07550	-733.68902	248.70981	1
O	62.78685	72.89572	79.45899	186.15459	-480.78787	-449.34387	1
N	62.09418	70.70682	79.63199	-487.53139	543.18517	42.03850	1
C	62.11025	70.64147	81.05445	-699.35871	69.34393	360.10701	1
H	62.10518	71.68797	81.43696	2.81126	-221.99807	-2715.58072	1
C	60.80200	69.77747	81.32096	-507.46439	-592.59194	122.07761	1
H	60.77507	69.33865	82.34328	-1975.08116	525.87560	-1801.71556	1
H	59.88105	70.39187	81.21073	1588.27743	-1913.22100	1113.83566	1
C	60.90294	68.70490	80.19234	-613.62442	-457.55993	-194.14490	1
H	61.62008	67.89645	80.46746	1707.54637	-721.27992	1996.92523	1
H	59.93415	68.20400	79.96591	758.23357	2100.81767	1560.55011	1
C	61.45262	69.57115	78.98207	-352.41524	704.96076	49.21465	1
H	62.22420	69.06187	78.35688	1714.76886	-2070.42778	443.35792	1
H	60.64301	70.00985	78.30667	1224.15040	1801.50881	1636.97633	1
C	63.38358	69.73585	81.58283	566.85117	537.05831	117.62814	1
O	63.64175	68.66813	81.08282	-550.26779	-406.08867	4.21666	1
N	63.96750	70.36478	82.59107	566.34832	-425.77725	180.18749	1
H	63.56169	71.25382	82.87980	644.44502	2646.80550	-52.73430	1
C	64.96926	69.74582	83.38791	612.34801	-281.48662	411.56221	1
H	65.25126	70.55323	84.09998	1667.23498	-1490.75142	1556.16753	1
C	64.30463	68.50203	84.14483	-714.45746	-175.43074	286.98927	1
H	64.08762	67.70471	83.38350	-787.09484	1763.03619	-1922.46060	1
O	63.06335	68.77873	84.81955	-429.46514	516.51932	-128.40526	1
H	62.45479	68.18193	84.34068	1981.82785	436.16847	-1818.18182	1
C	65.46892	68.02874	85.09003	630.79189	-468.94448	76.03230	1
H	65.18304	67.11748	85.66529	-1775.71861	2062.30353	131.89849	1
H	66.40509	67.78145	84.51080	-1855.14608	88.94118	1993.53760	1
H	65.73495	68.81557	85.83426	-1504.82350	-780.94249	-2132.90992	1
C	66.20028	69.29611	82.41476	288.57638	618.62814	-397.00270	1
O	66.91470	68.35043	82.67986	321.70495	-185.26452	574.37218	1
N	66.35052	70.19098	81.44536	-447.05792	-446.53431	367.76425	1
H	65.63176	70.90956	81.37301	350.31316	1975.63644	-1843.31522	1
C	67.18978	69.85678	80.34109	-511.23061	374.03917	-471.51480	1
H	67.88107	69.09359	80.76915	2234.79579	-898.70890	-1273.45190	1
C	66.22755	69.23978	79.23831	529.53781	-159.34528	563.72604	1
H	66.87015	68.90097	78.38564	654.59388	2347.94251	1217.51488	1
H	65.74166	68.32601	79.67045	-1526.55661	2103.42026	817.87394	1
O	65.16674	70.12861	78.71175	-554.84278	14.12789	399.58769	1
H	64.46627	69.91277	79.35791	1297.06236	2061.84854	1220.68812	1
C	67.94518	71.27321	79.99272	-662.15484	-412.13544	123.62687	1
O	68.87564	71.58660	80.70280	-540.01537	-317.05804	274.91456	1
N	67.38309	71.91619	78.98480	-202.76299	-690.00100	-131.49145	1
H	66.55746	71.48691	78.54764	-702.62132	-2617.37191	281.70413	1
C	67.79808	73.20807	78.57761	-491.73914	576.34187	222.73509	1
H	66.96853	73.55464	77.92276	1977.17978	-1823.58850	-434.68928	1
C	68.02807	74.11201	79.86374	-649.50797	-328.66843	306.11224	1
H	68.69125	73.58059	80.59751	2075.07803	1452.16490	-1004.45904	1
C	68.66203	75.48828	79.46682	-615.18735	-489.99689	-70.96352	1
H	68.69654	76.19008	80.33288	-1226.32661	-1724.38906	-1716.47170	1
H	69.71168	75.35458	79.11312	2381.64273	-969.84735	900.46860	1
H	68.08974	75.99082	78.65210	2122.74991	1702.23946	-141.36228	1
C	66.62137	74.40315	80.49129	-501.86074	-479.61276	-376.42482	1
H	66.70385	75.01002	81.42337	993.79261	-2246.95401	-1177.81305	1
H	65.96488	74.96420	79.78569	42.71279	-626.15391	-2651.37258	1
H	66.09225	73.45662	80.75389	-2026.39012	-1590.33709	-887.82922	1
C	69.21692	73.07153	77.75501	-672.89153	382.57394	-156.34072	1
O	70.29606	73.05319	78.30577	410.04627	221.86223	500.35950	1
N	68.96266	73.04935	76.45034	-378.11782	-443.79414	-441.10984	1

H	67.99889	73.09645	76.12249	2404.15973	-568.14894	1149.30085	1
C	70.06869	72.93781	75.56483	-544.54484	466.16019	331.29119	1
H	70.93905	72.73833	76.23681	-1409.80988	-170.17955	2325.32679	1
C	69.71073	71.79071	74.52342	-184.48284	116.78282	-758.89137	1
H	69.32886	70.88988	75.05493	1893.78641	1861.14652	611.04528	1
H	68.88921	72.12649	73.84866	-2170.79665	-974.19311	1327.50117	1
C	70.99034	71.45373	73.69760	389.51668	672.81878	-138.49152	1
H	71.29927	72.35179	73.11278	510.64577	1933.03327	1851.02484	1
H	71.84890	71.25687	74.37683	-2302.30869	-1105.72939	-948.89774	1
C	70.79963	70.22460	72.72972	-559.93427	-126.34590	542.30857	1
O	71.73349	69.84013	72.07515	-670.36003	-111.83166	76.36448	1
N	69.55557	69.69939	72.73610	-216.06729	-537.07037	446.51379	1
H	68.71525	70.00790	73.23913	-1251.96487	1074.12894	-2168.52500	1
H	69.26164	68.91705	72.14121	-2451.83023	-720.73851	944.84612	1
C	70.21947	74.28721	74.64658	546.24052	-563.88337	-85.11809	1
O	69.98088	74.18477	73.47146	71.70702	-307.40463	606.69673	1
N	70.59958	75.36677	75.34834	479.90863	-504.50129	222.86849	1
C	70.97556	76.59480	74.72774	75.51268	-600.91904	506.73700	1
H	70.58732	76.60453	73.68269	-2033.61562	1795.30103	254.89480	1
C	70.31709	77.63015	75.74966	523.79067	517.99274	284.45581	1
H	70.69474	78.66816	75.62086	1599.61665	-1127.00911	1895.98173	1
H	69.21156	77.67232	75.63575	-1881.83093	1275.82488	-1501.55016	1
C	70.72862	76.98573	77.10671	-600.05639	-404.05026	316.64485	1
H	71.79758	77.19821	77.33915	-2597.05260	496.36221	657.73249	1
H	70.13342	77.35170	77.97320	-1666.24518	1294.38985	-1723.90591	1
C	70.51667	75.44050	76.80207	493.57570	-129.95376	-602.56394	1
H	71.30944	74.77051	77.21603	-1469.08415	-637.84797	2204.22581	1
H	69.51212	75.03056	77.06851	-1038.97840	1068.88701	-2280.71789	1
C	72.59222	76.79560	74.86464	188.88100	241.05089	-727.87821	1
O	73.26102	76.21967	75.71887	531.88613	-49.19943	427.07960	1
N	72.97291	77.71976	74.01448	708.14833	26.93417	-179.73509	1
H	72.22365	78.06046	73.41581	-2232.69501	-1179.09831	1023.94816	1
C	74.32092	78.24788	73.93102	-738.32412	259.92548	104.42456	1
H	74.24504	79.09450	73.20727	2028.89123	1260.02983	1311.33324	1
C	74.76639	78.67347	75.32762	-258.65678	-345.52513	661.28459	1
H	74.63223	77.79955	76.01211	-1826.06090	-1801.46097	-918.64554	1
C	76.22503	79.07366	75.35895	-541.23400	531.92970	-218.41428	1
H	76.55168	79.36598	76.39546	1311.73218	-2219.18200	882.19023	1
H	76.91482	78.21396	75.01578	-1048.71298	1223.58118	-2196.97971	1
H	76.42841	79.95270	74.68707	419.06414	1903.92898	1903.44670	1
C	73.90210	79.79625	75.87128	-423.86431	152.66566	-648.55262	1
H	74.18981	80.07119	76.92344	105.68386	-2342.50080	1387.51238	1
H	73.99029	80.72763	75.24725	-1695.63603	-1484.73621	-1531.02793	1
H	72.81573	79.50575	75.88600	-1853.57121	-487.39031	1936.59343	1
C	75.26683	77.00906	73.33491	539.71428	-510.58232	267.58633	1
O	75.65311	77.06749	72.19777	-15.93758	-278.34085	-624.49402	1
N	75.45486	76.06252	74.23642	-676.81886	12.05292	276.17960	1
H	74.92885	76.07324	75.13892	-1694.44250	-373.84189	-2100.66098	1
C	76.42318	75.05875	73.95394	412.33078	-664.70360	108.35451	1
H	76.29592	74.33524	74.79106	1765.77476	1294.44366	1621.76594	1
C	76.02361	74.48511	72.52693	-179.66635	-721.95667	-264.73915	1
H	74.94666	74.68192	72.32430	-607.34922	2587.87032	-598.10018	1
H	76.57693	75.03035	71.72733	-359.69332	-1479.13954	-2259.74260	1
C	76.32209	72.95049	72.46770	-240.01076	616.02425	431.85322	1
H	77.38230	72.73238	72.73043	-1359.17131	782.54425	2227.99181	1
H	75.70356	72.38975	73.20385	-820.36667	85.81767	-2596.78715	1
C	76.00626	72.48817	70.98804	-513.07015	236.18570	-551.87280	1
H	74.90838	72.49254	70.79597	1816.11944	-1183.11722	-1650.94321	1
H	76.44510	73.19833	70.25020	-1800.04429	-1361.53473	1526.34643	1
C	76.60129	71.07437	70.79260	726.08972	-253.19732	-179.64713	1
H	77.70489	71.02276	70.88375	1224.27271	-456.51798	-2390.90304	1
H	76.13158	70.28316	71.41061	1206.40339	-1150.93514	-2154.90344	1

N	76.30745	70.65312	69.31448	329.70796	-633.68968	-155.67358	1
H	76.69939	69.71431	69.01457	1348.31305	1938.76845	1359.00643	1
H	75.28471	70.54370	69.05584	2096.92035	1446.54551	-966.48890	1
H	76.66608	71.28841	68.54301	-1197.07217	-2303.53719	827.28733	1
C	77.89928	75.76582	73.93454	-378.01186	-556.17301	-413.96481	1
O	78.10632	76.86133	74.42607	-409.44433	-307.55053	-453.30833	1
N	78.74984	74.96001	73.33083	-429.02477	524.70884	274.08502	1
H	78.38841	74.07521	72.98121	1706.34263	-1425.96036	1574.38926	1
C	80.14837	75.23024	73.32250	-45.25864	-626.22952	478.93267	1
H	80.54370	74.54611	72.53788	1988.63164	570.90773	1772.87287	1
C	80.29491	76.76727	72.97153	551.77128	-20.49526	-564.54996	1
H	79.44797	77.06355	72.31184	1434.61535	-1753.10253	1513.99471	1
H	80.17882	77.39371	73.88897	-714.67738	1819.09770	1898.36494	1
C	81.66455	77.06807	72.26532	446.99263	-555.77305	338.97221	1
H	82.47025	77.27212	73.00885	1953.68466	1892.25957	-161.67766	1
H	82.00811	76.19835	71.65932	-403.03785	2688.07032	-188.43591	1
C	81.43115	78.31289	71.34783	567.86262	335.74681	434.04421	1
H	80.68412	78.09782	70.55248	-1080.02275	1568.26081	-1948.78900	1
H	81.01490	79.16564	71.92778	-919.54108	2111.34692	1456.13463	1
C	82.84325	78.70480	70.71147	195.81897	173.33862	-745.11536	1
H	83.60579	78.97811	71.49348	1405.67949	-2301.21089	-390.08113	1
H	83.27836	77.91348	70.00834	-940.37328	-1118.32446	2299.72133	1
N	82.62189	79.86612	69.90225	319.26422	656.36007	-42.02424	1
H	85.09872	80.66994	69.97350	1961.03633	619.91968	1787.09422	2
H	83.22757	79.94131	69.08987	854.14343	-2156.80752	1429.08711	1
H	82.79869	80.75072	70.36968	1237.02905	-1378.50857	-1998.28500	1
C	80.72361	74.86082	74.80774	532.53319	-268.57236	517.55729	1
O	80.07884	74.27730	75.64811	266.16714	-290.73804	-558.88002	1
N	82.02117	75.18528	74.84733	-226.25352	640.49678	-270.32839	1
H	82.39451	75.65427	74.02421	2007.69063	-1035.27229	1523.50231	1
C	82.56310	75.46967	76.13844	-540.76542	-573.33303	49.50000	1
H	81.81978	75.04511	76.85259	-2374.20950	-1036.76023	843.75774	1
C	83.99242	74.76431	76.20198	-77.09082	666.87422	-415.84136	1
H	84.63585	75.15030	75.36338	-985.81834	617.19825	2463.91927	1
O	84.01037	73.33142	76.04424	293.29899	-391.65602	-477.80926	1
H	84.49602	73.23701	75.20195	2528.72714	-681.76262	-751.27122	1
C	84.51717	75.30683	77.58144	252.47363	-605.14473	440.05134	1
H	85.58385	75.03223	77.75131	-2052.65063	1456.49051	-1043.51830	1
H	84.44113	76.41734	77.64231	-2152.98800	-1661.97574	-161.70652	1
H	83.92840	74.88978	78.43107	-473.12726	-1584.27339	2165.61725	1
C	82.67649	77.10703	76.21318	360.36307	488.46713	505.10056	1
O	81.82904	77.73539	76.79823	-200.08817	-528.03552	-385.82850	1
N	83.74091	77.55681	75.55337	-597.54095	-226.44775	355.20479	1
H	84.36517	76.91560	75.06537	2179.23655	-1300.70981	-991.33835	1
C	83.94335	78.94103	75.45441	-687.68921	297.39807	-249.45179	1
H	83.08214	79.44714	75.94506	-349.16256	-2467.37713	-1101.72831	1
C	85.37211	79.27619	76.08828	672.47345	361.54328	-201.62880	1
H	86.14235	78.54983	75.74119	1541.41502	582.18425	2169.97026	1
H	85.70203	80.28945	75.76221	-2534.67717	60.25153	997.72295	1
C	85.21418	79.22846	77.61517	574.26774	-389.43119	-377.02542	1
C	84.46908	80.26287	78.25712	688.82679	-287.33580	-257.96133	1
H	84.06125	81.10865	77.67440	331.14182	1470.38163	2269.80013	1
C	85.75578	78.14951	78.37705	-383.52382	-675.04808	144.24917	1
H	86.35240	77.35859	77.88758	4.09847	1517.59662	2262.86397	1
C	84.24926	80.17362	79.63101	-134.76730	428.45285	-649.50303	1
H	83.66367	80.96857	80.12494	-903.23433	1600.49020	2011.53387	1
C	85.50009	78.09969	79.74993	-335.53281	-615.00040	-364.39058	1
H	85.90549	77.24968	80.32606	1596.45398	-1369.33862	1732.02659	1
C	84.73847	79.09699	80.42621	585.48547	-340.24194	-406.23938	1
O	84.46975	79.05585	81.81921	-419.15649	-173.04408	-511.94141	1
H	84.91903	78.20939	82.05092	-1071.84350	1559.71507	-1960.13012	1
C	84.16035	79.24959	73.82545	291.41878	-183.13091	710.72246	1

217	O	84.82116	78.44401	73.09547	379.87695	523.47396	-222.23622	1
218	N	83.67426	80.36592	73.46439	564.30591	373.09380	277.24371	1
219	H	83.21383	80.84455	74.21942	-120.59931	-2436.07824	-1214.35098	1
220	C	85.91716	80.23777	70.58827	404.07514	-299.37480	608.84009	2
221	H	85.47338	80.03499	71.59108	-1071.07882	-841.69025	2359.66467	2
222	C	87.07571	81.28282	70.69979	-683.12807	68.71770	-390.13096	2
223	H	86.78033	82.12591	71.36607	2068.88918	1596.58590	770.89603	2
224	H	87.97996	80.82227	71.16007	1379.86429	2342.49779	-179.85638	2
225	C	87.38284	81.80895	69.25599	-176.39967	537.49466	550.97268	2
226	H	87.69579	80.97323	68.58702	-396.28051	1942.22168	1869.33314	2
227	H	86.47347	82.24616	68.78739	-94.44700	586.00678	-2659.20016	2
228	C	88.53595	82.90019	69.40946	-291.69381	-718.18079	-150.72929	2
229	H	88.29034	83.76838	70.07339	-2303.38776	1089.76242	964.62032	2
230	H	89.53672	82.50590	69.72130	884.16729	2568.80533	-207.74283	2
231	N	88.65444	83.39434	68.10238	-594.49522	-252.81478	342.29276	2
232	H	88.03682	83.00299	67.40827	-1735.17426	2084.05705	-263.71605	2
233	C	89.47775	84.29458	67.74519	595.23177	-271.52487	442.22352	2
234	N	90.32678	84.82472	68.72328	585.53813	-424.79750	-105.81303	2
235	H	90.39739	84.59240	69.71311	959.88797	1327.56823	-2177.11905	2
236	H	91.02919	85.54637	68.55900	1578.48647	16.27378	-2220.76233	2
237	N	89.35169	84.58080	66.37840	-452.17763	-390.33541	-421.51853	2
238	H	88.73012	84.19557	65.66893	470.26107	1974.25629	-1817.92087	2
239	H	89.92219	85.27875	65.89965	1856.67307	-1573.97321	-1224.35416	2
240	C	86.44742	78.88946	69.93573	-531.47271	-581.45780	-55.06206	2
241	O	87.08342	78.83124	68.91782	-243.00837	192.73507	609.52485	2
242	N	86.04707	77.88222	70.74986	343.49288	-644.25607	38.09164	2
243	H	85.49501	78.10091	71.64120	-1691.16043	-1535.97525	-1484.73099	2
244	C	86.25617	76.47693	70.35573	542.66492	350.14337	454.42563	2
245	H	87.34241	76.39792	70.11862	1209.57979	-2396.95920	463.86744	2
246	C	85.79947	75.60730	71.50541	-737.14309	145.93010	242.71942	2
247	H	84.72842	75.84097	71.70368	-1050.18303	159.36419	-2509.06125	2
248	C	85.90520	74.13155	71.16228	-706.77696	-252.88343	-245.16230	2
249	H	85.50567	73.48282	71.98915	-1624.64039	-1665.49085	1417.86935	2
250	H	85.32915	73.87497	70.22703	2566.10521	598.51109	693.22305	2
251	H	86.97190	73.82399	70.98549	-1972.69758	107.10018	-1876.34312	2
252	C	86.56596	75.89430	72.78455	412.85148	672.58832	-27.69222	2
253	H	86.25447	75.21116	73.62118	1447.02277	1762.76533	-1490.78944	2
254	H	87.67354	75.76401	72.64172	1320.77379	-145.20455	2378.68491	2
255	H	86.39461	76.95344	73.13589	-1512.10848	1470.52031	1724.75147	2
256	C	85.30176	76.20101	69.03822	-607.11113	499.56689	73.73769	2
257	O	84.18160	76.66058	68.92543	357.97126	460.81534	356.68785	2
258	N	85.93462	75.35994	68.24272	536.55407	-368.62270	332.76306	2
259	H	86.84215	75.00316	68.53520	524.38866	2297.69985	1367.20941	2
260	C	85.31967	75.01554	67.01285	46.50277	-774.29190	-147.97904	2
261	H	84.59733	75.84434	66.83069	1676.51875	1883.29066	1032.55549	2
262	C	86.46515	74.83656	65.89137	-559.41456	-34.19198	-556.30433	2
263	H	87.03478	73.89949	66.09976	-64.66664	395.18769	2695.05373	2
264	H	86.00703	74.69346	64.88373	-2703.44278	-60.40056	333.79418	2
265	C	87.39606	76.02055	65.90292	-519.76435	377.31045	459.42366	2
266	H	87.74808	76.18356	66.94691	-1746.74323	1667.50644	-1261.73691	2
267	C	88.61091	75.74805	65.02481	570.95554	16.46261	-545.27700	2
268	H	89.33193	76.60574	65.03873	2391.58273	28.05277	-1305.07271	2
269	H	89.17092	74.83835	65.36342	175.75588	917.63458	2559.43867	2
270	H	88.31827	75.57813	63.95680	-1063.97851	-2155.34650	1283.00462	2
271	C	86.69506	77.29045	65.44089	193.76014	2.62946	-765.53141	2
272	H	87.39694	78.16425	65.42352	507.39394	-846.55087	2539.60089	2
273	H	86.27374	77.17986	64.40840	1337.57841	1818.19054	1526.01994	2
274	H	85.84828	77.56257	66.12335	1100.88635	-1172.91350	-2199.08909	2
275	C	84.58421	73.59202	67.22977	-549.21115	475.64486	-309.38218	2
276	O	84.83013	72.83694	68.13340	556.06106	-390.37755	-78.24250	2
277	O	83.67522	73.22445	66.20965	-572.27940	-310.19032	-209.75862	2
278	H	83.71738	74.05642	65.66027	-1380.08193	2212.14486	790.85944	2

Listing 68: C00068.xmolout

```
      276
   150.0000  150.0000  150.0000     0.0000     0.0000     0.0000**********     2.7750    11100
       0.2500    300.00   0.00   0.00   0.00  -1293.6004      0.0000    300.0000
H    60.36614  78.27556  70.64603    -2043.14101    -1757.64893     399.89339          1
N    60.50544  79.18841  71.07076      426.75047     490.45733    -334.42467          1
H    60.49184  78.96866  72.06287      442.25559   -1110.30854   -2448.52907          1
C    61.61101  79.93883  70.60383      415.32972    -666.39213      83.73172          1
H    61.65826  79.68466  69.52035      879.32219    2491.00447     667.35213          1
C    61.33517  81.51980  70.84406       31.35357     477.41777     628.23362          1
H    61.22878  81.71685  71.93664     1168.38489    2240.25337   -1019.71136          1
H    62.21575  82.11718  70.50896     -360.43770    2534.90498     931.67112          1
C    60.09234  81.92926  70.11074      463.18208     512.70242    -382.34388          1
H    59.28061  81.21346  70.37523     1725.29117    1863.52247    -987.07854          1
C    59.66322  83.33092  70.53097      710.42801    -144.27427    -313.15367          1
H    58.72358  83.64917  70.01082      364.20195   -1033.13725    2494.72524          1
H    59.47070  83.39285  71.63269     2567.61904     335.80679     847.48802          1
H    60.44936  84.09125  70.28871    -2587.99029     680.92867    -512.16411          1
C    60.29671  81.90108  68.60089      268.17616     741.64379     -40.42634          1
H    59.37999  82.24071  68.05387     1283.54314   -1945.37420   -1411.27812          1
H    61.13776  82.57099  68.28658    -1258.57796    1317.15249   -2026.02085          1
H    60.53789  80.87132  68.23293    -2502.84526     909.80597     575.92391          1
C    62.92879  79.52656  71.38078      619.36578    -458.64843    -172.09273          1
O    63.96294  80.28460  71.44491      -81.40433     433.87611     522.34594          1
N    62.89634  78.32644  71.88457      175.23996    -622.20190     341.55523          1
H    62.04554  77.77143  71.82930     -876.57256    -303.22879   -2561.90213          1
C    63.95344  78.07927  72.82363     -571.88214    -534.10783    -106.15033          1
H    63.48755  78.30574  73.80165    -2713.20841     -16.25800    -248.80809          1
C    64.29733  76.55171  72.68262      166.60354     288.16399    -716.09568          1
H    64.58199  76.34142  71.61507    -2097.85574    1636.78449    -586.18044          1
O    63.21970  75.62764  72.98430     -317.80580    -363.38227     484.43082          1
H    63.11990  75.16104  72.13534     1863.74748   -1451.84923    1357.29467          1
C    65.57728  76.46565  73.59671      503.80731     517.99032     318.51606          1
H    66.05045  75.45797  73.54124      640.61020   -2157.10410   -1536.29104          1
H    66.34474  77.21755  73.28888     1687.88455     465.60692    2087.56452          1
H    65.33098  76.65765  74.66664    -1293.07895   -1899.88581   -1463.57452          1
C    65.22465  79.11571  72.39881     -153.07550    -733.68902     248.70981          1
O    66.05359  78.69803  71.56901      186.15459    -480.78787    -449.34387          1
N    65.59203  79.84244  73.51506     -487.53139     543.18517      42.03850          1
C    66.56748  80.85732  73.41381     -699.35871      69.34393     360.10701          1
H    66.60823  81.10163  72.32605        2.81126    -221.99807   -2715.58072          1
C    66.00027  81.99100  74.38392     -507.46439    -592.59194     122.07761          1
H    66.78889  82.69685  74.72836    -1975.08116     525.87560   -1801.71556          1
H    65.21204  82.59934  73.88650     1588.27743   -1913.22100    1113.83566          1
C    65.40939  81.12217  75.53087     -613.62442    -457.55993    -194.14490          1
H    66.21408  80.74557  76.20153     1707.54637    -721.27992    1996.92523          1
H    64.68109  81.66386  76.17441      758.23357    2100.81767    1560.55011          1
C    64.74661  79.93770  74.68489     -352.41524     704.96076      49.21465          1
H    64.73995  78.94398  75.19532     1714.76886   -2070.42778     443.35792          1
H    63.72162  80.16326  74.29996     1224.15040    1801.50881    1636.97633          1
C    68.03567  80.34815  73.99521      566.85117     537.05831     117.62814          1
O    68.57207  80.93858  74.89904     -550.26779    -406.08867       4.21666          1
N    68.47506  79.32033  73.28851      566.34832    -425.77725     180.18749          1
H    67.89766  78.97398  72.51698      644.44502    2646.80550     -52.73430          1
C    69.75523  78.78507  73.57698      612.34801    -281.48662     411.56221          1
H    70.14018  79.33026  74.47079     1667.23498   -1490.75142    1556.16753          1
C    69.51570  77.21442  73.81817     -714.45746    -175.43074     286.98927          1
H    68.94295  76.80946  72.93987     -787.09484    1763.03619   -1922.46060          1
O    68.73900  76.89184  74.98564     -429.46514     516.51932    -128.40526          1
```

58	H	67.94952	76.48524	74.57795	1981.82785	436.16847	-1818.18182	1
59	C	70.97057	76.62209	73.80277	630.79189	-468.94448	76.03230	1
60	H	70.96655	75.51933	73.96409	-1775.71861	2062.30353	131.89849	1
61	H	71.49327	76.81302	72.83632	-1855.14608	88.94118	1993.53760	1
62	H	71.59648	77.07101	74.60543	-1504.82350	-780.94249	-2132.90992	1
63	C	70.65698	78.97222	72.22782	288.57638	618.62814	-397.00270	1
64	O	70.17327	78.97911	71.11874	321.70495	-185.26452	574.37218	1
65	N	71.95116	79.03309	72.55063	-447.05792	-446.53431	367.76425	1
66	H	72.22584	78.96200	73.52892	350.31316	1975.63644	-1843.31522	1
67	C	72.85417	78.80533	71.45373	-511.23061	374.03917	-471.51480	1
68	H	72.25980	78.08667	70.84281	2234.79579	-898.70890	-1273.45190	1
69	C	72.97987	80.20344	70.68273	529.53781	-159.34528	563.72604	1
70	H	73.41275	79.97965	69.67043	654.59388	2347.94251	1217.51488	1
71	H	71.93074	80.56244	70.51363	-1526.55661	2103.42026	817.87394	1
72	O	73.80803	81.21749	71.31758	-554.84278	14.12789	399.58769	1
73	H	73.12789	81.65320	71.86427	1297.06236	2061.84854	1220.68812	1
74	C	74.23037	78.17728	72.01672	-662.15484	-412.13544	123.62687	1
75	O	74.21723	77.24561	72.78212	-540.01537	-317.05804	274.91456	1
76	N	75.29264	78.71351	71.37731	-202.76299	-690.00100	-131.49145	1
77	H	75.07883	79.57214	70.85824	-702.62132	-2617.37191	281.70413	1
78	C	76.66328	78.46726	71.83979	-491.73914	576.34187	222.73509	1
79	H	76.71687	77.35948	71.93431	1977.17978	-1823.58850	-434.68928	1
80	C	77.58288	79.02957	70.77853	-649.50797	-328.66843	306.11224	1
81	H	77.33688	80.10874	70.65408	2075.07803	1452.16490	-1004.45904	1
82	C	79.05143	78.92623	71.14922	-615.18735	-489.99689	-70.96352	1
83	H	79.71391	79.33419	70.33706	-1226.32661	-1724.38906	-1716.47170	1
84	H	79.28533	79.50205	72.08866	2381.64273	-969.84735	900.46860	1
85	H	79.35765	77.85878	71.32434	2122.74991	1702.23946	-141.36228	1
86	C	77.35991	78.35324	69.43541	-501.86074	-479.61276	-376.42482	1
87	H	77.98109	78.82124	68.62384	993.79261	-2246.95401	-1177.81305	1
88	H	77.62918	77.26258	69.47430	42.71279	-626.15391	-2651.37258	1
89	H	76.28430	78.41679	69.11515	-2026.39012	-1590.33709	-887.82922	1
90	C	76.89960	79.18487	73.32238	-672.89153	382.57394	-156.34072	1
91	O	77.97124	79.07051	73.86729	410.04627	221.86223	500.35950	1
92	N	75.82297	79.81905	73.73990	-378.11782	-443.79414	-441.10984	1
93	H	75.02854	79.79369	73.09915	2404.15973	-568.14894	1149.30085	1
94	C	75.81428	80.55580	74.96890	-544.54484	466.16019	331.29119	1
95	H	76.69564	81.23106	74.89331	-1409.80988	-170.17955	2325.32679	1
96	C	74.41863	81.30288	74.97771	-184.48284	116.78282	-758.89137	1
97	H	74.38165	82.10399	74.20491	1893.78641	1861.14652	611.04528	1
98	H	73.60714	80.59599	74.69613	-2170.79665	-974.19311	1327.50117	1
99	C	74.17795	81.88207	76.40642	389.51668	672.81878	-138.49152	1
100	H	74.57216	81.17536	77.17409	510.64577	1933.03327	1851.02484	1
101	H	74.78382	82.80174	76.56294	-2302.30869	-1105.72939	-948.89774	1
102	C	72.65857	82.17342	76.70332	-559.93427	-126.34590	542.30857	1
103	O	72.35541	82.69873	77.74334	-670.36003	-111.83166	76.36448	1
104	N	71.80616	81.75527	75.74225	-216.06729	-537.07037	446.51379	1
105	H	71.99208	81.30993	74.83590	-1251.96487	1074.12894	-2168.52500	1
106	H	70.78430	81.83507	75.81120	-2451.83023	-720.73851	944.84612	1
107	C	76.00230	79.57336	76.25434	546.24052	-563.88337	-85.11809	1
108	O	76.57340	80.06964	77.19160	71.70702	-307.40463	606.69673	1
109	N	75.31841	78.41977	76.21651	479.90863	-504.50129	222.86849	1
110	C	75.67088	77.38620	77.14694	75.51268	-600.91904	506.73700	1
111	H	76.09056	77.94792	78.01409	-2033.61562	1795.30103	254.89480	1
112	C	74.30650	76.63625	77.43003	523.79067	517.99274	284.45581	1
113	H	74.44441	75.53812	77.56495	1599.61665	-1127.00911	1895.98173	1
114	H	73.83653	77.00576	78.36907	-1881.83093	1275.82488	-1501.55016	1
115	C	73.41737	76.97317	76.18864	-600.05639	-404.05026	316.64485	1
116	H	73.57050	76.23146	75.36977	-2597.05260	496.36221	657.73249	1
117	H	72.32924	76.95910	76.42592	-1666.24518	1294.38985	-1723.90591	1
118	C	73.93712	78.40032	75.74144	493.57570	-129.95376	-602.56394	1
119	H	73.97212	78.55896	74.63728	-1469.08415	-637.84797	2204.22581	1

120	H	73.42546	79.26206	76.23191	-1038.97840	1068.88701	-2280.71789	1
121	C	76.77020	76.33209	76.55033	188.88100	241.05089	-727.87821	1
122	O	77.52669	75.77870	77.31114	531.88613	-49.19943	427.07960	1
123	N	76.71938	76.16952	75.19363	708.14833	26.93417	-179.73509	1
124	H	75.87624	76.68283	74.92290	-2232.69501	-1179.09831	1023.94816	1
125	C	77.09213	74.83766	74.64782	-738.32412	259.92548	104.42456	1
126	H	76.60349	74.10964	75.33583	2028.89123	1260.02983	1311.33324	1
127	C	76.60474	74.74840	73.22268	-258.65678	-345.52513	661.28459	1
128	H	76.83339	75.71919	72.73003	-1826.06090	-1801.46097	-918.64554	1
129	C	77.30595	73.64772	72.44490	-541.23400	531.92970	-218.41428	1
130	H	76.98396	73.62624	71.36773	1311.73218	-2219.18200	882.19023	1
131	H	78.42437	73.77996	72.45905	-1048.71298	1223.58118	-2196.97971	1
132	H	77.09103	72.63356	72.88065	419.06414	1903.92898	1903.44670	1
133	C	75.10402	74.52813	73.14633	-423.86431	152.66566	-648.55262	1
134	H	74.73257	74.55594	72.08488	105.68386	-2342.50080	1387.51238	1
135	H	74.80976	73.53260	73.58301	-1695.63603	-1484.73621	-1531.02793	1
136	H	74.53781	75.31913	73.72249	-1853.57121	-487.39031	1936.59343	1
137	C	78.72886	74.70548	74.66923	539.71428	-510.58232	267.58633	1
138	O	79.24066	73.58994	74.58617	-15.93758	-278.34085	-624.49402	1
139	N	79.31615	75.87583	74.68132	-676.81886	12.05292	276.17960	1
140	H	78.68011	76.66266	74.79297	-1694.44250	-373.84189	-2100.66098	1
141	C	80.72119	76.01679	74.45181	412.33078	-664.70360	108.35451	1
142	H	80.91788	75.28131	73.63859	1765.77476	1294.44366	1621.76594	1
143	C	80.94046	77.53148	74.06804	-179.66635	-721.95667	-264.73915	1
144	H	80.30022	77.81681	73.20410	-607.34922	2587.87032	-598.10018	1
145	H	80.61722	78.19380	74.90388	-359.69332	-1479.13954	-2259.74260	1
146	C	82.45775	77.74379	73.74508	-240.01076	616.02425	431.85322	1
147	H	83.07943	77.49624	74.63706	-1359.17131	782.54425	2227.99181	1
148	H	82.80024	77.06670	72.93077	-820.36667	85.81767	-2596.78715	1
149	C	82.63741	79.26128	73.33938	-513.07015	236.18570	-551.87280	1
150	H	82.15515	79.47266	72.35708	1816.11944	-1183.11722	-1650.94321	1
151	H	82.13192	79.92867	74.07484	-1800.04429	-1361.53473	1526.34643	1
152	C	84.15525	79.55500	73.29650	726.08972	-253.19732	-179.64713	1
153	H	84.67037	79.45192	74.27260	1224.27271	-456.51798	-2390.90304	1
154	H	84.71325	79.00261	72.51442	1206.40339	-1150.93514	-2154.90344	1
155	N	84.32657	81.06562	72.92306	329.70796	-633.68968	-155.67358	1
156	H	85.32583	81.42010	72.89215	1348.31305	1938.76845	1359.00643	1
157	H	83.96581	81.37396	71.97362	2096.92035	1446.54551	-966.48890	1
158	H	83.87443	81.77447	73.57110	-1197.07217	-2303.53719	827.28733	1
159	C	81.53007	75.61132	75.81098	-378.01186	-556.17301	-413.96481	1
160	O	82.17888	76.41237	76.43591	-409.44433	-307.55053	-453.30833	1
161	N	81.43556	74.28815	75.98723	-429.02477	524.70884	274.08502	1
162	H	80.75926	73.74088	75.39265	1706.34263	-1425.96036	1574.38926	1
163	C	81.76079	73.83695	77.30663	-45.25864	-626.22952	478.93267	1
164	H	82.36234	74.66207	77.76018	1988.63164	570.90773	1772.87287	1
165	C	80.38372	73.59071	78.05787	551.77128	-20.49526	-564.54996	1
166	H	79.67287	74.43362	77.89348	1434.61535	-1753.10253	1513.99471	1
167	H	79.87661	72.69072	77.64332	-714.67738	1819.09770	1898.36494	1
168	C	80.71165	73.40218	79.57410	446.99263	-555.77305	338.97221	1
169	H	81.66142	72.81673	79.69126	1953.68466	1892.25957	-161.67766	1
170	H	80.89287	74.38302	80.06917	-403.03785	2688.07032	-188.43591	1
171	C	79.51430	72.62811	80.25910	567.86262	335.74681	434.04421	1
172	H	78.58602	73.24443	80.25096	-1080.02275	1568.26081	-1948.78900	1
173	H	79.26648	71.69929	79.69532	-919.54108	2111.34692	1456.13463	1
174	C	79.94880	72.28735	81.70391	195.81897	173.33862	-745.11536	1
175	H	80.78025	71.55810	81.78077	1405.67949	-2301.21089	-390.08113	1
176	H	80.16045	73.16766	82.34279	-940.37328	-1118.32446	2299.72133	1
177	N	78.74191	71.57319	82.40009	319.26422	656.36007	-42.02424	1
178	H	78.91472	71.25252	83.39640	1961.03633	619.91968	1787.09422	1
179	H	77.85026	72.13765	82.51454	854.14343	-2156.80752	1429.08711	1
180	H	78.38188	70.68065	81.95224	1237.02905	-1378.50857	-1998.28500	1
181	C	82.52553	72.39820	77.25188	532.53319	-268.57236	517.55729	1

O	82.81701	71.84842	78.31183	266.16714	-290.73804	-558.88002	1
N	82.69129	71.94992	76.02379	-226.25352	640.49678	-270.32839	1
H	82.38079	72.60622	75.31014	2007.69063	-1035.27229	1523.50231	1
C	83.51325	70.84602	75.67498	-540.76542	-573.33303	49.50000	1
H	83.62532	70.94239	74.57236	-2374.20950	-1036.76023	843.75774	1
C	84.91602	71.01952	76.42507	-77.09082	666.87422	-415.84136	1
H	84.72724	71.04640	77.53541	-985.81834	617.19825	2463.91927	1
O	85.62471	72.23166	76.10049	293.29899	-391.65602	-477.80926	1
H	85.60549	72.69362	76.96155	2528.72714	-681.76262	-751.27122	1
C	85.67752	69.69095	76.07209	252.47363	-605.14473	440.05134	1
H	86.68437	69.66077	76.55035	-2052.65063	1456.49051	-1043.51830	1
H	85.12089	68.78553	76.41509	-2152.98800	-1661.97574	-161.70652	1
H	85.83112	69.58920	74.97242	-473.12726	-1584.27339	2165.61725	1
C	82.78607	69.43787	76.06456	360.36307	488.46713	505.10056	1
O	83.08501	68.44853	75.43529	-200.08817	-528.03552	-385.82850	1
N	81.94738	69.52590	77.09443	-597.54095	-226.44775	355.20479	1
H	81.85560	70.41643	77.61033	2179.23655	-1300.70981	-991.33835	1
C	81.33607	68.30159	77.48880	-687.68921	297.39807	-249.45179	1
H	82.13332	67.54211	77.32165	-349.16256	-2467.37713	-1101.72831	1
C	80.78207	68.42862	78.97894	672.47345	361.54328	-201.62880	1
H	80.08561	69.29557	79.05091	1541.41502	582.18425	2169.97026	1
H	80.19287	67.51557	79.22684	-2534.67717	60.25153	997.72295	1
C	81.94009	68.59501	79.97393	574.26774	-389.43119	-377.02542	1
C	82.64814	67.45106	80.44987	688.82679	-287.33580	-257.96133	1
H	82.38990	66.43766	80.09239	331.14182	1470.38163	2269.80013	1
C	82.28975	69.90189	80.42681	-383.52382	-675.04808	144.24917	1
H	81.75721	70.78842	80.03674	4.09847	1517.59662	2262.86397	1
C	83.66787	67.64174	81.38199	-134.76730	428.45285	-649.50303	1
H	84.21216	66.75759	81.75695	-903.23433	1600.49020	2011.53387	1
C	83.31786	70.04184	81.36195	-335.53281	-615.00040	-364.39058	1
H	83.57641	71.05875	81.70550	1596.45398	-1369.33862	1732.02659	1
C	84.03940	68.92570	81.87677	585.48547	-340.24194	-406.23938	1
O	85.08338	69.05403	82.82968	-419.15649	-173.04408	-511.94141	1
H	85.07223	70.03184	82.95800	-1071.84350	1559.71507	-1960.13012	1
C	80.01580	67.97113	76.57026	291.41878	-183.13091	710.72246	1
O	79.81891	66.81822	76.25273	379.87695	523.47396	-222.23622	1
N	79.24694	69.03516	76.38817	564.30591	373.09380	277.24371	1
H	79.64644	69.91188	76.72225	-120.59931	-2436.07824	-1214.35098	1
C	78.10144	69.01030	75.55183	404.07514	-299.37480	608.84009	1
H	77.96532	70.07161	75.24593	-1071.07882	-841.69025	2359.66467	1
C	78.40457	67.99141	74.37852	-683.12807	68.71770	-390.13096	1
H	79.43974	68.13785	73.99324	2068.88918	1596.58590	770.89603	1
H	78.37123	66.94012	74.77235	1379.86429	2342.49779	-179.85638	1
C	77.34961	68.18059	73.24198	-176.39967	537.49466	550.97268	1
H	76.31391	68.04832	73.62670	-396.28051	1942.22168	1869.33314	1
H	77.38837	69.20663	72.81392	-94.44700	586.00678	-2659.20016	1
C	77.71369	67.07262	72.14963	-291.69381	-718.18079	-150.72929	1
H	78.72733	67.16849	71.68349	-2303.38776	1089.76242	964.62032	1
H	77.60161	66.01341	72.49633	884.16729	2568.80533	-207.74283	1
N	76.76389	67.28352	71.14166	-594.49522	-252.81478	342.29276	1
H	76.04666	67.96664	71.32877	-1735.17426	2084.05705	-263.71605	1
C	76.74818	66.66896	70.02929	595.23177	-271.52487	442.22352	1
N	77.75047	65.72027	69.79574	585.53813	-424.79750	-105.81303	1
H	78.51495	65.42308	70.40080	959.88797	1327.56823	-2177.11905	1
H	77.83806	65.16163	68.94635	1578.48647	16.27378	-2220.76233	1
N	75.67599	67.09166	69.23083	-452.17763	-390.33541	-421.51853	1
H	74.94378	67.77989	69.39895	470.26107	1974.25629	-1817.92087	1
H	75.49327	66.71343	68.30042	1856.67307	-1573.97321	-1224.35416	1
C	76.80644	68.39926	76.36206	-531.47271	-581.45780	-55.06206	1
O	76.64586	67.21117	76.53447	-243.00837	192.73507	609.52485	1
N	75.99672	69.38677	76.71549	343.49288	-644.25607	38.09164	1
H	76.26789	70.35741	76.56041	-1691.16043	-1535.97525	-1484.73099	1

```
C    74.86207   69.03357   77.51428      542.66492      350.14337      454.42563          1
H    74.59284   68.02399   77.12689     1209.57979    -2396.95920      463.86744          1
C    75.32909   68.92464   79.02949     -737.14309      145.93010      242.71942          1
H    75.80636   69.86964   79.36700    -1050.18303      159.36419    -2509.06125          1
C    74.08741   68.57496   79.91753     -706.77696     -252.88343     -245.16230          1
H    74.36914   68.43553   80.98797    -1624.64039    -1665.49085     1417.86935          1
H    73.31775   69.39100   79.87440     2566.10521      598.51109      693.22305          1
H    73.59220   67.63462   79.57823    -1972.69758      107.10018    -1876.34312          1
C    76.33120   67.72160   79.13091      412.85148      672.58832      -27.69222          1
H    76.57934   67.47435   80.18997     1447.02277     1762.76533    -1490.78944          1
H    75.90983   66.79975   78.65967     1320.77379     -145.20455     2378.68491          1
H    77.29119   67.94126   78.60260    -1512.10848     1470.52031     1724.75147          1
C    73.71956   70.17126   77.25293     -607.11113      499.56689       73.73769          1
O    72.97265   70.54101   78.13564      357.97126      460.81534      356.68785          1
N    73.67703   70.42173   75.94853      536.55407     -368.62270      332.76306          1
H    74.39834   69.94144   75.40942      524.38866     2297.69985     1367.20941          1
C    73.06999   71.59172   75.41743       46.50277     -774.29190     -147.97904          1
H    73.10179   71.42356   74.31910     1676.51875     1883.29066     1032.55549          1
C    71.57158   71.75824   75.99607     -559.41456      -34.19198     -556.30433          1
H    71.59180   71.62750   77.11506      -64.66664      395.18769     2695.05373          1
H    71.16868   72.78174   75.80434    -2703.44278      -60.40056      333.79418          1
C    70.70317   70.71319   75.35344     -519.76435      377.31045      459.42366          1
H    71.21818   69.72995   75.44628    -1746.74323     1667.50644    -1261.73691          1
C    69.35920   70.62611   76.06680      570.95554       16.46261     -545.27700          1
H    68.70371   69.83555   75.61882     2391.58273       28.05277    -1305.07271          1
H    69.48328   70.38044   77.15311      175.75588      917.63458     2559.43867          1
H    68.79856   71.59442   76.00777    -1063.97851    -2155.34650     1283.00462          1
C    70.47525   71.01637   73.87799      193.76014        2.62946     -765.53141          1
H    69.80723   70.25534   73.39916      507.39394     -846.55087     2539.60089          1
H    69.99418   72.01782   73.73388     1337.57841     1818.19054     1526.01994          1
H    71.43383   71.02387   73.29931     1100.88635    -1172.91350    -2199.08909          1
C    73.95331   72.87015   75.80735     -549.21115      475.64486     -309.38218          1
O    75.01455   72.83227   76.37093      556.06106     -390.37755      -78.24250          1
O    73.44466   74.11142   75.32129     -572.27940     -310.19032     -209.75862          1
H    72.62021   73.76730   74.88476    -1380.08193     2212.14486      790.85944          1
```

Listing 69: C00069.xmolout

```
      276
  150.0000  150.0000  150.0000     0.0000     0.0000     0.0000**********     1.0580     4232
      0.2500    300.00    0.00    0.00    0.00   -1293.5989       0.0000    300.0000
H    73.71273   64.84398   68.89238    -2043.14101    -1757.64893      399.89339          1
N    74.70404   64.68363   68.71632      426.75047      490.45733     -334.42467          1
H    74.69908   64.18461   67.81947      442.25559    -1110.30854    -2448.52907          1
C    75.46415   65.87047   68.62716      415.32972     -666.39213       83.73172          1
H    74.91973   66.66471   69.18367      879.32219     2491.00447      667.35213          1
C    76.93896   65.58563   69.22041       31.35357      477.41777      628.23362          1
H    77.36024   64.66912   68.74308     1168.38489     2240.25337    -1019.71136          1
H    77.63519   66.41994   68.96175     -360.43770     2534.90498      931.67112          1
C    76.83969   65.41320   70.71226      463.18208      512.70242     -382.34388          1
H    75.96959   64.75286   70.92929     1725.29117     1863.52247     -987.07854          1
C    78.09699   64.74882   71.25954      710.42801     -144.27427     -313.15367          1
H    78.03729   64.59883   72.36836      364.20195    -1033.13725     2494.72524          1
H    78.26820   63.74251   70.79724     2567.61904      335.80679      847.48802          1
H    79.01032   65.36571   71.05752    -2587.99029      680.92867     -512.16411          1
C    76.62911   66.75435   71.40270      268.17616      741.64379      -40.42634          1
H    76.55154   66.64009   72.51462     1283.54314    -1945.37420    -1411.27812          1
H    77.47710   67.45849   71.20014    -1258.57796     1317.15249    -2026.02085          1
H    75.69161   67.25932   71.05495    -2502.84526      909.80597      575.92391          1
C    75.57772   66.13423   67.02650      619.36578     -458.64843     -172.09273          1
O    75.33472   65.28014   66.19862      -81.40433      433.87611      522.34594          1
N    76.08116   67.35354   66.85261      175.23996     -622.20190      341.55523          1
```

H	76.20829	67.94147	67.67683	-876.57256	-303.22879	-2561.90213	1
C	75.99066	67.96611	65.56349	-571.88214	-534.10783	-106.15033	1
H	75.97819	67.05868	64.91378	-2713.20841	-16.25800	-248.80809	1
C	74.60288	68.78487	65.51840	166.60354	288.16399	-716.09568	1
H	74.82342	69.85257	65.22865	-2097.85574	1636.78449	-586.18044	1
O	73.85823	68.89187	66.73712	-317.80580	-363.38227	484.43082	1
H	73.90086	69.85714	66.90345	1863.74748	-1451.84923	1357.29467	1
C	73.90215	68.04606	64.31952	503.80731	517.99032	318.51606	1
H	73.61946	67.00519	64.60263	640.61020	-2157.10410	-1536.29104	1
H	72.96907	68.56693	64.00365	1687.88455	465.60692	2087.56452	1
H	74.56121	67.97243	63.42325	-1293.07895	-1899.88581	-1463.57452	1
C	77.32073	68.87380	65.43304	-153.07550	-733.68902	248.70981	1
O	78.31573	68.52232	66.01975	186.15459	-480.78787	-449.34387	1
N	77.21669	69.89953	64.56988	-487.53139	543.18517	42.03850	1
C	78.29485	70.84801	64.56360	-699.35871	69.34393	360.10701	1
H	79.20526	70.24898	64.80474	2.81126	-221.99807	-2715.58072	1
C	78.24767	71.45567	63.09817	-507.46439	-592.59194	122.07761	1
H	78.62190	72.50319	63.05432	-1975.08116	525.87560	-1801.71556	1
H	78.87956	70.86741	62.39592	1588.27743	-1913.22100	1113.83566	1
C	76.73475	71.32821	62.73951	-613.62442	-457.55993	-194.14490	1
H	76.13869	72.13080	63.22857	1707.54637	-721.27992	1996.92523	1
H	76.52510	71.39326	61.64813	758.23357	2100.81767	1560.55011	1
C	76.40548	69.90225	63.36102	-352.41524	704.96076	49.21465	1
H	75.33351	69.75500	63.62098	1714.76886	-2070.42778	443.35792	1
H	76.75580	69.03391	62.75487	1224.15040	1801.50881	1636.97633	1
C	78.05327	72.02618	65.67838	566.85117	537.05831	117.62814	1
O	79.02520	72.69542	66.03293	-550.26779	-406.08867	4.21666	1
N	76.78408	72.16939	65.98752	566.34832	-425.77725	180.18749	1
H	76.23254	71.46498	65.50136	644.44502	2646.80550	-52.73430	1
C	76.29100	72.85550	67.13094	612.34801	-281.48662	411.56221	1
H	75.22534	72.54031	67.18299	1667.23498	-1490.75142	1556.16753	1
C	77.12939	72.29716	68.37839	-714.45746	-175.43074	286.98927	1
H	78.22883	72.36051	68.14383	-787.09484	1763.03619	-1922.46060	1
O	76.87988	70.91141	68.68509	-429.46514	516.51932	-128.40526	1
H	77.73559	70.50709	68.43884	1981.82785	436.16847	-1818.18182	1
C	76.77457	73.32898	69.50664	630.79189	-468.94448	76.03230	1
H	77.24697	73.04293	70.47554	-1775.71861	2062.30353	131.89849	1
H	77.13188	74.37695	69.25937	-1855.14608	88.94118	1993.53760	1
H	75.67342	73.38014	69.67905	-1504.82350	-780.94249	-2132.90992	1
C	76.40745	74.46712	66.92410	288.57638	618.62814	-397.00270	1
O	75.36487	75.10997	66.98289	321.70495	-185.26452	574.37218	1
N	77.63471	74.86031	66.73706	-447.05792	-446.53431	367.76425	1
H	78.40611	74.14296	66.67196	350.31316	1975.63644	-1843.31522	1
C	78.00539	76.22876	66.67813	-511.23061	374.03917	-471.51480	1
H	79.05436	76.13843	66.31500	2234.79579	-898.70890	-1273.45190	1
C	77.05587	76.98961	65.63621	529.53781	-159.34528	563.72604	1
H	77.63158	77.83142	65.16138	654.59388	2347.94251	1217.51488	1
H	76.85219	76.24633	64.81940	-1526.55661	2103.42026	817.87394	1
O	75.84251	77.53801	66.15657	-554.84278	14.12789	399.58769	1
H	75.42216	76.66724	66.52353	1297.06236	2061.84854	1220.68812	1
C	77.95152	76.86556	68.17369	-662.15484	-412.13544	123.62687	1
O	77.90801	76.19450	69.18866	-540.01537	-317.05804	274.91456	1
N	78.11074	78.17584	68.07500	-202.76299	-690.00100	-131.49145	1
H	78.16084	78.60365	67.15301	-702.62132	-2617.37191	281.70413	1
C	78.02729	78.96473	69.25920	-491.73914	576.34187	222.73509	1
H	77.33110	78.42218	69.93743	1977.17978	-1823.58850	-434.68928	1
C	79.49677	79.02812	69.86436	-649.50797	-328.66843	306.11224	1
H	80.21005	79.45570	69.12687	2075.07803	1452.16490	-1004.45904	1
C	79.44259	79.89452	71.16755	-615.18735	-489.99689	-70.96352	1
H	80.44350	79.97277	71.65358	-1226.32661	-1724.38906	-1716.47170	1
H	79.09479	80.93274	70.95171	2381.64273	-969.84735	900.46860	1
H	78.74058	79.46091	71.91747	2122.74991	1702.23946	-141.36228	1

86	C	79.92168	77.57438	70.27122	-501.86074	-479.61276	-376.42482	1
87	H	80.87630	77.56482	70.84897	993.79261	-2246.95401	-1177.81305	1
88	H	79.13860	77.08317	70.89823	42.71279	-626.15391	-2651.37258	1
89	H	80.06551	76.92621	69.37286	-2026.39012	-1590.33709	-887.82922	1
90	C	77.54921	80.46035	68.78815	-672.89153	382.57394	-156.34072	1
91	O	78.08948	81.03091	67.86998	410.04627	221.86223	500.35950	1
92	N	76.60496	80.89724	69.61026	-378.11782	-443.79414	-441.10984	1
93	H	76.30709	80.30083	70.38156	2404.15973	-568.14894	1149.30085	1
94	C	75.73768	81.97387	69.20343	-544.54484	466.16019	331.29119	1
95	H	76.37164	82.88541	69.27555	-1409.80988	-170.17955	2325.32679	1
96	C	75.26404	81.74887	67.70979	-184.48284	116.78282	-758.89137	1
97	H	76.14134	81.59992	67.03393	1893.78641	1861.14652	611.04528	1
98	H	74.64962	80.82495	67.61732	-2170.79665	-974.19311	1327.50117	1
99	C	74.43697	83.00527	67.29080	389.51668	672.81878	-138.49152	1
100	H	73.61257	83.18221	68.02070	510.64577	1933.03327	1851.02484	1
101	H	75.06062	83.92330	67.36820	-2302.30869	-1105.72939	-948.89774	1
102	C	73.83878	82.89234	65.83902	-559.93427	-126.34590	542.30857	1
103	O	73.16107	83.78968	65.40825	-670.36003	-111.83166	76.36448	1
104	N	74.15795	81.75106	65.19002	-216.06729	-537.07037	446.51379	1
105	H	74.72829	80.95237	65.49198	-1251.96487	1074.12894	-2168.52500	1
106	H	73.84338	81.53025	64.23888	-2451.83023	-720.73851	944.84612	1
107	C	74.46391	81.99057	70.20640	546.24052	-563.88337	-85.11809	1
108	O	73.84645	83.02146	70.28828	71.70702	-307.40463	606.69673	1
109	N	74.14075	80.78514	70.69176	479.90863	-504.50129	222.86849	1
110	C	73.50609	80.65824	71.97755	75.51268	-600.91904	506.73700	1
111	H	72.66257	81.38689	71.97521	-2033.61562	1795.30103	254.89480	1
112	C	73.05874	79.13861	72.02254	523.79067	517.99274	284.45581	1
113	H	73.17092	78.69512	73.03784	1599.61665	-1127.00911	1895.98173	1
114	H	71.98298	79.03179	71.75813	-1881.83093	1275.82488	-1501.55016	1
115	C	73.96864	78.45076	70.95642	-600.05639	-404.05026	316.64485	1
116	H	74.97296	78.21637	71.37444	-2597.05260	496.36221	657.73249	1
117	H	73.55413	77.49576	70.56251	-1666.24518	1294.38985	-1723.90591	1
118	C	74.04816	79.59591	69.85630	493.57570	-129.95376	-602.56394	1
119	H	74.92680	79.54396	69.17226	-1469.08415	-637.84797	2204.22581	1
120	H	73.11991	79.72106	69.24930	-1038.97840	1068.88701	-2280.71789	1
121	C	74.58316	81.00099	73.14855	188.88100	241.05089	-727.87821	1
122	O	75.77712	81.02177	72.98991	531.88613	-49.19943	427.07960	1
123	N	73.88321	81.23697	74.27361	708.14833	26.93417	-179.73509	1
124	H	72.88309	81.06005	74.17466	-2232.69501	-1179.09831	1023.94816	1
125	C	74.54579	81.22001	75.58257	-738.32412	259.92548	104.42456	1
126	H	75.41545	81.90766	75.46326	2028.89123	1260.02983	1311.33324	1
127	C	73.52703	81.63902	76.61973	-258.65678	-345.52513	661.28459	1
128	H	72.63919	80.97422	76.49697	-1826.06090	-1801.46097	-918.64554	1
129	C	74.04822	81.44839	78.03289	-541.23400	531.92970	-218.41428	1
130	H	73.25551	81.67196	78.79957	1311.73218	-2219.18200	882.19023	1
131	H	74.38355	80.38539	78.19770	-1048.71298	1223.58118	-2196.97971	1
132	H	74.92647	82.11623	78.25001	419.06414	1903.92898	1903.44670	1
133	C	73.05502	83.06897	76.42780	-423.86431	152.66566	-648.55262	1
134	H	72.22586	83.32862	77.14252	105.68386	-2342.50080	1387.51238	1
135	H	73.88659	83.80700	76.59542	-1695.63603	-1484.73621	-1531.02793	1
136	H	72.66596	83.23694	75.38594	-1853.57121	-487.39031	1936.59343	1
137	C	75.00675	79.64986	75.86750	539.71428	-510.58232	267.58633	1
138	O	74.19060	78.78372	76.16384	-15.93758	-278.34085	-624.49402	1
139	N	76.30684	79.55697	75.75045	-676.81886	12.05292	276.17960	1
140	H	76.79305	80.41156	75.49110	-1694.44250	-373.84189	-2100.66098	1
141	C	76.96695	78.29703	75.87508	412.33078	-664.70360	108.35451	1
142	H	76.24330	77.58571	75.41651	1765.77476	1294.44366	1621.76594	1
143	C	78.34310	78.45286	75.11860	-179.66635	-721.95667	-264.73915	1
144	H	78.18089	78.76588	74.06218	-607.34922	2587.87032	-598.10018	1
145	H	78.95226	79.26194	75.58122	-359.69332	-1479.13954	-2259.74260	1
146	C	79.09658	77.08341	75.19344	-240.01076	616.02425	431.85322	1
147	H	79.25831	76.79210	76.25853	-1359.17131	782.54425	2227.99181	1

H	78.49726	76.26597	74.73347	-820.36667	85.81767	-2596.78715	1
C	80.47401	77.27243	74.43954	-513.07015	236.18570	-551.87280	1
H	80.31195	77.53533	73.36841	1816.11944	-1183.11722	-1650.94321	1
H	81.05191	78.11939	74.87569	-1800.04429	-1361.53473	1526.34643	1
C	81.25731	75.94652	74.57750	726.08972	-253.19732	-179.64713	1
H	81.51157	75.67024	75.62049	1224.27271	-456.51798	-2390.90304	1
H	80.79741	75.08169	74.05917	1206.40339	-1150.93514	-2154.90344	1
N	82.64274	76.14498	73.87589	329.70796	-633.68968	-155.67358	1
H	83.29053	75.30542	73.89962	1348.31305	1938.76845	1359.00643	1
H	82.63519	76.37399	72.83938	2096.92035	1446.54551	-966.48890	1
H	83.27435	76.90433	74.26498	-1197.07217	-2303.53719	827.28733	1
C	77.16216	77.98843	77.47495	-378.01186	-556.17301	-413.96481	1
O	78.25096	77.90100	77.98601	-409.44433	-307.55053	-453.30833	1
N	75.96063	77.81861	78.01708	-429.02477	524.70884	274.08502	1
H	75.10422	77.95785	77.43197	1706.34263	-1425.96036	1574.38926	1
C	75.89909	77.64755	79.42559	-45.25864	-626.22952	478.93267	1
H	76.91835	77.88527	79.81265	1988.63164	570.90773	1772.87287	1
C	74.73441	78.58695	79.95407	551.77128	-20.49526	-564.54996	1
H	74.87597	79.63490	79.60367	1434.61535	-1753.10253	1513.99471	1
H	73.76040	78.25823	79.52435	-714.67738	1819.09770	1898.36494	1
C	74.69260	78.53094	81.51533	446.99263	-555.77305	338.97221	1
H	74.86276	77.48622	81.87935	1953.68466	1892.25957	-161.67766	1
H	75.50307	79.14822	81.96425	-403.03785	2688.07032	-188.43591	1
C	73.26319	79.03979	81.96256	567.86262	335.74681	434.04421	1
H	73.11031	80.10774	81.68321	-1080.02275	1568.26081	-1948.78900	1
H	72.46444	78.47259	81.43345	-919.54108	2111.34692	1456.13463	1
C	73.15498	78.83599	83.49160	195.81897	173.33862	-745.11536	1
H	73.15619	77.77680	83.81954	1405.67949	-2301.21089	-390.08113	1
H	73.88734	79.41602	84.08784	-940.37328	-1118.32446	2299.72133	1
N	71.76031	79.38994	83.93778	319.26422	656.36007	-42.02424	1
H	71.55907	79.32560	84.97718	1961.03633	619.91968	1787.09422	1
H	71.56525	80.41660	83.74994	854.14343	-2156.80752	1429.08711	1
H	70.89868	78.92314	83.53173	1237.02905	-1378.50857	-1998.28500	1
C	75.40231	76.12761	79.80346	532.53319	-268.57236	517.55729	1
O	75.53649	75.73518	80.96252	266.16714	-290.73804	-558.88002	1
N	74.79965	75.54221	78.79266	-226.25352	640.49678	-270.32839	1
H	74.85143	76.10199	77.94439	2007.69063	-1035.27229	1523.50231	1
C	74.06451	74.33240	78.81684	-540.76542	-573.33303	49.50000	1
H	73.88574	74.11360	77.74107	-2374.20950	-1036.76023	843.75774	1
C	74.93493	73.19923	79.53659	-77.09082	666.87422	-415.84136	1
H	75.27177	73.58827	80.54026	-985.81834	617.19825	2463.91927	1
O	76.12357	72.80477	78.82955	293.29899	-391.65602	-477.80926	1
H	76.80737	72.95283	79.52037	2528.72714	-681.76262	-751.27122	1
C	73.87487	72.06218	79.76439	252.47363	-605.14473	440.05134	1
H	74.29865	71.23274	80.37841	-2052.65063	1456.49051	-1043.51830	1
H	72.96421	72.44141	80.29643	-2152.98800	-1661.97574	-161.70652	1
H	73.53532	71.62300	78.79793	-473.12726	-1584.27339	2165.61725	1
C	72.64031	74.53488	79.60370	360.36307	488.46713	505.10056	1
O	71.67693	73.95262	79.16470	-200.08817	-528.03552	-385.82850	1
N	72.75715	75.31350	80.66563	-597.54095	-226.44775	355.20479	1
H	73.69806	75.65710	80.92821	2179.23655	-1300.70981	-991.33835	1
C	71.69794	75.44427	81.60620	-687.68921	297.39807	-249.45179	1
H	72.03214	76.29422	82.24291	-349.16256	-2467.37713	-1101.72831	1
C	70.32352	75.74515	80.83374	672.47345	361.54328	-201.62880	1
H	70.05731	74.86909	80.18948	1541.41502	582.18425	2169.97026	1
H	69.48988	75.88181	81.55999	-2534.67717	60.25153	997.72295	1
C	70.49597	77.00478	79.97461	574.26774	-389.43119	-377.02542	1
C	70.37358	78.30614	80.54792	688.82679	-287.33580	-257.96133	1
H	70.19103	78.43153	81.62993	331.14182	1470.38163	2269.80013	1
C	70.78391	76.85621	78.58378	-383.52382	-675.04808	144.24917	1
H	70.91832	75.84979	78.14364	4.09847	1517.59662	2262.86397	1
C	70.47820	79.41750	79.71047	-134.76730	428.45285	-649.50303	1

H	70.36902	80.42393	80.15055	-903.23433	1600.49020	2011.53387	1
C	70.88106	77.99740	77.78538	-335.53281	-615.00040	-364.39058	1
H	71.09756	77.86781	76.71026	1596.45398	-1369.33862	1732.02659	1
C	70.70734	79.31192	78.30831	585.48547	-340.24194	-406.23938	1
O	70.76828	80.47392	77.48947	-419.15649	-173.04408	-511.94141	1
H	70.87087	80.04010	76.60694	-1071.84350	1559.71507	-1960.13012	1
C	71.63726	74.05843	82.43864	291.41878	-183.13091	710.72246	1
O	72.52400	73.20150	82.34213	379.87695	523.47396	-222.23622	1
N	70.58408	74.07290	83.21954	564.30591	373.09380	277.24371	1
H	70.01161	74.91343	83.17068	-120.59931	-2436.07824	-1214.35098	1
C	70.16239	72.97721	84.02131	404.07514	-299.37480	608.84009	1
H	69.12154	73.24445	84.31389	-1071.07882	-841.69025	2359.66467	1
C	70.26096	71.65678	83.14234	-683.12807	68.71770	-390.13096	1
H	69.64698	71.74744	82.21794	2068.88918	1596.58590	770.89603	1
H	71.31013	71.50324	82.78830	1379.86429	2342.49779	-179.85638	1
C	69.79109	70.45959	84.02270	-176.39967	537.49466	550.97268	1
H	70.36428	70.42217	84.97596	-396.28051	1942.22168	1869.33314	1
H	68.72437	70.57073	84.31893	-94.44700	586.00678	-2659.20016	1
C	70.02027	69.13038	83.16625	-291.69381	-718.18079	-150.72929	1
H	69.40149	69.03632	82.23802	-2303.38776	1089.76242	964.62032	1
H	71.08384	68.90963	82.89373	884.16729	2568.80533	-207.74283	1
N	69.59823	68.14541	84.07157	-594.49522	-252.81478	342.29276	1
H	69.31329	68.46063	84.98595	-1735.17426	2084.05705	-263.71605	1
C	69.55623	66.89962	83.81778	595.23177	-271.52487	442.22352	1
N	69.95912	66.48341	82.54394	585.53813	-424.79750	-105.81303	1
H	70.29363	67.04070	81.75935	959.88797	1327.56823	-2177.11905	1
H	69.96830	65.51634	82.21952	1578.48647	16.27378	-2220.76233	1
N	69.09263	66.18138	84.92947	-452.17763	-390.33541	-421.51853	1
H	68.79591	66.49624	85.85157	470.26107	1974.25629	-1817.92087	1
H	68.97878	65.16747	84.93091	1856.67307	-1573.97321	-1224.35416	1
C	71.13050	72.82105	85.32651	-531.47271	-581.45780	-55.06206	1
O	70.64332	72.76139	86.42151	-243.00837	192.73507	609.52485	1
N	72.41342	72.64914	84.97672	343.49288	-644.25607	38.09164	1
H	72.66329	72.69658	83.94880	-1691.16043	-1535.97525	-1484.73099	1
C	73.44568	73.03321	85.95987	542.66492	350.14337	454.42563	1
H	72.87220	73.61454	86.71889	1209.57979	-2396.95920	463.86744	1
C	74.00874	71.73946	86.50973	-737.14309	145.93010	242.71942	1
H	74.28052	71.08970	85.64827	-1050.18303	159.36419	-2509.06125	1
C	75.24975	71.96254	87.35326	-706.77696	-252.88343	-245.16230	1
H	75.65103	70.99538	87.76467	-1624.64039	-1665.49085	1417.86935	1
H	76.07750	72.43978	86.75897	2566.10521	598.51109	693.22305	1
H	75.03951	72.63567	88.22974	-1972.69758	107.10018	-1876.34312	1
C	72.95790	70.99575	87.31631	412.85148	672.58832	-27.69222	1
H	73.27837	69.94572	87.55995	1447.02277	1762.76533	-1490.78944	1
H	72.73933	71.51573	88.28892	1320.77379	-145.20455	2378.68491	1
H	71.98226	70.93825	86.75385	-1512.10848	1470.52031	1724.75147	1
C	74.50054	73.96528	85.08846	-607.11113	499.56689	73.73769	1
O	74.33570	75.16049	85.04399	357.97126	460.81534	356.68785	1
N	75.43657	73.24289	84.49563	536.55407	-368.62270	332.76306	1
H	75.45166	72.22849	84.58607	524.38866	2297.69985	1367.20941	1
C	76.39133	73.91163	83.68745	46.50277	-774.29190	-147.97904	1
H	75.89353	74.86006	83.37606	1676.51875	1883.29066	1032.55549	1
C	77.74372	74.15996	84.54398	-559.41456	-34.19198	-556.30433	1
H	78.18561	73.16946	84.80582	-64.66664	395.18769	2695.05373	1
H	78.50723	74.67483	83.91381	-2703.44278	-60.40056	333.79418	1
C	77.46450	74.94608	85.79374	-519.76435	377.31045	459.42366	1
H	76.58818	74.49082	86.30820	-1746.74323	1667.50644	-1261.73691	1
C	78.66186	74.88520	86.73509	570.95554	16.46261	-545.27700	1
H	78.46855	75.44101	87.68833	2391.58273	28.05277	-1305.07271	1
H	78.91762	73.83009	87.01207	175.75588	917.63458	2559.43867	1
H	79.57544	75.33537	86.26861	-1063.97851	-2155.34650	1283.00462	1
C	77.13142	76.39748	85.47556	193.76014	2.62946	-765.53141	1

```
H    76.98204  77.00060  86.40814      507.39394     -846.55087     2539.60089          1
H    77.94708  76.89099  84.88668     1337.57841     1818.19054     1526.01994          1
H    76.18522  76.47643  84.87574     1100.88635    -1172.91350    -2199.08909          1
C    76.80098  72.88529  82.51796     -549.21115      475.64486     -309.38218          1
O    76.64066  71.69636  82.59518      556.06106     -390.37755      -78.24250          1
O    77.54464  73.44693  81.44383     -572.27940     -310.19032     -209.75862          1
H    77.27641  74.40084  81.56152    -1380.08193     2212.14486      790.85944          1
```

Listing 70: C00070.xmolout

```
      276
  150.0000  150.0000  150.0000    0.0000    0.0000    0.0000***********   2.7750    11100
       0.2500    300.00    0.00    0.00    0.00  -1293.5878      0.0000    300.0000
H    67.86796  69.34608  69.51791    -2043.14101    -1757.64893      399.89339          1
N    68.05051  70.34421  69.41266      426.75047      490.45733     -334.42467          1
H    68.06388  70.71096  70.37677      442.25559    -1110.30854    -2448.52907          1
C    69.19287  70.63909  68.63437      415.32972     -666.39213       83.73172          1
H    68.90938  70.29183  67.61768      879.32219     2491.00447      667.35213          1
C    69.45866  72.22865  68.69826       31.35357      477.41777      628.23362          1
H    69.67214  72.53721  69.75077     1168.38489     2240.25337    -1019.71136          1
H    70.36575  72.50521  68.10886     -360.43770     2534.90498      931.67112          1
C    68.23119  72.93254  68.18244      463.18208      512.70242     -382.34388          1
H    67.33601  72.35781  68.51282     1725.29117     1863.52247     -987.07854          1
C    68.12932  74.33917  68.75808      710.42801     -144.27427     -313.15367          1
H    67.21207  74.86927  68.39252      364.20195    -1033.13725     2494.72524          1
H    68.08261  74.32641  69.87744     2567.61904      335.80679      847.48802          1
H    69.01201  74.96683  68.47175    -2587.99029      680.92867     -512.16411          1
C    68.24235  72.99521  66.66084      268.17616      741.64379      -40.42634          1
H    67.31259  73.47395  66.25857     1283.54314    -1945.37420    -1411.27812          1
H    69.11227  73.59059  66.28077    -1258.57796     1317.15249    -2026.02085          1
H    68.31549  71.97465  66.20442    -2502.84526      909.80597      575.92391          1
C    70.48417  69.82917  69.16417      619.36578     -458.64843     -172.09273          1
O    71.42176  69.70408  68.42147      -81.40433      433.87611      522.34594          1
N    70.31339  69.31273  70.40253      175.23996     -622.20190      341.55523          1
H    69.55378  69.75638  70.95734     -876.57256     -303.22879    -2561.90213          1
C    71.51371  68.98747  71.15706     -571.88214     -534.10783     -106.15033          1
H    71.82025  69.96152  71.58705    -2713.20841      -16.25800     -248.80809          1
C    71.03152  67.96610  72.26632      166.60354      288.16399     -716.09568          1
H    70.62698  67.04886  71.75776    -2097.85574     1636.78449     -586.18044          1
O    69.95940  68.41479  73.14172     -317.80580     -363.38227      484.43082          1
H    69.26645  67.76323  72.93216     1863.74748    -1451.84923     1357.29467          1
C    72.39224  67.61518  72.97068      503.80731      517.99032      318.51606          1
H    72.25214  66.84339  73.76277      640.61020    -2157.10410    -1536.29104          1
H    73.13755  67.21248  72.24244     1687.88455      465.60692     2087.56452          1
H    72.84910  68.50737  73.45752    -1293.07895    -1899.88581    -1463.57452          1
C    72.67475  68.31215  70.25498     -153.07550     -733.68902      248.70981          1
O    72.57068  67.15399  69.94153      186.15459     -480.78787     -449.34387          1
N    73.74486  69.13223  70.12714     -487.53139      543.18517       42.03850          1
C    74.84869  68.71143  69.33836     -699.35871       69.34393      360.10701          1
H    74.59121  67.69900  68.94379        2.81126     -221.99807    -2715.58072          1
C    74.87179  69.88766  68.26505     -507.46439     -592.59194      122.07761          1
H    75.81710  69.92266  67.67829    -1975.08116      525.87560    -1801.71556          1
H    74.03564  69.78001  67.53765     1588.27743    -1913.22100     1113.83566          1
C    74.65749  71.13206  69.17988     -613.62442     -457.55993     -194.14490          1
H    75.61985  71.46191  69.65990     1707.54637     -721.27992     1996.92523          1
H    74.26724  72.01827  68.62933      758.23357     2100.81767     1560.55011          1
C    73.62861  70.58098  70.25637     -352.41524      704.96076       49.21465          1
H    73.88173  70.84650  71.31029     1714.76886    -2070.42778      443.35792          1
H    72.56224  70.83711  70.04534     1224.15040     1801.50881     1636.97633          1
C    76.27009  68.77788  70.17921      566.85117      537.05831      117.62814          1
O    77.30768  68.82612  69.56364     -550.26779     -406.08867        4.21666          1
N    76.07265  68.79284  71.49659      566.34832     -425.77725      180.18749          1
```

H	75.08533	68.70082	71.73432	644.44502	2646.80550	-52.73430	1
C	77.09249	68.36020	72.40053	612.34801	-281.48662	411.56221	1
H	76.52071	68.21355	73.34352	1667.23498	-1490.75142	1556.16753	1
C	77.67997	66.97445	71.86332	-714.45746	-175.43074	286.98927	1
H	78.14908	67.15284	70.85660	-787.09484	1763.03619	-1922.46060	1
O	76.68242	65.94851	71.68336	-429.46514	516.51932	-128.40526	1
H	76.72679	65.81422	70.71724	1981.82785	436.16847	-1818.18182	1
C	78.81098	66.60109	72.88864	630.79189	-468.94448	76.03230	1
H	79.30588	65.64035	72.61273	-1775.71861	2062.30353	131.89849	1
H	79.61729	67.38835	72.93845	-1855.14608	88.94118	1993.53760	1
H	78.40273	66.47618	73.91866	-1504.82350	-780.94249	-2132.90992	1
C	78.17034	69.56045	72.54498	288.57638	618.62814	-397.00270	1
O	79.35090	69.33910	72.72664	321.70495	-185.26452	574.37218	1
N	77.54113	70.74467	72.64830	-447.05792	-446.53431	367.76425	1
H	76.56345	70.71854	72.35689	350.31316	1975.63644	-1843.31522	1
C	78.35827	71.89366	72.39767	-511.23061	374.03917	-471.51480	1
H	79.37344	71.57205	72.73055	2234.79579	-898.70890	-1273.45190	1
C	78.30239	72.14610	70.81947	529.53781	-159.34528	563.72604	1
H	79.17769	72.79585	70.55987	654.59388	2347.94251	1217.51488	1
H	78.45471	71.15522	70.31090	-1526.55661	2103.42026	817.87394	1
O	77.05466	72.70639	70.29477	-554.84278	14.12789	399.58769	1
H	77.20466	73.64313	70.52054	1297.06236	2061.84854	1220.68812	1
C	77.78560	73.19168	73.18306	-662.15484	-412.13544	123.62687	1
O	78.19270	74.29202	72.89367	-540.01537	-317.05804	274.91456	1
N	76.88086	72.86539	74.11988	-202.76299	-690.00100	-131.49145	1
H	76.76449	71.85601	74.21288	-702.62132	-2617.37191	281.70413	1
C	76.46251	73.79164	75.17769	-491.73914	576.34187	222.73509	1
H	75.90261	73.13205	75.88313	1977.17978	-1823.58850	-434.68928	1
C	77.68627	74.41077	75.82483	-649.50797	-328.66843	306.11224	1
H	78.28106	74.90077	75.01728	2075.07803	1452.16490	-1004.45904	1
C	77.33212	75.46371	76.85742	-615.18735	-489.99689	-70.96352	1
H	78.25057	75.96217	77.27238	-1226.32661	-1724.38906	-1716.47170	1
H	76.67802	76.27964	76.41420	2381.64273	-969.84735	900.46860	1
H	76.76811	75.02041	77.72394	2122.74991	1702.23946	-141.36228	1
C	78.56157	73.35380	76.47748	-501.86074	-479.61276	-376.42482	1
H	79.48892	73.79972	76.92977	993.79261	-2246.95401	-1177.81305	1
H	78.01483	72.81841	77.30136	42.71279	-626.15391	-2651.37258	1
H	78.88845	72.57462	75.73587	-2026.39012	-1590.33709	-887.82922	1
C	75.45897	74.89842	74.47220	-672.89153	382.57394	-156.34072	1
O	75.35480	76.02586	74.90834	410.04627	221.86223	500.35950	1
N	74.75699	74.34493	73.49999	-378.11782	-443.79414	-441.10984	1
H	75.00875	73.39753	73.23130	2404.15973	-568.14894	1149.30085	1
C	73.71741	75.13536	72.90143	-544.54484	466.16019	331.29119	1
H	73.23891	75.56496	73.81043	-1409.80988	-170.17955	2325.32679	1
C	74.48236	76.25465	72.08012	-184.48284	116.78282	-758.89137	1
H	75.22907	76.75267	72.74487	1893.78641	1861.14652	611.04528	1
H	75.06718	75.80277	71.24649	-2170.79665	-974.19311	1327.50117	1
C	73.43779	77.27702	71.53708	389.51668	672.81878	-138.49152	1
H	72.74677	76.76112	70.83063	510.64577	1933.03327	1851.02484	1
H	72.77950	77.63290	72.35828	-2302.30869	-1105.72939	-948.89774	1
C	74.09179	78.53323	70.84657	-559.93427	-126.34590	542.30857	1
O	73.37763	79.38733	70.38783	-670.36003	-111.83166	76.36448	1
N	75.44190	78.54052	70.86205	-216.06729	-537.07037	446.51379	1
H	76.10099	77.84522	71.23171	-1251.96487	1074.12894	-2168.52500	1
H	76.01421	79.29018	70.45864	-2451.83023	-720.73851	944.84612	1
C	72.74190	74.17603	72.02457	546.24052	-563.88337	-85.11809	1
O	72.58069	74.42570	70.85935	71.70702	-307.40463	606.69673	1
N	72.13980	73.23972	72.78217	479.90863	-504.50129	222.86849	1
C	71.47689	72.10425	72.23882	75.51268	-600.91904	506.73700	1
H	71.93233	71.89426	71.24362	-2033.61562	1795.30103	254.89480	1
C	71.74509	71.03499	73.39185	523.79067	517.99274	284.45581	1
H	71.05024	70.15656	73.34263	1599.61665	-1127.00911	1895.98173	1

114	H	72.78295	70.63626	73.34052	-1881.83093	1275.82488	-1501.55016	1
115	C	71.54058	71.88631	74.68167	-600.05639	-404.05026	316.64485	1
116	H	70.46673	71.93804	74.96891	-2597.05260	496.36221	657.73249	1
117	H	72.07612	71.47745	75.56779	-1666.24518	1294.38985	-1723.90591	1
118	C	72.07821	73.31481	74.23835	493.57570	-129.95376	-602.56394	1
119	H	71.36683	74.15913	74.45601	-1469.08415	-637.84797	2204.22581	1
120	H	73.09322	73.58136	74.61967	-1038.97840	1068.88701	-2280.71789	1
121	C	69.85732	72.32036	72.14911	188.88100	241.05089	-727.87821	1
122	O	69.15146	71.42985	71.66872	531.88613	-49.19943	427.07960	1
123	N	69.49258	73.47800	72.63743	708.14833	26.93417	-179.73509	1
124	H	70.29719	73.99338	72.98363	-2232.69501	-1179.09831	1023.94816	1
125	C	68.12473	73.90763	72.87851	-738.32412	259.92548	104.42456	1
126	H	67.62179	73.70380	71.90336	2028.89123	1260.02983	1311.33324	1
127	C	67.48004	73.12167	74.01152	-258.65678	-345.52513	661.28459	1
128	H	67.99799	73.40421	74.95538	-1826.06090	-1801.46097	-918.64554	1
129	C	66.01247	73.48246	74.15322	-541.23400	531.92970	-218.41428	1
130	H	65.52778	72.93898	75.00988	1311.73218	-2219.18200	882.19023	1
131	H	65.88023	74.59242	74.33773	-1048.71298	1223.58118	-2196.97971	1
132	H	65.43067	73.22588	73.22555	419.06414	1903.92898	1903.44670	1
133	C	67.61195	71.61725	73.85291	-423.86431	152.66566	-648.55262	1
134	H	67.05400	71.06049	74.65553	105.68386	-2342.50080	1387.51238	1
135	H	67.20837	71.27191	72.85953	-1695.63603	-1484.73621	-1531.02793	1
136	H	68.68626	71.28378	73.90069	-1853.57121	-487.39031	1936.59343	1
137	C	68.23015	75.51993	73.28271	539.71428	-510.58232	267.58633	1
138	O	67.35748	75.99830	73.97711	-15.93758	-278.34085	-624.49402	1
139	N	69.26301	76.11813	72.72331	-676.81886	12.05292	276.17960	1
140	H	69.81329	75.52635	72.10499	-1694.44250	-373.84189	-2100.66098	1
141	C	69.51606	77.50734	72.86922	412.33078	-664.70360	108.35451	1
142	H	70.14736	77.72861	71.97968	1765.77476	1294.44366	1621.76594	1
143	C	68.13692	78.27235	72.91845	-179.66635	-721.95667	-264.73915	1
144	H	67.45173	77.89407	72.12547	-607.34922	2587.87032	-598.10018	1
145	H	67.61823	78.06679	73.89215	-359.69332	-1479.13954	-2259.74260	1
146	C	68.38988	79.81056	72.76075	-240.01076	616.02425	431.85322	1
147	H	69.05867	80.17804	73.57407	-1359.17131	782.54425	2227.99181	1
148	H	68.90514	80.04257	71.80186	-820.36667	85.81767	-2596.78715	1
149	C	66.97285	80.50799	72.83384	-513.07015	236.18570	-551.87280	1
150	H	66.31897	80.16399	71.99964	1816.11944	-1183.11722	-1650.94321	1
151	H	66.44258	80.21975	73.77077	-1800.04429	-1361.53473	1526.34643	1
152	C	67.17391	82.04024	72.77692	726.08972	-253.19732	-179.64713	1
153	H	67.73404	82.46357	73.63448	1224.27271	-456.51798	-2390.90304	1
154	H	67.59743	82.41689	71.82454	1206.40339	-1150.93514	-2154.90344	1
155	N	65.75545	82.69617	72.87079	329.70796	-633.68968	-155.67358	1
156	H	65.73346	83.75675	72.86343	1348.31305	1938.76845	1359.00643	1
157	H	65.06145	82.46664	72.10099	2096.92035	1446.54551	-966.48890	1
158	H	65.19297	82.48183	73.74416	-1197.07217	-2303.53719	827.28733	1
159	C	70.30288	77.84818	74.28197	-378.01186	-556.17301	-413.96481	1
160	O	69.81495	78.62665	75.07315	-409.44433	-307.55053	-453.30833	1
161	N	71.48370	77.25714	74.30607	-429.02477	524.70884	274.08502	1
162	H	71.63594	76.63226	73.51727	1706.34263	-1425.96036	1574.38926	1
163	C	72.49613	77.54024	75.28011	-45.25864	-626.22952	478.93267	1
164	H	73.42291	77.47974	74.66222	1988.63164	570.90773	1772.87287	1
165	C	72.25198	78.97969	75.89105	551.77128	-20.49526	-564.54996	1
166	H	71.84814	79.67037	75.11448	1434.61535	-1753.10253	1513.99471	1
167	H	71.44101	78.91397	76.65428	-714.67738	1819.09770	1898.36494	1
168	C	73.57060	79.55520	76.50618	446.99263	-555.77305	338.97221	1
169	H	74.21106	78.74640	76.93507	1953.68466	1892.25957	-161.67766	1
170	H	74.20227	80.04667	75.73256	-403.03785	2688.07032	-188.43591	1
171	C	73.14014	80.57608	77.63460	567.86262	335.74681	434.04421	1
172	H	72.81061	81.54535	77.19470	-1080.02275	1568.26081	-1948.78900	1
173	H	72.26621	80.19334	78.21036	-919.54108	2111.34692	1456.13463	1
174	C	74.35564	80.75905	78.57500	195.81897	173.33862	-745.11536	1
175	H	74.58938	79.87033	79.19775	1405.67949	-2301.21089	-390.08113	1

H	75.28061	81.11520	78.07502	-940.37328	-1118.32446	2299.72133	1
N	73.98285	81.87961	79.60127	319.26422	656.36007	-42.02424	1
H	74.72156	82.11100	80.32639	1961.03633	619.91968	1787.09422	1
H	73.76112	82.84252	79.21280	854.14343	-2156.80752	1429.08711	1
H	73.13901	81.69299	80.21631	1237.02905	-1378.50857	-1998.28500	1
C	72.48146	76.33296	76.35522	532.53319	-268.57236	517.55729	1
O	72.19925	75.18590	75.97668	266.16714	-290.73804	-558.88002	1
N	72.93529	76.69272	77.52127	-226.25352	640.49678	-270.32839	1
H	73.11793	77.68176	77.67251	2007.69063	-1035.27229	1523.50231	1
C	72.97218	75.81143	78.65301	-540.76542	-573.33303	49.50000	1
H	71.94071	75.80579	79.06645	-2374.20950	-1036.76023	843.75774	1
C	73.41088	74.36369	78.15578	-77.09082	666.87422	-415.84136	1
H	73.99842	74.51692	77.20604	-985.81834	617.19825	2463.91927	1
O	72.33022	73.48526	77.82222	293.29899	-391.65602	-477.80926	1
H	72.11168	73.84613	76.88507	2528.72714	-681.76262	-751.27122	1
C	74.36942	73.83239	79.27974	252.47363	-605.14473	440.05134	1
H	74.80404	72.84151	79.01112	-2052.65063	1456.49051	-1043.51830	1
H	75.22472	74.52672	79.46583	-2152.98800	-1661.97574	-161.70652	1
H	73.83294	73.70506	80.24943	-473.12726	-1584.27339	2165.61725	1
C	74.01990	76.54597	79.66638	360.36307	488.46713	505.10056	1
O	73.71252	76.73705	80.82116	-200.08817	-528.03552	-385.82850	1
N	75.16740	76.81363	79.04283	-597.54095	-226.44775	355.20479	1
H	75.22422	76.60100	78.04704	2179.23655	-1300.70981	-991.33835	1
C	76.22196	77.44136	79.75100	-687.68921	297.39807	-249.45179	1
H	75.68765	77.87166	80.62918	-349.16256	-2467.37713	-1101.72831	1
C	77.34691	76.38315	80.19207	672.47345	361.54328	-201.62880	1
H	77.87914	75.99057	79.29860	1541.41502	582.18425	2169.97026	1
H	78.11561	76.90881	80.80653	-2534.67717	60.25153	997.72295	1
C	76.76293	75.21447	80.99498	574.26774	-389.43119	-377.02542	1
C	76.05587	75.45169	82.21197	688.82679	-287.33580	-257.96133	1
H	75.90512	76.48072	82.58528	331.14182	1470.38163	2269.80013	1
C	76.94498	73.88081	80.51421	-383.52382	-675.04808	144.24917	1
H	77.49755	73.69005	79.57635	4.09847	1517.59662	2262.86397	1
C	75.53695	74.36097	82.90798	-134.76730	428.45285	-649.50303	1
H	74.97945	74.54791	83.84253	-903.23433	1600.49020	2011.53387	1
C	76.40934	72.82037	81.25107	-335.53281	-615.00040	-364.39058	1
H	76.55096	71.79482	80.86835	1596.45398	-1369.33862	1732.02659	1
C	75.68762	73.01501	82.46465	585.48547	-340.24194	-406.23938	1
O	75.13659	71.94621	83.21860	-419.15649	-173.04408	-511.94141	1
H	75.39418	71.18719	82.64424	-1071.84350	1559.71507	-1960.13012	1
C	76.88550	78.51930	78.73629	291.41878	-183.13091	710.72246	1
O	76.24814	79.05596	77.85175	379.87695	523.47396	-222.23622	1
N	78.14470	78.76222	79.09403	564.30591	373.09380	277.24371	1
H	78.54606	78.28997	79.90439	-120.59931	-2436.07824	-1214.35098	1
C	78.91923	79.63162	78.28256	404.07514	-299.37480	608.84009	1
H	78.24438	79.91635	77.43962	-1071.07882	-841.69025	2359.66467	1
C	79.30072	80.85591	79.22097	-683.12807	68.71770	-390.13096	1
H	78.40169	81.20668	79.77568	2068.88918	1596.58590	770.89603	1
H	80.02807	80.53688	80.00053	1379.86429	2342.49779	-179.85638	1
C	79.89377	82.00510	78.35188	-176.39967	537.49466	550.97268	1
H	80.80716	81.66586	77.80584	-396.28051	1942.22168	1869.33314	1
H	79.17466	82.33642	77.56937	-94.44700	586.00678	-2659.20016	1
C	80.22584	83.18449	79.37849	-291.69381	-718.18079	-150.72929	1
H	79.35456	83.58659	79.95546	-2303.38776	1089.76242	964.62032	1
H	81.04256	82.95436	80.10941	884.16729	2568.80533	-207.74283	1
N	80.69003	84.20766	78.54114	-594.49522	-252.81478	342.29276	1
H	80.84929	83.96607	77.57536	-1735.17426	2084.05705	-263.71605	1
C	80.93413	85.39461	78.92440	595.23177	-271.52487	442.22352	1
N	80.71644	85.70087	80.27233	585.53813	-424.79750	-105.81303	1
H	80.40088	85.09715	81.03060	959.88797	1327.56823	-2177.11905	1
H	80.88847	86.61118	80.70024	1578.48647	16.27378	-2220.76233	1
N	81.39228	86.18448	77.86121	-452.17763	-390.33541	-421.51853	1

```
H    81.55833  85.95666  76.88189       470.26107      1974.25629     -1817.92087          1
H    81.64963  87.16677  77.96668      1856.67307     -1573.97321     -1224.35416          1
C    80.30678  78.89196  77.81248      -531.47271      -581.45780       -55.06206          1
O    81.16833  79.55670  77.27062      -243.00837       192.73507       609.52485          1
N    80.31233  77.59817  78.09025       343.49288      -644.25607        38.09164          1
H    79.43400  77.29437  78.50547     -1691.16043     -1535.97525     -1484.73099          1
C    81.38163  76.66326  77.80385       542.66492       350.14337       454.42563          1
H    80.86397  75.67514  77.81653      1209.57979     -2396.95920       463.86744          1
C    81.98139  76.98741  76.44632      -737.14309       145.93010       242.71942          1
H    82.19224  78.09427  76.42181     -1050.18303       159.36419     -2509.06125          1
C    83.28596  76.26020  76.18755      -706.77696      -252.88343      -245.16230          1
H    83.68152  76.46811  75.15465     -1624.64039     -1665.49085      1417.86935          1
H    84.08563  76.57272  76.91904      2566.10521       598.51109       693.22305          1
H    83.16657  75.14554  76.28363     -1972.69758       107.10018     -1876.34312          1
C    80.99183  76.68096  75.33602       412.85148       672.58832       -27.69222          1
H    81.38511  76.98816  74.32844      1447.02277      1762.76533     -1490.78944          1
H    80.75556  75.58263  75.28118      1320.77379      -145.20455      2378.68491          1
H    80.01824  77.22150  75.49590     -1512.10848      1470.52031      1724.75147          1
C    82.52302  76.70465  79.03332      -607.11113       499.56689        73.73769          1
O    83.42484  75.89690  78.99887       357.97126       460.81534       356.68785          1
N    82.23119  77.58932  79.96609       536.55407      -368.62270       332.76306          1
H    81.39647  78.12525  79.73925       524.38866      2297.69985      1367.20941          1
C    83.07813  77.91774  81.05305        46.50277      -774.29190      -147.97904          1
H    82.38978  78.40725  81.77567      1676.51875      1883.29066      1032.55549          1
C    83.81905  76.61292  81.64164      -559.41456       -34.19198      -556.30433          1
H    84.41351  76.13076  80.81857       -64.66664       395.18769      2695.05373          1
H    84.55778  76.90099  82.42836     -2703.44278       -60.40056       333.79418          1
C    82.81346  75.63618  82.19142      -519.76435       377.31045       459.42366          1
H    82.04417  75.45211  81.40768     -1746.74323      1667.50644     -1261.73691          1
C    83.49612  74.31397  82.52101       570.95554        16.46261      -545.27700          1
H    82.76828  73.55710  82.91191      2391.58273        28.05277     -1305.07271          1
H    83.98832  73.86755  81.61842       175.75588       917.63458      2559.43867          1
H    84.29003  74.44336  83.30121     -1063.97851     -2155.34650      1283.00462          1
C    82.12802  76.17865  83.43790       193.76014         2.62946      -765.53141          1
H    81.40876  75.43541  83.86934       507.39394      -846.55087      2539.60089          1
H    82.86971  76.42578  84.24084      1337.57841      1818.19054      1526.01994          1
H    81.54887  77.11261  83.22125      1100.88635     -1172.91350     -2199.08909          1
C    84.22542  78.93798  80.57192      -549.21115       475.64486      -309.38218          1
O    84.44449  79.24276  79.42942       556.06106      -390.37755       -78.24250          1
O    85.02194  79.47048  81.61934      -572.27940      -310.19032      -209.75862          1
H    84.56361  79.04120  82.39133     -1380.08193      2212.14486       790.85944          1
```

Listing 71: C00071.xmolout

```
      276
  150.0000  150.0000  150.0000     0.0000     0.0000     0.0000***********    2.7750    11100
      0.2500    300.00    0.00    0.00    0.00   -1293.5846       0.0000    300.0000
H    70.98780  76.29341  64.63402     -2043.14101     -1757.64893       399.89339          1
N    71.34539  77.14752  65.05520       426.75047       490.45733      -334.42467          1
H    71.65383  76.82109  65.96787       442.25559     -1110.30854     -2448.52907          1
C    72.34935  77.81718  64.31655       415.32972      -666.39213        83.73172          1
H    72.11617  77.68635  63.23580       879.32219      2491.00447       667.35213          1
C    72.33001  79.36200  64.81388        31.35357       477.41777       628.23362          1
H    72.41254  79.38452  65.92659      1168.38489      2240.25337     -1019.71136          1
H    73.22141  79.91187  64.42882      -360.43770      2534.90498       931.67112          1
C    71.05564  80.02195  64.36833       463.18208       512.70242      -382.34388          1
H    70.21382  79.31369  64.54202      1725.29117      1863.52247      -987.07854          1
C    70.80817  81.28205  65.19013       710.42801      -144.27427      -313.15367          1
H    69.87269  81.81206  64.87417       364.20195     -1033.13725      2494.72524          1
H    70.71101  81.04959  66.28378      2567.61904       335.80679       847.48802          1
H    71.65817  82.00933  65.09324     -2587.99029       680.92867      -512.16411          1
C    71.10746  80.37763  62.88856       268.17616       741.64379       -40.42634          1
```

H	70.16082	80.87171	62.54870	1283.54314	-1945.37420	-1411.27812	1
H	71.94838	81.08428	62.66569	-1258.57796	1317.15249	-2026.02085	1
H	71.25800	79.47077	62.24811	-2502.84526	909.80597	575.92391	1
C	73.75441	77.20763	64.77144	619.36578	-458.64843	-172.09273	1
O	74.14010	77.09688	65.98904	-81.40433	433.87611	522.34594	1
N	74.50868	76.92023	63.74922	175.23996	-622.20190	341.55523	1
H	74.12569	76.99460	62.80879	-876.57256	-303.22879	-2561.90213	1
C	75.58037	76.03161	64.10757	-571.88214	-534.10783	-106.15033	1
H	75.13509	75.02385	63.99863	-2713.20841	-16.25800	-248.80809	1
C	76.75098	76.27197	63.08410	166.60354	288.16399	-716.09568	1
H	77.00519	77.36766	63.06775	-2097.85574	1636.78449	-586.18044	1
O	76.47163	75.92053	61.70598	-317.80580	-363.38227	484.43082	1
H	76.61962	76.77073	61.25329	1863.74748	-1451.84923	1357.29467	1
C	77.90695	75.47675	63.80093	503.80731	517.99032	318.51606	1
H	78.87910	75.59659	63.26866	640.61020	-2157.10410	-1536.29104	1
H	78.04923	75.83437	64.85257	1687.88455	465.60692	2087.56452	1
H	77.69052	74.38392	63.84709	-1293.07895	-1899.88581	-1463.57452	1
C	75.97843	76.44125	65.70320	-153.07550	-733.68902	248.70981	1
O	76.73967	77.39963	65.88605	186.15459	-480.78787	-449.34387	1
N	76.05560	75.26566	66.44108	-487.53139	543.18517	42.03850	1
C	76.52869	75.31001	67.76540	-699.35871	69.34393	360.10701	1
H	76.42532	76.37907	68.06716	2.81126	-221.99807	-2715.58072	1
C	75.58502	74.27502	68.53054	-507.46439	-592.59194	122.07761	1
H	76.07673	73.82601	69.42275	-1975.08116	525.87560	-1801.71556	1
H	74.66890	74.78285	68.90525	1588.27743	-1913.22100	1113.83566	1
C	75.22413	73.22846	67.43596	-613.62442	-457.55993	-194.14490	1
H	75.96502	72.39780	67.39655	1707.54637	-721.27992	1996.92523	1
H	74.23677	72.74378	67.60387	758.23357	2100.81767	1560.55011	1
C	75.25792	74.11062	66.10313	-352.41524	704.96076	49.21465	1
H	75.74085	73.60715	65.23028	1714.76886	-2070.42778	443.35792	1
H	74.26599	74.51834	65.78820	1224.15040	1801.50881	1636.97633	1
C	78.13133	74.86876	67.80239	566.85117	537.05831	117.62814	1
O	78.92782	75.72425	68.09704	-550.26779	-406.08867	4.21666	1
N	78.33493	73.59944	67.49447	566.34832	-425.77725	180.18749	1
H	77.51885	73.06418	67.20351	644.44502	2646.80550	-52.73430	1
C	79.60600	72.98914	67.68317	612.34801	-281.48662	411.56221	1
H	79.43143	71.93205	67.38160	1667.23498	-1490.75142	1556.16753	1
C	80.64684	73.73710	66.72685	-714.45746	-175.43074	286.98927	1
H	80.75771	74.79425	67.09877	-787.09484	1763.03619	-1922.46060	1
O	80.25589	73.83110	65.34279	-429.46514	516.51932	-128.40526	1
H	80.14085	74.79781	65.25160	1981.82785	436.16847	-1818.18182	1
C	81.98295	72.94596	66.97321	630.79189	-468.94448	76.03230	1
H	82.82741	73.38642	66.39353	-1775.71861	2062.30353	131.89849	1
H	82.28064	72.95621	68.05529	-1855.14608	88.94118	1993.53760	1
H	81.89073	71.87999	66.65937	-1504.82350	-780.94249	-2132.90992	1
C	79.95888	73.14827	69.26910	288.57638	618.62814	-397.00270	1
O	81.08932	73.47359	69.65452	321.70495	-185.26452	574.37218	1
N	78.90644	72.82049	69.96516	-447.05792	-446.53431	367.76425	1
H	78.09977	72.58555	69.38891	350.31316	1975.63644	-1843.31522	1
C	78.81480	72.74628	71.38587	-511.23061	374.03917	-471.51480	1
H	78.45253	71.70803	71.54813	2234.79579	-898.70890	-1273.45190	1
C	80.22445	73.02635	72.07101	529.53781	-159.34528	563.72604	1
H	80.12525	72.75431	73.15802	654.59388	2347.94251	1217.51488	1
H	80.95561	72.30476	71.60945	-1526.55661	2103.42026	817.87394	1
O	80.74599	74.36383	71.99355	-554.84278	14.12789	399.58769	1
H	81.17017	74.29965	71.05937	1297.06236	2061.84854	1220.68812	1
C	77.62132	73.76924	71.82521	-662.15484	-412.13544	123.62687	1
O	76.61020	73.29552	72.28677	-540.01537	-317.05804	274.91456	1
N	77.89797	75.05556	71.59707	-202.76299	-690.00100	-131.49145	1
H	78.83516	75.26130	71.24341	-702.62132	-2617.37191	281.70413	1
C	76.95669	76.09825	71.98675	-491.73914	576.34187	222.73509	1
H	75.94803	75.64560	71.83682	1977.17978	-1823.58850	-434.68928	1

C	77.21675	76.48421	73.43385	-649.50797	-328.66843	306.11224	1
H	78.30615	76.69807	73.54561	2075.07803	1452.16490	-1004.45904	1
C	76.44303	77.73506	73.80628	-615.18735	-489.99689	-70.96352	1
H	76.61842	78.03238	74.87943	-1226.32661	-1724.38906	-1716.47170	1
H	76.74617	78.60768	73.16095	2381.64273	-969.84735	900.46860	1
H	75.33430	77.59378	73.67776	2122.74991	1702.23946	-141.36228	1
C	76.86911	75.35727	74.38796	-501.86074	-479.61276	-376.42482	1
H	77.13349	75.61278	75.45164	993.79261	-2246.95401	-1177.81305	1
H	75.77003	75.11943	74.36364	42.71279	-626.15391	-2651.37258	1
H	77.41320	74.40745	74.11635	-2026.39012	-1590.33709	-887.82922	1
C	77.28705	77.42224	71.02495	-672.89153	382.57394	-156.34072	1
O	78.40530	77.85949	70.92052	410.04627	221.86223	500.35950	1
N	76.14598	77.91156	70.57422	-378.11782	-443.79414	-441.10984	1
H	75.30951	77.37689	70.79896	2404.15973	-568.14894	1149.30085	1
C	76.15252	78.97701	69.61205	-544.54484	466.16019	331.29119	1
H	76.87900	78.64716	68.83342	-1409.80988	-170.17955	2325.32679	1
C	74.65872	79.07065	69.09808	-184.48284	116.78282	-758.89137	1
H	74.32041	78.09547	68.67853	1893.78641	1861.14652	611.04528	1
H	73.97648	79.28144	69.95149	-2170.79665	-974.19311	1327.50117	1
C	74.54680	80.19758	68.02361	389.51668	672.81878	-138.49152	1
H	75.10694	81.10485	68.34815	510.64577	1933.03327	1851.02484	1
H	75.05925	79.88261	67.08647	-2302.30869	-1105.72939	-948.89774	1
C	73.05449	80.59956	67.72191	-559.93427	-126.34590	542.30857	1
O	72.82528	81.53843	67.00146	-670.36003	-111.83166	76.36448	1
N	72.13608	79.80138	68.30557	-216.06729	-537.07037	446.51379	1
H	72.27779	78.96839	68.88820	-1251.96487	1074.12894	-2168.52500	1
H	71.12139	79.90967	68.20405	-2451.83023	-720.73851	944.84612	1
C	76.65777	80.37602	70.27223	546.24052	-563.88337	-85.11809	1
O	77.08878	81.17740	69.48262	71.70702	-307.40463	606.69673	1
N	76.36502	80.57172	71.56627	479.90863	-504.50129	222.86849	1
C	77.07337	81.60359	72.27288	75.51268	-600.91904	506.73700	1
H	77.26258	82.35360	71.46960	-2033.61562	1795.30103	254.89480	1
C	76.05990	82.05524	73.39790	523.79067	517.99274	284.45581	1
H	76.57264	82.34073	74.34428	1599.61665	-1127.00911	1895.98173	1
H	75.49393	82.95789	73.07523	-1881.83093	1275.82488	-1501.55016	1
C	75.09696	80.83605	73.57485	-600.05639	-404.05026	316.64485	1
H	75.50682	80.08665	74.28770	-2597.05260	496.36221	657.73249	1
H	74.09778	81.12199	73.97277	-1666.24518	1294.38985	-1723.90591	1
C	75.03585	80.27422	72.09210	493.57570	-129.95376	-602.56394	1
H	74.86530	79.17641	72.00812	-1469.08415	-637.84797	2204.22581	1
H	74.32405	80.81025	71.42069	-1038.97840	1068.88701	-2280.71789	1
C	78.49506	81.02819	72.85275	188.88100	241.05089	-727.87821	1
O	79.51859	81.29191	72.25904	531.88613	-49.19943	427.07960	1
N	78.36800	80.35182	73.98528	708.14833	26.93417	-179.73509	1
H	77.45020	80.22534	74.40553	-2232.69501	-1179.09831	1023.94816	1
C	79.56498	79.76216	74.49662	-738.32412	259.92548	104.42456	1
H	80.14840	79.50898	73.58080	2028.89123	1260.02983	1311.33324	1
C	80.29310	80.86452	75.38325	-258.65678	-345.52513	661.28459	1
H	79.59893	81.28194	76.14355	-1826.06090	-1801.46097	-918.64554	1
C	81.52463	80.18761	76.07387	-541.23400	531.92970	-218.41428	1
H	82.09506	80.91194	76.70167	1311.73218	-2219.18200	882.19023	1
H	81.20637	79.34959	76.73889	-1048.71298	1223.58118	-2196.97971	1
H	82.23321	79.76358	75.32421	419.06414	1903.92898	1903.44670	1
C	80.82294	82.00622	74.44817	-423.86431	152.66566	-648.55262	1
H	81.52057	82.69059	74.98678	105.68386	-2342.50080	1387.51238	1
H	81.37027	81.59190	73.56615	-1695.63603	-1484.73621	-1531.02793	1
H	79.98614	82.63125	74.05265	-1853.57121	-487.39031	1936.59343	1
C	79.16652	78.49159	75.45121	539.71428	-510.58232	267.58633	1
O	79.65268	77.40249	75.25222	-15.93758	-278.34085	-624.49402	1
N	78.36389	78.87007	76.44047	-676.81886	12.05292	276.17960	1
H	78.02259	79.83027	76.44616	-1694.44250	-373.84189	-2100.66098	1
C	77.80288	77.86433	77.27733	412.33078	-664.70360	108.35451	1

H	78.29152	76.90377	76.98424	1765.77476	1294.44366	1621.76594	1
C	78.06906	78.29205	78.77340	-179.66635	-721.95667	-264.73915	1
H	79.15350	78.47352	78.93622	-607.34922	2587.87032	-598.10018	1
H	77.57205	79.25894	79.00438	-359.69332	-1479.13954	-2259.74260	1
C	77.53964	77.13539	79.72767	-240.01076	616.02425	431.85322	1
H	76.42859	77.03521	79.63273	-1359.17131	782.54425	2227.99181	1
H	77.96010	76.14896	79.40947	-820.36667	85.81767	-2596.78715	1
C	77.92986	77.44576	81.15730	-513.07015	236.18570	-551.87280	1
H	79.03891	77.50277	81.24025	1816.11944	-1183.11722	-1650.94321	1
H	77.57027	78.46378	81.43026	-1800.04429	-1361.53473	1526.34643	1
C	77.40815	76.46860	82.11233	726.08972	-253.19732	-179.64713	1
H	76.29513	76.45041	82.18222	1224.27271	-456.51798	-2390.90304	1
H	77.79072	75.43711	81.97046	1206.40339	-1150.93514	-2154.90344	1
N	77.88807	76.86109	83.54661	329.70796	-633.68968	-155.67358	1
H	77.56750	76.22612	84.33625	1348.31305	1938.76845	1359.00643	1
H	78.93716	76.89067	83.71708	2096.92035	1446.54551	-966.48890	1
H	77.58151	77.81066	83.91216	-1197.07217	-2303.53719	827.28733	1
C	76.17309	77.90607	77.03990	-378.01186	-556.17301	-413.96481	1
O	75.55796	78.88878	76.71570	-409.44433	-307.55053	-453.30833	1
N	75.74705	76.67967	77.29500	-429.02477	524.70884	274.08502	1
H	76.36278	75.90065	77.60380	1706.34263	-1425.96036	1574.38926	1
C	74.35617	76.45730	77.53811	-45.25864	-626.22952	478.93267	1
H	73.88433	77.41940	77.84069	1988.63164	570.90773	1772.87287	1
C	73.82338	75.90841	76.15030	551.77128	-20.49526	-564.54996	1
H	74.20678	76.55241	75.32769	1434.61535	-1753.10253	1513.99471	1
H	74.24478	74.89715	75.95529	-714.67738	1819.09770	1898.36494	1
C	72.26197	75.86715	76.11303	446.99263	-555.77305	338.97221	1
H	71.85594	75.18519	76.89298	1953.68466	1892.25957	-161.67766	1
H	71.82348	76.87101	76.30993	-403.03785	2688.07032	-188.43591	1
C	71.90107	75.35962	74.65712	567.86262	335.74681	434.04421	1
H	72.33642	76.03705	73.88687	-1080.02275	1568.26081	-1948.78900	1
H	72.35668	74.36038	74.46885	-919.54108	2111.34692	1456.13463	1
C	70.36476	75.28850	74.50946	195.81897	173.33862	-745.11536	1
H	69.86689	74.57770	75.19860	1405.67949	-2301.21089	-390.08113	1
H	69.84935	76.26916	74.53881	-940.37328	-1118.32446	2299.72133	1
N	70.07137	74.72123	73.07760	319.26422	656.36007	-42.02424	1
H	69.04726	74.59901	72.83046	1961.03633	619.91968	1787.09422	1
H	70.42112	75.28462	72.24879	854.14343	-2156.80752	1429.08711	1
H	70.45364	73.75518	72.86026	1237.02905	-1378.50857	-1998.28500	1
C	74.51219	75.30913	78.69010	532.53319	-268.57236	517.55729	1
O	75.55809	74.67046	78.72699	266.16714	-290.73804	-558.88002	1
N	73.43361	75.19770	79.44307	-226.25352	640.49678	-270.32839	1
H	72.66022	75.83804	79.26484	2007.69063	-1035.27229	1523.50231	1
C	73.51734	74.45521	80.65710	-540.76542	-573.33303	49.50000	1
H	74.51473	73.96815	80.54322	-2374.20950	-1036.76023	843.75774	1
C	73.47729	75.48772	81.86972	-77.09082	666.87422	-415.84136	1
H	72.42154	75.85080	82.00259	-985.81834	617.19825	2463.91927	1
O	74.26606	76.69558	81.71688	293.29899	-391.65602	-477.80926	1
H	73.56294	77.37104	81.73247	2528.72714	-681.76262	-751.27122	1
C	73.90492	74.58393	83.08198	252.47363	-605.14473	440.05134	1
H	73.79692	75.12492	84.05137	-2052.65063	1456.49051	-1043.51830	1
H	73.29270	73.64938	83.16198	-2152.98800	-1661.97574	-161.70652	1
H	74.97068	74.26996	82.99762	-473.12726	-1584.27339	2165.61725	1
C	72.27435	73.38561	80.59616	360.36307	488.46713	505.10056	1
O	72.16759	72.66295	79.63219	-200.08817	-528.03552	-385.82850	1
N	71.55254	73.38717	81.70755	-597.54095	-226.44775	355.20479	1
H	71.78551	74.05693	82.44027	2179.23655	-1300.70981	-991.33835	1
C	70.31465	72.70536	81.84201	-687.68921	297.39807	-249.45179	1
H	69.86617	73.15739	82.75510	-349.16256	-2467.37713	-1101.72831	1
C	69.42108	72.93528	80.52596	672.47345	361.54328	-201.62880	1
H	69.89546	72.41238	79.66243	1541.41502	582.18425	2169.97026	1
H	68.41452	72.48114	80.67267	-2534.67717	60.25153	997.72295	1

C	69.31297	74.43748	80.23727	574.26774	-389.43119	-377.02542	1
C	68.38005	75.25954	80.93865	688.82679	-287.33580	-257.96133	1
H	67.69036	74.82315	81.68364	331.14182	1470.38163	2269.80013	1
C	70.20007	75.01226	79.27713	-383.52382	-675.04808	144.24917	1
H	70.93469	74.37312	78.74911	4.09847	1517.59662	2262.86397	1
C	68.36600	76.62780	80.66919	-134.76730	428.45285	-649.50303	1
H	67.64615	77.26486	81.21206	-903.23433	1600.49020	2011.53387	1
C	70.14961	76.38784	79.04275	-335.53281	-615.00040	-364.39058	1
H	70.84578	76.82223	78.30455	1596.45398	-1369.33862	1732.02659	1
C	69.23909	77.24533	79.72674	585.48547	-340.24194	-406.23938	1
O	69.18518	78.64591	79.50358	-419.15649	-173.04408	-511.94141	1
H	69.88229	78.73048	78.81047	-1071.84350	1559.71507	-1960.13012	1
C	70.52000	71.10713	81.99138	291.41878	-183.13091	710.72246	1
O	69.57275	70.39317	82.31204	379.87695	523.47396	-222.23622	1
N	71.75985	70.72652	81.72571	564.30591	373.09380	277.24371	1
H	72.40162	71.45871	81.41830	-120.59931	-2436.07824	-1214.35098	1
C	71.92580	69.35363	81.31664	404.07514	-299.37480	608.84009	1
H	70.91936	69.10582	80.90590	-1071.07882	-841.69025	2359.66467	1
C	73.11694	69.27496	80.27985	-683.12807	68.71770	-390.13096	1
H	72.92804	69.93254	79.40049	2068.88918	1596.58590	770.89603	1
H	74.06222	69.64418	80.73777	1379.86429	2342.49779	-179.85638	1
C	73.24647	67.77613	79.86134	-176.39967	537.49466	550.97268	1
H	73.38933	67.13632	80.76281	-396.28051	1942.22168	1869.33314	1
H	72.31079	67.41349	79.38032	-94.44700	586.00678	-2659.20016	1
C	74.49262	67.63465	78.87394	-291.69381	-718.18079	-150.72929	1
H	74.35986	68.08689	77.85803	-2303.38776	1089.76242	964.62032	1
H	75.47892	67.97650	79.27938	884.16729	2568.80533	-207.74283	1
N	74.54922	66.23872	78.73329	-594.49522	-252.81478	342.29276	1
H	73.82085	65.70437	79.18123	-1735.17426	2084.05705	-263.71605	1
C	75.44724	65.60867	78.08963	595.23177	-271.52487	442.22352	1
N	76.45119	66.35930	77.46765	585.53813	-424.79750	-105.81303	1
H	76.57779	67.36994	77.44117	959.88797	1327.56823	-2177.11905	1
H	77.21983	65.96897	76.92213	1578.48647	16.27378	-2220.76233	1
N	75.22628	64.22446	78.14395	-452.17763	-390.33541	-421.51853	1
H	74.49963	63.68182	78.60758	470.26107	1974.25629	-1817.92087	1
H	75.84088	63.54306	77.69786	1856.67307	-1573.97321	-1224.35416	1
C	72.25886	68.49635	82.65886	-531.47271	-581.45780	-55.06206	1
O	73.34079	68.01497	82.87212	-243.00837	192.73507	609.52485	1
N	71.13516	68.42339	83.37696	343.49288	-644.25607	38.09164	1
H	70.29915	68.96636	83.04693	-1691.16043	-1535.97525	-1484.73099	1
C	71.13963	67.84274	84.72386	542.66492	350.14337	454.42563	1
H	70.06288	67.84260	85.01555	1209.57979	-2396.95920	463.86744	1
C	71.71602	66.44053	84.61814	-737.14309	145.93010	242.71942	1
H	72.76751	66.53885	84.24471	-1050.18303	159.36419	-2509.06125	1
C	71.75236	65.71996	85.95159	-706.77696	-252.88343	-245.16230	1
H	72.20891	64.69568	85.85779	-1624.64039	-1665.49085	1417.86935	1
H	72.35713	66.29131	86.71614	2566.10521	598.51109	693.22305	1
H	70.72015	65.58666	86.37830	-1972.69758	107.10018	-1876.34312	1
C	70.95308	65.60150	83.60791	412.85148	672.58832	-27.69222	1
H	71.41988	64.58842	83.46721	1447.02277	1762.76533	-1490.78944	1
H	69.88765	65.43721	83.92740	1320.77379	-145.20455	2378.68491	1
H	70.92387	66.10141	82.60087	-1512.10848	1470.52031	1724.75147	1
C	72.03147	68.82721	85.71016	-607.11113	499.56689	73.73769	1
O	72.35504	68.42772	86.80993	357.97126	460.81534	356.68785	1
N	72.23720	70.02602	85.19147	536.55407	-368.62270	332.76306	1
H	71.88519	70.19060	84.24843	524.38866	2297.69985	1367.20941	1
C	73.13324	70.90979	85.83818	46.50277	-774.29190	-147.97904	1
H	73.16066	70.56505	86.89761	1676.51875	1883.29066	1032.55549	1
C	74.58030	70.84020	85.12020	-559.41456	-34.19198	-556.30433	1
H	74.47305	71.17117	84.05975	-64.66664	395.18769	2695.05373	1
H	75.27354	71.57209	85.59949	-2703.44278	-60.40056	333.79418	1
C	75.16447	69.45457	85.16759	-519.76435	377.31045	459.42366	1

```
H    74.46476   68.75898   84.64315    -1746.74323     1667.50644    -1261.73691          1
C    76.50720   69.42836   84.44494      570.95554       16.46261     -545.27700          1
H    76.95942   68.40330   84.45152     2391.58273       28.05277    -1305.07271          1
H    76.40488   69.73934   83.37354      175.75588      917.63458     2559.43867          1
H    77.24695   70.12017   84.92416    -1063.97851    -2155.34650     1283.00462          1
C    75.35483   68.96967   86.59742      193.76014        2.62946     -765.53141          1
H    75.92893   68.00777   86.63460      507.39394     -846.55087     2539.60089          1
H    75.91538   69.71477   87.21935     1337.57841     1818.19054     1526.01994          1
H    74.36018   68.78185   87.10008     1100.88635    -1172.91350    -2199.08909          1
C    72.62444   72.42035   85.60590     -549.21115      475.64486     -309.38218          1
O    71.88027   72.75057   84.71884      556.06106     -390.37755      -78.24250          1
O    73.24872   73.40157   86.41574     -572.27940     -310.19032     -209.75862          1
H    73.75663   72.79431   87.02282    -1380.08193     2212.14486      790.85944          1
```

Listing 72: C00072.xmolout

```
       276
  150.0000  150.0000  150.0000     0.0000     0.0000     0.0000***********    2.7750    11100
       0.2500    300.00   0.00   0.00   0.00  -1293.5749       0.0000    300.0000
H    68.66714   81.15212   64.89523    -2043.14101    -1757.64893      399.89339          1
N    69.57019   80.79999   65.20851      426.75047      490.45733     -334.42467          1
H    69.63235   79.89153   64.74438      442.25559    -1110.30854    -2448.52907          1
C    70.66804   81.62300   64.87644      415.32972     -666.39213       83.73172          1
H    70.31647   82.67763   64.84612      879.32219     2491.00447      667.35213          1
C    71.82668   81.36129   65.97109       31.35357      477.41777      628.23362          1
H    72.11397   80.25793   65.94740     1168.38489     2240.25337    -1019.71136          1
H    72.74768   81.93459   65.70815     -360.43770     2534.90498      931.67112          1
C    71.36148   81.73223   67.35208      463.18208      512.70242     -382.34388          1
H    70.39258   81.21837   67.54487     1725.29117     1863.52247     -987.07854          1
C    72.38247   81.25723   68.37976      710.42801     -144.27427     -313.15367          1
H    72.05282   81.47943   69.42718      364.20195    -1033.13725     2494.72524          1
H    72.55409   80.15166   68.30882     2567.61904      335.80679      847.48802          1
H    73.37541   81.75530   68.23215    -2587.99029      680.92867     -512.16411          1
C    71.15713   83.23448   67.49092      268.17616      741.64379      -40.42634          1
H    70.86146   83.51619   68.53459     1283.54314    -1945.37420    -1411.27812          1
H    72.09206   83.80210   67.24665    -1258.57796     1317.15249    -2026.02085          1
H    70.35266   83.60806   66.80635    -2502.84526      909.80597      575.92391          1
C    71.21423   81.04400   63.46114      619.36578     -458.64843     -172.09273          1
O    71.08796   79.86458   63.14191      -81.40433      433.87611      522.34594          1
N    71.90943   81.99242   62.86926      175.23996     -622.20190      341.55523          1
H    71.86104   82.92800   63.27093     -876.57256     -303.22879    -2561.90213          1
C    72.37845   81.82556   61.52108     -571.88214     -534.10783     -106.15033          1
H    71.44983   81.56337   60.96666    -2713.20841      -16.25800     -248.80809          1
C    72.97335   83.24524   61.10582      166.60354      288.16399     -716.09568          1
H    73.92447   83.42864   61.67631    -2097.85574     1636.78449     -586.18044          1
O    72.12012   84.37255   61.39477     -317.80580     -363.38227      484.43082          1
H    72.71227   84.92075   61.94573     1863.74748    -1451.84923     1357.29467          1
C    73.33095   83.02970   59.58969      503.80731      517.99032      318.51606          1
H    73.81074   83.93572   59.15178      640.61020    -2157.10410    -1536.29104          1
H    74.03747   82.17783   59.44278     1687.88455      465.60692     2087.56452          1
H    72.42047   82.81270   58.98399    -1293.07895    -1899.88581    -1463.57452          1
C    73.47091   80.62848   61.38763     -153.07550     -733.68902      248.70981          1
O    73.52949   80.12861   60.29338      186.15459     -480.78787     -449.34387          1
N    74.26290   80.40113   62.44515     -487.53139      543.18517       42.03850          1
C    75.16408   79.28968   62.48007     -699.35871       69.34393      360.10701          1
H    75.53650   79.14076   61.44035        2.81126     -221.99807    -2715.58072          1
C    76.24343   79.76368   63.54652     -507.46439     -592.59194      122.07761          1
H    76.81092   78.91536   63.99046    -1975.08116      525.87560    -1801.71556          1
H    76.99312   80.45208   63.09656     1588.27743    -1913.22100     1113.83566          1
C    75.33582   80.50676   64.57452     -613.62442     -457.55993     -194.14490          1
H    74.75959   79.78602   65.19874     1707.54637     -721.27992     1996.92523          1
H    75.89415   81.16689   65.27574      758.23357     2100.81767     1560.55011          1
```

C	74.38262	81.30390	63.58360	-352.41524	704.96076	49.21465	1
H	73.35358	81.49050	63.96513	1714.76886	-2070.42778	443.35792	1
H	74.80611	82.25646	63.18778	1224.15040	1801.50881	1636.97633	1
C	74.43657	77.95044	63.08102	566.85117	537.05831	117.62814	1
O	75.12399	76.98474	63.29670	-550.26779	-406.08867	4.21666	1
N	73.13325	78.11348	63.23451	566.34832	-425.77725	180.18749	1
H	72.66633	79.00897	62.97835	644.44502	2646.80550	-52.73430	1
C	72.34690	77.14616	63.93041	612.34801	-281.48662	411.56221	1
H	71.33471	77.55022	63.69570	1667.23498	-1490.75142	1556.16753	1
C	72.60722	75.70199	63.30311	-714.45746	-175.43074	286.98927	1
H	73.61937	75.35008	63.65445	-787.09484	1763.03619	-1922.46060	1
O	72.67463	75.64066	61.86018	-429.46514	516.51932	-128.40526	1
H	73.64340	75.71140	61.73297	1981.82785	436.16847	-1818.18182	1
C	71.46962	74.82373	63.94086	630.79189	-468.94448	76.03230	1
H	71.62290	73.74208	63.71857	-1775.71861	2062.30353	131.89849	1
H	71.41532	74.92889	65.04938	-1855.14608	88.94118	1993.53760	1
H	70.47025	75.10360	63.53451	-1504.82350	-780.94249	-2132.90992	1
C	72.70223	77.31819	65.51041	288.57638	618.62814	-397.00270	1
O	72.58277	78.40925	66.03105	321.70495	-185.26452	574.37218	1
N	73.05081	76.18185	66.07682	-447.05792	-446.53431	367.76425	1
H	73.18590	75.26414	65.61401	350.31316	1975.63644	-1843.31522	1
C	73.14593	76.13122	67.51110	-511.23061	374.03917	-471.51480	1
H	73.87303	76.92105	67.80737	2234.79579	-898.70890	-1273.45190	1
C	71.68749	76.42989	68.11318	529.53781	-159.34528	563.72604	1
H	71.81268	76.57833	69.22068	654.59388	2347.94251	1217.51488	1
H	71.36114	77.41614	67.68503	-1526.55661	2103.42026	817.87394	1
O	70.68181	75.40586	67.92306	-554.84278	14.12789	399.58769	1
H	70.39215	75.63178	67.01847	1297.06236	2061.84854	1220.68812	1
C	73.64525	74.61472	67.70135	-662.15484	-412.13544	123.62687	1
O	73.74878	73.89693	66.71546	-540.01537	-317.05804	274.91456	1
N	73.88905	74.30602	68.97736	-202.76299	-690.00100	-131.49145	1
H	73.81955	75.08991	69.62757	-702.62132	-2617.37191	281.70413	1
C	74.49953	73.02577	69.31580	-491.73914	576.34187	222.73509	1
H	74.16162	72.37783	68.47126	1977.17978	-1823.58850	-434.68928	1
C	76.00783	73.22007	69.30466	-649.50797	-328.66843	306.11224	1
H	76.23349	74.13773	69.89202	2075.07803	1452.16490	-1004.45904	1
C	76.74784	72.06087	69.94346	-615.18735	-489.99689	-70.96352	1
H	77.86406	72.19377	69.88671	-1226.32661	-1724.38906	-1716.47170	1
H	76.47845	71.94582	71.03319	2381.64273	-969.84735	900.46860	1
H	76.49983	71.08535	69.43917	2122.74991	1702.23946	-141.36228	1
C	76.52265	73.43606	67.89300	-501.86074	-479.61276	-376.42482	1
H	77.59962	73.76119	67.88207	993.79261	-2246.95401	-1177.81305	1
H	76.45171	72.49481	67.28063	42.71279	-626.15391	-2651.37258	1
H	75.92171	74.22091	67.34988	-2026.39012	-1590.33709	-887.82922	1
C	73.98923	72.46715	70.80329	-672.89153	382.57394	-156.34072	1
O	74.27936	71.32429	71.08480	410.04627	221.86223	500.35950	1
N	73.29055	73.33620	71.50246	-378.11782	-443.79414	-441.10984	1
H	73.11084	74.20185	71.00230	2404.15973	-568.14894	1149.30085	1
C	73.08024	73.24410	72.91435	-544.54484	466.16019	331.29119	1
H	72.09792	73.73746	73.07232	-1409.80988	-170.17955	2325.32679	1
C	73.13146	71.72148	73.32253	-184.48284	116.78282	-758.89137	1
H	72.56187	71.11624	72.57825	1893.78641	1861.14652	611.04528	1
H	74.18951	71.36146	73.26096	-2170.79665	-974.19311	1327.50117	1
C	72.58497	71.48298	74.76980	389.51668	672.81878	-138.49152	1
H	73.09076	72.15663	75.49828	510.64577	1933.03327	1851.02484	1
H	71.51223	71.77138	74.83334	-2302.30869	-1105.72939	-948.89774	1
C	72.78235	69.98528	75.19653	-559.93427	-126.34590	542.30857	1
O	72.59620	69.63719	76.34475	-670.36003	-111.83166	76.36448	1
N	73.13440	69.17888	74.17929	-216.06729	-537.07037	446.51379	1
H	73.34582	69.40397	73.19936	-1251.96487	1074.12894	-2168.52500	1
H	73.33878	68.17863	74.28516	-2451.83023	-720.73851	944.84612	1
C	74.30972	74.02202	73.66155	546.24052	-563.88337	-85.11809	1

108	O	75.41339	73.53983	73.65216	71.70702	-307.40463	606.69673	1
109	N	73.90734	75.17886	74.21742	479.90863	-504.50129	222.86849	1
110	C	74.86475	76.00061	74.88828	75.51268	-600.91904	506.73700	1
111	H	75.85527	75.73380	74.45109	-2033.61562	1795.30103	254.89480	1
112	C	74.32465	77.45864	74.55385	523.79067	517.99274	284.45581	1
113	H	74.67489	78.22331	75.28249	1599.61665	-1127.00911	1895.98173	1
114	H	74.66848	77.79565	73.55105	-1881.83093	1275.82488	-1501.55016	1
115	C	72.77826	77.25266	74.57623	-600.05639	-404.05026	316.64485	1
116	H	72.38908	77.25679	75.61899	-2597.05260	496.36221	657.73249	1
117	H	72.21763	78.04437	74.03075	-1666.24518	1294.38985	-1723.90591	1
118	C	72.63456	75.81744	73.91039	493.57570	-129.95376	-602.56394	1
119	H	71.81908	75.18694	74.33742	-1469.08415	-637.84797	2204.22581	1
120	H	72.55293	75.82225	72.79775	-1038.97840	1068.88701	-2280.71789	1
121	C	74.84375	75.77800	76.51221	188.88100	241.05089	-727.87821	1
122	O	75.83546	76.03344	77.14480	531.88613	-49.19943	427.07960	1
123	N	73.62607	75.47625	76.95145	708.14833	26.93417	-179.73509	1
124	H	72.86070	75.25969	76.28107	-2232.69501	-1179.09831	1023.94816	1
125	C	73.50359	74.82502	78.22399	-738.32412	259.92548	104.42456	1
126	H	74.48393	74.35771	78.46323	2028.89123	1260.02983	1311.33324	1
127	C	73.07743	75.91477	79.30108	-258.65678	-345.52513	661.28459	1
128	H	72.32336	76.60495	78.86542	-1826.06090	-1801.46097	-918.64554	1
129	C	72.53065	75.22659	80.59805	-541.23400	531.92970	-218.41428	1
130	H	72.25695	75.97729	81.37645	1311.73218	-2219.18200	882.19023	1
131	H	71.61803	74.62026	80.38812	-1048.71298	1223.58118	-2196.97971	1
132	H	73.28743	74.54473	81.05281	419.06414	1903.92898	1903.44670	1
133	C	74.35795	76.69628	79.75351	-423.86431	152.66566	-648.55262	1
134	H	74.11175	77.51079	80.47513	105.68386	-2342.50080	1387.51238	1
135	H	75.09408	76.02397	80.25499	-1695.63603	-1484.73621	-1531.02793	1
136	H	74.87813	77.16381	78.88204	-1853.57121	-487.39031	1936.59343	1
137	C	72.28399	73.80977	77.87065	539.71428	-510.58232	267.58633	1
138	O	71.62941	74.02028	76.85032	-15.93758	-278.34085	-624.49402	1
139	N	72.22057	72.81606	78.73393	-676.81886	12.05292	276.17960	1
140	H	72.86444	72.89156	79.52271	-1694.44250	-373.84189	-2100.66098	1
141	C	71.01807	72.03823	78.89353	412.33078	-664.70360	108.35451	1
142	H	70.28163	72.72122	79.37114	1765.77476	1294.44366	1621.76594	1
143	C	70.55346	71.56998	77.46637	-179.66635	-721.95667	-264.73915	1
144	H	70.42575	72.45386	76.77973	-607.34922	2587.87032	-598.10018	1
145	H	71.37464	70.93562	77.00824	-359.69332	-1479.13954	-2259.74260	1
146	C	69.22366	70.74465	77.52715	-240.01076	616.02425	431.85322	1
147	H	69.34984	69.81642	78.12994	-1359.17131	782.54425	2227.99181	1
148	H	68.40173	71.32080	78.00756	-820.36667	85.81767	-2596.78715	1
149	C	68.90486	70.42854	76.01012	-513.07015	236.18570	-551.87280	1
150	H	68.78585	71.37490	75.43398	1816.11944	-1183.11722	-1650.94321	1
151	H	69.76158	69.89502	75.53645	-1800.04429	-1361.53473	1526.34643	1
152	C	67.61917	69.57675	75.91169	726.08972	-253.19732	-179.64713	1
153	H	67.72641	68.53327	76.26918	1224.27271	-456.51798	-2390.90304	1
154	H	66.71880	70.04145	76.36048	1206.40339	-1150.93514	-2154.90344	1
155	N	67.27834	69.45103	74.38681	329.70796	-633.68968	-155.67358	1
156	H	66.42833	68.86268	74.15017	1348.31305	1938.76845	1359.00643	1
157	H	67.05846	70.35061	73.86782	2096.92035	1446.54551	-966.48890	1
158	H	68.01599	69.01352	73.76156	-1197.07217	-2303.53719	827.28733	1
159	C	71.36028	70.73988	79.81594	-378.01186	-556.17301	-413.96481	1
160	O	70.46523	70.17821	80.40664	-409.44433	-307.55053	-453.30833	1
161	N	72.65518	70.41298	79.77737	-429.02477	524.70884	274.08502	1
162	H	73.23945	70.97409	79.15941	1706.34263	-1425.96036	1574.38926	1
163	C	73.06363	69.13737	80.24232	-45.25864	-626.22952	478.93267	1
164	H	72.17930	68.75868	80.80682	1988.63164	570.90773	1772.87287	1
165	C	73.40016	68.26855	78.95907	551.77128	-20.49526	-564.54996	1
166	H	72.62211	68.39859	78.16757	1434.61535	-1753.10253	1513.99471	1
167	H	74.35527	68.60088	78.49221	-714.67738	1819.09770	1898.36494	1
168	C	73.49602	66.78583	79.44611	446.99263	-555.77305	338.97221	1
169	H	74.25792	66.71163	80.26568	1953.68466	1892.25957	-161.67766	1

H	72.53110	66.45459	79.89233	-403.03785	2688.07032	-188.43591	1
C	73.89651	65.84721	78.23820	567.86262	335.74681	434.04421	1
H	73.08353	65.79823	77.47752	-1080.02275	1568.26081	-1948.78900	1
H	74.79182	66.24092	77.70372	-919.54108	2111.34692	1456.13463	1
C	74.18543	64.45598	78.84969	195.81897	173.33862	-745.11536	1
H	75.08368	64.41923	79.49816	1405.67949	-2301.21089	-390.08113	1
H	73.32452	63.99314	79.37213	-940.37328	-1118.32446	2299.72133	1
N	74.50906	63.46755	77.67988	319.26422	656.36007	-42.02424	1
H	74.77181	62.48296	77.97526	1961.03633	619.91968	1787.09422	1
H	73.73510	63.27449	76.97957	854.14343	-2156.80752	1429.08711	1
H	75.32297	63.71903	77.04676	1237.02905	-1378.50857	-1998.28500	1
C	74.43800	69.26177	81.14068	532.53319	-268.57236	517.55729	1
O	75.03346	68.24520	81.43520	266.16714	-290.73804	-558.88002	1
N	74.68158	70.51259	81.48225	-226.25352	640.49678	-270.32839	1
H	73.97273	71.16634	81.15371	2007.69063	-1035.27229	1523.50231	1
C	75.75827	70.97345	82.28050	-540.76542	-573.33303	49.50000	1
H	75.37355	71.91365	82.73389	-2374.20950	-1036.76023	843.75774	1
C	76.18845	69.85636	83.33487	-77.09082	666.87422	-415.84136	1
H	76.57523	68.96792	82.75399	-985.81834	617.19825	2463.91927	1
O	75.13515	69.35772	84.18773	293.29899	-391.65602	-477.80926	1
H	74.88621	68.54773	83.69049	2528.72714	-681.76262	-751.27122	1
C	77.38537	70.56780	84.06700	252.47363	-605.14473	440.05134	1
H	77.88189	69.88349	84.79343	-2052.65063	1456.49051	-1043.51830	1
H	78.16713	70.91518	83.34992	-2152.98800	-1661.97574	-161.70652	1
H	77.04051	71.46184	84.63668	-473.12726	-1584.27339	2165.61725	1
C	77.05998	71.23568	81.30924	360.36307	488.46713	505.10056	1
O	77.64224	70.35453	80.72726	-200.08817	-528.03552	-385.82850	1
N	77.35438	72.52506	81.40972	-597.54095	-226.44775	355.20479	1
H	76.77154	73.18115	81.94330	2179.23655	-1300.70981	-991.33835	1
C	78.47741	73.01267	80.69226	-687.68921	297.39807	-249.45179	1
H	78.98226	72.07554	80.35777	-349.16256	-2467.37713	-1101.72831	1
C	77.89344	73.85266	79.45410	672.47345	361.54328	-201.62880	1
H	77.14180	74.59747	79.80237	1541.41502	582.18425	2169.97026	1
H	78.70696	74.41732	78.94380	-2534.67717	60.25153	997.72295	1
C	77.26661	72.83733	78.48935	574.26774	-389.43119	-377.02542	1
C	78.13848	72.15251	77.59010	688.82679	-287.33580	-257.96133	1
H	79.21986	72.37916	77.57030	331.14182	1470.38163	2269.80013	1
C	75.87454	72.51933	78.51126	-383.52382	-675.04808	144.24917	1
H	75.19936	73.02434	79.22762	4.09847	1517.59662	2262.86397	1
C	77.60296	71.17826	76.75117	-134.76730	428.45285	-649.50303	1
H	78.28387	70.64386	76.06610	-903.23433	1600.49020	2011.53387	1
C	75.38076	71.55224	77.62846	-335.53281	-615.00040	-364.39058	1
H	74.29811	71.33618	77.63424	1596.45398	-1369.33862	1732.02659	1
C	76.22071	70.83242	76.72946	585.48547	-340.24194	-406.23938	1
O	75.76789	69.81881	75.84928	-419.15649	-173.04408	-511.94141	1
H	74.79710	69.84673	76.02934	-1071.84350	1559.71507	-1960.13012	1
C	79.36423	73.87969	81.74750	291.41878	-183.13091	710.72246	1
O	79.49789	73.54298	82.90446	379.87695	523.47396	-222.23622	1
N	79.98542	74.88242	81.13151	564.30591	373.09380	277.24371	1
H	79.77134	75.11992	80.16541	-120.59931	-2436.07824	-1214.35098	1
C	80.95450	75.59699	81.89977	404.07514	-299.37480	608.84009	1
H	81.37863	74.84762	82.60792	-1071.07882	-841.69025	2359.66467	1
C	81.96004	76.22600	80.85462	-683.12807	68.71770	-390.13096	1
H	82.31131	75.45860	80.12867	2068.88918	1596.58590	770.89603	1
H	81.42762	76.99116	80.24537	1379.86429	2342.49779	-179.85638	1
C	83.16824	76.87123	81.60246	-176.39967	537.49466	550.97268	1
H	82.83471	77.45079	82.49053	-396.28051	1942.22168	1869.33314	1
H	83.86986	76.10088	81.99232	-94.44700	586.00678	-2659.20016	1
C	83.86383	77.81869	80.51921	-291.69381	-718.18079	-150.72929	1
H	84.29917	77.28981	79.63341	-2303.38776	1089.76242	964.62032	1
H	83.22622	78.65214	80.13242	884.16729	2568.80533	-207.74283	1
N	84.92079	78.42116	81.21221	-594.49522	-252.81478	342.29276	1

```
H    85.06120  78.13189  82.16744    -1735.17426     2084.05705     -263.71605          1
C    85.70148  79.29118  80.71333      595.23177     -271.52487      442.22352          1
N    85.48693  79.67364  79.38425      585.53813     -424.79750     -105.81303          1
H    84.78007  79.35220  78.72393      959.88797     1327.56823    -2177.11905          1
H    86.04783  80.35767  78.87538     1578.48647       16.27378    -2220.76233          1
N    86.66310  79.70729  81.64448     -452.17763     -390.33541     -421.51853          1
H    86.82619  79.42692  82.61049      470.26107     1974.25629    -1817.92087          1
H    87.38373  80.39772  81.42906     1856.67307    -1573.97321    -1224.35416          1
C    80.19174  76.83711  82.66754     -531.47271     -581.45780      -55.06206          1
O    79.49356  77.63675  82.09190     -243.00837      192.73507      609.52485          1
N    80.60326  76.84421  83.93283      343.49288     -644.25607       38.09164          1
H    81.23394  76.08282  84.18292    -1691.16043    -1535.97525    -1484.73099          1
C    80.02672  77.70690  84.95207      542.66492      350.14337      454.42563          1
H    80.68624  77.53638  85.83653     1209.57979    -2396.95920      463.86744          1
C    80.04272  79.14730  84.46958     -737.14309      145.93010      242.71942          1
H    79.35137  79.21012  83.59291    -1050.18303      159.36419    -2509.06125          1
C    79.55395  80.10588  85.53861     -706.77696     -252.88343     -245.16230          1
H    79.50335  81.16361  85.15976    -1624.64039    -1665.49085     1417.86935          1
H    78.51639  79.82312  85.89827     2566.10521      598.51109      693.22305          1
H    80.23111  80.10751  86.43665    -1972.69758      107.10018    -1876.34312          1
C    81.41790  79.58158  83.99377      412.85148      672.58832      -27.69222          1
H    81.42517  80.65566  83.66178     1447.02277     1762.76533    -1490.78944          1
H    82.19060  79.47696  84.80373     1320.77379     -145.20455     2378.68491          1
H    81.76115  78.96233  83.12167    -1512.10848     1470.52031     1724.75147          1
C    78.46617  77.18215  85.24483     -607.11113      499.56689       73.73769          1
O    77.64425  77.97973  85.64753      357.97126      460.81534      356.68785          1
N    78.35319  75.87874  85.09316      536.55407     -368.62270      332.76306          1
H    79.21175  75.44533  84.76056      524.38866     2297.69985     1367.20941          1
C    77.22197  75.13111  85.52053       46.50277     -774.29190     -147.97904          1
H    77.65086  74.11633  85.67777     1676.51875     1883.29066     1032.55549          1
C    76.53425  75.75636  86.84095     -559.41456      -34.19198     -556.30433          1
H    76.21912  76.81281  86.62520      -64.66664      395.18769     2695.05373          1
H    75.59620  75.20019  87.08180    -2703.44278      -60.40056      333.79418          1
C    77.48831  75.71988  88.00239     -519.76435      377.31045      459.42366          1
H    78.45009  76.17967  87.68049    -1746.74323     1667.50644    -1261.73691          1
C    76.93094  76.52954  89.16756      570.95554       16.46261     -545.27700          1
H    77.62777  76.52811  90.04480     2391.58273       28.05277    -1305.07271          1
H    76.75765  77.59903  88.88156      175.75588      917.63458     2559.43867          1
H    75.95100  76.11881  89.52288    -1063.97851    -2155.34650     1283.00462          1
C    77.75263  74.29100  88.45894      193.76014        2.62946     -765.53141          1
H    78.42805  74.26071  89.35232      507.39394     -846.55087     2539.60089          1
H    76.80339  73.76829  88.74379     1337.57841     1818.19054     1526.01994          1
H    78.23881  73.68074  87.65543     1100.88635    -1172.91350    -2199.08909          1
C    76.13854  75.12422  84.34631     -549.21115      475.64486     -309.38218          1
O    74.95973  75.25429  84.53639      556.06106     -390.37755      -78.24250          1
O    76.62613  74.75909  83.04663     -572.27940     -310.19032     -209.75862          1
H    77.59974  74.75724  83.25697    -1380.08193     2212.14486      790.85944          1
```

Listing 73: C00073.xmolout

```
       276
  150.0000  150.0000  150.0000     0.0000     0.0000     0.0000***********    2.7750    11100
       0.2500    300.00    0.00    0.00    0.00   -1293.5588       0.0000     300.0000
H    79.75613  61.21994  84.47265    -2043.14101    -1757.64893      399.89339          1
N    80.09836  61.17527  83.51549      426.75047      490.45733     -334.42467          1
H    79.24356  61.32853  82.98166      442.25559    -1110.30854    -2448.52907          1
C    81.13097  62.09573  83.20085      415.32972     -666.39213       83.73172          1
H    81.60806  62.35867  84.17376      879.32219     2491.00447      667.35213          1
C    82.18023  61.36810  82.19465       31.35357      477.41777      628.23362          1
H    81.65730  61.10729  81.24503     1168.38489     2240.25337    -1019.71136          1
H    82.99827  62.07047  81.91120     -360.43770     2534.90498      931.67112          1
C    82.73275  60.13568  82.84384      463.18208      512.70242     -382.34388          1
```

H	81.87642	59.50382	83.17366	1725.29117	1863.52247	-987.07854	1
C	83.57284	59.34212	81.84835	710.42801	-144.27427	-313.15367	1
H	83.97527	58.40143	82.30419	364.20195	-1033.13725	2494.72524	1
H	82.97797	59.04658	80.94653	2567.61904	335.80679	847.48802	1
H	84.45095	59.93588	81.48640	-2587.99029	680.92867	-512.16411	1
C	83.58773	60.47108	84.06005	268.17616	741.64379	-40.42634	1
H	84.03976	59.55132	84.51242	1283.54314	-1945.37420	-1411.27812	1
H	84.43112	61.15871	83.79457	-1258.57796	1317.15249	-2026.02085	1
H	82.98929	60.97256	84.86269	-2502.84526	909.80597	575.92391	1
C	80.42916	63.32653	82.48721	619.36578	-458.64843	-172.09273	1
O	79.16296	63.45391	82.30490	-81.40433	433.87611	522.34594	1
N	81.29636	64.21967	82.12290	175.23996	-622.20190	341.55523	1
H	82.28598	64.08714	82.31663	-876.57256	-303.22879	-2561.90213	1
C	80.74377	65.38753	81.51002	-571.88214	-534.10783	-106.15033	1
H	81.09219	66.21203	82.16257	-2713.20841	-16.25800	-248.80809	1
C	81.39692	65.49014	80.08183	166.60354	288.16399	-716.09568	1
H	81.03301	64.63265	79.45297	-2097.85574	1636.78449	-586.18044	1
O	82.84502	65.43382	80.03919	-317.80580	-363.38227	484.43082	1
H	82.99068	64.64052	79.49295	1863.74748	-1451.84923	1357.29467	1
C	80.75494	66.83693	79.57361	503.80731	517.99032	318.51606	1
H	81.04503	67.05325	78.51962	640.61020	-2157.10410	-1536.29104	1
H	79.63827	66.80710	79.61168	1687.88455	465.60692	2087.56452	1
H	81.08508	67.70422	80.19083	-1293.07895	-1899.88581	-1463.57452	1
C	79.06319	65.20009	81.42682	-153.07550	-733.68902	248.70981	1
O	78.51039	65.02243	80.33169	186.15459	-480.78787	-449.34387	1
N	78.52848	66.04362	82.39911	-487.53139	543.18517	42.03850	1
C	77.13133	66.17842	82.47204	-699.35871	69.34393	360.10701	1
H	76.73392	65.40801	81.76666	2.81126	-221.99807	-2715.58072	1
C	76.87627	65.93667	84.03863	-507.46439	-592.59194	122.07761	1
H	75.89028	66.32038	84.38126	-1975.08116	525.87560	-1801.71556	1
H	76.90749	64.85375	84.29082	1588.27743	-1913.22100	1113.83566	1
C	78.08037	66.69531	84.66078	-613.62442	-457.55993	-194.14490	1
H	77.88921	67.79404	84.68766	1707.54637	-721.27992	1996.92523	1
H	78.31439	66.38616	85.70387	758.23357	2100.81767	1560.55011	1
C	79.24215	66.33542	83.61943	-352.41524	704.96076	49.21465	1
H	79.95081	67.17435	83.41550	1714.76886	-2070.42778	443.35792	1
H	79.82263	65.41091	83.85947	1224.15040	1801.50881	1636.97633	1
C	76.68104	67.72653	82.08988	566.85117	537.05831	117.62814	1
O	76.87120	68.63608	82.89740	-550.26779	-406.08867	4.21666	1
N	76.19729	67.76101	80.88416	566.34832	-425.77725	180.18749	1
H	76.17151	66.84768	80.43046	644.44502	2646.80550	-52.73430	1
C	75.33491	68.79095	80.37691	612.34801	-281.48662	411.56221	1
H	74.32021	68.34242	80.44857	1667.23498	-1490.75142	1556.16753	1
C	75.46896	70.13228	81.21699	-714.45746	-175.43074	286.98927	1
H	76.56849	70.31847	81.38889	-787.09484	1763.03619	-1922.46060	1
O	74.88927	70.10769	82.52503	-429.46514	516.51932	-128.40526	1
H	75.70082	69.77421	83.05497	1981.82785	436.16847	-1818.18182	1
C	74.88477	71.21648	80.23941	630.79189	-468.94448	76.03230	1
H	74.95012	72.23798	80.67976	-1775.71861	2062.30353	131.89849	1
H	75.43054	71.24232	79.26783	-1855.14608	88.94118	1993.53760	1
H	73.81084	71.02210	80.01252	-1504.82350	-780.94249	-2132.90992	1
C	75.73719	68.97102	78.80512	288.57638	618.62814	-397.00270	1
O	74.91499	68.60400	77.96083	321.70495	-185.26452	574.37218	1
N	76.90605	69.53380	78.64653	-447.05792	-446.53431	367.76425	1
H	77.40075	69.71562	79.51937	350.31316	1975.63644	-1843.31522	1
C	77.49045	69.88643	77.40316	-511.23061	374.03917	-471.51480	1
H	78.56693	69.70231	77.61213	2234.79579	-898.70890	-1273.45190	1
C	76.91091	68.96273	76.24341	529.53781	-159.34528	563.72604	1
H	77.66170	68.97148	75.40246	654.59388	2347.94251	1217.51488	1
H	76.88110	67.92200	76.66787	-1526.55661	2103.42026	817.87394	1
O	75.64805	69.29900	75.64978	-554.84278	14.12789	399.58769	1
H	75.02974	68.92566	76.39121	1297.06236	2061.84854	1220.68812	1

```
C    77.28613   71.50113   77.16199     -662.15484     -412.13544      123.62687          1
O    78.28976   72.18105   77.18052     -540.01537     -317.05804      274.91456          1
N    76.01948   71.87354   77.05136     -202.76299     -690.00100     -131.49145          1
H    75.38599   71.07216   77.05731     -702.62132    -2617.37191      281.70413          1
C    75.53611   73.22540   76.81432     -491.73914      576.34187      222.73509          1
H    74.59275   73.27681   77.40741     1977.17978    -1823.58850     -434.68928          1
C    76.57022   74.25286   77.22751     -649.50797     -328.66843      306.11224          1
H    77.51753   74.03403   76.66744     2075.07803     1452.16490    -1004.45904          1
C    76.14416   75.66957   76.89852     -615.18735     -489.99689      -70.96352          1
H    76.87879   76.42157   77.29793    -1226.32661    -1724.38906    -1716.47170          1
H    76.06548   75.85170   75.76433     2381.64273     -969.84735      900.46860          1
H    75.14445   75.91679   77.34804     2122.74991     1702.23946     -141.36228          1
C    76.88393   74.16264   78.71203     -501.86074     -479.61276     -376.42482          1
H    77.67674   74.89959   79.01489      993.79261    -2246.95401    -1177.81305          1
H    75.97719   74.36615   79.34368       42.71279     -626.15391    -2651.37258          1
H    77.26131   73.13754   78.98434    -2026.39012    -1590.33709     -887.82922          1
C    75.22030   73.34260   75.17631     -672.89153      382.57394     -156.34072          1
O    76.09201   73.23348   74.35111      410.04627      221.86223      500.35950          1
N    73.94260   73.63012   74.99831     -378.11782     -443.79414     -441.10984          1
H    73.32925   73.64794   75.80910     2404.15973     -568.14894     1149.30085          1
C    73.46095   73.79959   73.66091     -544.54484      466.16019      331.29119          1
H    74.26433   74.40327   73.18230    -1409.80988     -170.17955     2325.32679          1
C    73.37792   72.35157   73.02524     -184.48284      116.78282     -758.89137          1
H    74.34580   71.81318   73.15424     1893.78641     1861.14652      611.04528          1
H    72.60973   71.73494   73.54555    -2170.79665     -974.19311     1327.50117          1
C    73.02530   72.53871   71.51642      389.51668      672.81878     -138.49152          1
H    72.06825   73.09796   71.41874      510.64577     1933.03327     1851.02484          1
H    73.77603   73.19751   71.02647    -2302.30869    -1105.72939     -948.89774          1
C    72.92201   71.18301   70.72115     -559.93427     -126.34590      542.30857          1
O    72.60747   71.20505   69.55907     -670.36003     -111.83166       76.36448          1
N    73.21830   70.08538   71.44969     -216.06729     -537.07037      446.51379          1
H    73.50511   70.00261   72.43232    -1251.96487     1074.12894    -2168.52500          1
H    73.21116   69.13079   71.07448    -2451.83023     -720.73851      944.84612          1
C    71.98981   74.50860   73.72498      546.24052     -563.88337      -85.11809          1
O    71.00334   73.82194   73.75381       71.70702     -307.40463      606.69673          1
N    72.09181   75.85318   73.78036      479.90863     -504.50129      222.86849          1
C    70.96124   76.71187   73.74716       75.51268     -600.91904      506.73700          1
H    70.07406   76.12693   74.07969    -2033.61562     1795.30103      254.89480          1
C    71.38540   77.96892   74.61683      523.79067      517.99274      284.45581          1
H    71.00920   78.92115   74.17841     1599.61665    -1127.00911     1895.98173          1
H    70.95614   77.92939   75.64198    -1881.83093     1275.82488    -1501.55016          1
C    72.94611   77.90869   74.63113     -600.05639     -404.05026      316.64485          1
H    73.40030   78.75328   74.06423    -2597.05260      496.36221      657.73249          1
H    73.33816   77.99755   75.66926    -1666.24518     1294.38985    -1723.90591          1
C    73.37074   76.51422   74.00682      493.57570     -129.95376     -602.56394          1
H    73.87389   76.59226   73.01297    -1469.08415     -637.84797     2204.22581          1
H    73.97218   75.85673   74.68062    -1038.97840     1068.88701    -2280.71789          1
C    70.79303   77.44728   72.27987      188.88100      241.05089     -727.87821          1
O    71.71658   78.01700   71.74281      531.88613      -49.19943      427.07960          1
N    69.52019   77.36035   71.91548      708.14833       26.93417     -179.73509          1
H    68.88345   76.85410   72.52997    -2232.69501    -1179.09831     1023.94816          1
C    69.02156   78.05983   70.78405     -738.32412      259.92548      104.42456          1
H    67.96111   77.73276   70.70501     2028.89123     1260.02983     1311.33324          1
C    69.11408   79.61084   71.14420     -258.65678     -345.52513      661.28459          1
H    70.16206   79.86214   71.40987    -1826.06090    -1801.46097     -918.64554          1
C    68.58711   80.50544   69.97274     -541.23400      531.92970     -218.41428          1
H    68.55525   81.58369   70.25952     1311.73218    -2219.18200      882.19023          1
H    69.23462   80.42208   69.06420    -1048.71298     1223.58118    -2196.97971          1
H    67.55451   80.21331   69.66912      419.06414     1903.92898     1903.44670          1
C    68.16791   79.91871   72.35423     -423.86431      152.66566     -648.55262          1
H    68.20322   80.99669   72.63932      105.68386    -2342.50080     1387.51238          1
H    67.10628   79.67415   72.11570    -1695.63603    -1484.73621    -1531.02793          1
```

H	68.45221	79.32602	73.25504	-1853.57121	-487.39031	1936.59343	1
C	69.88921	77.69581	69.45365	539.71428	-510.58232	267.58633	1
O	69.74764	78.38310	68.44152	-15.93758	-278.34085	-624.49402	1
N	70.61535	76.61602	69.62477	-676.81886	12.05292	276.17960	1
H	70.57363	76.24545	70.57653	-1694.44250	-373.84189	-2100.66098	1
C	71.42557	75.98295	68.65086	412.33078	-664.70360	108.35451	1
H	71.75315	75.04635	69.15604	1765.77476	1294.44366	1621.76594	1
C	70.49245	75.73334	67.39511	-179.66635	-721.95667	-264.73915	1
H	69.56818	75.19035	67.69561	-607.34922	2587.87032	-598.10018	1
H	70.14243	76.69797	66.95905	-359.69332	-1479.13954	-2259.74260	1
C	71.32639	74.90752	66.36241	-240.01076	616.02425	431.85322	1
H	72.19917	75.49699	66.00127	-1359.17131	782.54425	2227.99181	1
H	71.74824	73.99230	66.83584	-820.36667	85.81767	-2596.78715	1
C	70.36224	74.52428	65.16830	-513.07015	236.18570	-551.87280	1
H	69.50870	73.90475	65.52837	1816.11944	-1183.11722	-1650.94321	1
H	69.90674	75.43346	64.71264	-1800.04429	-1361.53473	1526.34643	1
C	71.21109	73.74912	64.13417	726.08972	-253.19732	-179.64713	1
H	72.00300	74.35080	63.64520	1224.27271	-456.51798	-2390.90304	1
H	71.63712	72.79698	64.50894	1206.40339	-1150.93514	-2154.90344	1
N	70.25634	73.32173	62.96937	329.70796	-633.68968	-155.67358	1
H	70.71501	72.79590	62.17049	1348.31305	1938.76845	1359.00643	1
H	69.45910	72.66665	63.21836	2096.92035	1446.54551	-966.48890	1
H	69.75013	74.09545	62.44799	-1197.07217	-2303.53719	827.28733	1
C	72.71405	76.91166	68.21998	-378.01186	-556.17301	-413.96481	1
O	73.82097	76.44123	68.30039	-409.44433	-307.55053	-453.30833	1
N	72.34307	78.10154	67.77192	-429.02477	524.70884	274.08502	1
H	71.33044	78.38416	67.77467	1706.34263	-1425.96036	1574.38926	1
C	73.34917	78.92465	67.18790	-45.25864	-626.22952	478.93267	1
H	74.15943	78.21478	66.89740	1988.63164	570.90773	1772.87287	1
C	72.65111	79.67227	65.97834	551.77128	-20.49526	-564.54996	1
H	72.13564	78.94324	65.31366	1434.61535	-1753.10253	1513.99471	1
H	71.84815	80.34387	66.35714	-714.67738	1819.09770	1898.36494	1
C	73.71729	80.49121	65.17969	446.99263	-555.77305	338.97221	1
H	74.26679	81.18450	65.86482	1953.68466	1892.25957	-161.67766	1
H	74.48929	79.82188	64.73739	-403.03785	2688.07032	-188.43591	1
C	72.95731	81.30442	64.05499	567.86262	335.74681	434.04421	1
H	72.53009	80.61907	63.28734	-1080.02275	1568.26081	-1948.78900	1
H	72.08947	81.85640	64.48369	-919.54108	2111.34692	1456.13463	1
C	73.96367	82.29227	63.42148	195.81897	173.33862	-745.11536	1
H	74.32812	83.08119	64.10950	1405.67949	-2301.21089	-390.08113	1
H	74.82042	81.81408	62.90645	-940.37328	-1118.32446	2299.72133	1
N	73.21064	83.07666	62.29356	319.26422	656.36007	-42.02424	1
H	73.78157	83.81542	61.79015	1961.03633	619.91968	1787.09422	1
H	72.83640	82.51122	61.47699	854.14343	-2156.80752	1429.08711	1
H	72.35654	83.63647	62.58299	1237.02905	-1378.50857	-1998.28500	1
C	73.88122	80.03611	68.26802	532.53319	-268.57236	517.55729	1
O	74.67565	80.88208	67.91383	266.16714	-290.73804	-558.88002	1
N	73.34900	79.87347	69.47064	-226.25352	640.49678	-270.32839	1
H	72.68933	79.10545	69.59282	2007.69063	-1035.27229	1523.50231	1
C	73.74752	80.77243	70.50209	-540.76542	-573.33303	49.50000	1
H	74.16713	81.66234	69.97616	-2374.20950	-1036.76023	843.75774	1
C	72.41108	81.08031	71.33635	-77.09082	666.87422	-415.84136	1
H	71.87749	80.09116	71.44884	-985.81834	617.19825	2463.91927	1
O	71.53002	82.01230	70.68531	293.29899	-391.65602	-477.80926	1
H	70.80370	81.42297	70.40990	2528.72714	-681.76262	-751.27122	1
C	72.92685	81.51832	72.75341	252.47363	-605.14473	440.05134	1
H	72.08300	81.70369	73.45830	-2052.65063	1456.49051	-1043.51830	1
H	73.57787	80.73757	73.22018	-2152.98800	-1661.97574	-161.70652	1
H	73.52822	82.45557	72.69779	-473.12726	-1584.27339	2165.61725	1
C	74.86949	80.07385	71.45459	360.36307	488.46713	505.10056	1
O	75.57888	80.82644	72.12361	-200.08817	-528.03552	-385.82850	1
N	74.79755	78.76193	71.46687	-597.54095	-226.44775	355.20479	1

198	H	74.06947	78.38963	70.85649	2179.23655	-1300.70981	-991.33835	1
199	C	75.83063	77.91602	71.93903	-687.68921	297.39807	-249.45179	1
200	H	75.58061	76.92808	71.49344	-349.16256	-2467.37713	-1101.72831	1
201	C	77.25755	78.47564	71.47390	672.47345	361.54328	-201.62880	1
202	H	77.38239	79.53094	71.81491	1541.41502	582.18425	2169.97026	1
203	H	78.06272	77.87548	71.95684	-2534.67717	60.25153	997.72295	1
204	C	77.37114	78.38723	69.94594	574.26774	-389.43119	-377.02542	1
205	C	77.70162	77.14729	69.32039	688.82679	-287.33580	-257.96133	1
206	H	77.85305	76.23394	69.92391	331.14182	1470.38163	2269.80013	1
207	C	77.14798	79.55382	69.15435	-383.52382	-675.04808	144.24917	1
208	H	76.85510	80.50836	69.62910	4.09847	1517.59662	2262.86397	1
209	C	77.83459	77.11243	67.93230	-134.76730	428.45285	-649.50303	1
210	H	78.09563	76.15373	67.45103	-903.23433	1600.49020	2011.53387	1
211	C	77.29594	79.46867	67.76845	-335.53281	-615.00040	-364.39058	1
212	H	77.11709	80.38067	67.17202	1596.45398	-1369.33862	1732.02659	1
213	C	77.65069	78.25834	67.10468	585.48547	-340.24194	-406.23938	1
214	O	77.81047	78.16987	65.69726	-419.15649	-173.04408	-511.94141	1
215	H	77.64441	79.11096	65.45163	-1071.84350	1559.71507	-1960.13012	1
216	C	75.88467	77.85771	73.56784	291.41878	-183.13091	710.72246	1
217	O	75.97234	76.76351	74.09517	379.87695	523.47396	-222.23622	1
218	N	75.87354	79.04374	74.14155	564.30591	373.09380	277.24371	1
219	H	75.83057	79.91853	73.56084	-120.59931	-2436.07824	-1214.35098	1
220	C	75.83446	79.09797	75.56157	404.07514	-299.37480	608.84009	1
221	H	76.00983	78.05947	75.92751	-1071.07882	-841.69025	2359.66467	1
222	C	76.93808	80.16183	75.98357	-683.12807	68.71770	-390.13096	1
223	H	77.90773	79.94887	75.48075	2068.88918	1596.58590	770.89603	1
224	H	76.64084	81.18111	75.64821	1379.86429	2342.49779	-179.85638	1
225	C	77.08114	80.10175	77.53322	-176.39967	537.49466	550.97268	1
226	H	76.09206	80.23980	78.02940	-396.28051	1942.22168	1869.33314	1
227	H	77.45186	79.10742	77.86847	-94.44700	586.00678	-2659.20016	1
228	C	78.09297	81.27400	77.93636	-291.69381	-718.18079	-150.72929	1
229	H	79.11973	81.21903	77.49435	-2303.38776	1089.76242	964.62032	1
230	H	77.68095	82.30165	77.77233	884.16729	2568.80533	-207.74283	1
231	N	78.18336	81.11950	79.32467	-594.49522	-252.81478	342.29276	1
232	H	77.40918	80.64378	79.76363	-1735.17426	2084.05705	-263.71605	1
233	C	79.11578	81.58042	80.05657	595.23177	-271.52487	442.22352	1
234	N	80.15583	82.27538	79.43137	585.53813	-424.79750	-105.81303	1
235	H	80.25687	82.53960	78.45232	959.88797	1327.56823	-2177.11905	1
236	H	80.92534	82.73507	79.91881	1578.48647	16.27378	-2220.76233	1
237	N	78.88255	81.27161	81.40274	-452.17763	-390.33541	-421.51853	1
238	H	78.09483	80.80263	81.84766	470.26107	1974.25629	-1817.92087	1
239	H	79.48722	81.58521	82.16283	1856.67307	-1573.97321	-1224.35416	1
240	C	74.41198	79.75496	76.04130	-531.47271	-581.45780	-55.06206	1
241	O	73.90849	80.72138	75.47679	-243.00837	192.73507	609.52485	1
242	N	73.96096	79.08508	77.08034	343.49288	-644.25607	38.09164	1
243	H	74.53285	78.33551	77.46276	-1691.16043	-1535.97525	-1484.73099	1
244	C	72.62369	79.33878	77.51603	542.66492	350.14337	454.42563	1
245	H	72.09497	79.57640	76.56554	1209.57979	-2396.95920	463.86744	1
246	C	72.04817	78.11641	78.34092	-737.14309	145.93010	242.71942	1
247	H	72.62715	78.01437	79.28401	-1050.18303	159.36419	-2509.06125	1
248	C	70.54726	78.46334	78.63809	-706.77696	-252.88343	-245.16230	1
249	H	70.03553	77.64276	79.19291	-1624.64039	-1665.49085	1417.86935	1
250	H	70.46283	79.38827	79.25968	2566.10521	598.51109	693.22305	1
251	H	69.96993	78.64014	77.70036	-1972.69758	107.10018	-1876.34312	1
252	C	72.04668	76.74151	77.58559	412.85148	672.58832	-27.69222	1
253	H	71.80724	75.90013	78.27699	1447.02277	1762.76533	-1490.78944	1
254	H	71.28664	76.70938	76.77028	1320.77379	-145.20455	2378.68491	1
255	H	73.03547	76.51862	77.12297	-1512.10848	1470.52031	1724.75147	1
256	C	72.65989	80.64166	78.51889	-607.11113	499.56689	73.73769	1
257	O	72.39779	80.52662	79.69026	357.97126	460.81534	356.68785	1
258	N	72.87270	81.71620	77.77655	536.55407	-368.62270	332.76306	1
259	H	73.07634	81.58747	76.75523	524.38866	2297.69985	1367.20941	1

```
C    73.16286   82.96912   78.36959        46.50277      -774.29190      -147.97904           1
H    72.99766   83.68396   77.53433      1676.51875      1883.29066      1032.55549           1
C    72.23148   83.24251   79.65450      -559.41456       -34.19198      -556.30433           1
H    72.48461   82.49392   80.45227       -64.66664       395.18769      2695.05373           1
H    72.45263   84.24625   80.09192     -2703.44278       -60.40056       333.79418           1
C    70.77677   83.12327   79.27916      -519.76435       377.31045       459.42366           1
H    70.64322   82.16869   78.72119     -1746.74323      1667.50644     -1261.73691           1
C    69.91396   83.06803   80.53377       570.95554        16.46261      -545.27700           1
H    68.82740   82.95305   80.28566      2391.58273        28.05277     -1305.07271           1
H    70.19886   82.20488   81.18983       175.75588       917.63458      2559.43867           1
H    70.01761   83.99993   81.14718     -1063.97851     -2155.34650      1283.00462           1
C    70.32777   84.27596   78.39248       193.76014         2.62946      -765.53141           1
H    69.24189   84.19408   78.12773       507.39394      -846.55087      2539.60089           1
H    70.47130   85.26539   78.89844      1337.57841      1818.19054      1526.01994           1
H    70.90192   84.30737   77.43093      1100.88635     -1172.91350     -2199.08909           1
C    74.70442   82.98316   78.85400      -549.21115       475.64486      -309.38218           1
O    75.31162   82.01785   79.23644       556.06106      -390.37755       -78.24250           1
O    75.29615   84.26831   78.91178      -572.27940      -310.19032      -209.75862           1
H    74.52408   84.80659   78.58213     -1380.08193      2212.14486       790.85944           1
```

Listing 74: C00074.xmolout

```
       276
   150.0000  150.0000  150.0000     0.0000     0.0000     0.0000***********    2.7750    11100
        0.2500     300.00    0.00    0.00    0.00  -1293.5562       0.0000     300.0000
H    79.43042   79.80407   82.18581     -2043.14101     -1757.64893       399.89339           1
N    80.26650   79.38661   81.78143       426.75047       490.45733      -334.42467           1
H    79.90413   78.93429   80.94196       442.25559     -1110.30854     -2448.52907           1
C    81.30797   80.29430   81.49116       415.32972      -666.39213        83.73172           1
H    81.24935   81.12174   82.23039       879.32219      2491.00447       667.35213           1
C    82.69400   79.47212   81.55588        31.35357       477.41777       628.23362           1
H    82.68350   78.67332   80.77600      1168.38489      2240.25337     -1019.71136           1
H    83.55386   80.13860   81.30431      -360.43770      2534.90498       931.67112           1
C    82.85990   78.86376   82.92471       463.18208       512.70242      -382.34388           1
H    81.94590   78.26892   83.15119      1725.29117      1863.52247      -987.07854           1
C    84.06452   77.93187   82.95511       710.42801      -144.27427      -313.15367           1
H    84.19266   77.45453   83.96106       364.20195     -1033.13725      2494.72524           1
H    83.96934   77.10468   82.20501      2567.61904       335.80679       847.48802           1
H    85.01480   78.47993   82.72595     -2587.99029       680.92867      -512.16411           1
C    83.01880   79.93348   83.99628       268.17616       741.64379       -40.42634           1
H    83.19137   79.48242   85.00772      1283.54314     -1945.37420     -1411.27812           1
H    83.88771   80.60752   83.78029     -1258.57796      1317.15249     -2026.02085           1
H    82.10685   80.57972   84.07428     -2502.84526       909.80597       575.92391           1
C    81.05263   80.74878   79.94941       619.36578      -458.64843      -172.09273           1
O    80.68275   79.92695   79.10840       -81.40433       433.87611       522.34594           1
N    81.30237   82.03184   79.82100       175.23996      -622.20190       341.55523           1
H    81.62246   82.51181   80.66221      -876.57256      -303.22879     -2561.90213           1
C    81.24324   82.74736   78.60124      -571.88214      -534.10783      -106.15033           1
H    81.34104   83.79476   78.96188     -2713.20841       -16.25800      -248.80809           1
C    82.50487   82.29904   77.73648       166.60354       288.16399      -716.09568           1
H    82.39760   81.20911   77.48762     -2097.85574      1636.78449      -586.18044           1
O    83.77645   82.44873   78.40333      -317.80580      -363.38227       484.43082           1
H    84.07392   81.52105   78.45321      1863.74748     -1451.84923      1357.29467           1
C    82.34209   83.16092   76.43103       503.80731       517.99032       318.51606           1
H    83.18156   82.98169   75.71968       640.61020     -2157.10410     -1536.29104           1
H    81.39201   82.92384   75.88838      1687.88455       465.60692      2087.56452           1
H    82.33234   84.25228   76.65960     -1293.07895     -1899.88581     -1463.57452           1
C    79.81008   82.48902   77.82833      -153.07550      -733.68902       248.70981           1
O    79.75397   81.86289   76.74434       186.15459      -480.78787      -449.34387           1
N    78.85080   83.06507   78.49313      -487.53139       543.18517        42.03850           1
C    77.06835   80.51926   77.75918      -699.35871        69.34393       360.10701           1
H    76.93050   79.44883   77.49281         2.81126      -221.99807     -2715.58072           1
```

C	76.54539	80.74655	79.21764	-507.46439	-592.59194	122.07761	1
H	75.44570	80.56559	79.25980	-1975.08116	525.87560	-1801.71556	1
H	77.01233	80.01071	79.91370	1588.27743	-1913.22100	1113.83566	1
C	76.87831	82.20063	79.66767	-613.62442	-457.55993	-194.14490	1
H	76.40520	82.94273	78.98951	1707.54637	-721.27992	1996.92523	1
H	76.45718	82.38694	80.67820	758.23357	2100.81767	1560.55011	1
C	78.49328	82.39621	79.68196	-352.41524	704.96076	49.21465	1
H	78.82778	83.09133	80.49635	1714.76886	-2070.42778	443.35792	1
H	79.08108	81.43401	79.72575	1224.15040	1801.50881	1636.97633	1
C	76.29413	81.43799	76.71439	566.85117	537.05831	117.62814	1
O	75.09111	81.56553	76.69783	-550.26779	-406.08867	4.21666	1
N	77.19850	81.94790	75.85510	566.34832	-425.77725	180.18749	1
H	78.23677	81.78047	75.96937	644.44502	2646.80550	-52.73430	1
C	76.67762	82.83065	74.85258	612.34801	-281.48662	411.56221	1
H	75.76246	83.26215	75.31983	1667.23498	-1490.75142	1556.16753	1
C	77.83089	83.89382	74.61047	-714.45746	-175.43074	286.98927	1
H	78.73767	83.33583	74.24823	-787.09484	1763.03619	-1922.46060	1
O	78.30242	84.56060	75.82271	-429.46514	516.51932	-128.40526	1
H	78.62776	83.79217	76.32721	1981.82785	436.16847	-1818.18182	1
C	77.31108	84.86636	73.49788	630.79189	-468.94448	76.03230	1
H	78.06113	85.66060	73.27143	-1775.71861	2062.30353	131.89849	1
H	77.09284	84.33551	72.53680	-1855.14608	88.94118	1993.53760	1
H	76.37289	85.38048	73.81488	-1504.82350	-780.94249	-2132.90992	1
C	76.29197	81.99315	73.50599	288.57638	618.62814	-397.00270	1
O	75.81011	82.62093	72.55137	321.70495	-185.26452	574.37218	1
N	76.52947	80.71281	73.60509	-447.05792	-446.53431	367.76425	1
H	76.91472	80.49758	74.52308	350.31316	1975.63644	-1843.31522	1
C	76.43218	79.71433	72.60070	-511.23061	374.03917	-471.51480	1
H	76.04569	78.83578	73.16232	2234.79579	-898.70890	-1273.45190	1
C	75.44795	80.16074	71.42590	529.53781	-159.34528	563.72604	1
H	75.17531	79.23540	70.82834	654.59388	2347.94251	1217.51488	1
H	74.51249	80.51553	71.93866	-1526.55661	2103.42026	817.87394	1
O	75.91286	81.13359	70.48768	-554.84278	14.12789	399.58769	1
H	75.82994	81.97590	71.08579	1297.06236	2061.84854	1220.68812	1
C	77.95192	79.40692	72.09031	-662.15484	-412.13544	123.62687	1
O	78.89301	80.16105	72.19530	-540.01537	-317.05804	274.91456	1
N	77.92273	78.17943	71.57869	-202.76299	-690.00100	-131.49145	1
H	77.03790	77.67291	71.57259	-702.62132	-2617.37191	281.70413	1
C	79.10642	77.62388	71.02613	-491.73914	576.34187	222.73509	1
H	79.83715	78.46675	71.03065	1977.17978	-1823.58850	-434.68928	1
C	79.54173	76.45252	72.01980	-649.50797	-328.66843	306.11224	1
H	78.67978	75.78091	72.22341	2075.07803	1452.16490	-1004.45904	1
C	80.77566	75.63731	71.50912	-615.18735	-489.99689	-70.96352	1
H	81.13509	74.91489	72.28016	-1226.32661	-1724.38906	-1716.47170	1
H	80.53097	75.04636	70.59201	2381.64273	-969.84735	900.46860	1
H	81.63082	76.30695	71.25414	2122.74991	1702.23946	-141.36228	1
C	80.03826	77.14824	73.33208	-501.86074	-479.61276	-376.42482	1
H	80.21523	76.41099	74.15028	993.79261	-2246.95401	-1177.81305	1
H	80.99191	77.70255	73.16845	42.71279	-626.15391	-2651.37258	1
H	79.28923	77.89104	73.69766	-2026.39012	-1590.33709	-887.82922	1
C	78.81158	77.02559	69.53595	-672.89153	382.57394	-156.34072	1
O	79.62884	76.31261	69.00544	410.04627	221.86223	500.35950	1
N	77.66179	77.44622	69.04478	-378.11782	-443.79414	-441.10984	1
H	77.00756	78.07135	69.56210	2404.15973	-568.14894	1149.30085	1
C	77.18030	76.98414	67.76648	-544.54484	466.16019	331.29119	1
H	77.92868	77.32845	67.01713	-1409.80988	-170.17955	2325.32679	1
C	77.15815	75.39618	67.85892	-184.48284	116.78282	-758.89137	1
H	77.67365	75.06778	68.79160	1893.78641	1861.14652	611.04528	1
H	76.11430	75.01351	67.92538	-2170.79665	-974.19311	1327.50117	1
C	77.89149	74.81887	66.60978	389.51668	672.81878	-138.49152	1
H	77.47739	75.23835	65.66827	510.64577	1933.03327	1851.02484	1
H	78.95131	75.15901	66.61323	-2302.30869	-1105.72939	-948.89774	1

C	77.84049	73.24458	66.53773	-559.93427	-126.34590	542.30857	1
O	78.18821	72.68599	65.52973	-670.36003	-111.83166	76.36448	1
N	77.42040	72.65355	67.67654	-216.06729	-537.07037	446.51379	1
H	77.14521	73.07308	68.57239	-1251.96487	1074.12894	-2168.52500	1
H	77.37159	71.63776	67.81054	-2451.83023	-720.73851	944.84612	1
C	75.77690	77.75995	67.72802	546.24052	-563.88337	-85.11809	1
O	75.44220	78.38934	68.72002	71.70702	-307.40463	606.69673	1
N	75.13017	77.63809	66.56614	479.90863	-504.50129	222.86849	1
C	73.95904	78.41927	66.30095	75.51268	-600.91904	506.73700	1
H	74.13515	79.40982	66.77747	-2033.61562	1795.30103	254.89480	1
C	73.91207	78.39797	64.71902	523.79067	517.99274	284.45581	1
H	72.90353	78.63553	64.31204	1599.61665	-1127.00911	1895.98173	1
H	74.61915	79.13564	64.27770	-1881.83093	1275.82488	-1501.55016	1
C	74.36424	76.93145	64.42792	-600.05639	-404.05026	316.64485	1
H	73.52768	76.21927	64.62112	-2597.05260	496.36221	657.73249	1
H	74.69384	76.76308	63.37784	-1666.24518	1294.38985	-1723.90591	1
C	75.52875	76.73941	65.48829	493.57570	-129.95376	-602.56394	1
H	75.60717	75.71403	65.91743	-1469.08415	-637.84797	2204.22581	1
H	76.53428	77.08395	65.15027	-1038.97840	1068.88701	-2280.71789	1
C	72.66966	77.62325	66.95393	188.88100	241.05089	-727.87821	1
O	72.13340	76.72780	66.34125	531.88613	-49.19943	427.07960	1
N	72.34540	78.18904	68.10020	708.14833	26.93417	-179.73509	1
H	72.96117	78.93401	68.42677	-2232.69501	-1179.09831	1023.94816	1
C	71.33641	77.69216	68.96693	-738.32412	259.92548	104.42456	1
H	70.96398	78.59473	69.49912	2028.89123	1260.02983	1311.33324	1
C	70.23188	76.90271	68.14903	-258.65678	-345.52513	661.28459	1
H	70.68010	75.99801	67.66550	-1826.06090	-1801.46097	-918.64554	1
C	69.10860	76.49546	69.16527	-541.23400	531.92970	-218.41428	1
H	68.29752	75.91284	68.66962	1311.73218	-2219.18200	882.19023	1
H	69.51541	75.86389	69.99001	-1048.71298	1223.58118	-2196.97971	1
H	68.63681	77.38966	69.63514	419.06414	1903.92898	1903.44670	1
C	69.57763	77.82878	67.06541	-423.86431	152.66566	-648.55262	1
H	68.74892	77.31043	66.52832	105.68386	-2342.50080	1387.51238	1
H	69.15716	78.75866	67.51462	-1695.63603	-1484.73621	-1531.02793	1
H	70.33041	78.13388	66.29841	-1853.57121	-487.39031	1936.59343	1
C	72.00790	76.59866	70.00539	539.71428	-510.58232	267.58633	1
O	72.64298	75.63700	69.64098	-15.93758	-278.34085	-624.49402	1
N	71.55371	76.90941	71.21036	-676.81886	12.05292	276.17960	1
H	70.98494	77.75101	71.28704	-1694.44250	-373.84189	-2100.66098	1
C	72.02030	76.26582	72.38890	412.33078	-664.70360	108.35451	1
H	71.37336	76.68868	73.19064	1765.77476	1294.44366	1621.76594	1
C	71.79281	74.70857	72.17575	-179.66635	-721.95667	-264.73915	1
H	70.73091	74.52223	71.89901	-607.34922	2587.87032	-598.10018	1
H	72.39247	74.34745	71.30251	-359.69332	-1479.13954	-2259.74260	1
C	72.16465	73.91003	73.46725	-240.01076	616.02425	431.85322	1
H	73.27325	73.89262	73.59322	-1359.17131	782.54425	2227.99181	1
H	71.74509	74.41537	74.36697	-820.36667	85.81767	-2596.78715	1
C	71.65127	72.41467	73.42152	-513.07015	236.18570	-551.87280	1
H	70.53993	72.35428	73.35714	1816.11944	-1183.11722	-1650.94321	1
H	72.04564	71.87142	72.53234	-1800.04429	-1361.53473	1526.34643	1
C	72.18750	71.81370	74.74443	726.08972	-253.19732	-179.64713	1
H	73.28480	71.90295	74.88322	1224.27271	-456.51798	-2390.90304	1
H	71.66719	72.16697	75.66060	1206.40339	-1150.93514	-2154.90344	1
N	71.93043	70.27576	74.73034	329.70796	-633.68968	-155.67358	1
H	72.18556	69.82149	75.65429	1348.31305	1938.76845	1359.00643	1
H	70.93241	69.93894	74.60157	2096.92035	1446.54551	-966.48890	1
H	72.46661	69.67867	74.03520	-1197.07217	-2303.53719	827.28733	1
C	73.58362	76.68050	72.61243	-378.01186	-556.17301	-413.96481	1
O	74.18032	77.51396	71.97136	-409.44433	-307.55053	-453.30833	1
N	74.00891	76.04052	73.70299	-429.02477	524.70884	274.08502	1
H	73.35860	75.45708	74.22802	1706.34263	-1425.96036	1574.38926	1
C	75.41730	75.79428	73.78889	-45.25864	-626.22952	478.93267	1

H	75.81598	76.16465	72.81639	1988.63164	570.90773	1772.87287	1
C	75.85802	76.68985	75.01129	551.77128	-20.49526	-564.54996	1
H	75.39713	77.69996	74.93423	1434.61535	-1753.10253	1513.99471	1
H	75.49378	76.27732	75.97719	-714.67738	1819.09770	1898.36494	1
C	77.42502	76.78416	74.98435	446.99263	-555.77305	338.97221	1
H	77.89726	75.78633	75.13198	1953.68466	1892.25957	-161.67766	1
H	77.78655	77.16821	74.00211	-403.03785	2688.07032	-188.43591	1
C	77.75285	77.76886	76.15295	567.86262	335.74681	434.04421	1
H	77.25562	78.75057	75.98288	-1080.02275	1568.26081	-1948.78900	1
H	77.33201	77.38470	77.10784	-919.54108	2111.34692	1456.13463	1
C	79.33053	77.97237	76.29762	195.81897	173.33862	-745.11536	1
H	79.89077	77.01871	76.46130	1405.67949	-2301.21089	-390.08113	1
H	79.78998	78.55658	75.46321	-940.37328	-1118.32446	2299.72133	1
N	79.47842	78.76340	77.48120	319.26422	656.36007	-42.02424	1
H	79.89185	79.70482	77.39253	1961.03633	619.91968	1787.09422	1
H	78.16439	80.70846	77.69985	854.14343	-2156.80752	1429.08711	1
H	79.99288	78.35878	78.27457	1237.02905	-1378.50857	-1998.28500	1
C	75.66447	74.18550	74.02522	532.53319	-268.57236	517.55729	1
O	75.88616	73.51310	73.05142	266.16714	-290.73804	-558.88002	1
N	75.49742	73.80767	75.28247	-226.25352	640.49678	-270.32839	1
H	75.17331	74.42952	76.03671	2007.69063	-1035.27229	1523.50231	1
C	75.97865	72.54330	75.72102	-540.76542	-573.33303	49.50000	1
H	75.93360	71.82612	74.87149	-2374.20950	-1036.76023	843.75774	1
C	77.46711	72.75979	76.28225	-77.09082	666.87422	-415.84136	1
H	77.44663	73.62444	77.00158	-985.81834	617.19825	2463.91927	1
O	78.46709	73.09757	75.30597	293.29899	-391.65602	-477.80926	1
H	78.78206	73.95815	75.64799	2528.72714	-681.76262	-751.27122	1
C	77.74371	71.43835	77.08707	252.47363	-605.14473	440.05134	1
H	78.74362	71.46531	77.57908	-2052.65063	1456.49051	-1043.51830	1
H	76.98884	71.27331	77.89146	-2152.98800	-1661.97574	-161.70652	1
H	77.72849	70.54385	76.42216	-473.12726	-1584.27339	2165.61725	1
C	74.94936	72.30502	76.97250	360.36307	488.46713	505.10056	1
O	74.48816	73.31253	77.48467	-200.08817	-528.03552	-385.82850	1
N	74.83371	71.03004	77.28408	-597.54095	-226.44775	355.20479	1
H	75.36757	70.38184	76.70296	2179.23655	-1300.70981	-991.33835	1
C	74.13440	70.51678	78.39967	-687.68921	297.39807	-249.45179	1
H	74.62124	69.53908	78.60742	-349.16256	-2467.37713	-1101.72831	1
C	74.17392	71.55099	79.61779	672.47345	361.54328	-201.62880	1
H	73.79205	72.54949	79.27451	1541.41502	582.18425	2169.97026	1
H	73.48674	71.19640	80.42089	-2534.67717	60.25153	997.72295	1
C	75.60217	71.68081	80.16905	574.26774	-389.43119	-377.02542	1
C	76.14546	70.65776	81.00250	688.82679	-287.33580	-257.96133	1
H	75.55937	69.75101	81.23815	331.14182	1470.38163	2269.80013	1
C	76.37219	72.84328	79.86329	-383.52382	-675.04808	144.24917	1
H	75.95713	73.63082	79.20548	4.09847	1517.59662	2262.86397	1
C	77.42635	70.83403	81.52670	-134.76730	428.45285	-649.50303	1
H	77.84230	70.04843	82.18144	-903.23433	1600.49020	2011.53387	1
C	77.65033	72.97487	80.41176	-335.53281	-615.00040	-364.39058	1
H	78.23080	73.88364	80.17506	1596.45398	-1369.33862	1732.02659	1
C	78.22255	71.98668	81.26503	585.48547	-340.24194	-406.23938	1
O	79.51734	72.11236	81.83348	-419.15649	-173.04408	-511.94141	1
H	79.75950	73.00836	81.50053	-1071.84350	1559.71507	-1960.13012	1
C	72.55197	70.34502	78.00265	291.41878	-183.13091	710.72246	1
O	71.84181	71.29798	77.76591	379.87695	523.47396	-222.23622	1
N	72.22011	69.05698	78.02673	564.30591	373.09380	277.24371	1
H	72.92580	68.35742	78.25181	-120.59931	-2436.07824	-1214.35098	1
C	70.89992	68.73812	77.60985	404.07514	-299.37480	608.84009	1
H	70.47349	69.73237	77.32460	-1071.07882	-841.69025	2359.66467	1
C	71.01200	67.69837	76.41536	-683.12807	68.71770	-390.13096	1
H	71.86114	67.95367	75.74424	2068.88918	1596.58590	770.89603	1
H	71.22901	66.67160	76.78490	1379.86429	2342.49779	-179.85638	1
C	69.65569	67.73755	75.64728	-176.39967	537.49466	550.97268	1

```
H   68.84981  67.23302  76.22598     -396.28051     1942.22168     1869.33314          1
H   69.30608  68.78332  75.49821      -94.44700      586.00678    -2659.20016          1
C   69.92234  67.01912  74.24526     -291.69381     -718.18079     -150.72929          1
H   70.69904  67.50051  73.59743    -2303.38776     1089.76242      964.62032          1
H   70.15757  65.92595  74.30157      884.16729     2568.80533     -207.74283          1
N   68.68681  67.15985  73.59723     -594.49522     -252.81478      342.29276          1
H   67.95070  67.61392  74.11517    -1735.17426     2084.05705     -263.71605          1
C   68.44264  66.75335  72.41776      595.23177     -271.52487      442.22352          1
N   69.47904  66.12515  71.71808      585.53813     -424.79750     -105.81303          1
H   70.43307  65.92518  72.01570      959.88797     1327.56823    -2177.11905          1
H   69.40251  65.75205  70.77139     1578.48647       16.27378    -2220.76233          1
N   67.11970  67.03656  72.05030     -452.17763     -390.33541     -421.51853          1
H   66.36483  67.49282  72.56035      470.26107     1974.25629    -1817.92087          1
H   66.72954  66.78605  71.14086     1856.67307    -1573.97321    -1224.35416          1
C   70.02964  67.99167  78.79586     -531.47271     -581.45780      -55.06206          1
O   68.91640  68.41247  79.03078     -243.00837      192.73507      609.52485          1
N   70.60821  66.88405  79.25282      343.49288     -644.25607       38.09164          1
H   71.54945  66.81074  78.86762    -1691.16043    -1535.97525    -1484.73099          1
C   70.24203  66.22389  80.50873      542.66492      350.14337      454.42563          1
H   70.83862  65.28539  80.42705     1209.57979    -2396.95920      463.86744          1
C   70.68519  67.08545  81.67530     -737.14309      145.93010      242.71942          1
H   70.16317  68.06525  81.59408    -1050.18303      159.36419    -2509.06125          1
C   70.31440  66.45409  83.00502     -706.77696     -252.88343     -245.16230          1
H   70.68565  67.06088  83.87551    -1624.64039    -1665.49085     1417.86935          1
H   69.19291  66.35872  83.11518     2566.10521      598.51109      693.22305          1
H   70.74790  65.42159  83.10935    -1972.69758      107.10018    -1876.34312          1
C   72.18091  67.34844  81.64204      412.85148      672.58832      -27.69222          1
H   72.51389  67.97190  82.51596     1447.02277     1762.76533    -1490.78944          1
H   72.76773  66.38978  81.67582     1320.77379     -145.20455     2378.68491          1
H   72.48411  67.89293  80.70640    -1512.10848     1470.52031     1724.75147          1
C   68.61173  65.92091  80.53057     -607.11113      499.56689       73.73769          1
O   67.90969  66.29394  81.44454      357.97126      460.81534      356.68785          1
N   68.27252  65.17110  79.49534      536.55407     -368.62270      332.76306          1
H   69.00027  64.94679  78.81941      524.38866     2297.69985     1367.20941          1
C   66.93590  64.71700  79.39555       46.50277     -774.29190     -147.97904          1
H   66.47218  64.95166  80.38168     1676.51875     1883.29066     1032.55549          1
C   66.22111  65.48199  78.16284     -559.41456      -34.19198     -556.30433          1
H   66.74635  65.22309  77.21296      -64.66664      395.18769     2695.05373          1
H   65.17101  65.12458  78.03994    -2703.44278      -60.40056      333.79418          1
C   66.26252  66.96670  78.39835     -519.76435      377.31045      459.42366          1
H   67.32848  67.27922  78.59047    -1746.74323     1667.50644    -1261.73691          1
C   65.76057  67.70561  77.16446      570.95554       16.46261     -545.27700          1
H   65.81481  68.81666  77.29980     2391.58273       28.05277    -1305.07271          1
H   66.36642  67.45205  76.25626      175.75588      917.63458     2559.43867          1
H   64.69441  67.44914  76.93425    -1063.97851    -2155.34650     1283.00462          1
C   65.44431  67.36168  79.61912      193.76014        2.62946     -765.53141          1
H   65.37595  68.47541  79.72562      507.39394     -846.55087     2539.60089          1
H   64.39703  66.96564  79.56678     1337.57841     1818.19054     1526.01994          1
H   65.91162  66.97069  80.56457     1100.88635    -1172.91350    -2199.08909          1
C   66.97030  63.14873  79.01302     -549.21115      475.64486     -309.38218          1
O   67.91458  62.60906  78.49949      556.06106     -390.37755      -78.24250          1
O   65.75429  62.44972  79.20411     -572.27940     -310.19032     -209.75862          1
H   65.22067  63.18042  79.62310    -1380.08193     2212.14486      790.85944          1
```

Listing 75: C00075.xmolout

```
      276
  150.0000  150.0000  150.0000    0.0000    0.0000    0.0000**********    2.7750    11100
      0.2500    300.00   0.00   0.00   0.00  -1293.5493      0.0000    300.0000
H   64.05977  78.49321  79.57454    -2043.14101    -1757.64893      399.89339          1
N   64.19204  78.57048  78.56809      426.75047      490.45733     -334.42467          1
H   63.23825  78.74533  78.25760      442.25559    -1110.30854    -2448.52907          1
```

C	65.10950	79.58213	78.19322	415.32972	-666.39213	83.73172	1
H	65.94767	79.39620	78.89930	879.32219	2491.00447	667.35213	1
C	65.54297	79.37197	76.65398	31.35357	477.41777	628.23362	1
H	64.67813	79.60721	75.98862	1168.38489	2240.25337	-1019.71136	1
H	66.35249	80.08583	76.36953	-360.43770	2534.90498	931.67112	1
C	65.99447	77.95149	76.44319	463.18208	512.70242	-382.34388	1
H	65.19541	77.27202	76.81777	1725.29117	1863.52247	-987.07854	1
C	66.20926	77.68478	74.95826	710.42801	-144.27427	-313.15367	1
H	66.53579	76.62964	74.76967	364.20195	-1033.13725	2494.72524	1
H	65.27229	77.85348	74.36731	2567.61904	335.80679	847.48802	1
H	66.99562	78.35869	74.52919	-2587.99029	680.92867	-512.16411	1
C	67.27890	77.65386	77.20513	268.17616	741.64379	-40.42634	1
H	67.63637	76.60837	77.01873	1283.54314	-1945.37420	-1411.27812	1
H	68.10680	78.34727	76.90615	-1258.57796	1317.15249	-2026.02085	1
H	67.13736	77.76138	78.31123	-2502.84526	909.80597	575.92391	1
C	64.36599	80.97893	78.50354	619.36578	-458.64843	-172.09273	1
O	63.66861	81.10847	79.51751	-81.40433	433.87611	522.34594	1
N	64.75409	81.91078	77.66518	175.23996	-622.20190	341.55523	1
H	65.34990	81.60214	76.89866	-876.57256	-303.22879	-2561.90213	1
C	63.96541	83.09142	77.42217	-571.88214	-534.10783	-106.15033	1
H	63.14439	82.74984	76.75381	-2713.20841	-16.25800	-248.80809	1
C	63.37291	83.63369	78.80279	166.60354	288.16399	-716.09568	1
H	64.10601	83.34400	79.61126	-2097.85574	1636.78449	-586.18044	1
O	62.13524	83.04931	79.21079	-317.80580	-363.38227	484.43082	1
H	62.49864	82.20776	79.69148	1863.74748	-1451.84923	1357.29467	1
C	63.35412	85.19314	78.61040	503.80731	517.99032	318.51606	1
H	63.04068	85.71598	79.54366	640.61020	-2157.10410	-1536.29104	1
H	64.35345	85.59481	78.32212	1687.88455	465.60692	2087.56452	1
H	62.63844	85.48982	77.80822	-1293.07895	-1899.88581	-1463.57452	1
C	64.94496	84.11118	76.62942	-153.07550	-733.68902	248.70981	1
O	64.41881	84.72306	75.73612	186.15459	-480.78787	-449.34387	1
N	66.18941	84.25087	77.11961	-487.53139	543.18517	42.03850	1
C	67.17076	84.98667	76.39111	-699.35871	69.34393	360.10701	1
H	66.62624	85.72994	75.76359	2.81126	-221.99807	-2715.58072	1
C	68.06986	85.57141	77.56268	-507.46439	-592.59194	122.07761	1
H	69.07950	85.88674	77.21561	-1975.08116	525.87560	-1801.71556	1
H	67.59917	86.46274	78.03464	1588.27743	-1913.22100	1113.83566	1
C	68.09767	84.35796	78.54346	-613.62442	-457.55993	-194.14490	1
H	68.78134	83.55386	78.17593	1707.54637	-721.27992	1996.92523	1
H	68.43714	84.62042	79.57066	758.23357	2100.81767	1560.55011	1
C	66.57579	83.90702	78.48404	-352.41524	704.96076	49.21465	1
H	66.39854	82.81738	78.63668	1714.76886	-2070.42778	443.35792	1
H	65.89481	84.48168	79.15343	1224.15040	1801.50881	1636.97633	1
C	68.14009	83.98951	75.50739	566.85117	537.05831	117.62814	1
O	68.92882	84.47716	74.73263	-550.26779	-406.08867	4.21666	1
N	67.93219	82.71271	75.80683	566.34832	-425.77725	180.18749	1
H	67.20051	82.56279	76.49934	644.44502	2646.80550	-52.73430	1
C	68.91440	81.71185	75.55604	612.34801	-281.48662	411.56221	1
H	68.43071	80.79271	75.95243	1667.23498	-1490.75142	1556.16753	1
C	69.13249	81.64719	73.97828	-714.45746	-175.43074	286.98927	1
H	69.48654	82.65521	73.61486	-787.09484	1763.03619	-1922.46060	1
O	67.94422	81.37933	73.19859	-429.46514	516.51932	-128.40526	1
H	67.80816	82.24602	72.77023	1981.82785	436.16847	-1818.18182	1
C	70.27667	80.58036	73.82884	630.79189	-468.94448	76.03230	1
H	70.64955	80.53370	72.77893	-1775.71861	2062.30353	131.89849	1
H	71.16966	80.78931	74.49289	-1855.14608	88.94118	1993.53760	1
H	69.90895	79.56080	74.08953	-1504.82350	-780.94249	-2132.90992	1
C	70.22118	82.10297	76.46031	288.57638	618.62814	-397.00270	1
O	70.18270	81.82503	77.66047	321.70495	-185.26452	574.37218	1
N	71.14327	82.73117	75.78010	-447.05792	-446.53431	367.76425	1
H	70.89332	82.94535	74.81619	350.31316	1975.63644	-1843.31522	1
C	72.49032	82.90826	76.19821	-511.23061	374.03917	-471.51480	1

H	72.86107	83.62165	75.42721	2234.79579	-898.70890	-1273.45190	1
C	72.49048	83.52624	77.67367	529.53781	-159.34528	563.72604	1
H	73.41508	84.15091	77.80427	654.59388	2347.94251	1217.51488	1
H	71.61954	84.23411	77.69659	-1526.55661	2103.42026	817.87394	1
O	72.42384	82.58602	78.75386	-554.84278	14.12789	399.58769	1
H	71.49006	82.18594	78.54397	1297.06236	2061.84854	1220.68812	1
C	73.27510	81.48967	76.11282	-662.15484	-412.13544	123.62687	1
O	72.86873	80.53260	75.48605	-540.01537	-317.05804	274.91456	1
N	74.45666	81.62243	76.71274	-202.76299	-690.00100	-131.49145	1
H	74.62382	82.51720	77.16696	-702.62132	-2617.37191	281.70413	1
C	75.40080	80.51399	76.79050	-491.73914	576.34187	222.73509	1
H	74.77996	79.65614	76.43175	1977.17978	-1823.58850	-434.68928	1
C	76.58959	80.80457	75.88027	-649.50797	-328.66843	306.11224	1
H	77.10086	81.70914	76.27949	2075.07803	1452.16490	-1004.45904	1
C	77.59430	79.67054	75.86694	-615.18735	-489.99689	-70.96352	1
H	78.51140	79.93385	75.27121	-1226.32661	-1724.38906	-1716.47170	1
H	77.93638	79.40830	76.91709	2381.64273	-969.84735	900.46860	1
H	77.17078	78.72456	75.41595	2122.74991	1702.23946	-141.36228	1
C	76.14110	81.11896	74.46560	-501.86074	-479.61276	-376.42482	1
H	77.00847	81.37563	73.79761	993.79261	-2246.95401	-1177.81305	1
H	75.60350	80.23860	73.99957	42.71279	-626.15391	-2651.37258	1
H	75.42800	81.98855	74.44514	-2026.39012	-1590.33709	-887.82922	1
C	75.88854	80.39021	78.36930	-672.89153	382.57394	-156.34072	1
O	76.92467	79.82517	78.64186	410.04627	221.86223	500.35950	1
N	74.98586	80.87087	79.20654	-378.11782	-443.79414	-441.10984	1
H	74.14375	81.31224	78.83189	2404.15973	-568.14894	1149.30085	1
C	75.18906	80.63819	80.60366	-544.54484	466.16019	331.29119	1
H	76.30229	80.66621	80.67473	-1409.80988	-170.17955	2325.32679	1
C	74.45541	81.83342	81.34637	-184.48284	116.78282	-758.89137	1
H	74.60123	82.78376	80.78433	1893.78641	1861.14652	611.04528	1
H	73.35444	81.66545	81.36683	-2170.79665	-974.19311	1327.50117	1
C	75.02407	81.95261	82.79421	389.51668	672.81878	-138.49152	1
H	74.84457	81.00682	83.35813	510.64577	1933.03327	1851.02484	1
H	76.13297	82.03756	82.76358	-2302.30869	-1105.72939	-948.89774	1
C	74.43462	83.16698	83.60670	-559.93427	-126.34590	542.30857	1
O	74.81349	83.36612	84.73220	-670.36003	-111.83166	76.36448	1
N	73.52685	83.90121	82.92814	-216.06729	-537.07037	446.51379	1
H	73.15260	83.77029	81.98106	-1251.96487	1074.12894	-2168.52500	1
H	73.04215	84.71620	83.32008	-2451.83023	-720.73851	944.84612	1
C	74.63247	79.21799	81.18250	546.24052	-563.88337	-85.11809	1
O	74.89403	79.02838	82.34701	71.70702	-307.40463	606.69673	1
N	73.95541	78.39255	80.36656	479.90863	-504.50129	222.86849	1
C	73.34692	77.21289	80.89918	75.51268	-600.91904	506.73700	1
H	73.03038	77.45445	81.94028	-2033.61562	1795.30103	254.89480	1
C	72.17584	76.94738	79.85686	523.79067	517.99274	284.45581	1
H	71.79090	75.90376	79.89745	1599.61665	-1127.00911	1895.98173	1
H	71.30734	77.62044	80.03371	-1881.83093	1275.82488	-1501.55016	1
C	72.89657	77.29036	78.51682	-600.05639	-404.05026	316.64485	1
H	73.56546	76.45874	78.19796	-2597.05260	496.36221	657.73249	1
H	72.20117	77.48375	77.66907	-1666.24518	1294.38985	-1723.90591	1
C	73.73734	78.57175	78.93816	493.57570	-129.95376	-602.56394	1
H	74.74047	78.64013	78.45622	-1469.08415	-637.84797	2204.22581	1
H	73.19953	79.54285	78.82652	-1038.97840	1068.88701	-2280.71789	1
C	74.36977	75.92969	80.84412	188.88100	241.05089	-727.87821	1
O	73.95597	74.84711	81.18980	531.88613	-49.19943	427.07960	1
N	75.60228	76.25310	80.45973	708.14833	26.93417	-179.73509	1
H	75.68763	77.24635	80.24642	-2232.69501	-1179.09831	1023.94816	1
C	76.58061	75.28018	79.97729	-738.32412	259.92548	104.42456	1
H	77.53093	75.86256	80.02485	2028.89123	1260.02983	1311.33324	1
C	76.56171	74.02875	80.83401	-258.65678	-345.52513	661.28459	1
H	75.58617	73.51245	80.64784	-1826.06090	-1801.46097	-918.64554	1
C	77.67813	73.07450	80.45035	-541.23400	531.92970	-218.41428	1

H	77.64333	72.12771	81.05548	1311.73218	-2219.18200	882.19023	1
H	77.60681	72.78019	79.36511	-1048.71298	1223.58118	-2196.97971	1
H	78.69209	73.53363	80.60730	419.06414	1903.92898	1903.44670	1
C	76.62183	74.34414	82.31739	-423.86431	152.66566	-648.55262	1
H	76.61588	73.41145	82.94498	105.68386	-2342.50080	1387.51238	1
H	77.54595	74.92562	82.58432	-1695.63603	-1484.73621	-1531.02793	1
H	75.73109	74.96286	82.62583	-1853.57121	-487.39031	1936.59343	1
C	76.16594	74.87248	78.42060	539.71428	-510.58232	267.58633	1
O	75.92835	73.72321	78.13222	-15.93758	-278.34085	-624.49402	1
N	76.15838	75.93273	77.62571	-676.81886	12.05292	276.17960	1
H	76.43846	76.82558	78.02829	-1694.44250	-373.84189	-2100.66098	1
C	76.07398	75.68627	76.22617	412.33078	-664.70360	108.35451	1
H	75.58493	74.68926	76.12098	1765.77476	1294.44366	1621.76594	1
C	75.29608	76.90249	75.58171	-179.66635	-721.95667	-264.73915	1
H	74.29071	77.03355	76.04116	-607.34922	2587.87032	-598.10018	1
H	75.84070	77.85864	75.76938	-359.69332	-1479.13954	-2259.74260	1
C	75.18785	76.60171	74.04941	-240.01076	616.02425	431.85322	1
H	76.18828	76.36139	73.62109	-1359.17131	782.54425	2227.99181	1
H	74.55442	75.70652	73.85939	-820.36667	85.81767	-2596.78715	1
C	74.58176	77.88816	73.36008	-513.07015	236.18570	-551.87280	1
H	73.56586	78.11754	73.75865	1816.11944	-1183.11722	-1650.94321	1
H	75.20750	78.79192	73.58533	-1800.04429	-1361.53473	1526.34643	1
C	74.53607	77.61065	71.83897	726.08972	-253.19732	-179.64713	1
H	75.52820	77.46618	71.36565	1224.27271	-456.51798	-2390.90304	1
H	73.85273	76.79154	71.53811	1206.40339	-1150.93514	-2154.90344	1
N	73.95154	78.89247	71.15865	329.70796	-633.68968	-155.67358	1
H	73.85533	78.84553	70.10333	1348.31305	1938.76845	1359.00643	1
H	72.97395	79.19018	71.44637	2096.92035	1446.54551	-966.48890	1
H	74.49958	79.79365	71.27839	-1197.07217	-2303.53719	827.28733	1
C	77.59862	75.74118	75.58877	-378.01186	-556.17301	-413.96481	1
O	78.33368	76.68539	75.74691	-409.44433	-307.55053	-453.30833	1
N	77.76864	74.65322	74.85359	-429.02477	524.70884	274.08502	1
H	77.02264	73.96039	74.82316	1706.34263	-1425.96036	1574.38926	1
C	78.91146	74.54362	74.01054	-45.25864	-626.22952	478.93267	1
H	79.26148	75.60140	73.96045	1988.63164	570.90773	1772.87287	1
C	79.94076	73.59967	74.75440	551.77128	-20.49526	-564.54996	1
H	79.90601	73.78087	75.85215	1434.61535	-1753.10253	1513.99471	1
H	79.63825	72.53728	74.61916	-714.67738	1819.09770	1898.36494	1
C	81.39459	73.81484	74.21801	446.99263	-555.77305	338.97221	1
H	81.43600	73.78833	73.10603	1953.68466	1892.25957	-161.67766	1
H	81.79556	74.80898	74.51719	-403.03785	2688.07032	-188.43591	1
C	82.23298	72.63176	74.85160	567.86262	335.74681	434.04421	1
H	82.20464	72.68423	75.96510	-1080.02275	1568.26081	-1948.78900	1
H	81.77956	71.64757	74.59071	-919.54108	2111.34692	1456.13463	1
C	83.68850	72.70557	74.33762	195.81897	173.33862	-745.11536	1
H	83.81354	72.46404	73.26318	1405.67949	-2301.21089	-390.08113	1
H	84.21967	73.64769	74.57951	-940.37328	-1118.32446	2299.72133	1
N	84.48734	71.59494	75.10210	319.26422	656.36007	-42.02424	1
H	85.51319	71.48823	74.85050	1961.03633	619.91968	1787.09422	1
H	84.54305	71.70866	76.15498	854.14343	-2156.80752	1429.08711	1
H	84.14006	70.59603	75.01185	1237.02905	-1378.50857	-1998.28500	1
C	78.36746	73.92110	72.59420	532.53319	-268.57236	517.55729	1
O	77.34764	74.34823	72.10401	266.16714	-290.73804	-558.88002	1
N	79.19524	73.00197	72.11109	-226.25352	640.49678	-270.32839	1
H	80.01762	72.73690	72.64764	2007.69063	-1035.27229	1523.50231	1
C	78.88488	72.37313	70.88184	-540.76542	-573.33303	49.50000	1
H	77.90039	72.80210	70.58185	-2374.20950	-1036.76023	843.75774	1
C	80.07354	72.71572	69.86544	-77.09082	666.87422	-415.84136	1
H	81.05005	72.37069	70.30473	-985.81834	617.19825	2463.91927	1
O	80.27093	74.11178	69.57525	293.29899	-391.65602	-477.80926	1
H	81.18106	74.24836	69.90583	2528.72714	-681.76262	-751.27122	1
C	79.70603	71.80542	68.63782	252.47363	-605.14473	440.05134	1

H	80.48284	71.86649	67.84044	-2052.65063	1456.49051	-1043.51830	1
H	79.61616	70.72994	68.92919	-2152.98800	-1661.97574	-161.70652	1
H	78.73643	72.11056	68.17976	-473.12726	-1584.27339	2165.61725	1
C	78.85893	70.74483	71.15458	360.36307	488.46713	505.10056	1
O	77.82312	70.14795	70.98584	-200.08817	-528.03552	-385.82850	1
N	80.04475	70.29186	71.53188	-597.54095	-226.44775	355.20479	1
H	80.79274	70.97393	71.64061	2179.23655	-1300.70981	-991.33835	1
C	80.29615	68.90155	71.68472	-687.68921	297.39807	-249.45179	1
H	81.35601	68.89846	72.02308	-349.16256	-2467.37713	-1101.72831	1
C	79.30085	68.34730	72.81198	672.47345	361.54328	-201.62880	1
H	78.23831	68.48577	72.49963	1541.41502	582.18425	2169.97026	1
H	79.46239	67.25593	72.97567	-2534.67717	60.25153	997.72295	1
C	79.59826	69.12790	74.10023	574.26774	-389.43119	-377.02542	1
C	80.80520	68.86131	74.81484	688.82679	-287.33580	-257.96133	1
H	81.49851	68.07115	74.47346	331.14182	1470.38163	2269.80013	1
C	78.69524	70.13269	74.56021	-383.52382	-675.04808	144.24917	1
H	77.76299	70.34604	74.00607	4.09847	1517.59662	2262.86397	1
C	81.09206	69.61765	75.95090	-134.76730	428.45285	-649.50303	1
H	82.02920	69.41349	76.49757	-903.23433	1600.49020	2011.53387	1
C	79.01514	70.84706	75.71790	-335.53281	-615.00040	-364.39058	1
H	78.30329	71.61450	76.07122	1596.45398	-1369.33862	1732.02659	1
C	80.22441	70.63563	76.44382	585.48547	-340.24194	-406.23938	1
O	80.57473	71.39307	77.59188	-419.15649	-173.04408	-511.94141	1
H	79.75761	71.93918	77.68457	-1071.84350	1559.71507	-1960.13012	1
C	80.14180	68.21058	70.22393	291.41878	-183.13091	710.72246	1
O	80.66236	68.73604	69.26364	379.87695	523.47396	-222.23622	1
N	79.60632	66.97643	70.28955	564.30591	373.09380	277.24371	1
H	79.23003	66.77488	71.21615	-120.59931	-2436.07824	-1214.35098	1
C	78.73639	66.59838	69.18306	404.07514	-299.37480	608.84009	1
H	77.82714	67.22008	69.33387	-1071.07882	-841.69025	2359.66467	1
C	79.48061	66.88434	67.82976	-683.12807	68.71770	-390.13096	1
H	79.55796	67.98576	67.64209	2068.88918	1596.58590	770.89603	1
H	80.52973	66.51637	67.90571	1379.86429	2342.49779	-179.85638	1
C	78.74498	66.20432	66.62631	-176.39967	537.49466	550.97268	1
H	78.63225	65.10737	66.78475	-396.28051	1942.22168	1869.33314	1
H	77.71391	66.60143	66.49745	-94.44700	586.00678	-2659.20016	1
C	79.66259	66.51226	65.35674	-291.69381	-718.18079	-150.72929	1
H	79.78721	67.59794	65.11164	-2303.38776	1089.76242	964.62032	1
H	80.68514	66.05620	65.38494	884.16729	2568.80533	-207.74283	1
N	78.97411	65.92211	64.28874	-594.49522	-252.81478	342.29276	1
H	78.09175	65.48369	64.50084	-1735.17426	2084.05705	-263.71605	1
C	79.38979	65.90993	63.08792	595.23177	-271.52487	442.22352	1
N	80.61778	66.52571	62.81812	585.53813	-424.79750	-105.81303	1
H	81.25958	66.99783	63.45367	959.88797	1327.56823	-2177.11905	1
H	81.05384	66.57815	61.89711	1578.48647	16.27378	-2220.76233	1
N	78.48756	65.25188	62.23994	-452.17763	-390.33541	-421.51853	1
H	77.59362	64.80217	62.43156	470.26107	1974.25629	-1817.92087	1
H	78.64630	65.14094	61.23760	1856.67307	-1573.97321	-1224.35416	1
C	78.45340	65.01166	69.37509	-531.47271	-581.45780	-55.06206	1
O	79.12932	64.17293	68.80987	-243.00837	192.73507	609.52485	1
N	77.41784	64.81327	70.18823	343.49288	-644.25607	38.09164	1
H	76.98323	65.65644	70.56434	-1691.16043	-1535.97525	-1484.73099	1
C	77.06627	63.48761	70.68756	542.66492	350.14337	454.42563	1
H	76.13701	63.65994	71.28140	1209.57979	-2396.95920	463.86744	1
C	76.85802	62.55663	69.50452	-737.14309	145.93010	242.71942	1
H	77.82622	62.51678	68.94231	-1050.18303	159.36419	-2509.06125	1
C	76.49724	61.15048	69.94105	-706.77696	-252.88343	-245.16230	1
H	76.41461	60.44878	69.06554	-1624.64039	-1665.49085	1417.86935	1
H	77.27985	60.72665	70.64571	2566.10521	598.51109	693.22305	1
H	75.51156	61.12347	70.48255	-1972.69758	107.10018	-1876.34312	1
C	75.79839	63.08367	68.55335	412.85148	672.58832	-27.69222	1
H	75.68611	62.42677	67.64768	1447.02277	1762.76533	-1490.78944	1

```
H    74.79078  63.14332  69.04898    1320.77379     -145.20455     2378.68491          1
H    76.05278  64.11732  68.19062   -1512.10848     1470.52031     1724.75147          1
C    78.33253  62.98603  71.63908    -607.11113      499.56689       73.73769          1
O    78.60352  61.80924  71.76204     357.97126      460.81534      356.68785          1
N    78.87253  64.01869  72.26219     536.55407     -368.62270      332.76306          1
H    78.48599  64.92962  72.02764     524.38866     2297.69985     1367.20941          1
C    80.03525  63.83602  73.04786      46.50277     -774.29190     -147.97904          1
H    80.09807  62.73675  73.21940    1676.51875     1883.29066     1032.55549          1
C    81.31281  64.43100  72.25435    -559.41456      -34.19198     -556.30433          1
H    81.19266  65.53434  72.13713     -64.66664      395.18769     2695.05373          1
H    82.23976  64.28744  72.85886   -2703.44278      -60.40056      333.79418          1
C    81.45013  63.78332  70.90389    -519.76435      377.31045      459.42366          1
H    80.50879  63.96601  70.31973   -1746.74323     1667.50644    -1261.73691          1
C    82.61180  64.40835  70.14134     570.95554       16.46261     -545.27700          1
H    82.72538  63.95845  69.12136    2391.58273       28.05277    -1305.07271          1
H    82.46537  65.51112  70.00257     175.75588      917.63458     2559.43867          1
H    83.58471  64.26532  70.67867   -1063.97851    -2155.34650     1283.00462          1
C    81.64941  62.27931  71.01785     193.76014        2.62946     -765.53141          1
H    81.86964  61.81595  70.02127     507.39394     -846.55087     2539.60089          1
H    82.49965  62.02118  71.70093    1337.57841     1818.19054     1526.01994          1
H    80.72398  61.77534  71.41808    1100.88635    -1172.91350    -2199.08909          1
C    79.88309  64.73622  74.37537    -549.21115      475.64486     -309.38218          1
O    79.05873  65.60400  74.50368     556.06106     -390.37755      -78.24250          1
O    80.87919  64.53908  75.36254    -572.27940     -310.19032     -209.75862          1
H    81.34123  63.75356  74.95758   -1380.08193     2212.14486      790.85944          1
```

Listing 76: C00076.xmolout

```
       276
   150.0000  150.0000  150.0000     0.0000     0.0000     0.0000***********    1.7470     6988
        0.2500    300.00    0.00    0.00    0.00  -1293.5411       0.0000    300.0000
H    82.71846  80.50228  86.82710   -2043.14101    -1757.64893      399.89339          1
N    83.20327  80.18903  85.98727     426.75047      490.45733     -334.42467          1
H    82.46463  80.25172  85.28124     442.25559    -1110.30854    -2448.52907          1
C    84.32230  80.98340  85.63981     415.32972     -666.39213       83.73172          1
H    84.35508  81.74596  86.44761     879.32219     2491.00447      667.35213          1
C    85.65567  80.07852  85.63149      31.35357      477.41777      628.23362          1
H    85.57642  79.31052  84.82512    1168.38489     2240.25337    -1019.71136          1
H    86.55106  80.69688  85.38294    -360.43770     2534.90498      931.67112          1
C    85.82969  79.41976  86.97436     463.18208      512.70242     -382.34388          1
H    84.89645  78.86003  87.21005    1725.29117     1863.52247     -987.07854          1
C    86.99359  78.43673  86.93555     710.42801     -144.27427     -313.15367          1
H    87.12839  77.91616  87.91860     364.20195    -1033.13725     2494.72524          1
H    86.84217  77.64485  86.15753    2567.61904      335.80679      847.48802          1
H    87.95995  78.95260  86.69982   -2587.99029      680.92867     -512.16411          1
C    86.07546  80.44361  88.07467     268.17616      741.64379      -40.42634          1
H    86.25963  79.95046  89.06381    1283.54314    -1945.37420    -1411.27812          1
H    86.96857  81.08238  87.85160   -1258.57796     1317.15249    -2026.02085          1
H    85.20067  81.13041  88.20723   -2502.84526      909.80597      575.92391          1
C    83.92933  81.61237  84.20086     619.36578     -458.64843     -172.09273          1
O    82.76967  81.79074  83.88325     -81.40433      433.87611      522.34594          1
N    85.02193  81.98345  83.53277     175.23996     -622.20190      341.55523          1
H    85.94257  81.74182  83.89326    -876.57256     -303.22879    -2561.90213          1
C    84.83570  82.44999  82.19266    -571.88214     -534.10783     -106.15033          1
H    83.94294  83.10927  82.25891   -2713.20841      -16.25800     -248.80809          1
C    86.19571  83.17189  81.76776     166.60354      288.16399     -716.09568          1
H    87.02926  82.42146  81.85017   -2097.85574     1636.78449     -586.18044          1
O    86.58726  84.28871  82.58908    -317.80580     -363.38227      484.43082          1
H    87.43776  83.96939  82.94931    1863.74748    -1451.84923     1357.29467          1
C    85.95435  83.49548  80.24739     503.80731      517.99032      318.51606          1
H    86.88224  83.87848  79.76258     640.61020    -2157.10410    -1536.29104          1
H    85.62918  82.59541  79.67182    1687.88455      465.60692     2087.56452          1
```

H	85.17060	84.27659	80.11529	-1293.07895	-1899.88581	-1463.57452	1
C	84.59125	81.13902	81.25190	-153.07550	-733.68902	248.70981	1
O	85.38137	80.23152	81.27980	186.15459	-480.78787	-449.34387	1
N	83.53579	81.32552	80.44897	-487.53139	543.18517	42.03850	1
C	83.15750	80.33659	79.48375	-699.35871	69.34393	360.10701	1
H	83.21999	79.34548	79.98653	2.81126	-221.99807	-2715.58072	1
C	81.70983	80.80658	79.04439	-507.46439	-592.59194	122.07761	1
H	81.42327	80.43393	78.03465	-1975.08116	525.87560	-1801.71556	1
H	80.92588	80.45413	79.75186	1588.27743	-1913.22100	1113.83566	1
C	81.88329	82.35560	79.10608	-613.62442	-457.55993	-194.14490	1
H	82.49610	82.71371	78.24502	1707.54637	-721.27992	1996.92523	1
H	80.92404	82.92016	79.08676	758.23357	2100.81767	1560.55011	1
C	82.67874	82.50220	80.47489	-352.41524	704.96076	49.21465	1
H	83.30864	83.41762	80.55995	1714.76886	-2070.42778	443.35792	1
H	82.05116	82.39470	81.39267	1224.15040	1801.50881	1636.97633	1
C	84.21539	80.48292	78.23052	566.85117	537.05831	117.62814	1
O	84.30325	81.48804	77.56684	-550.26779	-406.08867	4.21666	1
N	84.85100	79.32741	78.10700	566.34832	-425.77725	180.18749	1
H	84.64010	78.56045	78.74529	644.44502	2646.80550	-52.73430	1
C	85.93172	79.23619	77.19238	612.34801	-281.48662	411.56221	1
H	85.91117	80.18092	76.59870	1667.23498	-1490.75142	1556.16753	1
C	87.21916	79.07488	78.13795	-714.45746	-175.43074	286.98927	1
H	87.00054	78.23550	78.85443	-787.09484	1763.03619	-1922.46060	1
O	87.51401	80.21158	78.97382	-429.46514	516.51932	-128.40526	1
H	86.94787	80.02233	79.75468	1981.82785	436.16847	-1818.18182	1
C	88.33608	78.63944	77.12672	630.79189	-468.94448	76.03230	1
H	89.28860	78.39577	77.65289	-1775.71861	2062.30353	131.89849	1
H	88.03651	77.73585	76.54431	-1855.14608	88.94118	1993.53760	1
H	88.56107	79.44982	76.39450	-1504.82350	-780.94249	-2132.90992	1
C	85.63978	77.85813	76.35790	288.57638	618.62814	-397.00270	1
O	85.68334	76.76710	76.92373	321.70495	-185.26452	574.37218	1
N	85.47961	78.15195	75.08911	-447.05792	-446.53431	367.76425	1
H	85.54078	79.15131	74.88378	350.31316	1975.63644	-1843.31522	1
C	84.67403	77.37111	74.20896	-511.23061	374.03917	-471.51480	1
H	84.87347	77.84491	73.22153	2234.79579	-898.70890	-1273.45190	1
C	85.15340	75.84719	74.26481	529.53781	-159.34528	563.72604	1
H	84.88059	75.34756	73.29296	654.59388	2347.94251	1217.51488	1
H	86.27497	75.88794	74.30959	-1526.55661	2103.42026	817.87394	1
O	84.62643	75.02506	75.30976	-554.84278	14.12789	399.58769	1
H	85.03772	75.51833	76.12212	1297.06236	2061.84854	1220.68812	1
C	83.11402	77.54014	74.63051	-662.15484	-412.13544	123.62687	1
O	82.71840	78.11662	75.61432	-540.01537	-317.05804	274.91456	1
N	82.38447	77.00162	73.65002	-202.76299	-690.00100	-131.49145	1
H	82.88339	76.55389	72.88112	-702.62132	-2617.37191	281.70413	1
C	80.92580	76.96985	73.69906	-491.73914	576.34187	222.73509	1
H	80.65302	76.85436	74.77457	1977.17978	-1823.58850	-434.68928	1
C	80.39607	78.25533	73.08515	-649.50797	-328.66843	306.11224	1
H	80.83556	78.34443	72.06652	2075.07803	1452.16490	-1004.45904	1
C	78.88504	78.23022	72.95172	-615.18735	-489.99689	-70.96352	1
H	78.49333	79.17719	72.48784	-1226.32661	-1724.38906	-1716.47170	1
H	78.53621	77.36899	72.30362	2381.64273	-969.84735	900.46860	1
H	78.38327	78.11845	73.95221	2122.74991	1702.23946	-141.36228	1
C	80.81749	79.48046	73.87562	-501.86074	-479.61276	-376.42482	1
H	80.48748	80.43507	73.38052	993.79261	-2246.95401	-1177.81305	1
H	80.38171	79.47130	74.91261	42.71279	-626.15391	-2651.37258	1
H	81.93688	79.52907	73.99513	-2026.39012	-1590.33709	-887.82922	1
C	80.52407	75.69031	72.71646	-672.89153	382.57394	-156.34072	1
O	81.06557	75.52743	71.64944	410.04627	221.86223	500.35950	1
N	79.50687	75.02675	73.24090	-378.11782	-443.79414	-441.10984	1
H	79.15316	75.32523	74.14759	2404.15973	-568.14894	1149.30085	1
C	79.03467	73.86245	72.57621	-544.54484	466.16019	331.29119	1
H	79.59373	73.84387	71.61155	-1409.80988	-170.17955	2325.32679	1

96	C	79.34158	72.62961	73.53187	-184.48284	116.78282	-758.89137	1
97	H	80.43779	72.52819	73.69917	1893.78641	1861.14652	611.04528	1
98	H	78.89102	72.78788	74.53766	-2170.79665	-974.19311	1327.50117	1
99	C	78.74965	71.35892	72.85057	389.51668	672.81878	-138.49152	1
100	H	77.64650	71.47957	72.74868	510.64577	1933.03327	1851.02484	1
101	H	79.11565	71.27575	71.80339	-2302.30869	-1105.72939	-948.89774	1
102	C	79.06181	70.02119	73.62239	-559.93427	-126.34590	542.30857	1
103	O	78.64857	68.98030	73.18087	-670.36003	-111.83166	76.36448	1
104	N	79.79422	70.18348	74.74467	-216.06729	-537.07037	446.51379	1
105	H	80.16815	71.04199	75.16628	-1251.96487	1074.12894	-2168.52500	1
106	H	80.08248	69.41242	75.35667	-2451.83023	-720.73851	944.84612	1
107	C	77.40726	74.00102	72.43341	546.24052	-563.88337	-85.11809	1
108	O	76.70091	73.49548	73.26534	71.70702	-307.40463	606.69673	1
109	N	77.06918	74.72791	71.35615	479.90863	-504.50129	222.86849	1
110	C	75.70453	74.85771	70.95954	75.51268	-600.91904	506.73700	1
111	H	75.09061	74.76506	71.88423	-2033.61562	1795.30103	254.89480	1
112	C	75.72257	76.28137	70.25961	523.79067	517.99274	284.45581	1
113	H	74.85771	76.43964	69.57708	1599.61665	-1127.00911	1895.98173	1
114	H	75.69343	77.10339	71.00933	-1881.83093	1275.82488	-1501.55016	1
115	C	77.09436	76.24308	69.51528	-600.05639	-404.05026	316.64485	1
116	H	77.01370	75.65147	68.57322	-2597.05260	496.36221	657.73249	1
117	H	77.47562	77.24908	69.22751	-1666.24518	1294.38985	-1723.90591	1
118	C	78.02071	75.50341	70.57044	493.57570	-129.95376	-602.56394	1
119	H	78.76404	74.80103	70.12587	-1469.08415	-637.84797	2204.22581	1
120	H	78.54278	76.17542	71.30238	-1038.97840	1068.88701	-2280.71789	1
121	C	75.41040	73.65888	69.86928	188.88100	241.05089	-727.87821	1
122	O	75.90956	73.67457	68.76894	531.88613	-49.19943	427.07960	1
123	N	74.61510	72.74743	70.41054	708.14833	26.93417	-179.73509	1
124	H	74.26637	72.87959	71.35935	-2232.69501	-1179.09831	1023.94816	1
125	C	74.16127	71.68446	69.57609	-738.32412	259.92548	104.42456	1
126	H	74.82955	71.68754	68.68184	2028.89123	1260.02983	1311.33324	1
127	C	74.24285	70.35121	70.45099	-258.65678	-345.52513	661.28459	1
128	H	73.60944	70.44383	71.36987	-1826.06090	-1801.46097	-918.64554	1
129	C	73.78998	69.12926	69.58592	-541.23400	531.92970	-218.41428	1
130	H	73.87625	68.17411	70.15650	1311.73218	-2219.18200	882.19023	1
131	H	72.71150	69.22135	69.25011	-1048.71298	1223.58118	-2196.97971	1
132	H	74.41610	69.02363	68.66850	419.06414	1903.92898	1903.44670	1
133	C	75.74556	70.14083	70.83804	-423.86431	152.66566	-648.55262	1
134	H	75.88679	69.25398	71.50042	105.68386	-2342.50080	1387.51238	1
135	H	76.38500	69.98979	69.93668	-1695.63603	-1484.73621	-1531.02793	1
136	H	76.14559	71.02985	71.38020	-1853.57121	-487.39031	1936.59343	1
137	C	72.60825	72.01786	69.21855	539.71428	-510.58232	267.58633	1
138	O	71.82052	72.58918	69.97973	-15.93758	-278.34085	-624.49402	1
139	N	72.35468	71.47698	68.05513	-676.81886	12.05292	276.17960	1
140	H	73.10152	70.97533	67.57493	-1694.44250	-373.84189	-2100.66098	1
141	C	70.99630	71.43328	67.61299	412.33078	-664.70360	108.35451	1
142	H	70.56134	72.41954	67.89357	1765.77476	1294.44366	1621.76594	1
143	C	71.07301	71.14318	66.06297	-179.66635	-721.95667	-264.73915	1
144	H	71.80724	71.82610	65.57905	-607.34922	2587.87032	-598.10018	1
145	H	71.45423	70.11424	65.87467	-359.69332	-1479.13954	-2259.74260	1
146	C	69.65064	71.33277	65.43811	-240.01076	616.02425	431.85322	1
147	H	68.92589	70.60140	65.86118	-1359.17131	782.54425	2227.99181	1
148	H	69.24434	72.34247	65.67019	-820.36667	85.81767	-2596.78715	1
149	C	69.82237	71.13855	63.87753	-513.07015	236.18570	-551.87280	1
150	H	70.58965	71.84024	63.47686	1816.11944	-1183.11722	-1650.94321	1
151	H	70.19968	70.11603	63.64609	-1800.04429	-1361.53473	1526.34643	1
152	C	68.45682	71.38631	63.19673	726.08972	-253.19732	-179.64713	1
153	H	67.67237	70.64561	63.44962	1224.27271	-456.51798	-2390.90304	1
154	H	68.06050	72.41432	63.31664	1206.40339	-1150.93514	-2154.90344	1
155	N	68.67590	71.22680	61.65361	329.70796	-633.68968	-155.67358	1
156	H	67.81600	71.37097	61.04974	1348.31305	1938.76845	1359.00643	1
157	H	69.36619	71.88934	61.19396	2096.92035	1446.54551	-966.48890	1

H	69.01685	70.28240	61.30953	-1197.07217	-2303.53719	827.28733	1
C	70.25761	70.19058	68.40037	-378.01186	-556.17301	-413.96481	1
O	70.81183	69.13903	68.64626	-409.44433	-307.55053	-453.30833	1
N	69.00482	70.55417	68.60850	-429.02477	524.70884	274.08502	1
H	68.74154	71.47727	68.26639	1706.34263	-1425.96036	1574.38926	1
C	68.06640	69.72279	69.27365	-45.25864	-626.22952	478.93267	1
H	67.09310	70.22199	69.06569	1988.63164	570.90773	1772.87287	1
C	68.16289	68.26722	68.65787	551.77128	-20.49526	-564.54996	1
H	68.21695	68.31181	67.54671	1434.61535	-1753.10253	1513.99471	1
H	69.10739	67.76876	68.98383	-714.67738	1819.09770	1898.36494	1
C	66.90645	67.46391	69.13082	446.99263	-555.77305	338.97221	1
H	66.86541	67.43421	70.24836	1953.68466	1892.25957	-161.67766	1
H	65.96849	67.96500	68.80132	-403.03785	2688.07032	-188.43591	1
C	66.99814	66.00396	68.52943	567.86262	335.74681	434.04421	1
H	67.05330	66.03486	67.41691	-1080.02275	1568.26081	-1948.78900	1
H	67.93128	65.49546	68.86448	-919.54108	2111.34692	1456.13463	1
C	65.74805	65.22190	68.99531	195.81897	173.33862	-745.11536	1
H	65.68354	65.06524	70.09055	1405.67949	-2301.21089	-390.08113	1
H	64.78599	65.61864	68.61399	-940.37328	-1118.32446	2299.72133	1
N	65.84367	63.77864	68.39459	319.26422	656.36007	-42.02424	1
H	65.04269	63.12759	68.63921	1961.03633	619.91968	1787.09422	1
H	65.86356	63.69474	67.33662	854.14343	-2156.80752	1429.08711	1
H	66.68235	63.19245	68.67683	1237.02905	-1378.50857	-1998.28500	1
C	68.38232	69.66161	70.88425	532.53319	-268.57236	517.55729	1
O	67.90354	68.78199	71.56453	266.16714	-290.73804	-558.88002	1
N	69.07247	70.72275	71.27631	-226.25352	640.49678	-270.32839	1
H	69.40227	71.34352	70.53935	2007.69063	-1035.27229	1523.50231	1
C	69.42065	70.87555	72.65537	-540.76542	-573.33303	49.50000	1
H	69.61698	69.84244	73.02269	-2374.20950	-1036.76023	843.75774	1
C	70.70417	71.83184	72.67648	-77.09082	666.87422	-415.84136	1
H	70.55082	72.66234	71.92443	-985.81834	617.19825	2463.91927	1
O	71.91834	71.21509	72.24172	293.29899	-391.65602	-477.80926	1
H	72.03580	71.70008	71.33518	2528.72714	-681.76262	-751.27122	1
C	70.70844	72.43811	74.12365	252.47363	-605.14473	440.05134	1
H	71.65788	72.98559	74.33294	-2052.65063	1456.49051	-1043.51830	1
H	69.87333	73.16416	74.27782	-2152.98800	-1661.97574	-161.70652	1
H	70.60267	71.63868	74.89299	-473.12726	-1584.27339	2165.61725	1
C	68.19804	71.53908	73.48445	360.36307	488.46713	505.10056	1
O	68.29365	71.58657	74.71883	-200.08817	-528.03552	-385.82850	1
N	67.24762	71.99853	72.70900	-597.54095	-226.44775	355.20479	1
H	67.39629	71.82733	71.71480	2179.23655	-1300.70981	-991.33835	1
C	66.13516	72.74409	73.16223	-687.68921	297.39807	-249.45179	1
H	65.47005	72.80395	72.27330	-349.16256	-2467.37713	-1101.72831	1
C	65.46132	72.04435	74.43563	672.47345	361.54328	-201.62880	1
H	66.23389	71.86155	75.22338	1541.41502	582.18425	2169.97026	1
H	64.69843	72.72918	74.87256	-2534.67717	60.25153	997.72295	1
C	64.81047	70.71874	74.01993	574.26774	-389.43119	-377.02542	1
C	63.48989	70.70348	73.47829	688.82679	-287.33580	-257.96133	1
H	62.93142	71.64436	73.32259	331.14182	1470.38163	2269.80013	1
C	65.53667	69.50289	74.19973	-383.52382	-675.04808	144.24917	1
H	66.56986	69.52001	74.59313	4.09847	1517.59662	2262.86397	1
C	62.91729	69.47225	73.16103	-134.76730	428.45285	-649.50303	1
H	61.89284	69.45695	72.75006	-903.23433	1600.49020	2011.53387	1
C	64.91648	68.29556	73.87145	-335.53281	-615.00040	-364.39058	1
H	65.48301	67.36031	74.02411	1596.45398	-1369.33862	1732.02659	1
C	63.59182	68.23066	73.34852	585.48547	-340.24194	-406.23938	1
O	62.95396	67.00730	73.01562	-419.15649	-173.04408	-511.94141	1
H	63.66100	66.37359	73.28397	-1071.84350	1559.71507	-1960.13012	1
C	66.63754	74.22088	73.65485	291.41878	-183.13091	710.72246	1
O	66.24245	75.19805	73.06927	379.87695	523.47396	-222.23622	1
N	67.38176	74.14008	74.75977	564.30591	373.09380	277.24371	1
H	67.71238	73.19964	75.08291	-120.59931	-2436.07824	-1214.35098	1

```
C    68.10928   75.33851   75.11096       404.07514      -299.37480       608.84009            1
H    68.61887   75.66117   74.17670     -1071.07882      -841.69025      2359.66467            1
C    67.03680   76.39259   75.59455      -683.12807        68.71770      -390.13096            1
H    66.17273   76.41130   74.88740      2068.88918      1596.58590       770.89603            1
H    66.61192   76.09014   76.57853      1379.86429      2342.49779      -179.85638            1
C    67.68649   77.80817   75.68981      -176.39967       537.49466       550.97268            1
H    68.51967   77.82983   76.42968      -396.28051      1942.22168      1869.33314            1
H    68.13291   78.11679   74.71829       -94.44700       586.00678     -2659.20016            1
C    66.48654   78.77830   76.11212      -291.69381      -718.18079      -150.72929            1
H    65.59825   78.78256   75.43108     -2303.38776      1089.76242       964.62032            1
H    66.12725   78.62057   77.16125       884.16729      2568.80533      -207.74283            1
N    67.07901   80.04523   76.05677      -594.49522      -252.81478       342.29276            1
H    68.08364   80.07249   76.14314     -1735.17426      2084.05705      -263.71605            1
C    66.44605   81.14193   75.93951       595.23177      -271.52487       442.22352            1
N    65.05106   81.09701   75.84818       585.53813      -424.79750      -105.81303            1
H    64.42282   80.29521   75.88077       959.88797      1327.56823     -2177.11905            1
H    64.44472   81.91385   75.76620      1578.48647        16.27378     -2220.76233            1
N    67.32768   82.23133   75.92939      -452.17763      -390.33541      -421.51853            1
H    68.34216   82.26543   76.01832       470.26107      1974.25629     -1817.92087            1
H    67.01839   83.20185   75.86293      1856.67307     -1573.97321     -1224.35416            1
C    69.11551   74.97135   76.33036      -531.47271      -581.45780       -55.06206            1
O    70.22151   75.50797   76.37889      -243.00837       192.73507       609.52485            1
N    68.53146   74.16043   77.18766       343.49288      -644.25607        38.09164            1
H    67.58053   73.89800   76.92859     -1691.16043     -1535.97525     -1484.73099            1
C    69.20269   73.47251   78.22897       542.66492       350.14337       454.42563            1
H    68.44369   72.73492   78.57109      1209.57979     -2396.95920       463.86744            1
C    70.48962   72.77804   77.59537      -737.14309       145.93010       242.71942            1
H    70.84846   73.34337   76.70726     -1050.18303       159.36419     -2509.06125            1
C    71.63986   72.62855   78.64632      -706.77696      -252.88343      -245.16230            1
H    72.48119   72.01230   78.24987     -1624.64039     -1665.49085      1417.86935            1
H    72.06421   73.63121   78.92378      2566.10521       598.51109       693.22305            1
H    71.27889   72.14161   79.58334     -1972.69758       107.10018     -1876.34312            1
C    70.04665   71.32898   77.19986       412.85148       672.58832       -27.69222            1
H    70.90744   70.72332   76.82964      1447.02277      1762.76533     -1490.78944            1
H    69.60582   70.78065   78.06554      1320.77379      -145.20455      2378.68491            1
H    69.26786   71.35766   76.37391     -1512.10848      1470.52031      1724.75147            1
C    69.61289   74.47247   79.46097      -607.11113       499.56689        73.73769            1
O    69.31567   74.13435   80.58334       357.97126       460.81534       356.68785            1
N    70.30302   75.53648   79.07419       536.55407      -368.62270       332.76306            1
H    70.52752   75.71127   78.06059       524.38866      2297.69985      1367.20941            1
C    70.66424   76.44066   80.10609        46.50277      -774.29190      -147.97904            1
H    70.01620   76.16140   80.97017      1676.51875      1883.29066      1032.55549            1
C    72.23636   76.26196   80.44762      -559.41456       -34.19198      -556.30433            1
H    72.82889   76.37001   79.50875       -64.66664       395.18769      2695.05373            1
H    72.57668   77.08341   81.12155     -2703.44278       -60.40056       333.79418            1
C    72.49784   74.92469   81.08096      -519.76435       377.31045       459.42366            1
H    72.10105   74.12417   80.40606     -1746.74323      1667.50644     -1261.73691            1
C    73.99638   74.70943   81.25817       570.95554        16.46261      -545.27700            1
H    74.22059   73.70476   81.70044      2391.58273        28.05277     -1305.07271            1
H    74.54180   74.77125   80.28153       175.75588       917.63458      2559.43867            1
H    74.44569   75.47795   81.93813     -1063.97851     -2155.34650      1283.00462            1
C    71.79998   74.78397   82.42672       193.76014         2.62946      -765.53141            1
H    72.09604   73.83643   82.94685       507.39394      -846.55087      2539.60089            1
H    72.04684   75.63272   83.11528      1337.57841      1818.19054      1526.01994            1
H    70.68358   74.75589   82.30628      1100.88635     -1172.91350     -2199.08909            1
C    70.46377   77.95330   79.57915      -549.21115       475.64486      -309.38218            1
O    70.38766   78.26167   78.41947       556.06106      -390.37755       -78.24250            1
O    70.48265   78.94852   80.58751      -572.27940      -310.19032      -209.75862            1
H    70.55141   78.35478   81.38547     -1380.08193      2212.14486       790.85944            1
```

Listing 77: C00077.xmolout

```
      276
  150.0000  150.0000  150.0000    0.0000    0.0000    0.0000**********    0.9895    3958
       0.2500    300.00   0.00   0.00   0.00  -1293.5337      0.0000    300.0000
H   60.27662  77.58934  65.25431    -2043.14101    -1757.64893      399.89339         1
N   60.57299  78.39843  65.79713      426.75047      490.45733     -334.42467         1
H   60.91634  77.97449  66.66003      442.25559    -1110.30854    -2448.52907         1
C   61.55314  79.20249  65.17606      415.32972     -666.39213       83.73172         1
H   61.42972  79.10115  64.07536      879.32219     2491.00447      667.35213         1
C   61.35094  80.71706  65.70533       31.35357      477.41777      628.23362         1
H   61.48197  80.72715  66.81420     1168.38489     2240.25337    -1019.71136         1
H   62.14094  81.38798  65.29256     -360.43770     2534.90498      931.67112         1
C   59.98500  81.22970  65.33484      463.18208      512.70242     -382.34388         1
H   59.24155  80.42906  65.54941     1725.29117     1863.52247     -987.07854         1
C   59.63926  82.45167  66.17737      710.42801     -144.27427     -313.15367         1
H   58.62469  82.85545  65.92669      364.20195    -1033.13725     2494.72524         1
H   59.64143  82.21244  67.27235     2567.61904      335.80679      847.48802         1
H   60.37486  83.28207  66.01970    -2587.99029      680.92867     -512.16411         1
C   59.91114  81.59135  63.85763      268.17616      741.64379      -40.42634         1
H   58.89999  81.98648  63.57931     1283.54314    -1945.37420    -1411.27812         1
H   60.65926  82.38116  63.58881    -1258.57796     1317.15249    -2026.02085         1
H   60.11382  80.70559  63.20228    -2502.84526      909.80597      575.92391         1
C   62.97488  78.67632  65.75271      619.36578     -458.64843     -172.09273         1
O   63.11212  78.21689  66.88982      -81.40433      433.87611      522.34594         1
N   63.90201  78.97195  64.87047      175.23996     -622.20190      341.55523         1
H   63.58864  79.33259  63.97134     -876.57256     -303.22879    -2561.90213         1
C   65.24128  78.48452  65.05159     -571.88214     -534.10783     -106.15033         1
H   65.07726  77.43237  65.37798    -2713.20841      -16.25800     -248.80809         1
C   65.98755  78.62543  63.64232      166.60354      288.16399     -716.09568         1
H   66.18886  79.71349  63.44383    -2097.85574     1636.78449     -586.18044         1
O   65.26987  78.12807  62.50112     -317.80580     -363.38227      484.43082         1
H   65.24655  78.92311  61.93032     1863.74748    -1451.84923     1357.29467         1
C   67.35083  77.91074  63.96752      503.80731      517.99032      318.51606         1
H   68.08028  78.01984  63.13178      640.61020    -2157.10410    -1536.29104         1
H   67.82734  78.32648  64.88910     1687.88455      465.60692     2087.56452         1
H   67.20446  76.81859  64.13657    -1293.07895    -1899.88581    -1463.57452         1
C   66.04322  79.31877  66.18504     -153.07550     -733.68902      248.70981         1
O   66.78128  78.64817  66.89489      186.15459     -480.78787     -449.34387         1
N   65.91245  80.64465  66.14932     -487.53139      543.18517       42.03850         1
C   66.77979  81.50571  66.90199     -699.35871       69.34393      360.10701         1
H   67.79750  81.06215  66.81306        2.81126     -221.99807    -2715.58072         1
C   66.55704  82.90518  66.19087     -507.46439     -592.59194      122.07761         1
H   66.86583  83.76575  66.82630    -1975.08116      525.87560    -1801.71556         1
H   67.13072  82.98105  65.24007     1588.27743    -1913.22100     1113.83566         1
C   65.01926  82.85728  65.92665     -613.62442     -457.55993     -194.14490         1
H   64.45341  83.07087  66.86171     1707.54637     -721.27992     1996.92523         1
H   64.67041  83.59322  65.16755      758.23357     2100.81767     1560.55011         1
C   64.82914  81.34712  65.47181     -352.41524      704.96076       49.21465         1
H   63.88579  80.86507  65.81452     1714.76886    -2070.42778      443.35792         1
H   64.96370  81.16637  64.37971     1224.15040     1801.50881     1636.97633         1
C   66.24475  81.53292  68.45095      566.85117      537.05831      117.62814         1
O   65.82379  82.55189  68.93902     -550.26779     -406.08867        4.21666         1
N   66.42222  80.33371  69.00031      566.34832     -425.77725      180.18749         1
H   66.76796  79.53863  68.41241      644.44502     2646.80550      -52.73430         1
C   65.77969  80.11887  70.25623      612.34801     -281.48662      411.56221         1
H   65.29387  81.10034  70.47371     1667.23498    -1490.75142     1556.16753         1
C   64.71246  78.94982  70.01115     -714.45746     -175.43074      286.98927         1
H   65.20001  78.11451  69.43704     -787.09484     1763.03619    -1922.46060         1
O   63.57628  79.35684  69.25648     -429.46514      516.51932     -128.40526         1
H   63.61933  78.76316  68.43316     1981.82785      436.16847    -1818.18182         1
C   64.40676  78.47276  71.47479      630.79189     -468.94448       76.03230         1
H   63.67485  77.63193  71.48314    -1775.71861     2062.30353      131.89849         1
```

```
H   65.32253  78.11555  72.00041    -1855.14608      88.94118    1993.53760    1
H   63.97170  79.29640  72.08717    -1504.82350    -780.94249   -2132.90992    1
C   66.98015  79.81903  71.33243      288.57638     618.62814    -397.00270    1
O   67.36229  80.75769  71.98786      321.70495    -185.26452     574.37218    1
N   67.38314  78.56398  71.37801     -447.05792    -446.53431     367.76425    1
H   67.06298  77.79454  70.76337      350.31316    1975.63644   -1843.31522    1
C   68.48348  78.22452  72.25100     -511.23061     374.03917    -471.51480    1
H   69.33780  78.82860  71.86892     2234.79579    -898.70890   -1273.45190    1
C   68.14667  78.61796  73.77115      529.53781    -159.34528     563.72604    1
H   69.09764  78.49378  74.35911      654.59388    2347.94251    1217.51488    1
H   67.90463  79.71841  73.77921    -1526.55661    2103.42026     817.87394    1
O   67.12511  77.83265  74.43309     -554.84278      14.12789     399.58769    1
H   66.33304  78.32746  74.14787     1297.06236    2061.84854    1220.68812    1
C   68.60086  76.65644  71.96341     -662.15484    -412.13544     123.62687    1
O   67.97602  76.18756  71.02659     -540.01537    -317.05804     274.91456    1
N   69.50693  76.04477  72.74795     -202.76299    -690.00100    -131.49145    1
H   69.85685  76.62084  73.51678     -702.62132   -2617.37191     281.70413    1
C   69.45512  74.57861  72.88726     -491.73914     576.34187     222.73509    1
H   68.40310  74.33257  72.61157     1977.17978   -1823.58850    -434.68928    1
C   70.46656  73.99220  71.92528     -649.50797    -328.66843     306.11224    1
H   71.45677  74.45156  72.14089     2075.07803    1452.16490   -1004.45904    1
C   70.59491  72.48911  72.09585     -615.18735    -489.99689     -70.96352    1
H   71.30707  72.04667  71.34672    -1226.32661   -1724.38906   -1716.47170    1
H   70.97155  72.21933  73.12155     2381.64273    -969.84735     900.46860    1
H   69.60552  71.97234  71.95841     2122.74991    1702.23946    -141.36228    1
C   70.11835  74.32009  70.48385     -501.86074    -479.61276    -376.42482    1
H   70.89753  73.94561  69.76480      993.79261   -2246.95401   -1177.81305    1
H   69.13469  73.86394  70.18492       42.71279    -626.15391   -2651.37258    1
H   70.01781  75.43204  70.33114    -2026.39012   -1590.33709    -887.82922    1
C   69.82058  74.19694  74.45731     -672.89153     382.57394    -156.34072    1
O   69.00924  73.57608  75.10154      410.04627     221.86223     500.35950    1
N   71.02478  74.59072  74.83497     -378.11782    -443.79414    -441.10984    1
H   71.61348  75.08926  74.17196     2404.15973    -568.14894    1149.30085    1
C   71.40152  74.30364  76.19522     -544.54484     466.16019     331.29119    1
H   70.47260  74.52913  76.76883    -1409.80988    -170.17955    2325.32679    1
C   71.75531  72.75614  76.26937     -184.48284     116.78282    -758.89137    1
H   70.88406  72.16029  75.91352     1893.78641    1861.14652     611.04528    1
H   72.60160  72.49996  75.59361    -2170.79665    -974.19311    1327.50117    1
C   72.09318  72.42877  77.75794      389.51668     672.81878    -138.49152    1
H   73.06593  72.90257  78.06188      510.64577    1933.03327    1851.02484    1
H   71.34440  72.90412  78.43075    -2302.30869   -1105.72939    -948.89774    1
C   72.13650  70.89009  78.05918     -559.93427    -126.34590     542.30857    1
O   72.28031  70.51381  79.20059     -670.36003    -111.83166      76.36448    1
N   72.00483  70.11269  76.96803     -216.06729    -537.07037     446.51379    1
H   71.80916  70.38251  75.99715    -1251.96487    1074.12894   -2168.52500    1
H   71.96769  69.08735  76.99209    -2451.83023    -720.73851     944.84612    1
C   72.69893  75.18859  76.57533      546.24052    -563.88337     -85.11809    1
O   73.78177  74.95302  76.05056       71.70702    -307.40463     606.69673    1
N   72.38301  76.11127  77.47479      479.90863    -504.50129     222.86849    1
C   73.38238  76.89974  78.13592       75.51268    -600.91904     506.73700    1
H   74.21693  77.04351  77.40774    -2033.61562    1795.30103     254.89480    1
C   72.56081  78.20039  78.51382      523.79067     517.99274     284.45581    1
H   73.02926  78.78468  79.33717     1599.61665   -1127.00911    1895.98173    1
H   72.46747  78.88941  77.64517    -1881.83093    1275.82488   -1501.55016    1
C   71.17816  77.58751  78.90397     -600.05639    -404.05026     316.64485    1
H   71.22397  77.12341  79.91682    -2597.05260     496.36221     657.73249    1
H   70.34713  78.32801  78.92567    -1666.24518    1294.38985   -1723.90591    1
C   71.00217  76.48194  77.78232      493.57570    -129.95376    -602.56394    1
H   70.45738  75.56474  78.11091    -1469.08415    -637.84797    2204.22581    1
H   70.56597  76.84745  76.82242    -1038.97840    1068.88701   -2280.71789    1
C   73.85068  76.12316  79.50272      188.88100     241.05089    -727.87821    1
O   73.05640  75.84599  80.37189      531.88613     -49.19943     427.07960    1
```

N	75.17723	76.02799	79.51199	708.14833	26.93417	-179.73509	1
H	75.61676	76.35504	78.65056	-2232.69501	-1179.09831	1023.94816	1
C	75.92698	75.18003	80.43122	-738.32412	259.92548	104.42456	1
H	76.99022	75.46582	80.24597	2028.89123	1260.02983	1311.33324	1
C	75.46092	75.50000	81.83926	-258.65678	-345.52513	661.28459	1
H	74.35470	75.31473	81.87475	-1826.06090	-1801.46097	-918.64554	1
C	76.11883	74.64897	82.90899	-541.23400	531.92970	-218.41428	1
H	75.76535	74.93551	83.93768	1311.73218	-2219.18200	882.19023	1
H	75.89168	73.55597	82.77645	-1048.71298	1223.58118	-2196.97971	1
H	77.23791	74.76322	82.90204	419.06414	1903.92898	1903.44670	1
C	75.68566	76.96607	82.17009	-423.86431	152.66566	-648.55262	1
H	75.28675	77.22956	83.18768	105.68386	-2342.50080	1387.51238	1
H	76.77864	77.22985	82.16115	-1695.63603	-1484.73621	-1531.02793	1
H	75.17445	77.63477	81.42450	-1853.57121	-487.39031	1936.59343	1
C	75.65485	73.59622	80.00344	539.71428	-510.58232	267.58633	1
O	74.79921	73.26471	79.18233	-15.93758	-278.34085	-624.49402	1
N	76.52384	72.81183	80.61634	-676.81886	12.05292	276.17960	1
H	77.13262	73.43031	81.15178	-1694.44250	-373.84189	-2100.66098	1
C	76.25039	71.47891	81.03232	412.33078	-664.70360	108.35451	1
H	76.92163	71.31785	81.90524	1765.77476	1294.44366	1621.76594	1
C	74.71089	71.37245	81.37547	-179.66635	-721.95667	-264.73915	1
H	74.37831	72.19848	82.04313	-607.34922	2587.87032	-598.10018	1
H	74.10764	71.48612	80.43986	-359.69332	-1479.13954	-2259.74260	1
C	74.47879	69.97288	82.03809	-240.01076	616.02425	431.85322	1
H	75.00198	69.17581	81.45936	-1359.17131	782.54425	2227.99181	1
H	74.89768	69.93196	83.06866	-820.36667	85.81767	-2596.78715	1
C	72.92038	69.71756	82.03346	-513.07015	236.18570	-551.87280	1
H	72.39665	70.41729	82.72567	1816.11944	-1183.11722	-1650.94321	1
H	72.50483	69.93190	80.99518	-1800.04429	-1361.53473	1526.34643	1
C	72.67045	68.24501	82.43395	726.08972	-253.19732	-179.64713	1
H	73.11873	67.49797	81.74858	1224.27271	-456.51798	-2390.90304	1
H	72.92699	67.99878	83.48345	1206.40339	-1150.93514	-2154.90344	1
N	71.12892	68.00527	82.32060	329.70796	-633.68968	-155.67358	1
H	70.78714	67.03188	82.56791	1348.31305	1938.76845	1359.00643	1
H	70.49518	68.61169	82.91849	2096.92035	1446.54551	-966.48890	1
H	70.70968	68.13668	81.35509	-1197.07217	-2303.53719	827.28733	1
C	76.51031	70.39288	79.83157	-378.01186	-556.17301	-413.96481	1
O	76.81891	69.25540	80.08904	-409.44433	-307.55053	-453.30833	1
N	76.15687	70.92562	78.66920	-429.02477	524.70884	274.08502	1
H	75.81512	71.90923	78.65403	1706.34263	-1425.96036	1574.38926	1
C	76.61650	70.29372	77.46684	-45.25864	-626.22952	478.93267	1
H	76.59295	69.22251	77.77917	1988.63164	570.90773	1772.87287	1
C	75.53155	70.71418	76.39390	551.77128	-20.49526	-564.54996	1
H	74.53250	70.76209	76.88431	1434.61535	-1753.10253	1513.99471	1
H	75.73006	71.75024	76.03843	-714.67738	1819.09770	1898.36494	1
C	75.46380	69.72759	75.18258	446.99263	-555.77305	338.97221	1
H	76.43892	69.64374	74.64684	1953.68466	1892.25957	-161.67766	1
H	75.20336	68.69588	75.50957	-403.03785	2688.07032	-188.43591	1
C	74.33972	70.33945	74.25093	567.86262	335.74681	434.04421	1
H	73.36200	70.35420	74.78217	-1080.02275	1568.26081	-1948.78900	1
H	74.56383	71.40448	74.00983	-919.54108	2111.34692	1456.13463	1
C	74.23665	69.50140	72.95689	195.81897	173.33862	-745.11536	1
H	75.11875	69.57127	72.28965	1405.67949	-2301.21089	-390.08113	1
H	73.95965	68.43941	73.11070	-940.37328	-1118.32446	2299.72133	1
N	73.05333	70.10649	72.12644	319.26422	656.36007	-42.02424	1
H	72.87745	69.67989	71.17054	1961.03633	619.91968	1787.09422	1
H	72.08503	70.05522	72.55867	854.14343	-2156.80752	1429.08711	1
H	73.13216	71.13596	71.88571	1237.02905	-1378.50857	-1998.28500	1
C	78.13363	70.80246	77.19675	532.53319	-268.57236	517.55729	1
O	78.94332	70.91087	78.11458	266.16714	-290.73804	-558.88002	1
N	78.33588	71.04936	75.91352	-226.25352	640.49678	-270.32839	1
H	77.54775	70.86872	75.29499	2007.69063	-1035.27229	1523.50231	1

C	79.65604	70.77361	75.39786	-540.76542	-573.33303	49.50000	1
H	79.99699	69.92590	76.03326	-2374.20950	-1036.76023	843.75774	1
C	79.47576	70.42733	73.85836	-77.09082	666.87422	-415.84136	1
H	79.23216	71.36888	73.29349	-985.81834	617.19825	2463.91927	1
O	78.41039	69.50239	73.54504	293.29899	-391.65602	-477.80926	1
H	77.89975	70.01905	72.89102	2528.72714	-681.76262	-751.27122	1
C	80.93805	69.97203	73.49669	252.47363	-605.14473	440.05134	1
H	81.04323	69.76108	72.40745	-2052.65063	1456.49051	-1043.51830	1
H	81.68803	70.75844	73.75692	-2152.98800	-1661.97574	-161.70652	1
H	81.22405	69.04338	74.04313	-473.12726	-1584.27339	2165.61725	1
C	80.58330	72.10074	75.59128	360.36307	488.46713	505.10056	1
O	80.96045	72.73805	74.63884	-200.08817	-528.03552	-385.82850	1
N	80.88325	72.26139	76.88321	-597.54095	-226.44775	355.20479	1
H	80.41117	71.65746	77.60456	2179.23655	-1300.70981	-991.33835	1
C	81.36584	73.55519	77.23297	-687.68921	297.39807	-249.45179	1
H	81.27730	74.16829	76.30554	-349.16256	-2467.37713	-1101.72831	1
C	80.47255	74.05582	78.46388	672.47345	361.54328	-201.62880	1
H	80.20881	73.21086	79.14070	1541.41502	582.18425	2169.97026	1
H	81.03973	74.80294	79.06497	-2534.67717	60.25153	997.72295	1
C	79.21174	74.69468	77.86592	574.26774	-389.43119	-377.02542	1
C	79.31342	76.02280	77.36152	688.82679	-287.33580	-257.96133	1
H	80.27050	76.57429	77.40390	331.14182	1470.38163	2269.80013	1
C	77.98002	73.98242	77.78122	-383.52382	-675.04808	144.24917	1
H	77.90385	72.94507	78.16338	4.09847	1517.59662	2262.86397	1
C	78.17895	76.60248	76.80878	-134.76730	428.45285	-649.50303	1
H	78.25863	77.63360	76.42268	-903.23433	1600.49020	2011.53387	1
C	76.87590	74.62150	77.21226	-335.53281	-615.00040	-364.39058	1
H	75.91695	74.07133	77.17360	1596.45398	-1369.33862	1732.02659	1
C	76.92785	75.94736	76.70864	585.48547	-340.24194	-406.23938	1
O	75.83668	76.61608	76.14883	-419.15649	-173.04408	-511.94141	1
H	75.13683	75.83112	76.07475	-1071.84350	1559.71507	-1960.13012	1
C	82.91645	73.45577	77.74080	291.41878	-183.13091	710.72246	1
O	83.75186	74.17838	77.19541	379.87695	523.47396	-222.23622	1
N	83.05905	72.64011	78.75870	564.30591	373.09380	277.24371	1
H	82.18965	72.17623	79.02035	-120.59931	-2436.07824	-1214.35098	1
C	84.31294	72.17921	79.24208	404.07514	-299.37480	608.84009	1
H	84.03314	71.32140	79.89377	-1071.07882	-841.69025	2359.66467	1
C	85.19364	71.76625	77.98725	-683.12807	68.71770	-390.13096	1
H	84.62400	71.10079	77.29999	2068.88918	1596.58590	770.89603	1
H	85.45458	72.66682	77.38288	1379.86429	2342.49779	-179.85638	1
C	86.47221	71.06452	78.53345	-176.39967	537.49466	550.97268	1
H	86.95066	71.68652	79.32344	-396.28051	1942.22168	1869.33314	1
H	86.22208	70.09849	79.02520	-94.44700	586.00678	-2659.20016	1
C	87.47756	70.84438	77.30309	-291.69381	-718.18079	-150.72929	1
H	87.08195	70.26369	76.43253	-2303.38776	1089.76242	964.62032	1
H	87.94472	71.80207	76.95483	884.16729	2568.80533	-207.74283	1
N	88.44350	70.08992	77.97996	-594.49522	-252.81478	342.29276	1
H	88.67617	70.40956	78.90993	-1735.17426	2084.05705	-263.71605	1
C	89.06800	69.07475	77.52688	595.23177	-271.52487	442.22352	1
N	88.79572	68.62839	76.23257	585.53813	-424.79750	-105.81303	1
H	88.16382	69.00765	75.52856	959.88797	1327.56823	-2177.11905	1
H	89.26077	67.83955	75.78114	1578.48647	16.27378	-2220.76233	1
N	89.96545	68.58907	78.48606	-452.17763	-390.33541	-421.51853	1
H	90.16205	68.90419	79.43501	470.26107	1974.25629	-1817.92087	1
H	90.56711	67.77962	78.32990	1856.67307	-1573.97321	-1224.35416	1
C	85.14174	73.32775	80.06896	-531.47271	-581.45780	-55.06206	1
O	85.50908	73.04282	81.17839	-243.00837	192.73507	609.52485	1
N	85.32989	74.43236	79.36434	343.49288	-644.25607	38.09164	1
H	84.92349	74.47978	78.38972	-1691.16043	-1535.97525	-1484.73099	1
C	86.06908	75.59882	79.83484	542.66492	350.14337	454.42563	1
H	85.50722	76.45118	79.38296	1209.57979	-2396.95920	463.86744	1
C	86.12640	75.65995	81.34686	-737.14309	145.93010	242.71942	1

```
H    86.61841  74.72343  81.71589    -1050.18303      159.36419    -2509.06125          1
C    86.94550  76.84159  81.83532     -706.77696     -252.88343     -245.16230          1
H    87.00274  76.87294  82.95748    -1624.64039    -1665.49085     1417.86935          1
H    88.00127  76.80190  81.44940     2566.10521      598.51109      693.22305          1
H    86.50508  77.81935  81.49786    -1972.69758      107.10018    -1876.34312          1
C    84.73842  75.71989  81.96101      412.85148      672.58832      -27.69222          1
H    84.78118  75.78221  83.08259     1447.02277     1762.76533    -1490.78944          1
H    84.15939  76.61167  81.59630     1320.77379     -145.20455     2378.68491          1
H    84.14301  74.80026  81.69867    -1512.10848     1470.52031     1724.75147          1
C    87.60164  75.49985  79.18690     -607.11113      499.56689       73.73769          1
O    88.17996  74.45314  79.01098      357.97126      460.81534      356.68785          1
N    88.12316  76.74620  79.09128      536.55407     -368.62270      332.76306          1
H    87.34228  77.36267  79.32768      524.38866     2297.69985     1367.20941          1
C    88.88525  77.06947  77.91098       46.50277     -774.29190     -147.97904          1
H    89.27367  78.08085  78.15436     1676.51875     1883.29066     1032.55549          1
C    90.05973  76.00155  77.67179     -559.41456      -34.19198     -556.30433          1
H    89.61070  75.03641  77.32746      -64.66664      395.18769     2695.05373          1
H    90.74604  76.33590  76.85599    -2703.44278      -60.40056      333.79418          1
C    90.81976  75.78552  78.95773     -519.76435      377.31045      459.42366          1
H    90.07572  75.50217  79.73972    -1746.74323     1667.50644    -1261.73691          1
C    91.81542  74.64395  78.79941      570.95554       16.46261     -545.27700          1
H    92.37210  74.44451  79.75145     2391.58273       28.05277    -1305.07271          1
H    91.30403  73.69026  78.50677      175.75588      917.63458     2559.43867          1
H    92.57881  74.86846  78.01008    -1063.97851    -2155.34650     1283.00462          1
C    91.55022  77.04599  79.39761      193.76014        2.62946     -765.53141          1
H    92.12713  76.88112  80.34412      507.39394     -846.55087     2539.60089          1
H    92.28040  77.39164  78.62086     1337.57841     1818.19054     1526.01994          1
H    90.84013  77.89182  79.58647     1100.88635    -1172.91350    -2199.08909          1
C    87.85971  77.14378  76.69621     -549.21115      475.64486     -309.38218          1
O    86.68691  77.37238  76.85118      556.06106     -390.37755      -78.24250          1
O    88.40460  77.07246  75.38559     -572.27940     -310.19032     -209.75862          1
H    89.36217  76.97121  75.61661    -1380.08193     2212.14486      790.85944          1
```

Listing 78: C00078.xmolout

```
       276
  150.0000  150.0000  150.0000     0.0000     0.0000     0.0000***********    2.7750    11100
       0.2500     300.00    0.00    0.00    0.00  -1293.5304       0.0000     300.0000
H    80.62860  79.47171  76.41020    -2043.14101    -1757.64893      399.89339          1
N    81.21464  78.78709  75.93443      426.75047      490.45733     -334.42467          1
H    80.55577  78.36630  75.27195      442.25559    -1110.30854    -2448.52907          1
C    82.33167  79.34976  75.26651      415.32972     -666.39213       83.73172          1
H    82.18571  80.44353  75.40299      879.32219     2491.00447      667.35213          1
C    83.70431  78.83508  75.93764       31.35357      477.41777      628.23362          1
H    83.84318  77.75017  75.71411     1168.38489     2240.25337    -1019.71136          1
H    84.58151  79.35720  75.48563     -360.43770     2534.90498      931.67112          1
C    83.66163  79.06328  77.42506      463.18208      512.70242     -382.34388          1
H    82.74218  78.57767  77.82282     1725.29117     1863.52247     -987.07854          1
C    84.88235  78.43246  78.08424      710.42801     -144.27427     -313.15367          1
H    84.88283  78.58304  79.19415      364.20195    -1033.13725     2494.72524          1
H    84.93039  77.32883  77.89733     2567.61904      335.80679      847.48802          1
H    85.83387  78.87526  77.69207    -2587.99029      680.92867     -512.16411          1
C    83.61093  80.54829  77.75856      268.17616      741.64379      -40.42634          1
H    83.63157  80.72471  78.86442     1283.54314    -1945.37420    -1411.27812          1
H    84.48108  81.09971  77.31812    -1258.57796     1317.15249    -2026.02085          1
H    82.67873  81.02918  77.36569    -2502.84526      909.80597      575.92391          1
C    82.14841  78.86276  73.73987      619.36578     -458.64843     -172.09273          1
O    81.02567  78.63393  73.29226      -81.40433      433.87611      522.34594          1
N    83.31171  78.73462  73.12298      175.23996     -622.20190      341.55523          1
H    84.17253  78.94439  73.62501     -876.57256     -303.22879    -2561.90213          1
C    83.34730  78.21419  71.80282     -571.88214     -534.10783     -106.15033          1
H    82.30508  78.34600  71.43262    -2713.20841      -16.25800     -248.80809          1
```

```
C    84.50019  78.99899  71.01073      166.60354      288.16399     -716.09568          1
H    85.49412  78.67263  71.42972    -2097.85574     1636.78449     -586.18044          1
O    84.47005  80.43259  71.11690     -317.80580     -363.38227      484.43082          1
H    85.32956  80.61048  71.55078     1863.74748    -1451.84923     1357.29467          1
C    84.35002  78.42724  69.55318      503.80731      517.99032      318.51606          1
H    85.15685  78.80828  68.88456      640.61020    -2157.10410    -1536.29104          1
H    84.40638  77.31168  69.53952     1687.88455      465.60692     2087.56452          1
H    83.37679  78.72227  69.09705    -1293.07895    -1899.88581    -1463.57452          1
C    83.85187  76.65648  71.84261     -153.07550     -733.68902      248.70981          1
O    85.00590  76.44746  71.56877      186.15459     -480.78787     -449.34387          1
N    82.89847  75.77961  72.21340     -487.53139      543.18517       42.03850          1
C    83.08330  74.38307  72.02373     -699.35871       69.34393      360.10701          1
H    84.14785  74.22641  71.73100        2.81126     -221.99807    -2715.58072          1
C    82.65620  73.81175  73.44641     -507.46439     -592.59194      122.07761          1
H    82.42576  72.72337  73.42314    -1975.08116      525.87560    -1801.71556          1
H    83.45740  73.95722  74.20473     1588.27743    -1913.22100     1113.83566          1
C    81.41019  74.69689  73.75750     -613.62442     -457.55993     -194.14490          1
H    80.49401  74.32202  73.21220     1707.54637     -721.27992     1996.92523          1
H    81.14612  74.72925  74.83961      758.23357     2100.81767     1560.55011          1
C    81.85237  76.10785  73.18026     -352.41524      704.96076       49.21465          1
H    81.04572  76.65027  72.62147     1714.76886    -2070.42778      443.35792          1
H    82.32628  76.78658  73.92743     1224.15040     1801.50881     1636.97633          1
C    81.98592  73.77088  70.94912      566.85117      537.05831      117.62814          1
O    81.86819  72.56645  70.90291     -550.26779     -406.08867        4.21666          1
N    81.42108  74.71134  70.20880      566.34832     -425.77725      180.18749          1
H    81.75197  75.64447  70.45161      644.44502     2646.80550      -52.73430          1
C    80.18576  74.55436  69.51484      612.34801     -281.48662      411.56221          1
H    80.15201  75.41718  68.81399     1667.23498    -1490.75142     1556.16753          1
C    80.23955  73.16296  68.73016     -714.45746     -175.43074      286.98927          1
H    80.45083  72.33930  69.48012     -787.09484     1763.03619    -1922.46060          1
O    81.28710  73.11128  67.73623     -429.46514      516.51932     -128.40526          1
H    81.92680  72.52554  68.18819     1981.82785      436.16847    -1818.18182          1
C    78.79106  72.93359  68.16819      630.79189     -468.94448       76.03230          1
H    78.71445  71.95692  67.63601    -1775.71861     2062.30353      131.89849          1
H    78.02539  72.92506  68.97943    -1855.14608       88.94118     1993.53760          1
H    78.50294  73.72611  67.43893    -1504.82350     -780.94249    -2132.90992          1
C    78.99534  74.67742  70.61054      288.57638      618.62814     -397.00270          1
O    79.16688  74.54791  71.80917      321.70495     -185.26452      574.37218          1
N    77.85714  74.98628  69.99581     -447.05792     -446.53431      367.76425          1
H    77.84101  75.05348  68.97988      350.31316     1975.63644    -1843.31522          1
C    76.65364  74.73265  70.72834     -511.23061      374.03917     -471.51480          1
H    76.93512  74.96440  71.78253     2234.79579     -898.70890    -1273.45190          1
C    75.50898  75.63937  70.08241      529.53781     -159.34528      563.72604          1
H    74.60718  75.52849  70.74411      654.59388     2347.94251     1217.51488          1
H    75.80247  76.71958  70.14362    -1526.55661     2103.42026      817.87394          1
O    75.15962  75.30212  68.72944     -554.84278       14.12789      399.58769          1
H    75.01877  74.29923  68.85072     1297.06236     2061.84854     1220.68812          1
C    76.21624  73.17939  70.52017     -662.15484     -412.13544      123.62687          1
O    75.34387  72.89169  69.70602     -540.01537     -317.05804      274.91456          1
N    76.96246  72.40206  71.25595     -202.76299     -690.00100     -131.49145          1
H    77.73007  72.78169  71.85138     -702.62132    -2617.37191      281.70413          1
C    76.47556  71.11310  71.66145     -491.73914      576.34187      222.73509          1
H    75.38116  71.20880  71.84040     1977.17978    -1823.58850     -434.68928          1
C    76.78591  70.08252  70.48551     -649.50797     -328.66843      306.11224          1
H    77.64946  70.44695  69.88901     2075.07803     1452.16490    -1004.45904          1
C    77.04485  68.63087  71.01330     -615.18735     -489.99689      -70.96352          1
H    77.26412  67.92366  70.17921    -1226.32661    -1724.38906    -1716.47170          1
H    77.91248  68.59787  71.71296     2381.64273     -969.84735      900.46860          1
H    76.16125  68.22812  71.56169     2122.74991     1702.23946     -141.36228          1
C    75.49890  69.95078  69.60294     -501.86074     -479.61276     -376.42482          1
H    75.67716  69.32001  68.69995      993.79261    -2246.95401    -1177.81305          1
H    74.65826  69.48522  70.17042       42.71279     -626.15391    -2651.37258          1
```

H	75.14469	70.96380	69.25207	-2026.39012	-1590.33709	-887.82922	1
C	77.37350	70.87263	72.98665	-672.89153	382.57394	-156.34072	1
O	78.28434	71.67656	73.21258	410.04627	221.86223	500.35950	1
N	76.97385	69.83774	73.69469	-378.11782	-443.79414	-441.10984	1
H	76.14543	69.36984	73.32423	2404.15973	-568.14894	1149.30085	1
C	77.84218	69.07534	74.53502	-544.54484	466.16019	331.29119	1
H	77.54693	68.03195	74.28714	-1409.80988	-170.17955	2325.32679	1
C	79.35454	69.37633	74.18409	-184.48284	116.78282	-758.89137	1
H	79.59150	69.07275	73.13847	1893.78641	1861.14652	611.04528	1
H	79.56724	70.47617	74.21951	-2170.79665	-974.19311	1327.50117	1
C	80.25370	68.61646	75.21128	389.51668	672.81878	-138.49152	1
H	79.80493	68.65839	76.22869	510.64577	1933.03327	1851.02484	1
H	80.28221	67.52918	74.97928	-2302.30869	-1105.72939	-948.89774	1
C	81.71188	69.20871	75.26574	-559.93427	-126.34590	542.30857	1
O	82.48098	68.81859	76.10587	-670.36003	-111.83166	76.36448	1
N	81.97058	70.14145	74.32087	-216.06729	-537.07037	446.51379	1
H	81.39577	70.46090	73.53230	-1251.96487	1074.12894	-2168.52500	1
H	82.89668	70.55840	74.17881	-2451.83023	-720.73851	944.84612	1
C	77.41086	69.30046	76.10106	546.24052	-563.88337	-85.11809	1
O	76.96742	68.31343	76.63145	71.70702	-307.40463	606.69673	1
N	77.68156	70.49239	76.65120	479.90863	-504.50129	222.86849	1
C	77.10189	70.87434	77.90307	75.51268	-600.91904	506.73700	1
H	76.86369	69.91359	78.41802	-2033.61562	1795.30103	254.89480	1
C	78.29247	71.73223	78.51923	523.79067	517.99274	284.45581	1
H	77.99190	72.35460	79.39241	1599.61665	-1127.00911	1895.98173	1
H	79.12879	71.08326	78.86261	-1881.83093	1275.82488	-1501.55016	1
C	78.69968	72.56366	77.26543	-600.05639	-404.05026	316.64485	1
H	77.93656	73.35040	77.03317	-2597.05260	496.36221	657.73249	1
H	79.67882	73.08389	77.36778	-1666.24518	1294.38985	-1723.90591	1
C	78.71637	71.40328	76.17943	493.57570	-129.95376	-602.56394	1
H	78.43279	71.70757	75.12539	-1469.08415	-637.84797	2204.22581	1
H	79.66221	70.81193	76.15585	-1038.97840	1068.88701	-2280.71789	1
C	75.76967	71.77604	77.59208	188.88100	241.05089	-727.87821	1
O	74.99836	71.46453	76.71704	531.88613	-49.19943	427.07960	1
N	75.63797	72.73954	78.50332	708.14833	26.93417	-179.73509	1
H	76.37909	72.85740	79.19341	-2232.69501	-1179.09831	1023.94816	1
C	74.82316	73.86280	78.14388	-738.32412	259.92548	104.42456	1
H	74.37890	73.53531	77.17449	2028.89123	1260.02983	1311.33324	1
C	73.73139	74.01634	79.28683	-258.65678	-345.52513	661.28459	1
H	74.20773	74.17392	80.27815	-1826.06090	-1801.46097	-918.64554	1
C	72.73754	75.17540	78.92748	-541.23400	531.92970	-218.41428	1
H	71.93218	75.28059	79.69219	1311.73218	-2219.18200	882.19023	1
H	73.25037	76.16766	78.85248	-1048.71298	1223.58118	-2196.97971	1
H	72.24301	74.99599	77.94375	419.06414	1903.92898	1903.44670	1
C	72.87332	72.70413	79.28571	-423.86431	152.66566	-648.55262	1
H	72.07598	72.72908	80.06457	105.68386	-2342.50080	1387.51238	1
H	72.37626	72.54668	78.30011	-1695.63603	-1484.73621	-1531.02793	1
H	73.50720	71.80682	79.47842	-1853.57121	-487.39031	1936.59343	1
C	75.84036	75.12419	77.95917	539.71428	-510.58232	267.58633	1
O	76.67898	75.06804	77.05708	-15.93758	-278.34085	-624.49402	1
N	75.61052	76.07861	78.82541	-676.81886	12.05292	276.17960	1
H	74.88107	75.89188	79.51233	-1694.44250	-373.84189	-2100.66098	1
C	76.28765	77.32250	78.88708	412.33078	-664.70360	108.35451	1
H	75.91633	77.76463	79.83870	1765.77476	1294.44366	1621.76594	1
C	77.84569	77.03713	78.88572	-179.66635	-721.95667	-264.73915	1
H	78.10793	76.29542	79.67286	-607.34922	2587.87032	-598.10018	1
H	78.16305	76.57403	77.92190	-359.69332	-1479.13954	-2259.74260	1
C	78.56198	78.40571	79.13441	-240.01076	616.02425	431.85322	1
H	78.42338	79.08854	78.26610	-1359.17131	782.54425	2227.99181	1
H	78.10239	78.92635	80.00429	-820.36667	85.81767	-2596.78715	1
C	80.09619	78.13991	79.41082	-513.07015	236.18570	-551.87280	1
H	80.22514	77.38711	80.22189	1816.11944	-1183.11722	-1650.94321	1

H	80.59587	77.71017	78.51199	-1800.04429	-1361.53473	1526.34643	1
C	80.74029	79.48665	79.81660	726.08972	-253.19732	-179.64713	1
H	80.96037	80.16877	78.97139	1224.27271	-456.51798	-2390.90304	1
H	80.19967	80.03422	80.61433	1206.40339	-1150.93514	-2154.90344	1
N	82.12947	79.17098	80.46743	329.70796	-633.68968	-155.67358	1
H	82.69008	80.01385	80.78474	1348.31305	1938.76845	1359.00643	1
H	82.12329	78.58404	81.35175	2096.92035	1446.54551	-966.48890	1
H	82.84111	78.65742	79.87273	-1197.07217	-2303.53719	827.28733	1
C	75.95214	78.29954	77.60393	-378.01186	-556.17301	-413.96481	1
O	75.68413	79.45811	77.80071	-409.44433	-307.55053	-453.30833	1
N	76.11816	77.66415	76.45609	-429.02477	524.70884	274.08502	1
H	76.42253	76.66127	76.44537	1706.34263	-1425.96036	1574.38926	1
C	75.93861	78.39666	75.24966	-45.25864	-626.22952	478.93267	1
H	76.06001	79.46547	75.54575	1988.63164	570.90773	1772.87287	1
C	77.05743	77.83593	74.27905	551.77128	-20.49526	-564.54996	1
H	78.05245	77.89105	74.77547	1434.61535	-1753.10253	1513.99471	1
H	76.88786	76.75277	74.08509	-714.67738	1819.09770	1898.36494	1
C	77.07747	78.62981	72.93218	446.99263	-555.77305	338.97221	1
H	76.11177	78.50946	72.38728	1953.68466	1892.25957	-161.67766	1
H	77.20102	79.72256	73.10618	-403.03785	2688.07032	-188.43591	1
C	78.28857	78.04275	72.10238	567.86262	335.74681	434.04421	1
H	79.28404	78.28028	72.60523	-1080.02275	1568.26081	-1948.78900	1
H	78.23213	76.92938	72.05559	-919.54108	2111.34692	1456.13463	1
C	78.25903	78.63370	70.67455	195.81897	173.33862	-745.11536	1
H	77.42947	78.27108	70.03471	1405.67949	-2301.21089	-390.08113	1
H	78.32742	79.73928	70.62287	-940.37328	-1118.32446	2299.72133	1
N	79.56719	78.11715	69.98910	319.26422	656.36007	-42.02424	1
H	79.71503	78.36422	68.96752	1961.03633	619.91968	1787.09422	1
H	80.47460	78.44938	70.42425	854.14343	-2156.80752	1429.08711	1
H	79.69728	77.06526	69.98246	1237.02905	-1378.50857	-1998.28500	1
C	74.45118	78.10896	74.61744	532.53319	-268.57236	517.55729	1
O	74.15347	78.61255	73.55882	266.16714	-290.73804	-558.88002	1
N	73.72147	77.30309	75.37851	-226.25352	640.49678	-270.32839	1
H	74.12566	77.00207	76.26590	2007.69063	-1035.27229	1523.50231	1
C	72.36713	77.06312	75.03915	-540.76542	-573.33303	49.50000	1
H	72.15025	77.66921	74.12873	-2374.20950	-1036.76023	843.75774	1
C	72.24730	75.47872	74.80661	-77.09082	666.87422	-415.84136	1
H	72.55157	74.95833	75.75574	-985.81834	617.19825	2463.91927	1
O	73.09322	74.93374	73.77871	293.29899	-391.65602	-477.80926	1
H	73.66423	74.33596	74.30234	2528.72714	-681.76262	-751.27122	1
C	70.70294	75.29280	74.58088	252.47363	-605.14473	440.05134	1
H	70.43441	74.21753	74.46439	-2052.65063	1456.49051	-1043.51830	1
H	70.10383	75.69202	75.43222	-2152.98800	-1661.97574	-161.70652	1
H	70.36080	75.81877	73.65967	-473.12726	-1584.27339	2165.61725	1
C	71.48652	77.43742	76.36584	360.36307	488.46713	505.10056	1
O	71.92329	77.20859	77.47433	-200.08817	-528.03552	-385.82850	1
N	70.27502	77.87103	76.03485	-597.54095	-226.44775	355.20479	1
H	70.03428	77.99677	75.05268	2179.23655	-1300.70981	-991.33835	1
C	69.41004	78.21098	77.10979	-687.68921	297.39807	-249.45179	1
H	70.09826	78.40935	77.96406	-349.16256	-2467.37713	-1101.72831	1
C	68.49575	79.45160	76.67469	672.47345	361.54328	-201.62880	1
H	67.98611	79.25256	75.70460	1541.41502	582.18425	2169.97026	1
H	67.70019	79.61514	77.43776	-2534.67717	60.25153	997.72295	1
C	69.41110	80.67957	76.57765	574.26774	-389.43119	-377.02542	1
C	69.84343	81.32014	77.77789	688.82679	-287.33580	-257.96133	1
H	69.51704	80.94748	78.76593	331.14182	1470.38163	2269.80013	1
C	69.85502	81.15026	75.30539	-383.52382	-675.04808	144.24917	1
H	69.53612	80.64551	74.37567	4.09847	1517.59662	2262.86397	1
C	70.69311	82.42087	77.67759	-134.76730	428.45285	-649.50303	1
H	71.03180	82.91390	78.60567	-903.23433	1600.49020	2011.53387	1
C	70.70476	82.25799	75.25795	-335.53281	-615.00040	-364.39058	1
H	71.04175	82.61435	74.26887	1596.45398	-1369.33862	1732.02659	1

213	C	71.15121	82.93546	76.42991	585.48547	-340.24194	-406.23938	1
214	O	72.00475	84.06834	76.39071	-419.15649	-173.04408	-511.94141	1
215	H	72.16626	84.12241	75.41943	-1071.84350	1559.71507	-1960.13012	1
216	C	68.39279	76.97852	77.45311	291.41878	-183.13091	710.72246	1
217	O	68.22070	76.66253	78.60955	379.87695	523.47396	-222.23622	1
218	N	67.80327	76.44098	76.36999	564.30591	373.09380	277.24371	1
219	H	68.02051	76.92135	75.49634	-120.59931	-2436.07824	-1214.35098	1
220	C	66.50019	75.89096	76.66329	404.07514	-299.37480	608.84009	1
221	H	66.18661	76.41996	77.59320	-1071.07882	-841.69025	2359.66467	1
222	C	65.57974	76.15815	75.41129	-683.12807	68.71770	-390.13096	1
223	H	65.50971	77.24912	75.19888	2068.88918	1596.58590	770.89603	1
224	H	66.01901	75.70283	74.49505	1379.86429	2342.49779	-179.85638	1
225	C	64.16813	75.55471	75.70218	-176.39967	537.49466	550.97268	1
226	H	64.23932	74.46029	75.89342	-396.28051	1942.22168	1869.33314	1
227	H	63.72649	75.98979	76.62606	-94.44700	586.00678	-2659.20016	1
228	C	63.25643	75.86416	74.42742	-291.69381	-718.18079	-150.72929	1
229	H	63.01603	76.94244	74.24556	-2303.38776	1089.76242	964.62032	1
230	H	63.62834	75.42826	73.46671	884.16729	2568.80533	-207.74283	1
231	N	62.06344	75.19311	74.72884	-594.49522	-252.81478	342.29276	1
232	H	62.08216	74.56950	75.52092	-1735.17426	2084.05705	-263.71605	1
233	C	60.98296	75.30026	74.06805	595.23177	-271.52487	442.22352	1
234	N	60.97143	76.15867	72.96319	585.53813	-424.79750	-105.81303	1
235	H	61.72171	76.73108	72.57845	959.88797	1327.56823	-2177.11905	1
236	H	60.16587	76.31934	72.35757	1578.48647	16.27378	-2220.76233	1
237	N	59.97511	74.49011	74.61044	-452.17763	-390.33541	-421.51853	1
238	H	59.98437	73.85443	75.40665	470.26107	1974.25629	-1817.92087	1
239	H	59.03587	74.42872	74.21548	1856.67307	-1573.97321	-1224.35416	1
240	C	66.64467	74.28664	76.94019	-531.47271	-581.45780	-55.06206	1
241	O	65.94099	73.78404	77.81751	-243.00837	192.73507	609.52485	1
242	N	67.43669	73.69165	76.06047	343.49288	-644.25607	38.09164	1
243	H	67.81237	74.42517	75.45506	-1691.16043	-1535.97525	-1484.73099	1
244	C	68.25191	72.55765	76.35956	542.66492	350.14337	454.42563	1
245	H	68.99050	72.55449	75.52724	1209.57979	-2396.95920	463.86744	1
246	C	68.92843	72.82066	77.77849	-737.14309	145.93010	242.71942	1
247	H	68.14734	72.97361	78.55427	-1050.18303	159.36419	-2509.06125	1
248	C	69.83440	71.58824	78.11442	-706.77696	-252.88343	-245.16230	1
249	H	70.32007	71.69223	79.11246	-1624.64039	-1665.49085	1417.86935	1
250	H	69.24617	70.64056	78.12772	2566.10521	598.51109	693.22305	1
251	H	70.64448	71.45916	77.35826	-1972.69758	107.10018	-1876.34312	1
252	C	69.85331	74.08066	77.69058	412.85148	672.58832	-27.69222	1
253	H	70.36642	74.27511	78.66049	1447.02277	1762.76533	-1490.78944	1
254	H	70.64017	73.96072	76.90932	1320.77379	-145.20455	2378.68491	1
255	H	69.26625	74.99961	77.44095	-1512.10848	1470.52031	1724.75147	1
256	C	67.46605	71.14819	76.47156	-607.11113	499.56689	73.73769	1
257	O	67.96620	70.15559	75.93713	357.97126	460.81534	356.68785	1
258	N	66.38652	71.19857	77.21365	536.55407	-368.62270	332.76306	1
259	H	66.05675	72.11971	77.61181	524.38866	2297.69985	1367.20941	1
260	C	66.06178	69.99212	77.92739	46.50277	-774.29190	-147.97904	1
261	H	67.05023	69.51108	78.10195	1676.51875	1883.29066	1032.55549	1
262	C	65.26333	70.38865	79.27166	-559.41456	-34.19198	-556.30433	1
263	H	64.39803	71.04228	79.00613	-64.66664	395.18769	2695.05373	1
264	H	64.82456	69.47463	79.73961	-2703.44278	-60.40056	333.79418	1
265	C	66.18115	71.09070	80.23649	-519.76435	377.31045	459.42366	1
266	H	66.70736	71.90891	79.69584	-1746.74323	1667.50644	-1261.73691	1
267	C	65.38088	71.71115	81.37539	570.95554	16.46261	-545.27700	1
268	H	66.04581	72.23147	82.11226	2391.58273	28.05277	-1305.07271	1
269	H	64.64697	72.46976	80.99877	175.75588	917.63458	2559.43867	1
270	H	64.79982	70.93801	81.94144	-1063.97851	-2155.34650	1283.00462	1
271	C	67.21786	70.13277	80.80845	193.76014	2.62946	-765.53141	1
272	H	67.88484	70.64149	81.55127	507.39394	-846.55087	2539.60089	1
273	H	66.73356	69.27018	81.33507	1337.57841	1818.19054	1526.01994	1
274	H	67.87728	69.70558	80.01033	1100.88635	-1172.91350	-2199.08909	1

```
C    65.14165  69.09743  76.97395     -549.21115      475.64486     -309.38218          1
O    64.05253  68.73092  77.32493      556.06106     -390.37755      -78.24250          1
O    65.71674  68.72433  75.77121     -572.27940     -310.19032     -209.75862          1
H    66.62853  69.27183  75.81667    -1380.08193     2212.14486      790.85944          1
```

Listing 79: C00079.xmolout

```
      276
  150.0000  150.0000  150.0000     0.0000     0.0000     0.0000***********    0.6012     2405
      0.2500    300.00    0.00    0.00    0.00   -1293.5268       0.0000    300.0000
H    75.57644  83.27698  68.29153    -2043.14101    -1757.64893      399.89339          1
N    75.74152  83.95922  69.03344      426.75047      490.45733     -334.42467          1
H    75.87190  83.38944  69.88857      442.25559    -1110.30854    -2448.52907          1
C    76.83404  84.81634  68.77747      415.32972     -666.39213       83.73172          1
H    77.14503  84.64159  67.72401      879.32219     2491.00447      667.35213          1
C    76.40396  86.34689  69.04126       31.35357      477.41777      628.23362          1
H    76.18776  86.48428  70.12771     1168.38489     2240.25337    -1019.71136          1
H    77.25091  87.03565  68.80758     -360.43770     2534.90498      931.67112          1
C    75.18852  86.69121  68.22131      463.18208      512.70242     -382.34388          1
H    74.38974  85.95382  68.46328     1725.29117     1863.52247     -987.07854          1
C    74.69096  88.08680  68.57778      710.42801     -144.27427     -313.15367          1
H    73.76286  88.35336  68.00951      364.20195    -1033.13725     2494.72524          1
H    74.45033  88.17505  69.66854     2567.61904      335.80679      847.48802          1
H    75.45805  88.86926  68.34369    -2587.99029      680.92867     -512.16411          1
C    75.47425  86.62155  66.72749      268.17616      741.64379      -40.42634          1
H    74.57847  86.91659  66.12215     1283.54314    -1945.37420    -1411.27812          1
H    76.31222  87.30517  66.43449    -1258.57796     1317.15249    -2026.02085          1
H    75.76145  85.58669  66.40866    -2502.84526      909.80597      575.92391          1
C    77.89338  84.29995  69.89291      619.36578     -458.64843     -172.09273          1
O    77.54047  83.39157  70.64918      -81.40433      433.87611      522.34594          1
N    79.00642  84.99163  69.82843      175.23996     -622.20190      341.55523          1
H    79.01382  85.72077  69.11420     -876.57256     -303.22879    -2561.90213          1
C    80.17415  84.81022  70.61741     -571.88214     -534.10783     -106.15033          1
H    80.56874  85.84392  70.71247    -2713.20841      -16.25800     -248.80809          1
C    79.79376  84.15378  72.02412      166.60354      288.16399     -716.09568          1
H    79.27701  83.17053  71.82272    -2097.85574     1636.78449     -586.18044          1
O    78.86909  84.94054  72.80602     -317.80580     -363.38227      484.43082          1
H    78.03248  84.46795  72.59717     1863.74748    -1451.84923     1357.29467          1
C    81.18459  83.85494  72.69363      503.80731      517.99032      318.51606          1
H    81.07595  83.26238  73.63533      640.61020    -2157.10410    -1536.29104          1
H    81.85946  83.27655  72.01716     1687.88455      465.60692     2087.56452          1
H    81.71524  84.79568  72.96659    -1293.07895    -1899.88581    -1463.57452          1
C    81.19342  83.85517  69.77161     -153.07550     -733.68902      248.70981          1
O    80.79299  82.83661  69.27087      186.15459     -480.78787     -449.34387          1
N    82.44222  84.34061  69.81716     -487.53139      543.18517       42.03850          1
C    83.52915  83.62350  69.21995     -699.35871       69.34393      360.10701          1
H    83.14448  83.17947  68.27407        2.81126     -221.99807    -2715.58072          1
C    84.62650  84.76032  69.05552     -507.46439     -592.59194      122.07761          1
H    85.66147  84.35545  68.99570    -1975.08116      525.87560    -1801.71556          1
H    84.46524  85.35662  68.12979     1588.27743    -1913.22100     1113.83566          1
C    84.37597  85.61963  70.33209     -613.62442     -457.55993     -194.14490          1
H    84.77622  85.10909  71.23825     1707.54637     -721.27992     1996.92523          1
H    84.83779  86.63231  70.29406      758.23357     2100.81767     1560.55011          1
C    82.78658  85.65207  70.34766     -352.41524      704.96076       49.21465          1
H    82.33125  85.77172  71.35732     1714.76886    -2070.42778      443.35792          1
H    82.33134  86.39316  69.64794     1224.15040     1801.50881     1636.97633          1
C    84.07191  82.49440  70.27505      566.85117      537.05831      117.62814          1
O    84.32402  82.79073  71.43937     -550.26779     -406.08867        4.21666          1
N    84.20321  81.35285  69.63784      566.34832     -425.77725      180.18749          1
H    83.87905  81.38642  68.67113      644.44502     2646.80550      -52.73430          1
C    85.06094  80.28637  70.00954      612.34801     -281.48662      411.56221          1
H    85.10479  79.66542  69.08701     1667.23498    -1490.75142     1556.16753          1
```

C	86.47995	80.92334	70.38804	-714.45746	-175.43074	286.98927	1
H	86.32705	81.62782	71.25327	-787.09484	1763.03619	-1922.46060	1
O	87.11583	81.69481	69.35231	-429.46514	516.51932	-128.40526	1
H	87.16288	82.57425	69.77774	1981.82785	436.16847	-1818.18182	1
C	87.26932	79.65098	70.86818	630.79189	-468.94448	76.03230	1
H	88.27729	79.91832	71.26208	-1775.71861	2062.30353	131.89849	1
H	86.72809	79.10464	71.67768	-1855.14608	88.94118	1993.53760	1
H	87.42283	78.92996	70.03160	-1504.82350	-780.94249	-2132.90992	1
C	84.48867	79.42322	71.27802	288.57638	618.62814	-397.00270	1
O	84.65272	78.22817	71.28218	321.70495	-185.26452	574.37218	1
N	84.06264	80.20619	72.26556	-447.05792	-446.53431	367.76425	1
H	84.05139	81.24901	72.12221	350.31316	1975.63644	-1843.31522	1
C	83.11960	79.60992	73.16140	-511.23061	374.03917	-471.51480	1
H	83.48389	78.55821	73.21891	2234.79579	-898.70890	-1273.45190	1
C	83.27282	80.45119	74.51106	529.53781	-159.34528	563.72604	1
H	82.83773	79.84377	75.34903	654.59388	2347.94251	1217.51488	1
H	84.37339	80.53028	74.71317	-1526.55661	2103.42026	817.87394	1
O	82.64422	81.76200	74.50558	-554.84278	14.12789	399.58769	1
H	83.31931	82.25795	74.00359	1297.06236	2061.84854	1220.68812	1
C	81.65122	79.69790	72.47480	-662.15484	-412.13544	123.62687	1
O	81.51203	79.56833	71.27430	-540.01537	-317.05804	274.91456	1
N	80.64165	79.68466	73.40277	-202.76299	-690.00100	-131.49145	1
H	81.16359	79.88666	74.26581	-702.62132	-2617.37191	281.70413	1
C	79.53883	80.59182	73.12658	-491.73914	576.34187	222.73509	1
H	80.00636	81.57931	72.93756	1977.17978	-1823.58850	-434.68928	1
C	78.66898	80.07286	71.91969	-649.50797	-328.66843	306.11224	1
H	78.61982	78.96290	71.95162	2075.07803	1452.16490	-1004.45904	1
C	77.24396	80.71841	72.04948	-615.18735	-489.99689	-70.96352	1
H	76.50809	80.22485	71.37230	-1226.32661	-1724.38906	-1716.47170	1
H	76.84564	80.63892	73.09083	2381.64273	-969.84735	900.46860	1
H	77.26840	81.81739	71.77261	2122.74991	1702.23946	-141.36228	1
C	79.10629	80.57408	70.49665	-501.86074	-479.61276	-376.42482	1
H	78.29828	80.41626	69.74357	993.79261	-2246.95401	-1177.81305	1
H	79.33891	81.66812	70.49449	42.71279	-626.15391	-2651.37258	1
H	80.02514	80.03688	70.11248	-2026.39012	-1590.33709	-887.82922	1
C	78.61309	80.56490	74.45865	-672.89153	382.57394	-156.34072	1
O	78.01649	81.55497	74.82702	410.04627	221.86223	500.35950	1
N	78.45410	79.29801	74.87981	-378.11782	-443.79414	-441.10984	1
H	79.11455	78.65852	74.43496	2404.15973	-568.14894	1149.30085	1
C	78.06542	79.07061	76.25493	-544.54484	466.16019	331.29119	1
H	78.84035	79.61518	76.83605	-1409.80988	-170.17955	2325.32679	1
C	76.65006	79.73583	76.44288	-184.48284	116.78282	-758.89137	1
H	76.70768	80.84213	76.26101	1893.78641	1861.14652	611.04528	1
H	75.94761	79.35578	75.66548	-2170.79665	-974.19311	1327.50117	1
C	76.11212	79.44328	77.88324	389.51668	672.81878	-138.49152	1
H	76.37550	78.40987	78.20198	510.64577	1933.03327	1851.02484	1
H	76.62578	80.08910	78.62869	-2302.30869	-1105.72939	-948.89774	1
C	74.55003	79.63498	77.97473	-559.93427	-126.34590	542.30857	1
O	73.95356	79.26332	78.95228	-670.36003	-111.83166	76.36448	1
N	74.00804	80.23039	76.88832	-216.06729	-537.07037	446.51379	1
H	74.47691	80.64679	76.07582	-1251.96487	1074.12894	-2168.52500	1
H	73.02059	80.49737	76.81691	-2451.83023	-720.73851	944.84612	1
C	78.21178	77.46168	76.43021	546.24052	-563.88337	-85.11809	1
O	79.32852	77.09321	76.69650	71.70702	-307.40463	606.69673	1
N	77.12084	76.70512	76.21944	479.90863	-504.50129	222.86849	1
C	77.23992	75.28058	76.20564	75.51268	-600.91904	506.73700	1
H	78.07419	74.99413	76.88701	-2033.61562	1795.30103	254.89480	1
C	75.76456	74.86687	76.64134	523.79067	517.99274	284.45581	1
H	75.52205	73.80414	76.41392	1599.61665	-1127.00911	1895.98173	1
H	75.60567	75.00273	77.73656	-1881.83093	1275.82488	-1501.55016	1
C	74.90454	75.87828	75.82377	-600.05639	-404.05026	316.64485	1
H	74.71907	75.50515	74.78782	-2597.05260	496.36221	657.73249	1

117	H	73.90166	76.05248	76.27446	-1666.24518	1294.38985	-1723.90591	1
118	C	75.81067	77.18487	75.80761	493.57570	-129.95376	-602.56394	1
119	H	75.94987	77.64096	74.79831	-1469.08415	-637.84797	2204.22581	1
120	H	75.50259	77.97358	76.53669	-1038.97840	1068.88701	-2280.71789	1
121	C	77.46562	74.84910	74.64315	188.88100	241.05089	-727.87821	1
122	O	76.62973	75.11192	73.78821	531.88613	-49.19943	427.07960	1
123	N	78.60099	74.20322	74.48523	708.14833	26.93417	-179.73509	1
124	H	79.15535	74.06555	75.32678	-2232.69501	-1179.09831	1023.94816	1
125	C	79.00465	73.72338	73.16520	-738.32412	259.92548	104.42456	1
126	H	78.54666	74.49746	72.50291	2028.89123	1260.02983	1311.33324	1
127	C	80.52333	73.65779	73.11156	-258.65678	-345.52513	661.28459	1
128	H	80.86198	72.94239	73.89379	-1826.06090	-1801.46097	-918.64554	1
129	C	81.01284	73.15040	71.76820	-541.23400	531.92970	-218.41428	1
130	H	82.13559	73.08702	71.73040	1311.73218	-2219.18200	882.19023	1
131	H	80.60961	72.12089	71.54700	-1048.71298	1223.58118	-2196.97971	1
132	H	80.68902	73.82455	70.92758	419.06414	1903.92898	1903.44670	1
133	C	81.14930	75.00623	73.41647	-423.86431	152.66566	-648.55262	1
134	H	82.27240	74.95030	73.44392	105.68386	-2342.50080	1387.51238	1
135	H	80.87441	75.77398	72.64127	-1695.63603	-1484.73621	-1531.02793	1
136	H	80.80696	75.40759	74.41067	-1853.57121	-487.39031	1936.59343	1
137	C	78.24914	72.25122	72.97338	539.71428	-510.58232	267.58633	1
138	O	78.90009	71.25715	72.77154	-15.93758	-278.34085	-624.49402	1
139	N	76.95117	72.40914	73.09051	-676.81886	12.05292	276.17960	1
140	H	76.57825	73.36976	73.21540	-1694.44250	-373.84189	-2100.66098	1
141	C	76.04686	71.33103	72.78991	412.33078	-664.70360	108.35451	1
142	H	76.11231	71.27737	71.68162	1765.77476	1294.44366	1621.76594	1
143	C	76.55470	70.00373	73.46469	-179.66635	-721.95667	-264.73915	1
144	H	77.59782	69.75427	73.12829	-607.34922	2587.87032	-598.10018	1
145	H	76.62455	70.12385	74.56998	-359.69332	-1479.13954	-2259.74260	1
146	C	75.56711	68.84863	73.07596	-240.01076	616.02425	431.85322	1
147	H	74.52326	69.09530	73.37257	-1359.17131	782.54425	2227.99181	1
148	H	75.54427	68.69924	71.97362	-820.36667	85.81767	-2596.78715	1
149	C	76.06994	67.54477	73.81356	-513.07015	236.18570	-551.87280	1
150	H	77.11097	67.29329	73.50537	1816.11944	-1183.11722	-1650.94321	1
151	H	76.11143	67.70524	74.91562	-1800.04429	-1361.53473	1526.34643	1
152	C	75.10126	66.39036	73.46541	726.08972	-253.19732	-179.64713	1
153	H	74.07720	66.50823	73.87244	1224.27271	-456.51798	-2390.90304	1
154	H	75.05382	66.13609	72.38781	1206.40339	-1150.93514	-2154.90344	1
155	N	75.65754	65.09600	74.14978	329.70796	-633.68968	-155.67358	1
156	H	75.08564	64.21451	74.00410	1348.31305	1938.76845	1359.00643	1
157	H	76.62169	64.77001	73.84818	2096.92035	1446.54551	-966.48890	1
158	H	75.75313	65.11153	75.20679	-1197.07217	-2303.53719	827.28733	1
159	C	74.57872	71.84772	73.29033	-378.01186	-556.17301	-413.96481	1
160	O	74.00063	72.69579	72.65086	-409.44433	-307.55053	-453.30833	1
161	N	74.17781	71.24843	74.40541	-429.02477	524.70884	274.08502	1
162	H	74.75652	70.55744	74.88026	1706.34263	-1425.96036	1574.38926	1
163	C	72.92414	71.60772	74.96892	-45.25864	-626.22952	478.93267	1
164	H	72.65441	72.59946	74.53600	1988.63164	570.90773	1772.87287	1
165	C	71.91732	70.45267	74.55091	551.77128	-20.49526	-564.54996	1
166	H	71.90951	70.33061	73.44496	1434.61535	-1753.10253	1513.99471	1
167	H	72.25861	69.47427	74.95645	-714.67738	1819.09770	1898.36494	1
168	C	70.48914	70.80519	75.07908	446.99263	-555.77305	338.97221	1
169	H	70.49736	70.87187	76.19345	1953.68466	1892.25957	-161.67766	1
170	H	70.16991	71.80713	74.71407	-403.03785	2688.07032	-188.43591	1
171	C	69.47128	69.69373	74.60032	567.86262	335.74681	434.04421	1
172	H	69.38798	69.67750	73.48968	-1080.02275	1568.26081	-1948.78900	1
173	H	69.82279	68.67864	74.89347	-919.54108	2111.34692	1456.13463	1
174	C	68.10064	69.99995	75.25858	195.81897	173.33862	-745.11536	1
175	H	68.09895	69.91634	76.37894	1405.67949	-2301.21089	-390.08113	1
176	H	67.64361	70.96359	74.94850	-940.37328	-1118.32446	2299.72133	1
177	N	67.07814	68.93367	74.80435	319.26422	656.36007	-42.02424	1
178	H	66.05613	69.22288	75.05206	1961.03633	619.91968	1787.09422	1

```
H   67.02800  68.76674  73.75262     854.14343   -2156.80752    1429.08711          1
H   67.20976  67.93279  75.24021    1237.02905   -1378.50857   -1998.28500          1
C   73.15094  71.55048  76.59427     532.53319    -268.57236     517.55729          1
O   73.81414  70.67588  77.10350     266.16714    -290.73804    -558.88002          1
N   72.47957  72.53338  77.18462    -226.25352     640.49678    -270.32839          1
H   71.92107  73.17388  76.62299    2007.69063   -1035.27229    1523.50231          1
C   72.54751  72.60443  78.60197    -540.76542    -573.33303      49.50000          1
H   73.48941  72.06418  78.85180   -2374.20950   -1036.76023     843.75774          1
C   72.48848  74.12552  79.07038     -77.09082     666.87422    -415.84136          1
H   71.47493  74.54453  78.82462    -985.81834     617.19825    2463.91927          1
O   73.45074  75.01891  78.47543     293.29899    -391.65602    -477.80926          1
H   72.87107  75.74575  78.17317    2528.72714    -681.76262    -751.27122          1
C   72.61101  73.94404  80.62925     252.47363    -605.14473     440.05134          1
H   72.46818  74.91095  81.16480   -2052.65063    1456.49051   -1043.51830          1
H   71.85317  73.22885  81.02375   -2152.98800   -1661.97574    -161.70652          1
H   73.61410  73.55304  80.91692    -473.12726   -1584.27339    2165.61725          1
C   71.18924  71.84098  79.17558     360.36307     488.46713     505.10056          1
O   70.12579  72.40920  79.22451    -200.08817    -528.03552    -385.82850          1
N   71.56362  70.64867  79.60333    -597.54095    -226.44775     355.20479          1
H   72.54854  70.42007  79.46303    2179.23655   -1300.70981    -991.33835          1
C   70.68104  69.60656  79.97484    -687.68921     297.39807    -249.45179          1
H   71.31756  68.87829  80.52362    -349.16256   -2467.37713   -1101.72831          1
C   69.46048  70.18970  80.83018     672.47345     361.54328    -201.62880          1
H   68.89308  70.94288  80.22829    1541.41502     582.18425    2169.97026          1
H   68.74450  69.37359  81.08518   -2534.67717      60.25153     997.72295          1
C   70.05895  70.82527  82.09364     574.26774    -389.43119    -377.02542          1
C   70.64897  70.00850  83.10453     688.82679    -287.33580    -257.96133          1
H   70.63920  68.90671  83.01911     331.14182    1470.38163    2269.80013          1
C   70.08166  72.24755  82.20626    -383.52382    -675.04808     144.24917          1
H   69.64181  72.87763  81.40952       4.09847    1517.59662    2262.86397          1
C   71.25625  70.63476  84.19264    -134.76730     428.45285    -649.50303          1
H   71.72035  70.00612  84.97250    -903.23433    1600.49020    2011.53387          1
C   70.70034  72.82772  83.31603    -335.53281    -615.00040    -364.39058          1
H   70.72033  73.92924  83.38733    1596.45398   -1369.33862    1732.02659          1
C   71.31024  72.05093  84.34356     585.48547    -340.24194    -406.23938          1
O   71.95091  72.62996  85.46998    -419.15649    -173.04408    -511.94141          1
H   71.79779  73.58473  85.27570   -1071.84350    1559.71507   -1960.13012          1
C   70.02945  68.96454  78.61864     291.41878    -183.13091     710.72246          1
O   69.49705  69.63136  77.75411     379.87695     523.47396    -222.23622          1
N   70.09302  67.64474  78.71573     564.30591     373.09380     277.24371          1
H   70.56935  67.22725  79.51373    -120.59931   -2436.07824   -1214.35098          1
C   69.73448  66.88959  77.54083     404.07514    -299.37480     608.84009          1
H   70.21256  67.43223  76.69371   -1071.07882    -841.69025    2359.66467          1
C   70.26268  65.40902  77.73935    -683.12807      68.71770    -390.13096          1
H   71.35035  65.39566  77.97604    2068.88918    1596.58590     770.89603          1
H   69.75850  64.92562  78.60624    1379.86429    2342.49779    -179.85638          1
C   69.96053  64.63897  76.41666    -176.39967     537.49466     550.97268          1
H   68.88501  64.74542  76.14053    -396.28051    1942.22168    1869.33314          1
H   70.53204  65.06775  75.56358     -94.44700     586.00678   -2659.20016          1
C   70.35103  63.10979  76.66397    -291.69381    -718.18079    -150.72929          1
H   71.43568  62.91180  76.86017   -2303.38776    1089.76242     964.62032          1
H   69.76062  62.58411  77.45710     884.16729    2568.80533    -207.74283          1
N   70.02330  62.52305  75.43266    -594.49522    -252.81478     342.29276          1
H   69.62558  63.12974  74.73259   -1735.17426    2084.05705    -263.71605          1
C   70.18660  61.29464  75.14972     595.23177    -271.52487     442.22352          1
N   70.73705  60.46495  76.13286     585.53813    -424.79750    -105.81303          1
H   71.03298  60.69462  77.08064     959.88797    1327.56823   -2177.11905          1
H   70.90843  59.46480  76.02490    1578.48647      16.27378   -2220.76233          1
N   69.75921  61.01734  73.84328    -452.17763    -390.33541    -421.51853          1
H   69.34884  61.62097  73.13254     470.26107    1974.25629   -1817.92087          1
H   69.79802  60.08531  73.42917    1856.67307   -1573.97321   -1224.35416          1
C   68.12279  66.88707  77.39414    -531.47271    -581.45780     -55.06206          1
```

```
O    67.60643  66.68840  76.28761     -243.00837      192.73507      609.52485          1
N    67.52320  66.97413  78.56491      343.49288     -644.25607       38.09164          1
H    68.17877  67.13755  79.33146    -1691.16043    -1535.97525    -1484.73099          1
C    66.14259  67.41727  78.67332      542.66492      350.14337      454.42563          1
H    65.67331  67.09995  77.71067     1209.57979    -2396.95920      463.86744          1
C    65.51232  66.85493  79.93874     -737.14309      145.93010      242.71942          1
H    66.09916  67.24470  80.80160    -1050.18303      159.36419    -2509.06125          1
C    64.08087  67.33629  80.08908     -706.77696     -252.88343     -245.16230          1
H    63.61956  66.98164  81.05137    -1624.64039    -1665.49085     1417.86935          1
H    64.02336  68.46031  80.08521     2566.10521      598.51109      693.22305          1
H    63.42940  66.96709  79.24987    -1972.69758      107.10018    -1876.34312          1
C    65.56756  65.34038  79.99303      412.85148      672.58832      -27.69222          1
H    65.18164  64.94488  80.97338     1447.02277     1762.76533    -1490.78944          1
H    64.94697  64.87542  79.17813     1320.77379     -145.20455     2378.68491          1
H    66.61947  64.96187  79.86594    -1512.10848     1470.52031     1724.75147          1
C    66.19185  69.07504  78.90192     -607.11113      499.56689       73.73769          1
O    66.44759  69.54419  79.98570      357.97126      460.81534      356.68785          1
N    65.93392  69.68708  77.75387      536.55407     -368.62270      332.76306          1
H    65.76741  69.04840  76.97472      524.38866     2297.69985     1367.20941          1
C    65.12731  70.88497  77.81090       46.50277     -774.29190     -147.97904          1
H    64.33600  70.65921  78.56012     1676.51875     1883.29066     1032.55549          1
C    66.06775  72.10306  78.29307     -559.41456      -34.19198     -556.30433          1
H    67.13869  71.81397  78.15881      -64.66664      395.18769     2695.05373          1
H    65.90064  73.00829  77.66091    -2703.44278      -60.40056      333.79418          1
C    65.77253  72.40818  79.73778     -519.76435      377.31045      459.42366          1
H    65.81037  71.44253  80.31282    -1746.74323     1667.50644    -1261.73691          1
C    66.83967  73.33692  80.30318      570.95554       16.46261     -545.27700          1
H    66.69589  73.51305  81.40076     2391.58273       28.05277    -1305.07271          1
H    67.86863  72.91369  80.16256      175.75588      917.63458     2559.43867          1
H    66.82271  74.33788  79.80013    -1063.97851    -2155.34650     1283.00462          1
C    64.39951  73.04446  79.89932      193.76014        2.62946     -765.53141          1
H    64.18591  73.30367  80.96837      507.39394     -846.55087     2539.60089          1
H    64.31402  73.99215  79.30695     1337.57841     1818.19054     1526.01994          1
H    63.57761  72.36579  79.55416     1100.88635    -1172.91350    -2199.08909          1
C    64.56320  71.12702  76.35038     -549.21115      475.64486     -309.38218          1
O    64.92153  70.45966  75.38859      556.06106     -390.37755      -78.24250          1
O    63.63119  72.16416  76.18959     -572.27940     -310.19032     -209.75862          1
H    63.58045  72.47397  77.14328    -1380.08193     2212.14486      790.85944          1
```

Listing 80: C00080.xmolout

```
      276
  150.0000  150.0000  150.0000     0.0000     0.0000     0.0000***********    2.7750    11100
       0.2500     300.00    0.00    0.00   0.00   -1293.5263       0.0000     300.0000
H    79.02095  77.29813  83.72580    -2043.14101    -1757.64893      399.89339          1
N    79.97342  77.03808  83.47662      426.75047      490.45733     -334.42467          1
H    79.83678  76.53445  82.60082      442.25559    -1110.30854    -2448.52907          1
C    80.86905  78.12254  83.34954      415.32972     -666.39213       83.73172          1
H    80.53529  78.92242  84.04633      879.32219     2491.00447      667.35213          1
C    82.35210  77.56385  83.66986       31.35357      477.41777      628.23362          1
H    82.52957  76.63876  83.07163     1168.38489     2240.25337    -1019.71136          1
H    83.13159  78.30125  83.35513     -360.43770     2534.90498      931.67112          1
C    82.43414  77.27966  85.14531      463.18208      512.70242     -382.34388          1
H    81.49781  76.75553  85.44473     1725.29117     1863.52247     -987.07854          1
C    83.61015  76.36667  85.46815      710.42801     -144.27427     -313.15367          1
H    83.61630  76.07052  86.54875      364.20195    -1033.13725     2494.72524          1
H    83.58049  75.42249  84.86526     2567.61904      335.80679      847.48802          1
H    84.59191  76.86300  85.26138    -2587.99029      680.92867     -512.16411          1
C    82.55670  78.57079  85.94400      268.17616      741.64379      -40.42634          1
H    82.64891  78.36928  87.04235     1283.54314    -1945.37420    -1411.27812          1
H    83.45943  79.16011  85.63820    -1258.57796     1317.15249    -2026.02085          1
H    81.66516  79.23412  85.80319    -2502.84526      909.80597      575.92391          1
```

C	80.85277	78.54700	81.78483	619.36578	-458.64843	-172.09273	1
O	80.78575	77.72534	80.86771	-81.40433	433.87611	522.34594	1
N	81.00521	79.84985	81.72878	175.23996	-622.20190	341.55523	1
H	81.01675	80.37759	82.60007	-876.57256	-303.22879	-2561.90213	1
C	81.06251	80.48489	80.45296	-571.88214	-534.10783	-106.15033	1
H	80.40883	79.81556	79.84879	-2713.20841	-16.25800	-248.80809	1
C	80.48240	81.96823	80.63144	166.60354	288.16399	-716.09568	1
H	81.21341	82.57499	81.23105	-2097.85574	1636.78449	-586.18044	1
O	79.22000	82.05018	81.31557	-317.80580	-363.38227	484.43082	1
H	79.45284	82.62570	82.07108	1863.74748	-1451.84923	1357.29467	1
C	80.47248	82.50298	79.15317	503.80731	517.99032	318.51606	1
H	80.10472	83.55417	79.10443	640.61020	-2157.10410	-1536.29104	1
H	81.48935	82.48637	78.69350	1687.88455	465.60692	2087.56452	1
H	79.80433	81.89041	78.50418	-1293.07895	-1899.88581	-1463.57452	1
C	82.58136	80.48249	79.85394	-153.07550	-733.68902	248.70981	1
O	82.64083	80.52345	78.64810	186.15459	-480.78787	-449.34387	1
N	83.60448	80.51901	80.72177	-487.53139	543.18517	42.03850	1
C	84.94189	80.55371	80.21470	-699.35871	69.34393	360.10701	1
H	84.89953	81.08552	79.23573	2.81126	-221.99807	-2715.58072	1
C	85.71644	81.30616	81.38177	-507.46439	-592.59194	122.07761	1
H	86.81437	81.12352	81.35854	-1975.08116	525.87560	-1801.71556	1
H	85.57154	82.40823	81.33059	1588.27743	-1913.22100	1113.83566	1
C	85.01761	80.69841	82.63657	-613.62442	-457.55993	-194.14490	1
H	85.40730	79.66652	82.83408	1707.54637	-721.27992	1996.92523	1
H	85.17556	81.28559	83.56956	758.23357	2100.81767	1560.55011	1
C	83.50030	80.66078	82.16796	-352.41524	704.96076	49.21465	1
H	82.92028	79.79206	82.56003	1714.76886	-2070.42778	443.35792	1
H	82.92602	81.59958	82.34708	1224.15040	1801.50881	1636.97633	1
C	85.54549	79.03505	80.06185	566.85117	537.05831	117.62814	1
O	86.61251	78.88714	79.51312	-550.26779	-406.08867	4.21666	1
N	84.75252	78.10707	80.58995	566.34832	-425.77725	180.18749	1
H	83.88747	78.43254	81.01887	644.44502	2646.80550	-52.73430	1
C	85.29596	76.80804	80.81076	612.34801	-281.48662	411.56221	1
H	86.24556	76.83858	80.22260	1667.23498	-1490.75142	1556.16753	1
C	85.55672	76.60282	82.37525	-714.45746	-175.43074	286.98927	1
H	84.58162	76.35744	82.87338	-787.09484	1763.03619	-1922.46060	1
O	86.10098	77.73074	83.10067	-429.46514	516.51932	-128.40526	1
H	85.42457	77.83644	83.79736	1981.82785	436.16847	-1818.18182	1
C	86.46706	75.32357	82.37170	630.79189	-468.94448	76.03230	1
H	86.68928	74.97854	83.40856	-1775.71861	2062.30353	131.89849	1
H	85.97866	74.46833	81.82995	-1855.14608	88.94118	1993.53760	1
H	87.44602	75.52153	81.87488	-1504.82350	-780.94249	-2132.90992	1
C	84.21825	75.69757	80.29642	288.57638	618.62814	-397.00270	1
O	84.25121	74.56212	80.72802	321.70495	-185.26452	574.37218	1
N	83.38784	76.17849	79.37827	-447.05792	-446.53431	367.76425	1
H	83.51705	77.14564	79.08494	350.31316	1975.63644	-1843.31522	1
C	82.62169	75.20577	78.63780	-511.23061	374.03917	-471.51480	1
H	83.40666	74.42307	78.52019	2234.79579	-898.70890	-1273.45190	1
C	81.48862	74.68069	79.62204	529.53781	-159.34528	563.72604	1
H	81.23638	73.63143	79.30910	654.59388	2347.94251	1217.51488	1
H	81.97004	74.61097	80.63502	-1526.55661	2103.42026	817.87394	1
O	80.24406	75.40826	79.68971	-554.84278	14.12789	399.58769	1
H	80.57249	76.25943	80.16355	1297.06236	2061.84854	1220.68812	1
C	82.19266	75.88713	77.23729	-662.15484	-412.13544	123.62687	1
O	83.07810	76.34210	76.51443	-540.01537	-317.05804	274.91456	1
N	80.90397	75.74723	76.99471	-202.76299	-690.00100	-131.49145	1
H	80.34172	75.29631	77.72777	-702.62132	-2617.37191	281.70413	1
C	80.20375	76.58771	76.08590	-491.73914	576.34187	222.73509	1
H	79.14462	76.48765	76.41054	1977.17978	-1823.58850	-434.68928	1
C	80.75549	78.07172	76.21815	-649.50797	-328.66843	306.11224	1
H	81.84780	78.10458	75.99920	2075.07803	1452.16490	-1004.45904	1
C	79.96124	78.99614	75.23326	-615.18735	-489.99689	-70.96352	1

H	80.31590	80.05253	75.28718	-1226.32661	-1724.38906	-1716.47170	1
H	80.07625	78.65971	74.17572	2381.64273	-969.84735	900.46860	1
H	78.87006	78.99958	75.46326	2122.74991	1702.23946	-141.36228	1
C	80.48022	78.60014	77.66742	-501.86074	-479.61276	-376.42482	1
H	80.83930	79.65328	77.78477	993.79261	-2246.95401	-1177.81305	1
H	79.39251	78.58582	77.91390	42.71279	-626.15391	-2651.37258	1
H	81.00316	77.98217	78.43845	-2026.39012	-1590.33709	-887.82922	1
C	80.40021	76.07089	74.54046	-672.89153	382.57394	-156.34072	1
O	79.42836	75.96677	73.83595	410.04627	221.86223	500.35950	1
N	81.68139	75.92587	74.22737	-378.11782	-443.79414	-441.10984	1
H	82.42389	76.13114	74.94273	2404.15973	-568.14894	1149.30085	1
C	81.98351	75.21802	73.01542	-544.54484	466.16019	331.29119	1
H	81.32248	75.71133	72.26780	-1409.80988	-170.17955	2325.32679	1
C	83.53504	75.40493	72.76317	-184.48284	116.78282	-758.89137	1
H	83.82338	76.47997	72.80546	1893.78641	1861.14652	611.04528	1
H	84.12358	74.90237	73.56952	-2170.79665	-974.19311	1327.50117	1
C	83.86781	74.78076	71.37175	389.51668	672.81878	-138.49152	1
H	83.45692	73.74558	71.31147	510.64577	1933.03327	1851.02484	1
H	83.32972	75.32628	70.56555	-2302.30869	-1105.72939	-948.89774	1
C	85.41066	74.75444	71.05633	-559.93427	-126.34590	542.30857	1
O	85.79257	74.29459	70.01107	-670.36003	-111.83166	76.36448	1
N	86.18865	75.27056	72.03244	-216.06729	-537.07037	446.51379	1
H	85.92635	75.68761	72.93313	-1251.96487	1074.12894	-2168.52500	1
H	87.21131	75.33113	71.97625	-2451.83023	-720.73851	944.84612	1
C	81.65276	73.62310	73.08558	546.24052	-563.88337	-85.11809	1
O	81.38154	73.11037	72.02868	71.70702	-307.40463	606.69673	1
N	81.85915	73.01454	74.26677	479.90863	-504.50129	222.86849	1
C	81.48433	71.64982	74.44965	75.51268	-600.91904	506.73700	1
H	81.33783	71.22247	73.42892	-2033.61562	1795.30103	254.89480	1
C	82.71289	71.08666	75.28867	523.79067	517.99274	284.45581	1
H	82.46449	70.14327	75.82524	1599.61665	-1127.00911	1895.98173	1
H	83.58806	70.86859	74.63699	-1881.83093	1275.82488	-1501.55016	1
C	83.00177	72.28240	76.24901	-600.05639	-404.05026	316.64485	1
H	82.30506	72.27609	77.11624	-2597.05260	496.36221	657.73249	1
H	84.02910	72.26656	76.67680	-1666.24518	1294.38985	-1723.90591	1
C	82.73862	73.53366	75.30773	493.57570	-129.95376	-602.56394	1
H	82.18149	74.36786	75.79727	-1469.08415	-637.84797	2204.22581	1
H	83.64238	73.93981	74.78930	-1038.97840	1068.88701	-2280.71789	1
C	80.16339	71.53605	75.41676	188.88100	241.05089	-727.87821	1
O	80.05876	70.58927	76.16684	531.88613	-49.19943	427.07960	1
N	79.32228	72.54586	75.25715	708.14833	26.93417	-179.73509	1
H	79.55672	73.26220	74.57034	-2232.69501	-1179.09831	1023.94816	1
C	78.03464	72.43127	75.88342	-738.32412	259.92548	104.42456	1
H	77.91359	71.32318	75.87393	2028.89123	1260.02983	1311.33324	1
C	78.12643	72.94631	77.38390	-258.65678	-345.52513	661.28459	1
H	78.27213	74.04746	77.41748	-1826.06090	-1801.46097	-918.64554	1
C	76.83747	72.50070	78.15654	-541.23400	531.92970	-218.41428	1
H	76.85099	72.85758	79.21291	1311.73218	-2219.18200	882.19023	1
H	75.90528	72.89229	77.68618	-1048.71298	1223.58118	-2196.97971	1
H	76.74479	71.38969	78.18384	419.06414	1903.92898	1903.44670	1
C	79.31440	72.21223	78.09857	-423.86431	152.66566	-648.55262	1
H	79.44053	72.56220	79.15056	105.68386	-2342.50080	1387.51238	1
H	79.15320	71.10592	78.12805	-1695.63603	-1484.73621	-1531.02793	1
H	80.27702	72.38752	77.56108	-1853.57121	-487.39031	1936.59343	1
C	76.98346	73.20155	74.91675	539.71428	-510.58232	267.58633	1
O	76.93274	72.86709	73.73116	-15.93758	-278.34085	-624.49402	1
N	76.25733	74.08635	75.55810	-676.81886	12.05292	276.17960	1
H	76.47522	74.23259	76.54056	-1694.44250	-373.84189	-2100.66098	1
C	75.18528	74.80301	74.96368	412.33078	-664.70360	108.35451	1
H	74.93267	75.57195	75.72741	1765.77476	1294.44366	1621.76594	1
C	75.74458	75.41387	73.61412	-179.66635	-721.95667	-264.73915	1
H	76.76333	75.83393	73.78148	-607.34922	2587.87032	-598.10018	1

H	75.87912	74.62554	72.83520	-359.69332	-1479.13954	-2259.74260	1
C	74.74930	76.51810	73.12829	-240.01076	616.02425	431.85322	1
H	73.82812	76.06347	72.69746	-1359.17131	782.54425	2227.99181	1
H	74.40941	77.15239	73.97785	-820.36667	85.81767	-2596.78715	1
C	75.51698	77.39518	72.05832	-513.07015	236.18570	-551.87280	1
H	76.45387	77.81529	72.49199	1816.11944	-1183.11722	-1650.94321	1
H	75.83950	76.77163	71.19276	-1800.04429	-1361.53473	1526.34643	1
C	74.57411	78.53175	71.60087	726.08972	-253.19732	-179.64713	1
H	73.70276	78.19643	71.00388	1224.27271	-456.51798	-2390.90304	1
H	74.24011	79.20882	72.41216	1206.40339	-1150.93514	-2154.90344	1
N	75.39135	79.45908	70.63794	329.70796	-633.68968	-155.67358	1
H	74.85767	80.27497	70.21981	1348.31305	1938.76845	1359.00643	1
H	76.23504	79.95441	71.04989	2096.92035	1446.54551	-966.48890	1
H	75.80490	79.01144	69.76899	-1197.07217	-2303.53719	827.28733	1
C	73.91255	73.79218	74.69683	-378.01186	-556.17301	-413.96481	1
O	72.85858	74.04511	75.22187	-409.44433	-307.55053	-453.30833	1
N	74.25550	72.76644	73.93616	-429.02477	524.70884	274.08502	1
H	75.23179	72.70886	73.55044	1706.34263	-1425.96036	1574.38926	1
C	73.24567	71.92485	73.37981	-45.25864	-626.22952	478.93267	1
H	73.86759	71.08550	72.99402	1988.63164	570.90773	1772.87287	1
C	72.22560	71.48234	74.50757	551.77128	-20.49526	-564.54996	1
H	72.75251	71.29988	75.47085	1434.61535	-1753.10253	1513.99471	1
H	71.49865	72.30129	74.72221	-714.67738	1819.09770	1898.36494	1
C	71.50276	70.18591	74.00939	446.99263	-555.77305	338.97221	1
H	71.13395	70.31450	72.96750	1953.68466	1892.25957	-161.67766	1
H	72.21326	69.33061	73.96925	-403.03785	2688.07032	-188.43591	1
C	70.29491	69.86525	74.97852	567.86262	335.74681	434.04421	1
H	70.65756	69.62968	76.00553	-1080.02275	1568.26081	-1948.78900	1
H	69.62581	70.75018	75.08717	-919.54108	2111.34692	1456.13463	1
C	69.52276	68.67036	74.37189	195.81897	173.33862	-745.11536	1
H	68.99102	68.89974	73.42686	1405.67949	-2301.21089	-390.08113	1
H	70.12204	67.74564	74.25363	-940.37328	-1118.32446	2299.72133	1
N	68.38638	68.26923	75.37277	319.26422	656.36007	-42.02424	1
H	67.76492	67.46742	75.06211	1961.03633	619.91968	1787.09422	1
H	68.68690	67.93595	76.33473	854.14343	-2156.80752	1429.08711	1
H	67.66588	69.01135	75.61111	1237.02905	-1378.50857	-1998.28500	1
C	72.58084	72.77146	72.15224	532.53319	-268.57236	517.55729	1
O	73.26681	73.39758	71.38196	266.16714	-290.73804	-558.88002	1
N	71.26491	72.59278	72.11966	-226.25352	640.49678	-270.32839	1
H	70.69429	72.14601	72.84825	2007.69063	-1035.27229	1523.50231	1
C	70.55860	72.98769	70.95279	-540.76542	-573.33303	49.50000	1
H	71.16078	73.76762	70.43186	-2374.20950	-1036.76023	843.75774	1
C	70.31636	71.66035	70.08191	-77.09082	666.87422	-415.84136	1
H	69.63780	70.97716	70.66395	-985.81834	617.19825	2463.91927	1
O	71.47931	70.87301	69.77410	293.29899	-391.65602	-477.80926	1
H	71.26556	70.03276	70.22806	2528.72714	-681.76262	-751.27122	1
C	69.55120	72.22484	68.83227	252.47363	-605.14473	440.05134	1
H	69.28151	71.40677	68.12392	-2052.65063	1456.49051	-1043.51830	1
H	68.58449	72.75213	69.11563	-2152.98800	-1661.97574	-161.70652	1
H	70.17911	72.95210	68.26623	-473.12726	-1584.27339	2165.61725	1
C	69.15982	73.43650	71.68257	360.36307	488.46713	505.10056	1
O	68.82023	72.84298	72.68938	-200.08817	-528.03552	-385.82850	1
N	68.55636	74.42233	71.03400	-597.54095	-226.44775	355.20479	1
H	68.98128	74.78079	70.17793	2179.23655	-1300.70981	-991.33835	1
C	67.21032	74.72464	71.36284	-687.68921	297.39807	-249.45179	1
H	67.04970	74.25169	72.36196	-349.16256	-2467.37713	-1101.72831	1
C	67.01303	76.31752	71.33432	672.47345	361.54328	-201.62880	1
H	66.89909	76.66063	70.28113	1541.41502	582.18425	2169.97026	1
H	66.06611	76.57877	71.86412	-2534.67717	60.25153	997.72295	1
C	68.20868	77.02210	71.98591	574.26774	-389.43119	-377.02542	1
C	68.28013	77.16107	73.40511	688.82679	-287.33580	-257.96133	1
H	67.48033	76.75503	74.05287	331.14182	1470.38163	2269.80013	1

C	69.25547	77.52793	71.15577	-383.52382	-675.04808	144.24917	1
H	69.21105	77.40527	70.05872	4.09847	1517.59662	2262.86397	1
C	69.38066	77.82063	73.95335	-134.76730	428.45285	-649.50303	1
H	69.43440	77.93394	75.05037	-903.23433	1600.49020	2011.53387	1
C	70.33582	78.18027	71.75261	-335.53281	-615.00040	-364.39058	1
H	71.13592	78.57168	71.10003	1596.45398	-1369.33862	1732.02659	1
C	70.43982	78.35693	73.16375	585.48547	-340.24194	-406.23938	1
O	71.53455	79.02527	73.77186	-419.15649	-173.04408	-511.94141	1
H	72.03262	79.29478	72.96329	-1071.84350	1559.71507	-1960.13012	1
C	66.30237	74.09672	70.16492	291.41878	-183.13091	710.72246	1
O	66.80670	73.45432	69.26232	379.87695	523.47396	-222.23622	1
N	65.04434	74.41069	70.38938	564.30591	373.09380	277.24371	1
H	64.81266	74.94919	71.24237	-120.59931	-2436.07824	-1214.35098	1
C	64.00155	74.14627	69.44810	404.07514	-299.37480	608.84009	1
H	63.46499	75.11671	69.36638	-1071.07882	-841.69025	2359.66467	1
C	64.57180	73.58946	68.08844	-683.12807	68.71770	-390.13096	1
H	65.36395	74.26402	67.68595	2068.88918	1596.58590	770.89603	1
H	65.07394	72.60674	68.27294	1379.86429	2342.49779	-179.85638	1
C	63.41080	73.41390	67.05772	-176.39967	537.49466	550.97268	1
H	62.57990	72.81100	67.48663	-396.28051	1942.22168	1869.33314	1
H	62.96380	74.39102	66.76953	-94.44700	586.00678	-2659.20016	1
C	64.08146	72.67598	65.80978	-291.69381	-718.18079	-150.72929	1
H	64.85763	73.26431	65.25704	-2303.38776	1089.76242	964.62032	1
H	64.51512	71.66715	66.03140	884.16729	2568.80533	-207.74283	1
N	62.99342	72.48004	64.94888	-594.49522	-252.81478	342.29276	1
H	62.08823	72.76105	65.29245	-1735.17426	2084.05705	-263.71605	1
C	63.07623	71.96931	63.78745	595.23177	-271.52487	442.22352	1
N	64.34257	71.58266	63.33543	585.53813	-424.79750	-105.81303	1
H	65.24384	71.64365	63.80761	959.88797	1327.56823	-2177.11905	1
H	64.53627	71.16196	62.42616	1578.48647	16.27378	-2220.76233	1
N	61.81420	71.89964	63.18151	-452.17763	-390.33541	-421.51853	1
H	60.89473	72.18200	63.51775	470.26107	1974.25629	-1817.92087	1
H	61.66416	71.51998	62.24602	1856.67307	-1573.97321	-1224.35416	1
C	63.06089	72.97848	70.12055	-531.47271	-581.45780	-55.06206	1
O	63.39825	71.81677	70.18200	-243.00837	192.73507	609.52485	1
N	61.92782	73.54736	70.52973	343.49288	-644.25607	38.09164	1
H	61.88095	74.55616	70.38115	-1691.16043	-1535.97525	-1484.73099	1
C	60.97283	72.90342	71.42562	542.66492	350.14337	454.42563	1
H	60.06177	73.54413	71.34962	1209.57979	-2396.95920	463.86744	1
C	60.72671	71.47999	70.95491	-737.14309	145.93010	242.71942	1
H	61.72110	70.95803	70.90942	-1050.18303	159.36419	-2509.06125	1
C	59.82789	70.71360	71.90591	-706.77696	-252.88343	-245.16230	1
H	59.68662	69.64663	71.57768	-1624.64039	-1665.49085	1417.86935	1
H	60.25156	70.69264	72.95006	2566.10521	598.51109	693.22305	1
H	58.80633	71.17984	71.97450	-1972.69758	107.10018	-1876.34312	1
C	60.14082	71.45274	69.55453	412.85148	672.58832	-27.69222	1
H	60.01792	70.40219	69.17283	1447.02277	1762.76533	-1490.78944	1
H	59.12927	71.94265	69.51795	1320.77379	-145.20455	2378.68491	1
H	60.80208	71.99853	68.82669	-1512.10848	1470.52031	1724.75147	1
C	61.57407	72.94525	72.97399	-607.11113	499.56689	73.73769	1
O	60.82569	72.69547	73.89069	357.97126	460.81534	356.68785	1
N	62.85956	73.23637	73.02863	536.55407	-368.62270	332.76306	1
H	63.36754	73.40373	72.16160	524.38866	2297.69985	1367.20941	1
C	63.47712	73.40111	74.29739	46.50277	-774.29190	-147.97904	1
H	62.62940	73.55291	75.00483	1676.51875	1883.29066	1032.55549	1
C	64.33417	72.07022	74.63937	-559.41456	-34.19198	-556.30433	1
H	65.11720	71.92996	73.85683	-64.66664	395.18769	2695.05373	1
H	64.88257	72.20142	75.60194	-2703.44278	-60.40056	333.79418	1
C	63.43617	70.86687	74.69726	-519.76435	377.31045	459.42366	1
H	62.88184	70.79972	73.73296	-1746.74323	1667.50644	-1261.73691	1
C	64.26099	69.59703	74.87386	570.95554	16.46261	-545.27700	1
H	63.61276	68.68355	74.89723	2391.58273	28.05277	-1305.07271	1

```
H    64.99582  69.46200  74.03899       175.75588       917.63458      2559.43867           1
H    64.84436  69.61374  75.83004     -1063.97851     -2155.34650      1283.00462           1
C    62.42158  70.97227  75.82755       193.76014         2.62946      -765.53141           1
H    61.84911  70.01810  75.95947       507.39394      -846.55087      2539.60089           1
H    62.91230  71.20348  76.80804      1337.57841      1818.19054      1526.01994           1
H    61.66571  71.78256  75.62407      1100.88635     -1172.91350     -2199.08909           1
C    64.50838  74.62364  74.17064      -549.21115       475.64486      -309.38218           1
O    64.88288  75.06694  73.10387       556.06106      -390.37755       -78.24250           1
O    65.04237  75.11804  75.37502      -572.27940      -310.19032      -209.75862           1
H    64.53322  74.53693  76.01521     -1380.08193      2212.14486       790.85944           1
```

Listing 81: C00081.xmolout

```
       276
   150.0000  150.0000  150.0000     0.0000     0.0000     0.0000***********    0.2750     1100
       0.2500    300.00   0.00   0.00   0.00  -1293.5211      0.0000    300.0000
H    74.17324  77.32821  70.79415     -2043.14101     -1757.64893       399.89339           1
N    74.49287  77.73899  69.90037       426.75047       490.45733      -334.42467           1
H    73.63175  78.04654  69.43224       442.25559     -1110.30854     -2448.52907           1
C    75.47937  78.75883  69.96965       415.32972      -666.39213        83.73172           1
H    75.60893  79.00154  71.05019       879.32219      2491.00447       667.35213           1
C    76.84202  78.12605  69.34476        31.35357       477.41777       628.23362           1
H    76.66678  77.83199  68.28329      1168.38489      2240.25337     -1019.71136           1
H    77.66430  78.87748  69.32788      -360.43770      2534.90498       931.67112           1
C    77.20127  76.92789  70.16848       463.18208       512.70242      -382.34388           1
H    76.27862  76.32468  70.32906      1725.29117      1863.52247      -987.07854           1
C    78.21257  76.04510  69.44504       710.42801      -144.27427      -313.15367           1
H    78.50395  75.16027  70.06842       364.20195     -1033.13725      2494.72524           1
H    77.80216  75.65105  68.47988      2567.61904       335.80679       847.48802           1
H    79.15247  76.60424  69.20470     -2587.99029       680.92867      -512.16411           1
C    77.75814  77.31986  71.53218       268.17616       741.64379       -40.42634           1
H    78.17611  76.43340  72.07554      1283.54314     -1945.37420     -1411.27812           1
H    78.58667  78.06703  71.44174     -1258.57796      1317.15249     -2026.02085           1
H    76.97261  77.77134  72.19086     -2502.84526       909.80597       575.92391           1
C    74.86742  79.93108  69.11174       619.36578      -458.64843      -172.09273           1
O    73.64699  79.91459  68.64311       -81.40433       433.87611       522.34594           1
N    75.68246  80.90619  68.86402       175.23996      -622.20190       341.55523           1
H    76.61718  80.91017  69.26534      -876.57256      -303.22879     -2561.90213           1
C    75.04456  81.98278  68.16751      -571.88214      -534.10783      -106.15033           1
H    74.51772  82.50064  68.99331     -2713.20841       -16.25800      -248.80809           1
C    76.12225  82.94138  67.54742       166.60354       288.16399      -716.09568           1
H    76.90816  82.35411  66.99656     -2097.85574      1636.78449      -586.18044           1
O    76.88913  83.71109  68.51892      -317.80580      -363.38227       484.43082           1
H    77.79863  83.49284  68.25948      1863.74748     -1451.84923      1357.29467           1
C    75.27029  83.78576  66.52861       503.80731       517.99032       318.51606           1
H    75.90442  84.51734  65.97501       640.61020     -2157.10410     -1536.29104           1
H    74.76736  83.13989  65.77023      1687.88455       465.60692      2087.56452           1
H    74.47356  84.36591  67.05019     -1293.07895     -1899.88581     -1463.57452           1
C    73.82357  81.26402  67.20996      -153.07550      -733.68902       248.70981           1
O    72.80099  81.94669  67.08633       186.15459      -480.78787      -449.34387           1
N    74.35213  80.51860  66.17862      -487.53139       543.18517        42.03850           1
C    73.57971  80.04334  65.09536      -699.35871        69.34393       360.10701           1
H    72.92234  80.89404  64.80268         2.81126      -221.99807     -2715.58072           1
C    74.67551  79.59085  64.02998      -507.46439      -592.59194       122.07761           1
H    74.28648  78.82628  63.32006     -1975.08116       525.87560     -1801.71556           1
H    75.04037  80.44363  63.41623      1588.27743     -1913.22100      1113.83566           1
C    75.78991  79.04216  64.96715      -613.62442      -457.55993      -194.14490           1
H    75.52698  78.03571  65.36157      1707.54637      -721.27992      1996.92523           1
H    76.78529  78.94713  64.47986       758.23357      2100.81767      1560.55011           1
C    75.74969  80.15221  66.11848      -352.41524       704.96076        49.21465           1
H    76.07191  79.80491  67.12758      1714.76886     -2070.42778       443.35792           1
H    76.30173  81.08923  65.86349      1224.15040      1801.50881      1636.97633           1
```

C	72.69819	78.66054	65.32483	566.85117	537.05831	117.62814	1
O	72.08722	78.22384	64.38110	-550.26779	-406.08867	4.21666	1
N	72.84164	78.15753	66.53694	566.34832	-425.77725	180.18749	1
H	73.37707	78.70558	67.22300	644.44502	2646.80550	-52.73430	1
C	72.12839	76.97629	66.89561	612.34801	-281.48662	411.56221	1
H	71.60777	76.69982	65.94894	1667.23498	-1490.75142	1556.16753	1
C	73.29808	75.93069	67.20212	-714.45746	-175.43074	286.98927	1
H	74.04857	76.43903	67.86618	-787.09484	1763.03619	-1922.46060	1
O	74.04625	75.57110	66.01382	-429.46514	516.51932	-128.40526	1
H	74.90821	75.97957	66.20977	1981.82785	436.16847	-1818.18182	1
C	72.75325	74.66266	67.95244	630.79189	-468.94448	76.03230	1
H	73.45823	73.80872	67.82133	-1775.71861	2062.30353	131.89849	1
H	72.66979	74.82170	69.05697	-1855.14608	88.94118	1993.53760	1
H	71.76195	74.32560	67.56169	-1504.82350	-780.94249	-2132.90992	1
C	71.04719	77.46226	68.00551	288.57638	618.62814	-397.00270	1
O	70.52423	78.54912	67.91162	321.70495	-185.26452	574.37218	1
N	70.69046	76.49251	68.85558	-447.05792	-446.53431	367.76425	1
H	71.18598	75.60073	68.87280	350.31316	1975.63644	-1843.31522	1
C	70.14941	76.94643	70.11503	-511.23061	374.03917	-471.51480	1
H	69.28286	77.60888	69.88324	2234.79579	-898.70890	-1273.45190	1
C	69.82086	75.58191	70.86233	529.53781	-159.34528	563.72604	1
H	69.41201	75.85366	71.86838	654.59388	2347.94251	1217.51488	1
H	68.99327	75.02980	70.34302	-1526.55661	2103.42026	817.87394	1
O	70.94569	74.66333	70.98012	-554.84278	14.12789	399.58769	1
H	71.66108	75.30816	71.18995	1297.06236	2061.84854	1220.68812	1
C	71.33968	77.68937	70.92138	-662.15484	-412.13544	123.62687	1
O	72.42410	77.09816	71.11505	-540.01537	-317.05804	274.91456	1
N	70.95262	78.83305	71.42004	-202.76299	-690.00100	-131.49145	1
H	69.99609	79.08188	71.16699	-702.62132	-2617.37191	281.70413	1
C	71.91754	79.85374	71.72306	-491.73914	576.34187	222.73509	1
H	72.71421	79.67587	70.96556	1977.17978	-1823.58850	-434.68928	1
C	71.15966	81.23299	71.55244	-649.50797	-328.66843	306.11224	1
H	70.15524	81.17875	72.02604	2075.07803	1452.16490	-1004.45904	1
C	71.98264	82.42779	72.14885	-615.18735	-489.99689	-70.96352	1
H	71.53522	83.41325	71.87862	-1226.32661	-1724.38906	-1716.47170	1
H	72.02517	82.38707	73.27235	2381.64273	-969.84735	900.46860	1
H	73.03386	82.42539	71.77590	2122.74991	1702.23946	-141.36228	1
C	71.03706	81.51904	70.01458	-501.86074	-479.61276	-376.42482	1
H	70.46460	82.45563	69.81524	993.79261	-2246.95401	-1177.81305	1
H	72.03828	81.63371	69.53330	42.71279	-626.15391	-2651.37258	1
H	70.51846	80.68936	69.47750	-2026.39012	-1590.33709	-887.82922	1
C	72.51437	79.66531	73.22627	-672.89153	382.57394	-156.34072	1
O	72.40745	80.54636	74.05183	410.04627	221.86223	500.35950	1
N	73.16870	78.52109	73.32163	-378.11782	-443.79414	-441.10984	1
H	73.12996	77.81364	72.55506	2404.15973	-568.14894	1149.30085	1
C	73.79564	78.18502	74.57749	-544.54484	466.16019	331.29119	1
H	74.52756	78.99154	74.80297	-1409.80988	-170.17955	2325.32679	1
C	72.59949	78.17614	75.62305	-184.48284	116.78282	-758.89137	1
H	72.12370	79.18915	75.65090	1893.78641	1861.14652	611.04528	1
H	71.80386	77.47601	75.28148	-2170.79665	-974.19311	1327.50117	1
C	73.09017	77.79979	77.05425	389.51668	672.81878	-138.49152	1
H	73.45673	76.75027	77.06523	510.64577	1933.03327	1851.02484	1
H	73.97069	78.41027	77.34640	-2302.30869	-1105.72939	-948.89774	1
C	71.95170	78.00757	78.12311	-559.93427	-126.34590	542.30857	1
O	72.23217	78.02789	79.29778	-670.36003	-111.83166	76.36448	1
N	70.71585	78.10660	77.59644	-216.06729	-537.07037	446.51379	1
H	70.42264	78.13168	76.61213	-1251.96487	1074.12894	-2168.52500	1
H	69.86920	78.28172	78.14844	-2451.83023	-720.73851	944.84612	1
C	74.43573	76.72634	74.36020	546.24052	-563.88337	-85.11809	1
O	73.75764	75.88657	73.77672	71.70702	-307.40463	606.69673	1
N	75.64503	76.58626	74.89872	479.90863	-504.50129	222.86849	1
C	76.37732	75.35763	74.75144	75.51268	-600.91904	506.73700	1

H	76.20706	75.05322	73.69475	-2033.61562	1795.30103	254.89480	1
C	77.85840	75.78385	75.10834	523.79067	517.99274	284.45581	1
H	78.46782	74.93288	75.48938	1599.61665	-1127.00911	1895.98173	1
H	78.39726	76.18211	74.21671	-1881.83093	1275.82488	-1501.55016	1
C	77.62782	76.89933	76.17752	-600.05639	-404.05026	316.64485	1
H	77.37981	76.45302	77.16703	-2597.05260	496.36221	657.73249	1
H	78.51146	77.55779	76.33314	-1666.24518	1294.38985	-1723.90591	1
C	76.37829	77.65948	75.55731	493.57570	-129.95376	-602.56394	1
H	75.70403	78.14507	76.29993	-1469.08415	-637.84797	2204.22581	1
H	76.63674	78.39236	74.75598	-1038.97840	1068.88701	-2280.71789	1
C	75.77834	74.24867	75.80141	188.88100	241.05089	-727.87821	1
O	76.45599	73.88069	76.73437	531.88613	-49.19943	427.07960	1
N	74.55518	73.94517	75.43653	708.14833	26.93417	-179.73509	1
H	74.14553	74.43814	74.60230	-2232.69501	-1179.09831	1023.94816	1
C	73.73578	72.94707	76.03268	-738.32412	259.92548	104.42456	1
H	72.73578	73.30657	75.70006	2028.89123	1260.02983	1311.33324	1
C	74.04376	71.53254	75.36390	-258.65678	-345.52513	661.28459	1
H	75.13013	71.30506	75.38376	-1826.06090	-1801.46097	-918.64554	1
C	73.19439	70.46089	76.13225	-541.23400	531.92970	-218.41428	1
H	72.92601	69.58711	75.49385	1311.73218	-2219.18200	882.19023	1
H	73.74946	70.06715	77.02221	-1048.71298	1223.58118	-2196.97971	1
H	72.23896	70.89844	76.50451	419.06414	1903.92898	1903.44670	1
C	73.51248	71.56059	73.89265	-423.86431	152.66566	-648.55262	1
H	73.70487	70.59783	73.36531	105.68386	-2342.50080	1387.51238	1
H	72.41341	71.74469	73.85871	-1695.63603	-1484.73621	-1531.02793	1
H	74.00046	72.36811	73.29994	-1853.57121	-487.39031	1936.59343	1
C	73.87311	72.88092	77.64932	539.71428	-510.58232	267.58633	1
O	74.09029	71.79625	78.21287	-15.93758	-278.34085	-624.49402	1
N	73.51557	74.02924	78.19669	-676.81886	12.05292	276.17960	1
H	73.48571	74.74498	77.46752	-1694.44250	-373.84189	-2100.66098	1
C	73.97842	74.30417	79.53502	412.33078	-664.70360	108.35451	1
H	75.08036	74.15956	79.48915	1765.77476	1294.44366	1621.76594	1
C	73.53670	75.78145	79.85538	-179.66635	-721.95667	-264.73915	1
H	73.90388	76.48953	79.07899	-607.34922	2587.87032	-598.10018	1
H	72.42369	75.84505	79.81917	-359.69332	-1479.13954	-2259.74260	1
C	74.06551	76.20081	81.26984	-240.01076	616.02425	431.85322	1
H	73.78071	75.44605	82.03942	-1359.17131	782.54425	2227.99181	1
H	75.17663	76.25292	81.28569	-820.36667	85.81767	-2596.78715	1
C	73.43106	77.61180	81.59324	-513.07015	236.18570	-551.87280	1
H	73.79329	78.38373	80.87306	1816.11944	-1183.11722	-1650.94321	1
H	72.32105	77.57981	81.47009	-1800.04429	-1361.53473	1526.34643	1
C	73.80826	77.99695	83.04506	726.08972	-253.19732	-179.64713	1
H	73.20784	77.49495	83.82936	1224.27271	-456.51798	-2390.90304	1
H	74.88595	77.91342	83.28246	1206.40339	-1150.93514	-2154.90344	1
N	73.51864	79.52367	83.20921	329.70796	-633.68968	-155.67358	1
H	73.64961	79.91026	84.18775	1348.31305	1938.76845	1359.00643	1
H	74.13206	80.17839	82.64287	2096.92035	1446.54551	-966.48890	1
H	72.54502	79.87104	82.96815	-1197.07217	-2303.53719	827.28733	1
C	73.25004	73.30939	80.59742	-378.01186	-556.17301	-413.96481	1
O	73.68814	73.21686	81.72435	-409.44433	-307.55053	-453.30833	1
N	72.12868	72.78096	80.11009	-429.02477	524.70884	274.08502	1
H	71.91346	72.96074	79.13095	1706.34263	-1425.96036	1574.38926	1
C	71.29880	72.03377	80.99752	-45.25864	-626.22952	478.93267	1
H	71.54466	72.46261	81.99806	1988.63164	570.90773	1772.87287	1
C	69.80152	72.26062	80.52459	551.77128	-20.49526	-564.54996	1
H	69.57903	73.34770	80.43421	1434.61535	-1753.10253	1513.99471	1
H	69.64745	71.84161	79.50474	-714.67738	1819.09770	1898.36494	1
C	68.82451	71.58433	81.53956	446.99263	-555.77305	338.97221	1
H	69.03781	70.49068	81.60401	1953.68466	1892.25957	-161.67766	1
H	68.97497	71.98673	82.56624	-403.03785	2688.07032	-188.43591	1
C	67.34368	71.84682	81.04393	567.86262	335.74681	434.04421	1
H	67.10231	72.93385	81.07982	-1080.02275	1568.26081	-1948.78900	1

```
H    67.22177   71.54700   79.97825      -919.54108      2111.34692      1456.13463          1
C    66.38027   71.03477   81.93882       195.81897       173.33862      -745.11536          1
H    66.50931   69.93618   81.87238      1405.67949     -2301.21089      -390.08113          1
H    66.35864   71.35066   83.00003      -940.37328     -1118.32446      2299.72133          1
N    64.92022   71.28554   81.42762       319.26422       656.36007       -42.02424          1
H    64.15956   70.75850   81.94571      1961.03633       619.91968      1787.09422          1
H    64.54935   72.27896   81.47168       854.14343     -2156.80752      1429.08711          1
H    64.70964   71.01660   80.42308      1237.02905     -1378.50857     -1998.28500          1
C    71.69657   70.45209   80.93790       532.53319      -268.57236       517.55729          1
O    71.18038   69.70813   81.74077       266.16714      -290.73804      -558.88002          1
N    72.57709   70.15491   79.98566      -226.25352       640.49678      -270.32839          1
H    72.92686   70.90372   79.36456      2007.69063     -1035.27229      1523.50231          1
C    72.80019   68.76370   79.76362      -540.76542      -573.33303        49.50000          1
H    72.36382   68.26585   80.66527     -2374.20950     -1036.76023       843.75774          1
C    72.00458   68.37078   78.42458       -77.09082       666.87422      -415.84136          1
H    72.22672   69.13975   77.63833      -985.81834       617.19825      2463.91927          1
O    70.57127   68.37423   78.54640       293.29899      -391.65602      -477.80926          1
H    70.33494   69.12002   77.95836      2528.72714      -681.76262      -751.27122          1
C    72.64656   67.00357   77.99120       252.47363      -605.14473       440.05134          1
H    72.07587   66.53256   77.15759     -2052.65063      1456.49051     -1043.51830          1
H    73.69592   67.12833   77.62839     -2152.98800     -1661.97574      -161.70652          1
H    72.66568   66.27602   78.83726      -473.12726     -1584.27339      2165.61725          1
C    74.38708   68.45105   79.55891       360.36307       488.46713       505.10056          1
O    74.75824   67.30409   79.63906      -200.08817      -528.03552      -385.82850          1
N    75.08966   69.53783   79.27484      -597.54095      -226.44775       355.20479          1
H    74.65486   70.48588   79.21044      2179.23655     -1300.70981      -991.33835          1
C    76.46594   69.38622   78.95767      -687.68921       297.39807      -249.45179          1
H    76.71117   68.38442   79.37521      -349.16256     -2467.37713     -1101.72831          1
C    76.62910   69.45163   77.36698       672.47345       361.54328      -201.62880          1
H    76.08121   70.33408   76.96656      1541.41502       582.18425      2169.97026          1
H    77.70497   69.59671   77.11739     -2534.67717        60.25153       997.72295          1
C    76.16225   68.17291   76.64985       574.26774      -389.43119      -377.02542          1
C    76.71391   66.90102   76.99109       688.82679      -287.33580      -257.96133          1
H    77.40764   66.79750   77.84580       331.14182      1470.38163      2269.80013          1
C    75.26095   68.27808   75.54806      -383.52382      -675.04808       144.24917          1
H    74.84275   69.25616   75.25696         4.09847      1517.59662      2262.86397          1
C    76.37468   65.79189   76.21460      -134.76730       428.45285      -649.50303          1
H    76.80719   64.81064   76.47577      -903.23433      1600.49020      2011.53387          1
C    74.94261   67.13931   74.80343      -335.53281      -615.00040      -364.39058          1
H    74.24387   67.24579   73.95584      1596.45398     -1369.33862      1732.02659          1
C    75.49729   65.85766   75.09216       585.48547      -340.24194      -406.23938          1
O    75.20289   64.69672   74.33169      -419.15649      -173.04408      -511.94141          1
H    74.63324   65.09418   73.63180     -1071.84350      1559.71507     -1960.13012          1
C    77.22828   70.62736   79.69498       291.41878      -183.13091       710.72246          1
O    77.25395   70.65646   80.90186       379.87695       523.47396      -222.23622          1
N    77.86451   71.42804   78.82764       564.30591       373.09380       277.24371          1
H    77.67151   71.21772   77.84341      -120.59931     -2436.07824     -1214.35098          1
C    77.97375   72.83709   79.11144       404.07514      -299.37480       608.84009          1
H    77.44045   73.29473   78.21539     -1071.07882      -841.69025      2359.66467          1
C    77.39897   73.23905   80.52066      -683.12807        68.71770      -390.13096          1
H    76.37024   72.82777   80.67316      2068.88918      1596.58590       770.89603          1
H    78.03448   72.75962   81.30622      1379.86429      2342.49779      -179.85638          1
C    77.42806   74.79544   80.68450      -176.39967       537.49466       550.97268          1
H    78.41993   75.21320   80.39938      -396.28051      1942.22168      1869.33314          1
H    76.68688   75.30260   80.02661       -94.44700       586.00678     -2659.20016          1
C    77.12798   75.03788   82.23267      -291.69381      -718.18079      -150.72929          1
H    76.10230   74.75548   82.58337     -2303.38776      1089.76242       964.62032          1
H    77.86395   74.55415   82.92306       884.16729      2568.80533      -207.74283          1
N    77.29873   76.42017   82.38172      -594.49522      -252.81478       342.29276          1
H    77.75010   76.91700   81.62983     -1735.17426      2084.05705      -263.71605          1
C    76.95474   77.08618   83.40735       595.23177      -271.52487       442.22352          1
N    76.33650   76.38396   84.44921       585.53813      -424.79750      -105.81303          1
```

```
H    76.13039   75.38862   84.52947      959.88797     1327.56823    -2177.11905          1
H    76.02371   76.79691   85.32787     1578.48647       16.27378    -2220.76233          1
N    77.26501   78.44766   83.26286     -452.17763     -390.33541     -421.51853          1
H    77.74288   78.94750   82.51393      470.26107     1974.25629    -1817.92087          1
H    77.05743   79.15070   83.97385     1856.67307    -1573.97321    -1224.35416          1
C    79.55329   73.20777   79.20409     -531.47271     -581.45780      -55.06206          1
O    80.00920   73.57864   80.26650     -243.00837      192.73507      609.52485          1
N    80.19986   73.09666   78.03146      343.49288     -644.25607       38.09164          1
H    79.66697   72.64613   77.28613    -1691.16043    -1535.97525    -1484.73099          1
C    81.63075   73.00371   78.15565      542.66492      350.14337      454.42563          1
H    81.86585   73.71221   78.98334     1209.57979    -2396.95920      463.86744          1
C    82.00390   71.51139   78.56965     -737.14309      145.93010      242.71942          1
H    81.51311   70.77046   77.90369    -1050.18303      159.36419    -2509.06125          1
C    83.56337   71.37906   78.48335     -706.77696     -252.88343     -245.16230          1
H    83.93943   70.49495   79.05066    -1624.64039    -1665.49085     1417.86935          1
H    83.90493   71.25788   77.42647     2566.10521      598.51109      693.22305          1
H    84.07562   72.28506   78.88850    -1972.69758      107.10018    -1876.34312          1
C    81.51839   71.31556   80.04591      412.85148      672.58832      -27.69222          1
H    81.86659   70.34748   80.48164     1447.02277     1762.76533    -1490.78944          1
H    81.88218   72.13966   80.70439     1320.77379     -145.20455     2378.68491          1
H    80.40236   71.33048   80.10834    -1512.10848     1470.52031     1724.75147          1
C    82.42020   73.35121   76.78326     -607.11113      499.56689       73.73769          1
O    83.63300   73.44284   76.84059      357.97126      460.81534      356.68785          1
N    81.63829   73.39825   75.70844      536.55407     -368.62270      332.76306          1
H    80.63746   73.26893   75.86576      524.38866     2297.69985     1367.20941          1
C    82.01312   74.31522   74.66802       46.50277     -774.29190     -147.97904          1
H    82.49731   75.17112   75.18533     1676.51875     1883.29066     1032.55549          1
C    83.02404   73.59076   73.64492     -559.41456      -34.19198     -556.30433          1
H    82.76890   72.50424   73.60209      -64.66664      395.18769     2695.05373          1
H    82.87460   73.98860   72.61183    -2703.44278      -60.40056      333.79418          1
C    84.46095   73.78933   74.05360     -519.76435      377.31045      459.42366          1
H    84.56498   73.52575   75.14943    -1746.74323     1667.50644    -1261.73691          1
C    85.35503   72.87491   73.22643      570.95554       16.46261     -545.27700          1
H    86.43232   72.96302   73.52267     2391.58273       28.05277    -1305.07271          1
H    85.06514   71.79828   73.34078      175.75588      917.63458     2559.43867          1
H    85.29122   73.11944   72.13425    -1063.97851    -2155.34650     1283.00462          1
C    84.89749   75.23442   73.86626      193.76014        2.62946     -765.53141          1
H    85.99237   75.36664   74.06848      507.39394     -846.55087     2539.60089          1
H    84.70862   75.58944   72.82025     1337.57841     1818.19054     1526.01994          1
H    84.35146   75.92864   74.55608     1100.88635    -1172.91350    -2199.08909          1
C    80.66561   74.68519   73.88763     -549.21115      475.64486     -309.38218          1
O    79.71166   73.95140   73.83519      556.06106     -390.37755      -78.24250          1
O    80.67303   75.90369   73.16361     -572.27940     -310.19032     -209.75862          1
H    81.59889   76.20287   73.37560    -1380.08193     2212.14486      790.85944          1
```

Listing 82: C00082.xmolout

```
       276
  150.0000  150.0000  150.0000     0.0000     0.0000     0.0000***********    2.7750    11100
       0.2500    300.00    0.00    0.00    0.00  -1293.5150       0.0000    300.0000
H    68.47668   71.60640   66.00199    -2043.14101    -1757.64893      399.89339          1
N    68.39853   72.62203   66.01249      426.75047      490.45733     -334.42467          1
H    68.04860   72.81031   66.95495      442.25559    -1110.30854    -2448.52907          1
C    69.61628   73.30066   65.78637      415.32972     -666.39213       83.73172          1
H    70.19153   72.72999   65.02594      879.32219     2491.00447      667.35213          1
C    69.24416   74.80102   65.33003       31.35357      477.41777      628.23362          1
H    68.83606   75.35502   66.20934     1168.38489     2240.25337    -1019.71136          1
H    70.15844   75.35871   65.01428     -360.43770     2534.90498      931.67112          1
C    68.21772   74.75343   64.22681      463.18208      512.70242     -382.34388          1
H    67.42317   74.03379   64.52977     1725.29117     1863.52247     -987.07854          1
C    67.57280   76.11893   64.02856      710.42801     -144.27427     -313.15367          1
H    66.78631   76.09256   63.23051      364.20195    -1033.13725     2494.72524          1
```

H	67.08071	76.48491	64.96662	2567.61904	335.80679	847.48802	1
H	68.32599	76.89124	63.72581	-2587.99029	680.92867	-512.16411	1
C	68.82684	74.27636	62.91594	268.17616	741.64379	-40.42634	1
H	68.06173	74.23103	62.09802	1283.54314	-1945.37420	-1411.27812	1
H	69.64452	74.95894	62.56784	-1258.57796	1317.15249	-2026.02085	1
H	69.26743	73.25086	63.01518	-2502.84526	909.80597	575.92391	1
C	70.28916	73.32770	67.26943	619.36578	-458.64843	-172.09273	1
O	69.58245	73.53122	68.24685	-81.40433	433.87611	522.34594	1
N	71.60212	73.18811	67.19968	175.23996	-622.20190	341.55523	1
H	72.00188	73.02229	66.27683	-876.57256	-303.22879	-2561.90213	1
C	72.32301	72.80463	68.37912	-571.88214	-534.10783	-106.15033	1
H	71.63020	72.13691	68.93387	-2713.20841	-16.25800	-248.80809	1
C	73.67838	72.11058	67.90638	166.60354	288.16399	-716.09568	1
H	74.22235	72.80894	67.21286	-2097.85574	1636.78449	-586.18044	1
O	73.52537	70.87274	67.18805	-317.80580	-363.38227	484.43082	1
H	73.98342	71.07682	66.34876	1863.74748	-1451.84923	1357.29467	1
C	74.45544	72.04424	69.27356	503.80731	517.99032	318.51606	1
H	75.49209	71.65771	69.13847	640.61020	-2157.10410	-1536.29104	1
H	74.53185	73.05667	69.74425	1687.88455	465.60692	2087.56452	1
H	73.94377	71.37474	70.00312	-1293.07895	-1899.88581	-1463.57452	1
C	72.71650	74.15089	69.21387	-153.07550	-733.68902	248.70981	1
O	73.56328	74.88641	68.77493	186.15459	-480.78787	-449.34387	1
N	72.06903	74.22047	70.38855	-487.53139	543.18517	42.03850	1
C	72.11549	75.42304	71.16184	-699.35871	69.34393	360.10701	1
H	72.37103	76.23482	70.44144	2.81126	-221.99807	-2715.58072	1
C	70.64675	75.44796	71.76731	-507.46439	-592.59194	122.07761	1
H	70.57067	76.14351	72.65659	-1975.08116	525.87560	-1801.71556	1
H	69.89643	75.79090	71.01864	1588.27743	-1913.22100	1113.83566	1
C	70.44142	73.94435	72.12262	-613.62442	-457.55993	-194.14490	1
H	71.01846	73.66464	73.03589	1707.54637	-721.27992	1996.92523	1
H	69.38066	73.66441	72.30964	758.23357	2100.81767	1560.55011	1
C	71.04348	73.27715	70.81234	-352.41524	704.96076	49.21465	1
H	71.50525	72.27113	70.95366	1714.76886	-2070.42778	443.35792	1
H	70.32577	73.26201	69.92664	1224.15040	1801.50881	1636.97633	1
C	73.22931	75.24678	72.35432	566.85117	537.05831	117.62814	1
O	72.94865	74.70932	73.42596	-550.26779	-406.08867	4.21666	1
N	74.38637	75.66280	71.91337	566.34832	-425.77725	180.18749	1
H	74.35544	76.01817	70.95595	644.44502	2646.80550	-52.73430	1
C	75.38603	76.22181	72.78794	612.34801	-281.48662	411.56221	1
H	75.04893	77.25831	73.00663	1667.23498	-1490.75142	1556.16753	1
C	75.44500	75.34983	74.12089	-714.45746	-175.43074	286.98927	1
H	75.15244	74.29674	73.84050	-787.09484	1763.03619	-1922.46060	1
O	74.50007	75.74677	75.11833	-429.46514	516.51932	-128.40526	1
H	73.68702	75.20219	74.80076	1981.82785	436.16847	-1818.18182	1
C	76.95302	75.38070	74.55578	630.79189	-468.94448	76.03230	1
H	77.10644	74.85277	75.52609	-1775.71861	2062.30353	131.89849	1
H	77.60673	74.88092	73.80352	-1855.14608	88.94118	1993.53760	1
H	77.32173	76.42511	74.67942	-1504.82350	-780.94249	-2132.90992	1
C	76.69993	76.24784	71.84112	288.57638	618.62814	-397.00270	1
O	77.33959	77.31089	71.76761	321.70495	-185.26452	574.37218	1
N	76.93461	75.07700	71.29494	-447.05792	-446.53431	367.76425	1
H	76.20815	74.39341	71.52015	350.31316	1975.63644	-1843.31522	1
C	77.82851	74.82847	70.22519	-511.23061	374.03917	-471.51480	1
H	77.57941	73.77862	69.95030	2234.79579	-898.70890	-1273.45190	1
C	77.46333	75.84440	69.04893	529.53781	-159.34528	563.72604	1
H	77.87454	75.46007	68.07453	654.59388	2347.94251	1217.51488	1
H	76.34450	75.80670	68.94594	-1526.55661	2103.42026	817.87394	1
O	77.90152	77.20754	69.17416	-554.84278	14.12789	399.58769	1
H	77.72836	77.34543	70.17519	1297.06236	2061.84854	1220.68812	1
C	79.39258	74.97623	70.66721	-662.15484	-412.13544	123.62687	1
O	80.26522	74.94120	69.83166	-540.01537	-317.05804	274.91456	1
N	79.52591	74.96339	71.99610	-202.76299	-690.00100	-131.49145	1

77	H	78.65244	74.99746	72.52027	-702.62132	-2617.37191	281.70413	1
78	C	80.76388	75.40344	72.55461	-491.73914	576.34187	222.73509	1
79	H	81.22858	76.05727	71.78318	1977.17978	-1823.58850	-434.68928	1
80	C	80.41063	76.12396	73.92292	-649.50797	-328.66843	306.11224	1
81	H	79.67003	75.53011	74.49935	2075.07803	1452.16490	-1004.45904	1
82	C	81.71864	76.33504	74.76000	-615.18735	-489.99689	-70.96352	1
83	H	81.50406	76.81181	75.74526	-1226.32661	-1724.38906	-1716.47170	1
84	H	82.23409	75.36241	74.95836	2381.64273	-969.84735	900.46860	1
85	H	82.44642	76.98742	74.22331	2122.74991	1702.23946	-141.36228	1
86	C	79.82181	77.54104	73.60696	-501.86074	-479.61276	-376.42482	1
87	H	79.50921	78.06549	74.53923	993.79261	-2246.95401	-1177.81305	1
88	H	80.56752	78.19119	73.09200	42.71279	-626.15391	-2651.37258	1
89	H	78.91660	77.46833	72.93214	-2026.39012	-1590.33709	-887.82922	1
90	C	81.71381	74.10754	72.87422	-672.89153	382.57394	-156.34072	1
91	O	82.89829	74.27076	73.06123	410.04627	221.86223	500.35950	1
92	N	81.00130	72.99218	72.98559	-378.11782	-443.79414	-441.10984	1
93	H	80.01574	73.07550	72.74011	2404.15973	-568.14894	1149.30085	1
94	C	81.56726	71.78059	73.46322	-544.54484	466.16019	331.29119	1
95	H	80.71291	71.06652	73.43736	-1409.80988	-170.17955	2325.32679	1
96	C	82.73493	71.42208	72.44553	-184.48284	116.78282	-758.89137	1
97	H	82.39803	71.60460	71.39992	1893.78641	1861.14652	611.04528	1
98	H	83.60199	72.10707	72.60825	-2170.79665	-974.19311	1327.50117	1
99	C	83.13291	69.92840	72.64840	389.51668	672.81878	-138.49152	1
100	H	83.58932	69.78877	73.65796	510.64577	1933.03327	1851.02484	1
101	H	82.22181	69.29033	72.67303	-2302.30869	-1105.72939	-948.89774	1
102	C	84.11186	69.37753	71.54378	-559.93427	-126.34590	542.30857	1
103	O	84.43466	68.21779	71.56471	-670.36003	-111.83166	76.36448	1
104	N	84.50249	70.29948	70.63768	-216.06729	-537.07037	446.51379	1
105	H	84.28773	71.30206	70.58491	-1251.96487	1074.12894	-2168.52500	1
106	H	85.14868	70.10419	69.86511	-2451.83023	-720.73851	944.84612	1
107	C	82.23543	71.93869	74.94471	546.24052	-563.88337	-85.11809	1
108	O	83.29243	71.38944	75.12606	71.70702	-307.40463	606.69673	1
109	N	81.47126	72.59104	75.83348	479.90863	-504.50129	222.86849	1
110	C	81.90691	72.81523	77.17635	75.51268	-600.91904	506.73700	1
111	H	83.01519	72.93299	77.16630	-2033.61562	1795.30103	254.89480	1
112	C	81.06635	74.10991	77.56612	523.79067	517.99274	284.45581	1
113	H	80.97884	74.25500	78.66608	1599.61665	-1127.00911	1895.98173	1
114	H	81.53459	75.03338	77.16102	-1881.83093	1275.82488	-1501.55016	1
115	C	79.69659	73.81969	76.87899	-600.05639	-404.05026	316.64485	1
116	H	79.06357	73.15697	77.51308	-2597.05260	496.36221	657.73249	1
117	H	79.09710	74.74415	76.68859	-1666.24518	1294.38985	-1723.90591	1
118	C	80.13160	73.08366	75.54094	493.57570	-129.95376	-602.56394	1
119	H	79.50398	72.20738	75.25460	-1469.08415	-637.84797	2204.22581	1
120	H	80.25007	73.75208	74.65976	-1038.97840	1068.88701	-2280.71789	1
121	C	81.39643	71.57388	78.10861	188.88100	241.05089	-727.87821	1
122	O	80.49290	70.84272	77.79243	531.88613	-49.19943	427.07960	1
123	N	82.01069	71.67140	79.29676	708.14833	26.93417	-179.73509	1
124	H	82.76361	72.35739	79.31837	-2232.69501	-1179.09831	1023.94816	1
125	C	81.86154	70.61648	80.31301	-738.32412	259.92548	104.42456	1
126	H	82.13336	69.67454	79.78156	2028.89123	1260.02983	1311.33324	1
127	C	82.77846	70.94029	81.47121	-258.65678	-345.52513	661.28459	1
128	H	82.57950	71.99212	81.77743	-1826.06090	-1801.46097	-918.64554	1
129	C	82.51313	70.04419	82.66875	-541.23400	531.92970	-218.41428	1
130	H	83.17085	70.30711	83.54198	1311.73218	-2219.18200	882.19023	1
131	H	81.44462	70.12344	83.01675	-1048.71298	1223.58118	-2196.97971	1
132	H	82.69857	68.96256	82.42405	419.06414	1903.92898	1903.44670	1
133	C	84.24499	70.84009	81.08705	-423.86431	152.66566	-648.55262	1
134	H	84.91814	71.13848	81.93671	105.68386	-2342.50080	1387.51238	1
135	H	84.52282	69.79020	80.79646	-1695.63603	-1484.73621	-1531.02793	1
136	H	84.49486	71.50117	80.21335	-1853.57121	-487.39031	1936.59343	1
137	C	80.28467	70.58784	80.80413	539.71428	-510.58232	267.58633	1
138	O	79.86432	69.64481	81.43559	-15.93758	-278.34085	-624.49402	1

```
N    79.62976  71.76746  80.57438     -676.81886       12.05292      276.17960          1
H    80.31142  72.24442  79.96755    -1694.44250     -373.84189    -2100.66098          1
C    78.29069  71.57375  80.03619      412.33078     -664.70360      108.35451          1
H    78.42007  70.85142  79.20032     1765.77476     1294.44366     1621.76594          1
C    77.46197  70.96900  81.22330     -179.66635     -721.95667     -264.73915          1
H    77.79768  69.91825  81.42323     -607.34922     2587.87032     -598.10018          1
H    77.68711  71.53114  82.15868     -359.69332    -1479.13954    -2259.74260          1
C    75.91986  70.98776  80.94305     -240.01076      616.02425      431.85322          1
H    75.51883  72.02669  80.98664    -1359.17131      782.54425     2227.99181          1
H    75.67952  70.60110  79.92741     -820.36667       85.81767    -2596.78715          1
C    75.31028  70.06562  82.07442     -513.07015      236.18570     -551.87280          1
H    75.67848  69.01981  81.96061     1816.11944    -1183.11722    -1650.94321          1
H    75.66497  70.39893  83.07684    -1800.04429    -1361.53473     1526.34643          1
C    73.76614  70.09850  82.02237      726.08972     -253.19732     -179.64713          1
H    73.31396  71.07586  82.28468     1224.27271     -456.51798    -2390.90304          1
H    73.32294  69.71386  81.08232     1206.40339    -1150.93514    -2154.90344          1
N    73.26021  69.11514  83.13342      329.70796     -633.68968     -155.67358          1
H    72.20740  69.05534  83.24534     1348.31305     1938.76845     1359.00643          1
H    73.51868  68.09079  83.02922     2096.92035     1446.54551     -966.48890          1
H    73.56366  69.31675  84.13029    -1197.07217    -2303.53719      827.28733          1
C    77.71522  73.00216  79.51891     -378.01186     -556.17301     -413.96481          1
O    76.84930  73.01071  78.67359     -409.44433     -307.55053     -453.30833          1
N    78.32444  74.04114  80.09761     -429.02477      524.70884      274.08502          1
H    79.00925  73.78540  80.80978     1706.34263    -1425.96036     1574.38926          1
C    77.73779  75.34041  80.10016      -45.25864     -626.22952      478.93267          1
H    78.52419  75.92798  80.62478     1988.63164      570.90773     1772.87287          1
C    77.51307  75.79242  78.59990      551.77128      -20.49526     -564.54996          1
H    78.22585  75.26193  77.92443     1434.61535    -1753.10253     1513.99471          1
H    76.49896  75.49001  78.25058     -714.67738     1819.09770     1898.36494          1
C    77.70601  77.34189  78.48870      446.99263     -555.77305      338.97221          1
H    77.02710  77.88736  79.18408     1953.68466     1892.25957     -161.67766          1
H    78.73894  77.63597  78.78084     -403.03785     2688.07032     -188.43591          1
C    77.41040  77.74469  76.98853      567.86262      335.74681      434.04421          1
H    78.19844  77.34095  76.31259    -1080.02275     1568.26081    -1948.78900          1
H    76.44845  77.29875  76.64130     -919.54108     2111.34692     1456.13463          1
C    77.34478  79.28808  76.90715      195.81897      173.33862     -745.11536          1
H    76.39416  79.72493  77.27389     1405.67949    -2301.21089     -390.08113          1
H    78.20549  79.81779  77.36273     -940.37328    -1118.32446     2299.72133          1
N    77.41333  79.67593  75.39146      319.26422      656.36007      -42.02424          1
H    77.22759  80.69210  75.14124     1961.03633      619.91968     1787.09422          1
H    78.34662  79.53305  74.91264      854.14343    -2156.80752     1429.08711          1
H    76.75564  79.16408  74.73421     1237.02905    -1378.50857    -1998.28500          1
C    76.35811  75.27662  80.96925      532.53319     -268.57236      517.55729          1
O    76.17589  74.47789  81.85824      266.16714     -290.73804     -558.88002          1
N    75.54247  76.25005  80.56010     -226.25352      640.49678     -270.32839          1
H    75.83938  76.84652  79.78888     2007.69063    -1035.27229     1523.50231          1
C    74.57560  76.75159  81.47688     -540.76542     -573.33303       49.50000          1
H    74.49040  75.93883  82.23543    -2374.20950    -1036.76023      843.75774          1
C    75.14109  78.11714  82.08882      -77.09082      666.87422     -415.84136          1
H    75.05029  78.92181  81.30843     -985.81834      617.19825     2463.91927          1
O    76.52105  78.11519  82.49594      293.29899     -391.65602     -477.80926          1
H    76.88639  78.82498  81.93071     2528.72714     -681.76262     -751.27122          1
C    74.11010  78.39604  83.24353      252.47363     -605.14473      440.05134          1
H    74.32219  79.36476  83.75314    -2052.65063     1456.49051    -1043.51830          1
H    73.05556  78.44590  82.85559    -2152.98800    -1661.97574     -161.70652          1
H    74.14552  77.60034  84.02389     -473.12726    -1584.27339     2165.61725          1
C    73.20877  77.05267  80.62700      360.36307      488.46713      505.10056          1
O    72.44636  77.92893  80.98878     -200.08817     -528.03552     -385.82850          1
N    73.04850  76.15546  79.66702     -597.54095     -226.44775      355.20479          1
H    73.81680  75.48974  79.58714     2179.23655    -1300.70981     -991.33835          1
C    72.09284  76.29503  78.62719     -687.68921      297.39807     -249.45179          1
H    72.12668  75.32243  78.08635     -349.16256    -2467.37713    -1101.72831          1
```

C	70.66290	76.60637	79.29082	672.47345	361.54328	-201.62880	1
H	70.71098	77.58058	79.84159	1541.41502	582.18425	2169.97026	1
H	69.88414	76.71281	78.49939	-2534.67717	60.25153	997.72295	1
C	70.30285	75.46723	80.25255	574.26774	-389.43119	-377.02542	1
C	69.72871	74.25381	79.76655	688.82679	-287.33580	-257.96133	1
H	69.48728	74.13384	78.69348	331.14182	1470.38163	2269.80013	1
C	70.57830	75.62316	81.64549	-383.52382	-675.04808	144.24917	1
H	71.00980	76.56724	82.02860	4.09847	1517.59662	2262.86397	1
C	69.47916	73.22555	80.67632	-134.76730	428.45285	-649.50303	1
H	69.03664	72.28629	80.30145	-903.23433	1600.49020	2011.53387	1
C	70.31162	74.56639	82.51669	-335.53281	-615.00040	-364.39058	1
H	70.53920	74.70283	83.58859	1596.45398	-1369.33862	1732.02659	1
C	69.76280	73.32877	82.06971	585.48547	-340.24194	-406.23938	1
O	69.50464	72.24455	82.94819	-419.15649	-173.04408	-511.94141	1
H	69.75421	72.66383	83.80646	-1071.84350	1559.71507	-1960.13012	1
C	72.53357	77.53462	77.68386	291.41878	-183.13091	710.72246	1
O	73.49026	78.24285	77.90437	379.87695	523.47396	-222.23622	1
N	71.70885	77.56314	76.63809	564.30591	373.09380	277.24371	1
H	70.90799	76.90582	76.53692	-120.59931	-2436.07824	-1214.35098	1
C	71.68493	78.80145	75.88227	404.07514	-299.37480	608.84009	1
H	71.32980	79.59103	76.58066	-1071.07882	-841.69025	2359.66467	1
C	73.15681	79.04861	75.36877	-683.12807	68.71770	-390.13096	1
H	73.85442	79.27686	76.22135	2068.88918	1596.58590	770.89603	1
H	73.55848	78.12463	74.88965	1379.86429	2342.49779	-179.85638	1
C	73.11282	80.24650	74.36379	-176.39967	537.49466	550.97268	1
H	72.52080	79.98979	73.45836	-396.28051	1942.22168	1869.33314	1
H	72.61474	81.13488	74.81157	-94.44700	586.00678	-2659.20016	1
C	74.62830	80.55905	73.99948	-291.69381	-718.18079	-150.72929	1
H	75.26989	80.75105	74.89334	-2303.38776	1089.76242	964.62032	1
H	75.13309	79.77865	73.38004	884.16729	2568.80533	-207.74283	1
N	74.66412	81.78840	73.24912	-594.49522	-252.81478	342.29276	1
H	73.89909	82.44016	73.09858	-1735.17426	2084.05705	-263.71605	1
C	75.70945	82.29104	72.69995	595.23177	-271.52487	442.22352	1
N	76.95633	81.56431	72.78877	585.53813	-424.79750	-105.81303	1
H	76.93200	80.54898	72.82486	959.88797	1327.56823	-2177.11905	1
H	77.60993	81.76367	72.03571	1578.48647	16.27378	-2220.76233	1
N	75.67549	83.51837	72.03425	-452.17763	-390.33541	-421.51853	1
H	74.89657	84.16960	71.92229	470.26107	1974.25629	-1817.92087	1
H	76.50604	83.95673	71.63402	1856.67307	-1573.97321	-1224.35416	1
C	70.71310	78.54206	74.61635	-531.47271	-581.45780	-55.06206	1
O	70.89395	77.55802	73.88640	-243.00837	192.73507	609.52485	1
N	69.86703	79.52949	74.45043	343.49288	-644.25607	38.09164	1
H	69.92358	80.24715	75.17272	-1691.16043	-1535.97525	-1484.73099	1
C	68.79987	79.55414	73.46678	542.66492	350.14337	454.42563	1
H	68.39272	80.59189	73.53765	1209.57979	-2396.95920	463.86744	1
C	69.38483	79.22760	72.09837	-737.14309	145.93010	242.71942	1
H	70.04100	78.32579	72.21941	-1050.18303	159.36419	-2509.06125	1
C	68.30840	78.89606	71.08350	-706.77696	-252.88343	-245.16230	1
H	68.74854	78.59191	70.09304	-1624.64039	-1665.49085	1417.86935	1
H	67.65481	78.05016	71.43399	2566.10521	598.51109	693.22305	1
H	67.63357	79.77651	70.89398	-1972.69758	107.10018	-1876.34312	1
C	70.25315	80.35811	71.57899	412.85148	672.58832	-27.69222	1
H	70.80368	80.06524	70.64222	1447.02277	1762.76533	-1490.78944	1
H	69.64165	81.27037	71.33442	1320.77379	-145.20455	2378.68491	1
H	71.02222	80.66975	72.33745	-1512.10848	1470.52031	1724.75147	1
C	67.63818	78.43873	73.90010	-607.11113	499.56689	73.73769	1
O	66.48343	78.78648	73.91181	357.97126	460.81534	356.68785	1
N	68.14674	77.23800	74.09708	536.55407	-368.62270	332.76306	1
H	69.17310	77.09314	74.00707	524.38866	2297.69985	1367.20941	1
C	67.33534	76.29837	74.79283	46.50277	-774.29190	-147.97904	1
H	66.39118	76.84815	75.01853	1676.51875	1883.29066	1032.55549	1
C	67.09338	75.01218	73.83802	-559.41456	-34.19198	-556.30433	1

```
H    68.05809  74.47178  73.69910       -64.66664       395.18769      2695.05373          1
H    66.40680  74.28599  74.33323     -2703.44278       -60.40056       333.79418          1
C    66.54156  75.44756  72.51006      -519.76435       377.31045       459.42366          1
H    67.27169  76.14686  72.04423     -1746.74323      1667.50644     -1261.73691          1
C    66.36163  74.25220  71.58182       570.95554        16.46261      -545.27700          1
H    65.93984  74.55898  70.59026      2391.58273        28.05277     -1305.07271          1
H    67.33374  73.73340  71.38042       175.75588       917.63458      2559.43867          1
H    65.66312  73.49262  72.01773     -1063.97851     -2155.34650      1283.00462          1
C    65.21473  76.17645  72.67174       193.76014         2.62946      -765.53141          1
H    64.74062  76.40035  71.68154       507.39394      -846.55087      2539.60089          1
H    64.47827  75.57253  73.26225      1337.57841      1818.19054      1526.01994          1
H    65.35399  77.16265  73.20412      1100.88635     -1172.91350     -2199.08909          1
C    68.13896  75.80112  76.08782      -549.21115       475.64486      -309.38218          1
O    69.26229  76.17461  76.36887       556.06106      -390.37755       -78.24250          1
O    67.47955  74.87180  76.91116      -572.27940      -310.19032      -209.75862          1
H    66.60255  74.83765  76.42227     -1380.08193      2212.14486       790.85944          1
```

Listing 83: C00083.xmolout

```
       276
  150.0000  150.0000  150.0000     0.0000     0.0000     0.0000***********    2.7750    11100
      0.2500    300.00   0.00   0.00   0.00  -1293.5132       0.0000    300.0000
H    60.66996  78.22632  70.47444     -2043.14101     -1757.64893       399.89339          1
N    61.44609  78.22228  71.13595       426.75047       490.45733      -334.42467          1
H    61.84231  77.27864  71.02556       442.25559     -1110.30854     -2448.52907          1
C    62.42125  79.21167  70.87528       415.32972      -666.39213        83.73172          1
H    61.98947  79.89384  70.11024       879.32219      2491.00447       667.35213          1
C    62.77628  79.98422  72.24645        31.35357       477.41777       628.23362          1
H    63.10441  79.24138  73.01196      1168.38489      2240.25337     -1019.71136          1
H    63.64116  80.67364  72.09507      -360.43770      2534.90498       931.67112          1
C    61.56993  80.74274  72.73125       463.18208       512.70242      -382.34388          1
H    60.71778  80.02971  72.80487      1725.29117      1863.52247      -987.07854          1
C    61.83799  81.32958  74.11224       710.42801      -144.27427      -313.15367          1
H    60.94564  81.87854  74.50954       364.20195     -1033.13725      2494.72524          1
H    62.09882  80.53446  74.85729      2567.61904       335.80679       847.48802          1
H    62.69079  82.05621  74.09140     -2587.99029       680.92867      -512.16411          1
C    61.18477  81.86566  71.77745       268.17616       741.64379       -40.42634          1
H    60.32460  82.46530  72.17291      1283.54314     -1945.37420     -1411.27812          1
H    62.03510  82.57633  71.61216     -1258.57796      1317.15249     -2026.02085          1
H    60.87846  81.47412  70.77377     -2502.84526       909.80597       575.92391          1
C    63.68875  78.34928  70.34545       619.36578      -458.64843      -172.09273          1
O    63.65895  77.13509  70.24279       -81.40433       433.87611       522.34594          1
N    64.67925  79.17933  70.03615       175.23996      -622.20190       341.55523          1
H    64.56886  80.17548  70.21921      -876.57256      -303.22879     -2561.90213          1
C    65.90185  78.61250  69.57759      -571.88214      -534.10783      -106.15033          1
H    65.58446  77.66559  69.08715     -2713.20841       -16.25800      -248.80809          1
C    66.53215  79.70913  68.59558       166.60354       288.16399      -716.09568          1
H    66.54892  80.69796  69.13009     -2097.85574      1636.78449      -586.18044          1
O    65.78300  79.92738  67.38432      -317.80580      -363.38227       484.43082          1
H    65.53112  80.86623  67.48413      1863.74748     -1451.84923      1357.29467          1
C    68.01289  79.22248  68.40147       503.80731       517.99032       318.51606          1
H    68.57308  79.89541  67.71110       640.61020     -2157.10410     -1536.29104          1
H    68.57452  79.21552  69.36785      1687.88455       465.60692      2087.56452          1
H    68.05789  78.19519  67.97088     -1293.07895     -1899.88581     -1463.57452          1
C    66.83816  78.36044  70.89563      -153.07550      -733.68902       248.70981          1
O    67.06332  79.27586  71.64034       186.15459      -480.78787      -449.34387          1
N    67.32041  77.10964  70.91110      -487.53139       543.18517        42.03850          1
C    68.15296  76.63275  71.97555      -699.35871        69.34393       360.10701          1
H    67.78295  77.10117  72.91598         2.81126      -221.99807     -2715.58072          1
C    67.96532  75.05786  71.87478      -507.46439      -592.59194       122.07761          1
H    68.80960  74.49151  72.32702     -1975.08116       525.87560     -1801.71556          1
H    67.03940  74.72079  72.39211      1588.27743     -1913.22100      1113.83566          1
```

```
C   67.85448  74.86259  70.33146    -613.62442    -457.55993    -194.14490    1
H   68.85347  74.94383  69.84470    1707.54637    -721.27992    1996.92523    1
H   67.43133  73.87746  70.03181     758.23357    2100.81767    1560.55011    1
C   66.92020  76.09069  69.94945    -352.41524     704.96076      49.21465    1
H   67.06685  76.48716  68.91745    1714.76886   -2070.42778     443.35792    1
H   65.83037  75.92239  70.13117    1224.15040    1801.50881    1636.97633    1
C   69.72573  76.97347  71.70144     566.85117     537.05831     117.62814    1
O   70.56749  76.76224  72.57298    -550.26779    -406.08867       4.21666    1
N   69.90084  77.36612  70.46015     566.34832    -425.77725     180.18749    1
H   69.04478  77.44805  69.91814     644.44502    2646.80550     -52.73430    1
C   71.17520  77.78996  69.99879     612.34801    -281.48662     411.56221    1
H   71.90506  77.22651  70.62287    1667.23498   -1490.75142    1556.16753    1
C   71.22036  77.52375  68.42448    -714.45746    -175.43074     286.98927    1
H   70.48776  78.21735  67.92661    -787.09484    1763.03619   -1922.46060    1
O   70.86660  76.19856  67.99094    -429.46514     516.51932    -128.40526    1
H   70.09584  76.38490  67.41626    1981.82785     436.16847   -1818.18182    1
C   72.68472  78.00035  68.10030     630.79189    -468.94448      76.03230    1
H   72.87890  78.00191  67.00282   -1775.71861    2062.30353     131.89849    1
H   72.87624  79.03395  68.47271   -1855.14608      88.94118    1993.53760    1
H   73.44354  77.33171  68.56883   -1504.82350    -780.94249   -2132.90992    1
C   71.24499  79.41919  70.20392     288.57638     618.62814    -397.00270    1
O   70.65099  80.16692  69.46215     321.70495    -185.26452     574.37218    1
N   71.98692  79.70751  71.27732    -447.05792    -446.53431     367.76425    1
H   72.40701  78.88749  71.71857     350.31316    1975.63644   -1843.31522    1
C   72.79760  80.92168  71.17695    -511.23061     374.03917    -471.51480    1
H   73.33499  80.78745  70.21111    2234.79579    -898.70890   -1273.45190    1
C   71.83277  82.14190  71.14373     529.53781    -159.34528     563.72604    1
H   72.39301  83.09650  70.98629     654.59388    2347.94251    1217.51488    1
H   71.08681  82.02144  70.31972   -1526.55661    2103.42026     817.87394    1
O   71.03628  82.64390  72.46256    -554.84278      14.12789     399.58769    1
H   70.38098  81.96073  72.41916    1297.06236    2061.84854    1220.68812    1
C   73.79824  80.85856  72.45276    -662.15484    -412.13544     123.62687    1
O   74.81402  80.21055  72.40056    -540.01537    -317.05804     274.91456    1
N   73.29759  81.57224  73.45854    -202.76299    -690.00100    -131.49145    1
H   72.35525  82.03329  73.34180    -702.62132   -2617.37191     281.70413    1
C   73.97676  81.58389  74.77070    -491.73914     576.34187     222.73509    1
H   75.02732  81.87890  74.54289    1977.17978   -1823.58850    -434.68928    1
C   73.24283  82.57469  75.65641    -649.50797    -328.66843     306.11224    1
H   72.21860  82.17977  75.84096    2075.07803    1452.16490   -1004.45904    1
C   73.94072  82.71929  76.99739    -615.18735    -489.99689     -70.96352    1
H   73.37639  83.40248  77.68904   -1226.32661   -1724.38906   -1716.47170    1
H   74.04699  81.72712  77.51634    2381.64273    -969.84735     900.46860    1
H   74.97489  83.14403  76.88055    2122.74991    1702.23946    -141.36228    1
C   73.08808  83.93249  74.99527    -501.86074    -479.61276    -376.42482    1
H   72.55152  84.66135  75.66170     993.79261   -2246.95401   -1177.81305    1
H   74.08552  84.38366  74.74086      42.71279    -626.15391   -2651.37258    1
H   72.50097  83.86436  74.03722   -2026.39012   -1590.33709    -887.82922    1
C   73.88093  80.06845  75.35989    -672.89153     382.57394    -156.34072    1
O   74.87119  79.45796  75.92465     410.04627     221.86223     500.35950    1
N   72.71148  79.49626  75.22173    -378.11782    -443.79414    -441.10984    1
H   72.12095  80.21217  74.79399    2404.15973    -568.14894    1149.30085    1
C   72.53277  78.42497  76.15792    -544.54484     466.16019     331.29119    1
H   72.35167  78.96988  77.10657   -1409.80988    -170.17955    2325.32679    1
C   71.28596  77.57942  75.75103    -184.48284     116.78282    -758.89137    1
H   70.34092  78.08049  76.06741    1893.78641    1861.14652     611.04528    1
H   71.20317  77.47530  74.63663   -2170.79665    -974.19311    1327.50117    1
C   71.43656  76.18712  76.45764     389.51668     672.81878    -138.49152    1
H   72.40556  75.72756  76.16205     510.64577    1933.03327    1851.02484    1
H   71.50282  76.31164  77.56046   -2302.30869   -1105.72939    -948.89774    1
C   70.23579  75.21811  76.09596    -559.93427    -126.34590     542.30857    1
O   69.78635  74.46146  76.92076    -670.36003    -111.83166      76.36448    1
N   69.85707  75.30844  74.80788    -216.06729    -537.07037     446.51379    1
```

105	H	70.24686	75.89712	74.01829	-1251.96487	1074.12894	-2168.52500	1
106	H	69.11713	74.69532	74.44114	-2451.83023	-720.73851	944.84612	1
107	C	74.06826	77.70086	76.25124	546.24052	-563.88337	-85.11809	1
108	O	74.41339	77.26341	77.35261	71.70702	-307.40463	606.69673	1
109	N	74.33836	77.00979	75.07179	479.90863	-504.50129	222.86849	1
110	C	75.20669	75.91413	75.05771	75.51268	-600.91904	506.73700	1
111	H	75.28560	75.55063	76.11271	-2033.61562	1795.30103	254.89480	1
112	C	74.45620	74.97593	73.99491	523.79067	517.99274	284.45581	1
113	H	75.11437	74.17013	73.60054	1599.61665	-1127.00911	1895.98173	1
114	H	73.56612	74.48093	74.44204	-1881.83093	1275.82488	-1501.55016	1
115	C	74.04881	76.02063	72.91749	-600.05639	-404.05026	316.64485	1
116	H	74.88210	76.21186	72.20501	-2597.05260	496.36221	657.73249	1
117	H	73.17958	75.69635	72.30234	-1666.24518	1294.38985	-1723.90591	1
118	C	73.73008	77.31608	73.79872	493.57570	-129.95376	-602.56394	1
119	H	74.19203	78.26886	73.43380	-1469.08415	-637.84797	2204.22581	1
120	H	72.64126	77.49153	73.98673	-1038.97840	1068.88701	-2280.71789	1
121	C	76.67509	76.20128	74.32998	188.88100	241.05089	-727.87821	1
122	O	77.25924	75.28390	73.81024	531.88613	-49.19943	427.07960	1
123	N	77.14239	77.47740	74.50699	708.14833	26.93417	-179.73509	1
124	H	76.34908	77.88481	75.03364	-2232.69501	-1179.09831	1023.94816	1
125	C	77.44889	78.16894	73.26393	-738.32412	259.92548	104.42456	1
126	H	76.46082	78.35356	72.78874	2028.89123	1260.02983	1311.33324	1
127	C	78.19126	79.49960	73.67207	-258.65678	-345.52513	661.28459	1
128	H	79.03994	79.28368	74.35764	-1826.06090	-1801.46097	-918.64554	1
129	C	78.69081	80.19952	72.35848	-541.23400	531.92970	-218.41428	1
130	H	79.27173	81.12585	72.57760	1311.73218	-2219.18200	882.19023	1
131	H	79.35118	79.52095	71.76391	-1048.71298	1223.58118	-2196.97971	1
132	H	77.83694	80.48553	71.70059	419.06414	1903.92898	1903.44670	1
133	C	77.16299	80.47642	74.33806	-423.86431	152.66566	-648.55262	1
134	H	77.55764	81.51785	74.40653	105.68386	-2342.50080	1387.51238	1
135	H	76.20400	80.50191	73.73118	-1695.63603	-1484.73621	-1531.02793	1
136	H	76.90386	80.15012	75.37488	-1853.57121	-487.39031	1936.59343	1
137	C	78.47464	77.38404	72.29110	539.71428	-510.58232	267.58633	1
138	O	78.41840	77.54536	71.09009	-15.93758	-278.34085	-624.49402	1
139	N	79.54875	76.84817	72.98914	-676.81886	12.05292	276.17960	1
140	H	79.08761	76.69234	73.90090	-1694.44250	-373.84189	-2100.66098	1
141	C	80.13974	75.72193	72.28170	412.33078	-664.70360	108.35451	1
142	H	79.28815	75.06981	71.99108	1765.77476	1294.44366	1621.76594	1
143	C	80.92510	76.33163	71.06869	-179.66635	-721.95667	-264.73915	1
144	H	80.23031	76.86261	70.36599	-607.34922	2587.87032	-598.10018	1
145	H	81.63542	77.10792	71.43576	-359.69332	-1479.13954	-2259.74260	1
146	C	81.69591	75.18286	70.32949	-240.01076	616.02425	431.85322	1
147	H	82.33707	74.61670	71.04217	-1359.17131	782.54425	2227.99181	1
148	H	80.99244	74.44111	69.88991	-820.36667	85.81767	-2596.78715	1
149	C	82.57323	75.87588	69.21234	-513.07015	236.18570	-551.87280	1
150	H	81.92842	76.41516	68.48067	1816.11944	-1183.11722	-1650.94321	1
151	H	83.23657	76.65182	69.65944	-1800.04429	-1361.53473	1526.34643	1
152	C	83.41495	74.78783	68.50582	726.08972	-253.19732	-179.64713	1
153	H	84.14214	74.26857	69.16148	1224.27271	-456.51798	-2390.90304	1
154	H	82.82485	74.04553	67.93235	1206.40339	-1150.93514	-2154.90344	1
155	N	84.29851	75.51139	67.43378	329.70796	-633.68968	-155.67358	1
156	H	84.93944	74.88473	66.86671	1348.31305	1938.76845	1359.00643	1
157	H	83.79102	76.03989	66.66577	2096.92035	1446.54551	-966.48890	1
158	H	84.98239	76.24235	67.78716	-1197.07217	-2303.53719	827.28733	1
159	C	81.20738	75.02252	73.28764	-378.01186	-556.17301	-413.96481	1
160	O	82.21212	75.57383	73.67798	-409.44433	-307.55053	-453.30833	1
161	N	80.83128	73.75652	73.50473	-429.02477	524.70884	274.08502	1
162	H	79.92109	73.44498	73.16369	1706.34263	-1425.96036	1574.38926	1
163	C	81.69579	73.00565	74.36724	-45.25864	-626.22952	478.93267	1
164	H	82.71393	73.31253	74.03475	1988.63164	570.90773	1772.87287	1
165	C	81.43607	73.53567	75.83731	551.77128	-20.49526	-564.54996	1
166	H	81.57371	74.64049	75.87603	1434.61535	-1753.10253	1513.99471	1

167	H	80.38531	73.34857	76.15398	-714.67738	1819.09770	1898.36494	1
168	C	82.44567	72.80096	76.77925	446.99263	-555.77305	338.97221	1
169	H	82.19265	71.71127	76.83791	1953.68466	1892.25957	-161.67766	1
170	H	83.48299	72.85362	76.37803	-403.03785	2688.07032	-188.43591	1
171	C	82.35824	73.49355	78.19591	567.86262	335.74681	434.04421	1
172	H	82.75649	74.53375	78.15950	-1080.02275	1568.26081	-1948.78900	1
173	H	81.29293	73.59042	78.52203	-919.54108	2111.34692	1456.13463	1
174	C	83.17687	72.61173	79.17547	195.81897	173.33862	-745.11536	1
175	H	82.87100	71.54467	79.20414	1405.67949	-2301.21089	-390.08113	1
176	H	84.27453	72.68250	79.03194	-940.37328	-1118.32446	2299.72133	1
177	N	82.93055	73.11511	80.61824	319.26422	656.36007	-42.02424	1
178	H	83.70725	72.85364	81.29168	1961.03633	619.91968	1787.09422	1
179	H	82.91084	74.16469	80.76414	854.14343	-2156.80752	1429.08711	1
180	H	82.01209	72.72942	81.12946	1237.02905	-1378.50857	-1998.28500	1
181	C	81.42249	71.39432	74.30823	532.53319	-268.57236	517.55729	1
182	O	82.26084	70.67594	74.80059	266.16714	-290.73804	-558.88002	1
183	N	80.26129	71.07148	73.76230	-226.25352	640.49678	-270.32839	1
184	H	79.68916	71.81936	73.37128	2007.69063	-1035.27229	1523.50231	1
185	C	79.82698	69.70749	73.66974	-540.76542	-573.33303	49.50000	1
186	H	78.94795	69.79529	72.98864	-2374.20950	-1036.76023	843.75774	1
187	C	81.00601	68.81919	73.02246	-77.09082	666.87422	-415.84136	1
188	H	81.78409	68.67219	73.82281	-985.81834	617.19825	2463.91927	1
189	O	81.66899	69.40642	71.89198	293.29899	-391.65602	-477.80926	1
190	H	82.52452	69.65611	72.30350	2528.72714	-681.76262	-751.27122	1
191	C	80.29962	67.45042	72.71094	252.47363	-605.14473	440.05134	1
192	H	81.02576	66.70241	72.31595	-2052.65063	1456.49051	-1043.51830	1
193	H	79.82321	67.00071	73.61761	-2152.98800	-1661.97574	-161.70652	1
194	H	79.50190	67.57110	71.94156	-473.12726	-1584.27339	2165.61725	1
195	C	79.41134	69.22622	75.12955	360.36307	488.46713	505.10056	1
196	O	79.55708	67.93366	75.48923	-200.08817	-528.03552	-385.82850	1
197	N	78.82682	70.13499	75.80393	-597.54095	-226.44775	355.20479	1
198	H	78.79902	70.97602	75.20547	2179.23655	-1300.70981	-991.33835	1
199	C	78.35744	70.11541	77.14296	-687.68921	297.39807	-249.45179	1
200	H	77.42902	70.72546	77.12298	-349.16256	-2467.37713	-1101.72831	1
201	C	78.13866	68.60927	77.66059	672.47345	361.54328	-201.62880	1
202	H	79.11344	68.05852	77.66091	1541.41502	582.18425	2169.97026	1
203	H	77.76261	68.61870	78.71867	-2534.67717	60.25153	997.72295	1
204	C	77.15333	67.97161	76.69912	574.26774	-389.43119	-377.02542	1
205	C	75.75241	68.14032	76.71900	688.82679	-287.33580	-257.96133	1
206	H	75.25637	68.69383	77.54084	331.14182	1470.38163	2269.80013	1
207	C	77.83729	67.20394	75.62218	-383.52382	-675.04808	144.24917	1
208	H	78.57843	66.40065	75.96454	4.09847	1517.59662	2262.86397	1
209	C	75.00743	67.63935	75.63436	-134.76730	428.45285	-649.50303	1
210	H	73.91435	67.77881	75.63564	-903.23433	1600.49020	2011.53387	1
211	C	76.97848	66.79888	74.49568	-335.53281	-615.00040	-364.39058	1
212	H	77.43617	66.28776	73.63687	1596.45398	-1369.33862	1732.02659	1
213	C	75.59869	66.98565	74.51092	585.48547	-340.24194	-406.23938	1
214	O	74.73069	66.55218	73.42101	-419.15649	-173.04408	-511.94141	1
215	H	75.39454	66.16060	72.83219	-1071.84350	1559.71507	-1960.13012	1
216	C	79.48647	70.76206	78.11715	291.41878	-183.13091	710.72246	1
217	O	80.66885	70.50876	78.04388	379.87695	523.47396	-222.23622	1
218	N	78.85716	71.48337	79.05131	564.30591	373.09380	277.24371	1
219	H	77.84248	71.58697	79.00713	-120.59931	-2436.07824	-1214.35098	1
220	C	79.68094	72.36447	79.82233	404.07514	-299.37480	608.84009	1
221	H	80.67619	72.35182	79.31101	-1071.07882	-841.69025	2359.66467	1
222	C	78.97243	73.78490	79.86297	-683.12807	68.71770	-390.13096	1
223	H	78.77558	74.16952	78.83721	2068.88918	1596.58590	770.89603	1
224	H	77.97506	73.70996	80.35208	1379.86429	2342.49779	-179.85638	1
225	C	79.91584	74.73754	80.65865	-176.39967	537.49466	550.97268	1
226	H	80.21848	74.26901	81.62478	-396.28051	1942.22168	1869.33314	1
227	H	80.86423	74.92094	80.10692	-94.44700	586.00678	-2659.20016	1
228	C	79.11927	76.10005	80.90420	-291.69381	-718.18079	-150.72929	1

```
H   78.88947  76.70608  79.99111    -2303.38776     1089.76242      964.62032          1
H   78.17513  76.00209  81.49866      884.16729     2568.80533     -207.74283          1
N   80.04242  76.81269  81.68460     -594.49522     -252.81478      342.29276          1
H   80.87569  76.32046  81.96787    -1735.17426     2084.05705     -263.71605          1
C   79.89313  78.01760  82.06243      595.23177     -271.52487      442.22352          1
N   78.72836  78.68406  81.66710      585.53813     -424.79750     -105.81303          1
H   77.94793  78.34500  81.10586      959.88797     1327.56823    -2177.11905          1
H   78.49424  79.64756  81.90797     1578.48647       16.27378    -2220.76233          1
N   80.98416  78.43915  82.83549     -452.17763     -390.33541     -421.51853          1
H   81.82853  77.94727  83.12400      470.26107     1974.25629    -1817.92087          1
H   81.05604  79.37416  83.23834     1856.67307    -1573.97321    -1224.35416          1
C   79.80135  71.83812  81.35803     -531.47271     -581.45780      -55.06206          1
O   80.81959  72.10012  82.01451     -243.00837      192.73507      609.52485          1
N   78.70025  71.25464  81.76552      343.49288     -644.25607       38.09164          1
H   77.99917  71.24932  81.02713    -1691.16043    -1535.97525    -1484.73099          1
C   78.69076  70.29903  82.87597      542.66492      350.14337      454.42563          1
H   79.71902  69.86434  82.85351     1209.57979    -2396.95920      463.86744          1
C   78.41285  71.04841  84.17172     -737.14309      145.93010      242.71942          1
H   77.38241  71.46443  84.10677    -1050.18303      159.36419    -2509.06125          1
C   78.47465  70.12044  85.36978     -706.77696     -252.88343     -245.16230          1
H   78.22365  70.65490  86.32718    -1624.64039    -1665.49085     1417.86935          1
H   77.74609  69.26336  85.25860     2566.10521      598.51109      693.22305          1
H   79.49951  69.67391  85.49628    -1972.69758      107.10018    -1876.34312          1
C   79.36791  72.21233  84.36200      412.85148      672.58832      -27.69222          1
H   79.15956  72.78224  85.30907     1447.02277     1762.76533    -1490.78944          1
H   80.43719  71.86343  84.40137     1320.77379     -145.20455     2378.68491          1
H   79.30282  72.93824  83.50265    -1512.10848     1470.52031     1724.75147          1
C   77.49444  69.21921  82.50921     -607.11113      499.56689       73.73769          1
O   76.84029  68.70410  83.39028      357.97126      460.81534      356.68785          1
N   77.47049  68.96534  81.20075      536.55407     -368.62270      332.76306          1
H   78.18853  69.47989  80.68968      524.38866     2297.69985     1367.20941          1
C   76.20126  68.77285  80.56697       46.50277     -774.29190     -147.97904          1
H   76.43326  68.62665  79.48503     1676.51875     1883.29066     1032.55549          1
C   75.49495  67.48264  81.21403     -559.41456      -34.19198     -556.30433          1
H   75.50940  67.55127  82.33722      -64.66664      395.18769     2695.05373          1
H   74.42379  67.40075  80.90712    -2703.44278      -60.40056      333.79418          1
C   76.29938  66.29623  80.74445     -519.76435      377.31045      459.42366          1
H   77.37084  66.60044  80.71631    -1746.74323     1667.50644    -1261.73691          1
C   76.16898  65.13212  81.71744      570.95554       16.46261     -545.27700          1
H   76.77680  64.24938  81.38952     2391.58273       28.05277    -1305.07271          1
H   76.51930  65.41373  82.74451      175.75588      917.63458     2559.43867          1
H   75.10690  64.78787  81.81104    -1063.97851    -2155.34650     1283.00462          1
C   75.87574  65.86027  79.34877      193.76014        2.62946     -765.53141          1
H   76.50058  65.00952  78.97294      507.39394     -846.55087     2539.60089          1
H   74.80807  65.52075  79.32937     1337.57841     1818.19054     1526.01994          1
H   75.97079  66.69221  78.60559     1100.88635    -1172.91350    -2199.08909          1
C   75.40840  70.16149  80.75768     -549.21115      475.64486     -309.38218          1
O   75.52452  71.04544  79.94862      556.06106     -390.37755      -78.24250          1
O   74.61503  70.33264  81.91568     -572.27940     -310.19032     -209.75862          1
H   74.96556  69.57284  82.46299    -1380.08193     2212.14486      790.85944          1
```

Listing 84: C00084.xmolout

```
     276
  150.0000  150.0000  150.0000     0.0000     0.0000     0.0000***********    2.7750    11100
       0.2500     300.00    0.00    0.00    0.00   -1293.5003       0.0000     300.0000
H   74.99049  62.39079  74.38730    -2043.14101    -1757.64893      399.89339          1
N   74.91929  63.40443  74.31773      426.75047      490.45733     -334.42467          1
H   74.61490  63.66701  75.25539      442.25559    -1110.30854    -2448.52907          1
C   76.11801  64.05675  73.95908      415.32972     -666.39213       83.73172          1
H   76.66978  63.39464  73.25746      879.32219     2491.00447      667.35213          1
C   75.72620  65.49312  73.33932       31.35357      477.41777      628.23362          1
```

H	75.22750	66.09959	74.13331	1168.38489	2240.25337	-1019.71136	1
H	76.64416	66.05879	73.04904	-360.43770	2534.90498	931.67112	1
C	74.80008	65.31484	72.16317	463.18208	512.70242	-382.34388	1
H	74.00320	64.59446	72.45721	1725.29117	1863.52247	-987.07854	1
C	74.14230	66.64081	71.80288	710.42801	-144.27427	-313.15367	1
H	73.43243	66.53107	70.94279	364.20195	-1033.13725	2494.72524	1
H	73.55954	67.06072	72.66328	2567.61904	335.80679	847.48802	1
H	74.90170	67.41091	71.50966	-2587.99029	680.92867	-512.16411	1
C	75.53705	64.76057	70.95226	268.17616	741.64379	-40.42634	1
H	74.85373	64.64263	70.07161	1283.54314	-1945.37420	-1411.27812	1
H	76.37360	65.43538	70.63458	-1258.57796	1317.15249	-2026.02085	1
H	75.98288	63.75455	71.16376	-2502.84526	909.80597	575.92391	1
C	76.87865	64.34746	75.37110	619.36578	-458.64843	-172.09273	1
O	76.28551	64.82106	76.32532	-81.40433	433.87611	522.34594	1
N	78.17216	64.09436	75.23967	175.23996	-622.20190	341.55523	1
H	78.51533	63.73578	74.34961	-876.57256	-303.22879	-2561.90213	1
C	78.98174	64.07271	76.41206	-571.88214	-534.10783	-106.15033	1
H	78.25920	64.25095	77.24205	-2713.20841	-16.25800	-248.80809	1
C	79.73713	62.65729	76.47935	166.60354	288.16399	-716.09568	1
H	80.42293	62.58125	75.59209	-2097.85574	1636.78449	-586.18044	1
O	78.89442	61.49652	76.45113	-317.80580	-363.38227	484.43082	1
H	79.21334	61.04898	75.64070	1863.74748	-1451.84923	1357.29467	1
C	80.59791	62.84822	77.78140	503.80731	517.99032	318.51606	1
H	81.29760	61.99459	77.93829	640.61020	-2157.10410	-1536.29104	1
H	81.21237	63.78267	77.73726	1687.88455	465.60692	2087.56452	1
H	79.95534	62.91811	78.68953	-1293.07895	-1899.88581	-1463.57452	1
C	80.10283	65.23902	76.22547	-153.07550	-733.68902	248.70981	1
O	81.20216	64.95522	75.77679	186.15459	-480.78787	-449.34387	1
N	79.64130	66.42483	76.62343	-487.53139	543.18517	42.03850	1
C	80.30006	67.64777	76.26815	-699.35871	69.34393	360.10701	1
H	80.71850	67.48768	75.24774	2.81126	-221.99807	-2715.58072	1
C	79.10519	68.68394	76.38120	-507.46439	-592.59194	122.07761	1
H	79.44829	69.74021	76.44270	-1975.08116	525.87560	-1801.71556	1
H	78.42399	68.61989	75.50361	1588.27743	-1913.22100	1113.83566	1
C	78.40521	68.17110	77.67984	-613.62442	-457.55993	-194.14490	1
H	78.96774	68.49474	78.58495	1707.54637	-721.27992	1996.92523	1
H	77.36453	68.54650	77.80538	758.23357	2100.81767	1560.55011	1
C	78.48355	66.59723	77.49263	-352.41524	704.96076	49.21465	1
H	78.67655	66.01965	78.42771	1714.76886	-2070.42778	443.35792	1
H	77.61337	66.12930	76.94219	1224.15040	1801.50881	1636.97633	1
C	81.46923	67.89900	77.38967	566.85117	537.05831	117.62814	1
O	81.45243	68.86288	78.11314	-550.26779	-406.08867	4.21666	1
N	82.36767	66.92149	77.31303	566.34832	-425.77725	180.18749	1
H	82.22568	66.12321	76.65427	644.44502	2646.80550	-52.73430	1
C	83.47361	67.01181	78.22072	612.34801	-281.48662	411.56221	1
H	83.00284	67.42196	79.14359	1667.23498	-1490.75142	1556.16753	1
C	84.05723	65.53622	78.39729	-714.45746	-175.43074	286.98927	1
H	84.46436	65.19000	77.40796	-787.09484	1763.03619	-1922.46060	1
O	83.09440	64.53380	78.78999	-429.46514	516.51932	-128.40526	1
H	83.16770	63.91123	78.03968	1981.82785	436.16847	-1818.18182	1
C	85.24913	65.72034	79.40542	630.79189	-468.94448	76.03230	1
H	85.72690	64.74328	79.65040	-1775.71861	2062.30353	131.89849	1
H	86.04965	66.38325	78.99587	-1855.14608	88.94118	1993.53760	1
H	84.90490	66.16951	80.36600	-1504.82350	-780.94249	-2132.90992	1
C	84.57672	68.06165	77.65096	288.57638	618.62814	-397.00270	1
O	85.48440	68.41787	78.40153	321.70495	-185.26452	574.37218	1
N	84.36384	68.41374	76.40845	-447.05792	-446.53431	367.76425	1
H	83.52583	68.04892	75.95785	350.31316	1975.63644	-1843.31522	1
C	85.32777	69.08612	75.59456	-511.23061	374.03917	-471.51480	1
H	86.30568	68.67255	75.93124	2234.79579	-898.70890	-1273.45190	1
C	84.89739	68.69817	74.11026	529.53781	-159.34528	563.72604	1
H	85.51429	69.29523	73.39273	654.59388	2347.94251	1217.51488	1

H	85.19033	67.62993	73.91960	-1526.55661	2103.42026	817.87394	1
O	83.45948	68.77476	73.80585	-554.84278	14.12789	399.58769	1
H	83.50456	69.03990	72.87600	1297.06236	2061.84854	1220.68812	1
C	85.26148	70.68328	75.88109	-662.15484	-412.13544	123.62687	1
O	85.17776	71.48659	74.98549	-540.01537	-317.05804	274.91456	1
N	85.42250	70.88468	77.18850	-202.76299	-690.00100	-131.49145	1
H	85.56249	70.04034	77.80019	-702.62132	-2617.37191	281.70413	1
C	85.28378	72.19202	77.81935	-491.73914	576.34187	222.73509	1
H	85.66832	72.02507	78.85346	1977.17978	-1823.58850	-434.68928	1
C	86.12716	73.19108	77.04953	-649.50797	-328.66843	306.11224	1
H	85.79230	73.16832	75.97882	2075.07803	1452.16490	-1004.45904	1
C	85.97231	74.60858	77.56583	-615.18735	-489.99689	-70.96352	1
H	86.63331	75.32881	77.00866	-1226.32661	-1724.38906	-1716.47170	1
H	84.91228	74.97622	77.46059	2381.64273	-969.84735	900.46860	1
H	86.24129	74.68312	78.65569	2122.74991	1702.23946	-141.36228	1
C	87.59437	72.79802	77.07504	-501.86074	-479.61276	-376.42482	1
H	88.22363	73.48369	76.44432	993.79261	-2246.95401	-1177.81305	1
H	88.01230	72.82625	78.11863	42.71279	-626.15391	-2651.37258	1
H	87.74264	71.75283	76.68691	-2026.39012	-1590.33709	-887.82922	1
C	83.66550	72.59762	77.87142	-672.89153	382.57394	-156.34072	1
O	83.35048	73.62776	78.41602	410.04627	221.86223	500.35950	1
N	82.89341	71.69811	77.29347	-378.11782	-443.79414	-441.10984	1
H	83.38630	70.87714	76.94218	2404.15973	-568.14894	1149.30085	1
C	81.46843	71.77779	77.28969	-544.54484	466.16019	331.29119	1
H	81.19445	70.86807	76.70752	-1409.80988	-170.17955	2325.32679	1
C	81.00472	71.68728	78.81025	-184.48284	116.78282	-758.89137	1
H	81.50564	70.81740	79.29732	1893.78641	1861.14652	611.04528	1
H	81.33489	72.59245	79.37068	-2170.79665	-974.19311	1327.50117	1
C	79.45588	71.52341	78.85737	389.51668	672.81878	-138.49152	1
H	78.96073	72.44317	78.46116	510.64577	1933.03327	1851.02484	1
H	79.13716	70.71983	78.15937	-2302.30869	-1105.72939	-948.89774	1
C	78.88698	71.20390	80.29109	-559.93427	-126.34590	542.30857	1
O	77.70642	71.00716	80.42154	-670.36003	-111.83166	76.36448	1
N	79.81699	71.18188	81.26940	-216.06729	-537.07037	446.51379	1
H	80.82640	71.36284	81.21903	-1251.96487	1074.12894	-2168.52500	1
H	79.60308	71.01338	82.25859	-2451.83023	-720.73851	944.84612	1
C	80.93933	73.15590	76.62928	546.24052	-563.88337	-85.11809	1
O	80.06017	73.78048	77.20865	71.70702	-307.40463	606.69673	1
N	81.45576	73.39880	75.42887	479.90863	-504.50129	222.86849	1
C	81.31164	74.69301	74.81193	75.51268	-600.91904	506.73700	1
H	81.45773	75.41957	75.64431	-2033.61562	1795.30103	254.89480	1
C	82.45104	74.66467	73.70874	523.79067	517.99274	284.45581	1
H	82.25275	75.36730	72.86824	1599.61665	-1127.00911	1895.98173	1
H	83.43979	74.94546	74.13616	-1881.83093	1275.82488	-1501.55016	1
C	82.43119	73.16628	73.27255	-600.05639	-404.05026	316.64485	1
H	81.56346	72.95795	72.60651	-2597.05260	496.36221	657.73249	1
H	83.34532	72.84686	72.72257	-1666.24518	1294.38985	-1723.90591	1
C	82.27297	72.45179	74.68278	493.57570	-129.95376	-602.56394	1
H	81.76174	71.46051	74.66916	-1469.08415	-637.84797	2204.22581	1
H	83.22672	72.36645	75.26136	-1038.97840	1068.88701	-2280.71789	1
C	79.82084	74.80018	74.16747	188.88100	241.05089	-727.87821	1
O	79.65870	74.69923	72.98448	531.88613	-49.19943	427.07960	1
N	78.91667	75.03239	75.13669	708.14833	26.93417	-179.73509	1
H	79.21783	74.91348	76.13368	-2232.69501	-1179.09831	1023.94816	1
C	77.49174	75.05373	74.78299	-738.32412	259.92548	104.42456	1
H	77.51217	74.66118	73.73796	2028.89123	1260.02983	1311.33324	1
C	76.75323	74.16639	75.76120	-258.65678	-345.52513	661.28459	1
H	76.90027	74.57331	76.78644	-1826.06090	-1801.46097	-918.64554	1
C	75.26479	74.13451	75.46179	-541.23400	531.92970	-218.41428	1
H	74.69869	73.53656	76.22722	1311.73218	-2219.18200	882.19023	1
H	74.82036	75.16771	75.44664	-1048.71298	1223.58118	-2196.97971	1
H	75.05673	73.67371	74.45764	419.06414	1903.92898	1903.44670	1

C	77.30957	72.75264	75.77235	-423.86431	152.66566	-648.55262	1
H	76.79746	72.10755	76.53781	105.68386	-2342.50080	1387.51238	1
H	77.18638	72.25148	74.77370	-1695.63603	-1484.73621	-1531.02793	1
H	78.41028	72.74827	76.01188	-1853.57121	-487.39031	1936.59343	1
C	77.01371	76.65018	74.79164	539.71428	-510.58232	267.58633	1
O	77.27084	77.30942	73.80909	-15.93758	-278.34085	-624.49402	1
N	76.41205	77.03191	75.90038	-676.81886	12.05292	276.17960	1
H	76.33515	76.33954	76.64080	-1694.44250	-373.84189	-2100.66098	1
C	75.88941	78.34553	76.05460	412.33078	-664.70360	108.35451	1
H	75.77799	78.43856	77.15827	1765.77476	1294.44366	1621.76594	1
C	76.89656	79.37120	75.39575	-179.66635	-721.95667	-264.73915	1
H	77.94429	79.13663	75.69294	-607.34922	2587.87032	-598.10018	1
H	76.86929	79.27859	74.27692	-359.69332	-1479.13954	-2259.74260	1
C	76.49528	80.82302	75.82560	-240.01076	616.02425	431.85322	1
H	75.44590	81.04238	75.52036	-1359.17131	782.54425	2227.99181	1
H	76.52881	80.94556	76.93141	-820.36667	85.81767	-2596.78715	1
C	77.50575	81.80476	75.10808	-513.07015	236.18570	-551.87280	1
H	78.54296	81.65143	75.48542	1816.11944	-1183.11722	-1650.94321	1
H	77.54840	81.59333	74.01464	-1800.04429	-1361.53473	1526.34643	1
C	77.03634	83.25687	75.35692	726.08972	-253.19732	-179.64713	1
H	76.06206	83.51179	74.89413	1224.27271	-456.51798	-2390.90304	1
H	77.04460	83.57125	76.41952	1206.40339	-1150.93514	-2154.90344	1
N	78.06850	84.20160	74.65242	329.70796	-633.68968	-155.67358	1
H	77.86340	85.24028	74.71709	1348.31305	1938.76845	1359.00643	1
H	79.07041	84.16531	75.00093	2096.92035	1446.54551	-966.48890	1
H	78.19730	84.07899	73.60591	-1197.07217	-2303.53719	827.28733	1
C	74.45514	78.50705	75.27071	-378.01186	-556.17301	-413.96481	1
O	74.28348	79.38737	74.45752	-409.44433	-307.55053	-453.30833	1
N	73.58061	77.59971	75.69411	-429.02477	524.70884	274.08502	1
H	73.88043	76.97031	76.43731	1706.34263	-1425.96036	1574.38926	1
C	72.21445	77.79769	75.34272	-45.25864	-626.22952	478.93267	1
H	72.25856	78.40538	74.40882	1988.63164	570.90773	1772.87287	1
C	71.54696	76.37417	75.21162	551.77128	-20.49526	-564.54996	1
H	72.18212	75.69592	74.59825	1434.61535	-1753.10253	1513.99471	1
H	71.46563	75.89178	76.21223	-714.67738	1819.09770	1898.36494	1
C	70.13151	76.55651	74.56678	446.99263	-555.77305	338.97221	1
H	69.47967	77.21454	75.19187	1953.68466	1892.25957	-161.67766	1
H	70.20090	77.05093	73.57167	-403.03785	2688.07032	-188.43591	1
C	69.52425	75.10226	74.43938	567.86262	335.74681	434.04421	1
H	70.18435	74.45156	73.82075	-1080.02275	1568.26081	-1948.78900	1
H	69.46593	74.61101	75.43801	-919.54108	2111.34692	1456.13463	1
C	68.11890	75.21535	73.80463	195.81897	173.33862	-745.11536	1
H	67.37103	75.74201	74.43072	1405.67949	-2301.21089	-390.08113	1
H	68.10723	75.62683	72.77553	-940.37328	-1118.32446	2299.72133	1
N	67.57287	73.75494	73.65824	319.26422	656.36007	-42.02424	1
H	66.60144	73.66268	73.24292	1961.03633	619.91968	1787.09422	1
H	68.12704	73.09322	73.04006	854.14343	-2156.80752	1429.08711	1
H	67.47325	73.18727	74.54969	1237.02905	-1378.50857	-1998.28500	1
C	71.47788	78.57976	76.59854	532.53319	-268.57236	517.55729	1
O	70.95130	77.97607	77.50299	266.16714	-290.73804	-558.88002	1
N	71.58541	79.88823	76.41501	-226.25352	640.49678	-270.32839	1
H	72.10613	80.22404	75.60554	2007.69063	-1035.27229	1523.50231	1
C	70.98358	80.74783	77.36945	-540.76542	-573.33303	49.50000	1
H	70.83289	80.09814	78.26262	-2374.20950	-1036.76023	843.75774	1
C	71.95721	81.99531	77.57724	-77.09082	666.87422	-415.84136	1
H	72.06491	82.52925	76.59334	-985.81834	617.19825	2463.91927	1
O	73.29541	81.69158	78.01273	293.29899	-391.65602	-477.80926	1
H	73.82064	82.00894	77.25115	2528.72714	-681.76262	-751.27122	1
C	71.09260	82.87218	78.55633	252.47363	-605.14473	440.05134	1
H	71.56452	83.86370	78.74691	-2052.65063	1456.49051	-1043.51830	1
H	70.07591	83.05975	78.13680	-2152.98800	-1661.97574	-161.70652	1
H	70.95825	82.37313	79.54406	-473.12726	-1584.27339	2165.61725	1

C	69.56336	81.28022	76.70608	360.36307	488.46713	505.10056	1
O	69.53820	82.29610	76.05019	-200.08817	-528.03552	-385.82850	1
N	68.58541	80.47029	77.06831	-597.54095	-226.44775	355.20479	1
H	68.86189	79.65856	77.62121	2179.23655	-1300.70981	-991.33835	1
C	67.27427	80.53830	76.52946	-687.68921	297.39807	-249.45179	1
H	66.71364	79.93774	77.27926	-349.16256	-2467.37713	-1101.72831	1
C	66.75316	82.04510	76.40490	672.47345	361.54328	-201.62880	1
H	67.41099	82.61250	75.70080	1541.41502	582.18425	2169.97026	1
H	65.72260	82.05117	75.97596	-2534.67717	60.25153	997.72295	1
C	66.75228	82.72413	77.78320	574.26774	-389.43119	-377.02542	1
C	65.76304	82.41160	78.76286	688.82679	-287.33580	-257.96133	1
H	64.96653	81.67604	78.54883	331.14182	1470.38163	2269.80013	1
C	67.76953	83.68484	78.06129	-383.52382	-675.04808	144.24917	1
H	68.52780	83.92769	77.29168	4.09847	1517.59662	2262.86397	1
C	65.82672	83.05718	79.99774	-134.76730	428.45285	-649.50303	1
H	65.06187	82.81974	80.75756	-903.23433	1600.49020	2011.53387	1
C	67.79593	84.29944	79.31544	-335.53281	-615.00040	-364.39058	1
H	68.59396	85.03382	79.52227	1596.45398	-1369.33862	1732.02659	1
C	66.83335	84.01115	80.32633	585.48547	-340.24194	-406.23938	1
O	66.84976	84.62730	81.60504	-419.15649	-173.04408	-511.94141	1
H	67.62876	85.22170	81.49310	-1071.84350	1559.71507	-1960.13012	1
C	67.27743	79.78305	75.08773	291.41878	-183.13091	710.72246	1
O	67.82526	78.71397	74.93039	379.87695	523.47396	-222.23622	1
N	66.55465	80.46489	74.19952	564.30591	373.09380	277.24371	1
H	66.12453	81.34934	74.47144	-120.59931	-2436.07824	-1214.35098	1
C	66.18348	79.78004	73.00479	404.07514	-299.37480	608.84009	1
H	66.25055	78.69979	73.27206	-1071.07882	-841.69025	2359.66467	1
C	64.74483	80.30465	72.60238	-683.12807	68.71770	-390.13096	1
H	64.02396	80.17032	73.43992	2068.88918	1596.58590	770.89603	1
H	64.77759	81.40103	72.41166	1379.86429	2342.49779	-179.85638	1
C	64.26637	79.54527	71.32616	-176.39967	537.49466	550.97268	1
H	65.04158	79.58197	70.52556	-396.28051	1942.22168	1869.33314	1
H	64.10249	78.46499	71.53545	-94.44700	586.00678	-2659.20016	1
C	62.91794	80.26556	70.86059	-291.69381	-718.18079	-150.72929	1
H	62.07552	80.20861	71.59624	-2303.38776	1089.76242	964.62032	1
H	63.02081	81.33389	70.54148	884.16729	2568.80533	-207.74283	1
N	62.54277	79.51714	69.73625	-594.49522	-252.81478	342.29276	1
H	63.05543	78.66545	69.56839	-1735.17426	2084.05705	-263.71605	1
C	61.60623	79.83438	68.93754	595.23177	-271.52487	442.22352	1
N	60.89622	81.01232	69.19594	585.53813	-424.79750	-105.81303	1
H	60.99024	81.66642	69.97180	959.88797	1327.56823	-2177.11905	1
H	60.10916	81.35301	68.64293	1578.48647	16.27378	-2220.76233	1
N	61.47827	78.88865	67.91077	-452.17763	-390.33541	-421.51853	1
H	61.98917	78.02687	67.72527	470.26107	1974.25629	-1817.92087	1
H	60.77399	78.95911	67.17517	1856.67307	-1573.97321	-1224.35416	1
C	67.19100	80.15544	71.76501	-531.47271	-581.45780	-55.06206	1
O	67.29740	79.38559	70.84297	-243.00837	192.73507	609.52485	1
N	67.64126	81.44573	71.81616	343.49288	-644.25607	38.09164	1
H	67.35350	81.73492	72.75791	-1691.16043	-1535.97525	-1484.73099	1
C	69.00879	81.77569	71.34527	542.66492	350.14337	454.42563	1
H	69.05384	82.85480	71.61808	1209.57979	-2396.95920	463.86744	1
C	70.02799	80.94549	72.08023	-737.14309	145.93010	242.71942	1
H	69.81622	79.87316	71.86650	-1050.18303	159.36419	-2509.06125	1
C	71.44171	81.26004	71.61777	-706.77696	-252.88343	-245.16230	1
H	72.20893	80.66429	72.18404	-1624.64039	-1665.49085	1417.86935	1
H	71.57800	81.02932	70.52358	2566.10521	598.51109	693.22305	1
H	71.69138	82.34671	71.76174	-1972.69758	107.10018	-1876.34312	1
C	69.95182	81.15080	73.58505	412.85148	672.58832	-27.69222	1
[truncated: 706,021 more chars]
